# The impact of an intervention to introduce malaria rapid diagnostic tests on fever case management in a high transmission setting in Uganda: A mixed-methods cluster-randomized trial (PRIME)

**DOI:** 10.1371/journal.pone.0170998

**Published:** 2017-03-13

**Authors:** Clare I. R. Chandler, Emily L. Webb, Catherine Maiteki-Sebuguzi, Susan Nayiga, Christine Nabirye, Deborah D. DiLiberto, Emmanuel Ssemmondo, Grant Dorsey, Moses R. Kamya, Sarah G. Staedke

**Affiliations:** 1 London School of Hygiene & Tropical Medicine, London, United Kingdom; 2 Infectious Diseases Research Collaboration, Kampala, Uganda; 3 Department of Medicine, University of California, San Francisco, United States of America; 4 Makerere University College of Health Sciences, Kampala, Uganda; George Washington University School of Medicine and Health Sciences, UNITED STATES

## Abstract

**Background:**

Rapid diagnostic tests for malaria (mRDTs) have been scaled-up widely across Africa. The PRIME study evaluated an intervention aiming to improve fever case management using mRDTs at public health centers in Uganda.

**Methods:**

A cluster-randomized trial was conducted from 2010–13 in Tororo, a high malaria transmission setting. Twenty public health centers were randomized in a 1:1 ratio to intervention or control. The intervention included training in health center management, fever case management with mRDTs, and patient-centered services; plus provision of mRDTs and artemether-lumefantrine (AL) when stocks ran low. Three rounds of Interviews were conducted with caregivers of children under five years of age as they exited health centers (N = 1400); reference mRDTs were done in children with fever (N = 1336). Health worker perspectives on mRDTs were elicited through semi-structured questionnaires (N = 49) and in-depth interviews (N = 10). The primary outcome was inappropriate treatment of malaria, defined as the proportion of febrile children who were not treated according to guidelines based on the reference mRDT.

**Findings:**

There was no difference in inappropriate treatment of malaria between the intervention and control arms (24.0% versus 29.7%, adjusted risk ratio 0.81 [95% CI: 0.56, 1.17] p = 0.24). Most children (76.0%) tested positive by reference mRDT, but many were not prescribed AL (22.5% intervention versus 25.9% control, p = 0.53). Inappropriate treatment of children testing negative by reference mRDT with AL was also common (31.3% invention vs 42.4% control, p = 0.29). Health workers appreciated mRDTs but felt that integrating testing into practice was challenging given constraints on time and infrastructure.

**Conclusions:**

The PRIME intervention did not have the desired impact on inappropriate treatment of malaria for children under five. In this high transmission setting, use of mRDTs did not lead to the reductions in antimalarial prescribing seen elsewhere. Broader investment in health systems, including infrastructure and staffing, will be required to improve fever case management.

## Introduction

In 2010, the World Health Organization changed guidelines for management of malaria, recommending that all suspected cases be confirmed by a parasitological test before treatment, when possible [1]. Subsequently, there has been a strong drive to scale-up use of rapid diagnostic tests for malaria (mRDTs) in areas where microscopy is unavailable or unreliable, with a goal of providing universal access to malaria diagnosis [2]. Testing for malaria is now considered one of the central pillars of malaria control, aiming to target effective antimalarials to those with laboratory confirmed malaria [3], and allowing for improved management of non-malarial fevers as well as reduced selection pressure for resistant parasites [4]. Increased testing is also promoted for surveillance purposes [5], particularly as the burden of malaria has been declining in many countries [6,7]. Implementation of rapid testing for malaria is paving the way for other point of care tests to target antibiotic use. Much hope is pinned on diagnostic technologies to turn the tide of antimicrobial resistance around the globe [8,9].

Several major challenges to the introduction of mRDTs at scale have been recognised [2]. Once mRDTs are in stock, the focus of implementation programs has been on ensuring that all suspected malaria cases are tested with a mRDT prior to prescription of recommended antimalarial drugs. Early experiences with introducing mRDTs highlighted the potential for tests to remain unused, or for negative test results to be ignored and overridden by clinical judgement [10–13]. However, interventions that have supported the introduction of mRDTs with intensive training and close supervision have increased appropriate malaria case management in Uganda [14,15], and elsewhere [16,17]. Reductions in antimalarial prescribing have been as high as 68% [18]. However, the impact of introducing mRDTs into routine care for children in areas with intense malaria transmission remains unclear, including effects on antimalarial and antibiotic prescribing.

The PRIME intervention was designed to improve the quality of care delivered for malaria and other childhood febrile illnesses in Tororo, Uganda by training health workers in public health centers, and ensuring adequate supplies of mRDTs and artemisinin-based combination therapies (ACTs) [19,20]. We conducted a cluster-randomized controlled trial to evaluate the impact of the PRIME intervention on community-level health indicators (published elsewhere)[21], and treatment of malaria in children under five years, reported here. We aimed to test the hypothesis that inappropriate treatment of malaria would be lower in intervention health centers than in control health centers. We also conducted a mixed-methods process evaluation alongside the main trial to further our understanding about the implementation, mechanisms of effect and context of the intervention [22].

## Methods

**Fig 1:**
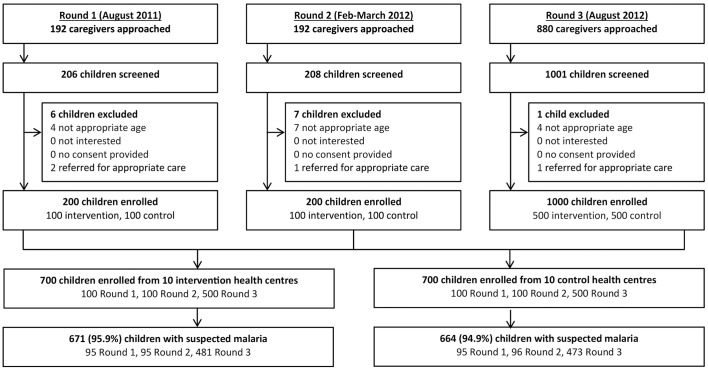
Trial profile for patient exit interviews.

The trial protocols have been published previously [19,22]. The original and final versions of the protocols can be found in S1, S2, S3 and S4. The trial was approved by the Ugandan National Council for Science and Technology (UNCST Ref HS 794), the Makerere University School of Medicine Research & Ethics Committee (SOMREC Ref 2010–108), The London School of Hygiene and Tropical Medicine Ethics Committee (LSHTM Ref 5779), and the University of California San Francisco Committee on Human Research (UCSF CHR Ref 006160). The trial profile is shown in Fig 1.

### Trial registration

This trial is registered at Clinicaltrials.gov (NCT01024426).

### Study site

**Fig 2:**
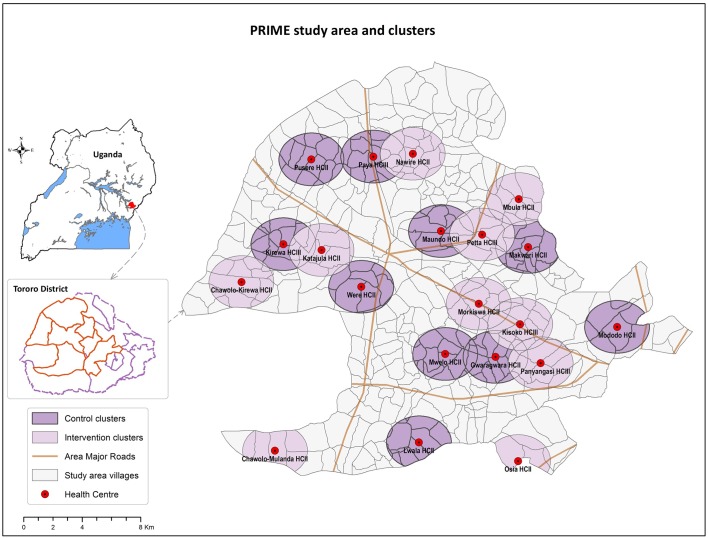
PRIME study area, health centers, and clusters in Tororo, Uganda. Reprinted from Staedke et al. [19], copyright of the authors.

Tororo district is a rural area in eastern Uganda with intense malaria transmission (estimated entomologic inoculation rate of 125 infective bites per person-year) [23]. The study area included seven sub-counties in Tororo district (Fig 2). Most local government-run health centers lack electricity and running water, and are under-staffed, run by nurses or nursing assistants [24].

### Cluster randomization

The cluster-randomized design was selected because the intervention was implemented at health centers. Of 22 health centers in the study area, two pairs of health centers had substantially overlapping catchment areas; one facility from each pair was randomly excluded. All other health centers were eligible for participation. Twenty government-run health centers (level II and III) were the units of randomization, and were assigned in a 1:1 ratio to intervention or control. Health centers were stratified by level, and restricted randomization was employed to ensure balance on geographical location and cluster size. The trial statistician generated the allocation sequence using random number generation in R version 2.11.1 (http://www.r-project.org/), and assigned health centers to study arms. Study personnel enrolled health centers after randomization; allocation was not blinded. Study personnel met with health leaders, health center in-charges, and community representatives to inform them about the study. An information sheet was used to describe the intervention, and verbal consent to participate in the study was obtained from the health center in-charges.

### PRIME intervention

Development of the PRIME intervention was guided by extensive formative research [20]. The intervention included: (1) training in-charges in health center management, (2) training health workers in fever case management and use of mRDTs, (3) training health workers in patient-centered services, and (4) ensuring adequate supplies of mRDTs and artemether-lumefantrine (AL). Intervention training delivery started in May 2011 and was completed by 1 July 2011, the start date of the evaluation period. Support for the supplies of mRDTs and AL continued until April 2013. The implementation of the intervention was monitored, following a process evaluation protocol [22].

The training workshops for health center management and patient centered services were developed specifically for the PRIME study [20], and the manuals are available online at www.actconsortium.org. For the fever case management module, we identified a training package developed by the Joint Uganda Malaria Training Program (JUMP) team utilising mRDT training guidelines and job aids adopted by Uganda’s Ministry of Health [25,26]. S5 File describes how the fever case management module was delivered. The module intended to improve antimalarial prescribing and included differential diagnoses for pneumonia, upper respiratory tract infection, otitis media, urinary tract infection, typhoid and bacterial meningitis, as well as recognition and referral of patients with severe illness.

### Patient exit interviews

**Fig 3:**
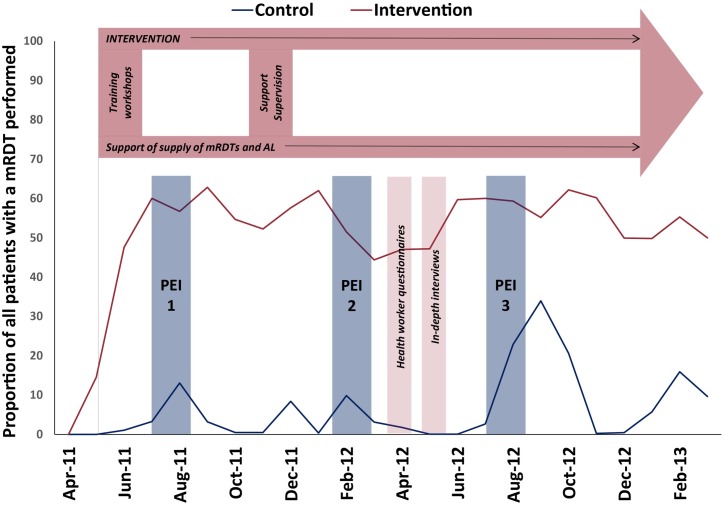
PRIME study timelines and activities. The purple figure at the top illustrates the delivery of the intervention. The blue columns represent the three rounds of the patient exit interviews (PEI). The pink columns represent the health worker questionnaires and in-depth interviews. The red (intervention) and blue (control) lines represent the proportion of all patients with a mRDT performed, which in the control arm, corresponds with availability of mRDTs.

Exit interviews were conducted with caregivers of children under-five to assess the impact of the intervention on malaria case management. Three rounds of surveys were conducted, 1, 7, and 13 months after the intervention was rolled out (Fig 3). On arrival, health workers on duty were informed of the study, and the study team approached caregivers as they left the health centers. When caregivers of young children were identified, study personnel briefly described the purpose of the study, and reviewed the eligibility criteria, which included: (1) age < 5 years, and (2) agreement of parent/guardian to provide written, informed consent. If eligibility criteria were met, a questionnaire was administered to the caregiver to gather information about the child’s illness and their experience at the health center, and the child underwent a clinical evaluation. If the child had a temperature of >38.0°C or a history of fever in the past 48 hours, a finger-prick blood sample was obtained to perform a reference mRDT. Children with a positive reference mRDT and no evidence of severe malaria, who had not been prescribed an ACT, were given AL.

### Health worker questionnaires and in-depth interviews

Approximately 10–11 months after the intervention was initiated, health workers from all 20 health centers were invited to complete a questionnaire about their current work and changes in the past year in their work at their health center after providing written informed consent (Fig 3). This included both open text questions, based on the Most Significant Change method [27], and a series of closed questions designed to assess the influence of each of the intervention components on its intended objectives. The questionnaire that evaluated confidence in following case management guidelines is included in S6 File [22]. In addition, the in-charge or acting in-charge health worker from each of the 10 intervention health centers was approached for an in-depth interview by a trained social scientist to reflect on changes at the health center over the past year. Written informed consent was provided by all interview participants.

### Outcomes

The primary outcome was the proportion of febrile children under five who were not treated according to malaria treatment guidelines, that is having either a negative reference mRDT and being prescribed AL or having a positive reference mRDT result and not being prescribed AL. Pre-specified secondary outcomes were: the proportion of children for whom AL was prescribed at the health center; the proportion of children for whom an mRDT was done at the health center; the proportion who were prescribed AL among those with a negative reference mRDT result; and among those with a positive reference mRDT results the proportion who were not prescribed AL; the proportion who were prescribed a non-ACT antimalarial; the proportion who were not prescribed any antimalarial and the proportion who were prescribed antibiotics. Additional outcomes examined included: the proportion of children prescribed other drugs, the mean number of drugs prescribed, and the mean number of antibiotics prescribed.

### Statistical analysis

We initially planned to interview 10 children and their caregivers in each of the 20 clusters at three different time points. Assuming the proportion inappropriately treated to be 50% in the control group, data from each time point would give 80% power to detect a difference in the proportion inappropriately treated for malaria between the two intervention arms of 24% (or more) at the 5% significance level, assuming a coefficient of variation between clusters of 0.2 and allowing for the stratified design.

However, data from the first two time points indicated a lower level of inappropriate treatment across both arms than anticipated in the original sample size calculation. Therefore, the sample size required for the third and final time point was increased to 50 per cluster. Assuming the proportion of children inappropriately treated to be 35% in the control group this would give 80% power to detect a difference in proportion inappropriately treated of 12% (or more) at the 5% significance level with coefficient of variation 0.2.

Trial analysis was done at the cluster level [28]. Data from the three time points were analyzed together due to the relatively small sample size for the first two time points. Cluster-level proportions for each outcome were calculated and log transformed to normalize their distributions. Crude risk ratios for the effect of the intervention were calculated by taking the exponential of the difference in the mean of the cluster-specific log proportion between the two arms [28]. Stratified t-tests were used to calculate p-values for the crude effect of the intervention, where the within-stratum between-cluster variance was estimated as the residual mean square from a two-way analysis of variance of the log-proportions on stratum and treatment arm, including an interaction term. Finally, 95% confidence intervals (CI) for crude risk ratios, adjusting for stratum, were calculated from this variance using a t-statistic with 16 degrees of freedom, and then applying the exponential transformation. Adjusted analyses for the effect of the intervention on each outcome was also performed, adjusting for child’s age and sex using a two-stage approach [19,28].

In further analyses, to assess plausibility of an effect of the intervention, we did a cluster-level analysis to evaluate the association between intervention “dose” (the proportion of health workers at a HC who received training in fever case management) and the proportion of children experiencing the primary and secondary outcomes at that HC, using linear regression. We also investigated the following cluster-level characteristics for associations with the outcomes: mean age of health workers, proportion male health workers, mean education level, mean health worker length of time working at health center, mean number of training workshops attended, and proportion of health workers who received mRDT training. Scores for confidence in malaria case management were calculated as non-weighted aggregates from the questionnaires (S2 File) and also considered as a covariate in this analysis.

Individual-level analyses investigating differences in ACT prescribing behaviour, comparing children who reportedly had an mRDT performed at the health centers to those who did not were done using logistic regression with random effects to allow for clustering.

Qualitative analysis of in-depth interviews and open-text questionnaire responses involved reviewing each health worker as a case, situated in the context of their health center, and then coding narratives and text line-by-line to generate lists of repeating ideas which were grouped into emerging themes. These themes were explored in relation to the quantitative findings arising, and vice versa. Analyses were done using Stata version 13 (StataCorp, College Station, Texas, US) and Nvivo version 10 (QSR International). A p-value of less than 0.05 was taken to indicate statistical significance.

## Results

### Study population

In all, 1400 children participated in the exit interviews, including 200 in round 1, 200 in round 2, and 1000 in round 3 (Fig 1). Overall, the prevalence of malaria was high: 1336 (95.4%) children were febrile or reported a history of fever in the last 48 hours, and among these, 1,006 (75.3%) had a positive reference mRDT (Table 1). Questionnaires were administered to 49 health workers (22 at control and 27 at intervention health centers) and 10 health center in-charges were interviewed.

**Table 1:** Characteristics of exit interview participants by intervention arm.

Characteristic		Control (n = 700)	Intervention (n = 700)
Sex of child^1^	Male, n (%)	342 (48.9%)	349 (49.9%)
Age of child, years	<1	225 (32.1%)	224 (32.0%)
	1	194 (27.7%)	202 (28.9%)
	2	108 (15.4%)	120 (17.1%)
	3	88 (12.6%)	80 (11.4%)
	4	85 (12.1%)	74 (10.6%)
Sex of caregiver	Male, n (%)	28 (4.0%)	37 (5.3%)
Age of caregiver, years	Median (IQR)	25 (21–31)	25 (21–30)
Fever or history of fever^2^	n (%)	665 (95.0%)	671 (95.9%)
mRDT result^3^^,^^4^	Positive, n (%)	495 (74.6%)	511 (76.2%)

^1^ Sex missing for 1 child in intervention arm round 2

^2^ Temperature taken if caregiver reported child fever in last 48 hours

^3^ mRDT done if child had fever or history of fever

^4^ No mRDT result for 1 child in control arm round 3

### Impact of the intervention on uptake of mRDTs and prescribing

In 2009–2010, when the formative research for the PRIME study was conducted, health centers were experiencing major stockouts of AL, the first-line recommended treatment for uncomplicated malaria, and lacked mRDTs. However, by the the time the PRIME trial began in 2011, a new ‘push’ delivery system had been implemented nationwide and stockouts of AL became less common. During the trial, mRDTs were generally unavailable in the control health centers but were intermittently delivered across the district (without training), including coinciding with our third round of exit interviews (Fig 3).

At the times of our exit interviews, the proportion of children tested with an mRDT was higher in the intervention health centers than in the controls (Table 2), but this difference was not statistically significant, and testing varied widely between health centers. Despite the differences in mRDT uptake, in both arms nearly two-thirds of consulations resulted in a prescription for AL. Children were prescribed an antimalarial or antibiotic in 86.4% of the consultations. Antibiotic prescriptions were higher in the intervention than the control arm (56.7% versus 47.1%) but this difference was not significant (Table 2).

**Table 2:** Effect of trial intervention on use of mRDTs and fever case management.

Trial arm	n/N	Proportion (range)^1^	Crude risk ratio (95% CI)	P-value	Adjusted risk ratio (95% CI)^2^	P-value
**mRDT done at health centers**^**3**^						
Control	287/698	41.2% (0%-83%)				
Intervention	475/696	68.4% (13%-97%)	1.66 (0.88, 3.13)	0.11	1.66 (0.88, 3.12)	0.11
**AL prescribed at health center**						
Control	443/700	63.3% (37%-90%)				
Intervention	450/696	64.6% (41%-81%)	1.02 (0.84, 1.24)	0.83	1.03 (0.84, 1.25)	0.79
**Antibiotic prescribed at health center**						
Control	330/700	47.1% (14%-83%)				
Intervention	397/700	56.7% (40%-81%)	1.20 (0.83, 1.74)	0.30	1.21 (0.83, 1.74)	0.30
**Inappropriate malaria treatment**^**4**^						
Control	197/664	29.7% (14%-50%)				
Intervention	162/671	24.0% (12%-43%)	0.81 (0.56, 1.17)	0.24	0.81 (0.56, 1.17)	0.24
**Reference mRDT- but received AL**						
Control	69/169	42.4% (0%-92%)				
Intervention	47/160	31.3% (5%-59%)	0.74 (0.38, 1.44)	0.35	0.71 (0.36, 1.38)	0.29
**Reference mRDT+ but did not receive AL**						
Control	128/495	26.1% (4%-51%)				
Intervention	115/511	22.5% (9%-52%)	0.86 (0.50, 1.48)	0.56	0.85 (0.50, 1.46)	0.53
**Reference mRDT+ received a non-ACT antimalarial**^**5**^^,^ ^**6**^						
Control	19/495	3.8% (0%-11%)				
Intervention	33/509	6.3% (0%-35%)	1.65 (0.29, 9.44)	0.55	1.61 (0.29, 9.08)	0.57
**Reference mRDT+ but did not receive any antimalarial**^**6**^						
Control	109/495	22.3% (4%-46%)				
Intervention	80/509	15.8% (7%-25%)	0.71 (0.41, 1.22)	0.20	0.71 (0.41, 1.21)	0.19

^1^ Arithmetic mean and range of cluster-specific proportions

^2^ Adjusted for age and sex of child

^3^ 6 missing values for mRDT done at health facility. In the control arm, availability of mRDTs varied over time, and uptake reflected this with 33% reporting an RDT done in round 1, 4% in round 2 and 50% in round 3. Levels of testing reported at intervention facilities were more stable at 79%, 71% and 66%

^4^ Inappropriate treatment defined as (number of children who were PRIME mRDT+ but did not receive an ACT + number of children who were PRIME mRDT- but received an ACT)/(number of children who had a PRIME mRDT done)

^5^ Inappropriate treatment with a non-ACT antimalarial defined as (number of children who were PRIME mRDT+ and received only a non-ACT antimalarial)/(number of children who were PRIME mRDT+)

^6^ Missing data on non-ACT treatment for two children in intervention arm.

### Impact of intervention on inappropriate treatment of malaria

Inappropriate treatment of malaria was slightly lower in the intervention arm than the control arm, but this difference was not statistically significant (24.1% and 29.7%, respectively, adjusted risk ratio 0.81 [95% CI: 0.56, 1.17] Table 2). Of children with a negative reference mRDT, fewer were inappropriately prescribed AL in the intervention arm than the control arm (31.3% and 42.4%, respectively), but the difference between the study arms was not significant (aRR 0.71 [95% CI: 0.36, 1.38]. Notably, not all children with a positive reference mRDT were prescribed AL. Of these children, some received a non-ACT antimalarial, but a substantial proportion did not receive any antimalarial treatment (15.7% intervention, 22.0% control).

**Fig 4:**
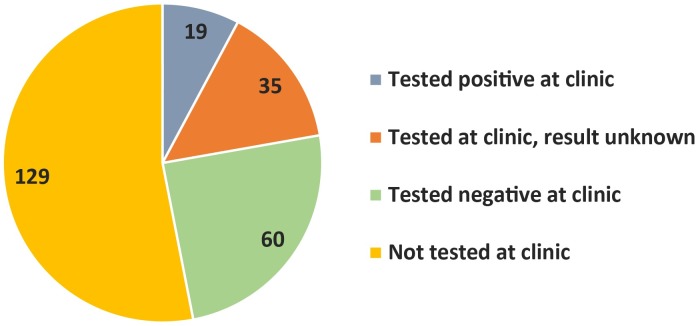
Children testing positive by reference mRDT but not prescribed AL at the health center.

Of the 241 children with a positive reference mRDT who were not prescribed AL, approximately half (53.5%) were not tested for malaria at the health center, and thus malaria may have been overlooked by the health worker (Fig 4). Of the 112 children tested for malaria, caregivers for just over half (53.6%) reported that the result was negative. Many caregivers did not know the test result (31.3%). Some reported that their child had tested positive but was not prescribed AL (17.0%); of these, most were prescribed quinine.

### Plausibility of intervention effect

To establish plausibility of the intervention effect on fever case management, we conducted a cluster-level analysis of the dose-response effect of participation in the intervention. At health centers where all health workers had attended the fever case management training, the proportion of children tested with an mRDT was 29% higher (an absolute difference), but not statistically significantly so, than in health centers where no health workers had attended training (95% CI: -27%, 85%; p = 0.26). At health centers where health workers scored higher on the questionnaire assessing their confidence and ability to use mRDTs in line with PRIME guidelines, children were more likely to be tested for malaria (an increase in testing of 16.6%, 95% CI: 2.8%, 30.6%; p = 0.02, for each absolute increase of 10% in confidence score) and were less likely to be prescribed AL in the setting of a negative reference mRDT (a decrease in prescribing of 12.9%, 95% CI: 3.7%, 22.0%; p = 0.008, for each absolute increase of 10% in confidence score). Consistent with this, those who had attended the fever case management training had on average a higher score in confidence in following guidelines than those who had not (73.7 compared with 57.8, p = 0.04). Taken together, those who attended the training were more confident implementing fever case management guidelines, and this was related to higher uptake of mRDTs and adherence to negative test results.

### Overall impact of mRDTs on prescribing

Because mRDTs and AL were available across health centers at the 13 month time point when a majority of the patient exit interviews took place (Fig 3), but they were not always taken up, we were able to explore how use of mRDTs affected prescription of AL across all health centers, independent of study arm assignment. Overall, targeting of AL was better amongst patients who were tested for malaria at the health center (n = 746) than those who were not tested (n = 579, Table 3). Children with a negative reference mRDT who were reportedly tested for malaria at the health center were much lesss likely to be prescribed AL than those who were not tested (18.1% vs 56.4%, p<0.001). However, use of mRDTs appeared to have had less of an impact on antibiotic prescribing. Fewer children with a positive reference mRDT were prescribed an antibiotic if they were tested at the health center than those not tested (44.9% vs 52.9%), but about two-thirds of children with a negative reference mRDT were prescribed an antibiotic regardless of whether they were tested for malaria at the health center. Overall, polypharmacy was common (Table 3). Children who were not tested for malaria at the health center were prescribed more drugs than those who were tested (mean 2.85 versus 2.56, p<0.001), mainly due to higher antimalarial prescribing.

**Table 3:** Prescriptions to children with fever or history of fever, by malaria and health center testing status.

	Overall		Tested at health center		Not tested at health center	
	Malaria^1^	Non-malaria	Malaria^1^	Non-malaria	Malaria^1^	Non-malaria
	(n = 1004)	(n = 327)	(n = 569)	(n = 177)	(n = 430)	(n = 149)
**Antimalarials**						
Prescribed AL (n, %)	763 (76.0%)	116 (35.5%)	456 (80.1%)	32 (18.1%)	305 (70.9%)	84 (56.4%)
Prescribed any antimalarial (n, %)	815 (81.2%)	124 (37.9%)	488 (85.8%)	34 (19.2%)	322 (74.9%)	90 (60.4%)
Prescribed quinine (n, %)	64 (6.4%)	8 (2.5%)	38 (6.7%)	2 (1.1%)	23 (5.4%)	6 (4.0%)
**Antibiotics**						
Prescribed any antibiotic (n, %)	485 (48.2%)	205 (62.3%)	256 (44.9%)	108 (61.0%)	228 (52.9%)	97 (64.2%)
Prescribed trimethoprim-sulfamethoxazole (n, %)	406 (40.4%)	158 (48.3%)	227 (39.9%)	93 (52.5%)	178 (41.4%)	65 (43.6%)
Prescribed amoxicillin (n, %)	50 (5.0%)	34 (10.4%)	20 (3.5%)	11 (6.2%)	30 (7.0%)	23 (15.4%)
**Other drugs**						
Prescribed any anthelminthic (n, %)	85 (8.5%)	25 (7.6%)	38 (6.7%)	12 (6.8%)	47 (10.9%)	13 (8.6%)
Prescribed any antifungal (n, %)	16 (1.6%)	9 (2.7%)	7 (1.2%)	4 (2.3%)	9 (2.1%)	5 (3.3%)
Prescribed panadol (n, %)	886 (88.1%)	284 (86.3%)	518 (90.9%)	157 (88.7%)	363 (84.2%)	126 (83.4%)
Prescribed eye ointment	36 (3.6%)	10 (3.0%)	11 (1.9%)	1 (0.6%)	25 (5.8%)	9 (6.0%)
Prescribed multivitamins	46 (4.6%)	13 (4.0%)	18 (3.2%)	8 (4.5%)	28 (6.5%)	5 (3.3%)
Prescribed zinc	47 (4.7%)	18 (5.5%)	14 (2.5%)	10 (5.7%)	33 (7.7%)	8 (5.3%)
Prescribed antihistamine	68 (6.8%)	36 (10.9%)	24 (4.4%)	18 (10.2%)	43 (10.0%)	18 (11.9%)
Prescribed other drug	21 (2.1%)	16 (4.9%)	3 (0.5%)	7 (4.0%)	17 (3.9%)	9 (6.0%)
**Overall**						
Total number of drugs prescribed (mean, SD, median, range [min/max])	2.78 (SD 0.93), median 3 (0–7)	2.48 (SD 0.90), median 2 (0–5)	2.67 (SD 0.79), median 3 (0–6)	2.25 (SD 0.73), median 2 (0–4)	2.94 (SD 1.07), median 3 (0–7)	2.77 (SD 0.98), median 3 (0–5)
Total number of antimicrobials prescribed (mean, SD, median, range [min/max])	1.40 (SD 0.63), median 1 (0–4)	1.11 (SD 0.73), median 1 (0–3)	1.39 (SD 0.59), median 1 (0–4)	0.89 (SD 0.66), median 1 (0–2)	1.41 (SD 1.69), median 1 (0–4)	1.38 (SD 0.72), median 1 (0–3)

^1^ Malaria diagnosis confirmed by reference mRDT.

### Health worker perceptions of incorporating mRDTs into practice

Intervention health workers identified mRDTs as the most significant change occurring at their health centers over the past year. At control health centers, where mRDTs were also delivered intermittently, mRDTs were occasionally mentioned. However, resoundingly, the biggest change for control health workers was the stable supply of AL due to the government’s new drug delivery (‘push’) system. Some health workers, mostly at control health centers, also mentioned the changes in management of medicines due to the support received from the SURE (Securing Ugandans’ Rights to Essential Medicines) NGO project, which was active at 19 of the participating health centers.

At some health centers, staff systematically used mRDTs to test every suspected malaria case. In these health centers, health workers appeared more confident managing patients with negative mRDTs. For example, the in-charge at one intervention health center with high testing rates explained how he consulted a guidebook to manage mRDT negative cases.

*HCW 801 (nursing assistant): Aaa the most important thing I got is when I have mRDT. I take, I prick someone and I take the blood then check it whether it’s malaria or another is not* *malaria*

*NC: So if it’s not malaria how do you know that it’s another* *disease?*

*HCW 801: That one I go back to my book, they call it ‘dictionary book’ then I read it, then I read what the complaint of the patient says, then **it makes me to know that this is not what I have ruled malaria**,* *I am to treat this.*

*NC: So where* *did you get that book from, the one that helps you?*

*HW 801: That one we got it from DMOs [District Medical* *Officer’s] office*

However, elsewhere, particularly the larger health centers and the overstretched smaller health centers, mRDTs were used less frequently. In these sites, staff felt that tests were too time-consuming in a context where they were overworked, with inadequate staffing levels. One intervention in-charge, who threatened to withdraw from the project due to the burden of testing and keeping records, described the burden of testing on her team.

*“It has given us it has added on our workload.* *You know we don’t have laboratory assistant, and now you have to test. I am not a lab personnel, I have to go in something which is out of my duties. So at the end of the day I have to see patients, I have to test, I have to do what, so **it becomes hectic for me**. Not only for me but for all the staff. And that is why we raised up an alarm the other time.” (HCW 201, midwife, emphasis added)*

mRDTs may not have been a priority for all health center managers, resulting in lower testing rates. At one intervention HCIII, where fewer than 50% children were tested, the project supervision team repeatedly noted that access to the store cupboard was limited in the absence of the in-charge. Here, one of the health workers complained that

*“Some busy days you find* *few mRDTs are left out compared to the **number of patients** and accessing the keys is very difficult.” (HCW 605, enrolled nurse)*

Testing seemed to be taken up either almost never or very frequently amongst control health centers. Half very rarely did mRDTs, not wanting to test without training and left the mRDTs in their boxes. At these facilities, health workers remained of the view that presumptive malaria treatment is the national guideline,

*“Treatment of malaria can also* *be given to a patient even if there is no mRDT, under national guidelines on management of common conditions.” (HCW 2002, nursing assistant)*

However, the other five control health centers tested over two-thirds of the children during our study. Here, individuals appeared active in wanting to gain skills in using the tests, and after the sporadic supplies of mRDTs from the government were finished spoke of how useful they had found the tests to be for case management.

### Health worker perceptions of the impact of mRDTs on prescribing

The responses about changes at health centers revolved around a key concern of availability of drugs, which are seen as an essential component of care. In this context, with a history of severe stockouts, medicines were seen as a precious resource. Health workers felt entrusted to provide these commodities to patients, and were happy to have more of them available. A key attractive feature of the mRDT was to ensure that the drugs were not ‘wasted’. The tests would ensure that the drugs went to ‘proper illness’ the ‘needy’ and those ‘deserving’ it because their diagnosis was ‘confirmed’, ‘definite’ and ‘proper’, as illustrated by this intervention health worker.

*“Use of mRDT has helped in effective management of especially malaria* *patients in that they are given the **right treatment** after testing not like way back when malaria treatment was on assumption making [for] **those who did not deserve malaria treatment”** (HCW 608, emphasis added)*

The process of rationing, or ‘controlling our number of patients who are taking Coartem’, was also appealing to health care workers who saw the test as an arbiter–able to assign patients to ‘deserving’ or ‘undeserving’ of these drugs. This ability to use (or at times ignore) a biomedical test strengthened health workers’ authority in two ways: by showing their association with biomedicine and access to its commodities, and by strengthening their ability to grant access of patients to the commodities they desired (medicines). By extension, health care workers reported that this had increased patients’ confidence in the care they received at health centers. Not only would patients be able to access drugs, which were now more reliably available, but they would only be able to do so after ‘proving’ that they ‘deserved’ these medicines.

*“The patients know now before getting* *treatment **you must be tested to confirm that you have malaria**” (HCW 210, emphasis added)*

Thus, the nature of biomedicine, as a priviledged and somewhat mystified method of managing illnesses, was reinforced through the presence and choice to use mRDTs.

### Patient perspectives of the impact of mRDTs on delivery of care

From a patient perspective, many who had their malaria status ‘confirmed’ through mRDTs were, as reported by the health care workers, pleased with their experience. The mRDTs were attractive as a marker of good biomedical care. However, at other times patients were frustrated with being unable to access the care they wished to received, being blocked by the mRDT. The focus on the mRDT and thereby on malaria / not malaria, appeared to draw focus away from other ailments.

*I would have loved it if the* *health worker had asked me or discussed about the hernia which is bothering the child. **We only talked about the fever**. (Mother at HC6, emphasis added)*

Indeed, health workers too recognised that patients could become discouraged by the insistence on use of mRDTs, as reported by this nurse.

*I am even seeing the numbers of patients, the many patients we have been having, at this moment it is a bit* *going down. Because now, **we are using the mRDTs to scrutinize them**. They come mostly–there are those who come aiming at the ACTs to go keep at their homes. So when you come and you are not given ACT today, you come and you are scrutinized, and you are not given the next day. So you find **people are getting discouraged to come** (HCW 1003, emphasis added)*

Thus, in this context, the introduction of mRDTs was largely seen as positive, and reinforced some of the desirable aspects of providing and accessing biomedical care. The main downsides for health workers were the work load they required and for patients their potential to overshadow their other health care needs.

## Discussion

In this cluster-randomized trial, we found that the PRIME intervention’s programme of training workshops and supervision, plus provision of mRDTs and AL when stocks ran low, increased uptake of mRDTs but did not have the desired impact on treatment of malaria. Although a reduction in inappropriate antimalarial treatment of 6% was observed in the intervention arm compared to the control arm, this difference was not statistically significant. In this high malaria transmission setting, we did not see the substantial reductions in antimalarial prescribing that have been observed in other scenarios with similarly intensive interventions but lower levels of malaria [16,17]. Concerningly, some patients with positive mRDT results did not receive the first-line antimalarial treatment. These findings demonstrate that diagnostic tests are not a straightforward solution to improving medicines use in all settings.

Given improved availability of AL and mRDTs in both intervention and control health centers at the time of the exit interviews, differences between study arms may be attributed to the training and supervision component of the intervention, which appeared to improve health worker confidence in testing and restricting antimalarials, supporting the plausibility of effect. However, the limited size of effect may reflect the large proportions of children for whom antimalarial prescription was classifed as appropriate, with three quarters of febrile children testing positive for malaria. This echos previous modelling exercises that have predicted the cost of introducing mRDTs in high transmission areas may outweigh the benefit [29]. In addition, although mRDTs can have several operational advantages, they may lack specificity. mRDTs that identify histidine rich protein II (HRP-2), a parasite antigen that may circulate for weeks following successful malaria treatment, may be falsely positive due to recent prior infection [30]. In high transmission settings like Tororo, where reinfection is common, a substantial proportion of patients presenting with conditions other than malaria may be over-diagnosed as malaria despite the use of an mRDT [31,32].

Across both trial arms, when mRDTs were used, targeting of AL improved substantially. However, testing rates were highly variable across the health centers in both study arms, reflecting the different contexts and needs of the health centers. Some health centers suffered from extremely poor infrastructure. Staff shortages and competing priorities left many health centers with too few staff working at any one time to allow for routine use of mRDTs. Other health centers benefited from highly motivated staff willing to extend themselves to take on this new technology. In order to increase frequency of testing, mRDTs would have to be supported by substantial improvements in infrastructure, personnel and ongoing supportive supervision, as documented elsewhere [33]. Such improvements are likely to require systemic change in health systems rather than to be addressed through single-disease focused programmes.

Of concern, a surprisingly large number of children with a positive reference mRDT were not prescribed AL. Much of the effort in malaria case management focuses on reducing inappropriate overuse of antimalarial drugs, assuming patients with a positive test will receive an ACT. However, ensuring that patients with malaria receive prompt effective treatment remains a major issue. In our setting, this undertreatment of malaria could not be attributed to ACT stockouts, as has been the case elsewhere [34], as supplies of AL were stable during the study. Lack of health worker knowledge of guidelines also seems unlikely; in their registers, health workers recorded prescribing AL to over 93% RDT positive cases (data not shown). It is possible that health workers incorrectly interpreted the mRDT results as negative, perhaps not waiting sufficient time for results to develop, or performing tests incorrectly. In many cases, caregivers did not know their child’s test results, which could reflect the challenging logistics of integrating even ‘simple’ and ‘rapid’ tests into resource and time constrained care scenarios. Finally, it is also possible that health workers witheld AL while the exit interviews were ongoing, knowing that the study team would test and treat patients.

The observation that antibiotics were so frequently prescribed, to around half of febrile children, is a concern in the context of mounting drug resistance. In other settings, it has been suggested that fewer than one in four febrile children require antibiotics [35,36]. While it is appealing to imagine that adding further point-of-care tests would limit overuse of antibiotics, the tendency to provide at least one antimicrobial to febrile children needs to be taken seriously. This is particularly true in low-resource settings where healthcare may consist of little more than provision of medicines [37].

This study had several limitations. The exit interviews were carried out at three timepoints, aiming to capture prescribing practices over time. However, this design raises several limitations in interpretation. First, given the unexpectedly low level of inappropriate malaria treatment and the different contextual scenarios that arose during the course of the trial, such as delivery of mRDTs to control health centers, it would have been desirable in hindsight to collect data on sufficient numbers of children to allow investigation of a change in the impact of the intervention over time. However, the sample size was not sufficient to allow this interaction analysis. The fact that a majority of our data are from 13 months post-training, however, means the findings presented are likely to be closer to a scale-up scenario than data collected at times of immediate post-intervention attentiveness. Second, slightly different study teams carried out the exit interviews at the different time points, partly due to the larger sample required for the third round of interviews, and thus the conduct of the intervivews across the three rounds may not have been universal. Third, because of changes in health center personnel over time, we were unable to link the exit interview data to specific health worker characteristics for more detailed analyses of explanatory variables such as intervention exposure.

## Conclusions

Although the PRIME intervention did not have the desired impact on inappropriate treatment of malaria, our results suggest that mRDTs have the capacity to improve targeting of ACTs. However, achieving this will require further efforts in supporting infrastructure as well as training and supervision programmes to ensure all eligible patients are tested. Such investments would also reap benefits in supporting the case management of other non-malarial illnesses amongst febrile patients, which is especially important if the specificity of mRDTs is low. In this high malaria transmission setting, benefits of mRDTs in terms of reducing ACT wastage are likely to be small. Rather, the benefit of mRDTs should be viewed in light of broader improvements to fever case management.

## Supporting information

S1 File PRIME protocol V1.1, 18 September 2010. **Original version of the PRIME protocol.** This file is a copy of the original study protocol of the PRIME cluster-randomized trial, which was approved by the overseeing ethics committees.
(PDF)

S2 File PRIME protocol V1.7, 15 February 2013. **Final version of the PRIME protocol.** This file is a copy of the final study protocol of the PRIME cluster-randomized trial, incorporating all amendments approved by the overseeing ethics committees.
(PDF)

S3 File PROCESS protocol. **V1.1, 23 February 2011. Original version of the PROCESS protocol.** This file is a copy of the original protocol of the PROCESS study which ran alongside the PRIME cluster-randomized trial, which was approved by the overseeing ethics committees.
(PDF)

S4 File PROCESS protocol V1.3, 15 April 2013. **Final version of the PROCESS protocol.** This file is a copy of the final protocol of the PROCESS study which ran alongside the PRIME cluster randomized trial, incorporating all amendments approved by the overseeing ethics committees.
(PDF)

S5 File Delivery of fever case management (FCM) module in the PRIME study. This file includes a description of the way the intervention delivery was monitored, results of how the training and supervision were delivered at intervention health centres, description of what happened at control health centres and movement between health centres. It also includes copies of the questionnaires filled by trainers and participants to monitor delivery.
(DOCX)

S6 File Questionnaire evaluating health worker confidence in following case management guidelines. This file shows the self-filled questionnaire health workers were asked to complete 10 months after the initial intervention training. These data were used to represent the confidence health workers had in the areas covered by the training and supervision.
(DOCX)

S7 File CONSORT 2010 checklist. This file includes a checklist of information to include when reporting a cluster-randomized trial, indicating where specific items are reported within the paper.
(DOCX)
