# Supplementary material for: The impact of an intervention to introduce malaria rapid diagnostic tests on fever case management in a high transmission setting in Uganda: A mixed-methods cluster-randomized trial (PRIME)
Source: PLoS One. 2017 Mar 13;12(3):e0170998. doi: 10.1371/journal.pone.0170998 (PMC5347994; doi:10.1371/journal.pone.0170998)
Supplement: S1 File — Original version of the PRIME protocol. This file is a copy of the original study protocol of the PRIME cluster-randomized trial, which was approved by the overseeing ethics committees. (PDF) [file pone.0170998.s001.pdf]

The ACT PRIME Study

Evaluating the impact of enhanced health facility-based care for malaria and febrile illnesses in children in Tororo, Uganda

18 September 2010
Protocol Version 1.1

Principal Investigator: Dr. Sarah Staedke
Co-investigators: Dr. Moses Kamya, Dr. Grant Dorsey, Dr. Clare Chandler

Funding Source: Gates Foundation, ACT Consortium

Institutional Review Boards

- Makerere University Faculty of Medicine Research and Ethical Committee
- Ugandan National Council for Science and Technology
- LSHTM Ethics Committee: 5779
- University of California, San Francisco Committee on Human Research: 006160

THE REPUBLIC OF UGANDA

CONTENTS

STUDY INFORMATION 6
PROJECT SYNOPSIS 7
PROJECT TEAM AND PARTICIPATING SITES 8
ABBREVIATIONS AND ACRONYMS 11

1 BACKGROUND 12
1.1. Introduction 12
1.2. Malaria diagnosis and treatment 12
1.2.1. Barriers to service in the formal healthcare sector 12
1.2.2. Universal diagnostic testing 12
1.2.3. Training health workers in malaria diagnosis 13
1.3. Malaria in Uganda 13
1.3.1. Overview 13
1.3.2. Antimalarial drug policy 14
1.3.3. Affordable Medicines Facility – malaria 14
1.4. Health facility interventions 15
1.4.1. What is quality care and what are the problems? 15
1.4.2. How can quality of care be improved? 15
1.5. Phase I results 17
1.5.1. Census survey 17
1.5.2. Public health facilities 17
1.5.3. Community medicine distributors 18
1.5.4. Qualitative study 18

2 RATIONALE 21
2.1. Problem statement 21
2.2. Study overview 21
2.3. Study population and design 22

3 STUDY OBJECTIVES 23

4 STUDY DESIGN 24
4.1. Overview 24
4.2. Study site 25
4.3. Study population 26
4.4. Cluster Randomisation 26
4.5. Outcome measures 28

5 HEALTH FACILITY INTERVENTION 29
5.1. Overview 29
5.2. Health Center Management 30
5.2.1. Overview 30
5.2.2. Human resource management 30
5.2.3. Financial management 31
5.2.4. Supply management 31
5.3. Health information management 31
5.4. Health worker training 32
5.4.1. Training in fever case management and use of RDTs 32
5.4.2. Training in patient-centered services 32

5.5. Consumables 35
5.5.1. Drug delivery from National Medical Stores 35
5.5.2. Supply artemether-lumefantrine 36
5.5.3. RDTs for malaria 36

6 STANDARD CARE 37
6.1. Overview 37
6.2. Staffing and patient attendance 37
6.3. Infrastructure and supplies 37
6.4. Standard care 37

7 CROSS-SECTIONAL SURVEYS 38
7.1. Overview 38
7.2. Definitions 38
7.3. Enrollment 38
7.3.1. Recruitment 38
7.3.2. Screening 38
7.3.3. Informed Consent 39
7.3.4. Enrollment 39
7.4. Survey procedures 39
7.4.1. Survey questionnaire 39
7.4.2. Clinical and laboratory assessment 40
7.4.3. Management of ill children 40

8 COHORT STUDY 41
8.1. Overview 41
8.2. Enrollment 41
8.2.1. Recruitment 41
8.2.2. Screening 41
8.2.3. Informed Consent 42
8.2.4. Enrollment 42
8.2.5. Management of ill children at enrollment 42
8.3. Household Surveys 42
8.4. Follow-up 43
8.4.1. Household diaries and monthly visits 43
8.4.2. Follow-up evaluations 43
8.4.3. Management of ill children during follow-up 44

9 M&E OF HEALTH FACILITY INTERVENTION 45
9.1. Overview 45
9.2. Patient Exit interviews 45
9.2.1. Overview 45
9.2.2. Recruitment 45
9.2.3. Informed Consent 45
9.2.4. Interview 46
9.2.5. RDT results 46
9.3. Health Facility Surveillance 47
9.3.1. All facilities 47
9.3.2. HFI facilities 47
9.4. Health worker knowledge questionnaires 48

10 ADVERSE EVENT MONITORING 49
10.1. Overview 49

10.2. Definitions 49
10.3. Identification of adverse events 49
10.4. Reporting of adverse events 49
10.5. ACT consortium drug safety register 50
10.5.1. Overview 50
10.5.2. Database 50
10.5.3. Mechanisms for data handling/sharing: 50

11 LABORATORY PROCEDURES 52
11.1. Microscopy 52
11.2. Rapid diagnostic tests for malaria 52
11.3. Hemoglobin measurement 52
11.4. Molecular studies 52

12 STATISTICAL ISSUES 54
12.1. Sample size calculations 54
12.1.1. Cluster randomized study 54
12.1.2. Cohort study 54
12.1.3. Patient exit interviews at health facilities 55
12.2. Analytical plan 55
12.2.1. Analytical approach 55
12.2.2. Baseline characteristics and trial profile 55
12.2.3. Cross-sectional survey outcomes 56
12.2.4. Cohort study outcomes 56
12.2.5. Outcomes measured at health facilities 57

13 DATA & SAFETY MONITORING BOARD 58
13.1. Overview 58
13.2. Monitoring plan 58
13.3. Stopping guidelines 58

14 DATA MANAGEMENT 59
14.1. Data management 59
14.1.1. Cross-sectional surveys 59
14.1.2. Cohort study 59
14.1.3. M&E of health facilities 59
14.2. quality assurance and quality control 60
14.3. Records and storage 60

15 PROTECTION OF HUMAN PARTICIPANTS 61
15.1. Institutional Review Boards 61
15.2. Informed consent process 61
15.3. Confidentiality 61
15.4. Risks and discomforts 61
15.4.1. Privacy 61
15.4.2. Risks of randomization 62
15.4.3. Fingerprick blood draws 62
15.4.4. Risk of artemether-lumefantrine 62
15.4.5. RDTs for malaria 62
15.4.6. Compensation 63
15.4.7. Alternatives 63

16 REFERENCES 64

17 APPENDICES 72

STUDY INFORMATION

Title	PRIME STUDY – Evaluating the impact of enhanced health facility-based care for malaria and febrile illnesses in children in Tororo, Uganda
Principal investigator	Sarah Staedke, MD, PhD<br>Clinical Senior Lecturer, London School of Hygiene and Tropical Medicine<br>Uganda Malaria Surveillance Project / Infectious Diseases Research Collaboration, Uganda
Co-investigators	Moses Kamya, MBChB, MPH, PhD; Professor, Makerere University<br>Grant Dorsey, MD, PhD; Associate Professor, University of California, San Francisco<br>Clare Chandler, PhD; Lecturer, London School of Hygiene and Tropical Medicine
Sponsor	London School of Hygiene & Tropical Medicine<br>Department of Clinical Research<br>Faculty of Infectious and Tropical Diseases<br>Keppel Street, London WC1E 7HT<br>United Kingdom
Funding agency	ACT Consortium grant from the Bill & Melinda Gates Foundation
Participating institutions	London School of Hygiene & Tropical Medicine, UK<br>Infectious Diseases Research Collaboration, Kampala, Uganda<br>University of California, San Francisco, USA
Institutional review boards	Makerere University Research and Ethics Committee<br>Uganda National Council for Science and Technology<br>London School of Hygiene & Tropical Medicine<br>University of California, San Francisco Committee on Human Research

PROJECT SYNOPSIS

Title	PRIME STUDY – Evaluating the impact of enhanced health facility-based care for malaria and febrile illnesses in children in Tororo, Uganda
Objectives	1. To compare the impact of enhanced health facility-based care to current standard of care on key population-based indicators, including the prevalence of anemia in children under five.<br>2. To compare the impact of enhanced health facility-based care to current standard of care on key longitudinal indicators, including antimalarial treatment incidence density, in a cohort of children under five.<br>3. To compare impact of enhanced health facility-based care to current standard of care on key indicators of case management for malaria and other illnesses, including the risk of inappropriate antimalarial treatment, in children under five treated at health facilities.
Description	Enhanced health facility care will be compared to the current standard of care provided by lower level government-run health facilities, supplemented by services provided through the private sector and community-based interventions, using a cluster-randomized design. Clusters will be defined as households located within a 2 km radius of the facilities.
Participants and Sample Size	1. Objective 1. Cross-sectional surveys in 400 children (200 under-fives + 200 aged 5-15 years) randomly selected from households in each cluster; 4000 children per study arm; 8000 total. Surveys will be conducted at baseline and then annually for 2 years. New populations of children will be selected for each survey.<br>2. Objective 2. Cohort of children under five recruited from 25 randomly selected households in each cluster; 250 households per study arm; 500 total.<br>3. Objective 3. Interviews will be conducted with children under five and their caregivers visiting health facilities on days selected for the exit interviews, including 10 patients per health facility. Exit interviews will be conducted every 6 months, for a total of four surveys; 200 patients will be interviewed during each survey; 800 total.
Study site	Tororo district, an area with very high malaria transmission intensity. The five sub-counties of West Budama North Health Sub-district (Nagongera, Paya, Kirewa, Kisoko, and Petta), and two sub-counties of West Budama South Health Sub-district (Mulanda and Rubongi) will be included.
Study period	The total duration of the study will be approximately 2.5 years. The health facility intervention will be scaled-up over 2 months and will run for approximately 2 years; the cross-sectional surveys will be conducted at baseline and then annually for 2 years; and each of the cohort study participants will be followed for 2 years.
Intervention	20 lower-level government-run health facilities in the area will be randomly assigned to one of two interventions: (1) health facility intervention (HFI), or (2) standard care. The HFI will focus on improving health center management, information management, health worker training, and ensuring adequate diagnostics and drug supplies.
Primary outcome	1. Objective 1. Prevalence of anemia<br>2. Objective 2. Antimalarial treatment incidence density<br>3. Objective 3. Inappropriate treatment of malaria
Secondary outcomes	1. Objective 1. Prevalence of parasitemia, prevalence of gametocytemia, all-cause mortality rate in children under five<br>2. Objective 2. Incidence of hospitalizations, illness, and febrile illness episodes, prompt effective treatment of fever, prompt effective treatment of malaria, incidence of serious adverse events, antibiotic treatment incidence density<br>3. Objective 3. Appropriate treatment of malaria, patient satisfaction, patient attendance, gaps in staffing, drug stock outs, health worker knowledge questionnaire scores

PROJECT TEAM AND PARTICIPATING SITES

INVESTIGATORS

Sarah Staedke, MD, PhD
Role in project: Principal investigator
Clinical Senior Lecturer, London School of Hygiene and Tropical Medicine, London, UK
Infectious Disease Research Collaboration / Uganda Malaria Surveillance Project, Kampala, Uganda
Email: sarah.staedke@lshtm.ac.uk

Moses Kamya, MBChB, MPH, PhD
Role in project: Co-investigator
Professor, Department of Medicine, Makerere University, Kampala, Uganda
Infectious Disease Research Collaboration / Uganda Malaria Surveillance Project, Kampala, Uganda
Email: mkamya@infocom.co.ug

Grant Dorsey, MD, PhD
Role in project: Co-investigator
Associate Professor, Department of Medicine, University of California, San Francisco
Infectious Disease Research Collaboration / Uganda Malaria Surveillance Project, Kampala, Uganda
Email: gdorsey@medsfgh.ucsf.edu

Clare Chandler, PhD
Role in project: Co-investigator
Senior Lecturer, London School of Hygiene and Tropical Medicine, London, UK
Social Scientist, ACT Consortium
Email: clare.chandler@lshtm.ac.uk

COLLABORATORS

Philip Rosenthal, MD
Role in project: Collaborator
Professor, Department of Medicine, University of California, San Francisco, USA
Infectious Disease Research Collaboration / Uganda Malaria Surveillance Project, Kampala, Uganda
Email: prosenthal@medsfgh.ucsf.edu

Ambrose Talisuna, MBChB, MSc, PhD
Role in project: Collaborator
Medicines for Malaria Venture (MMV) Representative in Africa
Infectious Disease Research Collaboration / Uganda Malaria Surveillance Project, Kampala, Uganda
Email: talisunaa@mmv.org; atalisun@yahoo.com

Fred Wabwire-Mangen, MBChB, MPH, PhD
Role in project: Collaborator
Associate Professor, School of Public Health, Makerere University, Kampala, Uganda
Infectious Disease Research Collaboration / Uganda Malaria Surveillance Project, Kampala, Uganda
Email: fwabwire@musph.ac.ug

Heidi Hopkins, MD, MPH
Role in project: Collaborator
FIND Diagnostics, Kampala, Uganda
Infectious Disease Research Collaboration / Uganda Malaria Surveillance Project, Kampala, Uganda
Email: hhopkins@medsfgh.ucsf.edu; heidi.hopkins@finddiagnostics.org

KEY PROJECT PERSONNEL

Personnel	Institution	Role
Deborah DiLiberto	London School of Hygiene & Tropical Medicine	Research Assistant
Rita Kabuleta-Luswata	Infectious Diseases Research Collaboration, Kampala	Project Officer
Dr. Catherine Maiteki-Sebuguzi	Infectious Diseases Research Collaboration, Kampala	Kampala Study Coordinator
Dr. Florence Nankya	Infectious Diseases Research Collaboration, Kampala	Tororo Study Coordinator
Dr. Fred Mudangha	Infectious Diseases Research Collaboration, Kampala	Tororo Study Coordinator
Lilian Taaka	Infectious Diseases Research Collaboration, Kampala	Social Scientist
Simon Peter Kigozi	Infectious Diseases Research Collaboration, Kampala	Kampala Data Manager
Faith Mukoya	Infectious Diseases Research Collaboration, Kampala	Tororo Data Manager
Dr. Immo Kleinschmidt	London School of Hygiene & Tropical Medicine	Statistician
Dr. Emily Webb	London School of Hygiene & Tropical Medicine	Statistician
Dr. Fred Matovu	London School of Hygiene & Tropical Medicine	Economic Consultant

PARTICIPATING SITES

Uganda Malaria Surveillance Project (UMSP)

Address: Infectious Disease Research Collaboration (IDRC), Mulago Hospital Complex, Malaria House, P.O. Box 7475, Kampala, Uganda
Contact Person: Catherine Tugaineyo
Phone Number: +256 (0) 414-530692
Fax Number: +256 (0) 414-540524
Email: ctugaineyo@muucsf.com

London School of Hygiene & Tropical Medicine (LSHTM)

Address: Keppel Street, London, WC1E 7HT, UK
Contact Person: Susan Sheedy
Phone Number: +44 (0) 20 7927 2256
Fax Number: +44 (0)20 7637 4314
Email: susan.sheedy@lshtm.ac.uk

University of California, San Francisco (UCSF)

Address: San Francisco General Hospital, 1001 Potrero Avenue, Building 30, Room 402/408, San Francisco, CA 94110, USA
Contact Person: Peter Padilla
Phone Number: +1 (415) 206-5438
Fax Number: +1 (415) 648-8425
Email: ppadilla@medsfgh.ucsf.edu

ABBREVIATIONS AND ACRONYMS

ACT	artemisinin-based combination therapy
ADDAT	ACT drug distribution assessment tool
AL	artemether-lumefantrine
AS+AQ	artesunate + amodiaquine
CAB	community advisory board
CBI	community-based intervention
CHW	community health worker
CMD	community medicine distributor
CQ	chloroquine
CQI	continuous quality improvement
FGD	focus group discussion
GIS	geographical information systems
GPS	global positioning system
HBMF	home-based management of fever/malaria
HC	health center
HFI	health facility intervention
ICCM	integrated community case management
IMCI	integrated management of childhood illnesses
JMS	joint medical stores
JUMP	Joint Uganda Malaria Program
IRB	institutional review board
LSHTM	London School of Hygiene and Tropical Medicine
MoH	Ministry of Health
MU	Makerere University (Kampala, Uganda)
MU-UCSF	Makerere University - UCSF Malaria Research Collaboration
M&E	monitoring and evaluation
NMS	national medical stores
PCS	patient-centered services
RDT	rapid diagnostic test
SOP	standard operating procedure
SP	sulfadoxine-pyrimethamine
UCSF	University of California, San Francisco
UMSP	Uganda Malaria Surveillance Project
UNCST	Uganda National Council of Science and Technology
VHT	village health team
WHO	World Health Organization

1 BACKGROUND

1.1. INTRODUCTION

Malaria remains one of the most serious global health problems.[1] Of the estimated 400 to 900 million episodes of fever that occur each year in African children, probably about half are due to malaria, resulting in over one million deaths.[2-4] Uncomplicated malaria can progress rapidly to severe disease, and most malaria deaths in young children occur within 2-3 days of onset of illness.[5] The first few days of illness present an important window of opportunity to reduce morbidity and mortality with early treatment.

Early effective antimalarial treatment is one of the key strategies for reducing the burden of malaria.[6] In the Global Strategic Plan for 2005-2015, the Roll Back Malaria Partnership has set a target to ensure that 80% of malaria episodes are adequately treated within 24 hours of onset of symptoms.[6] Despite increasing availability of effective artemisinin-based combination therapies (ACTs) and the emphasis on early antimalarial treatment, many patients with malaria do not benefit from this therapy. A recent UNICEF report of data collected between 2000 and 2006 estimated that 35% of febrile children in sub-Saharan Africa were treated with antimalarial medicines.[7] However, only 23% of these children were treated within 24 hours of onset of illness, and 60% of febrile children received chloroquine, rather than effective first-line therapies.

1.2. MALARIA DIAGNOSIS AND TREATMENT

1.2.1. Barriers to service in the formal healthcare sector

Diagnosis and treatment of malaria is often challenged by limited health-care infrastructure, particularly in Africa.[8,9] Substantial barriers to providing good quality health care exist, including logistical, cultural, and wider system barriers. As a result, few malaria patients receive treatment in the formal healthcare sector; most are treated at home with drugs purchased from informal drug shops.[2,10] Unfortunately, such treatment is often inadequate, with ineffective or poor quality drugs given at incorrect doses.[10-12] Community-based programs, such as home management of malaria (HMM), have been promoted by the World Health Organization (WHO) and others to extend care beyond the formal sector, and have been adopted in many African countries.[13-15]

1.2.2. Universal diagnostic testing

The WHO has recently released new guidelines for malaria treatment, recommending that suspected cases be confirmed by a parasitological test when possible.[16] Specifically, the WHO guidelines state that prompt parasitological confirmation by microscopy or alternatively by rapid diagnostic tests (RDTs) is recommended in all patients suspected of malaria before treatment is started; and that treatment solely on the basis of clinical suspicion should only be considered when a parasitological diagnosis is not accessible. The shift towards universal diagnostic testing for malaria is

very welcomed, but raises serious questions about the capacity for diagnosing malaria in endemic areas.

Currently, malaria diagnostics are generally only available in the formal healthcare sector, where a minority of antimalarial treatments are distributed. Even where diagnostic facilities are available, studies suggest that antimalarial treatment is given to at least half of patients with a negative test.[17-20] RDTs for malaria offer promise for extending diagnosis beyond hospitals and clinics, reaching the areas where many patients seek treatment. However, deployment of RDTs not straightforward, and introducing them into the periphery has several operational challenges. When RDTs have been introduced into settings where microscopy is available, studies suggest that providers often ignore negative results, misdiagnosing and overtreating malaria.[19,20]

1.2.3. Training health workers in malaria diagnosis

Limited training, supervision, and support for the shift from presumptive to diagnostic-driven treatment may be associated with poor provider adherence to test results. More recently, encouraging results have been seen in Uganda where a one-week integrated course for health workers at facilities with microscopy significantly decreased unnecessary ACT prescriptions.[21] Also in Uganda, data from a study evaluating an RDT training course targeted to health workers in lower-level public health centers, which included clear guidelines on management of positive and negative RDT results, dramatically decreased unnecessary antimalarial prescriptions while maintaining satisfactory patient outcomes [H Hopkins, unpublished data]. However, it remains to be seen whether these more intensive training programs can be taken to scale. One of the main challenges for the introduction of malaria diagnostics has been to concurrently enhance capacity to diagnose and treat alternative causes of fever.[22] The need to integrate training in case management of malaria with that of non-malaria febrile illnesses, and evidence that more supportive training packages increase effectiveness,[23-25] demonstrates the importance of integrating malaria programs with health services in general. A more comprehensive approach to health care that will attract more patients and manage multiple diseases effectively is called for to produce tangible health benefits in the population as a whole.

1.3. MALARIA IN UGANDA

1.3.1. Overview

Malaria is one of the most important health problems in Uganda and the leading cause of morbidity and mortality in children, accounting for up to 40% of outpatient visits, 20% of hospital admissions, and 14% of inpatient deaths.[26] Children in Uganda experience an estimated average of six episodes of malaria each year, resulting in between 70,000 and 110,000 deaths annually. Up to 90% of Uganda's population lives in highly endemic areas with perennial malaria transmission, while 10% live in areas at risk for epidemics.[27] The Ugandan Ministry of Health has developed a strategic plan for malaria control which focuses on intermittent presumptive treatment of pregnant women, vector control measures (including use of insecticide-treated bednets and indoor residual spraying), improved epidemic preparedness and response, and improved case management of malaria in health facilities and at home.[27] Access to care remains a major challenge in Uganda; only 49% of households live within a 5km radius of a public health facility.[28], and febrile children are frequently

treated outside of the formal sector.[29-32] Additional barriers to appropriate health care in Uganda include high costs, poor attitude of health workers, and stock-outs of drugs.[33]

1.3.2. Antimalarial drug policy

Artemether-lumefantrine (AL) was adopted as the new first-line treatment for uncomplicated malaria in 2004, with artesunate + amodiaquine (AS+AQ) as an alternative if AL was not available. Studies suggest that AL is highly effective for treating uncomplicated malaria in Uganda, with risk of recrudescence of 1% in Kampala and Tororo;[34-36] however, stock-outs of AL remain an issue.[33,37,38] A severe shortage of AL in Uganda has markedly limited the amount of drug available in the country.[39] Many public health facilities have been left without AL for months, and health care workers have been forced to resort to ineffective or inappropriate drugs for treatment of malaria.

In 2002, Uganda launched the national home-based management of fever (HBMF) program to address the challenge of poor access to antimalarial treatment, and to extend delivery of effective antimalarial drugs beyond the formal sector.[40] The aim of Uganda's HBMF program was to improve access to drugs and to treat all fevers in children under five within 24 hours of onset of symptoms by distributing antimalarials free of cost via volunteer community medicine distributors (CMDs). The program was scaled-up to country-wide coverage over approximately three years;[41] however, it was fully functional in only a few areas. Policy on community-based interventions in Uganda has recently changed expanding from malaria-only to integrated community case management (ICCM). Community health workers (CHWs), participating in village health teams (VHTs) will be trained to evaluate and provide presumptive treatment of malaria, pneumonia, and diarrhea based on clinical criteria. Community-based health care in Uganda is currently in transition; the HBMF program is being phased out and is inactive in most areas, but ICCM has not yet been rolled out. The anticipated launch date of the new ICCM policy is July 2010.

1.3.3. Affordable Medicines Facility – malaria

Another program that may increase access to antimalarials in Uganda is the Affordable Medicines Facility – malaria (AMFm), which is a new financing mechanism, intended to increase access to ACTs and reduce access to less effective antimalarial treatments, particularly artemisinin monotherapies (http://www.theglobalfund.org/en/amfm/). The AMFm is hosted by the Global Fund and is being piloted in 9 countries, beginning in June 2010, dependent upon agreements being signed between relevant parties. The mechanism of the AMFm is to make a co-payment towards the cost of ACTs with eligible first-line buyers in the public, private or not-for-profit sectors. This subsidy is intended to result in lower costs of ACTs being distributed through the different sectors. Alongside the subsidy, countries are required to implement 'supporting interventions' such as training providers and outreach to communities in order to improve malaria case management and to promote ACT use. Each country is responsible for the design and implementation of supporting interventions as well as for monitoring and evaluation activities over the period of the first phase of the pilot, lasting two years. Uganda's application to be a pilot AMFm country was accepted in November 2009 and the agreement for the contract with the Global Fund is in progress.

1.4. HEALTH FACILITY INTERVENTIONS

1.4.1. What is quality care and what are the problems?

The Institute of Medicine definition of quality of care incorporates six aims, that it be safe, effective, patient-centered, timely, efficient and equitable.[42] Evidence from increasing numbers of studies suggests quality of care by these measures is poor in many settings, including delivery of primary care in low-income countries. In terms of effectiveness and safety, direct observation studies of performance have identified severe deficiencies, particularly in history taking and examinations, diagnosis, and appropriate treatment choice and dosage.[43-48] This has been linked to low motivation of staff as well as poor resource availability in the work place. In terms of patient-centeredness and timeliness, meeting a population's expectations of how they should be treated by providers, including patient expectations for health care, is now seen as central to performance.[49] It has been argued that poor quality services fail to earn the population's trust, leading to clients seeking alternative sources of care,[50] or discontinuing care.[51] In contrast, the perception of good quality services, including inter-personal relationships, has been found to encourage patients to access care,[52] and demand for services.[53-55] Satisfied patients may be more likely to comply with treatment and maintain a continuing relationship with the health worker,[56] and loyalty to a clinic.[57], thus enjoying a better medical prognosis (presuming good technical quality of care).[58]

1.4.2. How can quality of care be improved?

Interventions to improve quality of care in low-resource settings have largely fallen into two categories: resource-based interventions and performance-based interventions. Resource-based interventions include the provision of equipment, infrastructure and drugs. Performance-based interventions have mostly been focused on clinical training and dissemination of guidelines. Far fewer studies have assessed interventions to improve aspects of quality care outside of clinical care.

1.4.2.1. Resource-based interventions

Resource-based interventions have shown that improved supplies together with guideline training can lead to improvements in quality of care,[59] and these improvements can be effective when solutions to resource needs are driven by local actors, for example using participatory research methods.[60] When resources, such as equipment, are delivered in a top-down fashion according to perceived needs, they may not be appropriate and may not be used.[61] In addition, resources such as RDTs for malaria need to be introduced in an appropriate manner, including generating local ownership and preparedness for change and providing troubleshooting and support in the longer term.[62] Further research to identify principle components for introducing equipment and supplies to primary health centers is needed to inform implementation across low-resource countries.

1.4.2.2. Performance-based interventions

Systematic reviews of evaluations of training-based interventions to change clinical behavior have produced mixed results in both developed,[63-67] and developing countries.[68] A training intervention to improve medical assistants' malaria prescribing practices in Ghana found initial improvements in practice deteriorated after twelve months, attributing the gap between knowledge and practice to socio-cultural factors including patient pressure, self-interested motivations and a

lack of supervision.[69] An evaluation of training for CHWs in Kenya found no improvement in overall process quality during consultations with pediatric patients after increasing numbers of refresher training series attended.[70] In fact, longitudinal analysis of this intervention showed that whilst initial refresher training led to improved management for severely ill children, adherence to guidelines for non-severe cases worsened, and after second refresher training the overall adherence declined rapidly.[71]

1.4.2.3. Why does training fail?

The failure of many training based programs to improve clinical care reflects a wider acknowledgement of a gap between knowledge and practice of health workers. Interpretation of the literature suggests that training can form the basis of effective strategies but only as long as other conditions are fulfilled. For example, the introduction of the integrated management of childhood illnesses (IMCI) scheme to primary health care facilities has had better results in countries with stronger health systems, and where support for the scheme has been strong, in terms of political advocacy, trainer support, and integration into current practices.[72-77] The importance of management is echoed in findings from Mexico where interactive education was combined with managerial interventions to improve prescribing practices for rhinopharyngitis at 18 primary health centers.[78] Similarly, in Kenya, treatment of uncomplicated malaria was better at facilities where health workers had undertaken in-service malaria training and where guidelines, wall charts, and more frequent supervision were provided.[79] However, evaluation of supervision and job aids for CHWs in Kenya found that these interventions did not improve adherence to guidelines for pediatric case management.[70] The authors suggest this may have been due to the quality of these interventions: relatively little time was spent on supervising clinical practice (co-examination of children by supervisor and health worker) compared to administrative tasks; quality of feedback from the supervisors may have been poor; and guidelines provided in job aides may not have been clear. In spite of the emphasis on training, clinical quality of care remains poor in many low-resource settings. Further research into how to support clinicians to improve performance, beyond didactic training, is urgently needed.

1.4.2.4. Patient-centeredness

Performance-based interventions tend to favor traditional continuing medical education training programs and are less focused on patient-centeredness. However, there is some evidence that participatory workshop interventions conducted over an extended period can improve communication with patients in different settings.[80] Ensuring that interventional methods used are appropriate to the local setting, and that participants are able to tackle wider issues that affect their ability to communicate effectively, have been shown to be important.[81,82] In high-resource settings, interventions targeted at provider communication have shown some success in targeting three aspects of the interaction: giving time to talk,[83] providing emotional care,[84,85] and giving positive communication.[86,87] Intervention methods to tackle these specific issues in patient-centeredness are based on adult learning theory, following principles of learning through experience and reflection. Self-observation, or 'mindfulness meditation' has formed an important part of some effective programs.[88] Further research is needed to identify intervention components that successfully achieve improved levels of patient-centeredness in a sustainable way that are replicable in low-resource settings.

1.5. PHASE I RESULTS

In the first phase of this project, we aimed to characterize the population and local health services in Tororo district by conducting a census survey, a survey of health services, and a qualitative study. All households within West Budama North health sub-district in Tororo, including Nagongera, Paya, Kirewa, Kisoko, and Petta sub-counties, were enumerated and mapped to provide a sampling frame for the main trial. To characterize the population, households were surveyed on basic demographic information, markers of socioeconomic status, vital statistics, and bednet practices. We also characterized the local health services, focusing on the public health facilities and the existing HBMF program, by surveying health care workers and community medicine distributors. In addition, we conducted a series of focus group discussions with primary caregivers, heads of households, and health care workers.

The initial field work was conducted from October 2009 to February 2010. Our census team consisted of surveyors paired with local research assistants who moved with the local leaders of each village to identify all households. The health services survey and qualitative study were conducted by a team of interviewers trained in social science and qualitative research.

Additional Phase I work, including (1) a census survey of two sub-counties in West Budama South health sub-district, (2) a brief survey of lower-level government-run health facilities in that area to evaluate their catchment areas, and (3) self-observational activities for health workers to increase their awareness about barriers to communicating with patients, will be conducted in August and September 2010.

1.5.1. Census survey

We enumerated 26,793 households in five sub-counties in Tororo district, including 144,216 residents and 26,905 children under five. Our results suggest that this area of Tororo is very rural, with limited infrastructure and education. Very few households have electricity (1%), or own a television (2%). Ownership of mobile phones (31%) and radios (43%) is also low. One-quarter of households have no toilet facilities, and only 30 households have a flush toilet. Overall, heads of household are not well-educated; one-quarter have received no formal education, and only 21% have received any secondary or higher education. Mortality in children under five was estimated to be 10.99%.

Although over half (63%) of households reported that they owned a bednet, only 37% of residents reported that they had slept under a bednet the previous night. Only 15% of residents and 20% of children under five slept under an insecticide-treated bednet (ITN) during the previous night, which is far below the targets of > 80%.

1.5.2. Public health facilities

We interviewed 81 (88%) of 92 health workers stationed at the 17 functional government-run health facilities in West Budama North. Most health workers (56 [69%]) were trained in management of malaria with AL, but only 29 (26%) had received training in RDTs for malaria.

Staffing shortages and absenteeism are a problem at most health centers; 16 (94%) reported that the number of staff working at the facility was insufficient. Drug stock-outs are also a major problem, particularly at HC IIs and HC IIIs. Nearly all health centers (94%) reported that they experienced

stock-outs. Only 29% of health centers reported that the supply of antimalarial drugs was adequate for treating their patients. Infrastructure at the health facilities in this area is also limited. Most lack electricity (88%) and running water (94%).

Overall, the knowledge of health workers about malaria case management was surprisingly poor. Out of a possible 178 points, the mean score achieved by health workers on a knowledge questionnaire was 51.6 (29%), ranging from 15 to 110. The in-charges of health centers scored unexpectedly low with a mean score of 60.5 (34%). When asked how to confirm the diagnosis of malaria, only eight (10%) health workers mentioned microscopy and two (2%) RDTs.

The results of knowledge questionnaire identified areas to target in training include physical examination skills, identification of danger signs for severe malaria, malaria diagnostics, differential diagnosis of non-malarial febrile illnesses, and key elements of managing uncomplicated and severe malaria (including administration of medications).

1.5.3. Community medicine distributors

We selected 100 community medicine distributors (CMDs) who had participated in the HBMF program using convenience sampling from five sub-counties in Tororo district. Major gaps in CMD training, supervision, and knowledge were identified. Interviews also revealed that CMDs are involved in implementing multiple programs led by different stakeholders, which are not integrated. Only four CMDs reported receiving support supervision in the last six months. Most CMDs (92%) said that they refer at least one patient each week, most commonly to a local HCIII. However, only 23% of CMDs said that they would refer the child after two days if no improvement was seen, suggesting that they refer sick children on initial review, but do not provide follow-up of patients.

Overall, CMDs scored poorly on the knowledge questionnaire (mean score 22%). Only 74% CMDs correctly identified fever as the most common symptom of malaria in children, and recognition of danger signs of severe malaria was poor. Although 61% of CMDs had received training on management of malaria with AL, few CMDs correctly described how AL should be administered. Recognition of non-malarial causes of fever in children was also poor.

Our results suggest that CMDs knowledge of appropriate management of malaria is limited, despite training, and they may be overstretched by stakeholders attempting to deliver community-based interventions.

1.5.4. Qualitative study

We conducted 69 in-depth interviews with health workers stationed at the 17 health facilities. In-depth interviews were conducted with 100 CMDs. A total of six FGDs, involving 65 participants, were conducted with health workers, representing all three health center levels (HC II, III, and IV) and higher and lower cadres of health workers. Five FGDs were conducted with 55 primary caregivers from the community, including two with caregivers under the age of 30 years, and three with older caregivers. Participants from four of these FGDs lived in parishes that had a public health facility. Five FGDs were conducted with 58 heads of household from the community, including two with female heads, and three with males. Participants from four of these FGDs lived in parishes that did not have a public health facility.

1.5.4.1. Defining good quality health care

When asked, 'what is good quality health care', community members, health workers and CMDs described similar values involving three themes: 1) comprehensive therapeutic process which describes the clinical treatment given to patients and the relationship between patient and health worker; 2) management of health facilities which describes operational components including staffing and facilities as well as availability of drugs and equipment, and 3) expectations of responsiveness which describes the provision of prompt and free services.

1.5.4.2. Attendance at public health centers

Community members report various and complex treatment seeking behaviors and outcomes. Most community members have visited their local health facility, but were dissatisfied with their experience. Barriers faced by patients in getting to health facilities were identified. In addition, we found that health facilities are not attractive to community members. Patients cannot get to health facilities because of logistical and cultural barriers, and are not attracted to health facilities because of poor management, poor interpersonal relationships with health workers, and local system failures.

1.5.4.3. Quality of care provided at health centers

We found that aspirations for good quality care were similar amongst health workers, CMDs, and community members. The most frequently discussed values include those involving the comprehensive therapeutic process (good clinical care and treatment, welcome and orientation, good interpersonal interactions between health workers and patients, and advice), responsiveness of health workers and the public health system (prompt and fair treatment, treatment free-of-charge), and management of health facilities (adequate staff, equipment, and infrastructure, availability of drugs, professional health workers).

At health centers, immediate barriers to quality care included drug stock-outs and lack of equipment; high patient to staff ratio; use of volunteer health workers; language barrier between health workers and patients and discriminatory treatment of patients. Underlying these barriers were poor motivation of staff; poor management of the health center; lack of patient-centerd culture and poor relationship between health workers and communities.

1.5.4.4. Treatment seeking behavior

Treatment seeking behavior of community members is largely driven by perceptions and understanding of illnesses and practical concerns, including accessibility, available resources, and prior experiences. In this area, first-line treatment for most conditions was with a biomedical drug. Nearly all community members had visited their local health center, but dissatisfaction with care was high. Other sources of health care include CMDs (when operational), private clinics, and drug shops. Herbal medicine was frequently used and interestingly, community members also relied heavily on shrines, churches and prayers for treatment.

Choice of health care was influenced by the following factors: (1) initial perceptions and beliefs about etiology and severity of the illness that would, from experience, require a particular source of treatment. Often, experience showed health centers to be a poorer source of care than other providers for common illnesses; (2) accessibility of the preferred treatment, which relied on distance to the provider as well as opening hours, spousal support in meeting costs, opportunity costs of

leaving the home and travelling to the provider, ability to negotiate the logistical and social rules of the provider's institution, and availability of treatment at that provider; and (3) trial and error in moving between treatment sources.

2 RATIONALE

2.1. PROBLEM STATEMENT

The current approach to management of malaria and febrile illnesses in Ugandan children is inadequate. Given the barriers to accessing good quality care through the formal heathcare sector, substantial attention and resources have been focused on developing community-based interventions to deliver antimalarial treatment and comprehensive care.[13,14,89] However, whether resources should be put into community-based programs or into improving the public health system is not clear; malaria case management could be strengthened by improving the quality and delivery of care in existing government-run health facilities. Our study is designed to assess whether an intervention to build capacity and improve delivery of drugs and diagnostics at government-run health facilities improves the health of children and quality of care delivered, as compared to 'standard care' currently available at health facilities, supplemented by services provided through the private sector and community-based interventions.

2.2. STUDY OVERVIEW

We are proposing to evaluate enhanced health facility-based care for malaria and febrile illnesses in children in Tororo district using a cluster randomized design. The health facility intervention (HFI) will aim to address barriers to achieving good quality health care that were identified in our formative research. A focus of our intervention will be providing RDTs and training health workers in management of both malaria and non-malarial febrile illnesses. We aim to implement an intervention which is sustainable and reproducible by the MoH in Uganda, working within the existing government systems in conjunction with the MoH and district teams.

Clusters, defined as health facilities and their catchment areas, will be randomized to the HFI or to standard care delivered from government-run health facilities, supplemented by services provided through the private sector and community-based interventions. Outcomes will be measured in three distinct populations: (1) cross-sectional surveys of children under 15 years randomly selected from households within the clusters; (2) a cohort of children under five randomly selected from households within the clusters and followed for 2 years; and (3) patients attending all government-run health facilities, including children under five and their caregivers participating in exit interviews on selected days every six months.

The primary outcome of the study is prevalence of anemia in children under five. We will test the primary hypothesis that the prevalence of anemia in children under five from clusters randomized to the HFI will be lower than in children randomized to the current standard of care. The study proposed here will benefit greatly from our formative research, prior longitudinal studies of antimalarial therapy conducted in Kampala, including two cohort studies evaluating health facility-based care,[34,90,91] and one cohort study evaluating HBMF, [92] and ongoing surveillance conducted by the Uganda Malaria Surveillance Project. The prior and ongoing studies are expected to inform the design and conduct of the planned research.

2.3. STUDY POPULATION AND DESIGN

In the cross-sectional surveys and cohort study, we plan to focus on children under five as young children bear the greatest burden of malaria in this endemic population. However, in our cross-sectional surveys we will also evaluate children aged 5-15 years, as this group contributes substantially to malaria transmission.

We have opted for a cluster randomized design rather than a non-randomized approach, in which the HFI would be implemented in all facilities, to minimize confounding. It is likely that other health-related interventions will be implemented in Tororo district by the MoH or other stakeholders, such as bednet distribution or safe water campaigns, which could have an impact on the health of children in the study area. If we relied on comparisons between measurements taken before and after implementation of the HFI, we could risk falsely attributing improvements to our intervention, when in fact the changes were due to factors outside of our study.

We have chosen the health facility as the unit of randomization, defining the clusters as catchment areas of these facilities, including households within a 2km radius of the health center. Only children from households within the clusters will be eligible for participation in the cross-sectional surveys and cohort study. We have opted to restrict the study population to children from villages near to facilities rather than including the full population of the study area to minimize risk of contamination between the study arms. We recognize that distance to the health facility is a major factor influencing utilization, which we will not be able to change with our intervention. Children from villages in the periphery of the health facility catchment areas may or may not be able to access services because of distance. If children who could not access the health facilities were included in the study population, we could risk underestimating the impact of the intervention, resulting in a Type II error in which we fail to identify a real difference between our HFI and standard care.

We also recognize the possibility of contamination in the other direction; primary caregivers of children in clusters randomized to standard care may seek care from facilities randomized to the HFI. Again, distance to the health center is likely to influence the likelihood of seeking care. By restricting the study population to children residing in households within a 2km radius of the health facility, we hope to focus our evaluation on children who are most likely to receive care from that facility. In addition, we plan to capture information on treatment seeking behavior during the cross-sectional surveys, and information on patient attendance and village of residence from all health facilities, to track patterns in utilization of services from various sources. We recognize that restricting the study population to households nearby the health facilities will limit our ability to generalize our results to more remote populations without access to health facilities. However, we plan to evaluate location of residence and distance to health facilities as a covariate in our analysis. In addition, when assessing outcomes for the cross-sectional survey and cohort study, analyses will be conducted primarily on an intention-to-treat basis, where data collected will be analyzed according to the assigned cluster. To address potential contamination issues due to children attending health facilities other than the one that defines their cluster, i.e. the health facility they live closest to, we shall also analyse data on a per-protocol basis. For this analysis, data collected will be analysed according to the facility at which the child accesses healthcare.

3 STUDY OBJECTIVES

1. To compare the impact of enhanced health facility-based care to current standard of care on key population-based indicators, including the prevalence of anemia in children under five, using a cluster randomized design. We will test the primary hypothesis that the prevalence of anemia will be lower in children under five from clusters randomized to the health facility intervention (HFI) than in children randomized to standard care. Children receiving care from the health facilities participating in the HFI may be more likely to receive good quality health care and appropriate treatment for malaria and non-malaria illnesses, including anemia and helminth infections. We will also test the secondary hypothesis that the prevalence of parasitemia in children under five, and children aged 5-15 years, from clusters that are randomized to the HFI will be lower than in children randomized to standard care.

2. To compare the impact of enhanced health facility-based care to current standard of care on key longitudinal indicators, including treatment incidence density, in a prospectively followed cohort of children under five. We will test the hypothesis that delivery of antimalarial treatment via current care will result in over-treatment and a higher incidence of antimalarial treatment, than antimalarial treatment delivered from health facilities randomized to the HFI, which will be targeted to lab-confirmed cases of malaria.

3. To compare impact of enhanced health facility-based care to current standard of care on key indicators of case management for malaria and other illnesses, including the risk of inappropriate antimalarial treatment, in children under five treated at health facilities. We will test the hypothesis that the health facility intervention decreases inappropriate treatment with ACTs, as measured by the proportion of children under five with suspected malaria and a negative RDT result that are inappropriately treated with an ACT plus the proportion of children under five with suspected malaria and a positive RDT result that are not prescribed an ACT, which we expect to be lower in the facilities randomized to the intervention than in those in the standard care group.

4 STUDY DESIGN

4.1. OVERVIEW

We propose to compare enhanced health facility care to the current standard of care using a cluster-randomized design in Tororo, Uganda. A census survey of the study area has been conducted to enumerate and map households, which will be used to create the sampling frame for the trial. The lower-level government-run health facilities (20 HC IIs and IIIs) in the area will be randomly assigned to one of two interventions: (1) health facility intervention (HFI), or (2) standard care. The HFI will focus on four components: (1) improving health center management, (2) improving information management, (3) providing health worker training, and (4) ensuring adequate malaria diagnostics and drug supplies. Standard care will include services provided by government-run facilities that are not randomized to the HFI. In addition, services provided by the private sector and through community-based interventions (CBI) implemented by the Ministry of Health and other partners will contribute to the health care provided in all areas. Currently, CBIs in Uganda are transitioning from home-based management of fever (HBMF) to village health teams (VHTs) delivering integrated community case management (ICCM). In Tororo district, CBIs are currently inactive but will likely be reactivated and scaled-up in Tororo district in a phased approach during the study period as follows: phase A: no CBI; phase B: malaria-only intervention; and phase C: VHT/ICCM.

Figure 1. Overview of study design

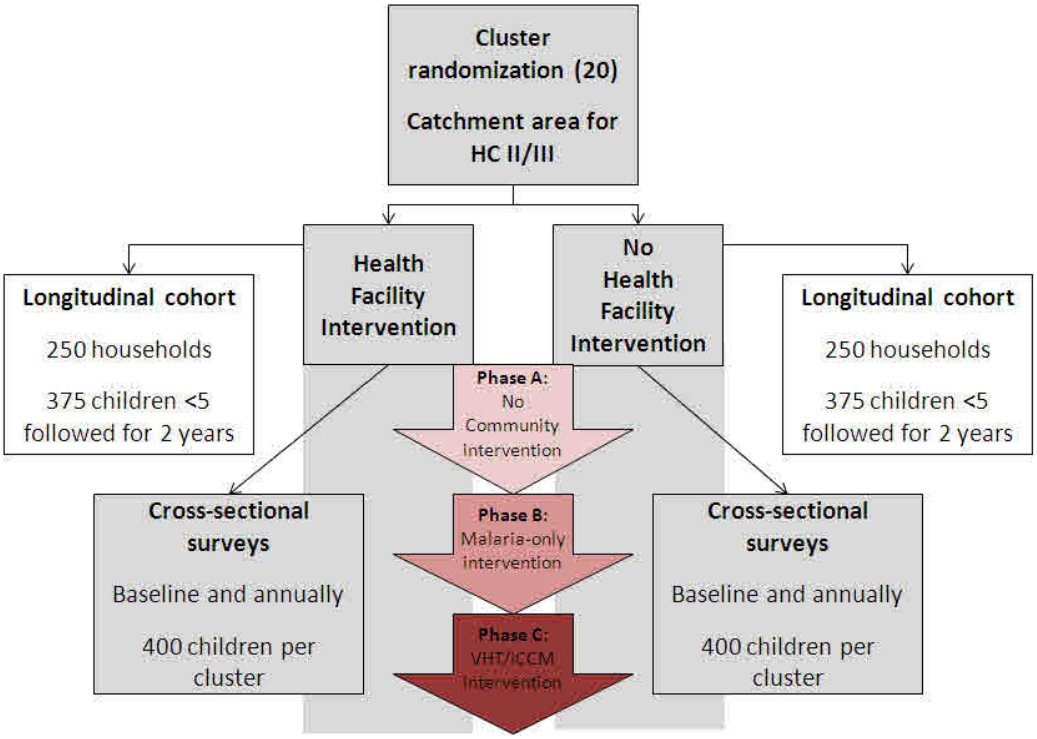

Information on changes in CBI policy and activity in the study area will be captured. In addition, AMFm may be scaled up in Uganda during the period of the study, which could include supporting interventions to train drug shop workers in the private sector and community awareness programs around ACTs. If AMFm is adopted in Uganda, these activities will be tracked as well.

Cross-sectional surveys will be conducted at baseline in 400 randomly selected children per cluster (8000 total per survey), and will be repeated annually. A sub-cohort of children will be recruited from 25 households randomly selected per cluster (500 total households) at the start of the intervention, and will closely followed for 2 years. All health facilities in the area will be assessed to monitor and evaluate the impact of the intervention; patient exit interviews will be conducted every 6 months in 10 patients from each health facility (200 total per survey).

4.2. STUDY SITE

The study will be conducted in Tororo district, an area with very high malaria transmission intensity The estimated entomologic inoculation rate (EIR) in Tororo is 562 infective bites per person-year, and the prevalence of parasitemia among children aged 5-9 years is 63.5%.[93,94] The five sub-counties of West Budama North Health Sub-district (Nagongera, Paya, Kirewa, Kisoko, and Petta), and two sub-counties of West Budama South Health Sub-district (Mulanda and Rubongi) will be included in the study population (Figure 2).

Figure 2. Study area

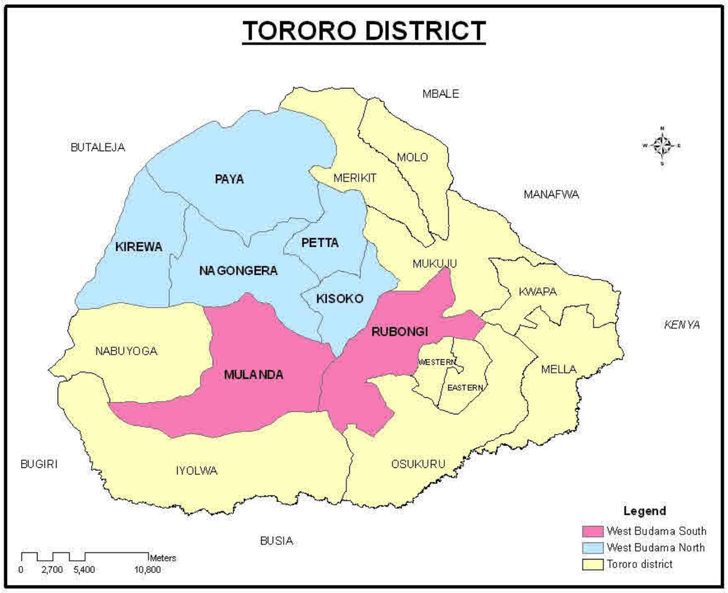

The results of our formative research suggest that this area is very rural, with limited infrastructure and education. Very few households have electricity (1%) and one-quarter have no toilet facilities. One-quarter of the heads of household have received no formal education, and only 21% have received any secondary or higher education.

4.3. STUDY POPULATION

Within the seven sub-counties, there are 22 lower-level government run health facilities, including 17 level II health centers (HC), and 5 level III HCs; 20 will be included in the randomization scheme. Clusters will be defined as the catchment areas of the health centers, including households that are located within a 2 km radius of the facilities. Only households located within the clusters will be included in the sampling frame for the cross-sectional surveys and the cohort study. The study population for each objective is listed in Table 1.

Table 1. Study objectives and populations

Objective	Study population and sample size
1. To compare the impact of enhanced health facility-based care to current standard of care on key population-based indicators, including the prevalence of anemia in children under five.	Cross-sectional surveys in 400 children (200 under-fives + 200 aged 5-15 years) randomly selected from households in each cluster (8000 children total); surveys will be conducted at baseline and then annually for 2 years (3 surveys in total)
2. To compare the impact of enhanced health facility-based care to current standard of care on key longitudinal indicators, including antimalarial treatment incidence density, in a cohort of children under five.	Cohort of children under five recruited from 25 households randomly selected from each cluster (500 total) and followed for two years; all children of appropriate age from each household will be eligible to participate
3. To compare impact of enhanced health facility-based care to current standard of care on key indicators of case management for malaria and other illnesses, including the risk of inappropriate antimalarial treatment, in children under five treated at health facilities.	Patients attending lower-level government-run health facilities (20 HC IIs and IIIs) in the study area, including 10 patients per health facility sampled in the exit interviews every six months (200 patients per survey, 4 surveys total)

4.4. CLUSTER RANDOMISATION

The lower-level government-run health facilities in the study area will be the unit of randomization. Clusters will be defined as the catchment areas of the health centers, including households located within a 2km radius of the facilities (Figure 3). The clusters will be defined prior to randomization using the full census survey database. All households and health facilities in West Budama North have already been mapped. The census survey and mapping of the two sub-counties in West Budama South (Mulanda and Rubongi) will be conducted in August and September 2010. The distances between every household and every lower-level government-run health facility in the area will be calculated. Households will be excluded from our sampling frame if they are $\geq$ 2km from any health facility. If a household is within 2km of a single health facility, the household will be considered to be within its catchment area and will be assigned to the cluster of that health facility. If a household is within 2km of more than one facility, the household will be assigned to the cluster of the closest health facility.

A total of 22 facilities are currently active in the area, however, 2 pairs of health centers are within close proximity (Soni HC II and Kirewa Chawolo HC II; Pokongo HC II and Morkiswa HC II) and have

substantially overlapping catchment areas. Given this, one facility from each pair will be randomly excluded from the randomization scheme.

Figure 3. Map of health facilities and clusters in West Budama North (mapping completed) and West Budama South (mapping in progress)

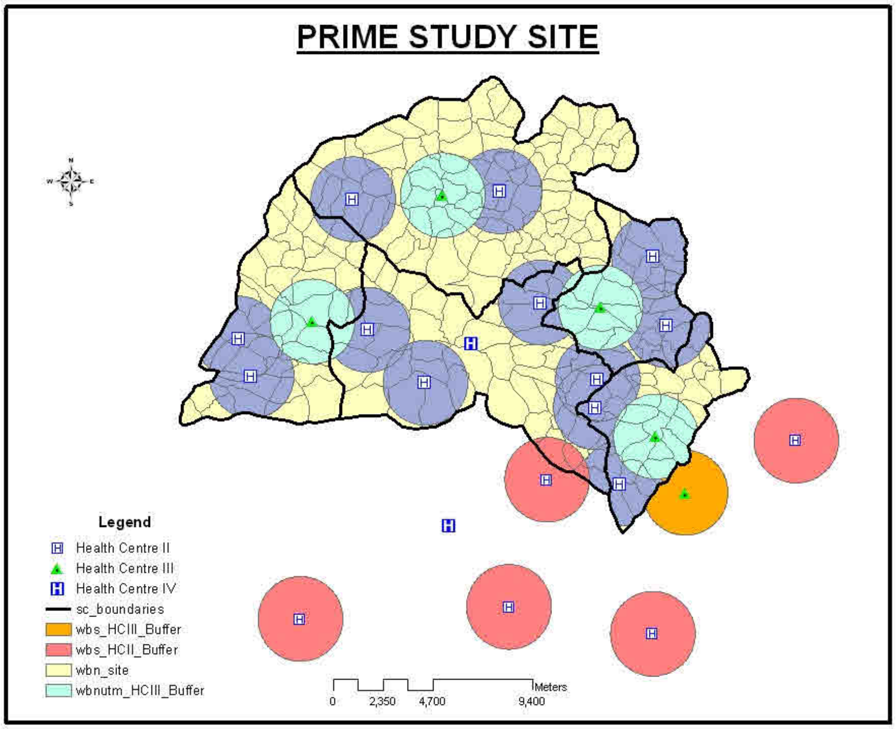

The randomization will be conducted by an investigator who is not directly involved in the project. Health facilities will be stratified by level (HC IIs and HC IIIs). Because of the uneven numbers of HC IIs and IIIs, one of the HC IIIs without a laboratory will be 'demoted' and paired with a HC II to ensure even numbers. Restricted randomization will be employed to ensure balance on geographical location. Specifically, restrictions will be applied that exclude the allocation of all clusters originating from a single sub-county, or that are otherwise in close geographical proximity from being allocated to the same arm of the trial.

4.5. OUTCOME MEASURES

The primary and secondary outcome measures for each study objective are listed below in Table 2.

Table 2. Study objectives and outcome measures

Outcomes	Indicator
Objective 1: Cross-sectional surveys	
<u>Prevalence of anemia</u>	Proportion of Hb measurements < 11.0 g/dL. Anemia will be classified according to severity: mild (Hb 8.0 – 10.9), moderate (Hb 5.0 – 7.9), severe (Hb < 5.0).
Prevalence of parasitemia	Proportion of thick blood smears that are positive for asexual parasites
Prevalence of gametocytemia	Proportion of thick blood smears that are positive for gametocytes
All-cause mortality	Probability of dying between birth and five years of age, expressed per 1,000 live births
Objective 2: Cohort study	
<u>Antimalarial treatment incidence density</u>	Number of antimalarial treatments given for fever/malaria over the period of follow-up
Incidence of hospitalizations	Overnight admission to a hospital or clinic
Incidence of illness episodes	Episode of illness as reported by primary caregiver
Incidence of febrile episodes	Episode of illness associated with fever as reported by primary caregiver
Prompt effective treatment of fever	Proportion of children with fever treated within 24 hours of onset of symptoms with an ACT
Prompt effective treatment of malaria	Proportion of children with malaria (confirmed by a parasitological test) treated within 24 hours of onset of symptoms with an ACT
Incidence of serious adverse events	Any experience that results in death, life-threatening experience, hospitalization, persistent or significant disability or incapacity, or specific medical or surgical intervention to prevent one of the other serious outcomes
Antibiotic treatment incidence density	Number of antibiotic treatments given for fever/bacterial illnesses over the period of follow-up
Objective 3: Health facilities	
Patient exit interviews conducted every 6 months	
<u>Inappropriate treatment of malaria</u>	Proportion of children under five with suspected malaria and a negative RDT result who are inappropriately given an ACT + Proportion of children under five with suspected malaria and a positive RDT result who are not prescribed an ACT
Appropriate treatment of malaria	Proportion of children under five with suspected malaria and a positive RDT result who are appropriately given an ACT + Proportion of children under five with suspected malaria and a negative RDT result who are not prescribed an ACT
Inappropriate treatment of malaria	Proportion of children under five with suspected malaria and a positive RDT result who are inappropriately given a non-ACT regimen
Patient satisfaction with health care	Proportion of patients indicating they were satisfied with care provided at the health center in exit interviews
Health facility surveillance conducted every 2 months	
Patient attendance	Total number of patients attending health facilities and their characteristics, including age, sex, village of residence, and diagnosis
Gaps in staffing requirements	Required positions, as indicated by the MoH staffing norms policy, which are unfilled for greater than one month
Stock-outs of ACTs	Days per month that AL supplied by NMS via the district is not available
Health worker knowledge questionnaires conducted annually	
Knowledge questionnaire scores	Proportion of questions answered correctly following training in fever case management

5 HEALTH FACILITY INTERVENTION

5.1. OVERVIEW

The health facility intervention (HFI) will be comprised of four components: 1) health center management training, 2) information management and continuous quality improvement (CQI); 3) health worker training, and 4) supply of consumables, including malaria diagnostics and antimalarial drugs. The goal of these components is to address the barriers to providing good quality care identified in our formative research (Appendix A). By addressing these barriers, we aim to provide good quality care as defined by health workers and community members in Tororo district, attracting them to health facilities and improving the case management of malaria and non-malarial febrile illnesses received when they attend facilities. The intervention package will be rolled out to all health centers randomized to the HFI over approximately 8-10 weeks (Table 3). Some activities will continue to be supported by the project for the duration of the study. We aim to implement an intervention which is sustainable and reproducible by the MoH in Uganda, working within the existing government systems in conjunction with the MoH and district teams.

Table 3. Health care intervention implementation plan

	Month 1				Month 2				
	Week 1	Week 2	Week 3	Week 4	Week 5	Week 6	Week 7	Week 8	Week 9
1) Health center management									
HC management training	■	■	■	■	■	■	■	■	■
2) Information management									
Continuous quality improvement	■	■	■	■	■	■	■	■	■
3) Health worker training									
Case management and RDTs		■		■					
Patient-centered services					■	■	■	■	■
4) Supply of consumables									
NMS drug distribution	■	■	■	■	■	■	■	■	■
Support for AL stock-outs	■	■	■	■	■	■	■	■	■
RDTs for malaria			■	■	■	■	■	■	■

Prior to the start of the study, investigators and key study personnel will meet with members of the MoH, the National Malaria Control Program, district and sub-county officials, and community representatives to inform them about the study objectives, plans for the intervention, and follow-up assessments. An information sheet will be used to describe the plans for the intervention to the in-charges of health facilities randomized to the HFI (Appendix B).

5.2. HEALTH CENTER MANAGEMENT

5.2.1. Overview

All in-charges of health centers randomized to the HFI will be trained in health center management. The purpose of this training is to equip in-charges with the skills and tools required to effectively and efficiently manage their health center. The training module will be based on the concepts presented in the Management Sciences (MSH) for Health guide, 'Health Systems in Action: an eHandbook for leaders and managers' (http://www.msh.org/Documents/upload/msh_eHandbook_complete.pdf). The information provided in the MSH guide will be adapted as appropriate to our study setting and the individual health facilities. The training will include three components (Table 4).

Table 4. Proposed content of Health Center Management training

Human resource management	Financial management	Supply management
— Staffing and allocation<br>— Using volunteers	— Financial reporting<br>— Budgeting and accounting	— Ordering and distributing<br>— Inventory management and stock control<br>— Rational use and dispensing<br>— Managing stock-outs

Our study staff will conduct a series of half-day group training workshops with the in-charges every 1-2 weeks. The sessions will be conducted using both formal classroom lecture sessions and informal practical methods with discussions, demonstrations, and role plays. The Health Center Management Training module will last approximately 8-10 weeks. Workshops will be held in convenient locations for the participants and all costs will be covered by the project.

5.2.2. Human resource management

Staffing needs and appropriate use of volunteers will be the focus of the human resource management component. Our formative research revealed that inadequate staffing at health centers had a major impact on provision of good quality care. In addition, health workers cited that irregular payment of salaries resulted in poor job motivation and prompted them to seek alternative sources of income, such as seeking other employment in place of attending their shifts at the health center. In addition, we found that a significant number of health workers in the area are actually untrained volunteers. These volunteers are engaged in various roles including dispensing medications, immunizing, delivering mothers, registering patients, and dressing wounds. We will work with in-charges to help finds ways to appropriately use volunteers and to address the current staffing gaps at their health centers, including methods of communicating with the district about staffing gaps and need for timely payment of salaries. The project team will work with the in-charges to determine the appropriate staffing requirements, using the MoH Staffing Norms policy as a guide (Appendix C), and with the Ministry and district officials to help meet staffing needs at the HFI centers.

5.2.3. Financial management

This component will focus on financial management and accounting including documentation of financial transactions and audit trails, procurement, and management of funds. In addition, the training will cover budgeting and accounting for the primary health care (PHC) funds. In our formative research, we found that management of the health centers was challenged by insufficient PHC funds. These funds are meant to cover the costs of a variety of activities at the health centers, including support staff, cleaning materials, transportation of drugs, and photocopying documents. Health centers are allocated an amount set by the district, and expenditure appears to vary depending on the individual needs of the facilities. As part of the HFI, we propose to train the in-charges to account for the PHC funds using a tool (Appendix D) designed to assist with the quantification and prioritization of the PHC funds. Information will be collected on the action or service required; expected benefit or consequence if action/service cannot be conducted; person responsible; cost; frequency; and total cost per month. The information collected using the tool will be used for accounting, planning and prioritizing, and justifying to the district and MoH the need for continued or increased PHC funding to the health centers. The sample tool provided in Appendix D will be piloted and adapted to each individual facility during the Health Center Management Training.

5.2.4. Supply management

Effective management of supplies is a crucial aspect of providing high quality health care services. This component will focus on enhancing the understanding and operation of the drug distribution cycle including barriers in ordering, receiving, and issuing of drugs; forecasting of supply needs; maintaining a regular inventory to prevent stock-outs; and completing and maintaining stock cards. In-charges will be trained to use the one-page ACT Drug Distribution Assessment Tool (ADDAT), which aims to identify and resolve issues in drug distribution from districts to local health facilities (Appendix E). The ADDAT is based on a literature review, in-depth interviews and focus group discussions (FGDs) with health workers and key informants, and the MoH 'Medicines Management Manual: Medicines Logistics and Store Management procedures for Districts and Health Facilities'. The ADDAT will be used monthly by in-charges or persons responsible for drug procurement and distribution at the health center. The sample tool provided in Appendix E will be piloted prior to the Health Center Management Training, and revised as appropriate.

5.3. HEALTH INFORMATION MANAGEMENT

This component will focus on the importance of monitoring and evaluation and managing health information, building on our experience with UMSP surveillance and CQI and other work done elsewhere in Africa (http://hciproject.org/). To make informed decisions, in-charges and health workers should have access to information about the health needs and priorities of the communities they serve; the quality and coverage of the services they offer; and available resources. In-charges will be trained to capture information on patients accessing their health facility, diagnoses made, and treatment provided, and how to utilize that information to generate reports, supervise staff, and provide feedback to the health facility team to ensure continuous quality improvement (CQI). Information will be captured using the patient, drug, and laboratory registers. In-charges will be taught to tally information at the end of each month and to complete a report (Appendix F) which will include data on

— Number of patients seen at the facility
— Number of children under five
— Number of patients with fever
— Number of patients who had a RDT performed
— Number of patients with a positive RDT
— Number of patients prescribed an ACT
— Number of patients diagnosed with malaria

Copies of the reports will be given to study personnel during the surveillance visits to the health facilities (Chapter 8). In addition, study personnel will provide detailed reports of RDT utilization and rational prescribing of ACTs bi-monthly. Training will be provided to a district-level employee, such as the malaria focal person, and the in-charges to help build capacity for report generation and how to utilize the data effectively to improve the quality of care provided at the facilities.

5.4. HEALTH WORKER TRAINING

5.4.1. Training in fever case management and use of RDTs

All clinical staff will receive training in fever case management. Training will be based on the Integrated Malaria Management training package developed by the JUMP team,[21] and the RDT training guidelines which have been adopted and implemented by Uganda's MoH 'User's manual: Use of Rapid Diagnostic Tests (RDTs) for malaria in fever case management in Uganda' (Appendix K). The training package will include the following sessions:

— Session 1: How to evaluate febrile patients and select patients for RDT testing
— Session 2: Performing and reading an RDT
— Session 3: Management of a patient with fever and a positive RDT
— Session 4: Management of a patient with fever and a negative RDT
— Session 5: Recognition and referral of patients with severe illness
— Session 6: Patient education
— Session 7: RDT storage and monitoring
— Session 8: Infection prevention

The training program will last one week; the first two days will focus on theory, and the remaining 3 days will include practical aspects. The impact of training on knowledge will be assessed using a pre- and post-training evaluation (Appendix H). The training will be conducted in Tororo at a local health facility by study personnel in collaboration with the JUMP training team. Health workers will be trained in two small groups to ensure that work at the health facilities continues alongside the training.

5.4.2. Training in patient-centered services

The purpose of the Patient-Centered Services (PCS) training module is to identify and improve interpersonal interactions between health workers and patients. The module builds on the results of our formative research which identified several barriers to proving good quality health care at health facilities, including poor interpersonal interactions between health workers and community

members resulting from poor communication skills, discriminatory behaviors of health workers, poor health worker motivation, and lack of patient-centered thinking. Through the PCS module, health workers will learn to recognize these challenges and develop skills for communicating and interacting with patients.

The PCS module training will be implemented in a tiered approach to (1) all clinical staff, and (2) all health center support staff. All clinical staff, including in-charges, will receive the full PCS training package which includes self-observation tasks and specific emphasis on clinical and patient interaction challenges. Support staff including volunteers will receive a scaled-down PCS training package with specific emphasis on welcoming and guiding patients at the health facility. All training activities and workshops will be lead by study personnel and trainers with experience in adult learning methodology.

5.4.2.1. PCS for clinical staff

The PCS module for all clinical staff starts with a series of four self-observation activities completed by health workers over a period of 8-12 weeks. Health workers will conduct the self-observation exercises individually during their routine work at health facilities. Most health workers will have conducted their self-observation activities during the formative research of this project. These observations serve three purposes: (1) to raise awareness of health workers about how their interactions with others affects their ability to work, (2) to begin to build a support network between colleagues, and (3) to help to identify issues directly relevant to the health workers in lower level health facilities. Through the self-observation activities, health workers are asked to become aware of their behavior around a particular topic and write a short summary of how their behavior affected those around them and their subsequent ability to achieve work goals. Topics include the following: (1) 'how you ask questions', (2) 'how you facilitate or hinder good communication', (3) 'what you do to relieve anxiety'; and (4) 'how you manage anger and irritation' (Appendices I & J). On completion of each self-observation, participants join other health worker colleagues to discuss their own observations and give support about how to carry out the next observations. The results of the observation activities are used to guide health workers through the one-week module.

Table 5: Clinical staff PCS module themes and competency aims

PCS theme	Competency aim
Coaching	In-charges will be able to:<br>• Motivate and inspire others to work better<br>• Facilitate meetings<br>• Give constructive feedback<br>• Feel empowered to bring about positive change
Communication with patients	Health workers (including in-charges) will be able to:<br>• Identify their own motivations for work<br>• Give patients time to talk<br>• Show compassion<br>• Communicate positively<br>• Identify strategies to ensure equity of services
Communication with colleagues	Health workers (including in-charges) will be able to:<br>• Develop self-awareness and respect for others in the workplace<br>• Contribute to a positive working environment<br>• Manage stress
Patient flow	Health workers (including in-charges) will be able to:<br>• Give a positive welcome to patients<br>• Find strategies to ensure that shorter and more equitable waiting times for patients<br>• Find strategies to improve patient navigation at the health center

The PCS module consists of four half-day day themed workshops, which build on the self-observation exercises. The four themes and competency aims are outlined in Table 5. The modules will use a combination of learning approaches including didactic material on each theme; appreciative enquiry exercises; role-plays or other participatory activities; and group discussions. The didactic component is important for framing the central concepts and theories around each theme. Appreciative enquiry exercises will allow participants to explore ways in which they successfully worked through difficult situations and identify what qualities they employed, using analysis and reinforcement of positive experiences to lead to behavior change, rather than focusing solely on negative experiences or deficiencies. Participants will work in small groups for these exercises. The group discussion will explore reactions to the participatory activities and develop methods that can be employed to achieve successful outcomes related to the theme. Additional ice-breaker and group-building activities will be used to engage and motivate participants throughout the workshop.

The half-day workshops will be approximately 3 hours in length and will be conducted over 2 weeks at a convenient location in the study area. Health workers will be trained in two groups to ensure that work at the health facilities continues alongside the training.

5.4.2.2. PCS for support staff

The PCS workshop for support staff will consist of two half-day themed modules using a combination of learning approaches. The modules are simplified versions of the clinical staff PCS workshop and will include the presentation of didactic material on the theme; appreciative enquiry exercises; role-

plays or other participatory activities; and group discussion. The two themes linked to each self-observation activity and learning outcomes are outlined in Table 6.

The half-day workshops will be approximately 3 hours in length and will be conducted over 1 week at a convenient location in the study area. Support staff will be trained in two groups to ensure that work at the health facilities continues alongside the training.

Table 6: Support staff PCS module themes and competency aims

PCS theme	Competency aim
Communication skills	Support staff (including volunteers) will be able to:<br>• Contribute to a positive working environment<br>• Manage stress
Patient flow	Support staff (including volunteers) will be able to:<br>• Give a positive welcome to patients<br>• Find strategies to ensure that shorter and more equitable waiting times for patients<br>• Find strategies to improve patient navigation at the health center

5.5. CONSUMABLES

Results from the formative research indicate that drug shortages and stock-outs at health facilities are a major problem, especially for AL, the first-line treatment for uncomplicated malaria. Both the quantitative and qualitative research show that these shortages have an impact on community members' perceptions of care provided at health centers and health workers' ability to provide good quality care. In addition, most health centers lack laboratory facilities and currently do not have the capacity to confirm the diagnosis of malaria. A major aim of the HFI is to ensure a continuous and adequate supply of drugs and RDTs for malaria.

5.5.1. Drug delivery from National Medical Stores

In Uganda, the National Medical Store (NMS) has recently adopted a new 'modified push' system for distributing drugs to lower-level health centers. Districts are to make an annual procurement plan, and NMS will deliver the drugs every 2 months, based on that plan. If no additional requests are made by health facilities or the district, NMS will provide a standard delivery of drugs based on the procurement plan, regardless of health facility need or disease prevalence. Once the procurement plan is developed, and annual orders have been made, health facilities will have limited power to change the drug deliveries.

As part of the HFI, the study team will work with the in-charges and the district health officer (DHO) to ensure the drug procurement system is working as planned including ordering, transporting, tracking, and storing of drugs. The in-charges of the health facilities will be trained to use the ADDAT tool (Appendix E, section 5.2.5). When required, the team will work with key stakeholders to facilitate drug delivery to the health centers.

5.5.2. Supply artemether-lumefantrine

If the amount of AL provided to the health centers by NMS is not adequate to meet demand, or if the procurement of AL fails, the project will supply supplemental AL obtained from Joint Medical Stores in Uganda. Batch numbers, expiry dates, date received, and date opened will be recorded for all AL supplied.

5.5.3. RDTs for malaria

Most health centers do not have access to a laboratory or malaria diagnostic facilities. As part of the HFI package, we will ensure adequate supplies of RDTs at all health centers. One of the HRP2-based tests, are recommended in the most recent WHO / FIND Product Testing Report (http://www.wpro.who.int/sites/rdt/home.htm) and are approved by the Uganda National Drug Authority, will be used. Batch numbers, expiry dates, date received, and date opened will be recorded for all RDTs supplied. RDTs may be introduced into lower-level health centers by the MoH. If so, we will track distribution and use of RDTs in a manner similar to that of drugs, as described above, and will supplement distribution of RDTs if needed.

6 STANDARD CARE

6.1. OVERVIEW

In this study, 10 health facilities will be randomly assigned to the standard care arm. Standard care will include services typically provided by government-run facilities.

6.2. STAFFING AND PATIENT ATTENDANCE

In our formative research, we interviewed 17 health workers, including the in-charges of health centers, to gain an understanding of the current situation at the local government-run health facilities. Not all health centres are open every day, depending on the availability of drugs and staff. The mean number of patients visiting the health centres each day is approximately 50-60, and staffing shortages are an issue at most health centers (Table 6.1).

Table 6.1: Patient attendance and staffing, stratified by level of health centre

	HC II<br>N=12	HC III<br>N=3
Patients visiting (mean, SD, range)	51.2 (20.4) 25-100	52.5 (15) 30-60
Staff stationed (mean, SD, range)	2.08 (0.66) 1-3	9.75 (0.95) 9-11
Staff available (mean, SD, range)	1.4 (0.51) 1-2	4.25 (0.96) 3-5

6.3. INFRASTRUCTURE AND SUPPLIES

Infrastructure at the health centres in this area is limited; most lack electricity and running water. Drug stock-outs are a common problem at HC IIs and IIIs. Most centres, but not all, stock AL but few centres stock artesunate + amodiaquine, the alternative treatment for uncomplicated malaria. Stock-outs of both parenteral and oral quinine are also common. Malaria is diagnosed clinically, without laboratory confirmation at most health centres, even at the two HCIIIs with laboratories.

6.4. STANDARD CARE

Standard care will include services typically provided by government-run facilities; we will not provide any additional support to these facilities. Health care will be provided to patients attending these facilities according to the usual standards; in-charges will continue to manage the facilities using their standard approach, no additional training will be provided to the health workers stationed at these facilities; and no support for staffing or supplies will be provided beyond what is supplied by the district and MoH.

7 CROSS-SECTIONAL SURVEYS

7.1. OVERVIEW

Cross-sectional surveys will be conducted in randomly selected children under fifteen years of age. We plan to sample 400 children from each cluster; 200 children under five and 200 children aged 5-15 years. A total of 8000 children will be sampled in each survey. Surveys will be conducted at baseline and then annually for each year; new populations of children will be selected for each survey. The survey will include a structured questionnaire administered to the primary caregiver, and a clinical and laboratory assessment of each child.

7.2. DEFINITIONS

Household: A household will be defined as any single permanent or semi-permanent dwelling structure acting as the primary residence for a person or group of people that generally cook and eat together. Some households may include members who sleep in other dwelling structures within the same compound, if the members are still dependent on the head of household in the main household.

Household resident: A resident within each household will be defined as a person who intends to have a sleeping place primarily at that location for a period of the next 6 months. This may include people who sleep in a separate house within the same compound, if they are still dependent on the head of household for decisions on finances and health care.

7.3. ENROLLMENT

7.3.1. Recruitment

All households enumerated during the census will be assigned a unique number. A random sample of households with at least one child under fifteen years of age will be selected from each cluster to generate a list of households to be approached. Three separate lists will be generated prior to each survey from the original census list. Study personnel will conduct door-to-door screening to identify those households with at least one child of appropriate age. Households without a child of appropriate age will be removed from the recruitment list. Residents not home during the initial contact will be re-visited on at least three other occasions over a six-week period before eliminating them from our sample selection process.

7.3.2. Screening

When a household with at least one child of appropriate age is identified, study personnel will briefly describe the purpose of the study in the appropriate language (usually Japadhola, Luganda or

Swahili) with parent(s) or guardian(s), and proceed with screening (Appendix K). The inclusion criteria are: 1) age < 15 years, 2) agreement of parents or guardians to provide informed consent, and 3) agreement of a child aged 8 years or older to provide assent. The exclusion criterion is: 1) inability to locate the child. One child under five and one child between the ages of 5-15 years from each household will be eligible for participation. If more than one child of appropriate age resides in the household, study personnel will record the gender and ages of all children under five and all children between the ages of 5-15 years, and one child from each age category will be randomly selected for participation. If only one child of appropriate age, or only one child in each age category, resides in the household they will be selected for participation. Previous participated in one of the cross-sectional surveys will not be an exclusion criterion; children who have previously participated in a survey will still be eligible to participate.

7.3.3. Informed Consent

Study personnel will conduct the informed consent discussion with the parent(s) or guardian(s). Informed consent will be conducted in the appropriate language and a translator will be used if necessary. Consent forms will be available in English and the local languages (Appendices L & M). Following the informed consent discussion, parents/guardians will be asked by the study personnel to sign a written consent form for their child to participate in a research study and a second approved consent form for the future use of biological specimens obtained during the course of the study. If the parent/guardian is unable to read or write, their fingerprint will substitute for a signature, and a signature from a witness to the informed consent procedures will be obtained. Written assent to participate in the study will also be obtained from children aged 8 years and older at the time of screening (Appendix N).

7.3.4. Enrollment

Children who fulfil the eligibility criteria will be assigned a study number and will undergo the survey procedures outlined below. If a child selected to participate is not at home on the day of the initial visit, the household will be re-visited on at least three other occasions over a six-week period before excluding the child.

7.4. SURVEY PROCEDURES

7.4.1. Survey questionnaire

Primary caregivers of selected children will be asked to complete a survey questionnaire to gather information about bednet use and management of child who was febrile within the last two weeks, including source of care, diagnostics test results, drug treatment, and actions taken for management of illnesses (Appendix O, Part 1). Data will be collected about management of fever in any child under the primary caregiver's care, and will not be restricted to children selected to participate in the survey. If more than one child has been febrile in the last two weeks, data will be collected on management of the most recent illness. If the primary caregiver is not available on the day of the initial visit, the household will be re-visited on at least three other occasions over a six-week period to administer the questionnaire. If the primary caregiver is not available after four visits, the survey

questionnaire will not be completed. However, the child or children selected for the cross-sectional survey from that household will not be excluded, and all additional information collected will be utilized. In the last annual cross-sectional survey, all women of child-bearing age (13-49 years) in the household will be asked to provide birth histories, which will allow us to estimate all-cause mortality in children under five (Appendix O, Part 2).

7.4.2. Clinical and laboratory assessment

Participating children will undergo a brief history and physical examination, including measurement of temperature, height, weight, mid-upper arm circumference, and spleen size (Appendix P). Blood will be collected by fingerprick for thick blood smear, hemoglobin, and for storage on filter paper for future molecular testing.

7.4.3. Management of ill children

Children enrolled in the survey who report fever in the past 48 hours, or who have a temperature of $\geq$ 38.0ºC will have an RDT performed. Febrile children will be treated with paracetamol as appropriate. Children with a positive RDT and no evidence of severe malaria will be treated with AL. Children with a positive RDT and evidence of danger signs of severe disease will be referred for further evaluation and treatment. Children with a hemoglobin level of < 5.0 g/dL will be referred for further evaluation and transfusion. Any child with other concerning clinical symptoms will also be referred to an appropriate health care facility at the discretion of the study personnel.

8 COHORT STUDY

8.1. OVERVIEW

A cohort of children under five will be enrolled from 25 households randomly selected from each cluster, for a total of 500 households. The cohort will be dynamic, in that all children within a household, who are under the age of five and who meet selection criteria, will be included. A household survey will be conducted at the start of the study. Children will undergo clinical and laboratory assessments at baseline and then every six months. Primary caregivers will be asked to prospectively collect information on the clinical symptoms of participating children and expenditures for health care using pictorial diaries. Study personnel will visit the households monthly to collect the diaries and administer a monthly questionnaire.

8.2. ENROLLMENT

8.2.1. Recruitment

All households enumerated during the census will be assigned a unique number. A random sample of households with at least one child under five will be selected from each cluster prior to randomization to generate a list of households to be approached. Study personnel will conduct door-to-door screening to identify those households with at least one child of appropriate age. Households without a child of appropriate age will be removed from the recruitment list. Residents not home during the initial contact will be re-visited on at least three other occasions over a six-week period before eliminating them from our sample selection process. When a household with at least one child of appropriate age is identified, study personnel will briefly describe the purpose of the study in the appropriate language (usually Japadhola, Luganda or Swahili) with parent(s) or guardian(s). If the parent(s)/guardian(s) are interested in the study, the study personnel will schedule an appointment date for screening. Residents not home during the initial contact will be re-visited on at least 3 other occasions over a 6-week period before eliminating them from our sample selection process. All children of appropriate age from a single household will be eligible for evaluation for study enrollment.

8.2.2. Screening

Children will be screened at convenient sites within the community. Interviews will be conducted in the appropriate language with parents or guardians. Selection criteria are based on the goal of recruiting a representative sample of children from our target population (Appendix Q). The inclusion criteria are: 1) age < 5 years, and 2) agreement of parents or guardians to provide informed consent. The exclusion criteria are: 1) intention to move during the follow-up period. During the screening process, study personnel will assess for initial eligibility criteria through conversation with the parent/guardian (including age of the child, willingness of the parent/guardian to participate in the study and to provide informed consent, and intention to move from Tororo). If the initial

screening criteria are met, the parent/guardian will be asked to provide informed consent for their child to participate in the study. If parents/guardians are undecided about consenting for their child (or children) to participate in the study at the initial screening visit, they will be allowed up to one week to make a final decision about study participation.

8.2.3. Informed Consent

Study personnel will conduct the informed consent discussion with the parent(s) or guardian(s). Informed consent will be conducted in the appropriate language and a translator will be used if necessary. Consent forms will be available in English and the local languages (Appendices R & S). Following the informed consent discussion, parents (or guardians) will be asked by the study personnel to sign a written consent form approved by the IRBs for their child to participate in a research study and a second approved consent form for the future use of biological specimens obtained during the course of the study. If the parent or guardian is unable to read or write, their fingerprint will substitute for a signature, and a signature from a witness to the informed consent procedures will be obtained.

8.2.4. Enrollment

Children who meet the eligibility criteria will be assigned a study number, and will undergo a clinical and laboratory evaluation. Height, weight, and temperature will be measured, and spleen size will be evaluated (Appendix T). Blood will be collected by finger prick for thick blood smear, hemoglobin, and for storage on filter paper for future molecular testing.

8.2.5. Management of ill children at enrollment

Children enrolled in the cohort study who report fever in the past 48 hours, or who have a temperature of $\geq 38.0^{o}C$ will have an RDT performed. Febrile children will be treated with paracetamol as appropriate. Children with a positive RDT and no evidence of severe malaria will be treated with AL. Children with a positive RDT and evidence of danger signs of severe disease will be referred for further evaluation and treatment. Children with a hemoglobin level of < 5.0 g/dL will be referred for further evaluation and transfusion. Any child with other concerning clinical symptoms will also be referred to an appropriate health care facility at the discretion of the study personnel.

8.3. HOUSEHOLD SURVEYS

Following enrollment, or within a 2 week period from the date of enrollment, a household survey will be performed at all participating households (Appendix U). Primary caregivers will be asked to complete a survey questionnaire to gather information about bednet use and management of a child who was febrile within the last two weeks, including source of care, diagnostics test results, drug treatment, and actions taken for management of illnesses. Data will be collected about management of fever in any child under the primary caregiver’s care, and will not be restricted to children selected to participate in the cohort study. If more than one child has been febrile in the last two weeks, data will be collected on management of the most recent illness. If the primary caregiver is not available on the day of the initial visit, the household will be re-visited on at least three other

occasions over a six-week period to administer the questionnaire. If the primary caregiver is not available after four visits, the household survey questionnaire will not be completed. However, the child or children selected for the cohort study from that household will not be excluded, and all additional information collected will be utilized.

8.4. FOLLOW-UP

8.4.1. Household diaries and monthly visits

Primary caregivers will be asked to keep a diary of health of study participants for the duration of the study (Appendix V). The diaries will be based on instruments previously developed and validated in studies in Uganda and elsewhere in Africa.[92,95] The diaries have been developed by a Ugandan artist with input from the community, and will be adapted to the local setting if necessary. The diaries will be used to collect information on clinical symptoms and health care expenditures. Households will be visited by study personnel every two weeks during the first two months, and then monthly, to collect completed diaries. At each monthly visit, questionnaires will also be administered to gather additional data on the health of the study participants, management of any illnesses, and health care expenditures (Appendix W). The information collected in the diaries and the questionnaires will be complementary. Study personnel will review the diaries with the primary caregivers at each monthly visit and caregivers will be allowed to refer to the diaries while the questionnaire is administered to help prompt their memory. Small incentives (including sugar, soap, or washing powder) will be provided to each household during the monthly visit to encourage completion of the diaries.

8.4.2. Follow-up evaluations

Clinical and laboratory evaluations will be repeated every 6 months over the 2 years of study follow-up in all cohort study participants (Table 7). At the end of study follow-up, all children will be given an insecticide-treated bednet.

Table 7. Study objectives and outcome measures

Evaluation	Baseline	Every 6 months
Clinical evaluations		
Height, weight	X	X
Temperature	X	X
Spleen size	X	X
Laboratory evaluations		
Thick blood smear	X	X
Hemoglobin	X	X
Filter paper sample	X	X
Household evaluations		
Household KAP survey	X	—
Pictorial diaries	—	Monthly
Household questionnaires	—	Monthly

8.4.3. Management of ill children during follow-up

At the follow-up assessments, children who report fever in the past 48 hours, or who have a temperature of $\geq$ 38.0ºC will have an RDT performed. Febrile children will be treated with paracetamol as appropriate. Children will be managed as described above in section 7.2.5. Any child with concerning clinical symptoms will be referred to an appropriate health care facility at the discretion of the study personnel.

9 M&E OF HEALTH FACILITY INTERVENTION

9.1. OVERVIEW

We will conduct monitoring and evaluation (M&E) activities at all government-run health facilities in the study area, including those that are randomized to the intervention and those that are not. Patient exit interviews will be conducted every 6 months to gather information on rational use of ACTs and patient satisfaction. In addition, standardized information will be captured from all facilities including data on patient attendance, drug stock-outs, and staffing shortages every two months. Additional, detailed information on RDT utilization and ACT prescribing practices will also be collected from HFI facilities. A knowledge questionnaire will be administered annually to consenting health workers at all facilities. M&E activities will begin one month after the roll-out period (lasting 8-10 weeks) for the HFI has been completed.

9.2. PATIENT EXIT INTERVIEWS

9.2.1. Overview

Every six months, exit interviews will be conducted with children under five and their caregivers at all health facilities. The purpose of the interviews is to evaluate for rational prescribing of ACTs, and to determine the level of satisfaction with the health facility visit. For each survey, 10 patients will be selected by convenience sampling from each facility to participate in the interviews (200 total per survey).

9.2.2. Recruitment

As children and their caregivers are leaving the health facility, study personnel will ask the caregiver if their child is under five and if they would be willing to participate in an interview regarding the health care visit (Figure 4). When a caregiver with a child of appropriate age is identified, study personnel will briefly describe the purpose of the study in the appropriate language (usually Japadhola, Luganda or Swahili) with parent(s) or guardian(s). The inclusion criteria are: 1) age < 5 years, and 2) agreement of parents or guardians to provide informed consent (Appendix X). There are no exclusion criteria. If more than one child of appropriate age was seen at the health facility that day, all children will be eligible to participate.

9.2.3. Informed Consent

Study personnel will conduct the informed consent discussion with the parent(s) or guardian(s). Informed consent will be conducted in the appropriate language and a translator will be used if necessary. Consent forms will be available in English and the local languages (Appendix Y). Following the informed consent discussion, parents/guardians will be asked by the study personnel to sign a written consent form for their child to participate in a research study. If the parent/guardian is

unable to read or write, their fingerprint will substitute for a signature, and a signature from a witness to the informed consent procedures will be obtained.

Figure 4. Patient exit interview algorithm

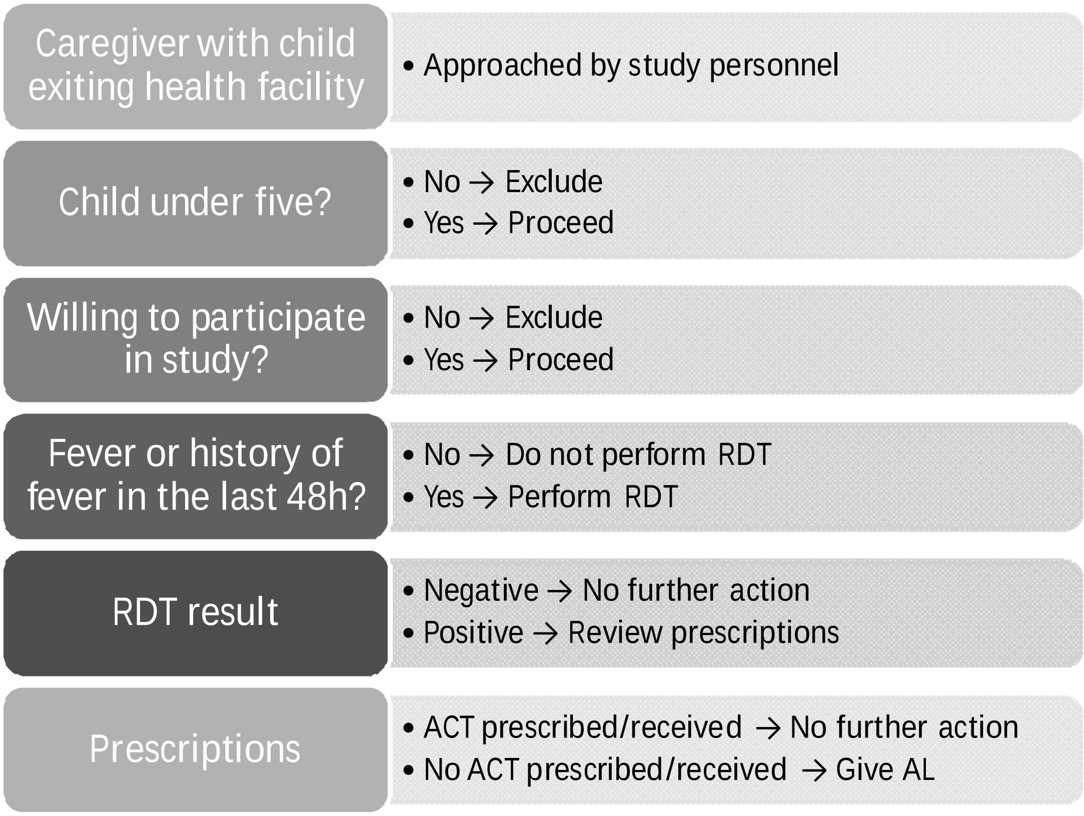

9.2.4. Interview

Children who fulfil the eligibility criteria will be assigned a study number and will undergo the survey procedures. Caregivers of enrolled children will be interviewed using a standardized questionnaire (Appendix Z) to gather information about the purpose of the visit, presenting complaint, the child's symptoms, whether a RDT or blood smear was done, the diagnosis given, medications prescribed, and medications received. Additional information about the satisfaction of the caregiver with the visit to the facility will also be obtained.

9.2.5. RDT results

If a child has a temperature of >38.0°C or a history of fever in the past 48 hours, a fingerprick blood sample will be obtained to perform a RDT. Febrile children will be treated with paracetamol as appropriate. The results of the RDTs performed by the study personnel will be compared to the results of RDTs performed by health facility staff, where possible. Children with a positive RDT and no evidence of severe malaria, who have not been prescribed or received an ACT, will be given AL (Figure 4). Children with a positive RDT and evidence of danger signs of severe disease will be

referred to an appropriate facility for further evaluation and treatment, regardless of the medicine prescribed.

9.3. HEALTH FACILITY SURVEILLANCE

9.3.1. All facilities

We plan to conduct routine surveillance at all health facilities every month. The purpose of the surveillance is to collect information about patient attendance, drug stocks, staffing gaps and health facility costs from all facilities. Study personnel will collect the information during a one-day visit to the health facilities. The in-charge of the facility will be approached and informed about the surveillance activities. An information sheet (Appendix AA) will be used to describe the purpose of the activities and verbal consent to collect information will be obtained from the in-charge. The surveillance form (Appendix BB, Part 1) contains three sections: (1) data on patient attendance including date visited, village of residence, age, sex, and diagnoses collected from the patient registers; (2) drug stock data collected from the drug register; and (3) staffing allocation data collected from the in-charge. The patient register and drug register are standard HMIS data collection tools, and are in place in all health facilities. The surveillance form will be used to collect information on health facility costs (Appendix BB, Part 3) including 1) drugs & supplies, 2) overheads (utilities, capital equipment, space), and 3) staff allocation and activities. Part 3 will only be conducted at baseline and annually for a total of three surveys.

9.3.2. HFI facilities

Additional information on utilization of RDTs and use of ACTs will be collected at facilities randomized to the HFI and any other facilities where RDTs are available (Appendix BB, Part 2). This information will be extracted from the patient, drug, and laboratory registers by study personnel. Study personnel will help the in-charges create the registers, to ensure that the appropriate information is captured. The data collected will complement the information tallied by the in-charges as part of the CQI activity in the health center management training module (section 5.2.6). Additional indicators evaluated in the health facility surveillance will include:

— Number (proportion) of patients who had a RDT performed
— Number (proportion) of patients with a positive RDT
— Number (proportion) of patients diagnosed with malaria
— Number (proportion) of patients diagnosed with malaria by RDT result
— Number (proportion) of patients with a positive RDT prescribed an ACT
— Number (proportion) of patients with a negative RDT prescribed an ACT
— Number (proportion) of patients without a RDT done prescribed an ACT

Reports of these indicators will be generated after each surveillance visit and will be provided to the in-charges of the facilities at the next visit as part of the ongoing CQI activities (section 5.2.6).

9.4. HEALTH WORKER KNOWLEDGE QUESTIONNAIRES

Questionnaires will be administered to health workers at all health facilities annually to assess their knowledge about fever case management. The first annual questionnaire will be administered after the intervention has been active for approximately one year. All available health workers will be approached to participate in the knowledge questionnaire. Information sheets will be used to describe the purpose of the knowledge questionnaire and verbal consent will be obtained from the health care worker before conducting the questionnaire (Appendix CC). The knowledge questionnaire includes structured questions allowing for open-ended answers to assess the health workers' knowledge about malaria transmission, symptoms, diagnosis, and treatment, and etiology of non-malaria fevers (Appendix DD).

10 ADVERSE EVENT MONITORING

10.1. OVERVIEW

Adverse event monitoring will be conducted in children enrolled into the cohort study. Data on serious adverse events and suspected adverse drug reactions will be collected retrospectively during the monthly interviews. Serious adverse event reports and summary reports of suspected adverse drug reactions will be submitted to the IRBs, the Data and Safety Monitoring Board (DSMB), and the ACT Consortium Drug Safety Register (ACTcDSR) according to their guidelines.

10.2. DEFINITIONS

Serious adverse event (SAE): An experience that results in any of the following outcomes:

- Death during the period of study follow-up
- Life-threatening experience (one that puts a participant at immediate risk of death at the time of the event)
- Inpatient hospitalization during the period of study follow-up
- Persistent or significant disability or incapacity
- Specific medical or surgical intervention to prevent one of the other serious outcomes listed in the definition.

Adverse drug reaction (ADR): A response to a medicine which is noxious and unintended, and which occurs at doses normally used in man.

10.3. IDENTIFICATION OF ADVERSE EVENTS

At each monthly visit, study personnel will inquire about the occurrence of any SAEs and any suspected ADRs. Two severity grading scales will be used to grade severity of events: 1) the WHO toxicity grading scale, which will be used by all projects involved in the ACT Consortium, and 2) a severity grading scale used previously in clinical trials in Uganda, which is based on the WHO toxicity grading scale and the National Institutes of Health, Division of Microbiology and Infectious Diseases grading scales (Appendices EE & FF).

10.4. REPORTING OF ADVERSE EVENTS

For each SAE and suspected ADR identified, an adverse event report form will be completed (Appendix GG & HH). The following information will be recorded for all experiences that are reported:

- Description of the patient (ID number, age, sex, weight)
- Description of the adverse event

— Date of event onset and date of resolution
— Date event reported
— Maximum severity of the event
— Causality and expectedness
— Whether the event was serious
— Outcome of the event
— Drug information (names, doses, dates administered for all drugs taken one month prior)
— Past medical history
— Known allergies and/or prior experience with drugs taken

10.5. ACT CONSORTIUM DRUG SAFETY REGISTER

10.5.1. Overview

Safety data collected in our study will be submitted to the central ACT Consortium Register within timescale agreed by the Consortium's Steering Committee. The Steering Committee will ensure the timely dissemination of any early warnings of emerging patient safety information within the project to all parties in the Consortium and to other relevant stakeholders. All individual patient data transferred to the ACT Consortium Register will be identifiable by study number only. No names or addresses of individuals will be stored. The adverse event data will be coded centrally using industry-standard MedDRA (Medical Dictionary of Drug Regulatory Authorities) coding systems.

10.5.2. Database

The database will fully comply with ICH – E6 Guideline for Good Clinical Practice,[96] in particular for the handling of electronic data (section 5.5.3), and will:

— Ensure and document that the electronic data processing system(s) conform to the sponsor's established requirements for completeness, accuracy, reliability, and consistent intended performance (i.e. validation).
— Maintain standard operation procedures for using these systems.
— Ensure that the systems are designed to permit data changes in such a way that the data changes are documented and that there is no deletion of entered data (i.e. maintain an audit trail, data trail, edit trail).
— Maintain a security system that prevents unauthorized access to the data.
— Maintain a list of the individuals who are authorized to make data changes
— Maintain adequate backup of the data.
— Safeguard the blinding, if any (e.g. maintain the blinding during data entry and processing).
— The individual patient data transferred to the central pharmacovigilance team will be identifiable by study number only. No names and addresses of individuals will be stored.

10.5.3. Mechanisms for data handling/sharing:

Each study will have ethical approval from all overseeing institutions, and sign an agreement regarding the data fields to be shared with the central database prior to the transfer of data to the

Liverpool School of Tropical Medicine (LSTM), who will manage the database. The agreement will be voluntary between the ACT Consortium study institution and the ACTc Pharmacovigilance database institution (LSTM). The study institution will remain the owner of their data at all times and the data will be used for pharmacovigilance monitoring within the ACT Consortium. Any future requests for information from the central database will require the permission of the owners of the data to enable access to the collated dataset.

11 LABORATORY PROCEDURES

11.1. MICROSCOPY

Thick blood smears will be stained with 2% Giemsa for 30 minutes and read by experienced laboratory technologists who are not involved in direct participant care. Parasite densities will be calculated by counting the number of asexual parasites per 200 leukocytes (or per 500 leukocytes, if the count is <10 asexual parasites/200 leukocytes), assuming a leukocyte count of 8,000/µl. A blood smear will be considered negative when the examination of 100 high power fields does not reveal asexual parasites. Gametocytemia will also be determined from thick smears. For quality control, all slides will be read by a second microscopist and a third reviewer will settle any discrepant readings.

11.2. RAPID DIAGNOSTIC TESTS FOR MALARIA

We will use RDTs when assessing febrile participants in the cross-sectional surveys, the cohort study, and in the patient exit interviews. In addition, we plan to support the distribution of RDTs to the HFI facilities. If RDTs are not distributed by the MoH to lower-level health facilities, we will provide RDTs to all HFI facilities. If RDTs are provided by the MoH, we will supply RDTs only when stock-outs occur. HRP2-based RDTs that have been approved by in the latest round of WHO / FIND product testing (http://www.wpro.who.int/sites/rdt/home.htm) will be used.

Each RDT will be interpreted by a health worker, and the results will be recorded in the appropriate log book. An RDT result will be considered positive if both the control line and the test line are visible after the development time. A result will be considered negative if the control line is visible, but no test line appears. The result will be considered invalid if no control line is visible, regardless of whether or not a test line appears.

11.3. HEMOGLOBIN MEASUREMENT

Hemoglobin will be measured from fingerprick blood samples using a portable spectrophotometer (HemoCue, Anglom, Sweden).

11.4. MOLECULAR STUDIES

Each time a thick blood smear is obtained in the cross-sectional survey and cohort study blood will also be collected onto filter paper. Samples will be collected by fingerprick sampling. Blood will be placed onto filter paper in approximately 25 ul aliquots per blood spot (4 blood spots per sample). The samples will be labeled with study numbers and dates, air-dried, and stored in small, sealed sample bags at ambient temperature with desiccant. Parasite DNA will subsequently be removed from the filter paper and prepared for molecular analysis using a chelex extraction method.

Molecular studies may include analyses of polymorphisms in parasite and/or human genes for mutations that may impact on clinical malaria, and genotyping of malaria parasites. Molecular studies will be performed only for research purposes and will have no impact on the clinical management of study participants.

12 STATISTICAL ISSUES

12.1. SAMPLE SIZE CALCULATIONS

12.1.1. Cluster randomized study

We plan to sample 200 children under five, and 200 children aged 5-15 years from each cluster in each cross-sectional survey, for a total of 8,000 children sampled annually. The primary outcome for the cluster randomized study will be the prevalence of anemia (hemoglobin < 11.0 g/dL) in children under five as measured in the cross-sectional surveys, which is assumed to be 65% at baseline estimated from data collected in a household survey conducted Mulanda sub-county in West Budama South (Rachel Pullen, unpublished data). We plan to test the primary hypothesis that children under five randomized to the HFI will have a lower prevalence of anemia than children randomized to receive standard care. With two study intervention arms, 10 clusters per intervention, a coefficient of variation between clusters of 0.2, and allowing for the stratified design, 200 children under five surveyed in each cluster will allow us to detect a difference in anemia prevalence between the two intervention arms of 17%, (or more) with power of 80% and significance of 5. If we assume that the clusters are more homogeneous, with a coefficient of variation of 0.1, then a difference of about 10% (or more) can be detected. As a secondary outcome, we will evaluate the prevalence of parasitemia in children under five, and in children aged 5-15 years. We will test the secondary hypothesis that children under five, and children aged 5-15 years, that are randomized to health facility-based care will have a lower prevalence of parasitemia than children randomized to receive standard care. In the older age group, the prevalence of parasitemia is estimated to be 60% at baseline based on data collected in Mulanda sub-county (Rachel Pullen, unpublished data). Again, with two study intervention arms, 10 clusters per intervention, and a coefficient of variation between clusters of 0.2, 200 children aged 5-15 years will need to be surveyed in each cluster to detect a difference in parasitemia prevalence in the two intervention arms of 16%, with power of 80% and significance of 5%.

12.1.2. Cohort study

The primary outcome for the longitudinal cohort study will be treatment incidence density, which is assumed to be 2.5 treatments per year at baseline. We plan to recruit 500 households into the longitudinal study (25 in each cluster), and estimate that the average number of children under five per household will be at least 1.5. Given this, 250 households, and at least 375 children, will be available in each intervention group. Assuming 30% loss to follow-up over 2 years, 262 children can be followed for a total of 2 years, resulting in 524 child-years of follow-up in total; shared between 10 clusters, this results in 52.4 person-years per cluster on average. If the baseline group experience 2.5 treatments per year, resulting in 5 treatments per child over 2 years, then a difference of 2 treatments on average over 2 years can be detected between the two interventions (for example if the HFI group experiences 5 treatments over 2 years and the standard care group experiences 7 treatments per two years, or approximately 3.5 per year per child), assuming a coefficient of variation between clusters of 0.2, power of 80%, significance level of 5%, and allowing

for the stratified design. This calculation does not include any partial follow-up data that are likely to be available from those that drop out over the course of the two years.

12.1.3. Patient exit interviews at health facilities

The primary outcome for the patient exit interviews will be the proportion of children under five with suspected malaria and a negative rapid diagnostic test result that are inappropriately treated with an ACT. The hypothesis is that the proportion of children inappropriately treated with an ACT will be lower in the facilities randomized to the intervention arm compared to those in the standard care group. Assuming this proportion to be 50% in the standard care group, interviewing 10 children and their caregivers in each of the 20 clusters will give 80% power to detect a difference in the proportion inappropriately treated for malaria between the two intervention arms of 24% (or more) at the 5% significance level, assuming a coefficient of variation between clusters of 0.2 and allowing for the stratified design.

12.2. ANALYTICAL PLAN

12.2.1. Analytical approach

Since this is a cluster randomised trial, analysis will be conducted at both the cluster level using summary statistics from each cluster, and at the individual level. The primary analysis will be based on the cluster-level results as this is expected to be more robust when the number of clusters randomised is not large. A two-stage approach based on cluster summaries will be used to adjust for individual- and cluster-level covariates, where appropriate.

Analysis will be conducted primarily on an intention to treat (ITT) basis, where data collected will be analysed according to the assigned cluster. To address potential contamination issues due to children attending health facilities other than the one that defines their cluster, i.e. the health facility they live closest to, we shall also analyse data on a per protocol (PP) basis. For this analysis, data collected will be analysed according to the facility at which the child accesses healthcare.

12.2.2. Baseline characteristics and trial profile

Baseline demographic characteristics will be available from the census survey conducted in the first phase of the project, the baseline cross-sectional survey, and the household survey for the cohort study. Data from each source will be separately tabulated by trial arm and by stratum. A trial profile will be produced. This will show, by trial arm, the numbers of households screened, eligible and enrolled for the cross-sectional surveys, plus numbers not enrolled (with reasons); the numbers of children within each age group (<5 years and 5-15 years) recruited for the cross-sectional surveys. For the cohort study, the trial profile will show the numbers of households and children screened, eligible and enrolled, plus numbers not enrolled (with reasons). The number of six-monthly visits at which clinical evaluations were conduced and the number of monthly visits at which household questionnaires were completed will be tabulated by study arm, both overall and separately for each time point.

12.2.3. Cross-sectional survey outcomes

The primary outcome for the cross-sectional surveys will be the prevalence of anemia (defined as hemoglobin < 11.0 g/dL). At each endpoint (one and two years after baseline), the crude prevalence of anemia will be tabulated for each cluster. There are equal numbers of clusters per intervention arm in each stratum and equal numbers of participants will be sampled from each cluster, so the simple mean prevalence across the 10 clusters per intervention arm will be used to calculate point estimates for overall prevalence of anemia in each intervention arm. A risk ratio for the effect of the intervention will then be calculated directly from these point estimates. If necessary, a logarithmic transformation will be applied to normalize cluster-specific prevalences before analyzing the data. A stratified t-test will be used to compare the means of the cluster-specific proportions, where the within-stratum between-cluster variance will be estimated as the residual mean square from a two-way analysis of variance of the log-prevalences on stratum and treatment arm, including an interaction term. A 95% confidence interval (CI) for the risk ratio, adjusting for stratum, will be calculated from this variance using a t-statistic with 16 degrees of freedom.

Adjusted analysis for the effect of the intervention on prevalence of anemia will also be performed to account for any baseline imbalances between groups. We will adjust for the cluster-specific prevalence of anemia collected at the baseline cross sectional survey. Additional a priori individual-level factors to be adjusted for are age group, sex, use of insecticide treated nets, and distance to the health facility. Any other individual- or cluster-specific factors found to be unbalanced at baseline will also be adjusted for. Adjustment will be performed by fitting a logistic regression model, including terms for stratum and the covariates to be adjusted for, but no term for intervention arm, to data from all clusters. From this the predicted prevalence of anemia for each cluster will be available. The ratio between the observed and predicted prevalence will be calculated (risk ratio-residuals). A stratified t-test will then be conducted on the risk ratio-residuals and the covariate-adjusted risk ratio (and 95% CI) for the effect of the intervention will be calculated by applying the two-way analysis of variance method described above to the residuals.

A secondary analysis of individual-level data will be implemented by fitting generalized estimating equation (GEE) models with an exchangeable correlation structure and robust standard errors, and including a fixed effect for stratum. Wald tests will be used to calculate p-values and 95% confidence intervals for the odds ratio for the effect of the intervention. Approaches based on individual-level data have been found to be less robust than the cluster-level approach, therefore results will be treated with caution.

It is possible that community-based interventions (CBI) may be scaled up during the study period. If this scaling-up is implemented approximately at the one year cross-sectional survey it will be possible to examine its impact by comparing outcomes from one to two years in the individual-level regression analysis described above, although the study has not been powered for this comparison. Otherwise it will not be possible to quantify the impact of any scaling-up of these activities on cross-sectionally collected outcomes.

12.2.4. Cohort study outcomes

The primary outcome for the cohort study will be antimalarial treatment incidence density, defined as the rate of antimalarial treatments administered to children in the cohort over the period of follow up. The number of events, child-years of follow-up and corresponding incidence rate will be tabulated by cluster. For each intervention arm, the cluster-specific rates will then be averaged to

give a point estimate of the rate for each intervention. Rate ratios for the effect of the intervention on each outcome will then be calculated from these point estimates. The distribution of cluster-specific rates is likely to be skewed, therefore a logarithmic transformation will be applied to normalize rates before analysis. A test of the null hypothesis that the rate ratio is equal to one will be conducted using a stratified t-test, where the within-stratum between-cluster variance will be estimated as the residual mean square from a two-way analysis of variance of the log-rates on stratum and treatment arm, including an interaction term. A 95% confidence interval (CI) for the rate ratio will be calculated from this variance using a t-statistic with 16 degrees of freedom.

Adjusted analysis will be performed to account for any baseline differences between groups. A priori individual-level factors to be adjusted for are baseline anemia, age group, sex, use of insecticide treated nets, and distance to the health facility. Any other individual- or cluster-specific factors found to be unbalanced between the intervention arms at baseline will also be adjusted for. Adjusted rate ratios will be calculated using a similar two-stage process as that described for the cross-sectional outcomes above, except Poisson regression will be used to calculate predicted rates and hence rate ratio residuals.

For cohort study outcomes, it will be possible to allow for changes in underlying rates of these events due to scaling-up of CBIs in the study area. This will be done by splitting the follow-up time for each cluster into periods representing pre- and post-scale-up, and examining the effect of the intervention on event rates, stratified by time-period.

12.2.5. Outcomes measured at health facilities

The primary outcome collected at health facilities will be inappropriate treatment of malaria, as quantified by the proportion of children under five with suspected malaria and a negative RDT result that are inappropriately treated with an ACT plus the proportion of children under five with suspected malaria and a positive RDT result that are not treated with an ACT. These data will be collected every six months at exit interviews from health facilities.

For each time point, the proportion will be tabulated by cluster, and the cluster-specific mean proportions will be averaged to give a point estimate of proportion in appropriately treated with an ACT in each intervention arm. The risk ratio for the impact of the intervention will then be calculated. The two-way analysis of variance and stratified t-test approach described above for the cross-sectional survey prevalence outcomes will then be applied to test the null hypothesis that the risk ratio equals one and to derive a 95% CI for the risk ratio. Adjusted analyses will also be performed to account for any baseline differences between the study arms, using the same two-stage approach described above. A priori individual-level factors to be adjusted for are age group and sex.

Scaling-up of CBIs in the study area is unlikely to impact on outcomes measured at exit interviews from health facilities, therefore no further analysis is planned to account for this.

13 DATA & SAFETY MONITORING BOARD

13.1. OVERVIEW

A data and safety monitoring board (DSMB) will be assembled in conjunction with the LSHTM and the ACT Consortium, consisting of a minimum of three members who are independent of the project and who have made no significant input into the project's design. Members will include a chairman, a statistician, a clinical monitor, and a local safety monitor, if needed. The DSMB, investigators, and the sponsor will agree on the rules for reporting safety data during the course of the project, and the rules for recommending premature termination of the project on grounds of safety or efficacy. The DSMB will review the analytical plan and will agree to review the interim reported data at pre-specified intervals.

13.2. MONITORING PLAN

We plan to prepare an interim report for review by the DSMB after approximately one half of the total projected follow-up time has been observed; specifically, after the HFI has been fully active for approximately one year, the first annual cross-sectional survey has been completed, and over 75% of the cohort study participants have been followed for one year. The interim report will contain information on study progress and data quality (including recruitment, follow-up, and protocol adherence), safety data (serious adverse events), and primary outcome data (prevalence of anemia, antimalarial treatment incidence density, inappropriate use of ACTs). A shell interim report will be prepared by project members in conjunction with our statisticians, and will be submitted to the DSMB for review prior to the start of the study.

13.3. STOPPING GUIDELINES

The DSMB will have the authority to recommend cessation of the project for reasons related to safety of trial subjects, and to notify the sponsor accordingly. Interpretation of results and decisions about discontinuation of the study will be made by the members of the DSMB.

14 DATA MANAGEMENT

14.1. DATA MANAGEMENT

14.1.1. Cross-sectional surveys

Screening forms, consent forms, survey questionnaires, and clinical case record forms will be completed by the field teams. Microscopy results will be recorded in a laboratory record book by lab technicians. All record forms will be reviewed by project coordinators for completeness and accuracy. Data entered onto paper record forms will be entered into a computerized database (Microsoft Access) by a data entry clerk and will be double-entered to verify accuracy. Electronic versions of the cross-sectional survey questionnaire will be created for personal digital assistants (PDAs) using appropriate software (Visual CE, Syware Inc). Survey teams will move in pairs; one team member will administer the survey questionnaire and record answers on a PDA, while another team member will record answers on a paper questionnaire. Data captured on PDAs will be downloaded daily to a Microsoft Access database. Data captured on paper record forms will be used as back-up if synchronization of the PDA to the computerized database fails.

14.1.2. Cohort study

Screening forms, consent forms, household survey forms, clinical case record forms, and monthly questionnaires will be completed by the field teams. Information recorded on the pictorial diaries by primary caregivers will be transferred onto standardized data extraction forms by study personnel. Microscopy results will be recorded in a laboratory record book by lab technicians. All record forms will be reviewed by project coordinators for completeness and accuracy. Data entered onto paper record forms and the data extraction forms will be entered into a computerized database (Microsoft Access) by a data entry clerk and will be double-entered to verify accuracy. Electronic versions of the household survey questionnaire and the monthly questionnaire will be created for the PDAs using appropriate software (Visual CE, Syware Inc). Field teams will move in pairs; one team member will administer the appropriate questionnaire and record answers on a PDA, while another team member will record answers on a paper version of the questionnaire. Data captured on PDAs will be downloaded daily to a Microsoft Access database. Data captured on paper record forms will be used as back-up if synchronization of the PDA to the computerized database fails.

14.1.3. M&E of health facilities

The patient exit interviews, health facility surveillance forms, and health worker knowledge questionnaires will be completed by field teams. All record forms will be reviewed by project coordinators for completeness and accuracy. Data entered onto paper record forms and the data extraction forms will be entered into a computerized database (Microsoft Access) by a data entry clerk and will be double-entered to verify accuracy. Electronic versions of the health facility surveillance forms will be created for the PDAs using appropriate software (Visual CE, Syware Inc). Data captured on PDAs will be downloaded daily to a Microsoft Access database. Data captured on

paper record forms will be used as back-up if synchronization of the PDA to the computerized database fails.

14.2. QUALITY ASSURANCE AND QUALITY CONTROL

All members of the study team will be trained in the project objectives, methods of effective communication with study participants, and collection of high quality data. Study team members will receive additional training specific to the tasks they will perform within the project including interviewing techniques, administration of surveys, completing questionnaires, and use of PDA devices. Standard Operating Procedures (SOPs) will be written for all project activities and booklets of all relevant documents will be provided to each member of the project team. Study group meetings will be conducted by the principal investigator to assess progress of the study, address any difficulties, and provide performance feedback to the members of the study group. Any corrections to data collection forms will be made by striking through the incorrect entry with a single line and entering the correct information adjacent to it, according to Good Clinical Practice guidelines.[96] The correction will be initialed and dated by the investigator. The investigators will allow all requested monitoring visits, audits or reviews.

14.3. RECORDS AND STORAGE

All study documents will be kept in secured filing cabinets in the Infectious Disease Research Collaboration offices. The principal investigator will be responsible for the security of all project documents. Back-up files of databases will be stored onto the main project server after each data entry session. Participants will be identified by their study ID number, and participant names will not be entered into the computerised database.

15 PROTECTION OF HUMAN PARTICIPANTS

15.1. INSTITUTIONAL REVIEW BOARDS

This study will be reviewed by the following organizations:

1. London School of Hygiene and Tropical Medicine Ethics Committee
2. Makerere University Faculty Research and Ethical Committee
3. Uganda National Council of Science and Technology
4. University of California, San Francisco Committee for Human Research

15.2. INFORMED CONSENT PROCESS

Study personnel will conduct screening interviews and informed consent discussions with individual children and their parent/guardian during the screening process for the cross-sectional surveys and the cohort study. Informed consent will be conducted in the appropriate language (usually Luganda) and a translator will be used if necessary. Consent forms will be available in both English and Luganda. Following the informed consent discussion, parents (or guardians) will be asked by the study personnel to provide their written consent on the approved informed consent document for their child to participate in a research study and a second approved consent form for the future use of biological specimens obtained during the course of the study. If the parent or guardian is unable to read or write, their fingerprint will substitute for a signature, and a signature from a witness to the informed consent procedures will be obtained. Written assent to participate in the cross-sectional surveys will also be obtained from children aged 8 years and older at the time of screening.

15.3. CONFIDENTIALITY

Parents and guardians will be informed that participation in a research study may involve a loss of privacy. All records will be kept as confidential as possible. Participants will be identified primarily by their study number and patient names will not be entered into the computerized database. No individual identities will be used in any reports or publications resulting from the study.

15.4. RISKS AND DISCOMFORTS

15.4.1. Privacy

Care will be taken to protect the privacy of participants, as described in this protocol. However, there is a risk that others may inadvertently see participants' information, and thus their privacy compromised.

15.4.2. Risks of randomization

This will be a randomized trial in which we propose to enhance the care provided at government-run health facilities. There is a risk that residents living in villages within clusters randomized to the standard care arm may receive health care that is inappropriate, ineffective, or delayed, given that care delivered from those facilities will not be enhanced. However, it is not clear if care provided by HFI facilities will improve the health of children or quality of care above care standardly available; this study is designed to answer that question.

15.4.3. Fingerprick blood draws

Participants in the cross-sectional surveys and cohort study will have blood removed by fingerprick for laboratory evaluations, which has been associated with discomfort, bleeding, bruising, and rarely infection.

15.4.4. Risk of artemether-lumefantrine

AL appears to be safe and well-tolerated,[97,98] which is supported by the results of studies in Uganda.[35,36,99,100] In one study, participants treated with AL were more likely to have elevated temperature than those treated with AQ+SP or AQ+AS, which may have been due to the known anti-pyretic properties of amodiaquine, a component of the other two study regimens.[100] In addition, a higher risk of delayed diarrhea was observed in AL-treated patients, which has previously been reported.[101,102]

In a review of the safety and tolerability of AL in 1869 patients (including 368 children aged < 5 years), the most commonly reported adverse events that were possibly related to AL included gastrointestinal events (abdominal pain, anorexia, nausea, vomiting, and diarrhea), headache, and dizziness, while pruritis and rash occurred uncommonly.[102] Most events were of mild or moderate severity and were similar to symptoms associated with clinical malaria. The tolerability profile of AL compared favourably to other antimalarial drugs, including CQ, mefloquine, SP, and quinine.[102] In a systematic review including eight comparative clinical trials, AL was found to be associated with less vomiting (mild or moderate) than CQ, mefloquine, halofantrine, and AS + mefloquine.[103]

The chemical structure of lumefantrine is similar to halofantrine, raising concern for possible cardiac toxicity with AL. Treatment with halofantrine is associated with changes in cardiac conduction, particularly prolongation of the QT interval, which can produce arrhythmias.[104] However, no serious cardiac toxicity has been reported with AL.[103] A study of cardiac effects in healthy volunteers found a concentration-dependent prolongation of the QTc interval in all participants treated with halofantrine, but no change in the QTc interval after treatment with AL.[105] Electrocardiographic data from clinical trials evaluating AL are encouraging, indicating that the frequency of QTc prolongation with AL is similar to that observed with CQ, mefloquine, and AQ + mefloquine.[102,106,107]

15.4.5. RDTs for malaria

The accuracy of RDTs for diagnosing malaria has been well-studied.[108-112] In Uganda, RDTs based on histidine-rich protein 2 (HRP2) and RDTs based on plasmodium lactate dehydrogenase (pLDH) were compared with expert microscopy and polymerase chain reaction (PCR)-corrected microscopy

for 7000 patients at sites of varying malaria transmission intensity across Uganda.[109] When all sites were considered, the sensitivity of the HRP2-based test was 97% when compared with microscopy and 98% when corrected by PCR; the sensitivity of the pLDH-based test was 88% when compared with microscopy and 77% when corrected by PCR. The specificity of the HRP2-based test was 71% when compared with microscopy and 88% when corrected by PCR; the specificity of the pLDH-based test was 92% when compared with microscopy and >98% when corrected by PCR. Based on Plasmodium falciparum PCR-corrected microscopy, the positive predictive value (PPV) of the HRP2-based test was high (93%) at all but the site with the lowest transmission rate; the pLDH-based test and expert microscopy offered excellent PPVs (98%) for all sites. The negative predictive value (NPV) of the HRP2-based test was consistently high (>97%); in contrast, the NPV for the pLDH-based test dropped significantly (from 98% to 66%) as transmission intensity increased, and the NPV for expert microscopy decreased significantly (99% to 54%) because of increasing failure to detect subpatent parasitemia

15.4.6. Compensation

Participants will not be paid for participating in this study. Most assessments will be conducted at households or health facilities, which will eliminate the need for travel and minimize opportunity costs for the participants. If cross-sectional survey or cohort study participants are referred by study personnel to a health facility for further assessment, transportation will either be provided by the study team, or the costs of transportation will be borne by the project. Any other costs of transportation related to project activities will be reimbursed by the project. Households participating in the cohort study will receive small incentives (soap, washing powder, or sugar) at monthly visits. All children participating in the cohort study will be given an insecticide-treated bednet at the end of study follow-up.

15.4.7. Alternatives

Children whose parents or guardians choose not to participate in this study will not be enrolled in the cross-sectional survey or cohort study. Children excluded from the study will still be eligible for standard care of medical problems as they arise at the government-run health facilities or other available health centers.

16 REFERENCES

1. Greenwood BM, Bojang K, Whitty CJ, Targett GA (2005) Malaria. Lancet 365: 1487-1498.

2. Breman J (2001) The ears of the hippopotamus: manifestations, determinants, and estimates of the malaria burden. Am J Trop Med Hyg 64: 1-11.

3. Breman JG, Alilio MS, Mills A (2004) Conquering the intolerable burden of malaria: what's new, what's needed: a summary. Am J Trop Med Hyg 71: 1-15.

4. Snow RW, Craig M, Deichmann U, Marsh K (1999) Estimating mortality, morbidity and disability due to malaria among Africa's non-pregnant population [see comments]. Bull World Health Organ 77: 624-640.

5. Greenwood BM, Bradley AK, Greenwood AM, Byass P, Jammeh K, et al. (1987) Mortality and morbidity from malaria among children in a rural area of The Gambia, West Africa. Trans R Soc Trop Med Hyg 81: 478-486.

6. Roll Back Malaria Partnership (2005) Global strategic plan 2005-2015. Geneva: Roll Back Malaria Partnership.

7. UNICEF (2007) Malaria & children: Progress in intervention coverage. New York, NY USA: UNICEF / Roll Back Malaria Partnership.

8. Kager PA (2002) Malaria control: constraints and opportunities. Trop Med Int Health 7: 1042-1046.

9. Moerman F, Lengeler C, Chimumbwa J, Talisuna A, Erhart A, et al. (2003) The contribution of health-care services to a sound and sustainable malaria-control policy. Lancet Infect Dis 3: 99-102.

10. McCombie SC (1996) Treatment seeking for malaria: a review of recent research. Soc Sci Med 43: 933-945.

11. Marsh VM, Mutemi WM, Muturi J, Haaland A, Watkins WM, et al. (1999) Changing home treatment of childhood fevers by training shop keepers in rural Kenya. Trop Med Int Health 4: 383-389.

12. Goodman C, Kachur SP, Abdulla S, Mwageni E, Nyoni J, et al. (2004) Retail supply of malaria-related drugs in rural Tanzania: risks and opportunities. Trop Med Int Health 9: 655-663.

13. World Health Organization (2004) Scaling up home-based management of malaria: from research to implementation. Geneva.

14. World Health Organization (2007) Lessons learned in home management of malaria: Implementation research in four African countries.

15. Ajayi IO, Browne EN, Garshong B, Bateganya F, Yusuf B, et al. (2008) Feasibility and acceptability of artemisinin-based combination therapy for the home management of malaria in four African sites. Malar J 7: 6.

16. World Health Organization (2010) Guidelines for the treatment of malaria. Geneva: World Health Organization.

17. Reyburn H, Ruanda J, Mwerinde O, Drakeley C (2006) The contribution of microscopy to targeting antimalarial treatment in a low transmission area of Tanzania. Malar J 5: 4.

18. Zurovac D, Midia B, Ochola SA, English M, Snow RW (2006) Microscopy and outpatient malaria case management among older children and adults in Kenya. Trop Med Int Health 11: 432-440.

19. Hamer DH, Ndhlovu M, Zurovac D, Fox M, Yeboah-Antwi K, et al. (2007) Improved diagnostic testing and malaria treatment practices in Zambia. Jama 297: 2227-2231.

20. Reyburn H, Mbakilwa H, Mwangi R, Mwerinde O, Olomi R, et al. (2007) Rapid diagnostic tests compared with malaria microscopy for guiding outpatient treatment of febrile illness in Tanzania: randomised trial. Bmj 334: 403.

21. Ssekabira U, Bukirwa H, Hopkins H, Namagembe A, Weaver MR, et al. (2008) Improved malaria case management after integrated team-based training of health care workers in Uganda. Am J Trop Med Hyg 79: 826-833.

22. Hopkins H, Asiimwe C, Bell D (2009) Access to antimalarial therapy: accurate diagnosis is essential to achieving long term goals. BMJ 339: b2606.

23. Msellem MI, Martensson A, Rotllant G, Bhattarai A, Stromberg J, et al. (2009) Influence of rapid malaria diagnostic tests on treatment and health outcome in fever patients, Zanzibar: a crossover validation study. PLoS Med 6: e1000070.

24. Williams HA, Causer L, Metta E, Malila A, O'Reilly T, et al. (2008) Dispensary level pilot implementation of rapid diagnostic tests: an evaluation of RDT acceptance and usage by providers and patients--Tanzania, 2005. Malar J 7: 239.

25. D'Acremont V (2009) Massive reduction of antimalarial prescription after rapid diagnostic test implementation in Dar es Salaam, Tanzania MIM Pan-African Malaria Conference. Nairobi, Kenya.

26. Uganda Ministry of Health (2008) The Burden of Malaria in Uganda.

27. Uganda Ministry of Health (2008) Malaria control and prevention.

28. Fapohunda BM, Plowman BA, Azairwe R, Bisorbowa G, Langi P, et al. (2004) Home-based management of fever strategy in Uganda: A report of the 2003 survey. Arlington, Virginia, USA: MOH, WHO, BASICS II.

29. Mbonye AK (2003) Prevalence of childhood illnesses and care-seeking practices in rural Uganda. ScientificWorldJournal 3: 721-730.

30. Kengeya-Kayondo JF, Seeley JA, Kajura-Bajenja E, Kabunga E, Mubiru E, et al. (1994) Recognition, treatment seeking behaviour and perception of cause of malaria among rural women in Uganda. Acta Trop 58: 267-273.

31. Tumwesigire S, Watson S (2002) Health seeking behavior by families of children suspected to have malaria in Kabale: Uganda. Afr Health Sci 2: 94-98.

32. Ndyomugyenyi R, Magnussen P, Clarke S (2007) Malaria treatment-seeking behaviour and drug prescription practices in an area of low transmission in Uganda: implications for prevention and control. Trans R Soc Trop Med Hyg 101: 209-215.

33. Tren R, Hess K, Bate R (2009) Drug procurement, the Global Fund and misguided competition policies. Malar J 8: 305.

34. Dorsey G, Staedke S, Clark TD, Njama-Meya D, Nzarubara B, et al. (2007) Combination therapy for uncomplicated falciparum malaria in Ugandan children: a randomized trial. Jama 297: 2210-2219.

35. Kamya MR, Yeka A, Bukirwa H, Lugemwa M, Rwakimari JB, et al. (2007) Artemether-Lumefantrine versus Dihydroartemisinin-Piperaquine for Treatment of Malaria: A Randomized Trial. PLoS Clin Trials 2: e20.

36. Yeka A, Dorsey G, Kamya MR, Talisuna A, Lugemwa M, et al. (2008) Artemether-lumefantrine versus dihydroartemisinin-piperaquine for treating uncomplicated malaria: a randomized trial to guide policy in Uganda. PLoS ONE 3: e2390.

37. Zurovac D, Tibenderana J, Nankabirwa J, Ssekitooleko J, Talisuna A, et al. (2008) Evaluation of outpatient malaria case-management under artemether-lumefantrine treatment policy in Uganda. Malaria Consortium, Uganda Ministry of Health, KEMRI/Wellcome Trust Collaborative Programme, Kenya.

38. Kindermans JM, Pilloy J, Olliaro P, Gomes M (2007) Ensuring sustained ACT production and reliable artemisinin supply. Malar J 6: 125.

39. Moszynski P Disappearance of drugs undermines Uganda's fight against malaria. BMJ 340: c2611.

40. Uganda Ministry of Health (2004) Implementation guidelines for the home based management of fever strategy. 2nd edition.

41. Kolaczinski JH, Ojok N, Opwonya J, Meek S, Collins A (2006) Adherence of community caretakers of children to pre-packaged antimalarial medicines (HOMAPAK) among internally displaced people in Gulu district, Uganda. Malar J 5: 40.

42. Institute of Medicine (2001) Crossing the quality chasm: a new health system for the 21st Century Washington, DC.

43. Kelly JM, Osamba B, Garg RM, Hamel MJ, Lewis JJ, et al. (2001) Community health worker performance in the management of multiple childhood illnesses: Siaya District, Kenya, 1997-2001. Am J Public Health 91: 1617-1624.

44. Zurovac D, Rowe AK (2006) Quality of treatment for febrile illness among children at outpatient facilities in sub-Saharan Africa. Ann Trop Med Parasitol 100: 283-296.

45. Leonard K, Masatu M, Vialou A (2005) Getting clinicians to do their best: Ability, Altruism and Incentives.: Working paper, University of Maryland.

46. Chakraborty S, Frick K (2002) Factors influencing private health providers' technical quality of care for acute respiratory infections among under-five children in rural West Bengal, India. Soc Sci Med 55: 1579-1587.

47. Arifeen SE, Bryce J, Gouws E, Baqui AH, Black RE, et al. (2005) Quality of care for under-fives in first-level health facilities in one district of Bangladesh. Bull World Health Organ 83: 260-267.

48. Krause G, Schleiermacher D, Borchert M, Benzler J, Heinmuller R, et al. (1998) Diagnostic quality in rural health centres in Burkina Faso. Trop Med Int Health 3: 100-107.

49. World Health Organisation (2000) World Health Report 2000. Health Systems: Improving Performance. Geneva.

50. Brown LD, Franco LM, Rafeh N, Hatzell T (1993) Quality Assurance of Health Care in Developing Countries. Bethesda, MD: Quality Assurance Project. Online at http://www.qaproject.org/pubs/PDFs/DEVCONT.pdf. Accessed 24 July 2008.

51. Hall JA, Epstein AM, DeCiantis ML, McNeil BJ (1993) Physicians' liking for their patients: more evidence for the role of affect in medical care. Health Psychol 12: 140-146.

52. Mbaruku G, Bergstrom S (1995) Reducing maternal mortality in Kigoma, Tanzania. Health Policy Plan 10: 71-78.

53. McPake B (1993) User charges for health services in developing countries: a review of the economic literature. Soc Sci Med 36: 1397-1405.

54. Wouters AV (1991) Essential national health research in developing countries: health care financing and the quality of care. Int J Health Planning and Management 6: 253-271.

55. El Arifeen S, Blum LS, Hoque DM, Chowdhury EK, Khan R, et al. (2004) Integrated Management of Childhood Illness (IMCI) in Bangladesh: early findings from a cluster-randomised study. Lancet 364: 1595-1602.

56. Deyo RA, Inui TS (1980) Dropouts and broken appointments. A literature review and agenda for future research. Med Care 18: 1146-1157.

57. Vera H (1993) The client's view of high-quality care in Santiago, Chile. Stud Fam Plann 24: 40-49.

58. Williams B (1994) Patient satisfaction: a valid concept? Soc Sci Med 38: 509-516.

59. Ashworth A, Chopra M, McCoy D, Sanders D, Jackson D, et al. (2004) WHO guidelines for management of severe malnutrition in rural South African hospitals: effect on case fatality and the influence of operational factors. Lancet 363: 1110-1115.

60. Puoane T, Sanders D, Ashworth A, Chopra M, Strasser S, et al. (2004) Improving the hospital management of malnourished children by participatory research. Int J Qual Health Care 16: 31-40.

61. Howie SR, Hill SE, Peel D, Sanneh M, Njie M, et al. (2008) Beyond good intentions: lessons on equipment donation from an African hospital. Bull World Health Organ 86: 52-56.

62. Chandler CI, Whitty CJ, Ansah EK (2010) How can malaria rapid diagnostic tests achieve their potential? A qualitative study of a trial at health facilities in Ghana. Malar J 9: 95.

63. Soumerai SB, McLaughlin TJ, Avorn J (1989) Improving drug prescribing in primary care: a critical analysis of the experimental literature. Milbank Q 67: 268-317.

64. Oxman AD, Thomson MA, Davis DA, Haynes RB (1995) No magic bullets: a systematic review of 102 trials of interventions to improve professional practice. Cmaj 153: 1423-1431.

65. Davis DA, Thomson MA, Oxman AD, Haynes RB (1995) Changing physician performance. A systematic review of the effect of continuing medical education strategies. JAMA 274: 700-705.

66. Grimshaw J, Shirran L, Thomas R, Mowatt G, Fraser C, et al. (2002) Changing provider behaviour: an overview of systematic reviews of interventions to promote implementation of research findings by healthcare professionals. In: Haines A, Donald A, editors. Getting Research Findings into Practice. 2nd ed. London: BMJ Books. pp. 29-68.

67. Smith LA, Jones C, Meek S, Webster J (2009) Review: Provider practice and user behavior interventions to improve prompt and effective treatment of malaria: do we know what works? Am J Trop Med Hyg 80: 326-335.

68. Ross-Degnan D, Laing R, Santoso B, Ofori-Adjei D, Lamoureux C, et al. (1997) Improving pharmaceutical use in primary care in developing counties: a critical review of experience and lack of experience. Chiang Mai, Thailand, April

69. Ofori-Adjei D, Arhinful DK (1996) Effect of training on the clinical management of malaria by medical assistants in Ghana. Soc Sci Med 42: 1169-1176.

70. Rowe SY, Kelly JM, Olewe MA, Kleinbaum DG, McGowan JE, Jr., et al. (2007) Effect of multiple interventions on community health workers' adherence to clinical guidelines in Siaya district, Kenya. Trans R Soc Trop Med Hyg 101: 188-202.

71. Rowe SY, Olewe MA, Kleinbaum DG, McGowan JE, Jr., McFarland DA, et al. (2007) Longitudinal analysis of community health workers' adherence to treatment guidelines, Siaya, Kenya, 1997-2002. Trop Med Int Health 12: 651-663.

72. Armstrong Schellenberg J, Bryce J, de Savigny D, Lambrechts T, Mbuya C, et al. (2004) The effect of Integrated Management of Childhood Illness on observed quality of care of under-fives in rural Tanzania. Health Policy Plan 19: 1-10.

73. Amaral J, Gouws E, Bryce J, Leite AJ, Cunha AL, et al. (2004) Effect of Integrated Management of Childhood Illness (IMCI) on health worker performance in Northeast-Brazil. Cad Saude Publica 20 Suppl 2: S209-219.

74. Gilroy K, Winch PJ, Diawara A, Swedberg E, Thiero F, et al. (2004) Impact of IMCI training and language used by provider on quality of counseling provided to parents of sick children in Bougouni District, Mali. Patient Educ Couns 54: 35-44.

75. Gouws E, Bryce J, Habicht JP, Amaral J, Pariyo G, et al. (2004) Improving antimicrobial use among health workers in first-level facilities: results from the multi-country evaluation of the Integrated Management of Childhood Illness strategy. Bull World Health Organ 82: 509-515.

76. Naimoli JF, Rowe AK, Lyaghfouri A, Larbi R, Lamrani LA (2006) Effect of the Integrated Management of Childhood Illness strategy on health care quality in Morocco. Int J Qual Health Care 18: 134-144.

77. Pariyo GW, Gouws E, Bryce J, Burnham G (2005) Improving facility-based care for sick children in Uganda: training is not enough. Health Policy Plan 20 Suppl 1: i58-i68.

78. Perez-Cuevas R, Guiscafre H, Munoz O, Reyes H, Tome P, et al. (1996) Improving physician prescribing patterns to treat rhinopharyngitis. Intervention strategies in two health systems of Mexico. Soc Sci Med 42: 1185-1194.

79. Zurovac D, Rowe AK, Ochola SA, Noor AM, Midia B, et al. (2004) Predictors of the quality of health worker treatment practices for uncomplicated malaria at government health facilities in Kenya. Int J Epidemiol 33: 1080-1091.

80. Fonn S, Mtonga AS, Nkoloma HC, Bantebya Kyomuhendo G, daSilva L, et al. (2001) Health providers' opinions on provider-client relations: results of a multi-country study to test Health Workers for Change. Health Policy Plan 16 Suppl 1: 19-23.

81. Manongi RN, Nasuwa FR, Mwangi R, Reyburn H, Poulsen A, et al. (2009) Conflicting priorities: evaluation of an intervention to improve nurse-parent relationships on a Tanzanian paediatric ward. Hum Resour Health 7: 50.

82. Labhardt ND, Manga E, Ndam M, Balo JR, Bischoff A, et al. (2009) Early assessment of the implementation of a national programme for the prevention of mother-to-child transmission of HIV in Cameroon and the effects of staff training: a survey in 70 rural health care facilities. Trop Med Int Health 14: 288-293.

83. Marvel MK, Epstein RM, Flowers K, Beckman HB (1999) Soliciting the patient's agenda - Have we improved? Jama-Journal of the American Medical Association 281: 283-287.

84. Fogarty LA, Curbow BA, Wingard JR, McDonnell K, Somerfield MR (1999) Can 40 seconds of compassion reduce patient anxiety? J Clin Oncol 17: 371-379.

85. Di Blasi Z, Harkness E, Ernst E, Georgiou A, Kleijnen J (2001) Influence of context effects on health outcomes: a systematic review. Lancet 357: 757-762.

86. Thomas KB (1987) General practice consultations: is there any point in being positive? Br Med J (Clin Res Ed) 294: 1200-1202.

87. Fassaert T, van Dulmen S, Schellevis F, van der Jagt L, Bensing J (2008) Raising positive expectations helps patients with minor ailments: a cross-sectional study. BMC Fam Pract 9: 38.

88. Krasner MS, Epstein RM, Beckman H, Suchman AL, Chapman B, et al. (2009) Association of an educational program in mindful communication with burnout, empathy, and attitudes among primary care physicians. Jama 302: 1284-1293.

89. World Health Organization (2002) Community involvement in rolling back malaria. Roll Back Malaria/World Health Organization. WHO/CDS/RBM/2002.2042 p.

90. Davis JC, Clark TD, Kemble SK, Talemwa N, Njama-Meya D, et al. (2006) Longitudinal study of urban malaria in a cohort of Ugandan children: description of study site, census and recruitment. Malar J 5: 18.

91. Dorsey G, Njama D, Kamya MR, Rosenthal P. A longitudinal assessment of different first-line therapies for uncomplicated falciparum malaria in Kampala, Uganda; 2001; Atlanta, Georgia.

92. Staedke SG, Mwebaza N, Kamya MR, Clark TD, Dorsey G, et al. (2009) Home management of malaria with artemether-lumefantrine compared with standard care in urban Ugandan children: a randomised controlled trial. Lancet 373: 1623-1631.

93. Okello PE, W VANB, Byaruhanga AM, Correwyn A, Roelants P, et al. (2006) Variation in malaria transmission intensity in seven sites throughout Uganda. Am J Trop Med Hyg 75: 219-225.

94. Pullan RL, Bukirwa H, Staedke SG, Snow RW, Brooker S (2010) Plasmodium infection and its risk factors in eastern Uganda. Malar J 9: 2.

95. Wiseman V, Conteh L, Matovu F (2005) Using diaries to collect data in resource-poor settings: questions on design and implementation. Health Policy Plan 20: 394-404.

96. International Conference on Harmonisation of Technical Requirements for Registration of Pharmaceuticals for Human Use (ICH) (1996) Guidance for Industry: Good Clinical Practice: Consolidated Guidance (ICH-E6). Rockville, MD: U.S. Department of Health and Human Services, Food and Drug Administration, Center for Drug Evaluation and Research (CDER), Center for Biologics Evaluation and Research (CBER).

97. Makanga M, Premji Z, Falade C, Karbwang J, Mueller EA, et al. (2006) Efficacy and safety of the six-dose regimen of artemether-lumefantrine in pediatrics with uncomplicated Plasmodium falciparum malaria: a pooled analysis of individual patient data. Am J Trop Med Hyg 74: 991-998.

98. Omari AA, Gamble C, Garner P (2005) Artemether-lumefantrine (six-dose regimen) for treating uncomplicated falciparum malaria. Cochrane Database Syst Rev: CD005564.

99. Piola P, Fogg C, Bajunirwe F, Biraro S, Grandesso F, et al. (2005) Supervised versus unsupervised intake of six-dose artemether-lumefantrine for treatment of acute, uncomplicated Plasmodium falciparum malaria in Mbarara, Uganda: a randomised trial. Lancet 365: 1467-1473.

100. Maiteki-Sebuguzi C, Jagannathan P, Yau VM, Clark TD, Njama-Meya D, et al. (2008) Safety and tolerability of combination antimalarial therapies for uncomplicated falciparum malaria in Ugandan children. Malar J 7: 106.

101. Novartis (2003) Coartem monograph. Basel, Switzerland.

102. Bakshi R, Hermeling-Fritz I, Gathmann I, Alteri E (2000) An integrated assessment of the clinical safety of artemether-lumefantrine: a new oral fixed-dose combination antimalarial drug. Trans R Soc Trop Med Hyg 94: 419-424.

103. Omari AA, Gamble C, Garner P (2004) Artemether-lumefantrine for uncomplicated malaria: a systematic review. Trop Med Int Health 9: 192-199.

104. Nosten F, Price RN (1995) New antimalarials. A risk-benefit analysis. Drug Saf 12: 264-273.

105. Bindschedler M, Lefevre G, Degen P, Sioufi A (2002) Comparison of the cardiac effects of the antimalarials co-artemether and halofantrine in healthy participants. Am J Trop Med Hyg 66: 293-298.

106. van Vugt M, Ezzet F, Nosten F, Gathmann I, Wilairatana P, et al. (1999) No evidence of cardiotoxicity during antimalarial treatment with artemether-lumefantrine. Am J Trop Med Hyg 61: 964-967.

107. Lefevre G, Looareesuwan S, Treeprasertsuk S, Krudsood S, Silachamroon U, et al. (2001) A clinical and pharmacokinetic trial of six doses of artemether-lumefantrine for multidrug-resistant Plasmodium falciparum malaria in Thailand. Am J Trop Med Hyg 64: 247-256.

108. Drakeley C, Reyburn H (2009) Out with the old, in with the new: the utility of rapid diagnostic tests for malaria diagnosis in Africa. Trans R Soc Trop Med Hyg 103: 333-337.

109. Hopkins H, Bebell L, Kambale W, Dokomajilar C, Rosenthal PJ, et al. (2008) Rapid diagnostic tests for malaria at sites of varying transmission intensity in Uganda. J Infect Dis 197: 510-518.

110. Hopkins H, Kambale W, Kamya MR, Staedke SG, Dorsey G, et al. (2007) Comparison of HRP2- and pLDH-based rapid diagnostic tests for malaria with longitudinal follow-up in Kampala, Uganda Am J Trop Med Hyg 76: 1092-1097.

111. Murray CK, Gasser RA, Jr., Magill AJ, Miller RS (2008) Update on rapid diagnostic testing for malaria. Clin Microbiol Rev 21: 97-110.

112. Wongsrichanalai C, Barcus MJ, Muth S, Sutamihardja A, Wernsdorfer WH (2007) A review of malaria diagnostic tools: microscopy and rapid diagnostic test (RDT). Am J Trop Med Hyg 77: 119-127.

17 APPENDICES

Appendix A: Intervention components, barriers addressed, and intended outcomes
Appendix B: Health facility intervention information sheet
Appendix C: MoH Staffing Norms policy
Appendix D: Primary health care funds accounting tool
Appendix E: ACT Drug Distribution Assessment Tool (ADDAT)
Appendix F: Health facility monthly report
Appendix G: User's manual: Use of Rapid Diagnostic Tests (RDTs) for malaria in fever case management in Uganda
Appendix H: Health worker pre-post training assessment
Appendix I: Health worker self-observational information sheet
Appendix J: Health worker self-observational task sheets (1-4)
Appendix K: Cross-sectional survey screening form
Appendix L: Cross-sectional survey informed consent to participate in a research study
Appendix M: Cross-sectional survey informed consent for future use of biological specimens
Appendix N: Cross-sectional survey assent form
Appendix O: Cross-sectional survey questionnaire form
Appendix P: Cross-sectional survey case record form
Appendix Q: Cohort study screening form
Appendix R: Cohort study informed consent to participate in a research study
Appendix S: Cohort study informed consent for future use of biological specimens
Appendix T: Cohort study case record form
Appendix U: Cohort study household survey
Appendix V: Cohort study pictorial diaries
Appendix W: Cohort study monthly questionnaires
Appendix X: Exit interview screening form
Appendix Y: Exit interview informed consent to participate in a research study
Appendix Z: Patient exit interview questionnaire form
Appendix AA: Health facility surveillance information sheet
Appendix BB: Health facility surveillance questionnaire form (Parts 1 & 2)
Appendix CC: Health care worker knowledge questionnaire information sheet
Appendix DD: Health care worker knowledge questionnaire form
Appendix EE: WHO toxicity grading scale
Appendix FF: UMSP toxicity grading scale
Appendix GG: Serious adverse event report form
Appendix HH: Suspected adverse drug reaction report form

APPENDIX A: Intervention components, barriers, and outcomes

Intervention Component	Barriers intended to address	Intended outcome
1. Health Centre Management		
Management training for in-charges:<br>• Human resource management (staffing)<br>• Financial management (budgeting and accounting)<br>• Supply management (drug stocking)	• Lack of in-charge skills to manage staff and HC<br>• Low motivation of staff due to poor HC administration<br>• Inappropriate use of volunteers<br>• Under-utilization or lack of appropriate tools to appropriately mange health centres	Good clinical care<br>Improved staff/patient ratio<br>Appropriate use of volunteers<br>Improved accounting of PHC funds<br>Improved forecasting and stocking of needed supplies
2. Information Management		
Monitoring and evaluating information for continuous quality improvement	• Low health worker motivation and performance with patients (clinical and wider therapeutic process)<br>• Lack of information to adequately manage the health facility and report to local and district stakeholders	Professional conduct<br>Good clinical care
3. Health Worker Training		
Training in fever case management and use of RDTs	• Poor knowledge of malaria case management<br>• Inadequate/unavailable infrastructure or diagnostic laboratory facilities	Good clinical care<br>Improved HW-patient interactions<br>Professional conduct<br>Prompt and fair treatment<br>Improved welcoming & guiding<br>Improved HW-patient interactions
Training in Patient-Centered Services	• Communication problems including language barrier<br>• Lack of patient-centred thinking<br>• Discrimination/preferential treatment of patients<br>• Poor patient flow and management<br>• Poor relationships between staff and communities	
4. Supply of Consumables		
Drug delivery from NMS	• Frequent stock-outs of drugs<br>• Poor procurement system<br>• Patients have to pay for drugs or services	Good clinical care<br>Free services<br>Availability of drugs<br>Appropriate treatment of malaria
Support supply artemether-lumefantrine	• Frequent stock-outs of first line antimalarial treatment	
RDTs for malaria	• Inadequate/unavailable infrastructure or diagnostic laboratory facilities<br>• RDTs not available nationally	

APPENDIX B. INFORMATION SHEET – Health facility intervention
ACT PRIME Study

<u>Introduction</u>

Dr. Sarah Staedke and colleagues from the Uganda Malaria Surveillance Project / Infectious Diseases Research Collaboration are investigating delivery of health care services in Tororo District. We are doing a research study to see if we can improve the health of children in this area by improving services at government-run health facilities.

<u>What will be done in this study?</u>

Certain health centers in Tororo district will be selected to take part in the intervention to improve services, or to continue with their current services. Assignment to the two groups has been determined by a lottery. The chance of being placed into either of the groups is the same. Your health center has been selected to take part in the intervention.

The health facility intervention will focus on 1) health center management training, 2) information management and quality improvement; 3) health worker training, and 4) supply of malaria diagnostics and antimalarial drugs. The intervention package will be introduced to all health centers selected to take part over approximately 8-10 weeks. After this time, all health centers in the area will be assessed every two months to determine how well the intervention is working. Our study personnel will continue to support the intervention at the selected health centers for the full duration of the study.

<u>What will happen if my health center takes part in this study?</u>

The intervention will include three different training packages, which you and other members of staff at your facility will be asked to take part in:

1) Health center management training. All in-charges of health centers assigned to the intervention will be trained in health center management. The purpose of this training is to equip in-charges with the skills and tools required to effectively and efficiently manage their health center. The training will include three components: human resource management, financial management, and supply management. Our study staff will carry out a series of half-day training workshops with the in-charges every 1-2 weeks, and the training module will last approximately 8-10 weeks. The in-charges will be taught how to collect data about patient attendance, primary health care (PHC) funds, and drug supplies during the training. The in-charges will also be asked to continue to collect data and complete forms during the full period of the study.

2) Fever case management training. All clinical staff, including in-charges and other health workers, will receive training in fever case management. The training program will last 1 week.

3) Patient-centered services (PCS) training. The purpose of this training is to identify and improve interpersonal interactions between health workers and patients. All clinical staff, including in-charges, will receive the full PCS training package which will start with a series of 4 self-observation activities completed over a period of 8-12 weeks. This will be followed by a PCS workshop which will consist of 4 half-day modules, lasting about 3 hours, carried out over 2 weeks. The modules will focus on coaching, communication with patients and colleagues, and patient flow. Support staff, including volunteers will receive a scaled-down PCS training package with specific emphasis on communication skills and patient flow. The PCS workshop for support staff will consist of 2 half-day themed modules carried out over 1 week. Health workers and support staff will be trained in two groups to ensure that work at the health centers continues alongside the training.

After beginning the intervention, we will collect information to see how well it is working. Every month, we will visit the health centers to gather information on patient attendance, drug stock-outs, staffing, and diagnosis and treatment of malaria. Every six months, we will interview patients as they are leaving the health center to learn more about their experience with the health center visit. Once a year, we will ask all health workers to take part in a survey about management of malaria and other fever illnesses to learn more about their knowledge and practices.

How long will this study last?

The intervention will be introduced over 2-3 months. The total duration of the study will be about 2 ½ years.

Can I stop being in the study?

You can decide to stop participating at any time. Just tell our study personnel right away if you wish to stop the activities.

What risks can I expect from participating in the study?

Participation in any research study may involve a loss of privacy. Information you provide about your health center will be recorded, but your name will not be used in any reports of the information provided. No quotes or other results arising from your participation in this study will be included in any reports, even anonymously, without your agreement. The information obtained from these study activities will only be used by the project researchers and will be locked up at our project offices. We will do our best to make sure that any personal information is kept private.

Are there benefits to taking part in the study?

Through the intervention, we aim to improve the health of children in this area by improving services at the health centers. As part of the intervention, our project will provide training to in-charges and other health workers, which will benefit you directly, and help provide malaria drugs and tests for diagnosing malaria, which will benefit the health facilities and patients. The information that we gather in this study will help researchers and policy-makers understand how best to improve health services in this area.

What other choices do I have if I do not take part in the study?

You are free to choose not to take part in the study. If you decide not to take part, there will be no penalty to you.

What are the costs of taking part in the study? Will I be paid for taking part in the study?

There are no costs to you for taking part in this study. You will not be paid for taking part in this study. The training activities will take place in convenient locations within Tororo district. Any transport costs incurred by trainees will be reimbursed by the project.

What are my rights if I take part in the study?

Taking part in this study is your choice. You may choose either to take part or not to take part. If you decide to take part in this study, you may change your mind at any time. No matter what decision you take, there will be no penalty to you in any way.

Who can answer my questions about the study?

You can talk to the researchers about any questions or concerns you have about these study activities. Contact Dr. Sarah Staedke or other members of the Uganda Malaria Surveillance Project / Infectious Disease Research Collaboration on telephone number 0414-530692. If you have any questions, comments or concerns about taking part in these self-observation activities, first talk to the researchers. If for any reason you do not wish to do this, or you still have concerns about doing so, you may contact Dr Charles Ibingira, Makerere University Faculty of Medicine Research and Ethical Committee at telephone number 0414-530020.

Giving verbal consent to take part in the study:

You may keep this information sheet if you wish. Participation in these activities is voluntary. You have the right to decline to take part in the activities, or to withdraw from them at any point without penalty. If you do not wish to take part in the activities, you should inform the researcher now. If you do wish to take part in these activities, you should tell the researcher now.

APPENDIX C: MOH STAFFING NORMS POLICY

STAFFING NORMS (AN EXCTRACT FROM THE OFFICIAL MOH HEALTH UNIT PROCEDURES MANUAL, June 2005)

Post	Number recommended per level							
	HC II		HC III		HC IV		General Hospital	
	HSSP I norms	Proposed LG Restr.	HSSP I norms	Proposed LG Restr	HSSP I norms	Proposed LG Restr	HSSP I norms (not indicated)	Proposed LG Restr
Medical Officers								
Principal Medical Officer								1
Medical Officers, special grade(community)								1
Senior Medical Officer						1		1
Medical Officer					1	1		4
Dental								
Dental Surgeon								1
Public Health Dental Officer					1	1		2
Dental Attendant								1
Pharmacy								
Pharmacist								1
Dispensers					1	1		2
Nursing								
Principal Nursing Officer								1
Senior Nursing Officer						1		5
Nursing Officer (Nursing)			1	[illegible]	1	1		17
Nursing Officer (Midwifery)					1	1		3
Nursing Officer (Psychiatry)						1		1
Public Health Nurse						1		1
Enrolled Psychiatric Nurse						1		2
Enrolled Nurse	1	2	2	3	2	3		46
Enrolled Midwives				2		3		25
Nursing Assistant	2	2	1	3	1	5		15
Allied Health Professional								
Senior Clinical Officer				1				1
Clinical Officer			1	1	2	2		5
Psychiatric Clinical Officer								1

Ophthalmic Clinical officer						1		1
Health Inspector					1	1		1
Health Assistant			1	1		1		
Medical Entomology Officer					0.5	1		1
Radiographers								2
Physiotherapist								1
Occupation Therapist								1
Orthopedic Officer								2
Health Educationist/Educator								1
Assistant Health Educator					1	1		1
Aneasthetic Officer					1	1		2
Aneasthetic Attendant/(Theater Attendant?)								2
Senior Laboratory Technologist								1
Laboratory Technologist								1
Laboratory Technician				1	1	1		2
Laboratory Assistant			1	1	1	1		1
Administrative and other staff								
Senior Hospital Administrator								1
Hospital Administrator								1
Personnel Officer								1
Medical Social Worker								1
Nutritionist								1
Supplies Officer								1
Steno-Secretary						1		1
Office Typist								1
Stores Assistant						1		2
Health Information Assistant (Records Assistant)				1	1	1		2
Senior Accounts Assistant								1
Accounts Assistant								2
Support staff								
Cold Chain Assistant						1		
Darkroom Attendant								1

Mortuary Attendant								1
Drivers						1		2
Cooks								3
Guards/Askari		2		2		3		2
Artisan								3
Porters		2		2		3		

APPENDIX D: PRIMARY HEALTH CARE (PHC) FUND ACCOUNTING TOOL

PART 1: Health Worker Information

Health centre code	Health worker ID	Health worker position	Reporting Month / Year
[____|____]	[____|____]	[____|____]<br>Pick code from list below	[____|____]/[____|____]<br>month year

1 = In-charge	4 = Senior clinical officer	7 = Enrolled nurse	10 = Nursing aide/assistant	13 = Health assistant
2 = Senior medical officer	5 = Clinical officer	8 = Midwife	11 = Laboratory technician	14 = Health educator
3 = Medical officer	6 = Nursing officer	9 = Public health nurse	12 = Laboratory assistant	15 = Other__________

The PHC Fund Tracking Tool should be completed monthly by the person responsible for managing the fund.

PART 2: PHC Fund Accounting Tool

Coding for Action or Service required

1 = community outreach	5 = paying volunteers	8 = buying supplies
2 = slashing around the health centre	6 = paying staff	9 = buying drugs
3 = cleaning the health centre	7 = buying fuel	10 = Other
4 = health centre repairs		

☐ Action or Service required<br>List code: [____]	Give details:	
Expected benefit or consequence if action/service cannot be conducted:		
Person responsible for completing the action/service:		
A Cost for action/service	B Frequency required per month (hours or days)	Cost per month (A x B)
/=		/= per month

☐ Action or Service required<br>List code: [____]	Give details:	
Expected benefit or consequence if action/service cannot be conducted:		
Person responsible for completing the action/service:		
A Cost for action/service	B Frequency required per month (hours or days)	Cost per month (A x B)
/=		/= per month

☐ Action or Service required<br>List code: [____]	Give details:	
Expected benefit or consequence if action/service cannot be conducted:		
Person responsible for completing the action/service:		
A Cost for action/service	B Frequency required per month (hours or days)	Cost per month (A x B)
/=		/= per month

Complete another Tracking Tool Sheet if there are additional services or actions required this month.

Total monthly cost for all services/actions	/=

APPENDIX E. ACT DRUG DISTRIBUTION ASSESSMENT TOOL (ADDAT)

PART 1: HEALTH WORKER INFORMATION

Health centre code	Health worker ID	Health worker position	Reporting Month / Year
[____|____]	[____|____]	[____|____]<br>Pick code from list below	[____|____]/[____|____]<br>month year

1 = In-charge	4 = Senior clinical officer	7 = Enrolled nurse	10 = Nursing aide/assistant	13 = Health assistant
2 = Senior medical officer	5 = Clinical officer	8 = Midwife	11 = Laboratory technician	14 = Health educator
3 = Medical officer	6 = Nursing officer	9 = Public health nurse	12 = Laboratory assistant	15 = Other___________

The ADDAT should be completed monthly by the person responsible for drug procurement and distribution.

PART 2: ADDAT

Green: The activity is on track. No intervention is required to ensure this node is operational for distribution of ACTs.	Yellow: The activity is partially on tract or only a component is on track. Some intervention is required for this activity to become operational.	Red: The activity not is not on track. Significant intervention is required for this activity to become operational.
Drug delivery from NMS to DHO	Date:	Green ☐ Yellow ☐ Red ☐
Issue if status is 'yellow' or 'red':		
How the issue was resolved:		Date:
Drug delivery to health center	Date:	Green ☐ Yellow ☐ Red ☐
Issue if status is 'yellow' or 'red':		
How the issue was resolved:		Date:
Adequate storage for drugs	Date:	Green ☐ Yellow ☐ Red ☐
Issue if status is 'yellow' or 'red':		
How the issue was resolved:		Date:
Adequate capacity for managing and distributing drugs	Date:	Green ☐ Yellow ☐ Red ☐
Issue if status is 'yellow' or 'red':		
How the issue was resolved:		Date:
Request re-supply of drugs from DHI	Date:	Green ☐ Yellow ☐ Red ☐
Issue if status is 'yellow' or 'red':		
How the issue was resolved:		Date:
Other activities this month	Date:	Green ☐ Yellow ☐ Red ☐
Describe action:		
Issue if status is 'yellow' or 'red':		
How the issue was resolved:		Date:

APPENDIX F: HEALTH FACILITY MONTHLY REPORT

Section 1: Health Center Information

Health centre code	Health worker ID	Date report completed
[____|____]	[____|____]	[____|____]/[____|____]/[____|____]<br>day month year

START date of report	STOP date of report
[____|____]/[____|____]/[____|____]<br>day month year	[____|____]/[____|____]/[____|____]<br>day month year

Section 2: Clinical Report

ALL PATIENTS	
Total number of patients visiting the health center this month	[____|____|____]
Total number of patients who had fever	[____|____|____]
Total number of patients who had a rapid diagnostic test for malaria done	[____|____|____]
Total number of patients who had a positive rapid diagnostic test result	[____|____|____]
Total number of patients who had a negative rapid diagnostic test result	[____|____|____]
Total number of patients who were prescribed an ACT* to treat malaria	[____|____|____]
Total number of patients who were diagnosed with malaria	[____|____|____]
CHILDREN UNDER FIVE	
Total number of children under five years of age visiting the health center this month	[____|____|____]
Total number of children under five years of age who had fever	[____|____|____]
Total number of children under five who had a rapid diagnostic test for malaria done	[____|____|____]
Total number of children under five who had a positive rapid diagnostic test result	[____|____|____]
Total number of children under five who had a negative rapid diagnostic test result	[____|____|____]
Total number of children under five who were prescribed an ACT* to treat malaria	[____|____|____]
Total number of children under five who were diagnosed with malaria	[____|____|____]

* ACT = artemisinin-based combination therapy for malaria. Examples include Coartem, Lumartem, Duocotexcin

Section 3: Extra comments

Describe any situation that had an impact on patient care at your health center this month, such as drug stock-outs, staff shortages, or political instability.

The Republic of Uganda

USER'S MANUAL:

Use of rapid diagnostic tests (RDTs) for malaria in fever case management in Uganda

Malaria Control Programme
Ministry of Health
January 2009

TRAINING PROGRAMME

Use of RDTs in fever case management in Uganda: INTRODUCTION

Welcome to the training course on “Fever case management with RDTs.” RDT stands for Rapid Diagnostic Test for malaria. RDTs are diagnostic tools that can diagnose malaria using finger prick blood. They are very accurate. They are easy to use, and do not require electricity or special equipment. They are very useful even in areas where there is no laboratory or trained laboratory staff.

As a health worker, you likely see patients with fever every day. Fever is a common and important sign of illness. In Uganda and other parts of Africa, a patient with fever is often assumed to have malaria. This is considered good clinical practice where no diagnostic tests are available. However, now RDTs can provide accurate diagnosis.

Although malaria is very common in many parts of Uganda, we know that not all fevers are caused by malaria. Malaria is more common in some areas, and less common in others. Look at the map on page 2 of this Introduction. Let’s imagine a health centre in Apac district. Apac is an example of an area in Uganda where malaria is very common. If a health worker in Apac sees 50 patients with fever, 35 of those are likely to have malaria parasites in their blood. As another example, let’s imagine a health centre in Kabale district. Kabale is an example of an area in Uganda where malaria is not very common. If a health worker there sees 50 patients with fever, only 2 are likely to have malaria parasites. The patients with malaria should be treated promptly with antimalarial drugs. But those without malaria should be given the correct treatment for their illness.

It is important to give prompt antimalarial treatment to any patient who truly has malaria. However, if we give antimalarial drugs to patients who do not have malaria

parasites in their blood, we have failed to give them the correct treatment. If all patients with fever are given antimalarial drugs, it is clear that many will not receive the correct treatment for their illness. This practice also wastes medicine, puts patients at risk of side effects, and increases drug stock-outs and health care costs.

Therefore, we can provide better health care if we can confirm whether a patient truly has malaria, or another illness. Most Health Centres II and III in Uganda do not have laboratories or microscopes or trained laboratory staff to perform blood smears. However, RDTs do not require these things. RDTs are ideal for use in health centres where diagnostic tests were not available before.

This training course will teach you how to perform and read RDTs, and how to use them to identify which patients have malaria and which do not have malaria.

TRAINING SESSIONS:

This course is made up of 7 sessions. The information for each session is presented in a training guide, which we shall read together. Each session will be followed by time to practice the knowledge and skills gained. The sessions include:

1) How to evaluate patients with fever and select patients for RDT testing
2) Performing and reading an RDT
3) Management of a patient with fever and a positive RDT
4) Management of a patient with fever and a negative RDT
5) Recognition and referral of patients with severe illness
6) Patient education
7) RDT storage and monitoring

Let's get started!

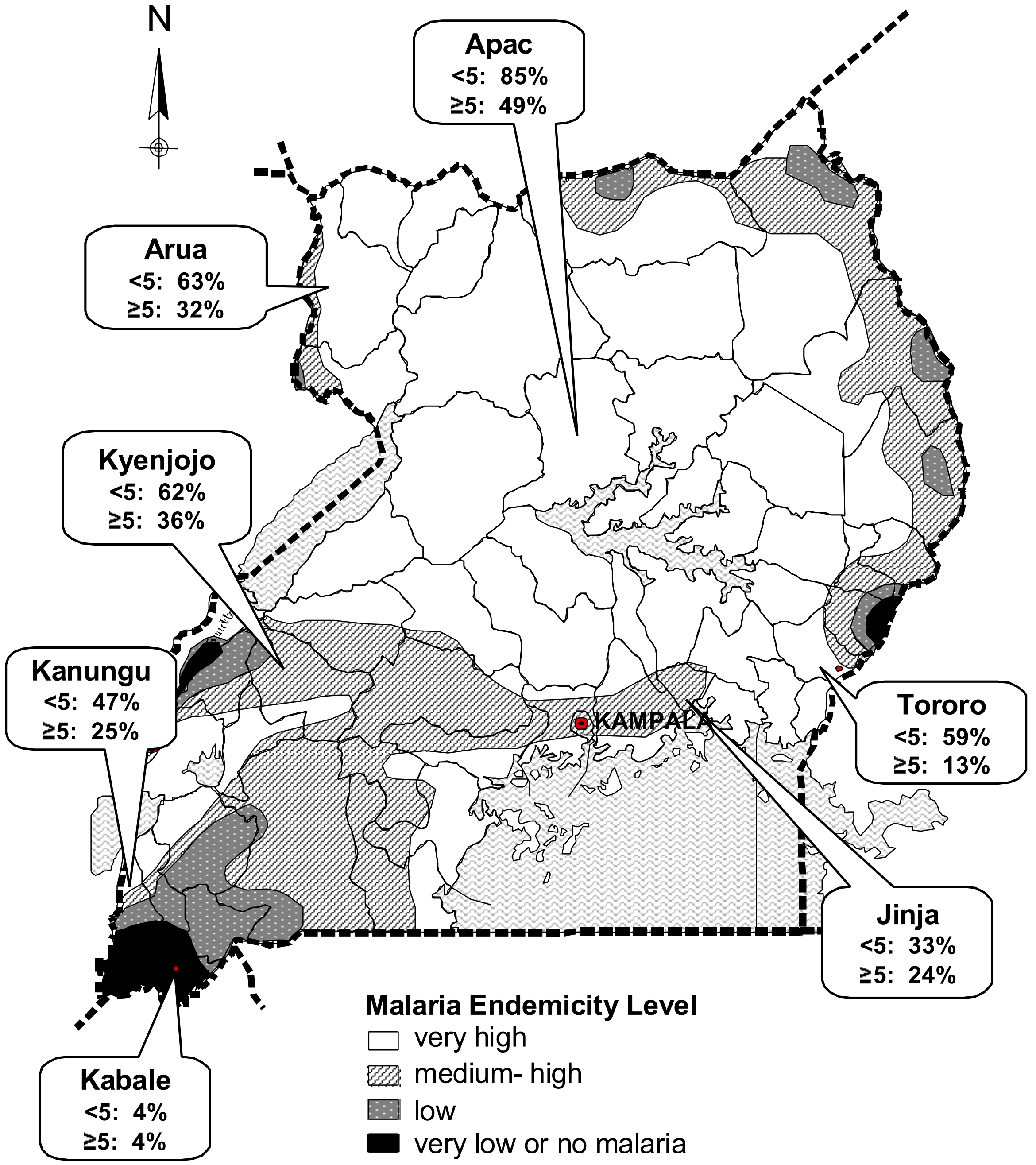

blood smear data from Uganda Malaria Surveillance Project,
Hopkins et al, *Journal of Infectious Diseases*, 2008

This map shows us some important facts about malaria Uganda:

1) **Not all fever is malaria.** Expert laboratory technicians read blood smears from patients with fever in each of these areas of Uganda. The number in each box shows the percentage of patients whose blood smear was positive. The results are grouped by patient age: <5 = children under 5 years old, and ≥5 = children 5 years and older and adults.

2) **Malaria risk is different in different parts of Uganda.** For example, in the mountain areas of Kabale, only 4% of patients with fever had malaria parasites. However, in Apac, 85% of young children with fever had malaria parasites. **According to this map, how common is malaria in your area?**

3) **Malaria risk is also different for older patients than for young children.** Older children and adults with fever are less likely to have malaria than young children.

Use of RDTs in fever case management in Uganda: PRE- and POST-TEST

Welcome to the pre-test for our training course in RDTs and fever case management. There are 16 questions in the pre-test. We will read each question together out loud. After we read the question, you should mark your answer on your own sheet. Don't worry if you don't know every answer. The reason for this test is not really to see how much you already know. The pre-test is designed to raise your interest in RDTs and fever cases. Each of these questions highlights an important learning point that we will cover during the course. By the end of the course, you will have learned everything you need to answer these questions correctly. On the last day of the course, we shall take this test again, and then discuss the answers as a group.

Instructions:

1. Read each question carefully along with the group.
2. Circle the letter in front of the answer you think is correct – for example **a)**

1. All cases of fever in Uganda are caused by malaria.

a) True

b) False

2. RDT stands for Rapid Diagnostic Test for malaria. Which of the following is **NOT** true about RDTs?

a) If performed correctly, they are very accurate in diagnosing which patients have malaria and which do not.

b) They can be performed in health centres that do not have power or laboratory equipment.

c) They can detect typhoid fever and pneumonia as well as malaria.

3. In order to perform an RDT, you will need to use finger prick blood. After you prick a patient's finger with a lancet, what should you do with the lancet?

a) Save it to wash and use again later.

b) Carefully put it immediately in the sharps container.

c) Put it on the table beside you, to use for the next patient.

4. We will pass an RDT around the room. Look carefully at it and read the result.

a) Positive
b) Negative
c) Invalid

5. Here is another RDT. Look carefully at it and read the result.

a) Positive
b) Negative
c) Invalid

6. RDTs should be stored in a cool, dry place.

a) True
b) False

7. You can use the same RDT for two different patients.

a) True
b) False

8. You should always check the expiry date on the package of an RDT before using it.

a) True
b) False

9. For which of the following patients should you perform an RDT for malaria?

a) A 4-year-old boy with fever and mild cough
b) An 8-month-old child with fever who refuses to breastfeed, is breathing rapidly and has very pale palms and sunken eyes
c) A 20-year-old woman who is pregnant and has fever
d) A 32-year-old man who is HIV-positive and has fever
e) A 45-year-old man with fever and joint paints
f) All of the above

10. A patient comes to your health centre with fever. She has taken chloroquine for two days but the fever has persisted. You perform an RDT and the result is positive. What should you do?

a) Tell the patient to complete the course of chloroquine.

b) Prescribe a full course of Coartem.

11. A patient comes to your health centre with fever. She took a complete, correct course of Coartem last week. She did not vomit any of the doses. You perform an RDT and the result is positive. What should you do?

a) Prescribe a full course of quinine.

b) Prescribe a second full course of Coartem.

c) Advise her to take Panadol and fluids only.

12. Which of the following is a symptom or sign of severe illness in a patient with fever?

a) Convulsions currently or within the past 2 days

b) Extreme weakness – patient is unable to sit or stand without support

c) Severe anaemia – patient's palms and conjunctivae are very pale

d) Unable to drink or breastfeed

e) All of the above

13. A 35-year-old man comes to your health centre. He tells you that he has fever and muscle aches. What should you do **FIRST**?

a) Prescribe Coartem immediately.

b) Prescribe chloroquine immediately – this regimen is best for adults.

c) Prescribe Panadol only and tell the patient to go home and rest.

d) Ask the patient how long he has had these symptoms, and ask if he has taken any drugs at home before coming to the health centre.

14. A mother brings her 8-year-old daughter to your health centre. Beginning yesterday, the girl has had fever and no appetite. Her mother has given Panadol and fluids, but the symptoms continue. There are no signs or symptoms of severe illness. On physical examination, you find no obvious cause of her fever. You perform an RDT, and the result is negative. Which of the following is **TRUE**?

a) This patient has a mild febrile illness, but the cause is not clear. The mother should continue to provide fluids and Panadol. You should advise her to bring the girl back to the health centre if the symptoms do not get better within 2 to 3 more days.

b) You should prescribe a course of chloroquine. Even though the RDT is negative, the girl may still have malaria, and chloroquine is appropriate for mild cases of malaria.

c) You should prescribe Coartem. Even though the RDT is negative, the girl may have malaria, and Coartem is the first-line regimen for malaria in Uganda.

15. A 10-year-old boy comes to your health centre with fever and joint pains. He looks weak, but is able to sit up easily by himself. After you take a history and do a physical examination, you perform an RDT. The RDT is positive, and you plan to treat for uncomplicated malaria. According to the Uganda Ministry of Health guidelines, which of the following is the **BEST** (first-line) treatment for this patient?

a) Chloroquine

b) Oral quinine

c) Injectable quinine

d) Coartem (artemether-lumefantrine)

e) Chloroquine + Fansidar (Homapak)

16. A mother brings her 1-year-old son to your health centre. She tells you he has not been feeding well. The child's body is hot to touch. He is weak and cannot sit up by himself, and he is breathing very rapidly. What should you do? Be sure to read all the statements below before answering.

a) Undress the child, and ask the mother to sponge him to help reduce the fever.

b) Immediately prepare and inject a dose of quinine AND antibiotic.

c) Perform an RDT and write the result on the referral note.

d) Write a referral note and ensure that the child is referred to a Health Centre IV or Hospital as soon as possible.

e) All of the above.

You have finished the pre-test – congratulations!

We hope these questions have raised your interest in RDTs and fever case management. Now, let's move ahead with the first session of the training course.

Session 1: HOW TO EVALUATE PATIENTS WITH FEVER AND SELECT PATIENTS FOR RDT TESTING

Content:

- ❑ **Introduction**
- ❑ **Learning objectives**
  1. **The importance of fever in selecting a patient for RDT testing**
  2. **Severe illness in a patient with fever**
  3. **Questions to ask when taking a history from a patient with fever**
  4. **Physical examination of a patient with fever**
  5. **Selecting a patient for RDT testing based on history and physical examination**
- ❑ **Summary**
- ❑ **References**

INTRODUCTION:

Welcome to Session 1 on "How to evaluate patients with fever and select patients for RDT testing." An RDT can tell you whether or not a patient has malaria. Therefore, when you are considering whether to test a patient with an RDT, the main question in your mind should be:

Based on the patient's symptoms, do I believe this patient may have malaria?

If the answer to the question is yes, then you can use an RDT to help determine whether or not the patient the patient actually has malaria.

Any time you are deciding whether a patient has malaria or another cause of fever, you can perform an RDT. The RDT result, together with the information you get from talking with the patient and examining him or her, will help you to make the correct diagnosis and provide the best treatment.

In this session, we will talk more about the symptoms of malaria, and the symptoms of other illnesses that may look like malaria. One of the most common symptoms that patients report is fever. Fever is an important signal that there is something wrong in the body. Fever does not always require treatment, but a health worker should try to understand the cause and to provide treatment if appropriate.

Frequently in Uganda, a patient with fever is assumed to have malaria. Although malaria is very common in many parts of Uganda, we know that not all fevers are caused by malaria. Many other diseases also cause fever. Therefore, it is important for you to be able to confirm whether a patient truly has malaria, or another illness. In this session, we will discuss the key points in taking a history and examining a patient with fever.

LEARNING OBJECTIVES:

By the end of this session, you should be able to:

1) Describe fever and explain how this symptom is important in selecting a patient for RDT testing
2) List signs of severe illness in a patient with fever
3) Outline important questions to ask when taking a history from a patient with fever
4) Describe how to carry out a physical examination of a patient with fever
5) Describe how to select a patient for RDT testing based on the history and physical examination

1.1 THE IMPORTANCE OF FEVER IN SELECTING A PATIENT FOR RDT TESTING

The main symptom that indicates that a patient may have malaria is **fever**. If a patient has fever, you should consider testing him or her with an RDT.

In our local languages, patients and their caregivers describe fever as a feeling that something is wrong in the body. Some of the local terms are *omusujja, kwaman lieth, omushwija* and *omutsusa*. These terms may describe body hotness, general body pain, or just feeling unwell. It is the responsibility of the health worker to determine whether the patient actually has fever by carefully asking questions of the patient or caregiver.

If you have a thermometer, you can measure the patient's temperature. Normal body temperature measured under the arm (axillary) is considered to be between 35.6°C to 37.0°C. Normal body temperature can vary over the course of the day in the same person, and can also vary from one person to the next.

Fever is present if:

- Axillary temperature is above 37°C
- The patient or caregiver describes body hotness

1.2 SEVERE ILLNESS IN A PATIENT WITH FEVER:

Most patients come to the health centre with illness that is not severe. However, some patients come with severe illness. When you assess a patient, you should first look for signs of severe illness.

If a patient has any of these symptoms or signs of severe illness, you should act quickly to **give pre-referral treatment** and **refer the patient to a Health Centre IV or Hospital**. We shall discuss severe illness in more detail in **Session 5**.

For now, let's review the symptoms and signs of severe illness in the following box. These signs are listed in the Uganda Ministry of Health's guidelines on "Management of Uncomplicated Malaria: A Practical Guide for Health Workers," 3rd edition, printed in 2005:

Danger signs of severe illness:

- ***Convulsions or fits – now, or within the past 2 days***
- ***Not able to drink or breast feed***
- ***Vomiting everything – not able to keep down food, fluid, or drugs***
- ***Changes in mental state – patient is confused, very sleepy = lethargic or drowsy, or in a coma = unconscious***
- ***Extreme weakness (prostration) – patient is unable to sit or stand without support***
- ***Severe difficulty breathing = respiratory distress***
- ***Severe anaemia – pale palms, fingernails, eyelids***
- ***Severe dehydration – coated tongue, sunken eyes, skin pinch***

If the patient shows any of the danger signs listed in the box, follow **the instructions in Session 5**. If the patient does not show danger signs, move on to take a history and perform a physical exam as below.

1.3 QUESTIONS TO ASK WHEN TAKING A HISTORY FROM A PATIENT WITH FEVER:

Your goal is to find clues in the patient's history that suggest a specific diagnosis. You should ask the following questions:

❑ **Characteristics of the fever**

- When did the fever start?
- How long has it lasted?

A fever that has persisted for more than 7 days may be malaria, but in this case you should also consider another illness such as typhoid fever, tuberculosis and other infectious diseases.

❑ **Past medical history**

- Does the patient have any chronic diseases?
- If the patient is a child, has he or she received the recommended schedule of immunizations?

For example, patients with a chronic disease such as HIV are more likely to suffer from malaria, as well as other infections like pneumonia and TB. Patients with sickle cell disease are also more in danger from malaria and other infections. As another example, a child who has not received immunizations is more likely to get measles and certain respiratory infections.

❑ Prior treatment

- What has been done to treat this illness before coming to your health centre today?
- What other medications have been taken?
- If medications were taken, was the dose complete, or partial?

This information will help to guide your treatment decisions. For example, if a patient has taken a full course of Septrin but has not improved, you should not prescribe Septrin again.

As another example, a patient may come to the health centre after swallowing only part of a dose of Coartem. (Note a complete course of Coartem requires 6 doses over 3 days.)

❑ Other symptoms

Many illnesses can cause fever. Only malaria can be treated with antimalarials. These other illnesses require other treatments. Once you know that your patient has fever, you should also ask whether he or she has any of the following symptoms. As we will see in Table 1 at the end of this session, these symptoms can be clues to help you diagnose the patient correctly.

- **Chills and rigors**
- **Headache**
- **General body weakness**
- **General body aches and joint pains**
- **Cough or flu**
- **Sore throat and painful swallowing**
- **Ear pain**
- **Loss of appetite**
- **Nausea**, **vomiting**, **abdominal pain**, and **diarrhoea**
- **Pain when passing urine (dysuria)**
- **Low abdominal pain**
- **Bone pain in a specific area**
- **Joint pain and/or swelling**
- **Painful swellings in the skin**
- **Skin rash**

Next we will discuss how to examine a patient with fever.

1.4 PHYSICAL EXAMINATION OF A PATIENT WITH FEVER:

In the physical examination of a patient with fever look for the following:

- **Measure the temperature**

- **Assess for danger signs**
  - As we discussed above in section 1.2, and as we shall discuss in more detail in Session 5.

- **Measure the weight (if you have a scales)**
  - You will need the body weight to give the correct dose of medication, especially in children.

- **Carefully examine the following systems**
  - **General**
    - Look for **pallor** = paleness of the palms, soles, fingernails and conjunctiva. Pallor indicates anaemia, which may be caused by malaria or other parasites.
    - Look for **jaundice** = abnormal yellow colour of the eyes. Jaundice may indicate either liver disease such as hepatitis, or very severe anaemia from malaria.
    - With your fingers, feel for **swollen or tender lymph nodes** at the neck, armpits, and groin area. Lymph nodes may be painful and swollen due to local bacterial infection or local TB infection.

  - **Central Nervous System**
    - Check for **neck stiffness**. Meningitis often causes painful neck stiffness.
    - Check for a **bulging fontanel** in very young children. This may also be caused by meningitis.

  - **Ears / Nose / Throat (ENT)**
    - Look for **redness in the throat or tonsils**. This indicates throat infection = pharyngitis, or tonsil infection = tonsillitis.
    - Look for **oral thrush** = white coating on the tongue and inner cheeks. Oral thrush may indicate immune suppression, such as HIV infection. If a patient has HIV, he or she is more likely to suffer from other infections such as pneumonia or malaria.
    - Look for **dryness of the tongue and mouth**. This may be a sign of dehydration.
    - Check **ears for redness and discharge**. These may be signs of ear infection = otitis media.

- **Respiratory**
  - Count the **number of breaths per minute**. Fast breathing in a patient with fever may be caused by severe pneumonia, severe malaria, or another serious infection. Fast breathing depends on the patient's age:

    - Younger than 2 months: 60 breaths per minute or more is fast breathing
    - 2 months to 12 months: 50 breaths per minute or more is fast breathing
    - 12 months to 5 years: 40 breaths per minute or more is fast breathing
    - 5 years or older: 20 or more breaths per minute is fast breathing

  - Look for signs of **difficulty breathing**: flaring of the nostrils, chest in-drawing. This may be caused by severe pneumonia or severe malaria.
  - If you know how to use a stethoscope, listen for any **abnormal chest sounds** such as rhonchi, wheezes, or crackling sounds = crepitations. Abnormal chest sounds in a patient with fever may be caused by pneumonia or bronchitis, or by severe malaria.

- **Abdomen**
  - Check for **pain on pressure**. Pain over the liver may indicate hepatitis. Lower abdominal pain may indicate urinary tract infection (UTI), or pelvic inflammatory disease (PID) in women.

- **Skin**
  - Look for **skin rashes**. Skin rash is not a sign of malaria. Skin rash may occur in measles, HIV infection, and other infections.
  - Look for any **painful swellings or abscesses**. These may indicate bacterial infection.
  - Examine any **wounds**: Is there redness or pain to pressure around the wound? Is there pus or discharge from the wound? If so, the wound is likely infected with bacteria.

Now we will look at Table 1 to see how the information from the history and physical examination can help us diagnose a patient with fever.

Table 1. Clinical signs associated with common causes of fever

Clinical sign	Common diagnosis
**Chills and rigors**	Malaria<br>Urinary tract infection (UTI)<br>Bacterial infection, for example sepsis
**Headache**	Malaria<br>Meningitis<br>Sinus infection (sinusitis)<br>Ear infection (otitis media)<br>Dental problems
**General body weakness**	Malaria<br>Other types of infection
**General body aches and joint pains**	Malaria<br>Viral infections
**Cough, difficult breathing, rapid breathing**	Pneumonia<br>Malaria<br>Tuberculosis<br>Measles
**Sore throat, painful swallowing**	Pharyngitis or tonsillitis<br>Thrush (candidiasis)
**Ear pain**	Ear infection (otitis media)
**Loss of appetite, nausea, vomiting, abdominal pain, diarrhoea**	Malaria<br>Gastro-enteritis<br>Hepatitis
**Bloody diarrhoea**	Dysentery – bacterial or amoebic
**Painful urination, frequent urination, flank pain**	Urinary tract infection (UTI)<br>Kidney infection (pyelonephritis or severe UTI)
**Low abdominal pain**	Urinary tract infection (UTI)<br>Pelvic inflammatory disease (PID)

(Table continues on next page…)

Clinical sign	Common diagnosis
**Bone pain, with increased pain if pressure is applied**	Osteomyelitis (bacterial bone infection)
**Joint pain and/or swelling, with pain on movement**	Bacterial infection of the joint<br>Rheumatic fever
**Painful and swollen lymph nodes**	Bacterial abscess<br>Tuberculosis (TB)
**Pain, swelling, hotness, and/or redness of the skin**	Bacterial skin infections (cellulitis)<br>Abscess
**Skin rash**	Measles<br>Chicken pox<br>HIV sero-conversion<br>Drug side effect or allergic reaction

1.5 SELECTING A PATIENT FOR RDT TESTING BASED ON HISTORY AND PHYSICAL EXAMINATION

As we have discussed, fever is an important symptom of malaria, but it is also a symptom of other illnesses. There are many reasons why a patient may present with fever. Therefore, it is important to carefully ask questions and examine any patient who presents with a fever.

After you have taken a history and performed a physical examination, you should ask yourself this question:

Based on the patient's symptoms, do I believe this patient may have malaria?

If the answer to the question is yes, then you can use an RDT to help determine whether or not the patient actually has malaria.

****Any time you are deciding whether a patient has malaria or another cause of fever, you can perform an RDT. The RDT result, together with the information you get from talking with the patient and examining him or her, will help you to make the correct diagnosis and provide the best treatment.****

You can use Table 1, along with IMCI and other guidelines you may have, to consider non-malaria diagnoses in patients with a negative RDT. Other diseases that are not listed in the table may also cause fever – but the diseases listed here are common and you should keep them in mind. If you see a patient with a condition you do not recognize, you should consider **REFERRING** the patient to a Health Centre IV or Hospital.

SUMMARY

We have come to the end of our session on "How to evaluate patients with fever and select patients for RDT testing." In this session we discussed fever and the usefulness of this symptom in determining whether to test a patient with an RDT. We discussed important questions and physical examination findings in patients with fever. We reviewed several common causes of fever.

Any time you are deciding whether a patient's fever is caused by malaria, or by another illness, you can perform an RDT. Together with the information you get from the history and physical examination, the RDT result will help you to make the correct diagnosis and provide the best treatment for the patient.

Keep the learning points from Session 1 in mind as we move on in this training course – we shall look back on this session when we discuss how to manage a patient with fever but a negative malaria RDT, in Session 4.

REFERENCES

1. Uganda Ministry of Health, "Management of uncomplicated malaria: A practical guide for health workers," 3rd edition, Dec 2005.
2. (1995). Lecture Notes on Tropical Medicine. Oxford, Blackwell Science.
3. Chandramohan, D., I. Carneiro, et al. (2001). "A clinical algorithm for the diagnosis of malaria: results of an evaluation in an area of low endemicity." Trop Med Int Health **6**(7): 505-10.
4. Chandramohan, D., S. Jaffar, et al. (2002). "Use of clinical algorithms for diagnosing malaria." Trop Med Int Health **7**(1): 45-52.
5. Kallander, K., J. Nsungwa-Sabiiti, et al. (2004). "Symptom overlap for malaria and pneumonia--policy implications for home management strategies." Acta Trop **90**(2): 211-4.
6. Marsh, K., D. Forster, et al. (1995). "Indicators of life-threatening malaria in African children." N Engl J Med **332**(21): 1399-404.
7. Nsungwa-Sabiiti, J., K. Kallander, et al. (2004). "Local fever illness classifications: implications for home management of malaria strategies." Trop Med Int Health **9**(11): 1191-9.
8. Warrell, D. A. and H. M. Gilles, Eds. (2002). Essential Malariology. London, Arnold.
9. World Health Organization (2000). "Severe falciparum malaria." Trans R Soc Trop Med Hyg **94**(Suppl 1): S1-60.
10. World Health Organization (2006). "Guidelines for the treatment of malaria." WHO/HTM/MAL/2006.1108.

Session 2: PERFORMING AND READING AN RDT

Content:

- ❑ **Introduction**
- ❑ **Learning objectives**
  1. **Description of RDTs – How do they work?**
  2. **Performing an RDT (WHO picture guide)**
  3. **Reading an RDT (WHO picture guide)**
  4. **Important tips for using RDTs**
  5. **Safe handling of blood and sharps**
- ❑ **Hands-on practice session**
- ❑ **Summary**
- ❑ **References**

INTRODUCTION:

Welcome to Session 2 on "Preparing and reading an RDT." RDT stands for *Rapid Diagnostic Test* for malaria. RDTs are useful to test patients for malaria, especially in areas where blood smears and microscopes are not available. RDTs detect the presence of malaria parasites in the blood. RDTs are called "rapid" because they give results within 15 minutes. RDTs have been studied and shown to be very accurate in areas around Uganda.

This session will provide you with the knowledge and skills to correctly and safely use RDTs to test patients for malaria.

LEARNING OBJECTIVES:

By the end of this session, you should be able to:

1. Describe an RDT and how it works
2. Perform an RDT correctly and safely
3. Read an RDT accurately and record the result as positive or negative
4. List some important tips for using RDTs
5. Describe safe handling of blood and sharps

2.1 DESCRIPTION OF RDTs – HOW DO THEY WORK?

RDTs are diagnostic tests that detect malaria in blood. The presence of malaria parasites in a person's blood is the cause of the fever and other symptoms we associate with malaria illness.

What do RDTs detect?

In the blood, malaria parasites make molecules called **antigens**. It is these antigens that RDTs detect. If a person is infected with malaria parasites, the parasites produce antigen in his or her blood, and the RDT result will be positive. If there are no parasites in the blood, there is no antigen, and the RDT result will be negative.

How do RDTS work?

RDTs contain molecules called **antibodies**. Antibodies fit together, or bind, with antigens. If the malaria antigen is present in the blood, the antibodies in the RDT can bind to it.

After we apply a drop of blood to the RDT, we add a special liquid called **buffer**. Buffer carries the blood, along with any antigen, and the antibodies, along the length of the RDT.

If antigen is present in the blood, and the antibodies in the RDT bind to it, the combination of antigen + antibody is trapped at the **test line (position T)** and forms a **red or purple line**. This gives a **positive RDT result**.

If there is no parasite antigen the antibodies have nothing to bind to, and they do not form a test line. This gives a **negative RDT result**.

The RDT also contains special molecules that are trapped at the **control line (position C)** if the buffer and blood have reached the end of the test strip. The control line tells us whether the RDT has worked correctly. All completed RDTs should show a red or purple control line. If we do not see a control line, the RDT result is **invalid**. In this case, we must repeat the patient's test with a new RDT.

Different types of RDTs are available.

You may see RDTs detecting different antigens, and made by different companies. Each brand or type of RDT has slightly different instructions for how to perform and read the tests. For now, let us focus on the RDT that will be used in this training.

This RDT has been studied carefully in Uganda and shown to be very accurate in diagnosing malaria. At this time it is the choice of the Malaria Control Program of the Uganda Ministry of Health.

2.2 PERFORMING AN RDT

For this part of the session, we will use the WHO picture guide. The picture guide has step-by-step instructions showing how to perform an RDT. The steps are listed here, along with some additional tips for some of the steps:

Before you begin, collect:

a. NEW unopened test packet
b. NEW unopened spirit swab (alcohol swab)
c. NEW unopened lancet
d. NEW pair of disposable gloves
e. Buffer
f. Timer or clock
g. Sharps container

Step 1: Check the expiry date on the test packet.

Step 2: Put on the gloves. Use new gloves for each patient.

Step 3: Open the packet and remove: test, loop, and desiccant sachet.

Extra tip for step 3: Each RDT packet contains a "desiccant sachet," which keeps the RDT dry until the packet is opened. The desiccant in our RDT packets should be **blue**. If the desiccant is purple, pink, or white, it means the test packet has been damaged. If the desiccant is not blue, throw away the test and open a new RDT packet.

Step 4: Write the patient's name on the cassette.

Extra tip for step 4: We will write the patient's **name and OPD number** on the cassette. Pencil works best for writing on the RDTs.

Step 5: Open the spirit swab (alcohol swab). Grasp the patient's ring finger. Clean the finger with the spirit swab. Allow the finger to dry before pricking.

Extra tip for step 5: After cleaning the patient's finger, allow it to air dry. Do <u>not</u> blow on the finger or wipe it – these actions make the finger dirty again.

Step 6: Open the lancet. Prick patient's finger to get a drop of blood.

Extra tip for step 6: When pricking the patient's finger, squeeze the tip of the finger with your own fingers and prick the **side** of the fleshy part. This is less painful than pricking in the middle or at the tip. Prick hard enough so that a drop of blood quickly appears on the skin.

Step 7: Discard the lancet in the sharps box immediately after pricking finger. Do **not** set down the lancet before discarding it.

Step 8: Use the loop to collect the drop of blood.

Extra tip for step 8: Touch the loop gently to the blood drop on the patient's finger. The loop will fill with the correct amount of blood.

Step 9: Use the loop to put the drop of blood into the square hole at **position A**.

Extra tip for step 9: Hold the RDT flat on the table top with one hand. With your other hand, carefully place the blood drop on the pad at position A. It is important to work quickly enough that the blood does not clot, but carefully so that all of the blood is absorbed into the pad. If most of the blood is accidentally wiped on the plastic edges of the well, the test will not work correctly.

Step 10: Immediately discard the loop in the sharps box.

Step 11: Put six (6) drops of buffer into the round hole at **position B.**

Extra tip for step 11: Check the time just after you add buffer to an RDT, and write the time on the RDT.

Step 12: Wait **15 minutes** after adding buffer.

Step 13: Read test results. Note: Do **not** read the test sooner than **15 minutes** after adding the buffer. You may get false results.

Extra tip for step 13: Before you read the RDT, check the time again to be sure that at least 15 minutes have passed.

2.3 READING AN RDT

For this part of the session, we will again use the WHO picture guide. We shall also use example results in the WHO training photographs and quiz, and actual RDTs.

See **point 14** on the WHO picture guide:

The RDT is **positive** if there is one red/purple line at position C and one red/purple line at position T. This means the patient **does** have malaria. The test is **positive** even if the red/purple line at position T is faint.

The RDT is **negative** if there is one red/purple line at position C and **NO** red/purple line at position T. This means the patient does **not** have malaria.

The RDT is **invalid** if there is **no** line at position C. This means the test is damaged. Even if there is a line at position T, if there is **no** line at position C it means the test is damaged. The results are invalid (false).

Immediately after you read the RDT, record the result on the outpatient form. Use the following symbols, and write clearly:

If the RDT is **positive**, write: **RDT pos**

If the RDT is **negative**, write: **RDT neg**

If the first test result is invalid, you should repeat with a new RDT. Then record the new test result in the patient's record. We shall discuss the importance of each test result in more detail in Sessions 3 and 4.

2.4 IMPORTANT TIPS FOR USING RDTs

With attention and practice, you will soon be very skilled at preparing and reading RDTs. Here are some important points to keep in mind:

- Always **check the expiry date**. An expired RDT may give a false result.
- Do **not** open an RDT packet until you are ready to use it for a patient. If a packet has been open for some time before the RDT is used, the RDT may give a false result.
- Do **not** put down the lancet or loop on the table after use. **Put the lancet and loop immediately into the sharps container.** If you put them on the table, you or someone else may accidentally be pricked. Accidental pricks can spread HIV, hepatitis viruses, and other diseases.
- Carefully collect the **correct amount of blood** and **place it neatly on the pad at position A**. The RDT may not work properly if you use too little or too much blood, or if the blood is not absorbed into the pad.
- Hold the bottle of buffer vertically over position B and add **exactly 6 drops of buffer**. The RDT may not work properly if you use too little or too much buffer.
- Be sure to wait **15 minutes** after adding buffer, before you read the RDT. Reading the RDT too soon can give a false result.
- Remember to **check for the control line**. If there is no control line, the RDT has not worked properly and the test result is invalid (false).

2.5 SAFE HANDLING OF BLOOD AND SHARPS

Correct handling of blood and sharps is very important for your safety and for the safety of your co-workers and patients. Safe handling involves protecting yourself and others from exposure to diseases that may be carried and transmitted by blood. Remember:

- **Always wear gloves** when working with blood, or with items that have touched blood. (This includes used alcohol swabs and cotton swabs.)
- **Put the lancet into the sharps box immediately after using it.** If you put down the lancet after using it, you or someone else may accidentally be pricked with it. Dirty lancets can spread HIV, hepatitis viruses, and other diseases.
- **Never use a lancet on more than one person.** Used lancets can spread HIV, hepatitis viruses, and other diseases. Even if you wash or clean the lancet, it still may carry disease.
- **Never put the lancet into the regular waste container – only use the sharps box.** If you put a used lancet with normal waste, anyone who handles the waste may be pricked and exposed to disease.
- **Put the blood loop into the sharps box immediately after the transfer of the blood to the test cassette**. The loop may also carry blood-borne diseases.
- During this study, we will save all RDTs after use, for research purposes. However, after the study has ended, you may discard used RDTs. After they study has ended, after you have read an RDT and recorded the result in the patient's record, **put the RDT into the waste container**. The RDT should be disposed of with the rest of the medical waste from the health centre, including used gloves, used spirit swabs (alcohol swabs) and other items. For example, many health centres discard waste in a garbage pit. The pit should be in a place where children and animals cannot easily reach it.

☺ **SUMMARY:**

We have come to the end of session on "Performing and reading an RDT." We reviewed how RDTs detect malaria parasite antigen in the blood of infected patients.

We learned and practiced the steps to perform and read RDTs correctly and safely.

Remember: Safe handling of blood and sharps is a very important part of the correct use of RDTs. Always wear gloves when working with blood – and always put lancets and blood loops into the sharps box immediately after use.

REFERENCES:

1. WHO website "Malaria Rapid Diagnostic Tests," www.wpro.who.int/sites/rdt

Session 3: MANAGEMENT OF A PATIENT WITH FEVER AND A POSITIVE RDT

Content:

- ❑ **Introduction**
- ❑ **Learning objectives**
  - **1 Meaning of a positive RDT in a patient with fever**
  - **2 How to treat a patient with fever and a positive RDT**
  - **3 Supportive treatment**
- ❑ **Summary**
- ❑ **References**

INTRODUCTION:

Welcome to Session 3 on "Management of a patient with fever and a positive RDT." As we will discuss below in more detail, a patient with fever, a positive RDT, and no signs of severe illness should be treated for uncomplicated malaria. In this session we will review Uganda Ministry of Health guidelines on treatment of patients with uncomplicated malaria. These guidelines are a review from the Ministry of Health's booklet "Management of Uncomplicated Malaria: A Practical Guide for Health Workers," 3rd edition, printed in 2005. The goals of treating a patient with a positive RDT are to:

- Cure the infection.
- Prevent progression to severe disease.

Correct treatment of patients with fever and a positive RDT also helps to reduce transmission of malaria infection to other people, and to prevent the spread of resistant malaria strains.

LEARNING OBJECTIVES:

By the end of this session, you should be able to:

1) Explain the meaning of a positive RDT result in a patient with fever
2) Describe how to treat a patient with fever and a positive RDT
3) Outline supportive treatments for a patient with fever and positive RDT

3.1 MEANING OF A POSITIVE RDT IN A PATIENT WITH FEVER

A patient with fever and a positive RDT is considered to have malaria. The malaria may be uncomplicated or severe.

Uncomplicated malaria is diagnosed when a patient has **all of the following**:

1) Symptoms of malaria (see box below).
2) Evidence of parasites in the blood – with a positive RDT (or blood smear).
3) No signs of severe illness. (For more details on severe illness, see Sessions 1 and 5.)

Review from the Ministry of Health's booklet "Management of Uncomplicated Malaria: A Practical Guide for Health Workers," 3rd edition, printed in 2005:

Common symptoms and signs of malaria include:

- *Fever*
- *Loss of appetite*
- *Weakness*
- *Nausea or vomiting*
- *Headache*
- *Joint pains*
- *Muscle aches*
- *Lethargy (tiredness*

***Remember: To correctly diagnose a patient with uncomplicated malaria, you should see both symptoms AND a positive RDT.**

If a patient has symptoms as in the box above, NO signs of severe illness, AND a positive RDT, you should prescribe antimalarial treatment.*

Now we shall review the recommended treatment for patients with uncomplicated malaria in Uganda.

3.2 HOW TO TREAT A PATIENT WITH FEVER AND A POSITIVE RDT

The Uganda national malaria treatment policy includes combination therapy. **A patient with fever and a positive RDT (and with no signs of severe illness) should be treated for uncomplicated malaria with combination therapy.**

Information on malaria and its treatment is presented in more detail in the Ministry of Health's booklet ""Management of Uncomplicated Malaria: A Practical Guide for Health Workers," 3rd edition, printed in 2005. Below, we will review some of the key points found in that booklet.

Combination therapy is the combination of two or more drugs given together to treat malaria. The main reason to give combination therapy to treat malaria is that it is more effective than using just one antimalarial drug. Think of an army is fighting an enemy – two soldiers are more effective than one.

Over the past several years, the malaria parasite has developed resistance to the older commonly used drugs (for example, chloroquine). Another important benefit of combination therapy is that it helps to prevent or delay the malaria parasite's development of resistance to the new drugs. For these reasons, the Uganda Ministry of Health now recommends combination therapy.

3.2.1 Types of antimalarial combination therapy

Currently, there are two types of antimalarial combination therapy:

- ❑ **Artemisinin-based combination therapy (ACT)**

  The ACT regimens are based on drugs called **artes**unate, **artem**ether, or dihydro**artem**isinin. ACTs recommended by the Uganda MoH include:

  1) **Artemether + lumefantrine = Coartem**
  2) **Amodiaquine + artesunate (AQ+AS)**

There are other ACTs available in Uganda. The ACTs listed here are those recommended by the MoH.

- ❑ **Other antimalarial therapy**

Sometimes your health centre may not have ACTs in stock. In this case, you may be forced to recommend an older antimalarial therapy. However, when available, ACT regimens are more effective, and are preferred.

3.2.2 Specific treatment

To treat a patient with uncomplicated malaria, you need to give specific treatment with effective antimalarials, which we shall discuss in this section. You should also give supportive treatment, which we shall discuss in the following section (3.4).

All of the information presented here is a review from the Ministry of Health's booklet "Management of Uncomplicated Malaria: A Practical Guide for Health Workers."

The recommended first-line regimen for Uganda (and many other African countries) is artemether-lumefantrine = Coartem.

❑ **Artemether-lumefantrine (Coartem)**

Coartem is a co-formulated drug. This means that two drugs are combined in each tablet. A complete treatment with Coartem requires 6 doses: one dose, given twice a day, over a period of 3 days.

Table 1. Treatment schedule for artemether-lumefantrine (Coartem)

Weight* in kg	Age*	Day 1	Day 2	Day 3	Colour code
**5-14**	4 months to 3 years	1 tablet x 2 12 hourly	1 tablet x 2 12 hourly	1 tablet x 2 12 hourly	**Yellow**
**15-24**	3 years to 7 years	2 tablets x 2 12 hourly	2 tablets x 2 12 hourly	2 tablets x 2 12 hourly	**Blue**
**25-34**	7 years to 12 years	3 tablets x 2 12 hourly	3 tablets x 2 12 hourly	3 tablets x 2 12 hourly	**Brown**
**>35**	12 years and older	4 tablets x 2 12 hourly	4 tablets x 2 12 hourly	4 tablets x 2 12 hourly	**Green**

*If you have a scales, it is best to use the patient's weight to determine the dose. If you don't have a scales, use the patient's age.

- ❑ **Artesunate + amodiaquine**

The alternative first-line treatment for uncomplicated malaria in Uganda is artesunate (AS) + amodiaquine (AQ). This combination can be used if Coartem is not available. AS + AQ must be given as separate tablets, as in Table 2. A complete treatment requires 3 doses of each drug: one dose of each, given once a day, over a period of 3 days.

Table 2. Treatment schedule for Artesunate + Amodiaquine

	**Dose in mg (Number of tablets)**					
	**Artesunate = 50 mg tabs**			**Amodiaquine = 153 mg tabs**		
**Age**	**Day 1**	**Day 2**	**Day 3**	**Day 1**	**Day 2**	**Day 3**
5 months to 11 months	25 mg (1/2 tab)	same as Day 1	same as Day 1	76 mg (1/2 tab)	same as Day 1	same as Day 1
1 year to 6 years	50 mg (1 tab)	same as Day 1	same as Day 1	153 mg (1 tab)	same as Day 1	same as Day 1
7 years to 13 years	100 mg (2 tabs)	same as Day 1	same as Day 1	306 mg (2 tabs)	same as Day 1	same as Day 1
13 years and older	200 mg (4 tabs)	same as Day 1	same as Day 1	612 mg (4 tabs)	same as Day 1	same as Day 1

3.2.3 Contra-indications of ACTs

Do not give an ACT medication to a patient who has had a bad reaction to the drug previously.

Do not give ACT at the same time with quinine or with other antimalarial drugs.

In addition, do not give ACT to:

1. Women in the first 3 months of pregnancy
2. Babies less than 4 months old
3. Children who weigh less than 5 kg

For patients in these 3 groups, you should give quinine.

Coartem is safe and effective for pregnant women after the 3rd month, for children older than 4 months, and for children weighing more than 5 kg.

3.2.4 Treatment administration

Treatment for uncomplicated malaria is taken orally (by mouth). Remember that Coartem should be taken with food or fluids. If possible the patient should take each dose of Coartem with milk or breast milk, or fatty or oily food (for example, meat or bean sauce made with cooking fat or oil, groundnut sauce, or odi). This improves absorption of the drug from the gut.

The first dose of any antimalarial treatment should be directly observed at the health centre. If the patient vomits in less than 30 minutes, wait 10 minutes and then give a second dose. If the second dose is vomited, change to injectable quinine or artemether.

3.3 SUPPORTIVE TREATMENT

Supportive treatment helps to relieve symptoms and improve recovery. Supportive treatment includes **relief of fever**, **relief of pain** (headache, body and joint pains), and attention to the patient's **fluid and nutritional intake**.

- **Use of antipyretics (drugs to decrease fever and pain)**

The following antipyretics are commonly used for patients with fever or malaria:

- paracetamol (Panadol) 10 mg/kg every 6 hours
- ibuprofen (Brufen) 5 mg/kg every 8 hours
- aspirin (*Note: do not give aspirin to patients younger than 8 years, as it can cause a serious side effect called Reye's syndrome)

- **Other measures:**

Other measures to relieve fever include **tepid sponging**, **fanning** and encouraging **fluid intake**.

REVIEW: Correct practices in treatment of uncomplicated malaria

- Start antimalarial therapy as **soon** as possible after diagnosing the patient.
- Give **antimalarial combination therapy** according to current guidelines.
- Ensure the patient has the **correct antimalarial dose** for his or her weight or age.
- Ensure the patient has a **complete antimalarial dose** and understands the importance of completing the full treatment.
- Give advice on **supportive treatment** to relieve symptoms and speed recovery.
- Watch for signs of **severe malaria**, **give pre-referral treatment** and **REFER** any patient with severe disease to a higher level facility immediately.

You can use the flow chart on the following page to manage a patient with a positive RDT result:

FLOW CHART: Management of patient with symptoms of uncomplicated malaria, no signs of severe illness, and a positive RDT for malaria

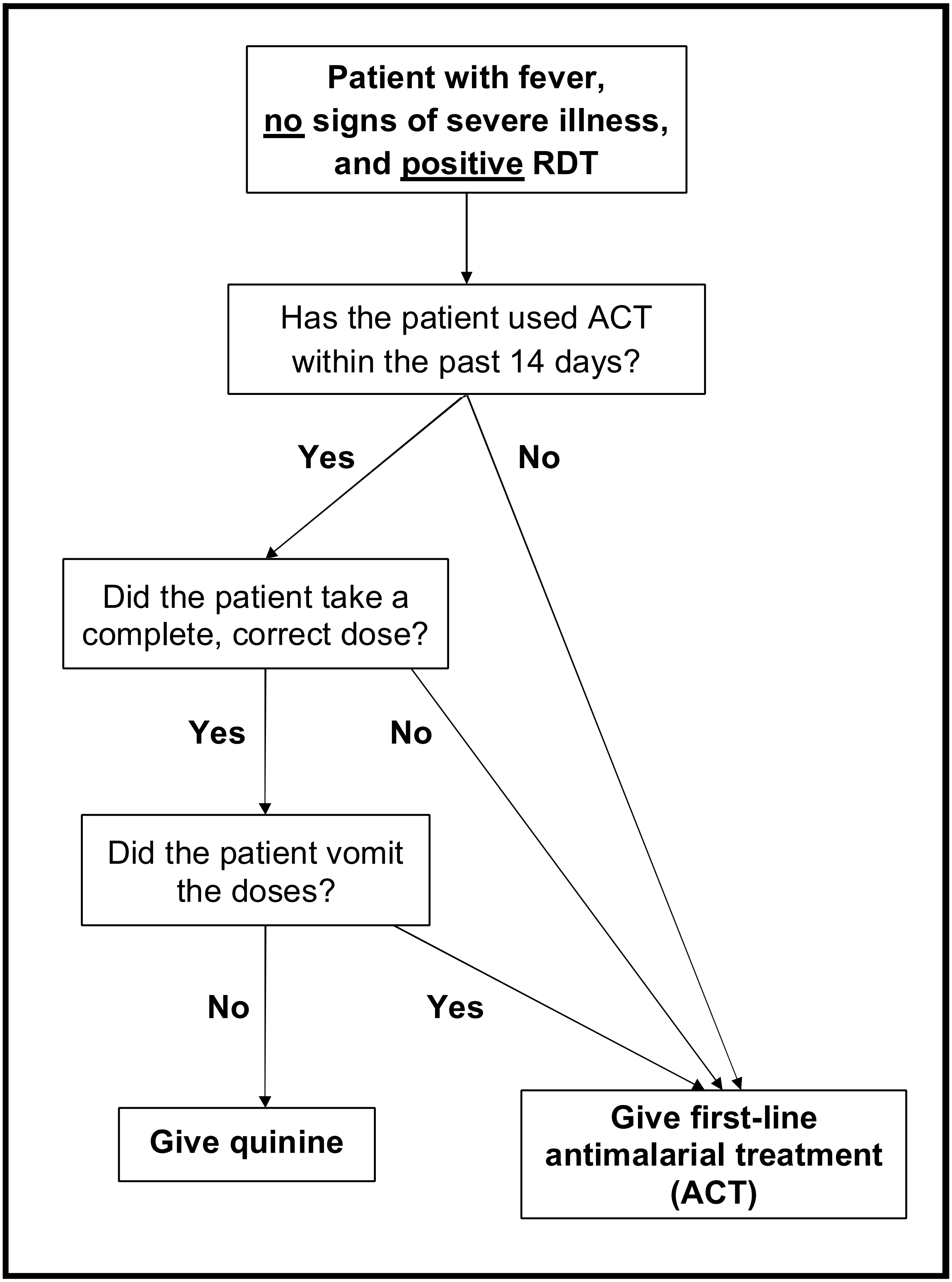

SUMMARY:

We have come to the end of our discussion on “Management of a patient with fever and positive RDT.” In this session we defined uncomplicated malaria, discussed antimalarial combination therapy, and reviewed the current guidelines for treatment of uncomplicated malaria in Uganda.

Remember: To correctly diagnose a patient with uncomplicated malaria, you should see symptoms including fever, NO signs of severe illness, AND a positive RDT.

REFERENCES:

1. Uganda Ministry of Health, “Management of Uncomplicated Malaria: A Practical Guide for Health Workers,” 3rd edition, Dec 2005.

Session 4: MANAGEMENT OF A PATIENT WITH FEVER BUT A NEGATIVE RDT

Content:

- ❑ **Introduction**
- ❑ **Learning objectives**
  1 **Benefits of treating on the basis of a RDT results**
  2 **Meaning of a negative RDT in a patient with fever**
  3 **Management of a patient with fever but a negative RDT**
  4 **Management of some common non-malaria febrile illnesses**
- ❑ **Summary**

INTRODUCTION:

Welcome to session 4 on "Management of a patient with fever but a negative RDT." Up to now, in many smaller health centres in Uganda, no diagnostic testing was available – so health workers gave antimalarial treatment based on symptoms and signs. This was recommended when older, safe and less expensive drugs like chloroquine (CQ) were effective against malaria.

However, now that artemisinin-based combination therapy (ACT) is required to effectively treat most true malaria cases, giving antimalarials without a using a diagnostic test is less accepted.

Giving antimalarial drugs to patients who do not have malaria leads to:

- Missing the opportunity to treat the true cause of a patient's illness
- Increased risk of drug stock-outs
- Increased risk of side effects
- Increased risk for development of drug resistant parasites

LEARNING OBJECTIVES:

By the end of this session, you should be able to:

1. Outline the benefits of treating patients on the basis of RDT results
2. Explain the meaning of a negative RDT in a patient with fever
3. Describe the management of a patient with fever but a negative RDT
4. List and describe the management of some common non-malaria febrile illnesses

4.1 BENEFITS OF TREATING ON THE BASIS OF RDT RESULTS

There are a number of possible benefits if you do not recommend antimalarial treatment for patients with negative RDTs. These include:

- You are more likely to focus on the true cause of fever.
- You may treat the true cause of fever in a timely manner.
- You can reduce the risk of antimalarial stock-outs in your health centre.
- You can help to limit the development and spread of drug resistance.
- You may reduce the patient's risk of side effects (drug reactions) due to unnecessary antimalarial treatments. A common example of a drug reaction is ringing in the ears after taking quinine.

4.2 MEANING OF A NEGATIVE RDT IN A PATIENT WITH FEVER

Let us begin this discussion by asking an important question: When caring for patients, why would we perform an RDT? Your response to this question might be something like: "To confirm the diagnosis and guide treatment decisions."

Then what does a negative RDT mean? **If performed correctly, a negative RDT means that the patient does not have malaria. The patient most likely has another disease that presents with similar symptoms as malaria.**

RDTs have been studied carefully in Uganda. If performed and read correctly, they are very accurate at diagnosing malaria. In different regions of Uganda, the RDT you have learned to use is able to detect *Plasmodium falciparum* infections even in patients with very low numbers of parasites in their blood. This gives us confidence that if the RDT is performed correctly, and the result is negative, the patient does not have malaria.

4.3 MANAGEMENT OF A PATIENT WITH FEVER BUT A NEGATIVE RDT

If a patient has fever but the RDT is negative, you should reconsider the history and clinical signs.

❑ **If the patient has a negative RDT, you can use the flow chart on the following page to manage his or her illness:**

FLOW CHART: Management of patient with a negative RDT for malaria

Patient with fever and negative RDT

↓

Does the patient show signs of non-malaria illness?

Refer to Session 1, Table 1; look carefully for other possible cause of fever

Yes → Treat non-malaria illness: use IMCI, Uganda Clinical Guidelines, and section 4.4 in this manual

No → Give supportive treatment for symptoms and ask patient to return in 1-2 days if symptoms continue or worsen

- Give paracetamol
- Counsel patient and be sure he or she understands the importance of returning to the health centre if symptoms worsen or do not resolve within 1-2 days

4.4 MANAGEMENT OF COMMON NON-MALARIA FEBRILE ILLNESSES

Let's return to Session 1, and review at the tables at the end of the session. As we discussed, the diseases listed in the tables include some of the most common causes of fever in children and adults. These are important diseases to consider when you are managing a patient with fever and a negative RDT.

In this section, we shall outline the management of some common non-malaria febrile illnesses:

1) Common cold (mild cough, runny nose = flu)
2) Pneumonia (lung infection)
3) Upper respiratory tract infection with sore throat = pharyngitis
4) Otitis media (ear infection)
5) Urinary tract infections
6) Typhoid
7) Bacterial meningitis

4.4.1 Common cold (flu)
(Uganda Clinical Guidelines pages 67-68 and IMCI page 10)

- ❑ **History:**

A patient with common cold has **flu (runny nose) with clear mucus**, and **mild cough**.

- ❑ **Physical examination:**

Look for clear or pale mucus in nostrils. If you have a stethoscope, listen to the patient's chest – if you hear rhonchi or crepitations, consider pneumonia (section 4.4.2) rather than common cold.

- ❑ **Treatment of common cold:**

- Give supportive treatment to relieve symptoms:
  - Relieve cough with fluids:
    - Tea with honey and lemon
    - Juice
    - For babies, breast milk
  - Panadol for fever
- No antibiotic is needed.

4.4.2 Pneumonia

(Uganda Clinical Guidelines pages 77-87 and IMCI pages 8 + 11)

Pneumonia is an inflammation of the lung tissue due to infection. The risk factors for pneumonia include:

- Age: young children are most in danger.
- Underlying medical illnesses: patients with HIV infection, diabetes, malnutrition, and sickle cell disease are at increased risk.
- Environmental conditions: smoke from wood or paraffin, especially in poorly ventilated dwellings may increase risk.
- Social conditions: over-crowding, smoking, alcohol and drug abuse, refugee conditions may increase risk.

❑ **History:**

A patient with pneumonia typically presents with **cough** and **fever**. The patient may or may not also have **thick pus-like sputum**, **chest pain that is worse with breathing and coughing**, and **difficulty breathing**.

❑ **Physical examination:**

- Examine for increased respiratory rate (i.e. fast breathing).
- Watch for signs of increased effort of breathing, such as nasal flaring, chest in-drawing, and use of neck and chest muscles.
- If you have a stethoscope, listen for decreased breath sounds and/or abnormal sounds such as rhonchi, crepitations and wheezes.

❑ **Treatment of pneumonia:**

Before you prescribe any treatment for a patient with pneumonia, you should consider whether the patient needs oral or injectable treatment. If the patient has severe illness, he or she will need injected drugs. If your health centre does not have injectable drugs, you should refer the patient urgently to a Health Centre IV or Hospital.

Table 4.1 Recommended antibiotic doses for treatment of pneumonia

Antibiotics	Adults	Children
**Amoxicillin**	500 mg 3 times a day (8 hourly) x 5 days	15-25 mg / kg 3 times a day (8 hourly) x 5 days
**Cotrimoxazole (Septrin)**	960 mg 2 times a day (12 hourly) x 5 days	24 mg / kg 2 times a day (12 hourly) x 5 days
**PPF**	20,000 IU / kg IM daily x 5 days	50,000 IU / kg IM daily x 5 days
**Chloramphenicol (oral or IV)**	1g 4 times a day (6 hourly) IV or IM then orally x 7 days	25 mg / kg 4 times a day (6 hourly) IV or IM then orally x 7 days

* IV = intravenous injection; IM = intramuscular injection

- **Other considerations in pneumonia treatment:**
  - Add **vitamin A** for children, if not received within the prior month.
    - For children aged 6-12 months, give 100,000 IU.
    - For children aged 1-5 years, give 200,000 IU.
  - If the respiratory illness does not respond to standard antibiotic treatment as above, consider tuberculosis. **If a patient's respiratory illness is rapidly progressing, or does not respond to standard antibiotic therapy, REFER him or her to a higher level health facility promptly.**
  - HIV-positive patients are at risk of a type of pneumonia called PCP. PCP cannot be treated with standard antibiotics. **If you suspect a patient may have PCP, REFER him or her promptly to a higher level health facility for high-dose cotrimoxazole treatment.**

4.4.3 Pharyngitis (upper respiratory infection with sore throat)
(Uganda Clinical Guidelines pages 237-238)

❑ **History:**

Ask about **throat pain**, **painful swallowing**, and **mild cough**. Patients may have a **hoarse voice** or feel **throat pain when talking**.

❑ **Physical examination:**

Look for **red throat and/or tonsils**. Patients may have a **white coating** over their throat and/or tonsils. Feel the anterior sides of the neck for **swollen and tender lymph nodes**.

❑ **Treatment of pharyngitis (sore throat):**

1) Give supportive treatment to relieve symptoms:
   - Relieve cough with fluids and/or linctus:
     - Tea with honey and lemon
     - Juice
     - Cough linctus
     - For babies, breast milk
   - Panadol for fever

2) If the patient has **fever, swollen lymph nodes in the anterior neck,** and **white coating over the throat,** the patient may have bacterial infection with **streptococcal pharyngitis (strep throat).** If so, give one of the antibiotics in Table 4.2.

Table 4.2 Antibiotic doses for treatment of streptococcal pharyngitis

Antibiotics	Adults	Children
**Benzathine penicillin**	1.2 million units x 1 injected (intramuscular) dose	If child weighs less than 30 kg: 30,000 units / kg x 1 injected (intramuscular) dose
**PPF**	20,000 IU / kg injected daily x 10 days	20,000 IU / kg injected daily x 10 days
**Phenoxymethyl-penicillin**	500 mg 6 hourly x 10 days	12.5 mg / kg 6 hourly x 10 days
**Erythromycin (if allergic to penicillin)**	500 mg 6 hourly x 10 days	12.5 mg / kg 6 hourly x 10 days

4.4.4 Otitis media (ear infection)
(Uganda Clinical Guidelines pages 223-224 and IMCI pages 8 + 11)

- **History:**

Ask about **ear pain**, **pulling** on ears, **pus discharge** from ear, **irritability**, and **fever**.

- **Physical examination:**

If you have an otoscope, examine for **bulging, irritated tympanic membrane**, with or without **pus discharge**.

- **Treatment of ear infection:**

- Give either cotrimoxazole or amoxicillin antibiotic therapy as in the tables:

Table 4.4 Cotrimoxazole (Septrin) doses for ear infection

Age or weight	Adult tablet<br>80 mg trimethoprim +<br>400 mg sulfamethoxazole	Pediatric tablet<br>20 mg trimethoprim +<br>100 mg sulfamethoxazole	Syrup<br>40 mg trimethroprim +<br>200 mg sulfamethoxazole<br>per 5 mL
2 to 12 months<br>(or 4 to 10 kg)	½ tab 2 times a day<br>(12 hourly) x 5 days	2 tabs 2 times a day<br>(12 hourly) x 5 days	5 mL 2 times a day<br>(12 hourly) x 5 days
1 year to 5 years<br>(or 10-19 kg)	1 tab 2 times a day<br>(12 hourly) x 5 days	3 tabs 2 times a day<br>(12 hourly) x 5 days	7.5 mL 2 times a day<br>(12 hourly) x 5 days
Patients weighing<br>more than 19 kg	2 tabs 2 times a day<br>(12 hourly) x 5 days	---	---

Table 4.4 Amoxicillin doses for ear infection

Age or weight	Tablet<br>250 mg	Syrup<br>125 mg per 5 mL
2 to 12 months<br>(or 4 to 10 kg)	½ tab 3 times a day<br>(8 hourly) x 5 days	5 mL 3 times a day<br>(8 hourly) x 5 days
1 year to 5 years<br>(or 10-19 kg)	1 tab 3 times a day<br>(8 hourly) x 5 days	10 mL 3 times a day<br>(8 hourly) x 5 days
Patients weighing<br>more than 19 kg	2 tabs (500 mg) 3 times a day<br>(8 hourly) x 5 days	---

- Advise the caregiver to dry the ear by **wicking** 3 times daily: Roll clean absorbent cloth or soft gauze into a wick. Place the wick in the patient's ear, and remove the wick when wet. Replace the wick with a clean one. Repeat these steps until the ear is dry.
- If possible, **follow up** and reassess the patient in clinic in 5-7 days.
- **Refer** the patient to a higher level health facility if the tympanic membrane is damaged, or if the patient returns repeatedly with signs of ear infection.

4.4.5 Urinary Tract Infection (UTI)

(Uganda Clinical Guidelines pages 241-246)

❑ **History:**

A patient with UTI typically presents with dysuria (**painful urination**) and **frequent urination**. He or she may also have haematuria (**blood in the urine**), and **fever**. Note that UTI is more common in women and girls than in men or boys.

❑ **Physical examination:**

On examination, the patient typically has lower abdominal tenderness (**pain on pressure over the urinary bladder**). Fever may or may not be present. The patient may also have renal angle tenderness (**pain with light pounding over the mid-back to either side of the spine**), which may indicate a more advanced infection reaching the kidney (pyelonephritis).

❑ **Treatment:**

To treat a patient with a UTI, you should consider the patient's age, and the severity of the disease.

See Table 4.5 for antibiotic doses in UTI treatment.

If possible, follow up the patient and reassess in 3-5 days.

- **If a patient with UTI does not respond to standard antibiotic therapy, REFER him or her promptly to a higher level health facility.**
- **If you suspect pyelonephritis (advanced infection reaching the kidney as above) or urosepsis (severe infection indicated by high fever and chills), injectable antibiotics should be used. REFER the patient promptly to a higher level health facility.**

Table 4.5 Recommended antibiotic doses for treatment of UTI

Antibiotic	Adults	Children
**Amoxicillin**	500 mg 3 times a day (8 hourly) x 5-7 days	15 mg / kg 3 times a day (8 hourly) x 7 days
**Cotrimoxazole (Septrin)**	960 mg 2 times a day (12 hourly) x 5-7 days	**By patient age:**<br>2 months to 12 months:<br>240 mg 2 times a day (12 hourly) x 5 -7 days<br>1 year to 5 years:<br>480 mg 2 times a day (12 hourly) x 5 -7 days

4.4.6 Typhoid fever (also known as **enteric fever**)

(Uganda Clinical Guidelines pages 25-26)

❑ History:

A patient with typhoid fever often reports **fever of gradual onset**, general **body aches** and pains, **loss of appetite**, **abdominal pain or discomfort**, **headache**, **diarrhoea or constipation** and **dry cough**.

❑ Physical examination:

The physical examination findings are often not specific, and depend on the duration and severity of the illness. Patients usually have **abdominal pain and tenderness.**

❑ Treatment:

See Table 4.6 for treatment recommendations for typhoid fever.

Table 4.6 Treatment of typhoid fever

Antibiotic	Adults	Children
**Chloramphenicol**	1000 mg 4 times a day (6 hourly) x 14 days	25 mg / kg 4 times a day (6 hourly) x 14 days
**Cotrimoxazole**	960 mg 2 times a day (12 hourly) x 14 days	24 mg / kg 2 times a day (12 hourly) x 14 days

4.4.7 Bacterial meningitis

(Uganda Clinical Guidelines pages 9-11)

❑ History:

A patient with bacterial meningitis typically presents with **fever**, **headache**, painful **stiff neck**, photophobia (desire to **avoid light due to eye pain**), and sometimes **vomiting** and **convulsions**. In babies, **failure to feed**, or in an older patient, **confusion**, may be the only signs of meningitis.

❑ Physical examination:

On examination, the patient may have **changed mental state** and may be **confused, irritable, very sleepy**, or **unconscious**. In children a **bulging anterior fontanel** may be seen. Patients with meningitis often have **severe neck pain and neck stiffness** so that the patient is **unable to touch the chin to the chest**.

❑ Treatment:

Bacterial meningitis is a very serious and dangerous disease. Treatment must be started as soon as possible. Delay in appropriate treatment could result in death or brain damage.

The only effective treatment for bacterial meningitis is injectable antibiotics. You should begin pre-referral treatment as in Table 4.7, and REFER the patient to a higher level facility IMMEDIATELY.

Table 4.7 Treatment of bacterial meningitis

Antibiotic	Adults	Children
**Chloramphenicol**	1000 mg 4 times a day (6 hourly) IV or IM then orally x 14 days	25 mg / kg 4 times a day (6 hourly) IV or IM then orally x 14 days

* IV = intravenous injection ; IM = intramuscular injection

☺ **SUMMARY:**

We have come to the end of our discussion on "Management of a patient with fever but a negative RDT." In this session, we have learned that in the era of artemisinin-based combination therapy (ACT), the best practice is to use a diagnostic test to determine whether a patient has malaria. This allows us to avoid giving antimalarials to patients who do not have malaria. We want to give antimalarial therapy to patients who truly have malaria, and avoid unnecessary antimalarial treatment of patients with a negative RDT.

When managing a patient with a negative RDT, you can follow the flow chart provided in this handout. You should always consider whether a non-malaria illness could be the cause of fever when taking history and examining a patient with fever. You can then treat or refer for non-malaria illness, as appropriate. Lastly, we emphasized that you should refer patients with fever and severe illness. You should also refer patients whose illness is not cured with initial treatment.

REFERENCES

1. Uganda Ministry of Health, *Uganda Clinical Guidelines: National Guidelines on Management of Common Conditions*, 2003.
2. WHO, Uganda Ministry of Health and UNICEF, "Integrated management of Childhood Illness" (IMCI) chart booklet.
3. *British National Formulary* (*BNF*) 51, BMJ Publishing Group Ltd, March 2006.
4. Lecture notes on Tropical Medicine, G.V. Gill and N.J. Beeching, 5th Edition
5. WHO guidelines for the treatment of malaria. WHO/ HTM/MAL/2006.1108
6. Uganda Ministry of Health, "Management of uncomplicated malaria: A practical guide for health workers," 3rd edition, December 2005.

Session 5: RECOGNITION AND REFERRAL OF PATIENTS WITH SEVERE ILLNESS

Content:

- **Introduction**
- **Learning objectives**
  1. **Symptoms and signs of severe illness**
  2. **Appropriate referral of patients with severe illness**
  3. **Pre-referral treatments for patients with severe illness**
- **Summary**
- **References**

INTRODUCTION:

Welcome to Session 5 on "Recognition and referral of patients with severe illness." In this session we will discuss how to recognize that a patient has severe illness that may be caused by malaria or another serious infection. We will also review guidelines on how to pre-treat and refer such patients to a higher level health care facility.

The symptoms and signs of severe illness that we will discuss here may result from malaria, from other infections, or more rarely from non-infectious illnesses. At lower level health centres, without laboratory diagnosis, it is often impossible to know the exact cause of such symptoms and signs. However, even without a definite diagnosis, **you can make an important difference in the patient's outcome by recognizing severe illness and acting quickly to offer pre-treatment and prompt referral to a Health Centre IV or Hospital.**

LEARNING OBJECTIVES:

By the end of this session, you should be able to:

1) List danger symptoms and signs of severe illness
2) Outline the steps to refer severely ill patients to higher level health facilities
3) Describe pre-referral treatments that may be given to severely ill patients before transfer to a higher level health centre

5.1 SYMPTOMS AND SIGNS OF SEVERE ILLNESS

The first step in helping patients with severe illness is to recognize the symptoms and signs that the patient is in danger. You should be especially careful to look for signs of severe illness in patients who are more likely to have severe infections including malaria: children younger than 5 years, pregnant women, and patients of all ages whose immune system is weak (e.g. HIV-positive patients). When evaluating a patient, you should ask about and watch for:

General danger signs:

1. **Convulsions** currently or at any time within the past two days
2. **Inability to drink or eat**, or **inability to breastfeed** for small children
3. **Vomiting everything** so that the patient is unable to keep down food, fluids, and medications
4. **Changed mental state** – confusion, lethargy (sleepiness), or unconsciousness (coma):

   a. In adults and older children, observe:
      - **Confusion**: Is the patient interacting with you and other individuals appropriately, or does he or she appear confused?
      - **Lethargy (sleepiness)**: Is the patient awake and attentive?
      - **Coma**: Can you wake the patient, or is he or she unconscious?

   b. In young children, observe:
      - **Confusion or lethargy (sleepiness)**: Does the child look at the mother or caregiver? Does the child follow an object moved in front of his or her eyes?
      - **Coma**: Does the child react to loud noises? If the child is sleeping, can you wake him or her with gentle shaking?

Other signs of severe illness:

5. **Extreme weakness** or prostration – unable to sit or stand without support
6. **Difficulty breathing** (respiratory distress):

   a. Is the patient **breathing faster than normal** for his or her age?
      - Younger than 2 months: 60 breaths per minute or more
      - 2 months to 12 months: 50 breaths per minute or more
      - 12 months to 5 years: 40 breaths per minute or more
      - 5 years or older: 20 or more breaths per minute

   b. In young children, watch for **chest in-drawing** and **nasal flaring**.
7. **Severe anaemia** – look for very pale palms, fingernails, tongue, and conjunctivae
8. **Severe dehydration** – look for dry mouth and tongue, sunken eyes, inability to drink or to keep down fluids, tenting of the skin (skin pinch goes back very slowly)

****If the patient shows any of the symptoms and signs listed above, you should consider the need for urgent treatment and referral to a higher level health facility.****

5.2 APPROPRIATE REFERRAL OF PATIENTS WITH SEVERE ILLNESS

The best treatment for a patient with severe illness requires injectable drugs and inpatient monitoring. If your health centre has facilities to administer injectable drugs and monitor patients in an inpatient setting, follow your usual practice to admit and treat the patient on the ward.

If your health centre does not have the facilities to administer injectable drugs and monitor patients in an inpatient setting, you should act as quickly as possible to REFER the patient to a higher level health centre. Follow the steps outlined here:

1) Provide pre-referral treatments as described in section 5.3 below.

2) Perform an RDT and write the result on the patient's referral note as below. Whether the RDT result is positive or negative, you should still **give all pre-referral treatments** as below.

2) Inform the patient and/or caregiver that the patient shows signs of severe illness, and requires urgent treatment including referral to a higher level facility.

3) Write a referral note stating:
   - Patient's name and age
   - Date and time
   - Symptoms and signs
   - RDT result
   - The name of any treatments/drugs given by you and the time they are given
   - Reason for referral

5.3 PRE-REFERRAL TREATMENTS FOR PATIENTS WITH SEVERE ILLNESS

It is often very difficult to determine the specific cause of illness in a patient with symptoms and signs of severe illness, as listed in section 5.1 above. Further diagnostic tests will be done at a higher level facility. **However, you should consider that any patient with the symptoms and signs above may have severe malaria.**

5.3.1 Pre-referral treatment with injectable antimalarial

Before referral, pre-treat the patient with **injectable quinine** as follows:

1) Obtain a 2 mL ampoule of quinine (600 mg total).

2) Add 4 mL sterile water to the ampoule. You now have a total of 6 mL of solution containing 600 mg quinine = 100 mg quinine per 1 mL.

3) The correct dose is **0.1 mL x body weight in kg, 8 hourly** until the patient can be referred or can take oral medicine. See Table 5.1 for some examples of how to calculate the dose:

Table 5.1 Example doses of injectable quinine

Patient weight	Dose of quinine
0.1 mL x 10 kg child	1 mL per injection
0.1 mL x 25 kg child	2.5 mL per injection
0.1 mL x 60 kg adult	6 mL per injection

4) Inject the dose into the **anterior (front) part of the thigh**. If the total dose is more than 3 mL, split the volume in two and inject half the dose into each thigh. **Do not inject into the buttock.**

5.3.2 Pre-referral treatment with injectable antibiotic

In addition to quinine as above, you should give an injectable antibiotic to any patient with symptoms and signs of severe illness. The antibiotic is given to treat a possible serious bacterial infection. Give benzyl penicillin or chloramphenicol as described in Table 5.2 until the patient can be transferred to a higher-level health center.

Table 5.2 Doses of pre-referral antibiotic

Antibiotics	Adults	Children
**Benzyl penicillin**	2 mega units IV or IM 4 hourly	100,000 IU / kg IV or IM 4 hourly
**Procaine penicillin**	---	4 to 10 kg (2 to 12 months): 400,000 units daily<br>10 to 19 kg (1 to 5 years): 800,000 units daily
**Chloramphenicol**	1g IV or IM 6 hourly	25 mg / kg (maximum 750 mg per dose) IV or IM 6 hourly

* IV = intravenous injection; IM = intramuscular injection

5.3.3 Pre-referral supportive treatment

While waiting for transfer to a higher level facility, you should also provide supportive treatment:

1) Reduce the fever – undress the patient, perform tepid sponging, fan the patient, and give Panadol (paracetamol).
2) If the patient has convulsions, give **diazepam**. Use solution with 10 mg / 2 mL, with a dose of 0.5 mg / kg as in Table 5.3. **Note: for patients older than 10 years, give 10 mg = 2 mL injection.**

Table 5.3 Doses of rectal diazepam (solution 10 mg / 2 mL)

Patient weight (or age)	Dose of quinine
4 to 6 kg (2 to 4 months)	0.25 mL
6 to 8 kg (4 to 12 months)	0.50 mL
10 to 14 kg (1 to 3 years)	0.50 mL
14 to 19 kg (3 to 5 years)	0.75 mL
20 to 40 kg (5 to 10 years)	1.5 mL

3) Prevent low blood sugar: give sugar solution orally if the patient is able (or ask the mother to breastfeed if the patient is a baby), or give sugar solution through an NG tube if the patient cannot drink.

4) If the patient is dehydrated (section 5.1), give fluids orally if the patient is able, or through an NG tube if the patient cannot drink.

*****For a patient with severe illness, every minute counts. Remember:**

- **Begin pre-referral treatment as soon as possible.**
- **Ensure referral to a Health Centre IV or Hospital as soon as possible.**

☺ **SUMMARY:**

We have come to the end of our discussion on "Recognition and referral of patients with severe illness." In this session, we have learned the symptoms and signs of severe illness, and how to refer such patients. We have also reviewed appropriate pre-referral treatment with quinine, and supportive treatments.

Be sure to ask about and watch for symptoms and signs of severe illness when evaluating febrile patients. Remember: severe illness is an emergency. Begin pre-referral treatment as soon as possible, and ensure referral to a higher level facility as soon as possible.

REFERENCES

1. Uganda Ministry of Health, "Management of Uncomplicated Malaria: A Practical Guide for Health Workers," 3rd edition, Dec 2005.
2. WHO, Uganda Ministry of Health and UNICEF, "Integrated management of Childhood Illness" (IMCI) chart booklet.
3. Uganda Ministry of Health, "Uganda Clinical Guidelines, 2003.

Session 6: PATIENT EDUCATION

Content:

- ❑ **Introduction**
- ❑ **Learning objectives**
  1. **Good communication skills**
  2. **Important messages to give a patient/caregiver to encourage adherence to treatment**
  3. **Important messages to give a patient/caregiver on symptoms that indicate a need to return for further care**
  4. **Messages on malaria prevention**
- ❑ **Summary checklist of key messages to give to patients**
- ❑ **Summary**

INTRODUCTION:

Welcome to Session 6 on "Patient education." Patient education is an exchange of information between the health worker and the patient and/or caregiver. As a health worker, an important part of your role is to provide information so that the patient or caregiver understands the diagnosis and treatment plan. A good health care provider shares information in a manner that is respectful and appropriate to the patient's or caregiver's level of understanding. Good communication skills are an important part of the health care process.

Our aim in patient education is to:

- ***Improve adherence to treatment***
- ***Improve patient follow-up care***
- ***Give preventive health messages***

LEARNING OBJECTIVES:

By the end of this session, you should be able to:

1. List 5 good communication skills
2. Outline important messages to give to a patient/caregiver to encourage adherence to treatment
3. Outline important messages to give a patient/caregiver on symptoms that indicate a need to return to for further care
4. Outline important messages to give a patient/caregiver on malaria prevention

6.1 GOOD COMMUNICATION SKILLS

In practicing good communication in patient education, you should:

1) **Ask questions and listen carefully** to answers given by the patient or caregiver. This ensures that you have as much information as possible to make a correct diagnosis and prescribe an effective treatment.

2) **Put the patient or caregiver at ease** so that he or she feels comfortable giving you honest and complete information about the illness, and trusting you to give correct advice and appropriate treatment.

3) **Give advice that is appropriate to the needs of each patient or caregiver.** Consider the messages below in sections 6.2, 6.3 and 6.4. Give explanations for your advice. If you need to correct the patient or caregiver, try not to criticize or to make him or her lose confidence.

4) **Use simple language** that can easily be understood by the patient/caregiver.

5) **Check to be sure the patient/caregiver understands the diagnosis made and treatment prescribed.** Encourage the patient/caregiver to ask any question he or she may have. You should also ask specific questions of the patient/caregiver to be sure he or she understands the treatment plan.

6.2 IMPORTANT MESSAGES TO GIVE A PATIENT OR CAREGIVER ON ADHERENCE TO TREATMENT

This section outlines the important messages you should give the patient or caregiver about the current episode of illness. These messages include the following:

6.2.1 The cause of this episode of illness:

- Based on your diagnosis, tell the patient or caregiver what the cause of the illness is – malaria, a viral illness, an ear infection, pneumonia, etc. This helps the patient or caregiver to understand the illness, and increases his or her confidence in the treatment recommendations.
- If the RDT result is positive, show the result to the patient/caregiver. Explain that the patient has malaria, and that you will prescribe antimalarial treatment.
- If the RDT result is negative, show the result to the patient/caregiver. Explain that the patient does not have malaria. Explain that not all fevers are caused by malaria. Tell the patient/caregiver that you will prescribe the appropriate treatment for the illness.

6.2.2 The treatment you recommend for this illness:

- The name of the specific treatment you are giving.
- The correct way to dose the drugs: number of tabs per dose, number of doses per day, number of days to complete the treatment.
- The correct way to take the drugs: for example, if you prescribe Coartem, tell the patient or caregiver to take each dose with milk (breastmilk for small children) or fatty or oily food (for example, meat or bean sauce made with cooking fat or oil, groundnut sauce, or odi) to improve absorption.
- Tell the patient about possible side effects of the treatment. However, mention that everyone is different and may react to a drug differently.
- Provide advice on storing medication: drugs should be stored in a clean and dry place, out of the reach of children.
- For any patient with fever, ensure the patient or caregiver understands how to use supportive treatment:
    - antipyretics (e.g. Panadol, ibuprofen)
    - adequate fluid intake: water, juice, and weak tea for adults and children who can drink, and breast milk for babies
    - tepid sponging for children and babies

6.2.3 Expected course of the illness:

Ensure that the patient or caregiver understands the following information:

- In order to be totally cured, the patient must take the full course of treatment.
- Symptoms may not disappear immediately after taking the first dose. Improvement may take up to two days.
- If symptoms worsen, or if they persist beyond two days, the patient should consult a health worker immediately.
- If the patient vomits the medicine within 30 minutes of taking the dose, he or she should take another dose.
- The patient should not change treatment by himself/herself. He or she should check first with a health worker.

6.3 IMPORTANT MESSAGES TO GIVE A PATIENT OR CAREGIVER ON SYMPTOMS THAT INDICATE A NEED TO RETURN FOR FURTHER CARE

A patient's condition may worsen even while he or she is on treatment. The patient should immediately go to a health facility if he or she has any of the following symptoms:

- Convulsions
- Persistent fever
- Severe vomiting or diarrhoea
- Becoming sicker
- Developing any new problem

6.3.1 How to tell that a child's condition is getting worse

You should advise the child's caregiver about symptoms that indicate that the child should go immediately to a health centre. Describe these symptoms in terms that the caregiver can understand so that she or he will recognize if the child requires urgent medical attention.

- Fever does not go away after two days of treatment
- Child develops convulsions
- Child is unable to eat, drink or breast feed
- Child becomes unconscious
- Child develops difficulty in breathing
- Vomiting begins or continues so that the child cannot keep down food, fluids and oral medications
- Child develops new symptoms
- Child becomes weaker or generally more ill
- Child develops a new rash – this may indicate an adverse drug reaction

6.3.2 How to tell that an adult's condition is getting worse

For an adult patient, you should advise him or her about symptoms that indicate a need to go to a health centre for further care:

- Fever does not go away after two days of treatment
- New symptoms occur
- Patient develops convulsions
- Patient is unable to drink or eat
- Vomiting begins or continues so that the patient cannot keep down food, fluids and oral medications
- Patient becomes weaker or generally more ill
- Patient develops a new rash – this may indicate an adverse drug reaction

6.4 MESSAGES TO GIVE A PATIENT OR CAREGIVER ON PREVENTION OF MALARIA

Your time with the patient or caregiver is an important opportunity to teach about ways to prevent malaria. Even if a patient has already heard these messages, being reminded by you as a health worker can help to reinforce the messages, and to encourage the patient's commitment to good prevention practices. You should counsel the patient or caregiver on the following topics:

- Explain the role of **mosquitoes** in malaria transmission (in particular, mosquitoes that spread malaria bite in the evening and night time)
- Explain the benefit of specific preventive measures such as:
  - Sleeping under an **insecticide treated net**.
  - The use of **intermittent presumptive treatment (IPT) in pregnant women** – this prevention method involves treatment with one dose of sulfadoxine-pyrimethamine (SP or Fansidar) in the second trimester, and one dose in the third trimester of pregnancy. IPT can be obtained from health centres and antenatal clinics across Uganda.
  - The Ugandan government is conducting **indoor residual spraying (IRS)** programs in some areas of Uganda. IRS sprays insecticide on the inside walls of a house to prevent and kill mosquitoes. If an IRS program is to be carried out in your area, the government will provide radio spots and community education to explain details of the program.

SUMMARY CHECKLIST OF KEY MESSAGES TO GIVE TO PATIENTS

☐ The treatment you are recommending and how to take it

☐ How to tell that a patient's illness is getting worse

☐ What to do if the patient's illness gets worse or does not improve

☐ Ways to prevent the illness from recurring

SUMMARY

We have come to the end of our session on "Patient education." In this session, we have reviewed important messages to give patients and caregivers. These messages encourage adherence to treatment, tell patients when to seek follow-up care, and teach about malaria prevention. Good communication skills are an essential part of fever case management, and of malaria prevention. Good communication involves asking and listening, putting the patient/caregiver at ease, offering advice, using simple language, and being sure the patient/caregiver understands what you have told him or her.

Session 7: RDT STORAGE AND MONITORING

Content:

- **Introduction**
- **Learning objectives**
  1. **Proper storage conditions for RDTs**
  2. **Monitoring RDT expiry dates at the health centre**
  3. **Ordering RDTs and disposing of used RDTs**
- **Summary**

INTRODUCTION:

Welcome to Session 7 on "RDT storage and monitoring." In this short session, we will discuss the proper way to store and monitor RDTs at the health centre.

LEARNING OBJECTIVES:

By the end of this session, you should be able to:

1. Describe the proper storage conditions for RDTs at your health centre
2. Explain how to monitor RDT expiry dates with the "FEFO" principle

7.1 PROPER STORAGE CONDITIONS FOR RDTS

RDTs must be stored in a **cool, dry place** in order to keep their ability to accurately diagnose malaria. Like drugs, RDTs' quality can be affected by heat and dampness. Therefore, the store room at your health centre, where the medicines are kept, is likely the best place to store RDTs.

RDT manufacturers recommend that the tests be stored between 4°C and 40°C. Uganda is a warm country, so we do not need to worry about RDTs reaching 4°C and freezing. It is more likely that RDTs could become too hot during transport or storage. However, in studies of RDTs in Uganda, the temperatures in storage rooms at health centres almost never went above 30°C. Therefore, the storage room at your health centre should provide a safe temperature for the RDTs.

Do not place RDTs near windows, where the sun can shine on them. Be sure that they are always kept as cool as possible in the storage room and in patient care areas.

7.2 MONITORING RDT EXPIRY DATES AT THE HEALTH CENTRE

During the monthly inventory at the health centre, **the expiry date on each RDT carton should be checked**.

Use the "**FEFO**" principle: First Expired, First Out. Put the carton with the earliest expiry date at the front of the storage area so that it is used soon.

If the expiry date on an RDT carton is past, do not use RDTs from the carton. If any RDTs have already been removed from the carton for use in the health centre, be sure they are removed from the health centre and replaced in the carton. Return the carton to Kampala.

☺ **SUMMARY**

We have come to the end of our session on "RDT storage and monitoring." In this session, we learned that RDTs should always be stored in a cool, dry place at the health centre. The expiry date on each carton should be checked during monthly inventory, using the "FEFO" principle.

Session 8: RDT LOGISTICS MANAGEMENT

Content:

- ❑ **Introduction**
- ❑ **Learning objectives**
  1. **Describe the purpose of a Laboratory Logistics System**
  2. **Outline the structure and flow of products/information for RDTs**
  3. **Describe some LMIS forms used in logistics management**
  4. **Perform stock status review**
  5. **Outline the procedure for ordering, receiving and storage of RDTs**
- ❑ **Summary**

INTRODUCTION:

Welcome to Session 8 on "RDT logistics management." In this session, we will discuss how to check RDT stocks at your health center, and how to order RDTs.

LEARNING OBJECTIVES:

By the end of this session you should be able to:

- Describe the purpose of a Laboratory Logistics System
- Outline the structure and flow of products/information for RDTs
- Describe some LMIS forms used in logistics management
- Perform stock status review
- Outline the procedure for ordering, receiving and storage of RDTs.

8.1 THE PURPOSE OF A LABORATORY LOGISTICS SYSTEM

The purpose of a logistics system is to ensure that products or supplies are available to the clients who will need them. It aims to provide the **Six Rights** of logistics system. A good logistics system provides excellent client/customer service by delivering:

- the **right product**
- in the **right quantity**
- in the **right condition**
- to the **right place**
- at the **right time** for the **right cost**

8.2 STRUCTURE AND FLOW OF PRODUCTS AND RDT INFORMATION

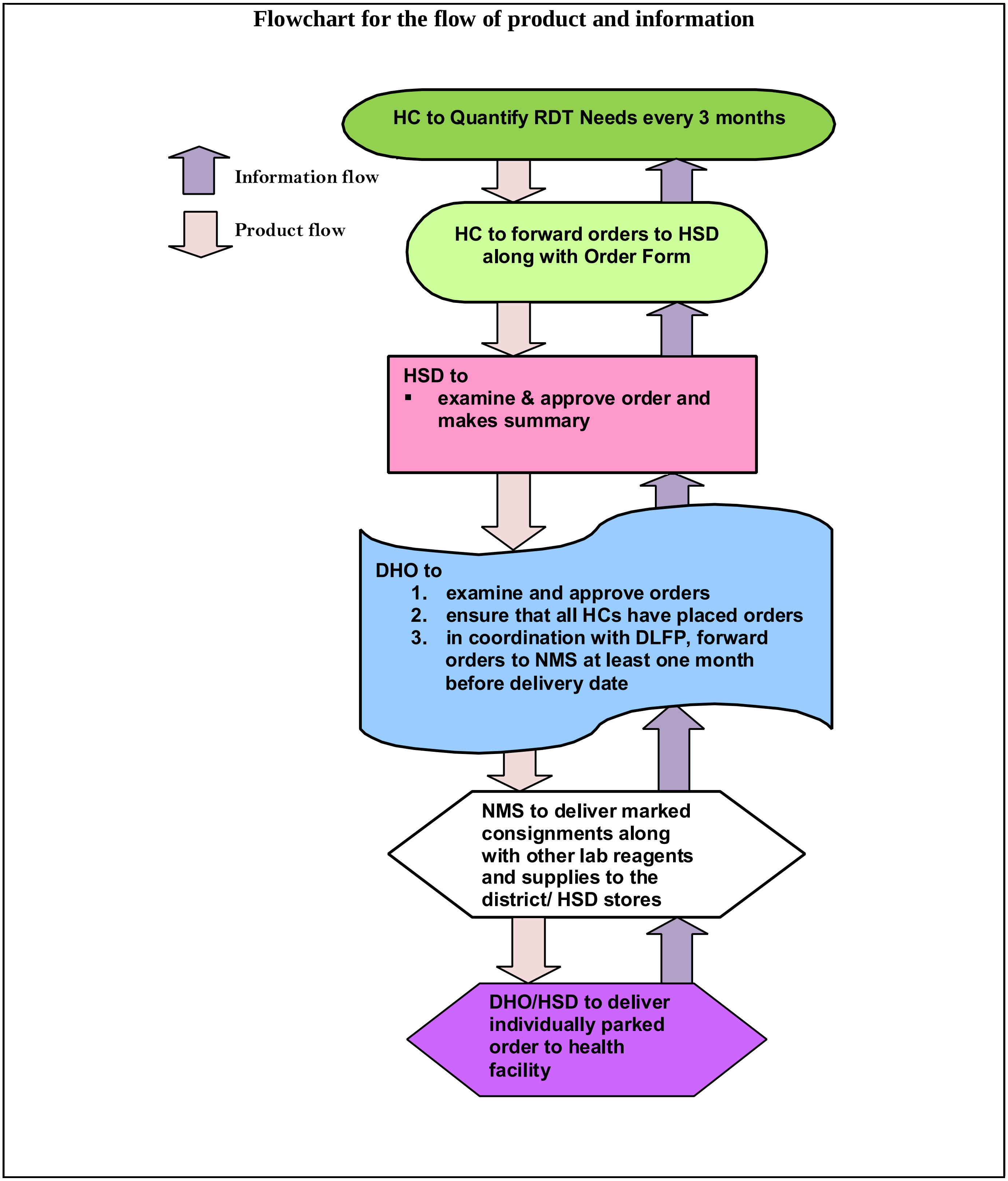

Note: Malaria RDTs are for use in Health Centers II and III, plus higher level health facilities that lack microscope services.

8.3 TYPES OF LABORATORY MANAGEMENT INFORMATION SYSTEM (LMIS) RECORDS

8.3.1 Stock keeping records

These are records for noting information about all products kept in storage (e.g. stock cards, ledgers, etc.)

8.3.2 Management of stock keeping records

A stock card is the basic record that is used to keep track of commodities that are found in storage or wherever commodities are kept.

HMIS 015: STOCK CARD

Health Unit Name: Financial Year___________ Page ______ of pages _____

Folio Number _________________ Card Number ____________

**Description:**	**Special Conditions**:
**Strength/ Size**	**Expiry Date(S)**

**Issue Unit:**	**AMC**	**Maximum Stock:**	**Minimum Stock:**	**Quantity To Order:**

**Date**	**To or From**	**Voucher Number**	**Quantity In**	**Quantity Out**	**Losses and Adjustments**	**Balance on Hand**	**Remarks / Batch Number**

8.3.3 Importance of stock card

- To track stock at hand, losses and adjustments
- To track quantity of RDTs issued (issue data) which may be used when calculating average monthly consumption when actual user data (dispensed data) is not available
- To keep continuous track of expiry dates

8.3.4 How to fill in the card

This should be demonstrated during the training.

8.3.5 When to complete the stock card

- At the time of physical count
- When products are received
- When products are issued
- Any other time there is a change in the status of the products in the store-room (example:

A physical count is the process of counting by hand the total number of units of each commodity in your storeroom or health facility at any given time.

- Do a physical count on a regular basis
- Check that recorded balances match actual quantities on the shelves
- Verify product quality
- Identify and correct errors in the stock card
- (other ideas given by the participants)

8.3.7 The link between a physical count and Product Quality Assurance

Conducting a physical count provides us an ideal opportunity to help ensure product quality

- Ensure that products are arranged according to FEFO
- Ensure that products do not show visible signs of damage
- Verify the product packaging before products are issued for use
- Read and record the room temperature if the thermometer is available
- (Other ideas given by the participants.)

8.3.8 Transaction records

These are records for keeping information about products being moved (e.g. packing slip, receiving report, issue voucher and requisition and issue voucher). Safe storage of all transaction records should be observed.

8.3.9 Consumption records

These are records for keeping information about products being consumed (daily consumption log, activity register etc.). Consumption refers to the quantities of RDTs used to perform tests over a given review period.

8.3.10 Types of consumption data

- **Dispensed-to-user data:** Information about the quantity of goods actually put in the hands of ultimate clients or end users (often shortened to "dispensed data").
- **Issues data:** Information about the quantity of goods shipped from one level of the system to another.

Note: This may involve malaria RDTs and Pregnancy tests depending on level of integration)

Name of health facility											
HSD											
District											
Daily Activity Register for Malaria RDT, TB and Pregnancy test											
Date	Lab No	Name	Age	Sex	Address	Serology					
						Malaria RDT		TB RDT		Pregnancy Test	
						Pos	Neg	Pos	Neg	Pos	Neg
Totals for each test done											

8.4 REVIEWING STOCK STATUS, CALCULATING AVERAGE MONTHLY CONSUMPTION, AND ORDERING

Before ordering new stock, review the following;

- How long is stock going to last?
- When do you need to order more stocks?
- What quantities of a particular item should be ordered?

To determine the **Average Monthly Consumption (AMC)**, add up the last three consecutive months' usage of a particular product, then divide by three. Get this data from the stock card (issues data) or from the RDT register (dispensed-to-user data)

Use the following formula to determine **Average Monthly Consumption**:

A) AMC = sum of 3 previous month's RDT usage = **AMC ÷3**

8.4.1 Reviewing stock status

What is your stock status?

When you review your stock status, you determine how much of each product you have available at your facility. You can review your stock status by counting the stock available, as you do during a physical inventory. When you finish, you will have an absolute quantity of stock available. But, when managing RDTs, it is much more important to know ***how long the stocks will last***. We refer to this as ***months of stock***.

What is "Months of Stock"?

Months of stock is the number of months a product will last based on the present consumption rate. The importance of Months of Stock inventory is:

To determine whether your facility/unit is

- Under stocked
- Overstocked
- Adequately stocked

If you are under stocked, you may need to place an emergency order. If you are overstocked, you may need to redistribute the stock to other facilities.

Use the following formula

$$\frac{\text{Stock on Hand}}{\text{Average Monthly Consumption}} = \text{Months of Stock}$$

Example:

If the health facility has 1,000 RDTs and the average consumption rate is 500 RDTs per month, the Months of Stock will be:

$$\frac{1{,}000}{500} = 2 \text{ Months of Stock}$$

8.5 Ordering RDTS and Disposing of used RDTS

- RDTs will be availed to the health centres through a push system until NMS finalizes plans for regular supplies.

Steps in completing the HMIS 018 B Order Form (Section for Donated RDTs)

National Medical Stores/Joint Medical Stores
RADPID DIAGNOSTIC TESTS (RDTs) Order form

Facility Name:

Level of Health Facility: ______________________

District______________________

			Number of Test Available at the Beginning of the two months	Total Number of Tests Received During the two months	Total Number of Tests Used During the two months	Losses / Adjustments (+/-)	Closing Balance	Maximum Stock Quantity	Quantity to Order (C-E)
NMS Code	Item Description	Basic Unit	A	B	C	D	E= A + B – C +/- D	F = C x 3	G
	Malaria RDT								
	Lancets								
	Others								
	Gloves								
	Cotton wool								
	Spirit								

**Order prepared by:**	**Date:**	**Phone No:**
**Facility in charge:**	**Date:**	**Phone No:**

National Medical Stores/Joint Medical Stores<br>HSD Order Summary- Rapid Diagnostic Tests (RDTs)					
Facility Name:-_______________________________________		Level of Health Facility_____________________________________<br>District____			
In the table below, fill in the name and level for each health unit ordering. Enter the quantities of each of the respective Rapid Diagnostic Tests (RDTs) ordered by the facilities.					
			Quantity of RDTs ordered		
No.	Name of Health Unit	Level (II,III)	Malaria RDT	TB RDTs	Pregnancy test
1					
2					
3					
4					
5					
6					
7					
8					
		Total RDTs			
	HSD in charge signature:	Date:		Phone No:	
	DHO Signature:	Date:		Phone No:	

8.5.2 Receiving and storage of RDTs

8.5.2.1 Coordinating receipt of RDTs and storage

Maintaining proper storage conditions for laboratory commodities is vital to ensuring their quality. Product expiry dates are based on ideal storage conditions and protecting product quality until their expiration date.

8.5.2.2 Procedures for receiving RDTs and other laboratory supplies

- Ensure that there is sufficient storage space available
- Prepare and clean the area used for receiving and storing the products
- Inspect the products for damage and expiry
- Separate the damaged or expired products from the usable stock
- Count the number of units for each product received and compares to issue voucher
- Inspect for completeness of the kit
- Record the date and quantity received on stock card, and bin card if applicable
- Ensure the expiry date is clearly marked and visible

8.5.2.3 Summary guidelines for proper storage of laboratory reagents and other supplies

These are the general storage procedures to be followed regardless of the size of facility. These rules can be adapted for each facility within reason.

- Clean and disinfect storeroom regularly
- Store lab supplies in a dry, well lit and well ventilated storeroom, out of direct light
- Secure the storeroom from water/animal (rodents) penetration
- Ensure that fire safety equipment is available and accessible and that personnel are trained to use it
- Store latex away from electric motors and florescent lights
- Maintain cooled storage for reagents which require cool chain e.g. some testing kits
- Limit storage area access to authorized personnel and lock up controlled lab reagents
- Store flammable reagents separately using appropriate safety precautions
- Store laboratory supplies away from pesticides, chemicals, and other hazardous material
- Arrange cartons/reagent bottles so that arrows are facing up, and ensure that identification labels, expiry dates, and manufacturing dates are visible
- Store laboratory reagents and supplies in a manner accessible for FEFO, accounting and general management
- Separate and dispose of damaged or expiry products

8.5.3 Disposing of expired RDTs

The RDT should be disposed of with the rest of the medical waste from the health centre, including used gloves, used spirit swabs (alcohol swabs) and other items. For example, many health centres discard waste in a garbage pit. The pit should be in a place where children and animals cannot easily reach it.

Annex 1: Exercise for completing the stock card for RDTs

Annex 2: Job Aid for completing logistic forms

Annex 2: Exercise for completing the Daily Activity Register for RDTs

APPENDIX H: USE OF RDTS IN FEVER CASE MANAGEMENT
PRE – POST TRAINING ASSESSMENT (1)

HEALTH WORKER INFORMATION

Cluster Number	Health Centre Code	Health Worker Study ID	Date
[____|____]	[____|____]	[___|____]	[____|____] / [____|____] / [___|____]<br>day month year

Health Worker Position			
[_____|_____]	1 = In-charge<br>2 = Senior medical officer<br>3 = Medical officer<br>4 = Senior clinical officer<br>5 = Clinical officer	6 = Nursing officer<br>7 = Enrolled nurse<br>8 = Midwife<br>9 = Public health nurse<br>10 = Nursing aide/assistant	11 = Laboratory technician<br>12 = Laboratory assistant<br>13 = Health assistant<br>14 = Health educator<br>15 = Other_______________

PART 1: INTRODUCTION

Welcome to the pre-test for our training course in RDTs and fever case management. There are 16 questions in the pre-test.

The reason for this test is not really to see how much you already know. The pretest is designed to raise your interest in RDTs and fever cases. Each of these questions highlights an important learning point that we will cover during the course. By the end of the course, you will have learned everything you need to answer these questions correctly. On the last day of the course, we shall take this test again, and then discuss the answers as a group.

Instructions:

1. Read each question carefully along with the group.
2. Put the letter to the answer you think is correct in the space on the right, for example: [__b__]

PART 2: QUESTIONS & ANSWERS

Questions	Answers
? All cases of fever in Uganda are caused by malaria.<br>a) True<br>b) False	[____]
? RDT stands for Rapid Diagnostic Test for malaria. Which of the following is NOT true about RDTs?<br>a) If performed correctly, they are very accurate in diagnosing which patients have malaria and which do not.<br>b) They can be performed in health centres that do not have power or laboratory equipment.<br>c) They can detect typhoid fever and pneumonia as well as malaria.	[____]
? In order to perform an RDT, you will need to use finger prick blood. After you prick a patient's finger with a lancet, what should you do with the lancet?<br>a) Save it to wash and use again later.<br>b) Carefully put it immediately in the sharps container.<br>c) Put it on the table beside you, to use for the next patient.	[____]
? We will pass an RDT around the room. Look carefully at it and read the result.<br>a) Positive<br>b) Negative<br>c) Invalid	[____]
? Here is another RDT. Look carefully at it and read the result.<br>a) Positive<br>b) Negative<br>c) Invalid	[____]

PRE – POST TRAINING ASSESSMENT (2)<br>HEALTH WORKER INFORMATION			
Cluster Number	Health Centre Code	Health Worker Study ID	Date
[____|____]	[____|____]	[____|____]	[____|____]/ [____|____]/ [____|____]<br>day month year

PART 2: QUESTIONS & ANSWERS CONTINUED		
?	RDTs should be stored in a cool, dry place.<br>a) True<br>b) False	[____]
?	You can use the same RDT for two different patients.<br>a) True<br>b) False	[____]
?	You should always check the expiry date on the package of an RDT before using it.<br>a) True<br>b) False	[____]
?	For which of the following patients should you perform an RDT for malaria?<br>a) A 4-year-old boy with fever and mild cough<br>b) An 8-month-old child with fever who refuses to breastfeed, is breathing rapidly and has very pale palms and sunken eyes<br>c) A 20-year-old woman who is pregnant and has fever<br>d) A 32-year-old man who is HIV-positive and has fever<br>e) A 45-year-old man with fever and joint paints<br>f) All of the above	[____]
?	A patient comes to your health centre with fever. She has taken chloroquine for two days but the fever has persisted. You perform an RDT and the result is positive. What should you do?<br>a) Tell the patient to complete the course of chloroquine.<br>b) Prescribe a full course of Coartem.	[____]
?	A patient comes to your health centre with fever. She took a complete, correct course of Coartem last week. She did not vomit any of the doses. You perform an RDT and the result is positive. What should you do?<br>a) Prescribe a full course of quinine.<br>b) Prescribe a second full course of Coartem.<br>c) Advise her to take Panadol and fluids only.	[____]
?	Which of the following is a symptom or sign of severe illness in a patient with fever?<br>a) Convulsions currently or within the past 2 days<br>b) Extreme weakness – patient is unable to sit or stand without support<br>c) Severe anaemia – patient's palms and conjunctivae are very pale<br>d) Unable to drink or breastfeed<br>e) All of the above	[____]
?	A 35-year-old man comes to your health centre. He tells you that he has fever and muscle aches. What should you do FIRST?<br>a) Prescribe Coartem immediately.<br>b) Prescribe chloroquine immediately – this regimen is best for adults.<br>c) Prescribe Panadol only and tell the patient to go home and rest.<br>d) Ask the patient how long he has had these symptoms, and ask if he has taken any drugs at home before coming to the health centre.	[____]

PRE – POST TRAINING ASSESSMENT (3)<br>HEALTH WORKER INFORMATION			
Cluster Number	Health Centre Code	Health Worker Study ID	Date
[____|____]	[____|____]	[____|____]	[____|____]/[____|____]/[____|____]<br>day month year

PART 2: QUESTIONS & ANSWERS CONTINUED		
?	A mother brings her 8-year-old daughter to your health centre. Beginning yesterday, the girl has had fever and no appetite. Her mother has given Panadol and fluids, but the symptoms continue. There are no signs or symptoms of severe illness. On physical examination, you find no obvious cause of her fever. You perform an RDT, and the result is negative. Which of the following is TRUE?<br><br>a) This patient has a mild febrile illness, but the cause is not clear. The mother should continue to provide fluids and Panadol. You should advise her to bring the girl back to the health centre if the symptoms do not get better within 2 to 3 more days.<br>b) You should prescribe a course of chloroquine. Even though the RDT is negative, the girl may still have malaria, and chloroquine is appropriate for mild cases of malaria.<br>c) You should prescribe Coartem. Even though the RDT is negative, the girl may have malaria, and Coartem is the first-line regimen for malaria in Uganda.	[___]
?	A 10-year-old boy comes to your health centre with fever and joint pains. He looks weak, but is able to sit up easily by himself. After you take a history and do a physical examination, you perform an RDT. The RDT is positive, and you plan to treat for uncomplicated malaria. According to the Uganda Ministry of Health guidelines, which of the following is the BEST (first-line) treatment for this patient?<br><br>a) Chloroquine<br>b) Oral quinine<br>c) Injectable quinine<br>d) Coartem (artemether-lumefantrine)<br>e) Chloroquine + Fansidar (Homapak)	[___]
?	A mother brings her 1-year-old son to your health centre. She tells you he has not been feeding well. The child's body is hot to touch. He is weak and cannot sit up by himself, and he is breathing very rapidly. What should you do? Be sure to read all the statements below before answering.<br><br>a) Undress the child, and ask the mother to sponge him to help reduce the fever.<br>b) Immediately prepare and inject a dose of quinine AND antibiotic.<br>c) Perform an RDT and write the result on the referral note.<br>d) Write a referral note and ensure that the child is referred to a Health Centre IV or Hospital as soon as possible.<br>e) All of the above.	[___]

Thank you!

APPENDIX I. INFORMATION SHEET
Health worker self-observation activities
ACT PRIME Study

Introduction

Dr. Sarah Staedke and colleagues from the Uganda Malaria Surveillance Project / Infectious Diseases Research Collaboration are investigating the provision of health care services in Tororo District. We are doing a research study to see if we can improve the health of children in this area by improving services at government-run health facilities. Certain health centers in Tororo district will be selected to take part in the intervention to improve services, or to continue with their current services. Assignment to the two groups has been determined by a lottery. The chance of being placed into either of the groups is the same.

Why are these self-observation activities being done?

We would like to know more about the interaction between health workers and patients in this area. To do this, we are asking health workers to take part in a series of self-observation activities about their behaviors and experiences interacting with patients. This information will help us to plan future training activities and health care studies in Tororo District.

What will happen if I take part in these self-observation activities?

We would like your participation in a series of self-observation activities in which you will reflect on your interpersonal behaviors and how you interact with patients. We will start with a two-hour introduction to the planned activities. You will then be asked to complete a series of tasks over a period of 8-12 weeks. The activities involve becoming aware of your behavior and writing a short summary of how your behavior affects those around you and your ability to achieve work goals. On completion of each task, you will be invited to join other health workers to discuss your observations and give support to each other. Over the 8-12 week period, we will carry out four meetings lasting approximately two hours each. We will ask you to give your written summaries to the workshop trainers. The summaries will be typed and stored electronically. We plan to use the summaries to help plan training activities for the future. All information gathered will be treated as confidential by the study personnel, and records of the interviews will be kept securely in locked filing cabinets and offices. No personal identification information such as names will be used in any reports arising out of this research.

How long will these self-observation activities last?

The introduction to the self-observation activities will last about 2 hours. Over the 8-12 week period, you will carry out four self-observation tasks and there will be four colleague meetings lasting approximately two hours each.

Can I stop being in the self-observation activities?

You can decide to stop participating at any time. Just tell the project researcher right away if you wish to stop the activities.

What risks can I expect from participating in the self-observation activities?

Participation in any research study may involve a loss of privacy. Information you provide about your experiences and opinions will be recorded, but your name will not be used in any reports of the information provided. No quotes or other results arising from your participation in this study will be included in any reports, even anonymously, without your agreement. The information obtained from these self-observation activities will only be used by the project researchers and will be locked at our project offices. We will do our best to make sure that the personal information gathered for this survey is kept private.

Are there benefits to taking part in these self-observation activities?

While we do not anticipate any immediate benefits to you, the self-observation activities may help you to develop an awareness of your interpersonal interactions with your colleagues and patients. Additionally, the information that you provide will help researchers plan for future training activities and health care studies in this area.

What other choices do I have if I do not take part in the self-observation activities?

You are free to choose not to take part in the self-observation activities. If you decide not to take part, there will be no penalty to you.

What are the costs of taking part in these self-observation activities? Will I be paid for taking part in these self-observation activities?

There are no costs to you for taking part in this survey. You will not be paid for taking part in this survey.

What are my rights if I take part in these self-observation activities?

Taking part in these self-observation activities is your choice. You may choose either to take part or not to take part. If you decide to take part in these self-observation activities, you may change your mind at any time. No matter what decision you take, there will be no penalty to you in any way.

Who can answer my questions about the self-observation activities?

You can talk to the researchers about any questions or concerns you have about these self-observation activities. Contact Dr. Sarah Staedke or other members of the Uganda Malaria Surveillance Project / Infectious Diseases Research Collaboration on telephone number 0414-530692. If you have any questions,

comments or concerns about taking part in these self-observation activities, first talk to the researchers. If for any reason you do not wish to do this, or you still have concerns about doing so, you may contact Dr Charles Ibingira, Makerere University Faculty of Medicine Research and Ethical Committee at telephone number 0414-530020.

Giving verbal consent to take part in the self-observation activities:

You may keep this information sheet if you wish. Participation in these activities is voluntary. You have the right to decline to take part in the activities, or to withdraw from them at any point without penalty. If you do not wish to take part in the activities, you should inform the researcher now. If you do wish to take part in these activities, you should tell the researcher now, and the introduction session will begin shortly. If you do not agree to quotes or other results arising from your participation in the study being included, even anonymously, in any reports about the study, please tell the researcher now.

THE REPUBLIC OF UGANDA

APPENDIX J. EXAMPLE HEALTH WORKER SELF-OBSERVATION TASK SHEET #1
Tororo District Survey Project

Observation Task 1: How you ask questions

Notes to the participant

Our personal communication style is an important factor in how we interact with others, whether we are consulting with patients, interacting with colleagues, or simply having a conversation. Developing awareness about how we communicate, and how our communication affects the person(s) we are communicating with, is a very important task in becoming a good health care worker. Understanding how we function, and what works well (and not so well) is the first step to understanding others. By paying conscious attention to how you communicate, you will become a better communicator.

Thus, you are invited to observe your own communication practices. An important key to making effective observations is focus: If you look for one or a few things, you will be able to see the pattern in what you are doing, and become aware of what you need to learn more about – and what you do well, and can help others learn from. If you look at too much at the same time, you will not see the patterns. Thus, you should follow the schedule proposed – look at one issue at a time for one week until you meet the facilitator again.

Looking at not only what you are saying or doing, but also at how you say and do it, is very important. The effect of what you say and do on others is also crucial. Start looking at this, and at the feelings you have – and at what you cause in the other person. Understanding the effect of feelings on communication outcome is key in this learning.

Tips for carrying out your observations:

- Carry this page of instructions in a notebook.
- When you plan your day, plot in one or two times or situations when you are know you will be interacting with patients or others in your workplace when you plan to observe yourself.
- Before the consultation/meeting, read the instructions again to remind yourself what you are looking for.
- Try to be aware during the meeting or conversation how you behave regarding the habit you are observing.
- After the meeting/event, reflect on what you have observed in your own behaviour, and make a few notes in your notebook.

THE REPUBLIC OF UGANDA

If you do this once or twice a day, you will start to see a pattern. And – discuss with your colleagues about how they do their observation, and may be what they have found out. This may help you to get used to doing this task.

Please observe the following when you interact with others:

1. When you ask questions of patients, do you usually:
    - Ask open-ended questions to allow the patient to tell their story in their own words?
    - Ask the patient a series of close-ended questions to get answers about specific symptoms?
    - Ask probing questions to find out more about what is troubling the patient if you feel that they haven't told you everything?
    - Listen patiently while the patient is speaking?
    - Tend to feel pressured by time, and become impatient if the patient is telling a long story or asking many questions?
    - Wait until the patient has completed their story, and you have asked any additional questions, before making a decision about the patient's likely diagnosis?
    - Often formulate your decision about a patient's diagnosis before you have finished taking the complete history, because you already know the problem?
    - Try to understand the patient's perspective and what their needs are while you take the history?
    - Do you notice any other patterns to your behavior when asking questions of patients? (Describe)

2. Observe in what type of situations you use the different methods, and what are the results or outcome (Do you feel good/bad/indifferent? Does the other person feel good/bad/indifferent?)

THE REPUBLIC OF UGANDA

APPENDIX J. EXAMPLE HEALTH WORKER SELF-OBSERVATION TASK SHEET #2
Tororo District Survey Project

Observation Task 2: How you facilitate or hinder good communication

Notes to the participant
During the last self-observation task, you started to observe your personal communication style by observing how you ask questions and how you listen to others: Do you try to really listen to find out what their ideas are, or are you more concerned about getting the other person to listen to your opinion and ideas? Or do you do a bit of both, depending on the situation? If both, you should be able to define situations for each habit and why.

This week, you are invited to observe how you facilitate or encourage communication with others and how you prevent or block good communication. You should pay attention to how your verbal and non-verbal actions affect your communication style and how you communicate with others. You can continue to pay attention to your listening habits as you start on this week's task of observing your discussion habits.

You will now be more comfortable with self-observation and understanding the feelings you have – and how they evoke feelings in another person. Understanding the effect of feelings on communication outcome is key in this learning.

Remember these tips for carrying out your observation tasks:

- Carry this page of instructions in a notebook.
- When you plan your day, plot in one or two times or situations when you are know you will be interacting with patients or others in your workplace when you plan to observe yourself.
- Before the consultation/meeting, read the instructions again to remind yourself what you are looking for.
- Try to be aware during the meeting or conversation how you behave regarding the habit you are observing.
- After the meeting/event, reflect on what you have observed in your own behaviour, and make a few notes in your notebook.

If you do this once or twice per day, you will start to see a pattern. Remember that the key to useful observation is to focus: concentrate on observing only one or two habits at a time. Continue to discuss with your colleagues about how they do their observation, and may be what they have found out. This may help you understand more about yourself.

THE REPUBLIC OF UGANDA

This week, observe how you facilitate or hinder good communication with others.

1. When you discuss with another person, do you usually:
   - Maintain eye contact?
   - Sit and face the person squarely or turn away from the person?
   - Use simple terms or complex medical jargon?
   - Ask which language the person would prefer to use, if you see the person is having difficulty speaking English?
   - Use non-verbal cues such as head nodding and smiling to show that you are paying attention?
   - Respond to the person's statements with your own opinions?
   - Ask questions to find out more what the other person is thinking?
   - Ask questions that lead to yes/no answers or to more descriptive answers?
   - Any other pattern? (Describe)

2. Observe in what type of situations you use the different methods, and what are the effects or results or outcome (Do you feel good/bad/indifferent? Does the other person feel good/bad/indifferent?). Don't forget to make notes!

THE REPUBLIC OF UGANDA

APPENDIX J. EXAMPLE HEALTH WORKER SELF-OBSERVATION TASK SHEET #3
Tororo District Survey Project

Observation Task 3: What do you do to relieve work-related stress and anxiety?

Notes to the participant

During the last self-observation task, you started to observe how your discussion habits may facilitate or hinder good communication: Do you use open and welcoming body language? Do you use simple language or complex medical terms? Do you practice active listening? Do you ask questions for clarification or make your own assumptions about what people are trying to tell you?

This week, you are invited to observe what you do to relieve work-related stress and anxiety. You should pay attention to the type of situations that cause you to feel stressed or anxious. Observe if you react in a way that has a positive or negative outcome on yourself and others around you. You can continue to pay attention to your discussion habits as you start on this week's task of observing your reactive emotions and behaviours.

Remember these tips for carrying out your observation tasks:

- Carry this page of instructions in a notebook.
- When you plan your day, plot in one or two times or situations when you are know you will be interacting with patients or others in your workplace when you plan to observe yourself.
- Before the consultation/meeting, read the instructions again to remind yourself what you are looking for.
- Try to be aware during the meeting or conversation how you behave regarding the habit you are observing.
- After the meeting/event, reflect on what you have observed in your own behaviour, and make a few notes in your notebook.

If you do this once or twice per day, you will start to see a pattern. Remember that the key to useful observation is to focus: concentrate on observing only one or two habits at a time. Continue to discuss with your colleagues about how they do their observation, and may be what they have found out. This may help you understand more about how to interact with your colleagues.

This week, observe what you do to relieve work-related stress and anxiety.

1. List situations that trigger work-related stress or anxiety. These may be issues to do with your relationship with your colleagues/boss, your relationship with patients, or with your daily duties.

2. When faced with these situations, do you usually:
   - Avoid the situation or interaction that triggers the anxiety?
   - Pretend nothing is wrong and simply proceed hoping that the situation will resolve on its own?
   - Seek for an opportunity to openly address the problem?
   - Seek help from colleagues or other sources about how to handle the stressful situation?
   - Redirect your stress or anxiety, taking it out on colleagues, patients, or others?
   - Find and perform an activity that relaxes you and relieves the stress or anxiety?
   - Any other pattern? (Describe)

3. Observe in what type of situations you use the different methods, and what are the effects or results or outcome (Do you feel good/bad/indifferent? Does the other person feel good/bad/indifferent?) Don't forget to make notes!

APPENDIX J. HEALTH WORKER SELF-OBSERVATION TASK SHEET #4
Tororo District Survey Project

Observation Task 4: How do you handle anger and irritation?

Notes to the participant

During the last self-observation task, you started to observe how your reactive emotions and behaviours impacted on how you relieve work-related stress and anxiety: Do you avoid situations that trigger anxiety or seek advice on how to face them? Do you find an activity that relaxes you? Do you avoid confrontation or establish open dialogue with colleague(s) involved in order to resolve the situation? Are you aware of how your stress and anxiety may interfere with your daily functions, job satisfaction as well as hinder good communication?

This week, you are invited to observe how you handle anger and irritation at work. You should pay attention to the difference between feelings of stress or anxiety and feelings of anger or irritation. These are two different types of emotions that can cause different types of reactions. Observe if feelings of stress or anxiety are related to your own actions while feelings of anger or irritation are related to the actions of others around you. Continue to pay attention to your reactive emotions and behaviours in these different situations.

Remember these tips for carrying out your observation tasks:

- Carry this page of instructions in a notebook.
- When you plan your day, plot in one or two times or situations when you are know you will be interacting with patients or others in your workplace when you plan to observe yourself.
- Before the consultation/meeting, read the instructions again to remind yourself what you are looking for.
- Try to be aware during the meeting or conversation how you behave regarding the habit you are observing.
- After the meeting/event, reflect on what you have observed in your own behaviour, and make a few notes in your notebook.

If you do this once or twice per day, you will start to see a pattern. Remember that the key to useful observation is to focus: concentrate on observing only one or two habits at a time. Continue to discuss with your colleagues about how they do their observation, and may be what they have found out. This may help you understand more about yourself and your colleagues.

THE REPUBLIC OF UGANDA

This week, observe what you in situations that cause anger and irritation.

1. List situations that may cause anger and irritation. These may be issues to do with your relationship with your colleagues/boss, your relationship with patients or simply your daily duties. Remember to try and differentiate between stress/anxiety and anger/irritation.

2. When faced with these situations, do you usually:

- Express your anger by yelling? Or react to your feelings by crying?
- Try to suppress your anger and bottle-up your feelings?
- Try to understand the reasons the situation or person made you angry?
- Try to understand what caused the situation, or the reasons for a person's behavior that made you angry?
- Avoid situations or people that might lead to conflict? Or do you work towards resolving conflict?
- Look for an opportunity to openly address problems in a constructive way?
- Find and perform an activity that relieves your anger or irritation?
- Seek for help from someone else about how to handle the crisis?
- Pretend nothing is wrong and simply proceed?
- Any other pattern? (Describe)

3. Observe in what type of situations you use the different methods, and what are the effects or results or outcome (Do you feel good/bad/indifferent? Does the other person feel good/bad/indifferent?) Don't forget to make notes!

APPENDIX K: CROSS-SECTIONAL SURVEY SCREENING FORM

PART 1: HOUSEHOLD & PARTICIPANT ID

Subcounty ID	Village ID	Compound number	Household number	Cluster number
[____]	[____|____]	[____|____|____|____]	[____|____]	[____|____]

Screening Date	Screening ID
[____|____]/[____|____]/[____|____] day month year	[____|____|____|____]

Age		Gender	
[____|____] / [____|____] years months	If child is less than 1 year, complete months, otherwise leave blank	[___]	1 = Male 2 = Female

PART 2: SCREENING INTERVIEW with PARENTS/GUARDIANS

Selection criteria	Include	Exclude	
1. Appropriate age			
a. Under five (aged 0 to less than 5 years)	1 = Yes	2 = No	[____]
b. Aged 5 to 15 years			
2. Willingness of parent(s)/guardian(s) to provide informed consent	1 = Yes	2 = No	[____]

If any answers are '2' from the EXCLUDE column, exclude from the study. If not, proceed to the next section.

PART 3: AVAILABILITY OF CHILD

	DATE APPROACHED:	Child available to participate in survey?		IF YES, go to Section 4. IF NO, GIVE REASON*	DATE TO RETURN:
1		Yes	No		
2		Yes	No		
3		Yes	No		
4		Yes	No		xxxxxxxxxxxxxxxxx

* 1=Not Home, 2=Come back later, 3=Not interested, 4=Vacant, 77=Other (Please specify above)

PART 4: SCREENING INTERVIEW with CHILD

Selection criteria	Include	Exclude	
3. Able to locate child	1 = Yes	2 = No	[____]
4. Willingness of child over 8 years to provide assent to participate	1 = Yes	2 = No	[____]

If any answers are '2' from the EXCLUDE column, exclude from the study. If not, proceed to the next section.

ASSIGN STUDY NUMBER	[____|____|____|____]

All criteria for study inclusion met?	Date of enrollment (date of survey)
1 = Yes<br>2 = No If no, exclude from the study [____]	[_____|_____]/[_____|_____]/[_____|_____] day month year

[_____]—[_____|_____]—[_____|_____|_____|_____]—[_____|_____]   [_____|_____|_____|_____]

Household ID   CSS Study ID

APPENDIX L. CROSS-SECTIONAL SURVEY
Research participant informed consent form

Protocol Title:	ACT PRIME Study: Evaluating the impact of enhanced health facility-based care for malaria and febrile illnesses in children
Site of Research:	Tororo, Uganda
Principal Investigators:	Dr. Sarah Staedke
Date:	18 September 2010

Introduction

Dr. Sarah Staedke and colleagues from the Uganda Malaria Surveillance Project / Infectious Diseases Research Collaboration are investigating delivery of healthcare services in Tororo District. We are doing a research study to see if we can improve the health of children in this area by improving services at government-run health facilities.

How is this survey being done?

Certain health centers in Tororo district will be selected to either take part in the intervention to improve services, or to continue with their current services. Assignment to the two groups has been determined by a lottery. The chance of being placed into either of the groups is the same. To find out how well the intervention is working, we would like to review the health of children living near the health centers. We plan to carry out three surveys over about 2 years in children from households in this area. We plan to sample 400 children from each area; 200 children under five and 200 children aged 5-15 years. A total of 8000 children will be sampled in each survey.

Households will be selected to participate by a lottery. In each household, one child under five and one child aged 5-15 years will be eligible to take part. If more than one child in the correct age group(s) lives in the house, we will select one child to take part by a lottery. For the survey, we would like to ask the primary caregiver some questions about bednets and treatment of fever in children. We would also like to examine your child and carry out some laboratory tests.

What will happen if my child takes part in this survey?

If you agree to let your child participate in this survey, the following will happen today:

a) We will ask the primary caregiver some questions about him/herself, use of bednets to prevent malaria, and practices for treatment of fever in children.

b) We will collect information on your child's general health.
c) We will briefly examine your child.
d) A blood sample will be taken from your child's finger to examine for malaria parasites, to measure blood counts, and to store on filter paper for future research purposes that will not impact on the health care of your child.
e) If your child has had a fever in the last 48 hours (2 days) or has a high temperature, we will do a rapid diagnostic test for malaria.
f) If your child has a positive test for malaria, we will provide treatment with artemether-lumefantrine (including Coartem or Lumatem), which is the recommended treatment for simple malaria in Uganda.
g) If your child has a negative test for malaria, has a low blood count, or has signs of severe malaria or another significant illness, we will refer you and your child to an appropriate health center or hospital for further care.

<u>How long will these activities last?</u>
Today the survey activities, including questions, examination, and blood tests will take about 1 hour.

<u>Can I stop my child from being in the survey?</u>
You can decide to stop participating at any time. Just tell our study personnel right away if you wish to stop the activities.

<u>What risks can I expect if my child participates in the survey?</u>
We will obtain one blood sample by fingerprick from your child. The risks of drawing blood from a fingerprick include temporary discomfort from the needle stick, bruising, and skin infection. The amount of blood removed will be too small to affect your child's health.

Participation in any research study may involve a loss of privacy. Information you provide will be recorded, but your name, and your child's name, will not be used in any reports of the information provided. The information obtained from these survey activities will only be used by the project researchers and will be locked at our project offices. We will do our best to make sure that any personal information is kept private.

<u>Are there benefits to letting my child take part in the survey?</u>
Through the intervention, we aim to improve the health of children in this area by improving services at the health centers. There will be no direct benefit to you from participating in this study. However, the information that we gather in this survey will help researchers and policy-makers understand how best to improve health services in this area.

<u>What other choices do I have if I do not allow my child to take part in the survey?</u>

THE REPUBLIC OF UGANDA

You are free to choose not to participate in the survey. If you decide not to take part, there will be no penalty to you.

What are the costs of taking part in the survey? Will I be paid for letting my child take part in the survey?
You and your child will not be charged for any of the treatments or procedures we perform today. However, if we refer your child for further evaluation and health care, you will be responsible for all costs for your child's health care. You and your child will not be paid for participation in the survey.

What are my rights if I allow my child to take part in the survey?
Taking part in this survey is your choice. You may choose either to take part or not to take part. If you decide to take part in this survey, you may change your mind at any time. If you decide to withdraw your child from the survey; your child will still be eligible for care at the local health facility and at Tororo District Hospital and at other local clinics. No matter what decision you take, there will be no penalty to you in any way.

What if my child is injured as result of being in this survey?
If your child is injured, or if you have questions about injuries as a result of being in the survey, please contact Dr. Sarah Staedke or other members of the Uganda Malaria Surveillance Project / Infectious Disease Research Collaboration on telephone number 0414-530692. The sponsoring organizations do not have a program to cover your costs if your child is hurt or has other bad results.

Who can answer my questions about the survey?
You can talk to the researchers about any questions or concerns you have about these survey activities. Contact Dr. Sarah Staedke or other members of the Uganda Malaria Surveillance Project / Infectious Disease Research Collaboration on telephone number 0414-530692. If you have any questions, comments or concerns about taking part in these activities, first talk to the researchers. If for any reason you do not wish to do this, or you still have concerns about doing so, you may contact Dr Charles Ibingira, Makerere University Faculty of Medicine Research and Ethical Committee at telephone number 0414-530020.

WHAT YOUR SIGNATURE OR THUMBPRINT MEANS

Your signature or thumbprint below means that you understand the information given to you in this consent form about your child's participation in the survey and agree with the following statements:

1. "I have read the consent form concerning this survey (or have understood the verbal explanation of the consent form) and I understand what will be required of me and what will happen to me and my child if we take part in it."

2. "My questions concerning this survey have been answered by Dr. Staedke or the person who signed below."

3. "I understand that at any time, I may withdraw my child from this survey without giving a reason and without affecting my child's normal health care and management."

4. "I agree that the child under my care will take part in this survey."

You will also be asked to sign another informed consent form for the use of stored specimens. If you wish your child to participate in this survey, you should sign or place your thumbprint below.

THE REPUBLIC OF UGANDA

WE WILL GIVE YOU A COPY OF THIS SIGNED AND DATED CONSENT FORM

____

Name of Participant (printed)

____

Name of Parent/Guardian

____

Signature or Fingerprint * of Parent/Guardian Date/Time

____

Name of Investigator Administering Consent (printed) Position/Title

____

Signature of Investigator Administering Consent Date/Time

*If the parent or guardian is unable to read and/or write, an impartial witness should be present during the informed consent discussion. After the written informed consent form is read and explained to the participant and parent or guardian, and after they have orally consented to their child's participation in the trial, and have either signed the consent form or provided their fingerprint, the witness should sign and personally date the consent form. By signing the consent form, the witness attests that the information in the consent form and any other written information was accurately explained to, and apparently understood by the parent or guardian, and that informed consent was freely given by the patient and parent or guardian.

____

Name of Person Witnessing Consent (printed)

____

Signature of Person Witnessing Consent Date/Time

[____]—[____|____]—[____|____|____|____]—[____|____]   [____|____|____|____]

Household ID   CSS Study ID

APPENDIX M. CROSS-SECTIONAL SURVEY
Informed consent for future use of biological specimens

Protocol Title:	ACT PRIME Study: Evaluating the impact of enhanced health facility-based care for malaria and febrile illnesses in children
Site of Research:	Tororo, Uganda
Principal Investigators:	Dr. Sarah Staedke
Date:	18 September 2010

INTRODUCTION

While your child is in this study, there may be blood samples taken from them that may be useful for future research. These samples will be stored long-term at Makerere University Medical School and the London School of Hygiene and Tropical Medicine, and the University of California, San Francisco. Samples may also be shared with investigators at other institutions.

WHAT SAMPLES WILL BE USED FOR

Your child's blood and the malaria parasites in it will be used to study malaria and the response of this disease to treatment. Results of these studies will not affect your child's care.

1. These samples will be used for future research to learn more about malaria and other diseases.
2. Your child's samples will be used only for research and will not be sold or used for the production of commercial products.
3. Genetic research may be performed on samples. However, no genetic information obtained from this research will be placed in your child's medical records. These samples will be identified only by codes so that they cannot be readily identified with your child.

LEVEL OF IDENTIFICATION

Your child's samples will be coded so that your child's name cannot be readily identified. Reports about research done with your child's samples will not be put in their medical record and will be kept confidential to the best of our ability. In the future, researchers studying your child's samples may need to know more about your child, such as their age, gender, and race. If this information is already available because of your child's participation in a study, it may be provided to the researcher. Your child's name or anything that might identify them personally will not be provided. You will not be asked to provide additional consent.

RISKS

There are few risks to your child from future use of their samples. A potential risk might be the release of information from your child's health or study records. Reports about research done with your child's samples will not be put in their health record, but will be kept with the study records. The study records will be kept confidential as far as possible.

BENEFITS

There will be no direct benefit to your child. From studying your child's samples we may learn more about malaria or other diseases: how to prevent them, how to treat them, how to cure them.

RESEARCH RESULTS/MEDICAL RECORDS

1. Results from future research using your child's samples may be presented in publications and meetings but patient names will not be identified.
2. Reports from future research done with your child's samples will not be given to you or your child's doctor. These reports will not be put in your child's medical record.

QUESTIONS

If you have any questions, comments or concerns about the future use of your child's specimen's, first talk to the researchers. You may also Contact Dr. Sarah Staedke or other members of the Uganda Malaria Surveillance Project on telephone number 0414-530692. If for any reason you do not wish to do this, or you still have concerns about the future use of your child's specimens, you may contact Dr Charles Ibingira, Makerere University Faculty of Medicine Research and Ethical Committee at telephone number 0414-530020.

FREEDOM TO REFUSE

You can change your mind at any time about allowing your child's samples to be used for future research. If you do, contact Dr. Staedke or other members of the Uganda Malaria Surveillance Project at the numbers listed above. Then your child's samples will no longer be made available for research and will be destroyed. Whether or not you allow us to use your child's samples in future research will not have any effect on your child's participation in this study or future participation in other studies.

WHAT YOUR SIGNATURE OR THUMBPRINT MEANS

Your signature or thumbprint below means that you understand the information given to you in this consent form about your child's specimens to be used for future research. If you wish to allow your child's specimens to be used for future research, you should sign or place your thumbprint below.

WE WILL GIVE YOU A COPY OF THIS SIGNED AND DATED CONSENT FORM

Name of Participant (printed)

Name of Parent/Guardian

Signature or Fingerprint * of Parent/Guardian   Date/Time

Name of Investigator Administering Consent (printed)   Position/Title

Signature of Investigator Administering Consent   Date/Time

*If the parent or guardian is unable to read and/or write, an impartial witness should be present during the informed consent discussion. After the written informed consent form is read and explained to the participant and parent or guardian, and after they have orally consented to their child's participation in the trial, and have either signed the consent form or provided their fingerprint, the witness should sign and personally date the consent form. By signing the consent form, the witness attests that the information in the consent form and any other written information was accurately explained to, and apparently understood by the parent or guardian, and that informed consent was freely given by the patient and parent or guardian.

Name of Person Witnessing Consent (printed)

Signature of Person Witnessing Consent   Date/Time

THE REPUBLIC OF UGANDA

[_____]—[_____|_____]—[_____|_____|_____|_____]—[_____|_____]

Household ID

[_____|_____|_____|_____]

CSS Study ID

APPENDIX N. CROSS-SECTIONAL SURVEY
Research participant assent form for children

Protocol Title:	ACT PRIME Study: Evaluating the impact of enhanced health facility-based care for malaria and febrile illnesses in children
Site of Research:	Tororo, Uganda
Principal Investigators:	Dr. Sarah Staedke
Date:	18 September 2010

— I am being asked to decide if I want to be in this research study.
— I know that I will have to see the survey field workers today.
— The field workers will talk to me, ask me questions, and examine me.
— I know I will have a few drops of blood drawn from my finger today.
— I asked and got answers to my questions. I know that I can ask questions about this survey at any time.
— I know that I can stop being in this survey at anytime without anyone being mad at me.

Mark one box with X:

I DO CONSENT: ☐ **I hereby agree to take part in this survey**

I DO NOT CONSENT: ☐ **I do not wish to take part in this survey**

Name of child:	
Signature or fingerprint of child:	Date:

Witness: I hereby confirm that the study has been explained to the child. All questions (if any) have also been answered to his/her satisfaction, and he/she has, of his own free will, consented to take part in the survey.

Name of witness:	
Signature of witness:	Date:

Name of person explaining study:	
Signature:	Date:

APPENDIX O: CROSS-SECTIONAL SURVEY QUESTIONNAIRE (1)

Subcounty ID	Village ID	Compound Number	Household Number	Date of visit
[___]	[___|___]	[___|___|___|___]	[___|___]	[___|___]/[___|___]/[___|___] day month year

SECTION 1: DATES VISITING THIS HOUSEHOLD

	DATE APPROACHED [dd/mm/yy]	Primary caregiver able to participate in survey? 1 = Yes 2 = No	IF YES, go to Section 4. IF NO, give reason* (see below)	DATE TO RETURN [dd/mm/yy]
1	[___ ___/___ ___/___ ___]	[___]	[___] ____________	[___ ___/___ ___/___ ___]
2	[___ ___/___ ___/___ ___]	[___]	[___] ____________	[___ ___/___ ___/___ ___]
3	[___ ___/___ ___/___ ___]	[___]	[___] ____________	[___ ___/___ ___/___ ___]
4	[___ ___/___ ___/___ ___]	[___]	[___] ____________	[___ ___/___ ___/___ ___]

* 1 = Not Home 2 = Come back later 3 = Not interested 4 = Vacant 77 = Other (Please specify above)

SECTION 2: PRIMARY CAREGIVER

1. "How old are you?" [____|____]years

2. Gender 1 = Male 2 = Female [____]

3. What is the highest level of school you completed?
0 = None; 1 = Primary (P1 — P4); 2 = Primary (P5 — P7); 3 = Secondary (S1 — S4); 4 = Secondary (S5 — S6); 10 = Certificate/Diploma; 11 = University; 77 = Other; 88 = Don't know; 99 = Refused to answer [____|____]

4. "What is the main activity or job you do to earn income" OR "If you do not have regular employment, what other things do you do to earn income?"
1 = Peasant farmer; 2 = Commercial farmer; 3 = Brew alcohol; 4 = Market vendor; 5 = Shop keeper; 6 = Transport(Driver/rider); 77 = Other; 88 = Don't know; 99 = Refused to answer [____|____]

SECTION 3: BEDNETS

5. "Does your household have any mosquito nets? 1 = Yes 2 = No 88 = Don't know 99 = Refused to answer [____|____]
If no, go to Section 5: Treatment seeking

6. "How many mosquito nets does your household have?" List number [____|____]

Net 1	Net 2 (if more than 2 nets, go to extra bednet form)
7a. "How many months ago did you obtain the mosquito net?" 88 = Don't know 99 = Refused to answer Insert number of months [____|____]	7b. "How many months ago did you obtain the mosquito net?" 88 = Don't know 99 = Refused to answer Insert number of months [____|____]
8a. "From where did you get the mosquito nets? [____|____] 1 = Government health center 2 = Government hospital 3 = Private hospital/clinic 4 = Private pharmacy 5 = Shop 6 = Open market 7 = Hawker 8 = Project/NGO 9 = Campaign 10 = Church 77 = Other ____________ 88 = Don't know 99 = Refused	8b. "From where did you get the mosquito nets? [____|____] 1 = Government health center 2 = Government hospital 3 = Private hospital/clinic 4 = Private pharmacy 5 = Shop 6 = Open market 7 = Hawker 8 = Project/NGO 9 = Campaign 10 = Church 77 = Other ____________ 88 = Don't know 99 = Refused
9a. "May I have a look at the mosquito net?" (Observe the net and record the status) 1 = Observed and intact 2 = Observed and has visible holes 3 = Not a net 4 = Not observed [____]	9b. "May I have a look at the mosquito net?" (Observe the net and record the status) 1 = Observed and intact 2 = Observed and has visible holes 3 = Not a net 4 = Not observed [____]
10a. "What is the brand of the mosquito net?" (If net was observed, record brand; if not, ask the respondent) If 1, go to Section 5 1 = Long lasting net (Permanet, Smartnet, Olyset) 2 = Factory net with insecticide kit (KO, Kooper, Ico, Safi) 3 = Factory net with no insecticide (B52, Bamboo, Century, Lucky, Victoria) 4 = Home made net 77 = Other________ [____]	10b. "What is the brand of the mosquito net?" (If net was observed, record brand; if not, ask the respondent) If 1, go to Section 5 1 = Long lasting net (Permanet, Smartnet, Olyset) 2 = Factory net with insecticide kit (KO, Kooper, Ico, Safi) 3 = Factory net with no insecticide (B52, Bamboo, Century, Lucky, Victoria) 4 = Home made net 77 = Other________ [____]
11a. How many months ago was the mosquito net last soaked or dipped in insecticide to repel mosquitos? 1 = < 6 mo 2 = ≥ 6 mo 3 = Never 88 = Don't know 99 = Refused to answer [____|____]	11b. How many months ago was the mosquito net last soaked or dipped in insecticide to repel mosquitos? 1 = < 6 mo 2 = ≥ 6 mo 3 = Never 88 = Don't know 99 = Refused to answer [____|____]

CROSS-SECTIONAL SURVEY QUESTIONNAIRE (2)

Subcounty ID	Village ID	Compound Number	Household Number	Date of visit
[___]	[___|___]	[___|___|___|___]	[___|___]	[___|___]/[___|___]/[___|___] day month year

SECTION 4: EXPERIENCE WITH ILLNESS DURING THE PAST TWO WEEKS

14. "Did any of the children under your care have fever in the past 2 weeks prior to this survey?"
1 = Yes
2 = No
88 = Don't know
99 = Refused to answer
[____|____]
If NO, stop here.

SECTION 4: PART 1: FIRST ACTION

"Now we would like to get a detailed step by step description of everything you did to care for your child who fell sick the most recently. There is no right or wrong answer to these questions. We want you to be as open and honest as possible."

15. "What did you do FIRST (including tepid sponging and herbs)?" (choose only one action)
1 = Nothing
2 = Tepid sponging
3 = Gave herbs kept at home
4 = Gave medicines kept at home
5 = Bought medicines from duka
6 = Bought medicines at drug shop/pharmacy
7 = Took to traditional healer
8 = Took to clinic or hospital
77 = Other _______________
88 = Don't know
99 = Refused to answer
[____|____]
If clinic or hospital, go to #16, otherwise skip to #17

16. "If you took your child to clinic or hospital, where did you go?"
1 = Tororo District Hospital
2 = Private hospital/clinic
77 = Other ________________
88 = Don't know
99 = Refused to answer
[____|____]

17. "How long had the child been ill when this FIRST action was taken?"
1 = < 24 hrs
2 = 1-3 days
3 = 4-7 days
4 = > 7 days
77 = Other _____________
88 = Don't know
99 = Refused to answer
[____|____]

MEDICINES GIVEN AS FIRST TREATMENT

"If your child took medicine FIRST, what did he/she take?" (Indicate all that were given as a first action)

DRUG	Number of times given per day	Number of days given	How long had the child been ill when the drug was started?<br>1 = < 24 hrs<br>2 = 1-3 days<br>3 = 4-7 days<br>4 = > 7 days<br>77 = Other (list)<br>88 = Don't know<br>99 = Refused to answer
18. Panadol	[____|____]	[____|____]	[____|____] ________________________
19. Aspirin	[____|____]	[____|____]	[____|____] ________________________
20. Chloroquine	[____|____]	[____|____]	[____|____] ________________________
21. Fansidar (SP)	[____|____]	[____|____]	[____|____] ________________________
22. CQ+SP	[____|____]	[____|____]	[____|____] ________________________
23. Amodiaquine	[____|____]	[____|____]	[____|____] ________________________
24. Quinine	[____|____]	[____|____]	[____|____] ________________________
25. Coartem	[____|____]	[____|____]	[____|____] ________________________
26. Septrin (Bactrim)	[____|____]	[____|____]	[____|____] ________________________
27. Amoxacillin	[____|____]	[____|____]	[____|____] ________________________
28. Other ________________	[____|____]	[____|____]	[____|____] ________________________
29. Other ________________	[____|____]	[____|____]	[____|____] ________________________
30. Other ________________	[____|____]	[____|____]	[____|____] ________________________
31. Unknown	[____|____]	[____|____]	[____|____] ________________________

IF ILLNESS RESOLVED, skip to Section 5 on treatment outcome

CROSS-SECTIONAL SURVEY QUESTIONNAIRE (3)

Subcounty ID	Village ID	Compound Number	Household Number	Date of visit
[___]	[___|___]	[___|___|___|___]	[___|___]	[___|___]/[___|___]/[___|___] day month year

SECTION 4: PART 2: SECOND ACTION

32. "What did you do SECOND (including tepid sponging and herbs)?" (choose only one action)

1 = Nothing
2 = Tepid sponging
3 = Gave herbs kept at home
4 = Gave medicines kept at home
5 = Bought medicines from duka
6 = Bought medicines at drug shop/pharmacy
7 = Took to traditional healer
8 = Took to clinic or hospital
77 = Other ______________
88 = Don't know
99 = Refused to answer

[____|____]

If clinic or hospital, go to #31, otherwise skip to #32

33. "If you took your child to clinic or hospital, where did you go?"

1 = Tororo District Hospital
2 = Private hospital/clinic
77 = Other _______________
88 = Don't know
99 = Refused to answer

[____|____]

34. "How long had the child been ill when this SECOND action was taken?"

1 = < 24 hrs
2 = 1-3 days
3 = 4-7 days
4 = > 7 days
77 = Other ___________
88 = Don't know
99 = Refused to answer

[____|____]

MEDICINES GIVEN AS SECOND TREATMENT

"If your child took medicine SECOND, what did he/she take?" (Indicate all that were given as a second action)

DRUG	Number of times given per day	Number of days given	How long had the child been ill when the drug was started? 1 = < 24 hrs; 2 = 1-3 days; 3 = 4-7 days; 4 = > 7 days; 77 = Other (list); 88 = Don't know; 99 = Refused to answer
35. Panadol	[____|____]	[____|____]	[____|____] ______________________
36. Aspirin	[____|____]	[____|____]	[____|____] ______________________
37. Chloroquine	[____|____]	[____|____]	[____|____] ______________________
38. Fansidar (SP)	[____|____]	[____|____]	[____|____] ______________________
39. CQ+SP	[____|____]	[____|____]	[____|____] ______________________
40. Amodiaquine	[____|____]	[____|____]	[____|____] ______________________
41. Quinine	[____|____]	[____|____]	[____|____] ______________________
42. Coartem	[____|____]	[____|____]	[____|____] ______________________
43. Septrin (Bactrim)	[____|____]	[____|____]	[____|____] ______________________
44. Amoxacillin	[____|____]	[____|____]	[____|____] ______________________
45. Other ________________	[____|____]	[____|____]	[____|____] ______________________
46. Other ________________	[____|____]	[____|____]	[____|____] ______________________
47. Other ________________	[____|____]	[____|____]	[____|____] ______________________
48. Other ________________	[____|____]	[____|____]	[____|____] ______________________
49. Unknown	[____|____]	[____|____]	[____|____] ______________________

IF ILLNESS RESOLVED, skip to Section 5 on treatment outcome

CROSS-SECTIONAL SURVEY QUESTIONNAIRE (4)				
Subcounty ID	Village ID	Compound Number	Household Number	Date of visit
[___]	[___|___]	[___|___|___|___]	[___|___]	[___|___]/[___|___]/[___|___] day month year

SECTION 4: PART 3: THIRD ACTION

50. "What did you do THIRD (including tepid sponging and herbs)?" (choose only one action)

1 = Nothing
2 = Tepid sponging
3 = Gave herbs kept at home
4 = Gave medicines kept at home
5 = Bought medicines from duka
6 = Bought medicines at drug shop/pharmacy
7 = Took to traditional healer
8 = Took to clinic or hospital
77 = Other _______________
88 = Don't know
99 = Refused to answer

[____|____]

If clinic or hospital, go to #46, otherwise skip to #47

33. "If you took your child to clinic or hospital, where did you go?"

1 = Tororo District Hospital
2 = Private hospital/clinic
77 = Other ________________
88 = Don't know
99 = Refused to answer

[____|____]

34. "How long had the child been ill when this THIRD action was taken?"

1 = < 24 hrs
2 = 1-3 days
3 = 4-7 days
4 = > 7 days
77 = Other ____________
88 = Don't know
99 = Refused to answer

[____|____]

MEDICINES GIVEN AS THIRD TREATMENT

"If your child took medicine THIRD, what did he/she take?" (Indicate all that were given as a third action)

DRUG	Number of times given per day	Number of days given	How long had the child been ill when the drug was started? 1 = < 24 hrs; 2 = 1-3 days; 3 = 4-7 days; 4 = > 7 days; 77 = Other (list); 88 = Don't know; 99 = Refused to answer
53. Panadol	[____|____]	[____|____]	[____|____] ________________________
54. Aspirin	[____|____]	[____|____]	[____|____] ________________________
55. Chloroquine	[____|____]	[____|____]	[____|____] ________________________
56. Fansidar (SP)	[____|____]	[____|____]	[____|____] ________________________
57. CQ+SP	[____|____]	[____|____]	[____|____] ________________________
58. Amodiaquine	[____|____]	[____|____]	[____|____] ________________________
59. Quinine	[____|____]	[____|____]	[____|____] ________________________
60. Coartem	[____|____]	[____|____]	[____|____] ________________________
61. Septrin (Bactrim)	[____|____]	[____|____]	[____|____] ________________________
62. Amoxacillin	[____|____]	[____|____]	[____|____] ________________________
63. Other ____________________	[____|____]	[____|____]	[____|____] ________________________
64. Other ____________________	[____|____]	[____|____]	[____|____] ________________________
65. Other ____________________	[____|____]	[____|____]	[____|____] ________________________
66. Other ____________________	[____|____]	[____|____]	[____|____] ________________________
67. Unknown	[____|____]	[____|____]	[____|____] ________________________

CROSS-SECTIONAL SURVEY QUESTIONNAIRE (5)				
Subcounty ID	Village ID	Compound Number	Household Number	Date of visit
[___]	[___|___]	[___|___|___|___]	[___|___]	[___|___]/[___|___]/[___|___]<br>day month year

SECTION 5: TREATMENT OUTCOME

68. "How long did the child's illness last?"	1 = < 24 hrs<br>2 = 1-3 days<br>3 = 4-7 days<br>4 = >7 days	5 = Ongoing at time of interview<br>77 = Other________________________<br>88 = Don't know<br>99 = Refused to answer	[____|____]
69. "Did you experience any delays in treating your child's illness?"	1 = Yes<br>2 = No	88 = Don't know<br>99 = Refused to answer	[____|____]<br>If no, skip to question #67
70. "What were the reasons for the delays?" (list all that apply)	1 = No transport available<br>2 = Not enough money available<br>3 = Needed to find coverage for work	4 = Needed to arrange for child care<br>5 = Waiting at the health facility<br>88 = Don't know<br>99 = Refused to answer	[____|____] [____|____]<br>[____|____] [____|____]<br>[____|____] [____|____]
71. "How much did you spend on management of this illness?"	Cost of drugs<br>[____|____|____|____|____]Ush<br>Fees (clinic, hospital, lab)<br>[____|____|____|____|____]Ush		Transport<br>[____|____|____|____|____]Ush<br>Other<br>[____|____|____|____|____]Ush<br>TOTAL<br>[____|____|____|____|____]Ush
72. "Did caring for your child and managing his/her illness prevent you from doing your usual activities this month?"	1 = Yes<br>2 = No	88 = Don't know<br>99 = Refused to answer	[____|____]
73. "If yes, how much time did you miss?"	If < 1 day, indicate number of hours otherwise, record 00		[____|____] hours<br>[____|____] days

Field worker initials	[____|____]	Initials of observers present at interview	[____|____] [____|____] [____|____]

APPENDIX P: CROSS-SECTIONAL SURVEY CLINICAL RECORD FORM (1)

Subcounty ID	Village ID	Compound Number	Household Number	Date of visit
[___]	[___|___]	[___|___|___|___]	[___|___]	[___|___]/[___|___]/[___|___]<br>day month year

Child's initials	Study ID	Gender		Age
[_____|_____]	[_____|_____|_____|_____]	[_____]	1 = Male<br>2 = Female	Age: [____|____]/[____|___]<br>years months

SECTION 1: BEDNETS

1. "Does this child have (sleep under) a mosquito net?	1 = Yes<br>2 = No	88 = Don't know<br>99 = Refused to answer<br>If no, go to Section 2: Case Record Form	[____|____]
2. "If yes, did the child sleep under the mosquito net last night?"	1 = Yes<br>2 = No	88 = Don't know<br>99 = Refused to answer	[____|____]
3. "How many months ago did you obtain the mosquito net for the child?"	88 = Don't know<br>99 = Refused to answer	Insert the number of months	[____|____]
4. "From where did you get the mosquito net?	1 = Government health center<br>2 = Government hospital<br>3 = Private hospital/clinic<br>4 = Private pharmacy<br>5 = Shop<br>6 = Open market<br>7 = Hawker<br>8 = Project/NGO<br>9 = Campaign	10 = Church<br>77 = Other____________<br>88 = Don't know<br>99 = Refused to answer	[____|____]
5. "May I have a look at the mosquito net?"<br>(Observe the net and record the status)	1 = Observed and intact<br>2 = Observed and has visible	3 = Not a net<br>4 = Not observed<br>If observed go to #6, if not observed skip to #7	[____|____]
6. "What is the brand of the mosquito net?"<br>(If net was observed, record brand; if not, ask the respondent)<br>If '1', go to Section 2: Case Record Form	1 = Long lasting net (Permanet, Smartnet, Olyset)<br>2 = Factory net with insecticide kit (KO net, Kooper net, Ico net, Safi net)<br>3 = Factory net with no insecticide (B52, Bamboo, Century, Lucky net, Victoria)<br>4 = Home made net<br>77 = Other____		[____|____]
7. How many months ago was the mosquito net last soaked or dipped with insecticide to repel mosquitos?	1 = < 6 months<br>2 = $\geq$ 6 months<br>3 = Never	88 = Don't know<br>99 = Refused to answer	[____|____]

SECTION 2: CASE RECORD FORM

10. Past medical history (list) _______________________ ____

____

____

11. "Does the child have any drug allergies?"	1 = Yes<br>2 = No	88 = Don't know<br>99 = Refused to answer	[____|____]

12. If yes, to which drugs? (list) ______________________ ____

13. Current medications

1 = Panadol	3 = Chloroquine	Drug 1	[____|____]
2 = Aspirin	4 = Fansidar (SP)	Drug 2	[____|____]
7 = Quinine	6 = Amodiaquine	Drug 3	[____|____]
9 = Septrin	10 = Amoxacillin	Drug 4	[____|____]
5 = CQ+SP	11 = None		
8 = Coartem	12 = Other (list) ________________	Drug 5	[____|____]

14. History of fever in the last 48 hours?	1 = Yes<br>2 = No	88 = Don't know<br>99 = Refused to answer	[____]
15. Temperature (°C), Severity*	[___|____]•[____]		Severity [____]
16. Weight (kg) [____|____]		17. Height (cm)	[____|____|___]
19. MUAC (mm) [____|____|____]		19. Spleen size, Severity*	[____] Severity [____]

CROSS-SECTIONAL SURVEY CLINICAL RECORD FORM (2)

Subcounty ID	Village ID	Compound Number	Household Number	Date of visit
[___]	[___|___]	[___|___|___|___]	[___|___]	[___|___] / [___|___] / [___|___]<br>day month year
Child's initials [_____|_____]			Study ID [_____|_____|_____|_____]	

SECTION 3: FEVER EPISODE

Complete if there is a history of fever or documented temperature (≥ 38.0°C)

20. Clinical Notes – History of present illness

21. Physical examination findings

22. RDT result	1 = Positive<br>2 = Negative	3 = Not performed<br>4 = Test failed	[____]
23. Diagnosis	1 = Uncomplicated malaria<br>2 = Severe malaria<br>3 = Otitis media<br>4 = Pharyngitis<br>5 = Upper respiratory tract infection<br>6 = Pneumonia<br>7 = Gastroenteritis<br>8 = Diarrhea	9 = Dysentery<br>10 = Urinary tract infection<br>11 = Skin infection<br>12 = Viral illness<br>77 = Other<br>88 = Unknown	[_____|_____]
24. Was the child referred for additional care?		1 = Yes<br>2 = No	[____]
25. If yes, where? (specify)			

26. Medications prescribed

1 = Panadol	3 = Chloroquine	Drug 1	[____|____]
2 = Aspirin	4 = Fansidar (SP)	Drug 2	[____|____]
7 = Quinine	6 = Amodiaquine	Drug 3	[____|____]
9 = Septrin	10 = Amoxacillin	Drug 4	[____|____]
5 = CQ+SP	11 = None	Drug 5	[____|____]
8 = Coartem	12 = Other (list) ______________		

Initials: [____|____]

APPENDIX Q: COHORT STUDY SCREENING FORM

PART 1: HOUSEHOLD & PARTICIPANT ID

Subcounty ID	Village ID	Compound number	Household number	Cluster number
[____]	[____|____]	[____|____|____|____]	[____|____]	[____|____]

Screening Date	Screening ID
[____|____]/[____|____]/[____|____]<br>day month year	[____|____|____|____]

Age		Gender	
[____|____] / [____|____]<br>years months	If child is less than 1 year, complete months, otherwise leave blank	[____]	1 = Male<br>2 = Female

PART 2: SCREENING INTERVIEW with PARENTS/GUARDIANS

Selection criteria	Include	Exclude	
1. Appropriate age<br>—Under five (aged 0 to less than 5 years)	1 = Yes	2 = No	[____]
2. Intention to move from Tororo during the follow-up period	1 = No	2 = Yes	[____]

If any answers are '2' from the EXCLUDE column, exclude from the study. If not, proceed to the next section.

PART 3: INFORMED CONSENT

Selection criteria	Include	Exclude	
3. Willingness of parent(s)/guardian(s) to provide informed consent	1 = Yes	2 = No	[____]

If any answers are '2' from the EXCLUDE column, exclude from the study. If not, proceed to the next section.

ASSIGN STUDY NUMBER	[____|____|____|____]

All criteria for study inclusion met?	Date of enrollment
1 = Yes<br>2 = No If no, exclude from the study<br>[____]	[____|____]/[____|____]/[____|____]<br>day month year

[_____]—[_____|_____]—[_____|_____|_____|_____]—[_____|_____]   [_____|_____|_____|_____]

Household ID   Cohort Study ID

APPENDIX R. COHORT STUDY
Research participant informed consent form

Protocol Title:	ACT PRIME Study: Evaluating the impact of enhanced health facility-based care for malaria and febrile illnesses in children
Site of Research:	Tororo, Uganda
Principal Investigators:	Dr. Sarah Staedke
Date:	18 September 2010

Introduction
Dr. Sarah Staedke and colleagues from the Uganda Malaria Surveillance Project / Infectious Diseases Research Collaboration are investigating delivery of healthcare services in Tororo District. We are doing a research study to see if we can improve the health of children in this area by improving services at government-run health facilities.

Why is this study being done?
Certain health centers in Tororo district will be selected to either take part in the intervention to improve services, or to continue with their current services. Assignment to the two groups has been determined by a lottery. The chance of being placed into either of the groups is the same. To find out how well the intervention is working, we would like to review the health of children living near the health centers. We plan to invite a group of children under five from 500 households near the health centers to take part in a 2-year study.

How is this study being done?
Households will be selected to participate by a lottery. In each household, all children under five will be eligible to take part. If a new child is born into your household, we will include them. When children reach the age of five, they will be taken out of the study. A household survey will be conducted at the start of the study to learn more about your household and how you manage illnesses in children. We will examine children and do blood tests five times during the study. We will ask members of your household to record information about the health of your children and how much you spend on their treatment using a diary with pictures. We will visit you once a month to collect the diaries and ask some questions about the health of your children.

What will happen if my child takes part in this study?

If you agree to let your child (or children) participate in this study, the following will happen today:

a) We will collect information on your child's general health and will briefly examine your child.
b) A blood sample will be taken from your child's finger to examine for malaria parasites and to measure blood counts.
c) We will also store a sample of blood on filter paper for future research purposes that will not impact on the health care of your child.
d) If your child has had a fever in the last 48 hours (2 days) or has a high temperature, we will do a rapid diagnostic test for malaria.
e) If your child has a positive test for malaria, we will provide treatment with artemether-lumefantrine (including Coartem or Lumatem), which is the recommended treatment for simple malaria in Uganda.
f) If your child has a negative test for malaria, has a low blood count, or has signs of severe malaria or another significant illness, we will refer you and your child to an appropriate health center or hospital for further care.
g) You will be asked to record information about the health of your child and certain problems using a diary with pictures. You will also be asked to record how much money you spend on your child's health care. We will give you pre-printed forms to help you record this information. You will be given instructions on how to complete the forms and will not be required to read in order to complete the forms. We will ask that you complete the forms for the duration of the study period (2 years). A member of the study staff will visit you at home at least once a month to collect completed diary forms, to give you new forms, and to ask additional questions about the health of your child during the previous month.
h) Either today or within the next two weeks, a member of the study staff will come to your home to ask you additional questions about your home and how members of your household manage malaria and other illnesses. We will also answer any questions you might have about how to fill in the diary forms.

Every six months for the next 2 years, we will schedule appointments for you and your child. At these appointments, the following will happen:

a) We will examine your child.
b) A blood sample will be taken from your child's finger to examine for malaria parasites and to measure blood counts.
c) We will also store a sample of blood on filter paper for future research purposes that will not impact on the health care of your child.
d) If your child has had a fever in the last 48 hours (2 days) or has a high temperature, we will do a rapid diagnostic test for malaria.

e) If your child has a positive test for malaria, we will provide treatment with artemether-lumefantrine (including Coartem or Lumatem), which is the recommended treatment for simple malaria in Uganda.
f) If your child has a negative test for malaria, has a low blood count, or has signs of severe malaria or another significant illness, we will refer you and your child to an appropriate health center or hospital for further care.

<u>How long will these activities last?</u>
The study will last for 2 years.

<u>Can I stop my child from being in the study?</u>
You can decide to stop participating at any time. Just tell our study personnel right away if you wish to stop the activities.

<u>What risks can I expect if my child participates in the study?</u>
We will obtain five blood samples by fingerprick from your child. The risks of drawing blood from a fingerprick include temporary discomfort from the needle stick, bruising, and skin infection. The amount of blood removed will be too small to affect your child's health.

Participation in any research study may involve a loss of privacy. Information you provide will be recorded, but your name, and your child's name, will not be used in any reports of the information provided. The information obtained from these study activities will only be used by the project researchers and will be locked up at our project offices. We will do our best to make sure that all personal information is kept private.

<u>Are there benefits to letting my child take part in the study?</u>
Through the intervention, we aim to improve the health of children in this area by improving services at the health centers. There will be no direct benefit to you from participating in this study. However, the information that we gather in this study will help researchers and policy-makers understand how best to improve health services in this area.

<u>What other choices do I have if I do not allow my child to take part in the study?</u>
You are free to choose not to participate in the study. If you decide not to take part, there will be no penalty to you.

What are the costs of taking part in the study? Will I be paid for letting my child take part in the study?
You and your child will not be charged for any of the treatments or procedures we perform today. However, if we refer your child for further evaluation and health care, you will be responsible for all costs for your child's health care. You and your child will not be paid for participation in the study.

What are my rights if I allow my child to take part in the study?
Taking part in this study is your choice. You may choose either to take part or not to take part. If you decide to take part in this study, you may change your mind at any time. If you decide to withdraw your child from the study; your child will still be eligible for care at the local health facility and at Tororo District Hospital and at other local clinics. No matter what decision you take, there will be no penalty to you in any way.

What if my child is injured as result of being in this study?
If your child is injured, or if you have questions about injuries as a result of being in the study, please contact Dr. Sarah Staedke or other members of the Uganda Malaria Surveillance Project / Infectious Disease Research Collaboration on telephone number 0414-530692. The sponsoring organizations do not have a program to cover your costs if your child is hurt or has other bad results.

Who can answer my questions about the study?
You can talk to the researchers about any questions or concerns you have about these study activities. Contact Dr. Sarah Staedke or other members of the Uganda Malaria Surveillance Project / Infectious Disease Research Collaboration on telephone number 0414-530692. If you have any questions, comments or concerns about taking part in these activities, first talk to the researchers. If for any reason you do not wish to do this, or you still have concerns about doing so, you may contact Dr Charles Ibingira, Makerere University Faculty of Medicine Research and Ethical Committee at telephone number 0414-530020.

WHAT YOUR SIGNATURE OR THUMBPRINT MEANS

Your signature or thumbprint below means that you understand the information given to you in this consent form about your child's participation in the study and agree with the following statements:

1. "I have read the consent form concerning this survey (or have understood the verbal explanation of the consent form) and I understand what will be required of me and what will happen to me and my child if we take part in it."

2. "My questions concerning this survey have been answered by Dr. Staedke or the person who signed below."

3. "I understand that at any time, I may withdraw my child from this survey without giving a reason and without affecting my child's normal health care and management."

4. "I agree that the child under my care will take part in this survey."

You will also be asked to sign another informed consent form for the use of stored specimens. If you wish your child to participate in this study, you should sign or place your thumbprint below.

THE REPUBLIC OF UGANDA

UCSF

WE WILL GIVE YOU A COPY OF THIS SIGNED AND DATED CONSENT FORM

Name of Participant (printed)

Name of Parent/Guardian

Signature or Fingerprint * of Parent/Guardian	Date/Time

Name of Investigator Administering Consent (printed)	Position/Title

Signature of Investigator Administering Consent	Date/Time

*If the parent or guardian is unable to read and/or write, an impartial witness should be present during the informed consent discussion. After the written informed consent form is read and explained to the participant and parent or guardian, and after they have orally consented to their child's participation in the trial, and have either signed the consent form or provided their fingerprint, the witness should sign and personally date the consent form. By signing the consent form, the witness attests that the information in the consent form and any other written information was accurately explained to, and apparently understood by the parent or guardian, and that informed consent was freely given by the patient and parent or guardian.

Name of Person Witnessing Consent (printed)

Signature of Person Witnessing Consent	Date/Time

THE REPUBLIC OF UGANDA

[____]—[____|____]—[____|____|____|____]—[____|____] [____|____|____|____]

Household ID Cohort Study ID

APPENDIX S. COHORT STUDY
Informed consent for future use of biological specimes

Protocol Title:	ACT PRIME Study: Evaluating the impact of enhanced health facility-based care for malaria and febrile illnesses in children
Site of Research:	Tororo, Uganda
Principal Investigators:	Dr. Sarah Staedke
Date:	18 September 2010

INTRODUCTION
While your child is in this study, there may be blood samples taken from them that may be useful for future research. These samples will be stored long-term at Makerere University Medical School and the London School of Hygiene and Tropical Medicine, and the University of California, San Francisco. Samples may also be shared with investigators at other institutions.

WHAT SAMPLES WILL BE USED FOR
Your child's blood and the malaria parasites in it will be used to study malaria and the response of this disease to treatment. Results of these studies will not affect your child's care.

1. These samples will be used for future research to learn more about malaria and other diseases.
2. Your child's samples will be used only for research and will not be sold or used for the production of commercial products.
3. Genetic research may be performed on samples. However, no genetic information obtained from this research will be placed in your child's medical records. These samples will be identified only by codes so that they cannot be readily identified with your child.

LEVEL OF IDENTIFICATION
Your child's samples will be coded so that your child's name cannot be readily identified. Reports about research done with your child's samples will not be put in their medical record and will be kept confidential to the best of our ability. In the future, researchers studying your child's samples may need to know more about your child, such as their age, gender, and race. If this information is already available because of your child's participation in a study, it may be provided to the researcher. Your child's name or anything that might identify them personally will not be provided. You will not be asked to provide additional consent.

RISKS

There are few risks to your child from future use of their samples. A potential risk might be the release of information from your child's health or study records. Reports about research done with your child's samples will not be put in their health record, but will be kept with the study records. The study records will be kept confidential as far as possible.

BENEFITS

There will be no direct benefit to your child. From studying your child's samples we may learn more about malaria or other diseases: how to prevent them, how to treat them, how to cure them.

RESEARCH RESULTS/MEDICAL RECORDS

1. Results from future research using your child's samples may be presented in publications and meetings but patient names will not be identified.
2. Reports from future research done with your child's samples will not be given to you or your child's doctor. These reports will not be put in your child's medical record.

QUESTIONS

If you have any questions, comments or concerns about the future use of your child's specimen's, first talk to the researchers. You may also Contact Dr. Sarah Staedke or other members of the Uganda Malaria Surveillance Project on telephone number 0414-530692. If for any reason you do not wish to do this, or you still have concerns about the future use of your child's specimens, you may contact Dr Charles Ibingira, Makerere University Faculty of Medicine Research and Ethical Committee at telephone number 0414-530020.

FREEDOM TO REFUSE

You can change your mind at any time about allowing your child's samples to be used for future research. If you do, contact Dr. Staedke or other members of the Uganda Malaria Surveillance Project at the numbers listed above. Then your child's samples will no longer be made available for research and will be destroyed. Whether or not you allow us to use your child's samples in future research will not have any effect on your child's participation in this study or future participation in other studies.

WHAT YOUR SIGNATURE OR THUMBPRINT MEANS

Your signature or thumbprint below means that you understand the information given to you in this consent form about your child's specimens to be used for future research. If you wish to allow your child's specimens to be used for future research, you should sign or place your thumbprint below.

WE WILL GIVE YOU A COPY OF THIS SIGNED AND DATED CONSENT FORM

Name of Participant (printed)

Name of Parent/Guardian

Signature or Fingerprint * of Parent/Guardian — Date/Time

Name of Investigator Administering Consent (printed) — Position/Title

Signature of Investigator Administering Consent — Date/Time

*If the parent or guardian is unable to read and/or write, an impartial witness should be present during the informed consent discussion. After the written informed consent form is read and explained to the participant and parent or guardian, and after they have orally consented to their child's participation in the trial, and have either signed the consent form or provided their fingerprint, the witness should sign and personally date the consent form. By signing the consent form, the witness attests that the information in the consent form and any other written information was accurately explained to, and apparently understood by the parent or guardian, and that informed consent was freely given by the patient and parent or guardian.

Name of Person Witnessing Consent (printed)

Signature of Person Witnessing Consent — Date/Time

APPENDIX T: COHORT STUDY CASE RECORD FORM (1)

Subcounty ID	Village ID	Compound #	Household #	Date ENROLLED: [____|____]/ [____|____]/ [____|____] (day / month / year)
[____|____]	[____|____]	[____|____|____|____]	[____|____]	
Study participant's initials [____|____]	Study ID [____|____|____|____]	Gender [____]	1 = Male<br>2 = Female	

SECTION 1: BEDNETS

1. "Does this child have (sleep under) a mosquito net?	1 = Yes<br>2 = No	88 = Don't know<br>99 = Refused to answer	[____|____]<br>If no, go to Section 2: Case Record Form
2. "If yes, did the child sleep under the mosquito net last night?"	1 = Yes<br>2 = No	88 = Don't know<br>99 = Refused to answer	[____|____]
3. "How many months ago did you obtain the mosquito net for the child?"	88 = Don't know<br>99 = Refused to answer	Insert the number of months	[____|____]
4. "From where did you get the mosquito net?	1 = Government health center<br>2 = Government hospital<br>3 = Private hospital/clinic<br>4 = Private pharmacy<br>5 = Shop<br>6 = Open market<br>7 = Hawker<br>8 = Project/NGO<br>9 = Campaign	10 = Church<br>77 = Other____________<br>88 = Don't know<br>99 = Refused to answer	[____|____]
5. "May I have a look at the mosquito net?"<br>(Observe the net and record the status)	1 = Observed and intact<br>2 = Observed and has visible	3 = Not a net<br>4 = Not observed	[____|____]
6. "What is the brand of the mosquito net?" (If net was observed, record brand; if not, ask the respondent)<br>If '1', go to Section 2: Case Record Form	1 = Long lasting net (Permanet, Smartnet, Olyset)<br>2 = Factory net with insecticide kit (KO net, Kooper net, Ico net, Safi net)<br>3 = Factory net with no insecticide (B52, Bamboo, Century, Lucky net, Victoria)<br>4 = Home made net<br>77 = Other____		[____|____]
7. How many months ago was the mosquito net last soaked or dipped with insecticide to repel mosquitos?	1 = < 6 months<br>2 = ≥ 6 months<br>3 = Never	88 = Don't know<br>99 = Refused to answer	[____|____]

COHORT STUDY CASE RECORD FORM (2)

Subcounty ID	Village ID	Compound #	Household #	Study ID
[___|___]	[___|___]	[___|___|___|___]	[___|___]	[___|___|___|___]

SECTION 2: CASE RECORD FROM

10. Past medical history (list) ____

11. "Does the child have any drug allergies?" 1 = Yes 2 = No 88 = I don't know 99 = Refused to answer [___|___]

12. If yes, to which drugs? (list) ____________________ ____________________

	ENROLMENT	6 MONTHS	12 MONTHS	18 MONTHS	24 MONTHS
DATE	[__ __/__ __/__ __]	[__ __/__ __/__ __]	[__ __/__ __/__ __]	[__ __/__ __/__ __]	[__ __/__ __/__ __]
13. Current medications<br>1 = Panadol 3 = Chloroquine<br>2 = Aspirin 4 = Fansidar (SP)<br>7 = Quinine 6 = Amodiaquine<br>9 = Septrin 10 = Amoxacillin<br>5 = CQ+SP 11 = None<br>8 = Coartem 12 = Other (list)	Drug 1 [___|___]<br>Drug 2 [___|___]<br>Drug 3 [___|___]<br>Drug 4 [___|___]<br>Drug 5 [___|___]<br>________	Drug 1 [___|___]<br>Drug 2 [___|___]<br>Drug 3 [___|___]<br>Drug 4 [___|___]<br>Drug 5 [___|___]<br>________	Drug 1 [___|___]<br>Drug 2 [___|___]<br>Drug 3 [___|___]<br>Drug 4 [___|___]<br>Drug 5 [___|___]<br>________	Drug 1 [___|___]<br>Drug 2 [___|___]<br>Drug 3 [___|___]<br>Drug 4 [___|___]<br>Drug 5 [___|___]<br>________	Drug 1 [___|___]<br>Drug 2 [___|___]<br>Drug 3 [___|___]<br>Drug 4 [___|___]<br>Drug 5 [___|___]<br>________
14. Fever in the past 48hrs?<br>1 = Yes* 2 = No	[___]	[___]	[___]	[___]	[___]
15. Temperature (°C), Severity†	[___|___].[___] Severity [___]	[___|___].[___] Severity [___]	[___|___].[___] Severity [___]	[___|___].[___] Severity [___]	[___|___].[___] Severity [___]
16. Weight (kg)	[___|___]	[___|___]	[___|___]	[___|___]	[___|___]
17. Height (cm)	[___|___|___]	[___|___|___]	[___|___|___]	[___|___|___]	[___|___|___]
18. MUAC (mm)	[___|___|___]	[___|___|___]	[___|___|___]	[___|___|___]	[___|___|___]
19. Spleen size, Severity†	[___] Severity [___]	[___] Severity [___]	[___] Severity [___]	[___] Severity [___]	[___] Severity [___]

* Fever = If child has fever or history of fever, complete Section 5, Fever Episode on page 4

†Rank severity on scale of 0-5: 0 = Absent 1 = Mild 2 = Moderate 3 = Severe 4 = Life-threatening 5 = Unable to assess

COHORT STUDY CASE RECORD FORM (3)

Subcounty ID	Village ID	Compound #	Household #	Study ID
[___|___]	[___|___]	[___|___|___|___]	[___|___]	[___|___|___|___]

SECTION 3: LABORATORY RESULTS

Rank severity on scale of 0-5: 0 = Absent 1 = Mild 2 = Moderate 3 = Severe 4 = Life-threatening 5 = Unable to assess

	ENROLMENT		6 MONTHS		12 MONTHS		18 MONTHS		24 MONTHS	
		Severity		Severity		Severity		Severity		Severity
25. Hemoglobin (g/dL)**	[___|___].[___]	[___]	[___|___].[___]	[___]	[___|___].[___]	[___]	[___|___].[___]	[___]	[___|___].[___]	[___]
26. Parasite density (/ul)	[__|__|__|__|__]		[__|__|__|__|__]		[__|__|__|__|__]		[__|__|__|__|__]		[__|__|__|__|__]	
27. Gametocytes<br>1 = Yes 2 = No 3 = N/A	[___]		[___]		[___]		[___]		[___]	

Hemoglobin ** = If Hb is < 5.0 g/dL, refer to Tororo District Hospital

SECTION 4: DATA ENTRY

FIRST DATA ENTRY	[__|__]/ [__|__]/[__|__]<br>day month year<br>Initials: [___|___]	[__|__]/ [__|__]/[__|__]<br>day month year<br>Initials: [___|___]	[__|__]/ [__|__]/[__|__]<br>day month year<br>Initials: [___|___]	[__|__]/ [__|__]/[__|__]<br>day month year<br>Initials: [___|___]	[__|__]/ [__|__]/[__|__]<br>day month year<br>Initials: [___|___]
SECOND DATA ENTRY	[__|__]/ [__|__]/[__|__]<br>day month year<br>Initials: [___|___]	[__|__]/ [__|__]/[__|__]<br>day month year<br>Initials: [___|___]	[__|__]/ [__|__]/[__|__]<br>day month year<br>Initials: [___|___]	[__|__]/ [__|__]/[__|__]<br>day month year<br>Initials: [___|___]	[__|__]/ [__|__]/[__|__]<br>day month year<br>Initials: [___|___]

COHORT STUDY CASE RECORD FORM (4)

Subcounty ID	Village ID	Compound #	Household #	Date of VISIT: [___|___]/[___|___]/[___|___] day month year
[___|___]	[___|___]	[___|___|___|___]	[___|___]	
Study ID [___|___|___|___]	Visit type [___]	1 = Enrolment<br>2 = 6 months<br>3 = 12 months	4 = 18 months<br>5 = 24 months	

SECTION 5: FEVER EPISODE

Complete ONLY if there is a history of fever or documented temperature (≥ 38.0°C)

28. Clinical Notes – History of present illness

29. Physical examination findings

30. RDT result		1 = Positive<br>2 = Negative	3 = Not performed<br>4 = Test failed	[___]
31. Diagnosis	1 = Uncomplicated malaria<br>2 = Severe malaria<br>3 = Otitis media<br>4 = Pharyngitis<br>5 = Upper respiratory tract infection	6 = Pneumonia<br>7 = Gastroenteritis<br>8 = Diarrhea<br>9 = Dysentery<br>10 = Urinary tract infection	11 = Skin infection<br>12 = Viral illness<br>77 = Other<br>88 = Unknown	[___|___]
32. Was the child referred for additional care?			1 = Yes<br>2 = No	[___]
33. If yes, where? (specify)				

34. Medications prescribed

1 = Panadol	3 = Chloroquine	Drug 1	[___|___]
2 = Aspirin	4 = Fansidar (SP)	Drug 2	[___|___]
7 = Quinine	6 = Amodiaquine	Drug 3	[___|___]
9 = Septrin	10 = Amoxacillin	Drug 4	[___|___]
5 = CQ+SP	11 = None	Drug 5	[___|___]
8 = Coartem	12 = Other (list) _______________		

Initials: [___\|___]

APPENDIX U: COHORT STUDY HOUSEHOLD SURVEY (1)

Subcounty ID	Village ID	Compound Number	Household Number	Date of visit
[___]	[___|___]	[___|___|___|___]	[___|___]	[___|___]/[___|___]/[___|___] day month year

SECTION 1: PRIMARY CAREGIVER

1. "How old are you?" [____|____]years	2. Gender	1 = Male<br>2 = Female	[____]
3. "What is the highest level of school you completed?"<br>0 = None<br>1 = Primary (P1 — P4)	2 = Primary (P5 — P7)<br>3 = Secondary (S1 — S4)<br>4 = Secondary (S5 — S6)<br>10 = Certificate/Diploma	11 = University<br>77 = Other<br>88 = Don't know<br>99 = Refused to answer	[____|____]
4. "What is the main activity or job you do to earn income" OR "If you do not have regular employment, what other things do you do to earn income?"	1 = Peasant farmer<br>2 = Commercial farmer<br>3 = Brew alcohol<br>4 = Market vendor	5 = Shop keeper<br>6 = Transport(Driver/rider)<br>77 = Other<br>88 = Don't know<br>99 = Refused to answer	[____|____]

SECTION 2: HEAD OF HOUSEHOLD

"Now I would like to ask you some questions about your household."

5. "Is the head of the household male or female?" Skip to SECTION 3 if the primary care giver is also the head of household	1 = Male<br>2 = Female<br>[____]	6. "How old is the head of household?"	[____|____] years
7. "What is the highest level of school completed by the head of household?"	0 = None<br>1 = Primary (P1 — P4)<br>2 = Primary (P5 — P7)<br>3 = Secondary (S1 — S4)<br>4 = Secondary (S5 — S6)	10 = Certificate/Diploma<br>11 = University<br>77 = Other<br>88 = Don't know<br>99 = Refused to answer	[____|____]
8. "What is the main activity or job the head of household does to earn income" OR "If he/she does not have regular employment, what other things does he/she do to earn income?"	1 = Peasant farmer<br>2 = Commercial farmer<br>3 = Brew alcohol<br>4 = Market vendor	5 = Shop keeper<br>6 = Transport(Driver/rider)<br>77 = Other<br>88 = Don't know<br>99 = Refused to answer	[____|____]

SECTION 3: TREATMENT SEEKING BEHAVIOR

Attendance of Public health facilities and satisfaction with health care provided at Public Health facilities

10. "Have you ever taken your child to a nearby public health facility for treatment?"	1 = Yes<br>2 = No	88 = Don't know<br>99 = Refused to answer	[____|____]
11. "If yes, how satisfied were you with the health care that your child received at the public health facility?"	1 = Very dissatisfied<br>2 = Dissatisfied<br>3 = Uncertain	4 = Satisfied<br>5 = Very satisfied<br>99 = Refused to answer	[____|____]
12. "If dissatisfied or very dissatisfied what were the reasons for being dissatisfied?" (List all that apply)<br>1 = Yes<br>2 = No	Long waiting time [____]<br>No trained professionals [____]<br>No drugs were given [____]<br>No lab tests done [____]<br>Rude HCW [____]	Had to pay for care [____]<br>Treatment was unsuccessful [____]<br>Other [____]<br>Refused to answer [____]	
13. "What was the treatment outcome when you took your child to the public health facility for health care?"	1= Recovered quickly<br>2 = Recovered slowly<br>3 = Did not recover, and so I had to take child back	4 = Did not recover, so I sought care elsewhere<br>77 = Other________________<br>99 = Refused to answer	[____|____]

COHORT STUDY HOUSEHOLD SURVEY (2)

Subcounty ID	Village ID	Compound Number	Household Number	Date of visit
[___]	[___|___]	[___|___|___|___]	[___|___]	[___|___]/[___|___]/[___|___] day month year

SECTION 4: BEDNETS

14. "Does your household have any mosquito nets?" If no, go to Section 5: Treatment seeking	1 = Yes 2 = No	88 = Don't know 99 = Refused to answer	[____|____]
15. "How many mosquito nets does your household have?"		List number	[____|____]

Net 1	Net 2 (if more than 2 nets, go to extra bednet form)
16a. "How many months ago did you obtain the mosquito net?" 88 = Don't know 99 = Refused to answer Insert number of months [____|____]	16b. "How many months ago did you obtain the mosquito net?" 88 = Don't know 99 = Refused to answer Insert number of months [____|____]
17a. "From where did you get the mosquito nets? [____|____] 1 = Government health center 2 = Government hospital 3 = Private hospital/clinic 4 = Private pharmacy 5 = Shop 6 = Open market 7 = Hawker 8 = Project/NGO 9 = Campaign 10 = Church 77 = Other ______________ 88 = Don't know 99 = Refused	17b. "From where did you get the mosquito nets? [____|____] 1 = Government health center 2 = Government hospital 3 = Private hospital/clinic 4 = Private pharmacy 5 = Shop 6 = Open market 7 = Hawker 8 = Project/NGO 9 = Campaign 10 = Church 77 = Other ______________ 88 = Don't know 99 = Refused
18a. "May I have a look at the mosquito net?" (Observe the net and record the status) 1 = Observed and intact 2 = Observed and has visible holes 3 = Not a net 4 = Not observed [____]	18b. "May I have a look at the mosquito net?" (Observe the net and record the status) 1 = Observed and intact 2 = Observed and has visible holes 3 = Not a net 4 = Not observed [____]
19a. "What is the brand of the mosquito net?" (If net was observed, record brand; if not, ask the respondent) If 1, go to Section 5 1 = Long lasting net (Permanet, Smartnet, Olyset) 2 = Factory net with insecticide kit (KO, Kooper, Ico, Safi) 3 = Factory net with no insecticide (B52, Bamboo, Century, Lucky, Victoria) 4 = Home made net 77 = Other________ [____]	19b. "What is the brand of the mosquito net?" (If net was observed, record brand; if not, ask the respondent) If 1, go to Section 5 1 = Long lasting net (Permanet, Smartnet, Olyset) 2 = Factory net with insecticide kit (KO, Kooper, Ico, Safi) 3 = Factory net with no insecticide (B52, Bamboo, Century, Lucky, Victoria) 4 = Home made net 77 = Other________ [____]
20a. How many months ago was the mosquito net last soaked or dipped in insecticide to repel mosquitos? 1 = < 6 mo 2 = ≥ 6 mo 3 = Never 88 = Don't know 99 = Refused to answer [____|____]	20b. How many months ago was the mosquito net last soaked or dipped in insecticide to repel mosquitos? 1 = < 6 mo 2 = ≥ 6 mo 3 = Never 88 = Don't know 99 = Refused to answer [____|____]

COHORT STUDY HOUSEHOLD SURVEY (3)

Subcounty ID	Village ID	Compound Number	Household Number	Date of visit
[___]	[___|___]	[___|___|___|___]	[___|___]	[___|___]/[___|___]/[___|___] (day / month / year)

SECTION 5: EXPERIENCE WITH ILLNESS DURING THE PAST TWO WEEKS

"Now I would like to ask you about your experiences with illness in this household and about what you do when your child is sick."

22. ""Have any of the children under your care been sick during the past two weeks?"
1 = Yes
2 = No
88 = Don't know
99 = Refused to answer
[____|____]
If no or unknown, skip to End.

SECTION 6: FIRST ACTION

Now we would like to get a detailed step by step description of everything you did to care for your child during the most recent illness. There is no right or wrong answer to these questions. We need you to be as open and honest as possible."

23."Did your child have fever with this episode of illness?"
1=Yes
2= No
88= Don't know
99= Refused to answer
[____|____]

24. "What did you do FIRST (including tepid sponging and herbs)?" (choose only one action)
1 = Nothing
2 = Tepid sponging
3 = Gave herbs kept at home
4 = Gave medicines kept at home
5 = Bought medicines from duka
6 = Bought medicines at drug shop/pharmacy
7 = Took to traditional healer
8 = Took to clinic or hospital
77 = Other _______________
88 = Don't know
99 = Refused to answer
[____|____]
If clinic or hospital, go to #25,otherwise skip to Qn # 26

16. "If you took your child to clinic or hospital, where did you go?"
1 = Tororo District Hospital
2 = Private hospital/clinic
77 = Other ______________
88 = Don't know
99 = Refused to answer
[____|____]

17. "How long had the child been ill when this FIRST action was taken?"
1 = < 24 hrs
2 = 1-3 days
3 = 4-7 days
4 = > 7 days
77 = Other ___________
88 = Don't know
99 = Refused to answer
[____|____]

MEDICINES GIVEN AS FIRST TREATMENT

"If your child took medicine FIRST, what did he/she take?" (Indicate all that were given as a first action)

DRUG	Number of times given per day	Number of days given	How long had the child been ill when the drug was started? 1 = < 24 hrs; 2 = 1-3 days; 3 = 4-7 days; 4 = > 7 days; 77 = Other (list); 88 = Don't know; 99 = Refused to answer
27. Panadol	[____|____]	[____|____]	[____|____] ____________________
28. Aspirin	[____|____]	[____|____]	[____|____] ____________________
29. Chloroquine	[____|____]	[____|____]	[____|____] ____________________
30. Fansidar (SP)	[____|____]	[____|____]	[____|____] ____________________
31. CQ+SP	[____|____]	[____|____]	[____|____] ____________________
32. Amodiaquine	[____|____]	[____|____]	[____|____] ____________________
33. Quinine	[____|____]	[____|____]	[____|____] ____________________
34. Coartem	[____|____]	[____|____]	[____|____] ____________________
35. Septrin (Bactrim)	[____|____]	[____|____]	[____|____] ____________________
36. Amoxacillin	[____|____]	[____|____]	[____|____] ____________________
37. Other ______________	[____|____]	[____|____]	[____|____] ____________________
38. Unknown	[____|____]	[____|____]	[____|____] ____________________

IF ILLNESS RESOLVED, skip to Part 1: Section 9: Treatment outcome

COHORT STUDY HOUSEHOLD SURVEY (4)

Subcounty ID	Village ID	Compound Number	Household Number	Date of visit
[___]	[___|___]	[___|___|___|___]	[___|___]	[___|___]/[___|___]/[___|___] day month year

SECTION 7: SECOND ACTION

39. "What did you do SECOND (including tepid sponging and herbs)?" (choose only one action)

1 = Nothing
2 = Tepid sponging
3 = Gave herbs kept at home
4 = Gave medicines kept at home
5 = Bought medicines from duka
6 = Bought medicines at drug shop/pharmacy
7 = Took to traditional healer
8 = Took to clinic or hospital
77 = Other ______________
88 = Don't know
99 = Refused to answer

[____|____]

If clinic or hospital, go to Qn.#40, otherwise skip to Qn # 41

40. "If you took your child to clinic or hospital, where did you go?"

1 = Tororo District Hospital
2 = Private hospital/clinic
77 = Other ______________
88 = Don't know
99 = Refused to answer

[____|____]

41. "How long had the child been ill when this SECOND action was taken?"

1 = < 24 hrs
2 = 1-3 days
3 = 4-7 days
4 = > 7 days
77 = Other ___________
88 = Don't know
99 = Refused to answer

[____|____]

MEDICINES GIVEN AS SECOND TREATMENT

"If your child took medicine SECOND, what did he/she take?" (Indicate all that were given as a second action)

DRUG	Number of times given per day	Number of days given	How long had the child been ill when the drug was started? 1 = < 24 hrs; 2 = 1-3 days; 3 = 4-7 days; 4 = > 7 days; 77 = Other (list); 88 = Don't know; 99 = Refused to answer
42. Panadol	[____|____]	[____|____]	[____|____] ____________________
43. Aspirin	[____|____]	[____|____]	[____|____] ____________________
44. Chloroquine	[____|____]	[____|____]	[____|____] ____________________
45. Fansidar (SP)	[____|____]	[____|____]	[____|____] ____________________
46. CQ+SP	[____|____]	[____|____]	[____|____] ____________________
47. Amodiaquine	[____|____]	[____|____]	[____|____] ____________________
48. Quinine	[____|____]	[____|____]	[____|____] ____________________
49. Coartem	[____|____]	[____|____]	[____|____] ____________________
50. Septrin (Bactrim)	[____|____]	[____|____]	[____|____] ____________________
51. Amoxacillin	[____|____]	[____|____]	[____|____] ____________________
52. Other ________________	[____|____]	[____|____]	[____|____] ____________________
53. Unknown	[____|____]	[____|____]	[____|____] ____________________

IF ILLNESS RESOLVED, skip to Part 1: Section 9: Treatment outcome

COHORT STUDY HOUSEHOLD SURVEY (5)

Subcounty ID	Village ID	Compound Number	Household Number	Date of visit
[___]	[___|___]	[___|___|___|___]	[___|___]	[___|___]/ [___|___]/[___|___] day month year

SECTION 8: THIRD ACTION

54. "What did you do THIRD (including tepid sponging and herbs)?" (choose only one action)

1 = Nothing
2 = Tepid sponging
3 = Gave herbs kept at home
4 = Gave medicines kept at home
5 = Bought medicines from duka
6 = Bought medicines at drug shop/pharmacy
7 = Took to traditional healer
8 = Took to clinic or hospital
77 = Other _______________
88 = Don't know
99 = Refused to answer

[____|____]

If clinic or hospital, go to Qn.#55,otherwise skip to Qn.#56

55. "If you took your child to clinic or hospital, where did you go?"

1 = Tororo District Hospital
2 = Private hospital/clinic
77 = Other ________________
88 = Don't know
99 = Refused to answer

[____|____]

56. "How long had the child been ill when this THIRD action was taken?"

1 = < 24 hrs
2 = 1-3 days
3 = 4-7 days
4 = > 7 days
77 = Other ____________
88 = Don't know
99 = Refused to answer

[____|____]

MEDICINES GIVEN AS THIRD TREATMENT

"If your child took medicine THIRD, what did he/she take?" (Indicate all that were given as a third action)

DRUG	Number of times given per day	Number of days given	How long had the child been ill when the drug was started? 1 = < 24 hrs; 2 = 1-3 days; 3 = 4-7 days; 4 = > 7 days; 77 = Other (list); 88 = Don't know; 99 = Refused to answer
57. Panadol	[____|____]	[____|____]	[____|____] ________________________
58. Aspirin	[____|____]	[____|____]	[____|____] ________________________
59. Chloroquine	[____|____]	[____|____]	[____|____] ________________________
60. Fansidar (SP)	[____|____]	[____|____]	[____|____] ________________________
61. CQ+SP	[____|____]	[____|____]	[____|____] ________________________
62. Amodiaquine	[____|____]	[____|____]	[____|____] ________________________
63. Quinine	[____|____]	[____|____]	[____|____] ________________________
64. Coartem	[____|____]	[____|____]	[____|____] ________________________
65. Septrin (Bactrim)	[____|____]	[____|____]	[____|____] ________________________
66. Amoxacillin	[____|____]	[____|____]	[____|____] ________________________
67. Other ____________________	[____|____]	[____|____]	[____|____] ________________________
68. Unknown	[____|____]	[____|____]	[____|____] ________________________

COHORT STUDY HOUSEHOLD SURVEY (6)				
Subcounty ID	Village ID	Compound Number	Household Number	Date of visit
[___]	[___|___]	[___|___|___|___]	[___|___]	[___|___] / [___|___] / [___|___]<br>day month year

SECTION 9: TREATMENT OUTCOME

69. "How long did the child's illness last?"	1 = < 24 hrs<br>2 = 1-3 days<br>3 = 4-7 days<br>4 = >7 days	5 = Ongoing at time of interview<br>77 = Other________________________<br>88 = Don't know<br>99 = Refused to answer	[____|____]
70. "Did you experience any delays in treating your child's illness?"	1 = Yes<br>2 = No	88 = Don't know<br>99 = Refused to answer	[____|____]<br>If no, skip to question #72
71. "What were the reasons for the delays?" (list all that apply)	1 = No transport available<br>2 = Not enough money available<br>3 = Needed to find coverage for work	4 = Needed to arrange for child care<br>5 = Waiting at the health facility<br>88 = Don't know<br>99 = Refused to answer	[____|____] [____|____]<br>[____|____] [____|____]<br>[____|____] [____|____]
72. "How much did you spend on management of this illness?"	Cost of drugs<br>[____|____|____|____|____]Ush<br>Fees (clinic, hospital, lab)<br>[____|____|____|____|____]Ush	Transport<br>[____|____|____|____|____]Ush<br>Other<br>[____|____|____|____|____]Ush<br>TOTAL<br>[____|____|____|____|____]Ush	
73. "Did caring for your child and managing his/her illness prevent you from doing your usual activities this month?"	1 = Yes<br>2 = No	88 = Don't know<br>99 = Refused to answer	[____|____]
74. "If yes, how much time did you miss?"	If < 1 day, indicate number of hours otherwise, record 00		[____|____] hours<br>[____|____] days

Field worker initials [____|____]	Initials of observers present at interview [____|____] [____|____] [____|____]

COHORT STUDY HOUSEHOLD SURVEY (7)				
**PART 2: WOMAN – BIRTH HISTORY**<br>PART 2 to be completed only on the last household survey interview.				
Subcounty ID<br>[___]	Village ID<br>[___|___]	Compound Number<br>[___|___|___|___]	Household Number<br>[___|___]	Date of visit<br>[___|___]/[___|___]/[___|___]<br>day month year

"Now I would like to ask you about all the births you have had during your life."			
75. "How many children have you given birth to in your life?"	[____|____]	0 = None<br>1 = One or more	99 = Refused to answer
If one or more, list total number of children	76. Birthed: [____|____]	77. Alive: [____|____]	78. Died: [____|____]

	Date of birth	Gender<br>1 = Male<br>2 = Female	Is the child alive now?<br>1 = Yes<br>2 = No	If the child has died, how old was the child when s(he) died?<br>(If <1 month at death, enter 0 YEARS 0 MONTHs)
79. Child 1	Month [___|___] Year [___|___]	[___]	[___]	Month [___|___] Year [___|___]
80. Child 2	Month [___|___] Year [___|___]	[___]	[___]	Month [___|___] Year [___|___]
81. Child 3	Month [___|___] Year [___|___]	[___]	[___]	Month [___|___] Year [___|___]
82. Child 4	Month [___|___] Year [___|___]	[___]	[___]	Month [___|___] Year [___|___]
83. Child 5	Month [___|___] Year [___|___]	[___]	[___]	Month [___|___] Year [___|___]
84. Child 6	Month [___|___] Year [___|___]	[___]	[___]	Month [___|___] Year [___|___]
85. Child 7	Month [___|___] Year [___|___]	[___]	[___]	Month [___|___] Year [___|___]
86. Child 8	Month [___|___] Year [___|___]	[___]	[___]	Month [___|___] Year [___|___]
87. Child 9	Month [___|___] Year [___|___]	[___]	[___]	Month [___|___] Year [___|___]

If more than 9 children born to the respondent, please continue on page 5 (supplement)

Key for Child Births and Deaths<br>If primary caregiver states:	'Beginning of the year'	For 'Month', enter: 2 for February
	'Middle of the year'	For 'Month', enter: 6 for June
	'End of year'	For 'Month', enter: 11 for November

Home visitor [____|____]	Observers present at interview [____|____] [____|____] [____|____]

APPENDIX V: COHORT STUDY PICTORIAL DIARIES

[____]–[____|____]–[____|____|____|____]–[____|____]   [____|____|____|____]   [___|___]/ [___|___]/ [___|___]

Household ID   Cohort Study ID   Start date (day / month / year)

SYMPTOMS	1<br>Monday<br>Tich achiel	2<br>Tuesday<br>Tich aryo	3<br>Wed<br>Tich adek	4<br>Thursday<br>Tich angwen	5<br>Friday<br>Tich abich	6<br>Saturday<br>Mukaaga	7<br>Sunday<br>Sabiti	1<br>Monday<br>Tich achiel	2<br>Tuesday<br>Tich aryo	3<br>Wed<br>Tich adek	4<br>Thursday<br>Tich angwen	5<br>Friday<br>Tich abich	6<br>Saturday<br>Mukaaga	7<br>Sunday<br>Sabiti
DATE														
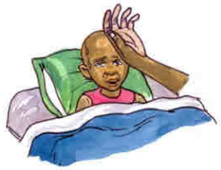														
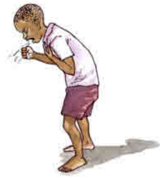														
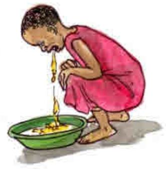														
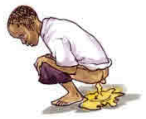														
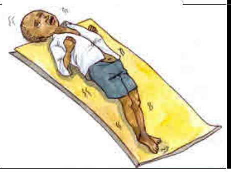														

APPENDIX V: COHORT STUDY PICTORIAL DIARIES

EXPENDITURES<br>Health care fees	1<br>Monday<br>Tich achiel	2<br>Tuesday<br>Tich aryo	3<br>Wed<br>Tich adek	4<br>Thursday<br>Tich angwen	5<br>Friday<br>Tich abich	6<br>Saturday<br>Mukaaga	7<br>Sunday<br>Sabiti	1<br>Monday<br>Tich achiel	2<br>Tuesday<br>Tich aryo	3<br>Wed<br>Tich adek	4<br>Thursday<br>Tich angwen	5<br>Friday<br>Tich abich	6<br>Saturday<br>Mukaaga	7<br>Sunday<br>Sabiti
DATE														
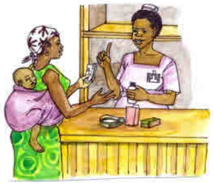														
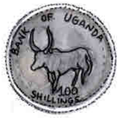 100														
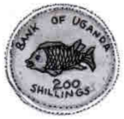 200														
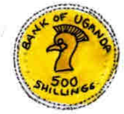 500														
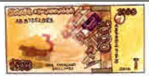 1000														
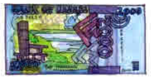 2000														
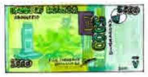 5000														
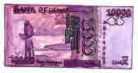 10000														

APPENDIX V: COHORT STUDY PICTORIAL DIARIES

EXPENDITURES<br>Transport	1<br>Monday<br>Tich achiel	2<br>Tuesday<br>Tich aryo	3<br>Wed<br>Tich adek	4<br>Thursday<br>Tich angwen	5<br>Friday<br>Tich abich	6<br>Saturday<br>Mukaaga	7<br>Sunday<br>Sabiti	1<br>Monday<br>Tich achiel	2<br>Tuesday<br>Tich aryo	3<br>Wed<br>Tich adek	4<br>Thursday<br>Tich angwen	5<br>Friday<br>Tich abich	6<br>Saturday<br>Mukaaga	7<br>Sunday<br>Sabiti
DATE														
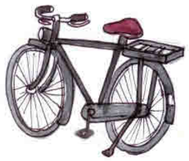	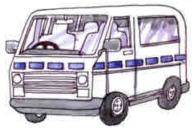	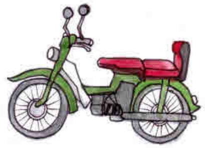												
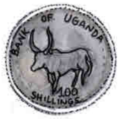 100														
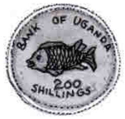 200														
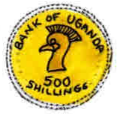 500														
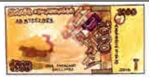 1000														
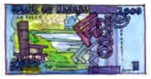 2000														
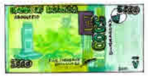 5000														
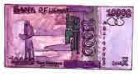 10000														

APPENDIX V: COHORT STUDY PICTORIAL DIARIES

EXPENDITURES<br>Medications	1<br>Monday<br>Tich achiel	2<br>Tuesday<br>Tich aryo	3<br>Wed<br>Tich adek	4<br>Thursday<br>Tich angwen	5<br>Friday<br>Tich abich	6<br>Saturday<br>Mukaaga	7<br>Sunday<br>Sabiti	1<br>Monday<br>Tich achiel	2<br>Tuesday<br>Tich aryo	3<br>Wed<br>Tich adek	4<br>Thursday<br>Tich angwen	5<br>Friday<br>Tich abich	6<br>Saturday<br>Mukaaga	7<br>Sunday<br>Sabiti
DATE														
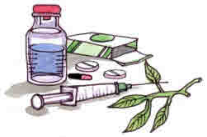														
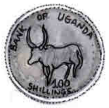 100														
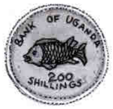 200														
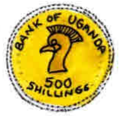 500														
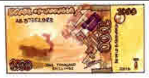 1000														
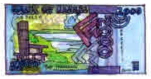 2000														
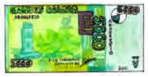 5000														
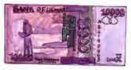 10000														

APPENDIX W: COHORT STUDY MONTHLY QUESTIONNAIRE (1)

Subcounty ID	Village ID	Compound #	Household #
[____|____]	[____|____]	[____|____|____|____]	[____|____]
Study ID [____|____|____|____]		Date of visit [___|___]/[___|___]/[___|___] day month year	Visit # [____|____]

PART 1: EXPERIENCE WITH ILLNESS DURING THE PAST MONTH

1. "Has your child been sick since our last visit?"	1 = Yes<br>2 = No	88 = Don't know<br>99 = Refused to answer	[____|____]
2. "If yes, how many episodes of illness did your child have since our last visit?"	Insert number	88 = Don't know<br>99= Refused to answer	[____|____]

PART 1: SECTION 1: FIRST ACTION

"Now we would like to get a detailed step by step description of everything you did to care for your child during each illness episode during the past one month. There is no right or wrong answer to these questions. We need you to be as open and honest as possible."

3."Did your child have fever with this episode of illness?"	1=Yes<br>2= No	88= Don't know<br>99= Refused to answer	[____|____]

4. "What did you do FIRST (including tepid sponging and herbs)?" (choose only one action)

1 = Nothing
2 = Tepid sponging
3 = Gave herbs kept at home
4 = Gave medicines kept at home
5 = Bought medicines from duka
6 = Bought medicines at drug shop/pharmacy
7 = Took to traditional healer
8 = Took to clinic or hospital
77 = Other ______________
88 = Don't know
99 = Refused to answer

[____|____]

If clinic or hospital, go to Qn.# 5,otherwise skip to Qn # 6

5. "If you took your child to clinic or hospital, where did you go?"	1 = Tororo District Hospital<br>2 = Private hospital/clinic<br>77 = Other ________________	88 = Don't know<br>99 = Refused to answer	[____|____]
6. "How long had the child been ill when this FIRST action was taken?"	1 = < 24 hrs<br>2 = 1-3 days<br>3 = 4-7 days<br>4 = > 7 days<br>77 = Other ____________	88 = Don't know<br>99 = Refused to answer	[____|____]

MEDICINES GIVEN AS FIRST TREATMENT

"If your child took medicine FIRST, what did he/she take?" (Indicate all that were given as a first action)

DRUG	Number of times given per day	Number of days given	How long had the child been ill when the drug was started? 1 = < 24 hrs; 2 = 1-3 days; 3 = 4-7 days; 4 = > 7 days; 77 = Other (list); 88 = Don't know; 99 = Refused to answer
7. Panadol	[____|____]	[____|____]	[____|____] ________________________
8. Aspirin	[____|____]	[____|____]	[____|____] ________________________
9. Chloroquine	[____|____]	[____|____]	[____|____] ________________________
10. Fansidar (SP)	[____|____]	[____|____]	[____|____] ________________________
11. CQ+SP	[____|____]	[____|____]	[____|____] ________________________
12. Amodiaquine	[____|____]	[____|____]	[____|____] ________________________
13. Quinine	[____|____]	[____|____]	[____|____] ________________________
14. Coartem	[____|____]	[____|____]	[____|____] ________________________
15. Septrin (Bactrim)	[____|____]	[____|____]	[____|____] ________________________
16. Amoxacillin	[____|____]	[____|____]	[____|____] ________________________
17. Other ________________	[____|____]	[____|____]	[____|____] ________________________
18. Unknown	[____|____]	[____|____]	[____|____] ________________________

COHORT STUDY MONTHLY QUESTIONNAIRE (2)

Study ID	Date of visit	Visit #
[___|___|___|___]	[___|___]/[___|___]/[___|___]<br>day month year	[___|___]

PART 1: SECTION 2: SECOND ACTION

19. "What did you do SECOND (including tepid sponging and herbs)?" (choose only one action)

1 = Nothing
2 = Tepid sponging
3 = Gave herbs kept at home
4 = Gave medicines kept at home
5 = Bought medicines from duka
6 = Bought medicines at drug shop/pharmacy
7 = Took to traditional healer
8 = Took to clinic or hospital
77 = Other ______________
88 = Don't know
99 = Refused to answer

[___|___]

If clinic or hospital, go to Qn. # 20,otherwise skip to Qn. # 21

20. "If you took your child to clinic or hospital, where did you go?"

1 = Tororo District Hospital
2 = Private hospital/clinic
77 = Other ________________
88 = Don't know
99 = Refused to answer

[___|___]

21. "How long had the child been ill when this SECOND action was taken?"

1 = < 24 hrs
2 = 1-3 days
3 = 4-7 days
4 = > 7 days
77 = Other ____________
88 = Don't know
99 = Refused to answer

[___|___]

MEDICINES GIVEN AS SECOND TREATMENT

"If your child took medicine SECOND, what did he/she take?" (Indicate all that were given as a second action)

DRUG	Number of times given per day	Number of days given	How long had the child been ill when the drug was started?<br>1 = < 24 hrs, 2 = 1-3 days, 3 = 4-7 days, 4 = > 7 days, 77 = Other (list), 88 = Don't know, 99 = Refused to answer
22. Panadol	[___|___]	[___|___]	[___|___] ________________
23. Aspirin	[___|___]	[___|___]	[___|___] ________________
24. Chloroquine	[___|___]	[___|___]	[___|___] ________________
25. Fansidar (SP)	[___|___]	[___|___]	[___|___] ________________
26. CQ+SP	[___|___]	[___|___]	[___|___] ________________
27. Amodiaquine	[___|___]	[___|___]	[___|___] ________________
28. Quinine	[___|___]	[___|___]	[___|___] ________________
29. Coartem	[___|___]	[___|___]	[___|___] ________________
30. Septrin (Bactrim)	[___|___]	[___|___]	[___|___] ________________
31. Amoxacillin	[___|___]	[___|___]	[___|___] ________________
32. Other ______________	[___|___]	[___|___]	[___|___] ________________
33. Unknown	[___|___]	[___|___]	[___|___] ________________

IF ILLNESS RESOLVED, skip to Part 1: Section 4: Treatment outcome

COHORT STUDY MONTHLY QUESTIONNAIRE (3)

Study ID	Date of visit	Visit #
[___|___|___|___]	[___|___]/ [___|___]/[___|___]<br>day month year	[___|___]

PART 1: SECTION 3: THIRD ACTION

34. "What did you do THIRD (including tepid sponging and herbs)?" (choose only one action)

1 = Nothing
2 = Tepid sponging
3 = Gave herbs kept at home
4 = Gave medicines kept at home
5 = Bought medicines from duka
6 = Bought medicines at drug shop/pharmacy
7 = Took to traditional healer
8 = Took to clinic or hospital
77 = Other ____________
88 = Don't know
99 = Refused to answer

[___|___]

If clinic or hospital, go to Qn.#35,otherwise skip to Qn # 36

35. "If you took your child to clinic or hospital, where did you go?"

1 = Tororo District Hospital
2 = Private hospital/clinic
77 = Other ______________
88 = Don't know
99 = Refused to answer

[___|___]

36. "How long had the child been ill when this THIRD action was taken?"

1 = < 24 hrs
2 = 1-3 days
3 = 4-7 days
4 = > 7 days
77 = Other ___________
88 = Don't know
99 = Refused to answer

[___|___]

MEDICINES GIVEN AS THIRD TREATMENT

"If your child took medicine THIRD, what did he/she take?" (Indicate all that were given as a third action)

DRUG	Number of times given per day	Number of days given	How long had the child been ill when the drug was started?<br>1 = < 24 hrs<br>2 = 1-3 days<br>3 = 4-7 days<br>4 = > 7 days<br>77 = Other (list)<br>88 = Don't know<br>99 = Refused to answer
37. Panadol	[___|___]	[___|___]	[___|___] ____________________
38. Aspirin	[___|___]	[___|___]	[___|___] ____________________
39. Chloroquine	[___|___]	[___|___]	[___|___] ____________________
40. Fansidar (SP)	[___|___]	[___|___]	[___|___] ____________________
41. CQ+SP	[___|___]	[___|___]	[___|___] ____________________
42. Amodiaquine	[___|___]	[___|___]	[___|___] ____________________
43. Quinine	[___|___]	[___|___]	[___|___] ____________________
44. Coartem	[___|___]	[___|___]	[___|___] ____________________
45. Septrin (Bactrim)	[___|___]	[___|___]	[___|___] ____________________
46. Amoxacillin	[___|___]	[___|___]	[___|___] ____________________
47. Other ________________	[___|___]	[___|___]	[___|___] ____________________
48. Unknown	[___|___]	[___|___]	[___|___] ____________________

IF ILLNESS RESOLVED, go to Part 1: Section 4: Treatment outcome, otherwise continue sequence of events on MONTHLY QUESTIONNAIRE form (PART 2).

COHORT STUDY MONTHLY QUESTIONNAIRE (4)

Study ID	Date of visit	Visit #
[___|___|___|___]	[__|__]/[__|__]/[__|__]<br>day month year	[___|___]

PART 1: SECTION 4: TREATMENT OUTCOME

Question	Codes		Response
49. "How long did the child's illness last?"	1 = < 24 hrs<br>2 = 1-3 days<br>3 = 4-7 days<br>4 = >7 days	5 = Ongoing at time of interview<br>77 = Other______________________<br>88 = Don't know<br>99 = Refused to answer	[___|___]
50. "Did you experience any delays in treating your child's illness?"	1 = Yes<br>2 = No	88 = Don't know<br>99 = Refused to answer	[___|___]<br>If no, skip to question #52
51. "What were the reasons for the delays?" (list all that apply)	1 = No transport available<br>2 = Not enough money available<br>3 = Needed to find coverage for work	4 = Needed to arrange for child care<br>5 = Waiting at the health facility<br>88 = Don't know<br>99 = Refused to answer	[___|___] [___|___]<br>[___|___] [___|___]<br>[___|___] [___|___]
52. "How much did you spend on management of this illness?"	Cost of drugs<br>[___|___|___|___|___]Ush<br>Fees (clinic, hospital, lab)<br>[___|___|___|___|___]Ush	Transport<br>[___|___|___|___|___]Ush<br>Other<br>[___|___|___|___|___]Ush<br>TOTAL<br>[___|___|___|___|___]Ush	
73. "Did caring for your child and managing his/her illness prevent you from doing your usual activities this month?"	1 = Yes<br>2 = No	88 = Don't know<br>99 = Refused to answer	[___|___]
74. "If yes, how much time did you miss?"	If < 1 day, indicate number of hours otherwise, record 00		[___|___] hours<br>[___|___] days

Field worker initials	[___|___]	Initials of observers present at interview [___|___] [___|___] [___|___]

If the child experienced an additional episode of illness, go to Part 2 and complete the information for additional episodes.

COHORT STUDY MONTHLY QUESTIONNAIRE (5)

Study ID	Date of visit	Visit #
[____|____|____|____]	[___|___]/ [___|___]/[___|___]<br>day month year	[____|____]

PART 2: EXPERIENCE WITH ILLNESS DURING THE PAST MONTH – Extra fever episodes

PART 2: SECTION 1: FIRST ACTION

"Now we would like to get a detailed step by step description of everything you did to care for your child during each illness episode during the past one month. There is no right or wrong answer to these questions. We nned you to be as open and honest as possible."

55."Did your child have fever with this episode of illness?"
1=Yes 88= Don't know
2= No 99= Refused to answer
[____|____]

56. "What did you do FIRST (including tepid sponging and herbs)?" (choose only one action)
1 = Nothing
2 = Tepid sponging
3 = Gave herbs kept at home
4 = Gave medicines kept at home
5 = Bought medicines from duka
6 = Bought medicines at drug shop/pharmacy
7 = Took to traditional healer
8 = Took to clinic or hospital
77 = Other ______________
88 = Don't know
99 = Refused to answer
[____|____]
If clinic or hospital, go to #57,otherwise skip to Qn # 58

57. "If you took your child to clinic or hospital, where did you go?"
1 = Tororo District Hospital
2 = Private hospital/clinic
77 = Other ______________
88 = Don't know
99 = Refused to answer
[____|____]

58. "How long had the child been ill when this FIRST action was taken?"
1 = < 24 hrs
2 = 1-3 days
3 = 4-7 days
4 = > 7 days
77 = Other ____________
88 = Don't know
99 = Refused to answer
[____|____]

MEDICINES GIVEN AS FIRST TREATMENT
"If your child took medicine FIRST, what did he/she take?" (Indicate all that were given as a first action)

DRUG	Number of times given per day	Number of days given	How long had the child been ill when the drug was started?<br>1 = < 24 hrs 2 = 1-3 days 3 = 4-7 days 4 = > 7 days 77 = Other (list) 88 = Don't know 99 = Refused to answer
59. Panadol	[___|___]	[___|___]	[___|___] ____________________
60. Aspirin	[___|___]	[___|___]	[___|___] ____________________
61. Chloroquine	[___|___]	[___|___]	[___|___] ____________________
62. Fansidar (SP)	[___|___]	[___|___]	[___|___] ____________________
63. CQ+SP	[___|___]	[___|___]	[___|___] ____________________
64. Amodiaquine	[___|___]	[___|___]	[___|___] ____________________
65. Quinine	[___|___]	[___|___]	[___|___] ____________________
66. Coartem	[___|___]	[___|___]	[___|___] ____________________
67. Septrin (Bactrim)	[___|___]	[___|___]	[___|___] ____________________
68. Amoxacillin	[___|___]	[___|___]	[___|___] ____________________
69. Other ______________	[___|___]	[___|___]	[___|___] ____________________
70. Unknown	[___|___]	[___|___]	[___|___] ____________________

IF ILLNESS RESOLVED, skip to Part 2: Section 4: Treatment outcome

COHORT STUDY MONTHLY QUESTIONNAIRE (6)

Study ID	Date of visit	Visit #
[___|___|___|___]	[___|___]/[___|___]/[___|___]<br>day month year	[___|___]

PART 2: SECTION 2: SECOND ACTION

71. "What did you do SECOND (including tepid sponging and herbs)?" (choose only one action)

1 = Nothing
2 = Tepid sponging
3 = Gave herbs kept at home
4 = Gave medicines kept at home
5 = Bought medicines from duka
6 = Bought medicines at drug shop/pharmacy
7 = Took to traditional healer
8 = Took to clinic or hospital
77 = Other _______________
88 = Don't know
99 = Refused to answer

[___|___]

If clinic or hospital, go to #72,otherwise skip to Qn # 73

72. "If you took your child to clinic or hospital, where did you go?"

1 = Tororo District Hospital
2 = Private hospital/clinic
77 = Other ________________
88 = Don't know
99 = Refused to answer

[___|___]

73. "How long had the child been ill when this SECOND action was taken?"

1 = < 24 hrs
2 = 1-3 days
3 = 4-7 days
4 = > 7 days
77 = Other ____________
88 = Don't know
99 = Refused to answer

[___|___]

74. Panadol	[___|___]	[___|___]	[___|___] ________________________
75. Aspirin	[___|___]	[___|___]	[___|___] ________________________
76. Chloroquine	[___|___]	[___|___]	[___|___] ________________________
77. Fansidar (SP)	[___|___]	[___|___]	[___|___] ________________________
78. CQ+SP	[___|___]	[___|___]	[___|___] ________________________
79. Amodiaquine	[___|___]	[___|___]	[___|___] ________________________
80. Quinine	[___|___]	[___|___]	[___|___] ________________________
81. Coartem	[___|___]	[___|___]	[___|___] ________________________
82. Septrin (Bactrim)	[___|___]	[___|___]	[___|___] ________________________
83. Amoxacillin	[___|___]	[___|___]	[___|___] ________________________
84. Other ____________________	[___|___]	[___|___]	[___|___] ________________________
85. Unknown	[___|___]	[___|___]	[___|___] ________________________

IF ILLNESS RESOLVED, skip to Part 2: Section 4: Treatment outcome

COHORT STUDY MONTHLY QUESTIONNAIRE (7)		
Study ID [___|___|___|___]	Date of visit [___|___]/[___|___]/[___|___] day month year	Visit # [___|___]

PART 2: SECTION 3: THIRD ACTION

86. "What did you do THIRD (including tepid sponging and herbs)?" (choose only one action)

1 = Nothing	6 = Bought medicines at drug shop/pharmacy	77 = Other ______________
2 = Tepid sponging	7 = Took to traditional healer	88 = Don't know
3 = Gave herbs kept at home	8 = Took to clinic or hospital	99 = Refused to answer
4 = Gave medicines kept at home		
5 = Bought medicines from duka		

[___|___]

If clinic or hospital, go to 87,otherwise skip to Qn #88

87. "If you took your child to clinic or hospital, where did you go?"

1 = Tororo District Hospital
2 = Private hospital/clinic
77 = Other ______________
88 = Don't know
99 = Refused to answer

[___|___]

88. "How long had the child been ill when this THIRD action was taken?"

1 = < 24 hrs
2 = 1-3 days
3 = 4-7 days
4 = > 7 days
77 = Other ___________
88 = Don't know
99 = Refused to answer

[___|___]

MEDICINES GIVEN AS THIRD TREATMENT

"If your child took medicine THIRD, what did he/she take?" (Indicate all that were given as a third action)

DRUG	Number of times given per day	Number of days given	How long had the child been ill when the drug was started? 1 = < 24 hrs; 2 = 1-3 days; 3 = 4-7 days; 4 = > 7 days; 77 = Other (list); 88 = Don't know; 99 = Refused to answer
89. Panadol	[___|___]	[___|___]	[___|___] ____________________
90. Aspirin	[___|___]	[___|___]	[___|___] ____________________
91. Chloroquine	[___|___]	[___|___]	[___|___] ____________________
92. Fansidar (SP)	[___|___]	[___|___]	[___|___] ____________________
93. CQ+SP	[___|___]	[___|___]	[___|___] ____________________
94. Amodiaquine	[___|___]	[___|___]	[___|___] ____________________
95. Quinine	[___|___]	[___|___]	[___|___] ____________________
96. Coartem	[___|___]	[___|___]	[___|___] ____________________
97. Septrin (Bactrim)	[___|___]	[___|___]	[___|___] ____________________
98. Amoxacillin	[___|___]	[___|___]	[___|___] ____________________
99. Other ________________	[___|___]	[___|___]	[___|___] ____________________
100. Unknown	[___|___]	[___|___]	[___|___] ____________________

COHORT STUDY MONTHLY QUESTIONNAIRE (8)

Study ID	Date of visit	Visit #
[____|____|____|____]	[__|___]/ [__|___]/[___|___] day month year	[____|____]

PART 2: SECTION 4: TREATMENT OUTCOME

101. "How long did the child's illness last?"	1 = < 24 hrs<br>2 = 1-3 days<br>3 = 4-7 days<br>4 = >7 days	5 = Ongoing at time of interview<br>77 = Other_______________________<br>88 = Don't know<br>99 = Refused to answer	[____|____]
102. "Did you experience any delays in treating your child's illness?"	1 = Yes<br>2 = No	88 = Don't know<br>99 = Refused to answer	[____|____]<br>If no, skip to question #104
103. "What were the reasons for the delays?" (list all that apply)	1 = No transport available<br>2 = Not enough money available<br>3 = Needed to find coverage for work	4 = Needed to arrange for child care<br>5 = Waiting at the health facility<br>88 = Don't know<br>99 = Refused to answer	[___|____] [____|____]<br>[___|____] [____|____]<br>[___|____] [____|____]
104. "How much did you spend on management of this illness?"	Cost of drugs<br>[____|____|____|____|____]Ush<br>Fees (clinic, hospital, lab)<br>[____|____|____|____|____]Ush		Transport<br>[____|____|____|____|____]Ush<br>Other<br>[____|____|____|____|____]Ush<br>TOTAL<br>[____|____|____|____|____]Ush
105. "Did caring for your child and managing his/her illness prevent you from doing your usual activities this month?"	1 = Yes<br>2 = No	88 = Don't know<br>99 = Refused to answer	[____|____]
106. "If yes, how much time did you miss?"	If < 1 day, indicate number of hours otherwise, record 00		[____|____] hours<br>[____|____] days

Field worker initials [____|____]	Initials of observers present at interview [___|___] [____|____] [____|____]

APPENDIX X: EXIT INTERVIEW SCREENING FORM

PART 1: PARTICIPANT INFORMATION

Screening Date<br>[_____|______]/[______|_____]/[______|______]<br>day month year		Screening ID<br>[______|______|______|______]	
Village name	______________________________	Sub-county ID<br>[____]	Village ID<br>[____|_____]
Child's age	[_____|_____] / [_____|_____]<br>years months	If child is less than 1 year, complete months, otherwise leave blank	
Gender	[____]	1 = Male<br>2 = Female	
Was your child referred to another health centre today?	[_____]	1 = Yes 88 = Don't know<br>2 = No<br>If yes, exclude and allow caregiver to leave	
If the caregiver is not answering your questions, indicate why	[_____|_____]	1 = Refused to talk<br>2 = Refused to wait<br>77 = Other: list ______________________	

SCREENING STAGE 1 – Appropriate age

Selection criteria	Include	Exclude	
Is the child the appropriate age<br>—Under five (aged 0 to less than 5 years)	1 = Yes	2 = No	[_____]
If the answer is '2' from the EXCLUDE column, exclude from the study. If not, proceed to the next section.			

INFORMATION ON STUDY

Read the following to the caregiver:	My name is ________________________. I work with the Uganda Malaria Surveillance Project which is part of the Infectious Disease Research Collaboration. We would like to ask you some questions about your experience today at this health centre and briefly examine your child. This should take about 30 minutes.<br>Would you be able participate now?			
Does the caregiver agree to continue?		Continue<br>1 = Yes	End interview<br>2 = No	[_____]
If No, indicate reason		1 = Refused to talk<br>2 = Refused to wait	77 = Other: list<br>________________________	[_____|_____]
Proceed with obtaining informed consent				

SCREENING STAGE 2 – Informed consent

Selection criteria	Include	Exclude	
Is the parent(s)/guardian(s) willing to provide informed consent?	1 = Yes	2 = No	[_____]
If the answer is '2' from the EXCLUDE column, exclude from the study. If not, proceed to the next section.			

ASSIGN STUDY NUMBER	[_____|_____|_____|_____]

All criteria for study inclusion met?<br>1 = Yes<br>2 = No If no, exclude from the study	[_____]	Date of enrollment<br>[______|______]/[______|______]/[______|______]<br>day month year

THE REPUBLIC OF UGANDA

[_____|_____|_____|_____]

Study ID

APPENDIX Y. PATIENT EXIT INTERVIEWS
Research participant informed consent form

Protocol Title:	ACT PRIME Study: Evaluating the impact of enhanced health facility-based care for malaria and febrile illnesses in children
Site of Research:	Tororo, Uganda
Principal Investigators:	Dr. Sarah Staedke
Date:	18 September 2010

Introduction

Dr. Sarah Staedke and colleagues from the Uganda Malaria Surveillance Project / Infectious Diseases Research Collaboration are investigating delivery of healthcare services in Tororo District. We are doing a research study to see if we can improve the health of children in this area by improving services at government-run health facilities.

Why is this survey being done?

Certain health centers in Tororo district will be selected to either take part in the intervention to improve services, or to continue with their current services. Assignment to the two groups has been determined by a lottery. The chance of being placed into either of the groups is the same. To find out how well the intervention is working, we would like to review the health of children under five who go to the health centers for care. We would like to know if caregivers of children are satisfied with their visit to the health centers. We would also like to hear any suggestions on how to improve visits to these health centers. We would like to interview the caregivers of 10 children under five who have visited the health centers; 200 children in total will be surveyed.

What will happen if my child takes part in this survey?

If you agree to let your child participate in this survey, the following will happen today:

a) We will ask you some questions about your visit to the health center today including the purpose of the visit, your child's symptoms, whether a test for malaria was done, and what treatment was given. We will also ask questions about whether you were satisfied with your visit or not.
b) We will collect information on your child's general health.
c) We will briefly examine your child.
d) If your child has had a fever in the last 48 hours (2 days) or has a high temperature, we will do a rapid diagnostic test for malaria.

e) If your child has a positive test for malaria, and appropriate treatment for malaria has not been given, we will provide treatment with artemether-lumefantrine (including Coartem or Lumatem), which is the recommended treatment for simple malaria in Uganda.
f) If your child has any signs of severe malaria or another significant illness, we will refer you and your child back to the health center or to the hospital for further care.

After today, we will not ask you to do anything further.

How long will this survey last?
Today, the interview will last about 30 minutes.

Can I stop my child from being in the survey?
You can decide to stop participating at any time. Just tell the project researcher right away if you wish to stop the activities.

What risks can I expect if my child participates in the survey?
We will obtain one blood sample by fingerprick from your child. The risks of drawing blood from a fingerprick include temporary discomfort from the needle stick, bruising, and skin infection. The amount of blood removed will be too small to affect your child's health.

Participation in any research study may involve a loss of privacy. Information you provide about your health center will be recorded, but your name will not be used in any reports of the information provided. The information obtained from these study activities will only be used by the project researchers and will be locked at our project offices. We will do our best to make sure that any personal information is kept private.

Are there benefits if my child takes part in the survey?
If we find that your child has malaria and has not been given appropriate treatment at the health center, we will give you medications and instructions on how to treat your child. Otherwise, there will be no direct benefit to you from participating in this study. However, the information that you provide will help researchers and policy-makers understand who best to improve health services in this area.

What other choices do I have if my child does not take part in the survey?
You are free to choose not to participate in the study. If you decide not to let your child take part, there will be no penalty to you.

THE REPUBLIC OF UGANDA

What are the costs of taking part in the survey? Will my child be paid for taking part in the survey?

There are no costs to you for taking part in this study. You and your child will not be paid for taking part in this study.

What are my rights if I allow my child to take part in the survey?

Taking part in this survey is your choice. You may choose either to take part or not to take part. If you decide to take part in this survey, you may change your mind at any time. If you decide to withdraw your child from the survey; your child will still be eligible for care at the local health facility and at Tororo District Hospital and at other local clinics. No matter what decision you take, there will be no penalty to you in any way.

What if my child is injured as result of being in this survey?

If your child is injured, or if you have questions about injuries as a result of being in the survey, please contact Dr. Sarah Staedke or other members of the Uganda Malaria Surveillance Project / Infectious Disease Research Collaboration on telephone number 0414-530692. The sponsoring organizations do not have a program to cover your costs if your child is hurt or has other bad results.

Who can answer my questions about the survey?

You can talk to the researchers about any questions or concerns you have about these survey activities. Contact Dr. Sarah Staedke or other members of the Uganda Malaria Surveillance Project / Infectious Disease Research Collaboration on telephone number 0414-530692. If you have any questions, comments or concerns about taking part in these activities, first talk to the researchers. If for any reason you do not wish to do this, or you still have concerns about doing so, you may contact Dr Charles Ibingira, Makerere University Faculty of Medicine Research and Ethical Committee at telephone number 0414-530020.

THE REPUBLIC OF UGANDA

WHAT YOUR SIGNATURE OR THUMBPRINT MEANS

Your signature or thumbprint below means that you understand the information given to you in this consent form about your child's participation in the survey and agree with the following statements:

1. "I have read the consent form concerning this survey (or have understood the verbal explanation of the consent form) and I understand what will be required of me and what will happen to me and my child if we take part in it."

2. "My questions concerning this survey have been answered by Dr. Staedke or the person who signed below."

3. "I understand that at any time, I may withdraw my child from this survey without giving a reason and without affecting my child's normal health care and management."

4. "I agree that the child under my care will take part in this survey."

You will also be asked to sign another informed consent form for the use of stored specimens. If you wish your child to participate in this survey, you should sign or place your thumbprint below.

THE REPUBLIC OF UGANDA

WE WILL GIVE YOU A COPY OF THIS SIGNED AND DATED CONSENT FORM

Name of Participant (printed)

Name of Parent/Guardian

Signature or Fingerprint * of Parent/Guardian — Date/Time

Name of Investigator Administering Consent (printed) — Position/Title

Signature of Investigator Administering Consent — Date/Time

*If the parent or guardian is unable to read and/or write, an impartial witness should be present during the informed consent discussion. After the written informed consent form is read and explained to the participant and parent or guardian, and after they have orally consented to their child's participation in the trial, and have either signed the consent form or provided their fingerprint, the witness should sign and personally date the consent form. By signing the consent form, the witness attests that the information in the consent form and any other written information was accurately explained to, and apparently understood by the parent or guardian, and that informed consent was freely given by the patient and parent or guardian.

Name of Person Witnessing Consent (printed)

Signature of Person Witnessing Consent — Date/Time

APPENDIX Z: PATIENT EXIT INTERVIEW QUESTIONNAIRE (1)

Section 1: Patient/Caregiver information

Health centre code	Study ID	Date of interview	
[____|____]	[____|____|____|____]	[____|____]/[____|____]/[____|____]<br>date month year	
Sub-county ID<br>[____]	Village name<br>______________________________	Village ID<br>[____|____|____]	
CHILD	If child is less than 1 year, complete months, otherwise leave blank	Age<br>[_____|_____] / [_____|_____]<br>years months	Gender<br>[____] 1 = Male<br>2 = Female
CAREGIVER		Age<br>[_____|_____]years	Gender<br>[____] 1 = Male<br>2 = Female

SECTION 2: CLINICAL HISTORY

1. What is the reason for your visit to the health center today? (Describe below)		
2. Did child have fever in the last 48 hours (2 days)?	1 = Yes 88 = Don't know<br>2 = No 99 = Refused	[_____|_____]
3. What other problems did the child have? (Describe below, and list all appropriate codes at right)	1 = Cough 6 = Weak (not playing)<br>2 = Flu 7 = Convulsions<br>3 = Not eating 8 = Other<br>4 = Vomiting 77 = None<br>5 = Diarrhea	[_____]<br>[_____]<br>[_____]
4. Did your child have a diagnostic test for malaria done today?<br>If no, skip to question 6.	1 = Yes 88 = Don't know<br>2 = No 99 = Refused	[_____|_____]
5. If yes, what was the result of the test?	1 = Positive 88 = Don't know<br>2 = Negative 99 = Refused	[_____|_____]
6. Were you told what is causing your child's illness?<br>If no, skip to question 8.	1 = Yes 88 = Don't know<br>2 = No 99 = Refused	[_____|_____]
7. What diagnoses was your child given?	1 = Malaria 7 = Measles<br>2 = Ear infection 8 = Urine infection<br>3 = Throat infection 9 = Other<br>4 = Pneumonia 88 = Don't know<br>5 = Gastroenteritis 99 = Refused<br>6 = Dysentery	[_____|_____]<br>[_____|_____]
8. What treatment was prescribed for your child?	1 = Panadol 11 = Amoxacillin<br>2 = AL (Coartem, Lumartem) 12 = Septrin<br>3 = DP (Duocotecxin) 13 = Iron (Ferrous)<br>4 = Quinine 14 = ORS<br>5 = Chloroquine only 15 = Vitamin A<br>6 = SP only (Fansidar) 20 = Other<br>7 = CQ+SP combination 88 = Don't know<br>8 = Amodiaquine (Camoquin) 99 = Refused	[_____|_____]<br>[_____|_____]<br>[_____|_____]<br>[_____|_____]
9. Did you receive the medications that were prescribed?<br>If yes, skip to next section.	1 = Yes 88 = Don't know<br>2 = No 99 = Refused	[_____|_____]
10. If no, why?	1 = Drug out of stock 88 = Don't know<br>2 = Couldn't pay for drug 99 = Refused<br>3 = Other	[_____|_____]

PATIENT EXIT INTERVIEW QUESTIONNAIRE (2)

Health centre code	Study ID	Date of interview
[_____|_____]	[_____|_____|_____|_____]	[_____|_____]/[_____|_____]/[_____|_____]<br>date month year

SECTION 3: SATISFACTION WITH HEALTH CARE<br>1 = very dissatisfied, 2 = dissatisfied, 3 = uncertain, 4 = satisfied, 5 = very satisfied					
1. How satisfied are you with the length of time you had to wait today?	1	2	3	4	5
2. How satisfied are you with the waiting space available?	1	2	3	4	5
3. How satisfied are you with the health center buildings and the consultation rooms?	1	2	3	4	5
4. How satisfied are you with the directions provided at the health center?	1	2	3	4	5
5. How satisfied are you with the attitude of health workers toward you?	1	2	3	4	5
6. How satisfied are you with the consultation and examination of your child?	1	2	3	4	5
7. How satisfied are you with the laboratory tests done?	1	2	3	4	5
8. How satisfied are you with the treatment prescribed?	1	2	3	4	5
9. How satisfied are you with the medications provided?	1	2	3	4	5
10. How satisfied are you with the explanation of why your child is sick?	1	2	3	4	5

SECTION 4: SUGGESTIONS

What do you think can be done to improve services at this health center?

Time of ARRIVAL to health center	Time of DEPARTURE from health center
[_____|_____]:[_____|_____]<br>hours minutes	[_____|_____]:[_____|_____]<br>hours minutes

PATIENT EXIT INTERVIEW QUESTIONNAIRE (3)

Health centre code	Study ID	Date of interview
[_____|_____]	[_____|_____|_____|_____]	[_____|_____]/[_____|_____]/[_____|_____]<br>date month year

SECTION 3: FEVER EPISODE

Complete ONLY if there is a history of fever or documented temperature (≥ 38.0°C)

1. Clinical Notes – History of present illness

2. Physical examination findings

3. Temperature (ºC) [____|____]•[____]

4. PRIME RDT result — If negative, skip to Qn 6. — 1 = Positive; 2 = Negative; 3 = Not performed; 4 = Test failed [____]

5. If the RDT is positive, was the child prescribed appropriate treatment for malaria at the health center? — 1 = Yes; 2 = No [____]

6. Diagnosis made by PRIME

1 = Uncomplicated malaria
2 = Severe malaria
3 = Otitis media
4 = Pharyngitis
5 = Upper respiratory tract infection
6 = Pneumonia
7 = Gastroenteritis
8 = Diarrhea
9 = Dysentery
10 = Urinary tract infection
11 = Skin infection
12 = Viral illness
77 = Other
88 = Unknown

[_____|_____]

8. Medications prescribed by PRIME

1 = Panadol
7 = Quinine
8 = Coartem
10 = Amoxacillin
11 = None
12 = Other (list)
____
____

Drug 1 [____|____]
Drug 2 [____|____]
Drug 3 [____|____]
Drug 4 [____|____]
Drug 5 [____|____]

9. Was the child referred for additional care by PRIME staff? — 1 = Yes; 2 = No [____]

10. If yes, where? (specify)

Initials: [____|____]

APPENDIX AA. INFORMATION SHEET - Health facility surveillance
ACT PRIME Study

Introduction

Dr. Sarah Staedke and colleagues from the Uganda Malaria Surveillance Project / Infectious Diseases Research Collaboration are investigating delivery of health care services in Tororo District. We are doing a research study to see if we can improve the health of children in this area by improving services at government-run health facilities. Certain health centers in Tororo district will be selected to participate in the intervention to improve services, or to continue with their current services. Assignment to the two groups has been determined by a lottery. The chance of being placed into either of the groups is the same.

Why is this surveillance being done?

As part of this study, we would like to know more about the types of patients seen, and the treatment provided, at lower-level health centers (HC IIs and HC IIIs) is this area. We would also like to know more about drug supplies, staffing and costs at HC IIs and IIIs.

What will happen if I take part in this surveillance?

We will ask you, or another available health worker, to provide us with your registers, including the patient, drug, and laboratory (if applicable) registers. We will review the registers and enter the data into a questionnaire. We estimate that it will take one day to enter the data. We will also ask you some questions about staffing at the health center over the past two months, which should last about 15 minutes. Once a year we will also ask you questions about routine costs at this health centre, which should last about 30-60 minutes.

How long will this surveillance last?

We plan to conduct the surveillance over 2 years. We will visit your health facility once every two months for a total of about 2 years.

Can I stop being in the surveillance?

You can decide to stop participating at any time. Just tell our study personnel right away if you wish to stop the activities.

What risks can I expect from participating in the surveillance?

Participation in any research study may involve a loss of privacy. Information you provide about your health center will be recorded, but your name will not be used in any reports of the information provided. No quotes or other results arising from your participation in this study will be included in any reports, even

anonymously, without your agreement. The information obtained from these surveillance activities will only be used by the project researchers and will be locked at our project offices. We will do our best to make sure that any personal information is kept private.

Are there benefits to taking part in the surveillance?

There will be no direct benefit to you from participating in this study. However, the information that you provide will help researchers and policy-makers understand who best to improve health services in this area.

What other choices do I have if I do not take part in the surveillance?

You are free to choose not to participate in the study. If you decide not to take part, there will be no penalty to you.

What are the costs of taking part in the surveillance? Will I be paid for taking part in the surveillance?

There are no costs to you for taking part in this study. You will not be paid for taking part in this study.

What are my rights if I take part in the surveillance?

Taking part in this study is your choice. You may choose either to take part or not to take part. If you decide to take part in this study, you may change your mind at any time. No matter what decision you take, there will be no penalty to you in any way.

Who can answer my questions about the surveillance?

You can talk to the researchers about any questions or concerns you have about these surveillance activities. Contact Dr. Sarah Staedke or other members of the Uganda Malaria Surveillance Project / Infectious Disease Research Collaboration on telephone number 0414-530692. If you have any questions, comments or concerns about taking part in these activities, first talk to the researchers. If for any reason you do not wish to do this, or you still have concerns about doing so, you may contact Dr Charles Ibingira, Makerere University Faculty of Medicine Research and Ethical Committee at telephone number 0414-530020.

Giving verbal consent to participate in the surveillance:

You may keep this information sheet if you wish. Participation in these activities is voluntary. You have the right to decline to participate in the activities, or to withdraw from them at any point without penalty. If you do not wish to participate in the activities, you should inform the researcher now. If you do wish to participate in these activities, you should tell the researcher now, and the interview will begin shortly.

APPENDIX BB: HEALTH FACILITY SURVEILLANCE FORM (1)

PART 1: Section 1: Health Center Information

Health centre code	Health worker ID	Date Report Completed
[_____|_____]	[_____|_____]	[_____|_____]/[_____|_____]/[_____|_____]<br>day month year

START date of report (this should be the STOP date from the previous months' report)	STOP date of report
[_____|_____]/[_____|_____]/[_____|_____]<br>day month year	[_____|_____]/[_____|_____]/[_____|_____]<br>day month year

Section 2: Review of Record Books

Assign each patient in the Patient Register a Patient Number and record into the PDA as much information as possible on each patient who attended the health facility since the date of the last visit using the Patient Register, Laboratory Log Book, and Dispensing Record.

From Patient Register		From Laboratory Log Book	From Dispensing Record
1. Date of visit<br>2. Age of patient<br>3. Gender of patient<br>4. Village of residence<br>5. Fever or history of fever	6. Diagnosis<br>(if health facility has RDTs or a laboratory, see the Laboratory Log Book)<br>7. Treatment prescribed	1. RDT performed<br>2. Result of RDT<br>3. Laboratory tests performed<br>4. Result of test	1. Drug dispensed<br>2. Amount

Section 3: Drug Stock Report

1. Was AL from NMS out-of-stock this month?	1 = Yes<br>2 = No	88 = Don't know<br>99 = Refused to answer	[____|____]<br>If No, skip to question 9

	2. List date when stock-out of AL began:	3. Was AL re-stocked after this stock-out?<br>1 = Yes<br>2 = No<br>88 = Don't know<br>99 = Refused to answer<br>If No, skip to question 9	4. If yes, who re-stocked your drugs?<br>1 = NMS<br>2 = ACT PRIME study (us)<br>3 = Used PHC or HF funds. List amount<br>77 = Other: list<br>88 = Don't know<br>99 = Refused to answer	5. What date was AL re-stocked?
1st	[__ ___/__ ___/___ __]<br>day month year	[____|____]	[___|____]<br>amount: UGX___________________<br>Other: list:_____________________	[__ ___/__ ___/___ __]<br>day month year
2nd	[__ ___/__ ___/___ __]<br>day month year	[____|____]	[___|____]<br>amount: UGX___________________<br>Other: list:_____________________	[__ ___/__ ___/___ __]<br>day month year
3rd	[__ ___/__ ___/___ __]<br>day month year	[____|____]	[___|____]<br>amount: UGX___________________<br>Other: list:_____________________	[__ ___/__ ___/___ __]<br>day month year

9. If your supply of AL ran low, did you ration or keep some drugs aside?	10. If yes, for what reasons did you ration or keep some drugs aside? List all that apply
[_____]<br>1 = Yes<br>2 = No<br>If no, skip to next section	[_____|_____] [_____|_____] [_____|_____] [_____|_____]<br>1 = To have drugs to treat children<br>2 = To have drugs to treat severe cases of malaria<br>3 = To have drugs available if a politician visits<br>77 = Other, ____________________________

HEALTH FACILITY SURVEILLANCE FORM (2)

Health centre code	Health worker ID	Date Report Completed
[____|____]	[____|____]	[____|____]/[____|____]/[____|____]<br>day month year

START date of report<br>(this should be the STOP date from the previous months' report)	STOP date of report
[____|____]/[____|____]/[____|____]<br>day month year	[____|____]/[____|____]/[____|____]<br>day month year

Section 4: Staff Availability

Speak with the in-charge to determine the availability of each staff person assigned to the health facility

1 = In-charge	5 = Clinical officer	9 = Public health nurse	13 = Health assistant
2 = Senior medical officer	6 = Nursing officer	10 = Nursing aide/assistant	14 = Health educator
3 = Medical officer	7 = Enrolled nurse	11 = Laboratory technician	15 = Other____________
4 = Senior clinical officer	8 = Midwife	12 = Laboratory assistant	16 = Other____________

	Position	Total number of days available		Position	Total number of days available
1)	[____|____]	[____|____]	4)	[____|____]	[____|____]
2)	[____|____]	[____|____]	5)	[____|____]	[____|____]
3)	[____|____]	[____|____]	6)	[____|____]	[____|____]

Section 5: Extra comments

Describe any situation that had an impact on patient care at your health center this month, such as drug stock-outs, staff shortages, or political instability.

HEALTH FACILITY SURVEILLANCE FORM – HEALTH CENTRE INDICATOR REPORTS (3)

PART 2: Section 1: Health Center Information

Health centre code	Health worker ID	Date Report Completed
[____|____]	[____|____]	[____|____]/[____|____]/[____|____]<br>day month year

START date of report<br>(this should be the STOP date from the previous months' report)	STOP date of report
[____|____]/[____|____]/[____|____]<br>day month year	[____|____]/[____|____]/[____|____]<br>day month year

Section 2: Indicator report – ALL PATIENTS

Total number of patients visiting the clinic this month	[____|____|____]	
Number (proportion) of patients who had fever	[____|____|____]	[____|____] %
Number (proportion) of febrile patients who had a RDT done	[____|____|____]	[____|____] %
Number (proportion) of patients who had a positive RDT result	[____|____|____]	[____|____] %
Number (proportion) of patients who had a negative RDT result	[____|____|____]	[____|____] %
Total number of patients who were diagnosed with malaria	[____|____|____]	
Number (proportion) of patients diagnosed with malaria who had a positive RDT	[____|____|____]	[____|____] %
Number (proportion) of patients diagnosed with malaria who had a negative RDT	[____|____|____]	[____|____] %
Total number of patients who were prescribed an ACT* to treat malaria	[____|____|____]	
Number (proportion) of patients who had a positive RDT result who were prescribed an ACT*	[____|____|____]	[____|____] %
Number (proportion) of patients who had a negative RDT result who were prescribed an ACT*	[____|____|____]	[____|____] %
Number (proportion) of patients without an RDT done who were prescribed an ACT*	[____|____|____]	[____|____] %

* ACT = artemisinin-based combination therapy for malaria. Examples include Coartem, Lumartem, Duocotexcin

HEALTH FACILITY SURVEILLANCE FORM – INDICATOR REPORTS (4)

Health centre code	Health worker ID	Date Report Completed
[____|____]	[____|____]	[____|____]/[____|____]/[____|____]<br>day month year
START date of report		STOP date of report
[____|____]/[____|____]/[____|____]<br>day month year		[____|____]/[____|____]/[____|____]<br>day month year

Section 3: Indicator report – CHILDREN UNDER FIVE

Total number of children under five visiting the clinic this month	[____|____|____]	
Number (proportion) of children under five who had fever	[____|____|____]	[____|____] %
Number (proportion) of febrile children under five who had a RDT done	[____|____|____]	[____|____] %
Number (proportion) of children under five who had a positive RDT result	[____|____|____]	[____|____] %
Number (proportion) of children under five who had a negative RDT result	[____|____|____]	[____|____] %
Total number of children under five who were diagnosed with malaria	[____|____|____]	
Number (proportion) of children under five diagnosed with malaria who had a positive RDT	[____|____|____]	[____|____] %
Number (proportion) of children under five diagnosed with malaria who had a negative RDT	[____|____|____]	[____|____] %
Total number of children under five who were prescribed an ACT* to treat malaria	[____|____|____]	
Number (proportion) of children under five who had a positive RDT result who were prescribed an ACT*	[____|____|____]	[____|____] %
Number (proportion) of children under five who had a negative RDT result who were prescribed an ACT*	[____|____|____]	[____|____] %
Number (proportion) of children under five without an RDT done who were prescribed an ACT*	[____|____|____]	[____|____] %

* ACT = artemisinin-based combination therapy for malaria. Examples include Coartem, Lumartem, Duocotexcin

HEALTH FACILITY SURVEILLANCE FORM – COST COLLECTION (1)

PART 3: Section 1: Health Center Information

This section to be completed in all health facilities at baseline and then annually (3 surveys in total)

Health centre code	Health worker ID	Date Report Completed
[_____|_____]	[_____|_____]	[_____|_____]/[_____|_____]/[_____|_____]<br>day month year

Section 2: DRUGS & SUPPLIES

#	Drugs	Ever stocked?<br>1 = Yes<br>2 = No<br>88 = Don't know<br>99 = Refused to answer	Total number of units available on day of survey	If 0 units available, how many days has this item been out of stock?	Source of payment for item<br>1 = Us, ACT PRIME study<br>2 = NGO/project<br>3 = PHC Fund<br>4 = Government<br>77 = Other, list<br>88 = Don't know<br>99 = Refused to answer
1.	Artemether-Lumefantrine – Coartem	[_____|_____]		days	[_____|_____] ____________________
2.	Artemether-Lumefantrine – Lumartem	[_____|_____]		days	[_____|_____] ____________________
3.	Amodiaquine-Artesunate	[_____|_____]		days	[_____|_____] ____________________
4.	Sulfadoxine-Pyrimethamine (Fansidar)	[_____|_____]		days	[_____|_____] ____________________
5.	Chloroquine 150 base tab	[_____|_____]		days	[_____|_____] ____________________
6.	Chloroquine 50mg base/5 Bottle 1000ml Syrup	[_____|_____]		days	[_____|_____] ____________________
7.	Chloroquine 50mg base/5 Bottle 1000ml Syrup	[_____|_____]		days	[_____|_____] ____________________
8.	Chloroquine inj 40 mg/ml; 39ml	[_____|_____]		days	[_____|_____] ____________________
9.	Acetylsalicylic acid 300mg tab	[_____|_____]		days	[_____|_____] ____________________
10.	Diazepam inj 5mg/ml	[_____|_____]		days	[_____|_____] ____________________
11.	Ferrous sulphate tab. 200 + 0.25mg	[_____|_____]		days	[_____|_____] ____________________
12.	Folic Acid 5mg	[_____|_____]		days	[_____|_____] ____________________

HEALTH FACILITY SURVEILLANCE FORM – COST COLLECTION (2)

Health centre code	Health worker ID	Date Report Completed
[_____|_____]	[_____|_____]	[_____|_____]/[_____|_____]/[_____|_____]<br>day month year

#	DRUGS	Ever stocked?<br>1 = Yes<br>2 = No<br>88 = Don't know<br>99 = Refused to answer	Total number of units available on day of survey	If 0 units available, how many days has this item been out of stock?	Source of payment for item<br>1 = Us, ACT PRIME study<br>2 = NGO/project<br>3 = PHC Fund<br>4 = Government<br>77 = Other, list<br>88 = Don't know<br>99 = Refused to answer
13.	Oral Rehydration Salts	[_____|_____]		days	[_____|_____] ____________________
14.	Paracetamol 120/5ml syrup	[_____|_____]		days	[_____|_____] ____________________
15.	Paracetamol tab 500 mg	[_____|_____]		days	[_____|_____] ____________________
16.	Quinine inj 300mg/ml	[_____|_____]		days	[_____|_____] ____________________
17.	Quinine tab 300mg	[_____|_____]		days	[_____|_____] ____________________
18.	Dextrose 5%	[_____|_____]		days	[_____|_____] ____________________
19.	Dextrose 50%	[_____|_____]		days	[_____|_____] ____________________
20.	Water for injection 10m	[_____|_____]		days	[_____|_____] ____________________
21.	Other ______________________________	[_____|_____]		days	[_____|_____] ____________________
22.	Other ______________________________	[_____|_____]		days	[_____|_____] ____________________
23.	Other ______________________________	[_____|_____]		days	[_____|_____] ____________________
24.	Other ______________________________	[_____|_____]		days	[_____|_____] ____________________
25.	Other ______________________________	[_____|_____]		days	[_____|_____] ____________________

HEALTH FACILITY SURVEILLANCE FORM – COST COLLECTION (3)

Health centre code	Health worker ID	Date Report Completed
[_____|_____]	[_____|_____]	[_____|_____]/[_____|_____]/[_____|_____]<br>day month year

#	SUPPLIES	Ever stocked?<br>1 = Yes<br>2 = No<br>88 = Don't know<br>99 = Refused to answer	Total number of units available on day of survey	If 0 units available, how many days has this item been out of stock?	Source of payment for item<br>1 = Us, ACT PRIME study<br>2 = NGO/project<br>3 = PHC Fund<br>4 = Government<br>77 = Other, list<br>88 = Don't know<br>99 = Refused to answer
26.	Syringe for needles	[_____|_____]		days	[_____|_____] ____________________
27.	Cotton Wool	[_____|_____]		days	[_____|_____] ____________________
28.	Plaster wound adhesive	[_____|_____]		days	[_____|_____] ____________________
29.	Surgical gloves	[_____|_____]		days	[_____|_____] ____________________
30.	Giving set	[_____|_____]		days	[_____|_____] ____________________
31.	Cannula	[_____|_____]		days	[_____|_____] ____________________
32.	Needle half circle 16 triangular	[_____|_____]		days	[_____|_____] ____________________
33.	Other ______________________________	[_____|_____]		days	[_____|_____] ____________________
34.	Other ______________________________	[_____|_____]		days	[_____|_____] ____________________
35.	Other ______________________________	[_____|_____]		days	[_____|_____] ____________________
36.	Other ______________________________	[_____|_____]		days	[_____|_____] ____________________
37.	Other ______________________________	[_____|_____]		days	[_____|_____] ____________________
38.	Other ______________________________	[_____|_____]		days	[_____|_____] ____________________
39.	Other ______________________________	[_____|_____]		days	[_____|_____] ____________________
40.	Other ______________________________	[_____|_____]		days	[_____|_____] ____________________

HEALTH FACILITY SURVEILLANCE FORM – COST COLLECTION (4)

Health centre code	Health worker ID	Date Report Completed
[____|____]	[____|____]	[____|____]/[____|____]/[____|____]<br>day month year

Section 3: OVERHEADS

	UTILITIES	Does this health facility have this utility?<br>1 = Yes 2 = No<br>88 = Don't know<br>99 = Refused to answer	Was this utility paid for this year?<br>1 = Yes<br>2 = No<br>88 = Don't know<br>99 = Refused to answer	Average price per month, in UGX	Average total price per year, in UGX	Source of payment for utility<br>1 = NGO<br>2 = PHC Fund<br>3 = Government<br>77 = Other, list<br>88 = Don't know<br>99 = Refused to answer
41.	Gas	[___|___]	[___|___]	UGX	UGX	[___|___] ____________________
42.	Electricity	[___|___]	[___|___]	UGX	UGX	[___|___] ____________________
43.	Water	[___|___]	[___|___]	UGX	UGX	[___|___] ____________________
44.	Other ______________	[___|___]	[___|___]	UGX	UGX	[___|___] ____________________
45.	Other ______________	[___|___]	[___|___]	UGX	UGX	[___|___] ____________________

HEALTH FACILITY SURVEILLANCE FORM – COST COLLECTION (5)

Health centre code	Health worker ID	Date Report Completed
[_____|_____]	[_____|_____]	[_____|_____]/[_____|_____]/[_____|_____]<br>day month year

	CAPITAL EQUIPMENT<br>Vehicles, furniture, generator,	Description<br>Make/model	# of units available	Date purchased<br>[Day/month/year]	Cost, in UGX	Source of payment for item<br>1 = NGO<br>2 = PHC Fund<br>3 = Government, list department<br>77 = Other, list<br>88 = Don't know<br>99 = Refused to answer	If this item is used in the treatment of malaria, indicate the % of time in a week the item is used to treat malaria.
46.				[__ __/__ __/__ __]	UGX	[___|___] _______________	%
47.				[__ __/__ __/__ __]	UGX	[___|___] _______________	%
48.				[__ __/__ __/__ __]	UGX	[___|___] _______________	%
49.				[__ __/__ __/__ __]	UGX	[___|___] _______________	%
50.				[__ __/__ __/__ __]	UGX	[___|___] _______________	%
51.				[__ __/__ __/__ __]	UGX	[___|___] _______________	%
52.				[__ __/__ __/__ __]	UGX	[___|___] _______________	%
53.				[__ __/__ __/__ __]	UGX	[___|___] _______________	%
54.				[__ __/__ __/__ __]	UGX	[___|___] _______________	%
55.				[__ __/__ __/__ __]	UGX	[___|___] _______________	%
56.				[__ __/__ __/__ __]	UGX	[___|___] _______________	%
57.				[__ __/__ __/__ __]	UGX	[___|___] _______________	%
58.				[__ __/__ __/__ __]	UGX	[___|___] _______________	%
59.				[__ __/__ __/__ __]	UGX	[___|___] _______________	%
60.				[__ __/__ __/__ __]	UGX	[___|___] _______________	%

HEALTH FACILITY SURVEILLANCE FORM – COST COLLECTION (6)

Health centre code	Health worker ID	Date Report Completed
[____|____]	[____|____]	[____|____]/[____|____]/[____|____]<br>day month year

	SPACE - Description<br>1 = waiting area; 2 = registration; 3 = examination room; 4 = dispensing room; 5 = laboratory; 6 = kitchen; 7 = laundry; 8 = pharmacy; 9 = storage; 10 = corridor; 11 = not in use; 77 = other, list	Size, in square meters	Price per square meter, in UGX	Source of payment for space<br>1 = NGO; 2 = PHC Fund; 3 = Government, list department; 77 = Other, list; 88 = Don't know; 99 = Refused to answer	If this space is used in the treatment of malaria, indicate the % of time in a week this space is used to treat malaria.
61.	[___|___] ____________	m^2	UGX/m^2	[___|___] ____________	%
62.	[___|___] ____________	m^2	UGX/m^2	[___|___] ____________	%
63.	[___|___] ____________	m^2	UGX/m^2	[___|___] ____________	%
64.	[___|___] ____________	m^2	UGX/m^2	[___|___] ____________	%
65.	[___|___] ____________	m^2	UGX/m^2	[___|___] ____________	%
66.	[___|___] ____________	m^2	UGX/m^2	[___|___] ____________	%
67.	[___|___] ____________	m^2	UGX/m^2	[___|___] ____________	%
68.	[___|___] ____________	m^2	UGX/m^2	[___|___] ____________	%
69.	[___|___] ____________	m^2	UGX/m^2	[___|___] ____________	%
70.	[___|___] ____________	m^2	UGX/m^2	[___|___] ____________	%

HEALTH FACILITY SURVEILLANCE FORM – COST COLLECTION (7)

Health centre code	Health worker ID	Date Report Completed
[_____|_____]	[_____|_____]	[_____|_____]/[_____|_____]/[_____|_____]<br>day month year

STAFF ALLOCATION & ACTIVITIES

List all of the staff people present at the health facility in an average week and then percentage of time in an average week they spend on each activity. Indicate 0, if no time is spent on an activity by a staff person. Repeat for each staff person.

1 = In-charge, 2 = Senior medical officer, 3 = Medical officer, 4 = Senior clinical officer, 5 = Clinical officer, 6 = Nursing officer, 7 = Enrolled nurse, 8 = Midwife, 9 = Public health nurse, 10 = Nursing aide/assistant, 11 = Laboratory technician, 12 = Laboratory assistant, 13 = Health assistant, 14 = Health educator, 15 = Other_____________

	Staff person 1: [___|___]	Staff person 2: [___|___]	Staff person 3: [___|___]	Staff person 4: [___|___]	Staff person 5: [___|___]	Staff person 6: [___|___]	Staff person 7: [___|___]	Staff person 8: [___|___]
Overhead activities								
Administration	%	%	%	%	%	%	%	%
Accounting	%	%	%	%	%	%	%	%
Compiling health information	%	%	%	%	%	%	%	%
Cleaning health centre	%	%	%	%	%	%	%	%
Laundry	%	%	%	%	%	%	%	%
Other ___________________	%	%	%	%	%	%	%	%
Other ___________________	%	%	%	%	%	%	%	%
Support activities								
Dispensary activities	%	%	%	%	%	%	%	%
Diagnostic services	%	%	%	%	%	%	%	%
Other ___________________	%	%	%	%	%	%	%	%
Other ___________________	%	%	%	%	%	%	%	%
Service activities								
Out-patient - Malaria	%	%	%	%	%	%	%	%
Out-patient - Other illnesses	%	%	%	%	%	%	%	%
Antenatal visits	%	%	%	%	%	%	%	%
Postnatal care	%	%	%	%	%	%	%	%
Vaccination	%	%	%	%	%	%	%	%
Family planning	%	%	%	%	%	%	%	%
Maternity care	%	%	%	%	%	%	%	%
Other ___________________	%	%	%	%	%	%	%	%
Other ___________________	%	%	%	%	%	%	%	%

APPENDIX CC. INFORMATION SHEET
Health worker knowledge questionnaire
ACT PRIME Study

Introduction

Dr. Sarah Staedke and colleagues from the Uganda Malaria Surveillance Project / Infectious Diseases Research Collaboration are investigating the delivery of health care services in Tororo District. We are doing a research study to see if we can improve the health of children in this area by improving services at government-run health facilities. Certain health centers in Tororo district will be selected to either take part in the intervention to improve services, or to continue with their current services. Assignment to the two groups has been determined by a lottery. The chance of being placed into either of the groups is the same.

Why is this study being done?

As part of this study, we would like to assess health workers' understanding of febrile illnesses, particularly malaria, including how it is transmitted, common symptoms, and approaches to management and treatment. We plan to carry out these surveys once a year for 2 years.

What will happen if I take part in this study?

Today, we would like to ask you some questions about fever and malaria using a questionnaire. We will take notes of the discussion. All information gathered will be treated as confidential by the study personnel, and records of the interviews will be kept securely in locked filing cabinets and offices. No personal identification information such as names will be used in any reports arising out of this research.

How long will the interview last?

Today, the interview will last about 30 minutes.

Can I stop being in the study?

You can decide to stop participating at any time. Just tell our study personnel right away if you wish to stop the activities.

What risks can I expect from participating in the study?

Participation in any research study may involve a loss of privacy. Information you provide will be recorded, but your name will not be used in any reports of the information provided. The information obtained from these study activities will only be used by the project researchers and will be locked at our project offices. We will do our best to make sure that any personal information is kept private.

Are there benefits to taking part in the study?

There will be no direct benefit to you from participating in this study. However, the information that you provide will help researchers and policy-makers understand how best to improve health services in this area.

What other choices do I have if I do not take part in the study?

You are free to choose not to take part in the study. If you decide not to take part, there will be no penalty to you.

What are the costs of taking part in the study? Will I be paid for taking part in the study?

There are no costs to you for taking part in this study. You will not be paid for taking part in this study.

What are my rights if I take part in the study?

Taking part in this study is your choice. You may choose either to take part or not to take part. If you decide to take part in this study, you may change your mind at any time. No matter what decision you take, there will be no penalty to you in any way.

Who can answer my questions about the study?

You can talk to the researchers about any questions or concerns you have about these study activities. Contact Dr. Sarah Staedke or other members of the Uganda Malaria Surveillance Project / Infectious Disease Research Collaboration on telephone number 0414-530692. If you have any questions, comments or concerns about taking part in these activities, first talk to the researchers. If for any reason you do not wish to do this, or you still have concerns about doing so, you may contact Dr Charles Ibingira, Makerere University Faculty of Medicine Research and Ethical Committee at telephone number 0414-530020.

Giving verbal consent to take part in the study:

You may keep this information sheet if you wish. Participation in these activities is voluntary. You have the right to decline to take part in the activities, or to withdraw from them at any point without penalty. If you do not wish to take part in the activities, you should inform the researcher now. If you do wish to take part in these activities, you should tell the researcher now, and the interview will begin shortly.

APPENDIX DD: HEALTH WORKER KNOWLEDGE QUESTIONNAIRE (1)

Date: [____|____] / [____|____] / [____|____]<br>day month year		Health worker position:<br>1 = In-charge<br>2 = Senior medical officer<br>3 = Medical officer<br>4 = Senior clinical officer<br>5 = Clinical officer<br>6 = Nursing officer<br>7 = Enrolled nurse<br>8 = Midwife<br>9 = Public health nurse<br>10 = Nursing aide/assistant<br>11 = Laboratory technician<br>12 = Laboratory assistant<br>13 = Health assistant<br>14 = Health educator<br>15 = Volunteer<br>16 = Other____________________<br>[____|____]
Health center code<br>[_____|_____]	HCW Study ID<br>[_____|_____]	

SECTION 1<br>Administer questionnaire by asking the questions below, without prompting for answers. Record all of the health worker's answers below during the interview.	SECTION 2<br>After the interview is finished, complete the questions in section 2	SECTION 3<br>Finally, score the questionnaire here in section 3	
1. "What is malaria?"<br>Record answer	Did the respondent's answer include: 1 = Yes 2 = No<br>[____] Fever or high temperature or hot body<br>[____] Parasites	Give 5 points for each Yes answer in SECTION 2.	[____|____]
2. "How does someone get malaria?"<br>Record answer	Did the respondent's answer include: 1 = Yes 2 = No<br>[____] Bite of a mosquito<br>[____] Female mosquito<br>[____] Anopheles mosquito	Give 5 points for each Yes answer in SECTION 2.	[____|____]
3. "What is the most common symptom of malaria in children?"<br>Record answer	Did the respondent's answer include: 1 = Yes 2 = No<br>[____] Fever or high temperature or hot body	Give 10 points for a Yes answer in SECTION 2.	[____|____]

HEALTH WORKER KNOWLEDGE QUESTIONNAIRE (2)

Date: [____|____] / [____|____] / [____|____]<br>day month year	Health center code: [____|____]	HCW Study ID: [____|____]

SECTION 1<br>Administer questionnaire by asking the questions below, without prompting for answers. Record all of the health worker's answers below during the interview.	SECTION 2<br>After the interview is finished, complete the questions in section 2	SECTION 3<br>Finally, score the questionnaire here in section 3	
4. **"**What are danger signs of severe malaria in children?**"**<br>Record answer	Did the respondent's answer include: 1 = Yes 2 = No<br>[____] Convulsions<br>[____] Altered consciousness (confusion, coma)<br>[____] Lethargy (prostration or extreme weakness)<br>[____] Unable to drink or breastfeed<br>[____] Vomiting everything<br>[____] Unable to sit/stand due to weakness<br>[____] Severe anemia<br>[____] Respiratory distress (labored breathing)<br>[____] Jaundice (yellow coloring of eyes)	Give 2 points for each Yes answer in SECTION 2.	[____|____]
5. **"**When you examine a child that you suspect has uncomplicated malaria, what signs do you look out for?"<br>Record answer	Did the respondent's answer include: 1 = Yes 2 = No<br>[____] Fever or high temperature or hot body<br>[____] Evidence of anemia (pallor)<br>[____] Enlargement of spleen	Give 5 points for each Yes answer in SECTION 2.	[____|____]
6. **"**When you examine a child that you suspect has severe malaria, what signs do you look out for?"<br>Record answer	Did the respondent's answer include: 1 = Yes 2 = No<br>[____] Mental status or level of consciousness<br>[____] Evidence of severe anemia (pallor)<br>[____] Dehydration	Give 5 points for each Yes answer in SECTION 2.	[____|____]

HEALTH WORKER KNOWLEDGE QUESTIONNAIRE (3)

Date: [____|____] / [____|____] / [____|____]<br>day month year	Health center code: [____|____]	HCW Study ID: [____|____]

SECTION 1<br>Administer questionnaire by asking the questions below, without prompting for answers. Record the health worker's answers below during the interview.	SECTION 2<br>After the interview is finished, complete the questions in section 2	SECTION 3<br>Finally, score the questionnaire here in section 3	
7. "What illnesses, other than malaria, commonly cause fever in children?"<br>Record answer	Did the respondent's answer include: 1 = Yes 2 = No<br>[____] Viral infection (common cold, HIV/AIDS, etc)<br>[____] Bacterial infection<br>[____] Measles<br>[____] Ear infection (otitis media or externa)<br>[____] Throat infection (pharyngitis, tonsillitis)<br>[____] Respiratory tract infection (pneumonia)<br>[____] Urinary tract infection<br>[____] Skin infection (abscess)<br>[____] Typhoid fever<br>[____] Brucellosis	Give 2 points for each Yes answer in SECTION 2.	[____|____]
8. "How can the diagnosis of malaria be confirmed?"<br>Record answer	Did the respondent's answer include: 1 = Yes 2 = No<br>[____] Positive blood slide for malaria parasites<br>[____] Positive rapid diagnostic test	Give 5 points for each Yes answer in SECTION 2.	[____|____]
9. "How should a child with uncomplicated malaria be treated?"<br>Record answer	Did the respondent's answer include: 1 = Yes 2 = No<br>[____] Coartem (artemether-lumefantrine)<br>[____] Dose based on child's age or weight<br>[____] Give Coartem tablets twice a day for 3 days	Give 5 points for each Yes answer in SECTION 2.	[____|____]

HEALTH WORKER KNOWLEDGE QUESTIONNAIRE (4)

Date: [____|____] / [____|____] / [____|____] (day, month, year)	Health center code: [____|____]	HCW Study ID: [____|____]

SECTION 1<br>Administer questionnaire by asking the questions below, without prompting for answers. Record the health worker's answers below during the interview.	SECTION 2<br>After the interview is finished, complete the questions in section 2	SECTION 3<br>Finally, score the questionnaire here in section 3	
10. **"**What information should be given to the primary caregiver as part of the counseling when administering treatment for uncomplicated malaria?"<br>Record answer	Did the respondent's answer include: 1 = Yes 2 = No<br>[____] Give Coartem tablets twice a day for 3 days<br>[____] Give Coartem with milk or meal if possible<br>[____] What to do if child does not improve<br>[____] Continue supportive treatment	Give 5 points for each Yes answer in SECTION 2.	[____|____]
11. **"**If Coartem is not available, what other antimalarials can be used to treat uncomplicated malaria?**"**<br>Record answer	Did the respondent's answer include: 1 = Yes 2 = No<br>[____] Amodiaquine + artesunate	Give 10 points for a Yes answer in SECTION 2.	[____|____]
12. **"**What medical treatment should be given to a child with severe malaria?"<br>Record answer	Did the respondent's answer include: 1 = Yes 2 = No<br>[____] Referral to a health center IV or hospital<br>[____] Treatment with quinine<br>[____] Quinine IV 10mg/kg every 8 hours<br>[____] Give quinine for total of 7 days	Give 5 points for each Yes answer in SECTION 2.	[____|____]

TOTAL SCORE [_____|_____|_____]

Appendix: WHO TOXICITY GRADING SCALE FOR DETERMINING THE SEVERITY OF ADVERSE EVENTS

WHO Toxicity Grading Scale for Determining The Severity of Adverse Events

**HEMATOLOGY**				
ITEM	Grade 1 Toxicity	Grade 2 Toxicity	Grade 3 Toxicity	Grade 4 Toxicity
Hemoglobin	9.5 - 10.5 gm/Dl	8.0 - 9.4 gm/Dl	6.5 - 7.9 gm/Dl	< 6.5 gm/Dl
Absolute Neutrophil Count	1000-1500/mm^3	750-999/mm^3	500-749/mm^3	<500/mm^3
Platelets	75000-99000/mm^3	50000-74999/mm^3	20000-49000/mm^3	<20000/mm^3
Prothrombin Time (PT)	1.01 - 1.25 x ULN	1.26-1.5 x ULN	1.51 -3.0 x ULN	>3 x ULN
Activated Partial Thromboplastin (APPT)	1.01 -1.66 x ULN	1.67 - 2.33 x ULN	2.34 - 3 x ULN	> 3 x ULN
Fibrinogen	0.75 - 0.99 X LLN	0.50 - 0.74 x LLN	0.25 - 0.49 x LLN	< 0.25 x LLN
Fibrin Split Product	20-40 mcg/ml	41-50 mcg/ml	51-60 mcg/ml	> 60 mcg/ml
Methemoglobin	5 - 9.9 %	10.0 - 14.9 %	15.0 - 19.9%	> 20 %
**LIVER ENZYMES**				
AST (SGOT)	1.25 - 2.5 x ULN	2.6 - 5 x ULN	5.1 - 10 x ULN	> 10 x ULN
ALT (SGPT)	1.25 - 2.5 x ULN	2.6 - 5 x ULN	5.1 - 10 x ULN	> 10 x ULN
GGT	1.25 -2.5 x ULN	1.6 - 5 x ULN	5.1 - 10 x ULN	> 10 x ULN
Alkaline Phosphatase	1.25 - 2. 5 x ULN	1.6 - 5 x ULN	5.1 - 10 x ULN	> 10 x ULN
Amylase	1.1 - 1.5 x ULN	1.6 - 2.0 x ULN	2.1 - 5.0 x ULN	> 5.1 x ULN
**CHEMISTRIES**				
Hyponatremia	130-135 mEq/L	123-129 mEq/L	116-122 mEq/L	< 116 or mental status changes or seizures
Hypernatremia	146-150 mEq/L	151-157 mEq/L	158-165 mEq/L	> 165 mEq/L or mental status changes or seizures
Hypokalemia	3.0 - 3.4 mEq/L	2.5 - 2.9 mEq/L	2.0 - 2.4 mEq/L or intensive replacement Rx required or hospitalization required.	< 2.0 mEq/L or paresis or ileus or life-threatening arrhythmia
Hyperkalemia	5.6 - 6.0 mEq/L	6.1 - 6.5 mEq/L	6.6 - 7.0 mEq/l	> 7.0 mEq/L or life-threatening arrhythmia
Hypoglycemia	55-64 mg/dL	40-54 mg/dL	30-39 mg/dL	<30 mg/dL or mental status changes or coma

**CHEMISTRIES (continued)**				
Hyperglycemia (note if fasting)	116 - 160 mg/dL	161- 250 mg/dL	251 - 500 mg/dL	> 500 mg/dL or ketoacidosis or seizures
Hypocalcemia (corrected for albumin)	8.4 - 7.8 mg/dL	7.7 - 7.0 mg/dL	6.9 - 6.1 mg/dL	< 6.1 mg/dL or life threatening arrhythmia or tetany
Hypercalcemia (correct for albumin)	10.6 - 11.5 mg/dL	11.6 - 12.5 mg/dL	12.6 - 13.5 mg/dL	> 13.5 mg/dL life-threatening arrhythmia
Hypomagnesemia	1.4 - 1.2 mEq/L	1.1 - 0.9 mEq/L	0.8 - 0.6 mEq/L	< 0.6 mEq/L or life-threatening arrhythmia
Hypophosphatemia	2.0 - 2.4 mg/dL	1.5 -1.9 mg/dL or replacement Rx required	1.0 -1.4 mg/dL intensive Rx or hospitalization required	< 1.0 mg/dL or life-threatening arrhythmia
Hyperbilirubinemia	1.1 - 1.5 x ULN	1.6 - 2.5 x ULN	2.6 - 5 x ULN	> 5 x ULN
BUN	1.25 - 2.5 x ULN	2.6 - 5 x ULN	5.1 - 10 x ULN	> 10 x ULN
Creatinine	1.1 x 1.5 x ULN	1.6 - 3.0 x ULN	3.1 - 6 x ULN	> 6 x ULN or required dialysis
**URINALYSIS**				
Proteinuria	1+ or < 0.3% or <3g/L or 200 mg - 1 gm loss/day	2 -3 + or 0.3 - 1.0% or 3-10 g/L 1- 2 gm loss/day	4+ or > 1.0% or > 10 g/L 2-3.5 gm loss/day	nephrotic syndrome or > 3.5 gm loss/day
Hematuria	microscopic only	gross, no clots	gross+ clots	obstructive or required transfusion
**CARDIAC DYSFUNCTION**				
Cardiac Rhythm		asymptomatic, transient signs, no Rx required	recurrent/persistent; No Rx required	requires treatment
Hypertension	transient inc. > 20 mm; no Rx	recurrent, chronic, > 20 mm, Rx required	requires acute Rx; No hospitalization	requires hospitalization
Hypotension	transient orthostatic hypotension, No Rx	symptoms correctable with oral fluids Rx	requires IV fluids; no hospitalization required	requires hospitalization
Pericarditis	minimal effusion	mild/moderate asymptomatic effusion, no Rx	symptomatic effusion; pain; EKG changes	tamponade; pericardiocentesis or surgery required
Hemorrhage, Blood Loss	microscopic/occult	mild, no transfusion	gross blood loss; 1-2 units transfused	massive blood loss; > 3 units transfused

Appendix: WHO TOXICITY GRADING SCALE FOR DETERMINING THE SEVERITY OF ADVERSE EVENTS

**RESPIRATORY**				
Cough	transient- no Rx	treatment associated cough<br>local Rx	uncontrolled	
Bronchospasm, Acute	transient; no Rx<br>< 80% - 70% FEV_1<br>(or peak flow)	requires Rx<br>normalizes with bronchodilator;<br>FEV_1 50% - 70%<br>(or peak Flow)	no normalization with bronchodilator;<br>FEV_1 25% - 50%<br>(or peak flow retractions)	cyanosis: FEV_1<br>< 25%<br>(or peak flow) or intubated
**GASTROINTESTINAL**				
Stomatitis	mild discomfort; no limits on activity	some limits on eating/drinking	eating/talking very limited	requires IV fluids
Nausea	mild discomfort; maintains reasonable intake	moderate discomfort; intake decreased significantly; some activity limited	severe discomfort; no significant intake; activities limited	minimal fluid intake
Vomiting	transient emesis	occasional/moderate vomiting	orthostatic hypotension or IV fluids required	hypotensive shock or hospitalization required for IV fluid therapy
Constipation	mild	moderate	severe	distensions w/vomiting
Diarrhea	transient 3-4 loose stools/day	5-7 loose stools/day	orthostatic hypotension or<br>> 7 loose stools/day or required IV fluids	hypotensive shock or hospitalization for IV fluid therapy required
**NEURO & NEUROMUSCULAR**				
Neuro-Cerebellar	slight incoordination dysdiadochokinesis	intention tremor, dysmetria, slurred speech; nystagmus	locomotor ataxia	incapacitated
Mood	mild anxiety or depression	moderate anxiety or depression and therapy required	severe anxiety or depression or mania; needs assistance	acute psychosis; incapacitated, requires hospitalization
Neuro Control<br>(ADL = activities of daily living)	mild difficulty concentrating; no Rx; mild confusion/agitation; ADL unaffected	moderate confusion/agitation some limitation of ADL; minimal Rx	severe confusion/agitation needs assistance for ADL; therapy required	toxic psychosis; hospitalization
Muscle Strength	subjective weakness no objective symptoms/ signs	mild objective signs/symptoms no decrease in function	objective weakness function limited	paralysis

OTHER PARAMETERS				
Fever: oral, > 12 hours	37.7 - 38.5 C or 100.0 - 101.5 F	38.6 - 39.5 C or 101.6 - 102.9 F	39.6 - 40.5 C or 103 - 105 F	> 40 C or > 105 F
Headache	mild, no Rx therapy	transient, moderate; Rx required	severe; responds to initial narcotic therapy	intractable; required repeated narcotic therapy
Fatigue	no decrease in ADL	normal activity decreased 25- 50%	normal activity decreased > 50% can't work	unable to care for self
Allergic Reaction	pruritus without rash	localized urticaria	generalized urticaria; angioedema	anaphylaxis
Local Reaction	tenderness or erythema	induration < 10 cm or phlebitis or inflammation	induration > 10 cm or ulceration	necrosis
Mucocutaneous	erythema; pruritus	diffuse, maculo papular rash, dry desquamation	vesiculation, moist desquamation, or ulceration	exfoliative dermatitis, mucous membrane involvement or erythema, multiforme or suspected Stevens-Johnson or necrosis requiring surgery

APPENDIX FF. Guidelines for grading symptoms, signs and laboratory findings

Table A. Guidelines for grading patient symptoms

	Grade 1<br>MILD	Grade 2<br>MODERATE	Grade 3<br>SEVERE	Grade 4<br>LIFE THREATENING
Subjective fever in the past 24 h	N/A	Present (Yes)	N/A	N/A
Weakness	Mild decrease in activity; For children – weak, but still playing	Moderate decrease in activity; For children – weak, and playing limited	Not participating in usual activities; For children – not playing	Prostration
Muscle and/or joint aches*	Mild and/or localized complaints	Diffuse complaints	Objective weakness; function limited	N/A
Headache*	Mild, no treatment required	Transient, moderate; treatment required	Severe, constant; requires narcotic therapy	Intractable; requires repeated narcotic therapy
Anorexia	Decreased appetite, but still taking solid food	Decreased appetite, avoiding solid food but taking liquids	Appetite very decreased; Refusing to breast feed, no solids or liquids taken (< 2 years ≤ 12 hr; > 2 years ≤ 24 hr)	Appetite very decreased; Refusing to breast feed, no solids or liquids taken (< 2 years > 12 hr; > 2 years > 24 hr)
Nausea*	Mild, transient feeling of impending vomiting; maintains reasonable intake	Moderate and/or constant feeling of impending vomiting; intake decreased	Severe, constant feeling of impending emesis; intake decreased significantly	N/A
Vomiting	1 episode per day	2-3 episodes per day	Orthostatic hypotension or IV fluids required	Hypotensive shock or nrolment ation required for IV fluid therapy
Abdominal pain*	Mild (1-3 on a scale of 1 to 10)	Moderate (4-6 on a scale of 1 to 10)	Moderate to severe (≥ 7 on a scale of 1 to 10)	Severe – nrolment at for treatment
Diarrhea	Transient 3-4 loose stools/day	5-7 loose stools/day	Orthostatic hypotension or > 7 loose stools/day or IV fluids required	Hypotensive shock or nrolment ation for IV fluid therapy required
Cough	Transient / intermittent	Persistent / constant	Uncontrolled	Cyanosis, stridor, severe shortness of breath
Pruritis	Transient pruritis	Pruritis that disturbs sleep	Severe, constant pruritis, sleep disturbed	N/A
Tinnitus*	Mild, transient ringing or roaring sound	Moderate, persistent ringing or roaring sound	Severe ringing or roaring sound with associated hearing loss	N/A
Behavioural changes	Mild difficulty concentrating; mild confusion or agitation; activities of daily living unaffected; no treatment	Moderate confusion or agitation; some limitation of activities of daily living; minimal treatment	Severe confusion or agitation; Needs assistance for activities of daily living; therapy required	Toxic psychosis; nrolment ation required
"Flu" (viral URI)	Mild nasal congestion, mild rhinorrhea	Moderate nasal congestion, moderate rhinorrhea	N/A	N/A
Allergic reaction	N/A	N/A	Urticaria	Severe urticaria anaphylaxis, angioedema
Convulsion	N/A	N/A	Localized or generalized seizure	Status epilepticus

* Assess only in children ≥ 3 years of age. Answer N/A for younger children and those unable to answer.

Table B. Guidelines for physical examination

Dehydration	Assess skin touch and turgor, mucous membranes, eyes, crying, fontanelle, pulse, urine output
Jaundice	Assess for yellowing of the sclera. Also evaluate the palpepral conjunctiva, lips, and skin.
Chest	Observe the rate, rhythm, depth, and effort of breathing. Check the patient's colour for cyanosis.<br><br>The maximum acceptable respiratory rate by age: < 2 months = 60, 2-12 months = 50, 1-5 years = 40, above 5 years = 30.<br><br>Inspect the neck for the position of the trachea, for supraclavicular retractions, and for contraction of the sternomastoid or other accessory muscles during inspiration.<br><br>Auscultate the anterior and posterior chest for normal breath sounds and any adventitious sounds (crackles or rales, wheezes, and rhonchi). Crackles are intermittent, non-musical, fine or coarse sounds that may be due to abnormalities of the lungs (pneumonia, fibrosis, early congestive heart failure) or airways (bronchitis or bronchiectasis). Wheezes are high-pitched and result from narrowed airways. Rhonchi are relatively low-pitched and suggest secretions in large airways.<br><br>If abnormalities are identified, evaluate for transmitted voice sounds. In addition, palpate the chest to assess for tactile fremitus, and percuss the chest to assess for areas of dullness. Normal, air-filled lungs emit predominantly vesicular breath sounds, transmit voice sounds poorly with "ee" = "ee", and have no tactile fremitus. Airless lung, as in lobar pneumonia, emits bronchial breath sounds, transmits spoken words clearly with "ee" = "aay" (egophany), and has an increase in tactile fremitus.
Abdomen	Inspect and ausculate the abdomen. Listen for bowel sounds in the abdomen before palpating it. Palpate the abdomen in all 4 quadrants lightly and then deeply. Assess the size of the liver and spleen. To assess for peritoneal inflammation, look for localised and rebound tenderness, and voluntary or involuntary rigidity.
Skin	Inspect the skin for colour, turgor, moisture, and lesions. If lesions are present, note their location and distribution (diffuse or localised), arrangement (linear, clustered, annular, dermatomal), type (macules, papules, vesicles) and colour.
Tablet test	For children $\geq$ 9 months of age, ask the patient to pick a tablet (or equivalent object) up off a flat surface using the thumb and index finger of their dominant hand. This tests for co-ordination of the upper extremity assessing the function of the motor system, cerebellar system, vestibular system (for coordinating eye and body movements) and the sensory system, for position sense. When testing small children, be aware that they will likely attempt to put the object into their mouth.

Table C. Grading physical examination findings

	Grade 1<br>MILD	Grade 2<br>MODERATE	Grade 3<br>SEVERE	Grade 4<br>LIFE-THREATENING
Temperature*<br>(axillary)	37.5-37.9°C	38.0-39.5°C	> 39.5°C	Sustained fever, equal or greater than 40.0°C for longer than 5 days
Dehydration	Less than 2 of the following:<br>Restless, irritable<br>Sunken eyes<br>Drinks eagerly, thirsty<br>Skin pinch goes back slowly	2 of the following:<br>Restless, irritable<br>Sunken eyes<br>Drinks eagerly, thirsty<br>Skin pinch goes back slowly	Two of the following:<br>Lethargic or unconscious<br>Sunken eyes<br>Not able to drink or drinking poorly<br>Skin pinch goes back very poorly	Two of the following + shock:<br>Lethargic or unconscious<br>Sunken eyes<br>Not able to drink or drinking poorly<br>Skin pinch goes back very poorly
Jaundice	Slight yellowing of sclera and conjunctiva	Moderate yellowing of sclera and conjunctiva, yellowing of mucous membranes	Severe yellowing of sclera and conjunctiva, yellowing of skin	N/A
Chest	Mildly increased RR (for age, temperature), transient or localised adventitious sounds	Moderately increased RR, diffuse or persistent adventitious sounds	Rapid RR (< 2 months > 60, 2-12 months > 50, 1-5 years > 40, adults > 30)* nasal flaring, retractions	Cyanosis
Abdomen	Normal bowel sounds, mild localised tenderness, and/or liver palpable 2-4 cm below the right costal margin (RCM), and/or spleen palpable, and/or umbilical hernia present	Normal or mildly abnormal bowel sounds, moderate or diffuse tenderness; and/or mild to moderately enlarged liver (4-6 cm below the RCM) and/or spleen palpable up to half-way between umbilicus and symphysis pubis	Severely abnormal bowel sounds, severe tenderness to palpation. Evidence of peritoneal irritation and/or significant enlargement of liver (> 6 cm below the RCM) and/or spleen palpable beyond half-way between umbilicus and symphysis pubis	Absent bowel sounds. Involuntary rigidity
Skin†	Localised rash, erythema, or pruritis	Diffuse, maculopapular rash, dry desquamation	Vesiculation, moist desquamation, or ulceration	Exfoliative dermatitis, mucous membrane involvement or erythema multiforme or suspected Stevens-Johnson or necrosis requiring surgery

Table C. Grading physical examination findings (continued)

	Grade 1<br>MILD	Grade 2<br>MODERATE	Grade 3<br>SEVERE	Grade 4<br>LIFE-THREATENING
Hearing	< 4 years: N/A<br>≥ 4 years: Decreased hearing in one ear	< 4 years: N/A<br>≥ 4 years: Decreased hearing in both ears or severe impairment in one ear	< 4 years: Any evidence of hearing impairment<br>≥ 4 years: Severe impairment in both ears	N/A
Tablet test	Difficulty grasping tablet but able to pick up	Unable to pick up tablet without dropping	Unable to grasp tablet	N/A
Clinical symptoms / sign (not otherwise specified)	No treatment required; monitor condition	Treatment required	Requires treatment and possible hospitalisation	Requires active medical intervention, hospitalisation, or hospice care

- Reference – The Harriet Lane Handbook, 15th edition, 2000

† Reference – WHO Toxicity Grading Scale for Determining the Severity of Adverse Events

TABLE D. Guidelines for grading of laboratory abnormalities

	Grade 1<br>MILD	Grade 2<br>MODERATE	Grade 3<br>SEVERE	Grade 4<br>LIFE-THREATENING
Haemoglobin (g/dL)	9.0 – 9.9	7.0 – 8.9	5.0 – 6.9	< 5.0

Reference – The Harriet Lane Handbook, 15th edition, 2000†
Reference – WHO Toxicity Grading Scale for Determining the Severity of Adverse Events

APPENDIX GG: SERIOUS ADVERSE EVENT FORM – INITIAL REPORT (1)

Study ID	Date of SAE Onset	Date of SAE Report
[____|____|____|____]	[___|___]/ [___|___]/[___|___]<br>day month year	[___|___]/ [___|___]/[___|___]<br>day month year

SAE number	Gender	Age	Weight
[____|____]	[____] 1 = Male<br>2 = Female	[____|____] [____|____]<br>years months (only if < 1 year)	[___|____] kg

SECTION 1: EVENT INFORMATION

Event description:____
(symptom, sign, or laboratory abnormality)

Indicate reason for serious AE: (list all that apply): 1 = Yes 2 = No

Death	[____]
Life-threatening	[____]
Resulted in or prolonged hospitalization	[____]
Required medical or surgical intervention	[____]
Resulted in significant or persistent disability	[____]
Other ____________________________	[____]

Maximum severity (WHO scale)	1 = Mild<br>2 = Moderate<br>3 = Severe<br>4 = Life-threatening	[____]
Maximum severity (Uganda scale)	1 = Mild<br>2 = Moderate<br>3 = Severe<br>4 = Life-threatening	[____]
Was the event expected?	1 = Yes<br>2 = No	[____]

SECTION 2: EVENT SUMMARY

EVENT SUMMARY (include details of event, associated signs and symptoms, possible alternative etiologies, relevant past medical history, and medical management):

____
____
____
____
____
____
____
____

Study product name: ARTEMETHER-LUMEFANTRINE	Route of study product: ORAL

Dosing schedule at SAE onset:
1 = 1 tab, 2x per day, for 3 days
2 = 1 tabs, 2x per day, for 3 days
3 = 3 tabs, 2x per day, for 3 days
4 = 4 tabs, 2x per day, for 3 days
77 = Other __________________________
88 = I don't know
[___\|____]

Date study product first started:	Date study product last taken prior to onset of SAE:
[____|____]/[____|____]/[____|____]<br>day month year	[____|____]/[____|____]/[____|____]<br>day month year

Relationship to study product:	If not associated, is event related to:
0 = None<br>1 = Unlikely<br>2 = Possible<br>3 = Probable<br>4 = Definite<br>[____]	1 = Study procedure<br>2 = Other condition or illness<br>3 = Other medication<br>4 = Other (specify)<br>________________________<br>[____]

**SERIOUS ADVERSE EVENT FORM – INITIAL REPORT (2)**	Study ID<br>[____|____|____|____]	Date of SAE Report<br>[___|___]/ [___|___]/ [___|___]<br>day month year

Study product status: (list all that apply) 1 = Yes 2 = No		Patient management: (list all that apply) 1 = Yes 2 = No	
No change in dose	[_____]	Patient hospitalized	[_____]
Study treatment held	[_____]	Blood transfusion given	[_____]
Study treatment discontinued permanently	[_____]	Intravenous fluids given	[_____]
Other_______________________________	[_____]	Parenteral quinine given	[_____]
		Other_________________________________	[_____]
		Other_________________________________	[_____]
		Other_________________________________	[_____]

RELEVANT LABORATORY TESTS

Test	Collection date [dd/mm/yy]	Result	Site normal range	Most recent value prior to SAE	Collection date [dd/mm/yy]
	[/ /]				[/ /]
	[/ /]				[/ /]
	[/ /]				[/ /]

RELEVANT DIAGNOSTIC TESTS

Test	Collection date [dd/mm/yy]	Results/Comments
	[/ /]	
	[/ /]	
	[/ /]	

CONCOMITANT MEDICATIONS

(List relevant concomitant medications the subject was taking up to 1 month prior to SAE onset.)

Medication	Start date [dd/mm/yy]	Stop date [dd/mm/yy]	Total daily dose	Indication	Suspect for SAE 1 = Yes 2 = No
	[/ /]	[/ /]			[___]
	[/ /]	[/ /]			[___]
	[/ /]	[/ /]			[___]
	[/ /]	[/ /]			[___]
	[/ /]	[/ /]			[___]

Outcome of event	1 = Ongoing<br>2 = Resolved without sequelae<br>3 = Resolved with sequelae<br>4 = Death [_____]	If resolved or died, indicate date:<br>[____|____]/[____|____]/[____|____]<br>day month year
Completed by (printed name):<br>Signature:	Date:<br>[__|__]/[__|__]/[__|__]<br>day month year	Investigator (printed name): Date:<br>Signature: [__|__]/[__|__]/[__|__]<br>day month year

APPENDIX GG: SERIOUS ADVERSE EVENT FORM – FOLLOW-UP REPORT (1)

Study ID	Date of SAE Onset	Date of SAE was first reported
[____|____|____|____]	[___|___]/ [___|___]/[___|___]<br>day month year	[___|___]/ [___|___]/[___|___]<br>day month year

SAE number	Gender	Age	Weight
[____|____]	[____] 1 = Male 2 = Female	[____|____] [____|____]<br>years months (only if < 1 year)	[____|____] kg

Date of follow-up<br>[___|___]/ [___|___]/[___|___]<br>day month year	Temperature<br>[____|____]•[____] ºC
Progress note:	Laboratory results:
Date of follow-up<br>[___|___]/ [___|___]/[___|___]<br>day month year	Temperature<br>[____|____]•[____] ºC
Progress note:	Laboratory results:
Date of follow-up<br>[___|___]/ [___|___]/[___|___]<br>day month year	Temperature<br>[____|____]•[____] ºC
Progress note:	Laboratory results:

Outcome of event 1 = Ongoing<br>2 = Resolved without sequelae<br>3 = Resolved with sequelae<br>4 = Death [_____]	If resolved or died, indicate date:<br>[____|____]/[____|____]/[____|____]<br>day month year
Completed by (printed name): Date:<br>Signature: [__|__]/[__|__]/[__|__]<br>day month year	Investigator (printed name): Date:<br>Signature: [__|__]/[__|__]/[__|__]<br>day month year

APPENDIX HH. ADVERSE DRUG REACTION REPORT FORM	Study ID	Age	Gender	Weight
	[___|___|___|___]	[____|____] years [____|____] months (only if < 1 year)	[____] 1 = Male 2 = Female	[____|____] kg

	Complete on day first reported and update as appropriate								Complete on final day	
Event description	Date of event onset [dd/mm/yy]	Date event reported [dd/mm/yy]	Maximum severity (WHO)*	Maximum severity (Uganda)*	Relationship†	Expected‡	Serious**	Initials	Outcome ††	Date event resolved [dd/mm/yy]
1.										
2.										
3.										
4.										
5.										
6.										
7.										
8.										
9.										
10.										
11.										
12.										

* Severity: Rank on scale of 1-4: 1 = mild; 2 = moderate; 3 = severe; 4 = life threatening
† Relationship: Rank on scale of 0-4: 0 = none; 1 = unlikely; 2 = possible; 3 = probable; 4 = definite
‡ Expectedness: Refer to the Coartem package insert
** Serious: Indicate 1=Yes, 2=No Criteria for SAE: (1) fatal, (2) life threatening, (3) results in or prolongs hospitalization, (4) results in significant or persistent disability or capacity, (5) requires medical / surgical intervention to prevent serious outcome. <u>If serious, report to Kampala core facility staff immediately.</u>
†† Outcome: Rank on scale of 1-5: 1 = resolved without sequelae; 2 = resolved with sequelae; 3 = AE still present at study end/discontinuation, but improving; 4 = subject died; 5 = unknown
