# Supplementary material for: The impact of an intervention to introduce malaria rapid diagnostic tests on fever case management in a high transmission setting in Uganda: A mixed-methods cluster-randomized trial (PRIME)
Source: PLoS One. 2017 Mar 13;12(3):e0170998. doi: 10.1371/journal.pone.0170998 (PMC5347994; doi:10.1371/journal.pone.0170998)
Supplement: S2 File — Final version of the PRIME protocol. This file is a copy of the final study protocol of the PRIME cluster-randomized trial, incorporating all amendments approved by the overseeing ethics committees. (PDF) [file pone.0170998.s002.pdf]

The ACT PRIME Study

Evaluating the impact of enhanced health facility-based care for malaria and febrile illnesses in children in Tororo, Uganda

15 February 2013
Protocol Version 1.7

Principal Investigator: Dr. Sarah Staedke
Co-investigators: Dr. Moses Kamya, Dr. Grant Dorsey, Dr. Clare Chandler

Funding Source: Gates Foundation, ACT Consortium

Institutional Review Boards

- Makerere University Faculty of Medicine Research & Ethical Committee: 2010-108
- Ugandan National Council for Science and Technology: HS 794
- LSHTM Ethics Committee: 5779
- University of California, San Francisco Committee on Human Research: 006160

ACT CONSORTIUM

THE REPUBLIC OF UGANDA

UCSF

CONTENTS

CONTENTS 2

STUDY INFORMATION 6

PROJECT SYNOPSIS 7

PROJECT TEAM AND PARTICIPATING SITES 8
Investigators 8
Collaborators 8
Key project personnel 9
Participating sites 10

ABBREVIATIONS AND ACRONYMS 11

1 BACKGROUND 12
1.1. Introduction 12
1.2. Malaria diagnosis and treatment 12
1.2.1. Barriers to service in the formal healthcare sector 12
1.2.2. Universal diagnostic testing 12
1.2.3. Training health workers in malaria diagnosis 13
1.3. Malaria in Uganda 13
1.3.1. Overview 13
1.3.2. Antimalarial drug policy 14
1.3.3. Affordable Medicines Facility – malaria 14
1.4. Health facility interventions 15
1.4.1. What is quality care and what are the problems? 15
1.4.2. How can quality of care be improved? 15
1.5. Phase I results 17
1.5.1. Census survey 17
1.5.2. Public health facilities 17
1.5.3. Community medicine distributors 18
1.5.4. Qualitative study 18

2 RATIONALE 21
2.1. Problem statement 21
2.2. Study overview 21
2.3. Study population and design 22

3 STUDY OBJECTIVES 23

4 STUDY DESIGN 24
4.1. Overview 24
4.2. Study site 25
4.3. Study population 26
4.4. Cluster Randomisation 26
4.5. Outcome measures 28

5 HEALTH FACILITY INTERVENTION 29
5.1. Overview 29

5.2. Health Center Management 30
5.2.1. Overview 30
5.2.2. Financial management 30
5.2.3. Supply management 30
5.2.4. Health information management 31
5.3. Health worker training 31
5.3.1. Training in fever case management and use of RDTs 31
5.3.2. Training in patient-centered services 32
5.4. Consumables 34
5.4.1. Drug delivery from National Medical Stores 34
5.4.2. Supply artemether-lumefantrine 35
5.4.3. RDTs for malaria 35

6 STANDARD CARE 36
6.1. Overview 36
6.2. Staffing and patient attendance 36
6.3. Infrastructure and supplies 36
6.4. Standard care 36

7 CROSS-SECTIONAL SURVEYS 37
7.1. Overview 37
7.2. Definitions 37
7.3. Enrollment 37
7.3.1. Recruitment 37
7.3.2. Screening 38
7.3.3. Informed Consent 38
7.3.4. Enrollment 38
7.4. Survey procedures 38
7.4.1. Survey questionnaire 38
7.4.2. Clinical and laboratory assessment 39
7.4.3. Management of ill children 39

8 COHORT STUDY 40
8.1. Overview 40
8.2. Enrollment 40
8.2.1. Recruitment 40
8.2.2. Screening 40
8.2.3. Informed Consent 41
8.2.4. Enrollment 41
8.2.5. Management of ill children at enrollment 41
8.3. Household Surveys 41
8.4. Follow-up 42
8.4.1. Household diaries and monthly visits 42
8.4.2. Follow-up evaluations 42
8.4.3. Management of ill children during follow-up 42

9 M&E OF HEALTH FACILITY INTERVENTION 44
9.1. Overview 44
9.2. Patient Exit interviews 44
9.2.1. Overview 44
9.2.2. Recruitment 44
9.2.3. Informed Consent 44
9.2.4. Interview 45

9.2.5. RDT results *45*
9.3. Health Facility Surveillance 46
9.3.1. All facilities *46*
9.3.2. HFI facilities *46*
9.3.3. Out-patient registers *47*
9.4. Health worker knowledge questionnaires 47

10 ADVERSE EVENT MONITORING 48
10.1. Overview 48
10.2. Definitions 48
10.3. Identification of adverse events 48
10.4. Reporting of adverse events 49
10.5. ACT consortium drug safety register 49
10.5.1. Overview *49*
10.5.2. Database *49*
10.5.3. Mechanisms for data handling/sharing: *50*

11 LABORATORY PROCEDURES 51
11.1. Microscopy 51
11.2. Rapid diagnostic tests for malaria 51
11.3. Hemoglobin measurement 51
11.4. Molecular studies 51

12 STATISTICAL ISSUES 53
12.1. Sample size calculations 53
12.1.1. Cluster randomized study *53*
12.1.2. Cohort study *54*
12.1.3. Patient exit interviews at health facilities *54*
12.2. Analytical plan 55
12.2.1. Analytical approach *55*
12.2.2. Baseline characteristics and trial profile *55*
12.2.3. Cross-sectional survey outcomes *55*
12.2.4. Cohort study outcomes *56*
12.2.5. Outcomes measured at health facilities *57*

13 DATA & SAFETY MONITORING BOARD 58
13.1. Overview 58
13.2. Monitoring plan 58
13.3. Stopping guidelines 58

14 DATA MANAGEMENT 59
14.1. Data management 59
14.1.1. Cross-sectional surveys *59*
14.1.2. Cohort study *59*
14.1.3. M&E of health facilities *60*
14.2. quality assurance and quality control 60
14.3. Records and storage 60
14.4. data sharing 61

15 PROTECTION OF HUMAN PARTICIPANTS 62
15.1. Institutional Review Boards 62
15.2. Informed consent process 62
15.3. Confidentiality 62

15.4. Risks and discomforts 63
15.4.1. Privacy 63
15.4.2. Risks of randomization 63
15.4.3. Fingerprick blood draws 63
15.4.4. Risk of artemether-lumefantrine 63
15.4.5. RDTs for malaria 64
15.4.6. Compensation 64
15.4.7. Alternatives 64

16 TIMELINE 65

17 REFERENCES 66

18 APPENDICES 74

STUDY INFORMATION

**Title**	PRIME STUDY – Evaluating the impact of enhanced health facility-based care for malaria and febrile illnesses in children in Tororo, Uganda
**Principal investigator**	Sarah Staedke, MD, PhD<br>Clinical Senior Lecturer, London School of Hygiene and Tropical Medicine<br>Uganda Malaria Surveillance Project / Infectious Diseases Research Collaboration, Uganda
**Co-investigators**	Moses Kamya, MBChB, MPH, PhD; Professor, Makerere University<br>Grant Dorsey, MD, PhD; Associate Professor, University of California, San Francisco<br>Clare Chandler, PhD; Lecturer, London School of Hygiene and Tropical Medicine
**Sponsor**	London School of Hygiene & Tropical Medicine<br>Department of Clinical Research<br>Faculty of Infectious and Tropical Diseases<br>Keppel Street, London WC1E 7HT<br>United Kingdom
**Funding agency**	ACT Consortium grant from the Bill & Melinda Gates Foundation
**Participating institutions**	London School of Hygiene & Tropical Medicine, UK<br>Infectious Diseases Research Collaboration, Kampala, Uganda<br>University of California, San Francisco, USA
**Institutional review boards**	Makerere University Research and Ethics Committee<br>Uganda National Council for Science and Technology<br>London School of Hygiene & Tropical Medicine<br>University of California, San Francisco Committee on Human Research

PROJECT SYNOPSIS

**Title**	PRIME STUDY – Evaluating the impact of enhanced health facility-based care for malaria and febrile illnesses in children in Tororo, Uganda
**Objectives**	1. To compare the impact of enhanced health facility-based care to current standard of care on key population-based indicators, including the prevalence of anemia in children under five.<br>2. To compare the impact of enhanced health facility-based care to current standard of care on key longitudinal indicators, including antimalarial treatment incidence density, in a cohort of children under five.<br>3. To compare impact of enhanced health facility-based care to current standard of care on key indicators of case management for malaria and other illnesses, including the risk of inappropriate antimalarial treatment, in children under five treated at health facilities.
**Description**	Enhanced health facility care will be compared to the current standard of care provided by lower level government-run health facilities, supplemented by services provided through the private sector and community-based interventions, using a cluster-randomized design. Clusters will be defined as households located within a 2 km radius of the facilities.
**Participants and Sample Size**	1. Objective 1. Cross-sectional surveys in children under five and aged 5-15 years randomly selected from households in each cluster; 4383 children per study arm; 8766 total. Surveys will be conducted at baseline and then annually for 2 years. New populations of children will be selected for each survey.<br>2. Objective 2. Cohort of children under five recruited from 25 randomly selected households in each cluster; 250 households per study arm; 500 total.<br>3. Objective 3. Interviews will be conducted with children under five and their caregivers visiting health facilities on days selected for the exit interviews. Three rounds of exit interviews will be conducted. In the first two surveys, 200 patients will be interviewed during each survey (10 patients per health facility). In the final survey, 1000 patients will be interviewed (50 patients per health facility). A total of 1400 patients will be enrolled into the exit interviews.
**Study site**	Tororo district, an area with very high malaria transmission intensity. The five sub-counties of West Budama North Health Sub-district (Nagongera, Paya, Kirewa, Kisoko, and Petta), and two sub-counties of West Budama South Health Sub-district (Mulanda and Rubongi) will be included.
**Study period**	The total duration of the study will be approximately 2.5 years. The health facility intervention will be scaled-up over 2 months and will run for approximately 2 years; the cross-sectional surveys will be conducted at baseline and then annually for 2 years; and each of the cohort study participants will be followed for approximately 18 months in total, 12 months following the roll-out of the intervention.
**Intervention**	20 lower-level government-run health facilities in the area will be randomly assigned to one of two interventions: (1) health facility intervention (HFI), or (2) standard care. The HFI will focus on improving health center management, health worker training, and ensuring adequate diagnostics and drug supplies.
**Primary outcome**	1. Objective 1. Prevalence of anemia<br>2. Objective 2. Antimalarial treatment incidence density<br>3. Objective 3. Inappropriate treatment of malaria
**Secondary outcomes**	1. Objective 1. Prevalence of parasitemia, prevalence of gametocytemia, all-cause mortality rate in children under five<br>2. Objective 2. Incidence of hospitalizations, illness, and febrile illness episodes, prompt effective treatment of fever, prompt effective treatment of malaria, incidence of serious adverse events, antibiotic treatment incidence density<br>3. Objective 3. Appropriate treatment of malaria, patient satisfaction, patient attendance, gaps in staffing, drug stock outs, health worker knowledge questionnaire scores

PROJECT TEAM AND PARTICIPATING SITES

INVESTIGATORS

Sarah Staedke, MD, PhD
Role in project: Principal investigator
Clinical Senior Lecturer, London School of Hygiene and Tropical Medicine, London, UK
Infectious Disease Research Collaboration / Uganda Malaria Surveillance Project, Kampala, Uganda
Email: sarah.staedke@lshtm.ac.uk

Moses Kamya, MBChB, MPH, PhD
Role in project: Co-investigator
Professor, Department of Medicine, Makerere University, Kampala, Uganda
Infectious Disease Research Collaboration / Uganda Malaria Surveillance Project, Kampala, Uganda
Email: mkamya@infocom.co.ug

Grant Dorsey, MD, PhD
Role in project: Co-investigator
Associate Professor, Department of Medicine, University of California, San Francisco
Infectious Disease Research Collaboration / Uganda Malaria Surveillance Project, Kampala, Uganda
Email: gdorsey@medsfgh.ucsf.edu

Clare Chandler, PhD
Role in project: Co-investigator
Senior Lecturer, London School of Hygiene and Tropical Medicine, London, UK
Social Scientist, ACT Consortium
Email: clare.chandler@lshtm.ac.uk

COLLABORATORS

Philip Rosenthal, MD
Role in project: Collaborator
Professor, Department of Medicine, University of California, San Francisco, USA
Infectious Disease Research Collaboration / Uganda Malaria Surveillance Project, Kampala, Uganda
Email: prosenthal@medsfgh.ucsf.edu

Ambrose Talisuna, MBChB, MSc, PhD
Role in project: Collaborator
Medicines for Malaria Venture (MMV) Representative in Africa
Infectious Disease Research Collaboration / Uganda Malaria Surveillance Project, Kampala, Uganda
Email: talisunaa@mmv.org; atalisun@yahoo.com

Fred Wabwire-Mangen, MBChB, MPH, PhD
Role in project: Collaborator
Associate Professor, School of Public Health, Makerere University, Kampala, Uganda
Infectious Disease Research Collaboration / Uganda Malaria Surveillance Project, Kampala, Uganda
Email: fwabwire@musph.ac.ug

Heidi Hopkins, MD, MPH
Role in project: Collaborator
FIND Diagnostics, Kampala, Uganda
Infectious Disease Research Collaboration / Uganda Malaria Surveillance Project, Kampala, Uganda
Email: hhopkins@medsfgh.ucsf.edu; heidi.hopkins@finddiagnostics.org

KEY PROJECT PERSONNEL

Personnel	Institution	Role
Deborah DiLiberto	London School of Hygiene & Tropical Medicine	Research Assistant
Rita Kabuleta-Luswata	Infectious Diseases Research Collaboration, Kampala	Project Officer
Dr. Catherine Maiteki-Sebuguzi	Infectious Diseases Research Collaboration, Kampala	Kampala Study Coordinator
Dr. Florence Nankya	Infectious Diseases Research Collaboration, Kampala	Tororo Study Coordinator
Dr. Samuel Gonahasa	Infectious Diseases Research Collaboration, Kampala	Tororo Study Coordinator
Dr. Edith Mbabazi	Infectious Diseases Research Collaboration, Kampala	Junior Study Coordinator
Dr. Emmanuel Ssemmondo	Infectious Diseases Research Collaboration, Kampala	Junior Study Coordinator
Dr. Damalie Nabukeera	Infectious Diseases Research Collaboration, Kampala	Junior Study Coordinator
Levi Mugenyi	Infectious Diseases Research Collaboration, Kampala	Statistician/Data Manager
Simon Peter Kigozi	Infectious Diseases Research Collaboration, Kampala	Kampala Data Officer
Faith Mukoya	Infectious Diseases Research Collaboration, Kampala	Tororo Data Manager
Eric Ochen	Infectious Diseases Research Collaboration, Kampala	Tororo Data Officer
Dr. Immo Kleinschmidt	London School of Hygiene & Tropical Medicine	Statistician
Dr. Emily Webb	London School of Hygiene & Tropical Medicine	Statistician
Dr. Fred Matovu	London School of Hygiene & Tropical Medicine	Economic Consultant

PARTICIPATING SITES

Uganda Malaria Surveillance Project (UMSP)

Address: Infectious Disease Research Collaboration (IDRC), Mulago Hospital Complex, Malaria House, P.O. Box 7475, Kampala, Uganda
Contact Person: Catherine Tugaineyo
Phone Number: +256 (0) 414-530692
Fax Number: +256 (0) 414-540524
Email: ctugaineyo@muucsf.com

London School of Hygiene & Tropical Medicine (LSHTM)

Address: Keppel Street, London, WC1E 7HT, UK
Contact Person: Susan Sheedy
Phone Number: +44 (0) 20 7927 2256
Fax Number: +44 (0)20 7637 4314
Email: susan.sheedy@lshtm.ac.uk

University of California, San Francisco (UCSF)

Address: San Francisco General Hospital, 1001 Potrero Avenue, Building 30, Room 402/408, San Francisco, CA 94110, USA
Contact Person: Peter Padilla
Phone Number: +1 (415) 206-5438
Fax Number: +1 (415) 648-8425
Email: ppadilla@medsfgh.ucsf.edu

ABBREVIATIONS AND ACRONYMS

ACT	artemisinin-based combination therapy
ADDAT	ACT drug distribution assessment tool
AL	artemether-lumefantrine
AS+AQ	artesunate + amodiaquine
CAB	community advisory board
CBI	community-based intervention
CHW	community health worker
CMD	community medicine distributor
CQ	chloroquine
CQI	continuous quality improvement
FGD	focus group discussion
GIS	geographical information systems
GPS	global positioning system
HBMF	home-based management of fever/malaria
HC	health center
HFI	health facility intervention
ICCM	integrated community case management
IMCI	integrated management of childhood illnesses
JMS	joint medical stores
JUMP	Joint Uganda Malaria Program
IRB	institutional review board
LSHTM	London School of Hygiene and Tropical Medicine
MoH	Ministry of Health
MU	Makerere University (Kampala, Uganda)
MU-UCSF	Makerere University - UCSF Malaria Research Collaboration
M&E	monitoring and evaluation
NMS	national medical stores
PCS	patient-centered services
RDT	rapid diagnostic test
SOP	standard operating procedure
SP	sulfadoxine-pyrimethamine
UCSF	University of California, San Francisco
UMSP	Uganda Malaria Surveillance Project
UNCST	Uganda National Council of Science and Technology
VHT	village health team
WHO	World Health Organization

1 BACKGROUND

1.1. INTRODUCTION

Malaria remains one of the most serious global health problems.[1] Of the estimated 400 to 900 million episodes of fever that occur each year in African children, probably about half are due to malaria, resulting in over one million deaths.[2-4] Uncomplicated malaria can progress rapidly to severe disease, and most malaria deaths in young children occur within 2-3 days of onset of illness.[5] The first few days of illness present an important window of opportunity to reduce morbidity and mortality with early treatment.

Early effective antimalarial treatment is one of the key strategies for reducing the burden of malaria.[6] In the Global Strategic Plan for 2005-2015, the Roll Back Malaria Partnership has set a target to ensure that 80% of malaria episodes are adequately treated within 24 hours of onset of symptoms.[6] Despite increasing availability of effective artemisinin-based combination therapies (ACTs) and the emphasis on early antimalarial treatment, many patients with malaria do not benefit from this therapy. A recent UNICEF report of data collected between 2000 and 2006 estimated that 35% of febrile children in sub-Saharan Africa were treated with antimalarial medicines.[7] However, only 23% of these children were treated within 24 hours of onset of illness, and 60% of febrile children received chloroquine, rather than effective first-line therapies.

1.2. MALARIA DIAGNOSIS AND TREATMENT

1.2.1. Barriers to service in the formal healthcare sector

Diagnosis and treatment of malaria is often challenged by limited health-care infrastructure, particularly in Africa.[8,9] Substantial barriers to providing good quality health care exist, including logistical, cultural, and wider system barriers. As a result, few malaria patients receive treatment in the formal healthcare sector; most are treated at home with drugs purchased from informal drug shops.[2,10] Unfortunately, such treatment is often inadequate, with ineffective or poor quality drugs given at incorrect doses.[10-12] Community-based programs, such as home management of malaria (HMM), have been promoted by the World Health Organization (WHO) and others to extend care beyond the formal sector, and have been adopted in many African countries.[13-15]

1.2.2. Universal diagnostic testing

The WHO has recently released new guidelines for malaria treatment, recommending that suspected cases be confirmed by a parasitological test when possible.[16] Specifically, the WHO guidelines state that prompt parasitological confirmation by microscopy or alternatively by rapid diagnostic tests (RDTs) is recommended in all patients suspected of malaria before treatment is started; and that treatment solely on the basis of clinical suspicion should only be considered when a parasitological diagnosis is not accessible. The shift towards universal diagnostic testing for malaria is

very welcomed, but raises serious questions about the capacity for diagnosing malaria in endemic areas.

Currently, malaria diagnostics are generally only available in the formal healthcare sector, where a minority of antimalarial treatments are distributed. Even where diagnostic facilities are available, studies suggest that antimalarial treatment is given to at least half of patients with a negative test.[17-20] RDTs for malaria offer promise for extending diagnosis beyond hospitals and clinics, reaching the areas where many patients seek treatment. However, deployment of RDTs not straightforward, and introducing them into the periphery has several operational challenges. When RDTs have been introduced into settings where microscopy is available, studies suggest that providers often ignore negative results, misdiagnosing and overtreating malaria.[19,20]

1.2.3. Training health workers in malaria diagnosis

Limited training, supervision, and support for the shift from presumptive to diagnostic-driven treatment may be associated with poor provider adherence to test results. More recently, encouraging results have been seen in Uganda where a one-week integrated course for health workers at facilities with microscopy significantly decreased unnecessary ACT prescriptions.[21] Also in Uganda, data from a study evaluating an RDT training course targeted to health workers in lower-level public health centers, which included clear guidelines on management of positive and negative RDT results, dramatically decreased unnecessary antimalarial prescriptions while maintaining satisfactory patient outcomes [H Hopkins, unpublished data]. However, it remains to be seen whether these more intensive training programs can be taken to scale. One of the main challenges for the introduction of malaria diagnostics has been to concurrently enhance capacity to diagnose and treat alternative causes of fever.[22] The need to integrate training in case management of malaria with that of non-malaria febrile illnesses, and evidence that more supportive training packages increase effectiveness,[23-25] demonstrates the importance of integrating malaria programs with health services in general. A more comprehensive approach to health care that will attract more patients and manage multiple diseases effectively is called for to produce tangible health benefits in the population as a whole.

1.3. MALARIA IN UGANDA

1.3.1. Overview

Malaria is one of the most important health problems in Uganda and the leading cause of morbidity and mortality in children, accounting for up to 40% of outpatient visits, 20% of hospital admissions, and 14% of inpatient deaths.[26] Children in Uganda experience an estimated average of six episodes of malaria each year, resulting in between 70,000 and 110,000 deaths annually. Up to 90% of Uganda’s population lives in highly endemic areas with perennial malaria transmission, while 10% live in areas at risk for epidemics.[27] The Ugandan Ministry of Health has developed a strategic plan for malaria control which focuses on intermittent presumptive treatment of pregnant women, vector control measures (including use of insecticide-treated bednets and indoor residual spraying), improved epidemic preparedness and response, and improved case management of malaria in health facilities and at home.[27] Access to care remains a major challenge in Uganda; only 49% of households live within a 5km radius of a public health facility.[28], and febrile children are frequently

treated outside of the formal sector.[29-32] Additional barriers to appropriate health care in Uganda include high costs, poor attitude of health workers, and stock-outs of drugs.[33]

1.3.2. Antimalarial drug policy

Artemether-lumefantrine (AL) was adopted as the new first-line treatment for uncomplicated malaria in 2004, with artesunate + amodiaquine (AS+AQ) as an alternative if AL was not available. Studies suggest that AL is highly effective for treating uncomplicated malaria in Uganda, with risk of recrudescence of 1% in Kampala and Tororo;[34-36] however, stock-outs of AL remain an issue.[33,37,38] A severe shortage of AL in Uganda has markedly limited the amount of drug available in the country.[39] Many public health facilities have been left without AL for months, and health care workers have been forced to resort to ineffective or inappropriate drugs for treatment of malaria.

In 2002, Uganda launched the national home-based management of fever (HBMF) program to address the challenge of poor access to antimalarial treatment, and to extend delivery of effective antimalarial drugs beyond the formal sector.[40] The aim of Uganda's HBMF program was to improve access to drugs and to treat all fevers in children under five within 24 hours of onset of symptoms by distributing antimalarials free of cost via volunteer community medicine distributors (CMDs). The program was scaled-up to country-wide coverage over approximately three years;[41] however, it was fully functional in only a few areas. Policy on community-based interventions in Uganda has recently changed expanding from malaria-only to integrated community case management (ICCM). Community health workers (CHWs), participating in village health teams (VHTs) will be trained to evaluate and provide presumptive treatment of malaria, pneumonia, and diarrhea based on clinical criteria. Community-based health care in Uganda is currently in transition; the HBMF program is being phased out and is inactive in most areas, but ICCM has not yet been rolled out. The anticipated launch date of the new ICCM policy is July 2010.

1.3.3. Affordable Medicines Facility – malaria

Another program that may increase access to antimalarials in Uganda is the Affordable Medicines Facility – malaria (AMFm), which is a new financing mechanism, intended to increase access to ACTs and reduce access to less effective antimalarial treatments, particularly artemisinin monotherapies (http://www.theglobalfund.org/en/amfm/). The AMFm is hosted by the Global Fund and is being piloted in 9 countries, beginning in June 2010, dependent upon agreements being signed between relevant parties. The mechanism of the AMFm is to make a co-payment towards the cost of ACTs with eligible first-line buyers in the public, private or not-for-profit sectors. This subsidy is intended to result in lower costs of ACTs being distributed through the different sectors. Alongside the subsidy, countries are required to implement 'supporting interventions' such as training providers and outreach to communities in order to improve malaria case management and to promote ACT use. Each country is responsible for the design and implementation of supporting interventions as well as for monitoring and evaluation activities over the period of the first phase of the pilot, lasting two years. Uganda's application to be a pilot AMFm country was accepted in November 2009 and the agreement for the contract with the Global Fund is in progress.

1.4. HEALTH FACILITY INTERVENTIONS

1.4.1. What is quality care and what are the problems?

The Institute of Medicine definition of quality of care incorporates six aims, that it be *safe, effective, patient-centered, timely, efficient* and *equitable*.[42] Evidence from increasing numbers of studies suggests quality of care by these measures is poor in many settings, including delivery of primary care in low-income countries. In terms of effectiveness and safety, direct observation studies of performance have identified severe deficiencies, particularly in history taking and examinations, diagnosis, and appropriate treatment choice and dosage.[43-48] This has been linked to low motivation of staff as well as poor resource availability in the work place. In terms of patient-centeredness and timeliness, meeting a population's expectations of how they should be treated by providers, including patient expectations for health care, is now seen as central to performance.[49] It has been argued that poor quality services fail to earn the population's trust, leading to clients seeking alternative sources of care,[50] or discontinuing care.[51] In contrast, the perception of good quality services, including inter-personal relationships, has been found to encourage patients to access care,[52] and demand for services.[53-55] Satisfied patients may be more likely to comply with treatment and maintain a continuing relationship with the health worker,[56] and loyalty to a clinic.[57], thus enjoying a better medical prognosis (presuming good technical quality of care).[58]

1.4.2. How can quality of care be improved?

Interventions to improve quality of care in low-resource settings have largely fallen into two categories: resource-based interventions and performance-based interventions. Resource-based interventions include the provision of equipment, infrastructure and drugs. Performance-based interventions have mostly been focused on clinical training and dissemination of guidelines. Far fewer studies have assessed interventions to improve aspects of quality care outside of clinical care.

1.4.2.1. Resource-based interventions

Resource-based interventions have shown that improved supplies together with guideline training can lead to improvements in quality of care,[59] and these improvements can be effective when solutions to resource needs are driven by local actors, for example using participatory research methods.[60] When resources, such as equipment, are delivered in a top-down fashion according to perceived needs, they may not be appropriate and may not be used.[61] In addition, resources such as RDTs for malaria need to be introduced in an appropriate manner, including generating local ownership and preparedness for change and providing troubleshooting and support in the longer term.[62] Further research to identify principle components for introducing equipment and supplies to primary health centers is needed to inform implementation across low-resource countries.

1.4.2.2. Performance-based interventions

Systematic reviews of evaluations of training-based interventions to change clinical behavior have produced mixed results in both developed,[63-67] and developing countries.[68] A training intervention to improve medical assistants' malaria prescribing practices in Ghana found initial improvements in practice deteriorated after twelve months, attributing the gap between knowledge and practice to socio-cultural factors including patient pressure, self-interested motivations and a

lack of supervision.[69] An evaluation of training for CHWs in Kenya found no improvement in overall process quality during consultations with pediatric patients after increasing numbers of refresher training series attended.[70] In fact, longitudinal analysis of this intervention showed that whilst initial refresher training led to improved management for severely ill children, adherence to guidelines for non-severe cases worsened, and after second refresher training the overall adherence declined rapidly.[71]

1.4.2.3. Why does training fail?

The failure of many training based programs to improve clinical care reflects a wider acknowledgement of a gap between knowledge and practice of health workers. Interpretation of the literature suggests that training can form the basis of effective strategies but only as long as other conditions are fulfilled. For example, the introduction of the integrated management of childhood illnesses (IMCI) scheme to primary health care facilities has had better results in countries with stronger health systems, and where support for the scheme has been strong, in terms of political advocacy, trainer support, and integration into current practices.[72-77] The importance of management is echoed in findings from Mexico where interactive education was combined with managerial interventions to improve prescribing practices for rhinopharyngitis at 18 primary health centers.[78] Similarly, in Kenya, treatment of uncomplicated malaria was better at facilities where health workers had undertaken in-service malaria training and where guidelines, wall charts, and more frequent supervision were provided.[79] However, evaluation of supervision and job aids for CHWs in Kenya found that these interventions did not improve adherence to guidelines for pediatric case management.[70] The authors suggest this may have been due to the quality of these interventions: relatively little time was spent on supervising clinical practice (co-examination of children by supervisor and health worker) compared to administrative tasks; quality of feedback from the supervisors may have been poor; and guidelines provided in job aides may not have been clear. In spite of the emphasis on training, clinical quality of care remains poor in many low-resource settings. Further research into how to support clinicians to improve performance, beyond didactic training, is urgently needed.

1.4.2.4. Patient-centeredness

Performance-based interventions tend to favor traditional continuing medical education training programs and are less focused on patient-centeredness. However, there is some evidence that participatory workshop interventions conducted over an extended period can improve communication with patients in different settings.[80] Ensuring that interventional methods used are appropriate to the local setting, and that participants are able to tackle wider issues that affect their ability to communicate effectively, have been shown to be important.[81,82] In high-resource settings, interventions targeted at provider communication have shown some success in targeting three aspects of the interaction: giving time to talk,[83] providing emotional care,[84,85] and giving positive communication.[86,87] Intervention methods to tackle these specific issues in patient-centeredness are based on adult learning theory, following principles of learning through experience and reflection. Self-observation, or ‘mindfulness meditation’ has formed an important part of some effective programs.[88] Further research is needed to identify intervention components that successfully achieve improved levels of patient-centeredness in a sustainable way that are replicable in low-resource settings.

1.5. PHASE I RESULTS

In the first phase of this project, we aimed to characterize the population and local health services in Tororo district by conducting a census survey, a survey of health services, and a qualitative study. All households within West Budama North health sub-district in Tororo, including Nagongera, Paya, Kirewa, Kisoko, and Petta sub-counties, were enumerated and mapped to provide a sampling frame for the main trial. To characterize the population, households were surveyed on basic demographic information, markers of socioeconomic status, vital statistics, and bednet practices. We also characterized the local health services, focusing on the public health facilities and the existing HBMF program, by surveying health care workers and community medicine distributors. In addition, we conducted a series of focus group discussions with primary caregivers, heads of households, and health care workers.

The initial field work was conducted from October 2009 to February 2010. Our census team consisted of surveyors paired with local research assistants who moved with the local leaders of each village to identify all households. The health services survey and qualitative study were conducted by a team of interviewers trained in social science and qualitative research.

Additional Phase I work, including (1) a census survey of two sub-counties in West Budama South health sub-district, (2) a brief survey of lower-level government-run health facilities in that area to evaluate their catchment areas, and (3) self-observational activities for health workers to increase their awareness about barriers to communicating with patients, will be conducted in August and September 2010.

1.5.1. Census survey

We enumerated 26,793 households in five sub-counties in Tororo district, including 144,216 residents and 26,905 children under five. Our results suggest that this area of Tororo is very rural, with limited infrastructure and education. Very few households have electricity (1%), or own a television (2%). Ownership of mobile phones (31%) and radios (43%) is also low. One-quarter of households have no toilet facilities, and only 30 households have a flush toilet. Overall, heads of household are not well-educated; one-quarter have received no formal education, and only 21% have received any secondary or higher education. Mortality in children under five was estimated to be 10.99%.

Although over half (63%) of households reported that they owned a bednet, only 37% of residents reported that they had slept under a bednet the previous night. Only 15% of residents and 20% of children under five slept under an insecticide-treated bednet (ITN) during the previous night, which is far below the targets of > 80%.

1.5.2. Public health facilities

We interviewed 81 (88%) of 92 health workers stationed at the 17 functional government-run health facilities in West Budama North. Most health workers (56 [69%]) were trained in management of malaria with AL, but only 29 (26%) had received training in RDTs for malaria.

Staffing shortages and absenteeism are a problem at most health centers; 16 (94%) reported that the number of staff working at the facility was insufficient. Drug stock-outs are also a major problem, particularly at HC IIs and HC IIIs. Nearly all health centers (94%) reported that they experienced

stock-outs. Only 29% of health centers reported that the supply of antimalarial drugs was adequate for treating their patients. Infrastructure at the health facilities in this area is also limited. Most lack electricity (88%) and running water (94%).

Overall, the knowledge of health workers about malaria case management was surprisingly poor. Out of a possible 178 points, the mean score achieved by health workers on a knowledge questionnaire was 51.6 (29%), ranging from 15 to 110. The in-charges of health centers scored unexpectedly low with a mean score of 60.5 (34%). When asked how to confirm the diagnosis of malaria, only eight (10%) health workers mentioned microscopy and two (2%) RDTs.

The results of knowledge questionnaire identified areas to target in training include physical examination skills, identification of danger signs for severe malaria, malaria diagnostics, differential diagnosis of non-malarial febrile illnesses, and key elements of managing uncomplicated and severe malaria (including administration of medications).

1.5.3. Community medicine distributors

We selected 100 community medicine distributors (CMDs) who had participated in the HBMF program using convenience sampling from five sub-counties in Tororo district. Major gaps in CMD training, supervision, and knowledge were identified. Interviews also revealed that CMDs are involved in implementing multiple programs led by different stakeholders, which are not integrated. Only four CMDs reported receiving support supervision in the last six months. Most CMDs (92%) said that they refer at least one patient each week, most commonly to a local HCIII. However, only 23% of CMDs said that they would refer the child after two days if no improvement was seen, suggesting that they refer sick children on initial review, but do not provide follow-up of patients.

Overall, CMDs scored poorly on the knowledge questionnaire (mean score 22%). Only 74% CMDs correctly identified fever as the most common symptom of malaria in children, and recognition of danger signs of severe malaria was poor. Although 61% of CMDs had received training on management of malaria with AL, few CMDs correctly described how AL should be administered. Recognition of non-malarial causes of fever in children was also poor.

Our results suggest that CMDs knowledge of appropriate management of malaria is limited, despite training, and they may be overstretched by stakeholders attempting to deliver community-based interventions.

1.5.4. Qualitative study

We conducted 69 in-depth interviews with health workers stationed at the 17 health facilities. In-depth interviews were conducted with 100 CMDs. A total of six FGDs, involving 65 participants, were conducted with health workers, representing all three health center levels (HC II, III, and IV) and higher and lower cadres of health workers. Five FGDs were conducted with 55 primary caregivers from the community, including two with caregivers under the age of 30 years, and three with older caregivers. Participants from four of these FGDs lived in parishes that had a public health facility. Five FGDs were conducted with 58 heads of household from the community, including two with female heads, and three with males. Participants from four of these FGDs lived in parishes that did not have a public health facility.

1.5.4.1. Defining good quality health care

When asked, 'what is good quality health care', community members, health workers and CMDs described similar values involving three themes: 1) *comprehensive therapeutic process* which describes the clinical treatment given to patients and the relationship between patient and health worker; 2) *management of health facilities* which describes operational components including staffing and facilities as well as availability of drugs and equipment, and 3) *expectations of responsiveness* which describes the provision of prompt and free services.

1.5.4.2. Attendance at public health centers

Community members report various and complex treatment seeking behaviors and outcomes. Most community members have visited their local health facility, but were dissatisfied with their experience. Barriers faced by patients in getting to health facilities were identified. In addition, we found that health facilities are not attractive to community members. Patients cannot get to health facilities because of logistical and cultural barriers, and are not attracted to health facilities because of poor management, poor interpersonal relationships with health workers, and local system failures.

1.5.4.3. Quality of care provided at health centers

We found that aspirations for good quality care were similar amongst health workers, CMDs, and community members. The most frequently discussed values include those involving the comprehensive therapeutic process (good clinical care and treatment, welcome and orientation, good interpersonal interactions between health workers and patients, and advice), responsiveness of health workers and the public health system (prompt and fair treatment, treatment free-of-charge), and management of health facilities (adequate staff, equipment, and infrastructure, availability of drugs, professional health workers).

At health centers, immediate barriers to quality care included drug stock-outs and lack of equipment; high patient to staff ratio; use of volunteer health workers; language barrier between health workers and patients and discriminatory treatment of patients. Underlying these barriers were poor motivation of staff; poor management of the health center; lack of patient-centerd culture and poor relationship between health workers and communities.

1.5.4.4. Treatment seeking behavior

Treatment seeking behavior of community members is largely driven by perceptions and understanding of illnesses and practical concerns, including accessibility, available resources, and prior experiences. In this area, first-line treatment for most conditions was with a biomedical drug. Nearly all community members had visited their local health center, but dissatisfaction with care was high. Other sources of health care include CMDs (when operational), private clinics, and drug shops. Herbal medicine was frequently used and interestingly, community members also relied heavily on shrines, churches and prayers for treatment.

Choice of health care was influenced by the following factors: (1) initial perceptions and beliefs about etiology and severity of the illness that would, from experience, require a particular source of treatment. Often, experience showed health centers to be a poorer source of care than other providers for common illnesses; (2) accessibility of the preferred treatment, which relied on distance to the provider as well as opening hours, spousal support in meeting costs, opportunity costs of

leaving the home and travelling to the provider, ability to negotiate the logistical and social rules of the provider's institution, and availability of treatment at that provider; and (3) trial and error in moving between treatment sources.

2 RATIONALE

2.1. PROBLEM STATEMENT

The current approach to management of malaria and febrile illnesses in Ugandan children is inadequate. Given the barriers to accessing good quality care through the formal heathcare sector, substantial attention and resources have been focused on developing community-based interventions to deliver antimalarial treatment and comprehensive care.[13,14,89] However, whether resources should be put into community-based programs or into improving the public health system is not clear; malaria case management could be strengthened by improving the quality and delivery of care in existing government-run health facilities. Our study is designed to assess whether an intervention to build capacity and improve delivery of drugs and diagnostics at government-run health facilities improves the health of children and quality of care delivered, as compared to 'standard care' currently available at health facilities, supplemented by services provided through the private sector and community-based interventions.

2.2. STUDY OVERVIEW

We are proposing to evaluate enhanced health facility-based care for malaria and febrile illnesses in children in Tororo district using a cluster randomized design. The health facility intervention (HFI) will aim to address barriers to achieving good quality health care that were identified in our formative research. A focus of our intervention will be providing RDTs and training health workers in management of both malaria and non-malarial febrile illnesses. We aim to implement an intervention which is sustainable and reproducible by the MoH in Uganda, working within the existing government systems in conjunction with the MoH and district teams.

Clusters, defined as health facilities and their catchment areas, will be randomized to the HFI or to standard care delivered from government-run health facilities, supplemented by services provided through the private sector and community-based interventions. Outcomes will be measured in three distinct populations: (1) cross-sectional surveys of children under 15 years randomly selected from households within the clusters; (2) a cohort of children under five randomly selected from households within the clusters and followed for approximately 18 months in total, 12 months following the roll-out of the intervention; and (3) patients attending all government-run health facilities, including children under five and their caregivers participating in exit interviews on selected days in three rounds of surveys.

The primary outcome of the study is prevalence of anemia in children under five. We will test the primary hypothesis that the prevalence of anemia in children under five from clusters randomized to the HFI will be lower than in children randomized to the current standard of care. The study proposed here will benefit greatly from our formative research, prior longitudinal studies of antimalarial therapy conducted in Kampala, including two cohort studies evaluating health facility-based care,[34,90,91] and one cohort study evaluating HBMF, [92] and ongoing surveillance conducted by the Uganda Malaria Surveillance Project. The prior and ongoing studies are expected to inform the design and conduct of the planned research.

2.3. STUDY POPULATION AND DESIGN

In the cross-sectional surveys and cohort study, we plan to focus on children under five as young children bear the greatest burden of malaria in this endemic population. However, in our cross-sectional surveys we will also evaluate children aged 5-15 years, as this group contributes substantially to malaria transmission.

We have opted for a cluster randomized design rather than a non-randomized approach, in which the HFI would be implemented in all facilities, to minimize confounding. It is likely that other health-related interventions will be implemented in Tororo district by the MoH or other stakeholders, such as bednet distribution or safe water campaigns, which could have an impact on the health of children in the study area. If we relied on comparisons between measurements taken before and after implementation of the HFI, we could risk falsely attributing improvements to our intervention, when in fact the changes were due to factors outside of our study.

We have chosen the health facility as the unit of randomization, defining the clusters as catchment areas of these facilities, including households within a 2km radius of the health center. Only children from households within the clusters will be eligible for participation in the cross-sectional surveys and cohort study. We have opted to restrict the study population to children from villages near to facilities rather than including the full population of the study area to minimize risk of contamination between the study arms. We recognize that distance to the health facility is a major factor influencing utilization, which we will not be able to change with our intervention. Children from villages in the periphery of the health facility catchment areas may or may not be able to access services because of distance. If children who could not access the health facilities were included in the study population, we could risk underestimating the impact of the intervention, resulting in a Type II error in which we fail to identify a real difference between our HFI and standard care.

We also recognize the possibility of contamination in the other direction; primary caregivers of children in clusters randomized to standard care may seek care from facilities randomized to the HFI. Again, distance to the health center is likely to influence the likelihood of seeking care. By restricting the study population to children residing in households within a 2km radius of the health facility, we hope to focus our evaluation on children who are most likely to receive care from that facility. In addition, we plan to capture information on treatment seeking behavior during the cross-sectional surveys, and information on patient attendance and village of residence from all health facilities, to track patterns in utilization of services from various sources. We recognize that restricting the study population to households nearby the health facilities will limit our ability to generalize our results to more remote populations without access to health facilities. However, we plan to evaluate location of residence and distance to health facilities as a covariate in our analysis. In addition, when assessing outcomes for the cross-sectional survey and cohort study, analyses will be conducted primarily on an intention-to-treat basis, where data collected will be analyzed according to the assigned cluster. To address potential contamination issues due to children attending health facilities other than the one that defines their cluster, i.e. the health facility they live closest to, we shall also analyse data on a per-protocol basis. For this analysis, data collected will be analysed according to the facility at which the child accesses healthcare.

3 STUDY OBJECTIVES

1. To compare the impact of enhanced health facility-based care to current standard of care on key population-based indicators, including the prevalence of anemia in children under five, using a cluster randomized design. We will test the primary hypothesis that the prevalence of anemia will be lower in children under five from clusters randomized to the health facility intervention (HFI) than in children randomized to standard care. Children receiving care from the health facilities participating in the HFI may be more likely to receive good quality health care and appropriate treatment for malaria and non-malaria illnesses, including anemia and helminth infections. We will also test the secondary hypothesis that the prevalence of parasitemia in children under five, and children aged 5-15 years, from clusters that are randomized to the HFI will be lower than in children randomized to standard care.

2. To compare the impact of enhanced health facility-based care to current standard of care on key longitudinal indicators, including treatment incidence density, in a prospectively followed cohort of children under five. We will test the hypothesis that delivery of antimalarial treatment via current care will result in over-treatment and a higher incidence of antimalarial treatment, than antimalarial treatment delivered from health facilities randomized to the HFI, which will be targeted to lab-confirmed cases of malaria.

3. To compare impact of enhanced health facility-based care to current standard of care on key indicators of case management for malaria and other illnesses, including the risk of inappropriate antimalarial treatment, in children under five treated at health facilities. We will test the hypothesis that the health facility intervention decreases inappropriate treatment with ACTs, as measured by the proportion of children under five with suspected malaria and a negative RDT result that are inappropriately treated with an ACT plus the proportion of children under five with suspected malaria and a positive RDT result that are not prescribed an ACT, which we expect to be lower in the facilities randomized to the intervention than in those in the standard care group.

4 STUDY DESIGN

4.1. OVERVIEW

We propose to compare enhanced health facility care to the current standard of care using a cluster-randomized design in Tororo, Uganda. A census survey of the study area has been conducted to enumerate and map households, which will be used to create the sampling frame for the trial. The lower-level government-run health facilities (20 HC IIs and IIIs) in the area will be randomly assigned to one of two interventions: (1) health facility intervention (HFI), or (2) standard care. The HFI will focus on three components: (1) improving health center management, (2) providing health worker training, and (3) ensuring adequate malaria diagnostics and drug supplies. Standard care will include services provided by government-run facilities that are not randomized to the HFI. In addition, services provided by the private sector and through community-based interventions (CBI) implemented by the Ministry of Health and other partners will contribute to the health care provided in all areas. Currently, CBIs in Uganda are transitioning from home-based management of fever (HBMF) to village health teams (VHTs) delivering integrated community case management (ICCM). In Tororo district, CBIs are currently inactive but will likely be reactivated and scaled-up in Tororo district in a phased approach during the study period as follows: phase A: no CBI; phase B: malaria-only intervention; and phase C: VHT/ICCM.

Figure 1. Overview of study design

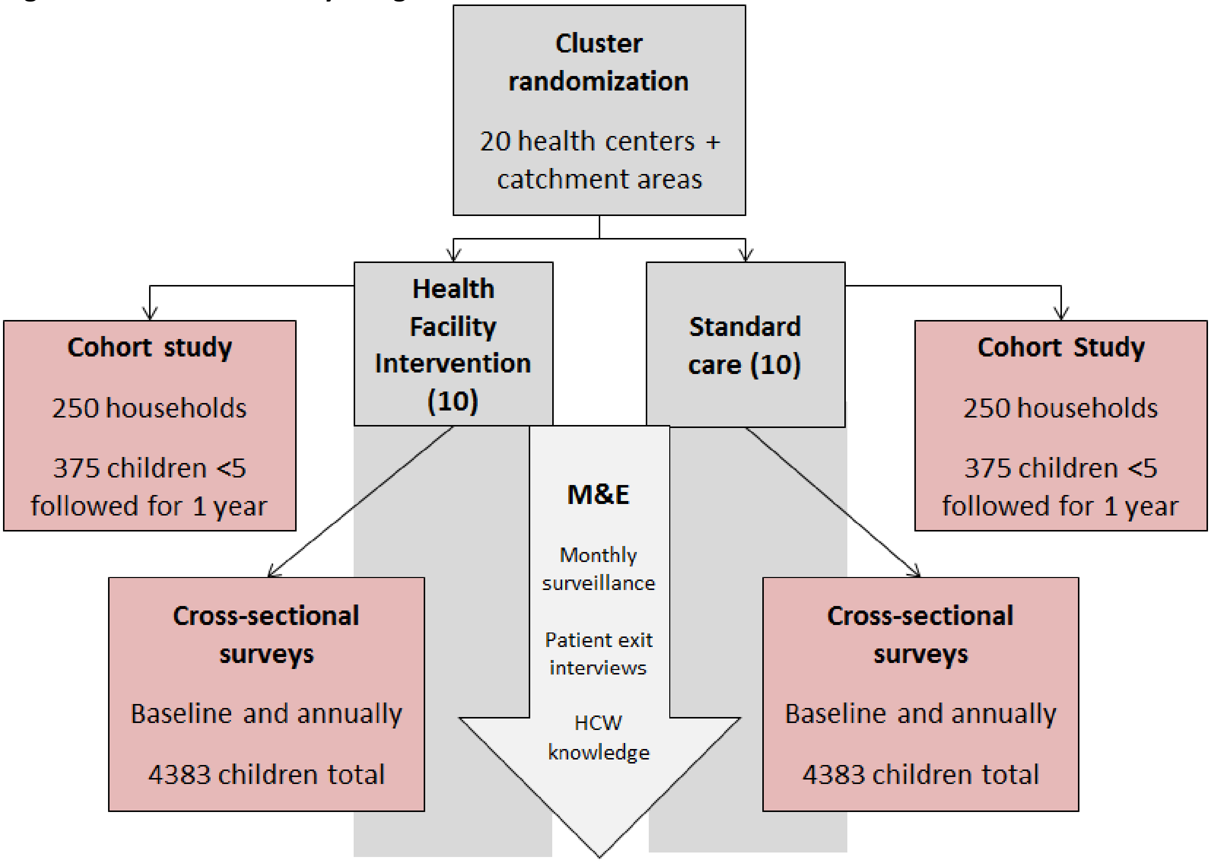

Information on changes in CBI policy and activity in the study area will be captured. In addition, AMFm may be scaled up in Uganda during the period of the study, which could include supporting interventions to train drug shop workers in the private sector and community awareness programs around ACTs. If AMFm is adopted in Uganda, these activities will be tracked as well.

Cross-sectional surveys will be conducted at baseline in randomly selected children from each cluster (8766 total per survey), and will be repeated annually. A sub-cohort of children will be recruited from 25 households randomly selected per cluster (500 total households) at the start of the intervention, and will closely followed for approximately 18 months in total, 12 months following the roll-out of the intervention. All health facilities in the area will be assessed to monitor and evaluate the impact of the intervention; three rounds of patient exit interviews will be conducted. In the first two surveys, 10 patients from each health facility (200 total per survey) will be interviewed. In the final survey, 50 patients from each facility (1000 total) will be interviewed. A total of 1400 patients will participate in the exit interviews.

4.2. STUDY SITE

The study will be conducted in Tororo district, an area with very high malaria transmission intensity The estimated entomologic inoculation rate (EIR) in Tororo is 562 infective bites per person-year, and the prevalence of parasitemia among children aged 5-9 years is 63.5%.[93,94] The five sub-counties of West Budama North Health Sub-district (Nagongera, Paya, Kirewa, Kisoko, and Petta), and two sub-counties of West Budama South Health Sub-district (Mulanda and Rubongi) will be included in the study population (Figure 2).

Figure 2. Study area

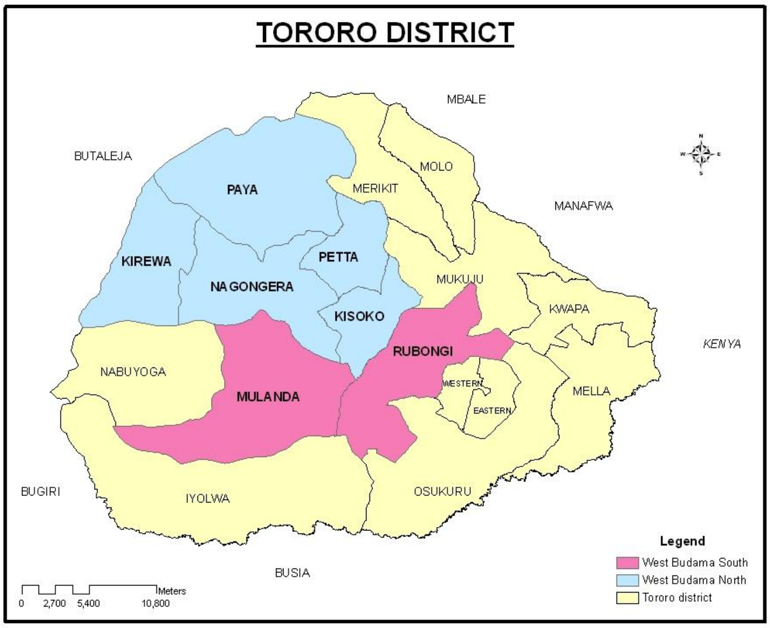

The results of our formative research suggest that this area is very rural, with limited infrastructure and education. Very few households have electricity (1%) and one-quarter have no toilet facilities. One-quarter of the heads of household have received no formal education, and only 21% have received any secondary or higher education.

4.3. STUDY POPULATION

Within the seven sub-counties, there are 22 lower-level government run health facilities, including 17 level II health centers (HC), and 5 level III HCs; 20 will be included in the randomization scheme. Clusters will be defined as the catchment areas of the health centers, including households that are located within a 2 km radius of the facilities. Only households located within the clusters will be included in the sampling frame for the cross-sectional surveys and the cohort study. The study population for each objective is listed in Table 1.

Table 1. Study objectives and populations

Objective	Study population and sample size
1. To compare the impact of enhanced health facility-based care to current standard of care on key population-based indicators, including the prevalence of anemia in children under five.	Cross-sectional surveys in children under five and aged 5-15 years randomly selected from households in each cluster (8766 children total); surveys will be conducted at baseline and then annually for 2 years (3 surveys in total)
2. To compare the impact of enhanced health facility-based care to current standard of care on key longitudinal indicators, including antimalarial treatment incidence density, in a cohort of children under five.	Cohort of children under five recruited from 25 households randomly selected from each cluster (500 total) and followed for approximately 18 months in total, 12 months following the roll-out of the intervention; all children of appropriate age from each household will be eligible to participate
3. To compare impact of enhanced health facility-based care to current standard of care on key indicators of case management for malaria and other illnesses, including the risk of inappropriate antimalarial treatment, in children under five treated at health facilities.	Exit interviews in patients attending lower-level government-run health facilities (20 HC IIs and IIIs) in the study area (3 surveys in total). In the first two surveys, including 10 patients per health facility (200 patients per survey). In the final survey, including 50 patients per health facility (1000 patients in survey, 1400 patients overall).

4.4. CLUSTER RANDOMISATION

The lower-level government-run health facilities in the study area will be the unit of randomization. Clusters will be defined as the catchment areas of the health centers, including households located within a 2km radius of the facilities (Figure 3). The clusters will be defined prior to randomization using the full census survey database. All households and health facilities in West Budama North have already been mapped. The census survey and mapping of the two sub-counties in West Budama South (Mulanda and Rubongi) will be conducted in August and September 2010. The distances between every household and every lower-level government-run health facility in the area

will be calculated. Households will be excluded from our sampling frame if they are ≥ 2km from any health facility. If a household is within 2km of a single health facility, the household will be considered to be within its catchment area and will be assigned to the cluster of that health facility. If a household is within 2km of more than one facility, the household will be assigned to the cluster of the closest health facility.

A total of 22 facilities are currently active in the area, however, 2 pairs of health centers are within close proximity (Soni HC II and Kirewa Chawolo HC II; Pokongo HC II and Morkiswa HC II) and have substantially overlapping catchment areas. Given this, one facility from each pair will be randomly excluded from the randomization scheme.

Figure 3. Map of health facilities and clusters in West Budama North and West Budama South

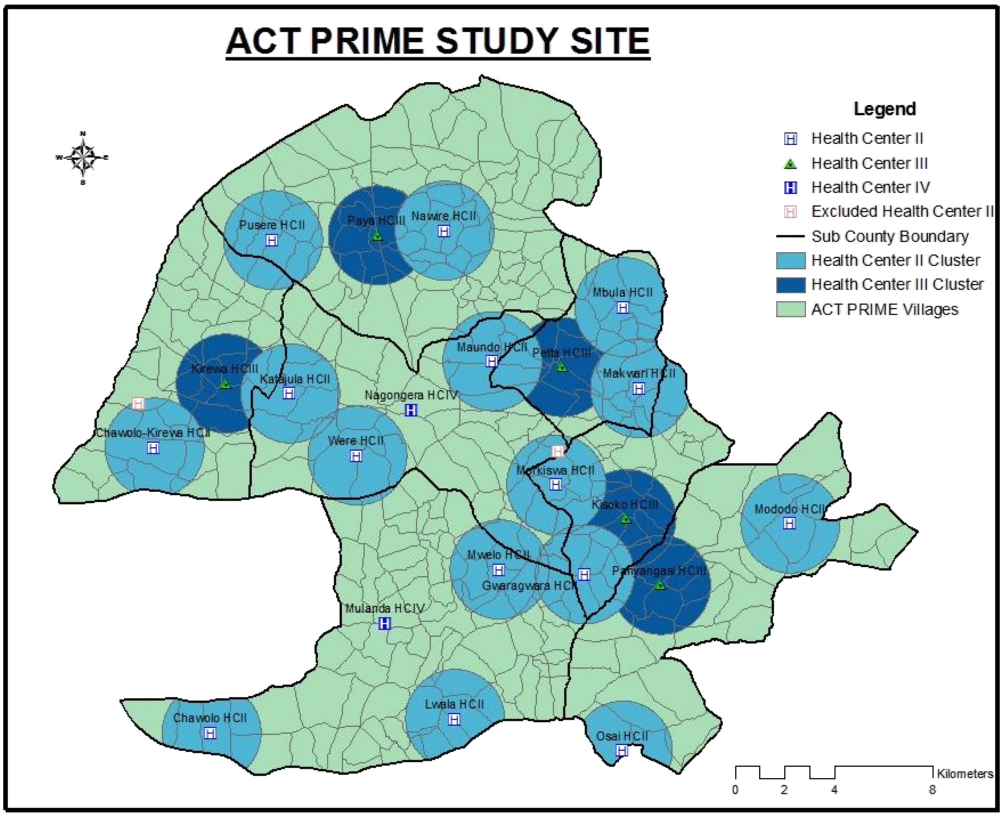

The randomization will be conducted by an investigator who is not directly involved in the project. Health facilities will be stratified by level (HC IIs and HC IIIs). Because of the uneven numbers of HC IIs and IIIs, one of the HC IIIs without a laboratory will be ‘demoted’ and paired with a HC II to ensure even numbers. Restricted randomization will be employed to ensure balance on geographical location. Specifically, restrictions will be applied that exclude the allocation of all clusters originating from a single sub-county, or that are otherwise in close geographical proximity from being allocated to the same arm of the trial.

4.5. OUTCOME MEASURES

The primary and secondary outcome measures for each study objective are listed below in Table 2.

Table 2. Study objectives and outcome measures

**Outcomes**	**Indicator**
**Objective 1: Cross-sectional surveys**	
Prevalence of anemia	Proportion of Hb measurements < 11.0 g/dL. Anemia will be classified according to severity: mild (Hb 8.0 – 10.9), moderate (Hb 5.0 – 7.9), severe (Hb < 5.0).
Prevalence of parasitemia	Proportion of thick blood smears that are positive for asexual parasites
Prevalence of gametocytemia	Proportion of thick blood smears that are positive for gametocytes
All-cause mortality	Probability of dying between birth and five years of age, expressed per 1,000 live births
**Objective 2: Cohort study**	
Antimalarial treatment incidence density	Number of antimalarial treatments given for fever/malaria over the period of follow-up
Incidence of hospitalizations	Overnight admission to a hospital or clinic
Incidence of illness episodes	Episode of illness as reported by primary caregiver
Incidence of febrile episodes	Episode of illness associated with fever as reported by primary caregiver
Prompt effective treatment of fever	Proportion of children with fever treated within 24 hours of onset of symptoms with an ACT
Prompt effective treatment of malaria	Proportion of children with malaria (confirmed by a parasitological test) treated within 24 hours of onset of symptoms with an ACT
Incidence of serious adverse events	Any experience that results in death, life-threatening experience, hospitalization, persistent or significant disability or incapacity, or specific medical or surgical intervention to prevent one of the other serious outcomes
Antibiotic treatment incidence density	Number of antibiotic treatments given for fever/bacterial illnesses over the period of follow-up
**Objective 3: Health facilities**	
**Patient exit interviews**	
Inappropriate treatment of malaria	Proportion of children under five with suspected malaria and a negative RDT result who are inappropriately given an ACT + Proportion of children under five with suspected malaria and a positive RDT result who are not prescribed an ACT
Appropriate treatment of malaria	Proportion of children under five with suspected malaria and a positive RDT result who are appropriately given an ACT + Proportion of children under five with suspected malaria and a negative RDT result who are not prescribed an ACT
Inappropriate treatment of malaria	Proportion of children under five with suspected malaria and a positive RDT result who are inappropriately given a non-ACT regimen
Patient satisfaction with health care	Proportion of patients indicating they were satisfied with care provided at the health center in exit interviews
**Health facility surveillance**	
Patient attendance	Total number of patients attending health facilities and their characteristics, including age, sex, village of residence, and diagnosis
Gaps in staffing requirements	Required positions, as indicated by the MoH staffing norms policy, which are unfilled for greater than one month
Stock-outs of ACTs	Days per month that AL supplied by NMS via the district is not available
**Health worker knowledge questionnaire**	
Knowledge questionnaire scores	Proportion of questions answered correctly following training in fever case management

5 HEALTH FACILITY INTERVENTION

5.1. OVERVIEW

The health facility intervention (HFI) will be comprised of three components: 1) health center management training, 2) health worker training, and 3) supply of consumables, including malaria diagnostics and antimalarial drugs. The goal of these components is to address the barriers to providing good quality care identified in our formative research (Appendix A). By addressing these barriers, we aim to provide good quality care as defined by health workers and community members in Tororo district, attracting them to health facilities and improving the case management of malaria and non-malarial febrile illnesses received when they attend facilities. The intervention package will be rolled out to all health centers randomized to the HFI over approximately 8-10 weeks (Table 3). Some activities will continue to be supported by the project for the duration of the study. We aim to implement an intervention which is sustainable and reproducible by the MoH in Uganda, working within the existing government systems in conjunction with the MoH and district teams.

Table 3. Health care intervention implementation plan

	Week 1	Week 2	Week 3	Week 4	Week 5	Week 6	Week 7	Week 8	Week 9	Week 10
**1) Health center management**										
Financial management										
Drug supply management										
Information management										
**2) Health worker training**										
**Fever case management and RDTs**										
**Patient-centered services:**										
Introduction to PCS and HCM										
Communication & building rapport										
Asking questions & giving advice										
Interactions with colleagues										
Improving the patient visit										
**3) Supply of consumables**										
National Medical Stores drug supply										
Support for AL stock-outs										
RDTs for malaria										

Prior to the start of the study, investigators and key study personnel will meet with members of the MoH, the National Malaria Control Program, district and sub-county officials, and community representatives to inform them about the study objectives, plans for the intervention, and follow-up assessments. An information sheet will be used to describe the plans for the intervention to the in-charges of health facilities randomized to the HFI (Appendix B).

5.2. HEALTH CENTER MANAGEMENT

5.2.1. Overview

All in-charges of health centers randomized to the HFI will be trained in health center management. The purpose of this training is to equip in-charges with the skills and tools required to effectively and efficiently manage their health center. The training modules will be developed by reviewing of the literature, holding discussions with the MoH and other stakeholders, reviewing policy guidelines, and piloting of materials with subsequent revisions. The training will include three components: (1) financial management, (2) supply management, and (3) information management (Table 4).

Table 4. Proposed content of Health Center Management training

Financial management	Supply management	Information management
— Financial reporting<br>— Budgeting and accounting	— Ordering and distributing<br>— Inventory management and stock control<br>— Managing stock-outs	— Collecting information accurately & timely<br>— Managing information<br>— Using information

Our study staff will conduct a series of half-day group training workshops with the in-charges every 1-2 weeks. The sessions will be conducted using both formal classroom lecture sessions and informal practical methods with discussions, demonstrations, and role plays. The Health Center Management Training module will last approximately 4-5 weeks. Workshops will be held in convenient locations for the participants and all costs will be covered by the project.

5.2.2. Financial management

This component will focus on financial management and accounting including documentation of financial transactions and audit trails and management of funds. In addition, the training will cover budgeting and accounting for the primary health care (PHC) funds. In our formative research, we found that management of the health centers was challenged by insufficient PHC funds. These funds are meant to cover the costs of a variety of activities at the health centers, including support staff, cleaning materials, transportation of drugs, and photocopying documents. Health centers are allocated an amount set by the district, and expenditure appears to vary depending on the individual needs of the facilities. As part of the HFI, we propose to train the in-charges to account for the PHC funds using a tool (Appendix C) designed to assist with the budgeting and accounting of the PHC funds. The sample tool provided in Appendix C will be piloted and during the Health Center Management Training, and revised as appropriate.

5.2.3. Supply management

Effective management of supplies is a crucial aspect of providing high quality health care services. This component will focus on enhancing the understanding and operation of the drug distribution cycle including barriers in ordering, receiving, and issuing of drugs; forecasting of supply needs; maintaining a regular inventory to prevent stock-outs; and completing and maintaining stock cards. In-charges will be trained to use the one-page ACT Drug Distribution Assessment Tool (ADDAT),

which aims to identify and resolve issues in drug distribution from districts to local health facilities (Appendix D). The ADDAT is based on a literature review, in-depth interviews and focus group discussions (FGDs) with health workers and key informants, and the MoH 'Medicines Management Manual: Medicines Logistics and Store Management procedures for Districts and Health Facilities'. The ADDAT will be used monthly by in-charges or persons responsible for drug procurement and distribution at the health center. The sample tool provided in Appendix D will be piloted prior to the Health Center Management Training, and revised as appropriate.

5.2.4. Health information management

This component will focus on the importance of monitoring and evaluation and managing health information, building on our experience with UMSP surveillance. In-charges and health workers should have access to information about the health needs and priorities of the communities they serve; the quality and coverage of the services they offer; and available resources. All health workers will be trained about the importance of data collected information about patients accessing their health facility, using the patient, drug, and laboratory registers, and the health unit outpatient monthly report, and how the data can be utilized to better manage patients and health facilities.

5.3. HEALTH WORKER TRAINING

5.3.1. Training in fever case management and use of RDTs

All clinical staff will receive training in fever case management. Training will be based on the Integrated Malaria Management training package developed by the Joint Uganda Malaria Training Program (JUMP) team,[21] and the RDT training guidelines which have been adopted and implemented by Uganda's MoH 'User's manual: Use of Rapid Diagnostic Tests (RDTs) for malaria in fever case management in Uganda' (Appendix E). The training package will include the following sessions:

- Session 1: How to evaluate febrile patients and select patients for RDT testing
- Session 2: Performing and reading an RDT
- Session 3: Management of a patient with fever and a positive RDT
- Session 4: Management of a patient with fever and a negative RDT
- Session 5: Recognition and referral of patients with severe illness
- Session 6: Patient education
- Session 7: RDT storage and monitoring
- Session 8: Infection prevention

The training program will be conducted by the JUMP training team over two weeks; the first two days will focus on theory, and will be followed by support supervision at the health facilities the next week. The training will be conducted in Tororo at a local health facility, and health workers will be trained in two small groups to ensure that work at the health facilities continues alongside the training. The impact of training on knowledge will be assessed using a pre-and post-training evaluation administered by the JUMP team. Additional support supervision will be conducted at 6 weeks and 6 months after the initial training and refresher training will be provided as needed.

5.3.2. Training in patient-centered services

The purpose of the Patient-Centered Services (PCS) training module is to identify and improve interpersonal interactions between health workers and patients. The module builds on the results of our formative research which identified several barriers to proving good quality health care at health facilities, including poor interpersonal interactions between health workers and community members resulting from poor communication skills, discriminatory behaviors of health workers, poor health worker motivation, and lack of patient-centered thinking. Through the PCS module, health workers will learn to recognize these challenges and develop skills for communicating and interacting with patients.

The PCS module training will be implemented in a tiered approach to (1) all clinical staff, and (2) all health center support staff. All clinical staff, including in-charges, will receive the full PCS training package which includes self-observation tasks and specific emphasis on clinical and patient interaction challenges. Support staff including volunteers will receive a scaled-down PCS training package with specific emphasis on welcoming and guiding patients at the health facility. All training activities and workshops will be led by study personnel and trainers with experience in adult learning methodology.

5.3.2.1. PCS for clinical staff

The PCS module for all clinical staff starts with a series of four self-observation activities completed by health workers over a period of 4-5 weeks. Health workers will conduct the self-observation exercises individually during their routine work at health facilities. These observations serve three purposes: (1) to raise awareness of health workers about how their interactions with others affects their ability to work, (2) to begin to build a support network between colleagues, and (3) to help to identify issues directly relevant to the health workers in lower level health facilities. Through the self-observation activities, health workers are asked to become aware of their behavior around a particular topic and write a short summary of how their behavior affected those around them and their subsequent ability to achieve work goals. Topics include the following: (1) 'how do you listen?', (2) 'how do you ask good questions?', (3) 'what happens when you are stressed?', and (4) 'how do you invite the patient to cooperate?' (Appendices F to J). On completion of each self-observation, participants join other health worker colleagues to discuss their own observations and give support about how to carry out the next observations. The results of the observation activities will be used to guide health workers through the PCS training modules.

Table 5: Clinical staff PCS module themes and learning outcomes

PCS module	Learning outcomes
**Introduction to PCS, self-observational activities, and HCM**	Health workers will be able to:<br>• Identify their own motivations for work<br>• Understand the meaning and importance of providing patient centered services<br>• Start developing self-awareness through self-observation activities<br>• Understand the meaning and importance health center management
**Improving communication and building rapport with patients**	Health workers will be able to:<br>• Apply skills in verbal and verbal communication to build rapport and active listening<br>• Identify ways to listen actively in spite of busy work environments<br>• Recognise how we think of people affects how we behave towards them<br>• Understand that respect is a core value for how we can put patients at ease and strengthen skills to show respect to others
**Asking good questions and giving advice to patients**	Health workers will be able to:<br>• Strengthen verbal and non-verbal communication skills in asking good questions<br>• Overcome barriers that prevent patients from giving good information to health workers<br>• Implement strategies to give appropriate information on diagnosis and treatment to patients
**Improving interactions with colleagues**	Health workers will be able to:<br>• Contribute to a positive working environment<br>• Motivate and inspire others to work better by identifying ways to bring about positive change<br>• Give constructive feedback<br>• Communicate effectively with colleagues by identifying how we react to stress impacts how we react to others
**Improving the patient visit**	Health workers will be able to:<br>• Implement strategies to improve the welcome of patients at the health facility to patients<br>• Implement strategies to ensure patients are seen fairly<br>• Implement strategies to improve the orientation of patients at the health center<br>• Appropriately utilize volunteers to address current staffing gaps<br>• Contribute to improving patient satisfaction by working together with all clinical and support staff

The PCS module consists of five half-day day themed workshops, which build on the self-observation exercises. The modules and learning outcomes are outlined in Table 5. The modules will use a combination of learning approaches including didactic material on each theme; appreciative enquiry exercises; role-plays or other participatory activities; and group discussions. The didactic component is important for framing the central concepts and theories around each theme. Appreciative enquiry exercises will allow participants to explore ways in which they successfully worked through difficult situations and identify what qualities they employed, using analysis and reinforcement of positive experiences to lead to behavior change, rather than focusing solely on negative experiences or deficiencies. Participants will work in small groups for these exercises. The group discussion will explore reactions to the participatory activities and develop methods that can be employed to achieve successful outcomes related to the theme. Additional ice-breaker and group-building activities will be used to engage and motivate participants throughout the workshop.

The half-day workshops will be approximately 3 hours in length and will be conducted over 4-5 weeks at a convenient location in the study area. Training dates will be selected with input from the in-charges to minimize disruption to the daily operations of the health facilities.

5.3.2.2. PCS for support staff

The PCS workshop for support staff will consist of one half-day module using a combination of learning approaches. The module is a simplified version of the clinical staff PCS workshop on improving the patient visit and will include the presentation of didactic material on the theme; appreciative enquiry exercises; role-plays or other participatory activities; and group discussion. The module themes and learning outcomes are outlined in Table 6.

The half-day workshop will be approximately 3 hours in length and will be conducted over 1 week at a convenient location in the study area. Training dates will be selected with input from the in-charges to minimize disruption to the daily operations of the health facilities.

Table 6: Support staff PCS module theme and learning outcomes

**PCS for support staff – Improving the patient visit**	Support staff will be able to:<br>• Understand the meaning and importance of providing patient centered services<br>• Implement strategies to improve the welcome of patients at the health facility to patients<br>• Implement strategies to ensure patients are seen fairly<br>• Implement strategies to improve the orientation of patients at the health center<br>• Appropriately utilize volunteers to address current staffing gaps<br>• Contribute to improving patient satisfaction by working together with all clinical and support staff

5.4. CONSUMABLES

Results from the formative research indicate that drug shortages and stock-outs at health facilities are a major problem, especially for AL, the first-line treatment for uncomplicated malaria. Both the quantitative and qualitative research show that these shortages have an impact on community members' perceptions of care provided at health centers and health workers' ability to provide good quality care. In addition, most health centers lack laboratory facilities and currently do not have the capacity to confirm the diagnosis of malaria. A major aim of the HFI is to ensure a continuous and adequate supply of drugs and RDTs for malaria.

5.4.1. Drug delivery from National Medical Stores

In Uganda, the National Medical Store (NMS) has recently adopted a new 'modified push' system for distributing drugs to lower-level health centers. Districts are to make an annual procurement plan, and NMS will deliver the drugs every 2 months, based on that plan. If no additional requests are made by health facilities or the district, NMS will provide a standard delivery of drugs based on the procurement plan, regardless of health facility need or disease prevalence. Once the procurement plan is developed, and annual orders have been made, health facilities will have limited power to change the drug deliveries.

As part of the HFI, in-charges will be training in drug supply management as outlined in section 5.2.3 and will be encouraged to complete drug stock cards and the ADDAT form (Appendix D) to help track the distribution of drugs from NMS.

5.4.2. Supply artemether-lumefantrine

If the amount of AL provided to the health centers by NMS is not adequate to meet demand, or if the procurement of AL fails, the project will supply supplemental AL obtained from Joint Medical Stores, Quality Chemicals, or other suitable supplier in Uganda. Batch numbers, expiry dates, date received, and date opened will be recorded for all AL supplied.

5.4.3. RDTs for malaria

Most health centers do not have access to a laboratory or malaria diagnostic facilities. As part of the HFI package, we will ensure adequate supplies of RDTs at all health centers. One of the HRP2-based tests, are recommended in the most recent WHO / FIND Product Testing Report (http://www.wpro.who.int/sites/rdt/home.htm) and are approved by the Uganda National Drug Authority, will be used. Batch numbers, expiry dates, date received, and date opened will be recorded for all RDTs supplied. RDTs may be introduced into lower-level health centers by the MoH. If so, we will track distribution and use of RDTs in a manner similar to that of drugs, as described above, and will supplement distribution of RDTs if needed.

6 STANDARD CARE

6.1. OVERVIEW

In this study, 10 health facilities will be randomly assigned to the standard care arm. Standard care will include services typically provided by government-run facilities.

6.2. STAFFING AND PATIENT ATTENDANCE

In our formative research, we interviewed 17 health workers, including the in-charges of health centers, to gain an understanding of the current situation at the local government-run health facilities. Not all health centres are open every day, depending on the availability of drugs and staff. The mean number of patients visiting the health centres each day is approximately 50-60, and staffing shortages are an issue at most health centers (Table 7).

Table 7: Patient attendance and staffing, stratified by level of health centre

	**HC II**<br>**N=12**	**HC III**<br>**N=3**
Patients visiting (mean, SD, range)	51.2 (20.4) 25-100	52.5 (15) 30-60
Staff stationed (mean, SD, range)	2.08 (0.66) 1-3	9.75 (0.95) 9-11
Staff available (mean, SD, range)	1.4 (0.51) 1-2	4.25 (0.96) 3-5

6.3. INFRASTRUCTURE AND SUPPLIES

Infrastructure at the health centres in this area is limited; most lack electricity and running water. Drug stock-outs are a common problem at HC IIs and IIIs. Most centres, but not all, stock AL but few centres stock artesunate + amodiaquine, the alternative treatment for uncomplicated malaria. Stock-outs of both parenteral and oral quinine are also common. Malaria is diagnosed clinically, without laboratory confirmation at most health centres, even at the two HCIIIs with laboratories.

6.4. STANDARD CARE

Standard care will include services typically provided by government-run facilities; we will not provide any additional support to these facilities. Health care will be provided to patients attending these facilities according to the usual standards; in-charges will continue to manage the facilities using their standard approach, no additional training will be provided to the health workers stationed at these facilities; and no support for staffing or supplies will be provided beyond what is supplied by the district and MoH.

7 CROSS-SECTIONAL SURVEYS

7.1. OVERVIEW

Cross-sectional surveys will be conducted in randomly selected children under-five, and those between the ages of 5-15 years of age. The number of children sampled will be weighted according to the total population of each cluster to achieve a harmonic mean of 200 for each age category. A total of 8766 children will be sampled in each survey. Surveys will be conducted at baseline and then annually for each year; new populations of children will be selected for each survey. The survey will include a structured questionnaire administered to the primary caregiver, and a clinical and laboratory assessment of each child.

7.2. DEFINITIONS

Household: A household will be defined as any single permanent or semi-permanent dwelling structure acting as the primary residence for a person or group of people that generally cook and eat together. Some households may include members who sleep in other dwelling structures within the same compound, if the members are still dependent on the head of household in the main household.

Household resident: A resident within each household will be defined as a person who intends to have a sleeping place primarily at that location for a period of the next 6 months. This may include people who sleep in a separate house within the same compound, if they are still dependent on the head of household for decisions on finances and health care.

7.3. ENROLLMENT

7.3.1. Recruitment

All households enumerated during the census will be assigned a unique number. A random sample of households with at least one child under fifteen years of age will be selected from each cluster to generate a list of households to be approached. Three separate lists will be generated prior to each survey from the original census list. Study personnel will conduct door-to-door screening to identify those households with at least one child of appropriate age. Households without a child of appropriate age will be removed from the recruitment list. Residents not home during the initial contact will be re-visited on at least three other occasions over a six-week period before eliminating them from our sample selection process.

7.3.2. Screening

When a household with at least one child of appropriate age is identified, study personnel will briefly describe the purpose of the study in the appropriate language (usually Japadhola, Luganda or Swahili) with parent(s) or guardian(s), and proceed with screening (Appendix K). The inclusion criteria are: 1) age < 15 years, 2) agreement of parents or guardians to provide informed consent, and 3) agreement of a child aged 8 years or older to provide assent. The exclusion criterion is: 1) inability to locate the child. One child under five and one child between the ages of 5-15 years from each household will be eligible for participation. If more than one child of appropriate age resides in the household, study personnel will record the gender and ages of all children under five and all children between the ages of 5-15 years, and one child from each age category will be randomly selected for participation. If only one child of appropriate age, or only one child in each age category, resides in the household they will be selected for participation. Previous participated in one of the cross-sectional surveys will not be an exclusion criterion; children who have previously participated in a survey will still be eligible to participate.

7.3.3. Informed Consent

Study personnel will conduct the informed consent discussion with the parent(s) or guardian(s). Informed consent will be conducted in the appropriate language and a translator will be used if necessary. Consent forms will be available in English and the local languages (Appendix L & M). Following the informed consent discussion, parents/guardians will be asked by the study personnel to sign a written consent form for their child to participate in a research study and a second approved consent form for the future use of biological specimens obtained during the course of the study in the baseline CSS. If the parent/guardian is unable to read or write, their fingerprint will substitute for a signature, and a signature from a witness to the informed consent procedures will be obtained. Written assent to participate in the study will also be obtained from children aged 8 years and older at the time of screening (Appendix N). In the repeat CSS, only Appendices L and N will be used.

7.3.4. Enrollment

Children who fulfil the eligibility criteria will be assigned a study number and will undergo the survey procedures outlined below. If a child selected to participate is not at home on the day of the initial visit, the household will be re-visited on at least three other occasions over a six-week period before excluding the child.

7.4. SURVEY PROCEDURES

7.4.1. Survey questionnaire

Primary caregivers of selected children will be asked to complete a survey questionnaire to gather information about bednet use and management of child who was febrile within the last two weeks, including source of care, diagnostics test results, drug treatment, and actions taken for management of illnesses (Appendix O, Part 1). Data will be collected about management of fever in any child

under the primary caregiver's care, and will not be restricted to children selected to participate in the survey. If more than one child has been febrile in the last two weeks, data will be collected on management of the most recent illness. If the primary caregiver is not available on the day of the initial visit, the household will be re-visited on at least three other occasions over a six-week period to administer the questionnaire. If the primary caregiver is not available after four visits, the survey questionnaire will not be completed. However, the child or children selected for the cross-sectional survey from that household will not be excluded, and all additional information collected will be utilized. In the last annual cross-sectional survey, all women of child-bearing age (13-49 years) in the household will be asked to provide birth histories, which will allow us to estimate all-cause mortality in children under five (Appendix O, Part 2).

7.4.2. Clinical and laboratory assessment

Participating children will undergo a brief history and physical examination, including measurement of temperature, height, weight, mid-upper arm circumference, and spleen size (Appendix P). Blood will be collected by fingerprick for thick blood smear, hemoglobin, and for storage on filter paper for future molecular testing. In the repeat CSS, filter paper samples will not be collected.

7.4.3. Management of ill children

Children enrolled in the survey who report fever in the past 48 hours, or who have a temperature of $\geq$ 38.0^{o}C will have an RDT performed. Febrile children will be treated with paracetamol as appropriate. Children with a positive RDT and no evidence of severe malaria will be treated with AL. Children with a positive RDT and evidence of danger signs of severe disease will be referred for further evaluation and treatment. Children with a hemoglobin level of < 5.0 g/dL will be referred for further evaluation and transfusion. Any child with other concerning clinical symptoms will also be referred to an appropriate health care facility at the discretion of the study personnel.

8 COHORT STUDY

8.1. OVERVIEW

A cohort of children under five will be enrolled from 25 households randomly selected from each cluster, for a total of 500 households. The cohort will be dynamic, in that all children within a household, who are under the age of five and who meet selection criteria, will be included. A household survey will be conducted at the start of the study. Children will undergo clinical and laboratory assessments at baseline and then every six months. Primary caregivers will be asked to prospectively collect information on the clinical symptoms of participating children and expenditures for health care using pictorial diaries. Study personnel will visit the households monthly to collect the diaries and administer a monthly questionnaire. Participants will be followed for approximately 18 months in total, the equivalent of approximately 12 months following roll-out of the intervention. At the end of study follow-up, all children enrolled from a participating household will be discontinued at the same time. Thus, children enrolled through dynamic recruitment will be followed for less than 18 months. Households will be discontinued at the 18-month clinical visit, or soon thereafter.

8.2. ENROLLMENT

8.2.1. Recruitment

All households enumerated during the census will be assigned a unique number. A random sample of households with at least one child under five will be selected from each cluster prior to randomization to generate a list of households to be approached. Study personnel will conduct door-to-door screening to identify those households with at least one child of appropriate age. Households without a child of appropriate age will be removed from the recruitment list. Residents not home during the initial contact will be re-visited on at least three other occasions over a six-week period before eliminating them from our sample selection process. When a household with at least one child of appropriate age is identified, study personnel will briefly describe the purpose of the study in the appropriate language (usually Japadhola, Luganda or Swahili) with parent(s) or guardian(s). If the parent(s)/guardian(s) are interested in the study, the study personnel will schedule an appointment date for screening. Residents not home during the initial contact will be re-visited on at least 3 other occasions over a 6-week period before eliminating them from our sample selection process. All children of appropriate age from a single household will be eligible for evaluation for study enrollment.

8.2.2. Screening

Children will be screened at convenient sites within the community. Interviews will be conducted in the appropriate language with parents or guardians. Selection criteria are based on the goal of recruiting a representative sample of children from our target population (Appendix Q). The

inclusion criteria are: 1) age < 5 years, and 2) agreement of parents or guardians to provide informed consent. The exclusion criteria are: 1) intention to move during the follow-up period, and 2) current enrollment in another research study. During the screening process, study personnel will assess for initial eligibility criteria through conversation with the parent/guardian (including age of the child, willingness of the parent/guardian to participate in the study and to provide informed consent, and intention to move from Tororo). If the initial screening criteria are met, the parent/guardian will be asked to provide informed consent for their child to participate in the study. If parents/guardians are undecided about consenting for their child (or children) to participate in the study at the initial screening visit, they will be allowed up to one week to make a final decision about study participation.

8.2.3. Informed Consent

Study personnel will conduct the informed consent discussion with the parent(s) or guardian(s). Informed consent will be conducted in the appropriate language and a translator will be used if necessary. Consent forms will be available in English and the local languages (Appendices R & S). Following the informed consent discussion, parents (or guardians) will be asked by the study personnel to sign a written consent form approved by the IRBs for their child to participate in a research study and a second approved consent form for the future use of biological specimens obtained during the course of the study. If the parent or guardian is unable to read or write, their fingerprint will substitute for a signature, and a signature from a witness to the informed consent procedures will be obtained.

8.2.4. Enrollment

Children who meet the eligibility criteria will be assigned a study number, and will undergo a clinical and laboratory evaluation. Height, weight, and temperature will be measured, and spleen size will be evaluated (Appendix T). Blood will be collected by finger prick for thick blood smear, hemoglobin, and for storage on filter paper for future molecular testing.

8.2.5. Management of ill children at enrollment

Children enrolled in the cohort study who report fever in the past 48 hours, or who have a temperature of $\geq 38.0^{o}C$ will have an RDT performed. Febrile children will be treated with paracetamol as appropriate. Children with a positive RDT and no evidence of severe malaria will be treated with AL. Children with a positive RDT and evidence of danger signs of severe disease will be referred for further evaluation and treatment. Children with a hemoglobin level of < 5.0 g/dL will be referred for further evaluation and transfusion. Any child with other concerning clinical symptoms will also be referred to an appropriate health care facility at the discretion of the study personnel.

8.3. HOUSEHOLD SURVEYS

Following enrollment, or within a 2 week period from the date of enrollment, a household survey will be performed at all participating households (Appendix U). Primary caregivers will be asked to complete a survey questionnaire to gather information about bednet use and management of a child

who was febrile within the last two weeks, including source of care, diagnostics test results, drug treatment, and actions taken for management of illnesses. Data will be collected about management of fever in any child under the primary caregiver's care, and will not be restricted to children selected to participate in the cohort study. If more than one child has been febrile in the last two weeks, data will be collected on management of the most recent illness. The household survey will then be repeated approximately 12 months after enrollment (Appendix UII). If the primary caregiver is not available on the day of the initial visit, the household will be re-visited on at least three other occasions over a six-week period to administer the questionnaire. If the primary caregiver is not available after four visits, the household survey questionnaire will not be completed. However, the child or children selected for the cohort study from that household will not be excluded, and all additional information collected will be utilized.

8.4. FOLLOW-UP

8.4.1. Household diaries and monthly visits

Primary caregivers will be asked to keep a diary of health of study participants for the duration of the study (Appendix V). The diaries will be based on instruments previously developed and validated in studies in Uganda and elsewhere in Africa.[92,95] The diaries have been developed by a Ugandan artist with input from the community, and will be adapted to the local setting if necessary. The diaries will be used to collect information on clinical symptoms and health care expenditures. Households will be visited by study personnel every two weeks during the first two months, and then monthly, to collect completed diaries. At each monthly visit, questionnaires will also be administered to gather additional data on the health of the study participants, management of any illnesses, and health care expenditures (Appendix W). The information collected in the diaries and the questionnaires will be complementary. Study personnel will review the diaries with the primary caregivers at each monthly visit and caregivers will be allowed to refer to the diaries while the questionnaire is administered to help prompt their memory. Small incentives (including sugar, soap, or washing powder) will be provided to each household during the monthly visit to encourage completion of the diaries.

8.4.2. Follow-up evaluations

Clinical and laboratory evaluations will be repeated every 6 months over the period of study follow-up in all cohort study participants (Table 8). At the end of study follow-up, all households will be given an insecticide-treated bednet (ITN), and each participating child will be given a small token of appreciation, such as a cup or plastic sandal shoes.

8.4.3. Management of ill children during follow-up

At the follow-up assessments, children who report fever in the past 48 hours, or who have a temperature of $\geq$ 38.0^{o}C will have an RDT performed. Febrile children will be treated with paracetamol as appropriate. Children will be managed as described above in section 7.2.5. Any child with concerning clinical symptoms will be referred to an appropriate health care facility at the discretion of the study personnel.

**Table 8. Study objectives and outcome measures**		
Evaluation	Baseline	Every 6 months
Clinical evaluations		
Height, weight	X	X
Temperature	X	X
Spleen size	X	X
Laboratory evaluations		
Thick blood smear	X	X
Hemoglobin	X	X
Filter paper sample	X	X
Household evaluations		
Household KAP survey	X	(~12 months post enrollment)
Pictorial diaries	—	Monthly
Household questionnaires	—	Monthly

9 M&E OF HEALTH FACILITY INTERVENTION

9.1. OVERVIEW

We will conduct monitoring and evaluation (M&E) activities at all government-run health facilities in the study area, including those that are randomized to the intervention and those that are not. Patient exit interviews will be conducted to gather information on rational use of ACTs and patient satisfaction. In addition, standardized information will be captured from all facilities including data on patient attendance, drug stock-outs, and staffing shortages every month. Additional, detailed information on RDT utilization and ACT prescribing practices will also be collected from HFI facilities. A knowledge questionnaire will be administered annually to consenting health workers at all facilities. M&E activities will begin within one month after the roll-out period for the HFI has been completed.

9.2. PATIENT EXIT INTERVIEWS

9.2.1. Overview

Exit interviews will be conducted with children under five and their caregivers at all health facilities. The purpose of the interviews is to evaluate for rational prescribing of ACTs, and to determine the level of satisfaction with the health facility visit. Three rounds of surveys are planned. In the first two surveys, 10 patients will be selected by convenience sampling from each facility to participate in the interviews (200 total per survey). In the final survey, 50 patients will be recruited to participate (1000 total in survey). In total, 1400 patients will participate in the interviews.

9.2.2. Recruitment

As children and their caregivers are leaving the health facility, study personnel will ask the caregiver if their child is under five and if they would be willing to participate in an interview regarding the health care visit (Figure 4). When a caregiver with a child of appropriate age is identified, study personnel will briefly describe the purpose of the study in the appropriate language (usually Japadhola, Luganda or Swahili) with parent(s) or guardian(s). The inclusion criteria are: 1) age < 5 years, and 2) agreement of parents or guardians to provide informed consent (Appendix X). There are no exclusion criteria. If more than one child of appropriate age was seen at the health facility that day, all children will be eligible to participate.

9.2.3. Informed Consent

Study personnel will conduct the informed consent discussion with the parent(s) or guardian(s). Informed consent will be conducted in the appropriate language and a translator will be used if necessary. Consent forms will be available in English and the local languages (Appendix Y). Following the informed consent discussion, parents/guardians will be asked by the study personnel to sign a

written consent form for their child to participate in a research study. If the parent/guardian is unable to read or write, their fingerprint will substitute for a signature, and a signature from a witness to the informed consent procedures will be obtained.

Figure 4. Patient exit interview algorithm

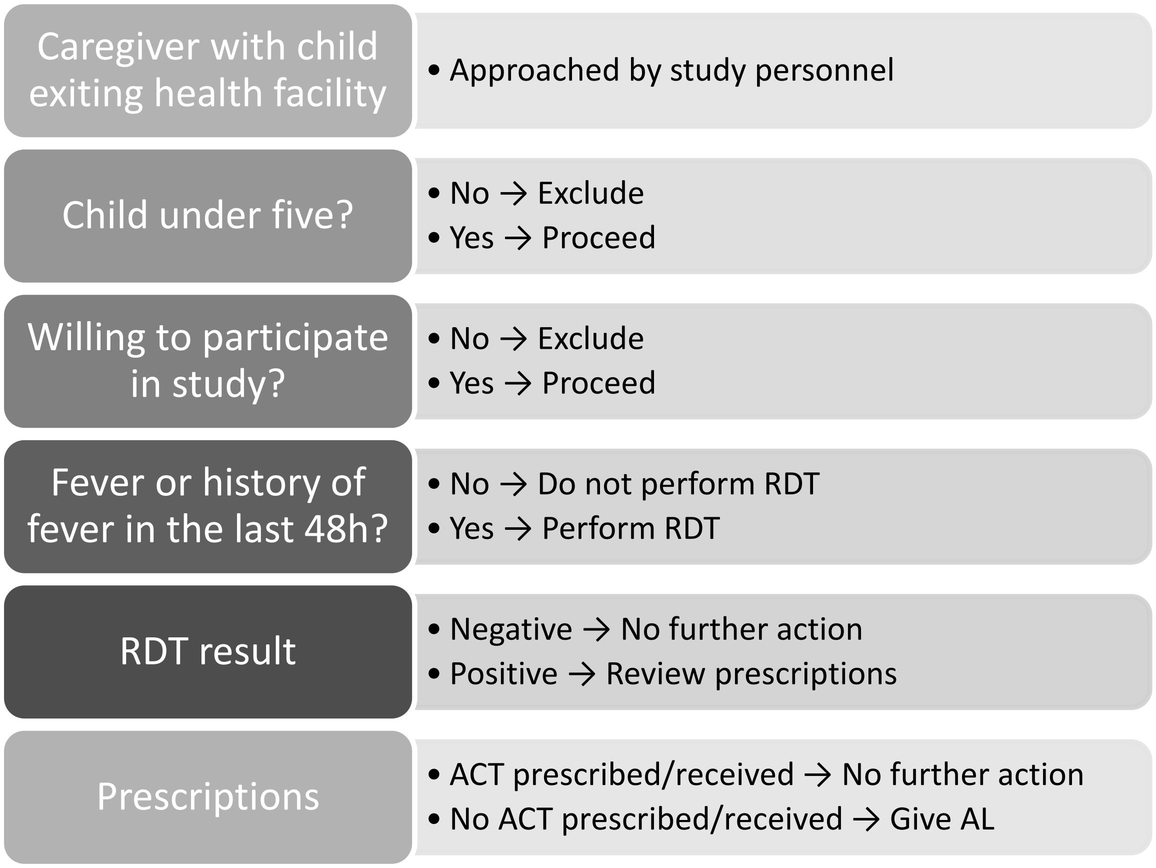

9.2.4. Interview

Children who fulfil the eligibility criteria will be assigned a study number and will undergo the survey procedures. Caregivers of enrolled children will be interviewed using a standardized questionnaire (Appendix Z) to gather information about the purpose of the visit, presenting complaint, the child's symptoms, whether a RDT or blood smear was done, the diagnosis given, medications prescribed, and medications received. Additional information about the satisfaction of the caregiver with the visit to the facility will also be obtained.

9.2.5. RDT results

If a child has a temperature of >38.0^{o}C or a history of fever in the past 48 hours, a fingerprick blood sample will be obtained to perform a RDT. Febrile children will be treated with paracetamol as appropriate. The results of the RDTs performed by the study personnel will be compared to the results of RDTs performed by health facility staff, where possible. Children with a positive RDT and no evidence of severe malaria, who have not been prescribed or received an ACT, will be given AL (Figure 4). Children with a positive RDT and evidence of danger signs of severe disease will be

referred to an appropriate facility for further evaluation and treatment, regardless of the medicine prescribed.

9.3. HEALTH FACILITY SURVEILLANCE

9.3.1. All facilities

We plan to conduct routine surveillance at all health facilities approximately every 1-3 months. The purpose of the surveillance is to collect information about patient attendance, drug stocks, and health facility costs from all facilities. Study personnel will collect the information during a routine visit to the health facilities. The in-charge of the facility will be approached and informed about the surveillance activities. An information sheet (Appendix AA) will be used to describe the purpose of the activities and verbal consent to collect information will be obtained from the in-charge. Data on patient attendance including date visited, village of residence, age, sex, and diagnoses will be collected from the out-patient registers (Appendix BB, Part 1, Section 2). Data on stocks of AL will be collected from the stock cards at each health facility. If patient registers are completed and the in-charge agrees, the patient registers may be taken to the PRIME study office for data entry and then returned to the health facilities. The patient register and drug stock cards are standard HMIS data collection tools, and are in place in all health facilities. Study personnel will work with the MoH, district officials, and in-charges of health facilities to ensure that the registers capture the essential information. Indicators that will be tracked at all health facilities will include (Appendix BB, Part 2):

— Number of patients seen at the facility
— Number of children under five
— Number of patients with fever
— Number of patients prescribed an ACT
— Number of patients diagnosed with malaria

The surveillance form will also be used to collect information on health facility costs (Appendix BB, Part 3) including 1) drugs & supplies, 2) overheads (utilities, capital equipment, space), and 3) staff allocation and activities. Part 3 will only be conducted once during the course of the study.

9.3.2. HFI facilities

Additional information on utilization of RDTs and use of ACTs will be collected at facilities randomized to the HFI and any other facilities where RDTs are available (Appendix BB, Part 2). This information will be extracted from the out-patient registers and drug stock cards by study personnel. The data collected will complement the information collected in the health center management training module (section 5.2.4). Additional indicators evaluated in the health facility surveillance will include:

— Number (proportion) of patients who had a RDT performed
— Number (proportion) of patients with a positive RDT
— Number (proportion) of patients diagnosed with malaria
— Number (proportion) of patients diagnosed with malaria by RDT result
— Number (proportion) of patients with a positive RDT prescribed an ACT
— Number (proportion) of patients with a negative RDT prescribed an ACT
— Number (proportion) of patients without a RDT done prescribed an ACT

9.3.3. Out-patient registers

Staff at all health facilities will be expected to complete the out-patient registers (OPD) provided by the MoH as part of HMIS. For this study, the standard HMIS forms have been modified slightly to include two additional columns to capture individual patient data on history of fever, temperature, whether an RDT was performed, and if so, the result. To help acknowledge the role that the in-charges and other members of staff play in completing the additional columns in the OPD, and in supporting our surveillance activities by providing information to complete Appendix BB, we propose to provide periodic recognition to all health facilities, including tea, coffee, sugar, milk, soap, and pens.

9.4. HEALTH WORKER KNOWLEDGE QUESTIONNAIRES

Questionnaires will be administered to health workers at all health facilities following implementation of the intervention to assess their knowledge about fever case management. All available health workers will be approached to participate in the knowledge questionnaire. Information sheets will be used to describe the purpose of the knowledge questionnaire and verbal consent will be obtained from the health care worker before conducting the questionnaire (Appendix CC). The knowledge questionnaire includes structured questions allowing for open-ended answers to assess the health workers' knowledge about malaria transmission, symptoms, diagnosis, and treatment, and etiology of non-malaria fevers (Appendix DD). Additional assessment of health worker knowledge will be carried out using the pre- and post-training questionnaires administered by the JUMP team in their training on fever case management at approximately one year after the roll-out of the intervention (Appendix E). Approximately two years after implementation of the intervention, assessment of health worker knowledge and skills for performing RDTs will be conducted using a modified version of Appendix E. Additional information about prior training on use of RDTs will be gathered, and health workers will be observed performing an RDT and managing a child with suspected malaria. Information sheets will be used to describe the purpose of the assessment to caretakers of children participating in the observation exercise (Appendix II).

10 ADVERSE EVENT MONITORING

10.1. OVERVIEW

Adverse event monitoring will be conducted in children enrolled into the cohort study. Data on serious adverse events and suspected adverse drug reactions will be collected retrospectively during the monthly interviews. Reports of serious adverse events that are classified as at least 'possibly' related to administration of AL, including those classified as possibly, probably, or definitely related, and reports of all 'Suspected Unexpected Serious Adverse Reactions (SUSARs) will be submitted to the IRBs according to their guidelines for expedited reporting. All serious adverse event reports and summary reports of suspected adverse drug reactions will be submitted to the IRBs, the Data and Safety Monitoring Board (DSMB), and the ACT Consortium Drug Safety Register (ACTcDSR) annually.

10.2. DEFINITIONS

Serious adverse event (SAE): An experience that results in any of the following outcomes:

- Death during the period of study follow-up
- Life-threatening experience (one that puts a participant at immediate risk of death at the time of the event)
- Inpatient hospitalization during the period of study follow-up
- Persistent or significant disability or incapacity
- Specific medical or surgical intervention to prevent one of the other serious outcomes listed in the definition.

Adverse drug reaction (ADR): A response to a medicine which is noxious and unintended, and which occurs at doses normally used in man.

Suspected Unexpected Serious Adverse Reactions (SUSAR): An ADR that is suspected of having a causal relationship to the trial medication and is unexpected.

10.3. IDENTIFICATION OF ADVERSE EVENTS

At each monthly visit, study personnel will inquire about the occurrence of any SAEs and any suspected ADRs. Two severity grading scales will be used to grade severity of events: 1) the WHO toxicity grading scale, which will be used by all projects involved in the ACT Consortium, and 2) a severity grading scale used previously in clinical trials in Uganda, which is based on the WHO toxicity grading scale and the National Institutes of Health, Division of Microbiology and Infectious Diseases grading scales (Appendices EE & FF).

10.4. REPORTING OF ADVERSE EVENTS

For each SAE and suspected ADR identified, an adverse event report form will be completed (Appendix GG). The following information will be recorded for all experiences that are reported:

- Description of the patient (ID number, age, sex, weight)
- Description of the adverse event
- Date of event onset and date of resolution
- Date event reported
- Maximum severity of the event
- Causality and expectedness
- Whether the event was serious
- Outcome of the event
- Drug information (names, doses, dates administered for all drugs taken one month prior)
- Past medical history
- Known allergies and/or prior experience with drugs taken

All adverse event report forms will be entered into a database and reported annually to the IRBs, the DSMB, and the ACTcDSR. Reports of serious adverse events that are classified as at least 'possibly' related to administration of AL and reports of all 'Suspected Unexpected Serious Adverse Reactions (SUSARs) will be submitted to the IRBs according to their guidelines for expedited reporting.

10.5. ACT CONSORTIUM DRUG SAFETY REGISTER

10.5.1. Overview

Safety data collected in our study will be submitted to the central ACT Consortium Register within timescale agreed by the Consortium's Steering Committee. The Steering Committee will ensure the timely dissemination of any early warnings of emerging patient safety information within the project to all parties in the Consortium and to other relevant stakeholders. All individual patient data transferred to the ACT Consortium Register will be identifiable by study number only. No names or addresses of individuals will be stored. The adverse event data will be coded centrally using industry-standard MedDRA (Medical Dictionary of Drug Regulatory Authorities) coding systems.

10.5.2. Database

The database will fully comply with ICH – E6 Guideline for Good Clinical Practice,[96] in particular for the handling of electronic data (section 5.5.3), and will:

- Ensure and document that the electronic data processing system(s) conform to the sponsor's established requirements for completeness, accuracy, reliability, and consistent intended performance (i.e. validation).
- Maintain standard operation procedures for using these systems.
- Ensure that the systems are designed to permit data changes in such a way that the data changes are documented and that there is no deletion of entered data (i.e. maintain an audit trail, data trail, edit trail).

— Maintain a security system that prevents unauthorized access to the data.
— Maintain a list of the individuals who are authorized to make data changes
— Maintain adequate backup of the data.
— Safeguard the blinding, if any (e.g. maintain the blinding during data entry and processing).
— The individual patient data transferred to the central pharmacovigilance team will be identifiable by study number only. No names and addresses of individuals will be stored.

10.5.3. Mechanisms for data handling/sharing:

Each study will have ethical approval from all overseeing institutions, and sign an agreement regarding the data fields to be shared with the central database prior to the transfer of data to the Liverpool School of Tropical Medicine (LSTM), who will manage the database. The agreement will be voluntary between the ACT Consortium study institution and the ACTc Pharmacovigilance database institution (LSTM). The study institution will remain the owner of their data at all times and the data will be used for pharmacovigilance monitoring within the ACT Consortium. Any future requests for information from the central database will require the permission of the owners of the data to enable access to the collated dataset.

11 LABORATORY PROCEDURES

11.1. MICROSCOPY

Thick blood smears will be stained with 2% Giemsa for 30 minutes and read by experienced laboratory technologists who are not involved in direct participant care. For the cohort study and first cross-sectional survey, parasite densities will be calculated by counting the number of asexual parasites per 200 leukocytes (or per 500 leukocytes, if the count is <10 asexual parasites/200 leukocytes), assuming a leukocyte count of 8,000/μl. A blood smear will be considered negative when the examination of 100 high power fields does not reveal asexual parasites. For the second and final cross-sectional survey, thick blood smears will be read only for presence or absence of asexual parasites, parasite density will not be determined. Gametocytemia will also be determined from thick smears. For quality control, all slides will be read by a second microscopist and a third reviewer will settle any discrepant readings.

11.2. RAPID DIAGNOSTIC TESTS FOR MALARIA

We will use RDTs when assessing febrile participants in the cross-sectional surveys, the cohort study, and in the patient exit interviews. In addition, we plan to support the distribution of RDTs to the HFI facilities. If RDTs are not distributed by the MoH to lower-level health facilities, we will provide RDTs to all HFI facilities. If RDTs are provided by the MoH, we will supply RDTs only when stock-outs occur. HRP2-based RDTs that have been approved by in the latest round of WHO / FIND product testing (http://www.wpro.who.int/sites/rdt/home.htm) will be used.

Each RDT will be interpreted by a health worker, and the results will be recorded in the appropriate log book. An RDT result will be considered positive if both the control line and the test line are visible after the development time. A result will be considered negative if the control line is visible, but no test line appears. The result will be considered invalid if no control line is visible, regardless of whether or not a test line appears.

11.3. HEMOGLOBIN MEASUREMENT

Hemoglobin will be measured from fingerprick blood samples using a portable spectrophotometer (HemoCue, Anglom, Sweden).

11.4. MOLECULAR STUDIES

Each time a thick blood smear is obtained in the baseline cross-sectional survey and cohort study blood will also be collected onto filter paper. Samples will be collected by fingerprick sampling. Blood will be placed onto filter paper in approximately 25 ul aliquots per blood spot (4 blood spots per sample). The samples will be labeled with study numbers and dates, air-dried, and stored in

small, sealed sample bags at ambient temperature with desiccant. Parasite DNA will subsequently be removed from the filter paper and prepared for molecular analysis using a chelex extraction method. Molecular studies may include analyses of polymorphisms in parasite and/or human genes for mutations that may impact on clinical malaria, and genotyping of malaria parasites. Molecular studies will be performed only for research purposes and will have no impact on the clinical management of study participants.

12 STATISTICAL ISSUES

12.1. SAMPLE SIZE CALCULATIONS

12.1.1. Cluster randomized study

Cross-sectional surveys of children under five, and children aged 5-15 years will be conducted annually. The primary outcome for the cluster randomized study will be the prevalence of anemia (hemoglobin < 11.0 g/dL) in children under five as measured in the cross-sectional surveys, which is assumed to be 65% at baseline estimated from data collected in a household survey conducted Mulanda sub-county in West Budama South (Rachel Pullen, unpublished data). We plan to test the primary hypothesis that children under five randomized to the HFI will have a lower prevalence of anemia than children randomized to receive standard care. There is some variability in total cluster sizes so that sampling an equal number of children from each cluster (the most efficient design for a cluster randomized trial) while maintaining power to detect differences between intervention arms is logistically infeasible. Therefore children will be sampled from each cluster in proportion to the total cluster size, with a planned harmonic mean of 200 children sampled from each cluster (Table 9). With two study intervention arms, 10 clusters per intervention, a coefficient of variation between clusters of 0.2, and allowing for the stratified design, a harmonic mean of 200 children under five surveyed in each cluster will allow us to detect a difference in anemia prevalence between the two intervention arms of 17%, (or more) with power of 80% and significance of 5. If we assume that the clusters are more homogeneous, with a coefficient of variation of 0.1, then a difference of about 10% (or more) can be detected. As a secondary outcome, we will evaluate the prevalence of parasitemia in children under five, and in children aged 5-15 years. We will test the secondary hypothesis that children under five, and children aged 5-15 years, that are randomized to health facility-based care will have a lower prevalence of parasitemia than children randomized to receive standard care. In the older age group, the prevalence of parasitemia is estimated to be 60% at baseline based on data collected in Mulanda sub-county (Rachel Pullen, unpublished data). Children aged 5-15 years will also be sampled from each cluster proportionally to the total cluster size. Again, with two study intervention arms, 10 clusters per intervention, and a coefficient of variation between clusters of 0.2, a harmonic mean of 200 children aged 5-15 years surveyed in each cluster will allow us to detect a difference in parasitemia prevalence in the two intervention arms of 16%, with power of 80% and significance of 5%.

Table 9. Target sample size by cluster			
Cluster	Health facility	Target sample size per age group	Total sample size per cluster
1	Maundo	261	522
2	Were	216	432
3	Katajula	247	494
4	Paya	215	430
5	Pusere	216	432
6	Nawire	228	456
7	Kirewa	227	454
8	Chawolo Kirewa	255	510
9	Kisoko	323	646
10	Morkiswa	245	490
11	Petta	192	384
12	Makawari	228	456
13	Mbula	218	436
14	Gwaragwara	261	522
15	Osia	136	272
16	Mwelo	221	442
17	Lwala	106	212
18	Panyangasi	255	510
19	Mudodo	240	480
20	Chawolo Mulanda	93	186

12.1.2. Cohort study

The primary outcome for the longitudinal cohort study will be treatment incidence density, which is assumed to be 2.5 treatments per year at baseline. We plan to recruit 500 households into the longitudinal study (25 in each cluster), and will recruit all children under five years in the selected households. The cohort is dynamic, so children who are born or move into participating households will be recruited, and children who die or move out of participating households will no longer contribute to follow-up. We conservatively estimate that at any one time the average number of children under five per household will be at least 1.6. Given this, 250 households, and at least 400 children at any one time, will be available in each intervention group. Since the cohort is dynamic, no allowance for losses to follow-up is required. A follow-up period of 1 year will result in 400 child-years of follow-up in total per study arm; shared between 10 clusters, this will result in 40 person-years per cluster on average. If the baseline group experience an average of 2.5 treatments per child per year, then a difference of one treatment on average over 1 year of follow-up can be detected between the two interventions (for example if the HFI group experiences 2.5 treatments during the year and the standard care group experiences 3.5 treatments during the year assuming a coefficient of variation between clusters of 0.2, power of 80%, significance level of 5%, and allowing for the stratified design.

12.1.3. Patient exit interviews at health facilities

The primary outcome for the patient exit interviews will be the proportion of children under five with suspected malaria, who are not treated according to malaria treatment guidelines during their

visit to the health facility, based on the result of a RDT performed by the PRIME study staff that is either inappropriately receiving an ACT with a negative RDT result, or not receiving an ACT with a positive RDT. The hypothesis is that the proportion of children inappropriately treated with an ACT will be lower in the facilities randomized to the intervention arm compared to those in the standard care group. Assuming this proportion to be 35% in the standard care group, interviewing 50 children and their caregivers in each of the 20 clusters will give 80% power to detect a difference in the proportion inappropriately treated between the two intervention arms of 12% (or more) at the 5% significance level, assuming a coefficient of variation between clusters of 0.2 and allowing for the stratified design.

12.2. ANALYTICAL PLAN

12.2.1. Analytical approach

Since this is a cluster randomised trial, analysis will be conducted at both the cluster level using summary statistics from each cluster, and at the individual level. The primary analysis will be based on the cluster-level results as this is expected to be more robust when the number of clusters randomised is not large. A two-stage approach based on cluster summaries will be used to adjust for individual- and cluster-level covariates, where appropriate.

Analysis will be conducted primarily on an intention to treat (ITT) basis, where data collected will be analysed according to the assigned cluster. To address potential contamination issues due to children attending health facilities other than the one that defines their cluster, i.e. the health facility they live closest to, we shall also analyse data on a per protocol (PP) basis. For this analysis, data collected will be analysed according to the facility at which the child accesses healthcare.

12.2.2. Baseline characteristics and trial profile

Baseline demographic characteristics will be available from the census survey conducted in the first phase of the project, the baseline cross-sectional survey, and the household survey for the cohort study. Data from each source will be separately tabulated by trial arm and by stratum. A trial profile will be produced. This will show, by trial arm, the numbers of households screened, eligible and enrolled for the cross-sectional surveys, plus numbers not enrolled (with reasons); the numbers of children within each age group (<5 years and 5-15 years) recruited for the cross-sectional surveys. For the cohort study, the trial profile will show the numbers of households and children screened, eligible and enrolled, plus numbers not enrolled (with reasons). The number of six-monthly visits at which clinical evaluations were conducted and the number of monthly visits at which household questionnaires were completed will be tabulated by study arm, both overall and separately for each time point.

12.2.3. Cross-sectional survey outcomes

The primary outcome for the cross-sectional surveys will be the prevalence of anemia (defined as hemoglobin < 11.0 g/dL). At each endpoint (one and two years after baseline), the crude prevalence of anemia will be tabulated for each cluster. Although there will be some variation in cluster sample sizes, this is not large and cluster size is unlikely to be associated with differences in prevalence of

anemia, therefore the simple mean prevalence across the 10 clusters per intervention arm will be used to calculate point estimates for the overall prevalence of anemia in each intervention arm. A weighted average of the cluster prevalences will also be calculated for comparison, with the weights provided by the sample size for each cluster. A risk ratio for the effect of the intervention will be calculated directly from the cluster-based point estimates. If necessary, a logarithmic transformation will be applied to normalize cluster-specific prevalences before analyzing the data. A stratified t-test will be used to compare the means of the cluster-specific proportions, where the within-stratum between-cluster variance will be estimated as the residual mean square from a two-way analysis of variance of the log-prevalences on stratum and treatment arm, including an interaction term. A 95% confidence interval (CI) for the risk ratio, adjusting for stratum, will be calculated from this variance using a t-statistic with 16 degrees of freedom.

Adjusted analysis for the effect of the intervention on prevalence of anemia will also be performed to account for any baseline imbalances between groups. We will adjust for the cluster-specific prevalence of anemia collected at the baseline cross sectional survey. Additional *a priori* individual-level factors to be adjusted for are age group, sex, use of insecticide treated nets, and distance to the health facility. Any other individual- or cluster-specific factors found to be unbalanced at baseline will also be adjusted for. Adjustment will be performed by fitting a logistic regression model, including terms for stratum and the covariates to be adjusted for, but no term for intervention arm, to data from all clusters. From this the predicted prevalence of anemia for each cluster will be available. The ratio between the observed and predicted prevalence will be calculated (risk ratio-residuals). A stratified t-test will then be conducted on the risk ratio-residuals and the covariate-adjusted risk ratio (and 95% CI) for the effect of the intervention will be calculated by applying the two-way analysis of variance method described above to the residuals.

A secondary analysis of individual-level data will be implemented by fitting generalized estimating equation (GEE) models with an exchangeable correlation structure and robust standard errors, and including a fixed effect for stratum. Wald tests will be used to calculate p-values and 95% confidence intervals for the odds ratio for the effect of the intervention. Approaches based on individual-level data have been found to be less robust than the cluster-level approach where the number of clusters is not large, therefore results will be treated with caution.

It is possible that community-based interventions (CBI) may be scaled up during the study period. If this scaling-up is implemented approximately at the one year cross-sectional survey it will be possible to examine its impact by comparing outcomes from one to two years in the individual-level regression analysis described above, although the study has not been powered for this comparison. Otherwise it will not be possible to quantify the impact of any scaling-up of these activities on cross-sectionally collected outcomes.

12.2.4. Cohort study outcomes

The primary outcome for the cohort study will be antimalarial treatment incidence density, defined as the rate of antimalarial treatments administered to children in the cohort over the period of follow up. The number of events, child-years of follow-up and corresponding incidence rate will be tabulated by cluster. For each intervention arm, the cluster-specific rates will then be averaged to give a point estimate of the rate for each intervention. Rate ratios for the effect of the intervention on each outcome will then be calculated from these point estimates. The distribution of cluster-specific rates is likely to be skewed, therefore a logarithmic transformation will be applied to normalize rates before analysis. A test of the null hypothesis that the rate ratio is equal to one will

be conducted using a stratified t-test, where the within-stratum between-cluster variance will be estimated as the residual mean square from a two-way analysis of variance of the log-rates on stratum and treatment arm, including an interaction term. A 95% confidence interval (CI) for the rate ratio will be calculated from this variance using a t-statistic with 16 degrees of freedom.

Adjusted analysis will be performed to account for any baseline differences between groups. *A priori* individual-level factors to be adjusted for are baseline anemia, age group, sex, use of insecticide treated nets, and distance to the health facility. Any other individual- or cluster-specific factors found to be unbalanced between the intervention arms at baseline will also be adjusted for. Adjusted rate ratios will be calculated using a similar two-stage process as that described for the cross-sectional outcomes above, except Poisson regression will be used to calculate predicted rates and hence rate ratio residuals.

For cohort study outcomes, it will be possible to allow for changes in underlying rates of these events due to scaling-up of CBIs in the study area. This will be done by splitting the follow-up time for each cluster into periods representing pre- and post-scale-up, and examining the effect of the intervention on event rates, stratified by time-period.

12.2.5. Outcomes measured at health facilities

The primary outcome collected at health facilities will be inappropriate treatment of malaria, as quantified by the proportion of children under five with suspected malaria and a negative RDT result that are inappropriately treated with an ACT plus the proportion of children under five with suspected malaria and a positive RDT result that are not treated with an ACT. These data will be collected every six months at exit interviews from health facilities.

For each time point, the proportion will be tabulated by cluster, and the cluster-specific mean proportions will be averaged to give a point estimate of proportion in appropriately treated with an ACT in each intervention arm. The risk ratio for the impact of the intervention will then be calculated. The two-way analysis of variance and stratified t-test approach described above for the cross-sectional survey prevalence outcomes will then be applied to test the null hypothesis that the risk ratio equals one and to derive a 95% CI for the risk ratio. Adjusted analyses will also be performed to account for any baseline differences between the study arms, using the same two-stage approach described above. *A priori* individual-level factors to be adjusted for are age group and sex.

Scaling-up of CBIs in the study area is unlikely to impact on outcomes measured at exit interviews from health facilities, therefore no further analysis is planned to account for this.

13 DATA & SAFETY MONITORING BOARD

13.1. OVERVIEW

A data and safety monitoring board (DSMB) will be assembled in conjunction with the LSHTM and the ACT Consortium, consisting of a minimum of three members who are independent of the project and who have made no significant input into the project's design. Members will include a chairman, a statistician, a clinical monitor, and a local safety monitor, if needed. The DSMB, investigators, and the sponsor will agree on the rules for reporting safety data during the course of the project, and the rules for recommending premature termination of the project on grounds of safety or efficacy. The DSMB will review the analytical plan and will agree to review the interim reported data at pre-specified intervals.

13.2. MONITORING PLAN

We plan to prepare an interim report for review by the DSMB after approximately one half of the total projected follow-up time has been observed; specifically, after the HFI has been fully active for approximately one year, the first annual cross-sectional survey has been completed, and over 75% of the cohort study participants have been followed for one year. The interim report will contain information on study progress and data quality (including recruitment, follow-up, and protocol adherence), safety data (serious adverse events), and primary outcome data (prevalence of anemia, antimalarial treatment incidence density, inappropriate use of ACTs). A shell interim report will be prepared by project members in conjunction with our statisticians, and will be submitted to the DSMB for review prior to the start of the study.

13.3. STOPPING GUIDELINES

The DSMB will have the authority to recommend cessation of the project for reasons related to safety of trial subjects, and to notify the sponsor accordingly. Interpretation of results and decisions about discontinuation of the study will be made by the members of the DSMB.

14 DATA MANAGEMENT

14.1. DATA MANAGEMENT

14.1.1. Cross-sectional surveys

Screening forms, consent forms, survey questionnaires, and clinical case record forms will be completed by the field teams. Microscopy results will be recorded in a laboratory record book by lab technicians. All record forms will be reviewed by project coordinators for completeness and accuracy. Data entered onto paper record forms will be entered into a computerized database (Microsoft Access) by a data entry clerk and will be double-entered to verify accuracy. Electronic versions of the baseline cross-sectional survey questionnaire will be created for personal digital assistants (PDAs) using appropriate software (Visual CE, Syware Inc). Survey teams will move in pairs; one team member will administer the survey questionnaire and record answers on a PDA, while another team member will record answers on a paper questionnaire. Data captured on PDAs will be downloaded daily to a Microsoft Access database. Data captured on paper record forms will be used as back-up if synchronization of the PDA to the computerized database fails. The repeat CSS will be completed by the field teams using hand-held computers (tablets). Prior to conducting the data collection, information from the questionnaires and fields for entering results of biomarker testing will be programmed into the hand-held computers. Programming will include range checks, structure checks and internal consistency checks. Data from these devices will be transferred at the end of every day to our core data facility and stored on a secure server.

14.1.2. Cohort study

Screening forms, consent forms, household survey forms, clinical case record forms, and monthly questionnaires will be completed by the field teams. Information recorded on the pictorial diaries by primary caregivers will be transferred onto standardized data extraction forms by study personnel. Microscopy results will be recorded in a laboratory record book by lab technicians. All record forms will be reviewed by project coordinators for completeness and accuracy. Data entered onto paper record forms and the data extraction forms will be entered into a computerized database (Microsoft Access) by a data entry clerk and will be double-entered to verify accuracy. Electronic versions of the initial household survey questionnaire and the monthly questionnaire will be created for the PDAs using appropriate software (Visual CE, Syware Inc). Field teams will move in pairs; one team member will administer the appropriate questionnaire and record answers on a PDA, while another team member will record answers on a paper version of the questionnaire. Data captured on PDAs will be downloaded daily to a Microsoft Access database. Data captured on paper record forms will be used as back-up if synchronization of the PDA to the computerized database fails. The repeat household survey will be completed by the field teams using hand-held computers (tablets). Prior to conducting the data collection, information from the questionnaires and fields for entering results of biomarker testing will be programmed into the hand-held computers. Programming will include range checks, structure checks and internal consistency checks. Data from these devices will be transferred at the end of every day to our core data facility and stored on a secure server.

14.1.3. M&E of health facilities

The patient exit interviews, health facility surveillance forms, and health worker knowledge questionnaires will be completed by field teams. All record forms will be reviewed by project coordinators for completeness and accuracy. Data from the M&E components will be collected using a combination of paper record forms, PDA questionnaires, and tablet questionnaires. Data entered onto paper record forms and the data extraction forms will be entered into a computerized database (Microsoft Access) by a data entry clerk and will be double-entered to verify accuracy. Electronic versions of the health facility surveillance forms will be created for the PDAs using appropriate software (Visual CE, Syware Inc). Data captured on PDAs will be downloaded daily to a Microsoft Access database. Data captured on paper record forms will be used as back-up if synchronization of the PDA to the computerized database fails. The final round of patient exit interviews will be completed by the field teams using hand-held computers (tablets). Some health facility surveillance data will also be collected using the tablets. Prior to conducting the data collection, information from the questionnaires and fields for entering results of biomarker testing will be programmed into the hand-held computers. Programming will include range checks, structure checks and internal consistency checks. Data from these devices will be transferred at the end of every day to our core data facility and stored on a secure server.

14.2. QUALITY ASSURANCE AND QUALITY CONTROL

All members of the study team will be trained in the project objectives, methods of effective communication with study participants, and collection of high quality data. Study team members will receive additional training specific to the tasks they will perform within the project including interviewing techniques, administration of surveys, completing questionnaires, and use of PDA and tablet devices. Standard Operating Procedures (SOPs) will be written for all project activities and booklets of all relevant documents will be provided to each member of the project team. Study group meetings will be conducted by the principal investigator to assess progress of the study, address any difficulties, and provide performance feedback to the members of the study group. Any corrections to data collection forms will be made by striking through the incorrect entry with a single line and entering the correct information adjacent to it, according to Good Clinical Practice guidelines.[96] The correction will be initialed and dated by the investigator. The investigators will allow all requested monitoring visits, audits or reviews.

14.3. RECORDS AND STORAGE

All study documents will be kept in secured filing cabinets in the Infectious Disease Research Collaboration offices. The principal investigator will be responsible for the security of all project documents. Back-up files of databases will be stored onto the main project server after each data entry session. Participants will be identified by their study ID number, and participant names will not be entered into the computerised database.

14.4. DATA SHARING

This project is one of 16 participating in the ACT Consortium (www.actconsortium.org/). As part of the ACT Consortium, a policy liaison network will be organized to help synthesize data from the multiple projects and communicate the results to policy makers. Consortium researchers will share data with the policy liaison network to facilitate analyses and ensure broad dissemination of the research findings.

15 PROTECTION OF HUMAN PARTICIPANTS

15.1. INSTITUTIONAL REVIEW BOARDS

This study will be reviewed by the following organizations:

1. London School of Hygiene and Tropical Medicine Ethics Committee
2. Makerere University Faculty Research and Ethical Committee
3. Uganda National Council of Science and Technology
4. University of California, San Francisco Committee for Human Research

15.2. INFORMED CONSENT PROCESS

Study personnel will conduct screening interviews and informed consent discussions with individual children and their parent/guardian during the screening process for the cross-sectional surveys and the cohort study. Informed consent will be conducted in the appropriate language (usually Luganda) and a translator will be used if necessary. Consent forms will be available in both English and Luganda. Following the informed consent discussion in the baseline CSS and cohort study, parents (or guardians) will be asked by the study personnel to provide their written consent on the approved informed consent document for their child to participate in a research study and a second approved consent form for the future use of biological specimens obtained during the course of the study. Following the informed consent discussion in the repeat CSS and the PEI, parents (or guardians) will be asked by the study personnel to provide their written consent on the approved informed consent document for their child to participate in a research study only. If the parent or guardian is unable to read or write, their fingerprint will substitute for a signature, and a signature from a witness to the informed consent procedures will be obtained. Written assent to participate in the cross-sectional surveys will also be obtained from children aged 8 years and older at the time of screening. An additional information sheet will be provided to the cohort study parents (or guardians) to update them on the plans to end the cohort study (Appendix HH), and written consent from the parents (or guardians) will be sought at the time of study termination.

15.3. CONFIDENTIALITY

Parents and guardians will be informed that participation in a research study may involve a loss of privacy. All records will be kept as confidential as possible. Participants will be identified primarily by their study number and patient names will not be entered into the computerized database. No individual identities will be used in any reports or publications resulting from the study.

15.4. RISKS AND DISCOMFORTS

15.4.1. Privacy

Care will be taken to protect the privacy of participants, as described in this protocol. However, there is a risk that others may inadvertently see participants' information, and thus their privacy compromised.

15.4.2. Risks of randomization

This will be a randomized trial in which we propose to enhance the care provided at government-run health facilities. There is a risk that residents living in villages within clusters randomized to the standard care arm may receive health care that is inappropriate, ineffective, or delayed, given that care delivered from those facilities will not be enhanced. However, it is not clear if care provided by HFI facilities will improve the health of children or quality of care above care standardly available; this study is designed to answer that question.

15.4.3. Fingerprick blood draws

Participants in the cross-sectional surveys and cohort study will have blood removed by fingerprick for laboratory evaluations, which has been associated with discomfort, bleeding, bruising, and rarely infection.

15.4.4. Risk of artemether-lumefantrine

AL appears to be safe and well-tolerated,[97,98] which is supported by the results of studies in Uganda.[35,36,99,100] In one study, participants treated with AL were more likely to have elevated temperature than those treated with AQ+SP or AQ+AS, which may have been due to the known anti-pyretic properties of amodiaquine, a component of the other two study regimens.[100] In addition, a higher risk of delayed diarrhea was observed in AL-treated patients, which has previously been reported.[101,102]

In a review of the safety and tolerability of AL in 1869 patients (including 368 children aged < 5 years), the most commonly reported adverse events that were possibly related to AL included gastrointestinal events (abdominal pain, anorexia, nausea, vomiting, and diarrhea), headache, and dizziness, while pruritis and rash occurred uncommonly.[102] Most events were of mild or moderate severity and were similar to symptoms associated with clinical malaria. The tolerability profile of AL compared favourably to other antimalarial drugs, including CQ, mefloquine, SP, and quinine.[102] In a systematic review including eight comparative clinical trials, AL was found to be associated with less vomiting (mild or moderate) than CQ, mefloquine, halofantrine, and AS + mefloquine.[103]

The chemical structure of lumefantrine is similar to halofantrine, raising concern for possible cardiac toxicity with AL. Treatment with halofantrine is associated with changes in cardiac conduction, particularly prolongation of the QT interval, which can produce arrhythmias.[104] However, no serious cardiac toxicity has been reported with AL.[103] A study of cardiac effects in healthy volunteers found a concentration-dependent prolongation of the QTc interval in all participants treated with halofantrine, but no change in the QTc interval after treatment with AL.[105]

Electrocardiographic data from clinical trials evaluating AL are encouraging, indicating that the frequency of QTc prolongation with AL is similar to that observed with CQ, mefloquine, and AQ + mefloquine.[102,106,107]

15.4.5. RDTs for malaria

The accuracy of RDTs for diagnosing malaria has been well-studied.[108-112] In Uganda, RDTs based on histidine-rich protein 2 (HRP2) and RDTs based on plasmodium lactate dehydrogenase (pLDH) were compared with expert microscopy and polymerase chain reaction (PCR)-corrected microscopy for 7000 patients at sites of varying malaria transmission intensity across Uganda.[109] When all sites were considered, the sensitivity of the HRP2-based test was 97% when compared with microscopy and 98% when corrected by PCR; the sensitivity of the pLDH-based test was 88% when compared with microscopy and 77% when corrected by PCR. The specificity of the HRP2-based test was 71% when compared with microscopy and 88% when corrected by PCR; the specificity of the pLDH-based test was 92% when compared with microscopy and >98% when corrected by PCR. Based on Plasmodium falciparum PCR-corrected microscopy, the positive predictive value (PPV) of the HRP2-based test was high (93%) at all but the site with the lowest transmission rate; the pLDH-based test and expert microscopy offered excellent PPVs (98%) for all sites. The negative predictive value (NPV) of the HRP2-based test was consistently high (>97%); in contrast, the NPV for the pLDH-based test dropped significantly (from 98% to 66%) as transmission intensity increased, and the NPV for expert microscopy decreased significantly (99% to 54%) because of increasing failure to detect subpatent parasitemia.

15.4.6. Compensation

Participants will not be paid for participating in this study. Most assessments will be conducted at households or health facilities, which will eliminate the need for travel and minimize opportunity costs for the participants. If cross-sectional survey or cohort study participants are referred by study personnel to a health facility for further assessment, transportation will either be provided by the study team, or the costs of transportation will be borne by the project. Any other costs of transportation related to project activities will be reimbursed by the project. Households participating in the cohort study will receive small incentives (soap, washing powder, or sugar) at monthly visits. All children participating in the cohort study will be given an insecticide-treated bednet at the end of study follow-up. Health facilities will also receive periodic recognition for their participation in the surveillance activities, including sugar, tea, coffee, or soap.

15.4.7. Alternatives

Children whose parents or guardians choose not to participate in this study will not be enrolled in the cross-sectional survey or cohort study. Children excluded from the study will still be eligible for standard care of medical problems as they arise at the government-run health facilities or other available health centers.

16 TIMELINE

Figure 5. ACT PRIME Study implementation

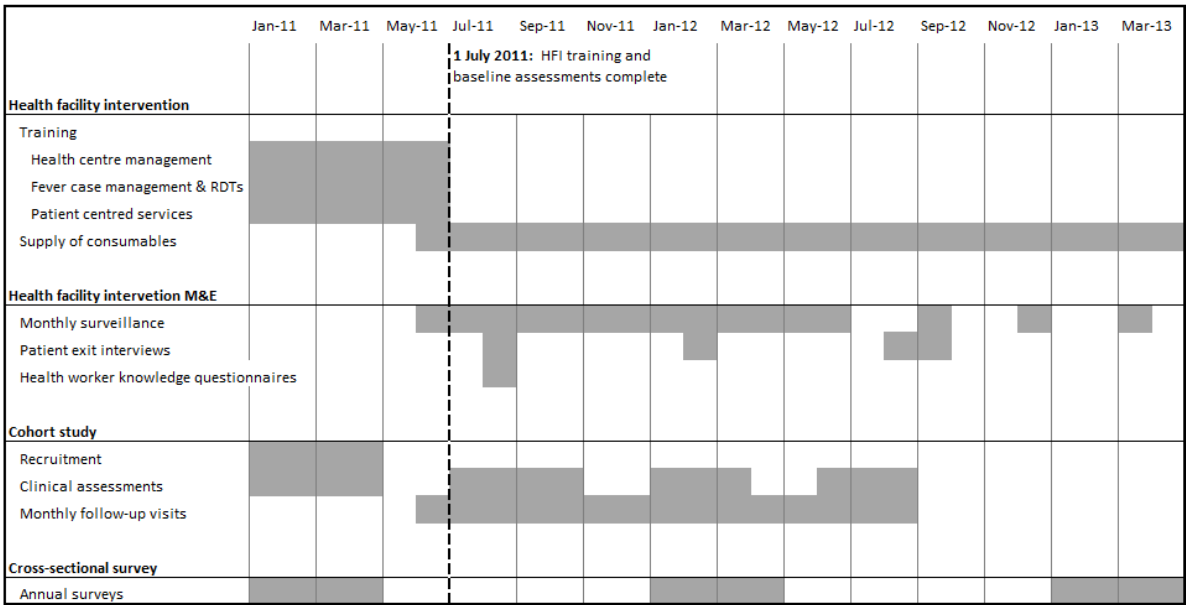

17 REFERENCES

1. Greenwood BM, Bojang K, Whitty CJ, Targett GA (2005) Malaria. Lancet 365: 1487-1498.

2. Breman J (2001) The ears of the hippopotamus: manifestations, determinants, and estimates of the malaria burden. Am J Trop Med Hyg 64: 1-11.

3. Breman JG, Alilio MS, Mills A (2004) Conquering the intolerable burden of malaria: what's new, what's needed: a summary. Am J Trop Med Hyg 71: 1-15.

4. Snow RW, Craig M, Deichmann U, Marsh K (1999) Estimating mortality, morbidity and disability due to malaria among Africa's non-pregnant population [see comments]. Bull World Health Organ 77: 624-640.

5. Greenwood BM, Bradley AK, Greenwood AM, Byass P, Jammeh K, et al. (1987) Mortality and morbidity from malaria among children in a rural area of The Gambia, West Africa. Trans R Soc Trop Med Hyg 81: 478-486.

6. Roll Back Malaria Partnership (2005) Global strategic plan 2005-2015. Geneva: Roll Back Malaria Partnership.

7. UNICEF (2007) Malaria & children: Progress in intervention coverage. New York, NY USA: UNICEF / Roll Back Malaria Partnership.

8. Kager PA (2002) Malaria control: constraints and opportunities. Trop Med Int Health 7: 1042-1046.

9. Moerman F, Lengeler C, Chimumbwa J, Talisuna A, Erhart A, et al. (2003) The contribution of health-care services to a sound and sustainable malaria-control policy. Lancet Infect Dis 3: 99-102.

10. McCombie SC (1996) Treatment seeking for malaria: a review of recent research. Soc Sci Med 43: 933-945.

11. Marsh VM, Mutemi WM, Muturi J, Haaland A, Watkins WM, et al. (1999) Changing home treatment of childhood fevers by training shop keepers in rural Kenya. Trop Med Int Health 4: 383-389.

12. Goodman C, Kachur SP, Abdulla S, Mwageni E, Nyoni J, et al. (2004) Retail supply of malaria-related drugs in rural Tanzania: risks and opportunities. Trop Med Int Health 9: 655-663.

13. World Health Organization (2004) Scaling up home-based management of malaria: from research to implementation. Geneva.

14. World Health Organization (2007) Lessons learned in home management of malaria: Implementation research in four African countries.

15. Ajayi IO, Browne EN, Garshong B, Bateganya F, Yusuf B, et al. (2008) Feasibility and acceptability of artemisinin-based combination therapy for the home management of malaria in four African sites. Malar J 7: 6.

16. World Health Organization (2010) Guidelines for the treatment of malaria. Geneva: World Health Organization.

17. Reyburn H, Ruanda J, Mwerinde O, Drakeley C (2006) The contribution of microscopy to targeting antimalarial treatment in a low transmission area of Tanzania. Malar J 5: 4.

18. Zurovac D, Midia B, Ochola SA, English M, Snow RW (2006) Microscopy and outpatient malaria case management among older children and adults in Kenya. Trop Med Int Health 11: 432-440.

19. Hamer DH, Ndhlovu M, Zurovac D, Fox M, Yeboah-Antwi K, et al. (2007) Improved diagnostic testing and malaria treatment practices in Zambia. Jama 297: 2227-2231.

20. Reyburn H, Mbakilwa H, Mwangi R, Mwerinde O, Olomi R, et al. (2007) Rapid diagnostic tests compared with malaria microscopy for guiding outpatient treatment of febrile illness in Tanzania: randomised trial. Bmj 334: 403.

21. Ssekabira U, Bukirwa H, Hopkins H, Namagembe A, Weaver MR, et al. (2008) Improved malaria case management after integrated team-based training of health care workers in Uganda. Am J Trop Med Hyg 79: 826-833.

22. Hopkins H, Asiimwe C, Bell D (2009) Access to antimalarial therapy: accurate diagnosis is essential to achieving long term goals. BMJ 339: b2606.

23. Msellem MI, Martensson A, Rotllant G, Bhattarai A, Stromberg J, et al. (2009) Influence of rapid malaria diagnostic tests on treatment and health outcome in fever patients, Zanzibar: a crossover validation study. PLoS Med 6: e1000070.

24. Williams HA, Causer L, Metta E, Malila A, O'Reilly T, et al. (2008) Dispensary level pilot implementation of rapid diagnostic tests: an evaluation of RDT acceptance and usage by providers and patients--Tanzania, 2005. Malar J 7: 239.

25. D'Acremont V (2009) Massive reduction of antimalarial prescription after rapid diagnostic test implementation in Dar es Salaam, Tanzania MIM Pan-African Malaria Conference. Nairobi, Kenya.

26. Uganda Ministry of Health (2008) The Burden of Malaria in Uganda.

27. Uganda Ministry of Health (2008) Malaria control and prevention.

28. Fapohunda BM, Plowman BA, Azairwe R, Bisorbowa G, Langi P, et al. (2004) Home-based management of fever strategy in Uganda: A report of the 2003 survey. Arlington, Virginia, USA: MOH, WHO, BASICS II.

29. Mbonye AK (2003) Prevalence of childhood illnesses and care-seeking practices in rural Uganda. ScientificWorldJournal 3: 721-730.

30. Kengeya-Kayondo JF, Seeley JA, Kajura-Bajenja E, Kabunga E, Mubiru E, et al. (1994) Recognition, treatment seeking behaviour and perception of cause of malaria among rural women in Uganda. Acta Trop 58: 267-273.

31. Tumwesigire S, Watson S (2002) Health seeking behavior by families of children suspected to have malaria in Kabale: Uganda. Afr Health Sci 2: 94-98.

32. Ndyomugyenyi R, Magnussen P, Clarke S (2007) Malaria treatment-seeking behaviour and drug prescription practices in an area of low transmission in Uganda: implications for prevention and control. Trans R Soc Trop Med Hyg 101: 209-215.

33. Tren R, Hess K, Bate R (2009) Drug procurement, the Global Fund and misguided competition policies. Malar J 8: 305.

34. Dorsey G, Staedke S, Clark TD, Njama-Meya D, Nzarubara B, et al. (2007) Combination therapy for uncomplicated falciparum malaria in Ugandan children: a randomized trial. Jama 297: 2210-2219.

35. Kamya MR, Yeka A, Bukirwa H, Lugemwa M, Rwakimari JB, et al. (2007) Artemether-Lumefantrine versus Dihydroartemisinin-Piperaquine for Treatment of Malaria: A Randomized Trial. PLoS Clin Trials 2: e20.

36. Yeka A, Dorsey G, Kamya MR, Talisuna A, Lugemwa M, et al. (2008) Artemether-lumefantrine versus dihydroartemisinin-piperaquine for treating uncomplicated malaria: a randomized trial to guide policy in Uganda. PLoS ONE 3: e2390.

37. Zurovac D, Tibenderana J, Nankabirwa J, Ssekitooleko J, Talisuna A, et al. (2008) Evaluation of outpatient malaria case-management under artemether-lumefantrine treatment policy in Uganda. Malaria Consortium, Uganda Ministry of Health, KEMRI/Wellcome Trust Collaborative Programme, Kenya.

38. Kindermans JM, Pilloy J, Olliaro P, Gomes M (2007) Ensuring sustained ACT production and reliable artemisinin supply. Malar J 6: 125.

39. Moszynski P Disappearance of drugs undermines Uganda's fight against malaria. BMJ 340: c2611.

40. Uganda Ministry of Health (2004) Implementation guidelines for the home based management of fever strategy. 2nd edition.

41. Kolaczinski JH, Ojok N, Opwonya J, Meek S, Collins A (2006) Adherence of community caretakers of children to pre-packaged antimalarial medicines (HOMAPAK) among internally displaced people in Gulu district, Uganda. Malar J 5: 40.

42. Institute of Medicine (2001) Crossing the quality chasm: a new health system for the 21st Century Washington, DC.

43. Kelly JM, Osamba B, Garg RM, Hamel MJ, Lewis JJ, et al. (2001) Community health worker performance in the management of multiple childhood illnesses: Siaya District, Kenya, 1997-2001. Am J Public Health 91: 1617-1624.

44. Zurovac D, Rowe AK (2006) Quality of treatment for febrile illness among children at outpatient facilities in sub-Saharan Africa. Ann Trop Med Parasitol 100: 283-296.

45. Leonard K, Masatu M, Vialou A (2005) Getting clinicians to do their best: Ability, Altruism and Incentives.: Working paper, University of Maryland.

46. Chakraborty S, Frick K (2002) Factors influencing private health providers' technical quality of care for acute respiratory infections among under-five children in rural West Bengal, India. Soc Sci Med 55: 1579-1587.

47. Arifeen SE, Bryce J, Gouws E, Baqui AH, Black RE, et al. (2005) Quality of care for under-fives in first-level health facilities in one district of Bangladesh. Bull World Health Organ 83: 260-267.

48. Krause G, Schleiermacher D, Borchert M, Benzler J, Heinmuller R, et al. (1998) Diagnostic quality in rural health centres in Burkina Faso. Trop Med Int Health 3: 100-107.

49. World Health Organisation (2000) World Health Report 2000. Health Systems: Improving Performance. Geneva.

50. Brown LD, Franco LM, Rafeh N, Hatzell T (1993) Quality Assurance of Health Care in Developing Countries. Bethesda, MD: Quality Assurance Project. Online at http://www.qaproject.org/pubs/PDFs/DEVCONT.pdf. Accessed 24 July 2008.

51. Hall JA, Epstein AM, DeCiantis ML, McNeil BJ (1993) Physicians' liking for their patients: more evidence for the role of affect in medical care. Health Psychol 12: 140-146.

52. Mbaruku G, Bergstrom S (1995) Reducing maternal mortality in Kigoma, Tanzania. Health Policy Plan 10: 71-78.

53. McPake B (1993) User charges for health services in developing countries: a review of the economic literature. Soc Sci Med 36: 1397-1405.

54. Wouters AV (1991) Essential national health research in developing countries: health care financing and the quality of care. Int J Health Planning and Management 6: 253-271.

55. El Arifeen S, Blum LS, Hoque DM, Chowdhury EK, Khan R, et al. (2004) Integrated Management of Childhood Illness (IMCI) in Bangladesh: early findings from a cluster-randomised study. Lancet 364: 1595-1602.

56. Deyo RA, Inui TS (1980) Dropouts and broken appointments. A literature review and agenda for future research. Med Care 18: 1146-1157.

57. Vera H (1993) The client's view of high-quality care in Santiago, Chile. Stud Fam Plann 24: 40-49.

58. Williams B (1994) Patient satisfaction: a valid concept? Soc Sci Med 38: 509-516.

59. Ashworth A, Chopra M, McCoy D, Sanders D, Jackson D, et al. (2004) WHO guidelines for management of severe malnutrition in rural South African hospitals: effect on case fatality and the influence of operational factors. Lancet 363: 1110-1115.

60. Puoane T, Sanders D, Ashworth A, Chopra M, Strasser S, et al. (2004) Improving the hospital management of malnourished children by participatory research. Int J Qual Health Care 16: 31-40.

61. Howie SR, Hill SE, Peel D, Sanneh M, Njie M, et al. (2008) Beyond good intentions: lessons on equipment donation from an African hospital. Bull World Health Organ 86: 52-56.

62. Chandler CI, Whitty CJ, Ansah EK (2010) How can malaria rapid diagnostic tests achieve their potential? A qualitative study of a trial at health facilities in Ghana. Malar J 9: 95.

63. Soumerai SB, McLaughlin TJ, Avorn J (1989) Improving drug prescribing in primary care: a critical analysis of the experimental literature. Milbank Q 67: 268-317.

64. Oxman AD, Thomson MA, Davis DA, Haynes RB (1995) No magic bullets: a systematic review of 102 trials of interventions to improve professional practice. Cmaj 153: 1423-1431.

65. Davis DA, Thomson MA, Oxman AD, Haynes RB (1995) Changing physician performance. A systematic review of the effect of continuing medical education strategies. JAMA 274: 700-705.

66. Grimshaw J, Shirran L, Thomas R, Mowatt G, Fraser C, et al. (2002) Changing provider behaviour: an overview of systematic reviews of interventions to promote implementation of research findings by healthcare professionals. In: Haines A, Donald A, editors. Getting Research Findings into Practice. 2nd ed. London: BMJ Books. pp. 29-68.

67. Smith LA, Jones C, Meek S, Webster J (2009) Review: Provider practice and user behavior interventions to improve prompt and effective treatment of malaria: do we know what works? Am J Trop Med Hyg 80: 326-335.

68. Ross-Degnan D, Laing R, Santoso B, Ofori-Adjei D, Lamoureux C, et al. (1997) Improving pharmaceutical use in primary care in developing counties: a critical review of experience and lack of experience. Chiang Mai, Thailand, April

69. Ofori-Adjei D, Arhinful DK (1996) Effect of training on the clinical management of malaria by medical assistants in Ghana. Soc Sci Med 42: 1169-1176.

70. Rowe SY, Kelly JM, Olewe MA, Kleinbaum DG, McGowan JE, Jr., et al. (2007) Effect of multiple interventions on community health workers' adherence to clinical guidelines in Siaya district, Kenya. Trans R Soc Trop Med Hyg 101: 188-202.

71. Rowe SY, Olewe MA, Kleinbaum DG, McGowan JE, Jr., McFarland DA, et al. (2007) Longitudinal analysis of community health workers' adherence to treatment guidelines, Siaya, Kenya, 1997-2002. Trop Med Int Health 12: 651-663.

72. Armstrong Schellenberg J, Bryce J, de Savigny D, Lambrechts T, Mbuya C, et al. (2004) The effect of Integrated Management of Childhood Illness on observed quality of care of under-fives in rural Tanzania. Health Policy Plan 19: 1-10.

73. Amaral J, Gouws E, Bryce J, Leite AJ, Cunha AL, et al. (2004) Effect of Integrated Management of Childhood Illness (IMCI) on health worker performance in Northeast-Brazil. Cad Saude Publica 20 Suppl 2: S209-219.

74. Gilroy K, Winch PJ, Diawara A, Swedberg E, Thiero F, et al. (2004) Impact of IMCI training and language used by provider on quality of counseling provided to parents of sick children in Bougouni District, Mali. Patient Educ Couns 54: 35-44.

75. Gouws E, Bryce J, Habicht JP, Amaral J, Pariyo G, et al. (2004) Improving antimicrobial use among health workers in first-level facilities: results from the multi-country evaluation of the Integrated Management of Childhood Illness strategy. Bull World Health Organ 82: 509-515.

76. Naimoli JF, Rowe AK, Lyaghfouri A, Larbi R, Lamrani LA (2006) Effect of the Integrated Management of Childhood Illness strategy on health care quality in Morocco. Int J Qual Health Care 18: 134-144.

77. Pariyo GW, Gouws E, Bryce J, Burnham G (2005) Improving facility-based care for sick children in Uganda: training is not enough. Health Policy Plan 20 Suppl 1: i58-i68.

78. Perez-Cuevas R, Guiscafre H, Munoz O, Reyes H, Tome P, et al. (1996) Improving physician prescribing patterns to treat rhinopharyngitis. Intervention strategies in two health systems of Mexico. Soc Sci Med 42: 1185-1194.

79. Zurovac D, Rowe AK, Ochola SA, Noor AM, Midia B, et al. (2004) Predictors of the quality of health worker treatment practices for uncomplicated malaria at government health facilities in Kenya. Int J Epidemiol 33: 1080-1091.

80. Fonn S, Mtonga AS, Nkoloma HC, Bantebya Kyomuhendo G, daSilva L, et al. (2001) Health providers' opinions on provider-client relations: results of a multi-country study to test Health Workers for Change. Health Policy Plan 16 Suppl 1: 19-23.

81. Manongi RN, Nasuwa FR, Mwangi R, Reyburn H, Poulsen A, et al. (2009) Conflicting priorities: evaluation of an intervention to improve nurse-parent relationships on a Tanzanian paediatric ward. Hum Resour Health 7: 50.

82. Labhardt ND, Manga E, Ndam M, Balo JR, Bischoff A, et al. (2009) Early assessment of the implementation of a national programme for the prevention of mother-to-child transmission of HIV in Cameroon and the effects of staff training: a survey in 70 rural health care facilities. Trop Med Int Health 14: 288-293.

83. Marvel MK, Epstein RM, Flowers K, Beckman HB (1999) Soliciting the patient's agenda - Have we improved? Jama-Journal of the American Medical Association 281: 283-287.

84. Fogarty LA, Curbow BA, Wingard JR, McDonnell K, Somerfield MR (1999) Can 40 seconds of compassion reduce patient anxiety? J Clin Oncol 17: 371-379.

85. Di Blasi Z, Harkness E, Ernst E, Georgiou A, Kleijnen J (2001) Influence of context effects on health outcomes: a systematic review. Lancet 357: 757-762.

86. Thomas KB (1987) General practice consultations: is there any point in being positive? Br Med J (Clin Res Ed) 294: 1200-1202.

87. Fassaert T, van Dulmen S, Schellevis F, van der Jagt L, Bensing J (2008) Raising positive expectations helps patients with minor ailments: a cross-sectional study. BMC Fam Pract 9: 38.

88. Krasner MS, Epstein RM, Beckman H, Suchman AL, Chapman B, et al. (2009) Association of an educational program in mindful communication with burnout, empathy, and attitudes among primary care physicians. Jama 302: 1284-1293.

89. World Health Organization (2002) Community involvement in rolling back malaria. Roll Back Malaria/World Health Organization. WHO/CDS/RBM/2002.2042 p.

90. Davis JC, Clark TD, Kemble SK, Talemwa N, Njama-Meya D, et al. (2006) Longitudinal study of urban malaria in a cohort of Ugandan children: description of study site, census and recruitment. Malar J 5: 18.

91. Dorsey G, Njama D, Kamya MR, Rosenthal P. A longitudinal assessment of different first-line therapies for uncomplicated falciparum malaria in Kampala, Uganda; 2001; Atlanta, Georgia.

92. Staedke SG, Mwebaza N, Kamya MR, Clark TD, Dorsey G, et al. (2009) Home management of malaria with artemether-lumefantrine compared with standard care in urban Ugandan children: a randomised controlled trial. Lancet 373: 1623-1631.

93. Okello PE, W VANB, Byaruhanga AM, Correwyn A, Roelants P, et al. (2006) Variation in malaria transmission intensity in seven sites throughout Uganda. Am J Trop Med Hyg 75: 219-225.

94. Pullan RL, Bukirwa H, Staedke SG, Snow RW, Brooker S (2010) Plasmodium infection and its risk factors in eastern Uganda. Malar J 9: 2.

95. Wiseman V, Conteh L, Matovu F (2005) Using diaries to collect data in resource-poor settings: questions on design and implementation. Health Policy Plan 20: 394-404.

96. International Conference on Harmonisation of Technical Requirements for Registration of Pharmaceuticals for Human Use (ICH) (1996) Guidance for Industry: Good Clinical Practice: Consolidated Guidance (ICH-E6). Rockville, MD: U.S. Department of Health and Human Services, Food and Drug Administration, Center for Drug Evaluation and Research (CDER), Center for Biologics Evaluation and Research (CBER).

97. Makanga M, Premji Z, Falade C, Karbwang J, Mueller EA, et al. (2006) Efficacy and safety of the six-dose regimen of artemether-lumefantrine in pediatrics with uncomplicated Plasmodium falciparum malaria: a pooled analysis of individual patient data. Am J Trop Med Hyg 74: 991-998.

98. Omari AA, Gamble C, Garner P (2005) Artemether-lumefantrine (six-dose regimen) for treating uncomplicated falciparum malaria. Cochrane Database Syst Rev: CD005564.

99. Piola P, Fogg C, Bajunirwe F, Biraro S, Grandesso F, et al. (2005) Supervised versus unsupervised intake of six-dose artemether-lumefantrine for treatment of acute, uncomplicated Plasmodium falciparum malaria in Mbarara, Uganda: a randomised trial. Lancet 365: 1467-1473.

100. Maiteki-Sebuguzi C, Jagannathan P, Yau VM, Clark TD, Njama-Meya D, et al. (2008) Safety and tolerability of combination antimalarial therapies for uncomplicated falciparum malaria in Ugandan children. Malar J 7: 106.

101. Novartis (2003) Coartem monograph. Basel, Switzerland.

102. Bakshi R, Hermeling-Fritz I, Gathmann I, Alteri E (2000) An integrated assessment of the clinical safety of artemether-lumefantrine: a new oral fixed-dose combination antimalarial drug. Trans R Soc Trop Med Hyg 94: 419-424.

103. Omari AA, Gamble C, Garner P (2004) Artemether-lumefantrine for uncomplicated malaria: a systematic review. Trop Med Int Health 9: 192-199.

104. Nosten F, Price RN (1995) New antimalarials. A risk-benefit analysis. Drug Saf 12: 264-273.

105. Bindschedler M, Lefevre G, Degen P, Sioufi A (2002) Comparison of the cardiac effects of the antimalarials co-artemether and halofantrine in healthy participants. Am J Trop Med Hyg 66: 293-298.

106. van Vugt M, Ezzet F, Nosten F, Gathmann I, Wilairatana P, et al. (1999) No evidence of cardiotoxicity during antimalarial treatment with artemether-lumefantrine. Am J Trop Med Hyg 61: 964-967.

107. Lefevre G, Looareesuwan S, Treeprasertsuk S, Krudsood S, Silachamroon U, et al. (2001) A clinical and pharmacokinetic trial of six doses of artemether-lumefantrine for multidrug-resistant Plasmodium falciparum malaria in Thailand. Am J Trop Med Hyg 64: 247-256.

108. Drakeley C, Reyburn H (2009) Out with the old, in with the new: the utility of rapid diagnostic tests for malaria diagnosis in Africa. Trans R Soc Trop Med Hyg 103: 333-337.

109. Hopkins H, Bebell L, Kambale W, Dokomajilar C, Rosenthal PJ, et al. (2008) Rapid diagnostic tests for malaria at sites of varying transmission intensity in Uganda. J Infect Dis 197: 510-518.

110. Hopkins H, Kambale W, Kamya MR, Staedke SG, Dorsey G, et al. (2007) Comparison of HRP2- and pLDH-based rapid diagnostic tests for malaria with longitudinal follow-up in Kampala, Uganda Am J Trop Med Hyg 76: 1092-1097.

111. Murray CK, Gasser RA, Jr., Magill AJ, Miller RS (2008) Update on rapid diagnostic testing for malaria. Clin Microbiol Rev 21: 97-110.

112. Wongsrichanalai C, Barcus MJ, Muth S, Sutamihardja A, Wernsdorfer WH (2007) A review of malaria diagnostic tools: microscopy and rapid diagnostic test (RDT). Am J Trop Med Hyg 77: 119-127.

18 APPENDICES

Appendix A:	Intervention components, barriers addressed, and intended outcomes
Appendix B:	Health facility intervention information sheet
Appendix C:	Primary health care funds accounting tool
Appendix D:	ACT Drug Distribution Assessment Tool (ADDAT)
Appendix E:	User's manual: Use of Rapid Diagnostic Tests (RDTs) for malaria in fever case management in Uganda
Appendix F:	Health worker self-observational information sheet
Appendix G:	Health worker self-observational task sheets (1)
Appendix H:	Health worker self-observational task sheets (2)
Appendix I:	Health worker self-observational task sheets (3)
Appendix J:	Health worker self-observational task sheets (4)
Appendix K:	Cross-sectional survey screening form
Appendix L:	Cross-sectional survey informed consent to participate in a research study
Appendix M:	Cross-sectional survey informed consent for future use of biological specimens
Appendix N:	Cross-sectional survey assent form
Appendix O:	Cross-sectional survey questionnaire form
Appendix P:	Cross-sectional survey case record form
Appendix Q:	Cohort study screening form
Appendix R:	Cohort study informed consent to participate in a research study
Appendix S:	Cohort study informed consent for future use of biological specimens
Appendix T:	Cohort study case record form
Appendix U:	Cohort study household survey
Appendix UII:	Revised Cohort study household survey
Appendix V:	Cohort study pictorial diaries
Appendix W:	Cohort study monthly questionnaires
Appendix X:	Exit interview screening form
Appendix Y:	Exit interview informed consent to participate in a research study
Appendix Z:	Patient exit interview questionnaire form
Appendix AA:	Health facility surveillance information sheet
Appendix BB:	Health facility surveillance questionnaire form (Parts 1 & 2)
Appendix CC:	Health care worker knowledge questionnaire information sheet
Appendix DD:	Health care worker knowledge questionnaire form
Appendix EE:	WHO toxicity grading scale
Appendix FF:	UMSP toxicity grading scale
Appendix GG:	Serious adverse event report form
Appendix HH:	Cohort study information sheet for termination of study activities
Appendix II:	Caregivers participating in health worker knowledge assessment information sheet

APPENDIX A: Intervention components, barriers, and outcomes

Intervention Component	Barriers intended to address	Intended outcome
**1. Health Centre Management**		
Training in health centre management:<br>• Financial management (budgeting and accounting)<br>• Supply management (drug stocking)<br>• Information management	• Lack of in-charge skills to manage the health centre<br>• Low motivation of staff due to poor health centre administration<br>• Under-utilization or lack of appropriate tools to appropriately mange health centres<br>• Lack of information to adequately manage the health facility and report to local and district stakeholders	Improved accounting of PHC funds<br>Improved forecasting and stocking of needed supplies<br>Improved utilization of health information
**2. Health Worker Training**		
Training in fever case management and use of RDTs	• Poor knowledge of malaria case management<br>• Inadequate/unavailable infrastructure or diagnostic laboratory facilities	Good clinical care<br>Improved management of malaria and non-malaria illnesses<br>Improved HW-patient interactions<br>Professional conduct<br>Prompt and fair treatment<br>Appropriate use of volunteers<br>Improved welcoming & guiding
Training in Patient-Centered Services	• Lack of patient-centred thinking<br>• Communication problems including language barrier<br>• Discrimination/preferential treatment of patients<br>• Poor relationships between staff and communities<br>• Inappropriate use of volunteers<br>• Poor patient flow and management	
**3. Supply of Consumables**		
Drug delivery from NMS	• Frequent stock-outs of drugs<br>• Poor procurement system<br>• Patients have to pay for drugs or services	Good clinical care<br>Free services<br>Improved availability of drugs<br>Appropriate treatment of malaria and non-malarial illnesses
Support supply of artemether-lumefantrine	• Frequent stock-outs of first line antimalarial treatment	
RDTs for malaria	• Inadequate/unavailable infrastructure or diagnostic laboratory facilities<br>• RDTs not available nationally	

THE REPUBLIC OF UGANDA

APPENDIX B. INFORMATION SHEET – Health facility intervention
ACT PRIME Study

Introduction

Dr. Sarah Staedke and colleagues from the Uganda Malaria Surveillance Project / Infectious Diseases Research Collaboration are investigating delivery of health care services in Tororo District. We are doing a research study to see if we can improve the health of children in this area by improving services at government-run health facilities.

What will be done in this study?

Certain health centers in Tororo district will be selected to take part in the intervention to improve services, or to continue with their current services. Assignment to the two groups has been determined by a lottery. The chance of being placed into either of the groups is the same. Your health center has been selected to take part in the intervention.

The health facility intervention will focus on 1) health center management training, 2) health worker training, and 3) supply of malaria diagnostics and antimalarial drugs. The intervention package will be introduced to all health centers selected to take part over approximately 8-10 weeks. After this time, all health centers in the area will be assessed every month to determine how well the intervention is working. Our study personnel will continue to support the intervention at the selected health centers for the full duration of the study.

What will happen if my health center takes part in this study?

The intervention will include three different training packages, which you and other members of staff at your facility will be asked to take part in:

1) Health center management training. All in-charges of health centers assigned to the intervention will be trained in health center management. The purpose of this training is to equip in-charges with the skills and tools required to effectively and efficiently manage their health center. The training will include three components: financial management, supply management, and information management. Our study staff will carry out a series of half-day training workshops with the in-charges every 1-2 weeks, and the training module will last approximately 4-5 weeks. The in-charges will be taught how to collect data about primary health care (PHC) funds and drug supplies during the training. All health workers will also be trained on the importance of information management and how to utilize data on patient attendance, diagnoses made, and treatment provided.

2) Fever case management training. All clinical staff, including in-charges and other health workers, will receive training in fever case management. The training program will last two weeks and will be followed by support supervision over the next six months and refresher training as required.

3) Patient-centered services (PCS) training. The purpose of this training is to identify and improve interpersonal interactions between health workers and patients. All clinical staff, including in-charges, will receive the full PCS training package which will start with a series of 4 self-observation activities completed over a period of 4-5 weeks. These activities will guide the health workers through a PCS workshop which will consist of 5 half-day modules, lasting about 3 hours, carried out over 4-5 weeks. The modules will focus on introduction to PCS, improving interactions with patients and colleagues, and improving the patient visit Support staff, including volunteers will receive a scaled-down PCS training package with specific emphasis on improving the patient visit. The PCS workshop for support staff will consist of 1 half-day themed module carried out over 1 week. Health workers and support staff will be trained in two groups to ensure that work at the health centers continues alongside the training.

After beginning the intervention, we will collect information to see how well it is working. Every month, we will visit the health centers to gather information on patient attendance, drug stock-outs, staffing, and diagnosis and treatment of malaria. Every six months, we will interview patients as they are leaving the health center to learn more about their experience with the health center visit. Once a year, we will ask all health workers to take part in a survey about management of malaria and other fever illnesses to learn more about their knowledge and practices. The data we collect will be used by project investigators and may be shared with other researchers and policy-makers to answer questions about how best to deliver health services.

How long will this study last?

The intervention will be introduced over 2-3 months. The total duration of the study will be about 2 ½ years.

Can I stop being in the study?

You can decide to stop participating at any time. Just tell our study personnel right away if you wish to stop the activities.

What risks can I expect from participating in the study?

Participation in any research study may involve a loss of privacy. Information you provide about your health center will be recorded, but your name will not be used in any reports of the information provided. The information obtained from these study activities will be locked up at our project offices. We will do our best to make sure that any personal information is kept private.

Are there benefits to taking part in the study?

Through the intervention, we aim to improve the health of children in this area by improving services at the health centers. As part of the intervention, our project will provide training to in-charges and other health workers, which will benefit you directly, and help provide malaria drugs and tests for diagnosing malaria, which will benefit the health facilities and patients. The information that we gather in this study will help researchers and policy-makers understand how best to improve health services in this area.

What other choices do I have if I do not take part in the study?

You are free to choose not to take part in the study. If you decide not to take part, there will be no penalty to you.

What are the costs of taking part in the study? Will I be paid for taking part in the study?

There are no costs to you for taking part in this study. You will not be paid for taking part in this study. The training activities will take place in convenient locations within Tororo district. Any transport costs incurred by trainees will be reimbursed by the project.

What are my rights if I take part in the study?

Taking part in this study is your choice. You may choose either to take part or not to take part. If you decide to take part in this study, you may change your mind at any time. No matter what decision you take, there will be no penalty to you in any way.

Who can answer my questions about the study?

You can talk to the researchers about any questions or concerns you have about these study activities. Contact Dr. Sarah Staedke or other members of the Uganda Malaria Surveillance Project / Infectious Disease Research Collaboration on telephone number 0414-530692. If you have any questions, comments or concerns about taking part in these self-observation activities, first talk to the researchers. If for any reason you do not wish to do this, or you still have concerns about doing so, you may contact Dr Charles Ibingira, Makerere University Faculty of Medicine Research and Ethical Committee at telephone number 0414-530020.

Giving verbal consent to take part in the study:

You may keep this information sheet if you wish. Participation in these activities is voluntary. You have the right to decline to take part in the activities, or to withdraw from them at any point without penalty. If you do not wish to take part in the activities, you should inform the researcher now. If you do wish to take part in these activities, you should tell the researcher now.

APPENDIX C: PRIMARY HEALTH CARE (PHC) FUND ACCOUNTING TOOL

Health centre code	Health worker ID	Reporting Month / Year	*Starting balance* (amount remaining from last quarter + PHC fund this quarter)	
[_____|_____]	[_____|_____]	[_____|_____]/[_____|_____] month year		

BUDGETING			
**Item**	**Cost for item**	**Number of items needed**	**Total cost for items**
**TOTAL BUDGET**			

ACCOUNTING			
**Item**	**Actual cost for item**	**Number of items purchased**	**Total amount spent**
**TOTAL AMOUNT SPENT**			
Remaining Balance *(Starting balance – Total Amount Spent)*			

**Did you have enough money to pay for all items and services budgeted for this quarter?**	☐ Yes ☐ No ☐ I don't know ☐ Refuse to answer
**If 'no', what items or services were you not able to pay for? List all that apply.**	

APPENDIX D: ACT DRUG DISTRIBUTION ASSESSMENT TOOL (ADDAT)

Health centre code	Health worker ID	ADDAT reporting month	GREEN: The activity is on track.	YELLOW: The activity is only partly on track.	RED: The activity not is not on track.
[_____|_____]	[_____|_____]	[____|____]/[____|____]/[____|____]<br>day month year	No work is required at this point to ensure this activity is on track.	Some action is required for this activity to become on track.	Much work is required for this activity to become on track.

Activity	Is the activity GREEN, YELLOW, or RED?	Describe status of the activity	Describe how was the issue was resolved	Date [day / month / year]
The date of drug pick-up from the HSD	☐ Green<br>☐ Yellow<br>☐ Red			
Transportation for drugs from HSD to health centre	☐ Green<br>☐ Yellow<br>☐ Red			
Storage of drugs at the health centre?	☐ Green<br>☐ Yellow<br>☐ Red			
The drug stock-card	☐ Green<br>☐ Yellow<br>☐ Red			
Stocks for AL (Coartem, Lumartem)	☐ Green<br>☐ Yellow<br>☐ Red			
Order for re-supply of AL/RDTs, if required	☐ Green<br>☐ Yellow<br>☐ Red			
Communication with staff regarding AL/RDT stocks	☐ Green<br>☐ Yellow<br>☐ Red			
Other activities this period	☐ Green<br>☐ Yellow<br>☐ Red			

THE REPUBLIC OF UGANDA

APPENDIX F. INFORMATION SHEET
Health worker self-observation activities
ACT PRIME Study

Introduction

Dr. Sarah Staedke and colleagues from the Uganda Malaria Surveillance Project / Infectious Diseases Research Collaboration are investigating the provision of health care services in Tororo District. We are doing a research study to see if we can improve the health of children in this area by improving services at government-run health facilities. Certain health centers in Tororo district will be selected to take part in the intervention to improve services, or to continue with their current services. Assignment to the two groups has been determined by a lottery. The chance of being placed into either of the groups is the same.

Why are these self-observation activities being done?

We would like to know more about the interaction between health workers and patients in this area. To do this, we are asking health workers to take part in a series of self-observation activities about their behaviors and experiences interacting with patients and to participate in training in patient-centered services. This information will help us to plan future training activities and health care studies in Tororo District.

What will happen if I take part in these self-observation activities?

We would like you to participate in a series of self-observation activities in which you will reflect on your interpersonal behaviors and how you interact with patients. We will start with a three hour introduction to the planned activities. You will then be asked to complete a series of tasks over a period of 4-5 weeks, which will be linked to training modules in patient-centered services. The self-observational activities involve becoming aware of your behavior and writing a short summary of how your behavior affects those around you and your ability to achieve work goals. On completion of each task, you will be invited to join other health workers to discuss your observations and give support to each other during 4 training sessions on patient-centered services that will cover improving interactions with patients and colleagues, and improving the patient visit. We will ask you to give your written summaries to the workshop trainers. The summaries will be typed and stored electronically. All information gathered will be treated as confidential by the study personnel, and records of the interviews will be kept securely in locked filing cabinets and offices. No personal identification information such as names will be used in any reports arising out of this research. . The data we collect will be used by project investigators and may be shared with other researchers and policy-makers to answer questions about how best to deliver health services.

How long will these self-observation activities last?

The introduction to the self-observation activities will last about 3 hours. Over the 4-5 week period, you will carry out four self-observation tasks and participate in five half-day training sessions in patient-centered services.

Can I stop being in the self-observation activities?

You can decide to stop participating at any time. Just tell the project researcher right away if you wish to stop the activities.

What risks can I expect from participating in the self-observation activities?

Participation in any research study may involve a loss of privacy. Information you provide about your experiences and opinions will be recorded, but your name will not be used in any reports of the information provided. No quotes or other results arising from your participation in this study will be included in any reports, even anonymously, without your agreement. The information obtained from these self-observation activities will be locked at our project offices. We will do our best to make sure that the personal information gathered for this survey is kept private.

Are there benefits to taking part in these self-observation activities?

While we do not anticipate any immediate benefits to you, the self-observation activities may help you to develop an awareness of your interpersonal interactions with your colleagues and patients. Additionally, the information that you provide will help researchers plan for future training activities and health care studies in this area.

What other choices do I have if I do not take part in the self-observation activities?

You are free to choose not to take part in the self-observation activities. If you decide not to take part, there will be no penalty to you.

What are the costs of taking part in these self-observation activities? Will I be paid for taking part in these self-observation activities?

There are no costs to you for taking part in these activities. You will not be paid for taking part in these activities.

What are my rights if I take part in these self-observation activities?

Taking part in these self-observation activities is your choice. You may choose either to take part or not to take part. If you decide to take part in these self-observation activities, you may change your mind at any time. No matter what decision you take, there will be no penalty to you in any way.

Who can answer my questions about the self-observation activities?

You can talk to the researchers about any questions or concerns you have about these self-observation activities. Contact Dr. Sarah Staedke or other members of the Uganda Malaria Surveillance Project / Infectious Diseases Research Collaboration on telephone number 0414-530692. If you have any questions, comments or concerns about taking part in these self-observation activities, first talk to the researchers. If for any reason you do not wish to do this, or you still have concerns about doing so, you may contact Dr Charles Ibingira, Makerere University Faculty of Medicine Research and Ethical Committee at telephone number 0414-530020.

Giving verbal consent to take part in the self-observation activities:

You may keep this information sheet if you wish. Participation in these activities is voluntary. You have the right to decline to take part in the activities, or to withdraw from them at any point without penalty. If you do not wish to take part in the activities, you should inform the researcher now. If you do wish to take part in these activities, you should tell the researcher now, and the introduction session will begin shortly. If you do not agree to quotes or other results arising from your participation in the study being included, even anonymously, in any reports about the study, please tell the researcher now.

THE REPUBLIC OF UGANDA

APPENDIX G: HEALTH WORKER SELF-OBSERVATION ACTIVITY #1: HOW DO YOU LISTEN?

Introduction

You have learned about the practice of self observation which will help you to develop awareness – to be able to "see" how you communicate with others.

When doing the self-observation activities, it is important to be ***focused***. Try to look at one thing so you will be able to see the pattern in what you are doing. You will see what you are doing well and what you need to learn more about. If you look at too much at the same time, you will not see the patterns.

This week we will start with focusing on the communication skill of ***listening***.

How do you listen to others and how do they react?

Think about how you listen to others every day. Do you

- Listen "with open ears and heart" until the person has finished?
- Listen "with your mouth full of words", impatient to explain your view?
- Give your answer or your next question as the person is talking because you believe you know what he/she will say?
- Listen with the intention to really understand the other person's point of view; ask questions to find out more, see the others' point of view and then offer your own ideas?
- Do some of each, depending on the situation and your mood?

There are 3 Steps for this self-observation activity which you can complete over one week as follows:

**Step 1** –How do you listen?	2-3 days
**Step 2** – What are the consequences of listening?	2-3 days
**Step 3** – How do your moods affect how you listen?	1-2 days

Remember these tips for carrying out your observations:

- Carry this page of instructions in a notebook.
- When you plan your workday, plot in one or two times or situations when you know you will be interacting with patients or others, and plan to observe yourself.
- Before the consultation/meeting, read the instructions again to remind yourself what you are looking for.
- Try to be aware during the meeting or conversation how you behave regarding the habit you are observing.

- After the meeting/event, reflect on what you have observed in your own behavior, and make a few notes in your notebook.

Step 1: How do you listen to others?

Here are two ways to help you look at ***how you listen to others***.

Try each of the following over the first 2-3 days.

1 – After a consultation with a patient, take a few minutes to ask yourself:

- How did I listen?
- Did I get the patient's ideas right?
- Did I make the patient feel I was interested in her problem?
- Did I really listen – or was I too much in a hurry?

Make your notes in your notebook about what you learned about your listening.

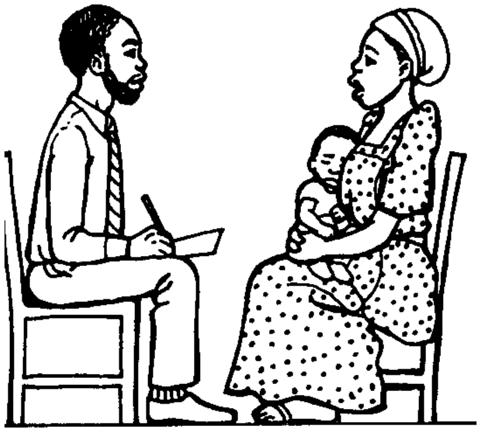

2 – During a meeting with your colleague(s), pay attention to how you listen. After the meeting, take a few moments to ask yourself:

- How well did I listen?
- Did I show interest in the other people's ideas?
- How did I do this, and how did it work?
- If I listened well – what happened to the other person(s)?

Make your notes in your notebook about what you learned about your listening.

Continue to observe how you listen to others as often as possible. Can you start to see your pattern?

After 2-3 days, move on to Step 2.

Step 2: What are the consequences of listening?

Now start looking at ***how your listening affects communication.***

Continue to observe how you listen to others using the instructions in Step 1.

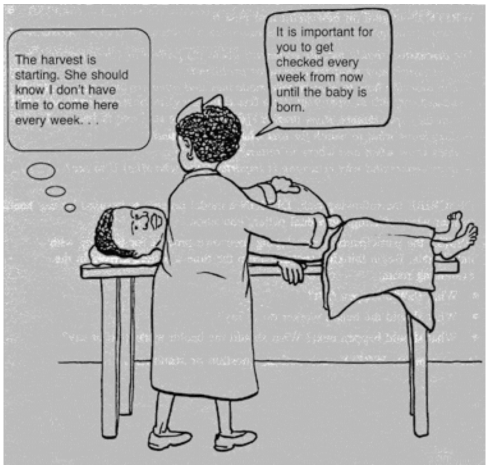

While thinking about how you listen to others, also ask yourself:

- What happens to the other person when I listen well?
- What happens if I don't listen well?

Try looking at yourself during the patient consultation. Try to see when you use the different listening methods and what are the results. Look at what feelings your different listening methods seem to bring out in other people.

Reflect and make notes in your notebook about how your listening affects others.

After 2-3 more days, move to Step 3.

Step 3: How do your moods affect how you listen?

Now start looking at how your different ***moods affect the way you listen***.

Continue to observe how you listen to others. While thinking about how you listen, ask yourself**:**

- What happens to my listening habits when I am feeling fresh & well rested?
- What happens to your listening habits when I am overworked?
- What happens to my listening habits after I have just had a negative interaction with my in-charge?
- What happens to my listening habits when I have many patients to see?
- What happens to my listening habits when I am hungry? Sad/feeling down? Angry/irritated? Frustrated? Worried?
- What effect does the way I am listening and communicating have on my interaction exchange with the patient?

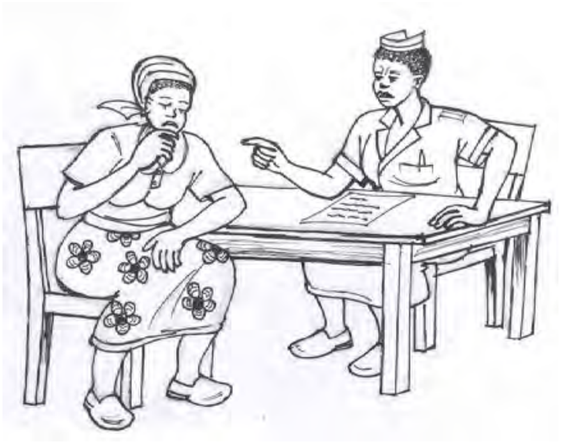

Reflect and makes notes in your notebook about how your moods affect how you listen and the quality of your communication.

Discovering the pattern

Complete Steps 1-3 once or twice a day for a whole week and you will start to see a pattern of how you behave "automatically" when you listen. You will also discover what the effect is on others when you listen well, and not so well.

Discussing with your colleagues

When you return to the next PCS training module, you will have the chance to discuss what you observed with you colleagues.

If you like, you can discuss with your colleagues before the next PCS training. You may want to see:

- How do your colleagues do their observations?
- What have you experienced and found out?

Use the tips on the next page for providing constructive feedback to your colleagues.

THE REPUBLIC OF UGANDA

Guidelines for constructive dialogue with colleagues

When discussing your observations with your colleague, there are two things that can happen depending on how you choose to provide feedback:

Choice 1: Supporting you colleague's motivations and insights

Learning about yourself can be a sensitive issue. When discussing observations with colleagues, show respect and appreciation – then they will feel safe, and you will help them (and yourself) learn well.

How do you do this?

- Ask **open ended questions** to understand more about what colleagues have observed, and listen actively.
- Help him/her think about what they have observed and how they feel about it. What was the effect on the patient/colleague? What did the person get from the experience?
- Be **encouraging and appreciative** to help your colleague talk and share. Then you can share from your observations, and let him/her ask you questions. This gives a good basis for a discussion of how you can use what you have seen, to improve practice.

Choice 2: Turning off your colleague's motivation

Many things we may do automatically without a bad intent, may make your colleague shut up rather than share from her/his experience of doing observations.

Things to avoid:

- Try not to judge or devalue what your colleague has seen and felt, it will hurt, and your colleague might get angry – or close up. With good reason! The message he/she might get from you is: "I know better what this means than you do". You can imagine how this feels and how it affects your communication.
- Try to avoid commenting about your own observations when your colleague has just started to describe his/her own findings. Your focus is then on getting her/him to listen to YOU, rather than you listening to him/her. You also know how this feels. Watch what it does to the communication when you fall into this trap.

Be respectful, appreciative, curious, and generous with your listening!

This does not mean you have to agree with each other - it just means you agree to try to see the other person's point of view, from his/her perspective. This will help us all to learn well together.

THE REPUBLIC OF UGANDA

APPENDIX H.
HEALTH WORKER SELF-OBSERVATION ACTIVITY #2: HOW DO YOU ASK GOOD QUESTIONS?

Introduction

Last week you looked at how you listen in different situations. You saw how you act in certain moods and what happened to the communication you when you listened in different ways.

For example, when listening to others:

- Do you try to really listen to find out what ***their*** ideas are, or are you more concerned about getting the other person to listen to ***your*** opinion and ideas?
- Or do you do a bit of both?
- Do you decide when to do what, or does it happen automatically?

This week we will focus on the “the companion skill” to listening – asking questions.

These two skills – listening and asking – are what you use most in your work. If you practice these skills, it will make a difference to the quality of your work.

How do you ask questions?

There are 4 Steps for this self-observation activity which you can complete over this week as follows:

**Step 1** – How do you ask questions to colleagues?	2 days
**Step 2** – How do you ask questions to patients?	2 days
**Step 3** – Asking questions with awareness	2-3 days
**Step 4** – Making a change	

<u>Remember these tips for carrying out your observations:</u>

- Carry this page of instructions in a notebook.
- When you plan your workday, plot in one or two times or situations when you know you will be interacting with patients or others, and plan to observe yourself.
- Before the consultation/meeting, read the instructions again to remind yourself what you are looking for.
- Try to be aware during the meeting or conversation how you behave regarding the habit you are observing.
- After the meeting/event, reflect on what you have observed in your own behavior, and make a few notes in your notebook.

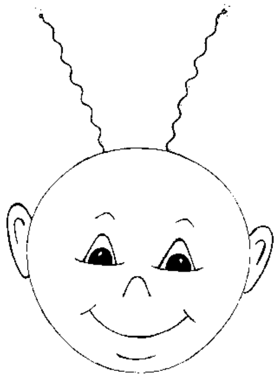

Keep your friend with you to help with your self-observations

THE REPUBLIC OF UGANDA

Step 1: How do you ask questions of *colleague*s?

Here are some ways to help you to see how you are asking questions to colleagues.

During a discussion with a colleague, pay attention to what happens when you ask questions.
Do you:

- Respond (automatically) to his/her statements with your own opinion?
- Ask questions to find out more about what the person is thinking?
- Ask questions that are open-ended and get more information from others?
- Ask closed questions that only have "yes" or "no" answers?

Make notes in your notebook about when you use the different ways of asking questions.

Continue to observe how you ask questions to colleagues as often as possible.

After a few discussions with colleagues, take a few moments to ask yourself:

- What are the ***results of asking questions in different ways***?
- How does it affect: 1) my own feelings?
  2) the other persons' feelings?

Make notes in your notebook about the affect of asking questions in different ways.

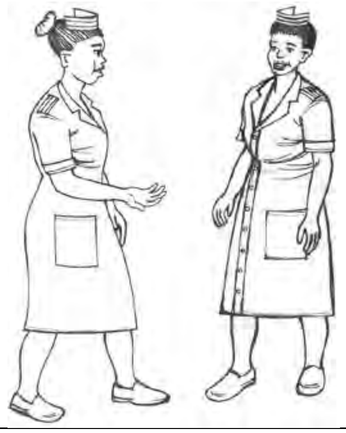

After 2 days, move on to Step 2.

THE REPUBLIC OF UGANDA

Step 2: How do you ask questions of *patients?*

Now try to look at how you ask questions to patients. Here are some ways to help you to see how you are asking questions of your colleagues.

During a patient consultation, pay attention to what happens when you ask questions. Do you:

- Respond (automatically) to his/her statements with your own opinion?
- Ask questions to find out more about what the person is thinking?
- Ask questions that are open-ended and get more information from others?
- Ask closed questions that only have “yes” or “no” answers?

Continue to observe how you ask questions to patients as often as possible.

After a few patient consultations, take a few moments to ask yourself:

- What are the ***results of asking questions in different ways***?
    - o Which questions did I ask that helped the patient to open up and give me information freely?
    - o Which questions did I ask that helped me to get a true picture of the patient’s situation, and helped me to make a good diagnosis?
    - o What kinds of questions did I ask that seemed to force the patient to just agree with me?

Make notes in your notebook about the ***effect*** of asking questions in different ways.

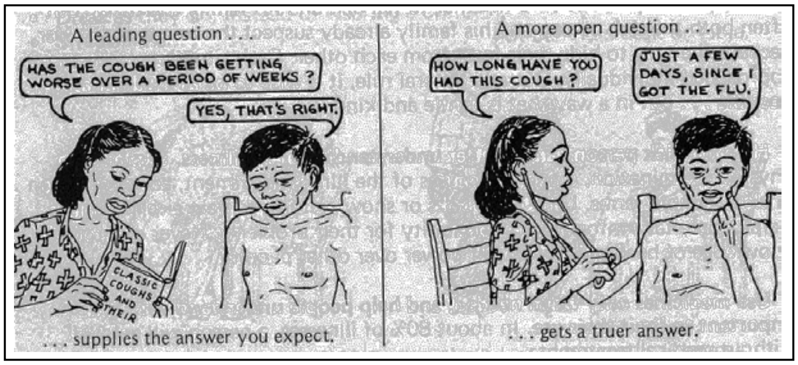

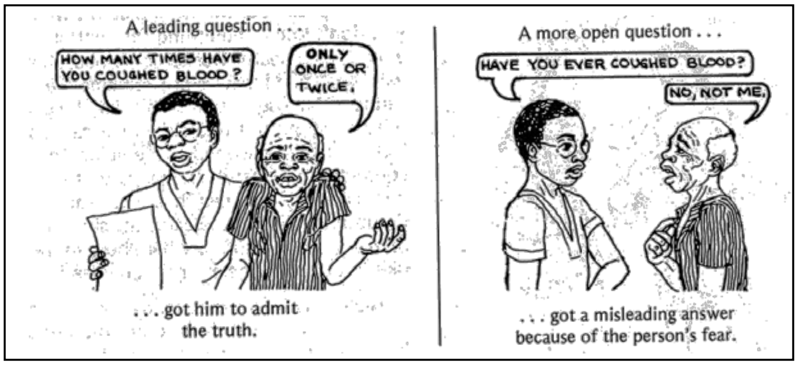

After 2 days, move on to Step 3.

THE REPUBLIC OF UGANDA

Complete Steps 3 and 4 together over the next 2-3 days.

Step 3: Asking questions with awareness

Over the next few days, look at your notes from Steps 1 and 2 to understand more about how you ask questions.

Think about each discussion with colleagues or consultation with patients and ask yourself:

- Did I ask questions automatically, or did I decide with awareness which question to ask? Why?
- Did I look at how the colleague or patient felt before deciding what question to ask?
- Are there times I am more likely to ask closed questions that only invite a "yes" or "no" answer?
- What is the effect of my mood on how I ask questions?

Write down answers to these questions in your notebook.

Do you see a pattern in how you ask questions?

- Is there a way you are asking questions that seems to function well?
- Are there ways that do not give you information?

Step 4 – Making a change

Finally, make "a picture in your head" of how you want your patients to see you.

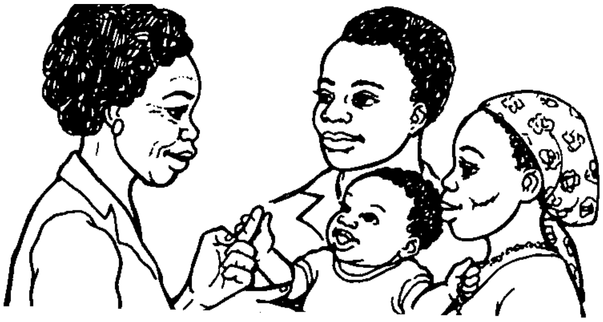

Try to behave in a way to achieve this.
Think about how you feel, when you do.

Think about how others respond to you.

Make notes in your notebook to share with your colleagues at the next PCS training module.

THE REPUBLIC OF UGANDA

APPENDIX I.
HEALTH WORKER SELF-OBSERVATION ACTIVITY #3: WHAT HAPPENS WHEN YOU ARE STRESSED?

Introduction

Over the last two weeks you have looked at how you listen and how you ask good questions of colleagues and patients. You may also already have made some changes in the way you use these skills.

We hope that you are having fun learning about your communication style and discussing what you have learned with your colleagues. We laugh at our experiences in our PCS training modules, and it is good to laugh with your colleagues about your experiences at work. Humor can be very motivating. Just be sure that you are always ‘being constructive’ so that you are not hurting others and making them turn off, if you are being “funny”/sarcastic on their behalf!

If learning is only serious, some people lose interest. A mixture of serious insights and some fun and laughter will bring you the best results when learning new skills.

Stress and how it affects you

This week we will focus on how being stressed influences you and your communication with patients and colleagues. Humor is a good method to prevent stress!

Stress is normal and will continue to come into your life from time to time. Being a health worker can be very stressful at times and many people are very unhappy and even get sick from too much stress. Stress can lead to conflicts with colleagues and patients and can make a health worker make wrong decisions that affect patients. If the stress is not taken care of, it can contribute to burnout and a number of other problems.

There are 4 Steps for this self-observation activity which you can complete over this week as follows:

**Step 1** – What makes you stressed?	2-3 days
**Step 2** – How do you communicate when you are stressed?	
**Step 3** – How does your stress affect others?	1-2 days
**Step 4** – Your stress-reaction pattern	1-2 days

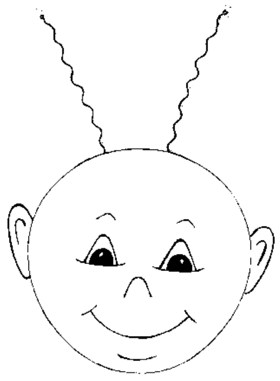

THE REPUBLIC OF UGANDA

Complete Steps 1 and 2 together over the first 2-3 days.

Step 1: What makes you stressed?

On your first day of this self-observation activity, answer the following question in your notebook:

- What is stress?
- How do you know when you are stressed?
- What are the “symptoms” of stress?

Then for the first 2-3 days, pay attention to ***what makes you stressed***.

When something happens that stresses you, make notes in your notebook:

- What was it that made you stressed?
- How did you react?
- Why did you react?

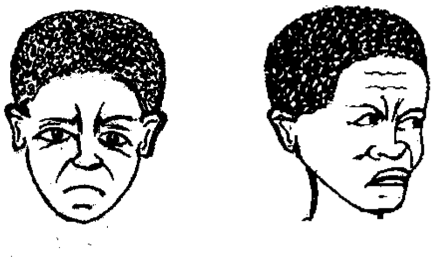

These can be issues related to your relationship with your colleagues or boss, with your patients, or with your daily duties. Or it can be a combination.

Step 2: How do you communicate when you are stressed?

After you have found out a few things that stress you, start to look at ***how you communicate when you are stressed***.

Each time you feel stressed, take a minute to look at yourself and see if you:

- Listen well?
- Ask questions? What kind of questions do you ask?
- Take control/talk a lot?
- Tell people what to do?
- Seek assistance?
- What else do you do?

Make notes in your notebook about the ***effect*** of stress on how you communicate with others.

After 2-3 days, move on to Step 3.

THE REPUBLIC OF UGANDA

Step 3: How does your stress affect others?

After Steps 1 and 2, you should understand what makes you stressed and how you communicate when you are stressed.

Now try to look at the ***effect of your stress on the reaction of the other person***. Each time you react to stress, take a minute to look at yourself and see if the other person:

- Gets defensive
- Starts arguing
- Goes quite and pulls away
- "Attacks" you
- Tries to convince you not to be stressed
- Sees that you are stressed, listens to you, and offers to help
- What else does the other person do?

Make notes in your notebook about how stress affects how others react to you.

After 1-2 days, move on to Step 4

Step 4: Your stress-reaction pattern

After looking at yourself and how you react to stress, you may start to see your ***"stress-reaction" pattern***. Your stress-reaction pattern also affects the reactions you get from the others.

Try to find your stress-reaction pattern.

Look through your notes and see:

- What have you done in each stress situation to reduce the stress or to solve the problem?
- Which strategies do you have that reduce your stress and the stress of patients and colleagues?
- Which reactions from others help you see and reduce your stress?

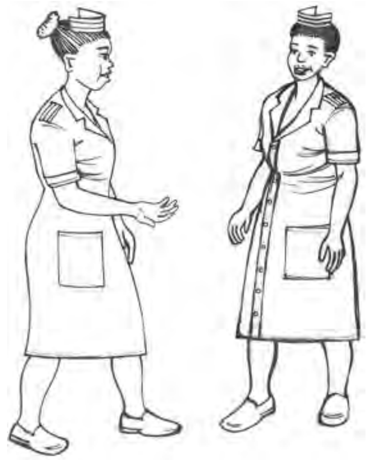

Make notes in your notebook to share with your colleagues at the next PCS training module.

THE REPUBLIC OF UGANDA

APPENDIX J.
HEALTH WORKER SELF-OBSERVATION ACTIVITY #4: HOW DO YOU INVITE THE PATIENT TO COOPERATE?

Introduction

All health workers probably sometimes experience patients as 'difficult' and at other times as very cooperative. This is normal – and it often has to do with how you feel and how well you cope with these feelings. Learning to recognize these feelings and take 'a step back' before you act, will help you maintain good relations with patients and colleagues, even if at first they seem 'difficult'.

Health workers try to see each patient as a person. But, when you are feeling tired and overworked you may 'stop seeing' your patients as 'Mrs Katanga' or 'Mr Kahane', and just see them as 'patients.' This is when you need to take 'a step back.'

Over the last three weeks, you have looked at how you listen, how you ask good questions, and how stress affects your communication with colleagues and patients. Maybe you have changed some of your methods to improve your communication. Many people say that the 'instant feedback' you get when you start listening with more attention, is so rewarding that they continue with the new habits. When they are being appreciated for their communication behavior, it feels good. ***It then becomes easier to treat the patient well.***

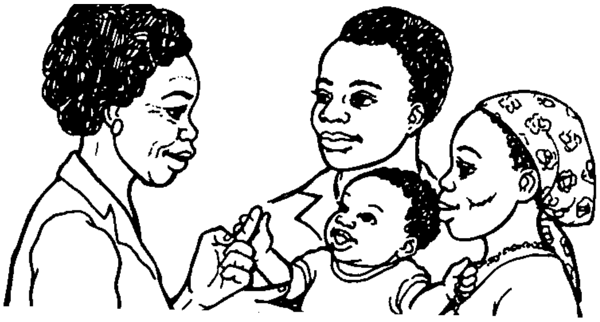

Cooperating with the patient

This week we will focus on putting together the communication skills you have learned over the last few weeks and practice them all with the patient. ***The goal is that that the patient will cooperate well with you and that you establish a good relationship which makes the patient satisfied, well informed about his disease and its treatment, and motivated to follow your advice.***

There are 4 Steps for this self-observation activity which you can complete over this week as follows:

Step 1 – Using your communication skills 2-3 days
Step 2 – Keeping your communication skills 2-3 days

THE REPUBLIC OF UGANDA

Step 1: Using your communication skills

For the first 2-3 days, pay attention to ***how you use the communication skills*** you have learned during your PCS training and self-observation activities.

Each time a patient comes to the health centre, take a few minutes to see how you:

- Greet the patient, and make him/her feel comfortable
- Help the patient to find their way around the health centre: where to go and what to do
- Listen to him/her to find out the patient's thoughts and ideas about the disease
- Ask questions
- Give information, advice, and say what action patient should take
- Any other action you take

Make your notes in your notebook about what you do that seems to have a ***positive effect*** on your communication with the patient and on how the patient cooperates with you.

Make your notes in your notebook about what you do that seems to have a ***negative effect***.
Ask yourself if your problems could have something to do with the way you use non-verbal communication or use medical terms and jargon, or use of a language the person does not understand well, or – anything else?

After 2-3 days, move on to Step 2.

Step 2: Keeping your communication skills

For the rest of the week, look at your overall communication style and interaction with patients. Try to focus on the following three questions:

1 – Do you see a pattern in how you invite the patient to cooperate? What is the pattern? When do you need to take 'a step back'?

- Make notes in your notebook of the actions you take to successfully invite the patient to cooperate.

2 – Do you have 'a picture in your head' of how you want your patients to see you? How do you behave to achieve this 'picture in your head'?

- Make notes in your notebook of the successes and 'failures' of behaving in this way.

3 – How can you work with others to improve the patients' visits to the health centre?

- Make notes about how you can work with other health centre staff and volunteers to make the patient feel more comfortable and willing to cooperate.

Make notes in your notebooks to discuss with your colleagues at the next PCS training module.

THE REPUBLIC OF UGANDA

SELF-OBSERVATION ACTIVITIES FOR THE FUTURE

Introduction

It is a good practice to continue with your self-observation activities and self-awareness building. It is important to always use your 'antennae', 'observer', or 'invisible friend' to help you maintain your self-awareness about how you interact with others.

Repeat the self-observation activity below once every several weeks to help you reflect on and maintain your good communication skills.

Activity: Reflect on your goal

Focus on your communication skills to a "whole". For a few days, reflect generally on your goal as a health worker, and about what kind of professional you want to be, and how you see yourself.

Look at your normal pattern when diagnosing and advising the patient and reflect on the following questions.

Do you sometimes find that you want to…

- Show the patient you are a good professional?
- Show him that you are the one who knows what the problem is, and what to do (rather than listen to his ideas about what is wrong)?
- Show him you have the power, and that he should respect you?
- Expect him to follow your advice, because you have the expertise (and regardless of how you act towards him)?
- Establish a dialogue and a common goal with the patient?

What effect or impact does it have, when you choose different options, on:

- Your communication with the patient
- The outcome of your interaction with the patient
- How you feel about yourself and the service you provided

Make notes in your notebooks and compare with your responses from previous self-observation activities.

Also try to discuss your successes and challenges with your colleagues. Your learning will be more effective if you discuss you observation and questions with your colleagues.

You may want to ask a colleague to observe you and give feedback on how you communicate. Remember to use the guideline on constructive dialogue from Self-Observation Activity #1.

APPENDIX K: CROSS-SECTIONAL SURVEY SCREENING FORM

PART 1: HOUSEHOLD & PARTICIPANT ID

Subcounty ID	Village ID	Compound number	Household number	Cluster number
[_____]	[_____|_____]	[_____|_____|_____|_____]	[_____|_____]	[_____|_____]

Screening Date	Screening ID
[_____|_____]/[_____|_____]/[_____|_____]<br>day month year	[_____|_____|_____|_____]

Age		Gender	
[_____|_____] / [_____|_____]<br>years months	If child is less than 1 year, complete months, otherwise leave blank	[____]	1 = Male<br>2 = Female

PART 2: SCREENING INTERVIEW with PARENTS/GUARDIANS

Selection criteria	Include	Exclude	
1. Appropriate age<br>a. Under five (aged 0 to less than 5 years)<br>b. Aged 5 to 15 years	1 = Yes	2 = No	[_____]
2. Willingness of parent(s)/guardian(s) to provide informed consent	1 = Yes	2 = No	[_____]

If any answers are '2' from the EXCLUDE column, exclude from the study. If not, proceed to the next section.

PART 3: AVAILABILITY OF CHILD

	DATE APPROACHED:	Child available to participate in survey?		**IF YES,** go to Section 4.<br>**IF NO,** GIVE REASON*	DATE TO RETURN:
**1**		Yes	No		
**2**		Yes	No		
**3**		Yes	No		
**4**		Yes	No		xxxxxxxxxxxxxxxxx

* **1**=Not Home, **2**=Come back later, **3**=Not interested, **4**=Vacant, **77**=Other (Please specify above)

PART 4: SCREENING INTERVIEW with CHILD

Selection criteria	Include	Exclude	
3. Able to locate child	1 = Yes	2 = No	[_____]
4. Willingness of child over 8 years to provide assent to participate	1 = Yes	2 = No	[_____]

If any answers are '2' from the EXCLUDE column, exclude from the study. If not, proceed to the next section.

ASSIGN STUDY NUMBER	[_____|_____|_____|_____]

All criteria for study inclusion met?	Date of enrollment *(date of survey)*
1 = Yes<br>2 = No *If no, exclude from the study* [_____]	[______|______]/[______|______]/[______|______]<br>day month year

Staff ID: [____|____] **Data entrant (1st):** [____|____] **Data entrant (2nd):** [____|____]

[____|____]　[____]—[____|____]—[____|____|____|____]—[____|____]　[____|____|____|____]

Cluster number　**Household ID**　**CSS Study ID**

APPENDIX L. CROSS-SECTIONAL SURVEY
Research participant informed consent form

**Protocol Title:**	ACT PRIME Study: Evaluating the impact of enhanced health facility-based care for malaria and febrile illnesses in children
**Site of Research:**	Tororo, Uganda
**Principal Investigators**:	Dr. Sarah Staedke
**Date:**	1 November 2012

Introduction

Dr. Sarah Staedke and colleagues from the Uganda Malaria Surveillance Project / Infectious Diseases Research Collaboration are investigating delivery of healthcare services in Tororo District. We are doing a research study to see if we can improve the health of children in this area by improving services at government-run health facilities.

How is this survey being done?

Certain health centers in Tororo district will be selected to either take part in the intervention to improve services, or to continue with their current services. Assignment to the two groups has been determined by a lottery. The chance of being placed into either of the groups is the same. To find out how well the intervention is working, we would like to review the health of children living near the health centers. We plan to carry out three surveys over about 2 years in children from households in this area. We plan to sample children under five and children aged 5-15 years. A total of 8766 children will be sampled in each survey.

Households will be selected to participate by a lottery. In each household, one child under five and one child aged 5-15 years will be eligible to take part. If more than one child in the correct age group(s) lives in the house, we will select one child to take part by a lottery. For the survey, we would like to ask the primary caregiver some questions about bednets and treatment of fever in children. We would also like to ask all women of child-bearing age living in your household some questions about their children. We would also like to examine your child and carry out some laboratory tests.

What will happen if my child takes part in this survey?

If you agree to let your child participate in this survey, the following will happen today:

a) We will ask the primary caregiver some questions about him/herself, use of bednets to prevent malaria, and practices for treatment of fever in children.
b) We will ask all women of child-bearing age living in your household some questions about their children.
c) We will collect information on your child's general health.
d) We will briefly examine your child.
e) A blood sample will be taken from your child's finger to examine for malaria parasites and to measure blood counts.
f) If your child has had a fever in the last 48 hours (2 days) or has a high temperature, we will do a rapid diagnostic test for malaria.
g) If your child has a positive test for malaria, we will provide treatment with artemether-lumefantrine (including Coartem or Lumatem), which is the recommended treatment for simple malaria in Uganda.
h) If your child has a negative test for malaria, has a low blood count, or has signs of severe malaria or another significant illness, we will refer you and your child to an appropriate health center or hospital for further care.
i) The data we collect will be used by project investigators and may be shared with other researchers and policy-makers to answer questions about how best to deliver health services.

How long will these activities last?
Today the survey activities, including questions, examination, and blood tests will take about 1 hour.

Can I stop my child from being in the survey?
You can decide to stop participating at any time. Just tell our study personnel right away if you wish to stop the activities.

What risks can I expect if my child participates in the survey?
We will obtain one blood sample by fingerprick from your child. The risks of drawing blood from a fingerprick include temporary discomfort from the needle stick, bruising, and skin infection. The amount of blood removed will be too small to affect your child's health.

Participation in any research study may involve a loss of privacy. Information you provide will be recorded, but your name, and your child's name, will not be used in any reports of the information provided. The information obtained from these survey activities will be locked at our project offices. We will do our best to make sure that any personal information is kept private.

Are there benefits to letting my child take part in the survey?

Through the intervention, we aim to improve the health of children in this area by improving services at the health centers. There will be no direct benefit to you from participating in this study. However, the information that we gather in this survey will help researchers and policy-makers understand how best to improve health services in this area.

What other choices do I have if I do not allow my child to take part in the survey?

You are free to choose not to participate in the survey. If you decide not to take part, there will be no penalty to you.

What are the costs of taking part in the survey? Will I be paid for letting my child take part in the survey?

You and your child will not be charged for any of the treatments or procedures we perform today. However, if we refer your child for further evaluation and health care, you will be responsible for all costs for your child's health care. You and your child will not be paid for participation in the survey.

What are my rights if I allow my child to take part in the survey?

Taking part in this survey is your choice. You may choose either to take part or not to take part. If you decide to take part in this survey, you may change your mind at any time. If you decide to withdraw your child from the survey; your child will still be eligible for care at the local health facility and at Tororo District Hospital and at other local clinics. No matter what decision you take, there will be no penalty to you in any way.

What if my child is injured as result of being in this survey?

If your child is injured, or if you have questions about injuries as a result of being in the survey, please contact Dr. Sarah Staedke or other members of the Uganda Malaria Surveillance Project / Infectious Disease Research Collaboration on telephone number 0414-530692. The sponsoring organizations do not have a program to cover your costs if your child is hurt or has other bad results.

Who can answer my questions about the survey?

You can talk to the researchers about any questions or concerns you have about these survey activities. Contact Dr. Sarah Staedke or other members of the Uganda Malaria Surveillance Project / Infectious Disease Research Collaboration on telephone number 0414-530692. If you have any questions, comments or concerns about taking part in these activities, first talk to the researchers. If for any reason you do not wish to do this, or you still have concerns about doing so, you may contact Professor James Tumwine, Makerere University School of Medicine Research and Ethical Committee at telephone number 0414-530020.

WHAT YOUR SIGNATURE OR THUMBPRINT MEANS

Your signature or thumbprint below means that you understand the information given to you in this consent form about your child's participation in the survey and agree with the following statements:

1. "I have read the consent form concerning this survey (or have understood the verbal explanation of the consent form) and I understand what will be required of me and what will happen to me and my child if we take part in it."

2. "My questions concerning this survey have been answered by Dr. Staedke or the person who signed below."

3. "I understand that at any time, I may withdraw my child from this survey without giving a reason and without affecting my child's normal health care and management."

4. "I agree that the child under my care will take part in this survey."

If you wish your child to participate in this survey, you should sign or place your thumbprint below.

THE REPUBLIC OF UGANDA

WE WILL GIVE YOU A COPY OF THIS SIGNED AND DATED CONSENT FORM

Name of Participant (printed)

Name of Parent/Guardian

Signature or Fingerprint * of Parent/Guardian Date/Time

Name of Investigator Administering Consent (printed) Position/Title

Signature of Investigator Administering Consent Date/Time

*If the parent or guardian is unable to read and/or write, an impartial witness should be present during the informed consent discussion. After the written informed consent form is read and explained to the participant and parent or guardian, and after they have orally consented to their child's participation in the trial, and have either signed the consent form or provided their fingerprint, the witness should sign and personally date the consent form. By signing the consent form, the witness attests that the information in the consent form and any other written information was accurately explained to, and apparently understood by the parent or guardian, and that informed consent was freely given by the patient and parent or guardian.

Name of Person Witnessing Consent (printed)

Signature of Person Witnessing Consent Date/Time

THE REPUBLIC OF UGANDA

[____|____]　[____]—[____|____]—[____|____|____|____]—[____|____]　[____|____|____|____]

Cluster number　**Household ID**　**CSS Study ID**

APPENDIX M. CROSS-SECTIONAL SURVEY
Informed consent for future use of biological specimens

**Protocol Title:**	ACT PRIME Study: Evaluating the impact of enhanced health facility-based care for malaria and febrile illnesses in children
**Site of Research:**	Tororo, Uganda
**Principal Investigators**:	Dr. Sarah Staedke
**Date:**	23 December 2010

INTRODUCTION

While your child is in this study, there may be blood samples taken from them that may be useful for future research. These samples will be stored long-term at Makerere University Medical School and the London School of Hygiene and Tropical Medicine, and the University of California, San Francisco. Samples may also be shared with investigators at other institutions.

WHAT SAMPLES WILL BE USED FOR

Your child's blood and the malaria parasites in it will be used to study malaria and the response of this disease to treatment. Results of these studies will not affect your child's care.

1. These samples will be used for future research to learn more about malaria and other diseases.
2. Your child's samples will be used only for research and will not be sold or used for the production of commercial products.
3. Genetic research may be performed on samples. However, no genetic information obtained from this research will be placed in your child's medical records. These samples will be identified only by codes so that they cannot be readily identified with your child.

LEVEL OF IDENTIFICATION

Your child's samples will be coded so that your child's name cannot be readily identified. Reports about research done with your child's samples will not be put in their medical record and will be kept confidential to the best of our ability. In the future, researchers studying your child's samples may need to know more about your child, such as their age, gender, and race. If this information is already available because of your child's participation in a study, it may be provided to the researcher. Your child's name or anything that might identify them personally will not be provided. You will not be asked to provide additional consent.

THE REPUBLIC OF UGANDA

RISKS

There are few risks to your child from future use of their samples. A potential risk might be the release of information from your child's health or study records. Reports about research done with your child's samples will not be put in their health record, but will be kept with the study records. The study records will be kept confidential as far as possible.

BENEFITS

There will be no direct benefit to your child. From studying your child's samples we may learn more about malaria or other diseases: how to prevent them, how to treat them, how to cure them.

RESEARCH RESULTS/MEDICAL RECORDS

1. Results from future research using your child's samples may be presented in publications and meetings but patient names will not be identified.
2. Reports from future research done with your child's samples will not be given to you or your child's doctor. These reports will not be put in your child's medical record.

QUESTIONS

If you have any questions, comments or concerns about the future use of your child's specimen's, first talk to the researchers. You may also Contact Dr. Sarah Staedke or other members of the Uganda Malaria Surveillance Project on telephone number 0414-530692. If for any reason you do not wish to do this, or you still have concerns about the future use of your child's specimens, you may contact Dr Charles Ibingira, Makerere University Faculty of Medicine Research and Ethical Committee at telephone number 0414-530020.

FREEDOM TO REFUSE

You can change your mind at any time about allowing your child's samples to be used for future research. If you do, contact Dr. Staedke or other members of the Uganda Malaria Surveillance Project at the numbers listed above. Then your child's samples will no longer be made available for research and will be destroyed. Whether or not you allow us to use your child's samples in future research will not have any effect on your child's participation in this study or future participation in other studies.

WHAT YOUR SIGNATURE OR THUMBPRINT MEANS

Your signature or thumbprint below means that you understand the information given to you in this consent form about your child's specimens to be used for future research. If you wish to allow your child's specimens to be used for future research, you should sign or place your thumbprint below.

THE REPUBLIC OF UGANDA

WE WILL GIVE YOU A COPY OF THIS SIGNED AND DATED CONSENT FORM

____
Name of Participant (printed)

____
Name of Parent/Guardian

____
Signature or Fingerprint * of Parent/Guardian Date/Time

____
Name of Investigator Administering Consent (printed) Position/Title

____
Signature of Investigator Administering Consent Date/Time

*If the parent or guardian is unable to read and/or write, an impartial witness should be present during the informed consent discussion. After the written informed consent form is read and explained to the participant and parent or guardian, and after they have orally consented to their child's participation in the trial, and have either signed the consent form or provided their fingerprint, the witness should sign and personally date the consent form. By signing the consent form, the witness attests that the information in the consent form and any other written information was accurately explained to, and apparently understood by the parent or guardian, and that informed consent was freely given by the patient and parent or guardian.

____
Name of Person Witnessing Consent (printed)

____
Signature of Person Witnessing Consent Date/Time

[____|____] [____]—[____|____]—[____|____|____|____]—[____|____] [____|____|____|____]

Cluster number **Household ID** **CSS Study ID**

APPENDIX N. CROSS-SECTIONAL SURVEY
Research participant assent form for children

**Protocol Title:**	ACT PRIME Study: Evaluating the impact of enhanced health facility-based care for malaria and febrile illnesses in children
**Site of Research:**	Tororo, Uganda
**Principal Investigators**:	Dr. Sarah Staedke
**Date:**	1 November 2012

Why have we met you ?
We want to tell you about a research study. A research study is when doctors collect information to learn more about something. After we tell you about it, we will ask if you'd like to be in this research study or not.

Why are doctors doing this research?
Dr. Sarah Staedke and colleagues from the Uganda Malaria Surveillance Project / Infectious Diseases Research Collaboration are doing this research study to see if we can improve the health of children in this area by improving health care given at government-run health facilities.

How is this research being done?
Certain health centers in Tororo district will be selected to either take part in the intervention to improve services, or to continue with their current services. Assignment to the two groups has been determined by a lottery. The chance of being placed into either of the groups is the same. To find out how well the intervention is working, we would like to review the health of children living near the health centers. We plan to carry out three research studies over about 2 years in children from households in this area. We plan to sample children under five and children aged 5-15 years. A total of 8766 children will be sampled in each survey.

Households will be selected to participate by a lottery. In each household, one child under five and one child aged 5-15 years will be eligible to take part. If more than one child in the correct age group(s) lives in the house, we will select one child to take part by a lottery. For the research, we would like to ask the primary caregiver some questions about bednets and treatment of fever in children. We would also like to ask all women of child-bearing age living in your household some questions about their children. We would also like to examine you and carry out some laboratory tests.

What will happen if I take part in this research?

If you agree to take part in this research, the following will happen today:

a) We will ask your primary caregiver some questions about him/herself, use of bednets to prevent malaria, and practices for treatment of fever in children.
b) We will ask all women of child-bearing age living in your household some questions about their children.
c) We will collect information on your general health.
d) We will briefly examine you.
e) A blood sample will be taken from your finger to examine for malaria parasites and to measure blood counts.
f) If you have had a fever in the last 48 hours (2 days) or have a high temperature, we will do a rapid diagnostic test for malaria.
g) If you have a positive test for malaria, we will provide treatment with artemether-lumefantrine (including Coartem or Lumatem), which is the recommended treatment for simple malaria in Uganda.
h) If you have a negative test for malaria, have a low blood count, or have signs of severe malaria or another significant illness, we will refer you to an appropriate health center or hospital for further care.
i) The data we collect will be used by project investigators and may be shared with other researchers and policy-makers to answer questions about how best to deliver health services.

How long will these research activities last?

Today the research activities, including questions, examination, and blood tests will take about 1 hour.

Can I stop being in the research?

You can decide to stop taking part in the research at any time. Just tell our study personnel right away if you wish to stop the activities.

What risks can I expect if I participate in the research?

We will obtain one blood sample by fingerprick from you. The risks of drawing blood from a finger prick include feeling pain, but it will stop quickly. You may also get a small wound from the needle stick. The amount of blood removed will be too small to affect your health.

THE REPUBLIC OF UGANDA

Participation in any research study means sometimes we may ask you questions that make you feel uncomfortable but we shall make sure this does not happen many times. Information you provide will be recorded, but your parent's/guardian's name, and your name, will not be used in any reports of the information provided. The information obtained from these research activities will be locked at our project offices. We will do our best to make sure that anything you tell us and your records are kept private.

What will you gain from this research?

Through the research, we aim to improve the health of children in this area by improving the health care given at the health centers. There will be no direct benefit to you from taking part in this study. However, the information that we gather in this research will help researchers and policy-makers understand how best to improve health services in this area.

What other choices do I have if I do not allow to take part in the research?

You are free to choose not to take part in the research. If you decide not to take part, there will be no punishment to you.

What are the costs of taking part in the research? Will I or my parents/guardians be paid if I take part in the research?

You and your parent's/guardians will not pay for any of the treatments or procedures we perform today. However, if we refer you for further check up and health care, your parent's/guardians will have to pay for all costs for your health care. You and your parent's/guardians will not be paid for taking part in the research.

What are my rights if I allow to take part in the research?

Taking part in this research is your choice. You may choose either to take part or not to take part. If you decide to take part in this research, you may change your mind at any time. If you decide to stop being in the research ; you will still be able to get care at the local health facility and at Tororo District Hospital and at other local clinics. No matter what decision you take, there will be no punishment to you in any way.

What if I am injured as result of being in this research?

If you are injured, or if you have questions about injuries as a result of being in the research, please call Dr. Sarah Staedke or other members of the Uganda Malaria Surveillance Project / Infectious Disease Research Collaboration on telephone number 0414-530692. The sponsoring organizations do not have a program to cover your costs if you are hurt or have other bad results.

THE REPUBLIC OF UGANDA

Who can answer my questions about the research?

You can talk to the researchers about any questions or concerns you have about these survey activities. Contact Dr. Sarah Staedke or other members of the Uganda Malaria Surveillance Project / Infectious Disease Research Collaboration on telephone number 0414-530692. If you have any questions, comments or concerns about taking part in these activities, first talk to the researchers. If for any reason you do not wish to do this, or you still have concerns about doing so, you may contact Professor James Tumwine, Makerere University School of Medicine Research and Ethical Committee at telephone number 0414-530020.

THE REPUBLIC OF UGANDA

WHAT YOUR SIGNATURE OR THUMBPRINT MEANS

Your signature or thumbprint below means that you understand the information given to you in this assent form about your taking part in the research and you agree with the following statements:

— I am being asked to decide if I want to be in this research study.
— I know that I will have to see the survey field workers today.
— The field workers will talk to me, ask me questions, and examine me.
— I know I will have a few drops of blood drawn from my finger today.
— I asked and got answers to my questions. I know that I can ask questions about this survey at any time.
— I know that I can stop being in this survey at any time without anyone being mad at me.

Mark one box with **X**:

I DO CONSENT: ☐ **I hereby agree to take part in this survey**

I DO <u>NOT</u> CONSENT: ☐ **I do not wish to take part in this survey**

Name of child:	
Signature or fingerprint of child:	Date:

<u>Witness</u>: I hereby confirm that the study has been explained to the child. All questions (if any) have also been answered to his/her satisfaction, and he/she has, of his own free will, consented to take part in the survey.

Name of witness:	
Signature of witness:	Date:

Name of person explaining study:	
Signature:	Date:

Variable Name	Question	Variable Type	Variable Codes	Accepted Values	Skip	Comments
**Appendix O: Part 1 - Household Survey**						
**Household Unique Identification**- *this information is unique for each household on recruitment list, to be programmed as a primary key (to avoid duplicates)*						
VISDATE	Date of visit	Date				
STARTIME	Start time of interview	String (HH:SS)				automatically generated
CLUSTER	Cluster number	Numeric		1 - 20		data is recorded twice to ensure data integrity
HHID	Household ID	Numeric		101000101 - 799999999		
STAFFID	Staff ID	Numeric		1 - 99		
NUMCHLDN	Number of children in household	Numeric		1 - 6		
STUDYID1	Study ID for child 1	Numeric		1001 - 1999		data is recorded twice to ensure data integrity
...	...	...		...		
STUDYID6	Study ID for child 1	Numeric		1001 - 1999		
AGREE	Are you going to conduct the interview with this household?	Numeric	1-Yes, 2-No	1, 2		
**Section 1: Head of household's (Respondent's) Background**						
HHEAD	Are you the head of the household?	numeric	1-Yes, 2-No	1, 2		
HHAGEYRS	How old are you?	Numeric	888 - Don't know<br>999 - Refused to answer	10 - 120, 888, 999		
HHGEND	Gender for head of household	Numeric	1- Male, 2-Female	1, 2		
HHSCHOOL	Have you ever attended school?	Numeric	1 - Yes<br>2 - No<br>99 - Refused to answer	1, 2, 3	if <> 1 then skip to HHJOBTRN	
HHHIGHEST	What is the highest level of school you attended?	Numeric	0 - None<br>1 - Primary (P1-P4)<br>2 - Primary (P5-P7)<br>3 - Secondary (S1-S4)<br>4 - Secondary(S5-S6)<br>10 - Certificate/Diploma<br>11 - University<br>88 - Don't know<br>99 - Refused to answer	0 - 11, 77, 88, 99		
HHJOBTRN	Have you completed any other type of job training school?	Numeric	1 - Yes<br>2 - No<br>99 - Refused to answer	1 , 2, 99	If 1is not head of household, skip HHREAD	
HHREAD	"Now I would like you to read a sentence. In what language do you read?" SELECT AND SHOW THE APPROPRIATE LITERACY CARD TO CAREGIVER. LISTEN AND GRADE THE READING EFFORT. *If cannot read skip to question #17	Numeric	1 - Cannot read at all*<br>2 - Can read only parts of the sentence<br>3 - Able to read the whole sentence<br>4 - No card with required language<br>99 - Refused to answer	1, 2, 3, 4, 99	if 1, skip to HHRADIO	

Variable Name	Question	Variable Type	Variable Codes	Accepted Values	Skip	Comments
HHNEWSPR	Do you read a newspaper or magazine almost every day, at least once a week, less than once a week or not at all?	Numeric	1 - Almost every day<br>2 - At least once a week<br>3 - At least once a month<br>4 - Not at all<br>5 - Cannot read<br>99 - Refused to answer	1,2,3,4,5,99		
HHRADIO	Do you listen to the radio almost every day, at least once week, less than once a week or not at all?	Numeric	1 - Almost every day<br>2 - At least once a week<br>3 - At least once a month<br>4 - Not at all<br>99 - Refused to answer	1,2,3,4,99		
HHTV	Do you watch television almost every day, at least once a week, less than once a week or not at all?	Numeric	1 - Almost every day<br>2 - At least once a week<br>3 - At least once a month<br>4 - Not at all<br>99 - Refused to answer	1,2,3,4,99		
HHJOB	What is the main activity or job you do to provide your family with income OR "*If you do not have regular employment, what other things do you do to help provide your family with income?*	Numeric	1 - Peasant farmer<br>2 - Student<br>3 - Military/police/security<br>4 - Market vendor<br>5 - Selling goods/shopkeeper/businessman<br>6 - Transport: Taxi, Bodaboda, bicycle rider<br>7 - Selling food sfuffs (catering/food vendor)<br>8 - Construction worker/Casual labor<br>77 - Other _________________	1,2,3,4,5,6,7,8,77	if <> 77, skip to HHWORK12	
OHHJOB	Specify other main activity or Job	string				
HHWORK12	Have you done any work in the last 12 months?	Numeric	1 - Yes<br>2 - No<br>99 - Refused to answer	1,2,99	if <> 1, SKIP to HHWORKJOB	
HHPAID	Are (were) you paid in cash or kind for this work or are (were) you not paid at all?<br><br>IF PAID, ASK WHETHER CASH, IN-KIND, OR BOTH.	Numeric	1 - Cash only<br>2 - Cash and kind<br>3 - In-kind only<br>4 - Not paid<br>99 - Refused to answer	1,2,3,4,99		
HHWORKJOB	if you have NOT done any work in the last 12 months,what have you been doing for most of the time during this period?	Numeric	1 - going to school/studying<br>2 - looking for work<br>3 - retired<br>4 - too ill to work<br>5 - handicapped, cannot work<br>6 - housework/child care<br>7- Peasant farmer<br>77 - other<br>99 - Refused to answer	1,2,3,4,5,6,7,77,99	if = 77, specify other	
HHWORKOT	Specify other non-work activity	String				

Variable Name	Question	Variable Type	Variable Codes	Accepted Values	Skip	Comments
RESIDENT	How many people usually live in your household (total number including children)?	Numeric		1-32		
WOMENCBA	How many women of child-bearing age (13-49 years) live in your household ?	Numeric		0 - 20		Refer to women that have lived in this HH for at least 6 months
INFOHEAD	Who is providing information for the Head of the household?	Numeric	1- Head of household<br>2- Primary caregive<br>3- Other available adult	1,2,3		
**Section 2: Primary Caregiver Background**						
GIVER	Are you the Primary care giver of this household?	Numeric	1-Yes, 2-No	1,2	If yes, and is head of household, [illegible]	
PCAGEYRS	How old are you?	Numeric	888- Don't know<br>999 - Refused to answer	10 - 120, 888, 999	- if < 13 or > 49, skip to section 6.<br>- if refuse to answer, skip to section 6	
PCSCHOOL	Have you ever attended school?	Numeric	1 - Yes<br>2 - No<br>99 - Refused to answer	1,2,99	if Not 1 then skip to PCJOBTRN	
PCHIGHEST	What is the highest level of school you attended?	Numeric	1 - Primary (P1-P4)<br>2 - Primary (P5-P7)<br>3 - Secondary (S1-S4)<br>4 - Secondary(S5-S6)<br>10 - Certificate/Diploma<br>11 - University<br>88 - Don't know<br>99 - Refused to answer	1,2,3,4,10,11,88,99		
PCJOBTRN	Have you completed any other type of job training school?	Numeric	1 - Yes<br>2 - No<br>9 - Refused to answer	as per variable codes		
PCREAD	"Now I would like you to read a sentence. In what language do you read?" SELECT AND SHOW THE APPROPRIATE LITERACY CARD TO CAREGIVER. LISTEN AND GRADE THE READING EFFORT.<br>*If cannot read skip to question #17	Numeric	1 - Cannot read at all*<br>2 - Can read only parts of the sentence<br>3 - Able to read the whole sentence<br>4 - No card with required language (specify language) ___________________<br>99 - Refused to answer	as per variable codes	if equal to 1, skip to PCRADIO<br>IF 77, specify other	
PCNEWSPR	Do you read a newspaper or magazine almost every day, at least once a week, less than once a week or not at all?	Numeric	1 - Almost every day<br>2 - At least once a week<br>3 - At least once a month<br>4 - Not at all<br>8 - Cannot read<br>99 - Refused to answer	as per variable codes		
PCRADIO	Do you listen to the radio almost every day, at least once week, less than once a week or not at all?	Numeric	1 - Almost every day<br>2 - At least once a week<br>3 - Less than once a week<br>4 - Not at all<br>99 - Refused to answer	as per variable codes		

Variable Name	Question	Variable Type	Variable Codes	Accepted Values	Skip	Comments
PCTV	Do you watch television almost every day, at least once a week, less than once a week or not at all?	Numeric	1 - Almost every day<br>2 - At least once a week<br>3 - At least once a month<br>4 - Not at all<br>99 - Refused to answer	as per variable codes		
PCJOB	What is your occupation, that is, what kind of work do you mainly do?<br><br>INTERVIEWER: PROBE TO OBTAIN DETAILED INFORMATION ON THE KIND OF WORK RESPONDENT DOES.	Numeric	1 - Peasant farmer<br>2 - Student<br>3 - Military/police/security<br>4 - Construction worker/casual labourer<br>5- Housekeeper<br>6 - Selling goods/shopkeeper/businessman<br>7- Selling food sfuffs (catering/food vendor)<br>8 - Taxi, bodaboda or bicycle rider<br>9 - Mechanic<br>10 - Government/clerical/secretarial work.<br>11 - Health worker<br>12 - Teacher<br>13 - None/unemployed<br>77 - Other ________________<br>99 - Refused to answer	as per variable codes		
OPCJOB	Specify other main activity or Job	String				
PCWORK12	Have you done any work in the last 12 months to earn you an income?	Numeric	1 - Yes<br>2 - No<br>9 - Refused to answer	as per variable codes	if Not equal to 1 , skip to PCWORKJB	
PCPAID	Are (were) you paid in cash or kind for this work or are (were) you not paid at all?<br><br>IF PAID, ASK WHETHER CASH, IN-KIND, OR BOTH.	Numeric	1 - Cash only<br>2 - Cash and kind<br>3 - In-kind only<br>4 - Not paid<br>99 - Refused to answer	as per variable codes	If answered, skip to Section 3	
PCWORKJB	if you have NOT done any work in the last 12 months,what have you been doing for most of the time during this period?	Numeric	1 - going to school/studying<br>2 - looking for work<br>3 - retired<br>4 - too ill to work<br>5 - handicapped, cannot work<br>6 - housework/child care<br>77 - other<br>97 - Not applicable	as per variable codes	if = 77, specify other	
PCWORKOT	Specify other non-work activity	String				
**Section 3: Household Characteristics (Wealth Indicators)**						

Variable Name	Question	Variable Type	Variable Codes	Accepted Values	Skip	Comments
SWATER	What is the main source of drinking water for members of your household?	Numeric	10 - borehole<br>11 - pipe into dwelling<br>12 - piped into yard/compound<br>13 - public tap<br>21 - open well in yard/compound<br>22 - open public well<br>31 - protected well in yard/compound<br>32 - protected public well<br>41 - protected spring<br>42 - unprotected spring<br>51 - Surface water(river,stream,lake,dam)<br>61 - Rain water<br>62 - Water from tanker truck<br>63 - Water from vendor<br>64 - Bottled water<br>77 - other<br>99 - Refused to answer	as per variable codes	if = 77 then specify Other	
OTHERSCS	Specify other source of water	String				
TFACLTY	What kind of toilet facility do members of your household usually use?	Numeric	1 - flush toilet<br>2 - vip latrine<br>3 - covered pit latrine no slab<br>4 - covered pit latrine w/slab<br>5 - uncovered pit latrine no slab<br>6 - uncovered pit latrine w/slab<br>7 - composting toilet<br>8 - no facility/bush/field<br>77 - other<br>88- Dont know<br>99 - Refused to answer	as per variable codes	if = 77 then specify Other	
OTHERFCY	Specify other kind of toilet facilities	String				
ELECTIRC	Does your household have...<br><br>...Electricity?	Numeric	1 - Yes<br>2 - No<br>88 - Don't know<br>99 - Refused to answer	as per variable codes		
RADIO	...Radio?	Numeric	1 - Yes<br>2 - No<br>88 - Don't know<br>99 - Refused to answer	as per variable codes		
TV	...Television?	Numeric	1 - Yes<br>2 - No<br>88 - Don't know<br>99 - Refused to answer	as per variable codes		
MOBILE	...Mobile phone?	Numeric	1 - Yes<br>2 - No<br>88 - Don't know<br>99 - Refused to answer	as per variable codes		

Variable Name	Question	Variable Type	Variable Codes	Accepted Values	Skip	Comments
FRIDGE	...Refrigerator?	Numeric	1 - Yes<br>2 - No<br>88 - Don't know<br>99 - Refused to answer	as per variable codes		
SOFA	...Sofa set?	Numeric	1 - Yes<br>2 - No<br>88 - Don't know<br>99 - Refused to answer	as per variable codes		
BED	...Bed?	Numeric	1 - Yes<br>2 - No<br>88 - Don't know<br>99 - Refused to answer	as per variable codes		
CLOCK	...Clock?	Numeric	1 - Yes<br>2 - No<br>88 - Don't know<br>99 - Refused to answer	as per variable codes		
FUELTYPE	What type of fuel does your household mainly use for cooking?	Numeric	1 - electricity<br>2 - lpg/natural gas<br>3 - biogas<br>4 - paraffin/kerosene<br>5 - charcoal<br>6 - firewood<br>7 - starw/shrubs/grass<br>8 - animal dung<br>95 - no food cooked in household<br>77 - other<br>99 - Refused to answer	as per variable codes	if = 77 then specify Other	
OTHERFUE	Specify other type of fuel used	String				
SENERGY	What do you use for lighting energy in this household?	Numeric	1 - electricity<br>2 - solar<br>3 - gas<br>4 - paraffin - hurricane lamp<br>5 - paraffin - pressure lamp<br>6 - paraffin - wick lamp<br>7 - firewood<br>8 - candles<br>77 - other<br>99 - Refused to answer/skipped	as per variable codes	if = 96 then specify Other	
OTHERENG	Specify other source of energy for lighting	String				
MMFLOOR	MAIN MATERIAL OF THE FLOOR<br><br>RECORD OBSERVATION.<br><br>MARK ONLY ONE.	Numeric	11 - earth/sand<br>12 - earth and dung<br>31 - parquet or polished wood<br>33 - mosaic or tiles<br>34 - bricks<br>35 - cement<br>36 - stones<br>77 - other	as per variable codes	if = 77 then specify Other	
OTHERMMF	Specify other material of the floor	String				

Variable Name	Question	Variable Type	Variable Codes	Accepted Values	Skip	Comments
MMROOF	MAIN MATERIAL OF THE ROOF.<br><br>RECORD OBSERVATION.<br><br>MARK ONLY ONE.	Numeric	11-thatched<br>12-mud<br>21-wood/planks<br>22-iron sheets<br>23-asbestos<br>24-tiles<br>25-tin<br>26-cement<br>77-other<br>99 - Refused to answer	as per variable codes	if = 77 then specify Other	
OTHERMMR	Specify other material of the roof	String				
MMEWALLS	MAIN MATERIAL OF THE EXTERIOR WALLS.<br><br>RECORD OBSERVATION.<br><br>MARK ONLY ONE.	Numeric	11 - thatched/straw<br>21 - mud and poles<br>22 - un-burnt bricks<br>23 - un-burnt bricks with plaster<br>24 - burnt bricks with mud<br>31 - cement blocks<br>32 - stone<br>33 - timber<br>34 - burnt bricks with cement<br>77 - other<br>99 - Refused to answer	as per variable codes	if = 77 then specify Other	
OTHERMME	Specify other material of the exterior walls	String				
HHROOMS	How many rooms in your household are used for sleeping?<br><br>(INCLUDING ROOMS OUTSIDE THE MAIN DWELLING)<br><br>If there are 15 or more rooms, enter 15	Numeric	88 - Don't know<br>99 - Refused to answer	1 - 15,98,99		
WATCH	Does any member of your household own or have…<br><br>…A watch?	Numeric	1 - Yes<br>2 - No<br>88 - Don't know<br>99 - Refused to answer	as per variable codes		
BICYCLE	….A bicycle?	Numeric	1 - Yes<br>2 - No<br>88 - Don't know<br>99 - Refused to answer	as per variable codes		
SCOOTER	…A motorcycle or motor scooter?	Numeric	1 - Yes<br>2 - No<br>88 - Don't know<br>99 - Refused to answer	as per variable codes		
CART	…An animal-drawn cart?	Numeric	1 - Yes<br>2 - No<br>88 - Don't know<br>99 - Refused to answer	as per variable codes		

Variable Name	Question	Variable Type	Variable Codes	Accepted Values	Skip	Comments
CAR	...A car or truck?	Numeric	1 - Yes<br>2 - No<br>88 - Don't know<br>99 - Refused to answer	as per variable codes		
BANKACCO	...A bank account?	Numeric	1 - Yes<br>2 - No<br>88 - Don't know<br>99 - Refused to answer	as per variable codes		
CATTLE	How many **Cattle** does this household own?<br><br>If none, enter 00<br>If more than 95, enter 95	Numeric	888 - Don't know<br>999 - Refused to answer	0 - 95, 888,999		
GOATS	How many **Goats** does this household own?<br><br>If none, enter 00<br>If more than 95, enter 95<br>If unknown, enter 98	Numeric	888- Don't know<br>999 - Refused to answer	0 - 95, 888,999		
SHEEP	How many **Sheep** does this household own?<br><br>If none, enter 00<br>If more than 95, enter 95<br>If unknown, enter 98	Numeric	88 - Don't know<br>99 - Refused to answer	0- 95, 888,999		
PIGS	How many **Pigs** does this household own?<br><br>If none, enter 00<br>If more than 95, enter 95<br>If unknown, enter 98	Numeric	888- Don't know<br>999 - Refused to answer	0 - 95, 888,999		
CHICKEN	How many **chickens** does this household own?<br><br>If none, enter 00<br>If more than 95, enter 95<br>If unknown, enter 98	Numeric	888- Don't know<br>999 - Refused to answer	0 - 95, 888,999		
HHPSF	How often in the last year did you have problems in satisfying the food needs of the household?	Numeric	1 - never<br>2 - seldom<br>3 - sometimes<br>4 - often<br>5 - always<br>88 - Don't know<br>99 - Refused to answer	as per variable codes		
DHFCTY	How far is it to the nearest health facility?<br><br>If less than 1 km, enter 00<br>If more than 95 km, enter 95<br>If Don't know, enter 98	Numeric	888- Don't know<br>999 - Refused to answer	0 - 95, 888,999		

Variable Name	Question	Variable Type	Variable Codes	Accepted Values	Skip	Comments
MTHFCTY	If you were to go this facility,how would you most likely go there?	Numeric	1 - car/motorcycle<br>2 - public transport(bus,taxi)<br>3 - animal/animal cart<br>4 - walking<br>5 - bicycle<br>77 - other<br>88 - Don't know<br>99 - Refused to answer	as per variable codes	if = 77 then specify Other	
OTHERMTH	Specify other means of transport to the health facility	String				
**Section 4: Bednets -** *All questions in this section will be repeated for each mosquito net in the household (hhnumnet)*						
HHAMNETS	Does this household have any mosquito nets ?	Numeric	1 - Yes<br>2 - No<br>88 - Don't know<br>99 - Refused to answer	as per variable codes	if NOT 1, skip to SECTION 5	
HNUMNETS	How many mosquito nets does your household have?<br><br>IF MORE THAN 12, ENTER 13	Numeric	88 - Don't know<br>99 - Refused to answer	1 - 13, 88, 99		List all nets
OBS1	NET #1: May I have a look at the net to establish the status? OBSERVE THE NET	Numeric	1 - Observed and intact<br>2 - Observed and has visible holes<br>3 - Not a net<br>4 - Not observed	As per variable codes		
BRAND1	OBSERVE OR ASK THE BRAND OR TYPE OF MOSQUITO NET.	Numeric	1 - Long lasting net (Permanet, Duranet, Interceptor, Netprotect, Olyset)<br>2 - Factory net **with** insecticide kit (KO- net, Kooper net, Iconet, Safi net)<br>3 - Factory net **with no** insecticide (B52, bamboo hut, century, lucky net, victoria)<br>4 - homemade net<br>77 - other<br>88 - Don't know brand<br>99 - Refused to answer	as per variable codes	if = 1 then skip to SLPNET1<br><br>if = 2, 3,4,77, 88 then GO to next question	
OTHERB1	NET #1: Specify other brands or types of mosquito net	string				
MNTHS1	NET #1: How many months ago did your household obtain the mosquito net?<br><br>IF LESS THAN ONE MONTH, WRITE '0'.<br>IF MORE THAN 36 MONTHS AGO RECORD 95<br>IF NOT SURE RECORD 888	Numeric	888 - Don't know<br>95 - More than 36 months<br>998 - Refused to answer	0-36,95, 888, 999		

Variable Name	Question	Variable Type	Variable Codes	Accepted Values	Skip	Comments
WHERE1	NET #1: Where did you get the mosquito net from?		1 - Gov't hospital<br>2 - Gov't health center<br>3 - Private hospital/clinic<br>4 - Private pharmacy<br>5 - Shop<br>6 - Open market<br>7 - Hawker<br>8 - Project/ngo<br>9 - Campaign/VHT<br>10 - Church<br>11 - Gov't distributed<br>77 - Other<br>88 - Don't know<br>99 - Refused to answer	as per variable codes	if = 77 then specify other	
WHER1OT	NET #1: Specify other source of mosquito net	string				
SMNET1	NET #1: Since you got the mosquito net, was it ever soaked or dipped in a liquid to repel mosquitoes or bugs?	Numeric	1 - Yes<br>2 - No<br>77 - Other<br>88 - Don't know	as per variable codes	if <> 1 then skip to SLPNET1	
TSMNET1	NET #1: How many months ago was the net last soaked or dipped?<br><br>IF LESS THAN 1 MONTH, RECORD '0'.<br>IF 25 OR MORE MONTHS AGO, RECORD 95<br>IF NOT SURE, RECORD 98	Numeric	1 - < 6 months<br>2 - 6 to 25 months<br>3 - Never<br>95 - More than 25 months ago<br>888 - Don't know<br>999 - Refused to answer	as per variable codes		
SLPNET1	NET #1: Did anyone sleep under this mosquito net last night?	Numeric	1 - Yes<br>2 - No<br>88 - Don't know<br>99 - Refused to answer	as per variable codes	if = 1 then skip to NEXT net	
NUSED1	NET #1: What are some of the reasons why this net was not used?<br><br>PROBE FOR THE MAIN REASON.<br><br>SELECT ONLY ONE.	Numeric	1 - Too hot<br>2 - Don't like smell<br>3 - No mosquitoes<br>4 - Net too old/too many holes<br>5 - Net not hang<br>6 - Don't want to hang<br>7- Net no longer kills insects<br>77 - Other<br>88 - Don't Know<br>99 - Refused to answer	as per variable codes		
OTHERN1	NET #1: Specify other reason	String				

Variable Name	Question	Variable Type	Variable Codes	Accepted Values	Skip	Comments
NTHUNG1	NET #1: If not hung, why not?<br><br>PROBE FOR THE MAIN REASON.<br><br>SELECT ONLY ONE.	Numeric	1 - Nowhere to hang<br>2 - Don't know how to hang net<br>3 - no tools to hang net<br>4 - shape did not fit<br>5 - size did not fit<br>6 - Don't want to hang<br>77 - other<br>88 - Don't know<br>99 - Refused to answer/skipped	as per variable codes	if <> 77 then repeat Section if there are still more nets in the house (based on HNUMNETS)	
OTHERNT1	NET # 1: Specify other reason why the net was not hung.	String			after this question, repeat Section 4 if there are still more nets in the house (based on	
**Section 5: Experience with managing an ill child in the last 2 weeks**						
FEVERCH	Has **any** of the children under your care been ill with a fever at any time in the past 2 weeks?	Numeric	1 - Yes<br>2 - No<br>88 - Don't know<br>99 - Refused to answer	as per variable codes	If NOT equal to 1, skip to END	
TMTCD	Did you seek advice or treatment for the illness from any source?	Numeric	1 - Yes<br>2 - No<br>88 - Don't know<br>99 - Refused to answer	as per variable codes	If NOT equal to 2, skip to FSEEK1	
NSTRT	Why have you not sought advice or treatment from any source? (Tick all that apply)					
NSTRTA	..... Child just fell ill	Numeric	1- Ticked<br>2 - Not ticked	1 , 2		
NSTRTB	..... Child not very ill	Numeric	1- Ticked<br>2 - Not ticked	1 , 2		
NSTRTC	..... Clinic too far	Numeric	1- Ticked<br>2 - Not ticked	1 , 2		
NSTRTD	..... Have no money	Numeric	1- Ticked<br>2 - Not ticked	1 , 2		
NSTRTE	..... Waiting for child's father	Numeric	1- Ticked<br>2 - Not ticked	1 , 2		
NSTRTF	..... Don't know what to do	Numeric	1- Ticked<br>2 - Not ticked	1 , 2		
NSTRTG	..... Already had medicine at home	Numeric	1- Ticked<br>2 - Not ticked	1 , 2		
NSTRTH	..... Other	Numeric	1- Ticked<br>2 - Not ticked	1 , 2		

Variable Name	Question	Variable Type	Variable Codes	Accepted Values	Skip	Comments
OCHOT	Specify other	string				
FSEEK1	ACTION #1: What did you do **FIRST**(Including tepid sponging and herbs)?	Numeric	1 - Nothing<br>2 - Tepid sponging<br>3 - Gave Herbs Kept at home<br>4 - Gave Medicines Kept at home<br>5 - Bought medicines from a Duka/shop<br>6 - Bought mediciness from drug shop/Pharmacy<br>7 - Took to Traditional healer<br>8 - Took to a public health centre,clinic or hospital<br>9 - Bought drugs from clinic<br>77 - Other<br>88 - Don't Know<br>99 - Refused to answer	as per variable codes	if equal to 77, specify other	
FSEEKO1	ACTION #1: Specify other action	string				
ACTN1	Action # 1: How long had the child been ill when this FIRST action was taken?	Numeric	1 - < 24 hrs<br>2 - 1-3 days<br>3 - 4-7 days<br>4- > 7 days<br>88 - Don't know<br>99 - Refused to answer			
SEEK1A	ACTION #1: If you took your child to a Public Health Centre,Clinic or Hospital,Where did you go?	Numeric	1 - Public health Centre<br>2 - Tororo District Hospital<br>3- Private Hospital/Clinic<br>77 - Other<br>88 -Don't Know<br>99-Refused to answer	as per variable codes	if = 1 go to NAMEPHC1<br>if = 1 or 2 go to SEEKPUBL<br>if =77, specify other	
SEEKO1	ACTION #1: Specify other public/private health center or NGO	string				

Variable Name	Question	Variable Type	Variable Codes	Accepted Values	Skip	Comments
NAMEPHC1	ACTION #1: If you went to public health center, What is the name of the public health center you went to?	Numeric	1 - Nawire HC II<br>2 -Panyangasi HC III<br>3 - Osia HC II<br>4 - Mbula HC II<br>5 - Chawolo-Mulanda HC II<br>6 - Kisoko HC III<br>7 - Katajula HC II<br>8 - Chawolo-Kirewa HC II<br>9 - Morkiswa HC II<br>10 - Petta HC III<br>11 - Paya HC III<br>12 - Pusere HC II<br>1 3- Gwaragwara HC II<br>14 - Mudodo HC II<br>15 - Mwelo HC II<br>16 - Kirewa HC III<br>17 - Maundo HC II<br>18 - Were HC II<br>19 - Lwala HC II<br>20 - Makawari HC II	as per variable codes		
SEEKPUBL	ACTION #1: Why did you go to the Public health centre or Public hospital ?	Numeric	1 - I heard there were drugs<br>2 - Someone told me to go<br>3 - It's closest to my home<br>4 - It's free(No cost)<br>5 - I always go to the Public health Centre<br>6 - They were nice to me the last time i went<br>7 - They told me to come back<br>8 - The drug given last time was unsuccessful<br>9 - There were no drugs at the health centre the last time i went<br>77 - Other<br>88 - Don't Know<br>99 - Refused to answer/skipped	as per variable codes	if equal to 77, specify other	
SEEKPBO1	Specify other reason for going to the public health center or hospital	String				
SERPUB1	ACTION #1: How satisfied were you with the health care that your child received at the Public health centre?	Numeric	1 - Very dissatisfied<br>2 - Dissatisfied<br>3 - Uncertain<br>4 - Satisfied<br>5 - Very satisfied<br>99 - Refused to answer	as per variable codes	if = 1 or 2 go to SEVPUB1 otherwise skip to OUTPUBL1	
SEVPUB1	ACTION #1: If dissatisfied or very dissatisfied, what were the reasons for being dissatisfied? LIST ALL THAT APPLY					

Variable Name	Question	Variable Type	Variable Codes	Accepted Values	Skip	Comments
SEVPUB1A	.... Long waiting time	Numeric	1 - Ticked<br>2 - Not ticked	1,2		
SEVPUB1B	.... No trained professionals	Numeric	1- Ticked<br>2 - Not ticked	1,2		
SEVPUB1C	.... No drugs were given	Numeric	1 - Ticked<br>2 - Not ticked	1,2		
SEVPUB1D	.... No lab tests done	Numeric	1 - Ticked<br>2 - Not ticked	1,2		
SEVPUB1E	.... Rude HCW	Numeric	1- Ticked<br>2 - Not ticked	1,2		
SEVPUB1F	.... Had to pay for care	Numeric	1 - Ticked<br>2 - Not ticked	1,2		
SEVPUB1G	.... Treatment was unsuccessful	Numeric	1 - Ticked<br>2 - Not ticked	1,2		
SEVPUB1H	.... Refused to answer	Numeric	1 - Ticked<br>2 - Not ticked	1,2		
OUTPUBL1	ACTION #1: What was the treatment outcome when you took your child to the public health facility for healthcare?	Numeric	1 - Recovered quickly<br>2 - Recovered slowly<br>3 - Did not recover, so had to take child back<br>4 - Did not recover so I sought care elsewhere<br>77 - Other<br>99 - Refused to answer	as per variable codes	if 77, specify other outcome<br>If action did not involve medication, skip to TESTMAL1	
OUTOTH1	ACTION #1: Specify other treatment outcome	string				
MEDGIV1	ACTION #1: If your child took medicine **FIRST**,what did he/she take?<br><br>RECORD ALL MENTIONED.					
MEDGIV1A	..... Panadol	Numeric	1 - Ticked<br>2 - Not ticked	1,2	if =1 then must answer DURTN1x, NUMTMESx, and [illegible]	
NUMTMS1A	ACTION #1: How many times was panadol given per day?	Numeric	88 - Don't know<br>99 - Refused to answer	1 - 7, 88, 99		
NUMDS1A	ACTION #1: For how many days was panadol given?	Numeric	88- Don't know<br>99 - Refused to answer	1 - 7, 88, 99		
DURTN1A	ACTION #1: How long had the child been ill when panadol was taken?	Numeric	1 - <24 hrs<br>2 - 1 - 3 days<br>3 - 4 - 7 days<br>4 - >7 days<br>88 - Don't know<br>99 - Refused to answer	1,2,3,4,88,99		
MEDGIV1B	..... Aspirin	Numeric	1 - Ticked<br>2 - Not ticked	1,2	if =1 then must answer DURTN1x, NUMTMESx, and [illegible]	

Variable Name	Question	Variable Type	Variable Codes	Accepted Values	Skip	Comments
NUMTMS1B	ACTION #1: How many times was Aspirin given per day?	Numeric	88 - Don't know<br>99 - Refused to answer	1 - 7, 88, 99		
NUMDS1B	ACTION #1: For how many days was Aspirin given?	Numeric	88- Don't know<br>99 - Refused to answer	1 - 7, 88, 99		
DURTN1B	ACTION #1: How long had the child been ill when Aspirin was taken?	Numeric	1 - <24 hrs<br>2 - 1 - 3 days<br>3 - 4 - 7 days<br>4 - >7 days<br>88 - Don't know<br>99 - Refused to answer	1,2,3,4,88,99		
MEDGIV1C	..... Chloroquine(CQ)	Numeric	1 - Ticked<br>2 - Not ticked	1,2	if =1 then must answer DURTN1x, NUMTMESx, and [illegible]	
NUMTMS1C	ACTION #1: How many times was Chloroquine(CQ) given per day?	Numeric	88 - Don't know<br>99 - Refused to answer	1 - 7, 88, 99		
NUMDS1C	ACTION #1: For how many days was Chloroquine(CQ) given?	Numeric	88- Don't know<br>99 - Refused to answer	1 - 7, 88, 99		
DURTN1C	ACTION #1: How long had the child been ill when Chloroquine(CQ) was taken?	Numeric	1 - <24 hrs<br>2 - 1 - 3 days<br>3 - 4 - 7 days<br>4 - >7 days<br>88 - Don't know<br>99 - Refused to answer	1,2,3,4,88,99		
MEDGIV1D	..... Fansidar(SP)	Numeric	1 - Ticked<br>2 - Not ticked	1,2	if =1 then must answer DURTN1x, NUMTMESx, and [illegible]	
NUMTMS1D	ACTION #1: How many times was Fansidar(SP) given per day?	Numeric	88 - Don't know<br>99 - Refused to answer	1 - 7, 88, 99		
NUMDS1D	ACTION #1: For how many days was Fansidar(SP) given?	Numeric	88- Don't know<br>99 - Refused to answer	1 - 7, 88, 99		
DURTN1D	ACTION #1: How long had the child been ill when Fansidar(SP) was taken?	Numeric	1 - <24 hrs<br>2 - 1 - 3 days<br>3 - 4 - 7 days<br>4 - >7 days<br>88 - Don't know<br>99 - Refused to answer	1,2,3,4,88,99		
MEDGIV1E	..... Chloroquine + Fansidar(CQ+SP)	Numeric	1 - Ticked<br>2 - Not ticked	1,2	if =1 then must answer DURTN1x, NUMTMESx, and [illegible]	
NUMTMS1E	ACTION #1: How many times was CQ+SP given per day?	Numeric	88 - Don't know<br>99 - Refused to answer	1 - 7, 88, 99		
NUMDS1E	ACTION #1: For how many days was CQ+SP given?	Numeric	88- Don't know<br>99 - Refused to answer	1 - 7, 88, 99		

Variable Name	Question	Variable Type	Variable Codes	Accepted Values	Skip	Comments
DURTN1E	ACTION #1: How long had the child been ill when CQ+SP was taken?	Numeric	1 - <24 hrs<br>2 - 1 - 3 days<br>3 - 4 - 7 days<br>4 - >7 days<br>88 - Don't know<br>99 - Refused to answer	1,2,3,4,88,99		
MEDGIV1F	..... Amodiaquine	Numeric	1 - Ticked<br>2 - Not ticked	1,2	if =1 then must answer DURTN1x, NUMTMESx, and [illegible]	
NUMTMS1F	ACTION #1: How many times was Amodiaquine given per day?	Numeric	88 - Don't know<br>99 - Refused to answer	1 - 7, 88, 99		
NUMDS1F	ACTION #1: For how many days was Amodiaquine given?	Numeric	88- Don't know<br>99 - Refused to answer	1 - 7, 88, 99		
DURTN1F	ACTION #1: How long had the child been ill when Amodiaquine was taken?	Numeric	1 - <24 hrs<br>2 - 1 - 3 days<br>3 - 4 - 7 days<br>4 - >7 days<br>88 - Don't know<br>99 - Refused to answer	1,2,3,4,88,99		
MEDGIV1G	..... Quinine	Numeric	1 - Ticked<br>2 - Not ticked	1,2	if =1 then must answer DURTN1x, NUMTMESx, and [illegible]	
NUMTMS1G	ACTION #1: How many times was Quinine given per day?	Numeric	88 - Don't know<br>99 - Refused to answer	1 - 7, 88, 99		
NUMDS1G	ACTION #1: For how many days was Quinine given?	Numeric	88- Don't know<br>99 - Refused to answer	1 - 7, 88, 99		
DURTN1G	ACTION #1: How long had the child been ill when Quinine was taken?	Numeric	1 - <24 hrs<br>2 - 1 - 3 days<br>3 - 4 - 7 days<br>4 - >7 days<br>88 - Don't know<br>99 - Refused to answer	1,2,3,4,88,99		
MEDGIV1H	..... Artemether-Lumefantrine (Coartem or Lumartem)	Numeric	1 - Ticked<br>2 - Not ticked	1,2	if =1 then must answer DURTN1x, NUMTMESx, and [illegible]	
NUMTMS1H	ACTION #1: How many times was Coartem given per day?	Numeric	88 - Don't know<br>99 - Refused to answer	1 - 7, 88, 99		
NUMDS1H	ACTION #1: For how many days was Coartem given?	Numeric	88- Don't know<br>99 - Refused to answer	1 - 7, 88, 99		
DURTN1H	ACTION #1: How long had the child been ill when Coartem was taken?	Numeric	1 - <24 hrs<br>2 - 1 - 3 days<br>3 - 4 - 7 days<br>4 - >7 days<br>88 - Don't know<br>99 - Refused to answer	1,2,3,4,88,99		

Variable Name	Question	Variable Type	Variable Codes	Accepted Values	Skip	Comments
MEDGIV1I	..... Cotrimoxazole	Numeric	1 - Ticked<br>2 - Not ticked	1,2	if =1 then must answer DURTN1x, NUMTMESx, and [illegible]	
NUMTMS1I	ACTION #1: How many times was Cotrimoxazole given per day?	Numeric	88 - Don't know<br>99 - Refused to answer	1 - 7, 88, 99		
NUMDS1I	ACTION #1: For how many days was Cotrimoxazole given?	Numeric	88- Don't know<br>99 - Refused to answer	1 - 7, 88, 99		
DURTN1I	ACTION #1: How long had the child been ill when Cotrimoxazole was taken?	Numeric	1 - <24 hrs<br>2 - 1 - 3 days<br>3 - 4 - 7 days<br>4 - >7 days<br>88 - Don't know<br>99 - Refused to answer	1,2,3,4,88,99		
MEDGIV1J	..... Amoxacillin	Numeric	1 - Ticked<br>2 - Not ticked	1,2	if =1 then must answer DURTN1x, NUMTMESx, and [illegible]	
NUMTMS1J	ACTION #1: How many times was Cotrimoxazole given per day?	Numeric	88 - Don't know<br>99 - Refused to answer	1 - 7, 88, 99		
NUMDS1J	ACTION #1: For how many days was Cotrimoxazole given?	Numeric	88- Don't know<br>99 - Refused to answer	1 - 7, 88, 99		
DURTN1J	ACTION #1: How long had the child been ill when Cotrimoxazole was taken?	Numeric	1 - <24 hrs<br>2 - 1 - 3 days<br>3 - 4 - 7 days<br>4 - >7 days<br>88 - Don't know<br>99 - Refused to answer	1,2,3,4,88,99		
MEDGIV1K	..... Other1	Numeric	1 - Ticked<br>2 - Not ticked	1,2	if =1 then must answer DURTN1x, NUMTMESx, and [illegible]	
NUMTMS1K	ACTION #1: How many times was the other 1 drug given per day?	Numeric	88 - Don't know<br>99 - Refused to answer	1 - 7, 88, 99		
NUMDS1K	ACTION #1: For how many days was the other1 drug given?	Numeric	88- Don't know<br>99 - Refused to answer	1 - 7, 88, 99		
DURTN1K	ACTION #1: How long had the child been ill when the other1 drug was taken?	Numeric	1 - <24 hrs<br>2 - 1 - 3 days<br>3 - 4 - 7 days<br>4 - >7 days<br>88 - Don't know<br>99 - Refused to answer	1,2,3,4,88,99		
MEDGIV1L	..... Other2	Numeric	1 - Ticked<br>2 - Not ticked	1,2	if =1 then must answer DURTN1x, NUMTMESx, and [illegible]	
NUMTMS1L	ACTION #1: How many times was the other 2 drug given per day?	Numeric	88 - Don't know<br>99 - Refused to answer	1 - 7, 88, 99		

Variable Name	Question	Variable Type	Variable Codes	Accepted Values	Skip	Comments
NUMDS1L	ACTION #1: For how many days was the other 2 drug given?	Numeric	88- Don't know<br>99 - Refused to answer	1 - 7, 88, 99		
DURTN1L	ACTION #1: How long had the child been ill when the other 2 drug was taken?	Numeric	1 - <24 hrs<br>2 - 1 - 3 days<br>3 - 4 - 7 days<br>4 - >7 days<br>88 - Don't know<br>99 - Refused to answer	1,2,3,4,88,99		
MEDGIV1M	..... Other3	Numeric	1 - Ticked<br>2 - Not ticked	1,2	if =1 then must answer DURTN1x, NUMTMESx, and [illegible]	
NUMTMS1M	ACTION #1: How many times was the other 3 drug given per day?	Numeric	88 - Don't know<br>99 - Refused to answer	1 - 7, 88, 99		
NUMDS1M	ACTION #1: For how many days was the other 3 drug given?	Numeric	88- Don't know<br>99 - Refused to answer	1 - 7, 88, 99		
DURTN1M	ACTION #1: How long had the child been ill when the other 3 drug was taken?	Numeric	1 - <24 hrs<br>2 - 1 - 3 days<br>3 - 4 - 7 days<br>4 - >7 days<br>88 - Don't know<br>99 - Refused to answer	1,2,3,4,88,99		
MEDGIV1N	..... Unknown	Numeric	1 - Ticked<br>2 - Not ticked	1,2	if =1 then must answer DURTN1x, NUMTMESx, and [illegible]	
NUMTMS1N	ACTION #1: How many times was the unknown drug given per day?	Numeric	88 - Don't know<br>99 - Refused to answer	1 - 7, 88, 99		
NUMDS1N	ACTION #1: For how many days was the unknown drug given?	Numeric	88- Don't know<br>99 - Refused to answer	1 - 7, 88, 99		
DURTN1N	ACTION #1: How long had the child been ill when the unknown drug was taken?	Numeric	1 - <24 hrs<br>2 - 1 - 3 days<br>3 - 4 - 7 days<br>4 - >7 days<br>88 - Don't know<br>99 - Refused to answer	1,2,3,4,88,99		
MEDGIV1O	..... Not Applicable	Numeric	1 - Ticked<br>2 - Not ticked	1,2	if =1 then must answer DURTN1x, NUMTMESx, and [illegible]	
TESTMAL1	ACTION #1: Was your child tested for malaria (finger or heel prick) when you went to this place?	Numeric	1 - Yes<br>2 - No<br>77 - Other<br>88 - Don't know<br>99 - Refused to answer	1,2,77,88,99	If NOT 1, skip to ILLRES1	

Variable Name	Question	Variable Type	Variable Codes	Accepted Values	Skip	Comments
TESTTYP1	ACTION #1: If yes, do you know what type of test was done? For example, was the blood placed onto a glass slide or onto a white stick?	Numeric	1 - Blood smear<br>2 - RDT<br>77 - Other<br>88 - Don't know	1,2,77,88	if 77, specify other test done	
TESTTYO1	ACTION #1: Specify other test done	string				
TOLDRES1	ACTION #1: Where you told the result of the test?	Numeric	1 - Yes<br>2 - No<br>88 - Don't know	1,2,88	If NOT 1, skip to ILLRES1	
TESTRES1	ACTION #1: If yes, what was the result of the test?	Numeric	1 - Positive for malaria<br>2 - Negative for malaria<br>77 - Other<br>88 - Don't know<br>99 - Refused to answer	1,2,77,88,99		
TESTREO1	ACTION #1: If other result, specify	string				
ILLRES1	ACTION #1: Did the child's illness resolve after this FIRST action?	Numeric	1 - Yes<br>2 - No<br>88 - Don't know	1,2,88		
ANYACTO1	Have you done any other action until now?	Numeric	1 - Yes<br>2 - No	1,2	If Yes, Continue to SECOND action, else skip to SCETION 6	
....	....	....	....	....	....	Variables for ACTION #2,3,.....,8; have index 2,3,...,8
BDTST	At any time during the illness, did the child have blood taken from his/her finger or heel for testing?	Numeric	1 - Yes<br>2 - No<br>88 - Don't know<br>99 - Refused to answer	1,2,88,99		
DURILL	How long did the child's illness last?	Numeric	1 - < 24 hrs<br>2 - 1-3 days<br>3- 4-7 days<br>4- > 7 days<br>5- Ongoing	1,2,3,4,5		
ILLDLYS	Did you experience any delays in treating your child's illness?	Numeric	1 - Yes<br>2 - No<br>88 - Don't know<br>99 - Refused to answer	1,2,88,99	If NOT 1, skip to SPEND	
RESDYS	What were the reasons for the delay?<br>**LIST ALL THAT APPLY**					
RESDYSA	... No transport available	Numeric	1 - Ticked<br>2 - Not ticked	1,2		
RESDYSB	... Not enough money available	Numeric	1 - Ticked<br>2 - Not ticked	1,2		

Variable Name	Question	Variable Type	Variable Codes	Accepted Values	Skip	Comments
RESDYSC	... Needed to find coverage for work	Numeric	1 - Ticked<br>2 - Not ticked	1,2		
RESDYSD	... Needed to arrange for child care	Numeric	1 - Ticked<br>2 - Not ticked	1,2		
RESDYSE	... Waiting at the health facility	Numeric	1 - Ticked<br>2 - Not ticked	1,2		
RESDYSF	... Was not at home	Numeric	1 - Ticked<br>2 - Not ticked	1,2		
RESDYSG	... Other	Numeric	1 - Ticked<br>2 - Not ticked	1,2	If ticked, specify other reason for delay	
RESDYSH	... Don't know	Numeric	1 - Ticked<br>2 - Not ticked	1,2		
RESDYSI	... Refused to answer	Numeric	1 - Ticked<br>2 - Not ticked	1,2		
RESDLYSO	specify other reason for the delay	string				
SPEND	Did you spend any money on management of this illness?	Numeric	1 - Yes<br>2 - No	1,2	If equal to 2, skip to RESDELY	
HWDRUGS	How much did you spend on Drugs? IF COST IS GREATER THAN 200000, ENTER 200000	Numeric		0 - 200000		
HWTRANS	How much did you spend on Transport?<br>IF COST IS GREATER THAN 200000, ENTER 200000	Numeric		0 - 200000		
HWFEES	How much did you spend on clinical,hospital and laboratory bills?<br>IF COST IS GREATER THAN 200000, ENTER 200000	Numeric		0 - 200000		
SPENDO	Did you spend on anything else in regards to treatment of the illness?	Numeric	1 - Yes<br>2 - No	1,2	If equal to 2, skip to RESDELY	
HWOTHER	How much was spent on other things?	Numeric		50 - 200000		
RESDELY	Did caring for your child and managing his/her illness prevent you from doing your usual activities ?	Numeric	1 - Yes<br>2 - No<br>88 - Don't know<br>99 - Refused to answer	1,2,88,99	If equal to 2, skip to END	
HCHMISS	If yes, how much time did you miss?	Numeric		1 - 100		
UNITS	Units of time missed (HRS, DAYS,....)	Numeric	1 - Hours<br>2 - Days<br>88 - Weeks	1,2,88		

Variable Name	Question	Variable Type	Variable Codes	Accepted Values	Skip	Comments
STOPTIME	STOPTIME = Current time	Date/Time				
VSTATUS	Result of visit?	Numeric	1 - Completed<br>2 - No household member at home or no competent respondent at home at time of visit<br>3 - Entire household absent for extended period of time<br>77 - Other<br>99 - Refused to be interviewed	1,2,3,77,99	If 77, specify other results of the visit	
OVSTATUS	Specify other result	string				
TOTVISIT	Total number of visits	Numeric		1 - 4		

APPENDIX P: CROSS-SECTIONAL SURVEY CLINICAL RECORD FORM (1)

Subcounty ID	Village ID	Compound Number	Household Number	Date of visit
[___]	[___|___]	[___|___|___|___]	[___|___]	[___|___]/ [___|___]/[___|___] day month year

Cluster number	Study ID	Gender	Age
[_____|_____]	[_____|_____|_____|_____]	[_____] 1 = Male 2 = Female	Age: [____|____]/[____|____] years months

SECTION 1: BEDNETS

1. "Does this child have (sleep under) a mosquito net?	1 = Yes<br>2 = No	88 = Don't know<br>99 = Refused to answer<br>*If no, go to Section 2: Case Record Form*	[____|____]
2. "If yes, did the child sleep under the mosquito net last night?"	1 = Yes<br>2 = No	88 = Don't know<br>99 = Refused to answer	[____|____]
3. "How many months ago did you obtain the mosquito net for the child?"	77 = Other ________________________<br>88 = Don't know<br>99 = Refused to answer	Insert the number of months	[____|____]
4. "From where did you get the mosquito net?	1 = Government health center<br>2 = Government hospital<br>3 = Private hospital/clinic<br>4 = Private pharmacy<br>5 = Shop<br>6 = Open market<br>7 = Hawker<br>8 = Project/NGO<br>9 = Campaign	10 = Church<br>77 = Other___________<br>88 = Don't know<br>99 = Refused to answer	[____|____]
5. "May I have a look at the mosquito net?"<br>*(Observe the net and record the status)*	1 = Observed and intact<br>2 = Observed and has visible	3 = Not a net<br>4 = Not observed<br>*If observed go to #6, if not observed skip to #7*	[____|____]
6. "What is the brand of the mosquito net?"<br>*(If net was observed, record brand; if not, ask the respondent)*<br>*If '1', go to Section 2: Case Record Form*	1 = Long lasting net (Permanet, Smartnet, Olyset)<br>2 = Factory net with insecticide kit (KO net, Kooper net, Ico net, Safi net)<br>3 = Factory net with no insecticide (B52, Bamboo, Century, Lucky net, Victoria)<br>4 = Home made net<br>77 = Other____		[____|____]
7. How many months ago was the mosquito net last soaked or dipped with insecticide to repel mosquitos?	1 = < 6months<br>2 = ≥ 6 months<br>3 = Never	88 = Don't know<br>99 = Refused to answer	[____|____]

SECTION 2: CASE RECORD FORM

10. Past medical history (list) ______________________ ____

____

____

11. "Does the child have any drug allergies?"	1 = Yes<br>2 = No	88 = Don't know<br>99 = Refused to answer	[____|____]

12. If yes, to which drugs? (list) ______________________ ____

13. Current medications

1 = Panadol<br>2 = Aspirin<br>3 = Chloroquine<br>4 = Fansidar (SP)<br>5 = CQ+SP<br>6 = Amodiaquine<br>7 = Quinine	8 = Coartem<br>9 = Septrin<br>10 = Amoxacillin<br>11 = None<br>12 = Other (list)<br>________________	Drug 1<br>Drug 2<br>Drug 3<br>Drug 4<br>Drug 5	[____|____]<br>[____|____]<br>[____|____]<br>[____|____]<br>[____|____]

**14. History of fever in the last 48 hours?**	1 = Yes<br>2 = No	88 = Don't know<br>99 = Refused to answer	[____]
**15. Temperature** (°C), **Severity***	[____|____]•[____]	Severity [____]	
**16. Weight** (kg)	[____|____]		
**18. MUAC** (mm) [____|____|____]	**19. Spleen size†**	[____] Hackett's score	

***Rank severity on scale of 0-5:** 0=Absent 1=Mild 2=Moderate 3=Severe 4=Life-threatening 5=Unable to assess

† Hackett's: 0=Normal, 1=palpable, 2= <½ to umbilicus, 3= >½ to umbilicus, 4= <½ to symphysis pubis, 5= >½ to symphysis pubis

CROSS-SECTIONAL SURVEY CLINICAL RECORD FORM (2)

Subcounty ID	Village ID	Compound Number	Household Number	Date of visit
[___]	[___|___]	[___|___|___|___]	[___|___]	[___|___]/ [___|___]/[___|___]<br>day month year

Cluster number	Study ID
[_____|_____]	[_____|_____|_____|_____]

SECTION 3: FEVER EPISODE

Complete if there is a history of fever or documented temperature (≥ 38.0°C)

20. Clinical Notes – History of present illness

21. Physical examination findings

22. RDT result

0 = Negative
1 = Positive (Pf only)
2 = Positive (Pan only)
3 = Positive (Pf + Pan)
4 = Invalid
77 = Other

[____|____]

23. Diagnosis

1 = Uncomplicated malaria
2 = Severe malaria
3 = Otitis media
4 = Pharyngitis
5 = Upper respiratory tract infection
6 = Pneumonia
7 = Gastroenteritis
8 = Diarrhea
9 = Dysentery
10 = Urinary tract infection
11 = Skin infection
12 = Viral illness
77 = Other
88 = Unknown

Diagnosis 1 [____|____]
Diagnosis 2 [____|____]
Diagnosis 3 [____|____]
Diagnosis 4 [____|____]
Diagnosis 5 [____|____]

24. Was the child referred for additional care?

1 = Yes
2 = No

[____]

25. If yes, where? (specify)

26. Medications prescribed

1 = Panadol
8 = Coartem (artemether-lumefantrine)
12 = Other (list)
____
____
____

Drug 1 [____|____]
Drug 2 [____|____]
Drug 3 [____|____]
Drug 4 [____|____]
Drug 5 [____|____]

SECTION 4: LABORATORY RESULTS

Complete for all participants

**27. Hemoglobin** (g/dL)**	**28. Parasite density** (/ul)	**29. Gametocytes**
Severity<br>[____|____]•[____] [____]	[____|____|____|____|____]	1 = Yes 2 = No 3 = N/A<br>[____]

Hemoglobin ** = If Hb is < 5.0 g/dL, refer to Tororo District Hospital

Staff ID: [____|____] **Data entrant (1st):** [____|____] **Data entrant (2nd):** [____|____]

APPENDIX Q: COHORT STUDY SCREENING FORM

PART 1: HOUSEHOLD & PARTICIPANT ID

Subcounty ID	Village ID	Compound number	Household number	Cluster number
[_____]	[_____|_____]	[_____|_____|_____|_____]	[_____|_____]	[_____|_____]

Screening Date	Screening ID
[_____|_____]/[_____|_____]/[_____|_____]<br>day month year	[_____|_____|_____|_____]

Age		Gender	
[_____|_____] / [_____|_____]<br>years months	***If child is less than 1 year, complete months, otherwise leave blank***	[_____]	1 = Male<br>2 = Female

PART 2: SCREENING INTERVIEW with PARENTS/GUARDIANS

**Selection criteria**	**Include**	**Exclude**	
1. Appropriate age<br>—Under five (aged 0 to less than 5 years)	1 = Yes	2 = No	[_____]
2. Intention to move from Tororo during the follow-up period	1 = No	2 = Yes	[_____]
3. Current enrollment in another research study	1 = No	2 = Yes	[_____]

If any answers are '2' from the EXCLUDE column, exclude from the study. If not, proceed to the next section.

PART 3: INFORMED CONSENT

**Selection criteria**	**Include**	**Exclude**	
4. Willingness of parent(s)/guardian(s) to provide informed consent	1 = Yes	2 = No	[_____]

If any answers are '2' from the EXCLUDE column, exclude from the study. If not, proceed to the next section.

**ASSIGN STUDY NUMBER**	**[_____|_____|_____|_____]**

**All criteria for study inclusion met?**	**Date of enrollment**
1 = Yes<br>2 = No ***If no, exclude from the study*** [_____]	[______|______]/[______|______]/[______|______]<br>day month year

Staff ID: [____|____] **Data entrant (1st):** [____|____] **Data entrant (2nd):** [____|____]

[____|____] [____]—[____|____]—[____|____|____|____]—[____|____] [____|____|____|____]

Cluster number **Household ID** **Cohort Study ID**

APPENDIX R. COHORT STUDY
Research participant informed consent form

**Protocol Title:**	ACT PRIME Study: Evaluating the impact of enhanced health facility-based care for malaria and febrile illnesses in children
**Site of Research:**	Tororo, Uganda
**Principal Investigators**:	Dr. Sarah Staedke
**Date:**	15 September 2011

Introduction

Dr. Sarah Staedke and colleagues from the Uganda Malaria Surveillance Project / Infectious Diseases Research Collaboration are investigating delivery of healthcare services in Tororo District. We are doing a research study to see if we can improve the health of children in this area by improving services at government-run health facilities.

Why is this study being done?

Certain health centers in Tororo district will be selected to either take part in the intervention to improve services, or to continue with their current services. Assignment to the two groups has been determined by a lottery. The chance of being placed into either of the groups is the same. To find out how well the intervention is working, we would like to review the health of children living near the health centers. We plan to invite a group of children under five from 500 households near the health centers to take part in a 2-year study.

How is this study being done?

Households will be selected to participate by a lottery. In each household, all children under five will be eligible to take part. If a new child is born into your household, they will be eligible to take part. A household survey will be conducted at the start of the study to learn more about your household and how you manage illnesses in children. The household survey will then be repeated approximately one year after enrollment, and we will also ask all women of child bearing age living in your household some questions about their children. We will examine children and do blood tests five times during the study. We will ask members of your household to record information about the health of your children and how much you spend on their treatment using a diary with pictures. We will visit you once a month to collect the diaries and ask some questions about the health of your children.

What will happen if my child takes part in this study?

If you agree to let your child (or children) participate in this study, the following will happen today:

a) We will collect information on your child's general health and will briefly examine your child.
b) A blood sample will be taken from your child's finger to examine for malaria parasites and to measure blood counts.
c) We will also store a sample of blood on filter paper for future research purposes that will not impact on the health care of your child.
d) If your child has had a fever in the last 48 hours (2 days) or has a high temperature, we will do a rapid diagnostic test for malaria.
e) If your child has a positive test for malaria, we will provide treatment with artemether-lumefantrine (including Coartem or Lumartem), which is the recommended treatment for simple malaria in Uganda.
f) If your child has a negative test for malaria, has a low blood count, or has signs of severe malaria or another significant illness, we will refer you and your child to an appropriate health center or hospital for further care.
g) You will be asked to record information about the health of your child and certain problems using a diary with pictures. You will also be asked to record how much money you spend on your child's health care. We will give you pre-printed forms to help you record this information. You will be given instructions on how to complete the forms and will not be required to read in order to complete the forms. We will ask that you complete the forms for the duration of the study period (2 years). A member of the study staff will visit you at home at least once a month to collect completed diary forms, to give you new forms, and to ask additional questions about the health of your child during the previous month.
h) Either today or within the next two weeks, a member of the study staff will come to your home to ask you additional questions about your home and how members of your household manage malaria and other illnesses. We will also answer any questions you might have about how to fill in the diary forms.
i) At the monthly visits to your home, we will also ask if your child has had any health problems after treatment for an illness. If your child has any serious health problems, or problems after treatment with artemether-lumefantrine (including Coartem or Lumartem), we will ask you some questions about the illness, the treatment, and the details of event.

Every six months for the next 2 years, we will schedule appointments for you and your child. At these appointments, the following will happen:

a) We will examine your child.
b) A blood sample will be taken from your child's finger to examine for malaria parasites and to measure blood counts.

c) We will also store a sample of blood on filter paper for future research purposes that will not impact on the health care of your child.
d) If your child has had a fever in the last 48 hours (2 days) or has a high temperature, we will do a rapid diagnostic test for malaria.
e) If your child has a positive test for malaria, we will provide treatment with artemether-lumefantrine (including Coartem or Lumartem), which is the recommended treatment for simple malaria in Uganda.
f) If your child has a negative test for malaria, has a low blood count, or has signs of severe malaria or another significant illness, we will refer you and your child to an appropriate health center or hospital for further care.

<u>How long will these activities last?</u>
The study will last for 2 years.

<u>How will the data be used?</u>
a) The data we collect will be used by project investigators and may be shared with other researchers and policy-makers to answer questions about how best to deliver health services.
b) Information about problems following treatment will be stored in a central register to help researchers understand more about the safety of drug treatments.

<u>Can I stop my child from being in the study?</u>
You can decide to stop participating at any time. Just tell our study personnel right away if you wish to stop the activities.

<u>What risks can I expect if my child participates in the study?</u>
We will obtain five blood samples by fingerprick from your child. The risks of drawing blood from a fingerprick include temporary discomfort from the needle stick, bruising, and skin infection. The amount of blood removed will be too small to affect your child's health.

Participation in any research study may involve a loss of privacy. Information you provide will be recorded, but your name, and your child's name, will not be used in any reports of the information provided. The information obtained from these study activities will be locked up at our project offices. We will do our best to make sure that all personal information is kept private.

<u>Are there benefits to letting my child take part in the study?</u>
Through the intervention, we aim to improve the health of children in this area by improving services at the health centers. There will be no direct benefit to you from participating in this study. However, the

information that we gather in this study will help researchers and policy-makers understand how best to improve health services in this area.

What other choices do I have if I do not allow my child to take part in the study?

You are free to choose not to participate in the study. If you decide not to take part, there will be no penalty to you.

What are the costs of taking part in the study? Will I be paid for letting my child take part in the study?

You and your child will not be charged for any of the treatments or procedures we perform today. However, if we refer your child for further evaluation and health care, you will be responsible for all costs for your child's health care. You and your child will not be paid for participation in the study.

What are my rights if I allow my child to take part in the study?

Taking part in this study is your choice. You may choose either to take part or not to take part. If you decide to take part in this study, you may change your mind at any time. If you decide to withdraw your child from the study; your child will still be eligible for care at the local health facility and at Tororo District Hospital and at other local clinics. No matter what decision you take, there will be no penalty to you in any way.

What if my child is injured as result of being in this study?

If your child is injured, or if you have questions about injuries as a result of being in the study, please contact Dr. Sarah Staedke or other members of the Uganda Malaria Surveillance Project / Infectious Disease Research Collaboration on telephone number 0414-530692. The sponsoring organizations do not have a program to cover your costs if your child is hurt or has other bad results.

Who can answer my questions about the study?

You can talk to the researchers about any questions or concerns you have about these study activities. Contact Dr. Sarah Staedke or other members of the Uganda Malaria Surveillance Project / Infectious Disease Research Collaboration on telephone number 0414-530692. If you have any questions, comments or concerns about taking part in these activities, first talk to the researchers. If for any reason you do not wish to do this, or you still have concerns about doing so, you may contact Professor James Tumwine, Makerere University School of Medicine Research and Ethical Committee at telephone number 0414-530020.

WHAT YOUR SIGNATURE OR THUMBPRINT MEANS

Your signature or thumbprint below means that you understand the information given to you in this consent form about your child's participation in the study and agree with the following statements:

1. "I have read the consent form concerning this survey (or have understood the verbal explanation of the consent form) and I understand what will be required of me and what will happen to me and my child if we take part in it."

2. "My questions concerning this survey have been answered by Dr. Staedke or the person who signed below."

3. "I understand that at any time, I may withdraw my child from this survey without giving a reason and without affecting my child's normal health care and management."

4. "I agree that the child under my care will take part in this survey."

You will also be asked to sign another informed consent form for the use of stored specimens. If you wish your child to participate in this study, you should sign or place your thumbprint below.

THE REPUBLIC OF UGANDA

WE WILL GIVE YOU A COPY OF THIS SIGNED AND DATED CONSENT FORM

Name of Participant (printed)

Name of Parent/Guardian

Signature or Fingerprint * of Parent/Guardian Date/Time

Name of Investigator Administering Consent (printed) Position/Title

Signature of Investigator Administering Consent Date/Time

*If the parent or guardian is unable to read and/or write, an impartial witness should be present during the informed consent discussion. After the written informed consent form is read and explained to the participant and parent or guardian, and after they have orally consented to their child's participation in the trial, and have either signed the consent form or provided their fingerprint, the witness should sign and personally date the consent form. By signing the consent form, the witness attests that the information in the consent form and any other written information was accurately explained to, and apparently understood by the parent or guardian, and that informed consent was freely given by the patient and parent or guardian.

Name of Person Witnessing Consent (printed)

Signature of Person Witnessing Consent Date/Time

THE REPUBLIC OF UGANDA

[____|____] [____]—[____|____]—[____|____|____|____]—[____|____] [____|____|____|____]

Cluster number **Household ID** **Cohort Study ID**

APPENDIX S. COHORT STUDY
Informed consent for future use of biological specimes

**Protocol Title:**	ACT PRIME Study: Evaluating the impact of enhanced health facility-based care for malaria and febrile illnesses in children
**Site of Research:**	Tororo, Uganda
**Principal Investigators**:	Dr. Sarah Staedke
**Date:**	15 September 2011

INTRODUCTION

While your child is in this study, there may be blood samples taken from them that may be useful for future research. These samples will be stored long-term at Makerere University Medical School and the London School of Hygiene and Tropical Medicine, and the University of California, San Francisco. Samples may also be shared with investigators at other institutions.

WHAT SAMPLES WILL BE USED FOR

Your child's blood and the malaria parasites in it will be used to study malaria and the response of this disease to treatment. Results of these studies will not affect your child's care.

1. These samples will be used for future research to learn more about malaria and other diseases.
2. Your child's samples will be used only for research and will not be sold or used for the production of commercial products.
3. Genetic research may be performed on samples. However, no genetic information obtained from this research will be placed in your child's medical records. These samples will be identified only by codes so that they cannot be readily identified with your child.

LEVEL OF IDENTIFICATION

Your child's samples will be coded so that your child's name cannot be readily identified. Reports about research done with your child's samples will not be put in their medical record and will be kept confidential to the best of our ability. In the future, researchers studying your child's samples may need to know more about your child, such as their age, gender, and race. If this information is already available because of your child's participation in a study, it may be provided to the researcher. Your child's name or anything that might identify them personally will not be provided. You will not be asked to provide additional consent.

RISKS

There are few risks to your child from future use of their samples. A potential risk might be the release of information from your child's health or study records. Reports about research done with your child's samples will not be put in their health record, but will be kept with the study records. The study records will be kept confidential as far as possible.

BENEFITS

There will be no direct benefit to your child. From studying your child's samples we may learn more about malaria or other diseases: how to prevent them, how to treat them, how to cure them.

RESEARCH RESULTS/MEDICAL RECORDS

1. Results from future research using your child's samples may be presented in publications and meetings but patient names will not be identified.
2. Reports from future research done with your child's samples will not be given to you or your child's doctor. These reports will not be put in your child's medical record.

QUESTIONS

If you have any questions, comments or concerns about the future use of your child's specimen's, first talk to the researchers. You may also Contact Dr. Sarah Staedke or other members of the Uganda Malaria Surveillance Project on telephone number 0414-530692. If for any reason you do not wish to do this, or you still have concerns about the future use of your child's specimens, you may contact Professor James Tumwine, Makerere University School of Medicine Research and Ethical Committee at telephone number 0414-530020.

FREEDOM TO REFUSE

You can change your mind at any time about allowing your child's samples to be used for future research. If you do, contact Dr. Staedke or other members of the Uganda Malaria Surveillance Project at the numbers listed above. Then your child's samples will no longer be made available for research and will be destroyed. Whether or not you allow us to use your child's samples in future research will not have any effect on your child's participation in this study or future participation in other studies.

WHAT YOUR SIGNATURE OR THUMBPRINT MEANS

Your signature or thumbprint below means that you understand the information given to you in this consent form about your child's specimens to be used for future research. If you wish to allow your child's specimens to be used for future research, you should sign or place your thumbprint below.

THE REPUBLIC OF UGANDA

WE WILL GIVE YOU A COPY OF THIS SIGNED AND DATED CONSENT FORM

Name of Participant (printed)

Name of Parent/Guardian

Signature or Fingerprint * of Parent/Guardian Date/Time

Name of Investigator Administering Consent (printed) Position/Title

Signature of Investigator Administering Consent Date/Time

*If the parent or guardian is unable to read and/or write, an impartial witness should be present during the informed consent discussion. After the written informed consent form is read and explained to the participant and parent or guardian, and after they have orally consented to their child's participation in the trial, and have either signed the consent form or provided their fingerprint, the witness should sign and personally date the consent form. By signing the consent form, the witness attests that the information in the consent form and any other written information was accurately explained to, and apparently understood by the parent or guardian, and that informed consent was freely given by the patient and parent or guardian.

Name of Person Witnessing Consent (printed)

Signature of Person Witnessing Consent Date/Time

APPENDIX T: COHORT STUDY CLINICAL RECORD FORM (1)

Visit Code*	Study ID	Gender	Date of BIRTH
[_____]	[_____|_____|_____|_____]	[_____] 1 = Male 2 = Female	[___|___]/ [___|___]/[___|___] day month year

Subcounty ID	Village ID	Compound number	Household #	Cluster #	Date of VISIT
[___]	[___|___]	[___|___|___|___]	[___|___]	[___|___]	[___|___]/ [___|___]/[___|___] day month year

***Indicate type of visit:** 1 = Enrollment 2 = 6 months 3 = 12 months 4 = 18 months 5 = 24 months

SECTION 1: BEDNETS

**1. "Does this child have (sleep under) a mosquito net?"**	1 = Yes<br>2 = No	88 = Don't know<br>99 = Refused to answer	[_____|_____]<br>*If no, go to Section 2: Case Record Form*
**2. "If yes, did the child sleep under the mosquito net last night?"**	1 = Yes<br>2 = No	88 = Don't know<br>99 = Refused to answer	[_____|_____]
**3. "How many months ago did you obtain the mosquito net for the child?"**	77 = Other ____________________<br>88 = Don't know<br>99 = Refused to answer		Insert the number of months<br>[_____|_____]
**4. "From where did you get the mosquito net?**	1 = Government health center<br>2 = Government hospital<br>3 = Private hospital/clinic<br>4 = Private pharmacy	5 = Shop<br>6 = Open market<br>7 = Hawker<br>8 = Project/NGO<br>9 = Campaign	10 = Church<br>77 = Other__________<br>88 = Don't know<br>99 = Refused to answer<br>[_____|_____]
**5. "What is the brand of the mosquito net?"**<br>*If '1', go to Section 2: Case Record Form*	1 = Long lasting net (Permanet, Smartnet, Olyset)<br>2 = Factory net with insecticide kit (KO net, Kooper net, Ico net, Safi net)<br>3 = Factory net with no insecticide (B52, Bamboo, Century, Lucky net, Victoria)<br>4 = Home made net<br>77 = Other___________________________________		[_____|_____]
**6. How many months ago was the mosquito net last soaked or dipped with insecticide to repel mosquitos?**	1 = < 6months<br>2 = ≥ 6 months<br>3 = Never	88 = Don't know<br>99 = Refused to answer	[_____|_____]

SECTION 2: CASE RECORD FORM

10. Past medical history (list) ____________________ ____

____

____

**11. "Does the child have any drug allergies?"**	1 = Yes<br>2 = No	88 = Don't know<br>99 = Refused to answer	[_____|_____]
**12. If yes, to which drugs? (list)** ____________________ ____			
**13. Current medications**<br>1 = Panadol<br>2 = Aspirin<br>3 = Chloroquine<br>4 = Fansidar (SP)<br>5 = CQ+SP<br>6 = Amodiaquine<br>7 = Quinine	8 = Coartem<br>9 = Septrin<br>10 = Amoxacillin<br>11 = None<br>12 = Other (list)<br>________________	Drug 1<br>Drug 2<br>Drug 3<br>Drug 4<br>Drug 5	[_____|_____]<br>[_____|_____]<br>[_____|_____]<br>[_____|_____]<br>[_____|_____]
**14. History of fever in the last 48 hours?**	1 = Yes<br>2 = No	88 = Don't know<br>99 = Refused to answer	[_____]
**15. Temperature** (°C), **Severity***		[____|____]•[____]	Severity<br>[_____]
**16. Weight** (kg)	[_____|_____]	**17. Height** (cm)	[_____|_____|_____]
**18. MUAC** (mm)	[_____|_____|_____]	**19. Spleen size†**	[_____] Hackett's score

***Rank severity on scale of 0-5:** 0=Absent 1=Mild 2=Moderate 3=Severe 4=Life-threatening 5=Unable to assess

† Hackett's: 0=Normal, 1=palpable, 2= <½ to umbilicus, 3= >½ to umbilicus, 4= <½ to symphysis pubis, 5= >½ to symphysis pubis

COHORT STUDY CLINICAL RECORD FORM (2)

Visit Code* [_____]			Study ID [_____|_____|_____|_____]		
**Subcounty ID**	**Village ID**	**Compound ID**	**Household ID**	**Cluster #**	**Date of VISIT**
[___]	[___|___]	[___|___|___|___]	[___|___]	[___|___]	[___|___]/ [___|___]/[___|___]<br>day month year

***Indicate type of visit:** 1 = Enrollment 2 = 6 months 3 = 12 months 4 = 18 months 5 = 24 months

SECTION 3: FEVER EPISODE

Complete if there is a history of fever or documented temperature (≥ 38.0°C)

20. Clinical Notes – History of present illness

21. Physical examination findings

22. RDT result

0 = Negative
1 = Positive (Pf only)
2 = Positive (Pan only)
3 = Positive (Pf + Pan)
4 = Invalid
77 = Other

[_____|_____]

23. Diagnosis

1 = Uncomplicated malaria
2 = Severe malaria
3 = Otitis media
4 = Pharyngitis
5 = Upper respiratory tract infection
6 = Pneumonia
7 = Gastroenteritis
8 = Diarrhea
9 = Dysentery
10 = Urinary tract infection
11 = Skin infection
12 = Viral illness
77 = Other
88 = Unknown

Diagnosis 1 [_____|_____]
Diagnosis 2 [_____|_____]
Diagnosis 3 [_____|_____]
Diagnosis 4 [_____|_____]
Diagnosis 5 [_____|_____]

24. Was the child referred for additional care?

1 = Yes
2 = No

[_____]

25. If yes, where? (specify)

26. Medications prescribed

1 = Panadol
8 = Coartem (artemether-lumefantrine)
12 = Other (list)
____
____
____

Drug 1 [_____|_____]
Drug 2 [_____|_____]
Drug 3 [_____|_____]
Drug 4 [_____|_____]
Drug 5 [_____|_____]

SECTION 4: LABORATORY RESULTS

Complete for all participants

**27. Hemoglobin** (g/dL)**	**28. Parasite density** (/ul)	**29. Gametocytes**<br>1 = Yes 2 = No 3 = N/A
[_____|_____]•[_____] Severity [_____]	[_____|_____|_____|_____|_____]	[_____]

Hemoglobin ** = If Hb is < 5.0 g/dL, refer to Tororo District Hospital

Staff ID: [_____|_____] **Data entrant (1st):** [_____|_____] **Data entrant (2nd):** [_____|_____]

APPENDIX U: COHORT STUDY HOUSEHOLD SURVEY (1)

Subcounty ID	Village ID	Compound #	Household #	Cluster #	Date of visit
[___]	[___|___]	[___|___|___|___]	[___|___]	[___|___]	[___|___]/ [___|___]/[___|___]<br>day month year

Study IDs	1. [___|___|___|___]<br>2. [___|___|___|___]	3. [___|___|___|___]<br>4. [___|___|___|___]	5. [___|___|___|___]<br>6. [___|___|___|___]

SECTION 1: PRIMARY CAREGIVER

**1. "How old are you?"** [_____|_____]years	**2. Gender**	1 = Male<br>2 = Female	[_____]
**3. "What is the highest level of school you completed?"**<br>0 = None<br>1 = Primary (P1 — P4)	2 = Primary (P5 — P7)<br>3 = Secondary (S1 — S4)<br>4 = Secondary (S5 — S6)<br>10 = Certificate/Diploma	11 = University<br>77 = Other<br>88 = Don't know<br>99 = Refused to answer	[_____|_____]
**4. "What is the main activity or job you do to earn income" OR "If you do not have regular employment, what other things do you do to earn income?"**	1 = Peasant farmer<br>2 = Commercial farmer<br>3 = Brew alcohol<br>4 = Market vendor	5 = Shop keeper<br>6 = Transport(Driver/rider)<br>77 = Other<br>88 = Don't know<br>99 = Refused to answer	[_____|_____]

SECTION 2: HEAD OF HOUSEHOLD

"Now I would like to ask you some questions about your household."

**5. "Is the head of the household male or female?"**<br>*If primary caregiver is also the household head, go to Section 3*	1 = Male<br>2 = Female [_____]	**6. "How old is the head of household?"**	[_____|_____] years
**7. "What is the highest level of school completed by the head of household?"**	0 = None<br>1 = Primary (P1 — P4)<br>2 = Primary (P5 — P7)<br>3 = Secondary (S1 — S4)<br>4 = Secondary (S5 — S6)	10 = Certificate/Diploma<br>11 = University<br>77 = Other<br>88 = Don't know<br>99 = Refused to answer	[_____|_____]
**8. "What is the main activity or job the head of household does to earn income" OR "If he/she does not have regular employment, what other things does he/she do to earn income?"**	1 = Peasant farmer<br>2 = Commercial farmer<br>3 = Brew alcohol<br>4 = Market vendor	5 = Shop keeper<br>6 = Transport(Driver/rider)<br>77 = Other<br>88 = Don't know<br>99 = Refused to answer	[_____|_____]

SECTION 3: TREATMENT SEEKING BEHAVIOR

Attendance of Public health facilities and satisfaction with health care provided at Public Health facilities

**10. "Have you ever taken your child to a nearby public health facility for treatment?"**	1 = Yes<br>2 = No	88 = Don't know<br>99 = Refused to answer	[_____|_____]<br>*If yes go to Qn 11. Otherwise, skip to Section 4*
**11. "If yes, how satisfied were you with the health care that your child received at the public health facility?"**	1 = Very dissatisfied<br>2 = Dissatisfied<br>3 = Uncertain	4 = Satisfied<br>5 = Very satisfied<br>99 = Refused to answer	[_____|_____]<br>*If dissatisfied or very dissatisfied go to Qn 12. Otherwise, skip to Qn 13*
**12. "If dissatisfied or very dissatisfied what were the reasons for being dissatisfied?"** (list all that apply)	1 = Long waiting time<br>2 = No trained professionals<br>3 = No drugs were given<br>4 = No lab tests done<br>5 = Rude HCW	6 = Had to pay for care<br>7 = Treatment was unsuccessful<br>77 = Other<br>______________________________<br>99 = Refused to answer	[_____|_____] [_____|_____]<br>[_____|_____] [_____|_____]<br>[_____|_____] [_____|_____]
**13. "What was the treatment outcome when you took your child to the public health facility for health care?"**	1= Recovered quickly<br>2 = Recovered slowly<br>3 = Did not recover, and so I had to take child back	4 = Did not recover, so I sought care elsewhere<br>77 = Other_________________<br>99 = Refused to answer	[_____|_____]

COHORT STUDY HOUSEHOLD SURVEY (2)

Subcounty ID	Village ID	Compound #	Household #	Cluster #	Date of visit
[___]	[___|___]	[___|___|___|___]	[___|___]	[___|___]	[___|___]/ [___|___]/[___|___]<br>day month year

SECTION 4: BEDNETS

14. "Does your household have any mosquito nets?
1 = Yes
2 = No
88 = Don't know
99 = Refused to answer
[_____|_____]
If no, go to Section 5

15. "How many mosquito nets does your household have?" List number [_____|_____]

Net 1	Net 2 *(if more than 2 nets, go to extra bednet form)*
**16a. "How many months ago did you obtain the mosquito net?"**<br>88 = Don't know 77 = Other Insert number of months<br>99 = Refused to answer ________________ [_____|_____]	**16b. "How many months ago did you obtain the mosquito net?"**<br>88 = Don't know 77 = Other Insert number of months<br>99 = Refused to answer ________________ [_____|_____]
**17a. "From where did you get the mosquito nets?** [_____|_____]<br>1 = Government health center<br>2 = Government hospital<br>3 = Private hospital/clinic<br>4 = Private pharmacy<br>5 = Shop<br>6 = Open market<br>7 = Hawker<br>8 = Project/NGO<br>9 = Campaign<br>10 = Church<br>77 = Other ____________________<br>88 = Don't know<br>99 = Refused	**17b. "From where did you get the mosquito nets?** [_____|_____]<br>1 = Government health center<br>2 = Government hospital<br>3 = Private hospital/clinic<br>4 = Private pharmacy<br>5 = Shop<br>6 = Open market<br>7 = Hawker<br>8 = Project/NGO<br>9 = Campaign<br>10 = Church<br>77 = Other ____________________<br>88 = Don't know<br>99 = Refused
**18a. "May I have a look at the mosquito net?"**<br>*(Observe the net and record the status)*<br>1 = Observed and intact<br>2 = Observed and has visible holes<br>3 = Not a net<br>4 = Not observed [_____]	**18b. "May I have a look at the mosquito net?"**<br>*(Observe the net and record the status)*<br>1 = Observed and intact<br>2 = Observed and has visible holes<br>3 = Not a net<br>4 = Not observed [_____]
**19a. "What is the brand of the mosquito net?"**<br>*(If net was observed, record brand; if not, ask the respondent)*<br>1 = Long lasting net (Permanet, Smartnet, Olyset)<br>2 = Factory net with insecticide kit (KO, Kooper, Ico, Safi)<br>3 = Factory net with no insecticide (B52, Bamboo, Century, Lucky, Victoria)<br>4 = Home made net<br>77 = Other ______________________________<br>[____|____]	**19b. "What is the brand of the mosquito net?"**<br>*(If net was observed, record brand; if not, ask the respondent)*<br>1 = Long lasting net (Permanet, Smartnet, Olyset)<br>2 = Factory net with insecticide kit (KO, Kooper, Ico, Safi)<br>3 = Factory net with no insecticide (B52, Bamboo, Century, Lucky, Victoria)<br>4 = Home made net<br>77 = Other ______________________________<br>[____|____]
**20a. How many months ago was the mosquito net last soaked or dipped in insecticide to repel mosquitos?**<br>1 = < 6 mo<br>2 = ≥ 6 mo<br>3 = Never<br>88 = Don't know<br>99 = Refused to answer [_____|_____]	**20b. How many months ago was the mosquito net last soaked or dipped in insecticide to repel mosquitos?**<br>1 = < 6 mo<br>2 = ≥ 6 mo<br>3 = Never<br>88 = Don't know<br>99 = Refused to answer [_____|_____]

COHORT STUDY HOUSEHOLD SURVEY (3)

Subcounty ID	Village ID	Compound #	Household #	Cluster #	Date of visit
[___]	[___|___]	[___|___|___|___]	[___|___]	[___|___]	[___|___]/ [___|___]/[___|___]<br>day month year

SECTION 5: EXPERIENCE WITH ILLNESS DURING THE PAST TWO WEEKS

"Now I would like to ask you about your experiences with illness in this household and about what you do when your child is sick."

22. ""Have any of the children under your care been sick during the past two weeks?"
1 = Yes
2 = No
88 = Don't know
99 = Refused to answer
[_____|_____]
If no or unknown, skip to End.

SECTION 6: FIRST ACTION

"Now we would like to get a detailed step by step description of everything you did to care for your child during the most recent illness. There is no right or wrong answer to these questions. We need you to be as open and honest as possible."

23."Did your child have fever with this episode of illness?"
1=Yes
2= No
88= Don't know
99= Refused to answer
[_____|_____]

24. "What did you do FIRST (including tepid sponging and herbs)?" (choose only one action)
1 = Nothing
2 = Tepid sponging
3 = Gave herbs kept at home
4 = Gave medicines kept at home
5 = Bought medicines from duka
6 = Bought medicines at drug shop/pharmacy
7 = Took to traditional healer
8 = Took to public health centre, clinic or hospital
77 = Other ____________________
88 = Don't know
99 = Refused to answer
[_____|_____]
If 8, go to #25, otherwise skip to #29

25. "If you took your child to public health centre, clinic or hospital, where did you go?"
1 = Public health centre
2 = Tororo District Hospital
3 = Private hospital/clinic
77 = Other ____________________________
88 = Don't know
99 = Refused to answer
[_____|_____]
If 1 or 2, go to #26, otherwise skip to #29

26. "Why did you go to the public health centre or public hospital?"
1 = I heard there were drugs
2 = Someone told me to go
3 = It's closest to my home
4 = It's free (no cost)
5 = I always go to the public health centre
6 = They were nice to me last time I went
77 = Other _________________________
88 = Don't know
99 = Refused to answer
[_____|_____]

27. "How satisfied were you with the health care that your child received at the public health centre?"
1 = Very dissatisfied
2 = Dissatisfied
3 = Uncertain
4 = Satisfied
5 = Very satisfied
99 = Refused to answer
[_____|_____]
If 1 or 2 go to # 28, otherwise, skip to #29

28. "If dissatisfied or very dissatisfied what were the reasons for being dissatisfied?" (list all that apply)
1 = Long waiting time
2 = No trained professionals
3 = No drugs were given
4 = No lab tests done
5 = Rude HCW
6 = Had to pay for care
7 = Treatment was unsuccessful
77 = Other _____________________________
99 = Refused to answer
[_____|_____] [_____|_____]
[_____|_____] [_____|_____]
[_____|_____] [_____|_____]

29. "How long had the child been ill when this FIRST action was taken?"
1 = < 24 hrs
2 = 1-3 days
3 = 4-7 days
4 = > 7 days
77 = Other ____________________
88 = Don't know
99 = Refused to answer
[_____|_____]

MEDICINES GIVEN AS FIRST TREATMENT

"If your child took medicine FIRST, what did he/she take?" *(Indicate all that were given as a first action)*

DRUG	Number of times given per day	Number of days given	How long had the child been ill when the drug was started?<br>1 = < 24 hrs; 2 = 1-3 days; 3 = 4-7 days; 4 = > 7 days; 77 = Other (list); 88 = Don't know; 99 = Refused to answer
30. Panadol	[_____|_____]	[_____|_____]	[_____|_____] ______________________
31. Aspirin	[_____|_____]	[_____|_____]	[_____|_____] ______________________
32. Chloroquine	[_____|_____]	[_____|_____]	[_____|_____] ______________________
33. Fansidar *(SP)*	[_____|_____]	[_____|_____]	[_____|_____] ______________________
34. CQ+SP	[_____|_____]	[_____|_____]	[_____|_____] ______________________
35. Amodiaquine	[_____|_____]	[_____|_____]	[_____|_____] ______________________
36. Quinine	[_____|_____]	[_____|_____]	[_____|_____] ______________________
37. Coartem	[_____|_____]	[_____|_____]	[_____|_____] ______________________
38. Septrin *(Bactrim)*	[_____|_____]	[_____|_____]	[_____|_____] ______________________
39. Amoxacillin	[_____|_____]	[_____|_____]	[_____|_____] ______________________
40. Other ______________________	[_____|_____]	[_____|_____]	[_____|_____] ______________________
41. Other ______________________	[_____|_____]	[_____|_____]	[_____|_____] ______________________
42. Other ______________________	[_____|_____]	[_____|_____]	[_____|_____] ______________________
43. Unknown	[_____|_____]	[_____|_____]	[_____|_____] ______________________

IF ILLNESS RESOLVED, skip to Section 5 on treatment outcome

COHORT STUDY HOUSEHOLD SURVEY (4)

Subcounty ID	Village ID	Compound #	Household #	Cluster #	Date of visit
[___]	[___|___]	[___|___|___|___]	[___|___]	[___|___]	[___|___]/ [___|___]/[___|___]<br>day month year

SECTION 7: SECOND ACTION

44. "What did you do SECOND (including tepid sponging and herbs)?" (choose only one action)

1 = Nothing
2 = Tepid sponging
3 = Gave herbs kept at home
4 = Gave medicines kept at home
5 = Bought medicines from duka
6 = Bought medicines at drug shop/pharmacy
7 = Took to traditional healer
8 = Took to public health centre, clinic or hospital
77 = Other ___________________
88 = Don't know
99 = Refused to answer

[_____|_____]

If 8, go to #45, otherwise skip to #49

45. "If you took your child to public health centre, clinic or hospital, where did you go?"

1 = Public health centre
2 = Tororo District Hospital
3 = Private hospital/clinic
77 = Other ___________________________
88 = Don't know
99 = Refused to answer

[_____|_____]

If 1 or 2, go to #46, otherwise skip to #49

46. "Why did you go to the public health centre or public hospital?"

1 = I heard there were drugs
2 = Someone told me to go
3 = It's closest to my home
4 = It's free (no cost)
5 = I always go to the public health centre
6 = They were nice to me last time I went
7 = They told me to come back
8 = The drug given last time was unsuccessful
9 = There were no drugs at the health centre the last time I went
77 = Other _______________________________
88 = Don't know
99 = Refused to answer

[_____|_____]

47. "How satisfied were you with the health care that your child received at the public health centre?"

1 = Very dissatisfied
2 = Dissatisfied
3 = Uncertain
4 = Satisfied
5 = Very satisfied
99 = Refused to answer

[_____|_____]

If 1 or 2 go to # 48, otherwise, skip to #49

48. "If dissatisfied or very dissatisfied what were the reasons for being dissatisfied?" (list all that apply)

1 = Long waiting time
2 = No trained professionals
3 = No drugs were given
4 = No lab tests done
5 = Rude HCW
6 = Had to pay for care
7 = Treatment was unsuccessful
77 = Other _____________________________
99 = Refused to answer

[_____|_____] [_____|_____]
[_____|_____] [_____|_____]
[_____|_____] [_____|_____]

49. "How long had the child been ill when this SECOND action was taken?"

1 = < 24 hrs
2 = 1-3 days
3 = 4-7 days
4 = > 7 days
77 = Other ____________________
88 = Don't know
99 = Refused to answer

[_____|_____]

MEDICINES GIVEN AS SECOND TREATMENT

"If your child took medicine SECOND, what did he/she take?" *(Indicate all that were given as a first action)*

DRUG	Number of times given per day	Number of days given	How long had the child been ill when the drug was started?<br>1 = < 24 hrs 3 = 4-7 days 77 = Other (list)<br>2 = 1-3 days 4 = > 7 days 88 = Don't know<br>99 = Refused to answer
**50. Panadol**	[_____|_____]	[_____|_____]	[_____|_____] ______________________
**51. Aspirin**	[_____|_____]	[_____|_____]	[_____|_____] ______________________
**52. Chloroquine**	[_____|_____]	[_____|_____]	[_____|_____] ______________________
**53. Fansidar *(SP)***	[_____|_____]	[_____|_____]	[_____|_____] ______________________
**54. CQ+SP**	[_____|_____]	[_____|_____]	[_____|_____] ______________________
**55. Amodiaquine**	[_____|_____]	[_____|_____]	[_____|_____] ______________________
**56. Quinine**	[_____|_____]	[_____|_____]	[_____|_____] ______________________
**57. Coartem**	[_____|_____]	[_____|_____]	[_____|_____] ______________________
**58. Septrin *(Bactrim)***	[_____|_____]	[_____|_____]	[_____|_____] ______________________
**59. Amoxacillin**	[_____|_____]	[_____|_____]	[_____|_____] ______________________
**60. Other** ________________________	[_____|_____]	[_____|_____]	[_____|_____] ______________________
**61. Other** ________________________	[_____|_____]	[_____|_____]	[_____|_____] ______________________
**62. Other** ________________________	[_____|_____]	[_____|_____]	[_____|_____] ______________________
**63. Unknown**	[_____|_____]	[_____|_____]	[_____|_____] ______________________

IF ILLNESS RESOLVED, skip to Section 9: Treatment outcome

COHORT STUDY HOUSEHOLD SURVEY (5)

Subcounty ID	Village ID	Compound #	Household #	Cluster #	Date of visit
[___]	[___|___]	[___|___|___|___]	[___|___]	[___|___]	[___|___]/ [___|___]/[___|___]<br>day month year

SECTION 8: THIRD ACTION

64. "What did you do THIRD (including tepid sponging and herbs)?" (choose only one action)

1 = Nothing
2 = Tepid sponging
3 = Gave herbs kept at home
4 = Gave medicines kept at home
5 = Bought medicines from duka
6 = Bought medicines at drug shop/pharmacy
7 = Took to traditional healer
8 = Took to public health centre, clinic or hospital
77 = Other _______________
88 = Don't know
99 = Refused to answer

[_____|_____]

If 8, go to #65, otherwise skip to #69

65. "If you took your child to public health centre, clinic or hospital, where did you go?"

1 = Public health centre
2 = Tororo District Hospital
3 = Private hospital/clinic
77 = Other ____________________
88 = Don't know
99 = Refused to answer

[_____|_____]

If 1 or 2, go to #66, otherwise skip to #69

66. "Why did you go to the public health centre or public hospital?"

1 = I heard there were drugs
2 = Someone told me to go
3 = It's closest to my home
4 = It's free (no cost)
5 = I always go to the public health centre
6 = They were nice to me last time
7 = They told me to come back
8 = The drug given last time was unsuccessful
9 = There were no drugs at the health centre the last time I went
77 = Other ____________________________
88 = Don't know
99 = Refused to answer

[_____|_____]

67. "How satisfied were you with the health care that your child received at the public health centre?"

1 = Very dissatisfied
2 = Dissatisfied
3 = Uncertain
4 = Satisfied
5 = Very satisfied
99 = Refused to answer

[_____|_____]

If 1 or 2 go to #68. Otherwise, skip to #69

68. "If dissatisfied or very dissatisfied what were the reasons for being dissatisfied?" (list all that apply)

1 = Long waiting time
2 = No trained professionals
3 = No drugs were given
4 = No lab tests done
5 = Rude HCW
6 = Had to pay for care
7 = Treatment was unsuccessful
77 = Other ____________________________
99 = Refused to answer

[_____|_____] [_____|_____]
[_____|_____] [_____|_____]
[_____|_____] [_____|_____]

69. "How long had the child been ill when this THIRD action was taken?"

1 = < 24 hrs
2 = 1-3 days
3 = 4-7 days
4 = > 7 days
77 = Other _____________
88 = Don't know
99 = Refused to answer

[_____|_____]

MEDICINES GIVEN AS THIRD TREATMENT

"If your child took medicine THIRD, what did he/she take?" *(Indicate all that were given as a third action)*

DRUG	Number of times given per day	Number of days given	How long had the child been ill when the drug was started?<br>1 = < 24 hrs, 2 = 1-3 days, 3 = 4-7 days, 4 = > 7 days, 77 = Other (list), 88 = Don't know, 99 = Refused to answer
70. Panadol	[____|____]	[____|____]	[____|____] ________________________
71. Aspirin	[____|____]	[____|____]	[____|____] ________________________
72. Chloroquine	[____|____]	[____|____]	[____|____] ________________________
73. Fansidar *(SP)*	[____|____]	[____|____]	[____|____] ________________________
74. CQ+SP	[____|____]	[____|____]	[____|____] ________________________
75. Amodiaquine	[____|____]	[____|____]	[____|____] ________________________
76. Quinine	[____|____]	[____|____]	[____|____] ________________________
77. Coartem	[____|____]	[____|____]	[____|____] ________________________
78. Septrin *(Bactrim)*	[____|____]	[____|____]	[____|____] ________________________
79. Amoxacillin	[____|____]	[____|____]	[____|____] ________________________
80. Other _________________________	[____|____]	[____|____]	[____|____] ________________________
81. Other _________________________	[____|____]	[____|____]	[____|____] ________________________
82. Other _________________________	[____|____]	[____|____]	[____|____] ________________________
83. Unknown	[____|____]	[____|____]	[____|____] ________________________

IF ILLNESS RESOLVED, skip to Section 9 on treatment outcome, otherwise go to Extra Actions Form

COHORT STUDY HOUSEHOLD SURVEY (6)					
Subcounty ID	Village ID	Compound #	Household #	Cluster #	Date of visit
[___]	[___|___]	[___|___|___|___]	[___|___]	[___|___]	[___|___]/ [___|___]/[___|___]<br>day month year

SECTION 9: TREATMENT OUTCOME

**84. "How long did the child's illness last?"**	1 = < 24 hrs<br>2 = 1-3 days<br>3 = 4-7 days<br>4 = >7 days	5 = Ongoing at time of interview<br>77 = Other_______________________________<br>88 = Don't know<br>99 = Refused to answer	[_____|_____]
**85. "Did you experience any delays in treating your child's illness?"**	1 = Yes<br>2 = No	77 = Other _____________________________<br>88 = Don't know<br>99 = Refused to answer	[_____|_____]<br>If no, skip to question #87
**86. "What were the reasons for the delays?"** (list all that apply)	1 = No transport available<br>2 = Not enough money available<br>3 = Needed to find coverage for work	4 = Needed to arrange for child care<br>5 = Waiting at the health facility<br>6 = Was not at home<br>77 = Other ___________________<br>88 = Don't know<br>99 = Refused to answer	[_____|_____] [_____|_____]<br>[_____|_____] [_____|_____]<br>[_____|_____] [_____|_____]
**87. "How much did you spend on management of this illness?"**	Cost of drugs<br>[_____|_____|_____|_____|_____]Ush<br>Fees (clinic, hospital, lab)<br>[_____|_____|_____|_____|_____]Ush	Transport<br>[_____|_____|_____|_____|_____]Ush<br>Other<br>[_____|_____|_____|_____|_____]Ush	**TOTAL** [_____|_____|_____|_____|_____|_____]**Ush**
**88. "Did caring for your child and managing his/her illness prevent you from doing your usual activities this month?"**	1 = Yes<br>2 = No	88 = Don't know<br>99 = Refused to answer	[_____|_____]
**89. "If yes, how much time did you miss?"**	If < 1 day, indicate number of hours otherwise, record 00		[_____|_____] hours<br>[_____|_____] days

**Field worker ID**	[_____|_____]	**ID of observers present at interview**	[_____|_____] [_____|_____] [_____|_____]

APPENDIX U: COHORT STUDY HOUSEHOLD SURVEY (7)

Subcounty ID	Village ID	Compound #	Household #	Cluster #	Date of visit
[___]	[___|___]	[___|___|___|___]	[___|___]	[___|___]	[___|___]/ [___|___]/[___|___]<br>day month year

SECTION 4 cont'd: EXTRA BEDNETS *(Record number of net)*

Net [____]	Net [____]
**16c. "How many months ago did you obtain the mosquito net?"**<br>88 = Don't know 77 = Other Insert number of months<br>99 = Refused to answer ______________ [____|____]	**16d. "How many months ago did you obtain the mosquito net?"**<br>88 = Don't know 77 = Other Insert number of months<br>99 = Refused to answer ______________ [____|____]
**17c. "From where did you get the mosquito nets?** [____|____]<br>1 = Government health center 5 = Shop 10 = Church<br>6 = Open market 77 = Other<br>2 = Government hospital 7 = Hawker ____________________<br>3 = Private hospital/clinic 8 = Project/NGO 88 = Don't know<br>4 = Private pharmacy 9 = Campaign 99 = Refused	**17d. "From where did you get the mosquito nets?** [____|____]<br>1 = Government health center 5 = Shop 10 = Church<br>6 = Open market 77 = Other<br>2 = Government hospital 7 = Hawker ____________________<br>3 = Private hospital/clinic 8 = Project/NGO 88 = Don't know<br>4 = Private pharmacy 9 = Campaign 99 = Refused
**18c. "May I have a look at the mosquito net?"**<br>***(Observe the net and record the status)***<br>1 = Observed and intact 3 = Not a net<br>2 = Observed and has visible holes 4 = Not observed [____]	**18d. "May I have a look at the mosquito net?"**<br>***(Observe the net and record the status)***<br>1 = Observed and intact 3 = Not a net<br>2 = Observed and has visible holes 4 = Not observed [____]
**19c. "What is the brand of the mosquito net?"**<br>***(If net was observed, record brand; if not, ask the respondent)***<br>1 = Long lasting net (Permanet, Smartnet, Olyset)<br>2 = Factory net with insecticide kit (KO, Kooper, Ico, Safi)<br>3 = Factory net with no insecticide (B52, Bamboo, Century, Lucky, Victoria)<br>4 = Home made net<br>77 = Other ________________________ [___|___]	**19d. "What is the brand of the mosquito net?"**<br>***(If net was observed, record brand; if not, ask the respondent)***<br>1 = Long lasting net (Permanet, Smartnet, Olyset)<br>2 = Factory net with insecticide kit (KO, Kooper, Ico, Safi)<br>3 = Factory net with no insecticide (B52, Bamboo, Century, Lucky, Victoria)<br>4 = Home made net<br>77 = Other ________________________ [___|___]
**20c. How many months ago was the mosquito net last soaked or dipped in insecticide to repel mosquitos?**<br>1 = < 6 mo 3 = Never 99 = Refused to answer<br>2 = $\geq$ 6 mo 88 = Don't know [____|____]	**20d. How many months ago was the mosquito net last soaked or dipped in insecticide to repel mosquitos?**<br>1 = < 6 mo 3 = Never 99 = Refused to answer<br>2 = $\geq$ 6 mo 88 = Don't know [____|____]

Net [____]	Net [____]
**16e. "How many months ago did you obtain the mosquito net?"**<br>88 = Don't know 77 = Other Insert number of months<br>99 = Refused to answer ______________ [____|____]	**16f. "How many months ago did you obtain the mosquito net?"**<br>88 = Don't know 77 = Other Insert number of months<br>99 = Refused to answer ______________ [____|____]
**17e. "From where did you get the mosquito nets?** [____|____]<br>1 = Government health center 5 = Shop 10 = Church<br>6 = Open market 77 = Other<br>2 = Government hospital 7 = Hawker ____________________<br>3 = Private hospital/clinic 8 = Project/NGO 88 = Don't know<br>4 = Private pharmacy 9 = Campaign 99 = Refused	**17f. "From where did you get the mosquito nets?** [____|____]<br>1 = Government health center 5 = Shop 10 = Church<br>6 = Open market 77 = Other<br>2 = Government hospital 7 = Hawker ____________________<br>3 = Private hospital/clinic 8 = Project/NGO 88 = Don't know<br>4 = Private pharmacy 9 = Campaign 99 = Refused
**18e. "May I have a look at the mosquito net?"**<br>***(Observe the net and record the status)***<br>1 = Observed and intact 3 = Not a net<br>2 = Observed and has visible holes 4 = Not observed [____]	**18f. "May I have a look at the mosquito net?"**<br>***(Observe the net and record the status)***<br>1 = Observed and intact 3 = Not a net<br>2 = Observed and has visible holes 4 = Not observed [____]
**19e. "What is the brand of the mosquito net?"**<br>***(If net was observed, record brand; if not, ask the respondent)***<br>1 = Long lasting net (Permanet, Smartnet, Olyset)<br>2 = Factory net with insecticide kit (KO, Kooper, Ico, Safi)<br>3 = Factory net with no insecticide (B52, Bamboo, Century, Lucky, Victoria)<br>4 = Home made net<br>77 = Other ________________________ [___|___]	**19f. "What is the brand of the mosquito net?"**<br>***(If net was observed, record brand; if not, ask the respondent)***<br>1 = Long lasting net (Permanet, Smartnet, Olyset)<br>2 = Factory net with insecticide kit (KO, Kooper, Ico, Safi)<br>3 = Factory net with no insecticide (B52, Bamboo, Century, Lucky, Victoria)<br>4 = Home made net<br>77 = Other ________________________ [___|___]
**20e. How many months ago was the mosquito net last soaked or dipped in insecticide to repel mosquitos?**<br>1 = < 6 mo 3 = Never 99 = Refused to answer<br>2 = $\geq$ 6 mo 88 = Don't know [____|____]	**20f. How many months ago was the mosquito net last soaked or dipped in insecticide to repel mosquitos?**<br>1 = < 6 mo 3 = Never 99 = Refused to answer<br>2 = $\geq$ 6 mo 88 = Don't know [____|____]

COHORT STUDY HOUSEHOLD SURVEY (8)

Subcounty ID	Village ID	Compound #	Household #	Cluster #	Date of visit
[___]	[___|___]	[___|___|___|___]	[___|___]	[___|___]	[___|___]/ [___|___]/[___|___]<br>day month year

SECTION 8 cont'd: EXTRA ACTION [___]

(Record number of action)

84. "What did you do NEXT (including tepid sponging and herbs)?" (choose only one action)

1 = Nothing
2 = Tepid sponging
3 = Gave herbs kept at home
4 = Gave medicines kept at home
5 = Bought medicines from duka
6 = Bought medicines at drug shop/pharmacy
7 = Took to traditional healer
8 = Took to public health centre, clinic or hospital
77 = Other _______________
88 = Don't know
99 = Refused to answer

[_____|_____]

If 8, go to #85,otherwise skip to #89

85. "If you took your child to public health centre, clinic or hospital, where did you go?"

1 = Public health centre
2 = Tororo District Hospital
3 = Private hospital/clinic
77 = Other ____________________
88 = Don't know
99 = Refused to answer

[_____|_____]

If 1 or 2, go to #86, otherwise skip to #89

86. "Why did you go to the public health centre or public hospital?"

1 = I heard there were drugs
2 = Someone told me to go
3 = It's closest to my home
4 = It's free (no cost)
5 = I always go to the public health centre
6 = They were nice to me last time
7 = They told me to come back
8 = The drug given last time was unsuccessful
9 = There were no drugs at the health centre the last time I went
77 = Other ____________________________
88 = Don't know
99 = Refused to answer

[_____|_____]

87. "How satisfied were you with the health care that your child received at the public health centre?"

1 = Very dissatisfied
2 = Dissatisfied
3 = Uncertain
4 = Satisfied
5 = Very satisfied
99 = Refused to answer

[_____|_____]

If 1 or 2 go to #88. Otherwise, skip to #89

88. "If dissatisfied or very dissatisfied what were the reasons for being dissatisfied?" (list all that apply)

1 = Long waiting time
2 = No trained professionals
3 = No drugs were given
4 = No lab tests done
5 = Rude HCW
6 = Had to pay for care
7 = Treatment was unsuccessful
77 = Other ____________________________
99 = Refused to answer

[_____|_____] [_____|_____]
[_____|_____] [_____|_____]
[_____|_____] [_____|_____]

89. "How long had the child been ill when this NEXT action was taken?"

1 = < 24 hrs
2 = 1-3 days
3 = 4-7 days
4 = > 7 days
77 = Other _____________
88 = Don't know
99 = Refused to answer

[_____|_____]

MEDICINES GIVEN AS NEXT TREATMENT

"If your child took medicine THIRD, what did he/she take?" *(Indicate all that were given as a third action)*

DRUG	Number of times given per day	Number of days given	How long had the child been ill when the drug was started?<br>1 = < 24 hrs 2 = 1-3 days 3 = 4-7 days 4 = > 7 days 77 = Other (list) 88 = Don't know 99 = Refused to answer
**90. Panadol**	[_____|_____]	[_____|_____]	[_____|_____] ________________________
**91. Aspirin**	[_____|_____]	[_____|_____]	[_____|_____] ________________________
**92. Chloroquine**	[_____|_____]	[_____|_____]	[_____|_____] ________________________
**93. Fansidar *(SP)***	[_____|_____]	[_____|_____]	[_____|_____] ________________________
**94. CQ+SP**	[_____|_____]	[_____|_____]	[_____|_____] ________________________
**95. Amodiaquine**	[_____|_____]	[_____|_____]	[_____|_____] ________________________
**96. Quinine**	[_____|_____]	[_____|_____]	[_____|_____] ________________________
**97. Coartem**	[_____|_____]	[_____|_____]	[_____|_____] ________________________
**98. Septrin *(Bactrim)***	[_____|_____]	[_____|_____]	[_____|_____] ________________________
**99. Amoxacillin**	[_____|_____]	[_____|_____]	[_____|_____] ________________________
**100. Other** ________________________	[_____|_____]	[_____|_____]	[_____|_____] ________________________
**101. Other** ________________________	[_____|_____]	[_____|_____]	[_____|_____] ________________________
**102. Other** ________________________	[_____|_____]	[_____|_____]	[_____|_____] ________________________
**103. Unknown**	[_____|_____]	[_____|_____]	[_____|_____] ________________________

IF ILLNESS RESOLVED, skip to Section 9 on treatment outcome, otherwise go to Extra Actions Form

Ques. No.	Variable Name	Question	Variable Type	Variable Codes	Accepted Values	Skip	Comments
**Appendix UII: Part 1 - Household Survey**							
**Household Unique Identification-** *this information is unique for each household on recruitment list, to be programmed as a primary key (to avoid duplicates)*							
1	DATEFIRST	Date of Initial visit	Date				Can be a date before today for a revisit
2	DATELAST	Date of final visit	Date		cannot be before todays date		automatically generated
3	STARTIME	Start time of interview	String (HH:SS)				automatically generated
4	CLUSTNUM	Cluster number	Numeric		1 - 20		
5	SUBCTYNUM	Sub-county number	Numeric	1 - Nagongera<br>2 - Paya<br>3 - Kisoko<br>4 - Kirewa<br>5 - Petta<br>6 - Mulanda 7<br>- Ruhongi	as per variable codes		data is recorded twice to ensure data integrity
6	VILLAGENUM	Village number	Numeric		1 - 999		
7	COMPNUM	Compound number	Numeric		1 - 9999		
8	HHHNUM	Household number	Numeric		1 - 99		
9	STAFFID	Staff ID	Numeric				
**Section 1: Head of household's (Respondent's) Background**							
10	HHAGEYRS	How old are you?	Numeric	88 - Don't know<br>99 - Refused to answer/skipped	10 - 120,999	- if < 15 or > 49, skip to section 6.<br>- if refuse to answer, skip to section 6.	
11	HHSCHOOL	Have you ever attended school?	Numeric	1 - Yes<br>2 - No<br>99 - Refused to answer/skipped	as per variable codes	if <> 1 then skip to NEWSPR	
12	HHHIGHEST	What is the highest level of school you attended?	Numeric	0 - None 1<br>- Primary (P1-P4)<br>2 - Primary (P5-P7)<br>3 - Secondary (S1-S4)<br>4 - Secondary(S5-S6)<br>10 - Certificate/Diploma<br>11 - University<br>77 - Other ________________________<br>88 - Don't know<br>99 - Refused to answer	as per variable codes		
13	HHJOBTRN	Have you completed any other type of job training school?	Numeric	1 - Yes<br>2 - No<br>99 - Refused to answer/skipped	as per variable codes		
14	HHREAD	"Now I would like you to read a sentence. In what language do you read?"<br>SELECT AND SHOW THE APPROPRIATE LITERACY CARD TO CAREGIVER. LISTEN AND GRADE THE READING EFFORT. *If cannot read skip to question #17	Numeric	1 - Cannot read at all*<br>2 - Can read only parts of the sentence<br>3 - Able to read the whole sentence<br>4 - No card with required language (specify language) ____________________<br>99 - Refused to answer/skipped	as per variable codes		
15	HHNEWSPR	Do you read a newspaper or magazine almost every day, at least once a week, less than once a week or not at all?	Numeric	1 - Almost every day<br>2 - At least once a week<br>3 - Less than once a week<br>4 - Not at all<br>8 - Cannot read<br>99 - Refused to answer/skipped	as per variable codes		
16	HHRADIO	Do you listen to the radio almost every day, at least once week, less than once a week or not at all?	Numeric	1 - Almost every day<br>2 - At least once a week<br>3 - Less than once a week<br>4 - Not at all<br>99 - Refused to answer/skipped	as per variable codes		
17	HHTV	Do you watch television almost every day, at least once a week, less than once a week or not at all?	Numeric	1 - Almost every day<br>2 - At least once a week<br>3 - Less than once a week<br>4 - Not at all<br>99 - Refused to answer/skipped	as per variable codes		
18	HHJOB	What is the main activity or job you do to provide your family with income OR "*If you do not have regular employment, what other things do you do to help provide your family with income?*	Numeric	1 - Peasant farmer<br>2 - Student<br>3 - Military/police/security<br>4 - Construction worker/casual labourer<br>5 - Housekeeper<br>6 - Selling goods/shopkeeper/businessman<br>7 - Selling food sfuffs (catering/food vendor)<br>8 - Taxi, bodaboda or bicycle rider<br>9 - Mechanic<br>10 - Government/clerical/secretarial work.<br>11 - Health worker<br>12 - Teacher<br>14 - None/unemployed<br>77 - Other __________________<br>99 - Refused to answer/skipped	as per variable codes	if = 1 then skip to PAID<br>if = 9 then skip to WORK3	

Ques. No.	Variable Name	Question	Variable Type	Variable Codes	Accepted Values	Skip	Comments
19	HHWORK12	Have you done any work in the last 12 months?	Numeric	1 - Yes<br>2 - No<br>99 - Refused to answer/skipped	as per variable codes	if = 2 or 9 skip to WKSTMT	
20	HHPAID	Are (were) you paid in cash or kind for this work or are (were) you not paid at all?<br><br>IF PAID, ASK WHETHER CASH, IN-KIND, OR BOTH.	Numeric	1 - Cash only<br>2 - Cash and kind<br>3 - In-kind only<br>4 - Not paid<br>99 - Refused to answer/skipped	as per variable codes		
21	HHWORKJOB	if you have done any work in the last 12 months,what have you been doing for most of the time during this period?	Numeric	1 - going to school/studying<br>2 - looking for work<br>3 - retired<br>4 - too ill to work<br>5 - handicapped, cannot work<br>6 - housework/child care<br>96 - other<br>99 - Refused to answer/skipped	as per variable codes	if = 96, specify other	
22	HHWORKOT	Specify other non-work activity	String				
23	RESIDENTS	How many people usually live in your household (total number including children)?	Numeric		1-32		
24	WOMENCBA	How many women of child-bearing age (13-49 years) live in your household ?	Numeric		1-32		
25	WOMENLIST	List all women of child-bearing age (13-49 years) in your household					
**Section 2: Primary Caregiver Background**							
22	PCAGEYRS	How old are you?	Numeric	88 - Don't know<br>99 - Refused to answer/skipped	10 - 120,999	- if < 13 or > 49, skip to section 6.<br>- if refuse to answer, skip to section 6.	
23	PCSCHOOL	Have you ever attended school?	Numeric	1 - Yes<br>2 - No<br>99 - Refused to answer/skipped	as per variable codes	if > 1 then skip to NEWSPR	
24	PCHIGHEST	What is the highest level of school you attended?	Numeric	0 - None 1<br>- Primary (P1-P4)<br>2 - Primary (P5-P7)<br>3 - Secondary (S1-S4)<br>4 - Secondary(S5-S6)<br>10 - Certificate/Diploma<br>11 - University<br>77 - Other ________________________<br>88 - Don't know<br>99 - Refused to answer	as per variable codes		
25	PCJOBTRN	Have you completed any other type of job training school?	Numeric	1 - Yes<br>2 - No<br>99 - Refused to answer/skipped	as per variable codes		
26	PCREAD	"Now I would like you to read a sentence. In what language do you read?"<br>SELECT AND SHOW THE APPROPRIATE LITERACY CARD TO CAREGIVER. LISTEN AND GRADE THE READING EFFORT.<br>*If cannot read skip to question #17	Numeric	1 - Cannot read at all*<br>2 - Can read only parts of the sentence<br>3 - Able to read the whole sentence<br>4 - No card with required language (specify language) ____________________<br>99 - Refused to answer/skipped	as per variable codes		
27	PCNEWSPR	Do you read a newspaper or magazine almost every day, at least once a week, less than once a week or not at all?	Numeric	1 - Almost every day<br>2 - At least once a week<br>3 - Less than once a week<br>4 - Not at all<br>8 - Cannot read<br>99 - Refused to answer/skipped	as per variable codes		
28	PCRADIO	Do you listen to the radio almost every day, at least once week, less than once a week or not at all?	Numeric	1 - Almost every day<br>2 - At least once a week<br>3 - Less than once a week<br>4 - Not at all<br>99 - Refused to answer/skipped	as per variable codes		
29	PCTV	Do you watch television almost every day, at least once a week, less than once a week or not at all?	Numeric	1 - Almost every day<br>2 - At least once a week<br>3 - Less than once a week<br>4 - Not at all<br>99 - Refused to answer/skipped	as per variable codes		

Ques. No.	Variable Name	Question	Variable Type	Variable Codes	Accepted Values	Skip	Comments
30	PCJOB	What is your occupation, that is, what kind of work do you mainly do?<br><br>INTERVIEWER: PROBE TO OBTAIN DETAILED INFORMATION ON THE KIND OF WORK RESPONDENT DOES.	String	1 - Peasant farmer<br>2 - Student<br>3 - Military/police/security<br>4 - Construction worker/casual labourer<br>5 - Housekeeper<br>6 - Selling goods/shopkeeper/businessman<br>7 - Selling food sfuffs (catering/food vendor)<br>8 - Taxi, bodaboda or bicycle rider<br>9 - Mechanic<br>10 - Government/clerical/secretarial work.<br>11 - Health worker<br>12 - Teacher<br>14 - None/unemployed<br>77 - Other ___________________<br>99 - Refused to answer/skipped		skip to Section 3	
31	PCWORK12	Have you done any work in the last 12 months?	Numeric	1 - Yes<br>2 - No<br>99 - Refused to answer/skipped	as per variable codes	if = 2 or 9 skip to WKSTMT	
32	PCPAID	Are (were) you paid in cash or kind for this work or are (were) you not paid at all?<br><br>IF PAID, ASK WHETHER CASH, IN-KIND, OR BOTH.	Numeric	1 - Cash only<br>2 - Cash and kind<br>3 - In-kind only<br>4 - Not paid<br>99 - Refused to answer/skipped	as per variable codes		
33	PCWORKJOB	if you have done any work in the last 12 months,what have you been doing for most of the time during this period?	Numeric	1 - going to school/studying<br>2 - looking for work<br>3 - retired<br>4 - too ill to work<br>5 - handicapped, cannot work<br>6 - housework/child care<br>96 - other<br>99 - Refused to answer/skipped	as per variable codes	if = 96, specify other	
34	PCWORKOT	Specify other non-work activity	String				
**Section 3: Household Characteristics (Wealth Indicators)**							
35	SWATER	What is the main source of drinking water for members of your household?	Numeric	10 - borehole<br>11 - pipe into dwelling<br>12 - piped into yard/compound<br>13 - public tap<br>21 - open well in yard/compound<br>22 - open public well<br>31 - protected well in yard/compound<br>32 - protected public well<br>41 - protected spring<br>42 - unprotected spring<br>43 - river/stream<br>44 - pond/lake<br>45 - dam<br>51 - rainwater<br>61 - water truck<br>71 - bottled water<br>96 - other	as per variable codes	if = 96 then specify Other	
36	OTHERSCS	Specify other source of water	String				
37	TFACLTY	What kind of toilet facility do members of your household usually use?	Numeric	1 - flush toilet<br>2 - vip latrine<br>3 - covered pit latrine no slab<br>4 - covered pit latrine w/slab<br>5 - uncovered pit latrine no slab<br>6 - uncovered pit latrine w/slab<br>7 - composting toilet<br>8 - no facility/bush/field<br>77 - other<br>99 - Refused to answer/skipped	as per variable codes	if = 96 then specify Other	
38	OTHERFCY	Specify other kind of toilet facilities	String				
39	ELECTIRC	Does your household have...<br><br>...Electricity?	Numeric	1 - Yes<br>2 - No<br>88 - Don't know<br>99 - Refused to answer/skipped	as per variable codes		
40	RADIO	...Radio?	Numeric	1 - Yes<br>2 - No<br>88 - Don't know<br>99 - Refused to answer/skipped	as per variable codes		
41	TV	...Television?	Numeric	1 - Yes<br>2 - No<br>88 - Don't know<br>99 - Refused to answer/skipped	as per variable codes		

Ques. No.	Variable Name	Question	Variable Type	Variable Codes	Accepted Values	Skip	Comments
42	MOBILE	...Mobile phone?	Numeric	1 - Yes<br>2 - No<br>88 - Don't know<br>99 - Refused to answer/skipped	as per variable codes		
43	FRIDGE	...Refrigerator?	Numeric	1 - Yes<br>2 - No<br>88 - Don't know<br>99 - Refused to answer/skipped	as per variable codes		
44	SOFA	...Sofa set?	Numeric	1 - Yes<br>2 - No<br>88 - Don't know<br>99 - Refused to answer/skipped	as per variable codes		
45	BED	...Bed?	Numeric	1 - Yes<br>2 - No<br>88 - Don't know<br>99 - Refused to answer/skipped	as per variable codes		
46	CLOCK	...Clock?	Numeric	1 - Yes<br>2 - No<br>88 - Don't know<br>99 - Refused to answer/skipped	as per variable codes		
47	FUELTYPE	What type of fuel does your household mainly use for cooking?	Numeric	1 - electricity<br>2 - lpg/natural gas<br>3 - biogas<br>4 - paraffin/kerosene<br>5 - charcoal<br>6 - firewood<br>7 - starw/shrubs/grass<br>8 - animal dung<br>95 - no food cooked in household<br>96 - other<br>99 - Refused to answer/skipped	as per variable codes	if = 96 then specify Other	
48	OTHERFUE	Specify other type of fuel used	String				
49	SENERGY	What do you use for lighting energy in this household?	Numeric	1 - electricity<br>2 - solar<br>3 - gas<br>4 - paraffin - hurricane lamp<br>5 - paraffin - pressure lamp<br>6 - paraffin - wick lamp<br>7 - firewood<br>8 - candles<br>96 - other<br>99 - Refused to answer/skipped	as per variable codes	if = 96 then specify Other	
50	OTHERENG	Specify other source of energy for lighting	String				
51	MMFLOOR	MAIN MATERIAL OF THE FLOOR<br><br>RECORD OBSERVATION.<br><br>MARK ONLY ONE.	Numeric	11 - earth/sand<br>12 - earth and dung<br>31 - parquet or polished wood<br>33 - mosaic or tiles<br>34 - bricks<br>35 - cement<br>36 - stones<br>96 - other<br>99 - Refused to answer/Skipped	as per variable codes	if = 96 then specify Other	
52	OTHERMMF	Specify other material of the floor	String				
53	MMROOF	MAIN MATERIAL OF THE ROOF.<br><br>RECORD OBSERVATION.<br><br>MARK ONLY ONE.	Numeric	11-thatched<br>12-mud<br>21-wood/planks<br>22-iron sheets<br>23-asbestos<br>24-tiles<br>25-tin<br>26-cement<br>96-other<br>99 - Refused to answer/Skipped	as per variable codes	if = 96 then specify Other	
54	OTHERMMR	Specify other material of the roof	String				
55	MMEWALLS	MAIN MATERIAL OF THE EXTERIOR WALLS.<br><br>RECORD OBSERVATION.<br><br>MARK ONLY ONE.	Numeric	11 - thatched/straw<br>21 - mud and poles<br>22 - un-burnt bricks<br>23 - un-burnt bricks with plaster<br>24 - burnt bricks with mud<br>31 - cement blocks<br>32 - stone<br>33 - timber<br>34 - burnt bricks with cement<br>96 - other<br>99 - Refused to answer/Skipped	as per variable codes	if = 96 then specify Other	
56	OTHERMME	Specify other material of the exterior walls	String				

Ques. No.	Variable Name	Question	Variable Type	Variable Codes	Accepted Values	Skip	Comments
57	HHROOMS	How many rooms in your household are used for sleeping?<br><br>(INCLUDING ROOMS OUTSIDE THE MAIN DWELLING)<br><br>If there are 15 or more rooms, enter 15	Numeric	88 - Don't know<br>99 - Refused to answer/skipped	1 - 15,98,99		
58	WATCH	Does any member of your household own or have…<br><br>…A watch?	Numeric	1 - Yes<br>2 - No<br>88 - Don't know<br>99 - Refused to answer/skipped	as per variable codes		
59	BICYCLE	….A bicycle?	Numeric	1 - Yes<br>2 - No<br>88 - Don't know<br>99 - Refused to answer/skipped	as per variable codes		
60	SCOOTER	…A motorcycle or motor scooter?	Numeric	1 - Yes<br>2 - No<br>88 - Don't know<br>99 - Refused to answer/skipped	as per variable codes		
61	CAR	…A car or truck?	Numeric	1 - Yes<br>2 - No<br>88 - Don't know<br>99 - Refused to answer/skipped	as per variable codes		
62	BANKACCO	…A bank account?	Numeric	1 - Yes<br>2 - No<br>88 - Don't know<br>99 - Refused to answer/skipped	as per variable codes		
63	CATTLE	How many **Cattle** does this household own?<br><br>If none, enter 00<br>If more than 95, enter 95	Numeric	88 - Don't know<br>99 - Refused to answer/skipped	0 - 95, 98,99		
64	GOATS	How many **Goats** does this household own?<br><br>If none, enter 00<br>If more than 95, enter 95<br>If unknown, enter 98	Numeric	88 - Don't know<br>99 - Refused to answer/skipped	0 - 95, 98,99		
65	SHEEP	How many **Sheep** does this household own?<br><br>If none, enter 00<br>If more than 95, enter 95<br>If unknown, enter 98	Numeric	88 - Don't know<br>99 - Refused to answer/skipped	0 - 95, 98,99		
66	PIGS	How many **Pigs** does this household own?<br><br>If none, enter 00<br>If more than 95, enter 95<br>If unknown, enter 98	Numeric	88 - Don't know<br>99 - Refused to answer/skipped	0 - 95, 98,99		
67	CHICKEN	How many **chickens** does this household own?<br><br>If none, enter 00<br>If more than 95, enter 95<br>If unknown, enter 98	Numeric	88 - Don't know<br>99 - Refused to answer/skipped	0 - 95, 98,99		
68	HHPSF	How often in the last year did you have problems in satisfying the food needs of the household?	Numeric	1 - never<br>2 - seldom<br>3 - sometimes<br>4 - often<br>5 - always<br>88 - Don't know<br>99 - Refused to answer/skipped	as per variable codes		
69	DHFCTY	How far is it to the nearest health facility?<br><br>If less than 1 km, enter 00<br>If more than 95 km, enter 95<br>If Don't know, enter 98	Numeric	88 - Don't know<br>99 - Refused to answer/skipped	0 - 95, 98,99		
70	MTHFCTY	If you were to go this facility,how would you <u>most likely</u> go there?	Numeric	1 - car/motorcycle<br>2 - public transport(bus,taxi)<br>3 - animal/animal cart<br>4 - walking<br>5 - bicycle<br>6 - other<br>88 - Don't know<br>99 - Refused to answer/skipped	as per variable codes	if = 6 then specify Other	Walk to trading center and take a car or bike is: "car/motorbike". Most common means used!
71	OTHERMTH	Specify other means of transport to the health facility	String				
**Section 4: Bednets -** *All questions in this section will be repeated for each mosquito net in the household (hhnumnet)*							

Ques. No.	Variable Name	Question	Variable Type	Variable Codes	Accepted Values	Skip	Comments
72	HHAMNETS	Does this household have any mosquito nets ?	Numeric	1 - Yes<br>2 - No<br>88 - Don't know<br>99 - Refused to answer/skipped	as per variable codes	if = 2 or 9 then skip to Next SECTION	
73	HNUMNETS	How many mosquito nets does your household have?<br><br>IF MORE THAN 12, ENTER 13	Numeric	99 - Refused to answer/skipped	1 - 13,99		Again, the software is not capable of an 'infinite' number of nets, therefore it has to be preprogrammed for the maximum number of nets we expect to find at a household: 12 was chosen
74	MNTH1	How many months ago did your household obtain the mosquito net?<br><br>IF LESS THAN ONE MONTH, WRITE '0'.<br>IF MORE THAN 36 MONTHS AGO RECORD 95<br>IF NOT SURE RECORD 98	Numeric	0 - less than 1 month<br>88 - Don't know<br>95 - More than 36 months<br>99 - Refused to answer/skipped	0-36,88,95,99		
75	WHERE1	Where did you get the mosquito net from?		1 - Gov't health center<br>2 - Gov't hospital<br>3 - Private hospital/clinic<br>4 - Private pharmacy<br>5 - Shop<br>6 - Open market<br>7 - Hawker<br>8 - Project/ngo<br>9 - Campaign/VHT<br>10 - Church<br>77 - Other<br>88 - Does not know<br>99 - Refused to answer/skipped	as per variable codes	if = 96 then specify other	
76	OBS1	May I have a look at (all) the net(s) to establish the brand?	Numeric	1 - Observed and intact<br>2 - Observed and has visible holes<br>3 - Not a net<br>4 - Not observed	as per variable codes		
77	BRAND1	OBSERVE OR ASK THE BRAND OR TYPE OF MOSQUITO NET.	Numeric	1 - Long lasting net (Permanet, Duranet, Interceptor, Netprotect, Olyset)<br>2 - Factory net **with** insecticide kit (KO-net, Kooper net, Iconet, Safi net)<br>3 - Factory net **with no** insecticide (B52, bamboo hut, century, lucky net, victoria)<br><br>4 - homemade net<br>77 - other<br>88 - Don't know brand<br>99 - Refused to answer/skipped	as per variable codes	if = 1 then skip to SLPNETx<br><br>if = 2, 3,4,77, 88 then skip to next question	
78	OTHERB1	Specify other brands or types of mosquitonet	String				
79	SMNET1	Since you got the mosquito net, was it ever soaked or dipped in a liquid to repel mosquitoes or bugs?	Numeric	1 - Yes<br>2 - No<br>77 - Other<br>88 - Don't know	as per variable codes	if <> 1 then skip to SLPNETx	
80	TSMNET1	How many months ago was the net last soaked or dipped?<br><br>IF LESS THAN 1 MONTH, RECORD '0'.<br>IF 25 OR MORE MONTHS AGO, RECORD 95<br>IF NOT SURE, RECORD 98	Numeric	1 - < 6 months<br>2 - ≥6 months<br>3 - Never<br>95 - More than 25 months ago<br>88 - Don't know<br>99 - Refused to answer	as per variable codes		
81	SLPNET1	Did anyone sleep under this mosquito net last night?	Numeric	1 - Yes<br>2 - No<br>8 - Not sure<br>9 - Refused to answer/skipped	as per variable codes	if = 1 then skip to UNETLxA<br>if = 8 or 9 then repeat Section 4 if there are still more nets in the house (based on HNUMNETS)	
82	NUSED1	What are some of the reasons why this net was not used?<br><br>PROBE FOR THE MAIN REASON.<br><br>SELECT ONLY ONE.	Numeric	1 - Too hot<br>2 - Don't like smell<br>3 - No mosquitoes<br>4 - Net too old/too many holes<br>5 - Net not hang<br>6 - Don't want to hang<br>7- Net no longer kills insects<br>77 - other<br>88 - Don't Know<br>99 - Refused to answer	as per variable codes		

Ques. No.	Variable Name	Question	Variable Type	Variable Codes	Accepted Values	Skip	Comments
83	NUSED1I	...Other	Numeric	1 - Yes<br>2 - No<br>99 - Refused to answer/skipped	as per variable codes	if = 1 then specify other	
84	OTHERN1	Specify other	String				
85	NTHUNG1	If not hung, why not?<br><br>PROBE FOR THE MAIN REASON.<br><br>SELECT ONLY ONE.	Numeric	1 - Nowhere to hang<br>2 - Don't know how to hang net<br>3 - no tools to hang net<br>4 - shape did not fit<br>5 - size did not fit<br>6 - Don't want to hang<br>77 - other<br>88 - Don't know<br>99 - Refused to answer/skipped	as per variable codes	if <> 77 then repeat Section if there are still more nets in the house (based on HNUMNETS)	
86	OTHERNT1	Specify other reason why the net was not hung.	String			after this question, repeat Section 4 if there are still more nets in the house (based on HNUMNETS)	
**Section 5: Experience with managing an ill child in the last 2 weeks**							
77	FEVCH1	Has **any** of the children under your care been ill with a fever at any time in the past 2 weeks?	Numeric	1 - Yes<br>2 - No<br>88 - Don't know<br>99 - Refused to answer/skipped	as per variable codes		
78	FSEEK1	What did you do **FIRST**(Including tepid sponging and herbs)?	Numeric	1 - Nothing<br>2 - Tepid sponging<br>3 - Gave Herbs Kept at home<br>4 - Gave Medicines Kept at home<br>5 - Bought medicines from a Duka/shop<br>6 - Bought mediciness from drug shop/Pharmacy<br>7 - Took to Traditional healer<br>8 - Took to a public health centre,clinic or hospital<br>77 - Other<br>88 - Don't Know<br>99 - Refused to answer/skipped	as per variable codes		
79	TMTCD1	Did you seek advice or treatment for the illness from any source?	Numeric	1 - Yes<br>2 - No<br>88 - Don't know<br>99 - Refused to answer/skipped	as per variable codes	if = 1 or 9 skip to SEEKAx	
80	NSTRT1A	Why have you not sought advice or treatment from any source?<br><br>...Child just fell ill	Numeric	1 - Yes<br>2 - No<br>88 - Don't know<br>99 - Refused to answer/skipped	as per variable codes		
81	NSTRT1B	...Child not very ill	Numeric	1 - Yes<br>2 - No<br>88 - Don't know<br>99 - Refused to answer/skipped	as per variable codes		
82	NSTRT1C	...Clinic too far	Numeric	1 - Yes<br>2 - No<br>88 - Don't know<br>99 - Refused to answer/skipped	as per variable codes		
83	NSTRT1D	...Have no money	Numeric	1 - Yes<br>2 - No<br>88 - Don't know<br>99 - Refused to answer/skipped	as per variable codes		
84	NSTRT1E	...Waiting for child's father	Numeric	1 - Yes<br>2 - No<br>88 - Don't know<br>99 - Refused to answer/skipped	as per variable codes		
85	NSTRT1F	...Don't know what to do	Numeric	1 - Yes<br>2 - No<br>88 - Don't know<br>99 - Refused to answer/skipped	as per variable codes		
86	NSTRT1G	...Already had medicine at home	Numeric	1 - Yes<br>2 - No<br>88 - Don't know<br>99 - Refused to answer/skipped	as per variable codes		
87	NSTRT1H	...Other	Numeric	1 - Yes<br>2 - No<br>88 - Don't know<br>99 - Refused to answer/skipped	as per variable codes	if = 2 or 9 skip to FVCHDx	
88	OCHOT1	Specify other	String				

Ques. No.	Variable Name	Question	Variable Type	Variable Codes	Accepted Values	Skip	Comments
89	ACTN1	How long had the child been ill when this FIRST action was taken?	Numeric	1 - < 24 hrs<br>2 - 1-3 days<br>3 - 4-7 days<br>4- > 7 days<br>88 - Don't know<br>99 - Refused to answer			
90	SEEK1A	If you took your child to a Public Health Centre,Clinic or Hospital,Where did you go?	Numeric	1 - Public health Centre<br>2 - Tororo District Hospital<br>3- Private Hospital/Clinic<br>77 - Other<br>88 -Don't Know<br>99-Refused to answer	as per variable codes	if = 1 or 2 go to SEEKPUBL otherwise skip to	
91	SEEKPUBL	Why did you go to the Public health centre or Public hospital ?	Numeric	1 - I heard there were drugs<br>2 - Someone told me to go<br>3 - It's closest to my home<br>4 - It's free(No cost)<br>5 - I always go to the Public health Centre<br>6 - They were nice to me the last time i went<br>77 - Other<br>88 - Don't Know<br>99 - Refused to answer/skipped	as per variable codes		
92	SERVPUBL1	How satisfied were you with the health care that your child received at the Public health centre?	Numeric	1 - Very dissatisfied<br>2 - Dissatisfied<br>3 - Uncertain<br>4 - Satisfied<br>5 - Very satisfied<br>99 - Refused to answer	as per variable codes	if = 1 or 2 go to SERVPUBL2 otherwise skip to	
93	SERVPUBL2	If dissatisfied or very dissatisfied, what were the reasons for being dissatisfied?	Numeric	1 - Long waiting time<br>2 - No trained professionals<br>3 - No drugs were given<br>4 - No lab tests done<br>5 - Rude HCW<br>6 - Had to pay for care<br>7- Treatment was unsuccessful<br>99 - Refused to answer	as per variable codes		
94	BDTST1	At any time during the illness, did the child have blood taken from his/her finger or heel for testing?	Numeric	1 - Yes<br>2 - No<br>88 - Don't know<br>99 - Refused to answer/skipped	as per variable codes		
95	FVCHD1	Is [NAME] still sick with a fever?	Numeric	1 - Yes<br>2 - No<br>88 - Don't know<br>99 - Refused to answer/skipped	as per variable codes		
96	MEDGIV	If your child took medicine **FIRST**,what did he/she take?<br><br>RECORD ALL MENTIONED.					
97	MED1	Panadol	Numeric	1 - Yes<br>2 - No<br>88 - Don't know<br>99 - Refused to answer/skipped	as per variable codes	if =1 then must answer DURTN1x, NUMTMESx, and NUMDAYSx	
98	MED2	Aspirin	Numeric	1 - Yes<br>2 - No<br>88 - Don't know<br>99 - Refused to answer/skipped	as per variable codes	if =1 then must answer DURTN1x, NUMTMESx, and NUMDAYSx	
99	MED3	Chloroquine(CQ)	Numeric	1 - Yes<br>2 - No<br>88 - Don't know<br>99 - Refused to answer/skipped	as per variable codes	if =1 then must answer DURTN1x, NUMTMESx, and NUMDAYSx	
100	MED4	Fansidar(SP)	Numeric	1 - Yes<br>2 - No<br>88 - Don't know<br>99 - Refused to answer/skipped	as per variable codes	if =1 then must answer DURTN1x, NUMTMESx, and NUMDAYSx	
101	MED5	Chloroquine + Fansidar(CQ+SP)	Numeric	1 - Yes<br>2 - No<br>88 - Don't know<br>99 - Refused to answer/skipped	as per variable codes	if =1 then must answer DURTN1x, NUMTMESx, and NUMDAYSx	

Ques. No.	Variable Name	Question	Variable Type	Variable Codes	Accepted Values	Skip	Comments
102	MED6	Amodiaquine	Numeric	1 - Yes<br>2 - No<br>88 - Don't know<br>99 - Refused to answer/skipped	as per variable codes	if =1 then must answer DURTN1x, NUMTMESx, and NUMDAYSx	
103	MED7	Quinine	Numeric	1 - Yes<br>2 - No<br>88 - Don't know<br>99 - Refused to answer/skipped	as per variable codes	if =1 then must answer DURTN1x, NUMTMESx, and NUMDAYSx	
104	MED8	Artemether-Lumefantrine (Coartem)	Numeric	1 - Yes<br>2 - No<br>88 - Don't know<br>99 - Refused to answer/skipped	as per variable codes	if =1 then must answer DURTN1x, NUMTMESx, and NUMDAYSx	
105	MED9	Cotrimoxazole(Septrin)	Numeric	1 - Yes<br>2 - No<br>88 - Don't know<br>99 - Refused to answer/skipped	as per variable codes	if =1 then must answer DURTN1x, NUMTMESx, and NUMDAYSx	
106	MED10	Amoxacillin	Numeric	1 - Yes<br>2 - No<br>88 - Don't know<br>99 - Refused to answer/skipped	as per variable codes	if =1 then must answer DURTN1x, NUMTMESx, and NUMDAYSx	
107	MED11	Other	Numeric	1 - Yes<br>2 - No<br>88 - Don't know<br>99 - Refused to answer/skipped	as per variable codes	if =1 then must answer DURTN1x, NUMTMESx, and NUMDAYSx	
108	MED12	Other	Numeric	1 - Yes<br>2 - No<br>88 - Don't know<br>99 - Refused to answer/skipped	as per variable codes	if =1 then must answer DURTN1x, NUMTMESx, and NUMDAYSx	
109	MED13	Other	Numeric	1 - Yes<br>2 - No<br>88 - Don't know<br>99 - Refused to answer/skipped	as per variable codes	if =1 then must answer DURTN1x, NUMTMESx, and NUMDAYSx	
110	MED14	Unknown	Numeric	1 - Yes<br>2 - No<br>88 - Don't know<br>99 - Refused to answer/skipped	as per variable codes	if =1 then must answer DURTN1x, NUMTMESx, and NUMDAYSx	
111	DURTN1	How long had the child been ill when the drug was taken?	Numeric	1 - < 24 hrs<br>2 - 1-3 days<br>3 - 4-7 days<br>4- > 7 days<br>77- other<br>88 - Don't know<br>99 - Refused to answer	as per variable codes		
112	NUMTMES	How many times was the drug given per day?	Numeric	88 - Don't know<br>99 - Refused to answer/skipped	1 - 7, 8,9	Record Number	
113	DURILL	How long did the child's illness last ?	Numeric	1 - < 24 hrs<br>2 - 1-3 days<br>3 - 4-7 days<br>4- > 7 days<br>5- Ongoing at time of interview<br>77- other<br>88 - Don't know<br>99 - Refused to answer	as per variable codes		
114	ILLDLYS	Did you experience anydelays in treating your child's illness?	Numeric	1 - Yes<br>2 - No<br>77- other<br>88 - Don't know<br>99 - Refused to answer	as per variable codes	Record Number	
115	RESDLYS	What were the reasons for the delay?(List all that apply)	Numeric	1 - No transport available<br>2 - Not enough money available<br>3 - Needed to find coverage for work<br>4- Needed to arrange for child care<br>5-Waiting at the health facility<br>6- Was not at home<br>77- other<br>88 - Don't know<br>99 - Refused to answer	as per variable codes	Record Number	
116	HWMCHILL	How much did you spend on the management of this illness?	Numeric(Ushs)	Cost of Drugs.........................<br>Transport...................... ......<br>Fees(Clinic,hospital,lab)......................<br>Other..<br>TOTAL..			
117	RESDELAY	Did caring for your child and managing his/her illness prevent you from doing your usual activities ?	Numeric	1 - Yes<br>2 - No<br>88 - Don't know<br>99 - Refused to answer	as per variable codes		

Ques. No.	Variable Name	Question	Variable Type	Variable Codes	Accepted Values	Skip	Comments
118	HWMCHMISS	If yes, how much time did you miss?	Numeric			Record Number (Hours,days,weeks)	
119	NUMDAYS	For how many days was the drug given ?	Numeric	88 - Don't know<br>99 - Refused to answer	1 - 7, 8,9	Record Number	
120	OTHERACTN1	Specify other ACTIONS taken	String			Record Number of ACTION and repeat questions from FSEEK	
**APPENDIX O: Part 2 - Women's Survey -** *Complete for all women of child-bearing age (13-49 years) within the household*							
**Section 1: Respondent's Background**							
121	WAGEYRS	How old are you?	Numeric	88 - Don't know<br>99 - Refused to answer/skipped	13-49, 88, 99	- if < 13 or > 49, skip PART 2 of Appendix O	
122	WSCHOOL	Have you ever attended school?	Numeric	1 - Yes<br>2 - No<br>88 - Don't know<br>99 - Refused to answer/skipped	as per variable codes		
123	WHIGHEST	What is the highest level of school you attended?	Numeric	0- None 1 - Primary(1-4)<br>2 - primary(5-7)<br>3 - Secoundary (S1-S4)<br>4 - Secoundary(S5-S6)<br>10-Certificate/Diploma<br>11 - University<br>77 - Other ________________________<br>88 - Don't know<br>99 - Refused to answer	as per variable codes		
**Section 2: Birth History -** *Repeat questions in this section for every child birthed to this woman*							
124	TTBTH	How many total births have you had in your lifetime, including children who were born alive but who later died?	Numeric	99 - Refused to answer/skipped	0 - 35,99		
125	DTRBTD	On what day, month and year was your child born, (even if this birth was a child who later died)?	Numeric	88 - Don't know	1 - 31, 88		
126	DTRBTM	....Month	Numeric	88 - Don't know	1 - 12, 88		
127	DTRBTY	....Year	Numeric	8888 - Don't know	1970 - 2011, 8888, 9999		
128	CHAGE	Child age	Numeric				automatically generated
129	CHDLV	Is this child still alive?	Numeric	1 - Yes<br>2 - No<br>9 - Refused to answer/skipped	as per variable codes		
130	CHDDD	If the child died, HOW OLD was the child when s(he) died?	Numeric	INSERT YEARS OR MONTHS		if <> less than 1 month at death, enter 0 years, 0 months	
**Section 3 : Malaria Knowledge**							
131	FEVLG	I would like to ask you a few questions about fever in children.<br><br>When a child is sick with fever, how long after the fever begins should the child be taken for treatment?	Numeric	1 - same day<br>2 - next day<br>3 - two days after onset of fever<br>4 - three or more days after onset of fever<br>5 - fever is normal in children no treatment necessary<br>6 - depends on how serious the fever is<br>77 - other<br>88 - Don't know<br>99 - Refused to answer/skipped	as per variable codes	if = 96, specify other	
132	FEVOT	specify other	String				
133	MALCSA	In your opinion, what causes malaria?<br><br>PROBE: ANYTHING ELSE?<br><br>RECORD ALL MENTIONED<br><br>...Mosquito bites	Numeric	1 - Yes<br>2 - No<br>99 - Refused to answer/skipped	as per variable codes		
134	MALCSB	...Eeating maize	Numeric	1 - Yes<br>2 - No<br>99 - Refused to answer/skipped	as per variable codes		
135	MALCSC	...Eating mangoes	Numeric	1 - Yes<br>2 - No<br>99 - Refused to answer/skipped	as per variable codes		
136	MALCSD	...Eating dirty food	Numeric	1 - Yes<br>2 - No<br>99 - Refused to answer/skipped	as per variable codes		
137	MALCSE	...Drinking unboiled water	Numeric	1 - Yes<br>2 - No<br>99 - Refused to answer/skipped	as per variable codes		

Ques. No.	Variable Name	Question	Variable Type	Variable Codes	Accepted Values	Skip	Comments
138	MALCSF	...Getting soaked with rain	Numeric	1 - Yes<br>2 - No<br>99 - Refused to answer/skipped	as per variable codes		
139	MALCSG	...Cold/changing weather	Numeric	1 - Yes<br>2 - No<br>99 - Refused to answer/skipped	as per variable codes		
140	MALCSH	...Witchcraft	Numeric	1 - Yes<br>2 - No<br>99 - Refused to answer/skipped	as per variable codes		
141	MALCSI	...Contact with infected person	Numeric	1 - Yes<br>2 - No<br>99 - Refused to answer/skipped	as per variable codes		
142	MALCSJ	...Dirty environment or poor hygiene	Numeric	1 - Yes<br>2 - No<br>99 - Refused to answer/skipped	as per variable codes		
143	MALCSK	...Bushes around the home	Numeric	1 - Yes<br>2 - No<br>99 - Refused to answer/skipped	as per variable codes		
144	MALCSL	...Stagnant Water	Numeric	1 - Yes<br>2 - No<br>99 - Refused to answer/skipped	as per variable codes		
145	MALCSM	...Poor feeding	Numeric	1 - Yes<br>2 - No<br>99 - Refused to answer/skipped	as per variable codes		
146	MALCSN	...Eating too much	Numeric	1 - Yes<br>2 - No<br>99 - Refused to answer/skipped	as per variable codes		
147	MALCSO	...Domestic Animals(e.g Cows) around home	Numeric	1 - Yes<br>2 - No<br>99 - Refused to answer/skipped	as per variable codes		
148	MALCSP	...Bedbugs	Numeric	1 - Yes<br>2 - No<br>99 - Refused to answer/skipped	as per variable codes		
149	MALCSQ	...Wounds	Numeric	1 - Yes<br>2 - No<br>99 - Refused to answer/skipped	as per variable codes		
150	MALCSR	...Not sleeping under a net	Numeric	1 - Yes<br>2 - No<br>99 - Refused to answer/skipped	as per variable codes		
151	MALCSS	...Other things cause malaria	Numeric	1 - Yes<br>2 - No<br>99 - Refused to answer/skipped	as per variable codes	if = 1 must answer MLOTHR	
152	MALCST	...Don't Know	Numeric	1 - Yes<br>2 - No<br>99 - Refused to answer/skipped	as per variable codes		
153	MLOTHR	Specify other causes of malaria	String				
154	AVDMAL	Are there ways to avoid getting malaria?	Numeric	1 - Yes<br>2 - No<br>9 - Refused to answer/skipped	as per variable codes	if = 2 or 9 skip to IPTPA	
155	AVMALA	What are the ways to avoid getting malaria?<br><br>PROBE: ANYTHING ELSE?<br><br>RECORD ALL MENTIONED<br><br>....Sleep under mosquito net	Numeric	1 - Yes<br>2 - No<br>99 - Refused to answer/skipped	as per variable codes		
156	AVMALB	...Sleep under an insectcide treated net	Numeric	1 - Yes<br>2 - No<br>99 - Refused to answer/skipped	as per variable codes		
157	AVMALC	...Taking preventive medication	Numeric	1 - Yes<br>2 - No<br>99 - Refused to answer/skipped	as per variable codes		
158	AVMALD	...Use mosquito repellant	Numeric	1 - Yes<br>2 - No<br>99 - Refused to answer/skipped	as per variable codes		
159	AVMALE	...Spraying house with insectcide	Numeric	1 - Yes<br>2 - No<br>99 - Refused to answer/skipped	as per variable codes		
160	AVMALF	...Using mosquito coils	Numeric	1 - Yes<br>2 - No<br>99 - Refused to answer/skipped	as per variable codes		
161	AVMALG	...Destroy mosquito breeding sites	Numeric	1 - Yes<br>2 - No<br>99 - Refused to answer/skipped	as per variable codes		
162	AVMALH	...Boiling/clean water	Numeric	1 - Yes<br>2 - No<br>99 - Refused to answer/skipped	as per variable codes		
163	AVMALI	...Good food preparation	Numeric	1 - Yes<br>2 - No<br>99 - Refused to answer/skipped	as per variable codes		

Ques. No.	Variable Name	Question	Variable Type	Variable Codes	Accepted Values	Skip	Comments
164	AVMALJ	...Clean home/good hygiene	Numeric	1 - Yes<br>2 - No<br>99 - Refused to answer/skipped	as per variable codes		
165	AVMALK	...Cleaning environment around house/clearing bushes	Numeric	1 - Yes<br>2 - No<br>99 - Refused to answer/skipped	as per variable codes		
166	AVMALL	...Closing windows and/or doors early	Numeric	1 - Yes<br>2 - No<br>99 - Refused to answer/skipped	as per variable codes		
167	AVMALM	...Don't know	String	1 - Yes<br>2 - No<br>99 - Refused to answer/skipped	as per variable codes		
168	AVMALN	...Other	Numeric	1 - Yes<br>2 - No<br>99 - Refused to answer/skipped	as per variable codes	if = 1, specify other	
169	SPOTAV	Specify other ways to avoid getting malaria	String				
**Interview Details**							
170	VSTATUS	Result of Visit	Numeric	1 - Completed<br>2 -Incompete/ Pending<br>3 -Return for visit<br>9 - Other	as per variable codes	if = 9, specify other ; if=3 specify dates to return	
171	OVSTATUS	Specify other result	String				
172	TOTVISIT	Total number of visits	Numeric		1 - 3		
173	COMMENTS	Interviewer's Comments	String				

APPENDIX V: COHORT STUDY PICTORIAL DIARIES (1)

[_____]–[_____|_____]–[_____|_____|_____|_____]–[_____|_____]
Household ID

[_____|_____|_____|_____]
Cohort Study ID

[____|____]/[____|____]/[____|____]
Start date (*day / month / year*)

SYMPTOMS	1 Monday Tich achiel	2 Tuesday Tich aryo	3 Wed Tich adek	4 Thursday Tich angwen	5 Friday Tich abich	6 Saturday Mukaaga	7 Sunday Sabiti	1 Monday Tich achiel	2 Tuesday Tich aryo	3 Wed Tich adek	4 Thursday Tich angwen	5 Friday Tich abich	6 Saturday Mukaaga	7 Sunday Sabiti
**DATE**														
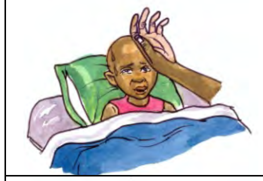														
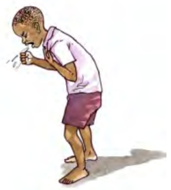														
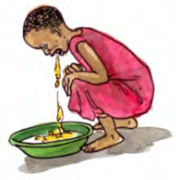														
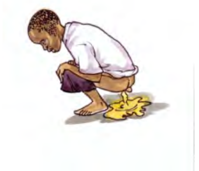														
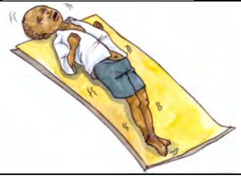														

[_____]–[_____|_____]–[_____|_____|_____|_____]–[_____|_____]
Household ID

[_____|_____|_____|_____]
Cohort Study ID

[____|____]/[____|____]/[____|____]
Start date (*day / month / year*)

EXPENDITURES<br>Health care fees	1<br>Monday<br>Tich achiel	2<br>Tuesday<br>Tich aryo	3<br>Wed<br>Tich adek	4<br>Thursday<br>Tich angwen	5<br>Friday<br>Tich abich	6<br>Saturday<br>Mukaaga	7<br>Sunday<br>Sabiti	1<br>Monday<br>Tich achiel	2<br>Tuesday<br>Tich aryo	3<br>Wed<br>Tich adek	4<br>Thursday<br>Tich angwen	5<br>Friday<br>Tich abich	6<br>Saturday<br>Mukaaga	7<br>Sunday<br>Sabiti
**DATE**														
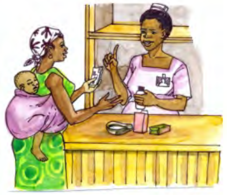														
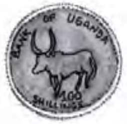 100														
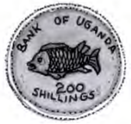 200														
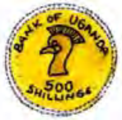 500														
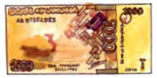 1000														
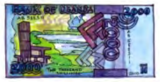 2000														
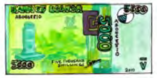 5000														
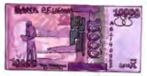 10000														

[____]–[____|____]–[____|____|____|____]–[____|____] **Household ID**

[____|____|____|____] **Cohort Study ID**

[____|____]/[____|____]/[____|____] **Start date (*day / month / year*)**

EXPENDITURES<br>Transport	1<br>Monday<br>Tich achiel	2<br>Tuesday<br>Tich aryo	3<br>Wed<br>Tich adek	4<br>Thursday<br>Tich angwen	5<br>Friday<br>Tich abich	6<br>Saturday<br>Mukaaga	7<br>Sunday<br>Sabiti	1<br>Monday<br>Tich achiel	2<br>Tuesday<br>Tich aryo	3<br>Wed<br>Tich adek	4<br>Thursday<br>Tich angwen	5<br>Friday<br>Tich abich	6<br>Saturday<br>Mukaaga	7<br>Sunday<br>Sabiti
**DATE**														
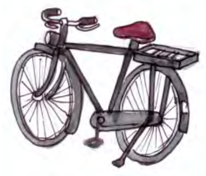	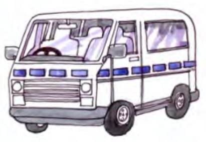		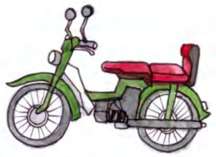											
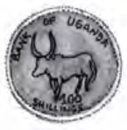 100														
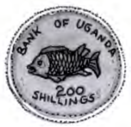 200														
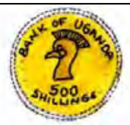 500														
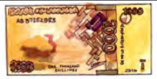 1000														
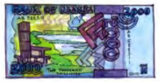 2000														
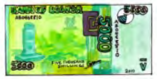 5000														
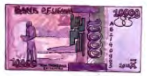 10000														

[____]–[____|____]–[____|____|____|____]–[____|____]
Household ID

[____|____|____|____]
Cohort Study ID

[____|____]/[____|____]/[____|____]
Start date (*day / month / year*)

EXPENDITURES Medications	1 Monday Tich achiel	2 Tuesday Tich aryo	3 Wed Tich adek	4 Thursday Tich angwen	5 Friday Tich abich	6 Saturday Mukaaga	7 Sunday Sabiti	1 Monday Tich achiel	2 Tuesday Tich aryo	3 Wed Tich adek	4 Thursday Tich angwen	5 Friday Tich abich	6 Saturday Mukaaga	7 Sunday Sabiti
**DATE**														
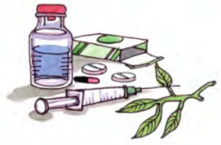														
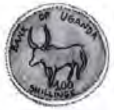 100														
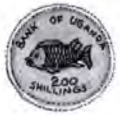 200														
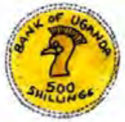 500														
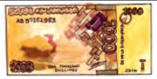 1000														
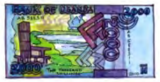 2000														
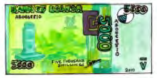 5000														
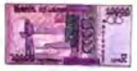 10000														

[____|____]/[____|____]/[____|____]
Stop date (day / month / year)

[____|____]
Staff ID

APPENDIX W: COHORT STUDY MONTHLY QUESTIONNAIRE (1)

Subcounty ID	Village ID	Compound #	Household #	Cluster #
[____|____]	[____|____]	[____|____|____|____]	[____|____]	[____|____]

Study ID	Date of visit	Visit #
[____|____|____|____]	[___|___]/[___|___]/[___|___]<br>day month year	[____|____]

PART 1: EXPERIENCE WITH ILLNESS DURING THE PAST MONTH

**1. "Has your child been sick since our last visit?"**	1 = Yes<br>2 = No	88 = Don't know<br>99 = Refused to answer	[____|____]<br>*If yes, go to #2, otherwise skip to end*
**2. "How many episodes of illness did your child have since our last visit?"**	Insert number	88 = Don't know<br>99= Refused to answer	[____|____]

PART 1: SECTION 1: FIRST ACTION

"Now we would like to get a detailed step by step description of everything you did to care for your child during each illness episode during the past one month. There is no right or wrong answer to these questions. We need you to be as open and honest as possible."

Record illness episode number

[____|____]

**3."Did your child have fever with this episode of illness?"**	1=Yes<br>2= No	88= Don't know<br>99= Refused to answer		[____|____]
**4a. "What did you do FIRST (including tepid sponging and herbs)?"** (choose only one action)	1 = Nothing<br>2 = Tepid sponging<br>3 = Gave herbs kept at home<br>4 = Gave medicines kept at home	5 = Bought medicines from duka<br>6 = Bought medicines at drug shop/pharmacy<br>7 = Took to traditional healer<br>8 = Took to public health centre, clinic or hospital	77 = Other ____________________<br>88 = Don't know<br>99 = Refused to answer	[____|____]<br>*If 8, go to #5, otherwise skip to #9*
**5a. "If you took your child to public health centre, clinic or hospital, where did you go?"**	1 = Public health centre<br>2 = Tororo District Hospital<br>3 = Private hospital/clinic	77 = Other ____________________________<br>88 = Don't know<br>99 = Refused to answer		[____|____]<br>*If 1 or 2, go to #5b, otherwise skip to #9*
**5b. "What is the name of the public health center you went to?"**	1 = Maundo HC II<br>2 = Were HC II<br>3 = Katajula HC II<br>4 = Paya HC III<br>5 = Pusere HC II<br>6 = Nawire HC II<br>7 = Kirewa HC III<br>8 = Chawolo-Kirewa HC II<br>9 = Kisoko HC III<br>10 = Morkiswa HC II	11 = Petta HC III<br>12 = Makawari HC II<br>13 = Mbula HC II<br>14 = Gwaragwara HC II<br>15 = Osia HC II<br>16 = Mwelo HC II<br>17 = Lwala HC II<br>18 = Panyangasi HC III<br>19 = Mudodo HC II<br>20 = Chawolo-Mulanda HC II	77 = Other _________________________<br>88 = Don't know<br>99 = Refused to answer	[____|____]
**6. "Why did you go to the public health centre or public hospital?"**	1 = I heard there were drugs<br>2 = Someone told me to go<br>3 = It's closest to my home<br>4 = It's free (no cost)	5 = I always go to the public health centre<br>6 = They were nice to me last time I went	77 = Other _________________________<br>88 = Don't know<br>99 = Refused to answer	[____|____]
**7. "How satisfied were you with the health care that your child received at the public health centre?"**	1= Very dissatisfied<br>2=Dissatisfied	3= Uncertain<br>4=Satisfied	5=Very satisfied<br>99 = Refused to answer	[____|____]<br>*If 1 or 2 go to # 8, otherwise, skip to #9*
**8. "If dissatisfied or very dissatisfied what were the reasons for being dissatisfied?"** (list all that apply)	1 = Long waiting time<br>2 = No trained professionals<br>3 = No drugs were given<br>4 = No lab tests done<br>5 = Rude HCW	6 = Had to pay for care<br>7 = Treatment was unsuccessful<br>77 = Other ____________________________<br>99 = Refused to answer	[____|____] [____|____] [____|____]	[____|____] [____|____] [____|____]
**9. "How long had the child been ill when this FIRST action was taken?"**	1 =< 24 hrs<br>2 = 1-3 days<br>3 = 4-7 days	4 => 7 days<br>77 = Other ________________	88 = Don't know<br>99 = Refused to answer	[____|____]
Ask this question only if the answer to question #4 was 6 or 8:<br>**4b. "Was your child tested for malaria (finger or heel prick) when you went to this place?"**	1=Yes<br>2= No	88= Don't know<br>99= Refused to answer		[____|____]<br>*If 1, go to #4c, otherwise, skip to #10*
**4c. "If yes, do you know what type of test was done? For example, was the blood placed onto a glass slide or onto a white stick?"**		1 = Blood smear<br>2 = RDT<br>77 = Other<br>88 = Don't know		[____|____]
**4d. "Were you told the result of the test?"** 1 = Yes	2 = No	88 = Don't know	99 = Refused	[____|____]
**4e. "If yes, what was the result of the test?"** 1 = positive for malaria	2 = negative for malaria	77 = Other<br>88 = Don't Know	99 = Refused to Answer	[____|____]

COHORT STUDY MONTHLY QUESTIONNAIRE (2)

Study ID	Date of visit	Visit #
[____|____|____|____]	[___|___]/[___|___]/[___|___]<br>day month year	[____|____]

MEDICINES GIVEN AS FIRST TREATMENT<br>**"If your child took medicine FIRST, what did he/she take?"** *(Indicate all that were given as a first action)*			
**DRUG**	**Number of times given per day**	**Number of days given**	**How long had the child been ill when the drug was started?**<br>1 = < 24 hrs 2 = 1-3 days 3 = 4-7 days 4 => 7 days 77 = Other (list) 88 = Don't know 99 = Refused to answer
**10. Panadol**	[____|____]	[____|____]	[____|____] ________________
**11. Aspirin**	[____|____]	[____|____]	[____|____] ________________
**12. Chloroquine**	[____|____]	[____|____]	[____|____] ________________
**13. Fansidar*(SP)***	[____|____]	[____|____]	[____|____] ________________
**14. CQ+SP**	[____|____]	[____|____]	[____|____] ________________
**15. Amodiaquine**	[____|____]	[____|____]	[____|____] ________________
**16. Quinine**	[____|____]	[____|____]	[____|____] ________________
**17. Coartem**	[____|____]	[____|____]	[____|____] ________________
**18. Septrin*(Bactrim)***	[____|____]	[____|____]	[____|____] ________________
**19. Amoxacillin**	[____|____]	[____|____]	[____|____] ________________
**20. Other**________________	[____|____]	[____|____]	[____|____] ________________
**21. Other**________________	[____|____]	[____|____]	[____|____] ________________
**22. Other**________________	[____|____]	[____|____]	[____|____] ________________
**23. Unknown**	[____|____]	[____|____]	[____|____] ________________

PART 1: SECTION 2: SECOND ACTION

24a. "What did you do SECOND (including tepid sponging and herbs)?" (choose only one action)

1 = Nothing
2 = Tepid sponging
3 = Gave herbs kept at home
4 = Gave medicines kept at home
5 = Bought medicines from duka
6 = Bought medicines at drug shop/pharmacy
7 = Took to traditional healer
8 = Took to public health centre clinic or hospital
77 = Other _____________
88 = Don't know
99 = Refused to answer

[____|____]

If 8, go to Qn #25, otherwise skip to Qn #29

25a. "If you took your child to public health centre, clinic or hospital, where did you go?"

1 = Public health centre
2 = Tororo District Hospital
3 = Private hospital/clinic
77 = Other ______________________
88 = Don't know
99 = Refused to answer

[____|____]

If 1 or 2, go to #26, otherwise skip to #29

25b. "What is the name of the public health center you went to?"

1 = Maundo HC II
2 = Were HC II
3 = Katajula HC II
4 = Paya HC III
5 = Pusere HC II
6 = Nawire HC II
7 = Kirewa HC III
8 = Chawolo-Kirewa HC II
9 = Kisoko HC III
10 = Morkiswa HC II
11 = Petta HC III
12 = Makawari HC II
13 = Mbula HC II
14 = Gwaragwara HC II
15 = Osia HC II
16 = Mwelo HC II
17 = Lwala HC II
18 = Panyangasi HC III
19 = Mudodo HC II
20 = Chawolo-Mulanda HC II
77 = Other ____________________
88 = Don't know
99 = Refused to answer

[____|____]

26. "Why did you go to the public health centre or public hospital?"

1 = I heard there were drugs
2 = Someone told me to go
3 = It's closest to my home
4 = It's free (no cost)
5 = I always go to the public health centre
6 = They were nice to me last time I went
7 = They told me to come back
8 = The drug given last time was unsuccessful
9 = There were no drugs at the health centre the last time I went
77 = Other ______________________
88 = Don't know
99 = Refused to answer

[____|____]

27. "How satisfied were you with the health care that your child received at the public health centre?"

1= Very dissatisfied
2=Dissatisfied
3= Uncertain
4=Satisfied
5=Very satisfied
99 = Refused to answer

[____|____]

If 1 or 2 go to # 28, otherwise, skip to #29

COHORT STUDY MONTHLY QUESTIONNAIRE (3)

Study ID	Date of visit	Visit #
[____|____|____|____]	[___|___]/[___|___]/[___|___]<br>day month year	[____|____]

**28. "If dissatisfied or very dissatisfied what were the reasons for being dissatisfied?"** (list all that apply)	1 = Long waiting time<br>2 = No trained professionals<br>3 = No drugs were given<br>4 = No lab tests done<br>5 = Rude HCW	6 = Had to pay for care<br>7 = Treatment was unsuccessful<br>77 = Other ____________________<br>99 = Refused to answer	[____|____] [____|____]<br>[____|____] [____|____]<br>[____|____] [____|____]
**29. "How long had the child been ill when this SECOND action was taken?"**	1 =< 24 hrs<br>2 = 1-3 days<br>3 = 4-7 days<br>4 => 7 days	77 = Other ____________________<br>88 = Don't know<br>99 = Refused to answer	[____|____]
Ask this question only if the answer to question #24 was 6 or 8:<br>**24b. "Was your child tested for malaria (finger or heel prick) when you went to this place?"**	1 =Yes<br>2 = No	88 = Don't know<br>99 = Refused to answer	[____|____]<br>*If 1, go to #24c, otherwise, skip to #30*
**24c. "If yes, do you know what type of test was done? For example, was the blood placed onto a glass slide or onto a white stick?"**		1 = Blood smear<br>2 = RDT<br>77 = Other<br>88 = Don't know	[____|____]
**24d. "Were you told the result of the test?"**	1 = Yes<br>2 = No	88 = Don't know<br>99 = Refused	[____|____]
**24e. "If yes, what was the result of the test?"**	1 = positive for malaria<br>2 = negative for malaria	77 = Other<br>88 = Don't Know<br>99 = Refused to Answer	[____|____]

MEDICINES GIVEN AS SECOND TREATMENT

"If your child took medicine SECOND, what did he/she take?" *(Indicate all that were given as a first action)*

DRUG	Number of times given per day	Number of days given	How long had the child been ill when the drug was started?<br>1 =< 24 hrs, 2 = 1-3 days, 3 = 4-7 days, 4 => 7 days, 77 = Other (list), 88 = Don't know, 99 = Refused to answer
**30. Panadol**	[____|____]	[____|____]	[____|____] ____________________
**31. Aspirin**	[____|____]	[____|____]	[____|____] ____________________
**32. Chloroquine**	[____|____]	[____|____]	[____|____] ____________________
**33. Fansidar*(SP)***	[____|____]	[____|____]	[____|____] ____________________
**34. CQ+SP**	[____|____]	[____|____]	[____|____] ____________________
**35. Amodiaquine**	[____|____]	[____|____]	[____|____] ____________________
**36. Quinine**	[____|____]	[____|____]	[____|____] ____________________
**37. Coartem**	[____|____]	[____|____]	[____|____] ____________________
**38. Septrin*(Bactrim)***	[____|____]	[____|____]	[____|____] ____________________
**39. Amoxacillin**	[____|____]	[____|____]	[____|____] ____________________
**40. Other____________________**	[____|____]	[____|____]	[____|____] ____________________
**41. Other____________________**	[____|____]	[____|____]	[____|____] ____________________
**42. Other____________________**	[____|____]	[____|____]	[____|____] ____________________
**43. Unknown**	[____|____]	[____|____]	[____|____] ____________________

IF ILLNESS RESOLVED, skip to Part 1: Section 4: Treatment outcome

COHORT STUDY MONTHLY QUESTIONNAIRE (4)

Study ID	Date of visit	Visit #
[____|____|____|____]	[___|___]/[___|___]/[___|___]<br>day month year	[____|____]

PART 1: SECTION 3: THIRD ACTION

44a. "What did you do THIRD (including tepid sponging and herbs)?" (choose only one action)

1 = Nothing
2 = Tepid sponging
3 = Gave herbs kept at home
4 = Gave medicines kept at home
5 = Bought medicines from duka
6 = Bought medicines at drug shop/pharmacy
7 = Took to traditional healer
8 = Took to public health centre, clinic or hospital
77 = Other ______________
88 = Don't know
99 = Refused to answer
[____|____]

If 8, go to #45,otherwise skip to #49

45a. "If you took your child to public health centre, clinic or hospital, where did you go?"

1 = Public health centre
2 = Tororo District Hospital
3 = Private hospital/clinic
77 = Other __________________
88 = Don't know
99 = Refused to answer
[____|____]

If 1 or 2, go to #46, otherwise skip to #49

45b. "What is the name of the public health center you went to?"

1 = Maundo HC II
2 = Were HC II
3 = Katajula HC II
4 = Paya HC III
5 = Pusere HC II
6 = Nawire HC II
7 = Kirewa HC III
8 = Chawolo-Kirewa HC II
9 = Kisoko HC III
10 = Morkiswa HC II
11 = Petta HC III
12 = Makawari HC II
13 = Mbula HC II
14 = Gwaragwara HC II
15 = Osia HC II
16 = Mwelo HC II
17 = Lwala HC II
18 = Panyangasi HC III
19 = Mudodo HC II
20 = Chawolo-Mulanda HC II
77 = Other ______________________
88 = Don't know
99 = Refused to answer
[____|____]

46. "Why did you go to the public health centre or public hospital?"

1 = I heard there were drugs
2 = Someone told me to go
3 = It's closest to my home
4 = It's free (no cost)
5 = I always go to the public health centre
6 = They were nice to me last time
7 = They told me to come back
8 = The drug given last time was unsuccessful
9 = There were no drugs at the health centre the last time I went
77 = Other ______________________
88 = Don't know
99 = Refused to answer
[____|____]

47. "How satisfied were you with the health care that your child received at the public health centre?"

1= Very dissatisfied
2=Dissatisfied
3= Uncertain
4=Satisfied
5=Very satisfied
99 = Refused to answer
[____|____]

If 1 or 2 go to #48. Otherwise, skip to #49

48. "If dissatisfied or very dissatisfied what were the reasons for being dissatisfied?" (list all that apply)

1 = Long waiting time
2 = No trained professionals
3 = No drugs were given
4 = No lab tests done
5 = Rude HCW
6 = Had to pay for care
7 = Treatment was unsuccessful
77 = Other ______________________
99 = Refused to answer
[____|____] [____|____]
[____|____] [____|____]
[____|____] [____|____]

49. "How long had the child been ill when this THIRD action was taken?"

1 =< 24 hrs
2 = 1-3 days
3 = 4-7 days
4 => 7 days
77 = Other ___________
88 = Don't know
99 = Refused to answer
[____|____]

Ask this question only if the answer to question #44 was 6 or 8:

44b. "Was your child tested for malaria (finger or heel prick) when you went to this place?"

1 =Yes
2 = No
88 = Don't know
99 = Refused to answer
[____|____]

If 1, go to #44c, otherwise, skip to #50

44c. "If yes, do you know what type of test was done? For example, was the blood placed onto a glass slide or onto a white stick?"

1 = Blood smear
2 = RDT
77 = Other
88 = Don't know
[____|____]

44d. "Were you told the result of the test?" 1 = Yes 2 = No 88 = Don't know 99 = Refused [____|____]

44e. "If yes, what was the result of the test?" 1 = positive for malaria 2 = negative for malaria 77 = Other 88 = Don't Know 99 = Refused to Answer [____|____]

MEDICINES GIVEN AS THIRD TREATMENT

"If your child took medicine THIRD, what did he/she take?"*(Indicate all that were given as a third action)*

DRUG	Number of times given per day	Number of days given	How long had the child been ill when the drug was started?<br>1 =< 24 hrs 2 = 1-3 days 3 = 4-7 days 4 => 7 days 77 = Other (list) 88 = Don't know 99 = Refused to answer
**50. Panadol**	[____|____]	[____|____]	[____|____] ____________________
**51. Aspirin**	[____|____]	[____|____]	[____|____] ____________________
**52. Chloroquine**	[____|____]	[____|____]	[____|____] ____________________
**53. Fansidar*(SP)***	[____|____]	[____|____]	[____|____] ____________________

COHORT STUDY MONTHLY QUESTIONNAIRE (5)

Study ID	Date of visit	Visit #
[____|____|____|____]	[___|___]/[___|___]/[___|___]<br>day month year	[____|____]

54. CQ+SP	[____|____]	[____|____]	[____|____] ____________________
55. Amodiaquine	[____|____]	[____|____]	[____|____] ____________________
56. Quinine	[____|____]	[____|____]	[____|____] ____________________
57. Coartem	[____|____]	[____|____]	[____|____] ____________________
58. Septrin*(Bactrim)*	[____|____]	[____|____]	[____|____] ____________________
59. Amoxacillin	[____|____]	[____|____]	[____|____] ____________________
60. Other____________________	[____|____]	[____|____]	[____|____] ____________________
61. Other____________________	[____|____]	[____|____]	[____|____] ____________________
62. Other____________________	[____|____]	[____|____]	[____|____] ____________________
63. Unknown	[____|____]	[____|____]	[____|____] ____________________

IF ILLNESS RESOLVED, go to Part 1: Section 4: Treatment outcome, otherwisego to Extra Action form.

PART 1: SECTION 4: TREATMENT OUTCOME

**64. "How long did the child's illness last?"**	1= < 24 hrs<br>2= 1-3 days<br>3= 4-7 days<br>4=>7 days	5= Ongoing at time of interview<br>77 = Other______________________<br>88 = Don't know<br>99= Refused to answer	[____|____]
**65. "Did you experience any delays in treating your child's illness?"**	1 = Yes<br>2 = No	77 = Other ______________________<br>88 = Don't know<br>99 = Refused to answer	[____|____]<br>*If no, skip to question #67*
**66. "What were the reasons for the delays?"** (list all that apply)	1= No transport available<br>2= Not enough money available<br>3 = Needed to find coverage for work	4 = Needed to arrange for child care<br>5= Waiting at the health facility<br>6 = Was not at home<br>77 = Other ________________<br>88= Don't know<br>99 = Refused to answer	[____|____] [____|____]<br>[____|____] [____|____]<br>[____|____] [____|____]
**67. "How much did you spend on management of this illness?"**	Cost of drugs<br>[____|____|____|____|____]Ush<br>Fees (clinic, hospital, lab)<br>[____|____|____|____|____]Ush	Transport<br>[____|____|____|____|____]Ush<br>Other<br>[____|____|____|____|____]Ush	**TOTAL** [____|____|____|____|____|____]**Ush**
**68. "Did caring for your child and managing his/her illness prevent you from doing your usual activities this month?"**	1 = Yes<br>2 = No	88 = Don't know<br>99 = Refused to answer	[____|____]
**69. "If yes, how much time did you miss?"**		If < 1 day, indicate number of hours otherwise, record 00	[____|____]hours<br>[____|____]days

PART 1: SECTION 5: ADVERSE EVENTS

**70. "Did your child have any problems after receiving treatment for this illness?"** *If yes, go to Qn #71, otherwise skip to end*	1 = Yes<br>2 = No	88 = Don't know<br>99 = Refused to answer	[____|____]
**71. "Did your child receive treatment with Coartem (artemether-lumefantrine) for this illness?"**	1 = Yes<br>2 = No	88 = Don't know<br>99 = Refused to answer	[____|____]
**** If any problems occurred after treatment with Coartem, complete an Adverse Drug Reaction Form (Appendix GG) and notify the medical officer and study coordinator***			
**72. "Did any of the following outcomes occur?"†**<br>*(list all that apply)*	1 = Death<br>2 = Life-threatening condition<br>3 = Admitted overnight to clinic or hospital	4 = Required surgery<br>88 = Don't know<br>99 = Refused to answer	[____|____] [____|____]<br>[____|____] [____|____]
† If any of the outcomes occurred, complete an Adverse Drug Reaction Form (Appendix GG) and notify the medical officer and study coordinator			

**Field worker ID**	[____|____]	**IDs of other team members present at interview**	[____|____][____|____][____|____]

If the child experienced an additional episode of illness, go to Part 2 and complete the information for additional episodes.

COHORT STUDY MONTHLY QUESTIONNAIRE (6)

Study ID	Date of visit	Visit #
[____|____|____|____]	[___|___]/[___|___]/[___|___] day month year	[____|____]

PART 2: EXPERIENCE WITH ILLNESS DURING THE PAST MONTH – Extra illness episodes

PART 2: SECTION 1: FIRST ACTION

"Now we would like to get a detailed step by step description of everything you did to care for your child during each illness episode during the past one month. There is no right or wrong answer to these questions. We need you to be as open and honest as possible."

Record illness episode number [____|____]

73."Did your child have fever with this episode of illness?"
1=Yes; 2= No; 88= Don't know; 99= Refused to answer [____|____]

74a. "What did you do FIRST (including tepid sponging and herbs)?" (choose only one action)
1 = Nothing
2 = Tepid sponging
3 = Gave herbs kept at home
4 = Gave medicines kept at home
5 = Bought medicines from duka
6 = Bought medicines at drug shop/pharmacy
7 = Took to traditional healer
8 = Took to public health centre, clinic or hospital
77 = Other ____________________
88 = Don't know
99 = Refused to answer
[____|____]
If 8, go to #75, otherwise skip to #79

75a. "If you took your child to public health centre, clinic or hospital, where did you go?"
1 = Public health centre
2 = Tororo District Hospital
3 = Private hospital/clinic
77 = Other ___________________________
88 = Don't know
99 = Refused to answer
[____|____]
If 1 or 2, go to #76, otherwise skip to #79

75b. "What is the name of the public health center you went to?"
1 = Maundo HC II
2 = Were HC II
3 = Katajula HC II
4 = Paya HC III
5 = Pusere HC II
6 = Nawire HC II
7 = Kirewa HC III
8 = Chawolo-Kirewa HC II
9 = Kisoko HC III
10 = Morkiswa HC II
11 = Petta HC III
12 = Makawari HC II
13 = Mbula HC II
14 = Gwaragwara HC II
15 = Osia HC II
16 = Mwelo HC II
17 = Lwala HC II
18 = Panyangasi HC III
19 = Mudodo HC II
20 = Chawolo-Mulanda HC II
77 = Other _________________________
88 = Don't know
99 = Refused to answer
[____|____]

76. "Why did you go to the public health centre or public hospital?"
1 = I heard there were drugs
2 = Someone told me to go
3 = It's closest to my home
4 = It's free (no cost)
5 = I always go to the public health centre
6 = They were nice to me last time I went
77 = Other _________________________
88 = Don't know
99 = Refused to answer
[____|____]

77. "How satisfied were you with the health care that your child received at the public health centre?"
1= Very dissatisfied
2=Dissatisfied
3= Uncertain
4=Satisfied
5=Very satisfied
99 = Refused to answer
[____|____]
If 1 or 2 go to # 78, otherwise, skip to #79

78. "If dissatisfied or very dissatisfied what were the reasons for being dissatisfied?" (list all that apply)
1 = Long waiting time
2 = No trained professionals
3 = No drugs were given
4 = No lab tests done
5 = Rude HCW
6 = Had to pay for care
7 = Treatment was unsuccessful
77 = Other ____________________________
99 = Refused to answer
[____|____] [____|____]
[____|____] [____|____]
[____|____] [____|____]

79. "How long had the child been ill when this FIRST action was taken?"
1 =< 24 hrs
2 = 1-3 days
3 = 4-7 days
4 => 7 days
77 = Other _____________
88 = Don't know
99 = Refused to answer
[____|____]

Ask this question only if the answer to question #74 was 6 or 8:
74b. "Was your child tested for malaria (finger or heel prick) when you went to this place?"
1 =Yes
2 = No
88 = Don't know
99 = Refused to answer
[____|____]
If 1, go to #74c, otherwise, skip to #80

74c. "If yes, do you know what type of test was done? For example, was the blood placed onto a glass slide or onto a white stick?"
1 = Blood smear
2 = RDT
77 = Other
88 = Don't know
[____|____]

74d. "Were you told the result of the test?" 1 = Yes; 2 = No; 88 = Don't know; 99 = Refused [____|____]

74e. "If yes, what was the result of the test?" 1 = positive for malaria; 2 = negative for malaria; 77 = Other; 88 = Don't Know; 99 = Refused to Answer [____|____]

MEDICINES GIVEN AS FIRST TREATMENT

"If your child took medicine FIRST, what did he/she take?" *(Indicate all that were given as a first action)*

DRUG	Number of times given per day	Number of days given	How long had the child been ill when the drug was started? 1 = < 24 hrs; 2 = 1-3 days; 3 = 4-7 days; 4 => 7 days; 77 = Other (list); 88 = Don't know; 99 = Refused to answer
**80. Panadol**	[____|____]	[____|____]	[____|____] ____________________
**81. Aspirin**	[____|____]	[____|____]	[____|____] ____________________
**82. Chloroquine**	[____|____]	[____|____]	[____|____] ____________________

COHORT STUDY MONTHLY QUESTIONNAIRE (7)

Study ID	Date of visit	Visit #
[_____|_____|_____|_____]	[___|___]/[___|___]/[___|___]<br>day month year	[_____|_____]

**83. Fansidar*(SP)***	[_____|_____]	[_____|_____]	[_____|_____]	________________________
**84. CQ+SP**	[_____|_____]	[_____|_____]	[_____|_____]	________________________
**85. Amodiaquine**	[_____|_____]	[_____|_____]	[_____|_____]	________________________
**86. Quinine**	[_____|_____]	[_____|_____]	[_____|_____]	________________________
**87. Coartem**	[_____|_____]	[_____|_____]	[_____|_____]	________________________
**88. Septrin*(Bactrim)***	[_____|_____]	[_____|_____]	[_____|_____]	________________________
**89. Amoxacillin**	[_____|_____]	[_____|_____]	[_____|_____]	________________________
**90. Other________________________**	[_____|_____]	[_____|_____]	[_____|_____]	________________________
**91. Other________________________**	[_____|_____]	[_____|_____]	[_____|_____]	________________________
**92. Other________________________**	[_____|_____]	[_____|_____]	[_____|_____]	________________________
**93. Unknown**	[_____|_____]	[_____|_____]	[_____|_____]	________________________

IF ILLNESS RESOLVED, skip to Part 2: Section 4: Treatment outcome

PART 2: SECTION 2: SECOND ACTION

94a. "What did you do SECOND (including tepid sponging and herbs)?" (choose only one action)

1 = Nothing
2 = Tepid sponging
3 = Gave herbs kept at home
4 = Gave medicines kept at home
5 = Bought medicines from duka
6 = Bought medicines at drug shop/pharmacy
7 = Took to traditional healer
8 = Took to public health centre, clinic or hospital
77 = Other _______________
88 = Don't know
99 = Refused to answer

[_____|_____]

If 8, go to Qn #95, otherwise skip to #99

95a. "If you took your child to public health centre, clinic or hospital, where did you go?"

1 = Public health centre
2 = Tororo District Hospital
3 = Private hospital/clinic
77 = Other____________________
88 = Don't know
99 = Refused to answer

[_____|_____]

If 1 or 2, go to #96, otherwise skip to #99

95b. "What is the name of the public health center you went to?"

1 = Maundo HC II
2 = Were HC II
3 = Katajula HC II
4 = Paya HC III
5 = Pusere HC II
6 = Nawire HC II
7 = Kirewa HC III
8 = Chawolo-Kirewa HC II
9 = Kisoko HC III
10 = Morkiswa HC II
11 = Petta HC III
12 = Makawari HC II
13 = Mbula HC II
14 = Gwaragwara HC II
15 = Osia HC II
16 = Mwelo HC II
17 = Lwala HC II
18 = Panyangasi HC III
19 = Mudodo HC II
20 = Chawolo-Mulanda HC II
77 = Other _________________________
88 = Don't know
99 = Refused to answer

[_____|_____]

96. "Why did you go to the public health centre or public hospital?"

1 = I heard there were drugs
2 = Someone told me to go
3 = It's closest to my home
4 = It's free (no cost)
5 = I always go to the public health centre
6 = They were nice to me last time I went
7 = They told me to come back
8 = The drug given last time was unsuccessful
9 = There were no drugs at the health centre the last time I went
77 = Other _________________________
88 = Don't know
99 = Refused to answer

[_____|_____]

97. "How satisfied were you with the health care that your child received at the public health centre?"

1= Very dissatisfied
2=Dissatisfied
3= Uncertain
4=Satisfied
5=Very satisfied
99 = Refused to answer

[_____|_____]

If 1 or 2 go to #98, otherwise, skip to #99

98. "If dissatisfied or very dissatisfied what were the reasons for being dissatisfied?" (list all that apply)

1 = Long waiting time
2 = No trained professionals
3 = No drugs were given
4 = No lab tests done
5 = Rude HCW
6 = Had to pay for care
7 = Treatment was unsuccessful
77 = Other ____________________________
99 = Refused to answer

[_____|_____] [_____|_____]
[_____|_____] [_____|_____]
[_____|_____] [_____|_____]

99. "How long had the child been ill when this SECOND action was taken?"

1 =< 24 hrs
2 = 1-3 days
3 = 4-7 days
4 => 7 days
77 = Other ____________________
88 = Don't know
99 = Refused to answer

[_____|_____]

Ask this question only if the answer to question #94 was 6 or 8:

94b. "Was your child tested for malaria (finger or heel prick) when you went to this place?"

1 =Yes
2 = No
88 = Don't know
99 = Refused to answer

[_____|_____]

If 1, go to #94c, otherwise, skip to #100

94c. "If yes, do you know what type of test was done? For example, was the blood placed onto a glass slide or onto a white stick?"

1 = Blood smear
2 = RDT
77 = Other
88 = Don't know

[_____|_____]

COHORT STUDY MONTHLY QUESTIONNAIRE (8)		
Study ID<br>[____|____|____|____]	Date of visit<br>[___|___]/[___|___]/[___|___]<br>day month year	Visit #<br>[____|____]

**94d. "Were you told the result of the test?"**	1 = Yes	2 = No	88 = Don't know	99 = Refused	[____|____]
**94e. "If yes, what was the result of the test?"**	1 = positive for malaria	2 = negative for malaria	77 = Other<br>88 = Don't Know	99 = Refused to Answer	[____|____]

MEDICINES GIVEN AS SECOND TREATMENT
"If your child took medicine SECOND, what did he/she take?"*(Indicate all that were given as a first action)*

DRUG	Number of times given per day	Number of days given	How long had the child been ill when the drug was started?<br>1 =< 24 hrs; 2 = 1-3 days; 3 = 4-7 days; 4 => 7 days; 77 = Other (list); 88 = Don't know; 99 = Refused to answer
**100. Panadol**	[____|____]	[____|____]	[____|____] ____________________
**101. Aspirin**	[____|____]	[____|____]	[____|____] ____________________
**102. Chloroquine**	[____|____]	[____|____]	[____|____] ____________________
**103. Fansidar*(SP)***	[____|____]	[____|____]	[____|____] ____________________
**104. CQ+SP**	[____|____]	[____|____]	[____|____] ____________________
**105. Amodiaquine**	[____|____]	[____|____]	[____|____] ____________________
**106. Quinine**	[____|____]	[____|____]	[____|____] ____________________
**107. Coartem**	[____|____]	[____|____]	[____|____] ____________________
**108. Septrin*(Bactrim)***	[____|____]	[____|____]	[____|____] ____________________
**109. Amoxacillin**	[____|____]	[____|____]	[____|____] ____________________
**110. Other____________________**	[____|____]	[____|____]	[____|____] ____________________
**111. Other____________________**	[____|____]	[____|____]	[____|____] ____________________
**112. Other____________________**	[____|____]	[____|____]	[____|____] ____________________
**113. Unknown**	[____|____]	[____|____]	[____|____] ____________________

IF ILLNESS RESOLVED, skip to Part 2: Section 4: Treatment outcome

PART 2: SECTION 3: THIRD ACTION

114a. "What did you do THIRD (including tepid sponging and herbs)?" (choose only one action)

1 = Nothing
2 = Tepid sponging
3 = Gave herbs kept at home
4 = Gave medicines kept at home
5 = Bought medicines from duka
6 = Bought medicines at drug shop/pharmacy
7 = Took to traditional healer
8 =Took to public health centre,clinic or hospital
77 = Other ______________
88 = Don't know
99 = Refused to answer

[____|____]

If 8, go to #115,otherwise skip to #119

115a. "If you took your child to public health centre, clinic or hospital, where did you go?"

1 = Public health centre
2 = Tororo District Hospital
3 = Private hospital/clinic
77 = Other __________________
88 = Don't know
99 = Refused to answer

[____|___|

If 1 or 2, go to #116, otherwise skip to #119

115b. "What is the name of the public health center you went to?"

1 = Maundo HC II
2 = Were HC II
3 = Katajula HC II
4 = Paya HC III
5 = Pusere HC II
6 = Nawire HC II
7 = Kirewa HC III
8 = Chawolo-Kirewa HC II
9 = Kisoko HC III
10 = Morkiswa HC II
11 = Petta HC III
12 = Makawari HC II
13 = Mbula HC II
14 = Gwaragwara HC II
15 = Osia HC II
16 = Mwelo HC II
17 = Lwala HC II
18 = Panyangasi HC III
19 = Mudodo HC II
20 = Chawolo-Mulanda HC II
77 = Other ________________________
88 = Don't know
99 = Refused to answer

[____|___|

COHORT STUDY MONTHLY QUESTIONNAIRE (9)

Study ID	Date of visit	Visit #
[____|____|____|____]	[___|___]/[___|___]/[___|___]<br>day month year	[____|____]

**116. "Why did you go to the public health centre or public hospital?"**	1 = I heard there were drugs<br>2 = Someone told me to go<br>3 = It's closest to my home<br>4 = It's free (no cost)<br>5 = I always go to the public health centre<br>6 = They were nice to me last time<br>7 = They told me to come back<br>8 = The drug given last time was unsuccessful<br>9 = There were no drugs at the health centre the last time I went<br>77 = Other ___________________<br>88 = Don't know<br>99 = Refused to answer	[____|____]
**117. "How satisfied were you with the health care that your child received at the public health centre?"**	1= Very dissatisfied<br>2=Dissatisfied<br>3= Uncertain<br>4=Satisfied<br>5=Very satisfied<br>99 = Refused to answer<br>*If 1 or 2 go to #118. Otherwise, skip to #119*	[____|____]
**118. "If dissatisfied or very dissatisfied what were the reasons for being dissatisfied?"** (list all that apply)	1 = Long waiting time<br>2 = No trained professionals<br>3 = No drugs were given<br>4 = No lab tests done<br>5 = Rude HCW<br>6 = Had to pay for care<br>7 = Treatment was unsuccessful<br>77 = Other ___________________________<br>99 = Refused to answer	[____|____] [____|____]<br>[____|____] [____|____]<br>[____|____] [____|____]
**119. "How long had the child been ill when this THIRD action was taken?"**	1 =< 24 hrs<br>2 = 1-3 days<br>3 = 4-7 days<br>4 => 7 days<br>77 = Other ____________<br>88 = Don't know<br>99 = Refused to answer	[____|____]
Ask this question only if the answer to question #114 was 6 or 8:<br>**114b. "Was your child tested for malaria (finger or heel prick) when you went to this place?"**	1 =Yes<br>2 = No<br>88 = Don't know<br>99 = Refused to answer<br>*If 1, go to #114c, otherwise, skip to #120*	[____|____]
**114c. "If yes, do you know what type of test was done? For example, was the blood placed onto a glass slide or onto a white stick?"**	1 = Blood smear<br>2 = RDT<br>77 = Other<br>88 = Don't know	[____|____]
**114d. "Were you told the result of the test?"**	1 = Yes<br>2 = No<br>88 = Don't know<br>99 = Refused	[____|____]
**114e. "If yes, what was the result of the test?"**	1 = positive for malaria<br>2 = negative for malaria<br>77 = Other<br>88 = Don't Know<br>99 = Refused to Answer	[____|____]

MEDICINES GIVEN AS THIRD TREATMENT

"If your child took medicine THIRD, what did he/she take?"*(Indicate all that were given as a third action)*

**DRUG**	**Number of times given per day**	**Number of days given**	**How long had the child been ill when the drug was started?**<br>1 =< 24 hrs<br>2 = 1-3 days<br>3 = 4-7 days<br>4 => 7 days<br>77 = Other (list)<br>88 = Don't know<br>99 = Refused to answer
**120. Panadol**	[____|____]	[____|____]	[____|____] ______________________
**121. Aspirin**	[____|____]	[____|____]	[____|____] ______________________
**122. Chloroquine**	[____|____]	[____|____]	[____|____] ______________________
**123. Fansidar*(SP)***	[____|____]	[____|____]	[____|____] ______________________
**124. CQ+SP**	[____|____]	[____|____]	[____|____] ______________________
**125. Amodiaquine**	[____|____]	[____|____]	[____|____] ______________________
**126. Quinine**	[____|____]	[____|____]	[____|____] ______________________
**127. Coartem**	[____|____]	[____|____]	[____|____] ______________________
**128. Septrin*(Bactrim)***	[____|____]	[____|____]	[____|____] ______________________
**129. Amoxacillin**	[____|____]	[____|____]	[____|____] ______________________
**130. Other**______________________	[____|____]	[____|____]	[____|____] ______________________
**131. Other**______________________	[____|____]	[____|____]	[____|____] ______________________
**132. Other**______________________	[____|____]	[____|____]	[____|____] ______________________
**133. Unknown**	[____|____]	[____|____]	[____|____] ______________________

IF ILLNESS RESOLVED, go to Part 1: Section 4: Treatment outcome, otherwise go to Extra Action form.

COHORT STUDY MONTHLY QUESTIONNAIRE (10)

Study ID	Date of visit	Visit #
[____|____|____|____]	[___|___]/[___|___]/[___|___]<br>day month year	[____|____]

PART 2: SECTION 4: TREATMENT OUTCOME

**134. "How long did the child's illness last?"**	1= < 24 hrs<br>2= 1-3 days<br>3= 4-7 days<br>4=>7 days	5= Ongoing at time of interview<br>77 = Other____________________<br>88 = Don't know<br>99= Refused to answer	[____|____]
**135. "Did you experience any delays in treating your child's illness?"**	1 = Yes<br>2 = No	77 = Other ____________________<br>88 = Don't know<br>99 = Refused to answer	[____|____]<br>*If no, skip to question #137*
**136. "What were the reasons for the delays?"** (list all that apply)	1= No transport available<br>2= Not enough money available<br>3 = Needed to find coverage for work	4 = Needed to arrange for child care<br>5= Waiting at the health facility<br>6 = Was not at home<br>77 = Other ____________<br>88= Don't know<br>99 = Refused to answer	[____|____] [____|____]<br>[____|____] [____|____]<br>[____|____] [____|____]
**137. "How much did you spend on management of this illness?"**	Cost of drugs<br>[____|____|____|____|____]Ush<br>Fees (clinic, hospital, lab)<br>[____|____|____|____|____]Ush	Transport<br>[____|____|____|____|____]Ush<br>Other<br>[____|____|____|____|____]Ush	**TOTAL** [____|____|____|____|____|____]**Ush**
**138. "Did caring for your child and managing his/her illness prevent you from doing your usual activities this month?"**	1 = Yes<br>2 = No	88 = Don't know<br>99 = Refused to answer	[____|____]
**139. "If yes, how much time did you miss?"**	If < 1 day, indicate number of hours otherwise, record 00		[____|____]hours<br>[____|____]days

PART 2: SECTION 5: ADVERSE EVENTS

**140. "Did your child have any problems after receiving treatment for this illness?"** *If yes, go to Qn #141, otherwise skip to end*	1 = Yes<br>2 = No	88 = Don't know<br>99 = Refused to answer	[____|____]
**141. "Did your child receive treatment with Coartem (artemether-lumefantrine) for this illness?"**	1 = Yes<br>2 = No	88 = Don't know<br>99 = Refused to answer	[____|____]
**** If any problems occurred after treatment with Coartem, complete an Adverse Drug Reaction Form (Appendix GG) and notify the medical officer and study coordinator***			
**142. "Did any of the following outcomes occur?"†** *(list all that apply)*	1 = Death<br>2 = Life-threatening condition<br>3 = Admitted overnight to clinic or hospital	4 = Required surgery<br>88 = Don't know<br>99 = Refused to answer	[____|____] [____|____]<br>[____|____] [____|____]
† If any of the outcomes occurred, complete an Adverse Drug Reaction Form (Appendix GG) and notify the medical officer and study coordinator			

**Field worker ID**	[____|____]	**IDs of other team members present at interview**	[____|____][____|____][____|____]

APPENDIX W: COHORT STUDY MONTHLY QUESTIONNAIRE (11)

Subcounty ID	Village ID	Compound ID	Household ID	Cluster #	Date of visit
[___]	[___|___]	[___|___|___|___]	[___|___]	[___|___]	[___|___]/ [___|___]/[___|___]<br>day month year

SECTION 8 cont'd: EXTRA ACTION [____]

(Record number of action)

64a. "What did you do NEXT(including tepid sponging and herbs)?" (choose only one action)

1 = Nothing
2 = Tepid sponging
3 = Gave herbs kept at home
4 = Gave medicines kept at home
5 = Bought medicines from duka
6 = Bought medicines at drug shop/pharmacy
7 = Took to traditional healer
8 =Took to public health centre, clinic or hospital
77 = Other _______________
88 = Don't know
99 = Refused to answer

[_____|_____]

If 8, go to #65,otherwise skip to #69

65a. "If you took your child to public health centre, clinic or hospital, where did you go?"

1 = Public health centre
2 = Tororo District Hospital
3 = Private hospital/clinic
77 = Other ____________________
88 = Don't know
99 = Refused to answer

[_____|_____]

If 1 or 2, go to #66, otherwise skip to #69

65b. "What is the name of the public health center you went to?"

1 = Maundo HC II
2 = Were HC II
3 = Katajula HC II
4 = Paya HC III
5 = Pusere HC II
6 = Nawire HC II
7 = Kirewa HC III
8 = Chawolo-Kirewa HC II
9 = Kisoko HC III
10 = Morkiswa HC II
11 = Petta HC III
12 = Makawari HC II
13 = Mbula HC II
14 = Gwaragwara HC II
15 = Osia HC II
16 = Mwelo HC II
17 = Lwala HC II
18 = Panyangasi HC III
19 = Mudodo HC II
20 = Chawolo-Mulanda HC II
77 = Other _________________________
88 = Don't know
99 = Refused to answer

[_____|____]

66. "Why did you go to the public health centre or public hospital?"

1 = I heard there were drugs
2 = Someone told me to go
3 = It's closest to my home
4 = It's free (no cost)
5 = I always go to the public health centre
6 = They were nice to me last time
7 = They told me to come back
8 = The drug given last time was unsuccessful
9 = There were no drugs at the health centre the last time I went
77 = Other ____________________________
88 = Don't know
99 = Refused to answer

[_____|_____]

67. "How satisfied were you with the health care that your child received at the public health centre?"

1= Very dissatisfied
2=Dissatisfied
3= Uncertain
4=Satisfied
5=Very satisfied
99 = Refused to answer

[_____|_____]

If 1 or 2 go to #68. Otherwise, skip to #69

68. "If dissatisfied or very dissatisfied what were the reasons for being dissatisfied?" (list all that apply)

1 = Long waiting time
2 = No trained professionals
3 = No drugs were given
4 = No lab tests done
5 = Rude HCW
6 = Had to pay for care
7 = Treatment was unsuccessful
77 = Other ____________________________
99 = Refused to answer

[_____|_____] [_____|_____]
[_____|_____] [_____|_____]
[_____|_____] [_____|_____]

69. "How long had the child been ill when this NEXT action was taken?"

1 =< 24 hrs
2 = 1-3 days
3 = 4-7 days
4 => 7 days
77 = Other _____________
88 = Don't know
99 = Refused to answer

[_____|_____]

Ask this question on if the answer to question #64 was 6 or 8:

64b. "Was your child tested for malaria (finger or heel prick) when you went to this place?"

1 =Yes
2 = No
88 = Don't know
99 = Refused to answer

[_____|_____]

If 1, go to #24c, otherwise, skip to #30

64c. "If yes, do you know what type of test was done? For example, was the blood placed onto a glass slide or onto a white stick?"

1 = Blood smear
2 = RDT
77 = Other
88 = Don't know

[_____|_____]

64d. "Were you told the result of the test?" 1 = Yes 2 = No 88 = Don't know 99 = Refused [_____|_____]

64e. "If yes, what was the result?" 1 = positive for malaria 2 = negative for malaria 77 = Other 88 = Don't Know 99 = Refused to Answer [_____|_____]

MEDICINES GIVEN AS NEXT TREATMENT

"If your child took medicine THIRD, what did he/she take?" *(Indicate all that were given as a third action)*

DRUG	Number of times given per day	Number of days given	How long had the child been ill when the drug was started?<br>1 =< 24 hrs 2 = 1-3 days 3 = 4-7 days 4 => 7 days 77 = Other (list) 88 = Don't know 99 = Refused to answer
**70. Panadol**	[_____|_____]	[_____|_____]	[_____|_____] ________________________
**71. Aspirin**	[_____|_____]	[_____|_____]	[_____|_____] ________________________
**72. Chloroquine**	[_____|_____]	[_____|_____]	[_____|_____] ________________________
**73. Fansidar *(SP)***	[_____|_____]	[_____|_____]	[_____|_____] ________________________

COHORT STUDY MONTHLY QUESTIONNAIRE (12)		
Study ID<br>[____|____|____|____]	Date of visit<br>[___|___]/[___|___]/[___|___]<br>day month year	Visit #<br>[____|____]

**74. CQ+SP**	[____|____]	[____|____]	[____|____] ______________________
**75. Amodiaquine**	[____|____]	[____|____]	[____|____] ______________________
**76. Quinine**	[____|____]	[____|____]	[____|____] ______________________
**77. Coartem**	[____|____]	[____|____]	[____|____] ______________________
**78. Septrin*(Bactrim)***	[____|____]	[____|____]	[____|____] ______________________
**79. Amoxacillin**	[____|____]	[____|____]	[____|____] ______________________
**80. Other______________________**	[____|____]	[____|____]	[____|____] ______________________
**81. Other______________________**	[____|____]	[____|____]	[____|____] ______________________
**82. Other______________________**	[____|____]	[____|____]	[____|____] ______________________
**83. Unknown**	[____|____]	[____|____]	[____|____] ______________________

IF ILLNESS RESOLVED, , go to Part 1/2: Section 4: Treatment outcome, otherwise go to Extra Actions Form

APPENDIX X: EXIT INTERVIEW SCREENING FORM

PART 1: PARTICIPANT INFORMATION

**Screening Date**<br>[______|______]/[______|______]/[______|______]<br>day month year		**Screening ID**<br>[______|______|______|______]	
**Village name**	____	**Sub-county ID**<br>[_____]	**Village ID**<br>[_____|_____]
**Child's age**	[_____|_____] / [_____|_____]<br>years months	**If child is less than 1 year, complete months, otherwise leave blank**	
**Gender**	[_____]	1 = Male<br>2 = Female	
**Was your child referred to another health centre today?**	[_____]	1 = Yes 88 = Don't know<br>2 = No<br>**If yes, exclude and allow caregiver to leave**	
**If the caregiver is not answering your questions, indicate why**	[_____|_____]	1 = Refused to talk<br>2 = Refused to wait<br>77 = Other: list ______________________________	

SCREENING STAGE 1 – Appropriate age

**Selection criteria**	**Include**	**Exclude**	
Is the child the appropriate age<br>—Under five (aged 0 to less than 5 years)	1 = Yes	2 = No	[_____]
If the answer is '2' from the EXCLUDE column, exclude from the study. If not, proceed to the next section.			

INFORMATION ON STUDY

**Read the following to the caregiver:**	**My name is ___________________________. I work with the Uganda Malaria Surveillance Project which is part of the Infectious Disease Research Collaboration. We would like to ask you some questions about your experience today at this health centre and briefly examine your child. This should take about 30 minutes.**<br>**Would you be able participate now?**			
**Does the caregiver agree to continue?**		**Continue**<br>1 = Yes	**End interview**<br>2 = No	[_____]
**If No, indicate reason**	1 = Refused to talk<br>2 = Refused to wait	77 = Other: list<br>______________________		[_____|_____]
**Proceed with obtaining informed consent**				

SCREENING STAGE 2 – Informed consent

**Selection criteria**	**Include**	**Exclude**	
Is the parent(s)/guardian(s) willing to provide informed consent?	1 = Yes	2 = No	[_____]
If the answer is '2' from the EXCLUDE column, exclude from the study. If not, proceed to the next section.			

**ASSIGN STUDY NUMBER**	[_____|_____|_____|_____]

**All criteria for study inclusion met?**<br>1 = Yes<br>2 = No ***If no, exclude from the study***	[_____]	**Date of enrollment**<br>[______|______]/[______|______]/[______|______]<br>day month year

Staff ID: [____\|____] **Data entrant (1st):** [____\|____] **Data entrant (2nd):** [____\|____]

THE REPUBLIC OF UGANDA

[_____|_____]

Health Facility ID

[_____|_____|_____|_____]

Study ID

APPENDIX Y. PATIENT EXIT INTERVIEWS
Research participant informed consent form

**Protocol Title:**	ACT PRIME Study: Evaluating the impact of enhanced health facility-based care for malaria and febrile illnesses in children
**Site of Research:**	Tororo, Uganda
**Principal Investigators**:	Dr. Sarah Staedke
**Date:**	15 May 2012

Introduction

Dr. Sarah Staedke and colleagues from the Uganda Malaria Surveillance Project / Infectious Diseases Research Collaboration are investigating delivery of healthcare services in Tororo District. We are doing a research study to see if we can improve the health of children in this area by improving services at government-run health facilities.

Why is this survey being done?

Certain health centers in Tororo district will be selected to either take part in the intervention to improve services, or to continue with their current services. Assignment to the two groups has been determined by a lottery. The chance of being placed into either of the groups is the same. To find out how well the intervention is working, we would like to review the health of children under five who go to the health centers for care. We would like to know if caregivers of children are satisfied with their visit to the health centers. We would also like to hear any suggestions on how to improve visits to these health centers. We would like to interview the caregivers of children under five who have visited the health centers. In the final survey, we will interview 50 caregivers from each participating health center, 1000 in total.

What will happen if my child takes part in this survey?

If you agree to let your child participate in this survey, the following will happen today:

a) We will ask you some questions about your visit to the health center today including the purpose of the visit, your child's symptoms, whether a test for malaria was done, and what treatment was given. We will also ask questions about whether you were satisfied with your visit or not.
b) We will collect information on your child's general health.
c) We will briefly examine your child.
d) If your child has had a fever in the last 48 hours (2 days) or has a high temperature, we will do a rapid diagnostic test for malaria.

e) If your child has a positive test for malaria, and appropriate treatment for malaria has not been given, we will provide treatment with artemether-lumefantrine (including Coartem or Lumartem), which is the recommended treatment for simple malaria in Uganda.
f) If your child has any signs of severe malaria or another significant illness, we will refer you and your child back to the health center or to the hospital for further care.
g) The data we collect will be used by project investigators and may be shared with other researchers and policy-makers to answer questions about how best to deliver health services.

After today, we will not ask you to do anything further.

How long will this survey last?

Today, the interview will last about 30 minutes.

Can I stop my child from being in the survey?

You can decide to stop participating at any time. Just tell the project researcher right away if you wish to stop the activities.

What risks can I expect if my child participates in the survey?

We will obtain one blood sample by fingerprick from your child. The risks of drawing blood from a fingerprick include temporary discomfort from the needle stick, bruising, and skin infection. The amount of blood removed will be too small to affect your child's health.

Participation in any research study may involve a loss of privacy. Information you provide about your health center will be recorded, but your name will not be used in any reports of the information provided. The information obtained from these study activities will be locked at our project offices. We will do our best to make sure that any personal information is kept private.

Are there benefits if my child takes part in the survey?

If we find that your child has malaria and has not been given appropriate treatment at the health center, we will give you medications and instructions on how to treat your child. Otherwise, there will be no direct benefit to you from participating in this study. However, the information that you provide will help researchers and policy-makers understand who best to improve health services in this area.

What other choices do I have if my child does not take part in the survey?

You are free to choose not to participate in the study. If you decide not to let your child take part, there will be no penalty to you.

What are the costs of taking part in the survey? Will my child be paid for taking part in the survey?

There are no costs to you for taking part in this study. You and your child will not be paid for taking part in this study.

What are my rights if I allow my child to take part in the survey?

Taking part in this survey is your choice. You may choose either to take part or not to take part. If you decide to take part in this survey, you may change your mind at any time. If you decide to withdraw your child from the survey; your child will still be eligible for care at the local health facility and at Tororo District Hospital and at other local clinics. No matter what decision you take, there will be no penalty to you in any way.

What if my child is injured as result of being in this survey?

If your child is injured, or if you have questions about injuries as a result of being in the survey, please contact Dr. Sarah Staedke or other members of the Uganda Malaria Surveillance Project / Infectious Disease Research Collaboration on telephone number 0414-530692. The sponsoring organizations do not have a program to cover your costs if your child is hurt or has other bad results.

Who can answer my questions about the survey?

You can talk to the researchers about any questions or concerns you have about these survey activities. Contact Dr. Sarah Staedke or other members of the Uganda Malaria Surveillance Project / Infectious Disease Research Collaboration on telephone number 0414-530692. If you have any questions, comments or concerns about taking part in these activities, first talk to the researchers. If for any reason you do not wish to do this, or you still have concerns about doing so, you may contact Professor James Tumwine, Makerere University School of Medicine Research and Ethical Committee at telephone number 0414-530020.

WHAT YOUR SIGNATURE OR THUMBPRINT MEANS

Your signature or thumbprint below means that you understand the information given to you in this consent form about your child's participation in the survey and agree with the following statements:

1. "I have read the consent form concerning this survey (or have understood the verbal explanation of the consent form) and I understand what will be required of me and what will happen to me and my child if we take part in it."

2. "My questions concerning this survey have been answered by Dr. Staedke or the person who signed below."

3. "I understand that at any time, I may withdraw my child from this survey without giving a reason and without affecting my child's normal health care and management."

4. "I agree that the child under my care will take part in this survey."

You will also be asked to sign another informed consent form for the use of stored specimens. If you wish your child to participate in this survey, you should sign or place your thumbprint below.

THE REPUBLIC OF UGANDA

WE WILL GIVE YOU A COPY OF THIS SIGNED AND DATED CONSENT FORM

Name of Participant (printed)

Name of Parent/Guardian

Signature or Fingerprint * of Parent/Guardian | Date/Time

Name of Investigator Administering Consent (printed) | Position/Title

Signature of Investigator Administering Consent | Date/Time

*If the parent or guardian is unable to read and/or write, an impartial witness should be present during the informed consent discussion. After the written informed consent form is read and explained to the participant and parent or guardian, and after they have orally consented to their child's participation in the trial, and have either signed the consent form or provided their fingerprint, the witness should sign and personally date the consent form. By signing the consent form, the witness attests that the information in the consent form and any other written information was accurately explained to, and apparently understood by the parent or guardian, and that informed consent was freely given by the patient and parent or guardian.

Name of Person Witnessing Consent (printed)

Signature of Person Witnessing Consent | Date/Time

APPENDIX Z: PATIENT EXIT INTERVIEW QUESTIONNAIRE (1)

Section 1: Patient/Caregiver information

Health facility ID	Study ID	Date of interview
[_____|_____]	[_____|_____|_____|_____]	[_____|_____]/[_____|_____]/[_____|_____]<br>date month year

Sub-county ID	Village name	Village ID
[_____]	______________________________	[_____|_____|_____]

CHILD	*If child is less than 1 year, complete months, otherwise leave blank*	**Age**<br>[______|______] / [______|______]<br>years months	**Gender**<br>[_____] 1 = Male 2 = Female
CAREGIVER		**Age**<br>[______|______]years	**Gender**<br>[_____] 1 = Male 2 = Female

SECTION 2: CLINICAL HISTORY

**1. What is the reason for your visit to the health center today?** *(Describe below)*			
**2. Did child have fever in the last 48 hours (2 days)?**	1 = Yes<br>2 = No	88 = Don't know<br>99 = Refused	[______|______]
**3. What other problems did the child have?** *(Describe below, and list all appropriate codes at right)*	1 = Cough<br>2 = Flu<br>3 = Not eating<br>4 = Vomiting<br>5 = Diarrhea	6 = Weak (not playing)<br>7 = Convulsions<br>8 = Other<br>77 = None	[______] [______]<br>[______] [______]<br>[______|______]
**4. Did your child have a diagnostic test for malaria done today?**<br>*If no, skip to question 6.*	1 = Yes<br>2 = No	88 = Don't know<br>99 = Refused	[______|______]
**5. If yes, what was the result of the test?**	1 = Positive<br>2 = Negative	88 = Don't know<br>99 = Refused	[______|______]
**6. Were you told what is causing your child's illness?**<br>*If no, skip to question 8.*	1 = Yes<br>2 = No	88 = Don't know<br>99 = Refused	[______|______]
**7. What diagnoses was your child given?**	1 = Malaria<br>2 = Ear infection<br>3 = Throat infection<br>4 = Pneumonia<br>5 = Gastroenteritis<br>6 = Dysentery	7 = Measles<br>8 = Urine infection<br>9 = Other<br>88 = Don't know<br>99 = Refused	[______|______]<br>[______|______]
**8. What treatment was prescribed for your child?**	1 = Panadol<br>2 = AL (Coartem, Lumartem)<br>3 = DP (Duocotecxin)<br>4 = Quinine<br>5 = Chloroquine only<br>6 = SP only (Fansidar)<br>7 = CQ+SP combination<br>8 = Amodiaquine (Camoquin)	11 = Amoxacillin<br>12 = Septrin<br>13 = Iron (Ferrous)<br>14 = ORS<br>15 = Vitamin A<br>20 = Other<br>88 = Don't know<br>99 = Refused	[______|______]<br>[______|______]<br>[______|______]<br>[______|______]
**9. Did you receive the medications that were prescribed?**<br>*If yes, skip to next section.*	1 = Yes<br>2 = No<br>3 = Received some	88 = Don't know<br>99 = Refused	[______|______]
**10. If no, why?**	1 = Drug out of stock<br>2 = Couldn't pay for drug<br>3 = Other	88 = Don't know<br>99 = Refused	[______|______]

PATIENT EXIT INTERVIEW QUESTIONNAIRE (2)

Health facility ID	Study ID	Date of interview
[_____|_____]	[_____|_____|_____|_____]	[_____|_____]/[_____|_____]/[_____|_____]<br>date month year

SECTION 3: SATISFACTION WITH HEALTH CARE

1 = very dissatisfied, 2 = dissatisfied, 3 = uncertain, 4 = satisfied, 5 = very satisfied

1. How satisfied are you with the length of time you had to wait today?	1	2	3	4	5
2. How satisfied are you with the waiting space available?	1	2	3	4	5
3. How satisfied are you with the health center buildings and the consultation rooms?	1	2	3	4	5
4. How satisfied are you with the directions provided at the health center?	1	2	3	4	5
5. How satisfied are you with the attitude of health workers toward you?	1	2	3	4	5
6. How satisfied are you with the consultation and examination of your child?	1	2	3	4	5
7. How satisfied are you with the laboratory tests done?	1	2	3	4	5
8. How satisfied are you with the treatment prescribed?	1	2	3	4	5
9. How satisfied are you with the medications provided?	1	2	3	4	5
10. How satisfied are you with the explanation of why your child is sick?	1	2	3	4	5

SECTION 4: SUGGESTIONS

What do you think can be done to improve services at this health center?

Time of ARRIVAL to health center	Time of DEPARTURE from health center
[_____|_____]:[_____|_____]<br>hours minutes	[_____|_____]:[_____|_____]<br>hours minutes

PATIENT EXIT INTERVIEW QUESTIONNAIRE (3)

Health facility ID	Study ID	Date of interview
[_____|_____]	[_____|_____|_____|_____]	[_____|_____]/[_____|_____]/[_____|_____]<br>date month year

SECTION 3: FEVER EPISODE

Complete ONLY if there is a history of fever or documented temperature (≥ 38.0°C)

1. Clinical Notes – History of present illness

2. Physical examination findings

3. Temperature (^{o}C) [____|____]•[____]

4. PRIME RDT result *If negative, skip to Qn 6.*
0 = Negative
1 = Positive (Pf only)
2 = Positive (Pan only)
3 = Positive (Pf + Pan)
4 = Invalid
77 = Other
[_____|_____]

5. If the RDT is positive, was the child prescribed appropriate treatment for malaria at the health center?
1 = Yes
2 = No
[_____]

6. Diagnosis made by PRIME
1 = Uncomplicated malaria
2 = Severe malaria
3 = Otitis media
4 = Pharyngitis
5 = Upper respiratory tract infection
6 = Pneumonia
7 = Gastroenteritis
8 = Diarrhea
9 = Dysentery
10 = Urinary tract infection
11 = Skin infection
12 = Viral illness
77 = Other
88 = Unknown
[______|______]

7. Medications prescribed by PRIME
1 = Panadol
2 = Coartem (artemether-lumefantrine)
12 = Other (list)
____
____
____

Drug 1 [_____|_____]
Drug 2 [_____|_____]
Drug 3 [_____|_____]
Drug 4 [_____|_____]
Drug 5 [_____|_____]

8. Was the child referred for additional care by PRIME staff?
1 = Yes
2 = No
[_____]

9. If yes, where? (specify)

Staff ID: [_____|_____] **Data entrant (1st):** [_____|_____] **Data entrant (2nd):** [_____|_____]

THE REPUBLIC OF UGANDA

APPENDIX AA. INFORMATION SHEET – Health facility surveillance ACT PRIME Study

Introduction

Dr. Sarah Staedke and colleagues from the Uganda Malaria Surveillance Project / Infectious Diseases Research Collaboration are investigating delivery of health care services in Tororo District. We are doing a research study to see if we can improve the health of children in this area by improving services at government-run health facilities. Certain health centers in Tororo district will be selected to participate in the intervention to improve services, or to continue with their current services. Assignment to the two groups has been determined by a lottery. The chance of being placed into either of the groups is the same.

Why is this surveillance being done?

As part of this study, we would like to know more about the types of patients seen, and the treatment provided, at lower-level health centers (HC IIs and HC IIIs) is this area. We would also like to know more about drug supplies, staffing and costs at HC IIs and IIIs.

What will happen if I take part in this surveillance?

We will visit your health center regularly, approximately every 1-3 months. We will ask you, or another available health worker, to provide us with your registers, including the patient, drug, and laboratory (if applicable) registers, drug stock cards, and drug stock book. We will review the registers and stock cards and will record the data. Depending on the frequency of our visits, we estimate that it will take 1-3 days to enter the data. We will also ask you some questions about any situation that had an impact on patient care at your health center this month, which should last about 15 minutes. During one visit we will also ask you questions about routine costs at this health center, which should last about 30-60 minutes. The data we collect will be used by project investigators and may be shared with other researchers and policy-makers to answer questions about how best to deliver health services.

How long will this surveillance last?

We plan to conduct the surveillance over 2 years. We will visit your health facility about once every 1-3 months for a total of about 2 years.

Can I stop being in the surveillance?

You can decide to stop participating at any time. Just tell our study personnel right away if you wish to stop the activities.

What risks can I expect from participating in the surveillance?

Participation in any research study may involve a loss of privacy. Information you provide about your health center will be recorded, but your name will not be used in any reports of the information provided. No

quotes or other results arising from your participation in this study will be included in any reports, even anonymously, without your agreement. The information obtained from these surveillance activities will be locked at our project offices. We will do our best to make sure that any personal information is kept private.

Are there benefits to taking part in the surveillance?

There will be no direct benefit to you from participating in this study. However, the information that you provide will help researchers and policy-makers understand who best to improve health services in this area.

What other choices do I have if I do not take part in the surveillance?

You are free to choose not to participate in the study. If you decide not to take part, there will be no penalty to you.

What are the costs of taking part in the surveillance? Will I be paid for taking part in the surveillance?

There are no costs to you for taking part in this study. You will not be paid for taking part in this study.
To recognize your role in supporting our surveillance activities, we will periodically provide tea, coffee, sugar, milk, soap, or pens to this health facility.

What are my rights if I take part in the surveillance?

Taking part in this study is your choice. You may choose either to take part or not to take part. If you decide to take part in this study, you may change your mind at any time. No matter what decision you take, there will be no penalty to you in any way.

Who can answer my questions about the surveillance?

You can talk to the researchers about any questions or concerns you have about these surveillance activities. Contact Dr. Sarah Staedke or other members of the Uganda Malaria Surveillance Project / Infectious Disease Research Collaboration on telephone number 0414-530692. If you have any questions, comments or concerns about taking part in these activities, first talk to the researchers. If for any reason you do not wish to do this, or you still have concerns about doing so, you may contact Dr James Tumwine, Makerere University Faculty of Medicine Research and Ethical Committee at telephone number 0414-530020.

Giving verbal consent to participate in the surveillance:

You may keep this information sheet if you wish. Participation in these activities is voluntary. You have the right to decline to participate in the activities, or to withdraw from them at any point without penalty. If you do not wish to participate in the activities, you should inform the researcher now. If you do wish to participate in these activities, you should tell the researcher now, and the interview will begin shortly. If you do not agree to quotes or other results arising from your participation in the study being included, even anonymously, in any reports about the study, please tell the researcher now.

APPENDIX BB: HEALTH FACILITY SURVEILLANCE FORM (1)

PART 1: Section 1: Health Center Information

Health centre code	Health worker ID	Date Report Completed
[_____|_____]	[_____|_____]	[_____|_____]/[_____|_____]/[_____|_____]<br>day month year

START date of report<br>(this should be the STOP date from the previous months' report)	STOP date of report
[_____|_____]/[_____|_____]/[_____|_____]<br>day month year	[_____|_____]/[_____|_____]/[_____|_____]<br>day month year

Section 2: Review of Record Books

Assign each patient in the Patient Register a Patient Number and record into the PDA as much information as possible on each patient who attended the health facility since the date of the last visit using the Patient Register, Laboratory Log Book, Drug Stock Cards, and Dispensing Record or Drug Stock Book.

From Patient Register		From Laboratory Log Book	From Dispensing Record
1. Date of visit	6. Diagnosis	(if health facility has a laboratory)	1. Drug dispensed
2. Age of patient	7. Treatment prescribed	1. RDT performed	2. Amount
3. Gender of patient	8. RDT performed	2. Result of RDT	
4. Village of residence	9. Result of RDT	3. Laboratory tests performed	
5. Fever or history of fever		4. Result of test	

Section 3: Drug Stock Report

Complete using Drug Stock Cards, Dispensing Record, and/or Drug Stock Book.

1. Was AL from NMS out-of-stock this month? 1 = Yes; 2 = No; 88 = Don't know; 99 = Refused to answer [_____|_____]

If No, skip to question 9

	**2. List date when stock-out of AL began:**	**3. Was AL re-stocked after this stock-out?**<br>1 = Yes<br>2 = No<br>88 = Don't know<br>99 = Refused to answer<br>*If No, skip to question 9*	**4. If yes, who re-stocked your drugs?**<br>1 = NMS<br>2 = ACT PRIME study (us)<br>3 = Used PHC or HF funds. List amount<br>77 = Other: list<br>88 = Don't know<br>99 = Refused to answer	**5. What date was AL re-stocked?**
1st	2a.<br>[__ ___/__ ___/___ __]<br>day month year	3a.<br>[_____|_____]	4a. [_____|_____]<br>amount: UGX____________________<br>Other: list:______________________	5a.<br>[__ ___/__ ___/___ __]<br>day month year
2nd	2b.<br>[__ ___/__ ___/___ __]<br>day month year	3b.<br>[_____|_____]	4b. [_____|_____]<br>amount: UGX____________________<br>Other: list:______________________	5b.<br>[__ ___/__ ___/___ __]<br>day month year
3rd	2c.<br>[__ ___/__ ___/___ __]<br>day month year	3c.<br>[_____|_____]	4c. [_____|_____]<br>amount: UGX____________________<br>Other: list:______________________	5c.<br>[__ ___/__ ___/___ __]<br>day month year

**9. If your supply of AL ran low, did you ration or keep some drugs aside?**	**10. If yes, for what reasons did you ration or keep some drugs aside? List all that apply**
[_____]	[_____|_____] [_____|_____] [_____|_____] [_____|_____]
1 = Yes<br>2 = No<br>*If no, skip to next section*	1 = To have drugs to treat children<br>2 = To have drugs to treat severe cases of malaria<br>3 = To have drugs available if a politician visits<br>77 = Other, ______________________________

HEALTH FACILITY SURVEILLANCE FORM (2)		
**Health centre code**<br>[______|______]	**Health worker ID**<br>[______|______]	**Date Report Completed**<br>[______|______]/[______|______]/[______|______]<br>day month year
**START date of report**<br>(this should be the STOP date from the previous months' report)<br>[______|______]/[______|______]/[______|______]<br>day month year		**STOP date of report**<br>[______|______]/[______|______]/[______|______]<br>day month year

Section 5: Extra comments
Describe any situation that had an impact on patient care at your health center this month, such as drug stock-outs, staff shortages, or political instability.

HEALTH FACILITY SURVEILLANCE FORM – HEALTH CENTRE INDICATOR REPORTS (3)

PART 2: Section 1: Health Center Information

Health centre code	Health worker ID	Date Report Completed
[_____|_____]	[_____|_____]	[_____|_____]/[_____|_____]/[_____|_____]<br>day month year

START date of report<br>(this should be the STOP date from the previous months' report)	STOP date of report
[_____|_____]/[_____|_____]/[_____|_____]<br>day month year	[_____|_____]/[_____|_____]/[_____|_____]<br>day month year

Section 2: Indicator report – ALL PATIENTS

Total number of patients visiting the clinic this month	[_____|_____|_____]	
Number (proportion) of patients who had fever	[_____|_____|_____]	[_____|_____] %
Number (proportion) of febrile patients who had a RDT done	[_____|_____|_____]	[_____|_____] %
Number (proportion) of patients who had a positive RDT result	[_____|_____|_____]	[_____|_____] %
Number (proportion) of patients who had a negative RDT result	[_____|_____|_____]	[_____|_____] %
Total number of patients who were diagnosed with malaria	[_____|_____|_____]	
Number (proportion) of patients diagnosed with malaria who had a positive RDT	[_____|_____|_____]	[_____|_____] %
Number (proportion) of patients diagnosed with malaria who had a negative RDT	[_____|_____|_____]	[_____|_____] %
Total number of patients who were prescribed an ACT* to treat malaria	[_____|_____|_____]	
Number (proportion) of patients who had a positive RDT result who were prescribed an ACT*	[_____|_____|_____]	[_____|_____] %
Number (proportion) of patients who had a negative RDT result who were prescribed an ACT*	[_____|_____|_____]	[_____|_____] %
Number (proportion) of patients without an RDT done who were prescribed an ACT*	[_____|_____|_____]	[_____|_____] %

*** ACT = artemisinin-based combination therapy for malaria. Examples include Coartem, Lumartem, Duocotexcin**

HEALTH FACILITY SURVEILLANCE FORM – INDICATOR REPORTS (4)		
**Health centre code**<br>[_____|_____]	**Health worker ID**<br>[_____|_____]	**Date Report Completed**<br>[_____|_____]/[_____|_____]/[_____|_____]<br>day month year
**START date of report**<br>[_____|_____]/[_____|_____]/[_____|_____]<br>**day month year**		**STOP date of report**<br>[_____|_____]/[_____|_____]/[_____|_____]<br>**day month year**

Section 3: Indicator report – CHILDREN UNDER FIVE		
Total number of children under five visiting the clinic this month	[_____|_____|_____]	
Number (proportion) of children under five who had fever	[_____|_____|_____]	[_____|_____] %
Number (proportion) of febrile children under five who had a RDT done	[_____|_____|_____]	[_____|_____] %
Number (proportion) of children under five who had a positive RDT result	[_____|_____|_____]	[_____|_____] %
Number (proportion) of children under five who had a negative RDT result	[_____|_____|_____]	[_____|_____] %
Total number of children under five who were diagnosed with malaria	[_____|_____|_____]	
Number (proportion) of children under five diagnosed with malaria who had a positive RDT	[_____|_____|_____]	[_____|_____] %
Number (proportion) of children under five diagnosed with malaria who had a negative RDT	[_____|_____|_____]	[_____|_____] %
Total number of children under five who were prescribed an ACT* to treat malaria	[_____|_____|_____]	
Number (proportion) of children under five who had a positive RDT result who were prescribed an ACT*	[_____|_____|_____]	[_____|_____] %
Number (proportion) of children under five who had a negative RDT result who were prescribed an ACT*	[_____|_____|_____]	[_____|_____] %
Number (proportion) of children under five without an RDT done who were prescribed an ACT*	[_____|_____|_____]	[_____|_____] %
*** ACT = artemisinin-based combination therapy for malaria. Examples include Coartem, Lumartem, Duocotexcin**		

HEALTH FACILITY SURVEILLANCE FORM – COST COLLECTION (1)

PART 3: Section 1: Health Center Information

This section to be completed in all health facilities at baseline and then annually (3 surveys in total)

Health centre code	Health worker ID	Date Report Completed
[_____|_____]	[_____|_____]	[_____|_____]/[_____|_____]/[_____|_____]<br>day month year

Section 2: DRUGS & SUPPLIES

#	Drugs	Ever stocked?<br>1 = Yes<br>2 = No<br>88 = Don't know<br>99 = Refused to answer	Total number of units available on day of survey	If 0 units available, how many days has this item been out of stock?	Source of payment for item<br>1 = Us, ACT PRIME study<br>2 = NGO/project<br>3 = PHC Fund<br>4 = Government<br>77 = Other, list<br>88 = Don't know<br>99 = Refused to answer
1.	Artemether-Lumefantrine – Coartem	[_____|_____]		days	[_____|_____] ____________________
2.	Artemether-Lumefantrine – Lumartem	[_____|_____]		days	[_____|_____] ____________________
3.	Amodiaquine-Artesunate	[_____|_____]		days	[_____|_____] ____________________
4.	Sulfadoxine-Pyrimethamine (Fansidar)	[_____|_____]		days	[_____|_____] ____________________
5.	Chloroquine 150 base tab	[_____|_____]		days	[_____|_____] ____________________
6.	Chloroquine 50mg base/5 Bottle 1000ml Syrup	[_____|_____]		days	[_____|_____] ____________________
7.	Chloroquine 50mg base/5 Bottle 1000ml Syrup	[_____|_____]		days	[_____|_____] ____________________
8.	Chloroquine inj 40 mg/ml; 39ml	[_____|_____]		days	[_____|_____] ____________________
9.	Acetylsalicylic acid 300mg tab	[_____|_____]		days	[_____|_____] ____________________
10.	Diazepam inj 5mg/ml	[_____|_____]		days	[_____|_____] ____________________
11.	Ferrous sulphate tab. 200 + 0.25mg	[_____|_____]		days	[_____|_____] ____________________
12.	Folic Acid 5mg	[_____|_____]		days	[_____|_____] ____________________

HEALTH FACILITY SURVEILLANCE FORM – COST COLLECTION (2)

Health centre code	Health worker ID	Date Report Completed
[_____|_____]	[_____|_____]	[_____|_____]/[_____|_____]/[_____|_____]<br>day month year

#	DRUGS	Ever stocked?<br>1 = Yes<br>2 = No<br>88 = Don't know<br>99 = Refused to answer	Total number of units available on day of survey	If 0 units available, how many days has this item been out of stock?	Source of payment for item<br>1 = Us, ACT PRIME study<br>2 = NGO/project<br>3 = PHC Fund<br>4 = Government<br>77 = Other, list<br>88 = Don't know<br>99 = Refused to answer
13.	Oral Rehydration Salts	[_____|_____]		days	[_____|_____] ______________________
14.	Paracetamol 120/5ml syrup	[_____|_____]		days	[_____|_____] ______________________
15.	Paracetamol tab 500 mg	[_____|_____]		days	[_____|_____] ______________________
16.	Quinine inj 300mg/ml	[_____|_____]		days	[_____|_____] ______________________
17.	Quinine tab 300mg	[_____|_____]		days	[_____|_____] ______________________
18.	Dextrose 5%	[_____|_____]		days	[_____|_____] ______________________
19.	Dextrose 50%	[_____|_____]		days	[_____|_____] ______________________
20.	Water for injection 10m	[_____|_____]		days	[_____|_____] ______________________
21.	Other ______________________________	[_____|_____]		days	[_____|_____] ______________________
22.	Other ______________________________	[_____|_____]		days	[_____|_____] ______________________
23.	Other ______________________________	[_____|_____]		days	[_____|_____] ______________________
24.	Other ______________________________	[_____|_____]		days	[_____|_____] ______________________
25.	Other ______________________________	[_____|_____]		days	[_____|_____] ______________________

HEALTH FACILITY SURVEILLANCE FORM – COST COLLECTION (3)		
**Health centre code** [_____|_____]	**Health worker ID** [_____|_____]	**Date Report Completed** [_____|_____]/[_____|_____]/[_____|_____] day month year

#	SUPPLIES	Ever stocked? 1 = Yes 2 = No 88 = Don't know 99 = Refused to answer	Total number of units available on day of survey	If 0 units available, how many days has this item been out of stock?	Source of payment for item 1 = Us, ACT PRIME study 2 = NGO/project 3 = PHC Fund 4 = Government 77 = Other, list 88 = Don't know 99 = Refused to answer
26.	Syringe for needles	[_____|_____]		days	[_____|_____] ________________________
27.	Cotton Wool	[_____|_____]		days	[_____|_____] ________________________
28.	Plaster wound adhesive	[_____|_____]		days	[_____|_____] ________________________
29.	Surgical gloves	[_____|_____]		days	[_____|_____] ________________________
30.	Giving set	[_____|_____]		days	[_____|_____] ________________________
31.	Cannula	[_____|_____]		days	[_____|_____] ________________________
32.	Needle half circle 16 triangular	[_____|_____]		days	[_____|_____] ________________________
33.	Other ___________________________________	[_____|_____]		days	[_____|_____] ________________________
34.	Other ___________________________________	[_____|_____]		days	[_____|_____] ________________________
35.	Other ___________________________________	[_____|_____]		days	[_____|_____] ________________________
36.	Other ___________________________________	[_____|_____]		days	[_____|_____] ________________________
37.	Other ___________________________________	[_____|_____]		days	[_____|_____] ________________________
38.	Other ___________________________________	[_____|_____]		days	[_____|_____] ________________________
39.	Other ___________________________________	[_____|_____]		days	[_____|_____] ________________________
40.	Other ___________________________________	[_____|_____]		days	[_____|_____] ________________________

HEALTH FACILITY SURVEILLANCE FORM – COST COLLECTION (4)

Health centre code	Health worker ID	Date Report Completed
[_____|_____]	[_____|_____]	[_____|_____]/[_____|_____]/[_____|_____]<br>day month year

Section 3: OVERHEADS

	UTILITIES	Does this health facility have this utility?<br>1 = Yes 2 = No<br>88 = Don't know<br>99 = Refused to answer	Was this utility paid for this year?<br>1 = Yes<br>2 = No<br>88 = Don't know<br>99 = Refused to answer	Average price per month, in UGX	Average total price per year, in UGX	Source of payment for utility<br>1 = NGO<br>2 = PHC Fund<br>3 = Government	77 = Other, list<br>88 = Don't know<br>99 = Refused to answer
41.	Gas	[___|___]	[___|___]	UGX	UGX	[___|___]	______________________________
42.	Electricity	[___|___]	[___|___]	UGX	UGX	[___|___]	______________________________
43.	Water	[___|___]	[___|___]	UGX	UGX	[___|___]	______________________________
44.	Other ____________________	[___|___]	[___|___]	UGX	UGX	[___|___]	______________________________
45.	Other ____________________	[___|___]	[___|___]	UGX	UGX	[___|___]	______________________________

HEALTH FACILITY SURVEILLANCE FORM – COST COLLECTION (5)

Health centre code	Health worker ID	Date Report Completed
[_____|_____]	[_____|_____]	[_____|_____]/[_____|_____]/[_____|_____]<br>day month year

	**CAPITAL EQUIPMENT**<br>Vehicles, furniture, generator,	**Description**<br>Make/model	**# of units available**	**Date purchased**<br>[Day/month/year]	**Cost, in UGX**	**Source of payment for item**<br>1 = NGO<br>2 = PHC Fund<br>3 = Government, list department<br>77 = Other, list<br>88 = Don't know<br>99 = Refused to answer	**If this item is used in the treatment of malaria, indicate the % of time in a week the item is used to treat malaria.**
46.				[__ __/__ __/__ __]	UGX	[___|___] ________________	%
47.				[__ __/__ __/__ __]	UGX	[___|___] ________________	%
48.				[__ __/__ __/__ __]	UGX	[___|___] ________________	%
49.				[__ __/__ __/__ __]	UGX	[___|___] ________________	%
50.				[__ __/__ __/__ __]	UGX	[___|___] ________________	%
51.				[__ __/__ __/__ __]	UGX	[___|___] ________________	%
52.				[__ __/__ __/__ __]	UGX	[___|___] ________________	%
53.				[__ __/__ __/__ __]	UGX	[___|___] ________________	%
54.				[__ __/__ __/__ __]	UGX	[___|___] ________________	%
55.				[__ __/__ __/__ __]	UGX	[___|___] ________________	%
56.				[__ __/__ __/__ __]	UGX	[___|___] ________________	%
57.				[__ __/__ __/__ __]	UGX	[___|___] ________________	%
58.				[__ __/__ __/__ __]	UGX	[___|___] ________________	%
59.				[__ __/__ __/__ __]	UGX	[___|___] ________________	%
60.				[__ __/__ __/__ __]	UGX	[___|___] ________________	%

HEALTH FACILITY SURVEILLANCE FORM – COST COLLECTION (6)

Health centre code	Health worker ID	Date Report Completed
[_____|_____]	[_____|_____]	[_____|_____]/[_____|_____]/[_____|_____]<br>day month year

	SPACE - Description<br>1 = waiting area<br>2 = registration<br>3 = examination room<br>4 = dispensing room<br>5 = laboratory<br>6 = kitchen<br>7 = laundry<br>8 = pharmacy<br>9 = storage<br>10 = corridor<br>11 = not in use<br>77 = other, list	Size, in square meters	Price per square meter, in UGX	Source of payment for space<br>1 = NGO<br>2 = PHC Fund<br>3 = Government, list department<br>77 = Other, list<br>88 = Don't know<br>99 = Refused to answer	If this space is used in the treatment of malaria, indicate the % of time in a week this space is used to treat malaria.
61.	[____|____] ______________________	m^2	UGX/m^2	[____|____] ______________________	%
62.	[____|____] ______________________	m^2	UGX/m^2	[____|____] ______________________	%
63.	[____|____] ______________________	m^2	UGX/m^2	[____|____] ______________________	%
64.	[____|____] ______________________	m^2	UGX/m^2	[____|____] ______________________	%
65.	[____|____] ______________________	m^2	UGX/m^2	[____|____] ______________________	%
66.	[____|____] ______________________	m^2	UGX/m^2	[____|____] ______________________	%
67.	[____|____] ______________________	m^2	UGX/m^2	[____|____] ______________________	%
68.	[____|____] ______________________	m^2	UGX/m^2	[____|____] ______________________	%
69.	[____|____] ______________________	m^2	UGX/m^2	[____|____] ______________________	%
70.	[____|____] ______________________	m^2	UGX/m^2	[____|____] ______________________	%

HEALTH FACILITY SURVEILLANCE FORM – COST COLLECTION (7)

Health centre code	Health worker ID	Date Report Completed
[_____|_____]	[_____|_____]	[_____|_____]/[_____|_____]/[_____|_____]<br>day month year

STAFF ALLOCATION & ACTIVITIES

List all of the staff people present at the health facility in an average week and then percentage of time in an average week they spend on each activity. Indicate 0, if no time is spent on an activity by a staff person. Repeat for each staff person.

1 = In-charge
2 = Senior medical officer
3 = Medical officer
4 = Senior clinical officer
5 = Clinical officer
6 = Nursing officer
7 = Enrolled nurse
8 = Midwife
9 = Public health nurse
10 = Nursing aide/assistant
11 = Laboratory technician
12 = Laboratory assistant
13 = Health assistant
14 = Health educator
15 = Other______________

	Staff person 1: [___|___]	Staff person 2: [___|___]	Staff person 3: [___|___]	Staff person 4: [___|___]	Staff person 5: [___|___]	Staff person 6: [___|___]	Staff person 7: [___|___]	Staff person 8: [___|___]
**Overhead activities**								
Administration	%	%	%	%	%	%	%	%
Accounting	%	%	%	%	%	%	%	%
Compiling health information	%	%	%	%	%	%	%	%
Cleaning health centre	%	%	%	%	%	%	%	%
Laundry	%	%	%	%	%	%	%	%
Other ____________________	%	%	%	%	%	%	%	%
Other ____________________	%	%	%	%	%	%	%	%
**Support activities**								
Dispensary activities	%	%	%	%	%	%	%	%
Diagnostic services	%	%	%	%	%	%	%	%
Other ____________________	%	%	%	%	%	%	%	%
Other ____________________	%	%	%	%	%	%	%	%
**Service activities**								
Out-patient - Malaria	%	%	%	%	%	%	%	%
Out-patient - Other illnesses	%	%	%	%	%	%	%	%
Antenatal visits	%	%	%	%	%	%	%	%
Postnatal care	%	%	%	%	%	%	%	%
Vaccination	%	%	%	%	%	%	%	%
Family planning	%	%	%	%	%	%	%	%
Maternity care	%	%	%	%	%	%	%	%
Other ____________________	%	%	%	%	%	%	%	%
Other ____________________	%	%	%	%	%	%	%	%

THE REPUBLIC OF UGANDA

APPENDIX CC. INFORMATION SHEET
Health worker knowledge questionnaire
ACT PRIME Study

Introduction

Dr. Sarah Staedke and colleagues from the Uganda Malaria Surveillance Project / Infectious Diseases Research Collaboration are investigating the delivery of health care services in Tororo District. We are doing a research study to see if we can improve the health of children in this area by improving services at government-run health facilities. Certain health centers in Tororo district will be selected to either take part in the intervention to improve services, or to continue with their current services. Assignment to the two groups has been determined by a lottery. The chance of being placed into either of the groups is the same.

Why is this study being done?

As part of this study, we would like to assess health workers' understanding of febrile illnesses, particularly malaria, including how it is transmitted, common symptoms, and approaches to management, diagnosis and treatment. We plan to carry out these surveys once a year for 3 years. As part of the final survey, we would also like to assess how well health workers have been trained to use rapid diagnostic tests (RDTs) for malaria.

What will happen if I take part in this study?

Today, we would like to ask you about training you have received and some questions about fever and malaria using a questionnaire. We will also watch you perform a RDT for malaria, interpret the result, and manage a child with fever. We will take notes while we watch you. All information gathered will be treated as confidential by the study personnel, and records of the assessment will be kept securely in locked filing cabinets and offices. No personal identification information such as names will be used in any reports arising out of this research. The data we collect will be used by project investigators and may be shared with other researchers and policy-makers to answer questions about how best to train health workers in fever case management and diagnosis of malaria, and how to improve delivery of health services.

How long will the assessment last?

Today, the assessment will last about 45 minutes.

Can I stop being in the study?

You can decide to stop participating at any time. Just tell our study personnel right away if you wish to stop the activities.

What risks can I expect from participating in the study?

Participation in any research study may involve a loss of privacy. Information you provide will be recorded, but your name will not be used in any reports of the information provided. No quotes or other results arising from your participation in this study will be included in any reports, even anonymously, without your agreement. The information

obtained from these study activities will be locked at our project offices. We will do our best to make sure that any personal information is kept private.

Are there benefits to taking part in the study?
There will be no direct benefit to you from participating in this study. However, the information that you provide will help researchers and policy-makers understand how best to train health workers in fever case management and diagnosis of malaria, and how to improve delivery of health services in this area.

What other choices do I have if I do not take part in the study?
You are free to choose not to take part in the study. If you decide not to take part, there will be no penalty to you.

What are the costs of taking part in the study? Will I be paid for taking part in the study?
There are no costs to you for taking part in this study. You will not be paid for taking part in this study.

What are my rights if I take part in the study?
Taking part in this study is your choice. You may choose either to take part or not to take part. If you decide to take part in this study, you may change your mind at any time. No matter what decision you take, there will be no penalty to you in any way.

Who can answer my questions about the study?
You can talk to the researchers about any questions or concerns you have about these study activities. Contact Dr. Sarah Staedke or other members of the Uganda Malaria Surveillance Project / Infectious Disease Research Collaboration on telephone number 0414-530692. If you have any questions, comments or concerns about taking part in these activities, first talk to the researchers. If for any reason you do not wish to do this, or you still have concerns about doing so, you may contact Professor James Tumwine, Makerere University School of Medicine Research and Ethical Committee at telephone number 0414-533541.

Giving verbal consent to take part in the study:
You may keep this information sheet if you wish. Participation in these activities is voluntary. You have the right to decline to take part in the activities, or to withdraw from them at any point without penalty. If you do not wish to take part in the activities, you should inform the researcher now. If you do wish to take part in these activities, you should tell the researcher now, and the assessment will begin shortly. If you do not agree to quotes or other results arising from your participation in the study being included, even anonymously, in any reports about the study, please tell the researcher now.

APPENDIX DD: HEALTH WORKER KNOWLEDGE QUESTIONNAIRE (1)

Date: [____|____] / [____|____] / [____|____]
day month year

**Health facility ID**	**HW Study ID**
[_____|_____]	[_____|_____]

Health worker position:

1 = In-charge
2 = Senior medical officer
3 = Medical officer
4 = Senior clinical officer
5 = Clinical officer
6 = Nursing officer
7 = Enrolled nurse
8 = Midwife
9 = Public health nurse
10 = Nursing aide/assistant
11 = Laboratory technician
12 = Laboratory assistant
13 = Health assistant
14 = Health educator
15 = Volunteer
16 = Other___________________________

[____|____]

**SECTION 1**<br>Administer questionnaire by asking the questions below, without prompting for answers. Record all of the health worker's answers below during the interview.	**SECTION 2**<br>After the interview is finished, complete the questions in section 2	**SECTION 3**<br>Finally, score the questionnaire here in section 3	
**1. "What is malaria?"**<br>Record answer	Did the respondent's answer include: 1 = Yes 2 = No<br>[____] Fever or high temperature or hot body<br>[____] Parasites	Give 5 points for each Yes answer in SECTION 2.	[____|____]
**2. "How does someone get malaria?"**<br>Record answer	Did the respondent's answer include: 1 = Yes 2 = No<br>[____] Bite of a mosquito<br>[____] Female mosquito<br>[____] Anopheles mosquito	Give 5 points for each Yes answer in SECTION 2.	[____|____]
**3. "What is the most common symptom of malaria in children?"**<br>Record answer	Did the respondent's answer include: 1 = Yes 2 = No<br>[____] Fever or high temperature or hot body	Give 10 points for a Yes answer in SECTION 2.	[____|____]

HEALTH WORKER KNOWLEDGE QUESTIONNAIRE (2)

**Date:** [____|____] / [____|____] / [____|____]<br>day month year	**Health facility ID**: [____|____]	**HW Study ID:** [____|____]

**SECTION 1**<br>Administer questionnaire by asking the questions below, without prompting for answers. Record all of the health worker's answers below during the interview.	**SECTION 2**<br>After the interview is finished, complete the questions in section 2	**SECTION 3**<br>Finally, score the questionnaire here in section 3	
**4. "What are danger signs of severe malaria in children?"**<br>Record answer	Did the respondent's answer include: 1 = Yes 2 = No<br>[____] Convulsions<br>[____] Altered consciousness (confusion, coma)<br>[____] Lethargy (prostration or extreme weakness)<br>[____] Unable to drink or breastfeed<br>[____] Vomiting everything<br>[____] Unable to sit/stand due to weakness<br>[____] Severe anemia<br>[____] Respiratory distress (labored breathing)<br>[____] Jaundice (yellow coloring of eyes)	Give 2 points for each Yes answer in SECTION 2.	[____|____]
**5. "When you examine a child that you suspect has uncomplicated malaria, what signs do you look out for?"**<br>Record answer	Did the respondent's answer include: 1 = Yes 2 = No<br>[____] Fever or high temperature or hot body<br>[____] Evidence of anemia (pallor)<br>[____] Enlargement of spleen	Give 5 points for each Yes answer in SECTION 2.	[____|____]
**6. "When you examine a child that you suspect has severe malaria, what signs do you look out for?"**<br>Record answer	Did the respondent's answer include: 1 = Yes 2 = No<br>[____] Mental status or level of consciousness<br>[____] Evidence of severe anemia (pallor)<br>[____] Dehydration	Give 5 points for each Yes answer in SECTION 2.	[____|____]

HEALTH WORKER KNOWLEDGE QUESTIONNAIRE (3)		
**Date:** [____|____] / [____|____] / [____|____] day month year	**Health facility ID**: [____|____]	**HW Study ID:** [____|____]

**SECTION 1**<br>Administer questionnaire by asking the questions below, without prompting for answers. Record the health worker's answers below during the interview.	**SECTION 2**<br>After the interview is finished, complete the questions in section 2	**SECTION 3**<br>Finally, score the questionnaire here in section 3	
**7. "What illnesses, other than malaria, commonly cause fever in children?"**<br>Record answer	Did the respondent's answer include: 1 = Yes 2 = No<br>[____] Viral infection (common cold, HIV/AIDS, etc)<br>[____] Bacterial infection<br>[____] Measles<br>[____] Ear infection (otitis media or externa)<br>[____] Throat infection (pharyngitis, tonsillitis)<br>[____] Respiratory tract infection (pneumonia)<br>[____] Urinary tract infection<br>[____] Skin infection (abscess)<br>[____] Typhoid fever<br>[____] Brucellosis	Give 2 points for each Yes answer in SECTION 2.	[____|____]
**8. "How can the diagnosis of malaria be confirmed?"**<br>Record answer	Did the respondent's answer include: 1 = Yes 2 = No<br>[____] Positive blood slide for malaria parasites<br>[____] Positive rapid diagnostic test	Give 5 points for each Yes answer in SECTION 2.	[____|____]
**9. "How should a child with uncomplicated malaria be treated?"**<br>Record answer	Did the respondent's answer include: 1 = Yes 2 = No<br>[____] Coartem (artemether-lumefantrine)<br>[____] Dose based on child's age or weight<br>[____] Give Coartem tablets twice a day for 3 days	Give 5 points for each Yes answer in SECTION 2.	[____|____]

HEALTH WORKER KNOWLEDGE QUESTIONNAIRE (4)

**Date:** [____|____] / [____|____] / [____|____]<br>day month year	**Health facility ID**: [____|____]	**HW Study ID:** [____|____]

**SECTION 1**<br>Administer questionnaire by asking the questions below, without prompting for answers. Record the health worker's answers below during the interview.	**SECTION 2**<br>After the interview is finished, complete the questions in section 2	**SECTION 3**<br>Finally, score the questionnaire here in section 3	
**10. "What information should be given to the primary caregiver as part of the counseling when administering treatment for uncomplicated malaria?"**<br>Record answer	Did the respondent's answer include: 1 = Yes 2 = No<br>[____] Give Coartem tablets twice a day for 3 days<br>[____] Give Coartem with milk or meal if possible<br>[____] What to do if child does not improve<br>[____] Continue supportive treatment	Give 5 points for each Yes answer in SECTION 2.	[____|____]
**11. "If Coartem is not available, what other antimalarials can be used to treat uncomplicated malaria?"**<br>Record answer	Did the respondent's answer include: 1 = Yes 2 = No<br>[____] Amodiaquine + artesunate	Give 10 points for a Yes answer in SECTION 2.	[____|____]
**12. "What medical treatment should be given to a child with severe malaria?"**<br>Record answer	Did the respondent's answer include: 1 = Yes 2 = No<br>[____] Referral to a health center IV or hospital<br>[____] Treatment with quinine<br>[____] Quinine IV 10mg/kg every 8 hours<br>[____] Give quinine for total of 7 days	Give 5 points for each Yes answer in SECTION 2.	[____|____]

TOTAL SCORE [____|____|____]

APPENDIX DD: HEALTH WORKER KNOWLEDGE QUESTIONNAIRE

Health centre code	Health Worker ID	Date
[____|____]	[____|____|____|____]	[____|____] / [____|____] / [____|____]<br>day month year

Position:

1 = In-charge	5 = Clinical officer	9 = Public health nurse	13 = Health assistant
2 = Senior medical officer	6 = Nursing officer	10 = Nursing aide/assistant	14 = Health educator
3 = Medical officer	7 = Enrolled nurse	11 = Laboratory technician	15 - Volunteer
4 = Senior clinical officer	8 = Midwife	12 = Laboratory assistant	15 = Other________________ [____|____]

PART 1: DEMOGRAPHIC INFORMATION

**1. Age**	Years [____|____]	**5. Highest level of education or qualification achieved**	
**2. Gender**	1 = Male<br>2 = Female [____]	0 = None<br>1 = Primary (P1 — P7)<br>2 = Secondary (S1 — S6)<br>3 = Certificate<br>77 = Other________________________	4 = Diploma<br>5 = Bachelor's degree<br>88 = Don't know<br>99 = Refused to answer<br>[____|____]
**3. 'Are you originally from this area?'**	1 = Yes<br>2 = No [____]		
**4. 'How long have you worked at this health centre?'**	[____|____]years<br>[____|____]months	**6. What year did you graduate from your course?**	[____|____|____|____]

PART 2: INSTRUCTIONS

Welcome to the Health Worker Knowledge questionnaire on RDTs and Fever Case Management.
Please take a moment to answer the following questions; there are 16 questions in the questionnaire. You may/may not have done this questionnaire in the past; however, you do not need to worry if you don't know every answer.

This is **NOT A TEST** that will affect your role or responsibilities and **WE VALUE THE TIME YOU TAKE** to complete this quiz. There is no need to write your name on this questionnaire.
All responses will be kept strictly confidential. Thank you!

GENERAL INSTRUCTIONS TO COMPLETE THIS QUESTIONNAIRE

1. *Please use a dark coloured pen to fill out the questionnaire*
2. *The health worker identity (ID) number is the unique number that was given to you at the start of this PRIME project. When we request you to give your ID number, please fill the boxes as below:*

   [_0_ | _5_ | _1_ | _0_]

   You are asked to enter your ID number like this at the top of each page of your questionnaire.
3. *In this questionnaire, we ask you to read each question carefully at the questions and decide which response you believe is correct. When you have decided, please circle the letter in the column beside that response. For example:*

**1. Today is Monday**	a) True (circled)<br>b) False

4. *If you change your mind and would like to circle a different response, please cross out your original choice and circle the choice that you believe is correct. For example, if you change your mind and you decide that your answer is 'false', cross through the original and circle the new response as below:*

**1. Today is Monday**	a) True (circled and crossed out)<br>b) False (circled)

HEALTH WORKER KNOWLEDGE QUESTIONNAIRE (2)

Health centre code	Health Worker ID	Date
[____|____]	[____|____|____|____]	[____|____] / [____|____] / [____|____]<br>day month year

PART 3: QUESTIONNAIRE

1	**All cases of fever in Uganda are caused by malaria.**	a) True<br>b) False
2	**RDT stands for Rapid Diagnostic Test for malaria. Which of the following is NOT true about RDTs?**	a) If performed correctly, they are very accurate in diagnosing which patients have malaria and which do not.<br>b) They can be performed in health centres that do not have power or laboratory equipment.<br>c) They can detect typhoid fever and pneumonia as well as malaria.
3	**In order to perform an RDT, you will need to use finger prick blood. After you prick a patient's finger with a lancet, what should you do with the lancet?**	a) Save it to wash and use again later.<br>b) Carefully put it immediately in the sharps container.<br>c) Put it on the table beside you, to use for the next patient.
4	**We have provided an RDT labelled A. Look carefully at it and read the result.**	a) Positive<br>b) Negative<br>c) Invalid
5	**Here is another RDT labelled B. Look carefully at it and read the result.**	a) Positive<br>b) Negative<br>c) Invalid
6	**RDTs should be stored in a cool, dry place.**	a) True<br>b) False
7	**You can use the same RDT for two different patients.**	a) True<br>b) False
8	**You should always check the expiry date on the package of an RDT before using it.**	a) True<br>b) False
9	**For which of the following patients should you perform an RDT for malaria?**	a) A 4-year-old boy with fever and mild cough<br>b) An 8-month-old child with fever who refuses to breastfeed, is breathing rapidly and has very pale palms and sunken eyes<br>c) A 20-year-old woman who is pregnant and has fever<br>a) A 32-year-old man who is HIV-positive and has fever<br>b) A 45-year-old man with fever and joint paints<br>c) All of the above

HEALTH WORKER KNOWLEDGE QUESTIONNAIRE (3)

Health centre code	Health Worker ID	Date
[____|____]	[____|____|____|____]	[____|____] / [____|____] / [____|____]<br>day month year

10	**A patient comes to your health centre with fever. She has taken chloroquine for two days but the fever has persisted. You perform an RDT and the result is positive. What should you do?**	a) Tell the patient to complete the course of chloroquine.<br>b) Prescribe a full course of Coartem.
11	**A patient comes to your health centre with fever. She took a complete, correct course of Co-artem last week. She did not vomit any of the doses. You perform an RDT and the result is positive. What should you do?**	a) Prescribe a full course of quinine.<br>b) Prescribe a second full course of Coartem.<br>c) Advise her to take Panadol and fluids only.
12	**Which of the following is a symptom or sign of severe illness in a patient with fever?**	a) Convulsions currently or within the past 2 days<br>b) Extreme weakness – patient is unable to sit or stand without support<br>c) Severe anaemia – patient's palms and conjunctivae are very pale<br>d) Unable to drink or breastfeed<br>e) All of the above
13	**A 35-year-old man comes to your health centre. He tells you that he has fever and muscle aches. What should you do FIRST?**	a) Prescribe Coartem immediately.<br>b) Prescribe chloroquine immediately – this regimen is best for adults.<br>c) Prescribe Panadol only and tell the patient to go home and rest.<br>d) Ask the patient how long he has had these symptoms, and ask if he has taken any drugs at home before coming to the health centre.
14	**A mother brings her 8-year-old daughter to your health centre. Beginning yesterday, the girl has had fever and no appetite. Her mother has given Panadol and fluids, but the symptoms continue. There are no signs or symptoms of severe illness. On physical examination, you find no obvious cause of her fever. You perform an RDT, and the result is negative. Which of the following is TRUE?**	a) This patient has a mild febrile illness, but the cause is not clear. The mother should continue to provide fluids and Panadol. You should advise her to bring the girl back to the health centre if the symptoms do not get better within 2 to 3 more days.<br>b) You should prescribe a course of chloroquine. Even though the RDT is negative, the girl may still have malaria, and chloroquine is appropriate for mild cases of malaria.<br>c) You should prescribe Coartem. Even though the RDT is negative, the girl may have malaria, and Coartem is the first-line regimen for malaria in Uganda.

HEALTH WORKER KNOWLEDGE QUESTIONNAIRE (2)

Health centre code	Health Worker ID	Date
[____|____]	[____|____|____|____]	[____|____] / [____|____] / [____|____]<br>day month year

15	**A 10-year-old boy comes to your health centre with fever and joint pains. He looks weak, but is able to sit up easily by himself. After you take a history and do a physical examination, you perform an RDT. The RDT is positive, and you plan to treat for uncomplicated malaria. According to the Uganda Ministry of Health guidelines, which of the following is the BEST (first-line) treatment for this patient?**	a) Chloroquine<br>b) Oral quinine<br>c) Injectable quinine<br>d) Coartem (artemether-lumefantrine)<br>e) Chloroquine + Fansidar (Homapak)
16	**A mother brings her 1-year-old son to your health centre. She tells you he has not been feeding well. The child's body is hot to touch. He is weak and cannot sit up by himself, and he is breathing very rapidly. What should you do? Be sure to read all the statements below before answering.**	a) Undress the child, and ask the mother to sponge him to help reduce the fever.<br>b) Immediately prepare and inject a dose of quinine AND antibiotic.<br>c) Perform an RDT and write the result on the referral note.<br>d) Write a referral note and ensure that the child is referred to a Health Centre IV or Hospital as soon as possible.<br>e) All of the above.

Thank you!

WHO Toxicity Grading Scale for Determining The Severity of Adverse Events

HEMATOLOGY				
ITEM	Grade 1 Toxicity	Grade 2 Toxicity	Grade 3 Toxicity	Grade 4 Toxicity
Hemoglobin	9.5 - 10.5 gm/Dl	8.0 - 9.4 gm/Dl	6.5 - 7.9 gm/Dl	< 6.5 gm/Dl
Absolute Neutrophil Count	1000-1500/mm^3	750-999/mm^3	500-749/mm^3	<500/mm^3
Platelets	75000-99000/mm^3	50000-74999/mm^3	20000-49000/mm^3	<20000/mm^3
Prothrombin Time (PT)	1.01 - 1.25 x ULN	1.26-1.5 x ULN	1.51 -3.0 x ULN	>3 x ULN
Activated Partial Thromboplastin (APPT)	1.01 -1.66 x ULN	1.67 - 2.33 x ULN	2.34 - 3 x ULN	> 3 x ULN
Fibrinogen	0.75 - 0.99 X LLN	0.50 - 0.74 x LLN	0.25 - 0.49 x LLN	< 0.25 x LLN
Fibrin Split Product	20-40 mcg/ml	41-50 mcg/ml	51-60 mcg/ml	> 60 mcg/ml
Methemoglobin	5 - 9.9 %	10.0 - 14.9 %	15.0 - 19.9%	> 20 %
**LIVER ENZYMES**				
AST (SGOT)	1.25 - 2.5 x ULN	2.6 - 5 x ULN	5.1 - 10 x ULN	> 10 x ULN
ALT (SGPT)	1.25 - 2.5 x ULN	2.6 - 5 x ULN	5.1 - 10 x ULN	> 10 x ULN
GGT	1.25 -2.5 x ULN	1.6 - 5 x ULN	5.1 - 10 x ULN	> 10 x ULN
Alkaline Phosphatase	1.25 - 2. 5 x ULN	1.6 - 5 x ULN	5.1 - 10 x ULN	> 10 x ULN
Amylase	1.1 - 1.5 x ULN	1.6 - 2.0 x ULN	2.1 - 5.0 x ULN	> 5.1 x ULN
**CHEMISTRIES**				
Hyponatremia	130-135 mEq/L	123-129 mEq/L	116-122 mEq/L	< 116 or mental status changes or seizures
Hypernatremia	146-150 mEq/L	151-157 mEq/L	158-165 mEq/L	> 165 mEq/L or mental status changes or seizures
Hypokalemia	3.0 - 3.4 mEq/L	2.5 - 2.9 mEq/L	2.0 - 2.4 mEq/L or intensive replacement Rx required or hospitalization required.	< 2.0 mEq/L or paresis or ileus or life-threatening arrhythmia
Hyperkalemia	5.6 - 6.0 mEq/L	6.1 - 6.5 mEq/L	6.6 - 7.0 mEq/l	> 7.0 mEq/L or life-threatening arrhythmia
Hypoglycemia	55-64 mg/dL	40-54 mg/dL	30-39 mg/dL	<30 mg/dL or mental status changes or coma

**CHEMISTRIES (continued)**				
Hyperglycemia<br>(note if fasting)	116 - 160 mg/dL	161- 250 mg/dL	251 - 500 mg/dL	> 500 mg/dL<br>or ketoacidosis<br>or seizures
Hypocalcemia<br>(corrected for albumin)	8.4 - 7.8 mg/dL	7.7 - 7.0 mg/dL	6.9 - 6.1 mg/dL	< 6.1 mg/dL<br>or life threatening arrhythmia or tetany
Hypercalcemia<br>(correct for albumin)	10.6 - 11.5 mg/dL	11.6 - 12.5 mg/dL	12.6 - 13.5 mg/dL	> 13.5 mg/dL<br>life-threatening arrhythmia
Hypomagnesemia	1.4 - 1.2 mEq/L	1.1 - 0.9 mEq/L	0.8 - 0.6 mEq/L	< 0.6 mEq/L or life-threatening arrhythmia
Hypophosphatemia	2.0 - 2.4 mg/dL	1.5 -1.9 mg/dL or replacement Rx required	1.0 -1.4 mg/dL intensive Rx or hospitalization required	< 1.0 mg/dL or life-threatening arrhythmia
Hyperbilirubinemia	1.1 - 1.5 x ULN	1.6 - 2.5 x ULN	2.6 - 5 x ULN	> 5 x ULN
BUN	1.25 - 2.5 x ULN	2.6 - 5 x ULN	5.1 - 10 x ULN	> 10 x ULN
Creatinine	1.1 x 1.5 x ULN	1.6 - 3.0 x ULN	3.1 - 6 x ULN	> 6 x ULN or required dialysis
**URINALYSIS**				
Proteinuria	1+ or < 0.3% or <3g/L or 200 mg - 1 gm loss/day	2 -3 + or 0.3 - 1.0% or 3-10 g/L<br>1- 2 gm loss/day	4+ or > 1.0% or > 10 g/L<br>2-3.5 gm loss/day	nephrotic syndrome or > 3.5 gm loss/day
Hematuria	microscopic only	gross, no clots	gross+ clots	obstructive or required transfusion
**CARDIAC DYSFUNCTION**				
Cardiac Rhythm		asymptomatic, transient signs, no Rx required	recurrent/persistent; No Rx required	requires treatment
Hypertension	transient inc. > 20 mm; no Rx	recurrent, chronic, > 20 mm, Rx required	requires acute Rx; No hospitalization	requires hospitalization
Hypotension	transient orthostatic hypotension, No Rx	symptoms correctable with oral fluids Rx	requires IV fluids; no hospitalization required	requires hospitalization
Pericarditis	minimal effusion	mild/moderate asymptomatic effusion, no Rx	symptomatic effusion; pain; EKG changes	tamponade; pericardiocentesis or surgery required
Hemorrhage, Blood Loss	microscopic/occult	mild, no transfusion	gross blood loss;<br>1-2 units transfused	massive blood loss;<br>> 3 units transfused

**RESPIRATORY**				
Cough	transient- no Rx	treatment associated cough<br>local Rx	uncontrolled	
Bronchospasm, Acute	transient; no Rx<br>< 80% - 70% FEV_1<br>(or peak flow)	requires Rx<br>normalizes with bronchodilator;<br>FEV_1 50% - 70%<br>(or peak Flow)	no normalization with bronchodilator;<br>FEV_1 25% - 50%<br>(or peak flow retractions)	cyanosis: FEV_1<br>< 25%<br>(or peak flow) or intubated
**GASTROINTESTINAL**				
Stomatitis	mild discomfort; no limits on activity	some limits on eating/drinking	eating/talking very limited	requires IV fluids
Nausea	mild discomfort; maintains reasonable intake	moderate discomfort; intake decreased significantly; some activity limited	severe discomfort; no significant intake; activities limited	minimal fluid intake
Vomiting	transient emesis	occasional/moderate vomiting	orthostatic hypotension or IV fluids required	hypotensive shock or hospitalization required for IV fluid therapy
Constipation	mild	moderate	severe	distensions w/vomiting
Diarrhea	transient 3-4 loose stools/day	5-7 loose stools/day	orthostatic hypotension or<br>> 7 loose stools/day or required IV fluids	hypotensive shock or hospitalization for IV fluid therapy required
**NEURO & NEUROMUSCULAR**				
Neuro-Cerebellar	slight incoordination dysdiadochokinesis	intention tremor, dysmetria, slurred speech; nystagmus	locomotor ataxia	incapacitated
Mood	mild anxiety or depression	moderate anxiety or depression and therapy required	severe anxiety or depression or mania; needs assistance	acute psychosis; incapacitated, requires hospitalization
Neuro Control<br>(ADL = activities of daily living)	mild difficulty concentrating; no Rx; mild confusion/agitation; ADL unaffected	moderate confusion/agitation some limitation of ADL; minimal Rx	severe confusion/agitation needs assistance for ADL; therapy required	toxic psychosis; hospitalization
Muscle Strength	subjective weakness<br>no objective symptoms/ signs	mild objective signs/symptoms<br>no decrease in function	objective weakness function limited	paralysis

OTHER PARAMETERS				
Fever: oral, > 12 hours	37.7 - 38.5 C or 100.0 - 101.5 F	38.6 - 39.5 C or 101.6 - 102.9 F	39.6 - 40.5 C or 103 - 105 F	> 40 C or > 105 F
Headache	mild, no Rx therapy	transient, moderate; Rx required	severe; responds to initial narcotic therapy	intractable; required repeated narcotic therapy
Fatigue	no decrease in ADL	normal activity decreased 25- 50%	normal activity decreased > 50% can't work	unable to care for self
Allergic Reaction	pruritus without rash	localized urticaria	generalized urticaria; angioedema	anaphylaxis
Local Reaction	tenderness or erythema	induration < 10 cm or phlebitis or inflammation	induration > 10 cm or ulceration	necrosis
Mucocutaneous	erythema; pruritus	diffuse, maculo papular rash, dry desquamation	vesiculation, moist desquamation, or ulceration	exfoliative dermatitis, mucous membrane involvement or erythema, multiforme or suspected Stevens-Johnson or necrosis requiring surgery

APPENDIX FF. Guidelines for grading symptoms, signs and laboratory findings

Table A. Guidelines for grading patient symptoms

	**Grade 1**<br>**MILD**	**Grade 2**<br>**MODERATE**	**Grade 3**<br>**SEVERE**	**Grade 4**<br>**LIFE THREATENING**
**Subjective fever in the past 24 h**	N/A	Present (Yes)	N/A	N/A
**Weakness**	Mild decrease in activity; For children – weak, but still playing	Moderate decrease in activity; For children – weak, and playing limited	Not participating in usual activities; For children – not playing	Prostration
**Muscle and/or joint aches***	Mild and/or localized complaints	Diffuse complaints	Objective weakness; function limited	N/A
**Headache***	Mild, no treatment required	Transient, moderate; treatment required	Severe, constant; requires narcotic therapy	Intractable; requires repeated narcotic therapy
**Anorexia**	Decreased appetite, but still taking solid food	Decreased appetite, avoiding solid food but taking liquids	Appetite very decreased; Refusing to breast feed, no solids or liquids taken (< 2 years ≤ 12 hr; > 2 years ≤ 24 hr)	Appetite very decreased; Refusing to breast feed, no solids or liquids taken (< 2 years > 12 hr; > 2 years > 24 hr)
**Nausea***	Mild, transient feeling of impending vomiting; maintains reasonable intake	Moderate and/or constant feeling of impending vomiting; intake decreased	Severe, constant feeling of impending emesis; intake decreased significantly	N/A
**Vomiting**	1 episode per day	2-3 episodes per day	Orthostatic hypotension or IV fluids required	Hypotensive shock or 1nrolment1ation required for IV fluid therapy
**Abdominal pain***	Mild (1-3 on a scale of 1 to 10)	Moderate (4-6 on a scale of 1 to 10)	Moderate to severe (≥ 7 on a scale of 1 to 10)	Severe – 1nrolment1at for treatment
**Diarrhea**	Transient 3-4 loose stools/day	5-7 loose stools/day	Orthostatic hypotension or > 7 loose stools/day or IV fluids required	Hypotensive shock or 1nrolment1ation for IV fluid therapy required
**Cough**	Transient / intermittent	Persistent / constant	Uncontrolled	Cyanosis, stridor, severe shortness of breath
**Pruritis**	Transient pruritis	Pruritis that disturbs sleep	Severe, constant pruritis, sleep disturbed	N/A
**Tinnitus***	Mild, transient ringing or roaring sound	Moderate, persistent ringing or roaring sound	Severe ringing or roaring sound with associated hearing loss	N/A
**Behavioural changes**	Mild difficulty concentrating; mild confusion or agitation; activities of daily living unaffected; no treatment	Moderate confusion or agitation; some limitation of activities of daily living; minimal treatment	Severe confusion or agitation; Needs assistance for activities of daily living; therapy required	Toxic psychosis; 1nrolment1ation required
**"Flu" (viral URI)**	Mild nasal congestion, mild rhinorrhea	Moderate nasal congestion, moderate rhinorrhea	N/A	N/A
**Allergic reaction**	N/A	N/A	Urticaria	Severe urticaria anaphylaxis, angioedema
**Convulsion**	N/A	N/A	Localized or generalized seizure	Status epilepticus
*** Assess only in children ≥ 3 years of age. Answer N/A for younger children and those unable to answer.**				

Table B. Guidelines for physical examination

**Dehydration**	Assess skin touch and turgor, mucous membranes, eyes, crying, fontanelle, pulse, urine output
**Jaundice**	Assess for yellowing of the sclera. Also evaluate the palpepral conjunctiva, lips, and skin.
**Chest**	Observe the rate, rhythm, depth, and effort of breathing. Check the patient's colour for cyanosis.<br><br>The maximum acceptable respiratory rate by age: < 2 months = 60, 2-12 months = 50, 1-5 years = 40, above 5 years = 30.<br><br>Inspect the neck for the position of the trachea, for supraclavicular retractions, and for contraction of the sternomastoid or other accessory muscles during inspiration.<br><br>Auscultate the anterior and posterior chest for normal breath sounds and any adventitious sounds (crackles or rales, wheezes, and rhonchi). *Crackles are intermittent, non-musical, fine or coarse sounds that may be due to abnormalities of the lungs (pneumonia, fibrosis, early congestive heart failure) or airways (bronchitis or bronchiectasis). Wheezes are high-pitched and result from narrowed airways. Rhonchi are relatively low-pitched and suggest secretions in large airways.*<br><br>If abnormalities are identified, evaluate for transmitted voice sounds. In addition, palpate the chest to assess for tactile fremitus, and percuss the chest to assess for areas of dullness. *Normal, air-filled lungs emit predominantly vesicular breath sounds, transmit voice sounds poorly with "ee" = "ee", and have no tactile fremitus. Airless lung, as in lobar pneumonia, emits bronchial breath sounds, transmits spoken words clearly with "ee" = "aay" (egophany), and has an increase in tactile fremitus.*
**Abdomen**	Inspect and ausculate the abdomen. Listen for bowel sounds in the abdomen before palpating it. Palpate the abdomen in all 4 quadrants lightly and then deeply. Assess the size of the liver and spleen. To assess for peritoneal inflammation, look for localised and rebound tenderness, and voluntary or involuntary rigidity.
**Skin**	Inspect the skin for colour, turgor, moisture, and lesions. If lesions are present, note their location and distribution (diffuse or localised), arrangement (linear, clustered, annular, dermatomal), type (macules, papules, vesicles) and colour.
**Tablet test**	For children $\geq$ 9 months of age, ask the patient to pick a tablet (or equivalent object) up off a flat surface using the thumb and index finger of their dominant hand. *This tests for co-ordination of the upper extremity assessing the function of the motor system, cerebellar system, vestibular system (for coordinating eye and body movements) and the sensory system, for position sense. When testing small children, be aware that they will likely attempt to put the object into their mouth.*

Table C. Grading physical examination findings

	Grade 1<br>MILD	Grade 2<br>MODERATE	Grade 3<br>SEVERE	Grade 4<br>LIFE-THREATENING
**Temperature* (axillary)**	37.5-37.9°C	38.0-39.5°C	> 39.5°C	Sustained fever, equal or greater than 40.0°C for longer than 5 days
**Dehydration**	Less than 2 of the following:<br>Restless, irritable<br>Sunken eyes<br>Drinks eagerly, thirsty<br>Skin pinch goes back slowly	2 of the following:<br>Restless, irritable<br>Sunken eyes<br>Drinks eagerly, thirsty<br>Skin pinch goes back slowly	Two of the following:<br>Lethargic or unconscious<br>Sunken eyes<br>Not able to drink or drinking poorly<br>Skin pinch goes back very poorly	Two of the following + shock:<br>Lethargic or unconscious<br>Sunken eyes<br>Not able to drink or drinking poorly<br>Skin pinch goes back very poorly
**Jaundice**	Slight yellowing of sclera and conjunctiva	Moderate yellowing of sclera and conjunctiva, yellowing of mucous membranes	Severe yellowing of sclera and conjunctiva, yellowing of skin	N/A
**Chest**	Mildly increased RR (for age, temperature), transient or localised adventitious sounds	Moderately increased RR, diffuse or persistent adventitious sounds	Rapid RR (< 2 months > 60, 2-12 months > 50, 1-5 years > 40, adults > 30)* nasal flaring, retractions	Cyanosis
**Abdomen**	Normal bowel sounds, mild localised tenderness, and/or liver palpable 2-4 cm below the right costal margin (RCM), and/or spleen palpable, and/or umbilical hernia present	Normal or mildly abnormal bowel sounds, moderate or diffuse tenderness; and/or mild to moderately enlarged liver (4-6 cm below the RCM) and/or spleen palpable up to half-way between umbilicus and symphysis pubis	Severely abnormal bowel sounds, severe tenderness to palpation. Evidence of peritoneal irritation and/or significant enlargement of liver (> 6 cm below the RCM) and/or spleen palpable beyond half-way between umbilicus and symphysis pubis	Absent bowel sounds. Involuntary rigidity
**Skin†**	Localised rash, erythema, or pruritis	Diffuse, maculopapular rash, dry desquamation	Vesiculation, moist desquamation, or ulceration	Exfoliative dermatitis, mucous membrane involvement or erythema multiforme or suspected Stevens-Johnson or necrosis requiring surgery
**Hearing**	*< 4 years: N/A*<br>≥ 4 years: Decreased hearing in one ear	*< 4 years: N/A*<br>≥ 4 years: Decreased hearing in both ears or severe impairment in one ear	*< 4 years: Any evidence of hearing impairment*<br>≥ 4 years: Severe impairment in both ears	N/A
**Tablet test**	Difficulty grasping tablet but able to pick up	Unable to pick up tablet without dropping	Unable to grasp tablet	N/A
**Clinical symptoms / sign *(not otherwise specified)***	No treatment required; monitor condition	Treatment required	Requires treatment and possible hospitalisation	Requires active medical intervention, hospitalisation, or hospice care

Reference – The Harriet Lane Handbook, 15th edition, 2000
† Reference – WHO Toxicity Grading Scale for Determining the Severity of Adverse Events

TABLE D. Guidelines for grading of laboratory abnormalities

	**Grade 1**<br>**MILD**	**Grade 2**<br>**MODERATE**	**Grade 3**<br>**SEVERE**	**Grade 4**<br>**LIFE-THREATENING**
**Haemoglobin**<br>*(g/dL)*	9.0 – 9.9	7.0 – 8.9	5.0 – 6.9	< 5.0

Reference – The Harriet Lane Handbook, 15th edition, 2000†
Reference – WHO Toxicity Grading Scale for Determining the Severity of Adverse Events

APPENDIX GG: ADVERSE EVENT REPORT FORM

Study ID	Date of Event Onset	Date of Event Report
[_____|_____|_____|_____]	[___|___]/ [___|___]/[___|___]<br>day month year	[___|___]/ [___|___]/[___|___]<br>day month year

Cluster number	AE number	Gender	Age	Weight
[_____|_____]	[_____|_____]	[_____] 1 = Male<br>2 = Female	[_____|_____] [_____|_____]<br>years months *(only if < 1 year)*	[_____|_____] kg

SECTION 1: EVENT INFORMATION

To be completed by field team

1. Event description:____

**2. Did this event occur after treatment with Coartem (artemether-lumefantrine)?**	1 = Yes<br>2 = No	88 = Don't know	[_____|_____]
**3. Was the AL obtained from the PRIME Study?**	1 = Yes<br>2 = No	88 = Don't know	[_____|_____]
**4. If no, from where was the AL obtained?**	1 = Coartem was kept at home<br>2 = Bought Coartem from Duka<br>3 = Bought Coartem from drug shop/pharmacy<br>4 = From Public Health center<br>5 = From Government Hospital<br>6 = From Private clinic/Hospital<br>7 = From ACT PRIME Study Clinic<br>8 = From Community Health Worker	77=Other<br>____________________<br>88=Don't know<br>99=Refused to answer	[_____|_____]
**5. Was this a serious adverse event?**	*If yes, go to Qn 4, otherwise skip to Qn 5.*	1 = Yes<br>2 = No<br>88 = Don't know	[_____|_____]
**6. Indicate reason for serious adverse event:**	1 = Death<br>2 = Life threatening condition<br>3 = Resulted in hospitalization (overnight stay at hospital or clinic)<br>4 = Prolonged hospitalization	5 = Required medical or surgical intervention to prevent serious outcome<br>6 = Resulted in significant or persistent disability<br>77 = Other<br>____________________	[_____|_____]<br>[_____|_____]

7. EVENT SUMMARY *(include details of event, associated signs and symptoms, and medical management):*

____

____

____

____

____

____

____

SECTION 2: TREATMENT INFORMATION

To be completed by medical officer

**8. Medication***	**10. Start date**<br>[___|___]/ [___|___]/[___|___]<br>day month year	**12. Daily dose**
**9. Indication**	**11. Stop date**<br>[___|___]/ [___|___]/[___|___]<br>day month year	**13. Duration**

***** *List medication that is suspected to be associated with the adverse event. Note that there is space to record all concomitant medications on page 2.***

AE REPORT FORM (2)	Cluster number [____|____]	Study ID [____|____|____|____]	Date of Event Report [___|___]/ [___|___]/[___|___] day month year

RELEVANT LABORATORY TESTS *(Complete if available)*		
**14. Test**	**15. Collection date** [dd/mm/yy]	**16. Results/Comments**
a)	[/ /]	a)
b)	[/ /]	b)
c)	[/ /]	c)

RELEVANT DIAGNOSTIC TESTS *(Complete if available)*		
**17. Test**	**18. Collection date** [dd/mm/yy]	**19. Results/Comments**
a)	[/ /]	a)
b)	[/ /]	b)
c)	[/ /]	c)

CONCOMITANT MEDICATIONS *(List all medications that the child was taking up to 1 month prior to event onset)*				
**20. Medication**	**21. Start date** [dd/mm/yy]	**22. Stop date** [dd/mm/yy]	**23. Total dose**	**24. Indication**
a)	[/ /]	[/ /]	a)	a)
b)	[/ /]	[/ /]	b)	b)
c)	[/ /]	[/ /]	c)	c)
d)	[/ /]	[/ /]	d)	d)

SECTION 3: GRADING & OUTCOME

To be completed by study coordinator

**25. Maximum severity (WHO scale)**	1 = Mild<br>2 = Moderate	3 = Severe<br>4 = Life-threatening	[____|____]
**26. Suspected relationship:**	0 = None<br>1 = Unlikely<br>2 = Possible	3 = Probable<br>4 = Definite	[____|____]
**27. Expected** *(If the event occurred in association with Coartem, was the event listed on Coartem package insert?):*		1 = Yes 3 = N/A<br>2 = No	[____]

**28. Outcome of event** 1 = Ongoing<br>2 = Resolved without sequelae<br>3 = Resolved with sequelae<br>4 = Death [_____]	**29. If resolved or died, indicate date:**<br>[____|____]/[____|____]/[____|____]<br>day month year
**Completed by** *(printed name):*<br><br>**Signature:**	**Investigator** *(printed name):*<br><br>**Signature:**
**Date:**<br>[____|____]/[____|____]/[____|____]<br>day month year	**Date:**<br>[____|____]/[____|____]/[____|____]<br>day month year

THE REPUBLIC OF UGANDA

APPENDIX HH. COHORT STUDY
Information Sheet for Termination of Study Activities

Introduction

Dr. Sarah Staedke and colleagues from the Uganda Malaria Surveillance Project / Infectious Diseases Research Collaboration are investigating delivery of healthcare services in Tororo District. We are doing a research study to see if we can improve the health of children in this area by improving services at government-run health facilities. Your child(ren) have been taking part in the ACT PRIME cohort study. The purpose of this information sheet is to tell you about our plans to end the cohort study.

Why are parts of the study ending?

ACT PRIME is a research study. Since May 2011, certain health centers in Tororo district have been selected to either take part in the intervention to improve services, or to continue with their current services. To find out how well the intervention is working, we have been reviewing the health of children living near the health centers including your children. We plan to end the cohort study by September 2012.

What parts of the study are ending?

Since your household was enrolled, we have been asking you to take part in different study activities. We have asked members of your household to record information about the health of your children and how much you spend on their treatment using a diary with pictures. We have visited your household once a month to collect the diaries and ask questions about the health of your children. We have also asked you take part in two household surveys so that we could learn more about your household and how you manage illnesses in children. We have asked you to bring your children to our clinic about every 6 months so that we could examine your child(ren) and do blood tests. All of these activities will be ending by September 2012. This means that your household and children enrolled in the study will no longer be required to take part in any of these activities.

When will my child stop participating in the study activities?

All children from your household that are taking part in the study will stop on the same day. We will schedule an appointment for you to bring your child(ren) to the study clinic. This may be at their scheduled clinical follow-up visit, or at a separate appointment. When you come to the clinic, we will tell you more about the plans to end the study and how this will affect your child(ren). We will also answer any questions.

How does the end of the study activities affect me and my child?

After the last clinic visit, your child(ren) will no longer take part in the cohort study activities. You will no longer need to complete the monthly diary, no one will come to your household to collect the diaries and ask questions about the health of your children, there will be no more household surveys, and there will be

no more clinic visits. Because these activities are ending, you will also stop receiving the soap, sugar, and salt that you have been receiving at the monthly visits to your household. At the end of the study, we will give each household an insecticide-treated bednet and each child a small token of appreciation for taking part in the study.

<u>What happens to my child after the study activities have ended?</u>

There is no future risk or benefit to your child(ren) or household after the study activities end. You will continue to be responsible for your child(ren)'s health care.

<u>Can I stop my child from being in the study now?</u>

We would like to make an appointment for you to bring your child(ren) to the study clinic so that we can talk to you more about our plans to end the study. But, you can decide to stop taking part at any time. Just tell our study personnel right away if you wish to stop taking part in the study now.

<u>Who can answer my questions about the study?</u>

You can talk to the researchers about any questions or concerns you have about these study activities. Contact Dr. Sarah Staedke or other members of the Uganda Malaria Surveillance Project / Infectious Disease Research Collaboration on telephone number 0414-530692. If you have any questions, comments or concerns about taking part in these activities, first talk to the researchers. If for any reason you do not wish to do this, or you still have concerns about doing so, you may contact Professor James Tumwine, Makerere University School of Medicine Research and Ethical Committee at telephone number 0414-530020.

THE REPUBLIC OF UGANDA

APPENDIX HH. COHORT STUDY
Signature Form for Termination of Study Activities

**Protocol Title:**	ACT PRIME Study: Evaluating the impact of enhanced health facility-based care for malaria and febrile illnesses in children
**Site of Research:**	Tororo, Uganda
**Principal Investigators**:	Dr. Sarah Staedke
**Date:**	15 May 2012

I understand that the ACT PRIME cohort study is ending and that as of today my child(ren) will no longer take part in the study. I understand that after today, I no longer need to fill in the monthly diary, no one will come to my household to collect the diaries and ask questions about the health of my children, there will be no more household surveys, and there will be no more clinic visits. I also understand that I will no longer receive soap, sugar, and salt each month. I understand that this applies to all of my children that have been enrolled in the cohort study.

Mark the box with **X**:

I UNDERSTAND that the cohort study is ending and that as of today my child(ren) will no longer take part in study activities: ☐

**Parent/Guardian's name:**		
**Parent/Guardian's Signature or Mark:**		**Date:**
**Cluster** [____|____]	**HH ID** [____]-[____|____]-[____|____|____|____]-[____|____]	
**Study ID for each child enrolled in the Cohort**	[____|____|____|____]	[____|____|____|____]
	[____|____|____|____]	[____|____|____|____]
	[____|____|____|____]	[____|____|____|____]

Name of person explaining information sheet:			
Signature:		Date:	

THE REPUBLIC OF UGANDA

APPENDIX II. INFORMATION SHEET
Caregivers participating in the health worker knowledge assessment
ACT PRIME Study

Introduction
Dr. Sarah Staedke and colleagues from the Uganda Malaria Surveillance Project/Infectious Diseases Research Collaboration are doing a study to evaluate activities that have been introduced at government-run health facilities in this area aiming to improve the health of children. We would like to understand the impact of these activities and why they are working, or not. This study involves several parts, including an assessment of the knowledge and skills of health workers for treating children with fever.

Why is this study being done?
As part of this study, we would like to know more about how well health workers know how to manage and treat children with fever. To do this we will ask health workers question about malaria, and ways to manage, diagnose and treat malaria. We would also like to know how well health workers have been trained to use rapid diagnostic tests (RDTs) for malaria.

What will happen today if I take part in this study?
If you agree to take part, we will watch the health worker as they interact with your child, including watching them test your child for malaria, decide the test result, and provide treatment for your child. The health worker has already agreed to be watched while they see your child. We would like you to interact with the health worker as you would normally; you are not expected to do anything differently while we are watching. After the visit with the health worker is over, we will not ask you to do anything further. The information we collect will be used by project investigators, and may be shared with other researchers and policy-makers to answer questions about how best to deliver health worker training and improve health services.

How long will the study last?
If you take part in the study, it will involve a one-time observation of your interaction with the health worker today. This should take about 30-45 minutes.

Can I stop my child from being in the study?
You can decide to stop participating at any time. Just tell our study personnel right away if you wish to stop the activities.

What risks can I expect from my child being in the study?
Participation in any research study may involve a loss of privacy. Information you provide will be recorded and we will observe the health worker testing your child's blood for malaria parasites but your name, and your child's name will not be used in any reports of the information provided. No quotes or other results arising from your child's consultation or your interaction with the health worker will be included in any reports, even anonymously, without your agreement.

The information obtained from these observations will be locked at our project offices. We will do our best to make sure that the personal information gathered for this study is kept private.

Are there benefits to taking part in the study?

There will be no direct benefit to you or your child from participating in this study. However, the information that we obtain will help researchers and policy-makers understand how best to deliver health worker training and improve health services in this area.

What other choices do I have if I do not take part in this study?

You are free to choose not to participate in the study. If you decide not to take part in this study, there will be no penalty to you. If you decide not to participate in the study your child will still be eligible to receive the best health care available at this or any other health facility today and in the future.

What are the costs of taking part in this study? Will I be paid for taking part in this study?

There are no costs to you for taking part in this study. You will not be paid for taking part in this study.

What are my rights if I take part in this study?

Taking part in this study is your choice. You may choose either to take part or not to take part in the study. If you decide to take part in this study, you may change your mind at any time. No matter what decision you take, there will be no penalty to you or to your child in any way.

Who can answer my questions about the study?

You can talk to the researchers about any questions or concerns you have about these study activities. Contact Dr. Sarah Staedke or other members of the Uganda Malaria Surveillance Project / Infectious Disease Research Collaboration on telephone number 0414-530692. If you have any questions, comments or concerns about taking part in these activities, first talk to the researchers. If for any reason you do not wish to do this, or you still have concerns about doing so, you may contact Professor James Tumwine , Makerere University Faculty of Medicine Research and Ethical Committee at telephone number 0414-533541.

Giving verbal consent to take part in the study:

You may keep this information sheet if you wish. Participation in these activities is voluntary. You have the right to decline to take part in the activities, or to withdraw from them at any point without penalty. If you do not wish to take part in the activities, you should inform the researcher now. If you do wish to take part in these activities, you should tell the researcher now, and the health worker knowledge assessment will begin shortly. If you do not agree to observations or other results arising from your participation in the study being included, even anonymously, in any reports about the study, please tell the researcher now.

APPENDIX E: USE OF RDTS FOR MALARIA IN FEVER CASE MANAGEMENT IN UGANDA - JUMP TRAINING TEST

Health centre code	Health Worker ID	Date
[____|____]	[____|____|____|____]	[____|____] / [____|____] / [____|____]<br>day month year

Position:

1 = In-charge	5 = Clinical officer	9 = Public health nurse	13 = Health assistant	17=other…
2 = Senior medical officer	6 = Nursing officer	10 = Nursing aide/assistant	14 = Health educator	
3 = Medical officer	7 = Enrolled nurse	11 = Laboratory technician	15 - Volunteer	
4 = Senior clinical officer	8 = Midwife	12 = Laboratory assistant	16 =vaccinator	[____|____]

PART 1 A: DEMOGRAPHIC INFORMATION

**1. Age**	years [____|____]	**5. What is the highest level of education or qualification achieved?**	0 = None; 1 = Primary (P1 — P7); 2 = Secondary (S1 — S6); 3 = Certificate; 4 = Diploma; 5 = Bachelor's degree; 77=Other____________; 88 = Don't know; 99 = Refused to answer [____|____]
**2.Gender**	1=Male<br>2=Female [____]		
**3. Are you originally from this area?**	1=Yes<br>2=No [____]		
**4. How long have you worked at this health centre?**	[____|____] years<br>[____|____] months	**6. What year did you graduate from your course?**	[____|____|____|____]

PART 1 B: TRAINING INFORMATION

Question	Options		Code
**7. Did you attend the ACT PRIME Training in use of RDTs for malaria in fever case management delivered by the JUMP team?**	1 = Yes<br>2 = No	88 = Don't know<br>99 = Refused to answer	[____|____]
**8. Have you attended any other training in fever/malaria case management in the last 5 years?**	1 = Yes<br>2 = No	88 = Don't know<br>99 = Refused to answer	[____|____]
**9. If yes, were any of the training sessions about use of RDTs for diagnosis of malaria?**	1 = Yes<br>2 = No	88 = Don't know<br>99 = Refused to answer	[____|____]
**10. If yes, how many RDT trainings did you attend?**			[____|____]
**11. For training #1, who organized the training?** *(Repeat Qns 11-17 for any other trainings #2,3….)*	1=MOH/District/HSD<br>2=IDRC/ACT PRIME<br>3=Malaria Consortium<br>4=PLAN<br>5=Heidi Hopkins	6=JUMP<br>77=Other_____________<br>88=Don't know<br>99=Refused to answer	[____|____]
**12. When was this training conducted (year)?**	1 = 2008<br>2 = 2009<br>3 = 2010<br>4 = 2011	5 = 2012<br>6 = 2013<br>77=Other____________<br>88=Don't know/can't remember	[____|____]
**13. How long was this training?**	1 =< 1 day<br>2 = 1-3 days<br>3 = 4-7 days<br>4 => 7 days	77=Other____________<br>88 = Don't know<br>99 = Refused to answer	[____|____]
**14. What materials were provided during and after the training?**	1 = MoH user's manual<br>2 = Job aide<br>3=Other user's manual	77=Other____________<br>88 = Don't know<br>99 = Refused to answer	[____|____]
**15. Did you receive any supervision following this training?**	1 = Yes<br>2 = No	88 = Don't know<br>99 = Refused to answer	[____|____]
**16. If yes, how many times were you supervised?**			[____|____]
**17. Who came for the supervision?**	1=MOH/District/HSD<br>2=IDRC/ACT PRIME<br>3=Malaria Consortium<br>4=PLAN	5=Dr. Heidi's team<br>6=JUMP<br>77=Other_____________<br>88=Don't know<br>99=Refused to answer	[____|____]

**18. What was done during the supervision? Select all that apply**	1=I was observed performing an RDT for malaria<br>2=I was re-taught the steps of performing an RDT<br>3=I was given feedback on areas I need to improve<br>4=OPD records were checked	5=we discussed how to organize the working area<br>6=We discussed patient care in general<br>77-Other<br>88-Don't know<br>99-Refused to answer	[____|____]<br>[____|____]<br>[____|____]
**19. Did you have access to RDTs at your health center after the RDT training?**		1=Yes<br>2=No<br>77=Other<br>88=Don't know/Can't remember<br>99=Refused to answer	[____|____]

PART 2 A : QUESTIONNAIRE INSTRUCTIONS

It is now time to take the written test

Please take a moment to answer the following questions. You may/may not have done this test in the past; however, you do not need to worry if you don't know every answer.

This **TEST** will not affect your role or responsibilities and **WE VALUE THE TIME YOU TAKE** to complete this test. There is no need to write your name on this test. **All responses will be kept strictly confidential.**

GENERAL INSTRUCTIONS TO COMPLETE THE JUMP TRAINING TEST

1. *Please use a dark coloured pen to fill out the test.*
2. *The health worker identity (ID) number is the unique number that was given to you at the start of this PRIME project. You are asked to enter your ID number like this at the top of each page of your questionnaire; you may refer to the top of page 1 for your Health worker ID. Please fill the boxes like this:* [_0_ | _5_ | _1_ | _0_]
3. *In this questionnaire, we ask you to read each question carefully at the questions and decide which response you believe is correct. When you have decided, please circle the letter in the column beside that response. For example:*

**1. Today is Monday**	(a) True	b) False

4. *If you change your mind and would like to circle a different response, please cross out your original choice and circle the choice that you believe is correct. For example, if you change your mind and you decide that your answer is 'false', cross through the original and circle the new response as below:*

**1. Today is Monday**	~~(a)~~ True	(b) False

PART 2B: QUESTIONS

**1**	**All cases of fever in Uganda are caused by malaria.**	a) True b) False
**2**	**RDT stands for Rapid Diagnostic Test for malaria. Which of the following is NOT true about RDTs?**	a) If performed correctly, they are very accurate in diagnosing which patients have malaria and which do not.<br>b) They can be performed in health centres that do not have power or laboratory equipment.<br>c) They can detect typhoid fever and pneumonia as well as malaria.
**3**	**In order to perform an RDT, you will need to use finger prick blood. After you prick a patient's finger with a lancet, what should you do with the lancet?**	a) Save it to wash and use again later.<br>b) Carefully put it immediately in the sharps container.<br>c) Put it on the table, to use for the next patient.

4	**We have provided pictures of RDTs on a computer labelled 1-10, look at each picture and interpret the result. For each test kit, choose from the options below;**<br>a) Positive<br>b) Negative<br>c) Positive – Pf/Pan<br>d) Invalid	**Test no.**	**Result (choose option a-d)**
		**1**	
		**2**	
		**3**	
		**4**	
		**5**	
		**6**	
		**7**	
		**8**	
		**9**	
		**10**	
5	**RDTs should be stored in a cool, dry place.**	a) True<br>b) False	
6	**You can use the same RDT for two different patients.**	a) True<br>b) False	
7	**You should always check the expiry date on the package of an RDT before using it.**	a) True<br>b) False	
8	**For which of the following patients should you perform an RDT for malaria?**	a) A 4-year-old boy with fever and mild cough<br>b) An 8-month-old child with fever who refuses to breastfeed, is breathing rapidly and has very pale palms and sunken eyes<br>c) A 20-year-old woman who is pregnant and has fever<br>a) A 32-year-old man who is HIV-positive and has fever<br>b) A 45-year-old man with fever and joint paints<br>c) All of the above	
9	**A patient comes to your health centre with fever. She has taken chloroquine for two days but the fever has persisted. You perform an RDT and the result is positive. What should you do?**	a) Tell the patient to complete the course of chloroquine.<br>b) Prescribe a full course of Coartem.	
10	**A patient comes to your health centre with fever. She took a complete, correct course of Co-artem last week. She did not vomit any of the doses. You perform an RDT and the result is positive. What should you do?**	a) Prescribe a full course of quinine.<br>b) Prescribe a second full course of Coartem.<br>c) Advise her to take Panadol and fluids only.	

**11**	**Which of the following is a symptom or sign of severe illness in a patient with fever?**	a) Convulsions currently or within the past 2 days<br>b) Extreme weakness – patient is unable to sit or stand without support<br>c) Severe anaemia – patient's palms and conjunctivae are very pale<br>d) Unable to drink or breastfeed<br>e) All of the above
**12**	**A 35-year-old man comes to your health centre. He tells you that he has fever and muscle aches. What should you do FIRST?**	a) Prescribe Coartem immediately.<br>b) Prescribe chloroquine immediately – this regimen is best for adults.<br>c) Prescribe Panadol only and tell the patient to go home and rest.<br>d) Ask the patient how long he has had these symptoms, and ask if he has taken any drugs at home before coming to the health centre.
**13**	**A mother brings her 8-year-old daughter to your health centre. Beginning yesterday, the girl has had fever and no appetite. Her mother has given Panadol and fluids, but the symptoms continue. There are no signs or symptoms of severe illness. On physical examination, you find no obvious cause of her fever. You perform an RDT, and the result is negative. Which of the following is TRUE?**	a) This patient has a mild febrile illness, but the cause is not clear. The mother should continue to provide fluids and Panadol. You should advise her to bring the girl back to the health centre if the symptoms do not get better within 2 to 3 more days.<br>b) You should prescribe a course of chloroquine. Even though the RDT is negative, the girl may still have malaria, and chloroquine is appropriate for mild cases of malaria.<br>c) You should prescribe Coartem. Even though the RDT is negative, the girl may have malaria, and Coartem is the first-line regimen for malaria in Uganda.
**14**	**A 10-year-old boy comes to your health centre with fever and joint pains. He looks weak, but is able to sit up easily by himself. After you take a history and do a physical examination, you perform an RDT. The RDT is positive, and you plan to treat for uncomplicated malaria. According to the Uganda Ministry of Health guidelines, which of the following is the BEST (first-line) treatment for this patient?**	a) Chloroquine<br>b) Oral quinine<br>c) Injectable quinine<br>d) Coartem (artemether-lumefantrine)<br>e) Chloroquine + Fansidar (Homapak)
**15**	**A mother brings her 1-year-old son to your health centre. She tells you he has not been feeding well. The child's body is hot to touch. He is weak and cannot sit up by himself, and he is breathing very rapidly. What should you do? Be sure to read all the statements below before answering.**	a) Undress the child, and ask the mother to sponge him to help reduce the fever.<br>b) Immediately prepare and inject a dose of quinine AND antibiotic.<br>c) Perform an RDT and write the result on the referral note.<br>d) Write a referral note and ensure that the child is referred to a Health Centre IV or Hospital as soon as possible.<br>e) All of the above.

PART 3: SKILLS ASSESSMENT

Explain to the health worker that you would like to observe them performing a RDT to test for malaria

- Ask if there is a child with suspected malaria that the health worker plans to test for malaria with a RDT
- If no child is available, ask the health worker to contact you by telephone when they next plan to test a child using an RDT
- Reassure the health worker that the information gathered during the assessment will be used to help improve training in use of RDTs
- Reassure the health worker that their individual performance will not negatively affect their employment or entitlement to benefits
- Use the checklist below to record the health worker's performance

RDT USER CHECKLIST

**1. What type of RDT is used?**	1=SD Bioline (Pf only)<br>2=SD Bioline (Pf and Pan)	3=Pf only (specify)______________________<br>4=Pf/pan (specify)______________________<br>77=other (specify)______________________	[____|____]
**Preparation phase: Does the health worker…?**			
**2. Prepare all supplies before starting the procedure?**	1 = Yes, prepares all supplies<br>2 = Yes, but some supplies are missing<br>3 = No, doesn't prepare supplies	66 = Not observed<br>77 = Other (specify)<br>_________________________________	[____|____]
**3. Check the expiry date of the RDT?**	1 = Yes<br>2 = No	88 = Don't know<br>99 = Refused to answer	[____|____]
**4. Read or consult the instructions prior to or while performing the RDT?**	1 = Yes, from the paper in the box<br>2 = Yes, from the poster on the wall<br>3 = Yes, from training material	4 = No, does not read instructions<br>66 = Not observed<br>77 = Other (specify)<br>_________________________________	[____|____]
**5. Check desiccant sachet for colour change?**	1 = Yes<br>2 = No	66 = Not observed<br>77 = Other (specify)<br>_________________________________	[____|____]
**6. Write down information to identify the patient on the RDT?**	1 = Yes, patient's name or initials<br>2 = Yes, patient's ID number<br>3 = No, no information recorded	66 = Not observed<br>77 = Other (specify)<br>_________________________________	[____|____]
**7. Wear gloves?**	1= Yes, new glove on one hand<br>2= Yes, new gloves on both hands<br>3= Yes, same glove(s) from previous patient	4 = No, does not wear any gloves<br>66 = Not observed<br>77 = Other (specify)<br>_________________________________	[____|____]
**8. Which finger does the health worker choose to prick?**	1 = Middle finger/Ring finger<br>2 = Index Finger<br>3 = Little finger/Thumb	66 = Not observed<br>77 = Other (specify)<br>_________________________________	[____|____]
**9. Clean the fingertip?**	1 = Yes, with alcohol swab<br>2 = Yes, with other disinfectant<br>3 = Yes, but with no disinfectant	4 = Did not clean at all<br>66 = Not observed<br>77 = Other (specify)<br>_________________________________	[____|____]
**Finger prick**			
**10. Does the health worker use a good finger prick technique to obtain adequate blood drop?**	1 = Yes<br>2 = No	66 = Not observed<br>77 = Other (specify)<br>_________________________________	[____|____]
**11. What does the health worker use to perform a finger prick?**	1 = lancet<br>2 = needle	66 = Not observed<br>77 = Other (specify)<br>_________________________________	[____|____]
**12. What does the health worker do with the item she/he uses for pricking?**	1 = Put it into special sharps box<br>2 = Put it into a plastic bottle<br>3 = Put it into the RDT package and into the rubbish bin/bag	4=Put it directly into the rubbish bin/bag without packaging<br>66=Not observed<br>77=Other(specify)<br>____________________	[____|____]
**13. When does the health worker dispose of the item used to perform the finger prick?**	1=Immediately after stabbing the finger<br>2=After obtaining blood but before adding the buffer<br>3=After adding the buffer, as he/she waits for the results	4=Disposes after recording the results<br>66=Not observed<br>77=Other(specify)<br>____________________	[____|____]

Finger prick (continued)			
**14. How does the health worker transfer blood from the patient to the RDT?**	1=Using a pipette from RDT kit<br>2=Using a loop from the RDT kit<br>3=Using a cupped loop from the RDT kit<br>4=Drops blood directly from the finger onto the RDT	5=Using a syringe<br>66 = Not observed<br>77 = Other (specify)<br>______________________	[____|____]
**15. How much blood is transferred to the RDT?**	1 = Appropriate amount of blood<br>2 = Less blood<br>3 = Too much blood<br>4 = Cannot quantify	66 = Not observed<br>77 = Other (specify)<br>______________________	[____|____]
**Using the RDT**			
**16. How many drops of buffer solution does the health worker add?**	Record number of drops	66 = Not observed	[____|____]
**17. Does the health worker place the RDT flat on the table and hold bottle vertically to dispense the buffer?**	1 = Yes<br>2 = No	66 = Not observed<br>77 = Other (specify)<br>______________________	[____|____]
**18. Does the health worker use a clock or watch to time the duration of the RDT?**	1 = Yes<br>2 = No	66 = Not observed<br>77 = Other (specify)<br>______________________	[____|____]
**19. How many minutes does the health worker wait before reading the test and determining the result?**	Record number of minutes	66 = Not observed	[____|____]
**20. How many RDTs does the health worker use on one patient?**	Record number of RDT test kits used		[____|____]
**Interpreting the test results**			
**21. What does the health worker tell the patient/caregiver about the result of the test?** *(Record exactly what was said below, then categorize the response using the options to the right – there can be more than one response.)*	1=Positive, falciparum malaria<br>2=Positive, species not specified<br>3=Positive, non-falciparum malaria<br>4=Positive, mixed species (more than one type of malaria)<br>5=Negative, no malaria	6=Little/scanty malaria present<br>7=Too much malaria<br>8=Did not tell the patient the result of the RDT<br>9=Test was invalid<br>66 = Not observed<br>77 = Other (specify)<br>______________________	[____|____]<br>[____|____]<br>[____|____]
**Health worker's statement:**			

Information given			
**22. What information or advice does the health worker give to the patient/caregiver?** *(Choose all that apply)*	1 = complete the prescribed drugs<br>2 = do not share the prescribed medication with another patient<br>3 = take AL with milk or eat a fatty meal before taking the medication<br>4 = Sleep under an ITN<br>5 = Bring the child back for review if there is no improvement	6 = repeat the dose if child vomits within 30 minutes of giving the medication<br>7 = Return for another dose if child vomits any of the doses<br>8 = No advice given<br>66 = Not observed<br>77 = Other (specify)<br>________________________________	[____|____]<br>[____|____]<br>[____|____]
**Treatment**			
**23. Review the prescription and record medications prescribed** *(Record all that are written)*	1 = Panadol<br>2 = AL (Coartem, Lumartem)<br>3 = DP (Duocotecxin)<br>4 = Quinine<br>5 = Chloroquine<br>6 = SP (Fansidar)<br>7 = CQ+SP combination<br>8 = Amodiaquine (Camoquin)	10 = Amoxacillin<br>11 = Septrin<br>12 = Iron (Ferrous)<br>13 = ORS<br>14 = Vitamin A<br>15= none<br>16=Metronidazole<br>17=Procaine Penicillin Fortified(PPF)<br>66= Not observed<br>77= Other (specify)________________<br>88 = Don't know	[____|____]<br>[____|____]<br>[____|____]<br>[____|____]<br>[____|____]
**HMIS – Recording information**			
**24. Does the health worker write down any information in the OPD or other HMIS register?**	1 = Yes<br>2 = No	66 = Not observed<br>77 = Other (specify)<br>________________________________	[____|____]

Thank you!

Staff ID: [____|____]
