# Supplementary material for: The impact of an intervention to introduce malaria rapid diagnostic tests on fever case management in a high transmission setting in Uganda: A mixed-methods cluster-randomized trial (PRIME)
Source: PLoS One. 2017 Mar 13;12(3):e0170998. doi: 10.1371/journal.pone.0170998 (PMC5347994; doi:10.1371/journal.pone.0170998)
Supplement: S3 File — V1.1, 23 February 2011. Original version of the PROCESS protocol. This file is a copy of the original protocol of the PROCESS study which ran alongside the PRIME cluster-randomized trial, which was approved by the overseeing ethics committees. (PDF) [file pone.0170998.s003.pdf]

The ACT PROCESS Study

Evaluating the process, context, and impact of interventions to enhance health facilities in Tororo, Uganda

23 February 2011
Protocol Version 1.1

Principal Investigator: Dr. Sarah Staedke
Co-investigators: Dr. Moses Kamya, Dr. Clare Chandler

Funding Source: Gates Foundation, ACT Consortium, LSHTM
Makerere University Research and Ethical Committee:
Ugandan National Council for Science and Technology:
London School of Hygiene & Tropical Medicine Ethics Committee: 5831

ACT CONSORTIUM

THE REPUBLIC OF UGANDA

UCSF

CONTENTS

STUDY INFORMATION **4**
PROJECT SYNOPSIS **5**
PROJECT TEAM AND PARTICIPATING SITES **6**
ABBREVIATIONS AND ACRONYMS **8**

1 BACKGROUND **9**
1.1. Malaria in Uganda 9
1.1.1. Barriers to diagnosing and treating malaria *9*
1.1.2. Improving quality of care through interventions *9*
1.2. The ACT PRIME study 10
1.3. Evaluating interventions 11
1.3.1. Comprehensive evaluation *11*

2 RATIONALE **13**

3 STUDY OBJECTIVES **14**
3.1. Primary objective 14
3.2. Secondary objectives 14

4 COMPREHENSIVE EVALUATION FRAMEWORK **15**
4.1. Overview 15
4.2. Logic model 16
4.3. Process evaluation 17
4.3.1. Documentation of implementation *17*
4.3.2. Assessment of intervention mechanisms *18*
4.4. Context evaluation 18
4.4.1. Structured contextual record *18*
4.4.2. Rich description of context *19*
4.5. Impact evaluation 19
4.5.1. Assessment of hypothesised impacts *19*
4.5.2. Assessment of undetermined impacts *20*

5 STUDY PROCEDURES **21**
5.1. Overview 21
5.2. Study site 22
5.3. Study population 22
5.4. Self-filled questionnaires 23
5.5. Health worker communication assessments and patient exit interviews 24
5.6. In-depth interviews 25
5.7. Semi-structured questionnaires 25
5.8. Focus group discussions 26
5.8.1. Primary caregiver FGDs *26*
5.8.2. Community health worker FGDs *27*
5.9. Structured contextual record 27
5.10. Supply of drugs and RDTs 28

6 DATA MANAGEMENT **29**
6.1. Data management 29
6.1.1. Quantitative data *29*
6.1.2. Qualitative data *29*
6.2. Quality assurance and quality control 30
6.3. Records and storage 30

6.4. Data sharing 31

7 ANALYTICAL PLAN 32
7.1. Quantitative data 32
7.2. Audio recordings 32
7.3. Qualitative data 32

8 PROTECTION OF HUMAN PARTICIPANTS 33
8.1. Institutional Review Boards 33
8.2. Informed consent process 33
8.2.1. Self-filled questionnaires 34
8.2.2. Health worker communication assessments and patient exit interviews 34
8.2.3. In-depth Interviews 34
8.2.4. Semi-structured questionnaires 34
8.2.5. Focus group Discussions 34
8.2.6. Supply of drugs and RDTs 35
8.3. Confidentiality 35
8.4. Risks and discomforts 35
8.4.1. Privacy 35
8.4.2. Compensation 35
8.4.3. Alternatives 36

9 REFERENCES 37

10 APPENDICES 40

STUDY INFORMATION

**Title**	**PROCESS Study – Evaluating the process, context, and impact of interventions to enhance health facilities in Tororo, Uganda**
**Principal investigator**	Sarah Staedke, MD, PhD<br>Clinical Senior Lecturer, London School of Hygiene and Tropical Medicine<br>Uganda Malaria Surveillance Project / Infectious Diseases Research Collaboration, Uganda
**Co-investigators**	Moses Kamya, MBChB, MPH, PhD; Professor, Makerere University<br>Clare Chandler, PhD; Lecturer, London School of Hygiene and Tropical Medicine
**Sponsor**	London School of Hygiene & Tropical Medicine<br>Department of Clinical Research<br>Faculty of Infectious and Tropical Diseases<br>Keppel Street, London WC1E 7HT<br>United Kingdom
**Funding agency**	ACT Consortium through a grant from the Bill & Melinda Gates Foundation to the London School of Hygiene and Tropical Medicine
**Participating institutions**	London School of Hygiene & Tropical Medicine, UK<br>Infectious Diseases Research Collaboration, Kampala, Uganda
**Institutional review boards**	Makerere University Research and Ethics Committee<br>Uganda National Council for Science and Technology<br>London School of Hygiene & Tropical Medicine

PROJECT SYNOPSIS

**Title**	**PROCESS Study – Evaluating the process, context, and impact of interventions to enhance health facilities in Tororo, Uganda**
**Description**	The study proposed here, ACT PROCESS, is a comprehensive evaluation to further our understanding about the outcomes of the ACT PRIME study in which an intervention will be implemented in lower level government run health centers in Tororo, Uganda. The aim of the PRIME health facility intervention (HFI) is to 1) improve health center management; 2) provide health worker training; and 3) stabilize supplies of drugs and rapid diagnostic tests (RDTs) for malaria.
**Study Design**	ACT PROCESS consists of a comprehensive evaluation framework to evaluate the process, context and impact of the ACT PRIME intervention. The evaluation framework includes: 1) a logic model to detail the components, effects and intended outcomes of the HFI; 2) a process evaluation to document the implementation of the HFI activities from the perspective of implementers, health workers, community members, and key stakeholders; 3) a context evaluation to capture information on factors that may have affected the HFI implementation or outcomes; and 4) an impact evaluation to assess the wider impact of the HFI beyond outcomes of the ACT PRIME study. These evaluation components will be assessed using self-filled questionnaires, health worker communication assessments and patient exit interviews, in-depth interviews and semi-structured questionnaires, focus group discussions, and a structured contextual record.
**Study site**	In Tororo District, the five sub-counties of West Budama North Health Sub-district (Nagongera, Paya, Kirewa, Kisoko, and Petta), and two sub-counties of West Budama South Health Sub-district (Mulanda and Rubongi) will be included.
**Primary objective**	To evaluate the process, context and impact of the HFI in the ACT PRIME study to further our understanding about why the HFI was effective, or not.
**Secondary objectives**	1. To develop a comprehensive logic model of the ACT PRIME HFI with intervention components mapped through to their intended effects and outcomes.<br>2. To evaluate the process of the HFI implementation including health worker training, health center management tools, supply of artemether-lumefantrine (AL) and rapid diagnostic tests (RDTs) for malaria, and interactions with local and district stakeholders.<br>3. To develop a rich contextual record of factors that may have affected the HFI implementation outcomes such as other interventions or programmes implemented in the area, changes to malaria case management guidelines, and other environmental, economic, political or individual factors.<br>4. To assess the wider expected and unexpected impacts of the HFI at the household, community, public health system, and private sector levels.
**Target population**	**Self-filled questionnaires:** all trainers and participants for each HFI training module; up to 350 self-filled questionnaires.<br>**Health worker communication assessments and patient exit interviews:** at least one health worker in the 20 health centers included in ACT PRIME; recording interactions with caregivers and conducting patient exit interviews with 3-5 patients per health worker at three time points; up to 125 assessments and interviews per time point, and up to 375 total.<br>**In-depth interviews:** selected HFI implementers, health workers in the HFI arm, and key local and district stakeholders; up to 25 in-depth interviews.<br>**Semi-structured questionnaires:** health workers in both the HFI and standard care arms, and private drug shops; up to 30 questionnaires.<br>**Focus group discussions:** selected primary caregivers and community health workers from the study area; up to 16 focus group discussions.
**Study period**	Implemented in parallel with the ACT PRIME study for approximately 1 ½ years.

PROJECT TEAM AND PARTICIPATING SITES

INVESTIGATORS

Sarah Staedke, MD, PhD
Role in project: Principal investigator
Clinical Senior Lecturer, London School of Hygiene and Tropical Medicine, London, UK
Infectious Disease Research Collaboration / Uganda Malaria Surveillance Project, Kampala, Uganda
Email: sarah.staedke@lshtm.ac.uk

Moses Kamya, MBChB, MPH, PhD
Role in project: Co-investigator
Professor, Department of Medicine, Makerere University, Kampala, Uganda
Infectious Disease Research Collaboration / Uganda Malaria Surveillance Project, Kampala, Uganda
Email: mkamya@infocom.co.ug

Clare Chandler, PhD
Role in project: Co-investigator
Senior Lecturer, London School of Hygiene and Tropical Medicine, London, UK
Social Scientist, ACT Consortium
Email: clare.chandler@lshtm.ac.uk

COLLABORATORS

Grant Dorsey, MD, PhD
Role in project: Collaborator
Associate Professor, Department of Medicine, University of California, San Francisco
Infectious Disease Research Collaboration / Uganda Malaria Surveillance Project, Kampala, Uganda
Email: gdorsey@medsfgh.ucsf.edu

Heidi Hopkins, MD, MPH
Role in project: Collaborator
FIND Diagnostics, Kampala, Uganda
Infectious Disease Research Collaboration / Uganda Malaria Surveillance Project, Kampala, Uganda
Email: hhopkins@medsfgh.ucsf.edu; heidi.hopkins@finddiagnostics.org

Anne Gasasira, MBChB, PhD
Role in project: Collaborator
Infectious Disease Research Collaboration / Uganda Malaria Surveillance Project, Kampala, Uganda
Email: agasasira@gmail.com

KEY PROJECT PERSONNEL

Personnel	Institution	Role
Susan Nayiga	Infectious Diseases Research Collaboration, Kampala	Social Scientist / Epidemiologist
Lilian Taaka	Infectious Diseases Research Collaboration, Kampala	Social Scientist
Christine Nabirye	Infectious Diseases Research Collaboration, Kampala	Social Scientist
Deborah DiLiberto	London School of Hygiene & Tropical Medicine, UK	Research Assistant

PARTICIPATING SITES

Uganda Malaria Surveillance Project (UMSP)

Address: Infectious Disease Research Collaboration (IDRC), Mulago Hospital Complex, Malaria House, P.O. Box 7475, Kampala, Uganda
Contact Person: Catherine Tugaineyo
Phone Number: +256 (0) 414-530692
Fax Number: +256 (0) 414-540524
Email: ctugaineyo@muucsf.com

London School of Hygiene & Tropical Medicine (LSHTM)

Address: Keppel Street, London, WC1E 7HT, UK
Contact Person: Susan Sheedy
Phone Number: +44 (0) 20 7927 2256
Fax Number: +44 (0)20 7637 4314
Email: susan.sheedy@lshtm.ac.uk

ABBREVIATIONS AND ACRONYMS

ACT	artemisinin-based combination therapy
AL	artemether-lumefantrine
CHW	community health worker
FGD	focus group discussion
FOMREC	Faculty of Medicine Research Ethics Committee, Makerere University
HFI	health facility intervention
HMM	home management of malaria
HW	health worker
IDI	in-depth interview
IRB	institutional review board
LSHTM	London School of Hygiene and Tropical Medicine
MCPP	Measuring Patient-Centered Communication
MoH	Ministry of Health
MU	Makerere University (Kampala, Uganda)
MU-UCSF	Makerere University - UCSF Malaria Research Collaboration
M&E	monitoring and evaluation
PCS	patient-centered services
RDT	rapid diagnostic test (for malaria)
SFQ	self-filled questionnaire
SOP	standard operating procedure
SSQ	semi-structured questionnaire
UMSP	Uganda Malaria Surveillance Project
UNCST	Uganda National Council of Science and Technology
WHO	World Health Organization

1 BACKGROUND

1.1. MALARIA IN UGANDA

Malaria remains one of the most serious global health problems [1]. Of the estimated 400 to 900 million episodes of fever that occur each year in African children, probably about half are due to malaria, resulting in over one million deaths [2-4]. In Uganda, malaria is one of the most important health problems and the leading cause of morbidity and mortality in children, accounting for up to 40% of outpatient visits, 20% of hospital admissions, and 14% of inpatient deaths [5]. Children in Uganda experience an estimated average of six episodes of malaria each year, resulting in between 70,000 and 110,000 deaths annually. Up to 90% of Uganda's population lives in highly endemic areas with perennial malaria transmission, while 10% live in areas at risk for epidemics [6].

1.1.1. Barriers to diagnosing and treating malaria

Diagnosis and treatment of malaria can be straightforward; however, it is often challenged by limited health-care infrastructure, particularly in Africa [7-8]. Substantial barriers to providing good quality health care exist, including logistical, cultural, and wider system barriers. As a result, few malaria patients receive treatment in the formal healthcare sector; most are treated at home with drugs purchased from informal drug shops [2, 9]. Unfortunately, such treatment is often inadequate, with ineffective or poor quality drugs given at incorrect doses [9-11]. Addressing these barriers and providing quality health care for malaria, and other illnesses that is safe, effective, patient-centered, timely, efficient and equitable is a necessity; however, evidence from increasing numbers of studies suggests quality of care by these measures is poor in many settings, including delivery of primary care in low-income countries. Direct observation studies of performance have identified severe deficiencies, particularly in history taking and examinations, diagnosis, and appropriate treatment choice and dosage [12-17]. This has been linked to low motivation of staff as well as poor resource availability in the work place. In terms of patient-centeredness and timeliness, meeting a population's expectations of how they should be treated by providers, including patient expectations for health care, is now seen as central to performance [18]. It has been argued that poor quality services fail to earn the population's trust, leading to clients seeking alternative sources of care [19], or discontinuing care [20]. In contrast, the perception of good quality services, including inter-personal relationships, has been found to encourage patients to access care [21], and demand for services [22-24]. Satisfied patients may be more likely to comply with treatment and maintain a continuing relationship with the health worker [25], and loyalty to a clinic [26], thus enjoying a better medical prognosis (presuming good technical quality of care) [27].

1.1.2. Improving quality of care through interventions

Interventions to improve quality of care in low-resource settings have largely fallen into two categories: resource-based interventions and performance-based interventions. Resource-based interventions include the provision of equipment, infrastructure and drugs. Performance-based interventions have mostly been focused on clinical training and dissemination of guidelines. Far fewer studies have assessed interventions to improve aspects of quality care outside of clinical care. The ACT PRIME study being conducted in Tororo, Uganda, on which the ACT PROCESS study proposed here is based, aims to improve quality of care at lower level government-run health

centers by implementing a health facility intervention which incorporates both resource-based and performance-based components.

1.2. THE ACT PRIME STUDY

In the ACT PRIME study, enhanced health facility care will be compared to the current standard of care provided by lower level government-run health facilities, supplemented by services provided through the private sector and community-based interventions, using a cluster-randomized design. There will be 20 health centers randomized to each study arm: 10 health centers in the health facility intervention arm and 10 health centers in the standard care arm. ACT PRIME began in December 2010, and the intervention will be rolled-out in March 2011. The objectives and outcomes of ACT PRIME are provided in Table 1.1 below.

Table 1.1 ACT PRIME objectives and outcomes

Objective	Primary outcome	Secondary outcomes
1. To compare the impact of enhanced health facility-based care to current standard of care on key population-based indicators in children under five.	Prevalence of anaemia	- Prevalence of parasitemia<br>- Prevalence of gametocytemia<br>- All-cause mortality rate in children under five
2. To compare the impact of enhanced health facility-based care to current standard of care on key longitudinal indicators, in a cohort of children under five.	Antimalarial treatment incidence density	- Incidence of hospitalizations,<br>- Illness and febrile illness episodes<br>- Prompt effective treatment of fever<br>- Prompt effective treatment of malaria<br>- Incidence of serious adverse events
3. To compare impact of enhanced health facility-based care to current standard of care on key indicators of case management for malaria and other illnesses, in children under five treated at health facilities.	Inappropriate treatment of malaria	- Appropriate treatment of malaria, patient satisfaction<br>- Patient attendance, gaps in staffing<br>- Drug stock outs<br>- Health worker knowledge questionnaire scores

The health facility intervention (HFI) will be comprised of three components: 1) health center management training, 2) health worker training, including fever case management and patient-centered services, and 3) supply of consumables, including malaria diagnostics and antimalarial drugs. The goal of these components is to address the barriers to providing good quality care identified in our formative research. By addressing these barriers, ACT PRIME aims to provide good quality care as defined by health workers and community members in Tororo district, attracting them to health facilities and improving the case management of malaria and non-malarial febrile illnesses received when they attend facilities. The intervention package will be rolled out to all health centers randomized to the HFI over approximately 8-10 weeks. Some activities will continue to be supported by the project for the duration of the study. ACT PRIME aims to implement an intervention which is sustainable and reproducible by the MoH in Uganda, working within the existing government systems in conjunction with the MoH and district teams.

This study, ACT PROCESS, is a parallel study intended to comprehensively evaluate the complex interventions of ACT PRIME being conducted in Tororo District, Uganda.

1.3. EVALUATING INTERVENTIONS

Research has shown that simple interventions such as basic training or health education have had limited effect on changing provider behaviour [28-29] or community behaviour [30]. The results from our formative research, the Tororo District Survey Project, echo these findings: the situation of providing and seeking health care whether in health facilities or in communities is far more complex, involving a range of actors, motivations, habits and logistics [31]. Achieving a change in behaviour requires complex interventions that address the multiple factors involved with access to appropriate treatment [32-33]. Evaluating the complexities of the intervention using a systematic approach is key to understanding *if*, *how* and *why* the intervention functioned.

Many authors and institutions are now arguing for more comprehensive evaluations of complex interventions that include a focus on process, context and impact [34]. Such comprehensive evaluations have been uncommon, and those that have existed alongside randomized controlled trials have been critiqued for poor integration with quantitative findings and methodological limitations [35], prompting the challenge for more carefully planned evaluations. We adopt a 'realist evaluation' approach to our study: to contribute to broader knowledge of 'What works for whom in what circumstances and in what respects, and how?' [36]. This involves understanding mechanisms of change by mapping out the intended intervention programme and contrasting this with the reality of implementation, analysing local interpretations of intervention effects, mapping and interpreting contextual influences and assessing impact within and outside of intended consequences of the intended intervention.

1.3.1. Comprehensive evaluation

Comprehensive evaluation can be considered in four components illustrated in Figure 1.1.

Figure 1.1 Framework for qualitative evaluation

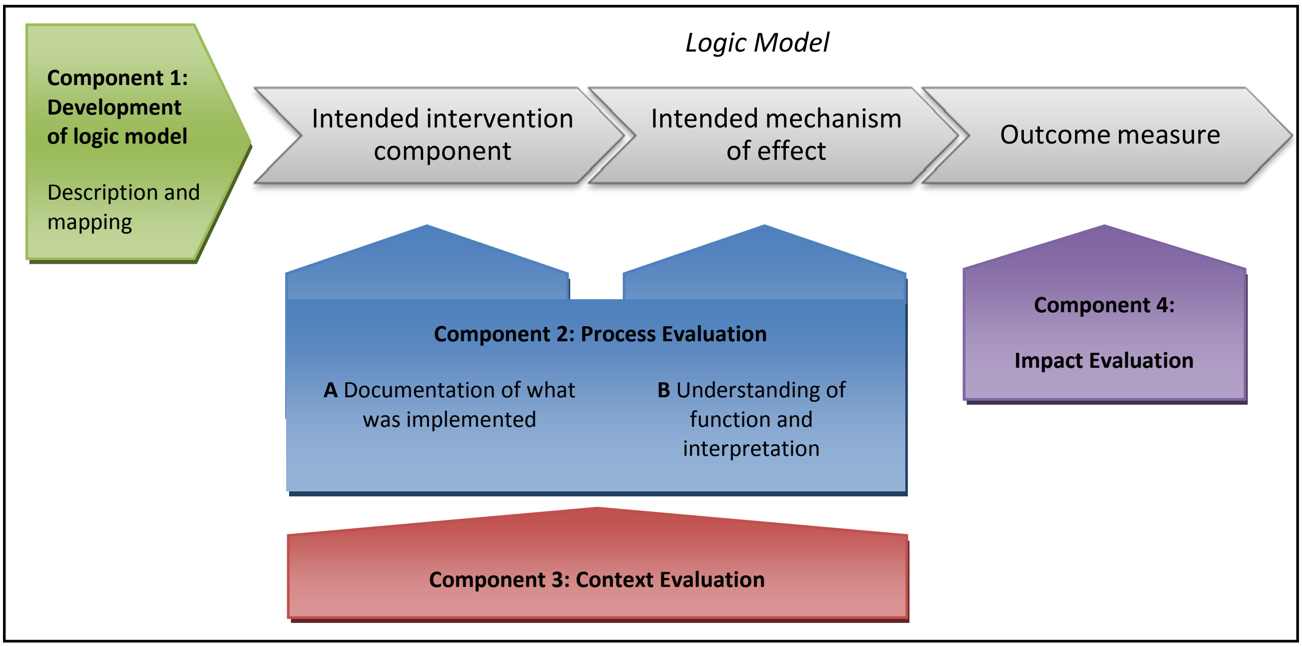

Component 1, *Development of logic model,* maps the intended pathway between intervention activities and outcomes, highlighting the mechanisms by which the intervention is intended to take effect and the assumptions that underlie each mechanism [36]. Once the intended intervention is mapped, it is then possible to identify factors that may explain the study outcomes.

Component 2, *Process Evaluation,* documents (a) how the intervention is implemented in reality, assessing this against the map of the planned intervention [37] and (b) how the intervention activities being implemented are functioning and being perceived, including whether intended mechanisms worked as planned [38].

Component 3, *Context Evaluation,* documents the context of the intervention process both in terms of the reasons that the implementation of the intervention occurs as it does in reality and how the intervention is interpreted and accommodated. The context documentation involves local factors as well as wider factors, including those outside of the PRIME study [39].

Component 4, *Impact Evaluation,* attempts to understand the depth and breadth of the impact of the intervention [40]. Specific outcome measurements are predicted and are measured quantitatively. However, the impact may be more far reaching and is likely to depend upon the way the intervention was implemented, interpretations of the intervention and how it is adopted as well as the local and broader context. It is therefore important that these other components are used to interpret any other outcomes and impacts of the intervention.

2 RATIONALE

The ACT PROCESS study proposed here is designed to evaluate the process, context and impact of the intervention implemented in the ACT PRIME study in order to further our understanding about why the HFI was effective, or not. This will be achieved through a comprehensive evaluation framework implemented in parallel with ACT PRIME.

ACT PROCESS consists of four linked evaluation components including: 1) logic model, 2) process evaluation, 3) context evaluation, 4) impact evaluation. The logic model is developed alongside the HFI intervention design stage and aims to detail the components, effects and intended outcomes of the HFI. The logic model informs the development of the data collection tools for the remaining components of the evaluation. The process evaluation will document the process of implementing the HFI including health worker training activities, health center management tools, supply of artemether-lumefantrine (AL) and rapid diagnostic tests (RDTs) for malaria, and interactions with local and district stakeholders from the perspective of implementers, health workers and community members. The context evaluation will capture information on factors that may have affected the HFI implementation or outcomes including other interventions or programmes implemented in the area, changes to malaria case management guidelines, and other environmental, economic, political or individual factors. The impact evaluation will assess the wider impact of the HFI beyond outcomes of ACT PRIME at the household, community, private sector, and public health system levels.

To facilitate our understanding about why the HFI was effective or not, links will be made between the clinical and economic outcomes of the ACT PRIME study and the process, context and impact outcomes of the ACT PROCESS study. This understanding is essential for interpreting and informing the development of a health facility intervention which is sustainable and reproducible by the MoH in Uganda, and elsewhere.

3 STUDY OBJECTIVES

3.1. PRIMARY OBJECTIVE

To evaluate the process, context and impact of the HFI in the ACT PRIME study in order to further our understanding about why the HFI was effective, or not.

3.2. SECONDARY OBJECTIVES

1. To develop a comprehensive logic model of the ACT PRIME HFI with intervention components mapped through to their intended effects and outcomes.

2. To evaluate the process of the HFI implementation including health worker training, health center management tools, supply of AL and RDTs for malaria, and interactions with local and district stakeholders.

3. To develop a rich contextual record of factors that may have affected the HFI implementation outcomes such as other interventions or programmes implemented in the area, changes to malaria case management guidelines, and other environmental, economic, political or individual factors.

4. To assess the wider expected and unexpected impacts of the HFI at the household, community, private sector, and public health system levels.

4 COMPREHENSIVE EVALUATION FRAMEWORK

4.1. OVERVIEW

Figure 4.1 illustrates the comprehensive evaluation framework for the study proposed here including the process, context and impact evaluation as they relate to the ACT PRIME study HFIs.

Figure 4.1 ACT PROCESS study comprehensive evaluation framework

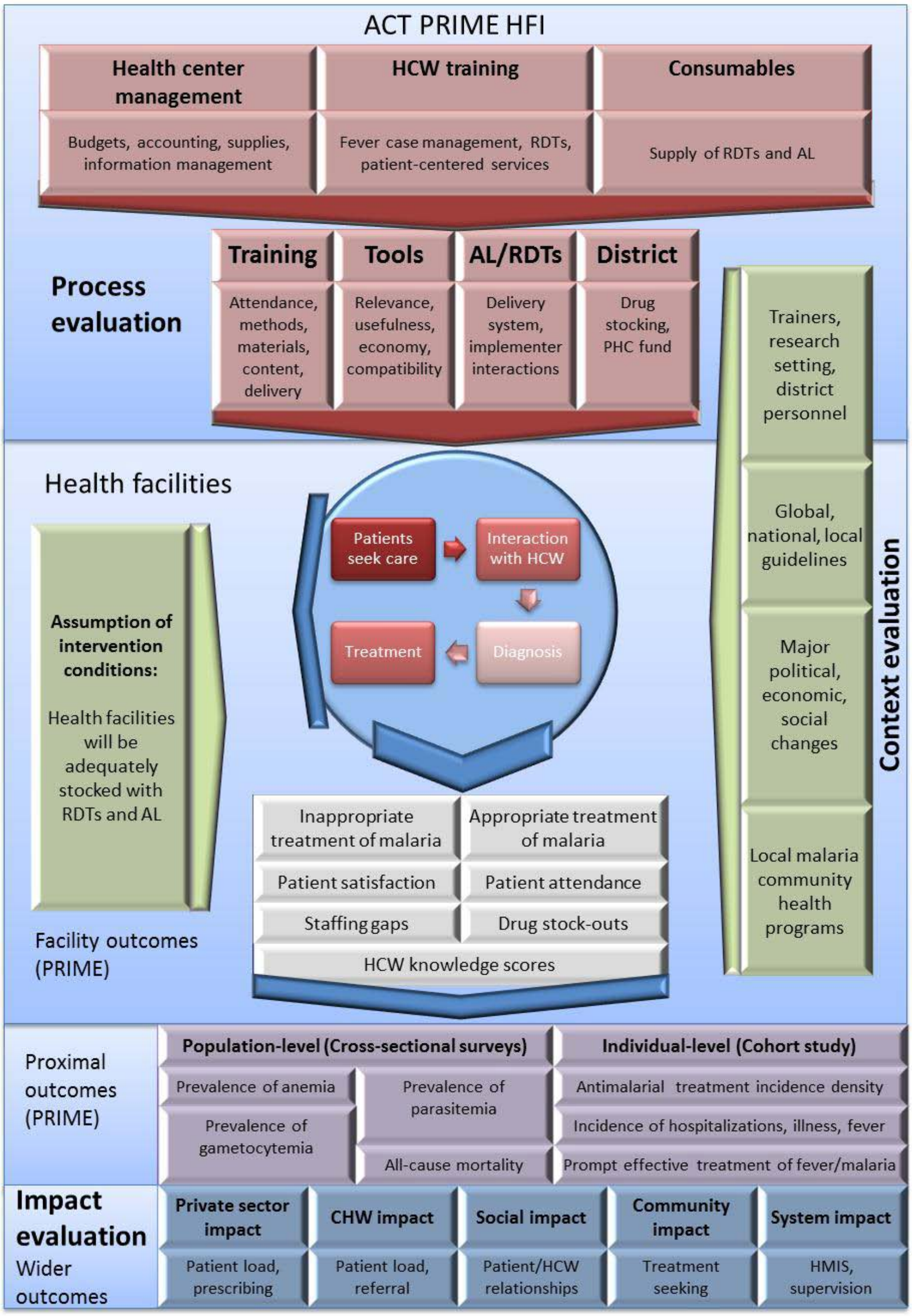

Data for the process, context and impact evaluations will be gathered through self-filled questionnaires, health worker communication assessments, IDIs, FGDs and a structured contextual record as outlined in Table 4.1 and described in Chapter 5.

Table 4.1 ACT PROCESS Evaluation methods

Evaluation method	Participants	Evaluation component			Maximum sample size
		Process evaluation	Context evaluation	Impact evaluation	
Self-filled questionnaires	- Trainees<br>- Trainers	X			350
Health worker communication assessments + patient exit interviews	- Caregivers & children under 5<br>- Health workers	X			375
In-depth interviews	- HFI Health workers	X	X	X	10
	- Implementers	X	X	X	5
	- Key stakeholders		X	X	10
Semi-structured questionnaires	- HFI Health workers	X	X	X	10
	- Standard care health workers	X	X	X	10
	- Private drug shops		X	X	10
Focus group discussions	- Primary caregivers	X	X	X	8
	- Community health workers		X	X	8
Structured contextual record	- Completed by study team		X		10

4.2. LOGIC MODEL

An initial logic model is illustrated in Figure 4.2. The logic model will be revised to ensure all components of the interventions are accurately mapped through to their intended effects and outcomes. The process of mapping the intervention started during the design phase for the ACT PRIME HFI. The design phase was a consultative process between investigators and implementers using our formative research, input from stakeholders, evidence from the literature, and behaviour change theory. Using these components, the intended mechanism of effect and the source for the hypothesised mechanism was specified. This informed the development of a logic model which describes in detail the proposed HFI components, mechanisms of effect and intended outcomes. This logic model forms the basis of subsequent evaluation components.

Figure 4.2 Logic model

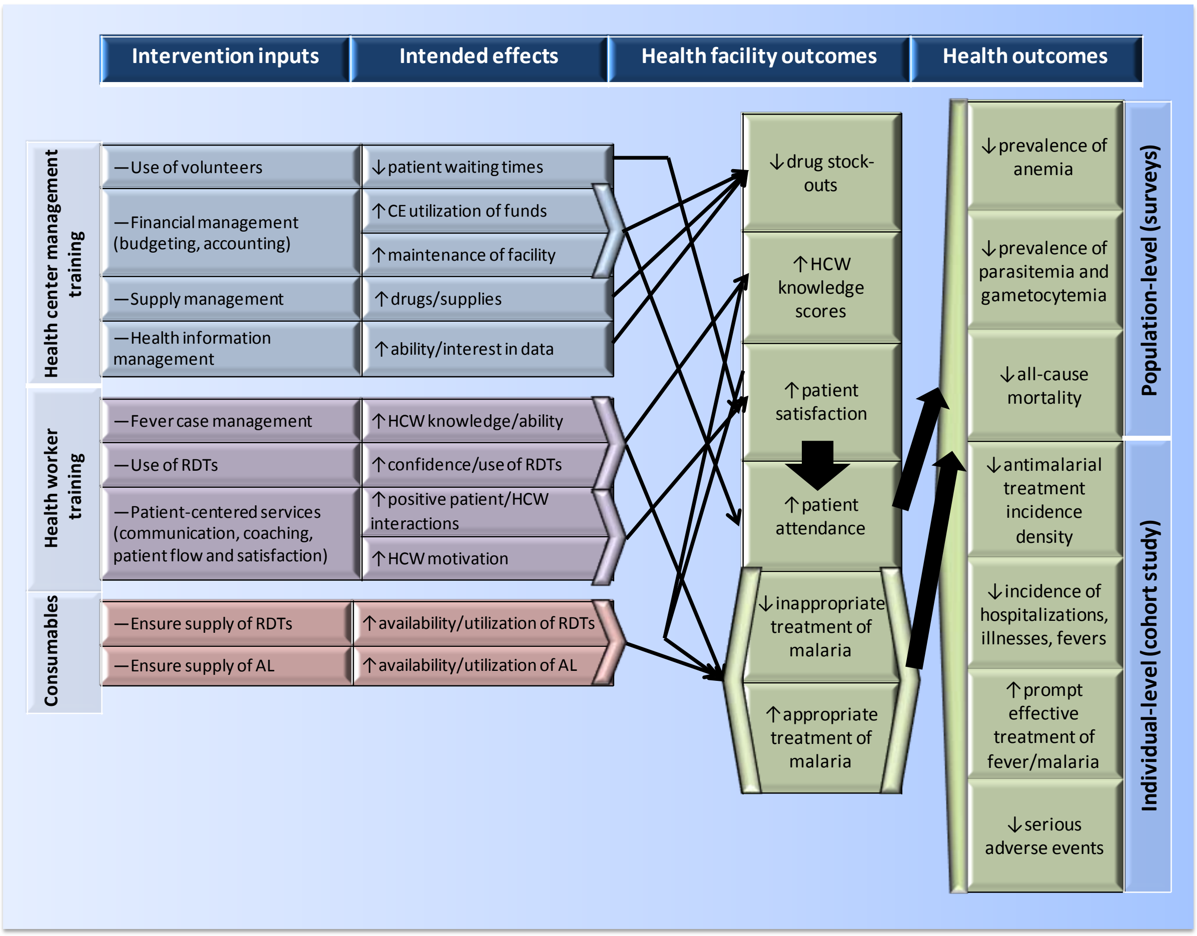

4.3. PROCESS EVALUATION

The process evaluation will involve two lines of research, (1) documentation of the implementation of the intervention as delivered by the study team and (2) qualitative study of the functioning and interpretation of the intervention by implementers and recipients. As outlined in Figure 4.1, we will be evaluating four aspects of the HFI including (1) health worker training modules, (2) health center management tools, (3) supply of AL and RDTs for malaria, and (4) interaction with the district regarding staffing and drug stocks.

4.3.1. Documentation of implementation

The logic model will be used to identify relevant activities for the process evaluation. For each activity, the following evaluation criteria will be used as categories of assessment [37].

- Fidelity (quality) — The extent to which the intervention was implemented as planned
- Dose delivered (completeness) — Amount or number of intended units of each intervention or component delivered or provided by interventionists
- Dose received (exposure) — Extent to which participants actively engage with, interact with, are receptive to and/or use materials or recommended resources. Can include ‘initial use’ and ‘continued use’

- Reach (participation rate)	Proportion of the intended priority audience that participates in the intervention; often measured by attendance; includes documentation of barriers to participation
- Recruitment	Procedures used to approach and attract participants at individual or organizational levels; includes maintenance of participant involvement in intervention
- Context	Aspects of the environment that may influence intervention implementation or study outcomes; includes contamination

Variables for data collection for each activity are formulated under each of the above headings by analysing the materials used for the HFI implementation including:

- Training packages: Health center management training; Training in fever case management and use of RDTs, Training in patient-centered services.
- Management tools: PHC Fund Accounting Tool, ACT Drug Distribution Assessment Tool
- Supply of AL and RDTs: drug stock cards, requisition and issue vouchers from health centers and the health sub-district records
- District and health sub-district interactions: Logs documenting interactions with district, health sub-district, and health center staff.

We will capture information on these variables during the implementation and monitoring and evaluation of the HFI using monthly health center records, health worker training evaluation self-filled questionnaires, IDIs with health workers and FGDs with primary caregivers. The data captured will be linked with other quantitative outcome data collected through ACT PRIME using unique identifiers of health center, community or individual health worker or intervention participant.

4.3.2. Assessment of intervention mechanisms

Perceptions of both recipients and implementers of interventions as the intervention is being rolled-out will be evaluated through the self-filled questionnaires, health worker communication assessments, IDIs and FGDs. Questions will explore the awareness, understanding of purpose, perception of relevance and usefulness, level of adoption and interpretation of importance in practice of each component of the intervention for respondents. All health worker training participants will complete the self-filled questionnaires; all health workers will be invited to participate in the health worker communication assessments; and a cross-section of participants will be invited to the FGDs (primary caregivers) and IDIs (health workers, implementers).

4.4. CONTEXT EVALUATION

Both local and regional/national contextual factors will be documented throughout ACT PRIME using a structured contextual record completed by the study team and rich contextual descriptions gathered through IDIs and FGDs.

4.4.1. Structured contextual record

The structured contextual record will involve the recording of details about factors that may affect ACT PRIME implementation and impact at three-monthly intervals by the implementing team. A structured record format will be used to document these contextual factors for each health center, and at the district level.

Factors may include the following:

- Other interventions involving malaria at the community level in the trial area
- Other research involving malaria at the community level in the trial area
- Other interventions at the health center level in the trial area
- Other research at the health center level in the trial area
- Other training programmes involving CMDs or health center staff involved in the trial
- Specific personalities or political problems at any communities/health center
- Change of staff at health center
- Change of community medicine distributor or village health team members
- Guideline changes about malaria testing and treatment at health centers/elsewhere
- Messages or news stories about malaria testing on radio/TV/newspapers
- Level of support (low, medium, high) from district health management team for the intervention
- Other local or national economic or political factors that may have impacted the delivery or receipt of this intervention

The source of each item added to the local context document will be noted on the document. The data collected in these tables will be assimilated into a report of concurrent activities and other contextual factors overall. Factors that varied widely will be used in the final analysis of the intervention impact as potential explanatory variables.

4.4.2. Rich description of context

Rich descriptions of contextual factors will be collected in all IDIs and FGDs in order to identify any contextual factors participants feel may have affected the impact of the intervention. Representatives from a randomly selected cross-section of health centers and communities will be invited to participate, and district officials will be purposively selected to represent those with most insight into the intervention process.

4.5. IMPACT EVALUATION

Clinical and economic outcomes will be collected as part of ACT PRIME. In addition, the intervention may have wider expected and unexpected impacts at the household level, community level, private sector level, and public health system level. We propose to evaluate the impact of the intervention amongst community members, health workers and others involved in providing health services using IDIs and FGDs.

4.5.1. Assessment of hypothesised impacts

Some impacts may be hypothesised in advance based on the predicted and potential mechanisms of change resulting from the intervention as described under 'intended effects' in the logic model (Figure 4.2). Both quantitative and qualitative methods will be used to assess these impacts. For quantitative measurements, existing data collection methods used in ACT PRIME will be utilized wherever possible including cross-sectional survey questionnaires, cohort household surveys, patient exit interviews, health worker knowledge questionnaires, and health facility surveillance questionnaires.

For qualitative assessments of intended effects, IDIs and FGDs will be used to assess whether and how the intervention affected specific hypothesised impacts. IDIs will be conducted with implementers and health workers in the HFI arm, and FGDs will be conducted with primary caregivers.

4.5.2. Assessment of undetermined impacts

Unexpected impacts will be assessed through a 'most significant change' (MSC) evaluation. MSC is a participatory evaluation technique that aims to collect and systematically analyse significant changes from the perspectives of those involved in a programme [41]. The technique aims to capture the values and perspectives of respondents, aiming to enrich the understanding of the intervention beyond intended changes and pre-defined indicators. A sample line of MSC questions include, "Looking back over the past three months what do you think was the most significant change in the way you managed illness in your household? Why is this significant to you? What difference has this made now or will it make in the future?" We will collect MSC stories from participants and use traditional qualitative data analysis approach to display the diversity and richness the responses. The MSC questions will be asked at the start of FGDs and IDIs that will then go on to ask directly about hypothesized impacts.

5 STUDY PROCEDURES

5.1. OVERVIEW

The ACT PROCESS study proposed here will be implemented in parallel with the ACT PRIME study, but will be carried out by a different team of field researchers. Self-filled questionnaires, health worker communication assessments and patient exit interviews, IDIs and semi-structured questionnaires, FGDs, and a contextual record will be used to evaluate the process, context and impact of the HFI in the ACT PRIME study. The self-filled questionnaires will be used to evaluate the HFI training and will be conducted during the HFI roll-out period in March-May 2011. The health worker communication assessments and patient exit interviews will be conducted immediately before and after the HFI training in patient-centered services, and then approximately six months following the training for a total of three cycles. The IDIs and semi-structured questionnaires, and FGDs will be conducted approximately 9-12 months after the HFI roll-out. The structured contextual record will be completed by the study team at three-monthly intervals. The timelines for the process, context and impact evaluations in relation to ACT PRIME are outlined in the study procedures timeline below.

Figure 5.1 Study procedures overview

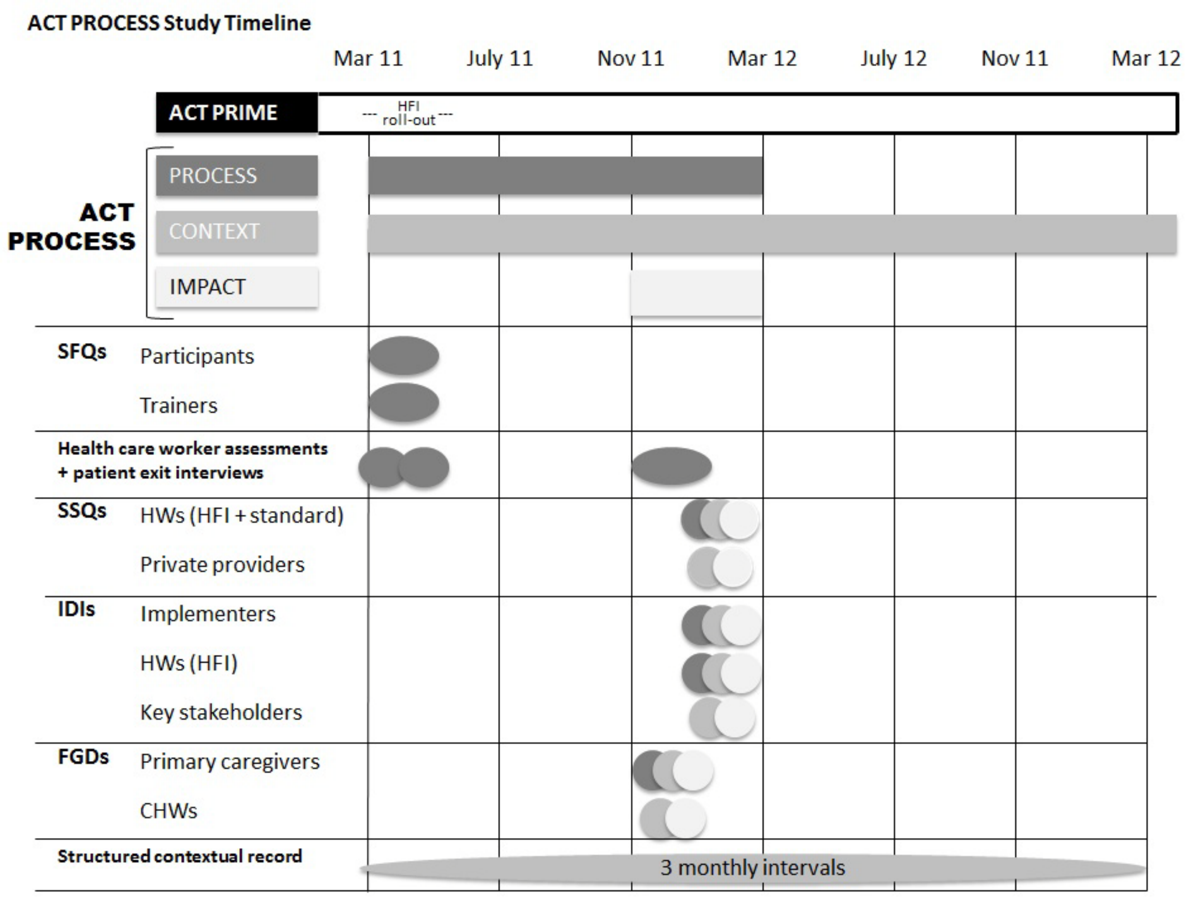

5.2. STUDY SITE

ACT PROCESS, in parallel with ACT PRIME, will be conducted in Tororo district, an area with very high malaria transmission intensity. The estimated entomologic inoculation rate (EIR) in Tororo is 562 infective bites per person-year, and the prevalence of parasitemia among children aged 5-9 years is 63.5% [42-43]. The five sub-counties of West Budama North Health Sub-district (Nagongera, Paya, Kirewa, Kisoko, and Petta), and two sub-counties of West Budama South Health Sub-district (Mulanda and Rubongi) will be included in the study population (Figure 5.2).

Figure 5.2 Study area

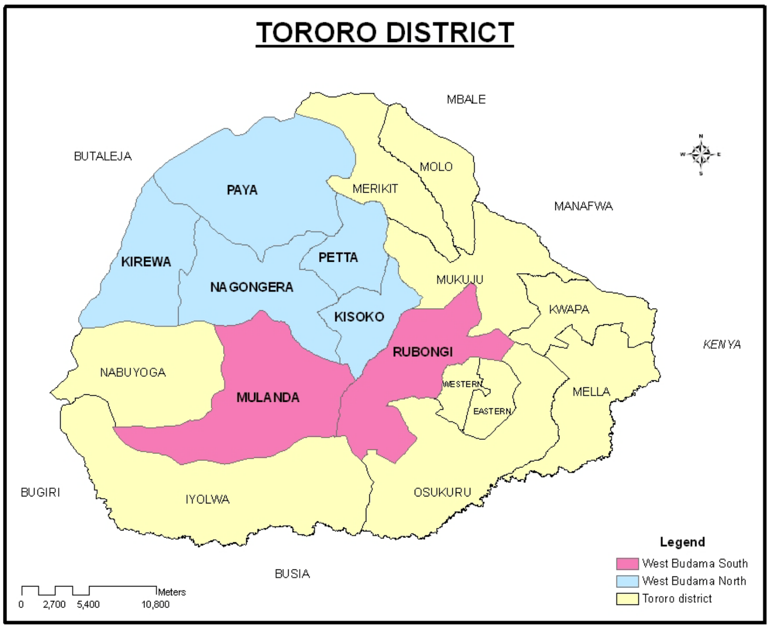

The results of our formative research suggest that this area is very rural, with limited infrastructure and education. Very few households have electricity (1%) and one-quarter have no toilet facilities. One-quarter of the heads of household have received no formal education, and only 21% have received any secondary or higher education [31].

5.3. STUDY POPULATION

Within the seven sub-counties of the study area, there are 22 lower-level government run health facilities, including 17 level II health centers, and 5 level III health centers; 20 will be included in ACT PRIME. These 20 health centers will be randomly allocated to the health facility intervention arm or the standard care arm for a total of 10 health facilities in each arm. Clusters to be included in ACT PRIME are defined as the catchment areas of the health centers including households that are located within a 2 km radius of the facilities. Only households located within the clusters will be included in the sampling frame for ACT PRIME. ACT PROCESS follows the same sampling frame as ACT PRIME; participants for each type of data collection methodology are defined below.

5.3.1. Self-filled questionnaires

Our target is to have one self-filled questionnaire completed by all HFI training participants and trainers for each module. There are 5 trainers and approximately 30 health center staff attending 10 module topics. We will conduct up to 350 self-filled questionnaires.

5.3.2. Health worker communication assessments + patient exit interviews

We aim to conduct communication assessments with at least one health worker in each health facility in both the HFI and standard care arms (10 in each arm). Each communication assessment will consist of at least three, and no more than five, health worker/caregiver interaction records. The communication assessments will be conducted immediately before and up to 6 weeks after the HFI training in patient-centered services, and then approximately six months following the training for a total of three cycles. The same health workers will be assessed at each time point to evaluate for changes over time. We will evaluate approximately 20-25 health workers chosen by convenience sampling. Therefore conducting up to 125 health worker/caregiver interactions in each time period; 375 in total, depending on the number of health workers and patients available at the health centers on the days of the study. One health worker will be chosen from each of the level II health centers, and 1-2 health workers will be chosen from each of the level III centers. Exit interviews will be conducted with all consenting caregivers who participated in the assessments, up to a maximum of 125 interviews at each time point, 375 in total.

5.3.3. In-depth interviews

We will conduct up to 25 IDIs with different target populations including HFI implementers (up to 5 interviews), health workers in the HFI arm (up to 10 interviews), and key local and district stakeholders (up to 10 interviews).

5.3.4. Semi-structured questionnaires

We will conduct up to 30 interviews to complete semi-structured questionnaires with different target populations including health workers in the HFI arm (up to 10 interviews), health workers in the standard care arm (up to 10 interviews), and private drug shop workers (up to 10 interviews). For the health worker interviews, we will target the in-charges of the health facilities.

5.3.5. Focus group discussions

We will conduct up to 16 FGDs with primary caregivers and community health workers from the study area selected by convenience sampling.

5.4. SELF-FILLED QUESTIONNAIRES

Self-filled questionnaires for each training module will be completed by all staff participants and trainers (Table 5.1). The purpose of the self-filled questionnaires is to gather opinions from participants and trainer on the objectives, content, materials, and implementation of the HFI training modules. Self-filled questionnaires will be completed at the end of each training topic. Training

modules, topics and associated self-filled questionnaires found in the Appendices are outlined in Table 5.1.

All health center staff will be invited to participate in the training module relevant to their position as outlined in Table 5.1. At the beginning of the training, written informed consent to complete the self-filled questionnaires will be obtained from all participants (Appendix B) as outlined in section 8.2. Self-filled questionnaires will be completed directly after the training topic and collected by the study team.

Table 5.1 Health worker training modules, topics and self-filled questionnaires

Module	Participants	Topics	Appendix	
			Trainers	Participants
Health center management	In-charges	Budgeting and accounting<br>Supply management<br>Information management	C	D
Fever case management	Clinical staff	Fever case management	E	F
Patient-centered services	Clinical staff	Introduction to PCS and self-observation<br>Improving interactions with patients I & II<br>Improving interactions with colleagues<br>Improving the patient visits	G	H
Patient-centered services	Support staff	Improving the patient visit	G	H

5.5. HEALTH WORKER COMMUNICATION ASSESSMENTS AND PATIENT EXIT INTERVIEWS

We plan to conduct communication assessments with health workers from both the HFI and standard care arms. The purpose of the assessments is to evaluate and compare the communication between health workers and patients immediately before and after HFI training in 'communicating with patients' and then during the study period. Health worker/caregiver interactions during consultations will be audiotaped and assessed using a validated measurement methodology, the Measurement of Patient-Centered Communication (MPCC) (Appendix I). The MPCC scores assessments according to three elements of patient-centered communication: 1) exploring the disease and the illness experience, 2) understanding the whole person, and 3) finding common ground [44]. In addition to the recorded interactions, consenting caregivers will be interviewed immediately on exit from the consultation to give their view of the quality of the interaction with the health worker. The purpose of the interviews is to determine the level of satisfaction of caregiver with the health facility visit.

Health workers from both the HFI and standard care arms available on the day of the communication assessment will be selected using convenience sampling, and invited to participate. At least one health worker from each facility, will be included. At least three interactions, and up to five interactions, will be recorded with each health worker. Written informed consent to conduct the assessments will be obtained from health workers before beginning (Appendix J) as outlined in section 8.2. Demographic information on the health worker will then be obtained (Appendix K).

Once a health worker has been selected and provided informed consent, caregivers who will have a consultation with that health worker will be selected using convenience sampling from the available patients visiting the health center on the day of the health worker communication assessment.

Patients to be included in the communication assessments will be 'typical uncomplicated malaria patients'. Inclusion criteria are 1) age: a child under five years of age, 2) fever or suspected fever, and 3) agreement of caregiver to provide informed consent. Exclusion criterion is 1) danger signs of severe disease (Appendix L). The same caregivers will be invited to participate in an exit interview after the consultation (Appendix O). Written informed consent to conduct the assessments, and the exit interviews, will be obtained from caregivers before beginning (Appendix M) as outlined in section 8.2. Demographic information on the caregiver will then be obtained (Appendix N).

The communication assessments will be conducted immediately before and up to 6 weeks after the HFI training in 'improving interactions with patients' and then approximately six months following the training for a total of three cycles. The same health workers will be evaluated in each cycle of the assessments.

5.6. IN-DEPTH INTERVIEWS

We plan to conduct IDIs with HFI implementers, health workers from the HFI arm and key stakeholders. The purpose of the IDIs is to collect information on the contextual factors and perceptions of the HFI as it is being implemented as well as the expected and unexpected impacts of the HFI. Written informed consent to conduct the IDI will be obtained from all participants before beginning (Appendix P) as outlined in section 8.2. The IDIs with health workers and implementers will be conducted approximately 9-12 months after the HFI roll-out. The IDIs with key stakeholders will be conducted approximately one year after the HFI roll-out.

Implementer participants for the IDIs will be purposively selected from the ACT PRIME implementation team. Any implementers who delivered health worker training, worked on drug distribution to health centers, or had significant interaction with health workers or district or local officials during the HFI implementation or follow-up period will be invited to participate. We plan to complete up to five IDIs with implementers, following the pre-defined topic guide (Appendix Q).

Health workers from the HFI arm will be selected by convenience sampling. We plan to complete up to 10 IDIs with health workers stationed at HFI health centers, following the pre-defined topic guide (Appendix R).

Key stakeholders will be purposively selected based on their involvement with the ACT PRIME study during the HFI implementation period or their role in the health system. For example, we plan to interview stakeholders involved with drug distribution and staffing for the HFI, district officials including the District Health Officer and Deputy District Health Officer, as well as key staff from the Tororo sub-district and sub-county level. We expect to complete up to ten IDIs with key stakeholders, following the pre-defined topic guide (Appendix S).

5.7. SEMI-STRUCTURED QUESTIONNAIRES

We plan to administer semi-structured questionnaires with health workers from HFI and standard care arms, and private drug shop workers. The purpose of the questionnaires is to collect information on the contextual factors and perceptions of the HFI as it is being implemented as well as the expected and unexpected impacts of the HFI. Written informed consent to conduct the interviews and administer the questionnaires will be obtained from all participants before beginning (Appendix T) as outlined in section 8.2. The interviews with health workers and private drug shop workers will be conducted approximately 9-12 months after the HFI roll-out.

Health workers from HFI and standard care arms will be selected by convenience sampling to ensure at least one health worker from each health facility participates in an interview. We plan to complete up to 10 interviews with health workers in both ACT PRIME arms, 20 in total. The draft questionnaire (Appendix U) will be piloted prior to the onset of the study, and will be refined if necessary.

Private drug shops workers will be randomly selected from the database developed for the Tororo District Survey Project. We plan to complete up to 10 interviews with private drug shop workers. The draft questionnaire (Appendix V) will be piloted prior to the onset of the study, and will be refined if necessary.

5.8. FOCUS GROUP DISCUSSIONS

We plan to conduct FGDs with primary caregivers and community health workers from both the HFI and standard care arms. The purpose of the FGDs is to collect information on the contextual factors and perceptions of the intervention as it is being implemented as well as the expected and unexpected impacts of the HFI on communities in the study area. Written informed consent to conduct the FGDs will be obtained from all participants before beginning (Appendix W), as outlined in section 8.2. Participants for FGDs will be selected from the HFI arm and the standard care arm; the same groups of participants will be invited to attend from each arm. The definition of each target population is provided in Table 5.2.

Table 5.2 FGD target populations

Target group	Definitions	FGD characteristics
Primary caregivers	Person primarily responsible for daily care of young children (generally female)	Groups to be stratified by age (< 30 years vs. > 30 years), health center (communities from each health center in ACT PRIME will be represented)
Community health worker	Person providing health care through the Village Health Team	Health center (communities from each health center in ACT PRIME study will be represented), distance to a health center (within or outside of a 2km radius of an ACT PRIME health center)

5.8.1. Primary caregiver FGDs

Eight FGDs will be conducted with primary caregivers approximately 9-12 months after the HFI roll-out; four FGDs will be held with primary caregivers over 30 years, and four with caregivers under 30 years. As outlined in Table 5.3, we have designed a matrix which distributes the FGDs across the desired categories.

Table 5.3 Sampling matrices for primary caregiver FGDs

	HFI health centers		Standard care health centers	
	<30 yrs	>30 yrs	<30 yrs	>30 yrs
HC # 1-5	FGD 1	FGD 2	FGD 5	FGD 6
HC # 6-10	FGD 3	FGD 4	FGD 7	FGD 8

The ten health centers in each of the ACT PRIME study arms will be numbered and inserted into the matrix above. One village in close proximity to one of the health facilities on each of the lists (health centers 1-5, and 6-10) will be selected using convenience sampling to include in each of the primary caregiver FGDs. Different health centers and different villages will be selected for each FGD. Local leaders will be asked to help identify and invite 6-12 representatives from the specified target group to participate in the FGDs.

A moderator and an assistant will lead the discussions in the local language (Japadhola or Swahili) using the FGD guides (Appendix X). During the FGDs, participants will be encouraged to share all thoughts and opinions. All FGDs will be recorded using a digital voice recorder, provided informed consent is given by participants. Hand-written notes detailing respondent identification numbers and verbal and non-verbal participant responses will be made in all FGDs.

5.8.2. Community health worker FGDs

We plan to conduct eight FGDs with CHWs approximately 9-12 months after the HFI roll-out. As outlined in Table 5.4, we have designed a matrix which distributes the 8 FGDs across the desired categories.

Table 5.4 Sampling matrices for CHW FGDs

	HFI health centers		Standard care health centers	
	< 2km	≥ 2km	< 2km	≥ 2km
HC # 1-5	FGD 1	FGD 2	FGD 5	FGD 6
HC # 6-10	FGD 3	FGD 4	FGD 7	FGD 8

The ten health centers in each of the ACT PRIME study arms will be numbered and inserted into the matrix above. One village located < 2km, and one ≥ 2km, from each of the health facilities, will be selected using convenience sampling from a list generated by previous GPS mapping. Local leaders will be asked to help identify and invite all CHWs from their village (typically two per village) to participate in the FGDs. A total of 5 villages will be represented in each FGD, representing the catchment areas of 5 different health centers.

A moderator and an assistant will lead the discussions in the local language (Japadhola or Swahili) using the FGD guides (Appendix Y). During the FGDs, participants will be encouraged to share all thoughts and opinions. All FGDs will be recorded using a digital voice recorder, provided informed consent is given by participants. Hand-written notes detailing respondent identification numbers and verbal and non-verbal participant responses will be made in all FGDs.

5.9. STRUCTURED CONTEXTUAL RECORD

A structured contextual record will be used to collect details about factors that may affect implementation and impact of the HFI. Sources of information for contextual details include published and grey literature; radio, local television and newspaper reports; notices from the Uganda MoH, National Malaria Control Programme, and other national departments; internet sites of organizations and NGOs active in the area including WHO, other UN organizations, Malaria Consortium, AMREF; and other relevant sources of information. These sources of information will

be reviewed on a three-monthly basis by the implementation team and details will be entered into the structured contextual record (Appendix Z).

In addition, detailed data will be collected prospectively on coverage levels of key malaria control interventions across Tororo district as detailed in Table 5.5. These data will be collected through the UMSP sentinel site at Nagongera Health Center IV in Tororo district. Data on IRS coverage will come from the Uganda MoH and implementing partners. Data on ITN coverage and ACT use will come from the cross sectional surveys and outpatient surveillance system operated by the UMSP.

Table 5.5 Malaria control intervention variables of interest

Category	Metric	Source of data
**IRS**	- Date, formulation, and proportion of households sprayed	MoH records
**ITNs**	- Proportion of households with at least one bednet<br>- Proportion of households with at least one ITN<br>- Average number of nets per household<br>- Average number of ITNs per household<br>- Proportion of children under five who slept under any net the prior night<br>- Proportion of children under five who slept under an ITN the prior night	Cross-sectional surveys
**ACTs**	- Proportion of febrile episodes in children treated with an ACT	Cross-sectional surveys
	- Proportion of antimalarial doses prescribed that were ACTs	Outpatient surveillance
	- Number of ACT doses prescribed at health care facility per month	Outpatient surveillance

5.10. SUPPLY OF DRUGS AND RDTS

We plan to collect data on the supply of artemether-lumefantrine and RDTs provided by the ACT PRIME Study to each of the HFI health centers using drug stock cards, and requisition and issue vouchers from the health centers and health sub-districts. Study personnel will collect the information during a one-day visit to the health facilities. The in-charge of the facility will be approached and informed about the surveillance activities. An information sheet (Appendix AA) will be used to describe the purpose of the activities and verbal consent to collect information will be obtained from the in-charge. Information will be collected on (1) supply and use of artemether-lumefantrine supplied by National Medical Stores (NMS) and ACT PRIME; and (2) supply and use of RDTs for malaria supplied by ACT PRIME (Appendix BB).

6 DATA MANAGEMENT

6.1. DATA MANAGEMENT

6.1.1. Quantitative data

Quantitative data from the self-filled questionnaires, patient exit interviews, semi-structured questionnaires, structured contextual record, and supply records will be collected by the study team. Data from the paper questionnaires and data collection forms will be entered into an Access database by a data entry clerk and will be double entered to verify accuracy. Back-up files of databases will be stored after each data entry session. For quality control, query programs will be written into the database to limit the entry of incorrect data and ensure entry of data into required fields.

6.1.2. Qualitative data

6.1.2.1. Self-filled questionnaires

Qualitative data from the self-filled questionnaires will be typed into Word documents for export to NVivo qualitative data management software. All typed records will be checked for accuracy against the original questionnaires. Back-up files of word documents will be stored after each data entry session.

6.1.2.2. Health worker communication assessment

All health worker communication assessments will be audiotaped using a digital voice recorder. Assessments will be transcribed and translated into English if necessary. Summaries of the assessments will be coded using an appropriate software package. All coded records will be checked for accuracy against the original recordings and field logs. The consultation recordings will be backed-up after each coding session.

6.1.2.3. Patient exit interviews

Any qualitative data arising from the patient exit interviews will be typed into Word documents for export to NVivo qualitative data management software. All typed records will be checked for accuracy against the original questionnaires. Back-up files of word documents will be stored after each data entry session.

6.1.2.4. IDIs

The IDIs will be administered using the appropriate topic guide by the interviewer. An assistant will take notes of the discussion. All interviews will also be recorded using a digital voice recorder. Summaries of the interviews will be written in the language of the interview, and will then be translated into English if necessary. Summaries of the interviews will typed into Word documents for export to NVivo qualitative data management software. All typed records will be checked for

accuracy against the original recordings and field notes of the assistant. Back-up files of word documents will be stored after each data entry session.

6.1.2.5. Semi-structured questionnaires

Any qualitative data arising from the semi-structured questionnaires will be typed into Word documents for export to NVivo qualitative data management software. All typed records will be checked for accuracy against the original questionnaires. Back-up files of word documents will be stored after each data entry session.

6.1.2.6. FGDs

The FGDs will be facilitated by a moderator and the assistant will take notes of the discussion and non-verbal communication in English. All FGDs will also be recorded using a digital voice recorder. Recordings of the FGDs will be transcribed into the local language, and then translated into English. The transcripts will be checked for accuracy against the original recordings and field notes by members of the field team. The transcripts and discussion notes will be reviewed for themes and re-organized according to discussion topics. Back-up files of transcripts will be stored after each data entry session.

6.1.2.7. Structured contextual record

Qualitative data from the contextual record will be typed into Word documents for export to NVivo qualitative data management software. All typed records will be checked for accuracy against the original questionnaires. Back-up files of word documents will be stored after each data entry session.

6.2. QUALITY ASSURANCE AND QUALITY CONTROL

All members of the study team will be trained in the project objectives, methods of effective communication with study participants, and collection of high quality data. Study team members will receive additional training specific to the tasks they will perform within the project including interviewing techniques and completing questionnaires. Standard Operating Procedures (SOPs) will be written for all project activities and booklets of all relevant documents will be provided to each member of the project team. Study group meetings will be conducted by the principal investigator to assess progress of the study, address any difficulties, and provide performance feedback to the members of the study group. Any corrections to data collection forms will be made by striking through the incorrect entry with a single line and entering the correct information adjacent to it, according to Good Clinical Practice guidelines [45]. The correction will be initialled and dated by the investigator. The investigators will allow all requested monitoring visits, audits or reviews.

6.3. RECORDS AND STORAGE

All study documents will be kept in secured filing cabinets in the Infectious Disease Research Collaboration offices. The principal investigator will be responsible for the security of all project documents. Back-up files of databases will be stored onto the main project server after each data entry session. Participants will be identified by their study ID number, and participant names will not be entered into the computerised database.

6.4. DATA SHARING

This project is one of 16 participating in the ACT Consortium (www.actconsortium.org/). As part of the ACT Consortium, a policy liaison network will be organized to help synthesize data from the multiple projects and communicate the results to policy makers. Consortium researchers will share data with the policy liaison network to facilitate analyses and ensure broad dissemination of the research findings.

7 ANALYTICAL PLAN

7.1. QUANTITATIVE DATA

Categorical variables from the self-filled questionnaires, patient exit interviews, semi-structured questionnaires, structured contextual record, and supply records, will be compared using the chi-square test or Fisher's exact test. Pairwise comparisons of continuous variables will be made using a two-sample t-test or non-parametric test when appropriate. A p-value < 0.05 (two-tailed) will be considered statistically significant. Analysis will be done using STATA (Stata, College Station, TX, USA).

7.2. AUDIO RECORDINGS

The health worker communication assessment audio recordings will be analyzed at the individual assessment level using the MPCC which has been validated and shows interrater reliabilities (interclass correlations) of 0.80-0.83. The coding is based on three components of patient-centered communication and produces a score for each component. These scores will be used to measure health worker responsiveness to patient concerns and produce a mean score of patient-centered communication ranging from 0 (not patient-centered) to 1 (very patient-centered) [44]. The audio recordings will be coded by trained social scientists, each coding one half of the assessments. All of the assessments will be dual-coded and compared for accuracy. Descriptive statistical analysis on MPCC scores will done using STATA (Stata, College Station, TX, USA).

7.3. QUALITATIVE DATA

Transcripts and interview notes from the self-filled questionnaires, patient exit interviews, IDIs, semi-structured questionnaires, FGDs, and structured contextual record will be analysed using a coding scheme developed from pre-defined topics together with themes emerging from the data. Coding will be done by hand, and using qualitative data analysis software, NVivo (QSR International, Cambridge, MA). We plan to prospectively label and code themes within topics as they emerge, resulting in a data-generated coding scheme. This stage of the analysis will be conducted independently by different members of the study team on different transcripts and then a final coding scheme will be agreed on and applied to all transcripts, with at least two members of the study team reviewing each transcript. The Nvivo software program will be used to aggregate the data by codes, and to assist with report writing.

8 PROTECTION OF HUMAN PARTICIPANTS

8.1. INSTITUTIONAL REVIEW BOARDS

This protocol and the information sheets will be reviewed and approved by all IRBs before the project begins. Any amendments or modifications to this material will also be reviewed and approved by the IRBs prior to implementation. The IRBs will include:

London School of Hygiene & Tropical Medicine (LSHTM) Ethics Committee
Address: Keppel Street, London, WC1E 7HT, UK
Contact Person: Paula Eliott
Phone Number: +44 (0) 20 7927 2256
Email: Ethics@lshtm.ac.uk

Faculty of Medicine Research and Ethics Committee (FOMREC), Makerere University
Address: Makerere University, Faculty of Medicine, Office of the Dean, PO Box 7072, Kampala, Uganda
Contact Person: Dr. Charles Ibingira
Phone Number: +256 (0) 414-530020
Fax Number: +256 (0) 414-531091

Uganda National Council of Science and Technology (UNCST)
Address: Plot 3/5/7 Nasser Road, PO Box 6884, Kampala, Uganda
Contact Person: Dr. Peter Ndmerere
Phone Number: +256 (0) 414-250499
Fax Number: +256 (0) 414-234579

8.2. INFORMED CONSENT PROCESS

Approval from local leaders will be sought before beginning activities in the project area. Written informed consent will be obtained from all participants for the self-filled questionnaires, health worker communication assessments and patient exit interviews, IDIs, semi-structured questionnaires, and FGDs. Study personnel will conduct informed consent discussions with potential study participants or their parent/guardian. Informed consent will be conducted in the appropriate language and a translator will be used if necessary. Consent forms will be available in English, Japadhola, and Swahili. During the consent discussion, the appropriate consent form will be read to the potential study participant (or parent/guardian) describing the purpose of the project, the procedures to be followed, and the risks and benefits of participation, and any questions raised will be answered. Following the informed consent discussion, the potential study participant (or parent/guardian) will be asked to provide their written consent on the approved informed consent document to participate in a research study. If the potential study participant (or parent/guardian) is unable to read or write, their fingerprint will substitute for a signature, and a signature from a witness to the informed consent procedures will be obtained.

Verbal consent will also be obtained prior to collecting information on the supply of drugs and RDTs from the health center in-charges using an information sheet. Information sheets in local languages

will be provided describing the purpose of the project and the procedures to be followed, and the risks and benefits of participation.

8.2.1. Self-filled questionnaires

Study personnel will seek informed consent from PRIME HFI training participants and from HFI trainers to complete the self-filled questionnaires. The informed consent discussion will be conducted with participants and trainers at the location of the training (health facility or other convenient location) prior to beginning the training. If the health worker cannot read, an impartial witness will be present during the entire consent process.

8.2.2. Health worker communication assessments and patient exit interviews

Study personnel will seek informed consent from health workers for participation in the health worker communication assessments and from caregivers for participation in both the health worker communication assessments and patient exit interviews. The informed consent discussion will be conducted with health workers at the health facility prior to beginning the assessment. If the health worker cannot read, an impartial witness will be present during the entire consent process. After a health worker has consented to participate, written informed consent will be sought from caregivers prior to each interaction. If a health worker or caregiver cannot read, an impartial witness will be present during the entire consent process.

8.2.3. In-depth Interviews

Study personnel, including a translator if necessary, will seek informed consent from potential study participants for participation in the IDIs. The informed consent discussion will be conducted with potential participants at a convenient location (health center, office, residence) and in a language that the participant is most comfortable with, using a translator if necessary. If the potential participant cannot read, an impartial witness will be present during the entire consent process.

8.2.4. Semi-structured questionnaires

Study personnel, including a translator if necessary, will seek informed consent from potential study participants for participation in the semi-structured questionnaires. The informed consent discussion will be conducted with potential participants at a convenient location (health center, office, residence) and in a language that the participant is most comfortable with, using a translator if necessary. If the potential participant cannot read, an impartial witness will be present during the entire consent process.

8.2.5. Focus group Discussions

Study personnel, including a translator if necessary, will seek informed consent from primary caregivers and CHWs for participation in the FGDs. The informed consent discussion will be conducted with primary caregivers at their residence and with CHWs at a convenient location in the language that the primary caregiver/CHW is most comfortable with, using a translator if necessary. If the primary caregiver/CHW cannot read, an impartial witness will be present during the entire consent process.

8.2.6. Supply of drugs and RDTs

Study personnel will collect the information about the supply and stocks of artemether-lumefantrine and RDTs during a one-day visit to the health facilities. The in-charge of the facility will be approached prior to the first visit and informed about the surveillance activities. An information sheet will be used to describe the purpose of the activities and verbal consent to collect information will be obtained from the in-charge. If the in-charge gives their verbal consent to participate in the study, their consent to participate in the study will be documented on the data collection log.

8.3. CONFIDENTIALITY

Participants in all study activities will be informed that participation in a research study may involve a loss of privacy. All records will be kept as confidential as possible. Participants will be identified by study numbers and participant names will not be entered into the computerized database. FGD participants will be referred to by their first names during the discussion, but names will not be recorded in the notes or transcripts; participants will be referred to by a participant ID only. In addition, participants providing qualitative data, including those involved in the self-filled questionnaires, patient exit interviews, IDIs, semi-structured questionnaires, and FGDs will be given the option of not being quoted at all, anonymously or otherwise, or included in any of the analyses. Completed questionnaires will be kept in secured filing cabinets in the study offices in Tororo and Kampala. Additional records will be stored in the log books, which will be stored securely in the study offices in Tororo. No individual identities will be recorded in the database or used in any reports or publications resulting from the study.

8.4. RISKS AND DISCOMFORTS

8.4.1. Privacy

Care will be taken to protect the privacy of participants, as described in this protocol. However, there is a risk that others may inadvertently see participants' information, and thus their privacy compromised. All information gathered will be treated as private by the study personnel, and records will be kept securely in locked filing cabinets and offices. No personal identification information such as names will be used in any reports arising out of this research.

8.4.2. Compensation

Self-filled questionnaires, health worker communication assessments and patient exit interviews, IDIs, semi-structures questionnaires, and FGDs will be held in venues central to participants' residences or place of work. There will be no cost to participants and participants will not be paid; however, 5000/= will be given to each FGD participant as compensation to refund their transport costs.

8.4.3. Alternatives

All identified participants may choose not to participate in any of the study activities. A decision not to participate will not have any impact on employment or eligibility for medical care or participation in future studies.

9 REFERENCES

1. Greenwood, B.M., et al., *Malaria.* Lancet, 2005. **365**(9469): p. 1487-98.

2. Breman, J., *The ears of the hippopotamus: manifestations, determinants, and estimates of the malaria burden.* Am J Trop Med Hyg, 2001. **64**: p. 1-11.

3. Breman, J.G., M.S. Alilio, and A. Mills, *Conquering the intolerable burden of malaria: what's new, what's needed: a summary.* Am J Trop Med Hyg, 2004. **71**(2 Suppl): p. 1-15.

4. Snow, R.W., et al., *Estimating mortality, morbidity and disability due to malaria among Africa's non-pregnant population [see comments].* Bull World Health Organ, 1999. **77**(8): p. 624-40.

5. Uganda Ministry of Health, *The Burden of Malaria in Uganda*. 2008.

6. Uganda Ministry of Health, *Malaria control and prevention*. 2008.

7. Kager, P.A., *Malaria control: constraints and opportunities.* Trop Med Int Health, 2002. **7**(12): p. 1042-6.

8. Moerman, F., et al., *The contribution of health-care services to a sound and sustainable malaria-control policy.* Lancet Infect Dis, 2003. **3**(2): p. 99-102.

9. McCombie, S.C., *Treatment seeking for malaria: a review of recent research.* Soc Sci Med, 1996. **43**(6): p. 933-45.

10. Marsh, V.M., et al., *Changing home treatment of childhood fevers by training shop keepers in rural Kenya.* Trop Med Int Health, 1999. **4**(5): p. 383-9.

11. Goodman, C., et al., *Retail supply of malaria-related drugs in rural Tanzania: risks and opportunities.* Trop Med Int Health, 2004. **9**(6): p. 655-63.

12. Kelly, J.M., et al., *Community health worker performance in the management of multiple childhood illnesses: Siaya District, Kenya, 1997-2001.* Am J Public Health, 2001. **91**(10): p. 1617-24.

13. Zurovac, D. and A.K. Rowe, *Quality of treatment for febrile illness among children at outpatient facilities in sub-Saharan Africa.* Ann Trop Med Parasitol, 2006. **100**(4): p. 283-96.

14. Leonard, K., M. Masatu, and A. Vialou, *Getting clinicians to do their best: Ability, Altruism and Incentives.* 2005, Working paper, University of Maryland.

15. Chakraborty, S. and K. Frick, *Factors influencing private health providers' technical quality of care for acute respiratory infections among under-five children in rural West Bengal, India.* Soc Sci Med, 2002. **55**(9): p. 1579-87.

16. Arifeen, S.E., et al., *Quality of care for under-fives in first-level health facilities in one district of Bangladesh.* Bull World Health Organ, 2005. **83**(4): p. 260-7.

17. Krause, G., et al., *Diagnostic quality in rural health centers in Burkina Faso.* Trop Med Int Health, 1998. **3**(2): p. 100-7.

18. World Health Organisation, *World Health Report 2000. Health Systems: Improving Performance*. 2000: Geneva.

19. Brown, L.D., et al., *Quality Assurance of Health Care in Developing Countries*. 1993, Quality Assurance Project. Online at http://www.qaproject.org/pubs/PDFs/DEVCONT.pdf. Accessed 24 July 2008: Bethesda, MD.

20. Hall, J.A., et al., *Physicians' liking for their patients: more evidence for the role of affect in medical care.* Health Psychol, 1993. **12**(2): p. 140-6.

21. Mbaruku, G. and S. Bergstrom, *Reducing maternal mortality in Kigoma, Tanzania.* Health Policy Plan, 1995. **10**(1): p. 71-8.

22. McPake, B., *User charges for health services in developing countries: a review of the economic literature.* Soc Sci Med, 1993. **36**(11): p. 1397-405.

23. Wouters, A.V., *Essential national health research in developing countries: health care financing and the quality of care.* Int J Health Planning and Management, 1991. **6**: p. 253-271.

24. El Arifeen, S., et al., *Integrated Management of Childhood Illness (IMCI) in Bangladesh: early findings from a cluster-randomised study.* Lancet, 2004. **364**(9445): p. 1595-602.

25. Deyo, R.A. and T.S. Inui, *Dropouts and broken appointments. A literature review and agenda for future research.* Med Care, 1980. **18**(11): p. 1146-57.

26. Vera, H., *The client's view of high-quality care in Santiago, Chile.* Stud Fam Plann, 1993. **24**(1): p. 40-9.

27. Williams, B., *Patient satisfaction: a valid concept?* Soc Sci Med, 1994. **38**(4): p. 509-16.

28. Grimshaw, J., et al., *Changing provider behaviour: an overview of systematic reviews of interventions to promote implementation of research findings by healthcare professionals*, in *Getting Research Findings into Practice*, A. Haines and A. Donald, Editors. 2002, BMJ Books: London. p. 29-68.

29. Oxman, A.D., et al., *No magic bullets: a systematic review of 102 trials of interventions to improve professional practice.* Cmaj, 1995. **153**(10): p. 1423-31.

30. Smith, L.A., et al., *Review: Provider practice and user behavior interventions to improve prompt and effective treatment of malaria: do we know what works?* Am J Trop Med Hyg, 2009. **80**(3): p. 326-35.

31. Staedke, S.G. and ACT Study Team, *Tororo District Survey Project: Characterizing the population and local health services*. 2010, Uganda Malaria Surveillance Project, Infectious Disease Research Collaboration.: Kampala.

32. Ross-Degnan, D., et al., *Improving pharmaceutical use in primary care in developing counties: a critical review of experience and lack of experience.*, in *Presented at the International Conference on Improving Use of Medicines*. 1997: Chiang Mai, Thailand, April

33. Power, R., et al., *Developing complex interventions for rigorous evaluation--a case study from rural Zimbabwe.* Health Educ Res, 2004. **19**(5): p. 570-5.

34. MRC, *Developing and Evaluating Complex Interventions: new guidance*. 2008, Medical Research Council. Available online at http://www.mrc.ac.uk/consumption/idcplg?IdcService=GET_FILE&dID=15585&dDocName=MRC004871&allowInterrupt=1: London.

35. Lewin, S., C. Glenton, and A.D. Oxman, *Use of qualitative methods alongside randomised controlled trials of complex healthcare interventions: methodological study.* BMJ, 2009. **339**: p. b3496.

36. Harris, M.J., *Evaluating Public and Community Health Programmes*. 2010, San Fransisco: Jossey-Bass.

37. Saunders, R.P., M.E. Evans, and P. Joshi, *Developing a Process-Evaluation Plan for Assessing Health Promotion Program Implementation: A How-To Guide.* Health Promotion Practice, 2005. **6**: p. 134-147.

38. Chandler, C.I.R., *ACT Consortium Social Science Guidance*. 2009.

39. Chandler, C.I.R., R. Hall-Clifford, and S. Yeung, *ACT Consortium Framework for Evaluation of RDT Interventions*. 2009.

40. Jones, N., et al., *Improving Impact Evaluation Production and Use*. 2009, Overseas Development Institute.

41. Davies, N. and J. Dart, *The 'Most Significant Change' (MSC) Technique. A guide to its use. Available online at http://www.mande.co.uk/docs/MSCGuide.pdf*. 2005.

42. Okello, P.E., et al., *Variation in malaria transmission intensity in seven sites throughout Uganda.* Am J Trop Med Hyg, 2006. **75**(2): p. 219-25.

43. Pullan, R.L., et al., *Plasmodium infection and its risk factors in eastern Uganda.* Malar J, 2010. **9**: p. 2.

44. Epstein, R.M., et al., *Measuring patient-centered communication in Patient-Physician consultations: Theoretical and practical issues.* Social Science & Medicine, 2005. **61**(7): p. 1516-1528.

45. International Conference on Harmonisation of Technical Requirements for Registration of Pharmaceuticals for Human Use (ICH), *Guidance for Industry: Good Clinical Practice: Consolidated Guidance (ICH-E6)*. 1996, Rockville, MD: U.S. Department of Health and Human Services, Food and Drug Administration, Center for Drug Evaluation and Research (CDER), Center for Biologics Evaluation and Research (CBER).

10 APPENDICES

Appendix A: Informed consent for SFQs – Trainers
Appendix B: Informed consent for SFQs – Participants
Appendix C: SFQ Health center management training – Trainers
Appendix D: SFQ Health center management training – Participants
Appendix E: SFQ Fever case management training – Trainers
Appendix F: SFQ Fever case management training – Participants
Appendix G: SFQ Patient-centered services training – Trainers
Appendix H: SFQ Patient-centered services training – Participants
Appendix I: Measuring Patient-Centered Communication coding scheme
Appendix J: Informed consent for HWCA – Health workers
Appendix K: HWCA demographic form – Health workers
Appendix L: HWCA Screening Form – Caregivers
Appendix M: Informed consent for HWCA – Caregivers
Appendix N: HWCA demographic form – Care givers
Appendix O: HWCA Patient Exit Interview
Appendix P: Informed consent for IDIs
Appendix Q: IDI Data collection tool – Implementers
Appendix R: IDI Data collection tool – Health workers (HFI)
Appendix S: IDI Data collection tool – Key stakeholders
Appendix T: Informed consent for SSQ
Appendix U: SSQ – Health workers, HFI and standard care
Appendix V: SSQ – Private drug shops
Appendix W: Informed consent for FGDs
Appendix X: FGD Data collection tool – Primary caregivers
Appendix Y: FGD Data collection tool – Community health workers
Appendix Z: Contextual record form
Appendix AA: Information sheet for surveillance of AL & RDTs
Appendix BB: Data collection form for surveillance of AL & RDTs

THE REPUBLIC OF UGANDA

[____]—[____|____|____|____]

SFQ Study ID

APPENDIX A. SELF-FILLED QUESTIONNAIRES
Informed consent form for participants

**Protocol Title:**	ACT PROCESS: Evaluating the process, context, and impact of interventions to enhance health facilities in Tororo, Uganda
**Site of Research:**	Tororo, Uganda
**Principal Investigators**:	Dr. Sarah Staedke
**Date:**	23 February 2011

Introduction

Dr. Sarah Staedke and colleagues from the Uganda Malaria Surveillance Project/Infectious Diseases Research Collaboration are conducting a research study to see if we can improve the health of children in this area by improving services at government-run health facilities. Part of this study intervention includes training for health workers on the following topics: (1) health centre management, (2) fever case management and use of rapid diagnostic tests for malaria, (3) patient-centred services. You have been invited to take part in these training sessions because of your role in the health facility. In addition to taking part in the training, we are asking for your feedback on the training session you attended.

Why is this study being done?

We would like to know more about how our training sessions were delivered in your area. To do this, we are asking all health workers who take part in the training for their opinions on the session they attended. This information will help us understand how the training session was delivered by our trainer and what you thought about the training methods, content, and objectives.

What will happen today if I take part in this study?

Today, we are inviting you to complete a questionnaire on your own regarding the training session. We can answer any questions you may have about the questionnaire or how to complete it. You will be asked to complete a questionnaire at the end of each training session. We will collect your questionnaires for analysis. After you complete the questionnaire, we will not ask you to do anything further. The information we collect will be used by project investigators, and may be shared with other researchers and policy-makers to answer questions about how best to deliver training and health services.

How long will the study last?

The training sessions will be conducted over 8-10 weeks. The number of training sessions that you will take part in will depend on the role you play at the health center. At the end of each session, you will be asked to complete a questionnaire. Each questionnaire will take about 30 minutes to complete.

Can I stop being in the study?

You can decide to stop participating at any time. Just tell the project researcher right away.

What risks can I expect from being in the study?

Participation in any research study may involve a loss of privacy. Your name will not be used in any reports of the information provided. No quotes or other results arising from your participation in this study will be included in any reports, even anonymously, without your agreement. The information obtained from these questionnaires will be locked at our project offices. We will do our best to make sure that the personal information gathered is kept private.

Are there benefits to taking part in the study?

By participating in the training session, you will have the chance to learn new information and skills, or refresh information and skills you have already learned. There will be no other direct benefit to you from participating in this study. However, the feedback that you provide about the training sessions will help researchers and policy-makers understand how best to improve health services in this area, especially in the treatment of malaria.

What other choices do I have if I do not take part in this study?

You are free to choose not to take part in the study. If you decide not to take part in this study, there will be no penalty to you.

What are the costs of taking part in this study? Will I be paid for taking part in this study?

There are no costs to you for taking part in this study. You will not be paid for taking part in this study.

What are my rights if I take part in this study?

Taking part in this study is your choice. You may choose either to take part or not to take part in the study. If you decide to take part in this study, you may change your mind at any time. No matter what decision you take, there will be no penalty to you in any way.

Who can answer my questions about the study?

You can talk to the researchers about any questions or concerns you have about these study activities. Contact Dr. Sarah Staedke or other members of the Uganda Malaria Surveillance Project / Infectious Disease Research Collaboration on telephone number 0414-530692. If you have any questions, comments or

concerns about taking part in these activities, first talk to the researchers. If for any reason you do not wish to do this, or you still have concerns about doing so, you may contact Dr Charles Ibingira, Makerere University Faculty of Medicine Research and Ethical Committee at telephone number 0414-530020.

WHAT YOUR SIGNATURE OR THUMBPRINT MEANS

Your signature or thumbprint below means that you understand the information given to you in this consent form about your participation in the study and agree with the following statements:

1. "I have read the consent form concerning this study (or have understood the verbal explanation of the consent form) and I understand what will be required of me and what will happen to me if I take part in it."

2. "My questions concerning this study have been answered by Dr. Staedke or the person who signed below."

3. "I understand that at any time, I may withdraw from this study without giving a reason."

4. "I agree to take part in this study."

If you wish to participate in this study, you should sign or place your thumbprint on the following page. If you do not agree to quotes or other results arising from your participation in the study being included, even anonymously, in any reports about the study, please tell the researcher now.

THE REPUBLIC OF UGANDA

WE WILL GIVE YOU A COPY OF THIS SIGNED AND DATED CONSENT FORM

____

Name of Participant (printed)

____

Signature or Fingerprint of Participant   Date/Time

____

Name of Investigator Administering Consent (printed)   Position/Title

____

Signature of Investigator Administering Consent   Date/Time

If the participant is unable to read and/or write, an impartial witness should be present during the entire informed consent discussion.

After the written informed consent form is read and explained to the participant, and after they have orally consented to participate in the study, the witness should print the name of the participant above, and then the participant should provide their fingerprint.

Then the witness should print their name, provide their signature, and date the consent form below.

By signing the consent form, the witness attests that the information in the consent form and any other written information was accurately explained to, and apparently understood by the participant, and that informed consent was freely given by the participant.

____

Name of Person Witnessing Consent (printed)

____

Signature of Person Witnessing Consent   Date/Time

THE REPUBLIC OF UGANDA

[____]—[____|____|____|____]

SFQ Study ID

APPENDIX B. SELF-FILLED QUESTIONNAIRES
Informed consent form for trainers

**Protocol Title:**	ACT PROCESS: Evaluating the process, context, and impact of interventions to enhance health facilities in Tororo, Uganda
**Site of Research:**	Tororo, Uganda
**Principal Investigators**:	Dr. Sarah Staedke
**Date:**	23 February 2011

Introduction

Dr. Sarah Staedke and colleagues from the Uganda Malaria Surveillance Project/Infectious Diseases Research Collaboration are conducting a research study to see if we can improve the health of children in this area by improving services at government-run health facilities. Part of this study intervention includes training for health workers on the following topics: (1) health centre management, (2) fever case management and use of rapid diagnostic tests for malaria, (3) patient-centred services. You have been invited to take part in this study because of your role in delivering the training. We are asking for your feedback on the training session you delivered.

Why is this study being done?

We would like to know more about how our training sessions were delivered in this area. To do this, we are asking all trainers who take part in delivering the training for their opinions on the session they delivered. This information will help us understand how the training session was delivered from your perspective and what you thought about the training methods, content, and objectives.

What will happen today if I take part in this study?

Today, we are inviting you to complete a questionnaire on your own regarding the training session. We can answer any questions you may have about the questionnaire or how to complete it. You will be asked to complete a questionnaire at the end of each training session you deliver. We will collect your questionnaires for analysis. After you complete the questionnaire, we will not ask you to do anything further. The information we collect will be used by project investigators, and may be shared with other researchers and policy-makers to answer questions about how best to deliver training and health services.

How long will the study last?
The training sessions will be conducted over 8-10 weeks. At the end of each training session you deliver, you will be asked to complete a questionnaire. Each questionnaire will take about 30 minutes to complete.

Can I stop being in the study?
You can decide to stop participating at any time. Just tell the project researcher right away.

What risks can I expect from being in the study?
Participation in any research study may involve a loss of privacy. Your name will not be used in any reports of the information provided. No quotes or other results arising from your participation in this study will be included in any reports, even anonymously, without your agreement. The information obtained from these questionnaires will be locked at our project offices. We will do our best to make sure that the personal information gathered is kept private.

Are there benefits to taking part in the study?
By participating as a trainer in our program, you will have the chance to learn new information and skills, or refresh information and skills you have already learned. There will be no other direct benefit to you from participating in this study. However, the feedback that you provide about the training sessions will help researchers and policy-makers understand how best to improve health services in this area, especially in the treatment of malaria.

What other choices do I have if I do not take part in this study?
You are free to choose not to take part in the study. If you decide not to take part in this study, there will be no penalty to you.

What are the costs of taking part in this study? Will I be paid for taking part in this study?
There are no costs to you for taking part in this study. You will not be paid for taking part in this study.

What are my rights if I take part in this study?
Taking part in this study is your choice. You may choose either to take part or not to take part in the study. If you decide to take part in this study, you may change your mind at any time. No matter what decision you take, there will be no penalty to you in any way.

Who can answer my questions about the study?
You can talk to the researchers about any questions or concerns you have about these study activities. Contact Dr. Sarah Staedke or other members of the Uganda Malaria Surveillance Project / Infectious Disease Research Collaboration on telephone number 0414-530692. If you have any questions, comments or concerns about taking part in these activities, first talk to the researchers. If for any reason you do not wish

to do this, or you still have concerns about doing so, you may contact Dr Charles Ibingira, Makerere University Faculty of Medicine Research and Ethical Committee at telephone number 0414-530020.

WHAT YOUR SIGNATURE OR THUMBPRINT MEANS

Your signature or thumbprint below means that you understand the information given to you in this consent form about your participation in the study and agree with the following statements:

1. "I have read the consent form concerning this study (or have understood the verbal explanation of the consent form) and I understand what will be required of me and what will happen to me if I take part in it."

2. "My questions concerning this study have been answered by Dr. Staedke or the person who signed below."

3. "I understand that at any time, I may withdraw from this study without giving a reason."

4. "I agree to take part in this study."

If you wish to participate in this study, you should sign or place your thumbprint on the following page. If you do not agree to quotes or other results arising from your participation in the study being included, even anonymously, in any reports about the study, please tell the researcher now.

THE REPUBLIC OF UGANDA

WE WILL GIVE YOU A COPY OF THIS SIGNED AND DATED CONSENT FORM

Name of Participant (printed)

Signature or Fingerprint of Participant Date/Time

Name of Investigator Administering Consent (printed) Position/Title

Signature of Investigator Administering Consent Date/Time

If the participant is unable to read and/or write, an impartial witness should be present during the entire informed consent discussion.

After the written informed consent form is read and explained to the participant, and after they have orally consented to participate in the study, the witness should print the name of the participant above, and then the participant should provide their fingerprint.

Then the witness should print their name, provide their signature, and date the consent form below.

By signing the consent form, the witness attests that the information in the consent form and any other written information was accurately explained to, and apparently understood by the participant, and that informed consent was freely given by the participant.

Name of Person Witnessing Consent (printed)

Signature of Person Witnessing Consent Date/Time

APPENDIX C: HEALTH CENTRE MANAGEMENT TRAINING SFQs

PART 1: TRAINER DEMOGRAPHIC BACKGROUND FORM

If you are leading multiple training modules, you only need to complete this form once.

Thank you for assisting with the delivery of these training workshops. Please take a moment to answer the following questions. There is no need to write your name on this questionnaire, and all responses will be kept strictly confidential. Thank you!

Trainer Study ID [____|____]

Please complete the boxes below with the relevant numbers	
**1. Age in years** [____|____]	**2. Gender** 1 = Male, 2 = Female [____]

3. Qualifications [____|____]

01 = Senior medical Officer
02 = Medical Officer
03 = Senior clinical Officer
04 = Clinical Officer
05 = Nursing Officer
06 = Enrolled nurse
07 = Comprehensive nurse
08 = Midwife
09 = Public health nurse
10 = Nursing aide/assistant
11 = Laboratory technician
12 = Laboratory assistant
13 = Health assistant
14 = Health educator
15 = Other ___________________________________

4. What training courses have you delivered in the past 3 years?

	Title of training delivered	Organization	Dates [dd/mm/yy] to [dd/mm/yy]
4a			[_____/_____/_____] to [_____/_____/_____]
4b			[_____/_____/_____] to [_____/_____/_____]
4c			[_____/_____/_____] to [_____/_____/_____]

5. What training courses have you attended in training methods?

	Title of training you attended	Organization	Dates [dd/mm/yy]
5a			[_____/_____/_____]
5b			[_____/_____/_____]
5c			[_____/_____/_____]

Please continue overleaf if you have delivered/attended more than 3 training workshops.

APPENDIX C: HEALTH CENTRE MANAGEMENT TRAINING
SFQ for TRAINERS – MODULE: PHC FUND ACCOUNTING (HCM01)

'Thank you for assisting with this health centre management training! We would appreciate your feedback on the training session you delivered today. Please take a moment to answer the following questions. Your comments will help us to know what information this group received and how well they achieved our intended learning objectives during this session. This will help us to know how to interpret any future change of practice amongst these health workers. There is no need to write your name on this questionnaire, and all responses will be kept strictly confidential. Thank you!'

**Trainer Study ID**	**Date of training**	**Study ID of other Trainers present**
[____|____]	[____|____]/ [____|____]/[____|____]<br>day month year	[____|____], [____|____]
**Training group #**	**Total # of participants invited**	**Total # of participants attended**
[____|____]	[____|____]	[____|____]

Participant Study IDs

[____|____|____|____]	[____|____|____|____]	[____|____|____|____]	[____|____|____|____]
[____|____|____|____]	[____|____|____|____]	[____|____|____|____]	[____|____|____|____]
[____|____|____|____]	[____|____|____|____]	[____|____|____|____]	[____|____|____|____]
[____|____|____|____]	[____|____|____|____]	[____|____|____|____]	[____|____|____|____]

TRAINER QUESTIONNAIRE

**1. Did the training start more than half an hour late?**	1 = Yes<br>2 = No	[____]
**2. If yes, what was the cause of the delay?**	1 = Participants arrived late<br>2 = Materials and supplies to be used were not delivered in time<br>3 = Trainer transport difficulties<br>4 = Other ______________________________	[____]

3. Please describe the consequence of the delay on your ability to deliver the training as intended.

Training component	Consequence of delay on trainer's ability to deliver the training as intended
**HCM00 – Introduction to HCM**	
**New topics introduced in the module**	
**Practice activities for the PHC Fund Management Tool**	

**4. What level of contribution did the participants have?**	1 = All contributed a lot<br>2 = All contributed at some point<br>3 = Only some contributed<br>4 = None contributed	[____]

5. For Qn. 4, responses 2 and 3 please list the 3 least active participants.

[____|____|____|____] [____|____|____|____] [____|____|____|____]

HEALTH CENTRE MANAGEMENT TRAINING
SFQ for TRAINERS – MODULE: PHC FUND ACCOUNTING (HCM01)

Trainer Study ID	Date of training
[____|____]	[____|____]/ [____|____]/[____|____]<br>day month year

6. Please complete the table below to describe how this training session went for each topic.

Topic	Teaching as planned?	Materials as planned?	If no, please explain
**Introduction to HCM - accountability**	1 = Yes<br>2 = No<br>[____]	1 = Yes<br>2 = No<br>[____]	
**Budgeting**	1 = Yes<br>2 = No<br>[____]	1 = Yes<br>2 = No<br>[____]	
**Accounting**	1 = Yes<br>2 = No<br>[____]	1 = Yes<br>2 = No<br>[____]	

7. Can you please describe any difficulties/barriers you encountered in delivery of the training?

**Barriers** to optimum implementation (e.g. - training interrupted, difficult or over bearing individuals, etc.)	**Impact** of barrier on the intended delivery of training and on learning

8. Please score how well you think the participants achieved each of the learning objectives today on a scale of 1-10.

(1=Not achieved, 10= Achieved in full. Use 'N/A' if the section is not included in topic)

Learning objectives	Score	Comments
Participants should be able to describe the Ministry of Health policy for PHC Funds for HC II/IIIs		
Participants should be able to Understand how budgeting and accounting contributes to showing accountability and skill as an in-charge		
Participants should be able to Describe the importance and purpose of budgeting and accounting for the PHC Fund		
Participants should be able to Develop and apply budgeting and accounting skills using the PHC Fund Management Tool		
Participants should be able to Plan and commit to completing the PHC Fund Management tool regularly at their health centres		

HEALTH CENTRE MANAGEMENT TRAINING<br>SFQ for TRAINERS – MODULE: PHC FUND ACCOUNTING (HCM01)	
**Trainer Study ID**<br>[____|____]	**Date of training**<br>[____|____]/ [____|____]/[____|____]<br>day month year

**9. How many questions or concerns were raised by this group about the topics covered today?**	1 = Many questions<br>2 = Few questions<br>3 = None	[____]
**10. What questions or concerns were raised by this group about the topics discussed today? (Please list)**		
**11. How did you address each of these concerns in this group?**		
**12. Which of these concerns do you think were still present at the end of the training?**		
**13. Would you change anything about the session you gave to this group today? If yes, what would you change? How would you change it?**		
**14. What is your overall assessment of how well the intended objectives of today's training were achieved?**	1 = Badly<br>2 = Fine<br>3 = Good	[____]

APPENDIX C: HEALTH CENTRE MANAGEMENT TRAINING
SFQ for TRAINERS – MODULE: DRUG SUPPLY MANAGEMENT (HCM02)

'Thank you for assisting with this health centre management training! We would appreciate your feedback on the training session you delivered today. Please take a moment to answer the following questions. Your comments will help us to know what information this group received and how well they achieved our intended learning objectives during this session. This will help us to know how to interpret any future change of practice amongst these health workers. There is no need to write your name on this questionnaire, and all responses will be kept strictly confidential. Thank you!'

Trainer Study ID	Date of training	Study ID of other Trainers present
[____|____]	[____|____]/ [____|____]/[____|____]<br>day month year	[____|____], [____|____]
**Training group #**	**Total # of participants invited**	**Total # of participants attended**
[____|____]	[____|____]	[____|____]

Participant Study IDs			
[____|____|____|____]	[____|____|____|____]	[____|____|____|____]	[____|____|____|____]
[____|____|____|____]	[____|____|____|____]	[____|____|____|____]	[____|____|____|____]
[____|____|____|____]	[____|____|____|____]	[____|____|____|____]	[____|____|____|____]
[____|____|____|____]	[____|____|____|____]	[____|____|____|____]	[____|____|____|____]

TRAINER QUESTIONNAIRE		
**1. Did the training start more than half an hour late?**	1 = Yes<br>2 = No	[____]
**2. If yes, what was the cause of the delay?**	1 = Participants arrived late<br>2 = Materials and supplies to be used were not delivered in time<br>3 = Trainer transport difficulties<br>4 = Other ____	[____]

3. Please describe the consequence of the delay on your ability to deliver the training as intended

Training component	Consequence of delay on trainer's ability to deliver the training as intended
**New topics introduced in the module**	
**Practice activities for completing the Stock Card and Requisition & Issue Voucher**	
**Practice activities for completing the ADDAT**	

**4. What level of contribution did the participants have?**	1 = All contributed a lot<br>2 = All contributed at some point<br>3 = Only some contributed<br>4 = None contributed	[____]
**5. For Qn. 4, responses 2 and 3 please list the 3 least active participants.**		
[____|____|____|____]	[____|____|____|____]	[____|____|____|____]

HEALTH CENTRE MANAGEMENT TRAINING
SFQ for TRAINERS – MODULE: DRUG SUPPLY MANAGEMENT (HCM02)

Trainer Study ID	Date of training
[____|____]	[____|____]/ [____|____]/[____|____]<br>day month year

6. Please complete the table below to describe how this training session went for each topic.

Topic	Teaching as planned?	Materials as planned?	If no, please explain
**Drug distribution system**	1 = Yes<br>2 = No<br>[____]	1 = Yes<br>2 = No<br>[____]	
**Stock Card and Requisition & Issue voucher**	1 = Yes<br>2 = No<br>[____]	1 = Yes<br>2 = No<br>[____]	
**ADDAT**	1 = Yes<br>2 = No<br>[____]	1 = Yes<br>2 = No<br>[____]	

7. Can you please describe any difficulties/barriers you encountered in delivery of the training?

**Barriers** to optimum implementation (e.g. - training interrupted, difficult or over bearing individuals, etc.)	**Impact** of barrier on the intended delivery of training and on learning

8. Please score how well you think the participants achieved each of the learning objectives today on a scale of 1-10.
(1=Not achieved, 10= Achieved in full. Use 'N/A' if the section is not included in topic)

Learning objectives	Score	Comments
Describe the main components of the drug distribution system		
Be motivated to actively participate in and keep the drug distribution system on track		
Describe the purpose and benefit of completing forms required in the drug distribution system		
Accurately complete and put in place a plan for completing the forms required in the drug distribution system		
Identify issues that prevent drugs from reaching the health centre		
Identify and implement solutions to the issues that prevent drugs from reaching the health centre		
Be motivated to complete the ADDAT regularly		

HEALTH CENTRE MANAGEMENT TRAINING<br>SFQ for TRAINERS – MODULE: DRUG SUPPLY MANAGEMENT (HCM02)	
**Trainer Study ID**<br>[____|____]	**Date of training**<br>[____|____]/ [____|____]/[____|____]<br>day month year

**9. How many questions or concerns were raised by this group about the topics covered today?**	1 = Many questions<br>2 = Few questions<br>3 = None [____]
**10. What questions or concerns were raised by this group about the topics discussed today? (Please list)**	
**11. How did you address each of these concerns in this group?**	
**12. Which of these concerns do you think were still present at the end of the training?**	
**13. Would you change anything about the session you gave to this group today? If yes, what would you change? How would you change it?**	
**14. What is your overall assessment of how well the intended objectives of today's training were achieved?**	1 = Badly<br>2 = Fine<br>3 = Good [____]

APPENDIX C: HEALTH CENTRE MANAGEMENT TRAINING
SFQ for TRAINERS – MODULE: INFORMATION MANAGEMENT (HCM03)

'Thank you for assisting with this health centre management training! We would appreciate your feedback on the training session you delivered today. Please take a moment to answer the following questions. Your comments will help us to know what information this group received and how well they achieved our intended learning objectives during this session. This will help us to know how to interpret any future change of practice amongst these health workers. There is no need to write your name on this questionnaire, and all responses will be kept strictly confidential. Thank you!'

Trainer Study ID	Date of training	Study ID of other Trainers present
[____|____]	[____|____]/ [____|____]/[____|____]<br>day month year	[____|____], [____|____]
**Training group #**	**Total # of participants invited**	**Total # of participants attended**
[____|____]	[____|____]	[____|____]

Participant Study IDs			
[____|____|____|____]	[____|____|____|____]	[____|____|____|____]	[____|____|____|____]
[____|____|____|____]	[____|____|____|____]	[____|____|____|____]	[____|____|____|____]
[____|____|____|____]	[____|____|____|____]	[____|____|____|____]	[____|____|____|____]
[____|____|____|____]	[____|____|____|____]	[____|____|____|____]	[____|____|____|____]

TRAINER QUESTIONNAIRE		
**1. Did the training start more than half an hour late?**	1 = Yes<br>2 = No	[____]
**2. If yes, what was the cause of the delay?**	1 = Participants arrived late<br>2 = Materials and supplies to be used were not delivered in time<br>3 = Trainer transport difficulties<br>4 = Other ______________________________	[____]

3. Please describe the consequence of the delay on your ability to deliver the training as intended

Training component	Consequence of delay on trainer's ability to deliver the training as intended
**New topics introduced in the module**	
**Practice activities for using information**	
**Planning activities for using information**	

**4. What level of contribution did the participants have?**	1 = All contributed a lot<br>2 = All contributed at some point<br>3 = Only some contributed<br>4 = None contributed	[____]
**5. For Qn. 4, responses 2 and 3 please list the 3 least active participants.**		
[____|____|____|____]	[____|____|____|____]	[____|____|____|____]

HEALTH CENTRE MANAGEMENT TRAINING<br>SFQ for TRAINERS – MODULE: INFORMATION MANAGEMENT (HCM03)	
**Trainer Study ID**<br>[____|____]	**Date of training**<br>[____|____]/ [____|____]/[____|____]<br>day month year

6. Please complete the table below to describe how this training session went for each topic.

Topic	Teaching as planned?	Materials as planned?	If no, please explain
**Information management**	1 = Yes<br>2 = No<br>[____]	1 = Yes<br>2 = No<br>[____]	
**Continuous quality improvement**	1 = Yes<br>2 = No<br>[____]	1 = Yes<br>2 = No<br>[____]	

7. Can you please describe any difficulties/barriers you encountered in delivery of the training?

<u>Barriers</u> to optimum implementation (e.g. - training interrupted, difficult or over bearing individuals, etc.)	<u>Impact</u> of barrier on the intended delivery of training and on learning

8. Please score how well you think the participants achieved each of the learning objectives today on a scale of 1-10.

(1=Not achieved, 10= Achieved in full. Use 'N/A' if the section is not included in topic)

Learning objectives	Score	Comments
Describe the importance of collecting complete & accurate information on each patient		
Understand what patient information is used for		
Understand how collecting information can beneficial to the health centre (drug quantification, predicting future needs)		
Understand how collecting information improves patient management		

HEALTH CENTRE MANAGEMENT TRAINING<br>SFQ for TRAINERS – MODULE: INFORMATION MANAGEMENT (HCM03)	
**Trainer Study ID**<br>[____|____]	**Date of training**<br>[____|____]/ [____|____]/[____|____]<br>day month year

**9. How many questions or concerns were raised by this group about the topics covered today?**	1 = Many questions<br>2 = Few questions<br>3 = None	[____]
**10. What questions or concerns were raised by this group about the topics discussed today? (Please list)**		
**11. How did you address each of these concerns in this group?**		
**12. Which of these concerns do you think were still present at the end of the training?**		
**13. Would you change anything about the session you gave to this group today? If yes, what would you change? How would you change it?**		
**14. What is your overall assessment of how well the intended objectives of today's training were achieved?**	1 = Badly<br>2 = Fine<br>3 = Good	[____]

APPENDIX D: HEALTH CENTRE MANAGEMENT TRAINING SFQ

PART 1: PARTICIPANT DEMOGRAPHIC BACKGROUND FORM

If you are attending multiple training modules, you only need to complete this form once.

Thank you for participating in this training course. We would like to collect some information about you and your work history. Please take a moment to answer the following questions. There is no need to write your name on this questionnaire. All responses will be kept strictly confidential. Thank you!

Please complete the boxes below with your own information. Please ask if you have any questions.

Participant PRIME Study ID [____|____]

Please complete the questions below

1. What is your age in years? [____|____] years

2. What is your gender? (please circle) Male Female

3. How long have you worked at this health centre? [____|____] and [____|____]
years months

4. If you are an in-charge, how long have you actively worked as an in-charge? [____|____] and [____|____]
years months

5. What is your education? Please circle all levels completed

Primary
Senior four
Senior six
Vocational certificate
University
Others *(please specify)*
____
____

6. What year did you completed your highest level of education (schooling)? [____|____|____|____]
year

7. What is your current position? Please select from the list below and write the appropriate number here: [____|____]

01 = Senior medical Officer
02 = Medical Officer
03 = Senior clinical Officer
04 = Clinical Officer
05 = Nursing Officer
06 = Enrolled nurse
07 = Comprehensive nurse
08 = Midwife
09 = Public health nurse
10 = Nursing aide/assistant
11 = Laboratory technician
12 = Laboratory assistant
13 = Health assistant
14 = Health educator
15 = Other
____

8. What year did you start working in this position? [____|____|____|____]
year

9. What training workshops have you attended in the past 3 years? Please complete the table below

	**Title of training you attended**	**Organization**	**Date** [dd/mm/yy]
**9a**			[_____/_____/_____]
**9b**			[_____/_____/_____]
**9c**			[_____/_____/_____]

APPENDIX D: HEALTH CENTRE MANAGEMENT TRAINING
SFQ for PARTICIPANTS – PHC FUND MANAGEMENT (HCM01)

Thank you for participating in this health centre management training! We would appreciate your feedback on the training session you have attended. Please take a moment to answer the following questions. This is **NOT A TEST** and **WE VALUE YOUR OPINION**. Your opinions will help us improve future trainings. There is no need to write your name on this questionnaire.
All responses will be kept strictly confidential. Thank you!

GENERAL INSTRUCTIONS TO COMPLETE THIS QUESTIONNAIRE

1. *Please use a dark coloured pen to fill out the questionnaire*

2. *The health worker identity (ID) number is the unique number that was given to you at the start of this PRIME project. When we request you to give your ID number, please fill the boxes as below:*

   [_0_ | _5_ | _1_ | _0_]

   You are asked to enter your ID number like this at the top of each page of your questionnaire.

3. *In this questionnaire, we ask you to read each question carefully and either write your response, or look at the statements and decide which response is closest to your own opinion. When you have decided, please circle the number in the column under that response. For example:*

Decide how much you agree with the statements below	***Strongly agree***	***Agree***	***Disagree***	***Strongly disagree***
I learned new ideas today	*1* (circled)	*2*	*3*	*4*

4. *If you change your mind and would like to circle a different response, please cross out your original choice and circle the choice that fits your opinion best. For example, if you change your mind and you decide that your answer is 'disagree', cross through the original and circle the new response as below:*

Decide how much you agree with the statements below	***Strongly agree***	***Agree***	***Disagree***	***Strongly disagree***
I learned new ideas today	*1* (circled and crossed out)	*2*	*3* (circled)	*4*

5. *When you have completed the questionnaire, please make sure you have completed everything in the following checklist:*

For the first time you attend a workshop <u>only</u>:	☐ *[For the first time only] I have signed and dated the consent form*<br>☐ *[For the first time only] I have completed my demographic details form*
For every workshop:	☐ *I have answered all of the questions in this questionnaire*<br>☐ *I have checked that I have circled the responses that are closest to my opinion*<br>☐ *I have written my health worker ID number on all of the pages of the questionnaire*<br>☐ *I have written my health worker ID number on the envelope and will place this completed form inside the envelope*

HEALTH CENTRE MANAGEMENT TRAINING<br>SFQ for PARTICIPANTS – PHC FUND MANAGEMENT (HCM01)	
**Health worker ID**<br>[____|____|____|____]	**Today's date**<br>[____|____]/ [____|____]/[____|____]<br>day month year

1. **Please complete the table below about your <u>learning today</u> by circling the response closest to your opinion.**

Decide how much you agree with the statements below	Strongly agree	Agree	Disagree	Strongly disagree
I learned new ideas today	1	2	3	4
I understood everything the trainer taught today	1	2	3	4
I felt the training was relevant to my daily work	1	2	3	4
The ideas I learned today will be useful for me in the future	1	2	3	4
I was given a chance to ask all of my questions	1	2	3	4
I felt that the trainer answered all our questions well	1	2	3	4
I felt able to practice new skills in the workshop today	1	2	3	4
I was given a chance to give my opinions	1	2	3	4
I will recommend my colleagues to attend this training	1	2	3	4
**If you have any comments on the above statements, Please write them below:**				

HEALTH CENTRE MANAGEMENT TRAINING<br>SFQ for PARTICIPANTS – PHC FUND MANAGEMENT (HCM01)	
**Health worker ID**	**Today's date**
[____|____|____|____]	[____|____]/ [____|____]/[____|____]<br>day month year

2. **Please complete the table below about the skills you have learnt from the training topic, by circling the response closest to your opinion.**

Decide how much you agree with the statements below	Strongly agree	Agree	Disagree	Strongly disagree
This training helped me to think about accountability	1	2	3	4
This training has helped me to see the importance of budgeting and accounting for the PHC Fund	1	2	3	4
This training has given me ideas for how to show accountability in my work as an in-charge	1	2	3	4
After this training, I feel able to change the way I manage PHC Funds at my health centre	1	2	3	4
After this training, I have found ways to management funds at my health centre using the PHC Fund Management Tool	1	2	3	4
**Please write any comments on the above statements in this section here:**				

3. **Please complete the table below about today's overall training by circling the response closest to your opinion.**

	Very good	Good	Fair	Poor	Very poor
What is your opinion of the trainer's skills today?	1	2	3	4	5
What is your opinion of the trainer's attitude today?	1	2	3	4	5
What is your opinion of the learner's manual you received today?	1	2	3	4	5
What is your opinion of the flip charts that were used today?	1	2	3	4	5
Overall, what is your opinion of the training today?	1	2	3	4	5

HEALTH CENTRE MANAGEMENT TRAINING<br>SFQ for PARTICIPANTS – PHC FUND MANAGEMENT (HCM01)	
**Health worker ID**<br>[____|____|____|____]	**Today's date**<br>[____|____]/ [____|____]/[____|____]<br>day month year

4. Please write anything else you would like to say about today's training

NOW PLEASE ENSURE YOU HAVE COMPLETED THIS FORM FULLY
BY GOING THROUGH THE CHECK SHEET ON THE FRONT PAGE

APPENDIX D: HEALTH CENTRE MANAGEMENT TRAINING

SFQ for PARTICIPANTS – DRUG SUPPLY MANAGEMENT(HCM02)

Thank you for participating in this health centre management training! We would appreciate your feedback on the training session you have attended. Please take a moment to answer the following questions. This is **NOT A TEST** and **WE VALUE YOUR OPINION**. Your opinions will help us improve future trainings. There is no need to write your name on this questionnaire.
All responses will be kept strictly confidential. Thank you!

GENERAL INSTRUCTIONS TO COMPLETE THIS QUESTIONNAIRE

6. *Please use a dark coloured pen to fill out the questionnaire*

7. *The health worker identity (ID) number is the unique number that was given to you at the start of this PRIME project. When we request you to give your ID number, please fill the boxes as below:*

   [_0_ | _5_ | _1_ | _0_]

   You are asked to enter your ID number like this at the top of each page of your questionnaire.

8. *In this questionnaire, we ask you to read each question carefully and either write your response, or look at the statements and decide which response is closest to your own opinion. When you have decided, please circle the number in the column under that response. For example:*

Decide how much you agree with the statements below	***Strongly agree***	***Agree***	***Disagree***	***Strongly disagree***
I learned new ideas today	*(1)*	*2*	*3*	*4*

9. *If you change your mind and would like to circle a different response, please cross out your original choice and circle the choice that fits your opinion best. For example, if you change your mind and you decide that your answer is 'disagree', cross through the original and circle the new response as below:*

Decide how much you agree with the statements below	***Strongly agree***	***Agree***	***Disagree***	***Strongly disagree***
I learned new ideas today	*~~(1)~~*	*2*	*(3)*	*4*

10. *When you have completed the questionnaire, please make sure you have completed everything in the following checklist:*

For the first time you attend a workshop <u>only</u>:	☐ *[For the first time only] I have signed and dated the consent form*<br>☐ *[For the first time only] I have completed my demographic details form*
For every workshop:	☐ *I have answered all of the questions in this questionnaire*<br>☐ *I have checked that I have circled the responses that are closest to my opinion*<br>☐ *I have written my health worker ID number on all of the pages of the questionnaire*<br>☐ *I have written my health worker ID number on the envelope and will place this completed form inside the envelope*

HEALTH CENTRE MANAGEMENT TRAINING<br>SFQ for PARTICIPANTS – DRUG SUPPLY MANAGEMENT(HCM02)	
**Health worker ID**	**Today's date**
[____|____|____|____]	[____|____]/ [____|____]/[____|____]<br>day month year

1. **Please complete the table below about your <u>learning today</u> by circling the response closest to your opinion.**

Decide how much you agree with the statements below	Strongly agree	Agree	Disagree	Strongly disagree
I learned new ideas today	1	2	3	4
I understood everything the trainer taught today	1	2	3	4
I felt the training was relevant to my daily work	1	2	3	4
The ideas I learned today will be useful for me in the future	1	2	3	4
I was given a chance to ask all of my questions	1	2	3	4
I felt that the trainer answered all our questions well	1	2	3	4
I felt able to practice new skills in the workshop today	1	2	3	4
I was given a chance to give my opinions	1	2	3	4
I will recommend my colleagues to attend this training	1	2	3	4
**If you have any comments on the above statements, Please write them below:**				

HEALTH CENTRE MANAGEMENT TRAINING
SFQ for PARTICIPANTS – DRUG SUPPLY MANAGEMENT(HCM02)

Health worker ID	Today's date
[____|____|____|____]	[____|____]/ [____|____]/[____|____]<br>day month year

2. Please complete the table below about the skills you have learnt from <u>the training topic</u>, by circling the response closest to your opinion.

Decide how much you agree with the statements below	Strongly agree	Agree	Disagree	Strongly disagree
This training helped me to think about my role as an in-charge in the drug distribution system	1	2	3	4
This training has helped me to see the importance of doing my part to keep the drug distribution system on track	1	2	3	4
This training has given me ideas for how to make sure drugs reach my health centre using the ADDAT	1	2	3	4
After this training, I feel able to change the way I manage drugs at my health centre	1	2	3	4
After this training, I have found ways to complete the Drug Stock Card and Requisition & Issue Voucher	1	2	3	4
**Please write any comments on the above statements in this section here:**				

3. Please complete the table below about today's <u>overall training</u> by circling the response closest to your opinion.

	Very good	Good	Fair	Poor	Very poor
What is your opinion of the trainer's skills today?	1	2	3	4	5
What is your opinion of the trainer's attitude today?	1	2	3	4	5
What is your opinion of the learner's manual you received today?	1	2	3	4	5
What is your opinion of the flip charts that were used today?	1	2	3	4	5
Overall, what is your opinion of the training today?	1	2	3	4	5

HEALTH CENTRE MANAGEMENT TRAINING<br>SFQ for PARTICIPANTS – DRUG SUPPLY MANAGEMENT(HCM02)	
**Health worker ID**<br>[_____|_____|_____|_____]	**Today's date**<br>[_____|_____]/ [_____|_____]/[_____|_____]<br>day month year

4. Please write anything else you would like to say about today's training

NOW PLEASE ENSURE YOU HAVE COMPLETED THIS FORM FULLY
BY GOING THROUGH THE <u>CHECK SHEET</u> ON THE FRONT PAGE

APPENDIX D: HEALTH CENTRE MANAGEMENT TRAINING
SFQ for PARTICIPANTS – INFORMATION MANAGEMENT (HCM03)

Thank you for participating in this health centre management training! We would appreciate your feedback on the training session you have attended. Please take a moment to answer the following questions. This is **NOT A TEST** and **WE VALUE YOUR OPINION**. Your opinions will help us improve future trainings. There is no need to write your name on this questionnaire. All responses will be kept strictly confidential. Thank you!

GENERAL INSTRUCTIONS TO COMPLETE THIS QUESTIONNAIRE

11. Please use a dark coloured pen to fill out the questionnaire

12. The health worker identity (ID) number is the unique number that was given to you at the start of this PRIME project. When we request you to give your ID number, please fill the boxes as below:

[_0_ | _5_ | _1_ | _0_]

You are asked to enter your ID number like this at the top of each page of your questionnaire.

13. In this questionnaire, we ask you to read each question carefully and either write your response, or look at the statements and decide which response is closest to your own opinion. When you have decided, please circle the number in the column under that response. For example:

Decide how much you agree with the statements below	***Strongly agree***	***Agree***	***Disagree***	***Strongly disagree***
I learned new ideas today	*(1)*	*2*	*3*	*4*

14. If you change your mind and would like to circle a different response, please cross out your original choice and circle the choice that fits your opinion best. For example, if you change your mind and you decide that your answer is 'disagree', cross through the original and circle the new response as below:

Decide how much you agree with the statements below	***Strongly agree***	***Agree***	***Disagree***	***Strongly disagree***
I learned new ideas today	*~~(1)~~*	*2*	*(3)*	*4*

15. When you have completed the questionnaire, please make sure you have completed everything in the following checklist:

For the first time you attend a workshop only:	☐ *[For the first time only] I have signed and dated the consent form*<br>☐ *[For the first time only] I have completed my demographic details form*
For every workshop:	☐ *I have answered all of the questions in this questionnaire*<br>☐ *I have checked that I have circled the responses that are closest to my opinion*<br>☐ *I have written my health worker ID number on all of the pages of the questionnaire*<br>☐ *I have written my health worker ID number on the envelope and will place this completed form inside the envelope*

HEALTH CENTRE MANAGEMENT TRAINING<br>SFQ for PARTICIPANTS – INFORMATION MANAGEMENT (HCM03)	
**Health worker ID**<br>[____|____|____|____]	**Today's date**<br>[____|____]/ [____|____]/[____|____]<br>day month year

1. **Please complete the table below about your <u>learning today</u> by circling the response closest to your opinion.**

Decide how much you agree with the statements below	Strongly agree	Agree	Disagree	Strongly disagree
I learned new ideas today	1	2	3	4
I understood everything the trainer taught today	1	2	3	4
I felt the training was relevant to my daily work	1	2	3	4
The ideas I learned today will be useful for me in the future	1	2	3	4
I was given a chance to ask all of my questions	1	2	3	4
I felt that the trainer answered all our questions well	1	2	3	4
I felt able to practice new skills in the workshop today	1	2	3	4
I was given a chance to give my opinions	1	2	3	4
I will recommend my colleagues to attend this training	1	2	3	4
**If you have any comments on the above statements, Please write them below:**				

HEALTH CENTRE MANAGEMENT TRAINING<br>SFQ for PARTICIPANTS – INFORMATION MANAGEMENT (HCM03)	
**Health worker ID**<br>[____|____|____|____]	**Today's date**<br>[____|____]/ [____|____]/[____|____]<br>day month year

2. **Please complete the table below about the skills you have learnt from <u>the training topic</u>, by circling the response closest to your opinion.**

Decide how much you agree with the statements below	Strongly agree	Agree	Disagree	Strongly disagree
This training helped me to think about	1	2	3	4
This training has helped me to see	1	2	3	4
This training has given me ideas for how to	1	2	3	4
After this training, I feel able to change	1	2	3	4
After this training, I have found ways to	1	2	3	4
**Please write any comments on the above statements in this section here:**				

3. **Please complete the table below about today's <u>overall training</u> by circling the response closest to your opinion.**

	Very good	Good	Fair	Poor	Very poor
What is your opinion of the trainer's skills today?	1	2	3	4	5
What is your opinion of the trainer's attitude today?	1	2	3	4	5
What is your opinion of the learner's manual you received today?	1	2	3	4	5
What is your opinion of the flip charts that were used today?	1	2	3	4	5
Overall, what is your opinion of the training today?	1	2	3	4	5

HEALTH CENTRE MANAGEMENT TRAINING<br>SFQ for PARTICIPANTS – INFORMATION MANAGEMENT (HCM03)	
**Health worker ID**<br>[____|____|____|____]	**Today's date**<br>[____|____]/ [____|____]/[____|____]<br>day month year

4. Please write anything else you would like to say about today's training

NOW PLEASE ENSURE YOU HAVE COMPLETED THIS FORM FULLY BY GOING THROUGH THE CHECK SHEET ON THE FRONT PAGE

APPENDIX E: FEVER CASE MANAGEMENT TRAINING
SFQ for TRAINERS (1)

Thank you for assisting with this health centre management training! We would appreciate your feedback on the training sessions you attended on staffing. Please take a moment to answer the following questions, as your comments will help us improve future trainings. There is no need to write your name on this questionnaire, and all responses will be kept strictly confidential. Thank you!

Trainer Study ID	Date of training	Study ID of other Trainers present
[____|____]	[____|____]/ [____|____]/[____|____]<br>day month year	[____|____], [____|____], [____|____]

Training group number	Participant health facility IDs				
[_____|_____]	[____|____]	[____|____]	[____|____]	[____|____]	[____|____]
	[____|____]	[____|____]	[____|____]	[____|____]	[____|____]
	**Participant Study IDs**				
	[____|____]	[____|____]	[____|____]	[____|____]	[____|____]
	[____|____]	[____|____]	[____|____]	[____|____]	[____|____]

Characteristics of participants

Age	Sex	Qualifications	Other trainings attended

TRAINER QUESTIONNAIRE

1. Did the training start on time? If not, what was the cause of the delay?

2. What was the general atmosphere during the session?

3. What level of contribution did the participants have? [____]

1 = All contributed a lot
2 = All contributed at some point
3 = Only some contributed
4 = None contributed
(please specify health centres/IDs if possible) [____|____], [____|____], [____|____]

Please summarise your opinion of the course by ranking the following using: 1 = Poor, 2 = Fair, 3 = Good, 4 = Very good, 5 = N/A

4. Attendance of the session by the in-charges [____]

5. How the training materials were received [____]

6. How the training aids used were received (if applicable) [____]

7. Your overall assessment of how the training went [____]

8. Did the training go as planned? Please explain why. 1 = Yes, 2 = No [____]

9. Can you please describe any difficulties you encountered?

10. What impact did these have on the quality of the information received by the participants?

FEVER CASE MANAGEMENT TRAINING
SFQ for TRAINERS (2)

Trainer Study ID	Date of training
[____|____]	[____|____]/ [____|____]/[____|____]<br>day month year

Please indicate if the following items were available at the training 1 = Yes 2 = No 3 = N/A

	Provided	Amount sufficient		Provided	Amount sufficient
**11. Sharps bin**	[_____]	[_____]	**16. Blood transfer devices**	[_____]	[_____]
**12. Standard waste bin**	[_____]	[_____]	**17. Sealed packets of alcohol swabs**	[_____]	[_____]
**13. Pairs of latex or nitrite gloves**	[_____]	[_____]	**18. RDT buffet (check it matches the RDT)**	[_____]	[_____]
**14. Sealed packets of RDTs**	[_____]	[_____]	**19.Timer**	[_____]	[_____]
**15. Sealed packets of lancets**	[_____]	[_____]	**20. RDT bench aid (check it matches the RDT type)**	[_____]	[_____]

How useful do you think the following training activity was for the health centre clinical staff? 1 = Not very useful 2 = Somewhat useful 3 = Very useful 4 = Don't know

**21. Discussion of evaluation of febrile patients and selection of patients for RDT testing**	[_____]	**25. Discussion of recognition and referral of patients with severe illness**	[_____]
**22. Practice of performing and reading an RDT**	[_____]	**26. Discussion of patient education**	[_____]
**23. Discussion of management of a patient with fever and a positive RDT**	[_____]	**27. Discussion of RDT storage and monitoring**	[_____]
**24. Discussion of management of a patient with fever and a negative RDT**	[_____]		[_____]

13. What questions or concerns were raised by this group about the training of health workers on fever case management? (Please list)

14. How were each of these concerns addressed, if at all?

15. Which of these concerns do you think were still present at the end of the training?

16. Would you change anything about the fever case management training sessions for clinical staff? If yes, what would you change? How would you change it?

17. Do you have any general comments on this course?

Thank you!

APPENDIX F: FEVER CASE MANAGEMENT TRAINING SFQ for PARTICIPANTS (1)

Thank you for participating in this training on staffing! We would appreciate your feedback on the training sessions you have attended. Please take a moment to answer the following questions, as your comments will help us improve future trainings. There is no need to write your name on this questionnaire, and all responses will be kept strictly confidential. Thank you!

Health worker ID	Today's date	Date training began
[____]	[____|____]/ [____|____]/[____|____] day month year	[____|____]/ [____|____]/[____|____] day month year

1. Your qualification [____]	1 = Clinical Officer<br>2 = Nurse<br>3 = Other (list)<br>____________________	2. What is your age? [____|____] years	3. How long have you worked actively as health centre in-charge? [____|____] OR [____|____] months years

4. How many trainings have you attended for health centre clinical staff in the past? [____|____]	5. When was the last training you attended and what was the topic of training? Topic [___|___]/ [___|___]/[___|___] day month year

6. What other PRIME courses have you attended so far?

TRAINEE QUESTIONNAIRE

Please summarise your opinion of the course by ranking the following using: 1 = Poor, 2 = Fair, 3 = Good, 4 = Very good, 5 = N/A

**7. Achievement of your aims when you enrolled in this training**	[____]	**10. Use of Training Aids (if applicable)**	[____]
**8. General achievements of the course objectives**	[____]	**11. Your overall assessment**	[____]
**9. Effectiveness of Trainer(s)**	[____]		

12. How interested do you think most of the other clinical staff in your group were throughout the training sessions? 1 = Not Very Interested, 2 = Somewhat Interested, 3 = Very Interested [____]

How useful did you find each of the following training activities? 1 = Not very useful, 2 = Somewhat useful, 3 = Very useful, 4 = Don't know

**13. Discussion of evaluation of febrile patients and selection of patients for RDT testing**	[____]	**17. Discussion of recognition and referral of patients with severe illness**	[____]
**14. Practice of performing and reading an RDT**	[____]	**18. Discussion of patient education**	[____]
**15. Discussion of management of a patient with fever and a positive RDT**	[____]	**19. Discussion of RDT storage and monitoring**	[____]
**16. Discussion of management of a patient with fever and a negative RDT**	[____]		[____]

15. What would you like to add or change about the training sessions?

16. Please write any concerns you have about fever case management or any other comments following up on this training?

17. Please write any general comments you have on this course?

FEVER CASE MANAGEMENT TRAINING
SFQ for PARTICIPANTS (2)

HW Study ID	Date of training
[____|____]	[____|____]/ [____|____]/[____|____]<br>day month year

Please summarise your opinion of the course by ranking the following using: 1 = Poor, 2 = Fair, 3 = Good, 4 = Very good, 5 = N/A

**18. I feel confident that I can do good history taking including asking good questions and active listening**	[____]	**24. I feel confident that I can manage the common non-malaria febrile illnesses according to treatment guidelines**	[____]
**19. I feel confident that I can perform a clinical examination on a patient with fever correctly**	[____]	**25. I feel confident that I can assess a patient for severe signs of illness**	[____]
**20. I feel confident that I can select a patient for RDT testing based on clinical evaluation**	[____]	**26. I feel confident that I can properly refer a patient when they are severely ill to higher level facilities**	[____]
**21. I feel confident that I can perform an RDT 22. correctly and safely**	[____]	**27. I feel confident that I can provide pre-referral treatments to severely ill patients**	[____]
**22. I feel confident that I can treat a patient with fever and a positive RDT according to national guidelines**	[____]	**28. I feel confident that I can use good communication skills when giving patients information about malaria and its treatment**	[____]
**23. I feel confident that I can manage a patient with fever but a negative RDT**	[____]	**29. I feel confident that I can store and monitor RDTs' expiry dates correctly**	[____]

Thank you!

APPENDIX G: PATIENT CENTRED SERVICES SFQs

PART 1: TRAINER DEMOGRAPHIC BACKGROUND FORM

If you are leading multiple training modules, you only need to complete this form once.

Thank you for assisting with the delivery of these training workshops. Please take a moment to answer the following questions. There is no need to write your name on this questionnaire, and all responses will be kept strictly confidential. Thank you!

Trainer Study ID [____|____]

Please complete the boxes below with the relevant numbers

**1. Age in years** [____|____]	**2. Gender** 1 = Male 2 = Female [____]

3. Qualifications [____|____]

01 = Senior medical Officer
02 = Medical Officer
03 = Senior clinical Officer
04 = Clinical Officer
05 = Nursing Officer
06 = Enrolled nurse
07 = Comprehensive nurse
08 = Midwife
09 = Public health nurse
10 = Nursing aide/assistant
11 = Laboratory technician
12 = Laboratory assistant
13 = Health assistant
14 = Health educator
15 = Other ________________________________

4. What training courses have you delivered in the past 3 years?

	**Title of training delivered**	**Organization**	**Dates** [dd/mm/yy] to [dd/mm/yy]
**4a**			[_____/_____/_____] to [_____/_____/_____]
**4b**			[_____/_____/_____] to [_____/_____/_____]
**4c**			[_____/_____/_____] to [_____/_____/_____]

5. What training courses have you attended in training methods?

	**Title of training you attended**	**Organization**	**Dates** [dd/mm/yy]
**5a**			[_____/_____/_____]
**5b**			[_____/_____/_____]
**5c**			[_____/_____/_____]

Please continue overleaf if you have delivered/attended more than 3 training workshops.

APPENDIX G: PATIENT CENTRED SERVICES

SFQ for TRAINERS – INTRODUCTION TO PCS & SOAs (PCS00)

'Thank you for assisting with this Patient Centered Services training! We would appreciate your feedback on the training session you delivered today. Please take a moment to answer the following questions. Your comments will help us to know what information this group received and how well they achieved our intended learning objectives during this session. This will help us to know how to interpret any future change of practice amongst these health workers. There is no need to write your name on this questionnaire, and all responses will be kept strictly confidential. Thank you!'

Trainer Study ID	Date of training	Study ID of other Trainers present
[____|____]	[____|____]/ [____|____]/[____|____]<br>day month year	[____|____], [____|____]
**Training group #**	**Total # of participants invited**	**Total # of participants attended**
[____|____]	[____|____]	[____|____]

Participant Study IDs

[____|____|____|____]	[____|____|____|____]	[____|____|____|____]	[____|____|____|____]
[____|____|____|____]	[____|____|____|____]	[____|____|____|____]	[____|____|____|____]
[____|____|____|____]	[____|____|____|____]	[____|____|____|____]	[____|____|____|____]
[____|____|____|____]	[____|____|____|____]	[____|____|____|____]	[____|____|____|____]

TRAINER QUESTIONNAIRE

**1. Did the training start more than half an hour late?**	1 = Yes<br>2 = No	[____]
**2. If yes, what was the cause of the delay?**	1 = Participants arrived late<br>2 = Materials and supplies to be used were not delivered in time<br>3 = Trainer transport difficulties<br>4 = Other ______________________________	[____]

3. Please describe the consequence of the delay on your ability to deliver the training as intended

Training component	Consequence of delay on trainer's ability to deliver the training as intended
**Introduction to the self-observation activities**	
**New topics introduced in the module**	
**Introduction to the first self-observation activity**	

**4. What level of contribution did the participants have?**	1 = All contributed a lot<br>2 = All contributed at some point<br>3 = Only some contributed<br>4 = None contributed	[____]
**5. For Qn. 4, responses 2 and 3 please list the 3 least active participants.**		
[____|____|____|____]	[____|____|____|____]	[____|____|____|____]

PATIENT CENTRED SERVICES<br>SFQ for TRAINERS – INTRODUCTION TO PCS & SOAs (PCS00)	
**Trainer Study ID**<br>[____|____]	**Date of training**<br>[____|____]/ [____|____]/[____|____]<br>day month year

6. Please complete the table below to describe how this training session went for each topic.

Topic	Teaching as planned?	Materials as planned?	If no, please explain
**Introduction to PCS**	1 = Yes<br>2 = No<br>[____]	1 = Yes<br>2 = No<br>[____]	
**Introduction to SOAs**	1 = Yes<br>2 = No<br>[____]	1 = Yes<br>2 = No<br>[____]	

7. Can you please describe any difficulties/barriers you encountered in delivery of the training?

**<u>Barriers</u> to optimum implementation (e.g. - training interrupted, difficult or over bearing individuals, etc.)**	**<u>Impact</u> of barrier on the intended delivery of training and on learning**

8. Please score how well you think the participants achieved each of the learning objectives today on a scale of 1-10.
(1=Not achieved, 10= Achieved in full. Use 'N/A' if the section is not included in topic)

Learning objectives	Score	Comments
Health workers will be able to identify their own motivations for work		
Health workers will be able to understand the meaning and importance of providing patient centred services		
Health workers will be able to start developing self-awareness through self-observation activities		

PATIENT CENTRED SERVICES
SFQ for TRAINERS – INTRODUCTION TO PCS & SOAs (PCS00)

Trainer Study ID	Date of training
[____|____]	[____|____]/ [____|____]/[____|____]<br>day month year

**9. How many questions or concerns were raised by this group about the topics covered today?**	1 = Many questions<br>2 = Few questions<br>3 = None [____]
**10. What questions or concerns were raised by this group about the topics discussed today? (Please list)**	
**11. How did you address each of these concerns in this group?**	
**12. Which of these concerns do you think were still present at the end of the training?**	
**13. Would you change anything about the session you gave to this group today? If yes, what would you change? How would you change it?**	
**14. What is your overall assessment of how well the intended objectives of today's training were achieved?**	1 = Badly<br>2 = Fine<br>3 = Good [____]

APPENDIX G: PATIENT CENTRED SERVICES
SFQ for TRAINERS – IMPROVING RELATIONSHIPS WITH PATIENTS I (PCS01)

'Thank you for assisting with this Patient Centered Services training! We would appreciate your feedback on the training session you delivered today. Please take a moment to answer the following questions. Your comments will help us to know what information this group received and how well they achieved our intended learning objectives during this session. This will help us to know how to interpret any future change of practice amongst these health workers. There is no need to write your name on this questionnaire, and all responses will be kept strictly confidential. Thank you!'

Trainer Study ID	Date of training	Study ID of other Trainers present
[____|____]	[____|____]/ [____|____]/[____|____]<br>day month year	[____|____], [____|____]
**Training group #**	**Total # of participants invited**	**Total # of participants attended**
[____|____]	[____|____]	[____|____]

Participant Study IDs

[____|____|____|____]	[____|____|____|____]	[____|____|____|____]	[____|____|____|____]
[____|____|____|____]	[____|____|____|____]	[____|____|____|____]	[____|____|____|____]
[____|____|____|____]	[____|____|____|____]	[____|____|____|____]	[____|____|____|____]
[____|____|____|____]	[____|____|____|____]	[____|____|____|____]	[____|____|____|____]

TRAINER QUESTIONNAIRE

Question	Responses	Code
**1. Did the training start more than half an hour late?**	1 = Yes<br>2 = No	[____]
**2. If yes, what was the cause of the delay?**	1 = Participants arrived late<br>2 = Materials and supplies to be used were not delivered in time<br>3 = Trainer transport difficulties<br>4 = Other ______________________________	[____]

3. Please describe the consequence of the delay on your ability to deliver the training as intended

Training component	Consequence of delay on trainer's ability to deliver the training as intended
**Reflection on self-observation activity**	
**New topics introduced in the module**	
**Introduction of the next self-observation activity**	

Question	Responses	Code
**4. What level of contribution did the participants have?**	1 = All contributed a lot<br>2 = All contributed at some point<br>3 = Only some contributed<br>4 = None contributed	[____]

5. For Qn. 4, responses 2 and 3 please list the 3 least active participants.

[____|____|____|____] [____|____|____|____] [____|____|____|____]

PATIENT CENTRED SERVICES
SFQ for TRAINERS – IMPROVING RELATIONSHIPS WITH PATIENTS I (PCS01)

Trainer Study ID	Date of training
[____|____]	[____|____]/ [____|____]/[____|____]<br>day month year

6. Please complete the table below to describe how this training session went for each topic.

Topic	Teaching as planned?	Materials as planned?	If no, please explain
**Building rapport**	1 = Yes<br>2 = No<br>[____]	1 = Yes<br>2 = No<br>[____]	
**Active listening**	1 = Yes<br>2 = No<br>[____]	1 = Yes<br>2 = No<br>[____]	

7. Can you please describe any difficulties/barriers you encountered in delivery of the training?

**Barriers** to optimum implementation (e.g. - training interrupted, difficult or over bearing individuals, etc.)	**Impact** of barrier on the intended delivery of training and on learning

8. Please score how well you think the participants achieved each of the learning objectives today on a scale of 1-10.
(1=Not achieved, 10= Achieved in full. Use 'N/A' if the section is not included in topic)

Learning objectives	Score	Comments
Health workers will be able to apply skills in verbal and verbal communication to build rapport and active listening		
Health workers will be able to identify ways to listen actively in spite of busy work environments		
Health workers will be able to recognise how we think of people affects how we behave towards them		
Health workers will be able to understand that respect is a core value for how we can put patients at ease and strengthen skills to show respect to others		

PATIENT CENTRED SERVICES<br>SFQ for TRAINERS – IMPROVING RELATIONSHIPS WITH PATIENTS I (PCS01)	
**Trainer Study ID**<br>[_____|_____]	**Date of training**<br>[_____|_____]/ [_____|_____]/[_____|_____]<br>day month year

**9. How many questions or concerns were raised by this group about the topics covered today?**	1 = Many questions<br>2 = Few questions<br>3 = None [_____]
**10. What questions or concerns were raised by this group about the topics discussed today? (Please list)**	
**11. How did you address each of these concerns in this group?**	
**12. Which of these concerns do you think were still present at the end of the training?**	
**13. Would you change anything about the session you gave to this group today? If yes, what would you change? How would you change it?**	
**14. What is your overall assessment of how well the intended objectives of today's training were achieved?**	1 = Badly<br>2 = Fine<br>3 = Good [_____]

APPENDIX G: PATIENT CENTRED SERVICES
SFQ for TRAINERS – IMPROVING RELATIONSHIPS WITH PATIENTS II (PCS02)

'Thank you for assisting with this patient centered services training! We would appreciate your feedback on the training session you delivered today. Please take a moment to answer the following questions. Your comments will help us to know what information this group received and how well they achieved our intended learning objectives during this session. This will help us to know how to interpret any future change of practice amongst these health workers. There is no need to write your name on this questionnaire, and all responses will be kept strictly confidential. Thank you!'

Trainer Study ID	Date of training	Study ID of other Trainers present
[____|____]	[____|____]/ [____|____]/[____|____]<br>day month year	[____|____], [____|____]
**Training group #**	**Total # of participants invited**	**Total # of participants attended**
[____|____]	[____|____]	[____|____]

Participant Study IDs

[____|____|____|____]	[____|____|____|____]	[____|____|____|____]	[____|____|____|____]
[____|____|____|____]	[____|____|____|____]	[____|____|____|____]	[____|____|____|____]
[____|____|____|____]	[____|____|____|____]	[____|____|____|____]	[____|____|____|____]
[____|____|____|____]	[____|____|____|____]	[____|____|____|____]	[____|____|____|____]

TRAINER QUESTIONNAIRE

Question	Options	
**1. Did the training start more than half an hour late?**	1 = Yes<br>2 = No	[____]
**2. If yes, what was the cause of the delay?**	1 = Participants arrived late<br>2 = Materials and supplies to be used were not delivered in time<br>3 = Trainer transport difficulties<br>4 = Other ______________________________	[____]

3. Please describe the consequence of the delay on your ability to deliver the training as intended

Training component	Consequence of delay on trainer's ability to deliver the training as intended
**Reflection on self-observation activity**	
**New topics introduced in the module**	
**Introduction of the next self-observation activity**	

Question	Options	
**4. What level of contribution did the participants have?**	1 = All contributed a lot<br>2 = All contributed at some point<br>3 = Only some contributed<br>4 = None contributed	[____]

5. For Qn. 4, responses 2 and 3 please list the 3 least active participants.

[____|____|____|____] [____|____|____|____] [____|____|____|____]

PATIENT CENTRED SERVICES

SFQ for TRAINERS – IMPROVING RELATIONSHIPS WITH PATIENTS II (PCS02)

Trainer Study ID	Date of training
[____|____]	[____|____]/ [____|____]/[____|____]<br>day month year

6. Please complete the table below to describe how this training session went for each topic.

Topic	Teaching as planned?	Materials as planned?	If no, please explain
**Giving information**	1 = Yes<br>2 = No<br>[____]	1 = Yes<br>2 = No<br>[____]	
**RDT negative results**	1 = Yes<br>2 = No<br>[____]	1 = Yes<br>2 = No<br>[____]	

7. Can you please describe any difficulties/barriers you encountered in delivery of the training?

**Barriers** to optimum implementation (e.g. - training interrupted, difficult or over bearing individuals, etc.)	**Impact** of barrier on the intended delivery of training and on learning

8. Please score how well you think the participants achieved each of the learning objectives today on a scale of 1-10.
(1=Not achieved, 10= Achieved in full. Use 'N/A' if the section is not included in topic)

Learning objectives	Score	Comments
Health workers will be able to strengthen verbal and non verbal communication skills in asking good questions		
Health workers will be able to implement strategies to give information effectively about diagnosis and treatment to patients		

PATIENT CENTRED SERVICES
SFQ for TRAINERS – IMPROVING RELATIONSHIPS WITH PATIENTS II (PCS02)

Trainer Study ID	Date of training
[____|____]	[____|____]/ [____|____]/[____|____]<br>day month year

**9. How many questions or concerns were raised by this group about the topics covered today?**	1 = Many questions<br>2 = Few questions<br>3 = None [____]
**10. What questions or concerns were raised by this group about the topics discussed today? (Please list)**	
**11. How did you address each of these concerns in this group?**	
**12. Which of these concerns do you think were still present at the end of the training?**	
**13. Would you change anything about the session you gave to this group today? If yes, what would you change? How would you change it?**	
**14. What is your overall assessment of how well the intended objectives of today's training were achieved?**	1 = Badly<br>2 = Fine<br>3 = Good [____]

APPENDIX G: PATIENT CENTRED SERVICES
SFQ for TRAINERS – IMPROVING RELATIONSHIPS WITH COLLEAGUES (PCS03)

'Thank you for assisting with this Patient Centered Services training! We would appreciate your feedback on the training session you delivered today. Please take a moment to answer the following questions. Your comments will help us to know what information this group received and how well they achieved our intended learning objectives during this session. This will help us to know how to interpret any future change of practice amongst these health workers. There is no need to write your name on this questionnaire, and all responses will be kept strictly confidential. Thank you!'

Trainer Study ID	Date of training	Study ID of other Trainers present
[____|____]	[____|____]/ [____|____]/[____|____]<br>day month year	[____|____], [____|____]
**Training group #**	**Total # of participants invited**	**Total # of participants attended**
[____|____]	[____|____]	[____|____]

Participant Study IDs

[____|____|____|____]	[____|____|____|____]	[____|____|____|____]	[____|____|____|____]
[____|____|____|____]	[____|____|____|____]	[____|____|____|____]	[____|____|____|____]
[____|____|____|____]	[____|____|____|____]	[____|____|____|____]	[____|____|____|____]
[____|____|____|____]	[____|____|____|____]	[____|____|____|____]	[____|____|____|____]

TRAINER QUESTIONNAIRE

Question	Response options	
**1. Did the training start more than half an hour late?**	1 = Yes<br>2 = No	[____]
**2. If yes, what was the cause of the delay?**	1 = Participants arrived late<br>2 = Materials and supplies to be used were not delivered in time<br>3 = Trainer transport difficulties<br>4 = Other ______________________________	[____]

3. Please describe the consequence of the delay on your ability to deliver the training as intended

Training component	Consequence of delay on trainer's ability to deliver the training as intended
**Reflection on self-observation activity**	
**New topics introduced in the module**	
**Introduction of the next self-observation activity**	

Question	Response options	
**4. What level of contribution did the participants have?**	1 = All contributed a lot<br>2 = All contributed at some point<br>3 = Only some contributed<br>4 = None contributed	[____]

5. For Qn. 4, responses 2 and 3 please list the 3 least active participants.

[____|____|____|____]   [____|____|____|____]   [____|____|____|____]

PATIENT CENTRED SERVICES
SFQ for TRAINERS – IMPROVING RELATIONSHIPS WITH COLLEAGUES (PCS03)

Trainer Study ID	Date of training
[____|____]	[____|____]/ [____|____]/[____|____]<br>day month year

6. Please complete the table below to describe how this training session went for each topic.

Topic	Teaching as planned?	Materials as planned?	If no, please explain
**Creating a positive working environment**	1 = Yes<br>2 = No<br>[____]	1 = Yes<br>2 = No<br>[____]	
**Wanting to stay at work all day**	1 = Yes<br>2 = No<br>[____]	1 = Yes<br>2 = No<br>[____]	

7. Can you please describe any difficulties/barriers you encountered in delivery of the training?

**Barriers** to optimum implementation (e.g. - training interrupted, difficult or over bearing individuals, etc.)	**Impact** of barrier on the intended delivery of training and on learning

8. Please score how well you think the participants achieved each of the learning objectives today on a scale of 1-10.
(1=Not achieved, 10= Achieved in full. Use 'N/A' if the section is not included in topic)

Learning objectives	Score	Comments
Health workers will be able to contribute to a positive working environment		
Health workers will be able to motivate and inspire others to work better by identifying ways to bring about positive change		
Health workers will be able to give constructive feedback		
Health workers will be able to communicate effectively with colleagues by identifying how we react to stress impacts how we react to others		

PATIENT CENTRED SERVICES
SFQ for TRAINERS – IMPROVING RELATIONSHIPS WITH COLLEAGUES (PCS03)

Trainer Study ID	Date of training
[_____|_____]	[_____|_____]/ [_____|_____]/[_____|_____]<br>day month year

**9. How many questions or concerns were raised by this group about the topics covered today?**	1 = Many questions<br>2 = Few questions<br>3 = None [_____]
**10. What questions or concerns were raised by this group about the topics discussed today? (Please list)**	
**11. How did you address each of these concerns in this group?**	
**12. Which of these concerns do you think were still present at the end of the training?**	
**13. Would you change anything about the session you gave to this group today? If yes, what would you change? How would you change it?**	
**14. What is your overall assessment of how well the intended objectives of today's training were achieved?**	1 = Badly<br>2 = Fine<br>3 = Good [_____]

APPENDIX G: PATIENT CENTRED SERVICES
SFQ for TRAINERS – IMPROVING THE PATIENT VISIT (PCS04)

'Thank you for assisting with this Patient Centered Services training! We would appreciate your feedback on the training session you delivered today. Please take a moment to answer the following questions. Your comments will help us to know what information this group received and how well they achieved our intended learning objectives during this session. This will help us to know how to interpret any future change of practice amongst these health workers. There is no need to write your name on this questionnaire, and all responses will be kept strictly confidential. Thank you!'

Trainer Study ID	Date of training	Study ID of other Trainers present
[____|____]	[____|____]/ [____|____]/[____|____]<br>day month year	[____|____], [____|____]
**Training group #**	**Total # of participants invited**	**Total # of participants attended**
[____|____]	[____|____]	[____|____]

Participant Study IDs

[____|____|____|____]	[____|____|____|____]	[____|____|____|____]	[____|____|____|____]
[____|____|____|____]	[____|____|____|____]	[____|____|____|____]	[____|____|____|____]
[____|____|____|____]	[____|____|____|____]	[____|____|____|____]	[____|____|____|____]
[____|____|____|____]	[____|____|____|____]	[____|____|____|____]	[____|____|____|____]

TRAINER QUESTIONNAIRE

**1. Did the training start more than half an hour late?**	1 = Yes<br>2 = No	[____]
**2. If yes, what was the cause of the delay?**	1 = Participants arrived late<br>2 = Materials and supplies to be used were not delivered in time<br>3 = Trainer transport difficulties<br>4 = Other ______________________________	[____]

3. Please describe the consequence of the delay on your ability to deliver the training as intended

Training component	Consequence of delay on trainer's ability to deliver the training as intended
**Reflection on self-observation activity**	
**New topics introduced in the module**	
**Introduction of the next self-observation activity**	

**4. What level of contribution did the participants have?**	1 = All contributed a lot<br>2 = All contributed at some point<br>3 = Only some contributed<br>4 = None contributed	[____]
**5. For Qn. 4, responses 2 and 3 please list the 3 least active participants.**<br>[____|____|____|____] [____|____|____|____] [____|____|____|____]		

PATIENT CENTRED SERVICES
SFQ for TRAINERS – IMPROVING THE PATIENT VISIT (PCS04)

Trainer Study ID	Date of training
[____|____]	[____|____]/ [____|____]/[____|____]<br>day month year

6. Please complete the table below to describe how this training session went for each topic.

Topic	Teaching as planned?	Materials as planned?	If no, please explain
**Patient welcome & orientation**	1 = Yes<br>2 = No<br>[_____]	1 = Yes<br>2 = No<br>[_____]	
	1 = Yes<br>2 = No<br>[_____]	1 = Yes<br>2 = No<br>[_____]	

7. Can you please describe any difficulties/barriers you encountered in delivery of the training?

**<u>Barriers</u> to optimum implementation (e.g. - training interrupted, difficult or over bearing individuals, etc.)**	**<u>Impact</u> of barrier on the intended delivery of training and on learning**

8. Please score how well you think the participants achieved each of the learning objectives today on a scale of 1-10.
(1=Not achieved, 10= Achieved in full. Use 'N/A' if the section is not included in topic)

Learning objectives	Score	Comments
Health workers will be able to implement strategies to improve the welcome of patients at the health facility to patients		
Health workers will be able to implement strategies to ensure patients are seen fairly		
Health workers will be able to implement strategies to improve the orientation of patients at the health center		
Health workers will be able to appropriately utilize volunteers to address current staffing gaps		
Health workers will be able to contribute to improving patient satisfaction by working together with all clinical and support staff		

PATIENT CENTRED SERVICES<br>SFQ for TRAINERS – IMPROVING THE PATIENT VISIT (PCS04)	
**Trainer Study ID**<br>[____|____]	**Date of training**<br>[____|____]/ [____|____]/[____|____]<br>day month year

**9. How many questions or concerns were raised by this group about the topics covered today?**	1 = Many questions<br>2 = Few questions<br>3 = None	[____]
**10. What questions or concerns were raised by this group about the topics discussed today? (Please list)**		
**11. How did you address each of these concerns in this group?**		
**12. Which of these concerns do you think were still present at the end of the training?**		
**13. Would you change anything about the session you gave to this group today? If yes, what would you change? How would you change it?**		
**14. What is your overall assessment of how well the intended objectives of today's training were achieved?**	1 = Badly<br>2 = Fine<br>3 = Good	[____]

APPENDIX G: PATIENT CENTRED SERVICES

SFQ for TRAINERS – VOLUNTEERS AND COMPREHENSIVE CARE (PCS05)

'Thank you for assisting with this patient centered services training! We would appreciate your feedback on the training session you delivered today. Please take a moment to answer the following questions. Your comments will help us to know what information this group received and how well they achieved our intended learning objectives during this session. This will help us to know how to interpret any future change of practice amongst these health workers. There is no need to write your name on this questionnaire, and all responses will be kept strictly confidential. Thank you!'

Trainer Study ID	Date of training	Study ID of other Trainers present
[____|____]	[____|____]/ [____|____]/[____|____]<br>day month year	[____|____], [____|____]
**Training group #**	**Total # of participants invited**	**Total # of participants attended**
[____|____]	[____|____]	[____|____]

Participant Study IDs

[____|____|____|____]	[____|____|____|____]	[____|____|____|____]	[____|____|____|____]
[____|____|____|____]	[____|____|____|____]	[____|____|____|____]	[____|____|____|____]
[____|____|____|____]	[____|____|____|____]	[____|____|____|____]	[____|____|____|____]
[____|____|____|____]	[____|____|____|____]	[____|____|____|____]	[____|____|____|____]

TRAINER QUESTIONNAIRE

**1. Did the training start more than half an hour late?**	1 = Yes<br>2 = No	[____]
**2. If yes, what was the cause of the delay?**	1 = Participants arrived late<br>2 = Materials and supplies to be used were not delivered in time<br>3 = Trainer transport difficulties<br>4 = Other ______________________________	[____]

3. Please describe the consequence of the delay on your ability to deliver the training as intended

Training component	Consequence of delay on trainer's ability to deliver the training as intended
**New topics introduced in the module**	
**Role play activities**	
**Planning activities**	

**4. What level of contribution did the participants have?**	1 = All contributed a lot<br>2 = All contributed at some point<br>3 = Only some contributed<br>4 = None contributed	[____]

5. For Qn. 4, responses 2 and 3 please list the 3 least active participants.

[____|____|____|____] [____|____|____|____] [____|____|____|____]

PATIENT CENTRED SERVICES<br>SFQ for TRAINERS – VOLUNTEERS AND COMPREHENSIVE CARE (PCS05)	
**Trainer Study ID**<br>[____|____]	**Date of training**<br>[____|____]/ [____|____]/[____|____]<br>day month year

6. Please complete the table below to describe how this training session went for each topic.

Topic	Teaching as planned?	Materials as planned?	If no, please explain
**Welcoming & greeting patients**	1 = Yes<br>2 = No<br>[____]	1 = Yes<br>2 = No<br>[____]	
**Patient navigation**	1 = Yes<br>2 = No<br>[____]	1 = Yes<br>2 = No<br>[____]	

7. Can you please describe any difficulties/barriers you encountered in delivery of the training?

**Barriers to optimum implementation (e.g. - training interrupted, difficult or over bearing individuals, etc.)**	**Impact of barrier on the intended delivery of training and on learning**

8. Please score how well you think the participants achieved each of the learning objectives today on a scale of 1-10.
(1=Not achieved, 10= Achieved in full. Use 'N/A' if the section is not included in topic)

Learning objectives	Score	Comments
Understand the importance of providing 'patient centred services'		
Recognise that we all have different perspectives, including volunteers and patients		
Implement strategies to improve the welcome of patients at health centres by establishing rapport		
Put themselves into the shoes of a patient approaching a health centre as an organisation with unspoken 'rules'		
Implement strategies to improve the orientation of patients at health centres		
Implement strategies to ensure patients can navigate the health centre		

PATIENT CENTRED SERVICES<br>SFQ for TRAINERS – VOLUNTEERS AND COMPREHENSIVE CARE (PCS05)	
**Trainer Study ID**<br>[____|____]	**Date of training**<br>[____|____]/ [____|____]/[____|____]<br>day month year

**9. How many questions or concerns were raised by this group about the topics covered today?**	1 = Many questions<br>2 = Few questions<br>3 = None [____]
**10. What questions or concerns were raised by this group about the topics discussed today? (Please list)**	
**11. How did you address each of these concerns in this group?**	
**12. Which of these concerns do you think were still present at the end of the training?**	
**13. Would you change anything about the session you gave to this group today? If yes, what would you change? How would you change it?**	
**14. What is your overall assessment of how well the intended objectives of today's training were achieved?**	1 = Badly<br>2 = Fine<br>3 = Good [____]

APPENDIX H: PATIENT CENTRED SERVICES SFQs

PART 1: PARTICIPANT DEMOGRAPHIC BACKGROUND FORM

If you are attending multiple training modules, you only need to complete this form once.

Thank you for participating in this training course. We would like to collect some information about you and your work history. Please take a moment to answer the following questions. There is no need to write your name on this questionnaire. All responses will be kept strictly confidential. Thank you!

Please complete the boxes below with your own information. Please ask if you have any questions.

Participant PRIME Study ID [____|____]

Please complete the questions below

1. What is your age in years? [____|____] years

2. What is your gender? (please circle) Male Female

3. How long have you worked at this health centre? [____|____] and [____|____]
years months

4. If you are an in-charge, how long have you actively worked as an in-charge? [____|____] and [____|____]
years months

5. What is your education? Please circle all levels completed

Primary
Senior four
Senior six
Vocational certificate
University
Others *(please specify)*
____
____

6. What year did you completed your highest level of education (schooling)? [____|____|____|____]
year

7. What is your current position? Please select from the list below and write the appropriate number here: [____|____]

01 = Senior medical Officer	06 = Enrolled nurse	11 = Laboratory technician
02 = Medical Officer	07 = Comprehensive nurse	12 = Laboratory assistant
03 = Senior clinical Officer	08 = Midwife	13 = Health assistant
04 = Clinical Officer	09 = Public health nurse	14 = Health educator
05 = Nursing Officer	10 = Nursing aide/assistant	15 = Other

____

8. What year did you start working in this position? [____|____|____|____]
year

9. What training workshops have you attended in the past 3 years? Please complete the table below

	Title of training you attended	Organization	Date [dd/mm/yy]
9a			[_____/_____/_____]
9b			[_____/_____/_____]
9c			[_____/_____/_____]

APPENDIX H: PATIENT CENTRED SERVICES

SFQ for PARTICIPANTS – INTRODUCTION TO PCS & SOAs (PCS00)

Thank you for participating in this health centre management training! We would appreciate your feedback on the training session you have attended. Please take a moment to answer the following questions. This is **NOT A TEST** and **WE VALUE YOUR OPINION**. Your opinions will help us improve future trainings. There is no need to write your name on this questionnaire. All responses will be kept strictly confidential. Thank you!

GENERAL INSTRUCTIONS TO COMPLETE THIS QUESTIONNAIRE

1. *Please use a dark coloured pen to fill out the questionnaire*

2. *The health worker identity (ID) number is the unique number that was given to you at the start of this PRIME project. When we request you to give your ID number, please fill the boxes as below:*

   [_0_ | _5_ | _1_ | _0_]

   You are asked to enter your ID number like this at the top of each page of your questionnaire.

3. *In this questionnaire, we ask you to read each question carefully and either write your response, or look at the statements and decide which response is closest to your own opinion. When you have decided, please circle the number in the column under that response. For example:*

Decide how much you agree with the statements below	***Strongly agree***	***Agree***	***Disagree***	***Strongly disagree***
I learned new ideas today	*1* (circled)	*2*	*3*	*4*

4. *If you change your mind and would like to circle a different response, please cross out your original choice and circle the choice that fits your opinion best. For example, if you change your mind and you decide that your answer is 'disagree', cross through the original and circle the new response as below:*

Decide how much you agree with the statements below	***Strongly agree***	***Agree***	***Disagree***	***Strongly disagree***
I learned new ideas today	~~1~~ (crossed out)	*2*	*3* (circled)	*4*

5. *When you have completed the questionnaire, please make sure you have completed everything in the following checklist:*

For the first time you attend a workshop <u>only</u>:	☐ *[For the first time only] I have signed and dated the consent form*<br>☐ *[For the first time only] I have completed my demographic details form*
For every workshop:	☐ *I have answered all of the questions in this questionnaire*<br>☐ *I have checked that I have circled the responses that are closest to my opinion*<br>☐ *I have written my health worker ID number on all of the pages of the questionnaire*<br>☐ *I have written my health worker ID number on the envelope and will place this completed form inside the envelope*

PATIENT CENTRED SERVICES
SFQ for PARTICIPANTS – INTRODUCTION TO PCS & SOAs (PCS00)

Health worker ID	Today's date
[____|____|____|____]	[____|____]/ [____|____]/[____|____]<br>day month year

1. **Please complete the table below about your <u>learning today</u> by circling the response closest to your opinion.**

Decide how much you agree with the statements below	Strongly agree	Agree	Disagree	Strongly disagree
I learned new ideas today	1	2	3	4
I understood everything the trainer taught today	1	2	3	4
I felt the training was relevant to my daily work	1	2	3	4
The ideas I learned today will be useful for me in the future	1	2	3	4
I was given a chance to ask all of my questions	1	2	3	4
I felt that the trainer answered all our questions well	1	2	3	4
I felt able to practice new skills in the workshop today	1	2	3	4
I was given a chance to give my opinions	1	2	3	4
I will recommend my colleagues to attend this training	1	2	3	4
**If you have any comments on the above statements, Please write them below:**				

PATIENT CENTRED SERVICES<br>SFQ for PARTICIPANTS – INTRODUCTION TO PCS & SOAs (PCS00)	
**Health worker ID**<br>[____|____|____|____]	**Today's date**<br>[____|____]/ [____|____]/[____|____]<br>day month year

2. **Please complete the table below about the skills you have learnt from <u>the training topic</u>, by circling the response closest to your opinion.**

Decide how much you agree with the statements below	Strongly agree	Agree	Disagree	Strongly disagree
This training helped me to think about	1	2	3	4
This training has helped me to see	1	2	3	4
This training has given me ideas for how to	1	2	3	4
After this training, I feel able to change	1	2	3	4
After this training, I have found ways to	1	2	3	4
**Please write any comments on the above statements in this section here:**				

3. **Please complete the table below about today's <u>overall training</u> by circling the response closest to your opinion.**

	Very good	Good	Fair	Poor	Very poor
What is your opinion of the trainer's skills today?	1	2	3	4	5
What is your opinion of the trainer's attitude today?	1	2	3	4	5
What is your opinion of the learner's manual you received today?	1	2	3	4	5
What is your opinion of the flip charts that were used today?	1	2	3	4	5
Overall, what is your opinion of the training today?	1	2	3	4	5

PATIENT CENTRED SERVICES<br>SFQ for PARTICIPANTS – INTRODUCTION TO PCS & SOAs (PCS00)	
**Health worker ID**<br>[____|____|____|____]	**Today's date**<br>[____|____]/ [____|____]/[____|____]<br>day month year

4. Please write anything else you would like to say about today's training

NOW PLEASE ENSURE YOU HAVE COMPLETED THIS FORM FULLY BY GOING THROUGH THE CHECK SHEET ON THE FRONT PAGE

APPENDIX H: PATIENT CENTRED SERVICES
SFQ for PARTICIPANTS – IMPROVING RELATIONSHIPS WITH PATIENTS I (PCS01)

Thank you for participating in this health centre management training! We would appreciate your feedback on the training session you have attended. Please take a moment to answer the following questions. This is **NOT A TEST** and **WE VALUE YOUR OPINION**. Your opinions will help us improve future trainings. There is no need to write your name on this questionnaire.
All responses will be kept strictly confidential. Thank you!

GENERAL INSTRUCTIONS TO COMPLETE THIS QUESTIONNAIRE

6. *Please use a dark coloured pen to fill out the questionnaire*

7. *The health worker identity (ID) number is the unique number that was given to you at the start of this PRIME project. When we request you to give your ID number, please fill the boxes as below:*
   [_0_ | _5_ | _1_ | _0_]

   You are asked to enter your ID number like this at the top of each page of your questionnaire.

8. *In this questionnaire, we ask you to read each question carefully and either write your response, or look at the statements and decide which response is closest to your own opinion. When you have decided, please circle the number in the column under that response. For example:*

Decide how much you agree with the statements below	***Strongly agree***	***Agree***	***Disagree***	***Strongly disagree***
I learned new ideas today	*(1)*	*2*	*3*	*4*

9. *If you change your mind and would like to circle a different response, please cross out your original choice and circle the choice that fits your opinion best. For example, if you change your mind and you decide that your answer is 'disagree', cross through the original and circle the new response as below:*

Decide how much you agree with the statements below	***Strongly agree***	***Agree***	***Disagree***	***Strongly disagree***
I learned new ideas today	*~~(1)~~*	*2*	*(3)*	*4*

10. *When you have completed the questionnaire, please make sure you have completed everything in the following checklist:*

For the first time you attend a workshop <u>only</u>:	☐ *[For the first time only] I have signed and dated the consent form*<br>☐ *[For the first time only] I have completed my demographic details form*
For every workshop:	☐ *I have answered all of the questions in this questionnaire*<br>☐ *I have checked that I have circled the responses that are closest to my opinion*<br>☐ *I have written my health worker ID number on all of the pages of the questionnaire*<br>☐ *I have written my health worker ID number on the envelope and will place this completed form inside the envelope*

PATIENT CENTRED SERVICES<br>SFQ for PARTICIPANTS – IMPROVING RELATIONSHIPS WITH PATIENTS I (PCS01)	
**Health worker ID**	**Today's date**
[____|____|____|____]	[____|____]/ [____|____]/[____|____]<br>day month year

1. **Please complete the table below about your <u>learning today</u> by circling the response closest to your opinion.**

Decide how much you agree with the statements below	Strongly agree	Agree	Disagree	Strongly disagree
I learned new ideas today	1	2	3	4
I understood everything the trainer taught today	1	2	3	4
I felt the training was relevant to my daily work	1	2	3	4
The ideas I learned today will be useful for me in the future	1	2	3	4
I was given a chance to ask all of my questions	1	2	3	4
I felt that the trainer answered all our questions well	1	2	3	4
I felt able to practice new skills in the workshop today	1	2	3	4
I was given a chance to give my opinions	1	2	3	4
I will recommend my colleagues to attend this training	1	2	3	4
**If you have any comments on the above statements, Please write them below:**				

PATIENT CENTRED SERVICES<br>SFQ for PARTICIPANTS – IMPROVING RELATIONSHIPS WITH PATIENTS I (PCS01)	
**Health worker ID**<br>[____|____|____|____]	**Today's date**<br>[____|____]/ [____|____]/[____|____]<br>day month year

2. Please complete the table below about the skills you have learnt from <u>the training topic</u>, by circling the response closest to your opinion.

Decide how much you agree with the statements below	Strongly agree	Agree	Disagree	Strongly disagree
This training helped me to think about	1	2	3	4
This training has helped me to see	1	2	3	4
This training has given me ideas for how to	1	2	3	4
After this training, I feel able to change	1	2	3	4
After this training, I have found ways to	1	2	3	4
**Please write any comments on the above statements in this section here:**				

3. Please complete the table below about today's <u>overall training</u> by circling the response closest to your opinion.

	Very good	Good	Fair	Poor	Very poor
What is your opinion of the trainer's skills today?	1	2	3	4	5
What is your opinion of the trainer's attitude today?	1	2	3	4	5
What is your opinion of the learner's manual you received today?	1	2	3	4	5
What is your opinion of the flip charts that were used today?	1	2	3	4	5
Overall, what is your opinion of the training today?	1	2	3	4	5

4. Please write anything else you would like to say about today's training

NOW PLEASE ENSURE YOU HAVE COMPLETED THIS FORM FULLY
BY GOING THROUGH THE CHECK SHEET ON THE FRONT PAGE

APPENDIX H: PATIENT CENTRED SERVICES

SFQ for PARTICIPANTS – IMPROVING RELATIONSHIPS WITH PATIENTS II (PCS02)

Thank you for participating in this health centre management training! We would appreciate your feedback on the training session you have attended. Please take a moment to answer the following questions. This is **NOT A TEST** and **WE VALUE YOUR OPINION**. Your opinions will help us improve future trainings. There is no need to write your name on this questionnaire.
All responses will be kept strictly confidential. Thank you!

GENERAL INSTRUCTIONS TO COMPLETE THIS QUESTIONNAIRE

11. Please use a dark coloured pen to fill out the questionnaire

12. The health worker identity (ID) number is the unique number that was given to you at the start of this PRIME project. When we request you to give your ID number, please fill the boxes as below:

[_0_ | _5_ | _1_ | _0_]

You are asked to enter your ID number like this at the top of each page of your questionnaire.

13. In this questionnaire, we ask you to read each question carefully and either write your response, or look at the statements and decide which response is closest to your own opinion. When you have decided, please circle the number in the column under that response. For example:

Decide how much you agree with the statements below	*Strongly agree*	*Agree*	*Disagree*	*Strongly disagree*
I learned new ideas today	*(1)*	*2*	*3*	*4*

14. If you change your mind and would like to circle a different response, please cross out your original choice and circle the choice that fits your opinion best. For example, if you change your mind and you decide that your answer is 'disagree', cross through the original and circle the new response as below:

Decide how much you agree with the statements below	*Strongly agree*	*Agree*	*Disagree*	*Strongly disagree*
I learned new ideas today	*~~(1)~~*	*2*	*(3)*	*4*

15. When you have completed the questionnaire, please make sure you have completed everything in the following checklist:

For the first time you attend a workshop only:	☐ *[For the first time only] I have signed and dated the consent form*<br>☐ *[For the first time only] I have completed my demographic details form*
For every workshop:	☐ *I have answered all of the questions in this questionnaire*<br>☐ *I have checked that I have circled the responses that are closest to my opinion*<br>☐ *I have written my health worker ID number on all of the pages of the questionnaire*<br>☐ *I have written my health worker ID number on the envelope and will place this completed form inside the envelope*

PATIENT CENTRED SERVICES<br>SFQ for PARTICIPANTS – IMPROVING RELATIONSHIPS WITH PATIENTS II (PCS02)	
**Health worker ID**<br>[____|____|____|____]	**Today's date**<br>[____|____]/ [____|____]/[____|____]<br>day month year

1. **Please complete the table below about your <u>learning today</u> by circling the response closest to your opinion.**

Decide how much you agree with the statements below	Strongly agree	Agree	Disagree	Strongly disagree
I learned new ideas today	1	2	3	4
I understood everything the trainer taught today	1	2	3	4
I felt the training was relevant to my daily work	1	2	3	4
The ideas I learned today will be useful for me in the future	1	2	3	4
I was given a chance to ask all of my questions	1	2	3	4
I felt that the trainer answered all our questions well	1	2	3	4
I felt able to practice new skills in the workshop today	1	2	3	4
I was given a chance to give my opinions	1	2	3	4
I will recommend my colleagues to attend this training	1	2	3	4
**If you have any comments on the above statements, Please write them below:**				

**PATIENT CENTRED SERVICES**<br>**SFQ for PARTICIPANTS – IMPROVING RELATIONSHIPS WITH PATIENTS II (PCS02)**	
**Health worker ID**<br>[____|____|____|____]	**Today's date**<br>[____|____]/ [____|____]/[____|____]<br>day month year

2. **Please complete the table below about the skills you have learnt from <u>the training topic</u>, by circling the response closest to your opinion.**

**Decide how much you agree with the statements below**	**Strongly agree**	**Agree**	**Disagree**	**Strongly disagree**
This training helped me to think about	1	2	3	4
This training has helped me to see	1	2	3	4
This training has given me ideas for how to	1	2	3	4
After this training, I feel able to change	1	2	3	4
After this training, I have found ways to	1	2	3	4
**Please write any comments on the above statements in this section here:**				

3. **Please complete the table below about today's <u>overall training</u> by circling the response closest to your opinion.**

	**Very good**	**Good**	**Fair**	**Poor**	**Very poor**
What is your opinion of the trainer's skills today?	1	2	3	4	5
What is your opinion of the trainer's attitude today?	1	2	3	4	5
What is your opinion of the learner's manual you received today?	1	2	3	4	5
What is your opinion of the flip charts that were used today?	1	2	3	4	5
Overall, what is your opinion of the training today?	1	2	3	4	5

PATIENT CENTRED SERVICES

SFQ for PARTICIPANTS – IMPROVING RELATIONSHIPS WITH PATIENTS II (PCS02)

Health worker ID	Today's date
[____|____|____|____]	[____|____]/ [____|____]/[____|____]<br>day month year

4. Please write anything else you would like to say about today's training

NOW PLEASE ENSURE YOU HAVE COMPLETED THIS FORM FULLY BY GOING THROUGH THE <u>CHECK SHEET</u> ON THE FRONT PAGE

APPENDIX H: PATIENT CENTRED SERVICES

SFQ for PARTICIPANTS – IMPROVING RELATIONSHIPS WITH COLLEAGUES (PCS03)

Thank you for participating in this health centre management training! We would appreciate your feedback on the training session you have attended. Please take a moment to answer the following questions. This is **NOT A TEST** and **WE VALUE YOUR OPINION**. Your opinions will help us improve future trainings. There is no need to write your name on this questionnaire.
All responses will be kept strictly confidential. Thank you!

GENERAL INSTRUCTIONS TO COMPLETE THIS QUESTIONNAIRE

16. *Please use a dark coloured pen to fill out the questionnaire*

17. *The health worker identity (ID) number is the unique number that was given to you at the start of this PRIME project. When we request you to give your ID number, please fill the boxes as below:*

    [_0_ | _5_ | _1_ | _0_]

    You are asked to enter your ID number like this at the top of each page of your questionnaire.

18. *In this questionnaire, we ask you to read each question carefully and either write your response, or look at the statements and decide which response is closest to your own opinion. When you have decided, please circle the number in the column under that response. For example:*

Decide how much you agree with the statements below	***Strongly agree***	***Agree***	***Disagree***	***Strongly disagree***
I learned new ideas today	*1* (circled)	*2*	*3*	*4*

19. *If you change your mind and would like to circle a different response, please cross out your original choice and circle the choice that fits your opinion best. For example, if you change your mind and you decide that your answer is 'disagree', cross through the original and circle the new response as below:*

Decide how much you agree with the statements below	***Strongly agree***	***Agree***	***Disagree***	***Strongly disagree***
I learned new ideas today	*1* (circled, crossed out)	*2*	*3* (circled)	*4*

20. *When you have completed the questionnaire, please make sure you have completed everything in the following checklist:*

For the first time you attend a workshop <u>only</u>:	☐ *[For the first time only] I have signed and dated the consent form*<br>☐ *[For the first time only] I have completed my demographic details form*
For every workshop:	☐ *I have answered all of the questions in this questionnaire*<br>☐ *I have checked that I have circled the responses that are closest to my opinion*<br>☐ *I have written my health worker ID number on all of the pages of the questionnaire*<br>☐ *I have written my health worker ID number on the envelope and will place this completed form inside the envelope*

PATIENT CENTRED SERVICES
SFQ for PARTICIPANTS – IMPROVING RELATIONSHIPS WITH COLLEAGUES (PCS03)

Health worker ID	Today's date
[____|____|____|____]	[____|____]/ [____|____]/[____|____]<br>day month year

4. **Please complete the table below about your <u>learning today</u> by circling the response closest to your opinion.**

Decide how much you agree with the statements below	Strongly agree	Agree	Disagree	Strongly disagree
I learned new ideas today	1	2	3	4
I understood everything the trainer taught today	1	2	3	4
I felt the training was relevant to my daily work	1	2	3	4
The ideas I learned today will be useful for me in the future	1	2	3	4
I was given a chance to ask all of my questions	1	2	3	4
I felt that the trainer answered all our questions well	1	2	3	4
I felt able to practice new skills in the workshop today	1	2	3	4
I was given a chance to give my opinions	1	2	3	4
I will recommend my colleagues to attend this training	1	2	3	4
**If you have any comments on the above statements, Please write them below:**				

PATIENT CENTRED SERVICES<br>SFQ for PARTICIPANTS – IMPROVING RELATIONSHIPS WITH COLLEAGUES (PCS03)	
**Health worker ID**	**Today's date**
[____|____|____|____]	[____|____]/ [____|____]/[____|____]<br>day month year

5. Please complete the table below about the skills you have learnt from <u>the training topic</u>, by circling the response closest to your opinion.

Decide how much you agree with the statements below	Strongly agree	Agree	Disagree	Strongly disagree
This training helped me to think about	1	2	3	4
This training has helped me to see	1	2	3	4
This training has given me ideas for how to	1	2	3	4
After this training, I feel able to change	1	2	3	4
After this training, I have found ways to	1	2	3	4
**Please write any comments on the above statements in this section here:**				

6. Please complete the table below about today's <u>overall training</u> by circling the response closest to your opinion.

	Very good	Good	Fair	Poor	Very poor
What is your opinion of the trainer's skills today?	1	2	3	4	5
What is your opinion of the trainer's attitude today?	1	2	3	4	5
What is your opinion of the learner's manual you received today?	1	2	3	4	5
What is your opinion of the flip charts that were used today?	1	2	3	4	5
Overall, what is your opinion of the training today?	1	2	3	4	5

PATIENT CENTRED SERVICES<br>SFQ for PARTICIPANTS – IMPROVING RELATIONSHIPS WITH COLLEAGUES (PCS03)	
**Health worker ID**	**Today's date**
[____|____|____|____]	[____|____]/ [____|____]/[____|____]<br>day month year

4. Please write anything else you would like to say about today's training

NOW PLEASE ENSURE YOU HAVE COMPLETED THIS FORM FULLY BY GOING THROUGH THE <u>CHECK SHEET</u> ON THE FRONT PAGE

APPENDIX H: PATIENT CENTRED SERVICES
SFQ for PARTICIPANTS – IMPROVING THE PATIENT VISIT (PCS04)

Thank you for participating in this health centre management training! We would appreciate your feedback on the training session you have attended. Please take a moment to answer the following questions. This is **NOT A TEST** and **WE VALUE YOUR OPINION**. Your opinions will help us improve future trainings. There is no need to write your name on this questionnaire.
All responses will be kept strictly confidential. Thank you!

GENERAL INSTRUCTIONS TO COMPLETE THIS QUESTIONNAIRE

21. Please use a dark coloured pen to fill out the questionnaire

22. The health worker identity (ID) number is the unique number that was given to you at the start of this PRIME project. When we request you to give your ID number, please fill the boxes as below:

[_0_ | _5_ | _1_ | _0_]

You are asked to enter your ID number like this at the top of each page of your questionnaire.

23. In this questionnaire, we ask you to read each question carefully and either write your response, or look at the statements and decide which response is closest to your own opinion. When you have decided, please circle the number in the column under that response. For example:

Decide how much you agree with the statements below	*Strongly agree*	*Agree*	*Disagree*	*Strongly disagree*
I learned new ideas today	*1* (circled)	*2*	*3*	*4*

24. If you change your mind and would like to circle a different response, please cross out your original choice and circle the choice that fits your opinion best. For example, if you change your mind and you decide that your answer is 'disagree', cross through the original and circle the new response as below:

Decide how much you agree with the statements below	*Strongly agree*	*Agree*	*Disagree*	*Strongly disagree*
I learned new ideas today	*1* (circled and crossed out)	*2*	*3* (circled)	*4*

25. When you have completed the questionnaire, please make sure you have completed everything in the following checklist:

For the first time you attend a workshop <u>only</u>:	☐ *[For the first time only] I have signed and dated the consent form*<br>☐ *[For the first time only] I have completed my demographic details form*
For every workshop:	☐ *I have answered all of the questions in this questionnaire*<br>☐ *I have checked that I have circled the responses that are closest to my opinion*<br>☐ *I have written my health worker ID number on all of the pages of the questionnaire*<br>☐ *I have written my health worker ID number on the envelope and will place this completed form inside the envelope*

PATIENT CENTRED SERVICES<br>SFQ for PARTICIPANTS – IMPROVING THE PATIENT VISIT (PCS04)	
**Health worker ID**<br>[____|____|____|____]	**Today's date**<br>[____|____]/ [____|____]/[____|____]<br>day month year

4. **Please complete the table below about your <u>learning today</u> by circling the response closest to your opinion.**

Decide how much you agree with the statements below	Strongly agree	Agree	Disagree	Strongly disagree
I learned new ideas today	1	2	3	4
I understood everything the trainer taught today	1	2	3	4
I felt the training was relevant to my daily work	1	2	3	4
The ideas I learned today will be useful for me in the future	1	2	3	4
I was given a chance to ask all of my questions	1	2	3	4
I felt that the trainer answered all our questions well	1	2	3	4
I felt able to practice new skills in the workshop today	1	2	3	4
I was given a chance to give my opinions	1	2	3	4
I will recommend my colleagues to attend this training	1	2	3	4
**If you have any comments on the above statements, Please write them below:**				

PATIENT CENTRED SERVICES

SFQ for PARTICIPANTS – IMPROVING THE PATIENT VISIT (PCS04)

Health worker ID	Today's date
[____|____|____|____]	[____|____]/ [____|____]/[____|____]<br>day month year

5. **Please complete the table below about the skills you have learnt from <u>the training topic</u>, by circling the response closest to your opinion.**

Decide how much you agree with the statements below	Strongly agree	Agree	Disagree	Strongly disagree
This training helped me to think about	1	2	3	4
This training has helped me to see	1	2	3	4
This training has given me ideas for how to	1	2	3	4
After this training, I feel able to change	1	2	3	4
After this training, I have found ways to	1	2	3	4

Please write any comments on the above statements in this section here:

6. **Please complete the table below about today's <u>overall training</u> by circling the response closest to your opinion.**

	Very good	Good	Fair	Poor	Very poor
What is your opinion of the trainer's skills today?	1	2	3	4	5
What is your opinion of the trainer's attitude today?	1	2	3	4	5
What is your opinion of the learner's manual you received today?	1	2	3	4	5
What is your opinion of the flip charts that were used today?	1	2	3	4	5
Overall, what is your opinion of the training today?	1	2	3	4	5

PATIENT CENTRED SERVICES<br>SFQ for PARTICIPANTS – IMPROVING THE PATIENT VISIT (PCS04)	
**Health worker ID**<br>[____|____|____|____]	**Today's date**<br>[____|____]/ [____|____]/[____|____]<br>day month year

4. Please write anything else you would like to say about today's training

NOW PLEASE ENSURE YOU HAVE COMPLETED THIS FORM FULLY BY GOING THROUGH THE <u>CHECK SHEET</u> ON THE FRONT PAGE

APPENDIX H: PATIENT CENTRED SERVICES

SFQ for PARTICIPANTS – VOLUNTEERS –IMPROVING THE PATIENT VISIT(PCS05)

Thank you for participating in this health centre management training! We would appreciate your feedback on the training session you have attended. Please take a moment to answer the following questions. This is **NOT A TEST** and **WE VALUE YOUR OPINION**. Your opinions will help us improve future trainings. There is no need to write your name on this questionnaire.
All responses will be kept strictly confidential. Thank you!

GENERAL INSTRUCTIONS TO COMPLETE THIS QUESTIONNAIRE

26. Please use a dark coloured pen to fill out the questionnaire

27. The health worker identity (ID) number is the unique number that was given to you at the start of this PRIME project. When we request you to give your ID number, please fill the boxes as below:

[_0_ | _5_ | _1_ | _0_]

You are asked to enter your ID number like this at the top of each page of your questionnaire.

28. In this questionnaire, we ask you to read each question carefully and either write your response, or look at the statements and decide which response is closest to your own opinion. When you have decided, please circle the number in the column under that response. For example:

Decide how much you agree with the statements below	***Strongly agree***	***Agree***	***Disagree***	***Strongly disagree***
I learned new ideas today	*1* (circled)	*2*	*3*	*4*

29. If you change your mind and would like to circle a different response, please cross out your original choice and circle the choice that fits your opinion best. For example, if you change your mind and you decide that your answer is 'disagree', cross through the original and circle the new response as below:

Decide how much you agree with the statements below	***Strongly agree***	***Agree***	***Disagree***	***Strongly disagree***
I learned new ideas today	*1* (circled and crossed out)	*2*	*3* (circled)	*4*

30. When you have completed the questionnaire, please make sure you have completed everything in the following checklist:

For the first time you attend a workshop only:	☐ *[For the first time only] I have signed and dated the consent form*<br>☐ *[For the first time only] I have completed my demographic details form*
For every workshop:	☐ *I have answered all of the questions in this questionnaire*<br>☐ *I have checked that I have circled the responses that are closest to my opinion*<br>☐ *I have written my health worker ID number on all of the pages of the questionnaire*<br>☐ *I have written my health worker ID number on the envelope and will place this completed form inside the envelope*

PATIENT CENTRED SERVICES

SFQ for PARTICIPANTS – VOLUNTEERS –IMPROVING THE PATIENT VISIT(PCS05)

Health worker ID	Today's date
[____|____|____|____]	[____|____]/ [____|____]/[____|____]<br>day month year

4. Please complete the table below about your <u>learning today</u> by circling the response closest to your opinion.

Decide how much you agree with the statements below	Strongly agree	Agree	Disagree	Strongly disagree
I learned new ideas today	1	2	3	4
I understood everything the trainer taught today	1	2	3	4
I felt the training was relevant to my daily work	1	2	3	4
The ideas I learned today will be useful for me in the future	1	2	3	4
I was given a chance to ask all of my questions	1	2	3	4
I felt that the trainer answered all our questions well	1	2	3	4
I felt able to practice new skills in the workshop today	1	2	3	4
I was given a chance to give my opinions	1	2	3	4
I will recommend my colleagues to attend this training	1	2	3	4
**If you have any comments on the above statements, Please write them below:**				

PATIENT CENTRED SERVICES
SFQ for PARTICIPANTS – VOLUNTEERS –IMPROVING THE PATIENT VISIT(PCS05)

Health worker ID	Today's date
[____|____|____|____]	[____|____]/ [____|____]/[____|____]<br>day month year

5. **Please complete the table below about the skills you have learnt from <u>the training topic</u>, by circling the response closest to your opinion.**

Decide how much you agree with the statements below	Strongly agree	Agree	Disagree	Strongly disagree
This training helped me to think about	1	2	3	4
This training has helped me to see	1	2	3	4
This training has given me ideas for how to	1	2	3	4
After this training, I feel able to change	1	2	3	4
After this training, I have found ways to	1	2	3	4

Please write any comments on the above statements in this section here:

6. **Please complete the table below about today's <u>overall training</u> by circling the response closest to your opinion.**

	Very good	Good	Fair	Poor	Very poor
What is your opinion of the trainer's skills today?	1	2	3	4	5
What is your opinion of the trainer's attitude today?	1	2	3	4	5
What is your opinion of the learner's manual you received today?	1	2	3	4	5
What is your opinion of the flip charts that were used today?	1	2	3	4	5
Overall, what is your opinion of the training today?	1	2	3	4	5

PATIENT CENTRED SERVICES<br>SFQ for PARTICIPANTS – VOLUNTEERS –IMPROVING THE PATIENT VISIT(PCS05)	
**Health worker ID**	**Today's date**
[____|____|____|____]	[____|____]/ [____|____]/[____|____]<br>day month year

4. Please write anything else you would like to say about today's training

NOW PLEASE ENSURE YOU HAVE COMPLETED THIS FORM FULLY BY GOING THROUGH THE <u>CHECK SHEET</u> ON THE FRONT PAGE

Appendix I: Measure of Patient-Centered Communication (MPCC) Coding Form

Coding form for component I. Exploring both the disease and the illness experience

Date:
Start Time:________________ Coder Initials:________
Stop Time:________________ Tape Code:________

COMPONENT I. EXPLORING BOTH THE DISEASE AND THE ILLNESS EXPERIENCE

Symptoms and/or Reason for Visit		Preliminary Exploration	Further Exploration	Validation	Cut-off	SCORE
1	______	Y N	Y N	Y N	Y N	______
2	______	Y N	Y N	Y N	Y N	______
3	______	Y N	Y N	Y N	Y N	______
4	______	Y N	Y N	Y N	Y N	______
5	______	Y N	Y N	Y N	Y N	______
					ST**	☐
Prompts						
1	______	Y N	Y N	Y N	Y N	______
2	______	Y N	Y N	Y N	Y N	______
3	______	Y N	Y N	Y N	Y N	______
4	______	Y N	Y N	Y N	Y N	______
5	______	Y N	Y N	Y N	Y N	______
					ST**	☐
Feelings						
1	______	Y N	Y N	Y N	Y N	______
2	______	Y N	Y N	Y N	Y N	______
3	______	Y N	Y N	Y N	Y N	______
4	______	Y N	Y N	Y N	Y N	______
5	______	Y N	Y N	Y N	Y N	______
					ST**	☐
Ideas						
1	______	Y N	Y N	Y N	Y N	______
2	______	Y N	Y N	Y N	Y N	______
3	______	Y N	Y N	Y N	Y N	______
4	______	Y N	Y N	Y N	Y N	______
5	______	Y N	Y N	Y N	Y N	______
					ST**	☐
Effect on Function						
1	______	Y N	Y N	Y N	Y N	______
2	______	Y N	Y N	Y N	Y N	______
3	______	Y N	Y N	Y N	Y N	______
4	______	Y N	Y N	Y N	Y N	______
5	______	Y N	Y N	Y N	Y N	______
					ST**	☐
Expectations						
1	______	Y N	Y N	Y N	Y N	______
2	______	Y N	Y N	Y N	Y N	______
3	______	Y N	Y N	Y N	Y N	______
4	______	Y N	Y N	Y N	Y N	______
5	______	Y N	Y N	Y N	Y N	______
					ST**	☐

GT*** ________ ÷ ________ = ☐

** Sub-total
**** Grand Total

Notes for completion of component 1

Score	Process Category
0	no preliminary exploration
0	preliminary exploration with cutoff
1	preliminary exploration and further exploration with cut off
2	preliminary exploration and validation with cut-off (no further exploration)
2	preliminary exploration without cut-off (no further exploration, no validation)
3	preliminary exploration and further exploration without cutoff (no validation)
3	preliminary exploration and further exploration and validation with cut-off
4	preliminary exploration and validation without cut off (no further exploration)
5	preliminary exploration and further exploration and validation without cut-off

Steps

1) Assign a score (X) for each statement listed.
2) For each heading (i.e. Symptoms and/or Reason for Visit, Prompts, Feelings, Ideas, Effect on Function, Expectations), add Xs and divide by the number of statements to calculate a subtotal (ST).
3) Add all STs.
4) Determine the appropriate denominator. The denominator is the number of applicable headings (maximum 6) multiplied by the score range (5). The denominator will be 30 (6 headings x 5) except where there are no “Symptoms and/or Reason for Visit” and/or “Prompts”. If there are neither “Symptoms and/or Reason for Visit” nor “Prompts”, the denominator will be 20 (4 headings x 5). If there is only one of “Symptoms and/or Reason for Visit” or “Prompts”, the denominator will be 25 (5 headings x 5).
5) Divide the sum of all STs by the appropriate denominator to calculate the grand total.

Table for scoring (KEY: Y = yes; N = no)

Preliminary Exploration	Further Exploration	Validation	Cut-off	Score (0-5)
N	N	N	Y	0
Y	N	N	Y	0
Y	Y	N	Y	1
Y	N	Y	Y	2
Y	N	N	N	2
Y	Y	N	N	3
Y	Y	Y	Y	3
Y	N	Y	N	4
Y	Y	Y	N	5

Coding form for component II. Understanding the whole person

Any statements relevant to FAMILY, LIFE CYCLE, SOCIAL SUPPORT, PERSONALITY, and CONTEXT are to be listed below:

		Preliminary Exploration	Further Exploration	Validation	Cut-off	SCORE
1	______	Y N	Y N	Y N	Y N	______
2	______	Y N	Y N	Y N	Y N	______
3	______	Y N	Y N	Y N	Y N	______
4	______	Y N	Y N	Y N	Y N	______
5	______	Y N	Y N	Y N	Y N	______
6	______	Y N	Y N	Y N	Y N	______
7	______	Y N	Y N	Y N	Y N	______
8	______	Y N	Y N	Y N	Y N	______
9	______	Y N	Y N	Y N	Y N	______
10	______	Y N	Y N	Y N	Y N	______

ST* ☐

GT** ______ ÷ 5 = ☐

* Sub-total
** Grand Total

Notes on completing coding for component II

Score	Process Category Meaning
0	no preliminary exploration
0	preliminary exploration with cutoff
1	preliminary exploration and further exploration with cut off
2	preliminary exploration and validation with cut-off (no further exploration)
2	preliminary exploration without cut-off (no further exploration, no validation)
3	preliminary exploration and further exploration without cutoff (no validation)
3	preliminary exploration and further exploration and validation with cut-off
4	preliminary exploration and validation without cut off (no further exploration)
5	preliminary exploration and further exploration and validation without cut-off

Steps

1) Assign a score (X) for each statement listed.
2) Add Xs and divide by the number of statements to calculate a subtotal (ST).
3) Divide the ST by 5 (the maximum possible score) to calculate the grand total (GT)

Table for scoring (KEY: Y = yes; N = no)

Preliminary Exploration	Further Exploration	Validation	Cut-off	Score (0-5)
N	N	N	Y	0
Y	N	N	Y	0
Y	Y	N	Y	1
Y	N	Y	Y	2
Y	N	N	N	2
Y	Y	N	N	3
Y	Y	Y	Y	3
Y	N	Y	N	4
Y	Y	Y	N	5

Coding form for component III. Finding common ground

Problem Definition:

		Clearly Expressed	Opportunity to Ask Questions	Mutual Discussion	Clarification of Agreement	SCORE
1	______	Y N	Y N	Y N	Y N	______
2	______	Y N	Y N	Y N	Y N	______
3	______	Y N	Y N	Y N	Y N	______
4	______	Y N	Y N	Y N	Y N	______
5	______	Y N	Y N	Y N	Y N	______
6	______	Y N	Y N	Y N	Y N	______
7	______	Y N	Y N	Y N	Y N	______
8	______	Y N	Y N	Y N	Y N	______
9	______	Y N	Y N	Y N	Y N	______
10	______	Y N	Y N	Y N	Y N	______
					ST**	☐

Goals of Treatment/Management

1	______	Y N	Y N	Y N	Y N	______
2	______	Y N	Y N	Y N	Y N	______
3	______	Y N	Y N	Y N	Y N	______
4	______	Y N	Y N	Y N	Y N	______
5	______	Y N	Y N	Y N	Y N	______
6	______	Y N	Y N	Y N	Y N	______
7	______	Y N	Y N	Y N	Y N	______
8	______	Y N	Y N	Y N	Y N	______
9	______	Y N	Y N	Y N	Y N	______
10	______	Y N	Y N	Y N	Y N	______
					ST**	☐

Responded Appropriately to Disagreement with Flexibility and Understanding

1	______	Y N	N/A	______
2	______	Y N	N/A	______
			ST**	☐

** Sub-total
*** Grand Total

GT*** ______ ÷ ______ = ☐

Notes for completing coding for component III

Scoring Guideline

For each statement under Problem Definition and Goals of Treatment and Management, each occurrence of Y (yes) is given a score of 1 for a maximum of 4 for each statement. Each occurrence of N (no) gets a score of 0. For Responded Appropriately to Disagreement with Flexibility and Understanding, each occurrence of Y (yes) is given a score of 4 and each occurrence of N (no) is given a score of 0.

Steps

1) Assign a score (X) for each statement listed using the scoring guideline above.
2) For each of the three headings (i.e. Problem Definition, Goals of Treatment and Management, Responded Appropriately to Disagreement with Flexibility and Understanding), add Xs and divide by the number of statements to calculate a subtotal (STs).
3) Determine the appropriate denominator. The highest possible score when using all three headings (Problem Definition, Goals of Treatment and Management, and Responded Appropriately to Disagreement with Flexibility and Understanding) is 12. If there are no statements for Responded Appropriately to Disagreement with Flexibility and Understanding, add the two other subtotals (Problem Definition and Goals of Treatment and Management) and divide by 8. If there are no Problem Definitions and no Responded Appropriately to Disagreement with Flexibility and Understanding, the denominator is 4.
4) Add the STs and divide by the appropriate denominator to obtain the Grand Total (GT) for Component III.
5) Multiply by 100 to obtain a percentage score.

THE REPUBLIC OF UGANDA

[____]—[____|____|____|____]

HCWA Study ID

APPENDIX J. HEALTH WORKER COMMUNICATION ASSESSMENTS
Informed consent form for health workers

**Protocol Title:**	ACT PROCESS: Evaluating the process, context, and impact of interventions to enhance health facilities in Tororo, Uganda
**Site of Research:**	Tororo, Uganda
**Principal Investigators**:	Dr. Sarah Staedke
**Date:**	23 February 2011

Introduction

Dr. Sarah Staedke and colleagues from the Uganda Malaria Surveillance Project/Infectious Diseases Research Collaboration are doing a study to evaluate activities that have been introduced at government-run health facilities in this area aiming to improve the health of children. We would like to understand the impact of these activities and why they are working, or not. This study involves several parts, including assessing the communication between health workers and their patients, holding interviews, and conducting group discussions with community members and health workers.

Why is this study being done?

For this part of the study, we would like to learn more about how well patients and health workers communicate. To do this, we would like to record interactions between health workers and caregivers of ill children at 20 health centers in this area. We are interested in recording your interactions with caregivers of typical malaria patients: children under five who have fever and no danger signs of severe disease. We would like to learn what usually happens in interactions between health workers, caregivers, and patients.

What will happen today if I take part in this study?

If you agree to take part, we will ask you to keep a digital voice recorder in the room where you see patients to record your interactions with patients and their caregivers. We will inform patients and their caregivers about this device and will also ask them if they agree to be recorded. We will let you know if they agree and will help you switch on the recording device. We would like you to conduct your consultation with the patient as you would normally; you are not expected to do anything differently to your usual practice while we are recording. We would like to record your interaction with at least three patients, and up to five patients. After we have recorded these interactions with you, we will not ask you to do anything further. The information we collect will be used by project investigators, and may be shared with other researchers and policy-makers to answer questions about how best to deliver training and health services.

<u>How long will the study last?</u>

We would like to record your interactions with patients on three occasions: today, within the next 2-3 months, and again in about 9 months. Each time, we would like to record your interaction with at least three, and up to five patients, which should take approximately 1-2 days to complete.

<u>Can I stop being in the study?</u>

You can decide to stop participating at any time. Just tell the project researcher right away if you wish to stop.

<u>What risks can I expect from being in the study?</u>

Participation in any research study may involve a loss of privacy. Information exchanged during the interactions with patients will be recorded, but your name will not be used in any reports of the information provided. The names of your patients, caregivers and colleagues will also not be used. No quotes or other results arising from your participation in this study will be included in any reports, even anonymously, without your agreement. The information obtained from these interviews will be locked at our project offices. We will do our best to make sure that the personal information gathered for this study is kept private.

<u>Are there benefits to taking part in the study?</u>

There will be no direct benefit to you from participating in this study. However, the information that you provide will help researchers and policy-makers understand how best to improve health services in this area.

<u>What other choices do I have if I do not take part in this study?</u>

You are free to choose not to participate in the study. If you decide not to take part in this study, there will be no penalty to you.

<u>What are the costs of taking part in this study? Will I be paid for taking part in this study?</u>

There are no costs to you for taking part in this study. You will not be paid for taking part in this study.

<u>What are my rights if I take part in this study?</u>

Taking part in this study is your choice. You may choose either to take part or not to take part in the study. If you decide to take part in this study, you may change your mind at any time. No matter what decision you take, there will be no penalty to you in any way.

<u>Who can answer my questions about the study?</u>

You can talk to the researchers about any questions or concerns you have about these study activities. Contact Dr. Sarah Staedke or other members of the Uganda Malaria Surveillance Project / Infectious Disease Research Collaboration on telephone number 0414-530692. If you have any questions, comments or

concerns about taking part in these activities, first talk to the researchers. If for any reason you do not wish to do this, or you still have concerns about doing so, you may contact Dr Charles Ibingira, Makerere University Faculty of Medicine Research and Ethical Committee at telephone number 0414-530020.

WHAT YOUR SIGNATURE OR THUMBPRINT MEANS

Your signature or thumbprint below means that you understand the information given to you in this consent form about your participation in the study and agree with the following statements:

1. "I have read the consent form concerning this study (or have understood the verbal explanation of the consent form) and I understand what will be required of me and what will happen to me if I take part in it."
2. "My questions concerning this study have been answered by Dr. Staedke or the person who signed below."
3. "I understand that at any time, I may withdraw from this study without giving a reason."
4. "I agree to take part in this study."

If you wish to participate in this study, you should sign or place your thumbprint on the following page. If you do not agree to quotes or other results arising from your participation in the study being included, even anonymously, in any reports about the study, please tell the researcher now.

THE REPUBLIC OF UGANDA

WE WILL GIVE YOU A COPY OF THIS SIGNED AND DATED CONSENT FORM

____
Name of Participant (printed)

____
Signature or Fingerprint of Participant　　　　Date/Time

____
Name of Investigator Administering Consent (printed)　　　　Position/Title

____
Signature of Investigator Administering Consent　　　　Date/Time

If the participant is unable to read and/or write, an impartial witness should be present during the entire informed consent discussion.

After the written informed consent form is read and explained to the participant, and after they have orally consented to participate in the study, the witness should print the name of the participant above, and then the participant should provide their fingerprint.

Then the witness should print their name, provide their signature, and date the consent form below.

By signing the consent form, the witness attests that the information in the consent form and any other written information was accurately explained to, and apparently understood by the participant, and that informed consent was freely given by the participant.

____
Name of Person Witnessing Consent (printed)

____
Signature of Person Witnessing Consent　　　　Date/Time

APPENDIX K. HEALTH WORKER COMMUNICATION ASSESSMENT

PART 1: HEALTH WORKER DETAILS

Health center code	HW Study ID	Interviewer code	Date
[_____|_____]	[_____|_____]	[_____|_____]	[____|____] / [____|____] / [____|____]<br>day month year

Health worker position: [____|____]

1 = In-charge	7=Nursing officer	12 = Laboratory technician
2 = Senior medical officer	8= Enrolled nurse	13 = Laboratory assistant
3 = Medical officer	9= Midwife	14= Health assistant
4 = Senior clinical officer	10= Public health nurse	15 = Health educator
5 = Clinical officer	11 = Nursing aide/assistant	16 = Other____
6 = Comprehensive nurse		

PART 2: DEMOGRAPHIC INFORMATION

1. Health worker age [____|____] years

2. Health worker gender 1 = Male 2 = Female [____]

3. Originally from this area? 1 = Yes 2 = No [____]

4. Number of years worked in this job [____|____] years

5. Highest level of education or qualification achieved [____|____]

0 = None	4 = Diploma	77 = Other
1 = Primary (P1 — P7)	5 = Bachelor's degree	
2 = Secondary (S1 — S6)	88 = Don't know	____
3 = Certificate	99 = Refused to answer	

6. Year graduated [____|____|____|____]
year

APPENDIX L: HEALTH WORKER COMMUNICATION ASSESSMENT
CAREGIVER & PATIENT SCREENING FORM

Health center code	Interviewer code	Date
[_____|_____]	[_____|_____]	[____|____] / [____|____] / [____|____]<br>day month year

Screening Date	Screening ID
[_____|_____]/[_____|_____]/[_____|_____]<br>day month year	[_____|_____|_____|_____]

Age		Gender	
[____|____] / [____|____]<br>years months	*If child is less than 1 year, complete months, otherwise leave blank*	[____]	1 = Male<br>2 = Female

PART 2: SCREENING INTERVIEW with PARENTS/GUARDIANS

Selection criteria	Include	Exclude	
1. Appropriate age<br>—Under five (aged 0 to less than 5 years)	1 = Yes	2 = No	[_____]
2. Fever or suspected fever	1 = Yes	2 = No	[_____]
3. Danger signs of severe disease? (Convulsions, severe weakness/lethargy, unable to feed, unable to stand/sit unsupported, vomiting everything, severe dehydration)	1 = No	2 = Yes	[_____]

If any answers are '2' from the EXCLUDE column, exclude from the study. If not, proceed to the next section.

PART 3: INFORMED CONSENT

Selection criteria	Include	Exclude	
4. Willingness of caregiver to provide informed consent	1 = Yes	2 = No	[_____]

If any answers are '2' from the EXCLUDE column, exclude from the study. If not, proceed to the next section.

ASSIGN STUDY NUMBER	[_____|_____|_____|_____]

All criteria for study inclusion met?	Date of enrollment
1 = Yes<br>2 = No *If no, exclude from the study*<br>[_____]	[______|______]/[______|______]/[______|______]<br>day month year

Staff ID: [____|____] **Data entrant (1st):** [____|____] **Data entrant (2nd):** [____|____]

THE REPUBLIC OF UGANDA

[____]—[____|____|____|____]

HCWA Study ID

APPENDIX M. HEALTH WORKER COMMUNICATION ASSESSMENTS and PATIENT EXIT INTERVIEWS
Informed consent form for caregivers

**Protocol Title:**	ACT PROCESS: Evaluating the process, context, and impact of interventions to enhance health facilities in Tororo, Uganda
**Site of Research:**	Tororo, Uganda
**Principal Investigators**:	Dr. Sarah Staedke
**Date:**	23 February 2011

Introduction

Dr. Sarah Staedke and colleagues from the Uganda Malaria Surveillance Project/Infectious Diseases Research Collaboration are doing a study to evaluate activities that have been introduced at government-run health facilities in this area aiming to improve the health of children. We would like to understand the impact of these activities and why they are working, or not. This study involves several parts, including assessing the communication between health workers and their patients, holding interviews, and conducting group discussions with community members and health workers.

Why is this study being done?

For this part of the study, we would like to learn more about how well patients and health workers communicate. To do this, we would like to record interactions between health workers and caregivers of ill children at 20 health centers in this area. We are interested in recording interactions between health workers and caregivers of typical malaria patients: children under five who have fever and no danger signs of severe disease. We would like to learn what usually happens in interactions between health workers, caregivers, and patients.

What will happen today if I take part in this study?

If you agree to take part, we will record your interaction with the health worker using a digital voice recorder. We have already informed the health worker about this device and they have agreed to be recorded. We would like you to interact with the health worker as you would normally; you are not expected to do anything differently while we are recording. After the consultation with the health worker is over, we would also like to ask you some questions about your visit to the health center today. We would like to ask questions about the purpose of your visit, and whether you were satisfied with your visit or not. After we have recorded your interaction with the health worker, and asked you the questions about your visit today,

we will not ask you to do anything further. The information we collect will be used by project investigators, and may be shared with other researchers and policy-makers to answer questions about how best to deliver training and health services.

How long will the study last?

If you take part in the study, it will involve a one-time recording of your interaction with the health worker today. This should take about 30 minutes. If you agree to stay after the consultation for the extra interview, it will last about 15 minutes.

Can I stop being in the study?

You can decide to stop participating at any time. Just tell the project researcher right away if you wish to stop.

What risks can I expect from being in the study?

Participation in any research study may involve a loss of privacy. Information exchanged during your interactions with the health worker will be recorded, but your name will not be used in any reports of the information provided. No quotes or other results arising from your participation in this study will be included in any reports, even anonymously, without your agreement. The information obtained from these interviews will be locked at our project offices. We will do our best to make sure that the personal information gathered for this study is kept private.

Are there benefits to taking part in the study?

There will be no direct benefit to you from participating in this study. However, the information that you provide will help researchers and policy-makers understand how best to improve health services in this area.

What other choices do I have if I do not take part in this study?

You are free to choose not to participate in the study. If you decide not to take part in this study, there will be no penalty to you.

What are the costs of taking part in this study? Will I be paid for taking part in this study?

There are no costs to you for taking part in this study. You will not be paid for taking part in this study.

What are my rights if I take part in this study?

Taking part in this study is your choice. You may choose either to take part or not to take part in the study. If you decide to take part in this study, you may change your mind at any time. No matter what decision you take, there will be no penalty to you in any way.

THE REPUBLIC OF UGANDA

Who can answer my questions about the study?

You can talk to the researchers about any questions or concerns you have about these study activities. Contact Dr. Sarah Staedke or other members of the Uganda Malaria Surveillance Project / Infectious Disease Research Collaboration on telephone number 0414-530692. If you have any questions, comments or concerns about taking part in these activities, first talk to the researchers. If for any reason you do not wish to do this, or you still have concerns about doing so, you may contact Dr Charles Ibingira, Makerere University Faculty of Medicine Research and Ethical Committee at telephone number 0414-530020.

WHAT YOUR SIGNATURE OR THUMBPRINT MEANS

Your signature or thumbprint below means that you understand the information given to you in this consent form about your participation in the study and agree with the following statements:

1. "I have read the consent form concerning this study (or have understood the verbal explanation of the consent form) and I understand what will be required of me and what will happen to me if I take part in it."

2. "My questions concerning this study have been answered by Dr. Staedke or the person who signed below."

3. "I understand that at any time, I may withdraw from this study without giving a reason."

4. "I agree to take part in this study."

If you wish to participate in this study, you should sign or place your thumbprint on the following page. If you do not agree to quotes or other results arising from your participation in the study being included, even anonymously, in any reports about the study, please tell the researcher now.

Mark each box with **X** if you agree:

☐ **I agree to let the researchers record my interaction with the health worker**

☐ **I agree to take part in the interview after my consultation with the health worker is over**

THE REPUBLIC OF UGANDA

WE WILL GIVE YOU A COPY OF THIS SIGNED AND DATED CONSENT FORM

Name of Participant (printed)

Signature or Fingerprint of Participant   Date/Time

Name of Investigator Administering Consent (printed)   Position/Title

Signature of Investigator Administering Consent   Date/Time

If the participant is unable to read and/or write, an impartial witness should be present during the entire informed consent discussion.

After the written informed consent form is read and explained to the participant, and after they have orally consented to participate in the study, the witness should print the name of the participant above, and then the participant should provide their fingerprint.

Then the witness should print their name, provide their signature, and date the consent form below.

By signing the consent form, the witness attests that the information in the consent form and any other written information was accurately explained to, and apparently understood by the participant, and that informed consent was freely given by the participant.

Name of Person Witnessing Consent (printed)

Signature of Person Witnessing Consent   Date/Time

APPENDIX N. HEALTH WORKER COMMUNICATION ASSESSMENT

PART 1: CAREGIVER DETAILS

Health center code	Patient ID code	Interviewer code	Date
[_____|_____]	[_____|_____]	[_____|_____]	[____|____] / [____|____] / [____|____]<br>day month year

**1. Number of patients brought by the caregiver today** [_____|_____]	**2. Number of eligible children being seen today** [_____|_____]

PART 2: CAREGIVER DEMOGRAPHIC INFORMATION

**1. Caregiver age**	[____|____] years	**2. Caregiver gender**	1 = Male<br>2 = Female [____]
**3. Been to this health centre before?**			1 = Yes<br>2 = No [____]
**3. Originally from this area?**			1 = Yes<br>2 = No [____]

5. Highest level of education or qualification achieved [____|____]

0 = None<br>1 = Primary (P1 — P7)<br>2 = Secondary (S1 — S6)<br>3 = Certificate	4 = Diploma<br>5 = Bachelor's degree<br>88 = Don't know<br>99 = Refused to answer	77 = Other<br>________________________________

5. Employment [____|____]

0 = Not employed<br>1 = Teacher<br>2 = Nurse<br>3 = Member of village health team<br>1 = Peasant farmer	2 = Commercial farmer<br>3 = Brew alcohol<br>4 = Market vendor<br>5 = Shop keeper<br>6 = Transport(Driver/rider)	77 = Other<br>________________________________<br>88 = Don't know<br>99 = Refused to answer

PART 3: PATIENT DEMOGRAPHIC INFORMATION

**1. Age of child enrolled**	[____|____] years	**2. Gender**	1 = Male<br>2 = Female [____]

3. What problems does the child have? (list all mentioned by the caregiver)

1 = None<br>2 = Cough<br>3 = Flu<br>4 = Not eating<br>5 = Vomiting<br>6 = Weak (not playing)<br>7 = Convulsions<br>8 = Fever	9 = Diarrhoea<br>10 = Skin infection<br>77 =Other<br>____________________________<br>____________________________<br>88 = I don't know<br>99 = Refused to answer	[____|____] [____|____]<br>[____|____] [____|____]<br>[____|____] [____|____]

APPENDIX O. HEALTH WORKER COMMUNICATION ASSESSMENT PATIENT EXIT INTERVIEW

Health Center ID	HW ID	Patient ID	Interviewer ID	Date
[____|____]	[____|____]	[____|____]	[____|____]	[____|____] / [____|____] / [____|____]<br>day month year

**1. Number of patients brought by the caregiver today**	[_____|_____]	**2. Number of eligible children being seen today**	[_____|_____]

**1. What was the reason you came here today?** *(List, in mother's words, below)*		
**2. Did you feel you were able to discuss this problem fully with the health worker?**	1 = Yes<br>2 = No	[_____|_____]
**3. Do you think that the health worker understood how important this problem is to you and your child?**	1 = Yes<br>2 = No	[_____|_____]
**4. Did you feel that the health worker was listening to you with full attention?**	1 = Yes<br>2 = No	[_____|_____]
**5. Did the health worker give you enough information about why he thinks the child is experiencing the problem(s)?**	1 = Yes<br>2 = No	[_____|_____]
**6. Do you agree with the health worker's opinion about the problem?**	1 = Yes<br>2 = No	[_____|_____]
**7. Do you feel the health worker could have done more to investigate the problem of your child today?**	1 = Yes<br>2 = No	[_____|_____]
**8. How satisfied were you with the treatment you were given?** *(Allocate category that fits best with response, check with mother)*	1 = Very satisfied<br>2 = Satisfied<br>3 = Somewhat satisfied<br>4 = Not satisfied	[_____|_____]
**Did the health worker explain what this medicine will do?**	1 = Yes<br>2 = No	[_____|_____]
**Did the health worker help you to understand how the child should take this medicine?**	1 = Yes<br>2 = No	[_____|_____]
**Did the health worker help you to understand what to expect during the child's illness?**	1 = Yes<br>2 = No	[_____|_____]
**How confident do you feel that your child will get better if you follow the health worker's advice?**<br>*(Allocate category that fits best with response, check with mother)*	1 = Very confident<br>2 = Somewhat confident<br>3 = Not confident	[_____|_____]
**Overall, how did the health worker make you feel?**<br>*(Allocate category that fits best with response, check with mother)*	1= Very happy<br>2 = Somewhat happy<br>3 = Unhappy	[_____|_____]
**Overall, how welcome did you feel at this health centre from start to finish?**	1 = Very welcome<br>2 = Welcome<br>3 = Somewhat welcome<br>4 = Unwelcome	[_____|_____]
**Next time your child is sick, will you come back to here?**	1 = Yes<br>2 = No	[_____|_____]
**Overall, how satisfied were you with the consultation today?**	1 = Very satisfied<br>2 = Satisfied<br>3 = Somewhat satisfied<br>4 = Not satisfied	[_____|_____]
**Do you have any additional comments about this consultation with the health worker?**		

THE REPUBLIC OF UGANDA

[____]—[____|____|____|____]

IDI Study ID

APPENDIX P. IN-DEPTH INTERVIEWS

Informed consent form for implementers, health workers, and stakeholders

**Protocol Title:**	ACT PROCESS: Evaluating the process, context, and impact of interventions to enhance health facilities in Tororo, Uganda
**Site of Research:**	Tororo, Uganda
**Principal Investigators**:	Dr. Sarah Staedke
**Date:**	23 February 2011

Introduction

Dr. Sarah Staedke and colleagues from the Uganda Malaria Surveillance Project/Infectious Diseases Research Collaboration are doing a study to evaluate activities that have been introduced at government-run health facilities in this area aiming to improve the health of children. We would like to understand the impact of these activities and why they are working, or not. This study involves several parts, including assessing the communication between health workers and their patients, holding interviews, and conducting group discussions with community members and health workers.

Why is this study being done?

For this part of the study, we would like to learn more about whether the activities that have been introduced at government-run health facilities have had an impact from the perspective of those involved in introducing the activities, health workers, and local and district stakeholders. This information will help us understand how and why the health facility activities have affected the health of children in this area.

What will happen today if I take part in this study?

Today, we would like to ask you some questions about the activities that have been introduced at the health facilities, provision of care for sick children in this area, and any changes that you have noticed recently. We will take notes of the discussion and a recording will also be made using a digital voice recorder. After we ask these questions today, we will not ask you to do anything further. The information we collect will be used by project investigators, and may be shared with other researchers and policy-makers to answer questions about how best to deliver training and health services.

How long will the study last?

Today, the interview will last about 60-90 minutes.

Can I stop being in the study?

You can decide to stop participating at any time. Just tell the project researcher right away if you wish to stop.

What risks can I expect from being in the study?

Participation in any research study may involve a loss of privacy. Your name will not be used in any reports of the information provided. No quotes or other results arising from your participation in this study will be included in any reports, even anonymously, without your agreement. The information obtained from these interviews will be locked at our project offices. We will do our best to make sure that the personal information gathered for this study is kept private.

Are there benefits to taking part in the study?

There will be no direct benefit to you from participating in this study. However, the information that you provide will help researchers and policy-makers understand how best to improve health services in this area.

What other choices do I have if I do not take part in this study?

You are free to choose not to participate in the study. If you decide not to take part in this study, there will be no penalty to you.

What are the costs of taking part in this study? Will I be paid for taking part in this study?

There are no costs to you for taking part in this study. You will not be paid for taking part in this study.

What are my rights if I take part in this study?

Taking part in this study is your choice. You may choose either to take part or not to take part in the study. If you decide to take part in this study, you may change your mind at any time. No matter what decision you take, there will be no penalty to you in any way.

Who can answer my questions about the study?

You can talk to the researchers about any questions or concerns you have about these study activities. Contact Dr. Sarah Staedke or other members of the Uganda Malaria Surveillance Project / Infectious Disease Research Collaboration on telephone number 0414-530692. If you have any questions, comments or concerns about taking part in these activities, first talk to the researchers. If for any reason you do not wish to do this, or you still have concerns about doing so, you may contact Dr Charles Ibingira, Makerere University Faculty of Medicine Research and Ethical Committee at telephone number 0414-530020.

WHAT YOUR SIGNATURE OR THUMBPRINT MEANS

Your signature or thumbprint below means that you understand the information given to you in this consent form about your participation in the study and agree with the following statements:

1. "I have read the consent form concerning this study (or have understood the verbal explanation of the consent form) and I understand what will be required of me and what will happen to me if I take part in it."

2. "My questions concerning this study have been answered by Dr. Staedke or the person who signed below."

3. "I understand that at any time, I may withdraw from this study without giving a reason."

4. "I agree to take part in this study."

If you wish to participate in this study, you should sign or place your thumbprint on the following page. If you do not agree to quotes or other results arising from your participation in the study being included, even anonymously, in any reports about the study, please tell the researcher now.

THE REPUBLIC OF UGANDA

WE WILL GIVE YOU A COPY OF THIS SIGNED AND DATED CONSENT FORM

Name of Participant (printed)

Signature or Fingerprint of Participant | Date/Time

Name of Investigator Administering Consent (printed) | Position/Title

Signature of Investigator Administering Consent | Date/Time

If the participant is unable to read and/or write, an impartial witness should be present during the entire informed consent discussion.

After the written informed consent form is read and explained to the participant, and after they have orally consented to participate in the study, the witness should print the name of the participant above, and then the participant should provide their fingerprint.

Then the witness should print their name, provide their signature, and date the consent form below.

By signing the consent form, the witness attests that the information in the consent form and any other written information was accurately explained to, and apparently understood by the participant, and that informed consent was freely given by the participant.

Name of Person Witnessing Consent (printed)

Signature of Person Witnessing Consent | Date/Time

APPENDIX Q: IDI DATA COLLECTION TOOL
HFI IMPLEMENTERS

Study ID	Date
[____|____]	[____|____] / [____|____] / [____|____]<br>day month year

Position:	
1 = Study coordinator<br>2 = Medical Officer<br>3 = Trainer<br>4 = Community Health worker<br>5 = Clinical officer<br>6 = Laboratory technician	7 = Laboratory Assistant<br>8 =Home Visitor<br>9 = Implementation support (administration, logistics, procurement, research assistant)<br>10 = Other_______________<br>[____|____]

DEMOGRAPHIC INFORMATION

**1. Age** Years [____|____]	**5. Highest level of education or qualification achieved**<br>0 = None<br>1 = Primary (P1 — P7)<br>2 = Secondary (S1 — S6)<br>3 = Certificate<br>4 = Diploma<br>5 = Bachelor's degree<br>88 = Don't know<br>99 = Refused to answer<br>77 = Other____________________________<br>[____|____]
**2. Gender** 1 = Male 2 = Female [____]	
**3. Originally from this area?** 1 = Yes 2 = No [____]	
**4. Number of years worked in this job** [____|____]	**6. Year graduated** [____|____|____|____]

PART 1: INTRODUCTION

Conduct the interview according to the directions below and record information as indicated.

Introduction to in-depth interview

"Hello, my name is ………. I am interested in interviewing you. I would like you to express your own views and experiences about your perspectives on the implementation of the ACT PRIME health facility intervention to improve the health of children in Tororo by improving services at government-run health facilities. A note-taker will be writing down what you say for our records, and we will record the interview using a digital recorder; these notes will be kept securely and your name will not be used anywhere. Your answers will be looked at together with those of many other implementers and you will not be identifiable in any reports that are published.

It is very important for us to hear your views and experiences because you have experience implementing the intervention and can give us this insight. We hope you will have time to spend with us now to complete this interview. The interview will take about 45 minutes; but if you prefer we can reschedule the interview for tomorrow or another day of your convenience.

Do you have any questions? Do you agree to continue before we start?

Now we request that we all switch off our mobile phones so that we are not distracted."

PART 2: IMPLEMENTER IN-DEPTH INTERVIEW (2)	
**Domains, topic questions, and probes:** Use the table below to help you administer the questions during the interview.	
**Domain**	**Topic and Probes**
**1. Role of the implementer**	a) What has been your role as an implementer of this intervention?
**2. Meeting participant expectations**	a) What do you think participants expected from the health facility intervention?
	b) Do you think this intervention met their expectations? *Probe: What expectations were met and what else happened that you think they were not expecting?*
**3. Implementation Process**	a) In your opinion, was the training component of the HFI implemented as planned? *Probe for specifics, why?*
	b) In your opinion, was the information management component of the HFI implemented as planned? *Probe for specifics, why?*
	c) In your opinion, was the supply of consumables including malaria diagnostics and antimalarial drugs component of the HFI implemented as planned? *Probe for specifics, why?*
	d) Looking back over the past two years, what component of the intervention do you think was most successfully implemented? *Probe for specifics.*
	e) Looking back over the past two years, what component of the intervention do you think was least successfully implemented? *Probe for specifics.*

PART 2: IMPLEMENTER IN-DEPTH INTERVIEW (3)

Domains, topic questions, and probes: Use the table below to help you administer the questions during the interview.

Domain	Topic and Probes
**4. Training**	Where you directly involved with the training components of the HFI?<br>**If no, skip to Question 4.**
	a) Can you describe the training sessions that you had with the participants? *Probe: what happened in the training?*
	b) What do you think worked particularly well in the training, which will ensure that the participants will take home specific messages?
	c) What impact do you think this training will have on the practices of participants in reality?
	d) What do you think can be strengthened in the training to enable health workers to really change their practice?
**5. Uptake of the intervention**	a) Aside from the training components, can you comment on whether the information and management tools provided were able to be taken up in practice?
	b) What are the enabling factors to using this new knowledge in practice?
	c) In your opinion, what things limit translation of this new knowledge into practice?
**6. Motivation towards job**	a) We want to know how we could do this programme elsewhere. What skills and characteristics do you think are needed to do your job really well?

PART 2: IMPLEMENTER IN-DEPTH INTERVIEW (4)

Domains, topic questions, and probes: Use the table below to help you administer the questions during the interview.

Domain	Topic and Probes
**7. Context**	a) Aside from the HFI, can you describe any other programmes/interventions involving malaria in the area? *Probe: is the programme at the community or health centre level?*
	b) Aside from the HFI, can you describe any other health-related programmes/interventions in the area? *Probe: is the programme at the community or health centre level?*
	c) Are there any other factors you think may have influenced the delivery or receipt of the ACT PRIME HFI?
**8. Closing**	Is there anything else you think is important about the implementation of the HFI intervention that we have not talked about?

- ✓ **Summarise**
- ✓ **Thank participant**

PART 3: CONTACT SUMMARY FORM (1)	
**Complete this form after the interview.**	
**Study ID**<br>[____|____]	**Date**<br>[____|____] / [____|____] / [____|____]<br>day month year
1. How would you describe the atmosphere and context of the interview (*Include interview location and how this may have affected responses)*?	
2. What were the main points made by the respondent during this interview?	

PART 3: CONTACT SUMMARY FORM (2)	
**Study ID**<br>[____|____]	**Date**<br>[____|____] / [____|____] / [____|____]<br>day month year
3. What new information did you gain through this interview compared to previous interviews?	
4. Was there anything surprising to you personally? Or that made you think differently?	
5. What messages did you take from this interview to improve the intervention design?	
6. Were there any problems with the topic guide (e.g. wording, order of topics, missing topics) you experienced in this interview?	

APPENDIX R: IDI DATA COLLECTION TOOL
HEALTH WORKERS (HFI)

Health centre code	Study ID	Date
[____|____]	[____|____]	[____|____] / [____|____] / [____|____]<br>day month year

Position:

1 = In-charge	5 = Clinical officer	9 = Public health nurse	13 = Health assistant	
2 = Senior medical officer	6 = Nursing officer	10 = Nursing aide/assistant	14 = Health educator	
3 = Medical officer	7 = Enrolled nurse	11 = Laboratory technician	15 - Volunteer	
4 = Senior clinical officer	8 = Midwife	12 = Laboratory assistant	15 = Other________________	[____|____]

DEMOGRAPHIC INFORMATION

**1. Age**	Years [____|____]	**5. Highest level of education or qualification achieved**	
**2. Gender**	1 = Male<br>2 = Female [____]	0 = None<br>1 = Primary (P1 — P7)<br>2 = Secondary (S1 — S6)<br>3 = Certificate<br>77 = Other____________________________	4 = Diploma<br>5 = Bachelor's degree<br>88 = Don't know<br>99 = Refused to answer<br>[____|____]
**3. Originally from this area?**	1 = Yes<br>2 = No [____]		
**4. Number of years worked in this job**	[____|____]	**6. Year graduated**	[____|____|____|____]

PART 1: INTRODUCTION

Conduct the interview according to the directions below and record information as indicated.

Introduction to in-depth interview

"Hello my name is ____________________ and I am interested in interviewing you. This interview will ask you to express your own views and experiences about your work and role at this health centre. We are interested in knowing whether improving the health services at this health centre has improved children's health in this area. We are specifically asking you about the ACT PRIME study activities which include 1) health center management training, 2) information management, 3) health worker training in fever case management and patient-centered services, and 4) supply of consumables, including malaria diagnostics and antimalarial drugs.

A note-taker will be writing down what you say for our records, and we will record the interview using a digital recorder; these notes will be kept securely and your name will not be used anywhere. Your answers will be looked at together with those of many other health workers from different facilities and you will not be identifiable in any reports that are published.

It is very important for us to hear your views and experiences because you have experience working here and can give us this insight. We hope you will have time to spend with us now to complete this interview. The interview will take about 45 minutes; if you prefer we can reschedule the interview for tomorrow or another day of your convenience.

Do you have any questions? Do you agree to continue before we start?

Now we request that we all switch off our mobile phones so that we are not distracted."

PART 2: IN-DEPTH INTERVIEW – HEALTH WORKERS (HFI) (1)	
**Domains, topic questions, and probes:** Use the table below to help you administer the questions during the interview.	
**Domain**	**Topic and Probes**
**1. Your role at work**	a) What do you do in your everyday work?
	b) What is the most important thing to you about the job?
	c) How do you feel about this job now?
**2. Significant events**	a) Looking back over the past few months, what do you think was the most significant change in the way you managed illness in your health centre?
	b) Why is this significant to you?
	c) What difference has this made now or will it make in the future?
**3. Reflection on training**	a) How do you feel the ACT PRIME study training you attended has impacted on your work?
	b) Was there anything that you learnt in principle that you have found difficult to put into practice?
	c) Have you attended any other training courses or received any materials or tools from other organizations to help you do your job? If yes, please list.

PART 2: IN-DEPTH INTERVIEW – HEALTH WORKERS (HFI) (2)	
**Domains, topic questions, and probes:** Use the table below to help you administer the questions during the interview.	
**Domain**	**Topic and Probes**
**4. Reflection on health centre management**	a) How would you describe the management of this health centre right now?
	b) Have there been any changes to the way the health centre is managed? *Probe for specifics.*
	c) What difference have these changes made to your work? *Probe: where do you see the greatest impact of health centre management in your every day work?*
	d) What are the greatest challenges that remain for you in the way this health centre is managed?
**Health centre management:** Staffing	a) How would you describe the staffing levels at the health centre right now?
	b) Have there been any changes recently to the staffing at this health centre?
	c) What difference have these changes made to your work? *Probe: where do you see the greatest impact of staffing levels in your everyday work?*
	d) What challenges do you still face in improving staffing levels at this health centre?
**Health centre management:** Budgeting and accounting	a) How would you describe the accounting and budgeting at this health centre right now?

PART 2: IN-DEPTH INTERVIEW – HEALTH WORKERS (HFI) (3)	
**Domains, topic questions, and probes:** Use the table below to help you administer the questions during the interview.	
**Domain**	**Topic and Probes**
	b) Have you made any changes recently to the way you undertake accounting and budgeting at this health centre? *Probe for specifics.*
	c) What difference have these changes made to your work? *Probe: where do you see the greatest impact of accounting and budgeting in your every day work?*
	d) How would you describe the function of the PHC Fund Accounting Tool for accounting activities? *Probe for specifics.*
**Health centre management:** Information management	a) How would you describe the way you manage the information you collect about patients at your health centre? (i.e. what you do with the information in the registers)
	b) What impact does the way you use this information have on your work?
	c) What problems did or have you experienced in implementing the form for recording malaria tests and treatment at the HC?
**Health centre management:** Drug stocking	a) Can you describe the way you re-stock drugs at this health centre?
	b) How have any changes in re-stocking affected your work?

PART 2: IN-DEPTH INTERVIEW – HEALTH WORKERS (HFI) (4)	
**Domains, topic questions, and probes:** Use the table below to help you administer the questions during the interview.	
**Domain**	**Topic and Probes**
	c) What challenges do you still face in re-stocking of drugs at this health centre?
	d) How would you describe the function of the ADDAT for re-stocking activities? *Probe for specifics.*
**5. Reflection on Patient-Centered Services:** Communication with patients	a) How would you describe your relationship with patients who come to this HC?
	b) What are the reasons for the nature of your relationship with patients as you describe?
	c) What is the most significant change you have experienced in the past few months in the way you interact with patients? *Probe: why do you think this change occurred and how did you achieve it?*
	d) Can you describe the impact your relationship with patients has on your work?
**Patient-Centered Services:** Communication with colleagues	a) How would you describe your relationship with your colleagues at your health centre?
	b) What do you think are the reasons for the nature of this relationship with colleagues, as you described it?

PART 2: IN-DEPTH INTERVIEW – HEALTH WORKERS (HFI) (5)	
**Domains, topic questions, and probes:** Use the table below to help you administer the questions during the interview.	
**Domain**	**Topic and Probes**
	c) What is the most significant change you have experienced in the past few months in the way you and your colleagues interact? *Probe: why do you think this change occurred and how are you all achieving it?*
	d) Can you describe the impact your relationship with colleagues has on your work?
**Patient-Centered Services:** Patient flow	a) Can you describe the patient flow at this health centre – which patients are seen in what order? *Probe: how do you achieve this patient flow?*
	b) What is the importance to you of having patients flow through your health centre in this way? *Probe for examples.*
**Patient-Centered Services:** Coaching	a) How would you describe satisfaction with your job at this health centre?
	b) What do you think are the reasons for your satisfaction/dissatisfaction, as you describe it?
	c) Can you describe the impact your satisfaction/dissatisfaction has on your work?
**6. Closing**	Is there anything else you think is important about working at this health centre that we have not talked about?

- ✓ **Summarise**
- ✓ **Thank participant**

PART 3: CONTACT SUMMARY FORM (1)	
**Interviewer to complete this form after the interview**	
**Study ID**<br>[____|____]	**Date**<br>[____|____] / [____|____] / [____|____]<br>day month year
1. How would you describe the atmosphere and context of the interview (*Include interview location and how this may have affected responses)*?	
2. What were the main points made by the respondent during this interview?	

PART 3: CONTACT SUMMARY FORM (2)

Study ID	Date
[____|____]	[____|____] / [____|____] / [____|____]<br>day month year
3. What new information did you gain through this interview compared to previous interviews?	
4. Was there anything surprising to you personally? Or that made you think differently?	
5. What messages did you take from this interview to improve the intervention design?	
6. Were there any problems with the topic guide (e.g. wording, order of topics, missing topics) you experienced in this interview?	

APPENDIX S: IDI DATA COLLECTION TOOL
KEY STAKEHOLDERS

Study ID	Date
[____|____]	[____|____] / [____|____] / [____|____]<br>day month year

Position:

1 = District Health Officer
2 = District Health Inspector
3 = Deputy District Health Officer
4 = Principal Nursing Officer
5 =Chief Administrative Officer
6 = Malaria Focal Person
7 = Local Chairman
8 = MoH staff, Dept_________________________
9 = Other_______________________

[____|____]

DEMOGRAPHIC INFORMATION

**1. Age**	Years [____|____]	**5. Highest level of education or qualification achieved**<br>0 = None<br>1 = Primary (P1 — P7)<br>2 = Secondary (S1 — S6)<br>3 = Certificate<br>4 = Diploma<br>5 = Bachelor's degree<br>88 = Don't know<br>99 = Refused to answer<br>77 = Other____________________________<br>[____|____]
**2. Gender**	1 = Male<br>2 = Female [____]	
**3. Originally from this area?**	1 = Yes<br>2 = No [____]	
**4. Number of years worked in this job**	[____|____]	**6. Year graduated** [____|____|____|____]

PART 1: INTRODUCTION

Conduct the interview according to the directions below and record information as indicated.

Introduction to in-depth interview

"Hello, my name is ………. I work with UMSP (Uganda malaria Surveillance project)/IDRC (Infectious Diseases Research Collaboration). I am interested in asking you a few questions about activities, programmes or other contextual factors occurring in Tororo District or across Uganda in the past year. A note-taker will be writing down what you say for our record and we will record the interview using a digital recorder; these notes will be kept securely and your name will not be used anywhere. Your answers will be looked at together with those of many other health workers from different facilities and you will not be identifiable in any reports that are published.

It is very important for us to hear your views and experiences because your knowledge and experience can give insight to our study. We hope you will have time to spend with us now to complete this interview. The interview will take about 45 minutes; if you prefer we can reschedule the interview for tomorrow or another day of your convenience.

Do you have any questions? Do you agree to continue before we start?

Now we request that we all switch off our mobile phones so that we are not distracted."

PART 2: STAKEHOLDERS IN-DEPTH INTERVIEW (1)

Domains, topic questions, and probes: Use the table below to help you administer the questions during the interview.

Domain	Topic and Probes
**1. Description of job**	a) Can you briefly describe your roles and responsibilities in your job?
	b) What specific role do you play in malaria-related programmes?
	c) Can you describe your involvement, if any, with the implementation of the ACT PRIME health facility intervention?
**2. Significant changes**	a) Looking back over the past year, what do you think is the most significant change in the way illnesses are managed in the area?
	b) Why is this significant to you?
	c) What difference has this made now or will it make in the future?
**3. Changes in HFI components**	a) Can you describe any actions taken to improve the staffing gaps at health centres in the area? *Probe: actions taken for all health centres or only some? Which ones?*
	b) Can you describe any changes to how drugs are re-stocked at health centres in the area? *Probe: changes in all health centres or only some? Which ones?*

PART 2: STAKEHOLDERS IN-DEPTH INTERVIEW (2)	
**Domains, topic questions, and probes:** Use the table below to help you administer the questions during the interview.	
**Domain**	**Topic and Probes**
	c) Can you describe any changes to the information you receive from health centres in the area? *Probe: changes in all health centres or only some? Which ones?*
	d) Can you describe any changes to the relationships between health workers and patients/community members in the area? *Probe: changes in all health centres or only some? Which ones?*
	e) How would you describe the levels of motivation of health workers at the different health centres here? *Probe: Please tell me what you think for each health centre and why you think they are more or less motivated there.*
**Now we would like to know what changes have been as a result of the PRIME study and what other things have also been happening in the area.**	
**4. Contextual factors**	a) Can you describe any changes to health centres in Tororo (i.e. opening or closing of health centres, improvements to health centres)? *Probe: Who is responsible for these changes?*
	b) Can you describe any changes to environmental conditions in Tororo (i.e. severe weather, new roads, swamps, agriculture)?
	c) Can you describe any changes to guidelines about malaria testing and treatment at health centres or the community level?
	d) Can you describe any messages or news stories on the radio, TV or newspaper about malaria testing/prevention/treatment or malaria programmes?

PART 2: STAKEHOLDERS IN-DEPTH INTERVIEW (3)

Domains, topic questions, and probes: Use the table below to help you administer the questions during the interview.

Domain	Topic and Probes
	e) Can you describe any other economic or political factors that you think may have impacted the delivery or receipt of the ACT PRIME health facility intervention?
**5. Other programmes**	a) Besides ACT PRIME, can you describe any other programmes involving malaria in the area? *Probe: are these programmes at the community or health centre level?*
	b) Besides ACT PRIME, can you describe any other health-related programmes in the area? *Probe: are these programmes at the community or health centre level?*
	c) What other training programs involving community health workers or health centre staff are taking place in the area?
**6. Support for the intervention**	a) In your opinion, what is the level of support from health workers for the ACT PRIME health facility intervention?
	b) In your opinion, what is the level of support from district-level staff for the ACT PRIME health facility intervention?
**7. Closing**	Are there any other important contextual factors we have not talked about?

- ✓ **Summarise**
- ✓ **Thank participant**

PART 3: CONTACT SUMMARY FORM (1)

Complete this form after the interview.	
**Study ID**<br>[____|____]	**Date**<br>[____|____] / [____|____] / [____|____]<br>day month year
1. How would you describe the atmosphere and context of the interview (*Include interview location and how this may have affected responses)*?	
2. What were the main points made by the respondent during this interview?	

PART 3: CONTACT SUMMARY FORM (2)	
**Study ID**<br>[____|____]	**Date**<br>[____|____] / [____|____] / [____|____]<br>day month year
3. What new information did you gain through this interview compared to previous interviews?	
4. Was there anything surprising to you personally? Or that made you think differently?	
5. What messages did you take from this interview to improve the intervention design?	
6. Were there any problems with the topic guide (e.g. wording, order of topics, missing topics) you experienced in this interview?	

THE REPUBLIC OF UGANDA

[____]—[____|____|____|____]

SSQ Study ID

APPENDIX T: SEMI-STRUCTURED QUESTIONNAIRES
Informed consent form for health workers and private providers

**Protocol Title:**	ACT PROCESS: Evaluating the process, context, and impact of interventions to enhance health facilities in Tororo, Uganda
**Site of Research:**	Tororo, Uganda
**Principal Investigators**:	Dr. Sarah Staedke
**Date:**	23 February 2011

Introduction

Dr. Sarah Staedke and colleagues from the Uganda Malaria Surveillance Project/Infectious Diseases Research Collaboration are doing a study to evaluate activities that have been introduced at government-run health facilities in this area aiming to improve the health of children. We would like to understand the impact of these activities and why they are working, or not. This study involves several parts, including assessing the communication between health workers and their patients, holding interviews, and conducting group discussions with community members and health workers.

Why is this study being done?

For this part of the study, we would like to learn more about whether the activities that have been introduced at government-run health facilities have had an impact from the perspective of health workers and private drug shop workers. This information will help us understand how and why the health facility activities have affected the health of children in this area.

What will happen today if I take part in this study?

The study will involve a one-time interview. Today, if you are a health worker, we would like to ask you some questions about drug stocks and health center management, diagnosis and treatment of fever and malaria, your attitudes and beliefs about your job, and any changes you have seen over the past few months at your health center. We may also leave some pages of the questionnaire for you to complete in your own time over the next three days. If you are a health worker who participated in the ACT PRIME training, we will also ask you some additional questions about the usefulness of the training. If you work in a private drug shop, we would like to ask you questions about drug stocks, and treatment of fever and malaria, patient attendance, and any changes you have seen over the past few months at your shop. The information we collect will be used by project investigators, and may be shared with other researchers and policy-makers to answer questions about how best to deliver training and health services.

How long will the study last?
Today, the interview will last about 60-90 minutes.

Can I stop being in the study?
You can decide to stop participating at any time. Just tell the project researcher right away if you wish to stop.

What risks can I expect from being in the study?
Participation in any research study may involve a loss of privacy. Your name will not be used in any reports of the information provided. No quotes or other results arising from your participation in this study will be included in any reports, even anonymously, without your agreement. The information obtained from these interviews will be locked at our project offices. We will do our best to make sure that the personal information gathered for this study is kept private.

Are there benefits to taking part in the study?
There will be no direct benefit to you from participating in this study. However, the information that you provide will help researchers and policy-makers understand how best to improve health services in this area.

What other choices do I have if I do not take part in this study?
You are free to choose not to participate in the study. If you decide not to take part in this study, there will be no penalty to you.

What are the costs of taking part in this study? Will I be paid for taking part in this study?
There are no costs to you for taking part in this study. You will not be paid for taking part in this study.

What are my rights if I take part in this study?
Taking part in this study is your choice. You may choose either to take part or not to take part in the study. If you decide to take part in this study, you may change your mind at any time. No matter what decision you take, there will be no penalty to you in any way.

Who can answer my questions about the study?
You can talk to the researchers about any questions or concerns you have about these study activities. Contact Dr. Sarah Staedke or other members of the Uganda Malaria Surveillance Project / Infectious Disease Research Collaboration on telephone number 0414-530692. If you have any questions, comments or concerns about taking part in these activities, first talk to the researchers. If for any reason you do not wish to do this, or you still have concerns about doing so, you may contact Dr Charles Ibingira, Makerere University Faculty of Medicine Research and Ethical Committee at telephone number 0414-530020.

THE REPUBLIC OF UGANDA

WHAT YOUR SIGNATURE OR THUMBPRINT MEANS

Your signature or thumbprint below means that you understand the information given to you in this consent form about your participation in the study and agree with the following statements:

1. "I have read the consent form concerning this study (or have understood the verbal explanation of the consent form) and I understand what will be required of me and what will happen to me if I take part in it."

2. "My questions concerning this study have been answered by Dr. Staedke or the person who signed below."

3. "I understand that at any time, I may withdraw from this study without giving a reason."

4. "I agree to take part in this study."

If you wish to participate in this study, you should sign or place your thumbprint on the following page. If you do not agree to quotes or other results arising from your participation in the study being included, even anonymously, in any reports about the study, please tell the researcher now.

THE REPUBLIC OF UGANDA

WE WILL GIVE YOU A COPY OF THIS SIGNED AND DATED CONSENT FORM

Name of Participant (printed)

Signature or Fingerprint of Participant	Date/Time

Name of Investigator Administering Consent (printed)	Position/Title

Signature of Investigator Administering Consent	Date/Time

If the participant is unable to read and/or write, an impartial witness should be present during the entire informed consent discussion.

After the written informed consent form is read and explained to the participant, and after they have orally consented to participate in the study, the witness should print the name of the participant above, and then the participant should provide their fingerprint.

Then the witness should print their name, provide their signature, and date the consent form below.

By signing the consent form, the witness attests that the information in the consent form and any other written information was accurately explained to, and apparently understood by the participant, and that informed consent was freely given by the participant.

Name of Person Witnessing Consent (printed)

Signature of Person Witnessing Consent	Date/Time

APPENDIX U: SEMI-STRUCTURED QUESTIONNAIRE
HEALTH WORKERS: HFI & STANDARD CARE

Health centre code	Health Worker ID	Date
[____|____]	[____|____|____|____]	[____|____] / [____|____] / [____|____]<br>day month year

Position:

1 = In-charge	5 = Clinical officer	9 = Public health nurse	13 = Health assistant
2 = Senior medical officer	6 = Nursing officer	10 = Nursing aide/assistant	14 = Health educator
3 = Medical officer	7 = Enrolled nurse	11 = Laboratory technician	15 - Volunteer
4 = Senior clinical officer	8 = Midwife	12 = Laboratory assistant	15 = Other________________

[____|____]

DEMOGRAPHIC INFORMATION

1. Age
Years [____|____]

2. Gender
1 = Male
2 = Female [____]

3. 'Are you originally from this area?'
1 = Yes
2 = No [____]

4. 'How long have you worked at this health centre?'
[____|____]years
[____|____]months

5. Highest level of education or qualification achieved

0 = None
1 = Primary (P1 — P7)
2 = Secondary (S1 — S6)
3 = Certificate
77 = Other________________________
4 = Diploma
5 = Bachelor's degree
88 = Don't know
99 = Refused to answer

[____|____]

6. What year did you graduate from your course? [____|____|____|____]

PART 1 (ALL HEALTH WORKERS)
SECTION 1: CHANGES AT WORK

To be administered by the study team

1. "Have you noticed any changes in the way patients are managed at this health center over the past few months?"
1 = Yes 88 = Don't know
2 = No 99 = Refused to answer [_____|_____]

If yes, go to Qn 2. Otherwise, skip to the next section

2. What do you think was the most significant or important change in the way patients are managed at this health center?

3. Why is this significant or important to you?

4. What difference has this made in how patients are managed at this health center?

SSQ: HEALTH WORKERS (2)

Health centre code	Health Worker ID	Date
[____|____]	[____|____|____|____]	[____|____] / [____|____] / [____|____]<br>day month year

SECTION 2: SUPPLY MANAGEMENT

**1. 'Does this health center typically stock artemether-lumefantrine (Coartem or Lumartem)?'**<br>*If yes, go to Qn 2, otherwise skip to Qn 4*	1 = Yes 88 = Don't know<br>2 = No 99 = Refused to answer	[_____|_____]
**2. 'Have there been stock-outs of artemether-lumefantrine (Coartem or Lumartem) in the last 6 months?'**	1 = Yes 88 = Don't know<br>2 = No 99 = Refused to answer	[_____|_____]
**3. 'On a typical day, is the supply of artemether-lumefantrine (Coartem or Lumartem) adequate to treat the number of malaria patients seen at this health center?'**	1 = Yes 88 = Don't know<br>2 = No 99 = Refused to answer	[_____|_____]
**4. 'Does this health center typically stock rapid diagnostic tests (RDTs) for malaria?'**<br>*If yes, go to Qn 5, otherwise skip to Qn 7*	1 = Yes 88 = Don't know<br>2 = No 99 = Refused to answer	[_____|_____]
**5. 'Have there been stock-outs of RDTs in the last 6 months?'**	1 = Yes 88 = Don't know<br>2 = No 99 = Refused to answer	[_____|_____]
**6. 'On a typical day, is the supply of RDTs adequate to manage the number of patients seen at this health center?'**	1 = Yes 88 = Don't know<br>2 = No 99 = Refused to answer	[_____|_____]
**7. 'On a typical day, is the supply of other drugs adequate to treat the number of patients seen at this health center?'**	1 = Yes 88 = Don't know<br>2 = No 99 = Refused to answer	[_____|_____]
**8. 'On a typical day, does this health center have adequate equipment and supplies to manage patients?'**	1 = Yes 88 = Don't know<br>2 = No 99 = Refused to answer	[_____|_____]

9. Please provide any additional comments about supply of drugs and other supplies at this health center.

SECTION 3: STAFFING

**1. "How many full-time staff members are stationed at this health facility?"**	Indicate number of staff	[_____|_____]
**2. "On a typical day, how many full-time staff members are available to work?"**	Indicate number of staff	[_____|_____]
**3. "How many volunteers are stationed at this health facility?"**	Indicate number of volunteers	[_____|_____]
**4. "On a typical day, how many volunteers are available?"**	Indicate number of volunteers	[_____|_____]
**5. "On a typical day, are the staff available at this health center adequate to manage the number of patients attending?"**	1 = Yes 88 = Don't know<br>2 = No 99 = Refused to answer	[_____|_____]

6. Please provide any additional comments about staffing and use of volunteers at this health center.

SSQ: HEALTH WORKERS (3)

Health centre code	Health Worker ID	Date
[____|____]	[____|____|____|____]	[____|____] / [____|____] / [____|____]<br>day month year

SECTION 4: FINANCIAL MANAGEMENT

**1. 'In the last 6 months, has this health center had enough money available to buy all of the supplies needed for day-to-day running of the facility (e.g. for soap, repairs, etc)?'**	1 = Yes 88 = Don't know<br>2 = No 99 = Refused to answer	[______|______]
**2. 'In the last 6 months, has this health center had enough money available to pay for support staff to help clean and maintain the health center?'**	1 = Yes 88 = Don't know<br>2 = No 99 = Refused to answer	[______|______]
**3. 'In general, how often does this health facility receive PHC funds?'**	1 = Regularly every quarter (4 times a year)<br>2 = Regularly every 3-6 mo (2-3 times a year)<br>3 = Irregularly, about 2 times a year<br>4 = Irregularly, about once a year<br>5 = Never<br>88 = Don't know<br>99 = Refused to answer	[______|______]
**4. 'In the last 6 months, has this health center received any PHC funds?'** *If yes, go to Qn 5, otherwise skip to Qn 6*	1 = Yes 88 = Don't know<br>2 = No 99 = Refused to answer	[______|______]
**5. 'In the last 6 months, have the PHC funds been adequate to cover your needs at this health center?'**	1 = Yes 88 = Don't know<br>2 = No 99 = Refused to answer	[______|______]
**6. 'In your opinion, when PHC funds are available, are they used in the right way at this health center?'**	1 = Yes 88 = Don't know<br>2 = No 99 = Refused to answer	[______|______]
**7. 'In your opinion, are staff members in this health center able to budget and account for funds well?'**	1 = Yes 88 = Don't know<br>2 = No 99 = Refused to answer	[______|______]

8. Please provide any additional comments about management of money and PHC funds at this health center.

SECTION 5: PATIENT MANAGEMENT

1. "Is it possible to test patients for malaria at this health center?" 1 = Yes 88 = Don't know; 2 = No 99 = Refused to answer [______|______]

If YES, go to Qn 2, otherwise skip to Qn 5

2. 'What tests for malaria can be done at this health center?' Read out each test and indicate for each 1 = Yes 88 = Don't know; 2 = No 99 = Refused to answer

[_____] Microscopy (blood smear)
[_____] Rapid diagnostic test for malaria
[_____] Other (describe)____

3. "Do you usually test patients for malaria before giving antimalarial treatment?" 1 = Yes 88 = Don't know; 2 = No 99 = Refused to answer [______|______]

If NO, go to Qn 4, otherwise skip to Qn 5

SSQ: HEALTH WORKERS (4)

Health centre code	Health Worker ID	Date
[____|____]	[____|____|____|____]	[____|____] / [____|____] / [____|____]<br>day month year

SECTION 5 cont: PATIENT MANAGEMENT

**4. "If you do not usually test patients for malaria before giving treatment, why?"**<br>*Record all answers given*	1 = There is not enough time to test all patients<br>2 = We lack the supplies needed to do the tests<br>3 = Patients/Caregivers are not willing to be tested<br>4 = I don't trust the results of the malaria tests<br>5 = I know better than the test when a patient has malaria<br>6 = In my experience, all fevers are due to malaria<br>7 = Malaria tests are not done correctly at this health center<br>77 = Other ________________________________<br>88 = Don't know<br>99 = Refused to answer	[_____|_____]<br>[_____|_____]<br>[_____|_____]<br>[_____|_____]
**5. "What treatment should be given to patients with uncomplicated malaria?"**<br>*Record all answers given*	1 = Artemether-lumefantrine (Coartem/Lumartem)<br>2 = Artesunate + amodiaquine<br>3 = Dihydroartemisinin-piperaquine (Duocotexcin)<br>4 = Any artemisinin-based combination therapy<br>5 = Quinine<br>6 = Chloroquine + sulfadoxine-pyrimethamine (Homapak)<br>77 = Other ________________________________<br>88 = Don't know<br>99 = Refused to answer	[_____|_____]<br>[_____|_____]<br>[_____|_____]<br>[_____|_____]
**6. 'Are you usually able to provide this treatment to your patients with uncomplicated malaria?'**<br>*If NO, go to Qn 7, otherwise skip to Qn 8*	1 = Yes<br>2 = No<br>88 = Don't know<br>99 = Refused to answer	[_____|_____]
**7. "If not, why?"**<br>*Record all answers given*	1 = The drug is often out of stock<br>2 = The patients can't afford to buy<br>77 = Other ________________________________<br>88 = Don't know<br>99 = Refused to answer	[_____|_____]<br>[_____|_____]
**8. 'How confident are you that you can correctly <u>diagnose</u> malaria in your patients?'**	1 = Very confident<br>2 = Confident<br>3 = Not confident<br>88 = Don't know<br>99 = Refused to answer	[_____|_____]
**9. 'How confident are you that you can correctly <u>treat</u> malaria in your patients?'**	1 = Very confident<br>2 = Confident<br>3 = Not confident<br>88 = Don't know<br>99 = Refused to answer	[_____|_____]
**10. 'How confident are you that you can correctly <u>diagnose</u> other illnesses (not malaria) in your patients?'**	1 = Very confident<br>2 = Confident<br>3 = Not confident<br>88 = Don't know<br>99 = Refused to answer	[_____|_____]
**11. 'How confident are you that you can correctly <u>treat</u> other illnesses (not malaria) in your patients?'**	1 = Very confident<br>2 = Confident<br>3 = Not confident<br>88 = Don't know<br>99 = Refused to answer	[_____|_____]

SSQ: HEALTH WORKERS (5)

Health centre code	Health Worker ID	Date
[____|____]	[____|____|____|____]	[____|____] / [____|____] / [____|____]<br>day month year

SECTION 5 cont: PATIENT MANAGEMENT

**12. "Are you able to give your patients a full explanation about their diagnosis and treatment?"**<br>*If NO, go to Qn 13. Otherwise, skip to Qn 14.*	1 = Yes 88 = Don't know<br>2 = No 99 = Refused to answer	[_____|_____]
**13. "If not, why?"**<br>*Record all answers given*	1 = I do not have enough time<br>2 = The patients are not willing to wait<br>3 = Sometimes I am not sure of the diagnosis<br>4 = Sometimes I am not sure of the treatment<br>77 = Other ________________________________<br>88 = Don't know<br>99 = Refused to answer	[_____|_____]<br>[_____|_____]<br>[_____|_____]
**14. "Are you able to refer severely ill patients to a higher-level health center when needed?"**<br>*If NO, go to Qn 15. Otherwise, skip to next section.*	1 = Yes 88 = Don't know<br>2 = No 99 = Refused to answer	[_____|_____]
**15. "If not, why?"**<br>*Record all answers given*	1 = The patients lack the money to go<br>2 = The patients don't have time to go<br>3 = The other health centers turn my patients away<br>4 = I don't know how to refer<br>77 = Other________________________________<br>88 = Don't know<br>99 = Refused to answer	[_____|_____]<br>[_____|_____]<br>[_____|_____]

16. Please provide any additional comments about patient management at this health center.

SSQ: HEALTH WORKERS (6)

Health centre code	Health Worker ID	Date
[____|____]	[____|____|____|____]	[____|____] / [____|____] / [____|____]<br>day month year

SECTION 6: PATIENT-CENTERED SERVICES (Self-filled)

This page may be left with the health worker to complete on their own (allow up to 3 days to complete)

Question	Response options		Code
**1. Patients should look up to health workers.**	1 = Strongly agree<br>2 = Agree<br>3 = Disagree	4 = Strongly disagree<br>88 = Don't know<br>99 = Refused to answer	[_____|_____]
**2. Health workers should be expected to help patients deal with non-medical problems.**	1 = Strongly agree<br>2 = Agree<br>3 = Disagree	4 = Strongly disagree<br>88 = Don't know<br>99 = Refused to answer	[_____|_____]
**3. Health workers should explain their diagnosis and treatment to all patients.**	1 = Strongly agree<br>2 = Agree<br>3 = Disagree	4 = Strongly disagree<br>88 = Don't know<br>99 = Refused to answer	[_____|_____]
**4. It is not necessary to explain diagnosis and treatment to all patients as some patients won't be able to understand.**	1 = Strongly agree<br>2 = Agree<br>3 = Disagree	4 = Strongly disagree<br>88 = Don't know<br>99 = Refused to answer	[_____|_____]
**5. If a health worker is uncertain about the cause of a patient's symptoms or diagnosis, this should be explained to the patient.**	1 = Strongly agree<br>2 = Agree<br>3 = Disagree	4 = Strongly disagree<br>88 = Don't know<br>99 = Refused to answer	[_____|_____]
**6. Patients have a right to ask their health worker for information about their health, diagnosis, and treatment.**	1 = Strongly agree<br>2 = Agree<br>3 = Disagree	4 = Strongly disagree<br>88 = Don't know<br>99 = Refused to answer	[_____|_____]

SECTION 7: ATTITUDE TOWARD WORK (Self-filled)

Question	Response options		Code
**1. Overall I am very satisfied in my job.**	1 = Strongly agree<br>2 = Agree<br>3 = Disagree	4 = Strongly disagree<br>88 = Don't know<br>99 = Refused to answer	[_____|_____]
**2. I am most motivated to do my job as health worker by the salary I receive.**	1 = Strongly agree<br>2 = Agree<br>3 = Disagree	4 = Strongly disagree<br>88 = Don't know<br>99 = Refused to answer	[_____|_____]
**3. I am most motivated to do my job as health worker by my desire to help people.**	1 = Strongly agree<br>2 = Agree<br>3 = Disagree	4 = Strongly disagree<br>88 = Don't know<br>99 = Refused to answer	[_____|_____]
**4. I am able to complete all of the work I am expected to do each day.**	1 = Strongly agree<br>2 = Agree<br>3 = Disagree	4 = Strongly disagree<br>88 = Don't know<br>99 = Refused to answer	[_____|_____]
**5. The health workers who get promoted are the ones that are best at their job.**	1 = Strongly agree<br>2 = Agree<br>3 = Disagree	4 = Strongly disagree<br>88 = Don't know<br>99 = Refused to answer	[_____|_____]
**6. The salary I receive is fair, given my level of training and experience.**	1 = Strongly agree<br>2 = Agree<br>3 = Disagree	4 = Strongly disagree<br>88 = Don't know<br>99 = Refused to answer	[_____|_____]
**7. This health center provides everything I need to do my job well.**	1 = Strongly agree<br>2 = Agree<br>3 = Disagree	4 = Strongly disagree<br>88 = Don't know<br>99 = Refused to answer	[_____|_____]
**8. The district management team communicates well with our health center**	1 = Strongly agree<br>2 = Agree<br>3 = Disagree	4 = Strongly disagree<br>88 = Don't know<br>99 = Refused to answer	[_____|_____]

SSQ: HEALTH WORKERS (7)		
**Health centre code** [____|____]	**Health Worker ID** [____|____|____|____]	**Date** [____|____] / [____|____] / [____|____] day month year

SECTION 8: ADDITIONAL COMMENTS (Self-filled)

This page may be left with the health worker to complete on their own (allow up to 3 days to complete)

Please use this space to provide any other comments about the quality of services provided at your health centre, any changes that have improved services, and how the quality of services offered to your local population could be improved.

SSQ: HEALTH WORKERS (8)

Health centre code	Health Worker ID	Date
[____|____]	[____|____|____|____]	[____|____] / [____|____] / [____|____]<br>day month year

PART 2 (HFI HEALTH WORKERS ONLY)

To be administered by the study team

1. Did you attend the following PRIME Training modules?

1 = Yes 88 = Don't know
2 = No 99 = Refused to answer

HCM 01 [______|______]	PCS 00 [______|______]	PCS 03 [______|______]
HCM 02 [______|______]	PCS 01 [______|______]	PCS 04 [______|______]
HCM 03 [______|______]	PCS 02 [______|______]	PCS 05 [______|______]
		JUMP [______|______]

2. Have you used the skills you learned in the training sessions in your everyday work?

Only ask questions related to the training module the health worker attended.

1 = Yes, regularly
2 = Yes, but infrequently
3 = No, never
88 = Don't know
99 = Refuse to answer

HCM 01	Budgeting & accounting for the PHC Fund using the PHC Fund Management Tool	[______|______]
HCM 02	Managing drug stocks using the Drug Stock Card and Requisition & Issue Voucher	[______|______]
	Managing distribution of drugs to your health centre using the ADDAT	[______|______]
HCM 03	Using patient information for clinical and health centre management decisions	[______|______]
PCS 00	Building self-awareness through self-observation activities	[______|______]
PCS 01	Building rapport with patients	[______|______]
	Active listening	[______|______]
PCS 02	Giving information to patients	[______|______]
	Managing RDT negative results	[______|______]
PCS 03	Creating a positive work environment	[______|______]
	Motivation towards your job	[______|______]
PCS 04 / PCS 05	Welcoming and orienting patients	[______|______]

**3. How often is the PHC Fund Management tool used?**	1 = Every week<br>2 = Every month<br>3 = Every time the PHC fund is expected	4 = Not often / Never<br>88 = Don't know<br>99 = Refused to answer	[______|______]
**4. How easy is the PHC Fund Management tool to use?**	1 = Easy<br>2 = Somewhat easy<br>3 = Not easy / difficult	88 = Don't know<br>99 = Refused to answer	[______|______]
**5. How useful is the PHC Fund Management tool for budgeting and accounting?**	1 = Useful<br>2 = Somewhat useful<br>3 = Not useful	88 = Don't know<br>99 = Refused to answer	[______|______]
**6. How often is the ADDAT tool used?**	1 = Every week<br>2 = Every month<br>3 = Every time a drug delivery is expected	4 = Not often / Never<br>88 = Don't know<br>99 = Refused to answer	[______|______]
**7. How easy is the ADDAT tool to use?**	1 = Easy<br>2 = Somewhat easy<br>3 = Not easy / difficult	88 = Don't know<br>99 = Refused to answer	[______|______]
**8. How useful is the ADDAT tool for managing issues in the distribution of drugs from the district/sub-district to your health centre?**	1 = Useful<br>2 = Somewhat useful<br>3 = Not useful	88 = Don't know<br>99 = Refused to answer	[______|______]

APPENDIX V: SEMI-STRUCTURED QUESTIONNAIRE
PRIVATE DRUG SHOP WORKERS

Drug shop code	Provider ID	Date
[____|____]	[____|____|____|____]	[____|____] / [____|____] / [____|____]<br>day month year

DEMOGRAPHIC INFORMATION

**1. Age** Years [____|____]	**5. Highest level of education or qualification achieved**<br>0 = None<br>1 = Primary (P1 — P7)<br>2 = Secondary (S1 — S6)<br>3 = Certificate<br>77 = Other________________________<br>4 = Diploma<br>5 = Bachelor's degree<br>88 = Don't know<br>99 = Refused to answer<br>[____|____]
**2. Gender** 1 = Male 2 = Female [____]	
**3. 'Are you originally from this area?'** 1 = Yes 2 = No [____]	
**4. 'How long have you worked at this drug shop?'** [____|____]years [____|____]months	**6. What year did you graduate from your course?** [____|____|____|____]

SECTION 1: CHANGES AT WORK

1. "Have you noticed any changes in the way patients are managed at this drug shop over the past few months?"

1 = Yes 88 = Don't know
2 = No 99 = Refused to answer [_____|_____]

If yes, go to Qn 2. Otherwise, skip to the next section

2. What do you think was the most significant or important change in the way patients are managed at this drug shop?

3. Why is this significant or important to you?

4. What difference has this made in how patients are managed at this drug shop?

SSQ: PRIVATE DRUG SHOPS (2)

Drug shop code	Provider ID	Date
[____|____]	[____|____|____|____]	[____|____] / [____|____] / [____|____]<br>day month year

SECTION 2: SUPPLY MANAGEMENT

**1. 'Does this drug shop typically stock artemether-lumefantrine?'**<br>*If yes, go to Qn 2, otherwise skip to Qn 5*	1 = Yes 88 = Don't know<br>2 = No 99 = Refused to answer	[_____|_____]
**2. 'Is artemether-lumefantrine available today?'**	1 = Yes 88 = Don't know<br>2 = No 99 = Refused to answer	[_____|_____]
**3. 'Have there been stock-outs of artemether-lumefantrine in the last 6 months?'**	1 = Yes 88 = Don't know<br>2 = No 99 = Refused to answer	[_____|_____]
**4. 'Does this drug shop stock 'Green Leaf' artemether-lumefantrine supported by AMFm?'**	1 = Yes 88 = Don't know<br>2 = No 99 = Refused to answer	[_____|_____]

ARTEMETHER-LUMEFANTRINE BRANDS AVAILABLE

	Stock	Available today	Cost per unit	Unit
	1=Always<br>2=Sometimes<br>3=Never	1=Yes<br>2=No	Ugandan shillings	1=Package<br>2=Tablet
5a. Coartem (Novartis, Switzerland)	[____]	[____]	[____|____],[____|____|____]	[____]
5b. Lumartem (Cipla, India)	[____]	[____]	[____|____],[____|____|____]	[____]
5c. Lomart (Agog, India)	[____]	[____]	[____|____],[____|____|____]	[____]
5d. Artefan (Ajanta Pharma, India)	[____]	[____]	[____|____],[____|____|____]	[____]
5e. Lumiter (Macleods, India)	[____]	[____]	[____|____],[____|____|____]	[____]
5f. Lonart (Milan Labs, India)	[____]	[____]	[____|____],[____|____|____]	[____]
5g. Green leaf (AMFm)	[____]	[____]	[____|____],[____|____|____]	[____]
5h. Other	[____]	[____]	[____|____],[____|____|____]	[____]
5i. Other	[____]	[____]	[____|____],[____|____|____]	[____]
5j. Other	[____]	[____]	[____|____],[____|____|____]	[____]

OTHER ARTEMISININ-BASED COMBINATION THERAPIES

**6. 'Does this drug shop typically stock other artemisinin-based drugs (ACTs)?'**	1 = Yes 88 = Don't know<br>2 = No 99 = Refused to answer	[_____|_____]

If YES, list other ACTs typically stocked at this drug shop, including: artesunate + amodiaquine, dihydroartemisinin + piperaquine, artemisinin + napthoquine (ARCO), artesunate + mefloquine

Brand Name	Manufacturer	Stock<br>1=Always<br>2=Sometimes<br>3=Never	Available today<br>1=Yes<br>2=No
7a.		[____]	[____]
7b.		[____]	[____]
7c.		[____]	[____]
7d.		[____]	[____]
7e.		[____]	[____]
7f.		[____]	[____]
7g.		[____]	[____]

SSQ: PRIVATE DRUG SHOPS (3)

Drug shop code	Provider ID	Date
[____|____]	[____|____|____|____]	[____|____] / [____|____] / [____|____]<br>day month year

SECTION 2 cont: SUPPLY MANAGEMENT

OTHER ANTIMALARIAL DRUGS

7. 'Does this drug shop typically stock other malaria drugs?' 1 = Yes 88 = Don't know 2 = No 99 = Refused to answer [_____|_____]

If YES, review the list below and complete the information on stocking	Stock<br>1=Always<br>2=Sometimes<br>3=Never	Available today<br>1=Yes<br>2=No
8a. Chloroquine	[____]	[____]
8b. Amodiaquine	[____]	[____]
8c. Sulfadoxine-pyrimethamine	[____]	[____]
8d. Quinine oral	[____]	[____]
8e. Quinine injectable	[____]	[____]
8f. Artesunate injectable	[____]	[____]
8g. Primaquine	[____]	[____]
8h. Mefloquine	[____]	[____]
8i. Other	[____]	[____]

9. Please provide any additional comments about supply of drugs at this drug shop.

SECTION 3: PATIENT LOAD

1. "On average, how many people visit this drug shop each week?" Total number of customers per week [_____|_____]

2. "Do you think that there has been a change in the number of people who visit your drug shop each week in the last year?" 1 = Yes 88 = Don't know 2 = No 99 = Refused to answer

If YES, go to Qn 3. Otherwise skip to next section. [_____|_____]

3. "If so, what kind of changes have you noticed?"

Record all answers given

1 = More customers come to the drug shop
2 = Fewer customers come to the drug shop
3 = More patients with malaria come to the drug shop
4 = Fewer patients with malaria come to the drug shop
5 = Customers ask for different drugs *(specify)*
____
77 = Other ___________________________________
88 = Don't know
99 = Refused to answer

[_____|_____]
[_____|_____]
[_____|_____]

SSQ: PRIVATE DRUG SHOPS (4)

Drug shop code	Provider ID	Date
[____|____]	[____|____|____|____]	[____|____] / [____|____] / [____|____]<br>day month year

SECTION 3 cont: PATIENT LOAD

4. "What do you think is the reason for this change?"

5. Please provide any additional comments about patient load at this drug shop.

SECTION 4: PATIENT MANAGEMENT

**1. "What treatments do you most commonly recommend for children with fever?"**<br><br>*Record all answers given*	1 = Artemether-lumefantrine (Coartem/Lumartem)<br>2 = Chloroquine + sulfadoxine-pyrimethamine (Homapak)<br>3 = Quinine<br>4 = Paracetamol (Panadol)<br>77 = Other ________________<br>88 = Don't know<br>99 = Refused to answer	[_____|_____]<br>[_____|_____]<br>[_____|_____]<br>[_____|_____]
**2. "What treatments do you most commonly recommend for adults with fever?"**<br><br>*Record all answers given*	1 = Artemether-lumefantrine (Coartem/Lumartem)<br>2 = Chloroquine + sulfadoxine-pyrimethamine (Homapak)<br>3 = Quinine<br>4 = Paracetamol (Panadol)<br>77 = Other ________________<br>88 = Don't know<br>99 = Refused to answer	[_____|_____]<br>[_____|_____]<br>[_____|_____]<br>[_____|_____]
**3. "What treatments do you most commonly recommend for a patient with uncomplicated (simple) malaria?"**<br><br>*Record all answers given*	1 = Artemether-lumefantrine (Coartem/Lumartem)<br>2 = Artesunate + amodiaquine<br>3 = Dihydroartemisinin-piperaquine (Duocotexcin)<br>4 = Any artemisinin-based combination therapy<br>5 = Quinine<br>6 = Chloroquine + sulfadoxine-pyrimethamine (Homapak)<br>77 = Other ________________<br>88 = Don't know<br>99 = Refused to answer	[_____|_____]<br>[_____|_____]<br>[_____|_____]<br>[_____|_____]

4. Please provide any additional comments about patient management at this drug shop.

SSQ: PRIVATE DRUG SHOPS (5)

Drug shop code	Provider ID	Date
[____|____]	[____|____|____|____]	[____|____] / [____|____] / [____|____]<br>day month year

SECTION 5: ADDITIONAL COMMENTS

Please use this space to provide any other comments

THE REPUBLIC OF UGANDA

[____|____|____|____|____|____|____|____]

FGD Study ID

APPENDIX W. FOCUS GROUP DISCUSSIONS
Informed consent form for caregivers and community health workers

**Protocol Title:**	ACT PROCESS: Evaluating the process, context, and impact of interventions to enhance health facilities in Tororo, Uganda
**Site of Research:**	Tororo, Uganda
**Principal Investigators**:	Dr. Sarah Staedke
**Date:**	23 February 2011

Introduction

Dr. Sarah Staedke and colleagues from the Uganda Malaria Surveillance Project/Infectious Diseases Research Collaboration are doing a study to evaluate activities that have been introduced at government-run health facilities in this area aiming to improve the health of children. We would like to understand the impact of these activities and why they are working, or not. This study involves several parts, including assessing the communication between health workers and their patients, holding interviews, and conducting group discussions with community members and health workers.

Why is this study being done?

For this part of the study, we would like to learn more about whether the activities that have been introduced at government-run health facilities have had an impact from the perspective of members of the community and health workers. This information will help us understand how and why the health facility activities have affected the health of children in this area. You are being asked to take part in a group discussion for this study because of your experiences with caring for and treating ill children.

What will happen today if I take part in this study?

If you agree, you will take part in a discussion about treating ill children and your experiences with health centers (if you are a primary caregiver) and about health services in this area and your experiences with your job (if you are a community health worker). We are interested to hear about your experiences and opinions; there are no right or wrong answers. We will take notes of the ideas discussed and a recording will be made of this discussion using a digital voice recorder. Afterwards, we will enter information from the discussion into a computer for analysis. The information we collect will be used by project investigators, and may be shared with other researchers and policy-makers to answer questions about how best to deliver training and health services.

What is the location of the study?
The discussion will take place in an agreed location within your area.

How long will the study last?
Participation in the study will involve a one-time discussion lasting about one to three hours.

Can I stop being in the study?
You can decide to stop participating at any time. Just tell the project researcher right away if you wish to stop.

What risks can I expect from being in the study?
Participation in any research study may involve a loss of privacy. Your name will not be used in any reports of the information provided. No quotes or other results arising from your participation in this study will be included in any reports, even anonymously, without your agreement. The information obtained from these interviews will be locked at our project offices. We will do our best to make sure that the personal information gathered for this study is kept private.

Are there benefits to taking part in the study?
There will be no direct benefit to you from participating in this study. However, the information that you provide will help researchers and policy-makers understand how best to improve health services in this area.

What other choices do I have if I do not take part in this study?
You are free to choose not to participate in the study. If you decide not to take part in this study, there will be no penalty to you.

What are the costs of taking part in this study? Will I be paid for taking part in this study?
There are no costs to you for taking part in this study. You will not be paid for taking part in this study, but you will be given 5,000/= Ush to refund the cost of your transport.

What are my rights if I take part in this study?
Taking part in this study is your choice. You may choose either to take part or not to take part in the study. If you decide to take part in this study, you may change your mind at any time. No matter what decision you take, there will be no penalty to you in any way.

Who can answer my questions about the study?
You can talk to the researchers about any questions or concerns you have about these study activities. Contact Dr. Sarah Staedke or other members of the Uganda Malaria Surveillance Project / Infectious Disease Research Collaboration on telephone number 0414-530692. If you have any questions, comments or

concerns about taking part in these activities, first talk to the researchers. If for any reason you do not wish to do this, or you still have concerns about doing so, you may contact Dr Charles Ibingira, Makerere University Faculty of Medicine Research and Ethical Committee at telephone number 0414-530020.

WHAT YOUR SIGNATURE OR THUMBPRINT MEANS

Your signature or thumbprint below means that you understand the information given to you in this consent form about your participation in the study and agree with the following statements:

1. "I have read the consent form concerning this study (or have understood the verbal explanation of the consent form) and I understand what will be required of me and what will happen to me if I take part in it."

2. "My questions concerning this study have been answered by Dr. Staedke or the person who signed below."

3. "I understand that at any time, I may withdraw from this study without giving a reason."

4. "I agree to take part in this study."

If you wish to participate in this study, you should sign or place your thumbprint on the following page. If you do not agree to quotes or other results arising from your participation in the study being included, even anonymously, in any reports about the study, please tell the researcher now.

THE REPUBLIC OF UGANDA

WE WILL GIVE YOU A COPY OF THIS SIGNED AND DATED CONSENT FORM

____

Name of Participant (printed)

____

Signature or Fingerprint of Participant — Date/Time

____

Name of Investigator Administering Consent (printed) — Position/Title

____

Signature of Investigator Administering Consent — Date/Time

> *If the participant is unable to read and/or write, an impartial witness should be present during the entire informed consent discussion.*
>
> *After the written informed consent form is read and explained to the participant, and after they have orally consented to participate in the study, the witness should print the name of the participant above, and then the participant should provide their fingerprint.*
>
> *Then the witness should print their name, provide their signature, and date the consent form below.*
>
> *By signing the consent form, the witness attests that the information in the consent form and any other written information was accurately explained to, and apparently understood by the participant, and that informed consent was freely given by the participant.*

____

Name of Person Witnessing Consent (printed)

____

Signature of Person Witnessing Consent — Date/Time

APPENDIX X: FGD DATA COLLECTION TOOL
Community health workers

PART 1: PARTICIPANT DETAILS

Record the demographic details for each participant using the primary caregiver or heads of household FGD participant log as appropriate.

PART 2: FGD INTRODUCTION

**Subcountry ID**	[_____]	**Moderator initials**	[_____|_____|_____]
**FGD ID number**	[_____|_____|_____]	**Note-taker initials**	[_____|_____|_____]
**Location of village** 1 = <2 km 2 = >2 km	[______|______]	**Gender of participants** 1 = Male 2 = Female	[______|______]
**HFI or Standard care** 1 = HFI 2 = Standard care	[______|______]	**Health center in parish** 1 = Yes 2 = No	[______|______]
**Date:**	[____|____] / [____|____] / [____|____] day month year	**Time start** / **Time end**	[_____|_____] : [_____|_____] / [_____|_____] : [_____|_____]

Introduction

I am ______________________________ from ______________________ (moderator)
I am ______________________________ from ______________________ (note-taker)

- *Hello. My name is.... and I work for UMSP (Uganda Malaria Surveillance Project)/IDRC (Infectious Diseases infectious Collaboration.*
- *Thank you very much for coming today. I am part of a team who are doing research to learn more about how people in Tororo treat illnesses in their children and the role of community health workers and health centers in this area. We would like to understand more about the situation of workers in health centres like yours in terms of treating sick children. In addition to our discussion with you today, we are talking with other health care workers, mothers and heads of households in this district.*
- *Today we would like you to take part in a discussion about treating sick children and your experiences with this in the context where you work. We have invited you to participate because you have experience with this here and we hope you will tell us the real situation from your point of view.*
- *The discussion will last for about one hour. We will take notes of the ideas discussed and, if you agree, a tape recording will be made of the discussion in order that we record what you say accurately. When the tape is on, the light will be red. If you wish to say anything 'off the record' this is fine, please indicate to the moderator or note taker. The audiotape will only be used by the study team: no one else will hear your voice. It will be kept for 2 years for our records and will then be erased or destroyed.*
- *We are not writing down your names here and no one will be able to identify you in any reports arising out of this research. All records of this discussion will be kept securely.*
- *Does anyone have any questions?*
- *Let's begin by setting some group rules"*
  - ✓ Ground rules set by group, e.g.
    - Only one person talks at a time.
    - Speak clearly
    - It is important for us to hear everyone's ideas and opinions. There is no right or wrong answers to questions – just ideas, experiences and opinions, which are all valuable.
    - It is important for us to hear all sides of an issue – the positive and the negative.
    - Confidentiality is assured. "What is shared in the room stays in the room."
    - TURN OFF MOBILE PHONES
  - ✓ Consent
  - ✓ Ask group to introduce themselves using first names and their role and health centre
  - ✓ Demographic details – please only use each others' first name for discussion

PART 3: FGD TOPIC GUIDES (1)

Domains, topic questions, and probes: Use the table below to help you administer the questions during the interview.

'Now I am going to introduce some topics one at a time about your experiences when your children are unwell, and I hope you can discuss them together.'

Domain	Topic and Probes
**1. Significant events**	a) Looking back over the past couple of years, what do you think was the most significant change in the way you managed illness in your community?
	b) Why is this significant to you?
	c) What difference has this made now or will it make in the future?
**2. Relationship with patients**	a) Can you describe the type of patients who come to visit you? *Probe: why do they come? how many come?*
	b) When do patients come to see you? *Probe: at what stage in their illness?*
	c) Do you think there has been a change over the past couple of years in the types of patients that come to see you?
	d) What do you think is the reason for this change?
	e) How do you manage patients who come to see you? *Probe: why do you refer? Where do you refer them to?*

PART 3: FGD TOPIC GUIDES (2)	
**Domains, topic questions, and probes:** Use the table below to help you administer the questions during the interview.	
**Domain**	**Topic and Probes**
	f) Why do you think people would choose to come to you rather than taking their child to other sources of treatment?
**3. CHW training**	a) What health related activities are you currently involved in?
	b) Can you describe the type of training you received in the last couple of years? *Probe: who provided the training? What was it about?*
	c) How has the training impacted on your job as a community health worker? *Probe for contribution and challenges.*
**4. Treatment**	b) Over the past couple of years, have there been any changes to the way you treat malaria?
	c) What do you think are the reasons for these changes?
**5. Relationship with community**	a) How would you describe your interactions with the community members in this area?
	b) Have your interactions changed over the past couple of years? *Probe for reasons and specifics.*

PART 3: FGD TOPIC GUIDES (3)	
**Domains, topic questions, and probes:** Use the table below to help you administer the questions during the interview.	
**Domain**	**Topic and Probes**
**6. Relationship with health workers**	a) How would you describe your interactions with the health workers at the nearest health centre?
	b) Have your interactions changed over the past couple of years? *Probe for reasons and specifics.*
**7. Drugs**	a) Have there been any changes to your drug supply over the past couple of years? *Probe for specifics, ie drugs always available, sometimes available, never available.*
	b) If yes, who supplied the drugs to you and what type of drugs did you receive?
**8. Closing**	We are now approaching the end of our discussion. Is there anything else anyone would like to add about your job that we have not talked about?

- ✓ **Summarise main points made by the participants**
- ✓ **Thank participants**

PART 4: NOTE-TAKER FORM

**FGD ID number** [_____|_____|_____]	**Moderator initials** [_____|_____|_____]
**Sub-county code** [_____]	**Note-taker initials** [_____|_____|_____]
**FGD type** 1 = Primary caregiver 2 = Heads of household [_____|_____]	**Gender of participants** 1 = Male 2 = Female [_____|_____]
**Age of participants** 1 = < 30 years 2 = ≥ 30 years [_____|_____]	**Health center in parish** 1 = Yes 2 = No [_____|_____]
**Date:** [____|____] / [____|____] / [____|____] day month year	**Time start** [_____|_____] : [_____|_____] **Time end** [_____|_____] : [_____|_____]
**1. Meeting place description: detail and description, e.g. location, size and accessibility, and how this could affect the discussion; interruptions during the discussion**	
**2. Participant seating diagram:**	
**3. Group dynamics: general description – level of participation, dominant and passive participants, interest level, boredom, anxiety – and how these relate to the different topics discussed**	
**4. Impressions and observations:**	
**5. Notes of comments provided AFTER the discussion is over (Include additional sheets if necessary):**	

PART 5: CONTACT SUMMARY FORM (1)

**FGD ID number** [____|____|____]	**Moderator initials** [____|____|____]
**Sub-county code** [____]	**Note-taker initials** [____|____|____]
**FGD type** 1 = Primary caregiver 2 = Heads of household [____|____]	**Gender of participants** 1 = Male 2 = Female [____|____]
**Age of participants** 1 = < 30 years 2 = ≥ 30 years [____|____]	**Health center in parish** 1 = Yes 2 = No [____|____]
**Date:** [____|____] / [____|____] / [____|____] day month year	**Time start** [____|____] : [____|____] **Time end** [____|____] : [____|____]

1. What were the main issues or points made by participants during this focus group?

PART 5: CONTACT SUMMARY FORM (2)

2. What new information did you gain through this focus group compared to previous focus groups in this study?

3. Was there anything surprising to you personally? Or that made you think differently about this research question?

4. What messages did you take from this interview for intervention design?

PART 5: CONTACT SUMMARY FORM (3)

5. How would you describe the general atmosphere and engagement of the focus group?

6. How would you describe the group dynamics? For example, were there dominant individuals (what was the result and what were their IDNOs)? Did all participants contribute? Did you feel there was pressure to adhere to dominant viewpoints (what topics)?

7. What else was important about this focus group?

8. Were there any problems with the topic guide (e.g. wording, order of topics, missing topics) you experienced in this focus group?

APPENDIX Y: FGD DATA COLLECTION TOOL

Primary caregivers

PART 1: PARTICIPANT DETAILS

Record the demographic details for each participant using the primary caregiver or heads of household FGD participant log as appropriate.

PART 2: FGD INTRODUCTION

**Subcounty ID**		[_____]	**Moderator initials**		[_____|_____|_____]
**FGD ID number**		[_____|_____|_____]	**Note-taker initials**		[_____|_____|_____]
**Age of participants**	1 = < 30 years<br>2 = > 30 years	[_____|_____]	**Gender of participants**	1 = Male<br>2 = Female	[_____|_____]
**HFI or Standard care**	1 = HFI<br>2 = Standard care	[_____|_____]	**Health center in parish**	1 = Yes<br>2 = No	[_____|_____]
**Date:**	[____|____] / [____|____] / [____|____]<br>day month year		**Time start**<br>**Time end**		[_____|_____] : [_____|_____]<br>[_____|_____] : [_____|_____]

Introduction

I am ______________________________ from ______________________ (moderator)
I am ______________________________ from ______________________ (note-taker)

- *Hello. My name is.... and I work for UMSP (Uganda Malaria Surveillance Project)/ IDRC (Infectious Diseases Research Collaboration).*
- *Thank you very much for coming today. I am part of a team who are doing research to learn more about how people in Tororo treat illnesses in children and their experiences with community medicine distributors and health centers. We would like to understand more about the situation of people in communities like yours in terms of options for seeking treatment for children when they are sick. In addition to our discussion with you today, we are talking with other mothers, heads of households, and health care workers in this district.*
- *Today we would like you to take part in a discussion about treating sick children and your experiences with different places you seek treatment. We have invited you to participate because you have experience with this here and we hope you will tell us the real situation from your point of view.*
- *The discussion will last for about one hour. We will take notes of the ideas discussed and, if you agree, a tape recording will be made of the discussion in order that we record what you say accurately. When the tape is on, the light will be red. If you wish to say anything 'off the record' this is fine, please indicate to the moderator or note taker. The audiotape will only be used by the study team: no one else will hear your voice. It will be kept for 2 years for our records and will then be erased or destroyed. We are not writing down your names here and no one will be able to identify you in any reports arising out of this research. All records of this discussion will be kept securely.*
- *Does anyone have any questions?*
- *Let us begin by setting some ground rules.*
  - ✓ Ground rules set by group, e.g.
    - Only one person talks at a time.
    - Speak clearly
    - It is important for us to hear everyone's ideas and opinions. There is no right or wrong answers to questions – just ideas, experiences and opinions, which are all valuable.
    - It is important for us to hear all sides of an issue – the positive and the negative.
    - Confidentiality is assured. "What is shared in the room stays in the room."
    - TURN OFF MOBILE PHONES
  - ✓ Consent
  - ✓ Ask group to introduce themselves using first names and their role and health centre
  - ✓ Demographic details – please only use each others' first name for discussion

PART 3: FGD TOPIC GUIDES (1)

Domains, topic questions, and probes: Use the table below to help you administer the questions during the interview.

'Now I am going to introduce some topics one at a time about your experiences when your children are unwell, and I hope you can discuss them together.'

Domain	Topic and Probes
**1. Common illnesses in children < 15 years**	a) What illnesses have been common in children under 5 years here for the last few months? *(Make a list, don't spend too long on this question)*
	b) What illnesses have been common in children aged between 5 and 15 years here in the last few months?
**2. Sources of treatment and provider roles**	a) In your experience, what sources have been most successful at treating these different illnesses? *(Start with malaria. Probe for different medicine, provider, and treatment types)*
	b) What is it about each of these different sources of treatment that is important to you? (*e.g. cost, expertise, interpersonal skills, etc. Probe for each source listed and for why different things are important for different illnesses)*
**3. Significant events**	a) Looking back over the past three months what do you think was the most significant change in the way you managed illness in your household?
	b) Why is this significant to you?
	c) What difference has this made now or will it make in the future?

PART 3: FGD TOPIC GUIDES (2)

Domains, topic questions, and probes: Use the table below to help you administer the questions during the interview.

Domain	Topic and Probes
**4. Use of CHWs**	a) What have been your experiences with Community health workers (CHWs) here?
	b) What role have the CHWs played in health care in this area?
**5. Use of health centres**	a) When do you feel it is necessary to go to a health centre with a child? *(Probe for specific illnesses and stages of illness, probe for examples)*
	b) If you feel that it is necessary to go, is it always possible to go? If not, what are the reasons that you don't go to the health centre? *(Probe for examples and stories)*
**6. Experience with health centres**	a) Which is the nearest health HC to your home? Probe: how long do you travel to reach the health centre?)
	b) How many of you have been to the health centre nearest to your home? Can you tell me about your experience when you have gone there with a sick child? *(Probe: What did you go there for, and what happened?)*
	c) Can you tell me about any really good experiences you have had at that health centre? *(Probe: What was it about that experience that made you feel satisfied?)*
**7. Change at Health centres**	a) What has been the most significant change at your nearest health centre? (Probe: what do you think brought the change?)

PART 3: FGD TOPIC GUIDES (3)	
**Domains, topic questions, and probes:** Use the table below to help you administer the questions during the interview.	
**Domain**	**Topic and Probes**
	b) What do these changes mean for you when you have a sick child?
	c) What improvements would you like to see at your nearest health centre?
**8. Closing**	We are now approaching the end of our discussion. Is there anything else anyone would like to add about the kind of diagnosis and treatment you get from health centres or CMDs that we have not talked about?
✓ **Summarize main points made by the participants;**<br>✓ **Thank participants**	

PART 4: NOTE-TAKER FORM

**FGD ID number**		[_____|_____|_____]	**Moderator initials**		[_____|_____|_____]
**Sub-county code**		[_____]	**Note-taker initials**		[_____|_____|_____]
**FGD type**	1 = Primary caregiver<br>2 = Heads of household	[_____|_____]	**Gender of participants**	1 = Male<br>2 = Female	[_____|_____]
**Age of participants**	1 = < 30 years<br>2 = ≥ 30 years	[_____|_____]	**Health center in parish**	1 = Yes<br>2 = No	[_____|_____]
**Date:**	[____|____] / [____|____] / [____|____]<br>day month year		**Time start**<br>**Time end**		[_____|_____] : [_____|_____]<br>[_____|_____] : [_____|_____]

1. Meeting place description: detail and description, e.g. location, size and accessibility, and how this could affect the discussion; interruptions during the discussion

2. Participant seating diagram:

3. Group dynamics: general description – level of participation, dominant and passive participants, interest level, boredom, anxiety – and how these relate to the different topics discussed

4. Impressions and observations:

5. Notes of comments provided AFTER the discussion is over (Include additional sheets if necessary):

PART 5: CONTACT SUMMARY FORM (1)

**FGD ID number** [_____|_____|_____]	**Moderator initials** [_____|_____|_____]
**Sub-county code** [_____]	**Note-taker initials** [_____|_____|_____]
**FGD type** 1 = Primary caregiver<br>2 = Heads of household [_____|_____]	**Gender of participants** 1 = Male<br>2 = Female [_____|_____]
**Age of participants** 1 = < 30 years<br>2 = ≥ 30 years [_____|_____]	**Health center in parish** 1 = Yes<br>2 = No [_____|_____]
**Date:** [____|____] / [____|____] / [____|____]<br>day month year	**Time start** [_____|_____] : [_____|_____]<br>**Time end** [_____|_____] : [_____|_____]

1. What were the main issues or points made by participants during this focus group?

PART 5: CONTACT SUMMARY FORM (2)

2. What new information did you gain through this focus group compared to previous focus groups in this study?

3. Was there anything surprising to you personally? Or that made you think differently about this research question?

4. What messages did you take from this interview for intervention design?

PART 5: CONTACT SUMMARY FORM (3)

5. How would you describe the general atmosphere and engagement of the focus group?

6. How would you describe the group dynamics? For example, were there dominant individuals (what was the result and what were their IDNOs)? Did all participants contribute? Did you feel there was pressure to adhere to dominant viewpoints (what topics)?

7. What else was important about this focus group?

8. Were there any problems with the topic guide (e.g. wording, order of topics, missing topics) you experienced in this focus group?

APPENDIX Z: STRUCTURED CONTEXTUAL RECORD

Staff ID	Date completed
[____|____]	[____|____] / [____|____] / [____|____]<br>day month year

TIME PERIOD COVERED:
1 = Baseline 2 = 0-3 months 3 = 4-6 months
4 = 7-9 months 5 = 10-12 months 6 = 13-15 months
7 = 16-18 months 8 = 19-21 months 9 = 21-24 months
[____|____]

SECTION 1: MALARIA CONTROL INTERVENTION COVERAGE

INDOOR RESIDUAL SPRAYING (IRS)

1. Has IRS been conducted in the study area in the last 3 months? 1 = Yes 88 = Don't know 2 = No [_____|_____]

If YES, go to Qn 2, otherwise skip to Qn 4

2. What is the source of the information? 1 = MoH 77 = Other 2 = USAID/PMI [_____|_____]

Location	Dates of spraying	Formulation<br>1=ICON CS; 2=DDT<br>77=Other	Total population	Proportion of households sprayed
3a.		[____|____]		[_____|_____] %
3b.		[____|____]		[_____|_____] %
3c.		[____|____]		[_____|_____] %
3d.		[____|____]		[_____|_____] %
3e.		[____|____]		[_____|_____] %

INSECTICIDE-TREATED BEDNETS (ITNs)

Indicator	Numerators (N) & Denominators (D)	Proportion or mean
**4. Proportion of households with at least one bednet**	N=[_____|_____|_____|_____|_____|_____]<br>D=[_____|_____|_____|_____|_____|_____]	[_____|_____] %
**5. Proportion of households with at least one ITN**	N=[_____|_____|_____|_____|_____|_____]<br>D=[_____|_____|_____|_____|_____|_____]	[_____|_____] %
**6. Mean number of nets per household**	D=[_____|_____|_____|_____|_____|_____]	[_____|_____]•[_____]
**7. Mean number of ITNs per household**	D=[_____|_____|_____|_____|_____|_____]	[_____|_____]•[_____]
**8. Proportion of children under five who slept under any net the prior night**	N=[_____|_____|_____|_____|_____|_____]<br>D=[_____|_____|_____|_____|_____|_____]	[_____|_____] %
**9. Proportion of children under five who slept under an ITN the prior night**	N=[_____|_____|_____|_____|_____|_____]<br>D=[_____|_____|_____|_____|_____|_____]	[_____|_____] %

ARTEMISININ-BASED COMBINATION THERAPY

Indicator	Numerators (N) & Denominators (D)	Proportion
**10. Proportion of febrile episodes in children treated with an ACT**	N=[_____|_____|_____|_____|_____|_____]<br>D=[_____|_____|_____|_____|_____|_____]	[_____|_____] %
**11. Proportion of malaria cases treated with an ACT**	N=[_____|_____|_____|_____|_____|_____]<br>D=[_____|_____|_____|_____|_____|_____]	[_____|_____] %

STRUCTURED CONTEXTUAL RECORD (2)

Staff ID	Date completed
[____|____]	[____|____] / [____|____] / [____|____]<br>day month year

SECTION 2: HEALTH FACILITY LEVEL

Health facility codes:

1 = Maundo	6 = Nawire	11 = Petta	16 = Mwelo
2 = Were	7 = Kirewa	12 = Makawari	17 = Lwala
3 = Katajula	8 = Chawolo Kirewa	13 = Mbula	18 = Panyangasi
4 = Paya	9 = Kisoko	14 = Gwaragwara	19 = Mudodo
5 = Pusere	10 = Morkiswa	15 = Osia	20 = Chawolo Mulanda

STAFFING

1. Have there been any changes to health center STAFF in the last 3 months? 1 = Yes 88 = Don't know 2 = No [_____|_____]

If YES, go to Qn 2, otherwise skip to Qn 3.

Health center code	HW position	Change	Source of information
	1=In-charge; 2 = Nursing aide/asst; 3 = Volunteer; 77 = Other (describe)	1=Joined HC; 2 = Left HC 3 = On leave/away	1 = HC in-charge; 2 = District; 3 = MoH ; 77 = Other
**2a.** [_____|_____]	[_____|_____] ________________________	[____]	[_____|_____]
**2b.** [_____|_____]	[_____|_____] ________________________	[____]	[_____|_____]
**2c.** [_____|_____]	[_____|_____] ________________________	[____]	[_____|_____]
**2d.** [_____|_____]	[_____|_____] ________________________	[____]	[_____|_____]
**2e.** [_____|_____]	[_____|_____] ________________________	[____]	[_____|_____]

INTERVENTIONS

3. Have any new INTERVENTIONS been introduced at the health centers in the last 3 months? 1 = Yes 88 = Don't know 2 = No [_____|_____]

If YES, go to Qn 4, otherwise skip to Qn 5.

Health center code	Name of program/donor	Intervention	Source of information
			1 = HC in-charge; 2 = District; 3 = MoH ; 77 = Other
**4a.** [_____|_____]			[_____|_____]
**4b.** [_____|_____]			[_____|_____]
**4c.** [_____|_____]			[_____|_____]
**4d.** [_____|_____]			[_____|_____]
**4e.** [_____|_____]			[_____|_____]

STRUCTURED CONTEXTUAL RECORD (3)

Staff ID	Date completed
[____|____]	[____|____] / [____|____] / [____|____]<br>day month year

SECTION 2 cont: HEALTH FACILITY LEVEL

Health facility codes:

1 = Maundo	6 = Nawire	11 = Petta	16 = Mwelo
2 = Were	7 = Kirewa	12 = Makawari	17 = Lwala
3 = Katajula	8 = Chawolo Kirewa	13 = Mbula	18 = Panyangasi
4 = Paya	9 = Kisoko	14 = Gwaragwara	19 = Mudodo
5 = Pusere	10 = Morkiswa	15 = Osia	20 = Chawolo Mulanda

TRAINING PROGRAMS

5. Have any new TRAINING PROGRAMS been introduced at the health centers in the last 3 months?

1 = Yes 88 = Don't know
2 = No

[_____|_____]

If YES, go to Qn 6, otherwise skip to Qn 7.

Health center code	HWs participating	Description of training	Source of information
			1 = HC in-charge; 2 = District; 3 = MoH ; 77 = Other
6a. [_____|_____]			[_____|_____]
6b. [_____|_____]			[_____|_____]
6c. [_____|_____]			[_____|_____]

RESEARCH PROGRAMS INVOLVING HEALTH CENTERS OR STAFF

7. Have any new RESEARCH PROGRAMS been introduced at the health centers in the last 3 months?

1 = Yes 88 = Don't know
2 = No

[_____|_____]

If YES, go to Qn 8, otherwise skip to next section.

Health center code	Name of research group	Project details	Source of information
			1 = HC in-charge; 2 = District; 3 = MoH ; 77 = Other
8a. [_____|_____]			[_____|_____]
8b. [_____|_____]			[_____|_____]
8c. [_____|_____]			[_____|_____]

STRUCTURED CONTEXTUAL RECORD (4)

Staff ID	Date completed
[____|____]	[____|____] / [____|____] / [____|____]<br>day month year

SECTION 3: COMMUNITY LEVEL

MALARIA-RELATED HEALTH PROGRAMS

9. Have any new health programs related to MALARIA been introduced in the community in the last 3 months? 1 = Yes 88 = Don't know 2 = No **[_____|_____]**

If YES, go to Qn 10, otherwise skip to Qn 11.

Sub-county code	Area involved	Summary of program	Source of information
	List parishes, or villages, if known		1 = HC in-charge; 2 = District; 3 = MoH ; 77 = Other
**10a.** [_____]			[____|____]
**10b.** [_____]			[____|____]
**10c.** [_____]			[____|____]
**10d.** [_____]			[____|____]
**10e.** [_____]			[____|____]

NON-MALARIA HEALTH PROGRAMS

11. Have any new health programs that are not related to malaria been introduced in the community in the last 3 months? 1 = Yes 88 = Don't know 2 = No **[_____|_____]**

If YES, go to Qn 12, otherwise skip to Qn 13.

Sub-county code	Area involved	Summary of program	Source of information
	List parishes, or villages, if known		1 = HC in-charge; 2 = District; 3 = MoH ; 77 = Other
**12a.** [_____]			[____|____]
**12b.** [_____]			[____|____]
**12c.** [_____]			[____|____]
**12d.** [_____]			[____|____]
**12e.** [_____]			[____|____]

STRUCTURED CONTEXTUAL RECORD (5)

Staff ID	Date completed
[____|____]	[____|____] / [____|____] / [____|____]<br>day month year

SECTION 3 cont: HEALTH FACILITY LEVEL

CHW TRAINING PROGRAMS

13. Have any new TRAINING PROGRAMS been introduced for community health workers in the last 3 months? 1 = Yes 88 = Don't know 2 = No [_____|_____]

If YES, go to Qn 14, otherwise skip to Qn 15.

Sub-county code	Area involved	Summary of program	Source of information
	List parishes, or villages, if known		1 = HC in-charge; 2 = District; 3 = MoH ; 77 = Other
**14a.** [_____]			[____|____]
**14b.** [_____]			[____|____]
**14c.** [_____]			[____|____]
**14d.** [_____]			[____|____]
**14e.** [_____]			[____|____]

RESEARCH PROGRAMS INVOLVING COMMUNITIES

15. Have any new RESEARCH PROGRAMS been introduced at the community level in the last 3 months? 1 = Yes 88 = Don't know 2 = No [_____|_____]

If YES, go to Qn 16, otherwise skip to next section.

Sub-county code	Area involved	Research group	Project details	Source of information
	List parishes, or villages, if known			1 = HC in-charge; 2 = District; 3 = MoH ; 77 = Other
**16a.** [_____]				[____|____]
**16b.** [_____]				[____|____]
**16c.** [_____]				[____|____]

STRUCTURED CONTEXTUAL RECORD (6)

Staff ID	Date completed
[____|____]	[____|____] / [____|____] / [____|____]<br>day month year

SECTION 4: DISTRICT and HEALTH SUB-DISTRICT LEVEL

STAFFING

17. Have there been any changes to key district or health sub-district STAFF in the last 3 months? 1 = Yes 88 = Don't know 2 = No **[_____|_____]**

If YES, go to Qn 18, otherwise skip to Qn 19.

Level	Position	Change	Source of information
1 = District;<br>2 = Sub-district		1=Joined; 2 = Left<br>3 = On leave/away	1 = HC in-charge; 2 = District;<br>3 = MoH ; 77 = Other
**18a.** [____]		[____]	[____|____]
**18b.** [____]		[____]	[____|____]
**18c.** [____]		[____]	[____|____]
**18d.** [____]		[____]	[____|____]
**18e.** [____]		[____]	[____|____]

DISTRICT & HEALTH SUB-DISTRICT POLICIES

19. Have any new policies been introduced at the district or health sub-district level in the last 3 months that would affect health centers, CHWs, or management of malaria? 1 = Yes 88 = Don't know 2 = No **[_____|_____]**

If YES, go to Qn 20, otherwise skip to Qn 21.

Level	Area involved	Summary of policy	Source of information
1 = District;<br>2 = Sub-district	List parishes, or villages, if known		1 = HC in-charge; 2 = District;<br>3 = MoH ; 77 = Other
**20a.** [____]			[____|____]
**20b.** [____]			[____|____]
**20c.** [____]			[____|____]

STRUCTURED CONTEXTUAL RECORD (7)

Staff ID	Date completed
[____|____]	[____|____] / [____|____] / [____|____]<br>day month year

SECTION 4: DISTRICT and HEALTH SUB-DISTRICT LEVEL

MESSAGES AND NEWS

21. Have there been any important messages or news stories about health centers, health workers, CHWs, malaria, malaria diagnosis or treatment in the radio/TV/newspapers in the last 3 months?

If YES, go to Qn 22, otherwise skip to Qn 23.

1 = Yes 88 = Don't know
2 = No

[_____|_____]

Description of story	Date of story	Source of information
	dd/mm/yyy	1 = Radio; 2 = TV;<br>3 =Newspaper; 77 = Other
**22a.**		[____|____]
**22b.**		[____|____]
**22c.**		[____|____]

ENVIRONMENTAL CHANGES

23. Have there been any significant environmental changes (e.g. changes in malaria transmission or new roads) in the last 3 months?

If YES, go to Qn 24, otherwise skip to next section.

1 = Yes 88 = Don't know
2 = No

[_____|_____]

Description of change	Date of change	Source of information
	dd/mm/yyy	1 = Radio; 2 = TV;<br>3 =Newspaper; 77 = Other
**24a.**		[____|____]
**24b.**		[____|____]
**24c.**		[____|____]

STRUCTURED CONTEXTUAL RECORD (8)

Staff ID	Date completed
[____|____]	[____|____] / [____|____] / [____|____]<br>day month year

SECTION 5: BROADER CONTEXT

MANAGEMENT GUIDELINES

25. Have there been any important changes in guidelines for health centers, health workers, CHWs, or malaria diagnosis or treatment in the last 3 months?

1 = Yes 88 = Don't know
2 = No

[_____|_____]

If YES, go to Qn 26, otherwise skip to Qn 27.

Description of guideline	Date announced	Source of information
	dd/mm/yyy	1 = HC in-charge; 2 = District; 3 = MoH ; 77 = Other
**26a.**		[____|____]
**26b.**		[____|____]
**26c.**		[____|____]

ECONOMIC AND POLITICAL FACTORS

27. Have there been any significant changes in economic or political factors in the last 3 months?

1 = Yes 88 = Don't know
2 = No

[_____|_____]

If YES, go to Qn 28, otherwise skip to END.

Description of factors and changes	Date of change	Source of information
	dd/mm/yyy	1 = Radio; 2 = TV; 3 =Newspaper; 77 = Other
**28a.**		[____|____]
**28b.**		[____|____]
**28c.**		[____|____]

THE REPUBLIC OF UGANDA

APPENDIX AA. INFORMATION SHEET
Surveillance of artemether-lumefantrine and rapid diagnostic tests

Introduction

Dr. Sarah Staedke and colleagues from the Uganda Malaria Surveillance Project/Infectious Diseases Research Collaboration are doing a study to evaluate activities that have been introduced at government-run health facilities in this area aiming to improve the health of children. We would like to understand the impact of these activities and why they are working, or not.

Why is this surveillance being done?

As part of this study, we would like to know more about the supply of malaria drugs (artemether-lumefantrine [Coartem or Lumartem]) and rapid diagnostic tests for malaria. We would also like to know more about the delivery of these supplies, any stock-outs, and the process of ordering for re-supply.

What will happen if I agree to take part in this surveillance?

We will ask you, or another available health worker, to provide us with your drug stock cards and requisition and issue vouchers. We will review the stock cards and vouchers and enter the data into a questionnaire. We estimate that it will take less than one day to enter the data. The information we collect will be used by project investigators and may be shared with other researchers and policy-makers to answer questions about how best to deliver health services.

How long will this surveillance last?

We plan to conduct the surveillance over about 1 ½ years. We will visit your health facility about once every one or two months to collect the information.

Can I stop being in the surveillance?

You can decide to stop participating at any time. Just tell our study personnel right away if you wish to stop the activities.

What risks can I expect from participating in the surveillance?

Participation in any research study may involve a loss of privacy. Information you provide about your health center will be recorded, but your name will not be used in any reports of the information provided. No quotes or other results arising from your participation in this study will be included in any reports, even anonymously, without your agreement. The information obtained from these surveillance activities will be locked at our project offices. We will do our best to make sure that any personal information is kept private.

THE REPUBLIC OF UGANDA

Are there benefits to taking part in the surveillance?

There will be no direct benefit to you from participating in this study. However, the information that you provide will help researchers and policy-makers understand how best to improve health services in this area.

What other choices do I have if I do not take part in the surveillance?

You are free to choose not to participate in the study. If you decide not to take part, there will be no penalty to you.

What are the costs of taking part in the surveillance? Will I be paid for taking part in the surveillance?

There are no costs to you for taking part in this study. You will not be paid for taking part in this study.

What are my rights if I take part in the surveillance?

Taking part in this study is your choice. You may choose either to take part or not to take part. If you decide to take part in this study, you may change your mind at any time. No matter what decision you take, there will be no penalty to you in any way.

Who can answer my questions about the surveillance?

You can talk to the researchers about any questions or concerns you have about these surveillance activities. Contact Dr. Sarah Staedke or other members of the Uganda Malaria Surveillance Project / Infectious Disease Research Collaboration on telephone number 0414-530692. If you have any questions, comments or concerns about taking part in these activities, first talk to the researchers. If for any reason you do not wish to do this, or you still have concerns about doing so, you may contact Dr Charles Ibingira, Makerere University Faculty of Medicine Research and Ethical Committee at telephone number 0414-530020.

Giving verbal consent to participate in the surveillance:

You may keep this information sheet if you wish. Participation in these activities is voluntary. You have the right to decline to participate in the activities, or to withdraw from them at any point without penalty. If you do not wish to participate in the activities, you should inform the researcher now. If you do wish to participate in these activities, you should tell the researcher now, and the interview will begin shortly. If you do not agree to quotes or other results arising from your participation in the study being included, even anonymously, in any reports about the study, please tell the researcher now.

APPENDIX BB: AL & RDT SURVEILLANCE DATA COLLECTION SHEET (1)

Health centre code	Staff ID	Data for the month of	Data Collection for (list AL package or RDT)
[____|____]	[____|____|____|____]	[____|____] / [____|____]<br>month year	1 = AL 6 tab pack (yellow)<br>2 = AL 12 tab pack (blue)<br>3 = AL 18 tab pack (green)<br>4 = AL 24 tab pack (brown)<br>5 = AL 24 tab pack (white)<br>6 = RDT<br>[____]

PART 1: STOCK CARD

Average Monthly consumption [____|____|____]	Minimum stock level [____|____|____]	Maximum stock level [____|____|____]

**Date card updated**	[____/____]<br>dd mm	[____/____]<br>dd mm	[____/____]<br>dd mm	[____/____]<br>dd mm	[____/____]<br>dd mm	[____/____]<br>dd mm
**Recorded Balance on hand**	[____|____|____]	[____|____|____]	[____|____|____]	[____|____|____]	[____|____|____]	[____|____|____]
**Losses / Adjustments**	[____]<br>1 = no issues<br>2 = unexplained loss<br>3 = explained loss<br>4 = explained adjustment<br>List details:	[____]<br>1 = no issues<br>2 = unexplained loss<br>3 = explained loss<br>4 = explained adjustment<br>List details:	[____]<br>1 = no issues<br>2 = unexplained loss<br>3 = explained loss<br>4 = explained adjustment<br>List details:	[____]<br>1 = no issues<br>2 = unexplained loss<br>3 = explained loss<br>4 = explained adjustment<br>List details:	[____]<br>1 = no issues<br>2 = unexplained loss<br>3 = explained loss<br>4 = explained adjustment<br>List details:	[____]<br>1 = no issues<br>2 = unexplained loss<br>3 = explained loss<br>4 = explained adjustment<br>List details:

Continue on a new page if stock card was completed more than 6 times in the month

PART 2: REQUISITION & ISSUE VOUCHER

**Order placed date**	[____/____]<br>dd mm	[____/____]<br>dd mm	[____/____]<br>dd mm	[____/____]<br>dd mm	[____/____]<br>dd mm	[____/____]<br>dd mm
**Balance on hand**	[____|____|____]	[____|____|____]	[____|____|____]	[____|____|____]	[____|____|____]	[____|____|____]
**Quantity requested**	[____|____|____]	[____|____|____]	[____|____|____]	[____|____|____]	[____|____|____]	[____|____|____]
**Order received date**	[____/____]<br>dd mm	[____/____]<br>dd mm	[____/____]<br>dd mm	[____/____]<br>dd mm	[____/____]<br>dd mm	[____/____]<br>dd mm
**Quantity received**	[____|____|____]	[____|____|____]	[____|____|____]	[____|____|____]	[____|____|____]	[____|____|____]

APPENDIX AA: AL & RDT SURVEILLANCE DATA COLLECTION SHEET (2)

Health centre code	Staff ID	Data for the month of	Data Collection for (list AL package or RDT)
[____|____]	[____|____|____|____]	[____|____] / [____|____]<br>month year	

PART 3: ADDITIONAL INFORMATION

Record any other comments or observations:

Stock card	Requisition & Issue Voucher
