# Supplementary material for: The impact of an intervention to introduce malaria rapid diagnostic tests on fever case management in a high transmission setting in Uganda: A mixed-methods cluster-randomized trial (PRIME)
Source: PLoS One. 2017 Mar 13;12(3):e0170998. doi: 10.1371/journal.pone.0170998 (PMC5347994; doi:10.1371/journal.pone.0170998)
Supplement: S4 File — Final version of the PROCESS protocol. This file is a copy of the final protocol of the PROCESS study which ran alongside the PRIME cluster randomized trial, incorporating all amendments approved by the overseeing ethics committees. (PDF) [file pone.0170998.s004.pdf]

The ACT PROCESS Study

Evaluating the process, context, and impact of interventions to enhance health facilities in Tororo, Uganda

15 April 2013
Protocol Version1.3

Principal Investigator: Dr. Sarah Staedke
Co-investigators: Dr. Moses Kamya, Dr. Clare Chandler

Funding Source: Gates Foundation, ACT Consortium, LSHTM
Makerere University Research and Ethical Committee:
Ugandan National Council for Science and Technology:
London School of Hygiene &Tropical Medicine Ethics Committee: 5831

CONTENTS

STUDY INFORMATION **4**
PROJECT SYNOPSIS **5**
PROJECT TEAM AND PARTICIPATING SITES **6**
ABBREVIATIONS AND ACRONYMS **8**

1 BACKGROUND **9**
1.1. Malaria in Uganda 9
1.1.1. Barriers to diagnosing and treating malaria *9*
1.1.2. Improving quality of care through interventions *9*
1.2. The ACT PRIME study 10
1.3. Evaluating interventions 11
1.3.1. Comprehensive evaluation *11*

2 RATIONALE **13**

3 STUDY OBJECTIVES **14**
3.1. Primary objective 14
3.2. Secondary objectives 14

4 COMPREHENSIVE EVALUATION FRAMEWORK **15**
4.1. Overview 15
4.2. Logic model 16
4.3. Process evaluation 17
4.3.1. Documentation of implementation *17*
4.3.2. Assessment of intervention mechanisms *18*
4.4. Context evaluation 18
4.4.1. Structured contextual record *18*
4.4.2. Rich description of context *19*
4.5. Impact evaluation 19
4.5.1. Assessment of hypothesised impacts *19*
4.5.2. Assessment of undetermined impacts *20*

5 STUDY PROCEDURES **21**
5.1. Overview 21
5.2. Study site 22
5.3. Study population 22
5.4. Self-filled questionnaires 23
5.5. Health worker communication assessments and patient exit interviews 24
5.6. In-depth interviews 25
5.7. Semi-structured questionnaires 25
5.8. Focus group discussions 26
5.8.1. Community FGDs *27*
5.9. Structured contextual record 27
5.10. Supply of drugs and RDTs 28

6 DATA MANAGEMENT **29**
6.1. Data management 29
6.1.1. Quantitative data *29*
6.1.2. Qualitative data *29*
6.2. Quality assurance and quality control 30
6.3. Records and storage 30
6.4. Data sharing 31

7 ANALYTICAL PLAN 32
7.1. Quantitative data 32
7.2. Audio recordings 32
7.3. Qualitative data 32

8 PROTECTION OF HUMAN PARTICIPANTS 33
8.1. Institutional Review Boards 33
8.2. Informed consent process 33
8.2.1. Self-filled questionnaires 34
8.2.2. Health worker communication assessments and patient exit interviews 34
8.2.3. In-depth Interviews 34
8.2.4. Semi-structured questionnaires 34
8.2.5. Focus group Discussions 34
8.2.6. Supply of drugs and RDTs 35
8.3. Confidentiality 35
8.4. Risks and discomforts 35
8.4.1. Privacy 35
8.4.2. Compensation 35
8.4.3. Alternatives 36

9 REFERENCES 37

10 APPENDICES 40

STUDY INFORMATION

**Title**	**PROCESS Study – Evaluating the process, context, and impact of interventions to enhance health facilities in Tororo, Uganda**
**Principal investigator**	Sarah Staedke, MD, PhD<br>Clinical Senior Lecturer, London School of Hygiene and Tropical Medicine<br>Uganda Malaria Surveillance Project / Infectious Diseases Research Collaboration, Uganda
**Co-investigators**	Moses Kamya, MBChB, MPH, PhD; Professor, Makerere University<br>Clare Chandler, PhD; Lecturer, London School of Hygiene and Tropical Medicine
**Sponsor**	London School of Hygiene & Tropical Medicine<br>Department of Clinical Research<br>Faculty of Infectious and Tropical Diseases<br>Keppel Street, London WC1E 7HT<br>United Kingdom
**Funding agency**	ACT Consortium through a grant from the Bill & Melinda Gates Foundation to the London School of Hygiene and Tropical Medicine
**Participating institutions**	London School of Hygiene & Tropical Medicine, UK<br>Infectious Diseases Research Collaboration, Kampala, Uganda
**Institutional review boards**	Makerere University Research and Ethics Committee<br>Uganda National Council for Science and Technology<br>London School of Hygiene & Tropical Medicine

PROJECT SYNOPSIS

Title	**PROCESS Study – Evaluating the process, context, and impact of interventions to enhance health facilities in Tororo, Uganda**
**Description**	The study proposed here, ACT PROCESS, is a comprehensive evaluation to further our understanding about the outcomes of the ACT PRIME study in which an intervention will be implemented in lower level government run health centers in Tororo, Uganda. The aim of the PRIME health facility intervention (HFI) is to 1) improve health center management; 2) provide health worker training;and 3) stabilize supplies of drugs and rapid diagnostic tests (RDTs) for malaria.
**Study Design**	ACT PROCESS consists of a comprehensive evaluation framework to evaluate the process, context and impact of the ACT PRIME intervention. The evaluation framework includes: 1) a logic model to detail the components, effects and intended outcomes of the HFI; 2) a process evaluation to document the implementation of the HFI activities from the perspective of implementers, health workers, community members, and key stakeholders; 3) a context evaluation to capture information on factors that may have affected the HFI implementation or outcomes; and 4) an impact evaluation to assess the wider impact of the HFI beyond outcomes of the ACT PRIME study. These evaluation components will be assessed using self-filled questionnaires, health worker communication assessments and patient exit interviews, in-depth interviews and semi-structured questionnaires, focus group discussions, and a structured contextual record.
**Study site**	In Tororo District, the five sub-counties of West Budama North Health Sub-district (Nagongera, Paya, Kirewa, Kisoko, and Petta), and two sub-counties of West Budama South Health Sub-district (Mulanda and Rubongi) will be included.
**Primary objective**	To evaluate the process, context and impact of the HFI in the ACT PRIME study to further our understanding about why the HFI was effective, or not.
**Secondary objectives**	1. To develop a comprehensive logic model of the ACT PRIME HFI with intervention components mapped through to their intended effects and outcomes.<br>2. To evaluate the process of the HFI implementation including health worker training, health center management tools, supply of artemether-lumefantrine (AL) and rapid diagnostic tests (RDTs) for malaria, and interactions with local and district stakeholders.<br>3. To develop a rich contextual record of factors that may have affected the HFI implementation outcomes such as other interventions or programmes implemented in the area, changes to malaria case management guidelines, and other environmental, economic, political or individual factors.<br>4. To assess the wider expected and unexpected impacts of the HFI at the household, community, public health system, and private sector levels.
**Target population**	**Self-filled questionnaires:** all trainers and participants for each HFI training module;up to 350 self-filled questionnaires.<br>**Health worker communication assessments and patient exit interviews:** at least one health worker in the 20 health centers included in ACT PRIME; recording interactions with caregivers at two time points; up to 125 assessments per time point and up to 250 total and conducting patient exit interviews with 3-5 patients per health worker at three time points; up to 125 interviews per time point, and up to 375 total.<br>**In-depth interviews:** selected HFI implementers, health workers in the HFI arm, and key local and district stakeholders; up to25in-depth interviews.<br>**Semi-structured questionnaires:** all health workers in both the HFI and standard care arms, and private drug shops; up to 70 questionnaires.<br>**Focus group discussions:** selected primary caregivers and heads of households from the study area; up to12focus group discussions.
**Study period**	Implemented in parallel with the ACT PRIME study for approximately 1 ½ years.

PROJECT TEAM AND PARTICIPATING SITES

INVESTIGATORS

Sarah Staedke, MD, PhD
Role in project: Principal investigator
Clinical Senior Lecturer, London School of Hygiene and Tropical Medicine, London, UK
Infectious Disease Research Collaboration / Uganda Malaria Surveillance Project, Kampala, Uganda
Email: sarah.staedke@lshtm.ac.uk

Moses Kamya, MBChB, MPH, PhD
Role in project: Co-investigator
Professor, Department of Medicine, Makerere University, Kampala, Uganda
Infectious Disease Research Collaboration / Uganda Malaria Surveillance Project, Kampala, Uganda
Email: mkamya@infocom.co.ug

Clare Chandler, PhD
Role in project: Co-investigator
Senior Lecturer, London School of Hygiene and Tropical Medicine, London, UK
Social Scientist, ACT Consortium
Email: clare.chandler@lshtm.ac.uk

COLLABORATORS

Grant Dorsey, MD, PhD
Role in project: Collaborator
Associate Professor, Department of Medicine, University of California, San Francisco
Infectious Disease Research Collaboration / Uganda Malaria Surveillance Project, Kampala, Uganda
Email: gdorsey@medsfgh.ucsf.edu

Heidi Hopkins, MD, MPH
Role in project: Collaborator
FIND Diagnostics, Kampala, Uganda
Infectious Disease Research Collaboration / Uganda Malaria Surveillance Project, Kampala, Uganda
Email: hhopkins@medsfgh.ucsf.edu; heidi.hopkins@finddiagnostics.org

Anne Gasasira, MBChB, PhD
Role in project: Collaborator
Infectious Disease Research Collaboration / Uganda Malaria Surveillance Project, Kampala, Uganda
Email: agasasira@gmail.com

KEY PROJECT PERSONNEL

Personnel	Institution	Role
Susan Nayiga	Infectious Diseases Research Collaboration, Kampala	Social Scientist / Epidemiologist
Lilian Taaka	Infectious Diseases Research Collaboration, Kampala	Social Scientist
Christine Nabirye	Infectious Diseases Research Collaboration, Kampala	Social Scientist
Deborah DiLiberto	London School of Hygiene & Tropical Medicine, UK	Research Assistant

PARTICIPATING SITES

Uganda Malaria Surveillance Project (UMSP)

Address: Infectious Disease Research Collaboration (IDRC), Mulago Hospital Complex, Malaria House, P.O. Box 7475, Kampala, Uganda
Contact Person: Catherine Tugaineyo
Phone Number: +256 (0) 414-530692
Fax Number: +256 (0) 414-540524
Email: ctugaineyo@muucsf.com

London School of Hygiene & Tropical Medicine (LSHTM)

Address: Keppel Street, London, WC1E 7HT, UK
Contact Person: Susan Sheedy
Phone Number: +44 (0) 20 7927 2256
Fax Number: +44 (0)20 7637 4314
Email: susan.sheedy@lshtm.ac.uk

ABBREVIATIONS AND ACRONYMS

ACT	artemisinin-based combination therapy
AL	artemether-lumefantrine
CHW	community health worker
FGD	focus group discussion
FOMREC	Faculty of Medicine Research Ethics Committee, Makerere University
HFI	health facility intervention
HMM	home management of malaria
HW	health worker
IDI	in-depth interview
IRB	institutional review board
LSHTM	London School of Hygiene and Tropical Medicine
MCPP	Measuring Patient-Centered Communication
MoH	Ministry of Health
MU	Makerere University (Kampala, Uganda)
MU-UCSF	Makerere University - UCSF Malaria Research Collaboration
M&E	monitoring and evaluation
PCS	patient-centeredservices
RDT	rapid diagnostic test (for malaria)
SFQ	self-filled questionnaire
SOP	standard operating procedure
SSQ	semi-structured questionnaire
UMSP	Uganda Malaria Surveillance Project
UNCST	Uganda National Council of Science and Technology
WHO	World Health Organization

1 BACKGROUND

1.1. MALARIA IN UGANDA

Malaria remains one of the most serious global health problems[1]. Of the estimated 400 to 900 million episodes of fever that occur each year in African children, probably about half are due to malaria, resulting in over one million deaths [2-4].In Uganda, malaria is one of the most important health problems and the leading cause of morbidity and mortality in children, accounting for up to 40% of outpatient visits, 20% of hospital admissions, and 14% of inpatient deaths [5]. Children in Uganda experience an estimated average of six episodes of malaria each year, resulting in between 70,000 and 110,000 deaths annually. Up to 90% of Uganda's population lives in highly endemic areas with perennial malaria transmission, while 10% live in areas at risk for epidemics [6].

1.1.1. Barriers to diagnosing and treating malaria

Diagnosis and treatment of malaria can be straight forward; however, it is often challenged by limited health-care infrastructure, particularly in Africa [7-8]. Substantial barriers to providing good quality health care exist, including logistical, cultural, and wider system barriers. As a result, few malaria patients receive treatment in the formal healthcare sector; most are treated at home with drugs purchased from informal drug shops[2, 9].Unfortunately, such treatment is often inadequate, with ineffective or poor quality drugs given at incorrect doses[9-11].Addressing these barriers and providing quality health care for malaria, and other illnesses that is safe, effective, patient-centered, timely, efficient and equitable is a necessity; however, evidence from increasing numbers of studies suggests quality of care by these measures is poor in many settings, including delivery of primary care in low-income countries. Direct observation studies of performance have identified severe deficiencies, particularly in history taking and examinations, diagnosis, and appropriate treatment choice and dosage [12-17]. This has been linked to low motivation of staff as well as poor resource availability in the work place. In terms of patient-centeredness and timeliness, meeting a population's expectations of how they should be treated by providers, including patient expectations for health care, is now seen as central to performance[18]. It has been argued that poor quality services fail to earn the population's trust, leading to clients seeking alternative sources of care[19], or discontinuing care[20]. In contrast, the perception of good quality services, including inter-personal relationships, has been found to encourage patients to access care [21], and demand for services [22-24]. Satisfied patients may be more likely to comply with treatment and maintain a continuing relationship with the health worker[25], and loyalty to a clinic[26], thus enjoying a better medical prognosis (presuming good technical quality of care)[27].

1.1.2. Improving quality of care through interventions

Interventions to improve quality of care in low-resource settings have largely fallen into two categories: resource-based interventions and performance-based interventions. Resource-based interventions include the provision of equipment, infrastructure and drugs. Performance-based interventions have mostly been focused on clinical training and dissemination of guidelines. Far fewer studies have assessed interventions to improve aspects of quality care outside of clinical care. The ACT PRIME study being conducted in Tororo, Uganda, on which the ACT PROCESS study proposed here is based, aims to improve quality of care at lower level government-run health

centers by implementing a health facility intervention which incorporates both resource-based and performance-based components.

1.2. THE ACT PRIME STUDY

In the ACT PRIME study, enhanced health facility care will be compared to the current standard of care provided by lower level government-run health facilities, supplemented by services provided through the private sector and community-based interventions, using a cluster-randomized design. There will be 20 health centers randomized to each study arm: 10 health centers in the health facility intervention arm and 10 health centers in the standard care arm. ACT PRIME began in December 2010, and the intervention will be rolled-out in March 2011. The objectives and outcomes of ACT PRIME are provided in Table 1.1 below.

Table 1.1 ACT PRIME objectives and outcomes

Objective	Primary outcome	Secondary outcomes
1. To compare the impact of enhanced health facility-based care to current standard of care on key population-based indicators in children under five.	Prevalence of anaemia	- Prevalence of parasitemia<br>- Prevalence of gametocytemia<br>- All-cause mortality rate in children under five
2. To compare the impact of enhanced health facility-based care to current standard of care on key longitudinal indicators, in a cohort of children under five.	Antimalarial treatment incidence density	- Incidence of hospitalizations,<br>- Illness and febrile illness episodes<br>- Prompt effective treatment of fever<br>- Prompt effective treatment of malaria<br>- Incidence of serious adverse events
3. To compare impact of enhanced health facility-based care to current standard of care on key indicators of case management for malaria and other illnesses, in children under five treated at health facilities.	Inappropriate treatment of malaria	- Appropriate treatment of malaria, patient satisfaction<br>- Patient attendance, gaps in staffing<br>- Drug stock outs<br>- Health worker knowledge questionnaire scores

The health facility intervention (HFI) will be comprised of three components: 1) health center management training, 2) health worker training, including fever case management and patient-centered services, and 3) supply of consumables, including malaria diagnostics and antimalarial drugs. The goal of these components is to address the barriers to providing good quality care identified in our formative research. By addressing these barriers, ACT PRIME aims to provide good quality care as defined by health workers and community members in Tororo district, attracting them to health facilities and improving the case management of malaria and non-malarial febrile illnesses received when they attend facilities. The intervention package will be rolled out to all health centers randomized to the HFI over approximately 8-10 weeks. Some activities will continue to be supported by the project for the duration of the study. ACT PRIME aims to implement an intervention which is sustainable and reproducible by the MoH in Uganda, working within the existing government systems in conjunction with the MoH and district teams.

This study, ACT PROCESS, is a parallel study intended to comprehensively evaluate the complex interventions of ACT PRIME being conducted in Tororo District, Uganda.

1.3. EVALUATING INTERVENTIONS

Research has shown that simple interventions such as basic training or health education have had limited effect on changing provider behaviour [28-29] or community behaviour[30]. The results from our formative research, the Tororo District Survey Project, echo these findings: the situation of providing and seeking health care whether in health facilities or in communities is far more complex, involving a range of actors, motivations, habits and logistics [31]. Achieving a change in behaviour requires complex interventions that address the multiple factors involved with access to appropriate treatment[32-33].Evaluating the complexities of the intervention using a systematic approach is key to understanding *if*, *how* and *why* the intervention functioned.

Many authors and institutions are now arguing for more comprehensive evaluations of complex interventions that include a focus on process, context and impact[34]. Such comprehensive evaluations have been uncommon, and those that have existed alongside randomized controlled trials have been critiqued for poor integration with quantitative findings and methodological limitations[35], prompting the challenge for more carefully planned evaluations. We adopt a 'realist evaluation' approach to our study: to contribute to broader knowledge of 'What works for whom in what circumstances and in what respects, and how?' [36]. This involves understanding mechanisms of change by mapping out the intended intervention programme and contrasting this with the reality of implementation, analysing local interpretations of intervention effects, mapping and interpreting contextual influences and assessing impact within and outside of intended consequences of the intended intervention.

1.3.1. Comprehensive evaluation

Comprehensive evaluation can be considered in four components illustrated inFigure 1.1.

Figure 1.1 Framework for qualitative evaluation

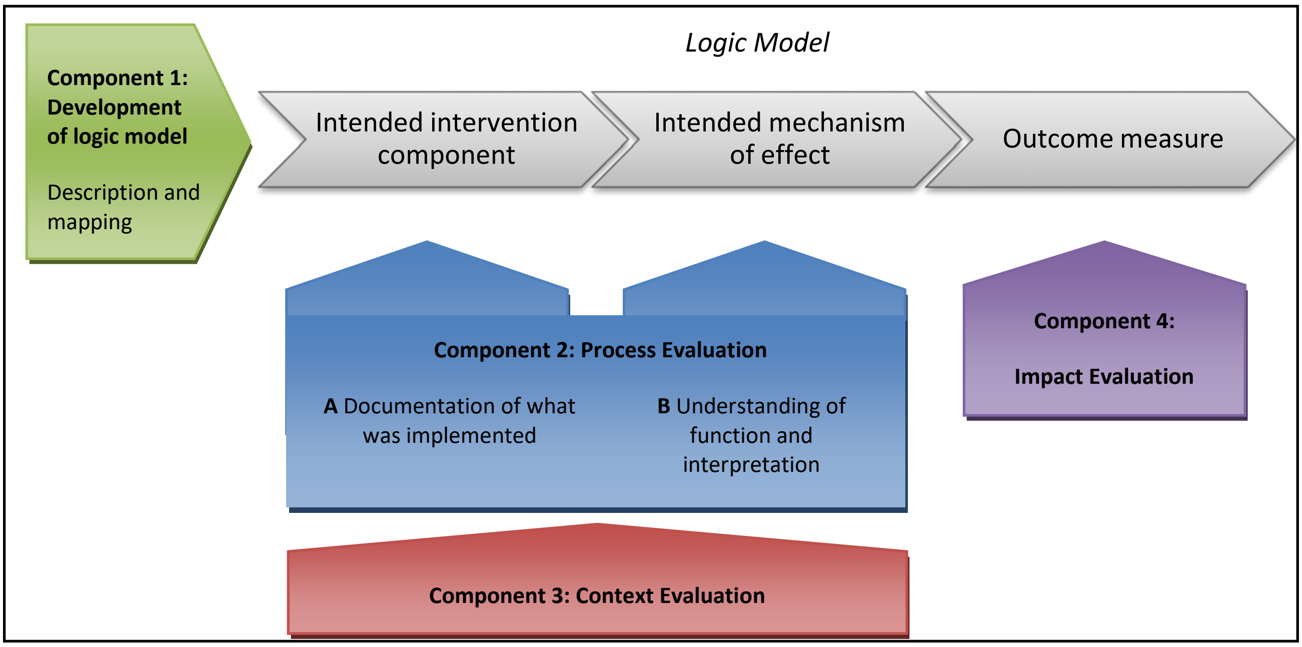

Component 1, *Development of logic model,* maps the intended pathway between intervention activities and outcomes, highlighting the mechanisms by which the intervention is intended to take effect and the assumptions that underlie each mechanism[36]. Once the intended intervention is mapped, it is then possible to identify factors that may explain the study outcomes.

Component 2, *Process Evaluation,* documents (a) how the intervention is implemented in reality, assessing this against the map of the planned intervention[37] and (b) how the intervention activities being implemented are functioning and being perceived, including whether intended mechanisms worked as planned[38].

Component 3, *Context Evaluation,* documents the context of the intervention process both in terms of the reasons that the implementation of the intervention occurs as it does in reality and how the intervention is interpreted and accommodated. The context documentation involves local factors as well as wider factors, including those outside of the PRIME study[39].

Component 4, *Impact Evaluation,* attempts to understand the depth and breadth of the impact of the intervention[40]. Specific outcome measurements are predicted and are measured quantitatively. However, the impact may be more far reaching and is likely to depend upon the way the intervention was implemented, interpretations of the intervention and how it is adopted as well as the local and broader context. It is therefore important that these other components are used to interpret any other outcomes and impacts of the intervention.

2 RATIONALE

The ACT PROCESS study proposed here is designed to evaluate the process, context and impact of the intervention implemented in the ACT PRIME study in order to further our understanding about why the HFI was effective, or not. This will be achieved through a comprehensive evaluation framework implemented in parallel with ACT PRIME.

ACT PROCESS consists of four linked evaluation components including: 1) logic model, 2) process evaluation, 3) context evaluation, 4) impact evaluation. The logic model is developed alongside the HFI intervention design stage and aims to detail the components, effects and intended outcomes of the HFI. The logic model informs the development of the data collection tools for the remaining components of the evaluation. The process evaluation will document the process of implementing the HFI including health worker training activities, health center management tools, supply of artemether-lumefantrine (AL) and rapid diagnostic tests (RDTs) for malaria, and interactions with local and district stakeholders from the perspective of implementers, health workers and community members. The context evaluation will capture information on factors that may have affected the HFI implementation or outcomes including other interventions or programmes implemented in the area, changes to malaria case management guidelines, and other environmental, economic, political or individual factors. The impact evaluation will assess the wider impact of the HFI beyond outcomes of ACT PRIME at the household, community, private sector, and public health system levels.

To facilitate our understanding about why the HFI was effective or not, links will be made between the clinical and economic outcomes of the ACT PRIME study and the process, context and impact outcomes of the ACT PROCESS study. This understanding is essential for interpreting and informing the development of a health facility intervention which is sustainable and reproducible by the MoH in Uganda, and elsewhere.

3 STUDY OBJECTIVES

3.1. PRIMARY OBJECTIVE

To evaluate the process, context and impact of the HFI in the ACT PRIME study in order to further our understanding about why the HFI was effective, or not.

3.2. SECONDARY OBJECTIVES

1. To develop a comprehensive logic model of the ACT PRIME HFI with intervention components mapped through to their intended effects and outcomes.

2. To evaluate the process of the HFI implementation including health worker training, health center management tools, supply of AL and RDTs for malaria, and interactions with local and district stakeholders.

3. To develop a rich contextual record of factors that may have affected the HFI implementation outcomes such as other interventions or programmes implemented in the area, changes to malaria case management guidelines, and other environmental, economic, political or individual factors.

4. To assess the wider expected and unexpected impacts of the HFI at the household, community, private sector, and public health system levels.

4 COMPREHENSIVE EVALUATION FRAMEWORK

4.1. OVERVIEW

Figure 4.1 illustrates the comprehensive evaluation framework for the study proposed here including the process, context and impact evaluation as they relate to the ACT PRIME study HFIs.

Figure 4.1 ACT PROCESS study comprehensive evaluation framework

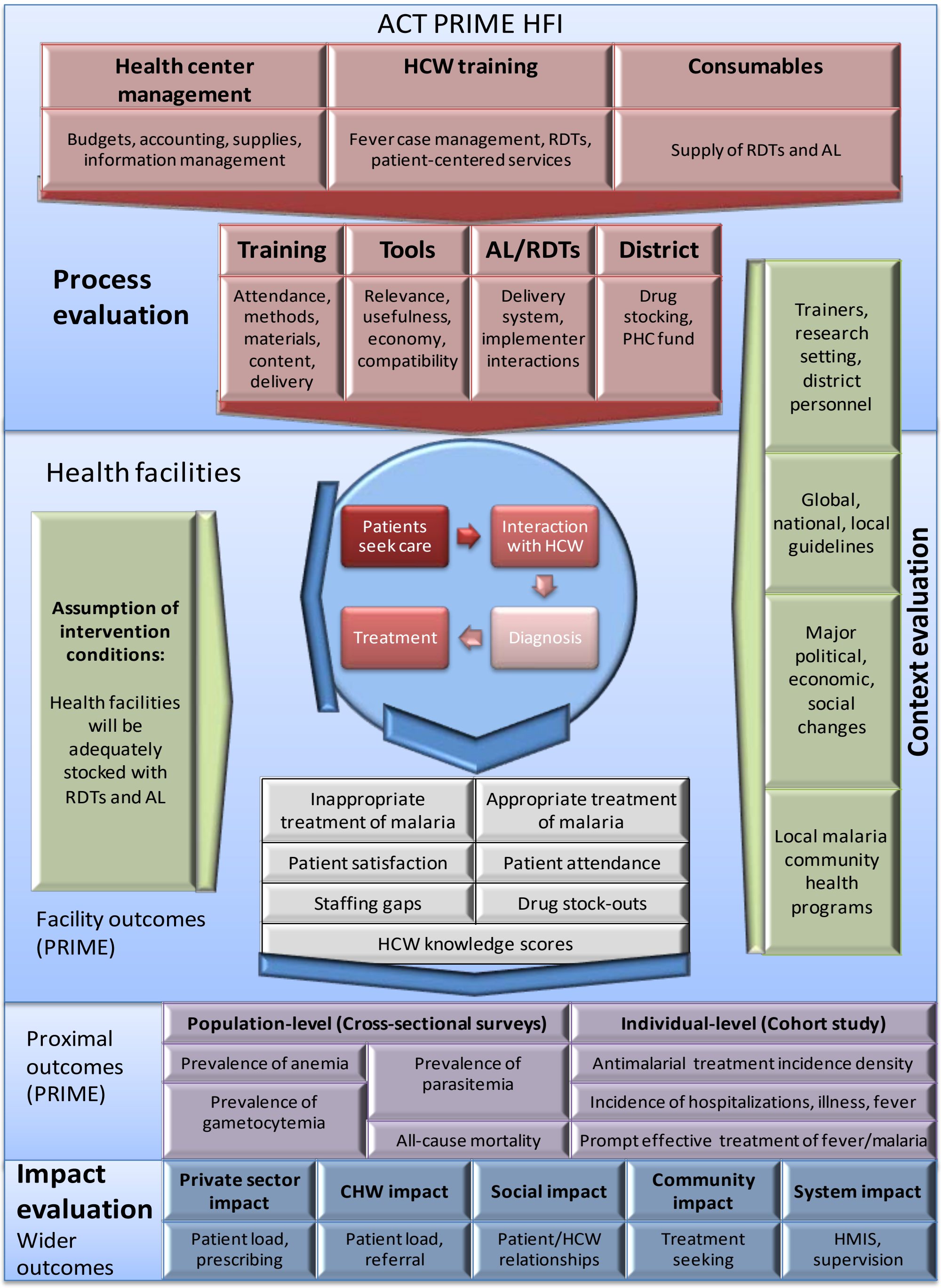

Data for the process, context and impact evaluations will be gathered through self-filled questionnaires, health worker communication assessments, IDIs, FGDs and a structured contextual record as outlined in Table4.1 and described in Chapter 5.

Table 4.1 ACT PROCESS Evaluation methods

Evaluation method	Participants	Evaluation component			Maximum sample size
		Process evaluation	Context evaluation	Impact evaluation	
Self-filled questionnaires	- Trainees<br>- Trainers	X			350
Health worker communication assessments + patient exit interviews	- Caregivers & children under 5<br>- Health workers	X			375
In-depth interviews	- HFI Health workers	X	X	X	10
	- Implementers	X	X	X	5
	- Key stakeholders		X	X	10
Semi-structured questionnaires	- HFI Health workers	X	X	X	30
	- Standard care health workers	X	X	X	30
	- Private drug shops		X	X	10
Focus group discussions	- Primary caregivers	X	X	X	9
	- Heads of households		X	X	3
Structured contextual record	- Completed by study team		X		10

4.2. LOGIC MODEL

An initial logic model is illustrated in Figure 4.2. The logic model will be revised to ensure all components of the interventions are accurately mapped through to their intended effects and outcomes. The process of mapping the intervention started during the design phase for the ACT PRIME HFI. The design phase was a consultative process between investigators and implementers using our formative research, input from stakeholders, evidence from the literature, and behaviour change theory. Using these components, the intended mechanism of effect and the source for the hypothesised mechanism was specified. This informed the development of a logic model which describes in detail the proposed HFI components, mechanisms of effect and intended outcomes. This logic model forms the basis of subsequent evaluation components.

Figure 4.2 Logic model

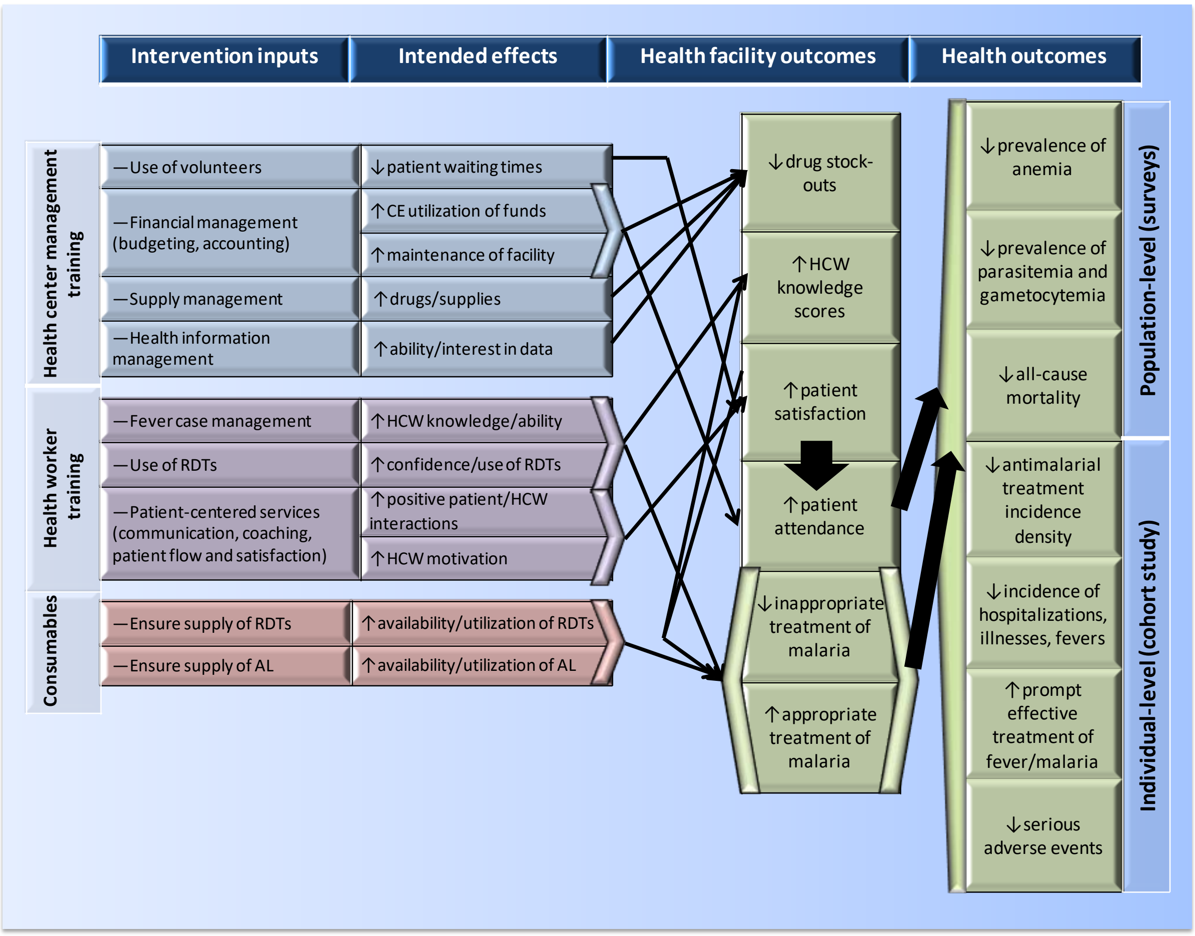

4.3. PROCESS EVALUATION

The process evaluation will involve two lines of research, (1) documentation of the implementation of the intervention as delivered by the study team and (2) qualitative study of the functioning and interpretation of the intervention by implementers and recipients. As outlined in Figure 4.1, we will be evaluating four aspects of the HFI including (1) health worker training modules, (2) health center management tools, (3) supply of AL and RDTs for malaria, and (4) interaction with the district regarding staffing and drug stocks.

4.3.1. Documentation of implementation

The logic model will be used to identify relevant activities for the process evaluation. For each activity, the following evaluation criteria will be used as categories of assessment[37].

- Fidelity (quality)	The extent to which the intervention was implemented as planned
- Dose delivered (completeness)	Amount or number of intended units of each intervention or component delivered or provided by interventionists
- Dose received (exposure)	Extent to which participants actively engage with, interact with, are receptive to and/or use materials or recommended resources. Can include ‘initial use’ and ‘continued use’

-	Reach (participation rate)	Proportion of the intended priority audience that participates in the intervention; often measured by attendance; includes documentation of barriers to participation
-	Recruitment	Procedures used to approach and attract participants at individual or organizational levels; includes maintenance of participant involvement in intervention
-	Context	Aspects of the environment that may influence intervention implementation or study outcomes; includes contamination

Variables for data collection for each activity are formulated under each of the above headings by analysing the materials used for the HFI implementation including:

- Training packages: Health center management training; Training in fever case management and use of RDTs, Training in patient-centered services.
- Management tools: PHC Fund Accounting Tool, ACT Drug Distribution Assessment Tool
- Supply of AL and RDTs: drug stock cards, requisition and issue vouchers from health centers and the health sub-district records
- District and health sub-district interactions: Logs documenting interactions with district, health sub-district, and health center staff.

We will capture information on these variables during the implementation and monitoring and evaluation of the HFI using monthly health center records, health worker training evaluation self-filled questionnaires, IDIs with health workers and FGDs with primary caregivers. The data captured will be linked with other quantitative outcome data collected through ACT PRIME using unique identifiers of health center, community or individual health worker or intervention participant.

4.3.2. Assessment of intervention mechanisms

Perceptions of both recipients and implementers of interventions as the intervention is being rolled-out will be evaluated through the self-filled questionnaires, health worker communication assessments, IDIs and FGDs. Questions will explore the awareness, understanding of purpose, perception of relevance and usefulness, level of adoption and interpretation of importance in practice of each component of the intervention for respondents. All health worker training participants will complete the self-filled questionnaires; all health workers will be invited to participate in the health worker communication assessments; and a cross-section of participants will be invited to the FGDs (primary caregivers, heads of households) and IDIs (health workers, implementers, key stakeholders).

4.4. CONTEXT EVALUATION

Both local and regional/national contextual factors will be documented throughout ACT PRIME using a structured contextual record completed by the study team and rich contextual descriptions gathered through IDIs and FGDs.

4.4.1. Structured contextual record

The structured contextual record will involve the recording of details about factors that may affect ACT PRIME implementation and impact at three-monthly intervals by the implementing team. A structured record format will be used to document these contextual factors for each health center, and at the district level.

Factors may include the following:

- — Other interventions involving malaria at the community level in the trial area
- — Other research involving malaria at the community level in the trial area
- — Other interventions at the health center level in the trial area
- — Other research at the health center level in the trial area
- — Other training programmes involving CMDs or health center staff involved in the trial
- — Specific personalities or political problems at any communities/health center
- — Change of staff at health center
- — Change of community medicine distributor or village health team members
- — Guideline changes about malaria testing and treatment at health centers/elsewhere
- — Messages or news stories about malaria testing on radio/TV/newspapers
- — Level of support (low, medium, high) from district health management team for the intervention
- — Other local or national economic or political factors that may have impacted the delivery or receipt of this intervention

The source of each item added to the local context document will be noted on the document. The data collected in these tables will be assimilated into a report of concurrent activities and other contextual factors overall. Factors that varied widely will be used in the final analysis of the intervention impact as potential explanatory variables.

4.4.2. Rich description of context

Rich descriptions of contextual factors will be collected in all IDIs and FGDs in order to identify any contextual factors participants feel may have affected the impact of the intervention. Representatives from a randomly selected cross-section of health centers and communities will be invited to participate, and district officials will be purposively selected to represent those with most insight into the intervention process.

4.5. IMPACT EVALUATION

Clinical and economic outcomes will be collected as part of ACT PRIME. In addition, the intervention may have wider expected and unexpected impacts at the household level, community level, private sector level, and public health system level. We propose to evaluate the impact of the intervention amongst community members, health workers and others involved in providing health services using IDIs and FGDs.

4.5.1. Assessment of hypothesised impacts

Some impacts may be hypothesised in advance based on the predicted and potential mechanisms of change resulting from the intervention as described under ‘intended effects’ in the logic model (Figure 4.2). Both quantitative and qualitative methods will be used to assess these impacts. For quantitative measurements, existing data collection methods used in ACT PRIME will be utilized wherever possible including cross-sectional survey questionnaires, cohort household surveys, patient exit interviews, health worker knowledge questionnaires, and health facility surveillance questionnaires.

For qualitative assessments of intended effects, IDIs and FGDs will be used to assess whether and how the intervention affected specific hypothesised impacts. IDIs will be conducted with implementers, health workers in the HFI arm and key stakeholders, and FGDs will be conducted with primary caregivers and heads of households.

4.5.2. Assessment of undetermined impacts

Unexpected impacts will be assessed through a 'most significant change' (MSC) evaluation. MSC is a participatory evaluation technique that aims to collect and systematically analyse significant changes from the perspectives of those involved in a programme[41]. The technique aims to capture the values and perspectives of respondents, aiming to enrich the understanding of the intervention beyond intended changes and pre-defined indicators. A sample line of MSC questions include, "Looking back over the past three months what do you think was the most significant change in the way you managed illness in your household? Why is this significant to you? What difference has this made now or will it make in the future?" We will collect MSC stories from participants and use traditional qualitative data analysis approach to display the diversity and richness the responses. The MSC questions will be asked at the start of FGDs and IDIs that will then go on to ask directly about hypothesized impacts.

5 STUDY PROCEDURES

5.1. OVERVIEW

The ACT PROCESS study proposed here will be implemented in parallel with the ACT PRIME study, but will be carried out by a different team of field researchers. Self-filled questionnaires, health worker communication assessments and patient exit interviews, IDIs and semi-structured questionnaires, FGDs, and a contextual record will be used to evaluate the process, context and impact of the HFI in the ACT PRIME study. The self-filled questionnaires will be used to evaluate the HFI training and will be conducted during the HFI roll-out period in March-May 2011. The health worker communication assessments and patient exit interviews will be conducted immediately before and after the HFI training in patient-centered services, and then approximately six months following the training for a total of three cycles. The IDIs and semi-structured questionnaires, and FGDs will be conducted approximately 9-12 months after the HFI roll-out. The structured contextual record will be completed by the study team at three-monthly intervals. The timelines for the process, context and impact evaluations in relation to ACT PRIME are outlined in the study procedures timeline below.

Figure 5.1 Study procedures overview

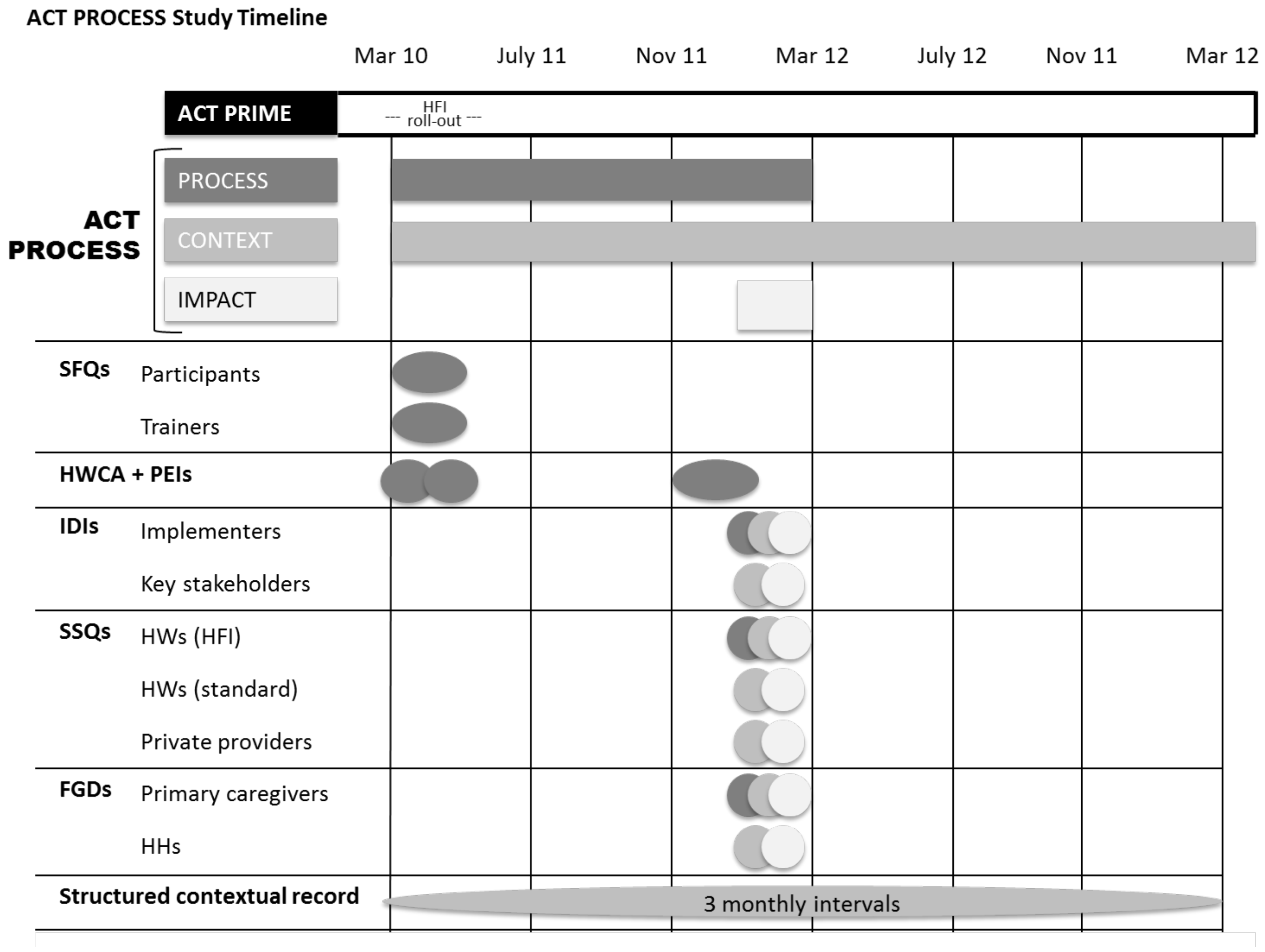

5.2. STUDY SITE

ACT PROCESS, in parallel with ACT PRIME, will be conducted in Tororo district, an area with very high malaria transmission intensity. The estimated entomologic inoculation rate (EIR) in Tororo is 562 infective bites per person-year, and the prevalence of parasitemia among children aged 5-9 years is 63.5%[42-43]. The five sub-counties of West Budama North Health Sub-district (Nagongera, Paya, Kirewa, Kisoko, and Petta), and two sub-counties of West Budama South Health Sub-district (Mulanda and Rubongi) will be included in the study population (Figure 5.2).

Figure 5.2 Study area

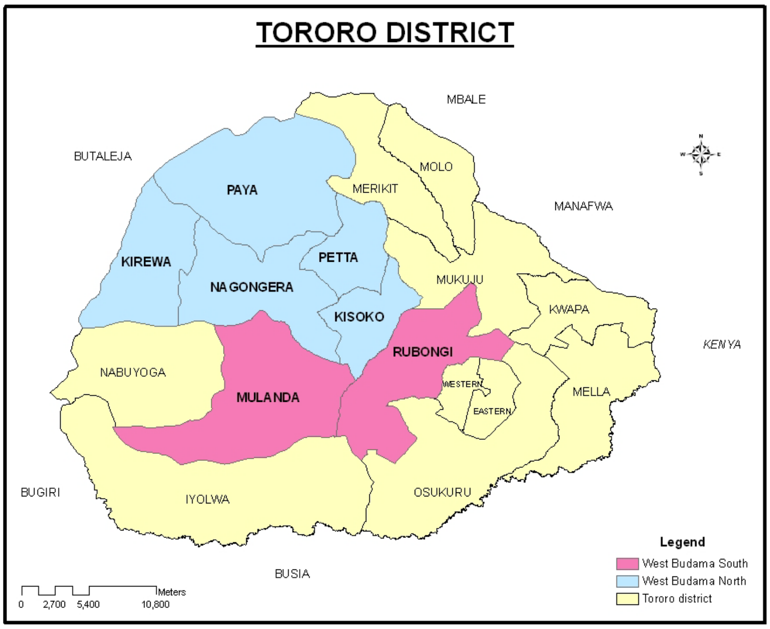

The results of our formative research suggest that this area is very rural, with limited infrastructure and education. Very few households have electricity (1%) and one-quarter have no toilet facilities. One-quarter of the heads of household have received no formal education, and only 21% have received any secondary or higher education[31].

5.3. STUDY POPULATION

Within the seven sub-counties of the study area, there are 22 lower-level government run health facilities, including 17 level II health centers, and 5 level III health centers; 20 will be included in ACT PRIME. These 20 health centers will be randomly allocated to the health facility intervention arm or the standard care arm for a total of 10 health facilities in each arm. Clusters to be included in ACT PRIME are defined as the catchment areas of the health centers including households that are located within a 2 km radius of the facilities. Only households located within the clusters will be included in the sampling frame for ACT PRIME. ACT PROCESS follows the same sampling frame as ACT PRIME; participants for each type of data collection methodology are defined below.

5.3.1. Self-filled questionnaires

Our target is to have one self-filled questionnaire completed by all HFI training participants and trainers for each module. There are 5 trainers and approximately 30 health center staff attending 10 module topics. We will conduct up to 350 self-filled questionnaires.

5.3.2. Health worker communication assessments + patient exit interviews

We aim to conduct communication assessments with at least one health worker in each health facility in both the HFI and standard care arms (10 in each arm). Each communication assessment will consist of at least three, and no more than five, health worker/caregiver interaction records. The communication assessments will be conducted immediately before and up to 6 weeks after the HFI training in patient-centered services, and then approximately six months following the training for a total of three cycles. The same health workers will be assessed at each time point to evaluate for changes over time. We will evaluate approximately 20-25 health workers chosen by convenience sampling. Therefore conducting up to 125health worker/caregiver interactions in each time period; 250 in total, depending on the number of health workers and patients available at the health centers on the days of the study. One health worker will be chosen from each of the level II health centers, and 1-2 health workers will be chosen from each of the level III centers. Exit interviews will be conducted with all consenting caregivers who participated in the assessments, up to a maximum of 125 interviews at each time point, 375 in total.

5.3.3. In-depth interviews

We will conduct up to25IDIs with different target populations including HFI implementers (up to 5 interviews), health workers in the HFI arm (up to 10 interviews), and key local and district stakeholders (up to 10 interviews).

5.3.4. Semi-structured questionnaires

We will administer up to 70semi-structured questionnaires to different target populations including health workers in the HFI arm (up to 30 interviews), health workers in the standard care arm (up to 30 interviews), and private drug shop workers (up to 10 interviews). For the health worker questionnaires, we will target all health workers at all the health facilities included in ACT PRIME.

5.3.5. Focus group discussions

We will conduct up to 12 FGDs with primary caregivers and heads of households from the study area selected by convenience sampling.

5.4. SELF-FILLEDQUESTIONNAIRES

Self-filled questionnaires for each training module will be completed by all staff participants and trainers (Table 5.1).The purpose of the self-filled questionnaires is to gather opinions from participants and trainer on the objectives, content, materials, and implementation of the HFI training modules. Self-filled questionnaires will be completed at the end of each training topic. Training

modules, topics and associated self-filled questionnaires found in the Appendices are outlined in Table 5.1.

All health center staff will be invited to participate in the training module relevant to their position as outlined in Table 5.1. At the beginning of the training, written informed consent to complete the self-filled questionnaires will be obtained from all participants (Appendix B) as outlined in section 8.2. Self-filled questionnaires will be completed directly after the training topic and collected by the study team.

Table 5.1 Health worker training modules, topics and self-filled questionnaires

Module	Participants	Topics	Appendix	
			Trainers	Participants
Health center management	In-charges	Budgeting and accounting<br>Supply management<br>Information management	C	D
Fever case management	Clinical staff	Fever case management	E	F
Patient-centered services	Clinical staff	Introduction to PCS and self-observation<br>Improving interactions with patients I & II<br>Improving interactions with colleagues<br>Improving the patient visits	G	H
Patient-centered services	Support staff	Improving the patient visit	G	H

5.5. HEALTH WORKER COMMUNICATION ASSESSMENTS AND PATIENT EXIT INTERVIEWS

We plan to conduct communication assessments with health workers from both the HFI and standard care arms. The purpose of the assessments is to evaluate and compare the communication between health workers and patients immediately before and after HFI training in ‘communicating with patients’ and then during the study period. Health worker/caregiver interactions during consultations will be audio taped and assessed using a validated measurement methodology, the Measurement of Patient-Centered Communication (MPCC) (Appendix I). The MPCC scores assessments according to three elements of patient-centered communication: 1)exploring the disease and the illness experience, 2) understanding the whole person, and 3) finding common ground[44]. In addition to the recorded interactions, consenting caregivers will be interviewed immediately on exit from the consultation to give their view of the quality of the interaction with the health worker. The purpose of the interviews is to determine the level of satisfaction of caregiver with the health facility visit.

Health workers from both the HFI and standard care arms available on the day of the communication assessment will be selected using convenience sampling, and invited to participate. At least one health worker from each facility, will be included. At least three interactions, and up to five interactions, will be recorded with each health worker. Written informed consent to conduct the assessments will be obtained from health workers before beginning (Appendix J) as outlined in section 8.2. Demographic information on the health worker will then be obtained (Appendix K).

Once a health worker has been selected and provided informed consent, caregivers who will have a consultation with that health worker will be selected using convenience sampling from the available patients visiting the health center on the day of the health worker communication assessment.

Patients to be included in the communication assessments will be 'typical uncomplicated malaria patients'. Inclusion criteria are 1) age: a child under five years of age, 2) fever or suspected fever, and 3) agreement of caregiver to provide informed consent. Exclusion criterion is 1) danger signs of severe disease (Appendix L). The same caregivers will be invited to participate in an exit interview after the consultation (Appendix O). Written informed consent to conduct the assessments, and the exit interviews, will be obtained from caregivers before beginning (Appendix M) as outlined in section 8.2. Demographic information on the caregiver will then be obtained (Appendix N).

The communication assessments will be conducted immediately before and up to 6 weeks after the HFI training in 'improving interactions with patients' and then approximately six months following the training for a total of three cycles. The same health workers will be evaluated in each cycle of the assessments.

5.6. IN-DEPTH INTERVIEWS

We plan to conduct IDIs with HFI implementers, health workers from the HFI arm and key stakeholders. The purpose of the IDIs is to collect information on the contextual factors and perceptions of the HFI as it is being implemented as well as the expected and unexpected impacts of the HFI. Written informed consent to conduct the IDI will be obtained from all participants before beginning (Appendix P) as outlined in section 8.2. The IDIs with health workers and implementers will be conducted approximately 9-12 months after the HFI roll-out. The IDIs with key stakeholders will be conducted approximately one year after the HFI roll-out.

Implementer participants for the IDIs will be purposively selected from the ACT PRIME implementation team. Any implementers who delivered health worker training, worked on drug distribution to health centers, or had significant interaction with health workers or district or local officials during the HFI implementation or follow-up period will be invited to participate. We plan to complete up to five IDIs with implementers, following the pre-defined topic guide (Appendix Q).

Health workers from the HFI arm will be selected by convenience sampling. We plan to complete up to 10 IDIs with health workers stationed at HFI health centers, following the pre-defined topic guide (Appendix R).

Key stakeholders will be purposively selected based on their involvement with the ACT PRIME study during the HFI implementation period or their role in the health system. For example, we plan to interview stakeholders involved with drug distribution and staffing for the HFI, district officials including the District Health Officer and Deputy District Health Officer, as well as key staff from the Tororo sub-district and sub-county level. We expect to complete up to ten IDIs with key stakeholders, following the pre-defined topic guide (Appendix S).

5.7. SEMI-STRUCTURED QUESTIONNAIRES

We plan to administer semi-structured questionnaires to all health workers from HFI and standard care arms, and private drug shop workers. The purpose of the questionnaires is to collect information on the contextual factors and perceptions of the HFI as it is being implemented as well as the expected and unexpected impacts of the HFI. Written informed consent to administer the questionnaires will be obtained from all participants before beginning (Appendix T) as outlined in section 8.2. The questionnaires will be administered to health workers and private drug shop workers approximately 9-12 months after the HFI roll-out.

All health workers from the HFI and standard care arms will be invited to complete a questionnaire. We plan to administer up to 30 questionnaires to health workers in both ACT PRIME arms, 60 in total. The draft questionnaire (Appendix U) will be piloted prior to the onset of the study, and will be refined if necessary.

Private drug shops workers will be randomly selected from the database developed for the Tororo District Survey Project. We plan to administer up to 10 questionnaires to private drug shop workers. The draft questionnaire (Appendix V) will be piloted prior to the onset of the study, and will be refined if necessary.

To better understand the current availability of antimalarial drugs & diagnostics in the private sector, we propose to repeat the survey of private providers in the PRIME study area to assess stocking of ACTs, the availability of AMFm subsidized drugs, and provision of rapid diagnostic tests (RDTs) for malaria.

As part of the repeat survey, we will identify and map all of the private drug shops that are currently active. The repeat survey will be conducted following the same procedures that were used initially. The ten providers who were interviewed initially will be approached, if they can be located, and asked to participate in a repeat interview. In addition, providers from all other drug shops identified will be approached for recruitment, in attempt to interview the full population of private providers. Written informed consent will be obtained from the participants using Appendix T. Interviews will be conducted using a translator if necessary, and data will be captured using the questionnaire in Appendix V, which will be loaded onto a tablet computer.

5.8. FOCUS GROUP DISCUSSIONS

We plan to conduct FGDs with primary caregivers and heads of households from both the HFI and standard care arms. The purpose of the FGDs is to collect information on the contextual factors and perceptions of the intervention as it is being implemented as well as the expected and unexpected impacts of the HFI on communities in the study area. Written informed consent to conduct the FGDs will be obtained from all participants before beginning (Appendix W), as outlined in section 8.2. Participants for FGDs will be selected from the HFI arm, the standard care arm and from areas outside of a 2km radius of an ACT PRIME health center; the same groups of participants will be invited to attend from each arm. The definition of each target population is provided in Table 5.2.

Table 5.2 FGD target populations

Target group	Definitions	FGD characteristics
Primary caregivers	Person primarily responsible for daily care of young children (generally female)	Health center (communities from each health center in ACT PRIME will be represented as well as from areas outside of a 2km radius of an ACT PRIME health center)
Heads of households	Is an adult person or persons who primarily make decisions for the general household(e.g. decisions on healthcare, income, etc)	Health center (communities from health centers in ACT PRIME study will be represented as well as from areas outside of a 2km radius of an ACT PRIME health center)

5.8.1. Community FGDs

Nine FGDs will be conducted with primary caregivers and three FGDs with heads of households approximately 9-12 months after the HFI roll-out. As outlined in Table 5.3, we have designed a matrix which distributes the FGDs across the desired categories.

Table 5.3 Sampling matrices for community FGDs

	**HFI health centers**	**Standard care health centers**	Area outside of a 2km radius of an ACT PRIME health center
PCGs	3	3	3
HHs	1	1	1

Villages in close proximity to the health facilities in ACT PRIME and those outside of a 2km radius of an ACT PRIME health center will be selected using convenience sampling to include in each of the primary caregiver and heads of households FGDs. Different health centers and different villages will be selected for each FGD. At least 3 of the community FGDs will be conducted in villages in close proximity to HC IIIs. Local leaders will be asked to help identify and invite 6-12 representatives from the specified target group to participate in the FGDs.

A moderator and an assistant will lead the discussions in the local language (Japadhola or Swahili) using the FGD guides (Appendix X, Appendix Y). During the FGDs, participants will be encouraged to share all thoughts and opinions. All FGDs will be recorded using a digital voice recorder, provided informed consent is given by participants. Hand-written notes detailing respondent identification numbers

5.9. STRUCTURED CONTEXTUAL RECORD

A structured contextual record will be used to collect details about factors that may affect implementation and impact of the HFI. Sources of information for contextual details include published and grey literature; radio, local television and newspaper reports; notices from the Uganda MoH, National Malaria Control Programme, and other national departments; internet sites of organizations and NGOs active in the area including WHO, other UN organizations, Malaria Consortium, AMREF; and other relevant sources of information. These sources of information will be reviewed on a three-monthly basis by the implementation team and details will be entered into the structured contextual record (Appendix Z).

In addition, detailed data will be collected prospectively on coverage levels of key malaria control interventions across Tororo district as detailed in Table 5.4. These data will be collected through the UMSP sentinel site at Nagongera Health Center IV in Tororo district. Data on IRS coverage will come from the Uganda MoH and implementing partners. Data on ITN coverage and ACT use will come from the cross sectional surveys and outpatient surveillance system operated by the UMSP.

Table 5.4 Malaria control intervention variables of interest

Category	Metric	Source of data

**IRS**	- Date, formulation, and proportion of households sprayed	MoH records
**ITNs**	- Proportion of households with at least one bednet<br>- Proportion of households with at least one ITN<br>- Average number of nets per household<br>- Average number of ITNs per household<br>- Proportion of children under five who slept under any net the prior night<br>- Proportion of children under five who slept under an ITN the prior night	Cross-sectional surveys
**ACTs**	- Proportion of febrile episodes in children treated with an ACT	Cross-sectional surveys
	- Proportion of antimalarial doses prescribed that were ACTs	Outpatient surveillance
	- Number of ACT doses prescribed at health care facility per month	Outpatient surveillance

5.10. SUPPLY OF DRUGS AND RDTS

We plan to collect data on the supply of artemether-lumefantrine and RDTs provided by the ACT PRIME Study to each of the HFI health centers using drug stock cards, and requisition and issue vouchers from the health centers and health sub-districts. Study personnel will collect the information during a one-day visit to the health facilities. The in-charge of the facility will be approached and informed about the surveillance activities. An information sheet (Appendix AA) will be used to describe the purpose of the activities and verbal consent to collect information will be obtained from the in-charge. Information will be collected on (1) supply and use of artemether-lumefantrine supplied by National Medical Stores (NMS) and ACT PRIME; and (2) supply and use of RDTs for malaria supplied by ACT PRIME (Appendix BB).

6 DATA MANAGEMENT

6.1. DATA MANAGEMENT

6.1.1. Quantitative data

Quantitative data from the self-filled questionnaires, patient exit interviews, semi-structured questionnaires, structured contextual record, and supply records will be collected by the study team. Data from the paper questionnaires and data collection forms will be entered into an Access database by a data entry clerk and will be double entered to verify accuracy. Back-up files of databases will be stored after each data entry session. For quality control, query programs will be written into the database to limit the entry of incorrect data and ensure entry of data into required fields.

6.1.2. Qualitative data

6.1.2.1. Self-filled questionnaires

Qualitative data from the self-filled questionnaires will be typed into Word documents for export to NVivo qualitative data management software. All typed records will be checked for accuracy against the original questionnaires. Back-up files of word documents will be stored after each data entry session.

6.1.2.2. Health worker communication assessment

All health worker communication assessments will be audio taped using a digital voice recorder. Assessments will be transcribed and translated into English if necessary. Summaries of the assessments will be coded using an appropriate software package. All coded records will be checked for accuracy against the original recordings and field logs. The consultation recordings will be backed-up after each coding session.

6.1.2.3. Patient exit interviews

Any qualitative data arising from the patient exit interviews will be typed into Word documents for export to NVivo qualitative data management software. All typed records will be checked for accuracy against the original questionnaires. Back-up files of word documents will be stored after each data entry session.

6.1.2.4. IDIs

The IDIs will be administered using the appropriate topic guide by the interviewer. An assistant will take notes of the discussion. All interviews will also be recorded using a digital voice recorder. Summaries of the interviews will be written in the language of the interview, and will then be translated into English if necessary.   Summaries of the interviews will typed into Word documents for export to NVivo qualitative data management software. All typed records will be checked for

accuracy against the original recordings and field notes of the assistant.Back-up files of word documents will be stored after each data entry session.

6.1.2.5. Semi-structured questionnaires

Any qualitative data arising from the semi-structured questionnaires will be typed into Word documents for export to NVivo qualitative data management software. All typed records will be checked for accuracy against the original questionnaires. Back-up files of word documents will be stored after each data entry session.

6.1.2.6. FGDs

The FGDs will be facilitated by a moderator and the assistant will take notes of the discussion and non-verbal communication in English. All FGDs will also be recorded using a digital voice recorder. Recordings of the FGDs will be transcribed into the local language, and then translated into English. The transcripts will be checked for accuracy against the original recordings and field notes by members of the field team. The transcripts and discussion notes will be reviewed for themes and re-organized according to discussion topics. Back-up files of transcripts will be stored after each data entry session.

6.1.2.7. Structured contextual record

Qualitative data from the contextual record will be typed into Word documents for export to NVivo qualitative data management software. All typed records will be checked for accuracy against the original questionnaires. Back-up files of word documents will be stored after each data entry session.

6.2. QUALITY ASSURANCE AND QUALITY CONTROL

All members of the study team will be trained in the project objectives, methods of effective communication with study participants, and collection of high quality data. Study team members will receive additional training specific to the tasks they will perform within the project including interviewing techniques and completing questionnaires. Standard Operating Procedures (SOPs) will be written for all project activities and booklets of all relevant documents will be provided to each member of the project team. Study group meetings will be conducted by the principal investigator to assess progress of the study, address any difficulties, and provide performance feedback to the members of the study group. Any corrections to data collection forms will be made by striking through the incorrect entry with a single line and entering the correct information adjacent to it, according to Good Clinical Practice guidelines[45]. The correction will be initialled and dated by the investigator. The investigators will allow all requested monitoring visits, audits or reviews.

6.3. RECORDS AND STORAGE

All study documents will be kept in secured filing cabinets in the Infectious Disease Research Collaboration offices. The principal investigator will be responsible for the security of all project documents. Back-up files of databases will be stored onto the main project server after each data entry session. Participants will be identified by their study ID number, and participant names will not be entered into the computerised database.

6.4. DATA SHARING

This project is one of 16 participating in the ACT Consortium (www.actconsortium.org/). As part of the ACT Consortium, a policy liaison network will be organized to help synthesize data from the multiple projects and communicate the results to policy makers. Consortium researchers will share data with the policy liaison network to facilitate analyses and ensure broad dissemination of the research findings.

7 ANALYTICAL PLAN

7.1. QUANTITATIVE DATA

Categorical variables from the self-filled questionnaires, patient exit interviews, semi-structured questionnaires, structured contextual record, and supply records, will be compared using the chi-square test or Fisher's exact test. Pair wise comparisons of continuous variables will be made using a two-sample t-test or non-parametric test when appropriate. A p-value < 0.05 (two-tailed) will be considered statistically significant. Analysis will be done using STATA (Stata, College Station, TX, USA).

7.2. AUDIO RECORDINGS

The health worker communication assessment audio recordings will be analyzed at the individual assessment level using the MPCC which has been validated and shows interrater reliabilities (interclass correlations) of 0.80-0.83. The coding is based on three components of patient-centered communication and produces a score for each component. These scores will be used to measure health worker responsiveness to patient concerns and produce a mean score of patient-centered communication ranging from 0 (not patient-centered) to 1 (very patient-centered) [44]. The audio recordings will be coded by trained social scientists, each coding one half of the assessments. All of the assessments will be dual-coded and compared for accuracy. Descriptive statistical analysis on MPCC scores will done using STATA (Stata, College Station, TX, USA).

7.3. QUALITATIVE DATA

Transcripts and interview notes from the self-filled questionnaires, patient exit interviews, IDIs, semi-structured questionnaires, FGDs, and structured contextual record will be analysed using a coding scheme developed from pre-defined topics together with themes emerging from the data. Coding will be done by hand, and using qualitative data analysis software, NVivo (QSR International, Cambridge, MA). We plan to prospectively label and code themes within topics as they emerge, resulting in a data-generated coding scheme. This stage of the analysis will be conducted independently by different members of the study team on different transcripts and then a final coding scheme will be agreed on and applied to all transcripts, with at least two members of the study team reviewing each transcript. The Nvivo software program will be used to aggregate the data by codes, and to assist with report writing.

8 PROTECTION OF HUMAN PARTICIPANTS

8.1. INSTITUTIONAL REVIEW BOARDS

This protocol and the information sheets will be reviewed and approved by all IRBs before the project begins. Any amendments or modifications to this material will also be reviewed and approved by the IRBs prior to implementation. The IRBs will include:

London School of Hygiene & Tropical Medicine (LSHTM) Ethics Committee

Address: Keppel Street, London, WC1E 7HT, UK
Contact Person: Paula Eliott
Phone Number: +44 (0) 20 7927 2256
Email: Ethics@lshtm.ac.uk

Faculty of Medicine Research and Ethics Committee (FOMREC), Makerere University

Address: Makerere University, Faculty of Medicine, Office of the Dean, PO Box 7072, Kampala, Uganda
Contact Person: Dr. Charles Ibingira
Phone Number: +256 (0) 414-530020
Fax Number: +256 (0) 414-531091

Uganda National Council of Science and Technology (UNCST)

Address: Plot 3/5/7 Nasser Road, PO Box 6884, Kampala, Uganda
Contact Person: Dr. Peter Ndmerere
Phone Number: +256 (0) 414-250499
Fax Number: +256 (0) 414-234579

8.2. INFORMED CONSENT PROCESS

Approval from local leaders will be sought before beginning activities in the project area. Written informed consent will be obtained from all participants for the self-filled questionnaires, health worker communication assessments and patient exit interviews, IDIs, semi-structured questionnaires, and FGDs. Study personnel will conduct informed consent discussions with potential study participants or their parent/guardian. Informed consent will be conducted in the appropriate language and a translator will be used if necessary. Consent forms will be available in English, Japadhola, and Swahili. During the consent discussion, the appropriate consent form will be read to the potential study participant (or parent/guardian) describing the purpose of the project, the procedures to be followed, and the risks and benefits of participation, and any questions raised will be answered. Following the informed consent discussion, the potential study participant (or parent/guardian) will be asked to provide their written consent on the approved informed consent document to participate in a research study. If the potential study participant (or parent/guardian) is unable to read or write, their fingerprint will substitute for a signature, and a signature from a witness to the informed consent procedures will be obtained.

Verbal consent will also be obtained prior to collecting information on the supply of drugs and RDTs from the health center in-charges using an information sheet. Information sheets in local

languageswill be provideddescribing the purpose of the project and the procedures to be followed, and the risks and benefits of participation.

8.2.1. Self-filled questionnaires

Study personnel will seek informed consent from PRIME HFI training participants and from HFI trainers to complete the self-filled questionnaires. The informed consent discussion will be conducted with participants and trainers at the location of the training (health facility or other convenient location) prior to beginning the training. If the health worker cannot read, an impartial witness will be present during the entire consent process.

8.2.2. Health worker communication assessments and patient exit interviews

Study personnel will seek informed consent from health workers for participation in the health worker communication assessments and from caregivers for participation in both the health worker communication assessments and patient exit interviews. The informed consent discussion will be conducted with health workers at the health facility prior to beginning the assessment. If the health worker cannot read, an impartial witness will be present during the entire consent process. After a health worker has consented to participate, written informed consent will be sought from caregivers prior to each interaction. If a health worker or caregiver cannot read, an impartial witness will be present during the entire consent process.

8.2.3. In-depth Interviews

Study personnel, including a translator if necessary, will seek informed consent from potential study participants for participation in the IDIs. The informed consent discussion will be conducted with potential participants at a convenient location (health center, office, residence) and in a language that the participant is most comfortable with, using a translator if necessary. If the potential participant cannot read, an impartial witness will be present during the entire consent process.

8.2.4. Semi-structured questionnaires

Study personnel, including a translator if necessary, will seek informed consent from potential study participants for participation in the semi-structured questionnaires. The informed consent discussion will be conducted with potential participants at a convenient location (health center, office, residence) and in a language that the participant is most comfortable with, using a translator if necessary. If the potential participant cannot read, an impartial witness will be present during the entire consent process.

8.2.5. Focus group Discussions

Study personnel, including a translator if necessary, will seek informed consent from primary caregivers and heads of households for participation in the FGDs. The informed consent discussion will be conducted with primary caregivers and heads of households at their residence in the language that the primary caregiver/head of household is most comfortable with, using a translator if necessary. If the primary caregiver/head of household cannot read, an impartial witness will be present during the entire consent process.

8.2.6. Supply of drugs and RDTs

Study personnel will collect the information about the supply and stocks of artemether-lumefantrine and RDTs during a one-day visit to the health facilities. The in-charge of the facility will be approached prior to the first visit and informed about the surveillance activities. An information sheet will be used to describe the purpose of the activities and verbal consent to collect information will be obtained from the in-charge. If the in-chargegives their verbal consent to participate in the study, their consent to participate in the study will be documented on the data collection log.

8.3. CONFIDENTIALITY

Participants in all study activities will be informed that participation in a research study may involve a loss of privacy. All records will be kept as confidential as possible. Participants will be identified by study numbers and participant names will not be entered into the computerized database. FGD participants will be referred to by their first names during the discussion, but names will not be recorded in the notes or transcripts; participants will be referred to by a participant ID only. In addition, participants providing qualitative data, including those involved in the self-filled questionnaires, patient exit interviews, IDIs, semi-structured questionnaires, and FGDs will be given the option of not being quoted at all, anonymously or otherwise, or included in any of the analyses. Completed questionnaires will be kept in secured filing cabinets in the study offices in Tororo and Kampala. Additional records will be stored in the log books, which will be stored securely in the study offices in Tororo. No individual identities will be recorded in the database or used in any reports or publications resulting from the study.

8.4. RISKS AND DISCOMFORTS

8.4.1. Privacy

Care will be taken to protect the privacy of participants, as described in this protocol. However, there is a risk that others may inadvertently see participants' information, and thus their privacy compromised. All information gathered will be treated as private by the study personnel, and records will be kept securely in locked filing cabinets and offices. No personal identification information such as names will be used in any reports arising out of this research.

8.4.2. Compensation

Self-filled questionnaires, health worker communication assessments and patient exit interviews, IDIs, semi-structures questionnaires, and FGDs will be held in venues central to participants' residences or place of work. There will be no cost to participants and participants will not be paid; however, 5000/= will be given to each FGD participant as compensation to refund their transport costs.

8.4.3. Alternatives

All identified participants may choose not to participate in any of the study activities. A decision not to participate will not have any impact on employment or eligibility for medical care or participation in future studies.

9 REFERENCES

1. Greenwood, B.M., et al., *Malaria.* Lancet, 2005. **365**(9469): p. 1487-98.

2. Breman, J., *The ears of the hippopotamus: manifestations, determinants, and estimates of the malaria burden.* Am J Trop Med Hyg, 2001. **64**: p. 1-11.

3. Breman, J.G., M.S. Alilio, and A. Mills, *Conquering the intolerable burden of malaria: what's new, what's needed: a summary.* Am J Trop Med Hyg, 2004. **71**(2 Suppl): p. 1-15.

4. Snow, R.W., et al., *Estimating mortality, morbidity and disability due to malaria among Africa's non-pregnant population [see comments].* Bull World Health Organ, 1999. **77**(8): p. 624-40.

5. Uganda Ministry of Health, *The Burden of Malaria in Uganda*. 2008.

6. Uganda Ministry of Health, *Malaria control and prevention*. 2008.

7. Kager, P.A., *Malaria control: constraints and opportunities.* Trop Med Int Health, 2002. **7**(12): p. 1042-6.

8. Moerman, F., et al., *The contribution of health-care services to a sound and sustainable malaria-control policy.* Lancet Infect Dis, 2003. **3**(2): p. 99-102.

9. McCombie, S.C., *Treatment seeking for malaria: a review of recent research.* Soc Sci Med, 1996. **43**(6): p. 933-45.

10. Marsh, V.M., et al., *Changing home treatment of childhood fevers by training shop keepers in rural Kenya.* Trop Med Int Health, 1999. **4**(5): p. 383-9.

11. Goodman, C., et al., *Retail supply of malaria-related drugs in rural Tanzania: risks and opportunities.* Trop Med Int Health, 2004. **9**(6): p. 655-63.

12. Kelly, J.M., et al., *Community health worker performance in the management of multiple childhood illnesses: Siaya District, Kenya, 1997-2001.* Am J Public Health, 2001. **91**(10): p. 1617-24.

13. Zurovac, D. and A.K. Rowe, *Quality of treatment for febrile illness among children at outpatient facilities in sub-Saharan Africa.* Ann Trop Med Parasitol, 2006. **100**(4): p. 283-96.

14. Leonard, K., M. Masatu, and A. Vialou, *Getting clinicians to do their best: Ability, Altruism and Incentives.* 2005, Working paper, University of Maryland.

15. Chakraborty, S. and K. Frick, *Factors influencing private health providers' technical quality of care for acute respiratory infections among under-five children in rural West Bengal, India.* Soc Sci Med, 2002. **55**(9): p. 1579-87.

16. Arifeen, S.E., et al., *Quality of care for under-fives in first-level health facilities in one district of Bangladesh.* Bull World Health Organ, 2005. **83**(4): p. 260-7.

17. Krause, G., et al., *Diagnostic quality in rural health centers in Burkina Faso.* Trop Med Int Health, 1998. **3**(2): p. 100-7.

18. World Health Organisation, *World Health Report 2000. Health Systems: Improving Performance*. 2000: Geneva.

19. Brown, L.D., et al., *Quality Assurance of Health Care in Developing Countries*. 1993, Quality Assurance Project. Online at http://www.qaproject.org/pubs/PDFs/DEVCONT.pdf. Accessed 24 July 2008: Bethesda, MD.

20. Hall, J.A., et al., *Physicians' liking for their patients: more evidence for the role of affect in medical care.* Health Psychol, 1993. **12**(2): p. 140-6.

21. Mbaruku, G. and S. Bergstrom, *Reducing maternal mortality in Kigoma, Tanzania.* Health Policy Plan, 1995. **10**(1): p. 71-8.

22. McPake, B., *User charges for health services in developing countries: a review of the economic literature.* Soc Sci Med, 1993. **36**(11): p. 1397-405.

23. Wouters, A.V., *Essential national health research in developing countries: health care financing and the quality of care.* Int J Health Planning and Management, 1991. **6**: p. 253-271.

24. El Arifeen, S., et al., *Integrated Management of Childhood Illness (IMCI) in Bangladesh: early findings from a cluster-randomised study.* Lancet, 2004. **364**(9445): p. 1595-602.

25. Deyo, R.A. and T.S. Inui, *Dropouts and broken appointments. A literature review and agenda for future research.* Med Care, 1980. **18**(11): p. 1146-57.

26. Vera, H., *The client's view of high-quality care in Santiago, Chile.* Stud Fam Plann, 1993. **24**(1): p. 40-9.

27. Williams, B., *Patient satisfaction: a valid concept?* Soc Sci Med, 1994. **38**(4): p. 509-16.

28. Grimshaw, J., et al., *Changing provider behaviour: an overview of systematic reviews of interventions to promote implementation of research findings by healthcare professionals*, in *Getting Research Findings into Practice*, A. Haines and A. Donald, Editors. 2002, BMJ Books: London. p. 29-68.

29. Oxman, A.D., et al., *No magic bullets: a systematic review of 102 trials of interventions to improve professional practice.* Cmaj, 1995. **153**(10): p. 1423-31.

30. Smith, L.A., et al., *Review: Provider practice and user behavior interventions to improve prompt and effective treatment of malaria: do we know what works?* Am J Trop Med Hyg, 2009. **80**(3): p. 326-35.

31. Staedke, S.G. and ACT Study Team, *Tororo District Survey Project: Characterizing the population and local health services*. 2010, Uganda Malaria Surveillance Project, Infectious Disease Research Collaboration.: Kampala.

32. Ross-Degnan, D., et al., *Improving pharmaceutical use in primary care in developing counties: a critical review of experience and lack of experience.*, in *Presented at the International Conference on Improving Use of Medicines*. 1997: Chiang Mai, Thailand, April

33. Power, R., et al., *Developing complex interventions for rigorous evaluation--a case study from rural Zimbabwe.* Health Educ Res, 2004. **19**(5): p. 570-5.

34. MRC, *Developing and Evaluating Complex Interventions: new guidance*. 2008, Medical Research Council. Available online at http://www.mrc.ac.uk/consumption/idcplg?IdcService=GET_FILE&dID=15585&dDocName=MRC004871&allowInterrupt=1: London.

35. Lewin, S., C. Glenton, and A.D. Oxman, *Use of qualitative methods alongside randomised controlled trials of complex healthcare interventions: methodological study.* BMJ, 2009. **339**: p. b3496.

36. Harris, M.J., *Evaluating Public and Community Health Programmes*. 2010, San Fransisco: Jossey-Bass.

37. Saunders, R.P., M.E. Evans, and P. Joshi, *Developing a Process-Evaluation Plan for Assessing Health Promotion Program Implementation: A How-To Guide.* Health Promotion Practice, 2005. **6**: p. 134-147.

38. Chandler, C.I.R., *ACT Consortium Social Science Guidance*. 2009.

39. Chandler, C.I.R., R. Hall-Clifford, and S. Yeung, *ACT Consortium Framework for Evaluation of RDT Interventions*. 2009.

40. Jones, N., et al., *Improving Impact Evaluation Production and Use*. 2009, Overseas Development Institute.

41. Davies, N. and J. Dart, *The 'Most Significant Change' (MSC) Technique. A guide to its use. Available online at http://www.mande.co.uk/docs/MSCGuide.pdf*. 2005.

42. Okello, P.E., et al., *Variation in malaria transmission intensity in seven sites throughout Uganda.* Am J Trop Med Hyg, 2006. **75**(2): p. 219-25.

43. Pullan, R.L., et al., *Plasmodium infection and its risk factors in eastern Uganda.* Malar J, 2010. **9**: p. 2.

44. Epstein, R.M., et al., *Measuring patient-centered communication in Patient-Physician consultations: Theoretical and practical issues.* Social Science & Medicine, 2005. **61**(7): p. 1516-1528.

45. International Conference on Harmonisation of Technical Requirements for Registration of Pharmaceuticals for Human Use (ICH), *Guidance for Industry: Good Clinical Practice: Consolidated Guidance (ICH-E6)*. 1996, Rockville, MD: U.S. Department of Health and Human Services, Food and Drug Administration, Center for Drug Evaluation and Research (CDER), Center for Biologics Evaluation and Research (CBER).

10 APPENDICES

Appendix A:	Informed consent for SFQs – Trainers
Appendix B:	Informed cdonsent for SFQs – Participants
Appendix C:	SFQ Health center management training – Trainers
Appendix D:	SFQ Health center management training – Participants
Appendix E:	SFQ Fever case management training – Trainers
Appendix F:	SFQ Fever case management training – Participants
Appendix G:	SFQ Patient-centeredservices training – Trainers
Appendix H:	SFQ Patient-centered services training – Participants
Appendix I:	Measuring Patient-Centered Communication coding scheme
Appendix J:	Informed consent for HWCA – Health workers
Appendix K:	HWCA demographic form – Health workers
Appendix L:	HWCA Screening Form – Caregivers
Appendix M:	Informed consent for HWCA – Caregivers
Appendix N:	HWCA demographic form – Care givers
Appendix O:	HWCA Patient Exit Interview
Appendix P:	Informed consent for IDIs
Appendix Q:	IDI Data collection tool – Implementers
Appendix R:	IDI Data collection tool – Health workers (HFI)
Appendix S:	IDI Data collection tool – Key stakeholders
Appendix T:	Informed consent for SSQ
Appendix U:	SSQ – Health workers, HFI and standard care
Appendix V:	SSQ – Private drug shops
Appendix W:	Informed consent for FGDs
Appendix X:	FGD Data collection tool – Primary caregivers
Appendix Y:	FGD Data collection tool – Heads of households
Appendix Z:	Contextual record form
Appendix AA:	Information sheet for surveillance of AL & RDTs
Appendix BB:	Data collection form for surveillance of AL & RDTs
Appendix CC:	SSQ – Health Centre in-charges HFI

[____]—[____|____|____|____]

SFQ Study ID

APPENDIX A. SELF-FILLED QUESTIONNAIRES
Informed consent form for participants

**Protocol Title:**	ACT PROCESS: Evaluating the process, context, and impact of interventions to enhance health facilities in Tororo, Uganda
**Site of Research:**	Tororo, Uganda
**Principal Investigators**:	Dr. Sarah Staedke
**Date:**	23 February 2011

Introduction

Dr. Sarah Staedke and colleagues from the Uganda Malaria Surveillance Project/Infectious Diseases Research Collaboration are conducting a research study to see if we can improve the health of children in this area by improving services at government-run health facilities. Part of this study intervention includes training for health workers on the following topics: (1) health centre management, (2) fever case management and use of rapid diagnostic tests for malaria, (3) patient-centred services. You have been invited to take part in these training sessions because of your role in the health facility. In addition to taking part in the training, we are asking for your feedback on the training session you attended.

Why is this study being done?

We would like to know more about how our training sessions were delivered in your area. To do this, we are asking all health workers who take part in the training for their opinions on the session they attended. This information will help us understand how the training session was delivered by our trainer and what you thought about the training methods, content, and objectives.

What will happen today if I take part in this study?

Today, we are inviting you to complete a questionnaire on your own regarding the training session. We can help you fill in the questionnaire if you would like. You will be asked to complete a questionnaire at the end of each training session. We will collect your questionnaires for analysis. After you complete the questionnaire, we will not ask you to do anything further. The information we collect will be used by project investigators, and may be shared with other researchers and policy-makers to answer questions about how best to deliver training and health services.

How long will the study last?

The training sessions will be conducted over 8-10 weeks. The number of training sessions that you will take part in will depend on the role you play at the health center. At the end of each session, you will be asked to complete a questionnaire. Each questionnaire will take about 30 minutes to complete.

Can I stop being in the study?

You can decide to stop participating at any time. Just tell the project researcher right away.

What risks can I expect from being in the study?

Participation in any research study may involve a loss of privacy. Information you provide about your experiences and opinions will be recorded, but your name will not be used in any reports of the information provided. No quotes or other results arising from your participation in this study will be included in any reports, even anonymously, without your agreement. The information obtained from these questionnaires will be locked at our project offices. We will do our best to make sure that the personal information gathered is kept private.

Are there benefits to taking part in the study?

By participating in the training session, you will have the chance to learn new information and skills, or refresh information and skills you have already learned. There will be no other direct benefit to you from participating in this study. However, the feedback that you provide about the training sessions will help researchers and policy-makers understand how best to improve health services in this area, especially in the treatment of malaria.

What other choices do I have if I do not take part in this study?

You are free to choose not to take part in the study. If you decide not to take part in this study, there will be no penalty to you.

What are the costs of taking part in this study? Will I be paid for taking part in this study?

There are no costs to you for taking part in this study. You will not be paid for taking part in this study.

What are my rights if I take part in this study?

Taking part in this study is your choice. You may choose either to take part or not to take part in the study. If you decide to take part in this study, you may change your mind at any time. No matter what decision you take, there will be no penalty to you in any way.

Who can answer my questions about the study?

You can talk to the researchers about any questions or concerns you have about these study activities. Contact Dr. Sarah Staedke or other members of the Uganda Malaria Surveillance Project / Infectious Disease

Research Collaboration on telephone number 0414-530692. If you have any questions, comments or concerns about taking part in these activities, first talk to the researchers. If for any reason you do not wish to do this, or you still have concerns about doing so, you may contact Dr Charles Ibingira, Makerere University Faculty of Medicine Research and Ethical Committee at telephone number 0414-530020.

WHAT YOUR SIGNATURE OR THUMBPRINT MEANS

Your signature or thumbprint below means that you understand the information given to you in this consent form about your participation in the study and agree with the following statements:

1. "I have read the consent form concerning this study (or have understood the verbal explanation of the consent form) and I understand what will be required of me and what will happen to me if I take part in it."

2. "My questions concerning this study have been answered by Dr. Staedke or the person who signed below."

3. "I understand that at any time, I may withdraw from this study without giving a reason."

4. "I agree to take part in this study."

If you wish to participate in this study, you should sign or place your thumbprint on the following page. If you do not agree to quotes or other results arising from your participation in the study being included, even anonymously, in any reports about the study, please tell the researcher now.

THE REPUBLIC OF UGANDA

WE WILL GIVE YOU A COPY OF THIS SIGNED AND DATED CONSENT FORM

____
Name of Participant (printed)

____
Signature or Fingerprint of Participant Date/Time

____
Name of Investigator Administering Consent (printed) Position/Title

____
Signature of Investigator Administering Consent Date/Time

If the participant is unable to read and/or write, an impartial witness should be present during the entire informed consent discussion.

After the written informed consent form is read and explained to the participant, and after they have orally consented to participate in the study, the witness should print the name of the participant above, and then the participant should provide their fingerprint.

Then the witness should print their name, provide their signature, and date the consent form below.

By signing the consent form, the witness attests that the information in the consent form and any other written information was accurately explained to, and apparently understood by the participant, and that informed consent was freely given by the participant.

____
Name of Person Witnessing Consent (printed)

____
Signature of Person Witnessing Consent Date/Time

THE REPUBLIC OF UGANDA

[____]—[____|____|____|____]

SFQ Study ID

APPENDIX B. SELF-FILLED QUESTIONNAIRES
Informed consent form for trainers

**Protocol Title:**	ACT PROCESS: Evaluating the process, context, and impact of interventions to enhance health facilities in Tororo, Uganda
**Site of Research:**	Tororo, Uganda
**Principal Investigators**:	Dr. Sarah Staedke
**Date:**	23 February 2011

Introduction

Dr. Sarah Staedke and colleagues from the Uganda Malaria Surveillance Project/Infectious Diseases Research Collaboration are conducting a research study to see if we can improve the health of children in this area by improving services at government-run health facilities. Part of this study intervention includes training for health workers on the following topics: (1) health centre management, (2) fever case management and use of rapid diagnostic tests for malaria, (3) patient-centred services. You have been invited to take part in this study because of your role in delivering the training. We are asking for your feedback on the training session you delivered.

Why is this study being done?

We would like to know more about how our training sessions were delivered in this area. To do this, we are asking all trainers who take part in delivering the training for their opinions on the session they delivered. This information will help us understand how the training session was delivered from your perspective and what you thought about the training methods, content, and objectives.

What will happen today if I take part in this study?

Today, we are inviting you to complete a questionnaire on your own regarding the training session. We can help you fill in the questionnaire if you would like. You will be asked to complete a questionnaire at the end of each training session you deliver. We will collect your questionnaires for analysis. After you complete the questionnaire, we will not ask you to do anything further. The information we collect will be used by project investigators, and may be shared with other researchers and policy-makers to answer questions about how best to deliver training and health services.

How long will the study last?

The training sessions will be conducted over 8-10 weeks. At the end of each training session you deliver, you will be asked to complete a questionnaire. Each questionnaire will take about 30 minutes to complete.

Can I stop being in the study?

You can decide to stop participating at any time. Just tell the project researcher right away.

What risks can I expect from being in the study?

Participation in any research study may involve a loss of privacy. Information you provide about your experiences and opinions will be recorded, but your name will not be used in any reports of the information provided. No quotes or other results arising from your participation in this study will be included in any reports, even anonymously, without your agreement. The information obtained from these questionnaires will be locked at our project offices. We will do our best to make sure that the personal information gathered is kept private.

Are there benefits to taking part in the study?

By participating as a trainer in our program, you will have the chance to learn new information and skills, or refresh information and skills you have already learned. There will be no other direct benefit to you from participating in this study. However, the feedback that you provide about the training sessions will help researchers and policy-makers understand how best to improve health services in this area, especially in the treatment of malaria.

What other choices do I have if I do not take part in this study?

You are free to choose not to take part in the study. If you decide not to take part in this study, there will be no penalty to you.

What are the costs of taking part in this study? Will I be paid for taking part in this study?

There are no costs to you for taking part in this study. You will not be paid for taking part in this study.

What are my rights if I take part in this study?

Taking part in this study is your choice. You may choose either to take part or not to take part in the study. If you decide to take part in this study, you may change your mind at any time. No matter what decision you take, there will be no penalty to you in any way.

Who can answer my questions about the study?

You can talk to the researchers about any questions or concerns you have about these study activities. Contact Dr. Sarah Staedke or other members of the Uganda Malaria Surveillance Project / Infectious Disease Research Collaboration on telephone number 0414-530692. If you have any questions, comments or

concerns about taking part in these activities, first talk to the researchers. If for any reason you do not wish to do this, or you still have concerns about doing so, you may contact Dr Charles Ibingira, Makerere University Faculty of Medicine Research and Ethical Committee at telephone number 0414-530020.

WHAT YOUR SIGNATURE OR THUMBPRINT MEANS

Your signature or thumbprint below means that you understand the information given to you in this consent form about your participation in the study and agree with the following statements:

1. "I have read the consent form concerning this study (or have understood the verbal explanation of the consent form) and I understand what will be required of me and what will happen to me if I take part in it."

2. "My questions concerning this study have been answered by Dr. Staedke or the person who signed below."

3. "I understand that at any time, I may withdraw from this study without giving a reason."

4. "I agree to take part in this study."

If you wish to participate in this study, you should sign or place your thumbprint on the following page. If you do not agree to quotes or other results arising from your participation in the study being included, even anonymously, in any reports about the study, please tell the researcher now.

THE REPUBLIC OF UGANDA

WE WILL GIVE YOU A COPY OF THIS SIGNED AND DATED CONSENT FORM

Name of Participant (printed)

Signature or Fingerprint of Participant Date/Time

Name of Investigator Administering Consent (printed) Position/Title

---

Signature of Investigator Administering Consent Date/Time

If the participant is unable to read and/or write, an impartial witness should be present during the entire informed consent discussion.

After the written informed consent form is read and explained to the participant, and after they have orally consented to participate in the study, the witness should print the name of the participant above, and then the participant should provide their fingerprint.

Then the witness should print their name, provide their signature, and date the consent form below.

By signing the consent form, the witness attests that the information in the consent form and any other written information was accurately explained to, and apparently understood by the participant, and that informed consent was freely given by the participant.

Name of Person Witnessing Consent (printed)

Signature of Person Witnessing Consent Date/Time

APPENDIX C: HEALTH CENTRE MANAGEMENT TRAINING SFQs

PART 1: TRAINER DEMOGRAPHIC BACKGROUND FORM

If you are leading multiple training modules, you only need to complete this form once.

Thank you for assisting with the delivery of these training workshops. Please take a moment to answer the following questions. There is no need to write your name on this questionnaire, and all responses will be kept strictly confidential. Thank you!

Trainer Study ID [____|____]

Please complete the boxes below with the relevant numbers

**1. Age in years** [____|____]	**2. Gender** 1 = Male<br>2 = Female [____]

3. Qualifications [____|____]

01 = Senior medical Officer	06 = Enrolled nurse	11 = Laboratory technician
02 = Medical Officer	07 = Comprehensive nurse	12 = Laboratory assistant
03 = Senior clinical Officer	08 = Midwife	13 = Health assistant
04 = Clinical Officer	09 = Public health nurse	14 = Health educator
05 = Nursing Officer	10 = Nursing aide/assistant	15 = Other ________________________________

4. What training courses have you delivered in the past 3 years?

	Title of training delivered	Organization	Dates [dd/mm/yy] to [dd/mm/yy]
4a			[_____/_____/_____]<br>to [_____/_____/_____]
4b			[_____/_____/_____]<br>to [_____/_____/_____]
4c			[_____/_____/_____]<br>to [_____/_____/_____]

5. What training courses have you attended in training methods?

	Title of training you attended	Organization	Dates [dd/mm/yy]
5a			[_____/_____/_____]
5b			[_____/_____/_____]
5c			[_____/_____/_____]

Please continue overleaf if you have delivered/attended more than 3 training workshops.

APPENDIX C: HEALTH CENTRE MANAGEMENT TRAINING
SFQ for TRAINERS – MODULE: PHC FUND MANAGEMENT (HCM01)

'Thank you for assisting with this health centre management training! We would appreciate your feedback on the training session you delivered today. Please take a moment to answer the following questions. Your comments will help us to know what information this group received and how well they achieved our intended learning objectives during this session. This will help us to know how to interpret any future change of practice amongst these health workers. There is no need to write your name on this questionnaire, and all responses will be kept strictly confidential. Thank you!'

**Trainer Study ID**	**Date of training**	**Study ID of other Trainers present**
[____|____]	[____|____]/ [____|____]/[____|____]<br>day month year	[____|____], [____|____]
**Training group #**	**Total # of participants invited**	**Total # of participants attended**
[____|____]	[____|____]	[____|____]

Participant Study IDs

[____|____|____|____]	[____|____|____|____]	[____|____|____|____]	[____|____|____|____]
[____|____|____|____]	[____|____|____|____]	[____|____|____|____]	[____|____|____|____]
[____|____|____|____]	[____|____|____|____]	[____|____|____|____]	[____|____|____|____]
[____|____|____|____]	[____|____|____|____]	[____|____|____|____]	[____|____|____|____]

TRAINER QUESTIONNAIRE

**1. Did the training start more than half an hour late?**	1 = Yes<br>2 = No	[____]
**2. If yes, what was the cause of the delay?**	1 = Participants arrived late<br>2 =Trainers arrived late<br>3 = Materials and supplies to be used were not delivered in time<br>4= Trainer transport difficulties<br>5= Other ____	[____]<br>[____]<br>[____]

3. Please describe the consequence of the delay on your ability to deliver the training as intended.

Training component	Consequence of delay on trainer's ability to deliver the training as intended
a) HCM00 – Introduction to HCM	
b) New topics introduced in the module	
c) Practice activities for the PHC Fund Management Tool	

**4. What level of contribution did the participants have?**	1 = All contributed a lot<br>2 = All contributed at some point<br>3 = Only some contributed<br>4 = None contributed	[____]

5. For Qn. 4, responses 2 and 3 please list the 3 least active participants.

[____|____|____|____] [____|____|____|____] [____|____|____|____]

HEALTH CENTRE MANAGEMENT TRAINING<br>SFQ for TRAINERS – MODULE: PHC FUND MANAGEMENT (HCM01)	
**Trainer Study ID**<br>[____|____]	**Date of training**<br>[____|____]/ [____|____]/[____|____]<br>day month year

6. Please complete the table below to describe how this training session went for each topic.

Topic	Teaching as planned?	Materials as planned?	If no, please explain
**a) Accountability**	1 = Yes<br>2 = No<br>[____]	1 = Yes<br>2 = No<br>[____]	
**b) The PHC Fund**	1 = Yes<br>2 = No<br>[____]	1 = Yes<br>2 = No<br>[____]	
**c) Budgeting and Accounting using the PHC Fund Management Tool**	1 = Yes<br>2 = No<br>[____]	1 = Yes<br>2 = No<br>[____]	
**d) Budgeting and Accounting – Putting it all together**	1 = Yes<br>2 = No<br>[____]	1 = Yes<br>2 = No<br>[____]	

7. Can you please describe any difficulties/barriers you encountered in delivery of the training?

**Barriers** to optimum implementation (e.g. - training interrupted, difficult or over bearing individuals, etc.)	**Impact** of barrier on the intended delivery of training and on learning

HEALTH CENTRE MANAGEMENT TRAINING
SFQ for TRAINERS – MODULE: PHC FUND MANAGEMENT (HCM01)

Trainer Study ID	Date of training
[____|____]	[____|____]/ [____|____]/[____|____]<br>day month year

8. Please score how well you think the participants achieved each of the learning objectives today on a scale of 1-10.
(1=Not achieved, 10= Achieved in full. Use 'N/A' if the section is not included in topic)

Learning objectives	Score	Comments
a) Understand the meaning and role of accountability for in-charges		
b) Recognize how being accountable impacts on others' perception of in-charges		
c) Describe the role of accountability in good health centre management		
d) Describe the Ministry of Health policy for PHC Funds for HC II/IIIs		
e) Understand the rationale for training in budgeting, accounting, and PHC Fund management		
f) Describe how the health centre uses its PHC Funds		
g) Recognize how in-charges can build trust and accountability in their roles through good PHC Fund management		
h) Describe the principles of budgeting and accounting		
i) Develop and apply budgeting and accounting skills using the PHC Fund Management Tool		
j) Describe the importance and benefit of budgeting and accounting for the PHC Fund		
k) Understand how budgeting and accounting contributes to showing accountability and skill as an in-charge		
l) Plan and commit to completing the PHC Fund Management tool regularly at their health centres		

**9. Please list commitments or plans made by participants during the workshop today. (List the commitments made and in brackets, list the representatives of each health centre who made that commitment. For example "To put up pictorial signs at the health centre (HC03, 05 and 06)"**	
**10. How many questions or concerns were raised by this group about the topics covered today?**	1 = Many questions<br>2 = Few questions<br>3 = None [_____]
**11. What questions or concerns were raised by this group about the topics discussed today? (Please list)**	
**12. How did you address each of these concerns in this group?**	
**13. Which of these concerns do you think were still present at the end of the training?**	
**14. Would you change anything about the session you gave to this group today? If yes, what would you change? How would you change it?**	
**15. What is your overall assessment of how well the intended objectives of today's training were achieved?**	1 = Badly<br>2 = Fine<br>3 = Good [_____]

APPENDIX C: HEALTH CENTRE MANAGEMENT TRAINING
SFQ for TRAINERS – MODULE: DRUG SUPPLY MANAGEMENT (HCM02)

'Thank you for assisting with this health centre management training! We would appreciate your feedback on the training session you delivered today. Please take a moment to answer the following questions. Your comments will help us to know what information this group received and how well they achieved our intended learning objectives during this session. This will help us to know how to interpret any future change of practice amongst these health workers. There is no need to write your name on this questionnaire, and all responses will be kept strictly confidential. Thank you!'

**Trainer Study ID**	**Date of training**	**Study ID of other Trainers present**
[____|____]	[____|____]/ [____|____]/[____|____]<br>day month year	[____|____], [____|____]
**Training group #**	**Total # of participants invited**	**Total # of participants attended**
[____|____]	[____|____]	[____|____]

Participant Study IDs

[____|____|____|____]	[____|____|____|____]	[____|____|____|____]	[____|____|____|____]
[____|____|____|____]	[____|____|____|____]	[____|____|____|____]	[____|____|____|____]
[____|____|____|____]	[____|____|____|____]	[____|____|____|____]	[____|____|____|____]
[____|____|____|____]	[____|____|____|____]	[____|____|____|____]	[____|____|____|____]

TRAINER QUESTIONNAIRE

**1. Did the training start more than half an hour late?**	1 = Yes<br>2 = No	[____]
**2. If yes, what was the cause of the delay?**	1 = Participants arrived late<br>2 =Trainers arrived late<br>3 = Materials and supplies to be used were not delivered in time<br>4 = Trainer transport difficulties<br>5 = Other ________________________________	[____]<br>[____]<br>[____]

3. Please describe the consequence of the delay on your ability to deliver the training as intended

Training component	Consequence of delay on trainer's ability to deliver the training as intended
a) New topics introduced in the module	
b) Practice activities for completing the Stock Card, OPD Register and order form	

**4. What level of contribution did the participants have?**	1 = All contributed a lot<br>2 = All contributed at some point<br>3 = Only some contributed<br>4 = None contributed	[____]
**5. For Qn. 4, responses 2 and 3 please list the 3 least active participants.**<br>[____|____|____|____] [____|____|____|____] [____|____|____|____]		

HEALTH CENTRE MANAGEMENT TRAINING<br>SFQ for TRAINERS – MODULE: DRUG SUPPLY MANAGEMENT (HCM02)	
**Trainer Study ID**<br>[____|____]	**Date of training**<br>[____|____]/ [____|____]/[____|____]<br>day month year

6. Please complete the table below to describe how this training session went for each topic.

Topic	Teaching as planned?	Materials as planned?	If no, please explain
a) Principles of the drug distribution system	1 = Yes<br>2 = No<br>[____]	1 = Yes<br>2 = No<br>[____]	
b) Forms required in drug distribution cycle	1 = Yes<br>2 = No<br>[____]	1 = Yes<br>2 = No<br>[____]	
c) Keeping track of drug distribution activities	1 = Yes<br>2 = No<br>[____]	1 = Yes<br>2 = No<br>[____]	

7. Can you please describe any difficulties/barriers you encountered in delivery of the training?

<u>Barriers</u> to optimum implementation (e.g. - training interrupted, difficult or over bearing individuals, etc.)	<u>Impact</u> of barrier on the intended delivery of training and on learning

HEALTH CENTRE MANAGEMENT TRAINING<br>SFQ for TRAINERS – MODULE: DRUG SUPPLY MANAGEMENT (HCM02)	
**Trainer Study ID**<br>[____|____]	**Date of training**<br>[____|____]/ [____|____]/[____|____]<br>day month year

8. Please score how well you think the participants achieved each of the learning objectives today on a scale of 1-10.
(1=Not achieved, 10= Achieved in full. Use 'N/A' if the section is not included in topic)

Learning objectives	Score	Comments
a) Describe the main components of the drug distribution system		
b) Be motivated to actively participate in and keep the drug distribution system on track		
c) Describe the purpose and benefit of completing forms required in the drug distribution system including:<br>o OPD register<br>o Stock-card (Form 015)<br>o Order Form (Form 085)		
d) Accurately complete the forms required in the drug distribution system		
e) Put in place a plan for completing the forms regularly at the heath centre		
f) Identify the activities required to get drugs from the District or Health Sub-District to the health centre		
g) Identify challenges and solutions to completing drug distribution system activities		
h) Be motivated to keep track of health centre level activities in the drug distribution system		

9. Please list commitments or plans made by participants during the workshop today. (List the commitments made and in brackets, list the representatives of each health centre who made that commitment. For example "To put up pictorial signs at the health centre (HC03, 05 and 06)"

**10. How many questions or concerns were raised by this group about the topics covered today?**	1 = Many questions<br>2 = Few questions<br>3 = None	[_____]
**11. What questions or concerns were raised by this group about the topics discussed today? (Please list)**		
**12. How did you address each of these concerns in this group?**		
**13. Which of these concerns do you think were still present at the end of the training?**		
**14. Would you change anything about the session you gave to this group today? If yes, what would you change? How would you change it?**		
**15. What is your overall assessment of how well the intended objectives of today's training were achieved?**	1 = Badly<br>2 = Fine<br>3 = Good	[_____]

APPENDIX C: HEALTH CENTRE MANAGEMENT TRAINING
SFQ for TRAINERS – MODULE: HEALTH INFORMATION MANAGEMENT (HCM03)

'Thank you for assisting with this health centre management training! We would appreciate your feedback on the training session you delivered today. Please take a moment to answer the following questions. Your comments will help us to know what information this group received and how well they achieved our intended learning objectives during this session. This will help us to know how to interpret any future change of practice amongst these health workers. There is no need to write your name on this questionnaire, and all responses will be kept strictly confidential. Thank you!'

**Trainer Study ID**	**Date of training**	**Study ID of other Trainers present**
[____|____]	[____|____]/ [____|____]/[____|____]<br>day month year	[____|____], [____|____]
**Training group #**	**Total # of participants invited**	**Total # of participants attended**
[____|____]	[____|____]	[____|____]

Participant Study IDs

[____|____|____|____]	[____|____|____|____]	[____|____|____|____]	[____|____|____|____]
[____|____|____|____]	[____|____|____|____]	[____|____|____|____]	[____|____|____|____]
[____|____|____|____]	[____|____|____|____]	[____|____|____|____]	[____|____|____|____]
[____|____|____|____]	[____|____|____|____]	[____|____|____|____]	[____|____|____|____]

TRAINER QUESTIONNAIRE

**1. Did the training start more than half an hour late?**	1 = Yes<br>2 = No	[____]
**2. If yes, what was the cause of the delay?**	1 = Participants arrived late<br>2 =Trainers arrived late<br>3 = Materials and supplies to be used were not delivered in time<br>4 = Trainer transport difficulties<br>5= Other ____	[____]<br>[____]<br>[____]

3. Please describe the consequence of the delay on your ability to deliver the training as intended

Training component	Consequence of delay on trainer's ability to deliver the training as intended
a) New topics introduced in the module	
b) Practice activities for using information	
c) Planning activities for using information	

**4. What level of contribution did the participants have?**	1 = All contributed a lot<br>2 = All contributed at some point<br>3 = Only some contributed<br>4 = None contributed
**5. For Qn. 4, responses 2 and 3 please list the 3 least active participants.**<br>[____|____|____|____] [____|____|____|____] [____|____|____|____]	

HEALTH CENTRE MANAGEMENT TRAINING<br>SFQ for TRAINERS – MODULE: HEALTH INFORMATION MANAGEMENT (HCM03)	
**Trainer Study ID**<br>[____|____]	**Date of training**<br>[____|____]/ [____|____]/[____|____]<br>day month year

6. Please complete the table below to describe how this training session went for each topic.

Topic	Teaching as planned?	Materials as planned?	If no, please explain
a) **Why quality information matters**	1 = Yes<br>2 = No<br>[____]	1 = Yes<br>2 = No<br>[____]	
b) **The information cycle – from patient to patient**	1 = Yes<br>2 = No<br>[____]	1 = Yes<br>2 = No<br>[____]	

7. Can you please describe any difficulties/barriers you encountered in delivery of the training?

**Barriers to optimum implementation (e.g. - training interrupted, difficult or over bearing individuals, etc.)**	**Impact of barrier on the intended delivery of training and on learning**

8. Please score how well you think the participants achieved each of the learning objectives today on a scale of 1-10.
(1=Not achieved, 10= Achieved in full. Use 'N/A' if the section is not included in topic)

Learning objectives	Score	Comments
a) Understand why we collect patient information		
b) Understand how collecting information can beneficial to the health centre (drug quantification, predicting future needs)		
c) Understand how collecting information improves patient management		

HEALTH CENTRE MANAGEMENT TRAINING<br>SFQ for TRAINERS – MODULE: INFORMATION MANAGEMENT (HCM03)	
**Trainer Study ID**<br>[_____|_____]	**Date of training**<br>[_____|_____]/ [_____|_____]/[_____|_____]<br>day month year

**9. Please list commitments or plans made by participants during the workshop today. (List the commitments made and in brackets, list the representatives of each health centre who made that commitment. For example "To put up pictorial signs at the health centre (HC03, 05 and 06)"**	
**10. How many questions or concerns were raised by this group about the topics covered today?**	1 = Many questions<br>2 = Few questions<br>3 = None [_____]
**11. What questions or concerns were raised by this group about the topics discussed today? (Please list)**	
**12. How did you address each of these concerns in this group?**	
**13. Which of these concerns do you think were still present at the end of the training?**	
**14. Would you change anything about the session you gave to this group today? If yes, what would you change? How would you change it?**	
**15. What is your overall assessment of how well the intended objectives of today's training were achieved?**	1 = Badly<br>2 = Fine<br>3 = Good [_____]

APPENDIX D: HEALTH CENTRE MANAGEMENT TRAINING SFQ

PART 1: PARTICIPANT DEMOGRAPHIC BACKGROUND FORM

If you are attending multiple training modules, you only need to complete this form once.

Thank you for participating in this training course. We would like to collect some information about you and your work history. Please take a moment to answer the following questions. There is no need to write your name on this questionnaire. All responses will be kept strictly confidential. Thank you!

Please complete the boxes below with your own information. Please ask if you have any questions.

Participant PRIME Study ID [____|____]

Please complete the questions below

1. What is your age in years? [____|____] years

2. What is your gender? (please circle) Male Female

3. How long have you worked at this health centre? [____|____] and [____|____]
years months

4. If you are an in-charge, how long have you actively worked as an in-charge? [____|____] and [____|____]
years months

5. What is your education? Please circle all levels completed

Primary
Senior four
Senior six
Vocational certificate
University
Others *(please specify)*
____
____

6. What year did you completed your highest level of education (schooling)? [____|____|____|____]
year

7. What is your current position? Please select from the list below and write the appropriate number here: [____|____]

01 = Senior medical Officer	06 = Enrolled nurse	11 = Laboratory technician
02 = Medical Officer	07 = Comprehensive nurse	12 = Laboratory assistant
03 = Senior clinical Officer	08 = Midwife	13 = Health assistant
04 = Clinical Officer	09 = Public health nurse	14 = Health educator
05 = Nursing Officer	10 = Nursing aide/assistant	15 = Other ________________

8. What year did you start working in this position? [____|____|____|____]
year

9. What training workshops have you attended in the past 3 years? Please complete the table below

	**Title of training you attended**	**Organization**	**Date** [dd/mm/yy]
**9a**			[_____/_____/_____]
**9b**			[_____/_____/_____]
**9c**			[_____/_____/_____]

APPENDIX D: HEALTH CENTRE MANAGEMENT TRAINING
SFQ for PARTICIPANTS – PHC FUND MANAGEMENT (HCM01)

Thank you for participating in this health centre management training! We would appreciate your feedback on the training session you have attended. Please take a moment to answer the following questions. This is **NOT A TEST** and **WE VALUE YOUR OPINION**. Your opinions will help us improve future trainings. There is no need to write your name on this questionnaire.
All responses will be kept strictly confidential. Thank you!

GENERAL INSTRUCTIONS TO COMPLETE THIS QUESTIONNAIRE

1. *Please use a dark coloured pen to fill out the questionnaire*

2. *The health worker identity (ID) number is the unique number that was given to you at the start of this PRIME project. When we request you to give your ID number, please fill the boxes as below:*

   [_0_ | _5_ | _1_ | _0_]

   You are asked to enter your ID number like this at the top of each page of your questionnaire.

3. *In this questionnaire, we ask you to read each question carefully and either write your response, or look at the statements and decide which response is closest to your own opinion. When you have decided, please circle the number in the column under that response. For example:*

Decide how much you agree with the statements below	***Strongly agree***	***Agree***	***Disagree***	***Strongly disagree***
I learned new ideas today	*(1)*	*2*	*3*	*4*

4. *If you change your mind and would like to circle a different response, please cross out your original choice and circle the choice that fits your opinion best. For example, if you change your mind and you decide that your answer is 'disagree', cross through the original and circle the new response as below:*

Decide how much you agree with the statements below	***Strongly agree***	***Agree***	***Disagree***	***Strongly disagree***
I learned new ideas today	*~~(1)~~*	*2*	*(3)*	*4*

5. *When you have completed the questionnaire, please make sure you have completed everything in the following checklist:*

For the first time you attend a workshop <u>only</u>:	☐ *[For the first time only] I have signed and dated the consent form*<br>☐ *[For the first time only] I have completed my demographic details form*
For every workshop:	☐ *I have answered all of the questions in this questionnaire*<br>☐ *I have checked that I have circled the responses that are closest to my opinion*<br>☐ *I have written my health worker ID number on all of the pages of the questionnaire*<br>☐ *I have written my health worker ID number on the envelope and will place this completed form inside the envelope*

HEALTH CENTRE MANAGEMENT TRAINING<br>SFQ for PARTICIPANTS – PHC FUND MANAGEMENT (HCM01)	
**Health worker ID**<br>[____|____|____|____]	**Today's date**<br>[____|____]/ [____|____]/[____|____]<br>day month year

1. **Please complete the table below about your <u>learning today</u> by circling the response closest to your opinion.**

Decide how much you agree with the statements below	Strongly agree	Agree	Disagree	Strongly disagree
I learned new ideas today	1	2	3	4
I understood everything the trainer taught today	1	2	3	4
I felt the training was relevant to my daily work	1	2	3	4
The ideas I learned today will be useful for me in the future	1	2	3	4
I was given a chance to ask all of my questions	1	2	3	4
I felt that the trainer answered all our questions well	1	2	3	4
I felt able to practice new skills in the workshop today	1	2	3	4
I was given a chance to give my opinions	1	2	3	4
I will recommend my colleagues to attend this training	1	2	3	4

If you have any comments on the above statements, Please write them below:

HEALTH CENTRE MANAGEMENT TRAINING<br>SFQ for PARTICIPANTS – PHC FUND MANAGEMENT (HCM01)	
**Health worker ID**<br>[____|____|____|____]	**Today's date**<br>[____|____]/ [____|____]/[____|____]<br>day month year

2. Please complete the table below about the skills you have learnt from <u>the training topic</u>, by circling the response closest to your opinion.

PLEASE REMEMBER, THERE IS NO RIGHT OR WRONG ANSWER!

Decide how much you agree with the statements below	Strongly agree	Agree	Disagree	Strongly disagree
This training helped me to think about accountability	1	2	3	4
This training has helped me to see the importance of budgeting and accounting for the PHC Fund	1	2	3	4
This training has given me ideas for how to show accountability in my work as an in-charge	1	2	3	4
After this training, I feel able to change the way I manage PHC Funds at my health centre	1	2	3	4
After this training, I have found ways to manage funds at my health centre using the PHC Fund Management Tool	1	2	3	4

Please write any comments on the above statements in this section here:

HEALTH CENTRE MANAGEMENT TRAINING<br>SFQ for PARTICIPANTS – PHC FUND MANAGEMENT (HCM01)	
**Health worker ID**	**Today's date**
[____|____|____|____]	[____|____]/ [____|____]/[____|____]<br>day month year

3. Please complete the table below about today's <u>overall training</u> by circling the response closest to your opinion.

	Very good	Good	Fair	Poor	Very poor
a) What is your opinion of trainer 1's skills today?	1	2	3	4	5
b) What is your opinion of trainer 1's attitude today?	1	2	3	4	5
c) What is your opinion of trainer 2's skills today?	1	2	3	4	5
d) What is your opinion of trainer 2's attitude today?	1	2	3	4	5
e) What is your opinion of the support given by the training assistant today?	1	2	3	4	5
f) What is your opinion about the discussions you had in your small groups today?	1	2	3	4	5
g) What is your opinion of the learner's manual you received today?	1	2	3	4	5
h) What is your opinion of the flip charts that were used today?	1	2	3	4	5
i) Overall, what is your opinion of the training today?	1	2	3	4	5

4. Please write anything else you would like to say about today's training

NOW PLEASE ENSURE YOU HAVE COMPLETED THIS FORM FULLY BY GOING THROUGH THE <u>CHECK SHEET</u> ON THE FRONT PAGE

APPENDIX D: HEALTH CENTRE MANAGEMENT TRAINING

SFQ for PARTICIPANTS – DRUG SUPPLY MANAGEMENT(HCM02)

Thank you for participating in this health centre management training! We would appreciate your feedback on the training session you have attended. Please take a moment to answer the following questions. This is **NOT A TEST** and **WE VALUE YOUR OPINION**. Your opinions will help us improve future trainings. There is no need to write your name on this questionnaire.
All responses will be kept strictly confidential. Thank you!

GENERAL INSTRUCTIONS TO COMPLETE THIS QUESTIONNAIRE

6. *Please use a dark coloured pen to fill out the questionnaire*

7. *The health worker identity (ID) number is the unique number that was given to you at the start of this PRIME project. When we request you to give your ID number, please fill the boxes as below:*

   [_0_ | _5_ | _1_ | _0_]

   You are asked to enter your ID number like this at the top of each page of your questionnaire.

8. *In this questionnaire, we ask you to read each question carefully and either write your response, or look at the statements and decide which response is closest to your own opinion. When you have decided, please circle the number in the column under that response. For example:*

Decide how much you agree with the statements below	***Strongly agree***	***Agree***	***Disagree***	***Strongly disagree***
I learned new ideas today	*(1)*	*2*	*3*	*4*

9. *If you change your mind and would like to circle a different response, please cross out your original choice and circle the choice that fits your opinion best. For example, if you change your mind and you decide that your answer is 'disagree', cross through the original and circle the new response as below:*

Decide how much you agree with the statements below	***Strongly agree***	***Agree***	***Disagree***	***Strongly disagree***
I learned new ideas today	*~~(1)~~*	*2*	*(3)*	*4*

10. *When you have completed the questionnaire, please make sure you have completed everything in the following checklist:*

For the first time you attend a workshop only:	☐ *[For the first time only] I have signed and dated the consent form*<br>☐ *[For the first time only] I have completed my demographic details form*
For every workshop:	☐ *I have answered all of the questions in this questionnaire*<br>☐ *I have checked that I have circled the responses that are closest to my opinion*<br>☐ *I have written my health worker ID number on all of the pages of the questionnaire*<br>☐ *I have written my health worker ID number on the envelope and will place this completed form inside the envelope*

HEALTH CENTRE MANAGEMENT TRAINING<br>SFQ for PARTICIPANTS – DRUG SUPPLY MANAGEMENT(HCM02)	
**Health worker ID**	**Today's date**
[____|____|____|____]	[____|____]/ [____|____]/[____|____]<br>day month year

1. **Please complete the table below about your <u>learning today</u> by circling the response closest to your opinion.**

Decide how much you agree with the statements below	Strongly agree	Agree	Disagree	Strongly disagree
I learned new ideas today	1	2	3	4
I understood everything the trainer taught today	1	2	3	4
I felt the training was relevant to my daily work	1	2	3	4
The ideas I learned today will be useful for me in the future	1	2	3	4
I was given a chance to ask all of my questions	1	2	3	4
I felt that the trainer answered all our questions well	1	2	3	4
I felt able to practice new skills in the workshop today	1	2	3	4
I was given a chance to give my opinions	1	2	3	4
I will recommend my colleagues to attend this training	1	2	3	4
**If you have any comments on the above statements, Please write them below:**				

HEALTH CENTRE MANAGEMENT TRAINING<br>SFQ for PARTICIPANTS – DRUG SUPPLY MANAGEMENT(HCM02)	
**Health worker ID**<br>[____|____|____|____]	**Today's date**<br>[____|____]/ [____|____]/[____|____]<br>day month year

2. Please complete the table below about the skills you have learnt from <u>the training topic</u>, by circling the response closest to your opinion.

PLEASE REMEMBER, THERE IS NO RIGHT OR WRONG ANSWER!

Decide how much you agree with the statements below	Strongly agree	Agree	Disagree	Strongly disagree
This training helped me to think about my role as an in-charge in the drug distribution system	1	2	3	4
This training has helped me to see the importance of doing my part to keep the drug distribution system on track	1	2	3	4
This training has given me ideas for how to make sure drugs reach my health centre	1	2	3	4
After this training, I feel able to change the way I manage drugs at my health centre	1	2	3	4
After this training, I have found ways to complete the OPD register, stock-card and order form	1	2	3	4

Please write any comments on the above statements in this section here:

HEALTH CENTRE MANAGEMENT TRAINING<br>SFQ for PARTICIPANTS – DRUG SUPPLY MANAGEMENT(HCM02)	
**Health worker ID**<br>[____|____|____|____]	**Today's date**<br>[____|____]/ [____|____]/[____|____]<br>day month year

3. Please complete the table below about today's <u>overall training</u> by circling the response closest to your opinion.

	Very good	Good	Fair	Poor	Very poor
a) What is your opinion of trainer 1's skills today?	1	2	3	4	5
b) What is your opinion of trainer 1's attitude today?	1	2	3	4	5
c) What is your opinion of trainer 2's skills today?	1	2	3	4	5
d) What is your opinion of trainer 2's attitude today?	1	2	3	4	5
e) What is your opinion of the support given by the training assistant today?	1	2	3	4	5
f) What is your opinion about the discussions you had in your small groups today?	1	2	3	4	5
g) What is your opinion of the learner's manual you received today?	1	2	3	4	5
h) What is your opinion of the flip charts that were used today?	1	2	3	4	5
i) Overall, what is your opinion of the training today?	1	2	3	4	5

4. Please write anything else you would like to say about today's training

NOW PLEASE ENSURE YOU HAVE COMPLETED THIS FORM FULLY BY GOING THROUGH THE <u>CHECK SHEET</u> ON THE FRONT PAGE

APPENDIX D: HEALTH CENTRE MANAGEMENT TRAINING
SFQ for PARTICIPANTS – HEALTH INFORMATION MANAGEMENT (HCM03)

Thank you for participating in this health centre management training! We would appreciate your feedback on the training session you have attended. Please take a moment to answer the following questions. This is **NOT A TEST** and **WE VALUE YOUR OPINION**. Your opinions will help us improve future trainings. There is no need to write your name on this questionnaire.
All responses will be kept strictly confidential. Thank you!

GENERAL INSTRUCTIONS TO COMPLETE THIS QUESTIONNAIRE

11. *Please use a dark coloured pen to fill out the questionnaire*

12. *The health worker identity (ID) number is the unique number that was given to you at the start of this PRIME project. When we request you to give your ID number, please fill the boxes as below:*

    [_0_ | _5_ | _1_ | _0_]

    You are asked to enter your ID number like this at the top of each page of your questionnaire.

13. *In this questionnaire, we ask you to read each question carefully and either write your response, or look at the statements and decide which response is closest to your own opinion. When you have decided, please circle the number in the column under that response. For example:*

Decide how much you agree with the statements below	***Strongly agree***	***Agree***	***Disagree***	***Strongly disagree***
I learned new ideas today	*(1)*	*2*	*3*	*4*

14. *If you change your mind and would like to circle a different response, please cross out your original choice and circle the choice that fits your opinion best. For example, if you change your mind and you decide that your answer is 'disagree', cross through the original and circle the new response as below:*

Decide how much you agree with the statements below	***Strongly agree***	***Agree***	***Disagree***	***Strongly disagree***
I learned new ideas today	*~~(1)~~*	*2*	*(3)*	*4*

15. *When you have completed the questionnaire, please make sure you have completed everything in the following checklist:*

For the first time you attend a workshop only:	☐ *[For the first time only] I have signed and dated the consent form*<br>☐ *[For the first time only] I have completed my demographic details form*
For every workshop:	☐ *I have answered all of the questions in this questionnaire*<br>☐ *I have checked that I have circled the responses that are closest to my opinion*<br>☐ *I have written my health worker ID number on all of the pages of the questionnaire*<br>☐ *I have written my health worker ID number on the envelope and will place this completed form inside the envelope*

HEALTH CENTRE MANAGEMENT TRAINING<br>SFQ for PARTICIPANTS – HEALTH INFORMATION MANAGEMENT (HCM03)	
**Health worker ID**<br>[____|____|____|____]	**Today's date**<br>[____|____]/ [____|____]/[____|____]<br>day month year

1. **Please complete the table below about your <u>learning today</u> by circling the response closest to your opinion.**

Decide how much you agree with the statements below	Strongly agree	Agree	Disagree	Strongly disagree
I learned new ideas today	1	2	3	4
I understood everything the trainer taught today	1	2	3	4
I felt the training was relevant to my daily work	1	2	3	4
The ideas I learned today will be useful for me in the future	1	2	3	4
I was given a chance to ask all of my questions	1	2	3	4
I felt that the trainer answered all our questions well	1	2	3	4
I felt able to practice new skills in the workshop today	1	2	3	4
I was given a chance to give my opinions	1	2	3	4
I will recommend my colleagues to attend this training	1	2	3	4
**If you have any comments on the above statements, Please write them below:**				

HEALTH CENTRE MANAGEMENT TRAINING<br>SFQ for PARTICIPANTS – HEALTH INFORMATION MANAGEMENT (HCM03)	
**Health worker ID**<br>[____|____|____|____]	**Today's date**<br>[____|____]/ [____|____]/[____|____]<br>day month year

2. **Please complete the table below about the skills you have learnt from <u>the training topic</u>, by circling the response closest to your opinion.**

PLEASE REMEMBER, THERE IS NO RIGHT OR WRONG ANSWER!

Decide how much you agree with the statements below	Strongly agree	Agree	Disagree	Strongly disagree
This training helped me to think about why we collect patient information	1	2	3	4
This training has helped me to see how collecting information can be beneficial to the health centre	1	2	3	4
This training has helped me to understand how collecting information improves patient management	1	2	3	4
**Please write any comments on the above statements in this section here:**				

HEALTH CENTRE MANAGEMENT TRAINING<br>SFQ for PARTICIPANTS – HEALTH INFORMATION MANAGEMENT (HCM03)	
**Health worker ID**	**Today's date**
[____|____|____|____]	[____|____]/ [____|____]/[____|____]<br>day month year

3. Please complete the table below about today's <u>overall training</u> by circling the response closest to your opinion.

	Very good	Good	Fair	Poor	Very poor
a) What is your opinion of trainer 1's skills today?	1	2	3	4	5
b) What is your opinion of trainer 1's attitude today?	1	2	3	4	5
c) What is your opinion of trainer 2's skills today?	1	2	3	4	5
d) What is your opinion of trainer 2's attitude today?	1	2	3	4	5
e) What is your opinion of the support given by the training assistant today?	1	2	3	4	5
f) What is your opinion about the discussions you had in your small groups today?	1	2	3	4	5
g) What is your opinion of the learner's manual you received today?	1	2	3	4	5
h) What is your opinion of the flip charts that were used today?	1	2	3	4	5
i) Overall, what is your opinion of the training today?	1	2	3	4	5

4. Please write anything else you would like to say about today's training

NOW PLEASE ENSURE YOU HAVE COMPLETED THIS FORM FULLY
BY GOING THROUGH THE <u>CHECK SHEET</u> ON THE FRONT PAGE

APPENDIX E: PATIENT CENTRED SERVICES SFQs

PART 1: TRAINER DEMOGRAPHIC BACKGROUND FORM

If you are leading multiple training modules, you only need to complete this form once.

Thank you for assisting with the delivery of these training workshops. Please take a moment to answer the following questions. There is no need to write your name on this questionnaire, and all responses will be kept strictly confidential. Thank you!

Trainer Study ID [____|____]

**Please complete the boxes below with the relevant numbers**	
**1. Age in years** [____|____]	**2. Gender** 1 = Male<br>2 = Female [____]

3. Qualifications [____|____]

01 = Senior medical Officer	06 = Enrolled nurse	11 = Laboratory technician
02 = Medical Officer	07 = Comprehensive nurse	12 = Laboratory assistant
03 = Senior clinical Officer	08 = Midwife	13 = Health assistant
04 = Clinical Officer	09 = Public health nurse	14 = Health educator
05 = Nursing Officer	10 = Nursing aide/assistant	15 = Other ________________________________

4. What training courses have you delivered in the past 3 years?

	**Title of training delivered**	**Organization**	**Dates** [dd/mm/yy] to [dd/mm/yy]
**4a**			[_____/_____/_____]<br>to [_____/_____/_____]
**4b**			[_____/_____/_____]<br>to [_____/_____/_____]
**4c**			[_____/_____/_____]<br>to [_____/_____/_____]

5. What training courses have you attended in training methods?

	**Title of training you attended**	**Organization**	**Dates** [dd/mm/yy]
**5a**			[_____/_____/_____]
**5b**			[_____/_____/_____]
**5c**			[_____/_____/_____]

Please continue overleaf if you have delivered/attended more than 3 training workshops.

APPENDIX F: FEVER CASE MANAGEMENT TRAINING SFQ for PARTICIPANTS (1)

Thank you for participating in this training on staffing! We would appreciate your feedback on the training sessions you have attended. Please take a moment to answer the following questions, as your comments will help us improve future trainings. There is no need to write your name on this questionnaire, and all responses will be kept strictly confidential. Thank you!

Health worker ID	Today's date	Date training began
[____]	[____|____]/ [____|____]/[____|____] day month year	[____|____]/ [____|____]/[____|____] day month year

1. Your qualification [____]	1 = Clinical Officer<br>2 = Nurse<br>3 = Other (list) ____________________	2. What is your age? [____|____] years	3. How long have you worked actively as health centre in-charge? [____|____] OR [____|____] months years

4. How many trainings have you attended for health centre clinical staff in the past? [____|____]	5. When was the last training you attended and what was the topic of training? Topic [___|___]/ [___|___]/[___|___] day month year

6. What other PRIME courses have you attended so far?

TRAINEE QUESTIONNAIRE

Please summarise your opinion of the course by ranking the following using: 1 = Poor, 2 = Fair, 3 = Good, 4 = Very good, 5 = N/A

**7. Achievement of your aims when you enrolled in this training**	[____]	**10. Use of Training Aids (if applicable)**	[____]
**8. General achievements of the course objectives**	[____]	**11. Your overall assessment**	[____]
**9. Effectiveness of Trainer(s)**	[____]		

12. How interested do you think most of the other clinical staff in your group were throughout the training sessions? 1 = Not Very Interested, 2 = Somewhat Interested, 3 = Very Interested [____]

How useful did you find each of the following training activities? 1 = Not very useful, 2 = Somewhat useful, 3 = Very useful, 4 = Don't know

**13. Discussion of evaluation of febrile patients and selection of patients for RDT testing**	[____]	**17. Discussion of recognition and referral of patients with severe illness**	[____]
**14. Practice of performing and reading an RDT**	[____]	**18. Discussion of patient education**	[____]
**15. Discussion of management of a patient with fever and a positive RDT**	[____]	**19. Discussion of RDT storage and monitoring**	[____]
**16. Discussion of management of a patient with fever and a negative RDT**	[____]		[____]

15. What would you like to add or change about the training sessions?

16. Please write any concerns you have about fever case management or any other comments following up on this training?

17. Please write any general comments you have on this course?

FEVER CASE MANAGEMENT TRAINING
SFQ for PARTICIPANTS (2)

HW Study ID	Date of training
[____|____]	[____|____]/ [____|____]/[____|____]<br>day month year

Please summarise your opinion of the course by ranking the following using:

1 = Poor 2 = Fair 3 = Good 4 = Very good 5 = N/A

**18. I feel confident that I can do good history taking including asking good questions and active listening**	[____]	**24. I feel confident that I can manage the common non-malaria febrile illnesses according to treatment guidelines**	[____]
**19. I feel confident that I can perform a clinical examination on a patient with fever correctly**	[____]	**25. I feel confident that I can assess a patient for severe signs of illness**	[____]
**20. I feel confident that I can select a patient for RDT testing based on clinical evaluation**	[____]	**26. I feel confident that I can properly refer a patient when they are severely ill to higher level facilities**	[____]
**21. I feel confident that I can perform an RDT 22. correctly and safely**	[____]	**27. I feel confident that I can provide pre-referral treatments to severely ill patients**	[____]
**22. I feel confident that I can treat a patient with fever and a positive RDT according to national guidelines**	[____]	**28. I feel confident that I can use good communication skills when giving patients information about malaria and its treatment**	[____]
**23. I feel confident that I can manage a patient with fever but a negative RDT**	[____]	**29. I feel confident that I can store and monitor RDTs' expiry dates correctly**	[____]

Thank you!

APPENDIX G: PATIENT CENTRED SERVICES SFQs

PART 1: TRAINER DEMOGRAPHIC BACKGROUND FORM

If you are leading multiple training modules, you only need to complete this form once.

Thank you for assisting with the delivery of these training workshops. Please take a moment to answer the following questions. There is no need to write your name on this questionnaire, and all responses will be kept strictly confidential. Thank you!

Trainer Study ID [____|____]

Please complete the boxes below with the relevant numbers

**1. Age in years** [____|____]	**2. Gender** 1 = Male 2 = Female [____]

3. Qualifications [____|____]

01 = Senior medical Officer	06 = Enrolled nurse	11 = Laboratory technician
02 = Medical Officer	07 = Comprehensive nurse	12 = Laboratory assistant
03 = Senior clinical Officer	08 = Midwife	13 = Health assistant
04 = Clinical Officer	09 = Public health nurse	14 = Health educator
05 = Nursing Officer	10 = Nursing aide/assistant	15 = Other ________________

4. What training courses have you delivered in the past 3 years?

	**Title of training delivered**	**Organization**	**Dates** [dd/mm/yy] to [dd/mm/yy]
**4a**			[_____/_____/_____] to [_____/_____/_____]
**4b**			[_____/_____/_____] to [_____/_____/_____]
**4c**			[_____/_____/_____] to [_____/_____/_____]

5. What training courses have you attended in training methods?

	**Title of training you attended**	**Organization**	**Dates** [dd/mm/yy]
**5a**			[_____/_____/_____]
**5b**			[_____/_____/_____]
**5c**			[_____/_____/_____]

Please continue overleaf if you have delivered/attended more than 3 training workshops.

APPENDIX G: PATIENT CENTRED SERVICES
SFQ for TRAINERS – INTRODUCTION TO PCS & SOAs (PCS00)

'Thank you for assisting with this Patient Centered Services training! We would appreciate your feedback on the training session you delivered today. Please take a moment to answer the following questions. Your comments will help us to know what information this group received and how well they achieved our intended learning objectives during this session. This will help us to know how to interpret any future change of practice amongst these health workers. There is no need to write your name on this questionnaire, and all responses will be kept strictly confidential. Thank you!'

ID for Trainer 1 [____|____] ID for Trainer 2 [____|____]	Date of training [____|____]/ [____|____]/[____|____] day month year	Study ID of other Trainers present [____|____], [____|____]
**Training group #** [____|____]	**Total # of participants invited** [____|____]	**Total # of participants attended** [____|____]

Participant Study IDs			
[____|____|____|____]	[____|____|____|____]	[____|____|____|____]	[____|____|____|____]
[____|____|____|____]	[____|____|____|____]	[____|____|____|____]	[____|____|____|____]
[____|____|____|____]	[____|____|____|____]	[____|____|____|____]	[____|____|____|____]
[____|____|____|____]	[____|____|____|____]	[____|____|____|____]	[____|____|____|____]

TRAINER QUESTIONNAIRE		
**1. Did the training start more than half an hour late?**	1 = Yes<br>2 = No	[____]
**2. If yes, what was the cause of the delay?**	1 = Participants arrived late<br>2 =Trainers arrived late<br>3 = Materials and supplies to be used were not delivered in time<br>4 = Trainer transport difficulties<br>5 = Other ____	[____]<br>[____]<br>[____]

3. Please describe the consequence of the delay on your ability to deliver the training as intended

Training component	Consequence of delay on trainer's ability to deliver the training as intended
**Introduction to the self-observation activities**	
**New topics introduced in the module**	
**Introduction to the first self-observation activity**	

**4. What level of contribution did the participants have?**	1 = All contributed a lot<br>2 = All contributed at some point<br>3 = Only some contributed<br>4 = None contributed	[____]
**5. For Qn. 4, responses 2 and 3 please list the 3 least active participants.**<br>[____|____|____|____] [____|____|____|____] [____|____|____|____]		

PATIENT CENTRED SERVICES<br>SFQ for TRAINERS – INTRODUCTION TO PCS & SOAs (PCS00)	
**Trainer Study ID**<br>[____|____]	**Date of training**<br>[____|____]/ [____|____]/[____|____]<br>day month year

6. Please complete the table below to describe how this training session went for each topic.

Topic	Teaching as planned?	Materials as planned?	If no, please explain
**Thinking about my role as a health worker**	1 = Yes<br>2 = No<br>[____]	1 = Yes<br>2 = No<br>[____]	
**Introduction to PCS**	1 = Yes<br>2 = No<br>[____]	1 = Yes<br>2 = No<br>[____]	
**Introduction to Self Observation Activities**	1 = Yes<br>2 = No<br>[____]	1 = Yes<br>2 = No<br>[____]	

7. Can you please describe any difficulties/barriers you encountered in delivery of the training?

**Barriers to optimum implementation (e.g. - training interrupted, difficult or over bearing individuals, etc.)**	**Impact of barrier on the intended delivery of training and on learning**

8. Please score how well you think the participants achieved each of the learning objectives today on a scale of 1-10.
(1=Not achieved, 10= Achieved in full. Use 'N/A' if the section is not included in topic)

By the end of today's session, to what extent did the participants learn to...	Score	Comments
Identify what is important to them about being a health worker		
Understand the meaning and importance of providing patient centred services		
Start developing self-awareness through self-observation activities		

PATIENT CENTRED SERVICES<br>SFQ for TRAINERS – INTRODUCTION TO PCS & SOAs (PCS00)	
**Trainer Study ID**<br>[____|____]	**Date of training**<br>[____|____]/ [____|____]/[____|____]<br>day month year

**9. Please list commitments or plans made by participants during the workshop today.**<br>*List the commitments made, and in brackets, list the representatives of each health centre who made that commitment. For example "To put up pictorial signs at the health centre (HC #03, HC #05 and HC #06)"*	
**10. How many questions or concerns were raised by this group about the topics covered today?**	1 = Many questions<br>2 = Few questions<br>3 = None [____]
**11. What questions or concerns were raised by this group about the topics discussed today? (Please list)**	
**12. How did you address each of these concerns in this group?**	
**13. Which of these concerns do you think were still present at the end of the training?**	
**14. Would you change anything about the session you gave to this group today? If yes, what would you change? How would you change it?**	
**15. What is your overall assessment of how well the intended objectives of today's training were achieved?**	1 = Badly<br>2 = Fine<br>3 = Good [____]

APPENDIX G: PATIENT CENTRED SERVICES
SFQ for TRAINERS – COMMUNICATIONS SKILLS PART I (PCS01)

'Thank you for assisting with this Patient Centered Services training! We would appreciate your feedback on the training session you delivered today. Please take a moment to answer the following questions. Your comments will help us to know what information this group received and how well they achieved our intended learning objectives during this session. This will help us to know how to interpret any future change of practice amongst these health workers. There is no need to write your name on this questionnaire, and all responses will be kept strictly confidential. Thank you!'

**ID for Trainer 1** [____|____]<br>**ID for Trainer 2** [____|____]	**Date of training**<br>[____|____]/ [____|____]/[____|____]<br>day month year	**Study ID of other Trainers present**<br>[____|____], [____|____]
**Training group #**<br>[____|____]	**Total # of participants invited**<br>[____|____]	**Total # of participants attended**<br>[____|____]

**Participant Study IDs**			
[____|____|____|____]	[____|____|____|____]	[____|____|____|____]	[____|____|____|____]
[____|____|____|____]	[____|____|____|____]	[____|____|____|____]	[____|____|____|____]
[____|____|____|____]	[____|____|____|____]	[____|____|____|____]	[____|____|____|____]
[____|____|____|____]	[____|____|____|____]	[____|____|____|____]	[____|____|____|____]

TRAINER QUESTIONNAIRE		
**1. Did the training start more than half an hour late?**	1 = Yes<br>2 = No	[____]
**2. If yes, what was the cause of the delay?**	1 = Participants arrived late<br>2 =Trainers arrived late<br>3 = Materials and supplies to be used were not delivered in time<br>4 = Trainer transport difficulties<br>5 = Other ______________________________	[____]<br>[____]<br>[____]

3. Please describe the consequence of the delay on your ability to deliver the training as intended

Training component	Consequence of delay on trainer's ability to deliver the training as intended
**Reflection on self-observation activity**	
**New topics introduced in the module**	
**Introduction of the next self-observation activity**	

**4. What level of contribution did the participants have?**	1 = All contributed a lot<br>2 = All contributed at some point<br>3 = Only some contributed<br>4 = None contributed	[____]
**5. For Qn. 4, responses 2 and 3 please list the 3 least active participants.**		
[____|____|____|____]	[____|____|____|____]	[____|____|____|____]

PATIENT CENTRED SERVICES<br>SFQ for TRAINERS – COMMUNICATIONS SKILLS PART I (PCS01)	
**Trainer Study ID**<br>[____|____]	**Date of training**<br>[____|____]/ [____|____]/[____|____]<br>day month year

6. Please complete the table below to describe how this training session went for each topic.

Topic	Teaching as planned?	Materials as planned?	If no, please explain
**Building Rapport**	1 = Yes<br>2 = No<br>[____]	1 = Yes<br>2 = No<br>[____]	
**Active listening**	1 = Yes<br>2 = No<br>[____]	1 = Yes<br>2 = No<br>[____]	

7. Can you please describe any difficulties/barriers you encountered in delivery of the training?

**Barriers to optimum implementation (e.g. - training interrupted, difficult or over bearing individuals, etc.)**	**Impact of barrier on the intended delivery of training and on learning**

PATIENT CENTRED SERVICES<br>SFQ for TRAINERS – COMMUNICATIONS SKILLS PART I (PCS01)	
**Trainer Study ID**<br>[_____|_____]	**Date of training**<br>[_____|_____]/ [_____|_____]/[_____|_____]<br>day month year

8.0 Please reflect on the uptake and usefulness of the SOA this week for your participants.

**8.1 How many participants do you think completed the SOAs in full this week?**
**8.2 How many participants do you think partially completed the SOAs this week?**
**8.3 Please list any participants who you think did not do the SOAs at all this week**
**8.4 To what extent did the SOA help participants to think about today's topic in advance? (a lot / somewhat / not much)**
**8.5 Please give examples of specific insights from the SOAs shared this week that you think had a significant impact on others in the group in terms of their own uptake of the ideas (describe the concept, story, and ID number(s) of those involved)**
**8.6 In your opinion, how important was the SOA to today's learning in this group? (essential / take it or leave it / unhelpful)**

9. Please score how well you think the participants achieved each of the learning objectives today on a scale of 1-10.
(1=Not achieved, 10= Achieved in full. Use 'N/A' if the section is not included in topic)

By the end of today's session, to what extent did the participants learn to...	Score	Comments
Recognise the impact of non-verbal and verbal behaviour on the patient and consultation outcome.		
Strengthen non-verbal and verbal skills in building rapport.		
Recognise that we think of different people in different ways, and this affects how we behave towards them.		
Understand that respect is a core value for how we can put patients at ease.		
Strengthen skills to show respect to patients		
Strengthen skills in self-reflection		
Strengthen non-verbal and verbal skills in active listening.		
Recognise the consequences of listening well, and less well, on the patient and consultation outcome.		
Identify ways to listen actively in spite of busy work environments.		

10. Please list commitments or plans made by participants during the workshop today.
(List the commitments made and in brackets, list the representatives of each health centre who made that commitment. For example "To put up pictorial signs at the health centre (HC #03, HC #05 and HC #06)"

**11. How many questions or concerns were raised by this group about the topics covered today?**	1 = Many questions<br>2 = Few questions<br>3 = None [____]
**12. What questions or concerns were raised by this group about the topics discussed today? (Please list)**	
**13. How did you address each of these concerns in this group?**	
**14. Which of these concerns do you think were still present at the end of the training?**	
**15. Would you change anything about the session you gave to this group today? If yes, what would you change? How would you change it?**	
**16. What is your overall assessment of how well the intended objectives of today's training were achieved?**	1 = Badly<br>2 = Fine<br>3 = Good [____]

APPENDIX G: PATIENT CENTRED SERVICES
SFQ for TRAINERS – COMMUNICATION SKILLS PART II (PCS02)

'Thank you for assisting with this patient centered services training! We would appreciate your feedback on the training session you delivered today. Please take a moment to answer the following questions. Your comments will help us to know what information this group received and how well they achieved our intended learning objectives during this session. This will help us to know how to interpret any future change of practice amongst these health workers. There is no need to write your name on this questionnaire, and all responses will be kept strictly confidential. Thank you!'

ID for Trainer 1 [____|____] ID for Trainer 2 [____|____]	Date of training [____|____]/ [____|____]/[____|____] day month year	Study ID of other Trainers present [____|____], [____|____]
Training group # [____|____]	Total # of participants invited [____|____]	Total # of participants attended [____|____]

Participant Study IDs			
[____|____|____|____]	[____|____|____|____]	[____|____|____|____]	[____|____|____|____]
[____|____|____|____]	[____|____|____|____]	[____|____|____|____]	[____|____|____|____]
[____|____|____|____]	[____|____|____|____]	[____|____|____|____]	[____|____|____|____]
[____|____|____|____]	[____|____|____|____]	[____|____|____|____]	[____|____|____|____]

TRAINER QUESTIONNAIRE

**1. Did the training start more than half an hour late?**	1 = Yes<br>2 = No	[____]
**2. If yes, what was the cause of the delay?**	1 = Participants arrived late<br>2 = Trainers arrived late<br>3 = Materials and supplies to be used were not delivered in time<br>4 = Trainer transport difficulties<br>5 = Other ____	[____]<br>[____]<br>[____]

3. Please describe the consequence of the delay on your ability to deliver the training as intended

Training component	Consequence of delay on trainer's ability to deliver the training as intended
**Reflection on self-observation activity**	
**New topics introduced in the module**	
**Introduction of the next self-observation activity**	

**4. What level of contribution did the participants have?**	1 = All contributed a lot<br>2 = All contributed at some point<br>3 = Only some contributed<br>4 = None contributed	[____]
**5. For Qn. 4, responses 2 and 3 please list the 3 least active participants.** [____|____|____|____] [____|____|____|____] [____|____|____|____]		

PATIENT CENTRED SERVICES
SFQ for TRAINERS – COMMUNICATION SKILLS PART II (PCS02)

Trainer Study ID	Date of training
[____|____]	[____|____]/ [____|____]/[____|____]<br>day month year

6. Please complete the table below to describe how this training session went for each topic.

Topic	Teaching as planned?	Materials as planned?	If no, please explain
**Asking good questions**	1 = Yes<br>2 = No<br>[____]	1 = Yes<br>2 = No<br>[____]	
**Giving good information**	1 = Yes<br>2 = No<br>[____]	1 = Yes<br>2 = No<br>[____]	

7. Can you please describe any difficulties/barriers you encountered in delivery of the training?

**Barriers** to optimum implementation (e.g. - training interrupted, difficult or over bearing individuals, etc.)	**Impact** of barrier on the intended delivery of training and on learning

8.0 Please reflect on the uptake and usefulness of the SOA this week for your participants.

**8.1 How many participants do you think completed the SOAs in full this week?**
**8.2 How many participants do you think partially completed the SOAs this week?**
**8.3 Please list any participants who you think did not do the SOAs at all this week**

**8.4 To what extent did the SOA help participants to think about today's topic in advance? (a lot / somewhat / not much)**
**8.5 Please give examples of specific insights from the SOAs shared this week that you think had a significant impact on others in the group in terms of their own uptake of the ideas (describe the concept, story, and ID number(s) of those involved)**
**8.6 In your opinion, how important was the SOA to today's learning in this group? (essential / take it or leave it / unhelpful)**

9. Please score how well you think the participants achieved each of the learning objectives today on a scale of 1-10.
(1=Not achieved, 10= Achieved in full. Use 'N/A' if the section is not included in topic)

By the end of today's session, to what extent did the participants learn to...	Score	Comments
Understand the importance of getting good information		
Be aware of the way and consequences of how they ask questions		
Know how to formulate open questions		
Ask questions without showing judgement		
Understand the importance of giving good information		
Be aware of the way and consequences of how they give information		
Know how to give good information to patients		
Understand how to empower patients to follow advice		

PATIENT CENTRED SERVICES
SFQ for TRAINERS – COMMUNICATION SKILLS PART II (PCS02)

Trainer Study ID	Date of training
[____|____]	[____|____]/ [____|____]/[____|____]<br>day month year

**10. Please list commitments or plans made by participants during the workshop today.**<br>*List the commitments made and in brackets, list the representatives of each health centre who made that commitment. For example "To put up pictorial signs at the health centre (HC #03, 05 and 06)"*

**11. How many questions or concerns were raised by this group about the topics covered today?**	1 = Many questions<br>2 = Few questions<br>3 = None	[____]

**12. What questions or concerns were raised by this group about the topics discussed today? (Please list)**
**13. How did you address each of these concerns in this group?**
**14. Which of these concerns do you think were still present at the end of the training?**
**15. Would you change anything about the session you gave to this group today? If yes, what would you change? How would you change it?**

**16. What is your overall assessment of how well the intended objectives of today's training were achieved?**	1 = Badly<br>2 = Fine<br>3 = Good	[____]

APPENDIX G: PATIENT CENTRED SERVICES
SFQ for TRAINERS – BUILDING A POSITIVE WORK ENVIRONMENT (PCS03)

'Thank you for assisting with this Patient Centered Services training! We would appreciate your feedback on the training session you delivered today. Please take a moment to answer the following questions. Your comments will help us to know what information this group received and how well they achieved our intended learning objectives during this session. This will help us to know how to interpret any future change of practice amongst these health workers. There is no need to write your name on this questionnaire, and all responses will be kept strictly confidential. Thank you!'

**ID for Trainer 1** [_____|_____]<br>**ID for Trainer 2** [_____|_____]	**Date of training**<br>[____|____]/ [____|____]/[____|____]<br>day month year	**Study ID of other Trainers present**<br>[____|____], [____|____]
**Training group #**<br>[____|____]	**Total # of participants invited**<br>[____|____]	**Total # of participants attended**<br>[____|____]

Participant Study IDs

[____|____|____|____]	[____|____|____|____]	[____|____|____|____]	[____|____|____|____]
[____|____|____|____]	[____|____|____|____]	[____|____|____|____]	[____|____|____|____]
[____|____|____|____]	[____|____|____|____]	[____|____|____|____]	[____|____|____|____]
[____|____|____|____]	[____|____|____|____]	[____|____|____|____]	[____|____|____|____]

TRAINER QUESTIONNAIRE

**1. Did the training start more than half an hour late?**	1 = Yes<br>2 = No	[____]
**2. If yes, what was the cause of the delay?**	1 = Participants arrived late<br>2 = Trainers arrived late<br>3 = Materials and supplies to be used were not delivered in time<br>4 = Trainer transport difficulties<br>5 = Other ______________________________________	[____]<br>[____]<br>[____]

3. Please describe the consequence of the delay on your ability to deliver the training as intended

Training component	Consequence of delay on trainer's ability to deliver the training as intended
**Reflection on self-observation activity**	
**New topics introduced in the module**	
**Introduction of the next self-observation activity**	

**4. What level of contribution did the participants have?**	1 = All contributed a lot<br>2 = All contributed at some point<br>3 = Only some contributed<br>4 = None contributed	[____]
**5. For Qn. 4, responses 2 and 3 please list the 3 least active participants.**<br>[____|____|____|____] [____|____|____|____] [____|____|____|____]		

PATIENT CENTRED SERVICES<br>SFQ for TRAINERS – BUILDING A POSITIVE WORK ENVIRONMENT (PCS03)	
**Trainer Study ID**<br>[____|____]	**Date of training**<br>[____|____]/ [____|____]/[____|____]<br>day month year

6. Please complete the table below to describe how this training session went for each topic.

Topic	Teaching as planned?	Materials as planned?	If no, please explain
**Health Centre Management Changes**	1 = Yes<br>2 = No<br>[____]	1 = Yes<br>2 = No<br>[____]	
**Dealing with stress at work**	1 = Yes<br>2 = No<br>[____]	1 = Yes<br>2 = No<br>[____]	

7. Can you please describe any difficulties/barriers you encountered in delivery of the training?

<u>Barriers</u> to optimum implementation (e.g. - training interrupted, difficult or over bearing individuals, etc.)	<u>Impact</u> of barrier on the intended delivery of training and on learning

8.0 Please reflect on the uptake and usefulness of the SOA this week for your participants.

**8.1 How many participants do you think completed the SOAs in full this week?**
**8.2 How many participants do you think partially completed the SOAs this week?**
**8.3 Please list any participants who you think did not do the SOAs at all this week**

PATIENT CENTRED SERVICES
SFQ for TRAINERS – BUILDING A POSITIVE WORK ENVIRONMENT (PCS03)

Trainer Study ID	Date of training
[____|____]	[____|____]/ [____|____]/[____|____]<br>day month year

8.4 To what extent did the SOA help participants to think about today's topic in advance? (a lot / somewhat / not much)

8.5 Please give examples of specific insights from the SOAs shared this week that you think had a significant impact on others in the group in terms of their own uptake of the ideas (describe the concept, story, and ID number(s) of those involved)

8.6 In your opinion, how important was the SOA to today's learning in this group? (essential / take it or leave it / unhelpful)

9. Please score how well you think the participants achieved each of the learning objectives today on a scale of 1-10.
(1=Not achieved, 10= Achieved in full. Use 'N/A' if the section is not included in topic)

By the end of today's session, to what extent did the participants learn to...	Score	Comments
Recognise their challenges at work		
Know about planned Health Centre Management changes		
Know their role in Health Centre Management changes		
To recognise stress by how we feel and behave		
To understand the effect of automatic reactions on us and others		
To know how to 'step back' and stop automatic reactions		
To carry a picture of best practice in dealing with difficult patients and situations		

10. Please list commitments or plans made by participants during the workshop today.
List the commitments made and in brackets, list the representatives of each health centre who made that commitment. For example "To put up pictorial signs at the health centre (HC #03, 05 and 06)"

11. How many questions or concerns were raised by this group about the topics covered today?

1 = Many questions
2 = Few questions
3 = None [_____]

12. What questions or concerns were raised by this group about the topics discussed today? (Please list)

13. How did you address each of these concerns in this group?

14. Which of these concerns do you think were still present at the end of the training?

15. Would you change anything about the session you gave to this group today? If yes, what would you change? How would you change it?

16. What is your overall assessment of how well the intended objectives of today's training were achieved?

1 = Badly
2 = Fine
3 = Good [_____]

APPENDIX G: PATIENT CENTRED SERVICES

SFQ for TRAINERS – IMPROVING THE PATIENT VISIT (PCS04)

'Thank you for assisting with this Patient Centered Services training! We would appreciate your feedback on the training session you delivered today. Please take a moment to answer the following questions. Your comments will help us to know what information this group received and how well they achieved our intended learning objectives during this session. This will help us to know how to interpret any future change of practice amongst these health workers. There is no need to write your name on this questionnaire, and all responses will be kept strictly confidential. Thank you!'

**ID for Trainer 1** [____|____]<br>**ID for Trainer 2** [____|____]	**Date of training**<br>[____|____]/ [____|____]/[____|____]<br>day month year	**Study ID of other Trainers present**<br>[____|____], [____|____]
**Training group #**<br>[____|____]	**Total # of participants invited**<br>[____|____]	**Total # of participants attended**<br>[____|____]

Participant Study IDs

[____|____|____|____]	[____|____|____|____]	[____|____|____|____]	[____|____|____|____]
[____|____|____|____]	[____|____|____|____]	[____|____|____|____]	[____|____|____|____]
[____|____|____|____]	[____|____|____|____]	[____|____|____|____]	[____|____|____|____]
[____|____|____|____]	[____|____|____|____]	[____|____|____|____]	[____|____|____|____]

TRAINER QUESTIONNAIRE

**1. Did the training start more than half an hour late?**	1 = Yes<br>2 = No	[____]
**2. If yes, what was the cause of the delay?**	1 = Participants arrived late<br>2 = Trainers arrived late<br>3 = Materials and supplies to be used were not delivered in time<br>4 = Trainer transport difficulties<br>5 = Other ____	[____]<br>[____]<br>[____]

3. Please describe the consequence of the delay on your ability to deliver the training as intended

Training component	Consequence of delay on trainer's ability to deliver the training as intended
**Reflection on self-observation activity**	
**New topics introduced in the module**	
**Introduction of the next self-observation activity**	

**4. What level of contribution did the participants have?**	1 = All contributed a lot<br>2 = All contributed at some point<br>3 = Only some contributed<br>4 = None contributed	[____]

5. For Qn. 4, responses 2 and 3 please list the 3 least active participants.

[____|____|____|____] [____|____|____|____] [____|____|____|____]

PATIENT CENTRED SERVICES<br>SFQ for TRAINERS – IMPROVING THE PATIENT VISIT (PCS04)	
**Trainer Study ID**<br>[____|____]	**Date of training**<br>[____|____]/ [____|____]/[____|____]<br>day month year

6. Please complete the table below to describe how this training session went for each topic.

Topic	Teaching as planned?	Materials as planned?	If no, please explain
**Communication Review**	1 = Yes<br>2 = No<br>[____]	1 = Yes<br>2 = No<br>[____]	
**Patient Welcome and Orientation**	1 = Yes<br>2 = No<br>[____]	1 = Yes<br>2 = No<br>[____]	

7. Can you please describe any difficulties/barriers you encountered in delivery of the training?

<u>Barriers</u> to optimum implementation (e.g. - training interrupted, difficult or over bearing individuals, etc.)	<u>Impact</u> of barrier on the intended delivery of training and on learning

PATIENT CENTRED SERVICES<br>SFQ for TRAINERS – IMPROVING THE PATIENT VISIT (PCS04)	
**Trainer Study ID**<br>[_____|_____]	**Date of training**<br>[_____|_____]/ [_____|_____]/[_____|_____]<br>day month year

8.0 Please reflect on the uptake and usefulness of the SOA this week for your participants.

**8.1 How many participants do you think completed the SOAs in full this week?**
**8.2 How many participants do you think partially completed the SOAs this week?**
**8.3 Please list any participants who you think did not do the SOAs at all this week**
**8.4 To what extent did the SOA help participants to think about today's topic in advance? (a lot / somewhat / not much)**
**8.5 Please give examples of specific insights from the SOAs shared this week that you think had a significant impact on others in the group in terms of their own uptake of the ideas (describe the concept, story, and ID number(s) of those involved)**
**8.6 In your opinion, how important was the SOA to today's learning in this group? (essential / take it or leave it / unhelpful)**

9. Please score how well you think the participants achieved each of the learning objectives today on a scale of 1-10.
(1=Not achieved, 10= Achieved in full. Use 'N/A' if the section is not included in topic)

By the end of today's session, to what extent did the participants learn to...	Score	Comments
Become aware of ways to invite their patients and colleagues to co-operate and the impact of doing this		
Recognise that we all have different perspectives, including as health workers and patients.		
Put themselves into the shoes of a patient approaching a health centre as an organisation with unspoken 'rules'.		
Explore reasons why patients have to wait long, and develop strategies that meet health workers' as well as patients' needs better.		
Implement strategies to improve the welcome of patients at health centres.		
Implement strategies to improve the orientation of patients at health centres.		
Implement strategies to ensure patients are seen fairly.		

10. Please list commitments or plans made by participants during the workshop today.
List the commitments made and in brackets, list the representatives of each health centre who made that commitment. For example "To put up pictorial signs at the health centre (HC #03, 05 and 06)"

11. How many questions or concerns were raised by this group about the topics covered today?

1 = Many questions
2 = Few questions
3 = None [_____]

**12. What questions or concerns were raised by this group about the topics discussed today? (Please list)**
**13. How did you address each of these concerns in this group?**
**14. Which of these concerns do you think were still present at the end of the training?**
**15. Would you change anything about the session you gave to this group today? If yes, what would you change? How would you change it?**

**16. What is your overall assessment of how well the intended objectives of today's training were achieved?**	1 = Badly<br>2 = Fine<br>3 = Good	[_____]

APPENDIX G: PATIENT CENTRED SERVICES

SFQ for TRAINERS – VOLUNTEERS: IMPROVING THE PATIENT VISIT (PCS05)

'Thank you for assisting with this patient centered services training! We would appreciate your feedback on the training session you delivered today. Please take a moment to answer the following questions. Your comments will help us to know what information this group received and how well they achieved our intended learning objectives during this session. This will help us to know how to interpret any future change of practice amongst these health workers. There is no need to write your name on this questionnaire, and all responses will be kept strictly confidential. Thank you!'

**ID for Trainer 1** [____|____] **ID for Trainer 2** [____|____]	**Date of training** [____|____]/ [____|____]/[____|____] day month year	**Study ID of other Trainers present** [____|____], [____|____]
**Training group #** [____|____]	**Total # of participants invited** [____|____]	**Total # of participants attended** [____|____]

**Participant Study IDs**			
[____|____|____|____]	[____|____|____|____]	[____|____|____|____]	[____|____|____|____]
[____|____|____|____]	[____|____|____|____]	[____|____|____|____]	[____|____|____|____]
[____|____|____|____]	[____|____|____|____]	[____|____|____|____]	[____|____|____|____]
[____|____|____|____]	[____|____|____|____]	[____|____|____|____]	[____|____|____|____]

TRAINER QUESTIONNAIRE

**1. Did the training start more than half an hour late?**	1 = Yes<br>2 = No	[____]
**2. If yes, what was the cause of the delay?**	1 = Participants arrived late<br>2 = Trainers arrived late<br>3 = Materials and supplies to be used were not delivered in time<br>4 = Trainer transport difficulties<br>5 = Other ____	[____]<br>[____]<br>[____]

3. Please describe the consequence of the delay on your ability to deliver the training as intended

Training component	Consequence of delay on trainer's ability to deliver the training as intended
**New topics introduced in the module**	
**Role play activities**	
**Planning activities**	

**4. What level of contribution did the participants have?**	1 = All contributed a lot<br>2 = All contributed at some point<br>3 = Only some contributed<br>4 = None contributed	[____]

5. For Qn. 4, responses 2 and 3 please list the 3 least active participants.

[____|____|____|____] [____|____|____|____] [____|____|____|____]

PATIENT CENTRED SERVICES<br>SFQ for TRAINERS – VOLUNTEERS: IMPROVING THE PATIENT VISIT (PCS05)	
**Trainer Study ID**	**Date of training**
[_____|_____]	[_____|_____]/ [_____|_____]/[_____|_____]<br>day month year

6. Please complete the table below to describe how this training session went for each topic.

Topic	Teaching as planned?	Materials as planned?	If no, please explain
**Patient Centres Services**	1 = Yes<br>2 = No<br>[_____]	1 = Yes<br>2 = No<br>[_____]	
**Welcoming and greeting patients**	1 = Yes<br>2 = No<br>[_____]	1 = Yes<br>2 = No<br>[_____]	
**Improving patient navigation**	1 = Yes<br>2 = No<br>[_____]	1 = Yes<br>2 = No<br>[_____]	

7. Can you please describe any difficulties/barriers you encountered in delivery of the training?

<u>Barriers</u> to optimum implementation (e.g. - training interrupted, difficult or over bearing individuals, etc.)	<u>Impact</u> of barrier on the intended delivery of training and on learning

8. Please score how well you think the participants achieved each of the learning objectives today on a scale of 1-10.
(1=Not achieved, 10= Achieved in full. Use 'N/A' if the section is not included in topic)

By the end of today's session, to what extent did the participants learn to...	Score	Comments
Understand the importance of providing 'patient centred services'		
Recognise that we all have different perspectives, including volunteers and patients		
Implement strategies to improve the welcome of patients at health centres by establishing rapport		
Put themselves into the shoes of a patient approaching a health centre as an organisation with unspoken 'rules'		
Implement strategies to improve the orientation of patients at health centres		
Implement strategies to ensure patients can navigate the health centre		

PATIENT CENTRED SERVICES<br>SFQ for TRAINERS – VOLUNTEERS: IMPROVING THE PATIENT VISIT (PCS05)	
**Trainer Study ID**<br>[____|____]	**Date of training**<br>[____|____]/ [____|____]/[____|____]<br>day month year

**9. Please list commitments or plans made by participants during the workshop today.**<br>*List the commitments made and in brackets, list the representatives of each health centre who made that commitment. For example "To put up pictorial signs at the health centre (HC #03, 05 and 06)"*		
**10. How many questions or concerns were raised by this group about the topics covered today?**	1 = Many questions<br>2 = Few questions<br>3 = None	[____]
**11. What questions or concerns were raised by this group about the topics discussed today? (Please list)**		
**12. How did you address each of these concerns in this group?**		
**13. Which of these concerns do you think were still present at the end of the training?**		
**14. Would you change anything about the session you gave to this group today? If yes, what would you change? How would you change it?**		
**15. What is your overall assessment of how well the intended objectives of today's training were achieved?**	1 = Badly<br>2 = Fine<br>3 = Good	[____]

APPENDIX H: PATIENT CENTRED SERVICES SFQs

PART 1: PARTICIPANT DEMOGRAPHIC BACKGROUND FORM

If you are attending multiple training modules, you only need to complete this form once.

Thank you for participating in this training course. We would like to collect some information about you and your work history. Please take a moment to answer the following questions. There is no need to write your name on this questionnaire. All responses will be kept strictly confidential. Thank you!

Please complete the boxes below with your own information. Please ask if you have any questions.

Participant PRIME Study ID [____|____]

Please complete the questions below

1. What is your age in years? [____|____] years

2. What is your gender? (please circle) Male Female

3. How long have you worked at this health centre? [____|____] and [____|____]
years months

4. If you are an in-charge, how long have you actively worked as an in-charge? [____|____] and [____|____]
years months

5. What is your education? Please circle all levels completed

Primary
Senior four
Senior six
Vocational certificate
University
Others *(please specify)*
____
____

6. What year did you completed your highest level of education (schooling)? [____|____|____|____]
year

7. What is your current position? Please select from the list below and write the appropriate number here: [____|____]

01 = Senior medical Officer	06 = Enrolled nurse	11 = Laboratory technician
02 = Medical Officer	07 = Comprehensive nurse	12 = Laboratory assistant
03 = Senior clinical Officer	08 = Midwife	13 = Health assistant
04 = Clinical Officer	09 = Public health nurse	14 = Health educator
05 = Nursing Officer	10 = Nursing aide/assistant	15 = Other ________________

8. What year did you start working in this position? [____|____|____|____]
year

9. What training workshops have you attended in the past 3 years? Please complete the table below

	**Title of training you attended**	**Organization**	**Date** [dd/mm/yy]
**9a**			[_____/_____/_____]
**9b**			[_____/_____/_____]
**9c**			[_____/_____/_____]

APPENDIX H: PATIENT CENTRED SERVICES
SFQ for PARTICIPANTS – INTRODUCTION TO PCS & SOAs (PCS00)

Thank you for participating in this patient centred services training! We would appreciate your feedback on the training session you have attended. Please take a moment to answer the following questions. This is **NOT A TEST** and **WE VALUE YOUR OPINION**. Your opinions will help us improve future trainings. There is no need to write your name on this questionnaire.
All responses will be kept strictly confidential. Thank you!

GENERAL INSTRUCTIONS TO COMPLETE THIS QUESTIONNAIRE

1. *Please use a dark coloured pen to fill out the questionnaire*

2. *The health worker identity (ID) number is the unique number that was given to you at the start of this PRIME project. When we request you to give your ID number, please fill the boxes as below:*

   [_0_ | _5_ | _1_ | _0_]

   You are asked to enter your ID number like this at the top of each page of your questionnaire.

3. *In this questionnaire, we ask you to read each question carefully, look at the statements and decide which response is closest to your own opinion. When you have decided, please circle the number in the column under that response. For example:*

Decide how much you agree with the statements below	*Strongly agree*	*Agree*	*Disagree*	*Strongly disagree*
I learned new ideas today	*(1)*	*2*	*3*	*4*

4. *If you change your mind and would like to circle a different response, please cross out your original choice and circle the choice that fits your opinion best. For example, if you change your mind and you decide that your answer is 'disagree', cross through the original and circle the new response as below:*

Decide how much you agree with the statements below	*Strongly agree*	*Agree*	*Disagree*	*Strongly disagree*
I learned new ideas today	~~1~~	*2*	*(3)*	*4*

5. *When you have completed the questionnaire, please make sure you have completed everything in the following checklist:*

For the first time you attend a workshop <u>only</u>:	☐ *[For the first time only] I have signed and dated the consent form*<br>☐ *[For the first time only] I have completed my demographic details form*
For every workshop:	☐ *I have answered all of the questions in this questionnaire*<br>☐ *I have checked that I have circled the responses that are closest to my opinion*<br>☐ *I have written my health worker ID number on all of the pages of the questionnaire*<br>☐ *I have written my health worker ID number on the envelope and will place this completed form inside the envelope*

PATIENT CENTRED SERVICES<br>SFQ for PARTICIPANTS – INTRODUCTION TO PCS & SOAs (PCS00)	
**Health worker ID**	**Today's date**
[____|____|____|____]	[____|____]/ [____|____]/[____|____]<br>day month year

1. **Please complete the table below about your <u>learning today</u> by circling the response closest to your opinion.**

Decide how much you agree with the statements below	Strongly agree	Agree	Disagree	Strongly disagree
I learned new ideas today	1	2	3	4
I understood everything the trainer taught today	1	2	3	4
I felt the training was relevant to my daily work	1	2	3	4
The ideas I learned today will be useful for me in the future	1	2	3	4
I was given a chance to ask all of my questions	1	2	3	4
I felt that the trainer answered all our questions well	1	2	3	4
I felt able to practice new skills in the workshop today	1	2	3	4
I was given a chance to give my opinions	1	2	3	4
I will recommend my colleagues to attend this training	1	2	3	4
**If you have any comments you would like to share, relevant to the above statements, please write them below:**				

PATIENT CENTRED SERVICES<br>SFQ for PARTICIPANTS – INTRODUCTION TO PCS & SOAs (PCS00)	
**Health worker ID**	**Today's date**
[____|____|____|____]	[____|____]/ [____|____]/[____|____]<br>day month year

2. **Please complete the table below about the skills you have learnt from <u>the training topic</u>, by circling the response closest to your opinion.**

PLEASE REMEMBER, THERE IS NO RIGHT OR WRONG ANSWER!

Decide how much you agree with the statements below	Strongly agree	Agree	Disagree	Strongly disagree
This workshop has helped me to think differently about what is important to me about being a health worker	1	2	3	4
Thinking from the patient's viewpoint today has changed how I feel about my patients	1	2	3	4
Today I learned something new about what it means to provide patient centred services	1	2	3	4
At my health centre, we are already giving patient centred services and do not need to change	1	2	3	4
Today I learned how to observe myself in my own practice through 'self-observation activities'	1	2	3	4
I do not think self-observation will be possible during my working week	1	2	3	4
After this training, I am keen to learn more about how to put patients at the centre of services at my health centre	1	2	3	4
**Please write any comments you would like to share, relevant to the statements in this section:**				

PATIENT CENTRED SERVICES<br>SFQ for PARTICIPANTS – INTRODUCTION TO PCS & SOAs (PCS00)	
**Health worker ID**<br>[____|____|____|____]	**Today's date**<br>[____|____]/ [____|____]/[____|____]<br>day month year

3. Please complete the table below about today's <u>overall training</u> by circling the response closest to your opinion.

	Very good	Good	Fair	Poor	Very poor
What is your opinion of trainer 1's skills today?	1	2	3	4	5
What is your opinion of trainer 1's attitude today?	1	2	3	4	5
What is your opinion of trainer 2's skills today?	1	2	3	4	5
What is your opinion of trainer 2's attitude today?	1	2	3	4	5
What is your opinion of the support given by the training assistant today?	1	2	3	4	5
What is your opinion about the discussions you had in your small groups today?	1	2	3	4	5
What is your opinion of the learner's manual you received today?	1	2	3	4	5
What is your opinion of the flip charts that were used today?	1	2	3	4	5
Overall, what is your opinion of the training today?	1	2	3	4	5

4. Please write anything else you would like to say about today's training

NOW PLEASE ENSURE YOU HAVE COMPLETED THIS FORM FULLY BY GOING THROUGH THE <u>CHECK SHEET</u> ON THE FRONT PAGE

APPENDIX H: PATIENT CENTRED SERVICES

SFQ for PARTICIPANTS – COMMUNICATION SKILLS PART 1 (PCS01)

Thank you for participating in this patient centred services training! We would appreciate your feedback on the training session you have attended. Please take a moment to answer the following questions. This is **NOT A TEST** and **WE VALUE YOUR OPINION**. Your opinions will help us improve future trainings. There is no need to write your name on this questionnaire.
All responses will be kept strictly confidential. Thank you!

GENERAL INSTRUCTIONS TO COMPLETE THIS QUESTIONNAIRE

1. *Please use a dark coloured pen to fill out the questionnaire*

2. *The health worker identity (ID) number is the unique number that was given to you at the start of this PRIME project. When we request you to give your ID number, please fill the boxes as below:*

   [0 | 5 | 1 | 0]

   You are asked to enter your ID number like this at the top of each page of your questionnaire.

3. *In this questionnaire, we ask you to read each question carefully, look at the statements and decide which response is closest to your own opinion. When you have decided, please circle the number in the column under that response. For example:*

Decide how much you agree with the statements below	***Strongly agree***	***Agree***	***Disagree***	***Strongly disagree***
I learned new ideas today	*1* (circled)	*2*	*3*	*4*

4. *If you change your mind and would like to circle a different response, please cross out your original choice and circle the choice that fits your opinion best. For example, if you change your mind and you decide that your answer is 'disagree', cross through the original and circle the new response as below:*

Decide how much you agree with the statements below	***Strongly agree***	***Agree***	***Disagree***	***Strongly disagree***
I learned new ideas today	*1* (circled, crossed out)	*2*	*3* (circled)	*4*

5. *When you have completed the questionnaire, please make sure you have completed everything in the following checklist:*

For the first time you attend a workshop only:	☐ *[For the first time only] I have signed and dated the consent form*<br>☐ *[For the first time only] I have completed my demographic details form*
For every workshop:	☐ *I have answered all of the questions in this questionnaire*<br>☐ *I have checked that I have circled the responses that are closest to my opinion*<br>☐ *I have written my health worker ID number on all of the pages of the questionnaire*<br>☐ *I have written my health worker ID number on the envelope and will place this completed form inside the envelope*

PATIENT CENTRED SERVICES<br>SFQ for PARTICIPANTS – COMMUNICATION SKILLS PART 1 (PCS01)	
**Health worker ID**	**Today's date**
[____|____|____|____]	[____|____]/ [____|____]/[____|____]<br>day month year

1. **Please complete the table below about your <u>learning today</u> by circling the response closest to your opinion.**

Decide how much you agree with the statements below	Strongly agree	Agree	Disagree	Strongly disagree
I learned new ideas today	1	2	3	4
I understood everything the trainer taught today	1	2	3	4
I felt the training was relevant to my daily work	1	2	3	4
The ideas I learned today will be useful for me in the future	1	2	3	4
I was given a chance to ask all of my questions	1	2	3	4
I felt that the trainer answered all our questions well	1	2	3	4
I felt able to practice new skills in the workshop today	1	2	3	4
I was given a chance to give my opinions	1	2	3	4
I will recommend my colleagues to attend this training	1	2	3	4

If you have any comments you would like to share, relevant to the above statements, please write them below:

PATIENT CENTRED SERVICES<br>SFQ for PARTICIPANTS – COMMUNICATION SKILLS PART 1 (PCS01)	
**Health worker ID**	**Today's date**
[____|____|____|____]	[____|____]/ [____|____]/[____|____]<br>day month year

2. Please complete the table below about the skills you have learnt from the training topic, by circling the response closest to your opinion.

PLEASE REMEMBER, THERE IS NO RIGHT OR WRONG ANSWER!

Decide how much you agree with the statements below	Strongly agree	Agree	Disagree	Strongly disagree
In today's training, I learned new ideas about how I can put patients at ease by showing respect to them	1	2	3	4
This training has helped me to think differently about the impact of non-verbal behaviour on the patient	1	2	3	4
After today's training, I am more aware of how my communication skills affect whether the patient gets useful treatment and management	1	2	3	4
Today, I found it useful to practice showing respect to patients	1	2	3	4
After this training, I feel I am able to listen to patients better than I did before	1	2	3	4
In spite of this training, I think it will be difficult to listen actively because of my busy work environment.	1	2	3	4
I did not find today's training helpful because I already knew about communication	1	2	3	4

Please write any comments you would like to share, relevant to the above statements in this section:

PATIENT CENTRED SERVICES<br>SFQ for PARTICIPANTS – COMMUNICATION SKILLS PART 1 (PCS01)	
**Health worker ID**<br>[____|____|____|____]	**Health worker ID**<br>[____|____|____|____]

3. **Please complete the table below about your self-observation activity (SOA) preparation this week. Please give a truthful response – we want to know if this is a useful part of the training.**

Decide how much you agree with the statements below	Strongly agree	Agree	Disagree	Strongly disagree
I completed all sections of the SOA this week (SOA #1: How Do I Listen?)	1	2	3	4
I found this week's SOA was very difficult to understand	1	2	3	4
I found it difficult to find time to complete this week's SOA	1	2	3	4
I feel like I learned a lot about my own skills when I was carrying out my self-observation this week	1	2	3	4
I feel like I have changed already as a result of my self-observation this week	1	2	3	4
I found today's feedback discussion on our SOAs helpful	1	2	3	4
I am looking forward to carrying out my next SOA	1	2	3	4

Please write any comments you would like to share, relevant to the above statements in this section:

PATIENT CENTRED SERVICES<br>SFQ for PARTICIPANTS – COMMUNICATION SKILLS PART 1 (PCS01)	
**Health worker ID**<br>[____|____|____|____]	**Health worker ID**<br>[____|____|____|____]

4. Please complete the table below about today's <u>overall training</u> by circling the response closest to your opinion.

	Very good	Good	Fair	Poor	Very poor
What is your opinion of trainer 1's skills today?	1	2	3	4	5
What is your opinion of trainer 1's attitude today?	1	2	3	4	5
What is your opinion of trainer 2's skills today?	1	2	3	4	5
What is your opinion of trainer 2's attitude today?	1	2	3	4	5
What is your opinion of the support given by the training assistant today?	1	2	3	4	5
What is your opinion about the discussions you had in your small groups today?	1	2	3	4	5
What is your opinion of the learner's manual you received today?	1	2	3	4	5
What is your opinion of the flip charts that were used today?	1	2	3	4	5
Overall, what is your opinion of the training today?	1	2	3	4	5

5. Please write anything else you would like to say about today's training

NOW PLEASE ENSURE YOU HAVE COMPLETED THIS FORM FULLY BY GOING THROUGH THE <u>CHECK SHEET</u> ON THE FRONT PAGE

APPENDIX H: PATIENT CENTRED SERVICES

SFQ for PARTICIPANTS – COMMUNICATION SKILLS PART 2 (PCS02)

Thank you for participating in this patient centred services training! We would appreciate your feedback on the training session you have attended. Please take a moment to answer the following questions. This is **NOT A TEST** and **WE VALUE YOUR OPINION**. Your opinions will help us improve future trainings. There is no need to write your name on this questionnaire.
All responses will be kept strictly confidential. Thank you!

GENERAL INSTRUCTIONS TO COMPLETE THIS QUESTIONNAIRE

1. *Please use a dark coloured pen to fill out the questionnaire*

2. *The health worker identity (ID) number is the unique number that was given to you at the start of this PRIME project. When we request you to give your ID number, please fill the boxes as below:*

   [_0_ | _5_ | _1_ | _0_]

   You are asked to enter your ID number like this at the top of each page of your questionnaire.

3. *In this questionnaire, we ask you to read each question carefully, look at the statements and decide which response is closest to your own opinion. When you have decided, please circle the number in the column under that response. For example:*

Decide how much you agree with the statements below	***Strongly agree***	***Agree***	***Disagree***	***Strongly disagree***
I learned new ideas today	*1* (circled)	*2*	*3*	*4*

4. *If you change your mind and would like to circle a different response, please cross out your original choice and circle the choice that fits your opinion best. For example, if you change your mind and you decide that your answer is 'disagree', cross through the original and circle the new response as below:*

Decide how much you agree with the statements below	***Strongly agree***	***Agree***	***Disagree***	***Strongly disagree***
I learned new ideas today	*1* (circled, crossed out)	*2*	*3* (circled)	*4*

5. *When you have completed the questionnaire, please make sure you have completed everything in the following checklist:*

For the first time you attend a workshop <u>only</u>:	☐ *[For the first time only] I have signed and dated the consent form*<br>☐ *[For the first time only] I have completed my demographic details form*
For every workshop:	☐ *I have answered all of the questions in this questionnaire*<br>☐ *I have checked that I have circled the responses that are closest to my opinion*<br>☐ *I have written my health worker ID number on all of the pages of the questionnaire*<br>☐ *I have written my health worker ID number on the envelope and will place this completed form inside the envelope*

PATIENT CENTRED SERVICES<br>SFQ for PARTICIPANTS – COMMUNICATION SKILLS PART 2 (PCS02)	
**Health worker ID**	**Today's date**
[____|____|____|____]	[____|____]/ [____|____]/[____|____]<br>day month year

1. **Please complete the table below about your <u>learning today</u> by circling the response closest to your opinion.**

Decide how much you agree with the statements below	Strongly agree	Agree	Disagree	Strongly disagree
I learned new ideas today	1	2	3	4
I understood everything the trainer taught today	1	2	3	4
I felt the training was relevant to my daily work	1	2	3	4
The ideas I learned today will be useful for me in the future	1	2	3	4
I was given a chance to ask all of my questions	1	2	3	4
I felt that the trainer answered all our questions well	1	2	3	4
I felt able to practice new skills in the workshop today	1	2	3	4
I was given a chance to give my opinions	1	2	3	4
I will recommend my colleagues to attend this training	1	2	3	4
**If you have any comments you would like to share, relevant to the above statements, please write them below:**				

PATIENT CENTRED SERVICES<br>SFQ for PARTICIPANTS – COMMUNICATION SKILLS PART 2 (PCS02)	
**Health worker ID**	**Today's date**
[____|____|____|____]	[____|____]/ [____|____]/[____|____]<br>day month year

2. Please complete the table below about the skills you have learnt from <u>the training topic</u>, by circling the response closest to your opinion.

PLEASE REMEMBER, THERE IS NO RIGHT OR WRONG ANSWER!

Decide how much you agree with the statements below	Strongly agree	Agree	Disagree	Strongly disagree
After this training, I now think differently about why it is important to get information from patients in a good way	1	2	3	4
After this training, I now think differently about why it is important to give information to patients in a good way	1	2	3	4
I found today's practice of using open questions very useful	1	2	3	4
After this training, I feel better able to ask questions without showing judgement	1	2	3	4
In today's training, I found ways I will use to empower patients to follow advice	1	2	3	4
In spite of today's training, I think it will be too difficult for me to give all the information each patient needs when I am back in my busy work environment	1	2	3	4
I did not find today's training helpful because I already knew about communication	1	2	3	4

Please write any comments you would like to share, relevant to the above statements in this section:

PATIENT CENTRED SERVICES<br>SFQ for PARTICIPANTS – COMMUNICATION SKILLS PART 2 (PCS02)	
**Health worker ID**	**Today's date**
[____|____|____|____]	[____|____]/ [____|____]/[____|____]<br>day month year

3. **Please complete the table below about your self-observation activity (SOA) preparation this week. Please give a truthful response – we want to know if this is a useful part of the training.**

Decide how much you agree with the statements below	Strongly agree	Agree	Disagree	Strongly disagree
I completed all sections of the SOA this week (SOA #2: How Do I Ask Good Questions?)	1	2	3	4
I found this week's SOA was very difficult to understand	1	2	3	4
I found it difficult to find time to complete this week's SOA	1	2	3	4
I feel like I learned a lot about my own skills when I was carrying out my self-observation this week	1	2	3	4
I feel like I have changed already as a result of my self-observation this week	1	2	3	4
I found today's feedback discussion on our SOAs helpful	1	2	3	4
I am looking forward to carrying out my next SOA	1	2	3	4

Please write any comments you would like to share, relevant to the above statements in this section:

PATIENT CENTRED SERVICES<br>SFQ for PARTICIPANTS – COMMUNICATION SKILLS PART 2 (PCS02)	
**Health worker ID**<br>[____|____|____|____]	**Today's date**<br>[____|____]/ [____|____]/[____|____]<br>day month year

4. Please complete the table below about today's <u>overall training</u> by circling the response closest to your opinion.

	**Very good**	**Good**	**Fair**	**Poor**	**Very poor**
What is your opinion of trainer 1's skills today?	1	2	3	4	5
What is your opinion of trainer 1's attitude today?	1	2	3	4	5
What is your opinion of trainer 2's skills today?	1	2	3	4	5
What is your opinion of trainer 2's attitude today?	1	2	3	4	5
What is your opinion of the support given by the training assistant today?	1	2	3	4	5
What is your opinion about the discussions you had in your small groups today?	1	2	3	4	5
What is your opinion of the learner's manual you received today?	1	2	3	4	5
What is your opinion of the flip charts that were used today?	1	2	3	4	5
Overall, what is your opinion of the training today?	1	2	3	4	5

5. Please write anything else you would like to say about today's training

NOW PLEASE ENSURE YOU HAVE COMPLETED THIS FORM FULLY BY GOING THROUGH THE <u>CHECK SHEET</u> ON THE FRONT PAGE

APPENDIX H: PATIENT CENTRED SERVICES
SFQ for PARTICIPANTS – BUILDING A POSITIVE WORK ENVIRONMENT (PCS03)

Thank you for participating in this patient centred services training! We would appreciate your feedback on the training session you have attended. Please take a moment to answer the following questions. This is **NOT A TEST** and **WE VALUE YOUR OPINION**. Your opinions will help us improve future trainings. There is no need to write your name on this questionnaire.
All responses will be kept strictly confidential. Thank you!

GENERAL INSTRUCTIONS TO COMPLETE THIS QUESTIONNAIRE

1. *Please use a dark coloured pen to fill out the questionnaire*
2. *The health worker identity (ID) number is the unique number that was given to you at the start of this PRIME project. When we request you to give your ID number, please fill the boxes as below:*

   [_0_ | _5_ | _1_ | _0_]

   You are asked to enter your ID number like this at the top of each page of your questionnaire.
3. *In this questionnaire, we ask you to read each question carefully, look at the statements and decide which response is closest to your own opinion. When you have decided, please circle the number in the column under that response. For example:*

Decide how much you agree with the statements below	***Strongly agree***	***Agree***	***Disagree***	***Strongly disagree***
I learned new ideas today	*(1)*	*2*	*3*	*4*

4. *If you change your mind and would like to circle a different response, please cross out your original choice and circle the choice that fits your opinion best. For example, if you change your mind and you decide that your answer is 'disagree', cross through the original and circle the new response as below:*

Decide how much you agree with the statements below	***Strongly agree***	***Agree***	***Disagree***	***Strongly disagree***
I learned new ideas today	*~~(1)~~*	*2*	*(3)*	*4*

5. *When you have completed the questionnaire, please make sure you have completed everything in the following checklist:*

For the first time you attend a workshop <u>only</u>:	☐ *[For the first time only] I have signed and dated the consent form*<br>☐ *[For the first time only] I have completed my demographic details form*
For every workshop:	☐ *I have answered all of the questions in this questionnaire*<br>☐ *I have checked that I have circled the responses that are closest to my opinion*<br>☐ *I have written my health worker ID number on all of the pages of the questionnaire*<br>☐ *I have written my health worker ID number on the envelope and will place this completed form inside the envelope*

PATIENT CENTRED SERVICES<br>SFQ for PARTICIPANTS – BUILDING A POSITIVE WORK ENVIRONMENT (PCS03)	
**Health worker ID**<br>[____|____|____|____]	**Today's date**<br>[____|____]/ [____|____]/[____|____]<br>day month year

1. Please complete the table below about your <u>learning today</u> by circling the response closest to your opinion.

Decide how much you agree with the statements below	Strongly agree	Agree	Disagree	Strongly disagree
I learned new ideas today	1	2	3	4
I understood everything the trainer taught today	1	2	3	4
I felt the training was relevant to my daily work	1	2	3	4
The ideas I learned today will be useful for me in the future	1	2	3	4
I was given a chance to ask all of my questions	1	2	3	4
I felt that the trainer answered all our questions well	1	2	3	4
I felt able to practice new skills in the workshop today	1	2	3	4
I was given a chance to give my opinions	1	2	3	4
I will recommend my colleagues to attend this training	1	2	3	4
**If you have any comments you would like to share, relevant to the above statements, please write them below:**				

PATIENT CENTRED SERVICES

SFQ for PARTICIPANTS – BUILDING A POSITIVE WORK ENVIRONMENT (PCS03)

Health worker ID	Today's date
[____|____|____|____]	[____|____]/ [____|____]/[____|____]<br>day month year

2. Please complete the table below about the skills you have learnt from <u>the training topic</u>, by circling the response closest to your opinion.

PLEASE REMEMBER, THERE IS NO RIGHT OR WRONG ANSWER!

Decide how much you agree with the statements below	Strongly agree	Agree	Disagree	Strongly disagree
I found it useful to discuss my challenges at work with other colleagues in the training today	1	2	3	4
I found it useful to hear from in-charges about plans for changes at our Health Centres today	1	2	3	4
After today's workshop, I know what my role will be in the proposed changes to managing stocks, finances and information at my health centre	1	2	3	4
I learned new ideas today about what is means to be stressed and burned out	1	2	3	4
This training has given me new ideas for how to recognise stress by how I feel and behave	1	2	3	4
In the role plays today, I found it helpful to practice stopping my automatic reactions	1	2	3	4
After this training, I feel able to 'step back' and stop automatic reactions	1	2	3	4
After this training, I feel more confident to deal with difficult patients and situations	1	2	3	4
In spite of today's training, I will find it too difficult to deal with my stressful environment when I return to my health centre	1	2	3	4
I did not find today's training helpful because I already knew about managing stress, or it is not relevant to me	1	2	3	4

Please write any comments you would like to share, relevant to the above statements in this section:

PATIENT CENTRED SERVICES
SFQ for PARTICIPANTS – BUILDING A POSITIVE WORK ENVIRONMENT (PCS03)

Health worker ID	Today's date
[____|____|____|____]	[____|____]/ [____|____]/[____|____]<br>day month year

3. **Please complete the table below about your self-observation activity (SOA) preparation this week. Please give a truthful response – we want to know if this is a useful part of the training.**

Decide how much you agree with the statements below	Strongly agree	Agree	Disagree	Strongly disagree
I completed all sections of the SOA this time (SOA #3: What Happens When I Am Stressed?)	1	2	3	4
I found this SOA was very difficult to understand	1	2	3	4
I found it difficult to find time to complete this SOA	1	2	3	4
I feel like I learned a lot about my own skills when I was carrying out my self-observation this week	1	2	3	4
I feel like I have changed already as a result of my self-observation this time	1	2	3	4
I found today's feedback discussion on our SOAs helpful	1	2	3	4
I am looking forward to carrying out my next SOA	1	2	3	4

Please write any comments you would like to share, relevant to the above statements in this section:

PATIENT CENTRED SERVICES<br>SFQ for PARTICIPANTS – BUILDING A POSITIVE WORK ENVIRONMENT (PCS03)	
**Health worker ID**<br>[____|____|____|____]	**Today's date**<br>[____|____]/ [____|____]/[____|____]<br>day month year

4. Please complete the table below about today's <u>overall training</u> by circling the response closest to your opinion.

	**Very good**	**Good**	**Fair**	**Poor**	**Very poor**
What is your opinion of trainer 1's skills today?	1	2	3	4	5
What is your opinion of trainer 1's attitude today?	1	2	3	4	5
What is your opinion of trainer 2's skills today?	1	2	3	4	5
What is your opinion of trainer 2's attitude today?	1	2	3	4	5
What is your opinion of the support given by the training assistant today?	1	2	3	4	5
What is your opinion about the discussions you had in your small groups today?	1	2	3	4	5
What is your opinion of the learner's manual you received today?	1	2	3	4	5
What is your opinion of the flip charts that were used today?	1	2	3	4	5
Overall, what is your opinion of the training today?	1	2	3	4	5

5. Please write anything else you would like to say about today's training

NOW PLEASE ENSURE YOU HAVE COMPLETED THIS FORM FULLY BY GOING THROUGH THE <u>CHECK SHEET</u> ON THE FRONT PAGE

APPENDIX H: PATIENT CENTRED SERVICES

SFQ for PARTICIPANTS – IMPROVING THE PATIENT VISIT (PCS04)

Thank you for participating in this patient centred services training! We would appreciate your feedback on the training session you have attended. Please take a moment to answer the following questions. This is **NOT A TEST** and **WE VALUE YOUR OPINION**. Your opinions will help us improve future trainings. There is no need to write your name on this questionnaire.
All responses will be kept strictly confidential. Thank you!

GENERAL INSTRUCTIONS TO COMPLETE THIS QUESTIONNAIRE

1. *Please use a dark coloured pen to fill out the questionnaire*

2. *The health worker identity (ID) number is the unique number that was given to you at the start of this PRIME project. When we request you to give your ID number, please fill the boxes as below:*

   [_0_ | _5_ | _1_ | _0_]

   You are asked to enter your ID number like this at the top of each page of your questionnaire.

3. *In this questionnaire, we ask you to read each question carefully, look at the statements and decide which response is closest to your own opinion. When you have decided, please circle the number in the column under that response. For example:*

Decide how much you agree with the statements below	***Strongly agree***	***Agree***	***Disagree***	***Strongly disagree***
I learned new ideas today	*(1)*	*2*	*3*	*4*

4. *If you change your mind and would like to circle a different response, please cross out your original choice and circle the choice that fits your opinion best. For example, if you change your mind and you decide that your answer is 'disagree', cross through the original and circle the new response as below:*

Decide how much you agree with the statements below	***Strongly agree***	***Agree***	***Disagree***	***Strongly disagree***
I learned new ideas today	*~~(1)~~*	*2*	*(3)*	*4*

5. *When you have completed the questionnaire, please make sure you have completed everything in the following checklist:*

For the first time you attend a workshop only:	☐ *[For the first time only] I have signed and dated the consent form*<br>☐ *[For the first time only] I have completed my demographic details form*
For every workshop:	☐ *I have answered all of the questions in this questionnaire*<br>☐ *I have checked that I have circled the responses that are closest to my opinion*<br>☐ *I have written my health worker ID number on all of the pages of the questionnaire*<br>☐ *I have written my health worker ID number on the envelope and will place this completed form inside the envelope*

PATIENT CENTRED SERVICES
SFQ for PARTICIPANTS – IMPROVING THE PATIENT VISIT (PCS04)

Health worker ID	Today's date
[____|____|____|____]	[____|____]/ [____|____]/[____|____]<br>day month year

1. **Please complete the table below about your <u>learning today</u> by circling the response closest to your opinion.**

Decide how much you agree with the statements below	Strongly agree	Agree	Disagree	Strongly disagree
I learned new ideas today	1	2	3	4
I understood everything the trainer taught today	1	2	3	4
I felt the training was relevant to my daily work	1	2	3	4
The ideas I learned today will be useful for me in the future	1	2	3	4
I was given a chance to ask all of my questions	1	2	3	4
I felt that the trainer answered all our questions well	1	2	3	4
I felt able to practice new skills in the workshop today	1	2	3	4
I was given a chance to give my opinions	1	2	3	4
I will recommend my colleagues to attend this training	1	2	3	4

If you have any comments you would like to share, relevant to the above statements, please write them below:

PATIENT CENTRED SERVICES

SFQ for PARTICIPANTS – IMPROVING THE PATIENT VISIT (PCS04)

Health worker ID	Today's date
[____|____|____|____]	[____|____]/ [____|____]/[____|____]<br>day month year

2. Please complete the table below about the skills you have learnt from <u>the training topic</u>, by circling the response closest to your opinion.

PLEASE REMEMBER, THERE IS NO RIGHT OR WRONG ANSWER!

Decide how much you agree with the statements below	Strongly agree	Agree	Disagree	Strongly disagree
This training helped me to understand why it is important to work together with patients and colleagues	1	2	3	4
Today, I have learned ways to invite patients and colleagues to co-operate	1	2	3	4
This training has helped me to put myself into the shoes of a patient approaching a health centre as an organisation with unspoken 'rules'	1	2	3	4
This training has given me ideas for how to to ensure patients are seen fairly	1	2	3	4
After this training, I feel able to implement strategies to improve the welcome of patients at health centres	1	2	3	4
After this training, I will implement strategies to improve the orientation of patients at health centres.	1	2	3	4
In spite of today's training, I will find it too difficult to implement the ideas we discussed into my busy work-life	1	2	3	4
I did not find the training helpful today because I already know how to improve the patient's visit	1	2	3	4

Please write any comments you would like to share, relevant to the above statements in this section:

PATIENT CENTRED SERVICES<br>SFQ for PARTICIPANTS – IMPROVING THE PATIENT VISIT (PCS04)	
**Health worker ID**<br>[____|____|____|____]	**Today's date**<br>[____|____]/ [____|____]/[____|____]<br>day month year

3. Please complete the table below about your self-observation activity (SOA) preparation this week. Please give a truthful response – we want to know if this is a useful part of the training.

Decide how much you agree with the statements below	Strongly agree	Agree	Disagree	Strongly disagree
I completed all sections of the SOA this week (SOA #4: How Do I Invite the Patient To Cooperate?)	1	2	3	4
I found this week's SOA was very difficult to understand	1	2	3	4
I found it difficult to find time to complete this week's SOA	1	2	3	4
I feel like I learned a lot about my own skills when I was carrying out my self-observation this week	1	2	3	4
I feel like I have changed already as a result of my self-observation this week	1	2	3	4
I found today's feedback discussion on our SOAs helpful	1	2	3	4
I plan to continue with the SOAs we have been given for the future	1	2	3	4

Please write any comments you would like to share, relevant to the above statements in this section:

PATIENT CENTRED SERVICES<br>SFQ for PARTICIPANTS – IMPROVING THE PATIENT VISIT (PCS04)	
**Health worker ID**<br>[____|____|____|____]	**Today's date**<br>[____|____]/ [____|____]/[____|____]<br>day month year

4. **Please complete the table below about today's <u>overall training</u> by circling the response closest to your opinion.**

	Very good	Good	Fair	Poor	Very poor
What is your opinion of trainer 1's skills today?	1	2	3	4	5
What is your opinion of trainer 1's attitude today?	1	2	3	4	5
What is your opinion of trainer 2's skills today?	1	2	3	4	5
What is your opinion of trainer 2's attitude today?	1	2	3	4	5
What is your opinion of the support given by the training assistant today?	1	2	3	4	5
What is your opinion about the discussions you had in your small groups today?	1	2	3	4	5
What is your opinion of the learner's manual you received today?	1	2	3	4	5
What is your opinion of the flip charts that were used today?	1	2	3	4	5
Overall, what is your opinion of the training today?	1	2	3	4	5

5. Please write anything else you would like to say about today's training

PATIENT CENTRED SERVICES<br>SFQ for PARTICIPANTS – IMPROVING THE PATIENT VISIT (PCS04)	
**Health worker ID**<br>[____|____|____|____]	**Today's date**<br>[____|____]/ [____|____]/[____|____]<br>day month year

6. Please complete the table below about the overall PRIME training by circling the response closest to your opinion.

Decide how much you agree with the statements below	Strongly agree	Agree	Disagree	Strongly disagree
Overall, the PRIME training has tackled topics that are very important to how I do my work	1	2	3	4
I have made significant changes in the way I interact with patients as a result of the PRIME training	1	2	3	4
We have made significant changes in the running of our health centre as a result of the PRIME training	1	2	3	4
I would have preferred the training to have all been in one week rather than over several weeks	1	2	3	4
The timings of the training sessions was not convenient for me	1	2	3	4
I found the opportunity to work with other colleagues at different health centres useful	1	2	3	4
I would recommend the use of self-observation activities in future trainings like this one	1	2	3	4
My colleagues and I area giving each other more support at work since starting this training	1	2	3	4

Please write any comments you would like to share, relevant to the above statements in this section:

PATIENT CENTRED SERVICES<br>SFQ for PARTICIPANTS – IMPROVING THE PATIENT VISIT (PCS04)	
**Health worker ID**	**Today's date**
[____|____|____|____]	[____|____]/ [____|____]/[____|____]<br>day month year

7. Please list the most important things you learned in the PRIME training that have changed how you go about your work

What I learned ***How it changed what I do***

8. Please use this space to give us any final feedback about the course, the trainers or the materials.

NOW PLEASE ENSURE YOU HAVE COMPLETED THIS FORM FULLY BY GOING THROUGH THE CHECK SHEET ON THE FRONT PAGE

APPENDIX H: PATIENT CENTRED SERVICES

SFQ for PARTICIPANTS – VOLUNTEERS: IMPROVING THE PATIENT VISIT(PCS05)

Thank you for participating in this patient centred services training! We would appreciate your feedback on the training session you have attended. Please take a moment to answer the following questions. This is **NOT A TEST** and **WE VALUE YOUR OPINION**. Your opinions will help us improve future trainings. There is no need to write your name on this questionnaire.
All responses will be kept strictly confidential. Thank you!

GENERAL INSTRUCTIONS TO COMPLETE THIS QUESTIONNAIRE

1. *Please use a dark coloured pen to fill out the questionnaire*

2. *The health worker identity (ID) number is the unique number that was given to you at the start of this PRIME project. When we request you to give your ID number, please fill the boxes as below:*

   [_0_ | _5_ | _1_ | _0_]

   You are asked to enter your ID number like this at the top of each page of your questionnaire.

3. *In this questionnaire, we ask you to read each question carefully, look at the statements and decide which response is closest to your own opinion. When you have decided, please circle the number in the column under that response. For example:*

Decide how much you agree with the statements below	***Strongly agree***	***Agree***	***Disagree***	***Strongly disagree***
I learned new ideas today	*1* (circled)	*2*	*3*	*4*

4. *If you change your mind and would like to circle a different response, please cross out your original choice and circle the choice that fits your opinion best. For example, if you change your mind and you decide that your answer is 'disagree', cross through the original and circle the new response as below:*

Decide how much you agree with the statements below	***Strongly agree***	***Agree***	***Disagree***	***Strongly disagree***
I learned new ideas today	~~*1*~~ (circled, crossed out)	*2*	*3* (circled)	*4*

5. *When you have completed the questionnaire, please make sure you have completed everything in the following checklist:*

For the first time you attend a workshop <u>only</u>:	☐ *[For the first time only] I have signed and dated the consent form*<br>☐ *[For the first time only] I have completed my demographic details form*
For every workshop:	☐ *I have answered all of the questions in this questionnaire*<br>☐ *I have checked that I have circled the responses that are closest to my opinion*<br>☐ *I have written my health worker ID number on all of the pages of the questionnaire*<br>☐ *I have written my health worker ID number on the envelope and will place this completed form inside the envelope*

PATIENT CENTRED SERVICES<br>SFQ for PARTICIPANTS – VOLUNTEERS:IMPROVING THE PATIENT VISIT(PCS05)	
**Health worker ID**<br>[____|____|____|____]	**Today's date**<br>[____|____]/ [____|____]/[____|____]<br>day month year

1. **Please complete the table below about your <u>learning today</u> by circling the response closest to your opinion.**

Decide how much you agree with the statements below	Strongly agree	Agree	Disagree	Strongly disagree
I learned new ideas today	1	2	3	4
I understood everything the trainer taught today	1	2	3	4
I felt the training was relevant to my daily work	1	2	3	4
The ideas I learned today will be useful for me in the future	1	2	3	4
I was given a chance to ask all of my questions	1	2	3	4
I felt that the trainer answered all our questions well	1	2	3	4
I felt able to practice new skills in the workshop today	1	2	3	4
I was given a chance to give my opinions	1	2	3	4
I will recommend my colleagues to attend this training	1	2	3	4

If you have any comments you would like to share, relevant to the above statements, please write them below:

PATIENT CENTRED SERVICES<br>SFQ for PARTICIPANTS – VOLUNTEERS: IMPROVING THE PATIENT VISIT(PCS05)	
**Health worker ID**	**Today's date**
[____|____|____|____]	[____|____]/ [____|____]/[____|____]<br>day month year

2. **Please complete the table below about the skills you have learnt from <u>the training topic</u>, by circling the response closest to your opinion.**

PLEASE REMEMBER, THERE IS NO RIGHT OR WRONG ANSWER!

Decide how much you agree with the statements below	Strongly agree	Agree	Disagree	Strongly disagree
This training helped me to understand the importance of providing 'patient centred services'	1	2	3	4
This training has helped me to see that we all have different perspectives, including volunteers and patients	1	2	3	4
This training has given me ideas for how to to improve the welcome of patients at my health centre	1	2	3	4
After this training, I feel able to build a good relationship with all types of patients attending my health centre	1	2	3	4
After this training, I have found ways to to ensure patients know where to go at my health centre	1	2	3	4
I will find it too difficult to carry out these new plans because I am too busy with other activities	1	2	3	4

Please write any comments on the above statements in this section here:

PATIENT CENTRED SERVICES<br>SFQ for PARTICIPANTS – VOLUNTEERS: IMPROVING THE PATIENT VISIT(PCS05)	
**Health worker ID**	**Today's date**
[____|____|____|____]	[____|____]/ [____|____]/[____|____]<br>day month year

3. Please complete the table below about today's <u>overall training</u> by circling the response closest to your opinion.

	Very good	Good	Fair	Poor	Very poor
What is your opinion of trainer 1's skills today?	1	2	3	4	5
What is your opinion of trainer 1's attitude today?	1	2	3	4	5
What is your opinion of trainer 2's skills today?	1	2	3	4	5
What is your opinion of trainer 2's attitude today?	1	2	3	4	5
What is your opinion of the support given by the training assistant today?	1	2	3	4	5
What is your opinion about the discussions you had in your small groups today?	1	2	3	4	5
What is your opinion of the learner's manual you received today?	1	2	3	4	5
What is your opinion of the flip charts that were used today?	1	2	3	4	5
Overall, what is your opinion of the training today?	1	2	3	4	5

4. Please write anything else you would like to say about today's training

NOW PLEASE ENSURE YOU HAVE COMPLETED THIS FORM FULLY BY GOING THROUGH THE <u>CHECK SHEET</u> ON THE FRONT PAGE

Appendix I: Measure of Patient-Centered Communication (MPCC) Coding Form

Coding form for component I. Exploring both the disease and the illness experience

Date:
Start Time:__________ Coder Initials:__________
Stop Time:__________ Tape Code:__________

COMPONENT I. EXPLORING BOTH THE DISEASE AND THE ILLNESS EXPERIENCE

Symptoms and/or Reason for Visit		Preliminary Exploration	Further Exploration	Validation	Cut-off	SCORE
1	______	Y N	Y N	Y N	Y N	______
2	______	Y N	Y N	Y N	Y N	______
3	______	Y N	Y N	Y N	Y N	______
4	______	Y N	Y N	Y N	Y N	______
5	______	Y N	Y N	Y N	Y N	______
					ST**	
**Prompts**						
1	______	Y N	Y N	Y N	Y N	______
2	______	Y N	Y N	Y N	Y N	______
3	______	Y N	Y N	Y N	Y N	______
4	______	Y N	Y N	Y N	Y N	______
5	______	Y N	Y N	Y N	Y N	______
					ST**	
**Feelings**						
1	______	Y N	Y N	Y N	Y N	______
2	______	Y N	Y N	Y N	Y N	______
3	______	Y N	Y N	Y N	Y N	______
4	______	Y N	Y N	Y N	Y N	______
5	______	Y N	Y N	Y N	Y N	______
					ST**	
**Ideas**						
1	______	Y N	Y N	Y N	Y N	______
2	______	Y N	Y N	Y N	Y N	______
3	______	Y N	Y N	Y N	Y N	______
4	______	Y N	Y N	Y N	Y N	______
5	______	Y N	Y N	Y N	Y N	______
					ST**	
**Effect on Function**						
1	______	Y N	Y N	Y N	Y N	______
2	______	Y N	Y N	Y N	Y N	______
3	______	Y N	Y N	Y N	Y N	______
4	______	Y N	Y N	Y N	Y N	______
5	______	Y N	Y N	Y N	Y N	______
					ST**	
**Expectations**						
1	______	Y N	Y N	Y N	Y N	______
2	______	Y N	Y N	Y N	Y N	______
3	______	Y N	Y N	Y N	Y N	______
4	______	Y N	Y N	Y N	Y N	______
5	______	Y N	Y N	Y N	Y N	______
					ST**	

GT*** ______ ÷ ______ =

** Sub-total
**** Grand Total

Notes for completion of component 1

Score	Process Category
0	no preliminary exploration
0	preliminary exploration with cutoff
1	preliminary exploration and further exploration with cut off
2	preliminary exploration and validation with cut-off (no further exploration)
2	preliminary exploration without cut-off (no further exploration, no validation)
3	preliminary exploration and further exploration without cutoff (no validation)
3	preliminary exploration and further exploration and validation with cut-off
4	preliminary exploration and validation without cut off (no further exploration)
5	preliminary exploration and further exploration and validation without cut-off

Steps

1) Assign a score (X) for each statement listed.
2) For each heading (i.e. Symptoms and/or Reason for Visit, Prompts, Feelings, Ideas, Effect on Function, Expectations), add Xs and divide by the number of statements to calculate a subtotal (ST).
3) Add all STs.
4) Determine the appropriate denominator. The denominator is the number of applicable headings (maximum 6) multiplied by the score range (5). The denominator will be 30 (6 headings x 5) except where there are no "Symptoms and/or Reason for Visit" and/or "Prompts". If there are neither "Symptoms and/or Reason for Visit" nor "Prompts", the denominator will be 20 (4 headings x 5). If there is only one of "Symptoms and/or Reason for Visit" or "Prompts", the denominator will be 25 (5 headings x 5).
5) Divide the sum of all STs by the appropriate denominator to calculate the grand total.

Table for scoring (KEY: Y = yes; N = no)

Preliminary Exploration	Further Exploration	Validation	Cut-off	Score (0-5)
N	N	N	Y	0
Y	N	N	Y	0
Y	Y	N	Y	1
Y	N	Y	Y	2
Y	N	N	N	2
Y	Y	N	N	3
Y	Y	Y	Y	3
Y	N	Y	N	4
Y	Y	Y	N	5

Coding form for component II. Understanding the whole person

Any statements relevant to FAMILY, LIFE CYCLE, SOCIAL SUPPORT, PERSONALITY, and CONTEXT are to be listed below:

		Preliminary Exploration	Further Exploration	Validation	Cut-off	SCORE
1	________	Y N	Y N	Y N	Y N	________
2	________	Y N	Y N	Y N	Y N	________
3	________	Y N	Y N	Y N	Y N	________
4	________	Y N	Y N	Y N	Y N	________
5	________	Y N	Y N	Y N	Y N	________
6	________	Y N	Y N	Y N	Y N	________
7	________	Y N	Y N	Y N	Y N	________
8	________	Y N	Y N	Y N	Y N	________
9	________	Y N	Y N	Y N	Y N	________
10	________	Y N	Y N	Y N	Y N	________
					ST*	☐

GT** ________ ÷ **5** = ☐

* Sub-total
** Grand Total

Notes on completing coding for component II

Score	Process Category Meaning
0	no preliminary exploration
0	preliminary exploration with cutoff
1	preliminary exploration and further exploration with cut off
2	preliminary exploration and validation with cut-off (no further exploration)
2	preliminary exploration without cut-off (no further exploration, no validation)
3	preliminary exploration and further exploration without cutoff (no validation)
3	preliminary exploration and further exploration and validation with cut-off
4	preliminary exploration and validation without cut off (no further exploration)
5	preliminary exploration and further exploration and validation without cut-off

<u>Steps</u>

1) Assign a score (X) for each statement listed.
2) Add Xs and divide by the number of statements to calculate a subtotal (ST).
3) Divide the ST by 5 (the maximum possible score) to calculate the grand total (GT)

<u>Table for scoring (KEY: Y = yes; N = no)</u>

Preliminary Exploration	Further Exploration	Validation	Cut-off	Score (0-5)
N	N	N	Y	0
Y	N	N	Y	0
Y	Y	N	Y	1
Y	N	Y	Y	2
Y	N	N	N	2
Y	Y	N	N	3
Y	Y	Y	Y	3
Y	N	Y	N	4
Y	Y	Y	N	5

Coding form for component III. Finding common ground

Problem Definition:

	Clearly Expressed	Opportunity to Ask Questions	Mutual Discussion	Clarification of Agreement	SCORE
1 ______	Y N	Y N	Y N	Y N	______
2 ______	Y N	Y N	Y N	Y N	______
3 ______	Y N	Y N	Y N	Y N	______
4 ______	Y N	Y N	Y N	Y N	______
5 ______	Y N	Y N	Y N	Y N	______
6 ______	Y N	Y N	Y N	Y N	______
7 ______	Y N	Y N	Y N	Y N	______
8 ______	Y N	Y N	Y N	Y N	______
9 ______	Y N	Y N	Y N	Y N	______
10 ______	Y N	Y N	Y N	Y N	______
				ST**	[ ]

Goals of Treatment/Management

	Clearly Expressed	Opportunity to Ask Questions	Mutual Discussion	Clarification of Agreement	SCORE
1 ______	Y N	Y N	Y N	Y N	______
2 ______	Y N	Y N	Y N	Y N	______
3 ______	Y N	Y N	Y N	Y N	______
4 ______	Y N	Y N	Y N	Y N	______
5 ______	Y N	Y N	Y N	Y N	______
6 ______	Y N	Y N	Y N	Y N	______
7 ______	Y N	Y N	Y N	Y N	______
8 ______	Y N	Y N	Y N	Y N	______
9 ______	Y N	Y N	Y N	Y N	______
10 ______	Y N	Y N	Y N	Y N	______
				ST**	[ ]

Responded Appropriately to Disagreement with Flexibility and Understanding

1 ______	Y N	N/A			______
2 ______	Y N	N/A			______
				ST**	[ ]

** Sub-total GT*** ______ ÷ ______ = [ ]

*** Grand Total

Notes for completing coding for component III

Scoring Guideline

For each statement under Problem Definition and Goals of Treatment and Management, each occurrence of Y (yes) is given a score of 1 for a maximum of 4 for each statement. Each occurrence of N (no) gets a score of 0. For Responded Appropriately to Disagreement with Flexibility and Understanding, each occurrence of Y (yes) is given a score of 4 and each occurrence of N (no) is given a score of 0.

Steps

1) Assign a score (X) for each statement listed using the scoring guideline above.
2) For each of the three headings (i.e. Problem Definition, Goals of Treatment and Management, Responded Appropriately to Disagreement with Flexibility and Understanding), add Xs and divide by the number of statements to calculate a subtotal (STs).
3) Determine the appropriate denominator. The highest possible score when using all three headings (Problem Definition, Goals of Treatment and Management, and Responded Appropriately to Disagreement with Flexibility and Understanding) is 12. If there are no statements for Responded Appropriately to Disagreement with Flexibility and Understanding, add the two other subtotals (Problem Definition and Goals of Treatment and Management) and divide by 8. If there are no Problem Definitions and no Responded Appropriately to Disagreement with Flexibility and Understanding, the denominator is 4.
4) Add the STs and divide by the appropriate denominator to obtain the Grand Total (GT) for Component III.
5) Multiply by 100 to obtain a percentage score.

[____]—[____|____|____|____]

HCWA Study ID

APPENDIX J. HEALTH WORKER COMMUNICATION ASSESSMENTS
Informed consent form for health workers

**Protocol Title:**	ACT PROCESS: Evaluating the process, context, and impact of interventions to enhance health facilities in Tororo, Uganda
**Site of Research:**	Tororo, Uganda
**Principal Investigators**:	Dr. Sarah Staedke
**Date:**	23 February 2011

Introduction

Dr. Sarah Staedke and colleagues from the Uganda Malaria Surveillance Project/Infectious Diseases Research Collaboration are doing a study to evaluate activities that have been introduced at government-run health facilities in this area aiming to improve the health of children. We would like to understand the impact of these activities and why they are working, or not. This study involves several parts, including assessing the communication between health workers and their patients, holding interviews, and conducting group discussions with community members and health workers.

Why is this study being done?

For this part of the study, we would like to learn more about how well patients and health workers communicate. To do this, we would like to record interactions between health workers and caregivers of ill children at 20 health centers in this area. We are interested in recording your interactions with caregivers of typical malaria patients: children under five who have fever and no danger signs of severe disease. We would like to learn what usually happens in interactions between health workers, caregivers, and patients.

What will happen today if I take part in this study?

If you agree to take part, we will ask you to keep a digital voice recorder in the room where you see patients to record your interactions with patients and their caregivers. We will inform patients and their caregivers about this device and will also ask them if they agree to be recorded. We will let you know if they agree and will help you switch on the recording device. We would like you to conduct your consultation with the patient as you would normally; you are not expected to do anything differently to your usual practice while we are recording. We would like to record your interaction with at least one patient, and up to three patients. After we have recorded these interactions with you, we will not ask you to do anything further. The information we collect will be used by project investigators, and may be shared with other researchers and policy-makers to answer questions about how best to deliver training and health services.

How long will the study last?

We would like to record your interactions with patients on three occasions: today, within the next 2-3 months, and again in about 9 months. Each time, we would like to record your interaction with at least three, and up to five patients, which should take approximately 1-2 days to complete.

Can I stop being in the study?

You can decide to stop participating at any time. Just tell the project researcher right away if you wish to stop.

What risks can I expect from being in the study?

Participation in any research study may involve a loss of privacy. Information exchanged during the interactions with patients will be recorded, but your name will not be used in any reports of the information provided. The names of your patients, caregivers and colleagues will also not be used. No quotes or other results arising from your participation in this study will be included in any reports, even anonymously, without your agreement. The information obtained from these interviews will be locked at our project offices. We will do our best to make sure that the personal information gathered for this study is kept private.

Are there benefits to taking part in the study?

There will be no direct benefit to you from participating in this study. However, the information that you provide will help researchers and policy-makers understand how best to improve health services in this area.

What other choices do I have if I do not take part in this study?

You are free to choose not to participate in the study. If you decide not to take part in this study, there will be no penalty to you.

What are the costs of taking part in this study? Will I be paid for taking part in this study?

There are no costs to you for taking part in this study. You will not be paid for taking part in this study.

What are my rights if I take part in this study?

Taking part in this study is your choice. You may choose either to take part or not to take part in the study. If you decide to take part in this study, you may change your mind at any time. No matter what decision you take, there will be no penalty to you in any way.

Who can answer my questions about the study?

You can talk to the researchers about any questions or concerns you have about these study activities. Contact Dr. Sarah Staedke or other members of the Uganda Malaria Surveillance Project / Infectious Disease Research Collaboration on telephone number 0414-530692. If you have any questions, comments or

concerns about taking part in these activities, first talk to the researchers. If for any reason you do not wish to do this, or you still have concerns about doing so, you may contact Dr Charles Ibingira, Makerere University Faculty of Medicine Research and Ethical Committee at telephone number 0414-530020.

WHAT YOUR SIGNATURE OR THUMBPRINT MEANS

Your signature or thumbprint below means that you understand the information given to you in this consent form about your participation in the study and agree with the following statements:

1. "I have read the consent form concerning this study (or have understood the verbal explanation of the consent form) and I understand what will be required of me and what will happen to me if I take part in it."
2. "My questions concerning this study have been answered by Dr. Staedke or the person who signed below."
3. "I understand that at any time, I may withdraw from this study without giving a reason."
4. "I agree to take part in this study."

If you wish to participate in this study, you should sign or place your thumbprint on the following page. If you do not agree to quotes or other results arising from your participation in the study being included, even anonymously, in any reports about the study, please tell the researcher now.

THE REPUBLIC OF UGANDA

WE WILL GIVE YOU A COPY OF THIS SIGNED AND DATED CONSENT FORM

Name of Participant (printed)

Signature or Fingerprint of Participant | Date/Time

Name of Investigator Administering Consent (printed) | Position/Title

Signature of Investigator Administering Consent | Date/Time

If the participant is unable to read and/or write, an impartial witness should be present during the entire informed consent discussion.

After the written informed consent form is read and explained to the participant, and after they have orally consented to participate in the study, the witness should print the name of the participant above, and then the participant should provide their fingerprint.

Then the witness should print their name, provide their signature, and date the consent form below.

By signing the consent form, the witness attests that the information in the consent form and any other written information was accurately explained to, and apparently understood by the participant, and that informed consent was freely given by the participant.

Name of Person Witnessing Consent (printed)

Signature of Person Witnessing Consent | Date/Time

APPENDIX K. HEALTH WORKER COMMUNICATION ASSESSMENT

PART 1: HEALTH WORKER DETAILS

Health center code	HW Study ID	Interviewer code	Date
[_____|_____]	[_____|_____]	[_____|_____]	[____|____] / [____|____] / [____|____]<br>day month year

Health worker position: [____|____]

1 = In-charge
2 = Senior medical officer
3 = Medical officer
4 = Senior clinical officer
5 = Clinical officer
6 = Comprehensive nurse
7=Nursing officer
8= Enrolled nurse
9= Midwife
10= Public health nurse
11 = Nursing aide/assistant
12 = Laboratory technician
13 = Laboratory assistant
14= Health assistant
15 = Health educator
16 = Other_______________________________________

PART 2: DEMOGRAPHIC INFORMATION

1. Health worker age [____|____] years

2. Health worker gender 1 = Male 2 = Female [____]

3. Originally from this area? 1 = Yes 2 = No [____]

4. Number of years worked in this job [____|____] years

5. Highest level of education or qualification achieved [____|____]

0 = None
1 = Primary (P1 — P7)
2 = Secondary (S1 — S6)
3 = Certificate
4 = Diploma
5 = Bachelor's degree
88 = Don't know
99 = Refused to answer
77 = Other ____

6. Year graduated [____|____|____|____]
year

7. Health worker language skills

1= Fluent
2=Some skills
3= Cannot speak

Japhadhola [____|____]
Ateso [____|____]
Swahilli [____|____]
Luganda [____|____]
Lusamya [____|____]
Lusoga [____|____]
Lugwere [____|____]
Lunyole [____|____]
Other_________________________________ [____|____]

APPENDIX L: HEALTH WORKER COMMUNICATION ASSESSMENT
CAREGIVER & PATIENT SCREENING FORM

Health center code	Interviewer code	Date
[_____|_____]	[_____|_____]	[____|____] / [____|____] / [____|____]<br>day month year

Screening Date	Screening ID
[_____|_____]/[_____|_____]/[_____|_____]<br>day month year	[_____|_____|_____|_____]

Age		Gender	
[____|____] / [____|____]<br>years months	*If child is less than 1 year, complete months, otherwise leave blank*	[____]	1 = Male<br>2 = Female

PART 2: SCREENING INTERVIEW with PARENTS/GUARDIANS

Selection criteria	Include	Exclude	
1. Appropriate age<br>—Under five (aged 0 to less than 5 years)	1 = Yes	2 = No	[_____]
2. Fever or suspected fever	1 = Yes	2 = No	[_____]
3. Danger signs of severe disease? (Convulsions, severe weakness/lethargy, unable to feed, unable to stand/sit unsupported, vomiting everything, severe dehydration)	1 = No	2 = Yes	[_____]

If any answers are '2' from the EXCLUDE column, exclude from the study. If not, proceed to the next section.

PART 3: INFORMED CONSENT

Selection criteria	Include	Exclude	
4. Willingness of caregiver to provide informed consent	1 = Yes	2 = No	[_____]

If any answers are '2' from the EXCLUDE column, exclude from the study. If not, proceed to the next section.

ASSIGN STUDY NUMBER	[_____|_____|_____|_____]

All criteria for study inclusion met?	Date of enrollment
1 = Yes<br>2 = No *If no, exclude from the study*<br>[_____]	[______|______]/[______|______]/[______|______]<br>day month year

Staff ID: [____|____] **Data entrant (1st):** [____|____] **Data entrant (2nd):** [____|____]

THE REPUBLIC OF UGANDA

[____]—[____|____|____|____]

HCWA Study ID

APPENDIX M. HEALTH WORKER COMMUNICATION ASSESSMENTS and PATIENT EXIT INTERVIEWS
Informed consent form for caregivers

**Protocol Title:**	ACT PROCESS: Evaluating the process, context, and impact of interventions to enhance health facilities in Tororo, Uganda
**Site of Research:**	Tororo, Uganda
**Principal Investigators**:	Dr. Sarah Staedke
**Date:**	20 February 2012

Introduction

Dr. Sarah Staedke and colleagues from the Uganda Malaria Surveillance Project/Infectious Diseases Research Collaboration are doing a study to evaluate activities that have been introduced at government-run health facilities in this area aiming to improve the health of children. We would like to understand the impact of these activities and why they are working, or not. This study involves several parts, including assessing the communication between health workers and their patients, holding interviews, and conducting group discussions with community members and health workers.

Why is this study being done?

For this part of the study, we would like to learn more about how well patients and health workers communicate. To do this, we would like to ask caregivers of ill children at 20 health centers in this area some questions about their visit to the health center today. We are interested in talking to caregivers of typical malaria patients: children under five who have fever and no danger signs of severe disease. We would like to learn about the purpose of their visit, and whether they were satisfied with their visit or not.

What will happen today if I take part in this study?

If you agree to take part, after the consultation with the health worker is over, we would like to ask you some questions about your visit to the health center today. We would like to ask questions about the purpose of your visit, and whether you were satisfied with your visit or not, particularly regarding the way the health worker communicated with you. After we have asked you the questions about your visit today, we will not ask you to do anything further. The information we collect will be used by project investigators, and may be shared with other researchers and policy-makers to answer questions about how best to deliver training and health services.

How long will the study last?

If you take part in the study, it will involve a one-time interview today. This should take about 15 minutes.

Can I stop being in the study?

You can decide to stop participating at any time. Just tell the project researcher right away if you wish to stop.

What risks can I expect from being in the study?

Participation in any research study may involve a loss of privacy. Information obtained from these interviews will be recorded on paper, but your name will not be used in any reports of the information provided. No quotes or other results arising from your participation in this study will be included in any reports, even anonymously, without your agreement. The information obtained from these interviews will be locked at our project offices. We will do our best to make sure that the personal information gathered for this study is kept private.

Are there benefits to taking part in the study?

There will be no direct benefit to you from participating in this study. However, the information that you provide will help researchers and policy-makers understand how best to improve health services in this area.

What other choices do I have if I do not take part in this study?

You are free to choose not to participate in the study. If you decide not to take part in this study, there will be no penalty to you.

What are the costs of taking part in this study? Will I be paid for taking part in this study?

There are no costs to you for taking part in this study. You will not be paid for taking part in this study.

What are my rights if I take part in this study?

Taking part in this study is your choice. You may choose either to take part or not to take part in the study. If you decide to take part in this study, you may change your mind at any time. No matter what decision you take, there will be no penalty to you in any way.

Who can answer my questions about the study?

You can talk to the researchers about any questions or concerns you have about these study activities. Contact Dr. Sarah Staedke or other members of the Uganda Malaria Surveillance Project / Infectious Disease Research Collaboration on telephone number 0414-530692. If you have any questions, comments or concerns about taking part in these activities, first talk to the researchers. If for any reason you do not wish

THE REPUBLIC OF UGANDA

to do this, or you still have concerns about doing so, you may contact Dr James Tumwine, Makerere University Faculty of Medicine Research and Ethical Committee at telephone number 0414-530020.

WHAT YOUR SIGNATURE OR THUMBPRINT MEANS

Your signature or thumbprint below means that you understand the information given to you in this consent form about your participation in the study and agree with the following statements:

1. "I have read the consent form concerning this study (or have understood the verbal explanation of the consent form) and I understand what will be required of me and what will happen to me if I take part in it."

2. "My questions concerning this study have been answered by Dr. Staedke or the person who signed below."

3. "I understand that at any time, I may withdraw from this study without giving a reason."

4. "I agree to take part in this study."

If you wish to participate in this study, you should sign or place your thumbprint on the following page. If you do not agree to quotes or other results arising from your participation in the study being included, even anonymously, in any reports about the study, please tell the researcher now.

Mark each box with **X** if you agree:

☐ **I agree to take part in the interview after my consultation with the health worker is over**

THE REPUBLIC OF UGANDA

WE WILL GIVE YOU A COPY OF THIS SIGNED AND DATED CONSENT FORM

Name of Participant (printed)

Signature or Fingerprint of Participant   Date/Time

Name of Investigator Administering Consent (printed)   Position/Title

Signature of Investigator Administering Consent   Date/Time

If the participant is unable to read and/or write, an impartial witness should be present during the entire informed consent discussion.

After the written informed consent form is read and explained to the participant, and after they have orally consented to participate in the study, the witness should print the name of the participant above, and then the participant should provide their fingerprint.

Then the witness should print their name, provide their signature, and date the consent form below.

By signing the consent form, the witness attests that the information in the consent form and any other written information was accurately explained to, and apparently understood by the participant, and that informed consent was freely given by the participant.

Name of Person Witnessing Consent (printed)

Signature of Person Witnessing Consent   Date/Time

APPENDIX N. HEALTH WORKER COMMUNICATION ASSESSMENT

PART 1: CAREGIVER DETAILS

Health center code	Patient ID code	Interviewer code	Date
[_____|_____]	[_____|_____]	[_____|_____]	[____|____] / [____|____] / [____|____]<br>day month year

**1. Number of patients brought by the caregiver today** [_____|_____]	**2. Number of eligible children being seen today** [_____|_____]

PART 2: CAREGIVER DEMOGRAPHIC INFORMATION

**1. Caregiver age** [____|____] years	**2. Caregiver gender** 1 = Male 2 = Female [____]

3. Been to this health centre before? 1 = Yes 2 = No [____]

4. Originally from this area? 1 = Yes 2 = No [____]

5. Highest level of education or qualification achieved [____|____]

0 = None
1 = Primary (P1 — P7)
2 = Secondary (S1 — S6)
3 = Certificate
4 = Diploma
5 = Bachelor's degree
88 = Don't know
99 = Refused to answer
77 = Other

____

6. Primary working activity [____|____]

0 =Unemployed
1 =Housewife/stay home mother
2 = Health worker
3 = Peasant farmer
4 = Small business owner/worker
77 = Other

____

88 = Don't know
99 = Refused to answer

7. Subjective impression of the caretaker's social economic status

1= Low
2=Medium
3=High [____|____]

PART 3: PATIENT DEMOGRAPHIC INFORMATION

**1. Age of child enrolled** [____|____] Months [____|____] Years	**2. Gender** 1 = Male 2 = Female [____]

3. What problems does the child have? (list all mentioned by the caregiver)

1 = None
2 = Cough
3 = Flu
4 = Not eating
5 = Vomiting
6 = Weak (not playing)
7 = Convulsions
8 = Fever
9 = Diarrhoea
10 = Skin infection
77 =Other

____

____

88 = I don't know
99 = Refused to answer

[____|____] [____|____]

[____|____] [____|____]

[____|____] [____|____]

APPENDIX O. HEALTH WORKER COMMUNICATION ASSESSMENT PATIENT EXIT INTERVIEW

Health Center ID	HW ID	Patient ID	Interviewer ID	Date
[____|____]	[____|____]	[____|____]	[____|____]	[____|____] / [____|____] / [____|____]<br>day month year

**1. Number of patients brought by the caregiver today** [_____|_____]	**2. Number of eligible children being seen today** [_____|_____]

3. Language of consultation ____

**1. What was the reason you came here today?** *(List, in mother's words, below)*		
**2. Did you feel you were able to discuss this problem fully with the health worker?**	1 = Yes<br>2 = No	[_____|_____]
**3. Do you think that the health worker understood how important this problem is to you and your child?**	1 = Yes<br>2 = No	[_____|_____]
**4. Did you feel that the health worker was listening to you with full attention?**	1 = Yes<br>2 = No	[_____|_____]
**5. Did the health worker give you enough information about why he thinks the child is experiencing the problem(s)?**	1 = Yes<br>2 = No	[_____|_____]
**6. Do you agree with the health worker's opinion about the problem?**	1 = Yes<br>2 = No	[_____|_____]
**7. Do you feel the health worker could have done more to investigate the problem of your child today?**	1 = Yes<br>2 = No	[_____|_____]
**8. How satisfied were you with the treatment you were given?** *(Allocate category that fits best with response, check with mother)*	1 = Very satisfied<br>2 = Satisfied<br>3 = Somewhat satisfied<br>4 = Not satisfied	[_____|_____]
**9. Did the health worker explain what this medicine will do?**	1 = Yes<br>2 = No	[_____|_____]
**10. Did the health worker help you to understand how the child should take this medicine?**	1 = Yes<br>2 = No	[_____|_____]
**11. Did the health worker help you to understand what to expect during the child's illness?**	1 = Yes<br>2 = No	[_____|_____]
**12. How confident do you feel that your child will get better if you follow the health worker's advice?**<br>*(Allocate category that fits best with response, check with mother)*	1 = Very confident<br>2 = Somewhat confident<br>3 = Not confident	[_____|_____]
**13. Overall, how did the health worker make you feel?**<br>*(Allocate category that fits best with response, check with mother)*	1= Very happy<br>2 = Somewhat happy<br>3 = Unhappy	[_____|_____]
**14. Overall, how welcome did you feel at this health centre from start to finish?**	1 = Very welcome<br>2 = Welcome<br>3 = Somewhat welcome<br>4 = Unwelcome	[_____|_____]
**15. Next time your child is sick, will you come back to here?**	1 = Yes<br>2 = No	[_____|_____]
**16. Overall, how satisfied were you with the consultation today?**	1 = Very satisfied<br>2 = Satisfied<br>3 = Somewhat satisfied<br>4 = Not satisfied	[_____|_____]
**17. Do you have any additional comments about this consultation with the health worker?**		

THE REPUBLIC OF UGANDA

[____]—[____|____|____|____]

IDI Study ID

APPENDIX P. IN-DEPTH INTERVIEWS
Informed consent form for implementers and stakeholders

**Protocol Title:**	ACT PROCESS: Evaluating the process, context, and impact of interventions to enhance health facilities in Tororo, Uganda
**Site of Research:**	Tororo, Uganda
**Principal Investigators**:	Dr. Sarah Staedke
**Date:**	23 February 2011

Introduction

Dr. Sarah Staedke and colleagues from the Uganda Malaria Surveillance Project/Infectious Diseases Research Collaboration are doing a study to evaluate activities that have been introduced at government-run health facilities in this area aiming to improve the health of children. We would like to understand the impact of these activities and why they are working, or not. This study involves several parts, including assessing the communication between health workers and their patients, holding interviews, and conducting group discussions with community members and health workers.

Why is this study being done?

For this part of the study, we would like to learn more about whether the activities that have been introduced at government-run health facilities have had an impact from the perspective of those involved in introducing the activities, and local and district stakeholders. This information will help us understand how and why the health facility activities have affected the health of children in this area.

What will happen today if I take part in this study?

Today, we would like to ask you some questions about the activities that have been introduced at the health facilities, provision of care for sick children in this area, and any changes that you have noticed recently. We will take notes of the discussion and a recording will also be made using a digital voice recorder. After we ask these questions today, we will not ask you to do anything further. The information we collect will be used by project investigators, and may be shared with other researchers and policy-makers to answer questions about how best to deliver training and health services.

How long will the study last?

Today, the interview will last about 60-90 minutes.

Can I stop being in the study?
You can decide to stop participating at any time. Just tell the project researcher right away if you wish to stop.

What risks can I expect from being in the study?
Participation in any research study may involve a loss of privacy. Information exchanged during the interactions with patients will be recorded, but your name will not be used in any reports of the information provided. The names of your patients, caregivers and colleagues will also not be used. No quotes or other results arising from your participation in this study will be included in any reports, even anonymously, without your agreement. The information obtained from these interviews will be locked at our project offices. We will do our best to make sure that the personal information gathered for this study is kept private.

Are there benefits to taking part in the study?
There will be no direct benefit to you from participating in this study. However, the information that you provide will help researchers and policy-makers understand how best to improve health services in this area.

What other choices do I have if I do not take part in this study?
You are free to choose not to participate in the study. If you decide not to take part in this study, there will be no penalty to you.

What are the costs of taking part in this study? Will I be paid for taking part in this study?
There are no costs to you for taking part in this study. You will not be paid for taking part in this study.

What are my rights if I take part in this study?
Taking part in this study is your choice. You may choose either to take part or not to take part in the study. If you decide to take part in this study, you may change your mind at any time. No matter what decision you take, there will be no penalty to you in any way.

Who can answer my questions about the study?
You can talk to the researchers about any questions or concerns you have about these study activities. Contact Dr. Sarah Staedke or other members of the Uganda Malaria Surveillance Project / Infectious Disease Research Collaboration on telephone number 0414-530692. If you have any questions, comments or concerns about taking part in these activities, first talk to the researchers. If for any reason you do not wish to do this, or you still have concerns about doing so, you may contact Dr Charles Ibingira, Makerere University Faculty of Medicine Research and Ethical Committee at telephone number 0414-530020.

WHAT YOUR SIGNATURE OR THUMBPRINT MEANS

Your signature or thumbprint below means that you understand the information given to you in this consent form about your participation in the study and agree with the following statements:

1. "I have read the consent form concerning this study (or have understood the verbal explanation of the consent form) and I understand what will be required of me and what will happen to me if I take part in it."

2. "My questions concerning this study have been answered by Dr. Staedke or the person who signed below."

3. "I understand that at any time, I may withdraw from this study without giving a reason."

4. "I agree to take part in this study."

If you wish to participate in this study, you should sign or place your thumbprint on the following page. If you do not agree to quotes or other results arising from your participation in the study being included, even anonymously, in any reports about the study, please tell the researcher now.

THE REPUBLIC OF UGANDA

WE WILL GIVE YOU A COPY OF THIS SIGNED AND DATED CONSENT FORM

Name of Participant (printed)

Signature or Fingerprint of Participant Date/Time

Name of Investigator Administering Consent (printed) Position/Title

Signature of Investigator Administering Consent Date/Time

If the participant is unable to read and/or write, an impartial witness should be present during the entire informed consent discussion.

After the written informed consent form is read and explained to the participant, and after they have orally consented to participate in the study, the witness should print the name of the participant above, and then the participant should provide their fingerprint.

Then the witness should print their name, provide their signature, and date the consent form below.

By signing the consent form, the witness attests that the information in the consent form and any other written information was accurately explained to, and apparently understood by the participant, and that informed consent was freely given by the participant.

Name of Person Witnessing Consent (printed)

Signature of Person Witnessing Consent Date/Time

APPENDIX Q: IDI DATA COLLECTION TOOL
HFI IMPLEMENTERS

Study ID	Date
[____|____]	[____|____] / [____|____] / [____|____]<br>day month year

Position:

1 = Study coordinator<br>2 = Medical Officer<br>3 = Trainer<br>4 = Community Health worker<br>5 = Clinical officer<br>6 = Laboratory technician	7 = Laboratory Assistant<br>8 =Home Visitor<br>9 = Implementation support (administration, logistics, procurement, research assistant)<br>10 = Other_______________<br>[____|____]

DEMOGRAPHIC INFORMATION

**1. Age**	Years	[____|____]	**5. Highest level of education or qualification achieved**<br>1 = Primary (P1 — P7)<br>2 = Secondary (S1 — S6)<br>3 = Certificate<br>77 = Other___________________________<br>4 = Diploma<br>5 = Bachelor's degree<br>6= Master's degree<br>99 = Refused to answer<br>[____|____]
**2. Gender**	1 = Male<br>2 = Female	[____]	
**3. Originally from this area?**	1 = Yes<br>2 = No	[____]	
**4. Number of years worked in this job**		[____|____]	**6. Year graduated** [____|____|____|____]

INTRODUCTION

Conduct the interview according to the directions below and record information as indicated.

Introduction to in-depth interview

"Hello, my name is ………. I am interested in interviewing you. I would like you to express your own views and experiences about your perspectives on the implementation of the ACT PRIME health facility intervention to improve the health of children in Tororo by improving services at government-run health facilities. A note-taker will be writing down what you say for our records, and we will record the interview using a digital recorder; these notes will be kept securely and your name will not be used anywhere. Your answers will be looked at together with those of many other implementers and you will not be identifiable in any reports that are published.

It is very important for us to hear your views and experiences because you have experience implementing the intervention and can give us this insight. We hope you will have time to spend with us now to complete this interview. The interview will take about 45 minutes; but if you prefer we can reschedule the interview for tomorrow or another day of your convenience.

Do you have any questions? Do you agree to continue before we start?

Now we request that we all switch off our mobile phones so that we are not distracted."

IMPLEMENTER IN-DEPTH INTERVIEW (1)

Domains, topic questions, and probes: Use the table below to help you administer the questions during the interview.

Domain	Topic and Probes
**1. Role of the implementer**	a) What has been your role as an implementer of this intervention?<br><br>**For HSD liaison person:** What has been your role as part of the ACT PRIME project?
**2. Meeting participant expectations**	a) What do you think health workers expected from this ACT PRIME health facility intervention?
	b) From your perspective, do you think this intervention met their expectations? *Probe: What expectations were met and what else happened that you think they were not expecting? Probe: challenges of motivating health workers at the HFI facilities*

IMPLEMENTER IN-DEPTH INTERVIEW (2)	
**Domains, topic questions, and probes:** Use the table below to help you administer the questions during the interview.	
**Domain**	**Topic and Probes**
**3. Training delivery**	a) Were you directly involved with the training components of the HFI?<br>**If no, skip to Question 5.**
	b) Can you please describe the main objectives for the implementation of the training component of the HFI, as you understand it?
	c) Can you describe each of the training sessions that you had with the participants? *Probe: what happened in the training?*
	d) What do you think worked particularly well in the training, which would ensure that the participants took home specific messages that helped to change their practice?
	e) In your opinion, was the PCS training component of the HFI implemented as planned? *Ask for specifics. Do you think it had the desired impacts? Why?*

IMPLEMENTER IN-DEPTH INTERVIEW (3)	
**Domains, topic questions, and probes:** Use the table below to help you administer the questions during the interview.	
**Domain**	**Topic and Probes**
	f) In your opinion, was the HCM training component of the HFI implemented as planned? *If not, ask for specifics. Do you think it had the desired impacts? Why?*
	g) In your opinion, was the JUMP training component of the HFI implemented as planned? *If not, ask for specifics. Do you think it had the desired impacts? Why?*
	h) [For Joseph and Lucas] In your opinion, was the training of the Health Sub-District Liaisons implemented as planned? *If not, ask for specifics. Do you think it had the desired impacts? Why?*
**4. Training impact**	a) What impact do you think these trainings will have on the practices of participants in reality?

IMPLEMENTER IN-DEPTH INTERVIEW (4)	
**Domains, topic questions, and probes:** Use the table below to help you administer the questions during the interview.	
**Domain**	**Topic and Probes**
	b) What do you think can be strengthened in the training to enable health workers to really change their practice?
	c) To what extent do you feel the training with *unsalaried staff* had the desired impact? Why/not?
**5. Uptake of the intervention**	a) Aside from the training components, can you comment on whether the information tools (OPD) and management tools (ADDAT, order forms) provided were able to be taken up in practice?
	b) What things do you think are needed to help health workers to apply what they learned to their everyday work?

IMPLEMENTER IN-DEPTH INTERVIEW (5)	
**Domains, topic questions, and probes:** Use the table below to help you administer the questions during the interview.	
**Domain**	**Topic and Probes**
	c) In your opinion, what things might prevent health workers from using what they learned to their everyday work?
	d) From your experience, how has this varied for the different health centres / types of health centres?
**6. Implementation Process – Supply of AL & RDTs**	a) Can you please describe the main objectives for the implementation of the supply of AL and RDTS to health facilities component of the HFI?
	b) In your opinion, was the supply of AL and RDTs component of the HFI implemented as planned? *Do you think it had the desired impacts? Why?*

IMPLEMENTER IN-DEPTH INTERVIEW (6)	
**Domains, topic questions, and probes:** Use the table below to help you administer the questions during the interview.	
**Domain**	**Topic and Probes**
	c) What would you say has been the impact of the way that AL and RDTs have been supplied to health facilities through this intervention? *Probe for changes over the past year, and reasons for differences. Probe for the role of the HSD as a person in this process.*
**7. Implementation Process**	a) Looking back over the past year, what component of the intervention do you think was most successfully implemented? *Probe for specifics: how they know it was successfully implemented, and what impacts they have observed it having.*
	b) Looking back over the past year, what component of the intervention do you think was least successfully implemented? *Probe for specifics*: how they know it was not successfully implemented, and what impacts this had for staff/facilities.
**8. Motivation towards job**	a) We want to know how we could do this programme elsewhere. What skills and characteristics do you think are needed to do your job as a trainer/ implementer really well?

IMPLEMENTER IN-DEPTH INTERVIEW (7)	
**Domains, topic questions, and probes:** Use the table below to help you administer the questions during the interview.	
**Domain**	**Topic and Probes**
**9. Context**	a) Aside from the HFI, can you describe any other programmes/interventions involving malaria in the area? *Probe: is the programme at the community or health centre level?*
	b) Aside from the HFI, can you describe any other health-related programmes/interventions in the area? *Probe: is the programme at the community or health centre level?*
	c) Are there any other factors you think may have influenced the delivery or receipt of the ACT PRIME HFI? *(Probe for; 1) the effect of stocking by NMS through the push system and ACT PRIME through HFI on the management and dispensing of drugs, 2) the effect of any changes to the system for the distribution or stocking of drugs 2) Interference of local leaders affecting the work at the facility 3) changes in working days and working in shifts and HWs having their own working schedule 4) Changes in staffing at the health centres effected by the subcounty)*
**10. Closing**	Is there anything else you think is important about the implementation of the Health Facility Intervention intervention that we have not talked about?
✓ **Summarise**<br>✓ **Thank participant**	

PART 3: CONTACT SUMMARY FORM (1)	
**Complete this form after the interview.**	
**Study ID**<br>[____|____]	**Date**<br>[____|____] / [____|____] / [____|____]<br>day month year
1. How would you describe the atmosphere and context of the interview (*Include interview location and how this may have affected responses)*?	
2. What were the main points made by the respondent during this interview?	

PART 3: CONTACT SUMMARY FORM (2)	
**Study ID**<br>[____|____]	**Date**<br>[____|____] / [____|____] / [____|____]<br>day month year
3. What new information did you gain through this interview compared to previous interviews?	
4. Was there anything surprising to you personally? Or that made you think differently?	
5. What messages did you take from this interview to improve the intervention design?	
6. Were there any problems with the topic guide (e.g. wording, order of topics, missing topics) you experienced in this interview?	

APPENDIX R: IDI DATA COLLECTION TOOL
HEALTH WORKERS (HFI)

Health centre code	Study ID	Date
[____|____]	[____|____]	[____|____] / [____|____] / [____|____]<br>day month year

Position:

1 = In-charge	5 = Clinical officer	9 = Public health nurse	13 = Health assistant	
2 = Senior medical officer	6 = Nursing officer	10 = Nursing aide/assistant	14 = Health educator	
3 = Medical officer	7 = Enrolled nurse	11 = Laboratory technician	15 - Volunteer	
4 = Senior clinical officer	8 = Midwife	12 = Laboratory assistant	15 = Other_______________	[____|____]

DEMOGRAPHIC INFORMATION

**1. Age**	Years	[____|____]
**2. Gender**	1 = Male<br>2 = Female	[____]
**3. Originally from this area?**	1 = Yes<br>2 = No	[____]
**4. Number of years worked in this job**		[____|____]

5. Highest level of education or qualification achieved

1 = Primary (P1 — P7)
2 = Secondary (S1 — S6)
3 = Certificate
4 = Diploma
5 = Bachelor's degree
6= Master's degree
99 = Refused to answer
77 = Other___________________________

[____|____]

6. Year graduated [____|____|____|____]

PART 1: INTRODUCTION

Conduct the interview according to the directions below and record information as indicated.

Introduction to in-depth interview

"Hello my name is ___________________ and I am interested in interviewing you. This interview will ask you to express your own views and experiences about your work and role at this health centre. We are interested in knowing whether improving the health services at this health centre has improved children's health in this area. We are specifically asking you about the ACT PRIME study activities which include 1) health center management training, 2) information management, 3) health worker training in fever case management and patient-centered services, and 4) supply of consumables, including malaria diagnostics and antimalarial drugs.

A note-taker will be writing down what you say for our records, and we will record the interview using a digital recorder; these notes will be kept securely and your name will not be used anywhere. Your answers will be looked at together with those of many other health workers from different facilities and you will not be identifiable in any reports that are published.

It is very important for us to hear your views and experiences because you have experience working here and can give us this insight. We hope you will have time to spend with us now to complete this interview. The interview will take about 45 minutes; if you prefer we can reschedule the interview for tomorrow or another day of your convenience.

Do you have any questions? Do you agree to continue before we start?

Now we request that we all switch off our mobile phones so that we are not distracted."

NOTE TO INTERVIEWER: For this interview, bring a copy of the ADDAT form, Stock Card, Order Form for EMHS and the two instruction sheets: 'using the stock card' and 'using the order form'

PART 2: IN-DEPTH INTERVIEW – HEALTH WORKERS (HFI) (1)	
**Domains, topic questions, and probes:** Use the table below to help you administer the questions during the interview.	
**Domain**	**Topic and Probes**
**1. Your role at work**	a) What does your usual day consist of at the health centre these days?
	b) What is the most important thing to you personally about doing this job?
	c) How do you feel about this job now? How has this changed over time?
**2. Significant events**	a) Looking back over the past year, what do you think was the most significant change in the way you managed illness in your health centre?
	b) Why is this significant to you?
	c) What difference has this made now or will it make in the future?
**3. Reflection on HFI**	a) The ACT PRIME project has carried out some activities at your health facility and others in this area since April 2011. Can you tell me about any that you have been involved with or that have affected you?<br><br>*Probe for all aspects of the intervention they can recall, and what they remember about each (it may be different from the way we frame the intervention, but we want to hear their description of what it meant to them)*

PART 2: IN-DEPTH INTERVIEW – HEALTH WORKERS (HFI) (2)	
**Domains, topic questions, and probes:** Use the table below to help you administer the questions during the interview.	
**Domain**	**Topic and Probes**
**4. Reflection on training**	a) What training did you attend with the ACT PRIME project since April last year? *Probe for a list of all components they can recall, in their own words.*<br><br>*Once they have listed them all, prompt them to see what they recall about: PCS training, PCS self-observation tasks, HCM training, RDT training, RDT supervision.* E.g. Between the PCS workshops, there were some suggestions for things to think about when you return to your individual health facilities that were written in your learner manuals. What can you tell me about those?'
	b) How do you feel the ACT PRIME study training you attended has impacted on your work?
	c) Was there anything that you learnt during the training that you have found difficult to put into practice? *Probe for each of the training sessions they can recall attending above- but further probes can be brought in under subsequent domains for each intervention components*
	d) Have you attended any other training courses or received any materials or tools from other organizations to help you do your job? *If yes, please list, and let us know what was most useful about each of those courses, materials or tools.*
**5.A Health centre management:** Staffing	a) How would you describe the staffing levels at your health centre right now? *Probe for number of staff, qualifications and status e.g permanent vs locum or temporary*
	b) Have there been any changes recently to the staffing at this health centre? *Probe: specifics and who effected these changes (district or Sub County)? How does the leave system operate, and how do you feel about this?*

PART 2: IN-DEPTH INTERVIEW – HEALTH WORKERS (HFI) (3)	
**Domains, topic questions, and probes:** Use the table below to help you administer the questions during the interview.	
**Domain**	**Topic and Probes**
	c) What difference have these changes made to your work? *Probe: Which part of your every day work is greatly affected by the staffing levels at your facility*
	d) What is the role of volunteers at your health centre? *Probe: how do you feel about the use of volunteers here?*
	e) What challenges do you still face in staffing at this health centre? *(try to keep this brief- there will be many challenges!)*
**5.B Health centre management:** Drug stocking implementation	a) Can you describe the way that drugs are stocked at this health centre? *Probe: how has this changed over the past year – including when changes were implemented.*
	b) Can you describe your relationship with the health sub-district (HSD) liaison, and what role he plays in stocking at your health facility?
	c) How often do you use the stock card? What do you think of this as a method to keep track of stocks? *NB: bring the one-pager instructions 'using the stock card' and see if they are familiar with this. What is useful/not useful about this instruction sheet? What was most useful for knowing how to complete the stock card?*
	d) How often do you use the 'order form for EMHS'? What do you think of this as a method to order supplies? *NB: bring the one-pager instructions 'using the order form' and see if they are familiar with this. What is useful/not useful about this instruction sheet? What was most useful for knowing how to complete the order form?*

PART 2: IN-DEPTH INTERVIEW – HEALTH WORKERS (HFI) (4)	
**Domains, topic questions, and probes:** Use the table below to help you administer the questions during the interview.	
**Domain**	**Topic and Probes**
**5.C Health centre management:** Drug stocking impact	a) How have any changes in stocking at your health facility affected your work? *Probe: What is the impact on hours spent at work, patient attendance etc?*
	b) What has been the effect of stocking by both NMS through the push system and ordering drugs from the HC IV on the management and dispensing of drugs? *Probe for sharing of drugs and RDTs and which HCs they usually share drugs with (between HFI and Standard care facilities?)*
	c) What challenges do you still face in stocking of drugs at this health centre?
	d) How would you describe the function of the ADDAT form for re-stocking activities? *Probe for whether they are using the ADDAT for restocking activities – show the ADDAT form if necessary and see if they recognize it.*
	e) When you run out of Lumartem or Coartem and RDTs, what method is your first choice for getting more supplies? What is the process of that method, and how did you learn about that process? *Probe for any trickle down of training on how to requisition for supplies from in-charges to other health workers at the health centre*
	f) How does the system for requisitioning supplies through the HC IV compare with other methods you use to get supplies including the the NMS system? *Probe for preferences and reasons; how could ordering supplies from the HC IV system be integrated with the NMS system?*

PART 2: IN-DEPTH INTERVIEW – HEALTH WORKERS (HFI) (5)	
**Domains, topic questions, and probes:** Use the table below to help you administer the questions during the interview.	
**Domain**	**Topic and Probes**
**5.D Health centre management:** Budgeting and accounting	a) How would you describe the financial situation at this health centre right now?
	b) How would you describe the accounting and budgeting for PHC funds or other funds at this health centre right now?
	c) Have you made any changes over the past year to the way you undertake accounting and budgeting of PHC funds or other funds at this health centre? *Probe for specifics.*
	d) What difference, if any, have these changes made to your work? *Probe: where do you see the greatest impact of accounting and budgeting in your every day work?*
	e) How would you describe the function of the PHC Fund Accounting Tool for accounting for activities at the health facility? *Probe for specifics.*
	f) How has the requisition for and delivery of PHC funds for your health centre been working in the past year? *Probe: what impact has this had on the operation of your health centre?*
**5.E Health centre management:** Information management	a) What is the information you collect about patients at your health centre in the OPD register used for?
	b) (How) have you and your colleagues at your health centre used the information documented in the OPDs? *Probe: how has the way you have used this information changed in the past year?*

PART 2: IN-DEPTH INTERVIEW – HEALTH WORKERS (HFI) (6)	
**Domains, topic questions, and probes:** Use the table below to help you administer the questions during the interview.	
**Domain**	**Topic and Probes**
	c) What impact does the way this information is used have on your work?
	d) What problems did or have you experienced in completing the OPD for recording fever, malaria tests and treatment at your health centre?
**6.A Reflection on Patient-Centered Services:** Communication with patients	a) How would you describe your relationship with the different types of patients who come to this health centre? *Probe for different types of patients (age, gender, type of illness, perceived social status) and how the relationship varies. Probe for the relationships they observe that their colleagues have with patients.*
	b) What is the most significant change in the past year in the way you interact with patients? *Probe: why do you think this change occurred and how did you achieve it?*
	c) Have you noticed any differences in the types of patients who attend at your health centre in the past year? *Probe: any differences in the socioeconomic status, catchment area or social group of patients coming now? If so, how are these different groups treated by colleagues at the health centre?*
	d) Can you describe the impact your relationship with patients has on your work? *Probe for any changes to this relationship. Probe for impact on own sense of wellbeing.*
	e) The unsalaried staff at different health centres were invited to some training last year about improving the way they welcome and interact with patients. Have you noticed any changes in these behaviours since then? *Probe for stories of examples of change/no change; probe for why they think these changes occurred and if they think the training was worthwhile*

PART 2: IN-DEPTH INTERVIEW – HEALTH WORKERS (HFI) (7)	
**Domains, topic questions, and probes:** Use the table below to help you administer the questions during the interview.	
**Domain**	**Topic and Probes**
	f) Have there been any changes in the order in which patients are seen at your health centre? If so, what is the reason, and what have been the consequences?
**6.B Patient-Centered Services:** Communication with colleagues	a) How would you describe your relationship with your colleagues at your health centre?
	b) What do you think are the reasons for the nature of this relationship with colleagues, as you described it?
	c) What is the most significant change you have experienced in the past year in the way you and your colleagues interact? *Probe: why do you think this change occurred and how are you all achieving it?*
	d) Can you describe the impact your relationship with colleagues has on your work?
**7.A Fever case management**	a) Can you tell me about your experiences with the RDT trainers since last year? *Probe for what their interactions with the RDT trainers consisted of (i.e. training, supervision, time points)*
	b) Can you tell me what were the most important things that you learned from the RDT trainers? *Probe for each: can you remember when you learnt that? Why do you think you can still remember that now?*

PART 2: IN-DEPTH INTERVIEW – HEALTH WORKERS (HFI) (8)	
**Domains, topic questions, and probes:** Use the table below to help you administer the questions during the interview.	
**Domain**	**Topic and Probes**
	c) Were there any parts of the recommendations made by the RDT trainers that you have found hard to put into practice? *Probe for any difficulties with mechanisms of doing the different types of tests (pf/pan (2 lines) vs bioline (3 lines), including loop vs dropper); how did these different methods affect your use of the tests (frequency/ inclination)*
	d) Did you receive a visit from the RDT trainers to your health facility? Can you tell me what they did when they came? What was most useful to you, and why?
	e) Can you make any recommendations for what could be improved about the RDT training?
**8. Satisfaction**	a) How would you describe your personal satisfaction with your job at this health centre? *Probe for reasons for satisfaction/dissatisfaction*
	b) Can you describe what impact your satisfaction/dissatisfaction has on your work?
**9. Closing**	Is there anything else you think is important about working at this health centre that we have not talked about? (*Probe for challenges like interference by political leaders, faultfinding supervision)*
✓ **Summarise**<br>✓ **Thank participant**	

PART 3: CONTACT SUMMARY FORM (1)	
**Interviewer to complete this form after the interview**	
**Study ID**<br>[____|____|____|____]	**Date**<br>[____|____] / [____|____] / [____|____]<br>day month year
1. How would you describe the atmosphere and context of the interview (*Include interview location and how this may have affected responses)*?	
2. What were the main points made by the respondent during this interview?	

PART 3: CONTACT SUMMARY FORM (2)	
**Study ID**<br>[____|____|____|____]	**Date**<br>[____|____] / [____|____] / [____|____]<br>day month year
3. What new information did you gain through this interview compared to previous interviews?	
4. Was there anything surprising to you personally? Or that made you think differently?	
5. What messages did you take from this interview to improve the intervention design?	
6. Were there any problems with the topic guide (e.g. wording, order of topics, missing topics) you experienced in this interview?	

APPENDIX S: IDI DATA COLLECTION TOOL
KEY STAKEHOLDERS

Study ID	Date
[____|____]	[____|____] / [____|____] / [____|____]<br>day month year

Position:

1 = District Health Officer 5 =Chief Administrative Officer 9 = Other_______________________
2 = District Health Inspector 6 = Malaria Focal Person
3 = Deputy District Health Officer 7 = Local Chairman
4 = Principal Nursing Officer 8 = MoH staff, Dept_________________________

[____|____]

DEMOGRAPHIC INFORMATION

**1. Age**	Years [____|____]	**5. Highest level of education or qualification achieved**<br>1 = Primary (P1 — P7)<br>2 = Secondary (S1 — S6)<br>3 = Certificate<br>77 = Other____________________________	4 = Diploma<br>5 = Bachelor's degree<br>6=Master's degree<br>99 = Refused to answer<br>[____|____]
**2. Gender**	1 = Male<br>2 = Female [____]		
**3. Originally from this area?**	1 = Yes<br>2 = No [____]		
**4. Number of years worked in this job**	[____|____]	**6. Year graduated**	[____|____|____|____]

PART 1: INTRODUCTION

Conduct the interview according to the directions below and record information as indicated.

Introduction to in-depth interview

"Hello, my name is ………. I work with UMSP (Uganda malaria Surveillance project)/IDRC (Infectious Diseases Research Collaboration). I am interested in asking you a few questions about activities, programmes or other contextual factors occurring in Tororo District or across Uganda in the past year. A note-taker will be writing down what you say for our record and we will record the interview using a digital recorder; these notes will be kept securely and your name will not be used anywhere. Your answers will be looked at together with those of many other health workers from different facilities and you will not be identifiable in any reports that are published.

It is very important for us to hear your views and experiences because your knowledge and experience can give insight to our study. We hope you will have time to spend with us now to complete this interview. The interview will take about 45 minutes; if you prefer we can reschedule the interview for tomorrow or another day of your convenience.

Do you have any questions? Do you agree to continue before we start?

Now we request that we all switch off our mobile phones so that we are not distracted."

PART 2: STAKEHOLDERS IN-DEPTH INTERVIEW (1)	
**Domains, topic questions, and probes:** Use the table below to help you administer the questions during the interview.	
**Domain**	**Topic and Probes**
**1. Description of job**	a) Can you briefly describe your roles and responsibilities in your job?
	b) What specific role do you play in malaria-related programmes?
	c) Can you describe your involvement, if any, with the implementation of the ACT PRIME health facility intervention?
**2. Significant changes**	a) Looking back over the past year, what do you think is the most significant change in the way illnesses are managed in Tororo, particularly in West-Budama North and South?
	b) Why is this significant to you?
	c) What difference has this made now or will it make in the future?
**3. Changes in HFI components:** Staffing	*For each of the following questions, probe for specific examples of health facilities in order to draw comparisons between intervention and standard care facilities.*
	a) Can you describe any actions taken to change the staffing at health centres in the area? *Probe: actions taken for all health centres or only some? Which ones, and detail of HWs?* <br>*Include sub county key informants to capture these changes effected at the sub county – kisoko, petta and Paya. Challenging areas –Petta, Makawari, Mbula*

PART 2: STAKEHOLDERS IN-DEPTH INTERVIEW (2)	
**Domains, topic questions, and probes:** Use the table below to help you administer the questions during the interview.	
**Domain**	**Topic and Probes**
Volunteers	b) Can you tell me about the status of volunteers at health facilities in Tororo over the past year? *Probe: any changes to their roles, numbers, remuneration?*
Absences	c) The district figures show a reduction in health worker numbers in the past year. What measures have been taken in this situation to ensure there are staff at the facilities? *Probe: how do you deal with leave requests?*
Drug stocking	d) Can you describe any changes to how drugs and other supplies are stocked at health centres in the area? *Probe: changes in all health centres or only some? Which ones? When were these changes implemented?*
PHC fund	e) Have there been any changes to the PHC fund requests you have received? Or change to the way the requisition and deliveries of funds have occurred? *Probe for any differences between intervention and standard care facilities.*
OPD register	f) Can you describe any changes to the attendance and case mix information (type, quality, quantity) you receive from health centres in the area? *Probe: changes in all health centres or only some? Which ones?*
Relationship with patients (skip for district officials)	g) Can you describe any changes you have observed in the relationships between health workers and patients/community members in the area? *Probe: changes in all health centres or only some? Which ones? What types of patients are affected by these relationships?*

PART 2: STAKEHOLDERS IN-DEPTH INTERVIEW (3)	
**Domains, topic questions, and probes:** Use the table below to help you administer the questions during the interview.	
**Domain**	**Topic and Probes**
Motivation of health workers (skip for district officials)	h) How would you describe the levels of motivation of health workers at the different health centres here? *Probe: Please tell me what you think for each health centre in West Budama North and South, and why you think they are more or less motivated there (prompt with a list of health centres if necessary).*
**The PRIME intervention consisted of a series of workshops last year, training health workers in malaria case management and use of RDTs, as well as in communication skills with patients and ways to improve health centre management. This was only done at 10 health centres. Now we would like to know what changes have been as a result of the PRIME study and what changes may be due to other activities happening here.** *For each of the following, probe for when, where and the scale of the change.*	
**4. Contextual factors** (skip for key informants taking part in the context record)	a) Can you describe any other changes to health centres in Tororo beyond those we just discussed (i.e. opening or closing of health centres, improvements to health centres)? *Probe: Who is responsible for these changes?*
	b) Can you describe any changes to environmental conditions in Tororo (i.e. severe weather, new roads, swamps, agriculture)?
	c) Can you describe any changes to guidelines or practices regarding malaria testing and treatment at health centres or the community level?
	d) Can you describe any messages or news stories on the radio, TV or newspaper about malaria testing/prevention/treatment or malaria programmes?
	e) Can you describe any other economic or political factors that you think may have impacted the delivery or receipt of the ACT PRIME health facility intervention?

PART 2: STAKEHOLDERS IN-DEPTH INTERVIEW (4)	
**Domains, topic questions, and probes:** Use the table below to help you administer the questions during the interview.	
**Domain**	**Topic and Probes**
**5. Other programmes** (skip for key informants taking part in the context record)	a) Besides ACT PRIME, can you describe any other programmes involving malaria in the area? *Probe: are these programmes at the community or health centre level?*
	b) Besides ACT PRIME, can you describe any other health-related programmes in the area? *Probe: are these programmes at the community or health centre level?*
	c) What other training programs involving community health workers or health centre staff are taking place in the area?
**6. Support for the intervention**	a) In your opinion, what is the level of support from health workers for the ACT PRIME health facility intervention?
	b) In your opinion, what is the level of support from the health sub district/ sub county staff for the ACT PRIME health facility intervention?
	c) In your opinion, what is the level of support from district-level staff for the ACT PRIME health facility intervention?
	d) Do you think the PRIME intervention should be scaled up to other health centres? Which components and why?
**7. Closing**	Are there any other issues about the PRIME intervention you would like to add?
✓ **Summarise**	

- ✓ **Thank participant**

PART 3: CONTACT SUMMARY FORM (1)	
**Complete this form after the interview.**	
**Study ID**<br>[____|____]	**Date**<br>[____|____] / [____|____] / [____|____]<br>day month year
1. How would you describe the atmosphere and context of the interview (*Include interview location and how this may have affected responses)*?	
2. What were the main points made by the respondent during this interview?	

PART 3: CONTACT SUMMARY FORM (2)	
**Study ID**<br>[____|____]	**Date**<br>[____|____] / [____|____] / [____|____]<br>day month year
3. What new information did you gain through this interview compared to previous interviews?	
4. Was there anything surprising to you personally? Or that made you think differently?	
5. What messages did you take from this interview to improve the intervention design?	
6. Were there any problems with the topic guide (e.g. wording, order of topics, missing topics) you experienced in this interview?	

THE REPUBLIC OF UGANDA

[____]—[____|____|____|____]

SSQ Study ID

APPENDIX T: SEMI-STRUCTURED QUESTIONNAIRES
Informed consent form for health workers and private providers

**Protocol Title:**	ACT PROCESS: Evaluating the process, context, and impact of interventions to enhance health facilities in Tororo, Uganda
**Site of Research:**	Tororo, Uganda
**Principal Investigators**:	Dr. Sarah Staedke
**Date:**	15 April 2013

Introduction

Dr. Sarah Staedke and colleagues from the Uganda Malaria Surveillance Project/Infectious Diseases Research Collaboration are doing a study to evaluate activities that have been introduced at government-run health facilities in this area aiming to improve the health of children. We would like to understand the impact of these activities and why they are working, or not. This study involves several parts, including assessing the communication between health workers and their patients, holding interviews, and conducting group discussions with community members and health workers.

Why is this study being done?

For this part of the study, we would like to learn more about whether the activities that have been introduced at government-run health facilities have had an impact from the perspective of health workers and private drug shop workers. This information will help us understand how and why the health facility activities have affected the health of children in this area.

What will happen today if I take part in this study?

The study will involve a one-time interview. Today, if you are a health worker, we would like to ask you some questions about drug stocks and health center management, diagnosis and treatment of fever and malaria, your attitudes and beliefs about your job, and any changes you have seen over the past few months at your health center. We may also leave some pages of the questionnaire for you to complete in your own time over the next three days. If you are a health worker who participated in the ACT PRIME training, we will also ask you some additional questions about the usefulness of the training. If you work in a private drug shop, we would like to ask you questions about stocks of drugs and diagnostics for malaria, treatment of fever and malaria, patient attendance, and any changes you have seen over the past few months at your shop. The information we collect will be used by project investigators, and may be shared with other researchers and policy-makers to answer questions about how best to deliver training and health services.

THE REPUBLIC OF UGANDA

How long will the study last?

Today, the interview will last about 60-90 minutes.

Can I stop being in the study?

You can decide to stop participating at any time. Just tell the project researcher right away if you wish to stop.

What risks can I expect from being in the study?

Participation in any research study may involve a loss of privacy. Your name will not be used in any reports of the information provided. No quotes or other results arising from your participation in this study will be included in any reports, even anonymously, without your agreement. The information obtained from these interviews will be locked at our project offices. We will do our best to make sure that the personal information gathered for this study is kept private.

Are there benefits to taking part in the study?

There will be no direct benefit to you from participating in this study. However, the information that you provide will help researchers and policy-makers understand how best to improve health services in this area.

What other choices do I have if I do not take part in this study?

You are free to choose not to participate in the study. If you decide not to take part in this study, there will be no penalty to you.

What are the costs of taking part in this study? Will I be paid for taking part in this study?

There are no costs to you for taking part in this study. You will not be paid for taking part in this study.

What are my rights if I take part in this study?

Taking part in this study is your choice. You may choose either to take part or not to take part in the study. If you decide to take part in this study, you may change your mind at any time. No matter what decision you take, there will be no penalty to you in any way.

Who can answer my questions about the study?

You can talk to the researchers about any questions or concerns you have about these study activities. Contact Dr. Sarah Staedke or other members of the Uganda Malaria Surveillance Project / Infectious Disease Research Collaboration on telephone number 0414-530692. If you have any questions, comments or concerns about taking part in these activities, first talk to the researchers. If for any reason you do not wish to do this, or you still have concerns about doing so, you may contact Dr Charles Ibingira, Makerere University Faculty of Medicine Research and Ethical Committee at telephone number 0414-530020.

THE REPUBLIC OF UGANDA

WHAT YOUR SIGNATURE OR THUMBPRINT MEANS

Your signature or thumbprint below means that you understand the information given to you in this consent form about your participation in the study and agree with the following statements:

1. "I have read the consent form concerning this study (or have understood the verbal explanation of the consent form) and I understand what will be required of me and what will happen to me if I take part in it."

2. "My questions concerning this study have been answered by Dr. Staedke or the person who signed below."

3. "I understand that at any time, I may withdraw from this study without giving a reason."

4. "I agree to take part in this study."

If you wish to participate in this study, you should sign or place your thumbprint on the following page. If you do not agree to quotes or other results arising from your participation in the study being included, even anonymously, in any reports about the study, please tell the researcher now.

WE WILL GIVE YOU A COPY OF THIS SIGNED AND DATED CONSENT FORM

Name of Participant (printed)

Signature or Fingerprint of Participant — Date/Time

Name of Investigator Administering Consent (printed) — Position/Title

Signature of Investigator Administering Consent — Date/Time

If the participant is unable to read and/or write, an impartial witness should be present during the entire informed consent discussion.

After the written informed consent form is read and explained to the participant, and after they have orally consented to participate in the study, the witness should print the name of the participant above, and then the participant should provide their fingerprint.

Then the witness should print their name, provide their signature, and date the consent form below.

By signing the consent form, the witness attests that the information in the consent form and any other written information was accurately explained to, and apparently understood by the participant, and that informed consent was freely given by the participant.

Name of Person Witnessing Consent (printed)

Signature of Person Witnessing Consent — Date/Time

APPENDIX U: SEMI-STRUCTURED QUESTIONNAIRE
HEALTH WORKERS: INTERVENTION ARM

Health centre code	Health Worker ID	Date
[____|____]	[____|____|____|____]	[____|____] / [____|____] / [____|____]<br>day month year

Position:

1 = In-charge	5 = Clinical officer	9 = Public health nurse	13 = Health assistant
2 = Senior medical officer	6 = Nursing officer	10 = Nursing aide/assistant	14 = Health educator
3 = Medical officer	7 = Enrolled nurse	11 = Laboratory technician	15 - Volunteer
4 = Senior clinical officer	8 = Midwife	12 = Laboratory assistant	15 = Other________________

[____|____]

DEMOGRAPHIC INFORMATION

**1. Age** Years [____|____]	**5. Highest level of education or qualification achieved**<br>0 = None<br>1 = Primary (P1 — P7)<br>2 = Secondary (S1 — S6)<br>3 = Certificate<br>77 = Other________________________<br>4 = Diploma<br>5 = Bachelor's degree<br>88 = Don't know<br>99 = Refused to answer<br>[____|____]
**2. Gender** 1 = Male 2 = Female [____]	
**3. 'Are you originally from this area?'** 1 = Yes 2 = No [____]	
**4. 'How long have you worked at this health centre?'** [____|____]years [____|____]months	**6. What year did you graduate from your course?** [____|____|____|____]
**7. How many training workshops have you attended in the past year?** [____|____]	**8. Have you received official training on doing malaria RDT?** 1 = Yes 2 = No [____]

INTRODUCTION TO QUESTIONNAIRE

Thank you for considering completing this questionnaire. The questions will ask you to express your own views and experiences about your work and role at your health centre. We are interested in knowing how the health services operate at this health centre and how this affects children's health in this area. This is ***NOT A TEST*** *and* ***WE VALUE YOUR OPINION.*** *We hope that the information you share with us will help us to know ways to improve services for health care in Tororo district.*

Your responses will be kept for our records. They will be kept securely and your name will not be used anywhere. Your answers will be looked at together with those of many other health workers from different facilities and you will not be identifiable in any reports that are published.

It is very important for us to hear your views and experiences because you have experience working here and can give us this insight. We hope you will have time to spend to complete this questionnaire in full. The questionnaire should take about half an hour.

Please read the instructions for completing the questionnaire on the next page carefully. If you have any queries about the questionnaire including the meaning of questions or how to make your response, please ask a member of the research team for clarification or make a note by the question on this questionnaire.

GENERAL INSTRUCTIONS TO COMPLETE THIS QUESTIONNAIRE

1. *Please use a dark coloured pen to fill out the questionnaire*

2. *Your health worker identity (ID) number is the unique number that was given to you at the start of this PRIME project. When we request you to give your ID number, please fill the boxes as below:*

   [_0_|_5_|_1_|_0_]

   You are asked to enter your ID number like this at the top of each page of your questionnaire.

3. *In this questionnaire, we ask you to read each question carefully and either write your response in the space, or look at the statements and decide which response is closest to your own opinion. When you have decided, please circle the number in the column under that response. For example:*

Decide how much you agree with the statements below	***Strongly agree***	***Agree***	***Disagree***	***Strongly disagree***
We always have enough ACTs in stock at this health centre	(1)	2	3	4

4. *If you change your mind and would prefer to circle a different response, please cross out your original choice and circle the choice that fits your opinion best. For example if you change your mind and you decide that your answer is 'disagree', cross through the original and circle the new response as below:*

Decide how much you agree with the statements below	***Strongly agree***	***Agree***	***Disagree***	***Strongly disagree***
We always have enough ACTs in stock at this health centre	~~(1)~~	2	(3)	4

5. *When you have completed the questionnaire, please ensure you have completed everything in the following checklist:*

- ☐ *I have completed my demographic details at the top of the form*
- ☐ *I have answered all of the questions in this questionnaire*
- ☐ *I have checked that I have circled the responses that are closest to my opinion*
- ☐ *I have written my health worker ID number on all of the pages of the questionnaire*
- ☐ *I have written my health worker ID number on the envelope and will place this completed form inside the envelope*

SSQ: HEALTH WORKERS (1)

Health centre code	Health Worker ID	Date
[____|____]	[____|____|____|____]	[____|____] / [____|____] / [____|____]<br>day month year

PART 1: ALL HEALTH WORKERS

1: CHANGES AT WORK

1.1. Think about changes at your health centre over the past year (since April 2011). Have there been any changes in the way patients are managed?

Yes	*No*
1	*2*

1.2. What do you think were the most significant or important changes in the way you managed patient illnesses at your health centre over the past year (since April 2011)?

1.3. Why are these changes significant or important to you?

1.4. What difference have these changes made to the patients coming to this health centre?

1.5. What difference have these changes made to you and your colleagues in your work at this health centre?

SSQ: HEALTH WORKERS (2)

Health centre code	Health Worker ID	Date
[____|____]	[____|____|____|____]	[____|____] / [____|____] / [____|____]<br>day month year

2. HEALTH FACILITY MANAGEMENT & SUPPLIES

Decide how much you agree with the statements below, and circle the number closest to your opinion on the right	Strongly agree	Agree	Disagree	Strongly disagree	Don't know
2.1. We always have enough ACTs in stock at this health centre	1	2	3	4	DK
2.2. We always have other drugs (apart from ACTs) that we need in stock at this health centre	1	2	3	4	DK
2.3. We always have enough RDTs in stock at this health centre	1	2	3	4	DK
2.4. At this health centre, we are often out of stock of supplies that are necessary for our work	1	2	3	4	DK
2.5. The storage facilities at this health centre are not suitable for storing all of our drugs, tests and other supplies	1	2	3	4	DK
2.6. We do not have enough money to purchase all of the supplies we need for the every-day running of this health centre (e.g. for soap, repairs)	1	2	3	4	DK
2.7. We do not have enough money to pay for the staff we need to clean and help us at this health centre	1	2	3	4	DK
2.8. There are staff working at this health centre who are good at completing budget and accounting forms	1	2	3	4	DK
2.9. In the last 6 months, the PHC fund has been enough for our needs at this health centre	1	2	3	4	DK
2.10. In the last 6 months, we have received PHC funds when we requested them	1	2	3	4	DK
2.11. In my opinion, the PHC funds are often used in the wrong way at this health centre	1	2	3	4	DK
2.12. I know the number of patients being seen at this health centre each month	1	2	3	4	DK
2.13. I know the number of patients who are confirmed with malaria at this health centre each month	1	2	3	4	DK
2.14. The reports of patient numbers at this health centre have been useful in knowing how many drugs to order	1	2	3	4	DK
2.15. The reports of patient numbers at this health centre have been useful in seeing how we are managing fever cases	1	2	3	4	DK

2.16. Please provide any further comments relating to health facility management & supplies or any of the questions above:

SSQ: HEALTH WORKERS (3)		
**Health centre code**	**Health Worker ID**	**Date**
[____|____]	[____|____|____|____]	[____|____] / [____|____] / [____|____]<br>day month year

**3. CASE MANAGEMENT**					
**Decide how much you agree with the statements below, and circle the number closest to your opinion on the right**		**Strongly agree**	**Agree**	**Disagre e**	**Strongly disagree**
3.1.	I am able to identify patients with malaria without using a malaria test	1	2	3	4
3.2.	I never give an antimalarial without first testing the patient for malaria	1	2	3	4
3.3.	It is not possible to test every patient for malaria because there is not enough time	1	2	3	4
3.4.	It is not possible to test every patient for malaria because sometimes we do not have the supplies we need	1	2	3	4
3.5.	Not all patients are willing to be tested	1	2	3	4
3.6.	Sometimes I think that malaria tests are not performed correctly at my health centre	1	2	3	4
3.7.	I do not always trust the quality of Rapid Diagnostic Tests (RDTs) for malaria	1	2	3	4
3.8.	If the malaria test is negative but the patient has fever, it is best to prescribe an antimalarial drug to be safe	1	2	3	4
3.9.	In my experience, fevers are usually due to malaria	1	2	3	4
3.10.	Patients who have uncomplicated malaria should always be given an antimalarial containing artemisinin	1	2	3	4
3.11.	I am confident that I can diagnose correctly those fevers that are not due to malaria	1	2	3	4
3.12.	I am confident that I can treat those fevers that are not due to malaria	1	2	3	4
3.13.	At my health centre, we do not have enough tests to diagnose fevers that are not due to malaria	1	2	3	4
3.14.	At my health centre, we do not have enough drugs to treat fevers that are not due to malaria	1	2	3	4
3.15.	I cannot give a full explanation to every patient about their diagnosis and treatment because I do not have time	1	2	3	4
3.16.	If a patient thinks that they have malaria, I find it hard to send them away without an antimalarial drug	1	2	3	4
3.17.	There are times when I want to refer a patient but cannot because of that patient's circumstances	1	2	3	4

3.18. Please provide any further comments relating to case management or any of the questions above:

SSQ: HEALTH WORKERS (4)

Health centre code	Health Worker ID	Date
[____|____]	[____|____|____|____]	[____|____] / [____|____] / [____|____]<br>day month year

4. INTERACTIONS WITH PATIENTS				
**Decide how much you agree with the statements below, and circle the number closest to your opinion on the right**	**Strongly agree**	**Agree**	**Disagree**	**Strongly disagree**
4.1. There is no harm in patients looking up to their health workers	1	2	3	4
4.2. A health worker should not always be expected to be willing to deal with non-medical problems	1	2	3	4
4.3. There are some patients to whom it would be useless to explain things as they would not understand	1	2	3	4
4.4. Health workers should always explain why they believe the patient is experiencing her or his symptoms	1	2	3	4
4.5. When a health worker prescribes medicine she or he must always explain what its effects are in detail	1	2	3	4
4.6. If a health worker is not sure about a patient’s diagnosis, she or he should not show this uncertainty to the patient	1	2	3	4
4.7. Patients have the right at all times to demand information from the health worker about their health	1	2	3	4
4.8. It is not the health worker’s job to provide emotional care to patients	1	2	3	4
4.9. If a patient takes herbs before coming to the health centre, she or he should be blamed if the sickness becomes worse	1	2	3	4

4.10. Please provide any further comments relating to your interactions with patients or any of the questions above:

5. FEELINGS ABOUT WORK				
**Decide how much you agree with the statements below, and circle the number closest to your opinion on the right**	**Strongly agree**	**Agree**	**Disagree**	**Strongly disagree**
5.1. Overall I am very satisfied with my job	1	2	3	4
5.2. These days, I don’t feel motivated to work as hard as I could	1	2	3	4
5.3. I only do this job so that I get paid at the end of the month	1	2	3	4
5.4. I am proud to be working for this health centre	1	2	3	4
5.5. I feel very little commitment to this health centre	1	2	3	4
5.6. I intend to leave this health centre	1	2	3	4
5.7. I feel emotionally worn out at the end of every day	1	2	3	4

SSQ: HEALTH WORKERS (5)		
**Health centre code**	**Health Worker ID**	**Date**
[____|____]	[____|____|____|____]	[____|____] / [____|____] / [____|____]<br>day month year

5. FEELINGS ABOUT WORK (CONTINUED)

**Decide how much you agree with the statements below, and circle the number closest to your opinion on the right**		**Strongly agree**	**Agree**	**Disagree**	**Strongly disagree**
5.8.	When I get up in the morning I fear having to face another day at work	1	2	3	4
5.9.	I would recommend to my children that they become a health worker like me	1	2	3	4
5.10.	I wish that I had chosen a different occupation from being a health worker	1	2	3	4
5.11.	I am afraid that I may not be able to stay in my current job in the future	1	2	3	4
5.12.	I cannot complete all of the work I am expected to do each day	1	2	3	4
5.13.	The health workers work well together in this health centre	1	2	3	4
5.14.	There is too much gossip in this health centre	1	2	3	4
5.15.	The health workers who are best at their job are the ones who get promoted	1	2	3	4
5.16.	High success on the job is shown in our pay	1	2	3	4
5.17.	The income I receive is fair, given my skills, knowledge and training	1	2	3	4
5.18.	This health centre provides everything I need to do my job well	1	2	3	4
5.19.	Suggestions made by health workers on how to improve their work are usually ignored by the district	1	2	3	4
5.20.	The district health management team communicates well with health workers in this health centre	1	2	3	4
5.21.	I like how this district health management team treats its employees	1	2	3	4
5.22.	We are well informed about guideline changes affecting our work	1	2	3	4
5.23.	I feel that at work things are going the way I would like them to	1	2	3	4

5.24. Please provide any further comments relating to your feelings about work or the questions in this section:

SSQ: HEALTH WORKERS (6)		
**Health centre code**	**Health Worker ID**	**Date**
[____|____]	[____|____|____|____]	[____|____] / [____|____] / [____|____]<br>day month year

PART 2 (HFI HEALTH WORKERS ONLY)

6. IMPACT OF PRIME TRAINING

Please circle to indicate (a) workshops attended and (b) how useful the skills taught in the workshop have been to you personally	(a) Attended the workshop	(b) Frequency I used skills learned in the workshops in my everyday work			
		Regularly	Infrequently	Never	Don't know
6.1. HCM 01 Budgeting & accounting for the PHC Fund using the PHC Fund Management Tool	Yes / No/ N/A	1	2	3	4
6.2. HCM 02 (a) Managing drug stocks using the Drug Stock Card and Requisition & Issue Voucher	Yes / No /N/A	1	2	3	4
6.3. HCM 02 (b) Managing distribution of drugs to your health centre using the ADDAT	Yes / No/ N/A	1	2	3	4
6.4. HCM 03 Using patient information for clinical and health centre management decisions	Yes / No/ N/A	1	2	3	4
6.5. PCS 00 Building self-awareness through self-observation activities	Yes / No	1	2	3	4
6.6. PCS 01 (a) Building rapport with patients	Yes / No	1	2	3	4
6.7. PCS 01 (b) Active listening	Yes / No	1	2	3	4
6.8. PCS 02 (a) Giving information to patients	Yes / No	1	2	3	4
6.9. PCS 02 (b) Managing RDT negative results	Yes / No	1	2	3	4
6.10. PCS 03 (a) Creating a positive work environment	Yes / No	1	2	3	4
6.11. PCS 03 (b) Motivation towards your job	Yes / No	1	2	3	4
6.12. PCS 04 / 05 Welcoming and orienting patients	Yes / No	1	2	3	4
6.13. RDT training (May 2011)	Yes / No	1	2	3	4
6.14. Supervision by the RDT trainers at the health centre (May 2011)	Yes / No	1	2	3	4
6.15. Supervision by the RDT trainers at the health centre (January 2012)	Yes / No	1	2	3	4

6.16. Please provide any further comments relating to the usefulness of the workshops:

SSQ: HEALTH WORKERS (7)		
**Health centre code**	**Health Worker ID**	**Date**
[____|____]	[____|____|____|____]	[____|____] / [____|____] / [____|____]<br>day month year

7. Please use this space to provide any further information or your opinions about the quality of services provided at your health centre, what has improved your services, and what else you think could improve the quality of services offered to your local population.

THANK YOU FOR YOUR PARTICIPATION

Now- please replace this form in the envelope and return it to a member of the research team.

APPENDIX V: SEMI-STRUCTURED QUESTIONNAIRE
PRIVATE DRUG SHOP WORKERS

Drug shop code	Provider ID	Date
[____|____]	[____|____|____|____]	[____|____] / [____|____] / [____|____]<br>day month year

DEMOGRAPHIC INFORMATION

**1. Age** Years [____|____]	**5. Highest level of education or qualification achieved**<br>0 = None<br>1 = Primary (P1 — P7)<br>2 = Secondary (S1 — S6)<br>3 = Certificate<br>77 = Other______________________<br>4 = Diploma<br>5 = Bachelor's degree<br>88 = Don't know<br>99 = Refused to answer<br>[____|____]
**2. Gender** 1 = Male 2 = Female [____]	
**3. 'Are you originally from this area?'** 1 = Yes 2 = No [____]	
**4. 'How long have you worked at this drug shop?'** [____|____]years [____|____]months	**6. What year did you graduate from your course?** [____|____|____|____]

SECTION 1: CHANGES AT WORK

1. "Have you noticed any changes in the services provided to patients at this drug shop over the past few months?" 1 = Yes 2 = No 88 = Don't know 99 = Refused to answer [_____|_____]

If yes, go to Qn 2. Otherwise, skip to the next section

2. What do you think was the most significant or important change in the way patients are managed at this drug shop?

3. Why is this significant or important to you?

4. What difference has this made in how patients are managed at this drug shop?

SSQ: PRIVATE DRUG SHOPS (2)

Drug shop code	Provider ID	Date
[____|____]	[____|____|____|____]	[____|____] / [____|____] / [____|____]<br>day month year

SECTION 2: SUPPLY MANAGEMENT

**1. 'Does this drug shop typically stock artemether-lumefantrine?'**<br>*If yes, go to Qn 2, otherwise skip to Qn 5*	1 = Yes 88 = Don't know<br>2 = No 99 = Refused to answer	[_____|_____]
**2. 'Is artemether-lumefantrine available today?'**	1 = Yes 88 = Don't know<br>2 = No 99 = Refused to answer	[_____|_____]
**3. 'Have there been stock-outs of artemether-lumefantrine in the last 6 months?'**	1 = Yes 88 = Don't know<br>2 = No 99 = Refused to answer	[_____|_____]
**4. 'Does this drug shop stock 'Green Leaf' artemether-lumefantrine supported by AMFm?'**	1 = Yes 88 = Don't know<br>2 = No 99 = Refused to answer	[_____|_____]

ARTEMETHER-LUMEFANTRINE BRANDS AVAILABLE

	Stock	Available today	Cost per unit	Unit
	1=Always<br>2=Sometimes<br>3=Never	1=Yes<br>2=No	Ugandan shillings	1=Package<br>2=Tablet
5a. Coartem (Novartis, Switzerland)				
6 pack	[____]	[____]	[____|____],[____|____|____]	[____]
12 pack	[____]	[____]	[____|____],[____|____|____]	[____]
18 pack	[____]	[____]	[____|____],[____|____|____]	[____]
24 pack	[____]	[____]	[____|____],[____|____|____]	[____]
5b. Lumartem (Cipla, India)	[____]	[____]	[____|____],[____|____|____]	[____]
5c. Lomart (Agog, India)	[____]	[____]	[____|____],[____|____|____]	[____]
5d. Artefan (Ajanta Pharma, India)	[____]	[____]	[____|____],[____|____|____]	[____]
5e. Lumiter (Macleods, India)	[____]	[____]	[____|____],[____|____|____]	[____]
5f. Lonart (Milan Labs, India)	[____]	[____]	[____|____],[____|____|____]	[____]
5g. Green leaf (AMFm) - CHILD	[____]	[____]	[____|____],[____|____|____]	[____]
5h. Green leaf (AMFm) - ADULT	[____]	[____]	[____|____],[____|____|____]	[____]
5i. Other	[____]	[____]	[____|____],[____|____|____]	[____]
5j. Other	[____]	[____]	[____|____],[____|____|____]	[____]

OTHER ARTEMISININ-BASED COMBINATION THERAPIES

**6. 'Does this drug shop typically stock other artemisinin-based drugs (ACTs)?'**	1 = Yes 88 = Don't know<br>2 = No 99 = Refused to answer	[_____|_____]

If YES, list other ACTs typically stocked at this drug shop, including: artesunate + amodiaquine, dihydroartemisinin + piperaquine, artemisinin + napthoquine (ARCO), artesunate + mefloquine

Brand Name	Manufacturer	Stock<br>1=Always<br>2=Sometimes<br>3=Never	Available today<br>1=Yes<br>2=No
7a.		[____]	[____]
7b.		[____]	[____]
7c.		[____]	[____]

SSQ: PRIVATE DRUG SHOPS (3)		
**Drug shop code**	**Provider ID**	**Date**
[____|____]	[____|____|____|____]	[____|____] / [____|____] / [____|____]<br>day month year

SECTION 2 cont: SUPPLY MANAGEMENT

OTHER ANTIMALARIAL DRUGS

7. 'Does this drug shop typically stock other malaria drugs?' 1 = Yes 2 = No 88 = Don't know 99 = Refused to answer [_____|_____]

If YES, review the list below and complete the information on stocking	**Stock**<br>1=Always<br>2=Sometimes<br>3=Never	**Available today**<br>1=Yes<br>2=No
8a. Chloroquine	[____]	[____]
8b. Amodiaquine	[____]	[____]
8c. Sulfadoxine-pyrimethamine	[____]	[____]
8d. Quinine oral	[____]	[____]
8e. Quinine injectable	[____]	[____]
8f. Artesunate injectable	[____]	[____]
8g. Primaquine	[____]	[____]
8h. Mefloquine	[____]	[____]
8i. Other	[____]	[____]

9. Please provide any additional comments about supply of drugs at this drug shop.

RAPID DIAGNOSTIC TESTS FOR MALARIA

10. 'Does this drug shop typically stock rapid diagnostic tests (RDTs) for malaria?' 1 = Yes 2 = No 88 = Don't know 99 = Refused to answer [_____|_____]

If YES, review the list below and complete the information on stocking	**Stock**<br>1=Always<br>2=Sometimes<br>3=Never	**Available today**<br>1=Yes<br>2=No
10a. Brand (specify)____	[____]	[____]
10b. Brand (specify)____	[____]	[____]
10c. Brand (specify)____	[____]	[____]
10d. Brand (specify)____	[____]	[____]
10e. Brand (specify)____	[____]	[____]

11. Please provide any additional comments about supply of RDTs at this drug shop.

SSQ: PRIVATE DRUG SHOPS (4)

Drug shop code	Provider ID	Date
[____|____]	[____|____|____|____]	[____|____] / [____|____] / [____|____]<br>day month year

SECTION 3: PATIENT LOAD

1. "On average, how many people visit this drug shop each week?"

Total number of customers per week [_____|_____]

2. "Do you think that there has been a change in the number of people who visit your drug shop each week in the last year?"

1 = Yes 88 = Don't know
2 = No 99 = Refused to answer

If YES, go to Qn 3. Otherwise skip to next section. [_____|_____]

3. "If so, what kind of changes have you noticed?"

Record all answers given

1 = More customers come to the drug shop
2 = Fewer customers come to the drug shop
3 = More patients with malaria come to the drug shop
4 = Fewer patients with malaria come to the drug shop
5 = Customers ask for different drugs *(specify)*
____
77 = Other ______________________________________
88 = Don't know
99 = Refused to answer

[_____|_____]
[_____|_____]
[_____|_____]

4. "What do you think is the reason for this change?"

5. Please provide any additional comments about patient load at this drug shop.

SECTION 4: PATIENT MANAGEMENT

1. "What treatments do you most commonly recommend for children under five years of age with fever?"

Record all answers given

1 = Artemether-lumefantrine (Coartem/Lumartem)
2 = Chloroquine + sulfadoxine-pyrimethamine (Homapak)
3 = Quinine
4 = Paracetamol (Panadol)
77 = Other ______________________________________
88 = Don't know
99 = Refused to answer

[_____|_____]
[_____|_____]
[_____|_____]
[_____|_____]

2. "What treatments do you most commonly recommend for adults with fever?"

Record all answers given

1 = Artemether-lumefantrine (Coartem/Lumartem)
2 = Chloroquine + sulfadoxine-pyrimethamine (Homapak)
3 = Quinine
4 = Paracetamol (Panadol)
77 = Other ______________________________________
88 = Don't know
99 = Refused to answer

[_____|_____]
[_____|_____]
[_____|_____]
[_____|_____]

SSQ: PRIVATE DRUG SHOPS (5)

Drug shop code	Provider ID	Date
[____|____]	[____|____|____|____]	[____|____] / [____|____] / [____|____]<br>day month year

SECTION 4 cont: PATIENT MANAGEMENT

**3. "What treatments do you most commonly recommend for a patient with uncomplicated (simple) malaria?"**<br><br>*Record all answers given*	1 = Artemether-lumefantrine (Coartem/Lumartem)<br>2 = Artesunate + amodiaquine<br>3 = Dihydroartemisinin-piperaquine (Duocotexcin)<br>4 = Any artemisinin-based combination therapy<br>5 = Quinine<br>6 = Chloroquine + sulfadoxine-pyrimethamine (Homapak)<br>77 = Other ____<br>88 = Don't know<br>99 = Refused to answer	[_____|_____]<br><br>[_____|_____]<br><br>[_____|_____]<br><br>[_____|_____]

4. Please provide any additional comments about patient management at this drug shop.

SECTION 5: ADDITIONAL COMMENTS

Please use this space to provide any other comments

INTERVIEWER'S COMMENTS

Please use this space to provide any observations or comments on the drug shop or the drug shop vendor

[____|____|____|____|____|____|____|____]

FGD Study ID

APPENDIX W. FOCUS GROUP DISCUSSIONS
Informed consent form for caregivers and heads of households

**Protocol Title:**	ACT PROCESS: Evaluating the process, context, and impact of interventions to enhance health facilities in Tororo, Uganda
**Site of Research:**	Tororo, Uganda
**Principal Investigators**:	Dr. Sarah Staedke
**Date:**	20 February 2012

Introduction

Dr. Sarah Staedke and colleagues from the Uganda Malaria Surveillance Project/Infectious Diseases Research Collaboration are doing a study to evaluate activities that have been introduced at government-run health facilities in this area aiming to improve the health of children. We would like to understand the impact of these activities and why they are working, or not. This study involves several parts, including assessing the communication between health workers and their patients, holding interviews, and conducting group discussions with community members and health workers.

Why is this study being done?

For this part of the study, we would like to learn more about whether the activities that have been introduced at government-run health facilities have had an impact from the perspective of members of the community and health workers. This information will help us understand how and why the health facility activities have affected the health of children in this area. You are being asked to take part in a group discussion for this study because of your experiences with caring for and treating ill children.

What will happen today if I take part in this study?

If you agree, you will take part in a discussion about treating ill children and your experiences with health centers. We are interested to hear about your experiences and opinions; there are no right or wrong answers. We will take notes of the ideas discussed and a recording will be made of this discussion using a digital voice recorder. Afterwards, we will enter information from the discussion into a computer for analysis. The information we collect will be used by project investigators, and may be shared with other researchers and policy-makers to answer questions about how best to deliver training and health services.

What is the location of the study?

The discussion will take place in an agreed location within your area.

How long will the study last?

Participation in the study will involve a one-time discussion lasting about one to three hours.

Can I stop being in the study?

You can decide to stop participating at any time. Just tell the project researcher right away if you wish to stop.

What risks can I expect from being in the study?

Participation in any research study may involve a loss of privacy. Your name will not be used in any reports of the information provided. No quotes or other results arising from your participation in this study will be included in any reports, even anonymously, without your agreement. The information obtained from these interviews will be locked at our project offices. We will do our best to make sure that the personal information gathered for this study is kept private.

Are there benefits to taking part in the study?

There will be no direct benefit to you from participating in this study. However, the information that you provide will help researchers and policy-makers understand how best to improve health services in this area.

What other choices do I have if I do not take part in this study?

You are free to choose not to participate in the study. If you decide not to take part in this study, there will be no penalty to you.

What are the costs of taking part in this study? Will I be paid for taking part in this study?

There are no costs to you for taking part in this study. You will not be paid for taking part in this study, but you will be given 5,000/= Ush to refund the cost of your transport.

What are my rights if I take part in this study?

Taking part in this study is your choice. You may choose either to take part or not to take part in the study. If you decide to take part in this study, you may change your mind at any time. No matter what decision you take, there will be no penalty to you in any way.

Who can answer my questions about the study?

You can talk to the researchers about any questions or concerns you have about these study activities. Contact Dr. Sarah Staedke or other members of the Uganda Malaria Surveillance Project / Infectious Disease Research Collaboration on telephone number 0414-530692. If you have any questions, comments or concerns about taking part in these activities, first talk to the researchers. If for any reason you do not wish

THE REPUBLIC OF UGANDA

to do this, or you still have concerns about doing so, you may contact Dr. James Tumwine, Makerere University Faculty of Medicine Research and Ethical Committee at telephone number 0414-530020.

WHAT YOUR SIGNATURE OR THUMBPRINT MEANS

Your signature or thumbprint below means that you understand the information given to you in this consent form about your participation in the study and agree with the following statements:

1. "I have read the consent form concerning this study (or have understood the verbal explanation of the consent form) and I understand what will be required of me and what will happen to me if I take part in it."

2. "My questions concerning this study have been answered by Dr. Staedke or the person who signed below."

3. "I understand that at any time, I may withdraw from this study without giving a reason."

4. "I agree to take part in this study."

If you wish to participate in this study, you should sign or place your thumbprint on the following page. If you do not agree to quotes or other results arising from your participation in the study being included, even anonymously, in any reports about the study, please tell the researcher now.

WE WILL GIVE YOU A COPY OF THIS SIGNED AND DATED CONSENT FORM

Name of Participant (printed)

Signature or Fingerprint of Participant — Date/Time

Name of Investigator Administering Consent (printed) — Position/Title

Signature of Investigator Administering Consent — Date/Time

If the participant is unable to read and/or write, an impartial witness should be present during the entire informed consent discussion.

After the written informed consent form is read and explained to the participant, and after they have orally consented to participate in the study, the witness should print the name of the participant above, and then the participant should provide their fingerprint.

Then the witness should print their name, provide their signature, and date the consent form below.

By signing the consent form, the witness attests that the information in the consent form and any other written information was accurately explained to, and apparently understood by the participant, and that informed consent was freely given by the participant.

Name of Person Witnessing Consent (printed)

Signature of Person Witnessing Consent — Date/Time

APPENDIX X: FGD DATA COLLECTION TOOL
Primary caregivers

PART 1: PARTICIPANT DETAILS

Record the demographic details for each participant using the primary caregiver FGD participant log as appropriate.

PART 2: FGD INTRODUCTION

**Subcounty ID**		[_____]	**Moderator initials**		[_____|_____|_____]
**FGD ID number**		[_____|_____|_____]	**Note-taker initials**		[_____|_____|_____]
**Age of participants**	1 = < 30 years<br>2 = $\geq$ 30 years	[_____|_____]	**Gender of participants**	1 = Male<br>2 = Female	[_____|_____]
**HFI or Standard care or Area outside of a 2km radius of an ACT PRIME health center**	1 = HFI<br>2 = Standard care<br>3= Area outside of a 2km radius of an ACT PRIME health center	[_____|_____]	**Health center in parish**	1 = Yes<br>2 = No	[_____|_____]
**Date:**	[____|____] / [____|____] / [____|____]<br>day month year		**Time start**<br>**Time end**		[_____|_____] : [_____|_____]<br>[_____|_____] : [_____|_____]

Introduction

I am ______________________________ from _____________________ (moderator)
I am ______________________________ from _____________________ (note-taker)

- *Hello. My name is.... and I work for UMSP (Uganda Malaria Surveillance Project)/ IDRC (Infectious Diseases Research Collaboration).*
- *Thank you very much for coming today. I am part of a team who are doing research to learn more about how people in Tororo treat illnesses in children and their experiences with community medicine distributors and health centers. We would like to understand more about the situation of people in communities like yours in terms of options for seeking treatment for children when they are sick. In addition to our discussion with you today, we are talking with other mothers, heads of households, and health care workers in this district.*
- *Today we would like you to take part in a discussion about treating sick children and your experiences with different places you seek treatment. We have invited you to participate because you have experience with this here and we hope you will tell us the real situation from your point of view.*
- *The discussion will last for about one hour. We will take notes of the ideas discussed and, if you agree, a tape recording will be made of the discussion in order that we record what you say accurately. When the tape is on, the light will be red. If you wish to say anything 'off the record' this is fine, please indicate to the moderator or note taker. The audiotape will only be used by the study team: no one else will hear your voice. It will be kept for 2 years for our records and will then be erased or destroyed. We are not writing down your names here and no one will be able to identify you in any reports arising out of this research. All records of this discussion will be kept securely.*
- *Does anyone have any questions?*
- *Let us begin by setting some ground rules.*
  - ✓ Ground rules set by group, e.g.
    - Only one person talks at a time.
    - Speak clearly
    - It is important for us to hear everyone's ideas and opinions. There is no right or wrong answers to questions – just ideas, experiences and opinions, which are all valuable.
    - It is important for us to hear all sides of an issue – the positive and the negative.
    - Confidentiality is assured. "What is shared in the room stays in the room."
    - TURN OFF MOBILE PHONES
  - ✓ Consent
  - ✓ Ask group to introduce themselves using first names and their role and health centre
  - ✓ Demographic details – please only use each others' first name for discussion

PART 3: FGD TOPIC GUIDES (1)

Domains, topic questions, and probes: Use the table below to help you administer the questions during the interview.

'Now I am going to introduce some topics one at a time about your experiences when your children are unwell, and I hope you can discuss them together.'

Domain	Topic and Probes
**1. Common illnesses in children < 15 years**	a) What illnesses have been common in children below 5 years here for the last one year? *(Make a list, don't spend too long on this question)*
	b) What illnesses have been common in children aged between 5 and 15 years here in the last few months?
**2. Sources of treatment and provider roles**	a) In your experience, what sources have been most successful at treating these different illnesses? *(Start with malaria. Probe for different medicine, provider, and treatment types)*
	b) What is it about each of these different sources of treatment that is important to you? (*e.g. cost, expertise, interpersonal skills, etc. Probe for each source listed and for why different things are important for different illnesses)*
**3. Significant events**	a) Looking back over the past year, what do you think was the most significant change in the way you managed illness in your household?
	b) Why is this significant to you?
	c) What difference has this made now or will it make in the future?

PART 3: FGD TOPIC GUIDES (2)	
**Domains, topic questions, and probes:** Use the table below to help you administer the questions during the interview.	
**Domain**	**Topic and Probes**
**4. Use of health centres**	a) When do you feel it is necessary to take a child to the health centre? *(Probe for specific illnesses and stages of illness, probe for examples)*
	b) If you feel that it is necessary to go, do you always go? If not, what are the reasons that you don't go to the health centre? *(Probe for examples and stories)*
**5. Experience with health centres**	a) How many of you have been to the government health centre nearest to your home? Can you tell me about your experience when you have gone there with a sick child?<br><br>(*Probe: What did you go there for, and what happened? Probe for issues regarding payment particularly for RDTs. Ask for the names of HCs mentioned.* Probe for issues regarding drugs and testing (were drugs available, was the child tested and did the drugs that were given work, did you have to pay for drugs or tests?)<br><br>*Probe for issues regarding relationship with health worker (how were you received, how did the HW speak with you, were you able to respond?)*<br><br>*Probe for issues regarding information (how did the HW give you information about the sickness?)*

PART 3: FGD TOPIC GUIDES (3)	
**Domains, topic questions, and probes:** Use the table below to help you administer the questions during the interview.	
**Domain**	**Topic and Probes**
	b) Based on this experience, would you go to that health centre again next time your child was sick with these symptoms? If yes, why? If not, where would you go, and why?
	c) Can you tell me about any really good experiences you have had at that health centre? *Probe: What was it about that experience that made you feel satisfied?*
**6. Change at Health centres**	a) What has been the most significant change at your nearest health centre? *Probe: what do you think brought the change? Repeat for all changes noted by participants. Ask for the names of HCs mentioned*
	b) What do these changes mean for you when you have a sick child? *Repeat for all changes noted by participants.*
	c) What improvements would you like to see at your nearest health centre?
**7. Context**	a) Can you describe any changes at other places you may have gone to for treatment (private clinic, traditional healer, place of worship) that has impacted on where you take a sick child?
**8. Closing**	We are now approaching the end of our discussion. Is there anything else anyone would like to add about the kind of diagnosis and treatment you get from health centres that we have not talked about?
✓ **Summarize main points made by the participants;**<br>✓ **Thank participants**	

PART 4: NOTE-TAKER FORM

**FGD ID number** [_____|_____|_____]	**Moderator initials** [_____|_____|_____]
**Sub-county code** [_____]	**Note-taker initials** [_____|_____|_____]
**FGD type** 1 = Primary caregiver 2 = Heads of household [_____|_____]	**Gender of participants** 1 = Male 2 = Female [_____|_____]
**Age of participants** 1 = < 30 years 2 = ≥ 30 years [_____|_____]	**Health center in parish** 1 = Yes 2 = No [_____|_____]
**Date:** [____|____] / [____|____] / [____|____] day month year	**Time start** [_____|_____] : [_____|_____] **Time end** [_____|_____] : [_____|_____]

1. Meeting place description: detail and description, e.g. location, size and accessibility, and how this could affect the discussion; interruptions during the discussion

2. Participant seating diagram:

3. Group dynamics: general description – level of participation, dominant and passive participants, interest level, boredom, anxiety – and how these relate to the different topics discussed

4. Impressions and observations:

5. Notes of comments provided AFTER the discussion is over (Include additional sheets if necessary):

PART 5: CONTACT SUMMARY FORM (1)			
**FGD ID number**	[_____|_____|_____]	**Moderator initials**	[_____|_____|_____]
**Sub-county code**	[_____]	**Note-taker initials**	[_____|_____|_____]
**FGD type** 1 = Primary caregiver 2 = Heads of household	[_____|_____]	**Gender of participants** 1 = Male 2 = Female	[_____|_____]
**Age of participants** 1 = < 30 years 2 = ≥ 30 years	[_____|_____]	**Health center in parish** 1 = Yes 2 = No	[_____|_____]
**Date:**	[____|____] / [____|____] / [____|____] day month year	**Time start** **Time end**	[_____|_____] : [_____|_____] [_____|_____] : [_____|_____]

1. What were the main issues or points made by participants during this focus group?

PART 5: CONTACT SUMMARY FORM (2)

2. What new information did you gain through this focus group compared to previous focus groups in this study?

3. Was there anything surprising to you personally? Or that made you think differently about this research question?

4. What messages did you take from this interview for intervention design?

PART 5: CONTACT SUMMARY FORM (3)

5. How would you describe the general atmosphere and engagement of the focus group?

6. How would you describe the group dynamics? For example, were there dominant individuals (what was the result and what were their IDNOs)? Did all participants contribute? Did you feel there was pressure to adhere to dominant viewpoints (what topics)?

7. What else was important about this focus group?

8. Were there any problems with the topic guide (e.g. wording, order of topics, missing topics) you experienced in this focus group?

APPENDIX Y: FGD DATA COLLECTION TOOL
Heads of households

PART 1: PARTICIPANT DETAILS

Record the demographic details for each participant using the heads of households FGD participant log as appropriate.

PART 2: FGD INTRODUCTION

**Subcounty ID** [_____]		**Moderator initials** [_____|_____|_____]	
**FGD ID number** [_____|_____|_____]		**Note-taker initials** [_____|_____|_____]	
**Age of participants**	1 = < 30 years<br>2 = $\geq$ 30 years [_____|_____]	**Gender of participants**	1 = Male<br>2 = Female [_____|_____]
**HFI or Standard care or Area outside of a 2km radius of an ACT PRIME health center**	1 = HFI<br>2 = Standard care<br>3= Area outside of a 2km radius of an ACT PRIME health center [_____|_____]	**Health center in parish**	1 = Yes<br>2 = No [_____|_____]
**Date:** [____|____] / [____|____] / [____|____]<br>day month year		**Time start**<br>**Time end**	[_____|_____] : [_____|_____]<br>[_____|_____] : [_____|_____]

Introduction

I am ______________________________ from ______________________ (moderator)
I am ______________________________ from ______________________ (note-taker)

- *Hello. My name is.... and I work for UMSP (Uganda Malaria Surveillance Project)/ IDRC (Infectious Diseases Research Collaboration).*
- *Thank you very much for coming today. I am part of a team who are doing research to learn more about how people in Tororo treat illnesses in children and their experiences with community medicine distributors and health centers. We would like to understand more about the situation of people in communities like yours in terms of options for seeking treatment for children when they are sick. In addition to our discussion with you today, we are talking with other mothers, heads of households, and health care workers in this district.*
- *Today we would like you to take part in a discussion about treating sick children and your experiences with different places you seek treatment. We have invited you to participate because you have experience with this here and we hope you will tell us the real situation from your point of view.*
- *The discussion will last for about one hour. We will take notes of the ideas discussed and, if you agree, a tape recording will be made of the discussion in order that we record what you say accurately. When the tape is on, the light will be red. If you wish to say anything 'off the record' this is fine, please indicate to the moderator or note taker. The audiotape will only be used by the study team: no one else will hear your voice. It will be kept for 2 years for our records and will then be erased or destroyed. We are not writing down your names here and no one will be able to identify you in any reports arising out of this research. All records of this discussion will be kept securely.*
- *Does anyone have any questions?*
- *Let us begin by setting some ground rules.*
  - ✓ Ground rules set by group, e.g.
    - Only one person talks at a time.
    - Speak clearly
    - It is important for us to hear everyone's ideas and opinions. There is no right or wrong answers to questions – just ideas, experiences and opinions, which are all valuable.
    - It is important for us to hear all sides of an issue – the positive and the negative.
    - Confidentiality is assured. "What is shared in the room stays in the room."
    - TURN OFF MOBILE PHONES
  - ✓ Consent
  - ✓ Ask group to introduce themselves using first names and their role and health centre
  - ✓ Demographic details – please only use each others' first name for discussion

PART 3: FGD TOPIC GUIDES (1)

Domains, topic questions, and probes: Use the table below to help you administer the questions during the interview.

'Now I am going to introduce some topics one at a time about your experiences when your children are unwell, and I hope you can discuss them together.'

Domain	Topic and Probes
**1. Common illnesses in children < 15 years**	a) What illnesses have been common in children below 5 years here for the last one year? *(Make a list, don't spend too long on this question)*
	b) What illnesses have been common in children aged between 5 and 15 years here in the last few months?
**2. Sources of treatment and provider roles**	a) In your experience, what sources have been most successful at treating these different illnesses? *(Start with malaria. Probe for different medicine, provider, and treatment types)*
	b) What is it about each of these different sources of treatment that is important to you? (*e.g. cost, expertise, interpersonal skills, etc. Probe for each source listed and for why different things are important for different illnesses)*
**3. Significant events**	a) Looking back over the past year, what do you think was the most significant change in the way you managed illness in your household?
	b) Why is this significant to you?
	c) What difference has this made now or will it make in the future?

PART 3: FGD TOPIC GUIDES (2)	
**Domains, topic questions, and probes:** Use the table below to help you administer the questions during the interview.	
**Domain**	**Topic and Probes**
**4. Use of health centres**	a) When do you feel it is necessary to take a child to the health centre? *(Probe for specific illnesses and stages of illness, probe for examples)*
	b) If you feel that it is necessary to go, do you always go? If not, what are the reasons that you don't go to the health centre? *(Probe for examples and stories)*
**5. Experience with health centres**	a) How many of you have been to the government health centre nearest to your home? Can you tell me about your experience when you have gone there with a sick child?<br><br>(*Probe: What did you go there for, and what happened? Probe for issues regarding payment particularly for RDTs. Ask for the names of HCs mentioned.* Probe for issues regarding drugs and testing (were drugs available, was the child tested and did the drugs that were given work, did you have to pay for drugs or tests?)<br><br>*Probe for issues regarding relationship with health worker (how were you received, how did the HW speak with you, were you able to respond?)*<br><br>*Probe for issues regarding information (how did the HW give you information about the sickness?)*

PART 3: FGD TOPIC GUIDES (3)	
**Domains, topic questions, and probes:** Use the table below to help you administer the questions during the interview.	
**Domain**	**Topic and Probes**
	b) Based on this experience, would you go to that health centre again next time your child was sick with these symptoms? If yes, why? If not, where would you go, and why?
	c) Can you tell me about any really good experiences you have had at that health centre? *Probe: What was it about that experience that made you feel satisfied?*
**6. Change at Health centres**	a) What has been the most significant change at your nearest health centre? *Probe: what do you think brought the change? Repeat for all changes noted by participants. Ask for the names of HCs mentioned*
	b) What do these changes mean for you when you have a sick child? *Repeat for all changes noted by participants.*
	c) What improvements would you like to see at your nearest health centre?
**7. Context**	a) Can you describe any changes at other places you may have gone to for treatment (private clinic, traditional healer, place of worship) that has impacted on where you take a sick child?
**8. Closing**	We are now approaching the end of our discussion. Is there anything else anyone would like to add about the kind of diagnosis and treatment you get from health centres that we have not talked about?
✓ **Summarize main points made by the participants;**<br>✓ **Thank participants**	

PART 4: NOTE-TAKER FORM			
**FGD ID number**	[_____|_____|_____]	**Moderator initials**	[_____|_____|_____]
**Sub-county code**	[_____]	**Note-taker initials**	[_____|_____|_____]
**FGD type** 1 = Primary caregiver 2 = Heads of household	[_____|_____]	**Gender of participants** 1 = Male 2 = Female	[_____|_____]
**Age of participants** 1 = < 30 years 2 = > 30 years	[_____|_____]	**Health center in parish** 1 = Yes 2 = No	[_____|_____]
**Date:**	[____|____] / [____|____] / [____|____] day month year	**Time start**	[_____|_____] : [_____|_____]
		**Time end**	[_____|_____] : [_____|_____]

1. Meeting place description: detail and description, e.g. location, size and accessibility, and how this could affect the discussion; interruptions during the discussion

2. Participant seating diagram:

3. Group dynamics: general description – level of participation, dominant and passive participants, interest level, boredom, anxiety – and how these relate to the different topics discussed

4. Impressions and observations:

5. Notes of comments provided AFTER the discussion is over (Include additional sheets if necessary):

PART 5: CONTACT SUMMARY FORM (1)

**FGD ID number**	[_____|_____|_____]	**Moderator initials**	[_____|_____|_____]
**Sub-county code**	[_____]	**Note-taker initials**	[_____|_____|_____]
**FGD type** 1 = Primary caregiver<br>2 = Heads of household	[_____|_____]	**Gender of participants** 1 = Male<br>2 = Female	[_____|_____]
**Age of participants** 1 = < 30 years<br>2 = ≥ 30 years	[_____|_____]	**Health center in parish** 1 = Yes<br>2 = No	[_____|_____]
**Date:**	[____|____] / [____|____] / [____|____]<br>day month year	**Time start**<br>**Time end**	[_____|_____] : [_____|_____]<br>[_____|_____] : [_____|_____]

1. What were the main issues or points made by participants during this focus group?

PART 5: CONTACT SUMMARY FORM (2)

2. What new information did you gain through this focus group compared to previous focus groups in this study?

3. Was there anything surprising to you personally? Or that made you think differently about this research question?

4. What messages did you take from this interview for intervention design?

PART 5: CONTACT SUMMARY FORM (3)

PART 5: CONTACT SUMMARY FORM (3)
**5. How would you describe the general atmosphere and engagement of the focus group?**
**6. How would you describe the group dynamics? For example, were there dominant individuals (what was the result and what were their IDNOs)? Did all participants contribute? Did you feel there was pressure to adhere to dominant viewpoints (what topics)?**
**7. What else was important about this focus group?**
**8. Were there any problems with the topic guide (e.g. wording, order of topics, missing topics) you experienced in this focus group?**

APPENDIX Z: STRUCTURED CONTEXTUAL RECORD (Form 1, Part A)

DISTRICT LEVEL: *To be administered to the DHO or district representative*	Staff ID [____|____]	Date completed [____|____] / [____|____] / [____|____] day month year

TIME PERIOD COVERED:	1 = Baseline	4 = 7-9 months	7 = 16-18 months	
	2 = 0-3 months	5 = 10-12 months	8 = 19-21 months	
	3 = 4-6 months	6 = 13-15 months	9 = 21-24 months	[____|____]

SECTION 1: RESPONDENT INFORMATION

**1. Respondent name**	**2. Contact information** *(cell phone or email address)*
**3. Respondent position**	1 = DHO 77 = Other *(list)* 2 = DHI ______________________________ [_____|_____]

SECTION 2: INTERVENTIONS

(1) BEDNETS

**1. Have bed nets been distributed in the study area in the last 3 months?** *If YES, go to Qn 2 (and complete Part B #1), otherwise skip to Qn 5.*	1 = Yes 88 = Don't know 2 = No	[_____|_____]
**2. Who was responsible for the distribution?**		
**3. Where were the nets distributed?**		
**4. Who do you suggest that we talk to for more information about the bed net distribution?**		

(2) INDOOR RESIDUAL SPRAYING (IRS)

**5. Has IRS been conducted in the study area in the last 3 months?** *If YES, go to Qn 6 (and complete Part B #2), otherwise skip to Qn 9.*	1 = Yes 88 = Don't know 2 = No	[_____|_____]
**6. Who conducted the IRS campaign?**		
**7. What areas were sprayed?**		
**8. Who do you suggest that we talk to for more information about IRS?**		

(3) ARTEMISININ COMBINATION THERAPY (ACTs)

**9. Have any programs to distribute ACTs been conducted in the study area in the last 3 months?** We are particularly interested in ACT distribution outside of the existing NMS supply to the public health centers, including the ACT study. *If YES, go to Qn 10 (and complete Part B #3), otherwise skip to Qn 13.*	1 = Yes 2 = No 88 = Don't know	[_____|_____]
**10. Who distributed the ACTs?**		
**11. Where were the ACTs distributed?**		
**12. Who do you suggest that we talk to for more information about ACT distribution?**		

(4) RAPID DIAGNOSTIC TESTS (RDTs)

**13. Have RDTs been distributed in the study area in the last 3 months?** We are particularly interested distribution of RDTs outside of the NMS supply to the public health centers, including the ACT Study. *If YES, go to Qn 14 (and complete Part B #4), otherwise skip to Qn 17.*	1 = Yes 2 = No 88 = Don't know	[_____|_____]
**14. Who distributed the RDTs?**		

STRUCTURED CONTEXTUAL RECORD (Form 1, Part A, Page 2)

Staff ID	Date completed
[____|____]	[____|____] / [____|____] / [____|____]<br>day month year

RAPID DIAGNOSTIC TESTS (RDTS) – CONTINUED		
**15. Where were the RDTs distributed?**		
**16. Who do you suggest that we talk to for more information about RDT distribution?**		
**(5) SCHOOL-BASED INTERVENTIONS**		
**17. Have any interventions targeting schools and/or school-aged children been conducted in the study area in the last 3 months?** Probe for programs involving health education, vaccination, de-worming, malaria, nutrition, sanitation, etc.<br>*If YES, go to Qn 18 (and complete Part B #5), otherwise skip to Qn 21.*	1 = Yes<br>2 = No<br>88 = Don't know	[_____|_____]
**18. What was the program(s) and who conducted it?**		
**19. What schools were involved?**		
**20. Who do you suggest that we talk to for more information about school-based interventions?**		
**(6) COMMUNITY-BASED INTERVENTIONS**		
**21. Have any interventions targeting the community been conducted in the study area in the last 3 months?** Probe for programs involving VHTs, ICCM, HBMF, CHWs, and/or those that target vaccination, de-worming, malaria, nutrition, sanitation, etc.<br>*If YES, go to Qn 22 (and complete Part B #6), otherwise skip to Qn 23.*	1 = Yes<br>2 = No<br>88 = Don't know	[_____|_____]
**22. What was the program(s) and who conducted it?**		
**23. Where were the program(s) conducted?**		
**24. Who do you suggest that we talk to for more information about community-based interventions?**		
**(7) HEALTH IEC CAMPAIGNS**		
**25. Have any IEC (information, education, and communication) campaigns been conducted in the study area in the last 3 months?** Probe for campaigns that target malaria treatment, prevention, or diagnostics (RDTs), and other health-related issues such as vaccination, de-worming, nutrition, sanitation, etc.<br>*If YES, go to Qn 26(and complete Part B #7), otherwise skip to Qn 29.*	1 = Yes<br>2 = No<br>88 = Don't know	[_____|_____]
**26. What was the campaign(s) and who conducted it?**		
**27. Where were the campaigns conducted?**		
**28. Who do you suggest that we talk to for more information about the IEC campaigns?**		
**(8) DISTRICT & HEALTH SUB-DISTRICT ISSUES**		
**29. Have any new policies been introduced at the district or health sub-district level in the last 3 months?** Probe for policies that might affect health.<br>*If YES, go to Qn 30 (and complete Part B #8), otherwise skip to Qn 31.*	1 = Yes<br>2 = No<br>88 = Don't know	[_____|_____]
**30. Who do you suggest that we talk to for more information about the policy changes?**		

STRUCTURED CONTEXTUAL RECORD (Form 1, Part A, Page 3)

Staff ID	Date completed
[____|____]	[____|____] / [____|____] / [____|____]<br>day month year

DISTRICT & HEALTH SUB-DISTRICT ISSUES - CONTINUED

**31. Have there been any important changes in guidelines for health centers, health workers, CHW, or on malaria diagnosis and treatment in the last 3 months?**<br>*If YES, go to Qn 32, otherwise skip to Qn 33.*	1 = Yes<br>2 = No<br>88 = Don't know	[_____|_____]
**32. Who do you suggest that we talk to for more information about the guideline changes?**		
**33. Have there been any important changes or gaps in staffing at the district or HSD level in the last 3 months?**<br>*If YES, go to Qn 34, otherwise skip to Qn 35.*	1 = Yes<br>2 = No<br>88 = Don't know	[_____|_____]
**34. Who do you suggest that we talk to for more information about district and HSD staffing?**		
**35. Have there been any important changes or gaps in staffing at the health centers in the study area in the last 3 months?**<br>*If YES, go to Qn 36, otherwise skip to Qn 37.*	1 = Yes<br>2 = No<br>88 = Don't know	[_____|_____]
**36. Who do you suggest that we talk to for more information about health center staffing?**		
**35. Have there been any important changes in supervision of health centers or health workers in the study area in the last 3 months?**<br>*If YES, go to Qn 36, otherwise skip to Qn 37.*	1 = Yes<br>2 = No<br>88 = Don't know	[_____|_____]
**36. Who do you suggest that we talk to for more information about supervision?**		

(9) ECONOMIC AND POLITICAL FACTORS

**37. Have there been any significant changes in economic or political factors that may have affected the performance of health workers, access to health centers, or health of the population (particularly children) in the last 3 months?**<br>*If YES, go to Qn 38 (and complete Part B #9), otherwise skip to Section 3.*	1 = Yes<br>2 = No<br>88 = Don't know	[_____|_____]
**38. Who do you suggest that we talk to for more information about these factors?**		

SECTION 3: ADDITIONAL COMMENTS

39. Is there anything that you think is important for us to know about the study area in the last 3 months that we've not asked?

THANK YOU FOR YOUR TIME AND SUPPORT

APPENDIX Z: STRUCTURED CONTEXTUAL RECORD (FORM 2)
DISTRICT LEVEL: COVERAGE INDICATORS

Interviewer ID	Date completed
[____|____]	[____|____] / [____|____] / [____|____]<br>day month year

TIME PERIOD COVERED:	1 = Baseline<br>2 = 0-3 months<br>3 = 4-6 months	4 = 7-9 months<br>5 = 10-12 months<br>6 = 13-15 months	7 = 16-18 months<br>8 = 19-21 months<br>9 = 21-24 months	[____|____]

INSECTICIDE-TREATED BEDNETS (ITNs) COVERAGE INDICATORS

**1. What is the source of the information about ITN coverage?**	Name of respondent or report<br>[____________________]	Location of respondent / report<br>[____________________]
**2. Has there been an ITN coverage survey in the study area in the last 3 months?**<br>*If YES, go to Qn 3, otherwise skip to next section*	1 = Yes 88 = Don't know<br>2 = No	[_____|_____]
**3. Date of Report**	**4. Date of Survey**	**5. Location(s) of survey(s)**

Indicator	Numerators (N) & Denominators (D)	Proportion or mean
**6. Proportion of households with at least one bed net**	N=[_____|_____|_____|_____|_____|_____]<br>D=[_____|_____|_____|_____|_____|_____]	[_____|_____] %
**7. Proportion of households with at least one ITN**	N=[_____|_____|_____|_____|_____|_____]<br>D=[_____|_____|_____|_____|_____|_____]	[_____|_____] %
**8. Mean number of nets per household**	D=[_____|_____|_____|_____|_____|_____]	[_____|_____]•[_____]
**9. Mean number of ITNs per household**	D=[_____|_____|_____|_____|_____|_____]	[_____|_____]•[_____]
**10. Proportion of children under five who slept under any net the prior night**	N=[_____|_____|_____|_____|_____|_____]<br>D=[_____|_____|_____|_____|_____|_____]	[_____|_____] %
**11. Proportion of children under five who slept under an ITN the prior night**	N=[_____|_____|_____|_____|_____|_____]<br>D=[_____|_____|_____|_____|_____|_____]	[_____|_____] %

INDOOR RESIDUAL SPRAYING (IRS) COVERAGE INDICATORS		
**12. What is the source of the information?**	Name of respondent or report<br>[________________________]	Location of respondent / report<br>[____________________]
**13. Is there any report on the coverage of IRS in the study area that is updated to include the last 3 months?**<br>*If YES, go to Qn 14, otherwise skip to next section*	1 = Yes 88 = Don't know<br>2 = No	[_____|_____]
**14. Date of Report**<br>[____________________]<br>dd/mm/yyyy	**15. Date of Survey (if appropriate)**<br>[____________________]<br>dd/mm/yyyy	**16. Author of report**<br>[____________________]
**Location**	**Proportion of households sprayed**	**Other information**
**17a. Nagongera**	[_____|_____] %	
**17b. Paya**	[_____|_____] %	
**17c. Kirewa**	[_____|_____] %	
**17d. Petta**	[_____|_____] %	
**17e. Kisoko**	[_____|_____] %	
**17f. Mulanda**	[_____|_____] %	
**17g. Rubongi**	[_____|_____] %	
**17h. Total**	[_____|_____] %	

POPULATION USE OF ACT AND RDT		
**18. What is the source of the information about ACT coverage?**	Name of respondent or report<br>[________________________]	Location of respondent / report<br>[____________________]
**19. Is there any report on use of ACT and RDT in the study area in the last 3 months?**<br>*If YES, go to Qn 20, otherwise skip to next section*	1 = Yes 88 = Don't know<br>2 = No	[_____|_____]
**20. What is the source of the data**	1 = Population survey<br>2 = HMIS data<br>3 = Other survey ____________<br>88 = Don't know	[_____|_____]
**Indicator**	**Numerators (N) & Denominators (D)**	**Proportion**
**20. Proportion of febrile episodes in children treated with an ACT**	N=[_____|_____|_____|_____|_____|_____]<br>D=[_____|_____|_____|_____|_____|_____]	[_____|_____] %
**21. Proportion of malaria cases treated with an ACT**	N=[_____|_____|_____|_____|_____|_____]<br>D=[_____|_____|_____|_____|_____|_____]	[_____|_____] %
**22. Proportion of febrile episodes tested with RDT**	N=[_____|_____|_____|_____|_____|_____]<br>D=[_____|_____|_____|_____|_____|_____]	[_____|_____] %
**23. Proportion of RDTs reported as positive**	N=[_____|_____|_____|_____|_____|_____]<br>D=[_____|_____|_____|_____|_____|_____]	[_____|_____] %

APPENDIX Z: STRUCTURED CONTEXTUAL RECORD (FORM 3) HEALTH CENTRES

**Interviewer ID**	**Respondent**	**Date completed**
[____|____]	[____________________/____________________] Name Position at HC	[____|____] / [____|____] / [____|____] day month year

**Health Centre ID**				
[____|____]	1 = Maundo<br>2 = Were<br>3 = Katajula<br>4 = Paya<br>5 = Pusere	6 = Nawire<br>7 = Kirewa<br>8 = Chawolo Kirewa<br>9 = Kisoko<br>**10 = Morkiswa**	11 = Petta<br>12 = Makawari<br>13 = Mbula<br>14 = Gwaragwara<br>**15 = Osia**	16 = Mwelo<br>17 = Lwala<br>18 = Panyangasi<br>19 = Mudodo<br>**20 = Chawolo Mulanda**

**TIME PERIOD COVERED:**				
	1 = Baseline<br>2 = 0-3 months<br>3 = 4-6 months	4 = 7-9 months<br>5 = 10-12 months<br>6 = 13-15 months	7 = 16-18 months<br>8 = 19-21 months<br>9 = 21-24 months	[____|____]

STAFFING

1. Have there been any changes or absences in health center STAFF in the last 3 months? 1 = Yes 88 = Don't know 2 = No [_____|_____]

If YES, go to Qn 2, otherwise skip to Qn 3.

HW position	Change	Replacement
1=In-charge; 2 = Nursing aide/asst;<br>3 = Volunteer; 77 = Other (describe)	1=Joined HC; 2 = Left HC<br>3 = On leave/away	1 = Replacement supplied;<br>2 = Empty position
**2a.** [____|____] ____________________	[____]	[____|____]
**2b.** [____|____] ____________________	[____]	[____|____]
**2c.** [____|____] ____________________	[____]	[____|____]
**2d.** [____|____] ____________________	[____]	[____|____]
**2e.** [____|____] ____________________	[____]	[____|____]

INTERVENTIONS

3. Have any new CHANGES been introduced at the health centers in the last 3 months? 1 = Yes 88 = Don't know 2 = No [_____|_____]

If YES, go to Qn 4, otherwise skip to Qn 5.

Name of program/donor	Intervention	Impact of Intervention
**4a.**		Summary of impact: on HW performance, patient access
**4b.**		
**4c.**		
**4d.**		

TRAINING PROGRAMS

5. Have any new TRAINING PROGRAMS been introduced at the health centers in the last 3 months? 1 = Yes 88 = Don't know 2 = No [_____|_____]

If YES, go to Qn 6, otherwise skip to Qn 7.

Description of training	Organisation running training	HWs participating	Impact of training
		Insert HW IDs (can be multiple)	Summary of impact: on HW performance, patient access
6a.			
6b.			
6c.			

RESEARCH PROGRAMS INVOLVING HEALTH CENTERS OR STAFF

7. Have any new RESEARCH PROGRAMS been introduced at the health centers in the last 3 months? 1 = Yes 88 = Don't know 2 = No [_____|_____]

If YES, go to Qn 8, otherwise skip to next section.

Project details	Name of research group	HWs participating	Impact of project
		Insert HW IDs (can be multiple)	Summary of impact: on HW performance, patient access
8a.			
8b.			
8c.			

OTHER CHANGES

9. Have there been any other changes in the last 3 months, aside from those above, that you think may have affected: 1 = Yes 88 = Don't know 2 = No [_____|_____]

(a) performance of health workers

(b) population access to health centres

(c) health status of the population, particularly children

Description of factors and changes	Impact of Change
	How affects HW performance, access or health status
10a.	
10b.	
10c.	

APPENDIX Z: STRUCTURED CONTEXTUAL RECORD (FORM 4) COMMUNITY LEVEL

Interviewer ID	Informant type<br>1 = Health officer<br>2 = Local informant	Informant sub-county code	Informant ID	Date completed
[_____|_____]	[____]	[_____]	[_____|_____]	[____|____] / [____|____] / [____|____]<br>day month year

TIME PERIOD COVERED:	1 = Baseline<br>2 = 0-3 months<br>3 = 4-6 months	4 = 7-9 months<br>5 = 10-12 months<br>6 = 13-15 months	7 = 16-18 months<br>8 = 19-21 months<br>9 = 21-24 months	[_____|_____]

We would like to know about three issues that we hope you can give us insight into in this local area. First, about changes that have affected the health of people here recently; second about changes that have affected access to health care services for people here recently; and lastly about changes that have affected the quality of services that people here have received for their health care recently.

We are interested in all aspects of health and all types of health care services that people use here.

PART 1: IMPACTS ON HEALTH

1. Can you tell us about anything that has happened in the past three months that you think has affected the health of people around here?

1 = Yes 88 = Don't know
2 = No

[______|______]

If YES, complete table, otherwise skip to next question.

**Event/activity/date**<br>List problems/ interventions that have changed, and have affected health locally	**Area involved/target group**<br>List parishes, or villages, if known, and nearest health centres	**Summary of event/activity**<br>Give details of what happened that the informant feels affected health.<br>Include how informant knows about this event/activity and **who initiated activity**.	**Impact on health**<br>Give details of what informer feels the impact on health is – who it affects

PART 2: IMPACTS ON ACCESS TO HEALTH CARE

2. Can you tell us about anything that has happened in the past three months that you think has affected how people here have accessed health care?

1 = Yes 88 = Don't know
2 = No

[_____|_____]

If YES, complete table, otherwise skip to next question.

**Programme/activity/date** List interventions/ events that changed health care access locally*	**Area involved** List parishes, or villages, if known, and nearest health centres	**Summary of programme/activity** Give details of what happened that the informant feels affected health care access.** Include how informant knows about this event/activity and **who initiated programme**.	**Impact on health** Give details of what informer feels the impact on health care access is – who it affects and how

*Probe for potential changes in access to the following:

- ☐ NGO campaigns or trainings
- ☐ CHW programmes
- ☐ Drug shop changes
- ☐ Private clinic changes
- ☐ Health centre changes
- ☐ Research activities changes

**Probe for barriers and facilitators including environmental, economic, political to each of the above providers of health care.

Continue on additional page if more information provided.

PART 3: IMPACTS ON QUALITY OF HEALTH CARE			
**3. Can you tell us about anything that you have heard in the past three months about the quality of health care and commodities* provided at different health care providers in this area?** *If YES, complete table, otherwise skip to next question.*	1 = Yes 88 = Don't know<br>2 = No		[_____|_____]
**Message/story/date**<br>List stories heard / messages interpreted about quality of care at health care providers**	**Area involved**<br>List provider/HC names, types and parishes, or villages, if known	**Summary of message/story**<br>Give details of the message/story and where the informant says the story originated.<br>Add **how far-reaching the message/story has gone according to informant.**	**Impact on health seeking**<br>Give details of what informer feels the impact of stories on behaviour of population locally in relation to health seeking

* Commodities include drugs, tests, other equipment, personnel etc.

** Probe for stories from the following sources:

- ☐ Local meetings
- ☐ Newspapers
- ☐ Television/radio
- ☐ General discussions

APPENDIX AA: AL & RDT SURVEILLANCE DATA COLLECTION SHEET

Health centre code	Staff ID	Data for the month of	Data Collection for (list AL package or RDT)
[____|____]	[____|____|____|____]	[____|____] / [____|____]<br>month year	1 = AL 6 tab pack (yellow)<br>2 = AL 12 tab pack (blue)<br>3 = AL 18 tab pack (green)<br>4 = AL 24 tab pack (brown)<br>5 = AL 24 tab pack (white)<br>6 = RDT<br>[____]

PART 1: STOCK CARD

Average Monthly consumption [____|____|____]	Minimum stock level [____|____|____]	Maximum stock level [____|____|____]

Date card updated	[____/____]<br>dd mm	[____/____]<br>dd mm	[____/____]<br>dd mm	[____/____]<br>dd mm	[____/____]<br>dd mm	[____/____]<br>dd mm
Recorded Balance on hand	[____|____|____]	[____|____|____]	[____|____|____]	[____|____|____]	[____|____|____]	[____|____|____]
Losses / Adjustments	[____]<br>1 = no issues<br>2 = unexplained loss<br>3 = explained loss<br>4 = explained adjustment<br>List details:	[____]<br>1 = no issues<br>2 = unexplained loss<br>3 = explained loss<br>4 = explained adjustment<br>List details:	[____]<br>1 = no issues<br>2 = unexplained loss<br>3 = explained loss<br>4 = explained adjustment<br>List details:	[____]<br>1 = no issues<br>2 = unexplained loss<br>3 = explained loss<br>4 = explained adjustment<br>List details:	[____]<br>1 = no issues<br>2 = unexplained loss<br>3 = explained loss<br>4 = explained adjustment<br>List details:	[____]<br>1 = no issues<br>2 = unexplained loss<br>3 = explained loss<br>4 = explained adjustment<br>List details:

Continue on a new page if stock card was completed more than 6 times in the month

PART 2: REQUISITION & ISSUE VOUCHER

Order placed date	[____/____]<br>dd mm	[____/____]<br>dd mm	[____/____]<br>dd mm	[____/____]<br>dd mm	[____/____]<br>dd mm	[____/____]<br>dd mm
Balance on hand	[____|____|____]	[____|____|____]	[____|____|____]	[____|____|____]	[____|____|____]	[____|____|____]
Quantity requested	[____|____|____]	[____|____|____]	[____|____|____]	[____|____|____]	[____|____|____]	[____|____|____]
Order received date	[____/____]<br>dd mm	[____/____]<br>dd mm	[____/____]<br>dd mm	[____/____]<br>dd mm	[____/____]<br>dd mm	[____/____]<br>dd mm
Quantity received	[____|____|____]	[____|____|____]	[____|____|____]	[____|____|____]	[____|____|____]	[____|____|____]

APPENDIX AA: AL & RDT SURVEILLANCE DATA COLLECTION SHEET

Health centre code	Staff ID	Data for the month of	Data Collection for (list AL package or RDT)
[____|____]	[____|____|____|____]	[____|____] / [____|____]<br>month year	

PART 3: ADDITIONAL INFORMATION

Record any other comments or observations:

Stock card	Requisition & Issue Voucher

APPENDIX BB: AL & RDT SURVEILLANCE DATA COLLECTION SHEET (1)

Health centre code	Staff ID	Data for the month of	Data Collection for (list AL package or RDT)
[____|____]	[____|____|____|____]	[____|____] / [____|____]<br>month year	1 = AL 6 tab pack (yellow)<br>2 = AL 12 tab pack (blue)<br>3 = AL 18 tab pack (green)<br>4 = AL 24 tab pack (brown)<br>5 = AL 24 tab pack (white)<br>6 = RDT<br>[____]

PART 1: STOCK CARD

Average Monthly consumption [____|____|____]	Minimum stock level [____|____|____]	Maximum stock level [____|____|____]

**Date card updated**	[____/____]<br>dd mm	[____/____]<br>dd mm	[____/____]<br>dd mm	[____/____]<br>dd mm	[____/____]<br>dd mm	[____/____]<br>dd mm
**Recorded Balance on hand**	[____|____|____]	[____|____|____]	[____|____|____]	[____|____|____]	[____|____|____]	[____|____|____]
**Losses / Adjustments**	[____]<br>1 = no issues<br>2 = unexplained loss<br>3 = explained loss<br>4 = explained adjustment<br>List details:	[____]<br>1 = no issues<br>2 = unexplained loss<br>3 = explained loss<br>4 = explained adjustment<br>List details:	[____]<br>1 = no issues<br>2 = unexplained loss<br>3 = explained loss<br>4 = explained adjustment<br>List details:	[____]<br>1 = no issues<br>2 = unexplained loss<br>3 = explained loss<br>4 = explained adjustment<br>List details:	[____]<br>1 = no issues<br>2 = unexplained loss<br>3 = explained loss<br>4 = explained adjustment<br>List details:	[____]<br>1 = no issues<br>2 = unexplained loss<br>3 = explained loss<br>4 = explained adjustment<br>List details:

Continue on a new page if stock card was completed more than 6 times in the month

PART 2: REQUISITION & ISSUE VOUCHER

**Order placed date**	[____/____]<br>dd mm	[____/____]<br>dd mm	[____/____]<br>dd mm	[____/____]<br>dd mm	[____/____]<br>dd mm	[____/____]<br>dd mm
**Balance on hand**	[____|____|____]	[____|____|____]	[____|____|____]	[____|____|____]	[____|____|____]	[____|____|____]
**Quantity requested**	[____|____|____]	[____|____|____]	[____|____|____]	[____|____|____]	[____|____|____]	[____|____|____]
**Order received date**	[____/____]<br>dd mm	[____/____]<br>dd mm	[____/____]<br>dd mm	[____/____]<br>dd mm	[____/____]<br>dd mm	[____/____]<br>dd mm
**Quantity received**	[____|____|____]	[____|____|____]	[____|____|____]	[____|____|____]	[____|____|____]	[____|____|____]

APPENDIX AA: AL & RDT SURVEILLANCE DATA COLLECTION SHEET (2)			
**Health centre code**	**Staff ID**	**Data for the month of**	**Data Collection for (list AL package or RDT)**
[____|____]	[____|____|____|____]	[____|____] / [____|____]<br>month year	

PART 3: ADDITIONAL INFORMATION	
**Record any other comments or observations:**	
**Stock card**	**Requisition & Issue Voucher**

APPENDIX CC: SEMI-STRUCTURED QUESTIONNAIRE
HEALTH FACILITY IN-CHARGES: HFI & STANDARD CARE

Health centre code	Health Worker ID	Date
[____|____]	[____|____|____|____]	[____|____] / [____|____] / [____|____]<br>day month year

Are you the in-charge of this health centre?
1 = In-charge
2 = Acting in-charge
3 = No
If 'no', please terminate interview [____|____]

SECTION 1: FINANCIAL MANAGEMENT

**1. 'In the last 6 months, has this health center had enough money available to buy all of the supplies needed for day-to-day running of the facility (e.g. for soap, repairs, etc)?'**	1 = Yes 88 = Don't know<br>2 = No 99 = Refused to answer	[_____|_____]
**2. 'In the last 6 months, has this health center had enough money available to pay for support staff to help clean and maintain the health center?'**	1 = Yes 88 = Don't know<br>2 = No 99 = Refused to answer	[_____|_____]
**3. 'In general, how often does this health facility receive PHC funds?'**	1 = Regularly every quarter (4 times a year)<br>2 = Regularly every 3-6 months (2-3 times a year)<br>3 = Irregularly, about 2 times a year<br>4 = Irregularly, about once a year<br>5 = Never<br>88 = Don't know<br>99 = Refused to answer	[_____|_____]
**4. 'In the last 6 months, has this health center received any PHC funds?'** *If yes, go to Qn 5, otherwise skip to Qn 6*	1 = Yes 88 = Don't know<br>2 = No 99 = Refused to answer	[_____|_____]
**5. 'In the last 6 months, have the PHC funds been adequate to cover your needs at this health center?'**	1 = Yes 88 = Don't know<br>2 = No 99 = Refused to answer	[_____|_____]
**6. 'In your opinion, when PHC funds are available, are they used in the right way at this health center?'**	1 = Yes 88 = Don't know<br>2 = No 99 = Refused to answer	[_____|_____]
**7. 'In your opinion, are staff members in this health center able to budget and account for funds well?'**	1 = Yes 88 = Don't know<br>2 = No 99 = Refused to answer	[_____|_____]

8. Please provide any additional comments about management of money and PHC funds at this health center.

SSQ: HEALTH FACILITIES (2)

Health centre code	Health Worker ID	Date
[____|____]	[____|____|____|____]	[____|____] / [____|____] / [____|____]<br>day month year

SECTION 2: PATIENT MANAGEMENT

1. "Is it possible to test patients for malaria at this health center?" (*Based on availability of the equipment and staff to carry out the test*)
If YES, go to Qn 2, otherwise skip to Qn 5

1 = Yes 88 = Don't know
2 = No 99 = Refused to answer
[_____|_____]

2. 'What tests for malaria can be done at this health center?' Read out each test and indicate for each

1 = Yes 88 = Don't know
2 = No 99 = Refused to answer

[____] Microscopy (blood smear)
[____] Rapid diagnostic test for malaria
[____] Other (describe)____

3. "Do health workers at your health facility usually test patients for malaria before giving antimalarial treatment?"
If NO, go to Qn 4, otherwise skip to Qn 5

1 = Yes 88 = Don't know
2 = No 99 = Refused to answer
[_____|_____]

4. "If health workers at your health facility do not usually test patients for malaria before giving treatment, why?"
Record all answers given

1 = There is not enough time to test all patients
2 = We lack the supplies needed to do the tests
3 = Patients/Caregivers are not willing to be tested
4 = I don't trust the results of the malaria tests
5 = I know better than the test when a patient has malaria
6 = In my experience, all fevers are due to malaria
7 = Malaria tests are not done correctly at this health center
77 = Other ______________________________
88 = Don't know
99 = Refused to answer

[_____|_____]
[_____|_____]
[_____|_____]
[_____|_____]

5. "What treatment should be given to patients with uncomplicated malaria?"
Record all answers given

1 = Artemether-lumefantrine (Coartem/Lumartem)
2 = Artesunate + amodiaquine
3 = Dihydroartemisinin-piperaquine (Duocotexcin)
4 = Any artemisinin-based combination therapy
5 = Quinine
6 = Chloroquine + sulfadoxine-pyrimethamine (Homapak)
77 = Other ______________________________
88 = Don't know
99 = Refused to answer

[_____|_____]
[_____|_____]
[_____|_____]
[_____|_____]

6. 'Are you usually able to provide this treatment to your patients with uncomplicated malaria at your health facility?' *If NO, go to Qn 7, otherwise skip to Qn 8*

1 = Yes 88 = Don't know
2 = No 99 = Refused to answer
[_____|_____]

7. "If not, why?"
Record all answers given

1 = The drug is often out of stock
2 = The patients can't afford to buy
77 = Other ______________________________
88 = Don't know
99 = Refused to answer

[_____|_____]
[_____|_____]

8. 'How confident are you that staff at your health centre can correctly <u>diagnose</u> malaria in patients?'

1 = Very confident 88 = Don't know
2 = Confident 99 = Refused to answer
3 = Not confident
[_____|_____]

SSQ: HEALTH FACILITIES (3)		
**Health centre code**	**Health Worker ID**	**Date**
[____|____]	[____|____|____|____]	[____|____] / [____|____] / [____|____]<br>day month year

SECTION 2 cont: PATIENT MANAGEMENT		
**9. 'How confident are you that staff at your health centre can correctly treat malaria in patients?'**	1 = Very confident<br>2 = Confident<br>3 = Not confident<br>88 = Don't know<br>99 = Refused to answer	[_____|_____]
**10. 'How confident are you that staff at your health centre can correctly diagnose other illnesses (not malaria) in patients?'**	1 = Very confident<br>2 = Confident<br>3 = Not confident<br>88 = Don't know<br>99 = Refused to answer	[_____|_____]
**11. 'How confident are you that staff at your health centre can correctly treat other illnesses (not malaria) in patients?'**	1 = Very confident<br>2 = Confident<br>3 = Not confident<br>88 = Don't know<br>99 = Refused to answer	[_____|_____]
**12. 'Are staff at your health centre able to give your patients a FULL explanation about their diagnosis and treatment?"**<br>*If NO, go to Qn 13. Otherwise, skip to Qn 14.*	1 = Yes<br>2 = No<br>88 = Don't know<br>99 = Refused to answer	[_____|_____]
**13. "If not, why?"**<br>*Record all answers given*	1 = They do not have enough time<br>2 = The patients are not willing to wait<br>3 = Sometimes they are not sure of the diagnosis<br>4 = Sometimes they are not sure of the treatment<br>77 = Other ____<br>88 = Don't know<br>99 = Refused to answer	[_____|_____]<br>[_____|_____]<br>[_____|_____]
**14. 'Are staff at your health centre able to refer severely ill patients to a higher-level health center when needed?'**<br>*If YES, go on to Qn 15, if NO, go to Qn 16.*	1 = Yes<br>2 = No<br>88 = Don't know<br>99 = Refused to answer	[_____|_____]
**15. 'If yes, which health facility do you routinely refer to?'**	[_______________________________]<br>[_______________________________]<br>*HC Name*	[_____|_____]<br>[_____|_____]<br>*HC Study ID where possible*
**16. 'Why do you choose to refer to that health facility?'**	1 = It is the closest health facility<br>2 = I know and trust the health workers there<br>77 = Other ___________________________	[_____|_____]
**17. "If not, why?"**<br>*Record all answers given*	1 = The patients lack the money to go<br>2 = The patients don't have time to go<br>3 = The other health centers turn my patients away<br>4 = I don't know how to refer<br>77 = Other____<br>88 = Don't know<br>99 = Refused to answer	[_____|_____]<br>[_____|_____]<br>[_____|_____]
**16. Please provide any additional comments about patient management at this health center.**		

SSQ: HEALTH FACILITIES (3)

Health centre code	Health Worker ID	Date
[____|____]	[____|____|____|____]	[____|____] / [____|____] / [____|____]<br>day month year

SECTION 3: HEALTH WORKER ATTITUDES

**1. 'How motivated would you say the health workers are at this health center?'**	1 = Very motivated<br>2 = Satisfactorily motivated<br>3 = Not motivated	88 = Don't know<br>99 = Refused to answer	**[_____|_____]**
**2. 'Have you observed any improvement in motivation towards work amongst health workers at this health centre in the past year?'**	1 = Significant improvement<br>2 = Some improvement<br>3 = No change<br>4 = decrease in motivation	88 = Don't know<br>99 = Refused to answer	**[_____|_____]**
**3. 'How often would you say that the health workers at this health center put their own priorities before the needs of patients?'**	1 = Always<br>2 = Sometimes<br>3 = Never	88 = Don't know<br>99 = Refused to answer	**[_____|_____]**

EVALUATION OF PRIME TOOLS: HFI health facilities only

**1. How often is the PHC Fund Management tool used?**	1 = Every week<br>2 = Every month<br>3 = Every time the PHC fund is expected	4 = Not often<br>5= Never<br>88 = Don't know<br>99 = Refused to answer	[_____|_____]
**2. How easy is the PHC Fund Management tool to use?**	1 = Easy<br>2 = Somewhat easy<br>3 = Not easy / difficult	88 = Don't know<br>99 = Refused to answer	[_____|_____]
**3. How useful is the PHC Fund Management tool for budgeting and accounting?**	1 = Useful<br>2 = Somewhat useful<br>3 = Not useful	88 = Don't know<br>99 = Refused to answer	[_____|_____]
**4. How often is the ADDAT tool used?**<br>*If 'never', end here, otherwise go to Qn 5*	1 = Every week<br>2 = Every month<br>3 = Every time a drug delivery is expected	4 = Not often<br>5 = Never<br>88 = Don't know<br>99 = Refused to answer	[_____|_____]
**5. How easy is the ADDAT tool to use?**	1 = Easy<br>2 = Somewhat easy<br>3 = Not easy / difficult	88 = Don't know<br>99 = Refused to answer	[_____|_____]
**6. How useful is the ADDAT tool for managing issues in the distribution of drugs from the district/sub-district to your health centre?**	1 = Useful<br>2 = Somewhat useful<br>3 = Not useful	88 = Don't know<br>99 = Refused to answer	[_____|_____]
