# Supplementary material for: The impact of an intervention to introduce malaria rapid diagnostic tests on fever case management in a high transmission setting in Uganda: A mixed-methods cluster-randomized trial (PRIME)
Source: PLoS One. 2017 Mar 13;12(3):e0170998. doi: 10.1371/journal.pone.0170998 (PMC5347994; doi:10.1371/journal.pone.0170998)
Supplement: S5 File — This file includes a description of the way the intervention delivery was monitored, results of how the training and supervision were delivered at intervention health centres, description of what happened at control health centres and movement between health centres. It also includes copies of the questionnaires filled by trainers and participants to monitor delivery. (DOCX) [file pone.0170998.s005.docx]

**Supplemental File 5**

**Delivery of fever case management (FCM) module in the PRIME study**

**Monitoring delivery.** The delivery of the PRIME intervention was closely monitored and documented as part of the process evaluation. To monitor the delivery of the FCM training, both trainers and trainees completed questionnaires (appended below), which captured attendance, participation levels, the use of planned materials and activities, challenges in implementing intended training, the perceived relevance of training components to the participants, concerns about issues arising in the training, and levels of confidence of participants in the domains of focus in the training. In addition, the PROCESS study team attended the training sessions as observers and took fieldnotes about the ways in which participants engaged with the training and topics. To monitor the delivery of the supervision visits, the supervisory team kept narrative records of each visit for each health centre. In addition, the PROCESS study team were visiting each health centre at least 3-monthly and took informal fieldnotes about the ways in which mRDTs were, or were not, becoming a part of health centre practices, as well as comments, complaints and requests from health centre staff.

**Training delivered.** All components of the FCM training were delivered, with only minor adaptations made in order to keep to time when several sessions started late. In all, 25 of 29 invited health workers attended the two-day fever case management training, which was held in two groups one week apart in order to allow health centers to remain open. These health workers represented all 10 intervention health centers.

The first day consisted of classroom lectures, including how to manage febrile patients with positive and negative mRDT results, with seven key alternative diagnoses defined. All participants practiced performing mRDTs on their colleagues. The second day involved clinical placement at a large health center where each participant tested children with suspected malaria and practiced management of negative and positive mRDT results.

**Supervision delivered.** One week after the training, each health facility was visited by the training team to provide mentorship on how to set up the working area for mRDTs and how to record their use in registers, how to manage fever patients with positive or negative results and to review stocks of ACTs and mRDTs. A key challenge noted was the low cadre of some of the staff at smaller health centers, who struggled to identify alternative causes of fever for mRDT negative patients.

One month after this (July 2011), the training team re-visited each health center to provide support supervision. One health center (#20) was closed for the week at the time of the supervisory visit, with the staff absent. This HC did not receive this follow up supervision. During their visits training team observed consultations, performance of mRDTs, reviewed records, gave feedback to staff and responded to issues raised by staff. They noted that at some health centers the staff treating patients were different to those who had attended training. The team provided summary training to these staff during supervisory visits. They also noted infrastructural issues preventing appropriate storage or use of mRDTs, and took action to tackle these where possible. For example, in some cases storage of mRDTs was sub-optimal (by a window), keys to store cupboards were missing, clocks or timers were not available, and some health centers had multiple types of mRDTs from different sources, which required guidance in performance differences.

Six months later (January 2012), the training team re-visited each intervention health center a final time for support supervision. One health center (#20) was again closed at the time of the visit with both staff absent and therefore did not receive the supervisory support. The trainers compared performance at each health center with the previous visits and provided feedback to staff. In many health centers, the team noted that performance indicators remained similar and infrastructural challenges persisted, although all had sufficient mRDTs and ACTs in stock. Some health centers had carried out few or no mRDTs in previous months, which staff reported during the supervisory visits to be due to high workload.

**Control health centres.** Health centers that did not receive the intervention were classified as ‘control’. These health centers received no training or supervision to improve quality of care but they did receive training in completing consultation registers, which were to be used to monitor case load and case management. This training, and on-the-job feedback on record keeping in these registers, was repeated at all intervention and control health centers.

**Movement between health centres.** The study team worked closely with the District Health Management Team, to reduce likelihood of contamination between intervention and control health centres. This included an agreement that health worker reassignment within the district would be restricted such that health workers at intervention facilities could move only to other intervention facilities, and likewise control health workers would be moved only to other control facilities. This was implemented in the main, with limited cross-over during the evaluation period, although this was challenging for the DHMT who were keen that the new skills learned at intervention facilities be dispersed to the other health centres. From our records, only 1 health worker was moved from an intervention to a control health centre between the training delivery and final round of patient exit interviews.

| FEVER CASE MANAGEMENT TRAINING  SELF-FILLED QUESTIONNAIRE for TRAINERS (1) | | | | | | | | | | | | | |
| --- | --- | --- | --- | --- | --- | --- | --- | --- | --- | --- | --- | --- | --- |
| Thank you for assisting with this health centre management training! We would appreciate your feedback on the training sessions you attended on staffing. Please take a moment to answer the following questions, as your comments will help us improve future trainings. There is no need to write your name on this questionnaire, and all responses will be kept strictly confidential. Thank you! | | | | | | | | | | | | | |
| Trainer Study ID  [____\|____] | Date of training  [____\|____]**/ ­­**[____\|____]**/**[____\|____]  day month year | | | | | | | Study ID of other Trainers present  [____\|____], [____\|____], [____\|____] | | | | | |
| Training group number  [_____\|_____] | Participant health facility IDs | | | | | | | |  | | | | |
|  | [____\|____]  [____\|____] | | [____\|____]    [____\|____] | | | [____\|____]  [____\|____] | | | | [____\|____]  [____\|____] | | | [____\|____]  [____\|____] |
|  | Participant Study IDs | | | | | | | | |  | | |  |
|  | [____\|____]  [____\|____] | | [____\|____]    [____\|____] | | | [____\|____]  [____\|____] | | | | [____\|____]  [____\|____] | | | [____\|____]  [____\|____] |
| Characteristics of participants (range) | | | |  | | |  | | | | |  | |
| Age | | Sex | | | Qualifications | | | | | | Other trainings attended | | |

| TRAINER QUESTIONNAIRE | | | | | | | | | | | | | | | | | | |
| --- | --- | --- | --- | --- | --- | --- | --- | --- | --- | --- | --- | --- | --- | --- | --- | --- | --- | --- |
| 1. Did the training start on time? If not, what was the cause of the delay? | | | | | | | | | | | | | | | | | | |
| 2. What was the general atmosphere during the session? | | | | | | | | | | | | | | | | | | |
| 3. What level of contribution did the participants have? | | | | | [____] | | | | | | | | | | | | | |
| 1 = All contributed a lot | 3 = Only some contributed | | | |  |  |  |  |  |  |  |  |  |  |  |  |  |  |
| 2 = All contributed at some point | 4 = None contributed  (please specify health centres/IDs if possible) | | | | | | | [____\|____], [____\|____], [____\|____] | | | | | | | | | | |
| Please summarise your opinion of the course by ranking the following using: | | | | | | | | | | 1 = Poor  2 = Fair | | 3 = Good  4 = Very good | | | 5 = N/A | | | |
| 4. Attendance of the session by the in-charges | | | | | | | | | | | | | | | | | [____] | |
| 5. How the training materials were received | | | | | | | | | | | | | | | | | [____] | |
| 6. How the training aids used were received (if applicable) | | | | | | | | | | | | | | | | | [____] | |
| 7. Your overall assessment of how the training went | | | | | | | | | | | | | | | | | [____] | |
| 8. Did the training go as planned? Please explain why. | | | | | | | | | | | | | | | | | 1 = Yes  2 = No  [____] | |
| 9. Can you please describe any difficulties you encountered? | | | | | | | | | | | | | | | | | | |
| 10. What impact did these have on the quality of the information received by the participants? | | | | | | | | | | | | | | | | | | |
| Please indicate if the following items were available at the training | | | | | | | | | 1 = Yes | | 2 = No | | | 3 = N/A | | | | |
|  | | **Provided** | | **Amount sufficient** | | |  | | | | | | **Provided** | | | | | **Amount sufficient** |
| 11. Sharps bin | | [____] | | [____] | | | 16. Blood transfer devices | | | | | | [____] | | | | | [____] |
| 12. Standard waste bin | | [____] | | [____] | | | 17. Sealed packets of alcohol swabs | | | | | | [____] | | | | | [____] |
| 13. Pairs of latex or nitrite gloves | | [____] | | [____] | | | 18. RDT buffet (check it matches the RDT) | | | | | | [____] | | | | | [____] |
| 14. Sealed packets of RDTs | | [____] | | [____] | | | 19.Timer | | | | | | [____] | | | | | [____] |
| 15. Sealed packets of lancets | | [____] | | [____] | | | 20. RDT bench aid (check it matches the RDT type) | | | | | | [____] | | | | | [____] |
| How useful do you think the following training activity was for the health centre clinical staff? | | | | | | | | | | | 1 = Not very useful  2 = Somewhat useful | | | 3 = Very useful  4 = Don’t know | | | | |
| 21. Discussion of evaluation of febrile patients and selection of patients for RDT testing | | | [____] | | | 25. Discussion of recognition and referral of patients with severe illness | | | | | | | | | | [____] | | |
| 22. Practice of performing and reading an RDT | | | [____] | | | 26. Discussion of patient education | | | | | | | | | | [____] | | |
| 23. Discussion of management of a patient with fever and a positive RDT | | | [____] | | | 27. Discussion of RDT storage and monitoring | | | | | | | | | | [____] | | |
| 24. Discussion of management of a patient with fever and a negative RDT | | | [____] | | |  | | | | | | | | | |  | | |
| 13. What questions or concerns were raised by this group about the training of health workers on fever case management? (Please list) | | | | | | | | | | | | | | | | | | |
| 14. How were each of these concerns addressed, if at all? | | | | | | | | | | | | | | | | | | |
| 15. Which of these concerns do you think were still present at the end of the training? | | | | | | | | | | | | | | | | | | |
| 16. Would you change anything about the fever case management training sessions for clinical staff? If yes, what would you change? How would you change it? | | | | | | | | | | | | | | | | | | |
| 17. Do you have any general comments on this course? | | | | | | | | | | | | | | | | | | |

Thank you!

| FEVER CASE MANAGEMENT TRAINING  SELF-FILLED QUESTIONNAIRE for PARTICIPANTS (1) | | | | | |
| --- | --- | --- | --- | --- | --- |
| Thank you for participating in this training on staffing! We would appreciate your feedback on the training sessions you have attended. Please take a moment to answer the following questions, as your comments will help us improve future trainings. There is no need to write your name on this questionnaire, and all responses will be kept strictly confidential. Thank you! | | | | | |
| Health worker ID  [____] | | Today’s date  [____\|____]**/ ­­**[____\|____]**/**[____\|____]  day month year | | Date training began  [____\|____]**/ ­­**[____\|____]**/**[____\|____]  day month year | |
| 1. Your qualification  [____] | 1 = Clinical Officer  2 = Nurse  3 = Other (list)  _________________ | | **2. What is your age?**  [____\|____] years | 3. How long have you worked actively as health centre in-charge?  [____\|____] OR [____\|____]  months years | |
| 4. How many trainings have you attended for health centre clinical staff in the past?  [____\|____] | | | 5. When was the last training you attended and what was the topic of training? | | |
|  |  |  | Topic | | [___\|___]**/ ­­**[___\|___]**/**[___\|___]  day month year |
| **6. What other PRIME courses have you attended so far?** | | | | | |

| TRAINEE QUESTIONNAIRE | | | | | | | | | | | | | |
| --- | --- | --- | --- | --- | --- | --- | --- | --- | --- | --- | --- | --- | --- |
| Please summarise your opinion of the course by ranking the following using: | | | | | 1 = Poor  2 = Fair | | | 3 = Good  4 = Very good | | 5 = N/A | | | |
| 7. Achievement of your aims when you enrolled in this training | | [____] | | 10. Use of Training Aids (if applicable) | | | | | | | | | [____] |
| 8. General achievements of the course objectives | | [____] | | 11. Your overall assessment | | | | | | | | | [____] |
| 9. Effectiveness of Trainer(s) | | [____] | |  | | | | | | | | |  |
| 12. How interested do you think most of the other clinical staff in your group were throughout the training sessions? | | | | 1 = Not Very Interested  2 = Somewhat Interested | | | 3 = Very Interested | | | | | [____] | |
| How useful did you find each of the following training activities? | | | | 1 = Not very useful  2 = Somewhat useful | | | 3 = Very useful  4 = Don’t know | | | | | | |
| 13. Discussion of evaluation of febrile patients and selection of patients for RDT testing | | [____] | | 17. Discussion of recognition and referral of patients with severe illness | | | | | | | | [____] | |
| 14. Practice of performing and reading an RDT | | [____] | | 18. Discussion of patient education | | | | | | | | [____] | |
| 15. Discussion of management of a patient with fever and a positive RDT | | [____] | | 19. Discussion of RDT storage and monitoring | | | | | | | | [____] | |
| 16. Discussion of management of a patient with fever and a negative RDT | | [____] | |  | | | | | | | | [____] | |
| 15. What would you like to add or change about the training sessions? | | | | | | | | | | | | | |
| 16. Please write any concerns you have about fever case management or any other comments following up on this training? | | | | | | | | | | | | | |
| 17. Please write any general comments you have on this course? | | | | | | | | | | | | | |
| Please summarise your opinion of the course by ranking the following using: | | | | | | 1 = Poor  2 = Fair | | | 3 = Good  4 = Very good | | 5 = N/A | | |
| 18. I feel confident that I can do good history taking including asking good questions and active listening | [____] | | 24. I feel confident that I can manage the common non-malaria febrile illnesses according to treatment guidelines | | | | | | | | | | [____] |
| 19. I feel confident that I can perform a clinical examination on a patient with fever correctly | [____] | | 25. I feel confident that I can assess a patient for severe signs of illness | | | | | | | | | | [____] |
| 20. I feel confident that I can select a patient for RDT testing based on clinical evaluation | [____] | | 26. I feel confident that I can properly refer a patient when they are severely ill to higher level facilities | | | | | | | | | | [____] |
| 21. I feel confident that I can perform an RDT 22. correctly and safely | [____] | | 27. I feel confident that I can provide pre-referral treatments to severely ill patients | | | | | | | | | | [____] |
| 22. I feel confident that I can treat a patient with fever and a positive RDT according to national guidelines | [____] | | 28. I feel confident that I can use good communication skills when giving patients information about malaria and its treatment | | | | | | | | | | [____] |
| 23. I feel confident that I can manage a patient with fever but a negative RDT | [____] | | 29. I feel confident that I can store and monitor RDTs’ expiry dates correctly | | | | | | | | | | [____] |

**Thank you!**
