# Supplementary material for: The impact of an intervention to introduce malaria rapid diagnostic tests on fever case management in a high transmission setting in Uganda: A mixed-methods cluster-randomized trial (PRIME)
Source: PLoS One. 2017 Mar 13;12(3):e0170998. doi: 10.1371/journal.pone.0170998 (PMC5347994; doi:10.1371/journal.pone.0170998)
Supplement: S6 File — This file shows the self-filled questionnaire health workers were asked to complete 10 months after the initial intervention training. These data were used to represent the confidence health workers had in the areas covered by the training and supervision. (DOCX) [file pone.0170998.s006.docx]

**Supplemental File 6**

**Questionnaire evaluating health worker confidence in following case management guidelines**

| CASE MANAGEMENT GUIDELINES |  |  |  |  | |
| --- | --- | --- | --- | --- | --- |
| Decide how much you agree with the statements below, and circle the number closest to your opinion on the right | Strongly agree | Agree | Disagree | Strongly disagree | |
| 1. I am able to identify patients with malaria without using a malaria test | 1 | 2 | 3 | 4 | |
| 1. I never give an antimalarial without first testing the patient for malaria | 1 | 2 | 3 | 4 | |
| 1. *It is not possible to test every patient for malaria because there is not enough time | 1 | 2 | 3 | 4 | |
| 1. *It is not possible to test every patient for malaria because sometimes we do not have the supplies we need | 1 | 2 | 3 | 4 | |
| 1. *Not all patients are willing to be tested | 1 | 2 | 3 | 4 | |
| 1. *Sometimes I think that malaria tests are not performed correctly at my health centre | 1 | 2 | 3 | 4 | |
| 1. *I do not always trust the quality of Rapid Diagnostic Tests (mRDTs) for malaria | 1 | 2 | 3 | 4 | |
| 1. *If the malaria test is negative but the patient has fever, it is best to prescribe an antimalarial drug to be safe | 1 | 2 | 3 | 4 | |
| 1. *In my experience, fevers are usually due to malaria | 1 | 2 | 3 | 4 | |
| 1. Patients who have uncomplicated malaria should always be given an antimalarial containing artemisinin | 1 | 2 | 3 | 4 | |
| 1. I am confident that I can diagnose correctly those fevers that are not due to malaria | 1 | 2 | 3 | 4 | |
| 1. I am confident that I can treat those fevers that are not due to malaria | 1 | 2 | 3 | 4 | |
| 1. *At my health centre, we do not have enough tests to diagnose fevers that are not due to malaria | 1 | 2 | 3 | 4 | |
| 1. *At my health centre, we do not have enough drugs to treat fevers that are not due to malaria | 1 | 2 | 3 | 4 | |
| 1. *I cannot give a full explanation to every patient about their diagnosis and treatment because I do not have time | 1 | 2 | 3 | 4 | |
| 1. *If a patient thinks that they have malaria, I find it hard to send them away without an antimalarial drug | 1 | 2 | 3 | 4 | |
| 1. *There are times when I want to refer a patient but cannot because of that patient’s circumstances | 1 | 2 | 3 | 4 | |
| 1. Please provide any further comments relating to case management or any of the questions above: | | | | |  |

*Note: for analysis, responses for each item were recoded, with strongly agree/agree merged and scoring ‘1’, and disagree/strongly disagree merged and scoring ‘0’. Items indicated above with an * were reverse coded. The total score for each respondent was an aggregate.*
